# Prognostic and therapeutic implications of extracellular matrix associated gene signature in renal clear cell carcinoma

**DOI:** 10.1038/s41598-021-86888-7

**Published:** 2021-04-07

**Authors:** Pankaj Ahluwalia, Meenakshi Ahluwalia, Ashis K. Mondal, Nikhil Sahajpal, Vamsi Kota, Mumtaz V. Rojiani, Amyn M. Rojiani, Ravindra Kolhe

**Affiliations:** 1 Department of Pathology, Medical College of Georgia, Augusta University, Augusta, GA USA; 2 Department of Medicine, Medical College of Georgia, Augusta University, Augusta, GA USA

**Keywords:** Cancer, Genetics

## Abstract

Complex interactions in tumor microenvironment between ECM (extra-cellular matrix) and cancer cell plays a central role in the generation of tumor supportive microenvironment. In this study, the expression of ECM-related genes was explored for prognostic and immunological implication in clear cell renal clear cell carcinoma (ccRCC). Out of 964 ECM genes, higher expression (*z*-score > 2) of 35 genes showed significant association with overall survival (OS), progression-free survival (PFS) and disease-specific survival (DSS). On comparison to normal tissue, 12 genes (*NUDT1*, *SIGLEC1*, *LRP1*, *LOXL2*, *SERPINE1*, *PLOD3*, *ZP3*, *RARRES2*, *TGM2*, *COL3A1*, *ANXA4*, and *POSTN*) showed elevated expression in kidney tumor (n = 523) compared to normal (n = 100). Further, Cox proportional hazard model was utilized to develop 12 genes ECM signature that showed significant association with overall survival in TCGA dataset (HR = 2.45; 95% CI [1.78–3.38]; *p* < 0.01). This gene signature was further validated in 3 independent datasets from GEO database. Kaplan–Meier log-rank test significantly associated patients with elevated expression of this gene signature with a higher risk of mortality. Further, differential gene expression analysis using DESeq2 and principal component analysis (PCA) identified genes with the highest fold change forming distinct clusters between ECM-rich high-risk and ECM-poor low-risk patients. Geneset enrichment analysis (GSEA) identified significant perturbations in homeostatic kidney functions in the high-risk group. Further, higher infiltration of immunosuppressive T-reg and M2 macrophages was observed in high-risk group patients. The present study has identified a prognostic signature with associated tumor-promoting immune niche with clinical utility in ccRCC. Further exploration of ECM dynamics and validation of this gene signature can assist in design and application of novel therapeutic approaches.

## Introduction

Cancer is one of the leading causes of mortality worldwide among which renal cancer constitutes about 5% of all cancers in men and 3% in women with about 140,000 deaths annually worldwide^1^. Renal cancers have very low 5-year survivability in metastatic renal cell carcinoma (12%)^2^. Current prognostic methods are based on clinicopathological features based on TNM (Tumor Node Metastasis staging and grading) which are effective, but the survival of the patients vary even in patients with similar prognosis^3^. The inherent heterogeneity in renal cell carcinoma has been shown to be a primary factor for failure of treatment and development of resistance^4^. Thus, despite several breakthroughs in diagnosis and treatment, high heterogeneity in renal cancer demands further exploration. In this direction, there is still a need to understand the interactions of the molecular players involved in tumorigenesis and its clinical relevance. Extracellular matrix (ECM) is one such entity that forms the biological framework of the tissue and plays a role in its carcinogenesis. ECM is a non-cellular component essential to maintain physical and biochemical stability of the tissue. Its composition varies depending on the tissue and developmental trajectory, but major components are fibrous proteins, proteoglycans, water, and minerals. The bidirectional interaction between cells and ECM influences cell adhesion and migration^5^. ECM is a dynamic entity, and its components are constantly in flux whereby it undergoes deposition and degradation that is significantly altered in diseased conditions. The change in ECM homeostasis has an important role in tumor growth and metastasis. The dynamic nature of ECM influences all the hallmarks of cancer that include uncontrolled growth, angiogenesis, invasion, disruption of metabolomics, and prevention of deadly immune response^6^.

Tumor microenvironment (TME) is a surrounding area of tumor composed of immune cells, blood vessels, cytokines, chemokines, other signalling molecules and ECM^7^. Along with malignant cells, other cells like endothelial cells, fibroblasts, immune cells interact in ECM in cancer. Several cells like T-regulatory cells, Myeloid-derived suppressor cells (MDSCs), Tumor-associated macrophages (TAMs) create an immunosuppressive environment that is conducive for tumor growth. CD8 T cells, CD4 T-cells, DCs and matrix remodelling enzymes like MMPs and ADAMTs play an important role in maintaining pro-inflammatory immunogenic environment^8^. These variations are important to understand as they are known to play a critical role in tumor growth and invasion^9^. Therefore, the interaction of immune cells and tumor growth and its relevance to ECM is an interesting area of exploration.

The advent of genomics and transcriptomics has led to identification of several gene signatures with prognostic utility in several cancers^10–12^. Here, we explored variation in the expression of extracellular matrix genes in renal clear cell carcinoma. In this study, we developed a 12-gene ECM gene prognostic signature based on TCGA dataset and validated it in 3 independent GEO datasets. These genes had significantly higher expression levels in RCC tumor compared to normal tissue. Further, differential gene analysis identified significant dysregulation of homeostatic functions in high-risk patients. The patients with high ECM signature also showed a higher fraction of tumor-promoting T regulatory and M2 macrophages. These results suggest that the 12-gene ECM signature can be used for prognosis as well as identify patients with suppressive immune phenotypes which can benefit from emerging therapies under research such as T-reg depletion immunotherapies.

## Results

### Identification of survival-associated ECM genes

**Figure 1:**
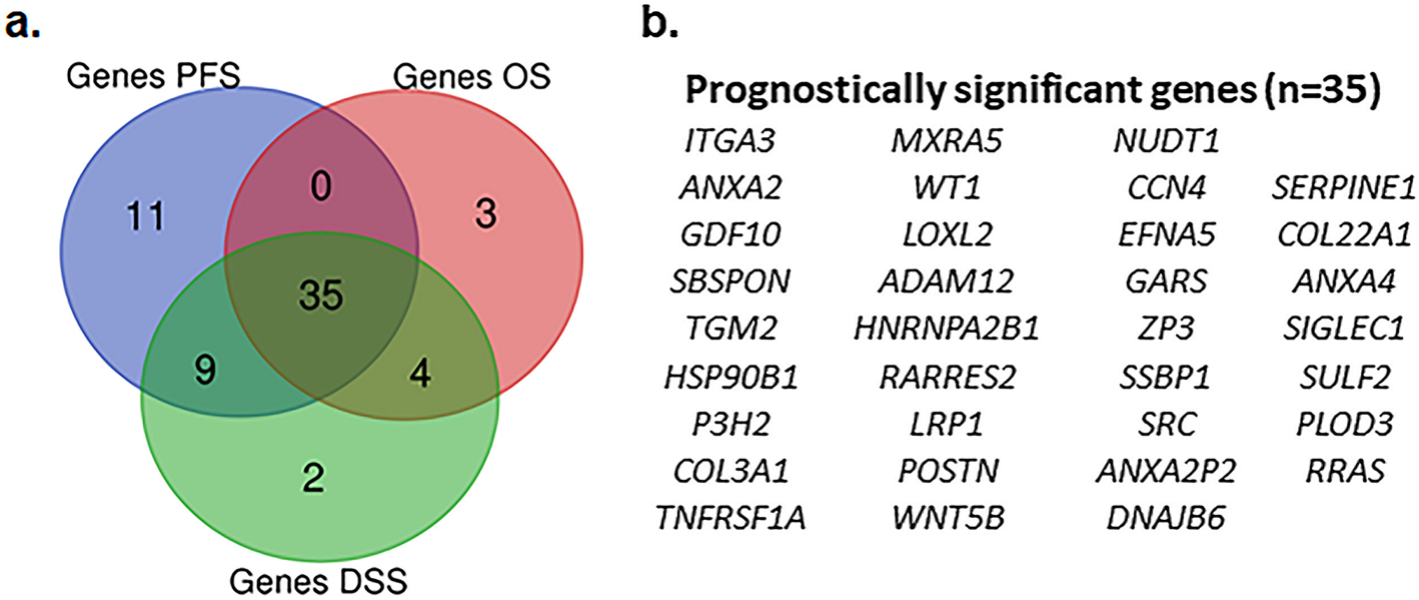
(**a**) Venn diagram of survival-related ECM genes in renal cancer in TCGA dataset (z score > 2). A total of 35 genes showed significant association with PFS, OS and DSS. (**b**) List of overlapping 35 genes that showed a significant association with survival. (patients with z > 2 score, *p* < 0.05).

**Figure 2:**
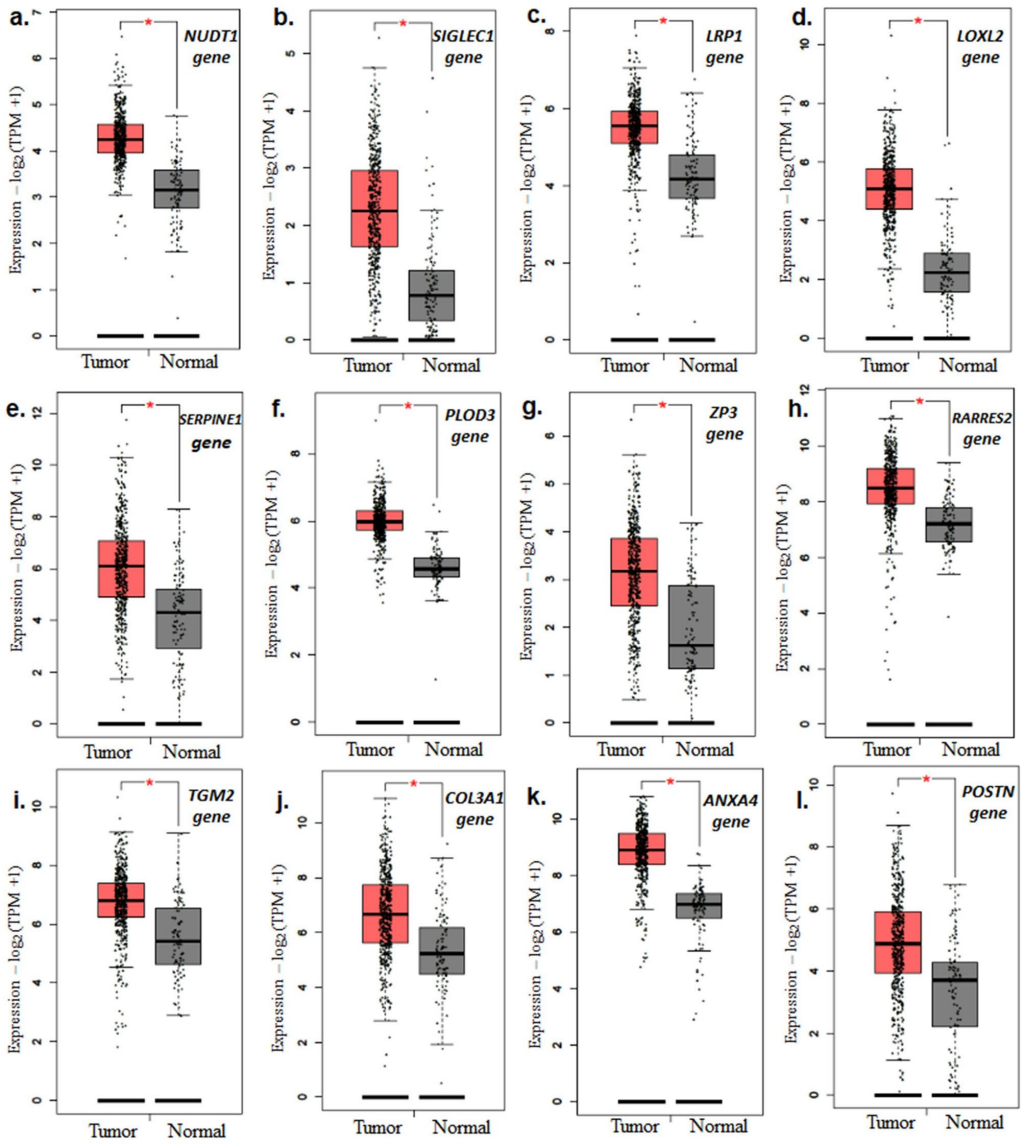
12 genes with higher expression in tumor (523 patients) compared to normal tissue (100 tissue). (**a**) *NUDT1*, (**b**) *SIGLEC1*, (**c**) *LRP1*, (**d**) *LOXL2*, (**e**) *SERPINE1*, (**f**) *PLOD3*, (**g**) *ZP3*, (**h**) *RARRES2*, (**i**) *TGM2*, (**j**) *COL3A1*, (**k**) *ANXA4*, and (**l**) *POSTN*.

**Figure 3:**
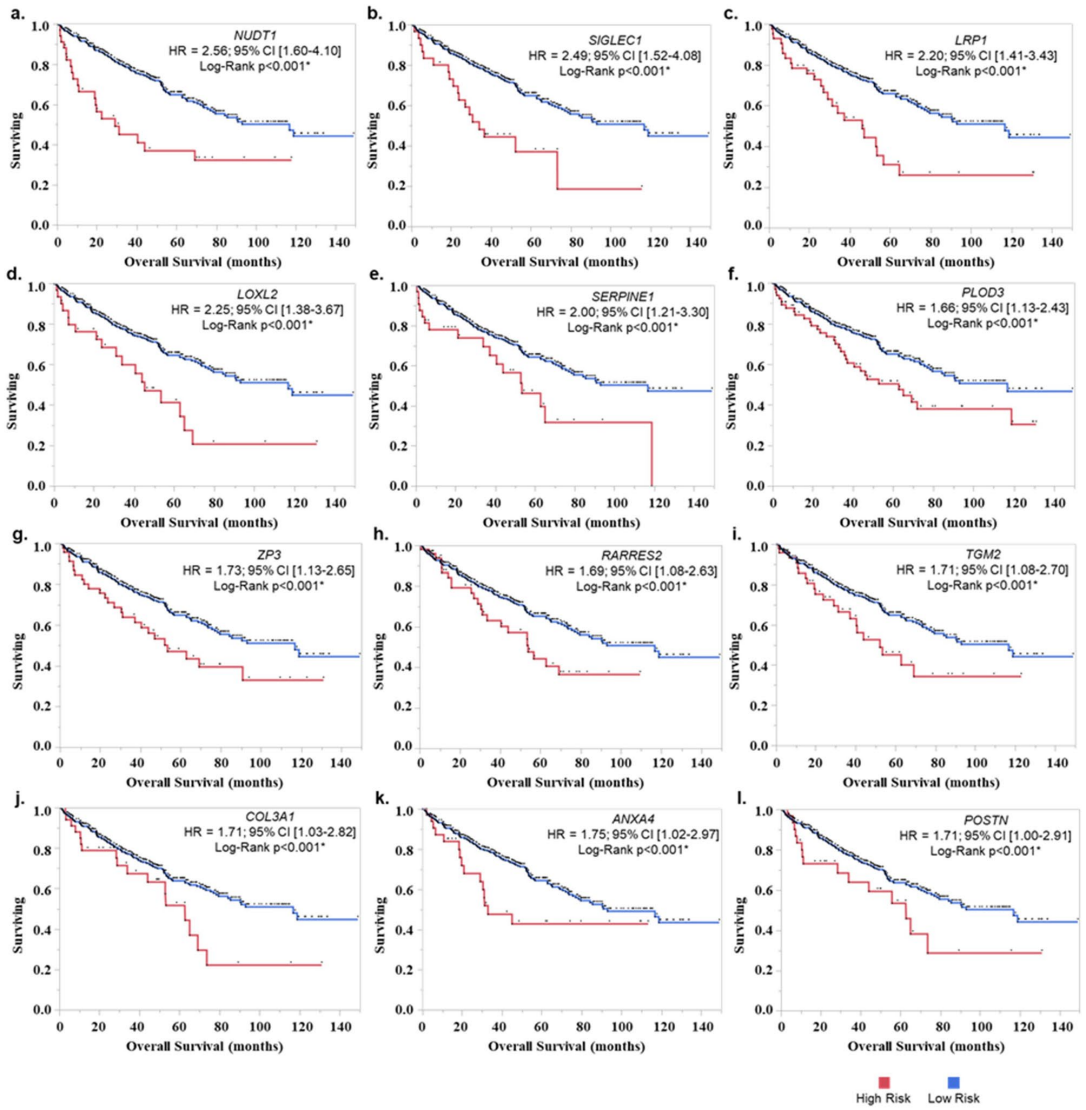
KM analysis of ECM genes with higher expression (z > 2 score) in RCC patients. (**a**) *NUDT1*, (**b**) *SIGLEC1*, (**c**) *LRP1*, (**d**) *LOXL2*, (**e**) *SERPINE1*, (**f**) *PLOD3*, (**g**) *ZP3*, (**h**) *RARRES2*, (**i**) *TGM2*, (**j**) *COL3A1*, (**k**) *ANXA4*, and (**l**) *POSTN*. The patients with higher expression (cutoff: z-score > 2) were grouped in high risk and other patients were in the low-risk group.

The survival analysis using cBioportal identified 106 ECM genes with higher prevalence in TCGA-KIRC patients (z-score > 2, prevalence > 5% of KIRC dataset (n = 510) (supp. Table 2). Among these 35 genes showed significant association with PFS, OS, and DSS (Fig. 1a,b and supp. Table 3). Out of these, 12 genes showed higher expression in ccRCC (Clear cell renal cell carcinoma) tumor compared to normal tissues (Fig. 2a–l). The gene products of these genes are found majorly in extra-cellular compartment and play an oncogenic role in several tumors (Table 1). Univariate and KM analysis identified patients with z > 2 score showed a significant association with a higher risk of mortality (Fig. 3a–l). Among these genes, *NUDT1* gene showed the highest risk with (H.R 2.56, 95% CI 1.60 – 4.10, *p* < 0.01*).

**Table 1:** Biological role of 12 genes.

Gene symbol	ECM conf. value	Role in tumorigenesis	References
*NUDT1* *(*Nudix Hydrolase 1*)*	3	NUDT1 enzyme removes oxidized nucleotide pools and prevents its subsequent misincorporation into DNA. Its higher expression has been associated with several cancers including renal cancer, brain, lung, and liver cancer	^13,14^
*SIGLEC1* (Sialic Acid Binding Ig Like Lectin 1)	5	SIGLEC1 expressing cells, like macrophages provide anchorage for metastatic cells. SIGLEC1 overexpression has been associated with poorer survival in several cancers	^15,16^
*LRP1* *(LDL Receptor Related Protein )*	3	LRP1 plays a significant role in the endocytosis of lipoproteins. LRP1 overexpression has been associated with invasion and migration of tumor cells	^17,18^
*LOXL2* *(Lysyl Oxidase Like 2)*	5	LOXL2 is part of extracellular enzyme family which cross-links elastin and fibrillar collagen. LOXL2 overexpression has been associated with several cancers including breast, gastric, colon, esophageal and pancreatic cancer	^19^
*SERPINE1* *(Serpin Family E Member 1)*	5	SERPINE1 is an inhibitor of urokinase and tissue plasminogen activator. It is associated with poorer prognosis in several cancer including stomach, head and neck, sarcoma, urothelial and testicular cancer	^20^
*PLOD3* *(Procollagen-Lysine*,*2-Oxoglutarate 5-Dioxygenase 3)*	5	PLOD enzymes perform Lysyl hydroxylation of collagen. PLOD3 also performs glycosylation activity of collagen hydroxylysines. Higher expression of PLOD3 has been associated with several cancers	^21,22^
*ZP3* *(Zona Pellucida Glycoprotein 3)*	5	ZP3 was found to be part of core secretome signature across multiple cancer types	^23^
*RARRES2* *(Retinoic Acid Receptor Responder 2)*	5	RARRES2 is secreted ligand for CMKLR1 expressing immune cells. Its expression is associated with the chemoattraction of immune cells	^24^
*TGM2 (Transglutaminase 2)*	5	TGM2 assists in posttranslational modification of proteins. Its overexpression is involved in several cancers including pancreatic, prostate, ovarian and breast cancer	^25^
*COL3A1* *(Collagen Type III Alpha 1 Chain)*	5	COL3A1 expresses components of fibrillar collagen which forms the skin and other tissues. Its over-expression in involved in multiple cancers	^26^
*ANXA4* *(Annexin A4)*	4	Annexin A4 is calcium-regulated phospholipid-binding protein. ANXA4 upregulation is involved in multiple cancers and is a biomarker for drug resistance	^27,28^
*POSTN* *(Periostin)*	5	POSTN is a multimodular protein which interacts with ECM proteins such as tenascin C, collagen, and fibronectin. Its expression is deregulated in multiple pathologies including inflammation and malignant transformation	^29^

The ECM confidence value ranged from 0 (absence of any extracellular evidence), 1 (low confidence of extracellular evidence) to 5 (highest confidence of extracellular evidence). Cellular localization of these genes was incorporated from Genecards (https://www.genecards.org/).

### Development of ECM signature with clinical application and its validation

**Figure 4:**
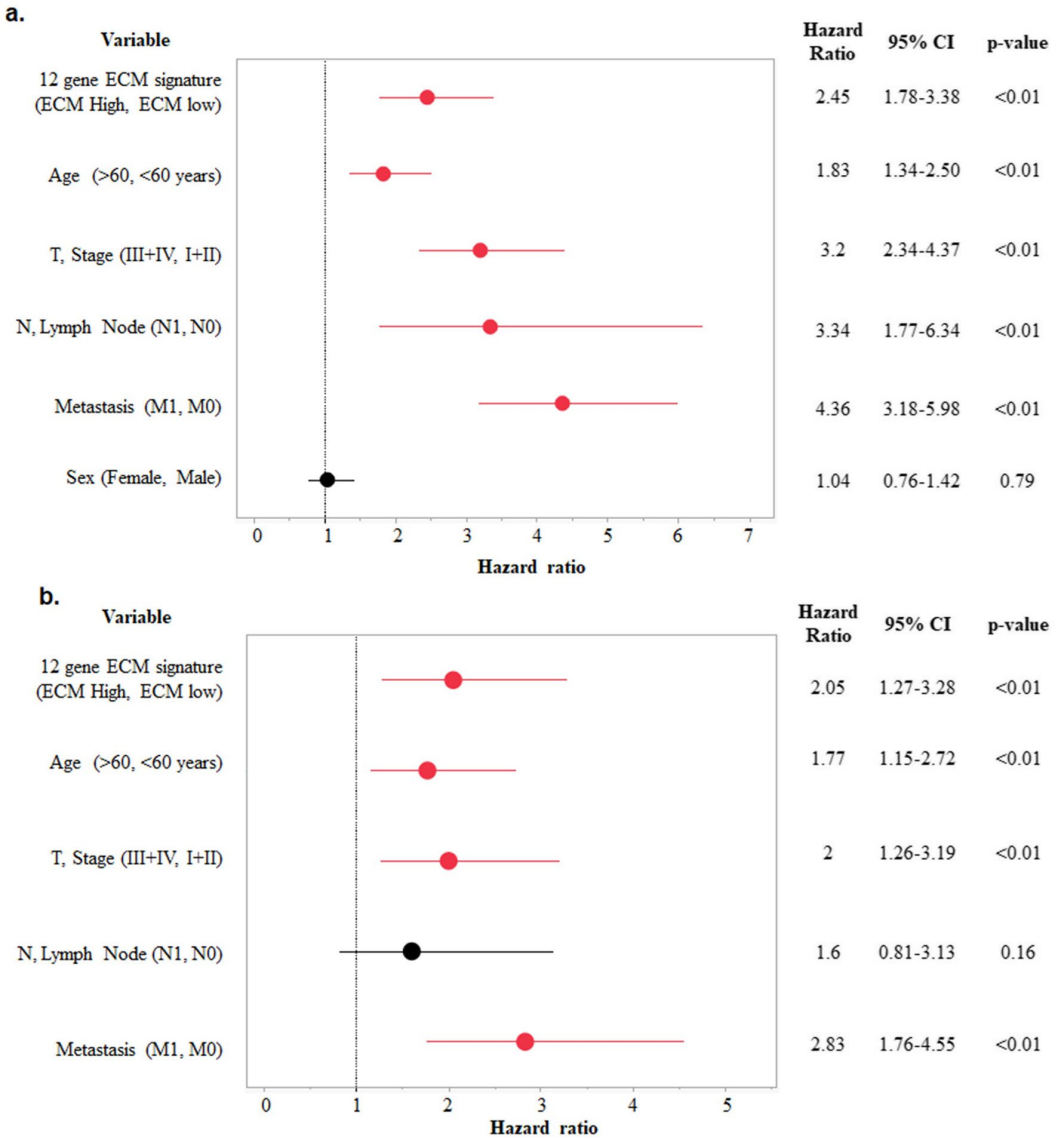
(**a**) Univariate and (**b**) Multivariate cox regression analysis of 12 gene ECM signature with age, Stage, N, Metastasis, and sex.

**Figure 5:**
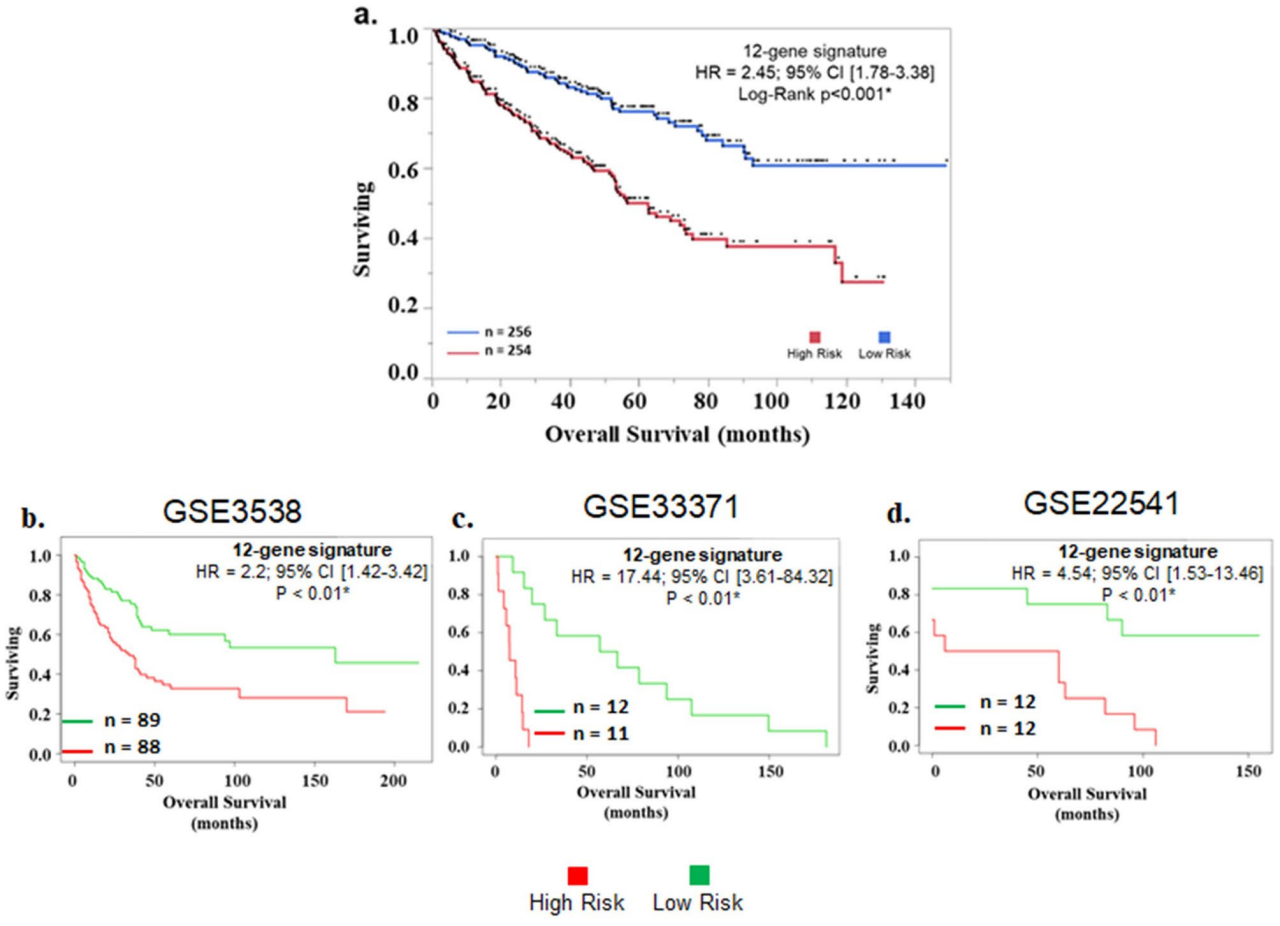
Survival analysis of 12 gene signature with kidney renal cell cancer patients in (**a**) TCGA dataset, (**b**) GSE3538, (**c**) GSE33371, and (**d**) GSE22541. Patients were divided using median risk-score value as cut-off dividing in high-risk and low-risk subgroups.

The TCGA-KIRC cancer cohort (n = 510) was used to assess risk based on Cox proportional hazard model. The survival risk scores were split at median (cut-off = 1.34) to divide patients into low-risk group and high-risk group ( range: 0.18 to 5.90). In univariate Cox proportion hazard analysis, the high-risk group was associated with worse survival (H.R 2.45, 95% CI 1.78–2.45, *p* < 0.01*). Other significant variables associated with poorer survival were higher age (H.R 1.83, 95% CI 1.34 – 2.50, *p* < 0.01*), advanced stage (H.R 3.2, 95% CI 2.34 – 4.37, *p* < 0.01*), lymph node involvement H.R 3.34, 95% CI 1.77 – 6.34, *p* < 0.01*), and metastasis (H.R 4.36, 95% CI 3.18 – 5.98, *p* < 0.01*) (Fig. 4a). In multivariate Cox proportion hazard only four variables showed association with poorer survival:—Higher risk (H.R 2.05, 95% CI 1.27–3.28, *p* < 0.01*), higher age (H.R 1.77, 95% CI 1.15 – 2.72, *p* < 0.01*), advanced stage (H.R 2, 95% CI 1.26 – 3.19, *p* < 0.01*), and metastasis (H.R 2.83, 95% CI 1.76 – 4.55, *p* < 0.01*) (Fig. 4b). Further, KM analysis significantly differentiated patients based on overall survival (Fig. 5a).

For external validation, this 12 gene signature was validated in 3 independent GEO datasets. GSE3538 (177 patients) HR = 2.2; 95% CI [1.42–3.42; *p* < 0.01*, GSE33371 (23 patients) HR = 17.44; 95% CI [3.61–84.32; *p* < 0.01*, GSE22541 (24 patients) HR = 4.54; 95% CI [1.53–13.46]; *p* < 0.05* (Fig. 5b–d). χ2 test between high and low risk categorical variables found significant association with ethnicity, stage, metastasis, aneuploidy score, fraction genome altered, OS status, PFS status and DSS status (Table 2).

**Table 2:** Comparison of clinicopathological features of patients with enriched ECM and low ECM gene signature using Pearson’s chi-square analysis (TCGA-KIRC dataset).

Clinical parameter	High ECM (n = 255)	Low ECM (n = 255)	Pearson χ2 p-value	Clinical parameter	High ECM (n = 255)	Low ECM (n = 255)	Pearson χ2 p-value
**Age**			1	**Fraction genome altered**			< 0.01*
< 66 years	128	128		< 0.120	108	150	
> 66 years	127	127		> 0.120	147	105	
**Ethnicity**			< 0.01*	**OS**			0.05
African American	36	19		< 5 years	191	171	
Caucasian	213	227		> 5 years	64	84	
**Sex**			0.51	**OS status**			< 0.01*
Female	89	96		Living	150	192	
Male	166	159		Deceased	105	63	
**Stage**			< 0.01*	**PFS**			< 0.02
I + II	132	171		< 5 years	206	185	
III + IV	123	84		> 5 years	49	70	
**Lymph Node**			0.059	**PFS Status**			< 0.01*
N0	117	111		No progression	153	199	
N1	12	4		Progression	102	56	
**Metastasis**			< 0.01*	**DSS**			0.05
No Metastasis M0	188	213		< 5 years	191	171	
Metastasis M1	51	27		> 5 years	64	84	
**Aneuploidy score**			< 0.01*	**DSS status**			< 0.01*
> 4	143	81		Tumor free	172	221	
< 4	112	174		Dead with tumor	76	31	

### Differentially expressed genes (DEGs) in high and low-risk patients

**Figure 6:**
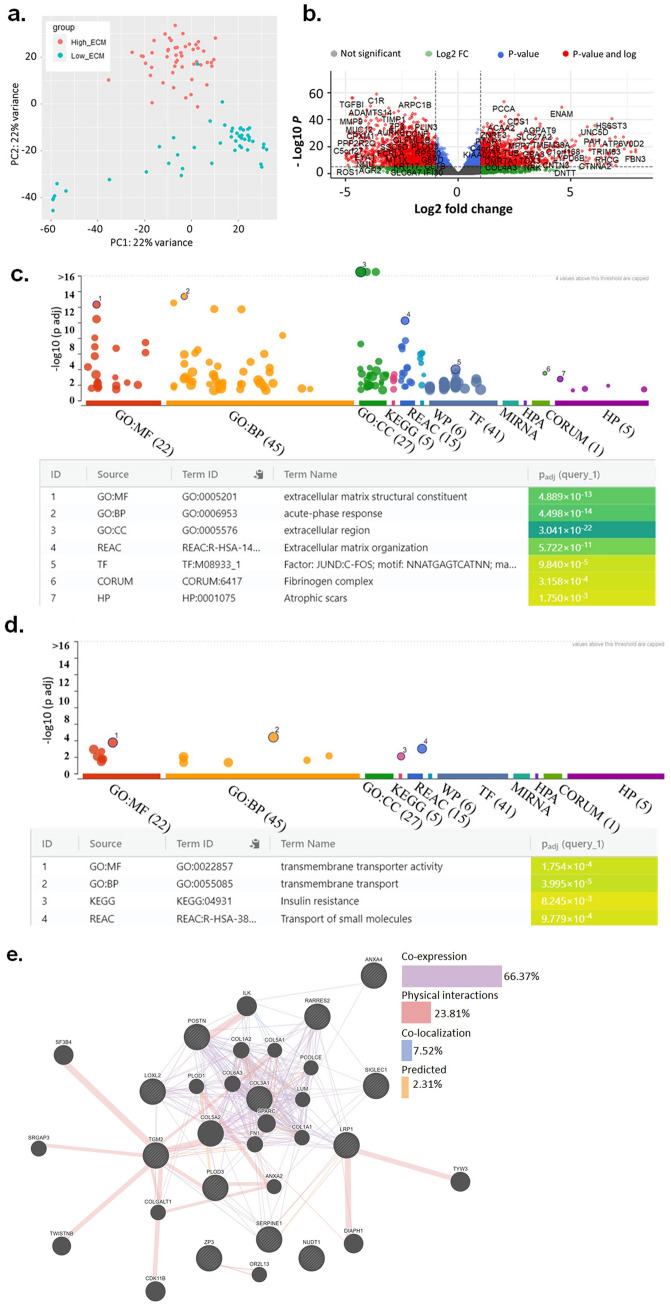
(**a**) Principal component analysis (PCA) showing distinct clustering between RNA-seq expression of high CDI patients (topmost 50 patients) compared to low CDI patients (bottom 50 patients). (**b**) Volcano plot showing differential expression of genes between High CDI and low CDI (*p* < 0.01, log2 fold-change > 2). (**c**) Functional enrichment analysis of highly expressed genes (log2 fold-change > 2) in (**c**). High ECM group with most hits for extracellular matrix and (**d**) Low ECM group with maximum hits for transmembrane transport activity. (**e**) GeneMANIA gene–gene interaction network showed multiple connections, pre-dominantly co-expression and physical interaction among genes in prognostic signature.

DEseq2 analysis was performed to screen differentially expressed genes between high and low-risk patients’ group (supp Table 4). Among 20,501 protein-coding genes, 1818 genes were upregulated at > twofold in high-risk patients compared to lower risk group. In the low-risk group, a total of 159 genes were upregulated at > twofold compared to the high-risk group (supp Table 4). Principal component analysis (PCA) showed a clear separation between the high-risk group and low-risk group based on ECM signature (Fig. 6a). Volcano plot of differentially expressed genes between high-risk and low-risk patients is depicted in Fig. 6b. The enriched gene ontology terms in high ECM group were extracellular matrix structural constituent and acute phase response pathways (Fig. 6c). The enriched gene ontology terms in low ECM group were transmembrane transporter activity and transport (Fig. 6d). Further network analysis using GENEmania portal revealed co-expression based on protein–protein interaction to be high (66.37%) & the physical interaction was at 23.81% (Fig. 6e).

### Key pathways associated with high and low-risk patients using Gene Set Enrichment Analysis

**Figure 7:**
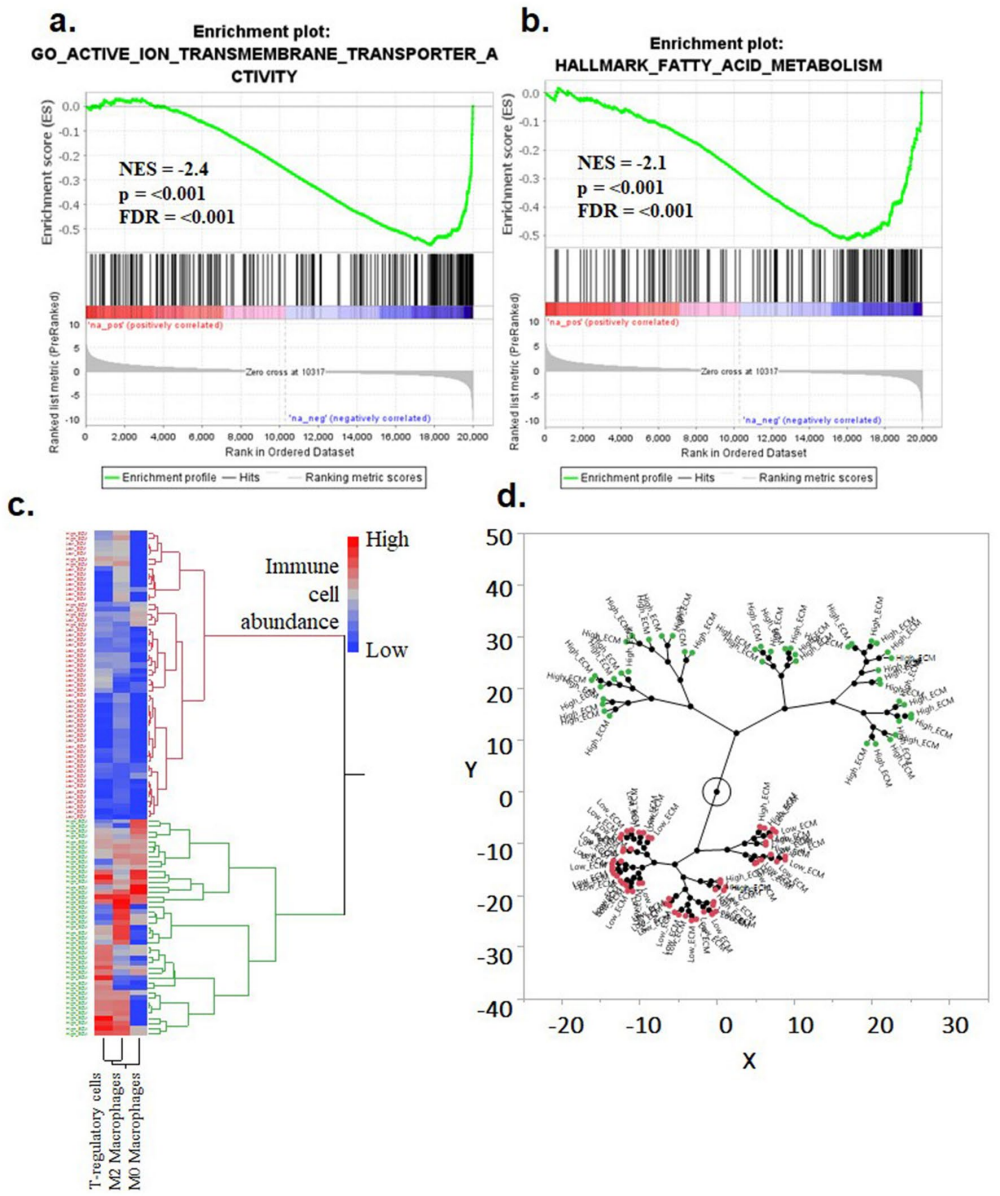
GSEA analysis of the differentially expressed genes between the two risk groups. (**a**) Pathways enriched in the low-risk group were (**b**). Active ion transmembrane transporter and (**b**). Hallmark of fatty acid metabolism. (**c**) Hierarchical tree structure and (**d**) constellation plot classifying the high-risk patients according to correlation with tumor-promoting immune cell infiltration.

GSEA analysis was performed to study two risk clusters in greater resolution. The analysis indicated significant perturbations of molecular pathways in the high-risk group. The pathways with highest normalized enrichment scores (NES) in the low risk ccRCC patients were GO active ion transmembrane transporter (NES = -2.4, FDR =  < 0.001), Hallmark fatty acid metabolism gene signature (NES = -2.1, FDR =  < 0.001), (Fig. 7a,b). Among other pathways were GO active transmembrane transporter activity (NES = -2.38), GO secondary active transmembrane transporter activity (NES = -2.27) and Hallmark oxidative phosphorylation (NES = -2.07) (suppl tables 5, 6). Interestingly, patients in low risk were enriched in transportation-related kidney pathways, whereas these homeostatic pathways were absent in high-risk patients which included diverse pathways with low NES such as Extracellular matrix (NES = 0.64), Hallmark Epithelial-mesenchymal transition (NES = 0.69) and Hallmark allograft rejection (NES = 0.63).

### Correlation analysis of ECM gene signature and infiltration of immune cells

To analyze the distribution of immune cells in these two risk groups, CIBERSORT-ABS algorithm was utilized (suppl Fig. 1). It identified patients in high risk with higher abundance of T regulatory cells, M0 and M2 macrophages (Fig. 7c). Although tumor-promoting immune features were found to be correlated with the high-risk group there was no association of inflammatory immune cells with any group (suppl Fig. 2). The constellation plot showed 2 distinct clusters depicting high-risk ECM rich patients and low-risk patients (Fig. 7d).

## Discussion

Extracellular matrix (ECM) has been commonly viewed as inert scaffolding for growth and development of tissues^30^. This view is rapidly being revised due to its emerging active roles in health and disease^31^. ECM plays an important role in maintaining structural integrity and amplification of growth signals by providing binding sites for various growth factors. For instance, FGF (fibroblast growth factors) and VEGF (vascular endothelial growth factors) have dual binding sites for cell adhesion and growth factors^32^. This localization of growth factors plays a critical role in the maintenance of gradients, which is essential in cell signalling. Further, the extracellular matrix and its perturbation plays a significant role in tumor invasion and metastasis especially in solid cancer^31^. In cancer, epithelial cells can remodel ECM to pro-tumorigenic tumor microenvironment through activation of stromal cells^33^. This transition of normal ECM due to altered expression of ECM genes can assist in prognostication of renal cancer. In the present study, the expression of ECM-related genes was explored to develop a prognostic signature using TCGA and other independent gene expression datasets.

Apart from the critical role of ECM plasticity in the spread of cells and its malignant transformation, it has important immunological implications as well^34,35^. It has been shown that ECM can negatively affect the infiltration and distribution of immune cells such as CD8 + T cells and NK cells in solid tumors^36,37^. Abnormal ECM components can alter mechanical and biochemical properties of TME which can affect immune cells function^38^. Additionally, immunosuppressive molecules such as IL-10 and TGF-β accumulate in ECM rich tumor partly due to low diffusion and buildup of hypoxic and metabolic stress^37,39^. The deposition of ECM components can lead to accumulation of immunosuppressive T-regulatory cells and M2 polarized macrophages^8^. Thus, variations in ECM components can affect distribution, activation, and polarization of immune cells.

In the current study, we explored the prognostic potential of genes related to ECM remodelling and its correlation to immune cell infiltration in ccRCC. We have identified 12 ECM gene signatures in ccRCC with prognostic significance in multiple datasets. Before this study, a comprehensive analysis of ECM genes to identify prognostic gene signature and its immune characterization was lacking. In this study, we have addressed the issue by exploring 964 ECM related genes and developed a 12 gene prognostic signature which was derived from TCGA and validated using independent GEO datasets. Further, these 12 genes were found to be tumorigenic as their expression levels were found to be significantly higher levels in renal cancer compared to normal tissue. Secretion of ECM modifying enzyme families such as LOX, PLOD, and collagen among others can lead to higher invasiveness, migration, proliferation, and survival of cancer cells^35^. The protein products of this gene signature are pre-dominantly secreted in extra-cellular spaces and play tumorigenic role. Further, network analysis of this prognostic disease module identified higher co-expression of these genes. The protein co-expression networks are generally used to identify genes which are functionally relevant and are under similar transcriptional program^40^. The expression level of these genes has been found to be similar across several conditions which is indicated by its higher co-expression (> 66.37%). In a separate study, high ECM gene expression was found to be prognostic for breast cancer^41^ and gastric cancer^42^. Interestingly one of the studies has identified correlation of aggressive ECM characteristics with immunosuppressive features in glioblastoma^43^. In renal cancer, gene like collagen 1 has been explored in enhanced metastasis and invasion in RCC^44^. Another study has identified a higher expression of 10 genes associated with cell adhesion and ECM regulation in renal cancer^45^.

In GSEA analysis, the patients in the high-risk group showed significant perturbation in normal homeostasis kidney function such as active ion transportation and fatty acid metabolism. These functions are known to be associated with kidney functions and renal epithelial cells utilize fatty acids are the source of energy^46^. One of the striking features in low-risk group was the preservation of kidney function genes. Chronic kidney disease (CKD) and lower estimated glomerular filtration rate (eGFR) is associated with increased risk of renal cancer^47^. Further, the clinical management of ccRCC patients includes maximization of kidney function preservation and management of long-term CKD^48^. This gene signature identified patients with high ECM at a higher risk of renal complications compared to the low-risk patient group.

Additional analysis of immune abundance using CIBERSORT-ABS revealed that T-regulatory, M2 macrophages and M0 macrophages were found to be higher in high-risk patients. In tumor microenvironment, T-reg and suppression of the immune system plays a significant role in progression of cancer^49^. Recently, high infiltration of M2 macrophages and FOXP3 + T-regulatory cells was associated with adverse clinical outcome in renal carcinoma^50^. The combination of anti-CTLA-4 and anti-PD-1 in a CheckMate 214 trial, was found to achieve objective response rate in only 40% of patients^51^. In our study, the association of M2 macrophages and T regulatory cells in ECM high-risk patients represent a risk population which might not benefit from these immunotherapies. These patients can be targeted for emerging novel therapeutic approaches currently under investigation such as T-reg depletion strategies or anti-FoxP3 Treg vaccines^52^.

Further, expanded knowledge of variables in ECM distribution can illuminate 2 critical aspects of novel immunotherapeutic approaches: its design and application. First, a recent study has identified that targeted blocking of ECM molecules can lead to a response in immune-refractive patients. In this study, integrin αvβ6/8-specific monoclonal antibody (mAb) led to higher survival in TNBC metastatic mouse models which responded poorly to PD-1 blockade^53^. So, direct inhibition of specific ECM components can lead to higher survival in cancer models. Secondly, understanding of physical and biochemical constraints posed by ECM can assist in generating better ways to deliver novel drugs to the tumors. Abnormal ECM matrix leads to poor diffusion of drugs, nutritional supply, and immune mediators^37^. Active ECM modulation has shown promising response to the efficacy of chemotherapeutic regimes in various models. Inhibition of LOX gene led to an improved response to chemotherapy in tumor models^54^. Comparatively, for an effective response to an immunotherapy regime, there is an addition of another variable i.e., immune cells. Immune modulators (mAbs) and immune cells are required to be in proximity with tumor for an effective response, but the ECM is known to interfere with both. Physically, ECM prevents interaction between immune cells such as T cells and tumor cells^55^. Further, ECM also negatively affects the diffusion of immunotherapy molecules in tissues. Dense, highly cross-linked, and stiff ECM matrix interferes with the diffusion of immunomodulatory drugs like ipilimumab (anti-CTLA-4) due to their larger hydrodynamic diameter^37^. Further, novel ECM-targeting drugs such as collagenase-infused nanogels can penetrate ECM-rich tumors and could be explored for such therapies^56^. Thus, further understanding of survival-related ECM genes in ccRCC and its effect on immune cells can assist in the design and application of novel therapeutics.

## Conclusion

In the current study, we developed a 12-ECM gene prognostic signature which was able to discriminate high-risk renal cell carcinoma patients from low-risk ones. Besides, this signature was validated in 3 independent datasets. Moreover, the tumorigenic role of the genes included in this signature showed elevated expression in tumor tissues compared to normal. PCA and differential gene expression analysis identified significant perturbation of normal homeostatic functions of high-risk patients. Further, immune-suppressive cells such as T-reg, M2 and M0 macrophages showed higher infiltration in high-risk patients. The prognostic signature and immune features can identify important sub-section of ccRCC patients which can benefit from emerging immunotherapies.

## Methods

### Data acquisition and pre-processing

A total of 964 extracellular matrix and remodeling genes were downloaded from Gene Ontology (http://geneontology.org)^57^ (suppl. Table 1). The gene list was curated based on range of evidence for their involvement in the ECM pathway. The evidence annotation of these genes included were: ‘Inferred from Experiment (EXP)’, ‘Inferred from Direct Assay (IDA)’, ‘Inferred from Physical Interaction (IPI)’, ‘Inferred from Mutant Phenotype (IMP)’, ‘Inferred from Genetic Interaction (IGI)’ and ‘Inferred from Expression Pattern (IEP)’ (http://geneontology.org/docs/guide-go-evidence-codes/). This approach led to inclusion of only specific genes with evidence of their involvement in ECM interaction pathways. Transcriptome RNA-sequencing and clinical data of kidney renal cell carcinoma cancer (KIRC) patients (n = 510) was extracted from the TCGA data portal (https://portal.gdc.cancer.gov/). This dataset was used for GSEA analysis, immune cell infiltration analysis and clustering analysis. The clinical information of patients and z-scores of individual genes was downloaded from cBioportal (https://www.cbioportal.org/)^58^. The z-scores were divided into categorical variables (z-score > 2 and z-score < 2) for survival analysis. A total of 523 ccRCC cancer patients and 100 normal kidney tissues from GTEx portal was compared using GEPIA portal (http://gepia2.cancer-pku.cn/) ^59^. GEO (Gene expression Omnibus) datasets (GSE3538, n = 177, GSE33371, n = 23 and GSE22541, n = 24) were explored for validation of prognostic signature^60^.

### Identification of survival-associated ECM genes

964 extracellular matrix and remodeling genes were analyzed in cBioportal to quantify perturbations in TCGA—renal cell carcinoma (TCGA-KIRC RNA Seq V2, n = 510 patients) based on z score more than 2 as has been used previously to screen prognostic genes^61^.

### Comparison of prognostic-related genes between tumor and normal tissue

The expression of the genes associated with overall survival (OS) of ECM genes in ccRCC were compared to normal tissue using Gene Expression Profiling Interactive Analysis. For this study, the Kidney tumor (n = 523) dataset was compared against combined gene expression data of normal tissues from TCGA and Genotype-Tissue Expression (GTEx) data (n = 100), respectively. In GEPIA, parameters with Log2FC (Fold change) cutoff were set at 1 with a p-value cut-off at 0.01.

### Development of 12 gene ECM signature

Independent prognostic potential of 12 genes was analyzed in renal cancer (TCGA-KIRC-cBioportal) based on log-rank p-values. A total of 510 patients with RNA-Seq data were included in this analysis. KM curves with overall survival (OS) and Progression-free survival (DFS) were generated. OS shows total duration for which patients were alive from date of diagnosis. To develop a 12 gene signature, Cox regression model was developed that divided the cohort into low- and high-risk groups ECM risk score based on median as previously published^62–64^. Briefly, the prognostic score was calculated by multiplying the expression value of a gene with its corresponding Cox proportion regression coefficient (Prognostic score = Σ Cox regression coefficient of Gene_i_ * expression value of gene Gene_i_). For validation of gene signature, GEO (Gene expression Omnibus) datasets were explored GSE3538 (n = 177), GSE33371 (n = 23) and GSE22541 (n = 24) using SurvExpress tool (http://bioinformatica.mty.itesm.mx:8080/BiomatecSurvivaX.jsp). The microarray expression data used in these studies were acquired from Affymetrix Human Genome array [HG-U133_Plus_2, GPL platform].

### Biological role of 12 genes

To further evaluate the biological roles of 12 genes and analyze predominant cellular compartment of their expressed proteins, information was extracted from literature and genecards web portal (https://www.genecards.org/Guide/GeneCard#compartments). The confidence score at genecards web-portal was incorporated from literature, high throughput screen and similar text mining analyses. The ‘unified confidence score’ ranged from absence of localization evidence (value = 0) and range from low confidence (value = 1) to high confidence (value = 5).

### Identification of differentially expressed mRNAs

The differentially expressed genes (DEGs) were analyzed using DEseq2 package in R (http://bioconductor.org/packages/ release/bioc/html/DESeq2.html). The results were analyzed and interpreted in R using ‘plotPCA’ function for Principal component analysis and volcano plot was generated using ‘enhancedvolcano’ package (http://bioconductor.org/ packages/ EnhancedVolcano.html).

### GSEA (Gene Set Enrichment Analysis) and network analysis

To explore the pathways associated changes in patients with high-risk score, GSEA software was downloaded from the Broad Institute ( http://www.broadinstitute.org/gsea). For this analysis, patients with highest expression of ECM signature (top 50) were compared with patients with lowest expression (bottom 50). All genes were pre-ranked based on DESeq2-calculated log2 fold changes using R and target gene sets were “H: hallmark gene sets” and “C5.GO.MF.v7.2” with the number of permutations set at 1000. Enrichment statistic was set to ‘weighted’ and ‘Signal2Noise’ metric was used for ranking genes. H (hallmark gene sets, 50 gene sets), and C5 (ontology gene sets, 14,765 gene sets).

### Gene interaction network analysis

Network analysis was performed to identify the interactions between the three genes with highest log-fold change compared to tumor using g:profiler^65^. GeneMANIA was used to analyze the string network analysis (http://genemania.org/)^66^.

### Immune cell infiltration analysis

In this study, the immune cell infiltration landscape of high-and low- risk patients were obtained via CIBERSORT-ABS algorithm which quantifies absolute proportion of cells. Briefly, it is determined by calculating the median expression of all the genes in signature and is divided by gene expression level in whole sample^67^. The absolute scores calculated using this method can be used for both inter- and intra-sample comparisons.

### Clustering analysis

Hierarchal clustering of correlation between immune cells and risk groups was generated using ward’s methods. Hierarchal clusters and constellation plots were prepared using JMP-Pro (version 14.0.0, SAS Institute, Cary, USA).

### Kaplan–Meier survival analysis

The Log-rank test was used to compare the survival distribution using Kaplan–Meier analysis using JMP-Pro (version 14.0.0, SAS Institute, Cary, USA).

### Statistical analysis

The statistical significance of OS (Overall survival), DFS (Disease -free survival), PFS (Progression free-survival) and DSS (Disease-specific survival) was computed using Log-rank t-test through cBioportal. For Pearson's chi-square (χ2) test, the numeric values of the risk score were split at the median and divided in 2 groups for comparison of patients in high and low-risk groups. GEPIA web-portal utilizes one-way ANOVA for calculation of differential expression between tumor and normal. GeneMANIA utilizes Geno-Ontology based weighing method that assigns score based on biological processes behind the interacting genes. For Gene Set enrichment analysis, the normalized gene enrichment score was kept at greater than > 2 was considered strong. *p* values of < 0.05 were considered statistically significant.

## Supplementary Information

Supplementary Information 1.

Supplementary Information 2.

Supplementary Information 3.

Supplementary Information 4.

Supplementary Information 5.

Supplementary Information 6.

Supplementary Information 7.

Supplementary Information 8.

## Data Availability

The study utilized The Cancer Genome Atlas (TCGA) Program and Gene Expression Omnibus (GEO) repositories, which are freely available to the public.
