# Supplementary material for: Prognostic and therapeutic implications of extracellular matrix associated gene signature in renal clear cell carcinoma
Source: Sci Rep. 2021 Apr 7;11:7561. doi: 10.1038/s41598-021-86888-7 (PMC8026590; doi:10.1038/s41598-021-86888-7)
Supplement: Supplementary file 3 — Supplementary Information 3. [file 41598_2021_86888_MOESM3_ESM.pdf]

Prognostic and therapeutic implications of extracellular matrix associated gene signature in renal clear cell carcinoma

Pankaj Ahluwalia[1], Meenakshi Ahluwalia[1], Ashis K. Mondal[1], Nikhil Sahajpal[1], Vamsi Kota[2], Mumtaz V. Rojiani[1], Amyn M. Rojiani[1], and Ravindra Kolhe[1]*

[1] Department of Pathology, Medical College of Georgia, Augusta University, GA, U.S.A.
[2] Department of Medicine, Medical College of Georgia, Augusta University, GA, U.S.A.
[*] Correspondence: rkolhe@augusta.edu; Tel.: (706)-721-2771; Fax :(706)-434-6053

Supplementary Table 1: ECM genes analyzed in this study.

ECM2	*FIBCD1*	*CILP*	*S100A4*	*C1QC*	*PCSK6*	*APOD*	*CSGALNACT1*	*NOTCH1*	*FZD4*
FRAS1	*COL20A1*	*HMCN1*	*ANXA5*	*NCAN*	*ITGA6*	*FGFR4*	*BLOC1S1*	*ITGAM*	*FGF1*
ECM1	*LGALS4*	*COL28A1*	*ANXA2P2*	*ANGPTL4*	*TNR*	*L1CAM*	*MPV17*	*NFKB2*	*NF1*
MEPE	*TECTB*	*COL8A1*	*S100A6*	*INHBE*	*CTSG*	*VCAM1*	*ATXN1L*	*JAM2*	*BMP7*
MUC5B	*ZPLD1*	*COL5A2*	*ITIH2*	*ADAMTS18*	*TIMP1*	*UMODL1*	*SKP2*	*TFIP11*	*TEK*
BMPER	*CHADL*	*FMOD*	*LOXL4*	*MBL2*	*PLG*	*PECAM1*	*CER1*	*ITGAX*	*VEGFA*
VWF	*COL14A1*	*ADAMTS5*	*LAMB2*	*ADAMTS16*	*CTSL*	*CCN1*	*PTX3*	*ERO1A*	*APOE*
ZAN	*NYX*	*RTBDN*	*LUM*	*GPC5*	*ITGB6*	*CCN2*	*F7*	*GLRX2*	*WNT11*
FCGBP	*KAZALD1*	*COL16A1*	*POSTN*	*F13A1*	*PHOSPHO1*	*LAMP2*	*VCL*	*WNT4*	*RELN*
MUC6	*VIT*	*PRG2*	*GFUS*	*PAPLN*	*SERPINB5*	*SPINT1*	*RARRES2*	*GLUD1*	*WNT3A*
MUC2	*COL10A1*	*OGN*	*ADAMTSL2*	*ADAMTS7*	*CRTAP*	*NRROS*	*GH1*	*THY1*	*LRP5*
OTOGL	*COL4A4*	*EYS*	*FGL1*	*CFP*	*ACHE*	*MYO1E*	*ACTG1*	*JUP*	*FGF10*
SSPOP	*COL9A1*	*ACAN*	*CPA3*	*BCAN*	*SPOCK2*	*PRDX4*	*SPACA3*	*IQGAP1*	*LGALS9*
MUC5AC	*COL9A2*	*CLEC14A*	*ZG16*	*ITGB4*	*IDH2*	*NPHS1*	*ADGRG6*	*PTPRJ*	*CD4*
OTOG	*COL9A3*	*ADAMTSL4*	*TLL2*	*ANXA7*	*CTHRC1*	*FCN1*	*PKM*	*IFNA2*	*ERCC2*
FREM2	*COL19A1*	*TNC*	*TFPI2*	*ANXA4*	*ADAMTS1*	*LEFTY2*	*ADAM10*	*SMPD3*	*KARS1*
FREM1	*LAMA4*	*STATH*	*KLK4*	*ENTPD2*	*MYOC*	*SOST*	*FLOT1*	*CXCL12*	*SCX*
FREM3	*LAMB4*	*ADAMTSL1*	*EGFL7*	*SERPINB1*	*ITGB5*	*SEMA3B*	*TGFB1*	*ITGB2*	*CAV2*
FAM20C	*BCAM*	*ADAMTS14*	*SERAC1*	*CSTB*	*SERPINA5*	*ANGPT2*	*AGT*	*ANGPT1*	*FOXF1*
EFEMP1	*HAPLN2*	*HPSE2*	*C1QB*	*DMBT1*	*SLPI*	*MST1*	*UMOD*	*F2*	*CYP1B1*
EFEMP2	*ANGPTL6*	*SCARA3*	*AMELX*	*CLEC3B*	*THBS4*	*HSD17B12*	*TGFB1I1*	*CALR*	*SRC*
SULF1	*OIT3*	*ADAMTSL3*	*NTNG2*	*ITIH4*	*TGM2*	*GPC6*	*ZP3*	*SMAD3*	*ABL1*
SULF2	*GP2*	*TINAG*	*LAMA2*	*ORM1*	*ANXA6*	*SPP1*	*SPN*	*KDR*	*PTK2*
LACRT	*SMOC1*	*ADAMTS6*	*IGFBP7*	*ORM2*	*KRT1*	*P4HA1*	*S100A9*	*ITGA8*	*CX3CL1*
SOD3	*ADAMTS15*	*TLL1*	*ITGA2B*	*PHLDB2*	*SERPINA3*	*ERO1B*	*S100A8*	*HPSE*	*OGG1*
MGP	*TPSAB1*	*ADAMTSL5*	*LINGO2*	*SLK*	*SERPINA1*	*ICAM2*	*CTSH*	*LOXL3*	*TYMS*
IMPG2	*PRSS1*	*DGCR6*	*LRRC24*	*PLET1*	*AZGP1*	*TST*	*APOA4*	*TSC1*	*CPS1*
MXRA5	*LYPD5*	*COL4A2*	*CCN5*	*DDR1*	*SERPINB6*	*GAS2*	*PLSCR1*	*TGFBR3*	*DNMT3A*
IMPG1	*NID1*	*PXDN*	*LRRN1*	*CLASP1*	*CTSV*	*MRPL18*	*B4GALT1*	*CAT*	*TENM1*
MATN1	*PODN*	*COL18A1*	*LRRN3*	*LAMA1*	*ITGAL*	*RIC8A*	*F11R*	*IL6*	*SOD1*
MMP23B	*MFAP5*	*HSPG2*	*LRIG1*	*ITGA7*	*LMAN1*	*LOX*	*CD47*	*CD200*	*TLR2*
MMP20	*MATN3*	*LAMB1*	*THBS2*	*RTN4RL2*	*GPC4*	*LOXL2*	*CDH1*	*PTN*	*TLR3*
MMP21	*RBP3*	*COL11A1*	*SRPX*	*VASN*	*CTSD*	*CASK*	*SLC12A2*	*BMP2*	*SHH*
MMP19	*EDIL3*	*SNED1*	*VWC2*	*DAG1*	*WNT5B*	*BCKDHB*	*JAM3*	*SFRP2*	*FGFR2*
MMP28	*SNORC*	*THBS3*	*ENAM*	*HAS3*	*CTSB*	*SIGLEC1*	*CD34*	*GPC3*	*ATP7A*
MMP24	*LYPD3*	*IGFALS*	*TNFRSF11B*	*WNT2B*	*PRTN3*	*RPSA*	*S100A7*	*CDH2*	*WNT5A*
MMP17	*COCH*	*CAPNS1*	*PLEKHA2*	*MUC3A*	*AMBP*	*WNT6*	*F3*	*GAS6*	*SNW1*

MMP27	*COL15A1*	*KLK2*	*ITGB7*	*VTN*	*F12*	*ADGRG1*	*TIMP3*	*CIB1*	*ABAT*
MMP26	*CCDC80*	*EMILIN1*	*HNRNPM*	*FAP*	*DEFA1*	*FURIN*	*WNT2*	*PSMA6*	*NOS2*
MMP16	*VWA1*	*LAMC2*	*FBN1*	*GPC1*	*FN1*	*GDF15*	*SEMA7A*	*BCR*	*PRKCD*
MMP15	*COL13A1*	*COLQ*	*ANTXR1*	*CD151*	*FGG*	*BSG*	*IL7*	*NUMA1*	*RPS3*
MMP25	*COL17A1*	*ADAMTS3*	*MUC17*	*CLC*	*GLG1*	*HPN*	*ANGPT4*	*RUVBL2*	*CDK1*
COMP	*COL5A3*	*VCAN*	*UTRN*	*P3H2*	*SPON2*	*CAPN2*	*PKHD1*	*CSNK2B*	*ROCK1*
MMP9	*HAPLN3*	*LRIG3*	*ADAM15*	*FKBP10*	*RTN4RL1*	*TNFRSF1A*	*LRP1*	*CCL21*	*NRP1*
MMP14	*LRRTM4*	*ICAM4*	*CTSS*	*RELL2*	*GP1BA*	*BST1*	*CCN3*	*ENG*	*CD36*
MXRA7	*LRRN2*	*SSC5D*	*COL2A1*	*AGRN*	*FGA*	*HRG*	*ITGB1*	*PKD1*	*FGF2*
MMP10	*COL22A1*	*DSPP*	*COL7A1*	*SPARC*	*ITGA4*	*SERPINF2*	*LRRTM1*	*HSPD1*	*FOXC1*
MMP7	*EGFL6*	*ADAMTS2*	*COL4A3*	*CEACAM6*	*DCN*	*FLRT1*	*FLRT3*	*SLC25A5*	*C1QBP*
MMP13	*LAMC1*	*ADAMTS8*	*HTRA1*	*ADTRP*	*SH3PXD2A*	*LRRC32*	*LRIG2*	*UQCC2*	*PDGFRA*
MMP11	*BGN*	*WDR72*	*A2M*	*AHCTF1*	*POMZP3*	*COLEC12*	*CCBE1*	*ARL2BP*	*FOXC2*
MXRA8	*ADAMTS19*	*NTN5*	*FBLN1*	*ALDH1L2*	*OC90*	*TIMP4*	*RECK*	*ARL2*	*NR2E1*
DMP1	*ADAMTS17*	*ADAMDEC1*	*LAMA5*	*TOMM70*	*VIPAS39*	*PI3*	*CCN4*	*RAC1*	*FOXO3*
MMP1	*TPSB2*	*ADAM11*	*LTBP1*	*MPST*	*NDUFAF7*	*TNFRSF1B*	*SPINT2*	*PRKCZ*	*WT1*
UCMA	*MFAP4*	*FGFBP3*	*PLOD3*	*GSR*	*MTHFD2*	*SH3PXD2B*	*PF4*	*GPLD1*	*RHOA*
MMP2	*COL27A1*	*ATRNL1*	*RRAS*	*SSBP1*	*MFAP1*	*NCAM1*	*HADH*	*BCAS3*	*FER*
MMP3	*COL11A2*	*CAPNS2*	*AMTN*	*ECH1*	*PMPCA*	*CHI3L1*	*SEMA3E*	*SPINK5*	*NLGN1*
MMP8	*COL4A6*	*A1BG*	*TMEFF1*	*FH*	*P3H4*	*SERPING1*	*FAM107A*	*DMTN*	*TIAM1*
HPX	*COL8A2*	*SERPINB8*	*EXOC8*	*PEX1*	*TMEFF2*	*SERPINB9*	*WNT3*	*ANXA1*	*APP*
MMP12	*COL4A5*	*OMD*	*NTN3*	*ALDH7A1*	*SDC2*	*CTSC*	*ACAT1*	*CSF1*	*LRRK2*
FAM20A	*MIA*	*FCN2*	*ZP2*	*ITGA2*	*APLP1*	*HSP90B1*	*CAD*	*PDGFB*	*PARK7*
C17orf58	*NTN4*	*ATRN*	*ITGA9*	*HAS1*	*ANXA8*	*KNG1*	*GREM1*	*ADIPOQ*	*TLR4*
ABI3BP	*LAMC3*	*PZP*	*KIF9*	*CTSK*	*USH2A*	*SERPINF1*	*EFNA5*	*TGFB2*	*PTEN*
PODNL1	*SPARCL1*	*ITIH1*	*FLG*	*KLK7*	*SPP2*	*CSPG4*	*HMGCL*	*ADAM8*	*CTNNB1*
SBSPON	*ADAMTS10*	*ANXA11*	*SDC3*	*PRG3*	*DST*	*APOC3*	*ANXA2*	*GSTP1*	*BAX*
MARCOL	*PRELP*	*CTSF*	*NOXO1*	*PTPRZ1*	*ADAMTS12*	*AHSG*	*CDON*	*SOX9*	*ERBB4*
ANGPTL5	*LAMB3*	*LGALS3BP*	*LMAN1L*	*PLAU*	*ANGPTL3*	*CTSZ*	*WASHC1*	*JAG1*	*TGFBR1*
OLFML2A	*TGM4*	*F9*	*ITIH5*	*NPNT*	*TNN*	*APOH*	*RAMP2*	*CD3E*	*SRF*
COL6A5	*ANGPTL1*	*NTNG1*	*MADCAM1*	*RIC1*	*FGB*	*APCS*	*TMEM38B*	*EDA*	*TNF*
AMELY	*CPN2*	*PCOLCE*	*ALPL*	*COL3A1*	*COL1A1*	*CD44*	*ERBIN*	*GNPAT*	*PTK2B*
EMILIN3	*TGFBI*	*MFGE8*	*BMP1*	*LRRC17*	*CD180*	*ITGB3*	*CREB3L1*	*ACAA1*	*TP53*
EMILIN2	*COL6A3*	*MMRN2*	*GSTK1*	*ELFN2*	*ADAM12*	*LGALS3*	*NTN1*	*HAT1*	*BCL2*
PRG4	*COL6A1*	*CS*	*SERPINH1*	*TRIL*	*LRRTM3*	*RGCC*	*TCF15*	*PCK2*	*CAV1*
COL6A6	*TUFT1*	*FBLN5*	*PRSS2*	*KERA*	*ELFN1*	*SDC4*	*SLC2A10*	*APOA1*	*SNCA*
C6orf15	*MPZL3*	*COL5A1*	*CCL28*	*LINGO1*	*HAS2*	*EPHX2*	*IHH*	*TGFB3*	
GFOD2	*HAPLN4*	*COL4A1*	*LYVE1*	*CCN6*	*TIMP2*	*CLIC4*	*IDE*	*EPHA3*	

NID2	*EMID1*	*LTBP3*	*BTD*	*CRISP3*	*CDH13*	*KRT8*	*CHMP1A*	*NPPA*	
LINGO4	*ST7*	*LAMA3*	*ALDH2*	*GPM6B*	*ITGA1*	*CD63*	*VDAC1*	*PDGFA*	
LRRC3C	*LTBP2*	*MATN2*	*GRHPR*	*P3H1*	*ACOT11*	*ACSM1*	*VIM*	*ALOX5*	
LINGO3	*ZP1*	*MMRN1*	*MDH2*	*CARMIL2*	*GARS1*	*GOT2*	*CFL1*	*SLC9A1*	
LRRC3B	*MEGF9*	*EGFLAM*	*CST3*	*TTR*	*SUCLA2*	*CRYM*	*ATP5F1B*	*DLG1*	
MATN4	*COL26A1*	*COLGALT<br>1*	*CPB2*	*PDPN*	*NUDT9*	*ACR*	*SHMT2*	*CLU*	
OPTC	*CRELD1*	*AMBN*	*SMOC2*	*DDR2*	*NAXE*	*ELANE*	*BASP1*	*SOD2*	
FBN3	*CBLN4*	*AEBP1*	*S100A10*	*ITGBL1*	*HSPE1*	*ADAM9*	*HSPA9*	*SLIT2*	
COL21A1	*ELN*	*COL1A2*	*OTOA*	*DEFB118*	*PPFIA2*	*SERPINE<br>2*	*FABP1*	*WNT7A*	
COL23A1	*CHAD*	*MELTF*	*STRCP1*	*EPDR1*	*FERMT3*	*MINK1*	*RIDA*	*ACTN2*	
COL24A1	*OTOL1*	*FSCN1*	*ITGB8*	*LCP1*	*CFL2*	*ETS1*	*SPTBN4*	*PDK2*	
TINAGL1	*ADAMTS4*	*ADAMTS2<br>0*	*KLKB1*	*SERPINC1*	*COASY*	*THBS1*	*ACTN3*	*SLC39A8*	
HAPLN1	*ANGPTL7*	*LOXL1*	*MYH11*	*QSOX1*	*TUFM*	*PSAP*	*ATP5F1A*	*ITGA5*	
TECTA	*PHLDB1*	*THSD1*	*DPP4*	*FLRT2*	*SERPINE1*	*ITGAV*	*RUVBL1*	*FOXF2*	
FBLN2	*ITGA10*	*IBSP*	*ICAM3*	*CAPN1*	*NBL1*	*CCL25*	*ACTN1*	*NOX1*	
DPT	*FBN2*	*ITGA11*	*ITGAE*	*ANOS1*	*DAND5*	*MYF5*	*HNRNPA2B<br>1*	*EXT1*	
VWA2	*SCUBE3*	*MUC4*	*MSLN*	*HDGF*	*TMPRSS6*	*DNAJC15*	*IDH1*	*ELF3*	
HMCN2	*CTRB2*	*ICAM5*	*ITGAD*	*GDF10*	*CBLN1*	*NUDT1*	*SFRP1*	*PDK4*	
LAD1	*CTRB1*	*SCUBE1*	*CLASP2*	*NDNF*	*PRDX5*	*OXCT1*	*FASTKD2*	*DNAJB6*	
ANGPTL<br>2	*COL25A1*	*CRISPLD2*	*LTBP4*	*GPC2*	*KLK5*	*FERMT1*	*HLCS*	*SIRT4*	
TNXB	*ASPN*	*CMA1*	*NDP*	*FCN3*	*PTPRA*	*APBB2*	*SIRT5*	*SIRT3*	
COL12A1	*MFAP2*	*SPON1*	*SRPX2*	*WNT8A*	*ITGA3*	*HSPA1L*	*OTC*	*MDK*	
THSD4	*LRRC15*	*ADAM19*	*ADAMTS1<br>3*	*SERPINB1<br>2*	*FGL2*	*POMT1*	*ICAM1*	*CFLAR*	
COL6A2	*CD248*	*HRNR*	*ADAMTS9*	*C1QA*	*LGALS1*	*TXN2*	*ANG*	*RB1*	
