# Supplementary material for: Prognostic and therapeutic implications of extracellular matrix associated gene signature in renal clear cell carcinoma
Source: Sci Rep. 2021 Apr 7;11:7561. doi: 10.1038/s41598-021-86888-7 (PMC8026590; doi:10.1038/s41598-021-86888-7)
Supplement: Supplementary file 4 — Supplementary Information 4. [file 41598_2021_86888_MOESM4_ESM.pdf]

Prognostic and therapeutic implications of extracellular matrix associated gene signature in renal clear cell carcinoma

Pankaj Ahluwalia[1], Meenakshi Ahluwalia[1], Ashis K. Mondal[1], Nikhil Sahajpal[1], Vamsi Kota[2], Mumtaz V. Rojiani[1], Amyn M. Rojiani[1], and Ravindra Kolhe[1]*

[1] Department of Pathology, Medical College of Georgia, Augusta University, GA, U.S.A.
[2] Department of Medicine, Medical College of Georgia, Augusta University, GA, U.S.A.
[*] Correspondence: rkolhe@augusta.edu; Tel.: (706)-721-2771; Fax :(706)-434-6053

Supplementary Table 2: Alterations of ECM genes using cBioportal in TCGA KIRC dataset (n = 510).

Gene Symbol	OQL Line	Num Samples Altered	Percent Samples Altered
PLOD3	PLOD3: EXP>=2;	65	13.00%
VDAC1	VDAC1: EXP>=2;	56	11.00%
DNAJB6	DNAJB6: EXP>=2;	51	10.00%
CCL28	CCL28: EXP>=2;	50	10.00%
RARRES2	RARRES2: EXP>=2;	48	9.00%
TGFBI	TGFBI: EXP>=2;	47	9.00%
ZP3	ZP3: EXP>=2;	47	9.00%
MMP24	MMP24: EXP>=2;	46	9.00%
TGM2	TGM2: EXP>=2;	46	9.00%
SSBP1	SSBP1: EXP>=2;	44	9.00%
ALDH7A1	ALDH7A1: EXP>=2;	44	9.00%
GARS	GARS: EXP>=2;	44	9.00%
LRP1	LRP1: EXP>=2;	44	9.00%
SLC2A10	SLC2A10: EXP>=2;	44	9.00%
SULF2	SULF2: EXP>=2;	42	8.00%
PEX1	PEX1: EXP>=2;	42	8.00%
MDH2	MDH2: EXP>=2;	41	8.00%
EFNA5	EFNA5: EXP>=2;	41	8.00%
ITGB8	ITGB8: EXP>=2;	40	8.00%
HDGF	HDGF: EXP>=2;	40	8.00%
RAC1	RAC1: EXP>=2;	40	8.00%
GNPAT	GNPAT: EXP>=2;	39	8.00%
ANXA2P2	ANXA2P2: EXP>=2;	38	7.00%
TUFM	TUFM: EXP>=2;	38	7.00%
HSPD1	HSPD1: EXP>=2;	38	7.00%
COL23A1	COL23A1: EXP>=2;	37	7.00%
KAZALD1	KAZALD1: EXP>=2;	37	7.00%
EPDR1	EPDR1: EXP>=2;	37	7.00%
HSP90B1	HSP90B1: EXP>=2;	37	7.00%
FER	FER: EXP>=2;	37	7.00%
SBSPON	SBSPON: EXP>=2;	36	7.00%
OPTC	OPTC: EXP>=2;	36	7.00%
HAPLN1	HAPLN1: EXP>=2;	36	7.00%
EDIL3	EDIL3: EXP>=2;	36	7.00%
WNT5B	WNT5B: EXP>=2;	36	7.00%
FGL2	FGL2: EXP>=2;	36	7.00%

ADGRG1	ADGRG1: EXP>=2;	36	7.00%
TNFRSF1A	TNFRSF1A: EXP>=2;	36	7.00%
FERMT1	FERMT1: EXP>=2;	36	7.00%
SHH	SHH: EXP>=2;	36	7.00%
COL3A1	COL3A1: EXP>=2;	35	7.00%
CTSZ	CTSZ: EXP>=2;	35	7.00%
NUDT1	NUDT1: EXP>=2;	35	7.00%
ARL2BP	ARL2BP: EXP>=2;	35	7.00%
CAV2	CAV2: EXP>=2;	35	7.00%
MXRA5	MXRA5: EXP>=2;	34	7.00%
COL4A2	COL4A2: EXP>=2;	34	7.00%
POSTN	POSTN: EXP>=2;	34	7.00%
POMZP3	POMZP3: EXP>=2;	34	7.00%
ANXA2	ANXA2: EXP>=2;	34	7.00%
FASTKD2	FASTKD2: EXP>=2;	34	7.00%
CAV1	CAV1: EXP>=2;	34	7.00%
EGFLAM	EGFLAM: EXP>=2;	33	6.00%
ELFN2	ELFN2: EXP>=2;	33	6.00%
PRDX4	PRDX4: EXP>=2;	33	6.00%
SHMT2	SHMT2: EXP>=2;	33	6.00%
HLCS	HLCS: EXP>=2;	33	6.00%
ITGA5	ITGA5: EXP>=2;	33	6.00%
LRRK2	LRRK2: EXP>=2;	33	6.00%
ASPN	ASPN: EXP>=2;	32	6.00%
CS	CS: EXP>=2;	32	6.00%
COL4A1	COL4A1: EXP>=2;	32	6.00%
RRAS	RRAS: EXP>=2;	32	6.00%
ALDH2	ALDH2: EXP>=2;	32	6.00%
ANXA4	ANXA4: EXP>=2;	32	6.00%
ITGA2	ITGA2: EXP>=2;	32	6.00%
ANXA6	ANXA6: EXP>=2;	32	6.00%
SERPINE1	SERPINE1: EXP>=2;	32	6.00%
ITGA3	ITGA3: EXP>=2;	32	6.00%
SIGLEC1	SIGLEC1: EXP>=2;	32	6.00%
CHMP1A	CHMP1A: EXP>=2;	32	6.00%
HNRNPA2B1	HNRNPA2B1: EXP>=2;	32	6.00%
PDGFA	PDGFA: EXP>=2;	32	6.00%
PDK4	PDK4: EXP>=2;	32	6.00%
KARS	KARS: EXP>=2;	32	6.00%

SRC	SRC: EXP>=2;	32	6.00%
MXRA7	MXRA7: EXP>=2;	31	6.00%
ACAN	ACAN: EXP>=2;	31	6.00%
ADAMTSL3	ADAMTSL3: EXP>=2;	31	6.00%
ADAMTS3	ADAMTS3: EXP>=2;	31	6.00%
LGALS3BP	LGALS3BP: EXP>=2;	31	6.00%
VASN	VASN: EXP>=2;	31	6.00%
ADAM12	ADAM12: EXP>=2;	31	6.00%
COASY	COASY: EXP>=2;	31	6.00%
TIMP4	TIMP4: EXP>=2;	31	6.00%
ITGB2	ITGB2: EXP>=2;	31	6.00%
BMP2	BMP2: EXP>=2;	31	6.00%
WT1	WT1: EXP>=2;	31	6.00%
LRRC3B	LRRC3B: EXP>=2;	30	6.00%
LGALS4	LGALS4: EXP>=2;	30	6.00%
TSTA3	TSTA3: EXP>=2;	30	6.00%
CST3	CST3: EXP>=2;	30	6.00%
GDF10	GDF10: EXP>=2;	30	6.00%
LOXL2	LOXL2: EXP>=2;	30	6.00%
CRYM	CRYM: EXP>=2;	30	6.00%
CD3E	CD3E: EXP>=2;	30	6.00%
LAD1	LAD1: EXP>=2;	29	6.00%
COL22A1	COL22A1: EXP>=2;	29	6.00%
AEBP1	AEBP1: EXP>=2;	29	6.00%
CTSS	CTSS: EXP>=2;	29	6.00%
FAP	FAP: EXP>=2;	29	6.00%
P3H2	P3H2: EXP>=2;	29	6.00%
CCN4	CCN4: EXP>=2;	29	6.00%
LGALS9	LGALS9: EXP>=2;	29	6.00%
SOD1	SOD1: EXP>=2;	29	6.00%
PTK2B	PTK2B: EXP>=2;	29	6.00%
