# Supplementary material for: Prognostic and therapeutic implications of extracellular matrix associated gene signature in renal clear cell carcinoma
Source: Sci Rep. 2021 Apr 7;11:7561. doi: 10.1038/s41598-021-86888-7 (PMC8026590; doi:10.1038/s41598-021-86888-7)
Supplement: Supplementary file 5 — Supplementary Information 5. [file 41598_2021_86888_MOESM5_ESM.pdf]

Prognostic and therapeutic implications of extracellular matrix associated gene signature in renal clear cell carcinoma

Pankaj Ahluwalia[1], Meenakshi Ahluwalia[1], Ashis K. Mondal[1], Nikhil Sahajpal[1], Vamsi Kota[2], Mumtaz V. Rojiani[1], Amyn M. Rojiani[1], and Ravindra Kolhe[1]*

[1] Department of Pathology, Medical College of Georgia, Augusta University, GA, U.S.A.
[2] Department of Medicine, Medical College of Georgia, Augusta University, GA, U.S.A.
[*] Correspondence: rkolhe@augusta.edu; Tel.: (706)-721-2771; Fax :(706)-434-6053

Supplementary Table 3: Survival analysis of ECM genes using cBioportal in TCGA KIRC dataset (n = 510).

Gene Symbol	OQL Line	Num Samples Altered	Percent Samples Altered	PFS (Log-rank)	OS (Log-rank)	DSS (Log-rank)	DFS (Log-rank)
NUDT1	NUDT1: EXP>=2;	35	0.07	0.000000	0.000042	0.000000	0.396000
SIGLEC1	SIGLEC1: EXP>=2;	32	0.06	0.003083	0.000159	0.002584	0.022100
LRP1	LRP1: EXP>=2;	44	0.09	0.000061	0.000310	0.000025	0.949000
LOXL2	LOXL2: EXP>=2;	30	0.06	0.000005	0.000815	0.000001	0.725000
SERPINE 1	SERPINE1: EXP>=2;	32	0.06	0.000197	0.005720	0.000102	0.477000
PLOD3	PLOD3: EXP>=2;	65	0.13	0.003759	0.008215	0.003305	0.266000
ZP3	ZP3: EXP>=2;	47	0.09	0.000002	0.010100	0.000012	0.489000
RARRES 2	RARRES2: EXP>=2;	48	0.09	0.001234	0.018000	0.001376	0.906000
TGM2	TGM2: EXP>=2;	46	0.09	0.015600	0.019800	0.000677	0.176000
COL3A1	COL3A1: EXP>=2;	35	0.07	0.009221	0.033700	0.014800	0.307000
ANXA4	ANXA4: EXP>=2;	32	0.06	0.000012	0.036100	0.007233	0.004350
POSTN	POSTN: EXP>=2;	34	0.07	0.014700	0.043700	0.030800	0.516000
HNRNPA 2B1	HNRNPA2B1: EXP>=2;	32	0.06	0.000020	0.000000	0.000000	0.763000
LGALS3 BP	LGALS3BP: EXP>=2;	31	0.06	0.000001	0.000012	0.000000	0.559000
COL22A1	COL22A1: EXP>=2;	29	0.06	0.000002	0.000028	0.000001	0.435000
SRC	SRC: EXP>=2;	32	0.06	0.000855	0.000028	0.000001	0.491000
ANXA2P 2	ANXA2P2: EXP>=2;	38	0.07	0.000000	0.000078	0.000000	0.704000
EFNA5	EFNA5: EXP>=2;	41	0.08	0.000000	0.000082	0.000000	0.034200
GARS	GARS: EXP>=2;	44	0.09	0.000000	0.000124	0.000003	0.645000
CCN4	CCN4: EXP>=2;	29	0.06	0.001131	0.000237	0.001074	0.922000
HSP90B1	HSP90B1: EXP>=2;	37	0.07	0.000000	0.000500	0.000002	0.046900
TNFRSF1 A	TNFRSF1A: EXP>=2;	36	0.07	0.000000	0.000590	0.000000	0.642000
OPTC	OPTC: EXP>=2;	36	0.07	0.198000	0.000813	0.047600	0.316000

WNT5B	WNT5B: EXP>=2;	36	0.07	0.009201	0.001024	0.000316	0.790000
MXRA5	MXRA5: EXP>=2;	34	0.07	0.000199	0.001160	0.000353	0.781000
ANXA2	ANXA2: EXP>=2;	34	0.07	0.000011	0.001751	0.000002	0.903000
SULF2	SULF2: EXP>=2;	42	0.08	0.004177	0.001838	0.000026	0.676000
RRAS	RRAS: EXP>=2;	32	0.06	0.000003	0.001973	0.000068	0.731000
GDF10	GDF10: EXP>=2;	30	0.06	0.017900	0.003357	0.039800	0.005096
PTK2B	PTK2B: EXP>=2;	29	0.06	0.112000	0.004207	0.001129	0.661000
COASY	COASY: EXP>=2;	31	0.06	0.238000	0.004982	0.005449	0.195000
ADAM12	ADAM12: EXP>=2;	31	0.06	0.000334	0.008762	0.000112	0.272000
PDK4	PDK4: EXP>=2;	32	0.06	0.368000	0.011200	0.065600	0.622000
WT1	WT1: EXP>=2;	31	0.06	0.011200	0.019000	0.007564	0.534000
SLC2A10	SLC2A10: EXP>=2;	44	0.09	0.181000	0.029300	0.009072	0.311000
ITGA3	ITGA3: EXP>=2;	32	0.06	0.008317	0.032200	0.007755	0.472000
VASN	VASN: EXP>=2;	31	0.06	0.299000	0.034200	0.163000	0.319000
SSBP1	SSBP1: EXP>=2;	44	0.09	0.003413	0.034400	0.000370	0.663000
ITGA2	ITGA2: EXP>=2;	32	0.06	0.816000	0.039800	0.260000	0.166000
P3H2	P3H2: EXP>=2;	29	0.06	0.000079	0.042200	0.001206	0.507000
DNAJB6	DNAJB6: EXP>=2;	51	0.1	0.000130	0.042900	0.002325	0.949000
SBSPON	SBSPON: EXP>=2;	36	0.07	0.000567	0.049500	0.003332	0.359000
HDGF	HDGF: EXP>=2;	40	0.08	0.002804	0.056000	0.010600	0.651000
ITGB2	ITGB2: EXP>=2;	31	0.06	0.267000	0.077600	0.177000	0.029700
EPDR1	EPDR1: EXP>=2;	37	0.07	0.001403	0.078300	0.003252	0.874000
ITGA5	ITGA5: EXP>=2;	33	0.06	0.000142	0.082700	0.001016	0.302000
ADAMTS3	ADAMTS3: EXP>=2;	31	0.06	0.034400	0.089100	0.795000	0.017500
FAP	FAP: EXP>=2;	29	0.06	0.037800	0.102000	0.085600	0.967000
KAZALD1	KAZALD1: EXP>=2;	37	0.07	0.037100	0.108000	0.048900	0.729000
TSTA3	TSTA3: EXP>=2;	30	0.06	0.005213	0.113000	0.012900	0.893000

HLCS	HLCS: EXP>=2;	33	0.06	0.583000	0.113000	0.542000	0.838000
CRYM	CRYM: EXP>=2;	30	0.06	0.374000	0.118000	0.047300	0.428000
MMP24	MMP24: EXP>=2;	46	0.09	0.552000	0.128000	0.641000	0.831000
CTSS	CTSS: EXP>=2;	29	0.06	0.159000	0.132000	0.156000	0.566000
RAC1	RAC1: EXP>=2;	40	0.08	0.001018	0.134000	0.008401	0.436000
HAPLN1	HAPLN1: EXP>=2;	36	0.07	0.001282	0.145000	0.024200	0.252000
CD3E	CD3E: EXP>=2;	30	0.06	0.209000	0.155000	0.192000	0.446000
ALDH7A1	ALDH7A1: EXP>=2;	44	0.09	0.338000	0.155000	0.331000	0.110000
PEX1	PEX1: EXP>=2;	42	0.08	0.120000	0.160000	0.406000	0.204000
CHMP1A	CHMP1A: EXP>=2;	32	0.06	0.140000	0.172000	0.032800	0.226000
CAV1	CAV1: EXP>=2;	34	0.07	0.000493	0.172000	0.066400	0.061500
SHMT2	SHMT2: EXP>=2;	33	0.06	0.182000	0.175000	0.258000	0.092400
KARS	KARS: EXP>=2;	32	0.06	0.105000	0.182000	0.257000	0.630000
FER	FER: EXP>=2;	37	0.07	0.284000	0.190000	0.360000	0.459000
COL23A1	COL23A1: EXP>=2;	37	0.07	0.040000	0.200000	0.123000	0.320000
TGFBI	TGFBI: EXP>=2;	47	0.09	0.001741	0.225000	0.023900	0.014000
LGALS9	LGALS9: EXP>=2;	29	0.06	0.005980	0.231000	0.070600	0.235000
ELFN2	ELFN2: EXP>=2;	33	0.06	0.833000	0.251000	0.716000	0.163000
SOD1	SOD1: EXP>=2;	29	0.06	0.041300	0.306000	0.083000	0.965000
FERMT1	FERMT1: EXP>=2;	36	0.07	0.352000	0.306000	0.762000	0.141000
CS	CS: EXP>=2;	32	0.06	0.133000	0.310000	0.105000	0.221000
ACAN	ACAN: EXP>=2;	31	0.06	0.758000	0.336000	0.308000	0.150000
ASPN	ASPN: EXP>=2;	32	0.06	0.009322	0.359000	0.139000	0.254000
LRRK2	LRRK2: EXP>=2;	33	0.06	0.044200	0.390000	0.333000	0.320000
CCL28	CCL28: EXP>=2;	50	0.1	0.956000	0.413000	0.611000	0.262000
EDIL3	EDIL3: EXP>=2;	36	0.07	0.620000	0.508000	0.632000	0.076200
ADAMTSL3	ADAMTSL3: EXP>=2;	31	0.06	0.822000	0.531000	0.927000	0.391000

ADGRG1	ADGRG1: EXP>=2;	36	0.07	0.458000	0.550000	0.810000	0.731000
AEBP1	AEBP1: EXP>=2;	29	0.06	0.371000	0.555000	0.567000	0.938000
CAV2	CAV2: EXP>=2;	35	0.07	0.235000	0.571000	0.437000	0.468000
PRDX4	PRDX4: EXP>=2;	33	0.06	0.044500	0.595000	0.034000	0.718000
CTSZ	CTSZ: EXP>=2;	35	0.07	0.680000	0.613000	0.660000	0.477000
MDH2	MDH2: EXP>=2;	41	0.08	0.962000	0.645000	0.980000	0.799000
ITGB8	ITGB8: EXP>=2;	40	0.08	0.490000	0.656000	0.676000	0.921000
COL4A2	COL4A2: EXP>=2;	34	0.07	0.612000	0.672000	0.784000	0.444000
FASTKD2	FASTKD2: EXP>=2;	34	0.07	0.019100	0.679000	0.106000	0.775000
LRRC3B	LRRC3B: EXP>=2;	30	0.06	0.245000	0.691000	0.188000	0.397000
COL4A1	COL4A1: EXP>=2;	32	0.06	0.408000	0.738000	0.794000	0.377000
HSPD1	HSPD1: EXP>=2;	38	0.07	0.793000	0.774000	0.440000	0.909000
TIMP4	TIMP4: EXP>=2;	31	0.06	0.181000	0.795000	0.696000	0.983000
ARL2BP	ARL2BP: EXP>=2;	35	0.07	0.034100	0.799000	0.440000	0.237000
FGL2	FGL2: EXP>=2;	36	0.07	0.507000	0.835000	0.960000	0.339000
CST3	CST3: EXP>=2;	30	0.06	0.622000	0.852000	0.379000	0.268000
VDAC1	VDAC1: EXP>=2;	56	0.11	0.012700	0.878000	0.510000	0.011600
BMP2	BMP2: EXP>=2;	31	0.06	0.401000	0.881000	0.491000	0.854000
MXRA7	MXRA7: EXP>=2;	31	0.06	0.197000	0.884000	0.968000	0.724000
LGALS4	LGALS4: EXP>=2;	30	0.06	0.158000	0.927000	0.328000	0.807000
PDGFA	PDGFA: EXP>=2;	32	0.06	0.592000	0.928000	0.552000	0.440000
ANXA6	ANXA6: EXP>=2;	32	0.06	0.981000	0.939000	0.996000	0.357000
TUFM	TUFM: EXP>=2;	38	0.07	0.921000	0.942000	0.520000	0.294000
ALDH2	ALDH2: EXP>=2;	32	0.06	0.693000	0.944000	0.557000	0.666000
GNPAT	GNPAT: EXP>=2;	39	0.08	0.119000	0.962000	0.477000	0.136000
POMZP3	POMZP3: EXP>=2;	34	0.07	0.807000	0.972000	0.794000	0.681000
EGFLAM	EGFLAM: EXP>=2;	33	0.06	0.304000	0.974000	0.301000	0.756000

SHH	SHH: EXP>=2;	36	0.07	0.905000	0.984000	0.877000	0.994000
LAD1	LAD1: EXP>=2;	29	0.06	0.322000	0.994000	0.335000	0.971000
