# Supplementary material for: Prognostic and therapeutic implications of extracellular matrix associated gene signature in renal clear cell carcinoma
Source: Sci Rep. 2021 Apr 7;11:7561. doi: 10.1038/s41598-021-86888-7 (PMC8026590; doi:10.1038/s41598-021-86888-7)
Supplement: Supplementary file 6 — Supplementary Information 6. [file 41598_2021_86888_MOESM6_ESM.pdf]

Prognostic and therapeutic implications of extracellular matrix associated gene signature in renal clear cell carcinoma

Pankaj Ahluwalia[1], Meenakshi Ahluwalia[1], Ashis K. Mondal[1], Nikhil Sahajpal[1], Vamsi Kota[2], Mumtaz V. Rojiani[1], Amyn M. Rojiani[1], and Ravindra Kolhe[1]*

[1] Department of Pathology, Medical College of Georgia, Augusta University, GA, U.S.A.
[2] Department of Medicine, Medical College of Georgia, Augusta University, GA, U.S.A.
[*] Correspondence: rkolhe@augusta.edu; Tel.: (706)-721-2771; Fax :(706)-434-6053

Supplementary Table 4: Differentially expressed genes between two risk groups.

Gene ID	Gene	baseMean	log2FoldChange	lfcSE	stat	pvalue	padj
785	ITGB1BP3	335.4729484	10.3627916	1.178637	8.792184	1.47E-18	3.66E-17
1420	KLK15	76.10125777	9.927385627	1.292855	7.678654	1.61E-14	2.22E-13
1033	KLK3	34.06250894	9.815475576	1.185492	8.279663	1.24E-16	2.34E-15
398	CFTR	973.6655222	8.724207943	0.868659	10.04331	9.83E-24	4.84E-22
1715	BSND	162.0951966	8.475490637	1.163703	7.283205	3.26E-13	3.73E-12
4153	SLC38A8	13.89975653	8.4704135	1.613467	5.24982	1.52E-07	7.19E-07
678	NR0B2	133.6150616	8.14878715	0.897816	9.076233	1.12E-19	3.25E-18
1012	FBN3	174.6528876	7.891082996	0.948153	8.322588	8.61E-17	1.67E-15
787	IEPACAM	145.2686421	7.882196625	0.89717	8.785618	1.56E-18	3.87E-17
1027	CLDN8	284.3507112	7.671202534	0.925133	8.291998	1.11E-16	2.13E-15
562	SFTPB	176.067388	7.65749331	0.811765	9.433135	3.98E-21	1.39E-19
1744	LRRC52	11.69866368	7.653440085	1.055715	7.249531	4.18E-13	4.70E-12
410	ATP6V0A4	2217.793229	7.644762826	0.763913	10.00738	1.41E-23	6.76E-22
394	PVALB	1118.056516	7.508234217	0.74501	10.07804	6.91E-24	3.44E-22
753	PART1	106.8539674	7.366615154	0.829244	8.883532	6.48E-19	1.69E-17
232	ATP6V0D2	2818.781964	7.32854618	0.667891	10.97267	5.17E-28	4.37E-26
527	CLNK	117.3548309	7.154939101	0.750981	9.527463	1.61E-21	5.99E-20
613	TMEM213	1586.811102	7.081374579	0.763357	9.276628	1.75E-20	5.59E-19
2870	LRRTM1	14.6938765	7.015238777	1.144496	6.129542	8.81E-10	6.02E-09
4781	GPRC6A	19.50823766	6.990741234	1.428079	4.895207	9.82E-07	4.03E-06
242	FOXI2	172.8596158	6.985200322	0.639552	10.92202	9.05E-28	7.33E-26
894	DMRT2	593.7962897	6.967001576	0.815979	8.538216	1.36E-17	2.99E-16
909	KLK1	508.8954724	6.900025202	0.811031	8.507721	1.77E-17	3.82E-16
1949	LOC38949	21.20064074	6.830638173	0.97074	7.036525	1.97E-12	1.98E-11
37	HS6ST3	275.8655878	6.828158086	0.507225	13.46178	2.63E-41	1.39E-38
4373	MOG	10.55323877	6.789090956	1.326393	5.118462	3.08E-07	1.38E-06
102	SLC26A7	1398.358078	6.754387491	0.549097	12.3009	8.96E-35	1.72E-32
286	CLCNKB	1820.25338	6.725249465	0.631757	10.64531	1.83E-26	1.26E-24
1157	KLK4	310.0364801	6.724200281	0.832282	8.079238	6.52E-16	1.10E-14
1453	FAM184B	21.00865761	6.68971691	0.876511	7.632208	2.31E-14	3.11E-13
4070	PNLIPRP3	10.7633977	6.638092904	1.253303	5.296479	1.18E-07	5.69E-07
1154	RHCG	2918.983646	6.619039574	0.818665	8.085162	6.21E-16	1.05E-14
486	TRIM63	216.1544357	6.590856874	0.681544	9.670474	4.03E-22	1.62E-20
3534	CALCA	17.77067775	6.570397211	1.164047	5.644443	1.66E-08	9.19E-08
1795	SYT10	49.13455778	6.547257482	0.90987	7.19582	6.21E-13	6.78E-12
547	SLC4A1	1795.97597	6.362603008	0.672338	9.463392	2.98E-21	1.07E-19
6731	ATP6V1G3	209.1881373	6.360999089	1.619171	3.928552	8.55E-05	0.000249
1884	PLA2G4F	331.2688743	6.345789802	0.893565	7.101655	1.23E-12	1.28E-11
318	TRIM50	218.9746822	6.257583128	0.598927	10.44799	1.50E-25	9.22E-24
946	CWH43	103.7663882	6.255742616	0.74002	8.453474	2.83E-17	5.86E-16
3718	FOXI1	1217.581029	6.230232945	1.127191	5.527218	3.25E-08	1.72E-07
2346	CTNNA2	44.01784185	6.088368671	0.920945	6.611005	3.82E-11	3.19E-10
73	UNC5D	148.4983357	6.079682985	0.481641	12.62285	1.58E-36	4.24E-34

524	KBTBD12	58.15854188	6.032903257	0.63289	9.532305	1.54E-21	5.75E-20
321	STAP1	633.644774	6.00504452	0.57502	10.4432	1.57E-25	9.61E-24
194	PAH	1962.601085	5.955173921	0.527013	11.29985	1.31E-29	1.33E-27
6710	C8orf71	6.166553531	5.92866895	1.505845	3.937104	8.25E-05	0.000241
815	FGF9	302.31359	5.871912709	0.673269	8.721496	2.75E-18	6.60E-17
793	CTXN3	85.38282536	5.84527512	0.666536	8.769637	1.79E-18	4.43E-17
555	AQP6	265.190141	5.839783963	0.618267	9.445401	3.54E-21	1.25E-19
367	ATP6V1B1	1342.916715	5.826965863	0.571133	10.20248	1.93E-24	1.03E-22
2039	RHBG	203.1411286	5.819085453	0.840665	6.922001	4.45E-12	4.28E-11
2705	PAK7	17.4932273	5.758020153	0.917529	6.27557	3.48E-10	2.52E-09
93	ERP27	1340.890683	5.734687205	0.462053	12.41131	2.27E-35	4.78E-33
1885	ASB5	54.60322678	5.679490462	0.799911	7.100157	1.25E-12	1.30E-11
4228	KLKP1	7.338375212	5.644760428	1.086617	5.194802	2.05E-07	9.50E-07
2903	GCGR	95.0656568	5.6210038	0.920089	6.109194	1.00E-09	6.76E-09
915	CYP4A22	6.995200575	5.515237567	0.649295	8.49419	1.99E-17	4.26E-16
207	INPP5J	702.8525092	5.417560543	0.484232	11.18795	4.67E-29	4.42E-27
6990	C10orf71	4.314311514	5.411286076	1.413264	3.828929	0.000129	0.000361
349	SLC5A8	612.3511503	5.217185502	0.506847	10.29342	7.54E-25	4.24E-23
502	CYP4A11	2340.527181	5.167973187	0.537879	9.608061	7.39E-22	2.89E-20
1303	KRTAP5-8	28.58065454	5.092446773	0.647883	7.860138	3.84E-15	5.77E-14
575	GGT6	119.9608711	5.069707943	0.539964	9.388974	6.06E-21	2.07E-19
311	HMGCS2	1477.285038	5.061500359	0.483348	10.47174	1.16E-25	7.34E-24
2747	PCP4	190.755522	5.01892797	0.804198	6.240907	4.35E-10	3.10E-09
625	SLC22A8	88.80568044	4.957595894	0.536872	9.234225	2.60E-20	8.16E-19
1542	RGS7	44.43229176	4.938373764	0.658015	7.504962	6.14E-14	7.81E-13
1052	SLC13A2	44.80171837	4.926787181	0.597924	8.239821	1.72E-16	3.21E-15
921	SLC9A2	164.2621468	4.923699267	0.579901	8.490589	2.06E-17	4.38E-16
937	LYPD6B	55.33963874	4.919822893	0.580992	8.467972	2.50E-17	5.22E-16
3318	SLC4A9	31.2778042	4.850406394	0.835516	5.805285	6.43E-09	3.80E-08
5236	DNTT	4.610272794	4.775898942	1.025354	4.657807	3.20E-06	1.20E-05
343	SLC22A24	9.797853194	4.761431271	0.461267	10.32251	5.57E-25	3.19E-23
984	TMPRSS2	606.9574178	4.742862917	0.566806	8.3677	5.88E-17	1.17E-15
1001	C9orf84	31.95903174	4.731954363	0.567222	8.342334	7.28E-17	1.43E-15
1076	CYP17A1	175.3668595	4.691780361	0.572288	8.198283	2.44E-16	4.44E-15
1855	CNTNAP5	82.97987611	4.690518649	0.657577	7.133032	9.82E-13	1.04E-11
6132	HHATL	599.9663402	4.6854538	1.113775	4.206821	2.59E-05	8.28E-05
488	C1orf168	73.64749184	4.676061094	0.483681	9.667656	4.14E-22	1.66E-20
2335	GPR110	255.252372	4.637063899	0.700528	6.619383	3.61E-11	3.03E-10
12	ENAM	261.5549193	4.599630033	0.308899	14.89042	3.80E-50	6.21E-47
522	SLC13A1	437.5808905	4.484640003	0.470192	9.537885	1.46E-21	5.47E-20
6202	PIK3C2G	9.582349049	4.481188566	1.073394	4.174783	2.98E-05	9.43E-05
1284	FLJ42875	41.78140802	4.480282991	0.568438	7.88175	3.23E-15	4.93E-14
626	SLC22A12	968.6450464	4.475595134	0.485182	9.224568	2.85E-20	8.91E-19
1585	MAG	19.43873561	4.464060992	0.600008	7.440005	1.01E-13	1.25E-12
1812	SLC22A6	679.7235055	4.463414342	0.621925	7.176774	7.14E-13	7.72E-12
287	FREM1	155.8528217	4.398382528	0.413276	10.64272	1.89E-26	1.29E-24
6910	SLC24A2	3.0205681	4.393336254	1.138333	3.859446	0.000114	0.000322

579	RANBP3L	51.49148582	4.355301819	0.464465	9.377033	6.79E-21	2.30E-19
2097	CNTN3	164.0684001	4.35364568	0.635411	6.851699	7.30E-12	6.82E-11
420	SLC22A13	29.25745169	4.343300102	0.436092	9.959593	2.29E-23	1.07E-21
386	C15orf59	170.2756633	4.33444853	0.429117	10.10086	5.48E-24	2.78E-22
370	SLC9A3	170.7564158	4.322790463	0.424605	10.18073	2.42E-24	1.28E-22
600	TMEM174	315.4056023	4.32077302	0.463767	9.316685	1.20E-20	3.92E-19
3000	PCGEM1	5.180821599	4.295376576	0.71208	6.032152	1.62E-09	1.06E-08
10547	ODAM	7.39530022	4.291716152	1.749066	2.453719	0.014139	0.026276
554	GCOM1	376.0417069	4.254779371	0.450292	9.448924	3.42E-21	1.21E-19
8971	C11orf53	2.006528004	4.229231099	1.398886	3.023285	0.0025	0.005463
636	TCL6	48.93680545	4.226595613	0.459467	9.198903	3.62E-20	1.11E-18
3182	SLC6A18	119.4365504	4.218928751	0.715883	5.893324	3.79E-09	2.33E-08
1893	OR1L8	6.021737976	4.174007853	0.588567	7.091816	1.32E-12	1.37E-11
2147	MAPK4	56.15605846	4.173186027	0.61278	6.810255	9.74E-12	8.89E-11
261	TMEM38A	246.8874231	4.123105919	0.382419	10.78166	4.20E-27	3.16E-25
875	REN	1117.571827	4.113441742	0.479411	8.580196	9.47E-18	2.12E-16
873	SLC10A2	489.0691868	4.110851931	0.478272	8.595224	8.31E-18	1.87E-16
8573	MUCL1	3.491663262	4.10991657	1.291665	3.181876	0.001463	0.003346
4141	STXBP5L	9.430101992	4.087977968	0.777395	5.258558	1.45E-07	6.87E-07
1438	G6PC	206.4078206	4.058424201	0.530621	7.64845	2.03E-14	2.77E-13
550	ELMOD1	65.50382363	4.052617195	0.428516	9.457332	3.16E-21	1.13E-19
10515	43901	1.859396024	4.051412727	1.642735	2.466261	0.013653	0.025451
805	LOC38838	208.2619085	4.027957763	0.460574	8.74551	2.22E-18	5.41E-17
993	SCGN	673.0436517	3.999427017	0.47854	8.357559	6.40E-17	1.26E-15
556	HAO2	489.9675818	3.987724529	0.422255	9.443876	3.59E-21	1.27E-19
963	CR1L	39.59945592	3.985202501	0.473056	8.424386	3.63E-17	7.38E-16
2877	C13orf35	2.951801972	3.983780767	0.650393	6.125187	9.06E-10	6.17E-09
6245	SLC9A4	21.56148001	3.955732865	0.95174	4.156318	3.23E-05	0.000102
2429	LPPR1	72.47995599	3.946244641	0.603875	6.534868	6.37E-11	5.14E-10
3089	C4BPB	14.42420692	3.939967287	0.65989	5.970641	2.36E-09	1.50E-08
2543	SH3GL2	141.0014649	3.935028215	0.612047	6.429296	1.28E-10	9.88E-10
3778	PGPEP1L	3.498045095	3.929345153	0.716007	5.487855	4.07E-08	2.11E-07
7317	CNGB3	2.036770034	3.929017478	1.063764	3.693503	0.000221	0.000593
9073	LOC40079	1.543917818	3.910158602	1.308236	2.988878	0.0028	0.006049
7994	C4orf51	1.539590658	3.905660664	1.144654	3.412089	0.000645	0.001581
4916	OVCH2	4.823824507	3.88398499	0.806081	4.818355	1.45E-06	5.77E-06
3465	FAM83B	27.80209918	3.880832443	0.681111	5.6978	1.21E-08	6.87E-08
2008	CLVS2	2.050795077	3.872048917	0.557178	6.949398	3.67E-12	3.58E-11
2864	MSLN	865.1259724	3.849678482	0.627729	6.132709	8.64E-10	5.91E-09
9020	SPACA1	1.410017701	3.844818641	1.279691	3.00449	0.00266	0.005781
672	TMED6	291.1820074	3.827988281	0.420915	9.094443	9.51E-20	2.77E-18
2422	SLC34A1	161.0582612	3.825224169	0.58502	6.538621	6.21E-11	5.02E-10
3978	SCRT1	3.194526104	3.792148903	0.70821	5.354552	8.58E-08	4.23E-07
306	MYRIP	311.1058537	3.784985773	0.359809	10.51942	7.03E-26	4.50E-24
2517	TRIM71	10.79421347	3.782355142	0.586631	6.447586	1.14E-10	8.85E-10
4221	VWA5B1	11.7626759	3.76440082	0.724157	5.19832	2.01E-07	9.34E-07
333	C1orf95	168.9518957	3.739079169	0.359878	10.38984	2.76E-25	1.62E-23

1363	SIM1	140.8424477	3.738352286	0.480937	7.773061	7.66E-15	1.10E-13
2764	UPF0639	8.621070332	3.726825321	0.598651	6.225374	4.80E-10	3.41E-09
3368	TPTE2P3	2.569422143	3.707561244	0.642881	5.767102	8.06E-09	4.69E-08
3090	TMEM61	62.61763774	3.701965206	0.620067	5.970269	2.37E-09	1.50E-08
418	TMEM72	106.3562313	3.686999049	0.37009	9.962433	2.23E-23	1.04E-21
1225	PACRG	108.2061736	3.682183919	0.461137	7.985003	1.41E-15	2.25E-14
538	HSD11B2	3298.418496	3.649041264	0.383956	9.503793	2.02E-21	7.37E-20
2489	SMOC1	551.4072258	3.63935186	0.561529	6.481152	9.10E-11	7.17E-10
2832	SLC6A19	1023.847081	3.636753269	0.590123	6.162705	7.15E-10	4.95E-09
85	C1orf210	168.8107311	3.633491404	0.290369	12.51337	6.31E-36	1.45E-33
64	AGPAT9	644.7315537	3.632985909	0.282825	12.84533	9.14E-38	2.80E-35
206	IRF6	301.9202893	3.622390885	0.323696	11.19072	4.53E-29	4.31E-27
817	NEURL	65.18892073	3.621551787	0.415457	8.717039	2.86E-18	6.85E-17
1822	TAF7L	11.68605954	3.620281994	0.504906	7.170204	7.49E-13	8.06E-12
8398	LHX9	3.214009385	3.616016045	1.112888	3.249219	0.001157	0.002701
1588	SERPINA7	6.405541611	3.603873567	0.484442	7.439229	1.01E-13	1.25E-12
1490	FAM70A	1045.070736	3.598299397	0.474395	7.58503	3.32E-14	4.37E-13
2413	OXGR1	27.21320611	3.596441676	0.549388	6.546268	5.90E-11	4.79E-10
2437	EPN3	64.41652135	3.59050572	0.550304	6.524581	6.82E-11	5.48E-10
5826	C1orf64	6.747295716	3.577277717	0.820439	4.360202	1.30E-05	4.37E-05
1369	DUSP15	269.7487528	3.572361171	0.460027	7.765552	8.13E-15	1.16E-13
3246	GRHL2	58.56422976	3.566801437	0.609311	5.853829	4.80E-09	2.90E-08
707	MCOLN3	117.7949776	3.559506491	0.395568	8.998472	2.29E-19	6.35E-18
2355	LRRC14B	54.97651531	3.558844796	0.539078	6.601724	4.06E-11	3.38E-10
601	LOC15332ε	25.06786296	3.543098239	0.380489	9.311951	1.26E-20	4.09E-19
7018	SPINK7	9.215063162	3.534951242	0.926449	3.815593	0.000136	0.000379
8729	PLA2G3	2.006232686	3.514832053	1.127983	3.116032	0.001833	0.004116
296	TRPM3	122.1622593	3.494082889	0.330267	10.57958	3.71E-26	2.45E-24
8977	KRT20	19.16749618	3.471519115	1.149055	3.021194	0.002518	0.005498
7191	SLC6A2	4.30090381	3.456160291	0.921924	3.748857	0.000178	0.000484
34	WDR72	1704.114801	3.451112109	0.255896	13.48637	1.88E-41	1.08E-38
6668	TTR	82.87874356	3.436733445	0.86903	3.954678	7.66E-05	0.000225
3487	THRSP	69.71545357	3.398564132	0.598003	5.683186	1.32E-08	7.43E-08
6536	CHIA	3.596961884	3.393533618	0.846265	4.010015	6.07E-05	0.000182
2975	SLC12A1	238.1576091	3.388738065	0.559945	6.051908	1.43E-09	9.43E-09
2104	F7	14.73096932	3.385897315	0.494657	6.844935	7.65E-12	7.13E-11
125	SLC27A2	806.1793389	3.383010289	0.282898	11.95842	5.87E-33	9.20E-31
2033	TFCP2L1	1975.935555	3.361822045	0.485479	6.92475	4.37E-12	4.21E-11
9412	SPATS1	1.895110679	3.360128952	1.177684	2.853166	0.004329	0.009015
640	GBA3	1291.365053	3.357041153	0.365123	9.194267	3.78E-20	1.16E-18
914	MUC20	3183.098801	3.344670516	0.393729	8.494845	1.98E-17	4.25E-16
193	OXCT1	2103.977645	3.343956113	0.295898	11.30106	1.30E-29	1.32E-27
1047	ELAVL2	69.22728832	3.337749263	0.404911	8.243164	1.68E-16	3.14E-15
1971	SPSB4	32.81224602	3.336462429	0.476643	6.999923	2.56E-12	2.55E-11
2941	MLNR	2.107486069	3.333502247	0.54861	6.076268	1.23E-09	8.20E-09
361	LHFPL3	9.207697745	3.331567876	0.325618	10.23152	1.43E-24	7.78E-23
5073	ATP4B	2.375439764	3.323090135	0.701742	4.73549	2.19E-06	8.44E-06

2607	NRK	82.63028056	3.322732136	0.521519	6.371263	1.87E-10	1.41E-09
8020	PIP	7.44501202	3.321220889	0.975617	3.404227	0.000664	0.001622
6071	SVOPL	13.57227541	3.316826184	0.78303	4.235885	2.28E-05	7.35E-05
8242	DPP10	3.924298042	3.316803181	1.000583	3.314871	0.000917	0.00218
5414	AGXT2L1	20.26574169	3.310469913	0.72612	4.559125	5.14E-06	1.86E-05
359	SLC2A4	212.4009748	3.304509172	0.32291	10.23354	1.40E-24	7.66E-23
1931	RPS6KA6	40.52011356	3.292801992	0.46671	7.055344	1.72E-12	1.75E-11
2197	CASR	99.33813339	3.283071421	0.485656	6.760075	1.38E-11	1.23E-10
2587	ALDOB	2934.379265	3.280991112	0.513162	6.393677	1.62E-10	1.23E-09
3085	TRIM67	15.47711823	3.268649587	0.547229	5.973092	2.33E-09	1.48E-08
1643	VIL1	367.2076198	3.264939342	0.44296	7.370736	1.70E-13	2.02E-12
366	PRLR	818.3840597	3.26225584	0.319619	10.20671	1.85E-24	9.91E-23
675	IQSEC3	207.9232795	3.257675563	0.358555	9.085579	1.03E-19	3.00E-18
1031	KIT	1581.848357	3.25068331	0.392451	8.283029	1.20E-16	2.28E-15
433	PPARGC1A	1587.553812	3.247248561	0.32844	9.886874	4.75E-23	2.15E-21
409	FREM2	559.035177	3.244329682	0.324011	10.01304	1.34E-23	6.40E-22
8289	CT62	1.915330245	3.243928942	0.983723	3.297605	0.000975	0.002306
1476	NTN1	329.1283144	3.241157028	0.426409	7.601049	2.94E-14	3.90E-13
4652	MCCD1	25.42227233	3.234252304	0.652135	4.959485	7.07E-07	2.98E-06
794	OGDHL	3132.929713	3.233309087	0.368722	8.768969	1.80E-18	4.45E-17
160	LIN7A	293.4700578	3.22595117	0.27831	11.59122	4.57E-31	5.59E-29
4366	PRMT8	4.561745312	3.224282253	0.629494	5.122024	3.02E-07	1.36E-06
9749	NTSR2	2.191256707	3.200261607	1.171018	2.732888	0.006278	0.012623
1107	CHL1	269.2854481	3.196446173	0.392093	8.152262	3.57E-16	6.32E-15
1267	TOX3	172.4879762	3.195329795	0.404323	7.902912	2.72E-15	4.22E-14
10781	RPL13AP17	0.951226895	3.195225752	1.349282	2.368094	0.01788	0.032508
1896	FTCD	348.954957	3.192979255	0.450308	7.090661	1.33E-12	1.38E-11
7735	MYH8	142.9449603	3.188638775	0.904884	3.523809	0.000425	0.001078
3066	ASB17	1.045480301	3.187868018	0.53242	5.987501	2.13E-09	1.36E-08
3169	LOC1001289	5.167941646	3.179560794	0.53836	5.906014	3.50E-09	2.17E-08
10643	C12orf77	1.189672289	3.175141878	1.313949	2.416487	0.015671	0.028861
4243	CLCNKA	435.9056092	3.1690982	0.611139	5.185564	2.15E-07	9.95E-07
2032	SLC5A1	1228.303931	3.168860583	0.457566	6.925473	4.35E-12	4.19E-11
7306	FRMD7	3.849102103	3.168091506	0.856675	3.698125	0.000217	0.000583
4101	BMP7	66.58365547	3.14467965	0.595495	5.280779	1.29E-07	6.15E-07
1065	SLC16A7	306.2946834	3.139910505	0.382047	8.218642	2.06E-16	3.79E-15
2744	FER1L6	11.80687732	3.135123909	0.502295	6.241599	4.33E-10	3.09E-09
1199	PPM1E	86.72267966	3.129437137	0.390463	8.014688	1.10E-15	1.81E-14
6216	UGT2A1	2.102356029	3.126505744	0.749714	4.170266	3.04E-05	9.59E-05
6131	KLHL34	2.439840909	3.126186817	0.743039	4.207301	2.58E-05	8.26E-05
4866	SLC22A10	0.95526035	3.124456507	0.643702	4.85389	1.21E-06	4.88E-06
4791	GABRG1	5.971060773	3.12285332	0.638527	4.890717	1.00E-06	4.11E-06
7639	NXPH2	7.424869371	3.119134137	0.874929	3.565014	0.000364	0.000934
11281	FXYD4	10.86741866	3.115278565	1.425567	2.185291	0.028868	0.050158
7255	LCN8	1.425335718	3.111816285	0.835792	3.723193	0.000197	0.000531
9795	TMPRSS7	1.597383257	3.092092483	1.137567	2.718163	0.006565	0.013136
1005	FAM189A2	158.8797753	3.087305674	0.370236	8.338745	7.51E-17	1.46E-15

4988	ABCB5	29.49810517	3.084424803	0.645224	4.780391	1.75E-06	6.88E-06
2435	LRRN1	41.82664379	3.08302701	0.472427	6.52593	6.76E-11	5.44E-10
635	ANO4	259.8153549	3.076870452	0.334361	9.202242	3.51E-20	1.08E-18
6241	ZNF804B	2.139032594	3.051371432	0.733785	4.158399	3.20E-05	0.000101
654	ESRRG	456.5933319	3.047785423	0.332751	9.159349	5.22E-20	1.56E-18
1976	HAP1	15.78542558	3.044396746	0.435279	6.994132	2.67E-12	2.65E-11
2010	ANO5	199.6710371	3.022618844	0.435044	6.947845	3.71E-12	3.62E-11
3958	SLC14A2	13.4317311	3.020842908	0.562366	5.371665	7.80E-08	3.86E-07
10722	OTC	0.804340932	3.018860922	1.263716	2.388876	0.0169	0.030895
7870	ACOT12	3.539483244	3.018450268	0.871331	3.464182	0.000532	0.001325
745	PLCG2	2787.973709	3.017438536	0.338877	8.904223	5.38E-19	1.41E-17
3535	SPAG6	10.48924901	3.017001015	0.53453	5.644209	1.66E-08	9.20E-08
1819	PCK1	3038.348173	3.01251731	0.420013	7.172433	7.37E-13	7.94E-12
582	SLC16A9	2025.974841	3.011116143	0.32131	9.371386	7.16E-21	2.41E-19
2662	GRM3	5.409409997	3.010041698	0.476373	6.318662	2.64E-10	1.94E-09
694	BSPRY	121.5399467	2.98279266	0.330198	9.033342	1.67E-19	4.70E-18
13058	GJD2	0.743383891	2.981088629	1.833348	1.626036	0.103942	0.156025
3446	GPR143	577.125642	2.976254631	0.521252	5.709815	1.13E-08	6.43E-08
2026	C4orf49	238.9833081	2.97523252	0.42915	6.932842	4.12E-12	3.99E-11
2735	C19orf46	19.84295898	2.97488507	0.475844	6.251812	4.06E-10	2.91E-09
11552	KRTAP5-3	0.862610049	2.974567269	1.418488	2.096999	0.035994	0.061067
2784	SV2B	260.5319452	2.97110677	0.478497	6.209254	5.32E-10	3.75E-09
7390	PLG	339.3898076	2.968499781	0.810833	3.661052	0.000251	0.000666
8442	C20orf151	1.475580098	2.963815179	0.917458	3.230463	0.001236	0.002869
4550	DPP6	53.3163099	2.96294103	0.591494	5.009249	5.46E-07	2.35E-06
3253	LCN10	55.96674786	2.961606007	0.506243	5.850171	4.91E-09	2.96E-08
1635	ZNF385B	283.0215416	2.951906737	0.399871	7.382146	1.56E-13	1.87E-12
638	NPR3	686.6934156	2.950396271	0.320774	9.197748	3.66E-20	1.12E-18
9160	CHRNA2	1.222416349	2.939326526	0.994478	2.955647	0.00312	0.006677
4398	SEZ6L	7.038487713	2.936334246	0.575235	5.104584	3.32E-07	1.48E-06
497	LDHD	414.5356975	2.934579494	0.304611	9.633844	5.75E-22	2.27E-20
316	C11orf92	217.560016	2.929034601	0.280001	10.4608	1.31E-25	8.11E-24
3802	SCG3	38.83839846	2.921431857	0.533779	5.473109	4.42E-08	2.28E-07
2773	EPB41L4B	119.407191	2.92070626	0.469746	6.217628	5.05E-10	3.57E-09
5634	CKMT1B	257.8358338	2.916274025	0.654816	4.453578	8.45E-06	2.94E-05
2982	FRMD1	22.05305168	2.915712864	0.482115	6.047751	1.47E-09	9.65E-09
6785	TTPA	1.210811918	2.915601734	0.746678	3.904763	9.43E-05	0.000272
3399	PAPPA2	64.01812323	2.902011417	0.504831	5.748482	9.00E-09	5.19E-08
4674	CDH17	158.0153978	2.899461763	0.58594	4.948391	7.48E-07	3.14E-06
3237	TJP3	69.31862505	2.899131627	0.494551	5.862151	4.57E-09	2.77E-08
6791	ANKRD30I	3.289201824	2.888304614	0.739994	3.903145	9.50E-05	0.000274
1984	MYT1	10.45213162	2.888251073	0.413469	6.98541	2.84E-12	2.81E-11
9365	GALNTL6	1.186484638	2.885649141	1.004051	2.874007	0.004053	0.008483
55	FZp686O24	289.1814038	2.884094812	0.221725	13.00753	1.11E-38	3.95E-36
9360	CYP1A2	3.142584288	2.884078671	1.002703	2.876303	0.004024	0.008426
10211	SYT14	20.04686067	2.872861269	1.114693	2.577266	0.009959	0.019116
7713	CKMT1A	147.4426285	2.870439262	0.812157	3.534341	0.000409	0.001039

2255	F11	36.68357867	2.862276828	0.427655	6.692952	2.19E-11	1.90E-10
1207	OVOL1	64.82561007	2.861547435	0.357515	8.004001	1.20E-15	1.96E-14
6051	MTNR1A	3.256708219	2.858166976	0.673732	4.242292	2.21E-05	7.17E-05
2175	B4GALNT3	149.4519446	2.856973251	0.421048	6.785393	1.16E-11	1.04E-10
1283	CA4	123.7039302	2.843598929	0.36065	7.884647	3.15E-15	4.82E-14
3597	NAT8L	44.77805507	2.843500243	0.507172	5.606584	2.06E-08	1.12E-07
2218	ADRB1	18.74646891	2.836498042	0.420949	6.738346	1.60E-11	1.42E-10
8016	CA10	6.519907041	2.835290146	0.832582	3.405419	0.000661	0.001615
184	NR3C2	462.407865	2.826800077	0.248524	11.37434	5.61E-30	5.95E-28
13068	CTAG2	0.820168633	2.822492597	1.739375	1.622705	0.104652	0.156969
3892	LOC15165[illegible]	0.902507512	2.818767736	0.521239	5.407823	6.38E-08	3.21E-07
1887	SLC17A1	403.7612513	2.804262155	0.395088	7.097808	1.27E-12	1.32E-11
13363	HTR3B	0.656963498	2.800111845	1.83899	1.522636	0.12785	0.187532
2917	CAMK2B	74.78432873	2.796532752	0.458511	6.099168	1.07E-09	7.16E-09
11522	C14orf53	0.693464871	2.796260978	1.325155	2.110138	0.034846	0.059275
1902	IGSF11	27.6496821	2.795038123	0.394536	7.084365	1.40E-12	1.44E-11
168	ANKRD56	161.2124129	2.78932908	0.241847	11.53346	8.95E-31	1.04E-28
4182	GLRA2	1.495354296	2.776824549	0.5318	5.221557	1.77E-07	8.32E-07
3174	MFSD6L	15.21197448	2.772220809	0.469697	5.902148	3.59E-09	2.22E-08
1111	TMEM27	3053.241419	2.762954852	0.339337	8.142211	3.88E-16	6.85E-15
414	ACSS3	1055.038141	2.760629152	0.276445	9.986171	1.75E-23	8.30E-22
13196	ADAM2	0.74703005	2.758481392	1.746406	1.579519	0.114217	0.169655
5492	BMPR1B	434.9017072	2.756744756	0.609159	4.525494	6.03E-06	2.15E-05
2098	EGF	385.8388302	2.755862306	0.402282	6.850565	7.36E-12	6.87E-11
1487	FSTL4	31.662498	2.754920212	0.362847	7.592502	3.14E-14	4.14E-13
5812	SLC22A9	1.85739693	2.749828835	0.62947	4.368479	1.25E-05	4.22E-05
6820	ASCL4	1.721831513	2.74716497	0.705986	3.891247	9.97E-05	0.000287
1421	SLC26A9	133.5186773	2.742728476	0.357204	7.678318	1.61E-14	2.22E-13
1830	LOC72380[illegible]	71.52781395	2.740513008	0.382486	7.165011	7.78E-13	8.32E-12
3073	SLC38A4	157.2550633	2.734344619	0.457167	5.981069	2.22E-09	1.41E-08
9967	SLC26A3	0.981255924	2.732403847	1.02557	2.664279	0.007715	0.015173
282	MPP7	258.1602243	2.730374991	0.255775	10.6749	1.33E-26	9.27E-25
4102	C6orf168	73.23108875	2.726903153	0.516409	5.280513	1.29E-07	6.16E-07
2251	SIX4	43.32047348	2.724578335	0.406946	6.695177	2.15E-11	1.88E-10
720	GYLTL1B	187.4543943	2.718196655	0.30316	8.966198	3.07E-19	8.36E-18
3263	STK32A	32.2854491	2.713323149	0.464624	5.839827	5.23E-09	3.14E-08
13642	CTAG1B	0.721920048	2.706803395	1.879099	1.440479	0.149732	0.215121
1482	ATP6V1C2	343.7432551	2.701879171	0.35572	7.595515	3.07E-14	4.05E-13
9293	SCEL	3.896092297	2.701129341	0.930683	2.902309	0.003704	0.007813
3030	ACE2	1850.561249	2.698643102	0.448982	6.010578	1.85E-09	1.20E-08
10341	LHCGR	6.937986481	2.698528197	1.065409	2.532858	0.011314	0.021445
5922	C14orf180	64.33133539	2.696991339	0.625475	4.311912	1.62E-05	5.36E-05
6885	TMPRSS11[illegible]	1.680255516	2.696612865	0.696905	3.869415	0.000109	0.000311
4222	ARHGEF3[illegible]	9.086341388	2.694871496	0.518483	5.197605	2.02E-07	9.37E-07
4858	CHRM1	1.6722926	2.694756986	0.554495	4.859839	1.17E-06	4.74E-06
1520	NRG2	45.32753379	2.690809872	0.357172	7.533643	4.93E-14	6.37E-13
5529	AFM	15.96556204	2.688449527	0.596571	4.506505	6.59E-06	2.34E-05

3441	FCN2	9.781344743	2.687347733	0.470405	5.712838	1.11E-08	6.33E-08
25	CDS1	634.9064636	2.683625101	0.19193	13.98234	2.00E-44	1.57E-41
4391	OR2T10	8.962163986	2.680957348	0.524798	5.108554	3.25E-07	1.45E-06
1383	RDH12	48.11992681	2.671938808	0.345063	7.743329	9.68E-15	1.37E-13
2805	LOC338651	12.10813129	2.659678991	0.429784	6.18841	6.08E-10	4.25E-09
2568	SEMA3D	192.0021846	2.656909878	0.414396	6.411521	1.44E-10	1.10E-09
6657	CDH9	8.623011374	2.654766846	0.670389	3.960037	7.49E-05	0.000221
1595	SLC16A11	29.6085122	2.644513596	0.356042	7.427539	1.11E-13	1.36E-12
905	FAM169A	181.3254284	2.64062069	0.310246	8.511369	1.72E-17	3.72E-16
3242	FA2H	136.1255839	2.636777357	0.449988	5.859662	4.64E-09	2.80E-08
4831	PSCA	14.73729995	2.636192339	0.540802	4.874597	1.09E-06	4.42E-06
2320	COL19A1	16.66285291	2.628495924	0.395836	6.640374	3.13E-11	2.64E-10
4203	SORCS1	33.81635943	2.623414277	0.503908	5.206136	1.93E-07	8.99E-07
3230	ATRNL1	115.2317744	2.620784306	0.446807	5.86559	4.48E-09	2.72E-08
1774	TSPAN5	206.952909	2.612523012	0.362147	7.213978	5.43E-13	6.00E-12
39	ACAT1	4769.774192	2.611135976	0.194627	13.41609	4.87E-41	2.45E-38
3580	SLC5A12	1424.414721	2.609663319	0.464615	5.616831	1.94E-08	1.06E-07
947	C5orf23	2150.262699	2.605656337	0.308397	8.449042	2.94E-17	6.08E-16
2150	ABO	138.0669831	2.604164156	0.382461	6.808958	9.83E-12	8.96E-11
5867	ESRP1	115.1905305	2.60258578	0.600077	4.337087	1.44E-05	4.82E-05
1110	PKHD1	920.3030673	2.600743552	0.319164	8.14861	3.68E-16	6.50E-15
6984	NRAP	6.759198275	2.594028143	0.676865	3.832413	0.000127	0.000356
68	C6orf155	86.88886912	2.594023631	0.203309	12.75899	2.78E-37	8.01E-35
6964	ZNF536	4.93370696	2.593347505	0.675156	3.841107	0.000122	0.000345
13187	PSG3	0.859803588	2.592210946	1.636962	1.58355	0.113296	0.168389
1030	AGTR1	256.6192094	2.591251847	0.312685	8.287096	1.16E-16	2.21E-15
5326	CCBE1	63.4444511	2.588812979	0.561606	4.609657	4.03E-06	1.48E-05
5807	LOC286002	3.354407891	2.588256536	0.592116	4.371199	1.24E-05	4.17E-05
315	TRIM2	2062.566845	2.585802712	0.247128	10.46342	1.27E-25	7.91E-24
7635	C11orf16	0.895878275	2.580581167	0.723624	3.566191	0.000362	0.00093
2958	PROC	64.56661387	2.574239239	0.424538	6.063629	1.33E-09	8.82E-09
11641	ZNF705D	1.021099612	2.571819595	1.246267	2.063618	0.039054	0.065759
1584	MCF2	10.72471927	2.570651624	0.345399	7.442564	9.87E-14	1.22E-12
1539	KLHL3	392.5258044	2.564339008	0.341566	7.507598	6.02E-14	7.67E-13
6364	STOML3	1.211757819	2.563364776	0.625854	4.095786	4.21E-05	0.00013
1061	PFKFB2	1131.860855	2.561272486	0.311402	8.224967	1.95E-16	3.61E-15
12132	OR1N1	0.689379416	2.556210237	1.3334	1.917061	0.05523	0.089232
7055	NOTUM	4.130979905	2.551372066	0.671626	3.798801	0.000145	0.000404
9669	C10orf27	1.100930843	2.54935855	0.924417	2.7578	0.005819	0.011797
2396	NAT8B	120.3265229	2.548064907	0.388336	6.5615	5.33E-11	4.36E-10
40	ALDH6A1	640.9104551	2.538506853	0.190108	13.353	1.14E-40	5.58E-38
1793	LRRC19	349.6503871	2.527542011	0.351191	7.197068	6.15E-13	6.73E-12
7898	LOC285796	1.39533125	2.524925804	0.732059	3.449074	0.000563	0.001396
2553	SLC6A4	6.051479037	2.515966678	0.391664	6.42379	1.33E-10	1.02E-09
430	EMX2OS	1416.098811	2.513929565	0.25368	9.909846	3.77E-23	1.72E-21
305	RCAN2	2720.90124	2.513721562	0.23895	10.51988	7.00E-26	4.50E-24
4630	ST8SIA5	79.17005458	2.513482971	0.505337	4.973874	6.56E-07	2.78E-06

3150	ARSF	56.63537676	2.511223696	0.424035	5.922206	3.18E-09	1.98E-08
2469	ATP13A4	29.06199752	2.507142548	0.385796	6.498617	8.11E-11	6.44E-10
2130	SLC22A11	1535.304487	2.503112865	0.366826	6.823715	8.87E-12	8.16E-11
14232	PSKH2	0.506263095	2.500376413	2.001998	1.248941	0.211687	0.291545
7305	SNCB	3.538210852	2.498318769	0.675514	3.698397	0.000217	0.000582
5511	GAL3ST3	9.289992969	2.495924278	0.552926	4.514026	6.36E-06	2.26E-05
7445	SLC36A2	78.46513737	2.490593892	0.684472	3.638711	0.000274	0.000721
5242	SLC30A8	5.738082606	2.489281871	0.53483	4.654339	3.25E-06	1.22E-05
1510	WNK3	34.97801083	2.486642956	0.329169	7.554309	4.21E-14	5.47E-13
6030	WFIKKN2	3.960089693	2.486136016	0.584678	4.252147	2.12E-05	6.88E-05
3383	OMG	17.66989957	2.480923261	0.430889	5.75769	8.53E-09	4.94E-08
4159	C1orf161	93.81739654	2.480641258	0.472933	5.245233	1.56E-07	7.36E-07
6345	LOC14814[illegible]	8.30065856	2.476986323	0.603346	4.105419	4.04E-05	0.000125
9095	IGFBPL1	1.242618231	2.470668336	0.829512	2.978458	0.002897	0.006243
7189	SST	213.9495778	2.466028604	0.657721	3.749354	0.000177	0.000483
8179	ALLC	1.569336189	2.465806824	0.737598	3.34302	0.000829	0.001986
3745	EDAR	15.58851471	2.463885847	0.447181	5.509812	3.59E-08	1.88E-07
1562	MPPED2	124.2487797	2.463706433	0.329493	7.477275	7.59E-14	9.52E-13
126	ENPP5	678.8495856	2.45936174	0.205758	11.95267	6.29E-33	9.78E-31
1228	PHYHIPL	580.3005362	2.45425347	0.307658	7.977213	1.50E-15	2.39E-14
977	ACADL	836.3446086	2.451515271	0.292517	8.380751	5.26E-17	1.06E-15
2808	TMEM30B	343.1894694	2.443816016	0.395284	6.182425	6.31E-10	4.41E-09
13985	ASB11	0.577370926	2.443318884	1.845838	1.323691	0.185606	0.260122
2740	OSTBETA	43.87080022	2.441899568	0.391066	6.244214	4.26E-10	3.05E-09
5786	HABP2	3189.977952	2.441005407	0.557447	4.378903	1.19E-05	4.04E-05
1787	GLYATL1	1188.878342	2.432435556	0.337831	7.20015	6.01E-13	6.60E-12
6482	GSDMC	11.46147518	2.427107752	0.601355	4.036067	5.44E-05	0.000164
1009	NSUN7	159.4422556	2.426679572	0.291314	8.330112	8.08E-17	1.57E-15
2782	TUBB4	523.330502	2.426159374	0.390549	6.212179	5.23E-10	3.68E-09
452	MAPT	1441.399507	2.423390585	0.247445	9.793651	1.20E-22	5.20E-21
1593	GRB14	401.8579014	2.42308391	0.326067	7.431245	1.08E-13	1.32E-12
4093	LOC64300[illegible]	131.3920884	2.422707312	0.458451	5.284556	1.26E-07	6.03E-07
2897	CCDC158	15.20611095	2.422308467	0.39624	6.11323	9.76E-10	6.60E-09
4083	C5orf47	1.617890632	2.41918194	0.4573	5.290144	1.22E-07	5.87E-07
1059	TMEM171	350.7050385	2.411463833	0.293044	8.229028	1.89E-16	3.49E-15
695	RLN2	11.02658825	2.410854354	0.26691	9.032452	1.68E-19	4.73E-18
11038	IL20	0.596777649	2.410222881	1.058203	2.277657	0.022747	0.040394
2086	ILDR2	31.30273727	2.408341859	0.350968	6.861992	6.79E-12	6.38E-11
3981	KGFLP1	8.366778985	2.406444078	0.449523	5.353333	8.63E-08	4.25E-07
3912	SLC17A3	3747.166679	2.405534017	0.445832	5.395604	6.83E-08	3.42E-07
469	EXPH5	312.8886	2.40446251	0.246902	9.738525	2.07E-22	8.63E-21
4091	GRIK5	47.27324573	2.402813393	0.454539	5.286263	1.25E-07	5.98E-07
6500	RIMS1	6.857790577	2.401739374	0.596157	4.028704	5.61E-05	0.000169
2636	MGAM	1029.636148	2.393909968	0.3776	6.3398	2.30E-10	1.71E-09
1014	KL	2125.488956	2.393890207	0.28791	8.31471	9.20E-17	1.78E-15
4845	LUZP2	7.595641548	2.392589433	0.491405	4.868877	1.12E-06	4.54E-06
7251	STAC2	436.3094537	2.392190122	0.642297	3.72443	0.000196	0.000529

1447	SMTNL2	323.9645814	2.38961723	0.312831	7.638679	2.19E-14	2.97E-13
5062	ERBB4	98.04588249	2.38711275	0.503556	4.740515	2.13E-06	8.25E-06
8227	LPA	1.516084954	2.385180886	0.717988	3.322035	0.000894	0.002129
3712	GPR81	54.70879254	2.38474939	0.431147	5.531174	3.18E-08	1.68E-07
2722	BBOX1	2873.92993	2.381310431	0.380261	6.262313	3.79E-10	2.73E-09
14118	MMP26	0.493007176	2.375862868	1.853508	1.28182	0.199906	0.277543
4867	LHFPL4	2.71518483	2.375476192	0.489443	4.853426	1.21E-06	4.89E-06
1050	MIA2	263.8626135	2.365469697	0.287018	8.241541	1.70E-16	3.17E-15
336	PAIP2B	408.4582163	2.363674203	0.227786	10.37674	3.16E-25	1.85E-23
2034	UBE2QL1	238.3057142	2.36325702	0.341307	6.924132	4.39E-12	4.23E-11
1251	THSD7A	492.4400473	2.360809259	0.297652	7.931443	2.17E-15	3.39E-14
4959	DIRAS1	106.1288124	2.36029737	0.492484	4.792636	1.65E-06	6.51E-06
3690	MIOX	1178.596446	2.359365212	0.425915	5.539524	3.03E-08	1.61E-07
12645	PLK5P	1.371790925	2.354968155	1.339161	1.75854	0.078656	0.121924
3471	MYL3	67.70191951	2.353527963	0.413277	5.694796	1.24E-08	6.98E-08
8463	KNG1	155.4941222	2.353284064	0.730464	3.221631	0.001275	0.002952
551	TUB	461.4990843	2.35327355	0.248865	9.456041	3.20E-21	1.14E-19
4532	MYO16	15.07863114	2.34636162	0.467566	5.018249	5.21E-07	2.26E-06
1214	AQP7	142.5071862	2.342255519	0.292856	7.99799	1.26E-15	2.04E-14
438	MOBKL1A	2259.823215	2.339832911	0.237097	9.868661	5.69E-23	2.55E-21
12653	CCKAR	0.92353062	2.337164512	1.330804	1.756204	0.079054	0.122454
5269	ASPG	171.3878522	2.331422105	0.502288	4.641609	3.46E-06	1.29E-05
3444	C9orf71	191.317581	2.330247723	0.408079	5.710281	1.13E-08	6.42E-08
6680	CPEB1	65.29209118	2.328882882	0.589675	3.949437	7.83E-05	0.00023
12389	FAM123A	0.626081022	2.327052807	1.265144	1.839358	0.065862	0.104203
7707	WBSCR17	408.2899998	2.326661602	0.657845	3.536792	0.000405	0.00103
3015	PTGER3	2241.814783	2.316137172	0.384538	6.023164	1.71E-09	1.11E-08
546	SLC25A21	9.624663843	2.308340925	0.243832	9.466951	2.88E-21	1.03E-19
5138	SYNGR3	102.036549	2.306030732	0.489582	4.710206	2.47E-06	9.44E-06
2059	COL25A1	65.78871879	2.304757812	0.334587	6.888364	5.64E-12	5.37E-11
3161	TRHDE	411.475134	2.302176419	0.389413	5.911919	3.38E-09	2.10E-08
5836	CYP4Z2P	2.15118414	2.298955581	0.528041	4.353744	1.34E-05	4.49E-05
4690	PROZ	5.421064682	2.296966735	0.465579	4.933573	8.07E-07	3.37E-06
2433	LOC145837	161.7622045	2.290564957	0.350843	6.528751	6.63E-11	5.34E-10
618	RALGPS1	193.4728226	2.289310993	0.247363	9.254873	2.14E-20	6.80E-19
858	TBC1D14	3960.441185	2.286134453	0.265116	8.623153	6.51E-18	1.49E-16
2404	GABRB3	340.8413307	2.282415722	0.348243	6.554095	5.60E-11	4.56E-10
4115	TMEM132D	15.00645562	2.279438517	0.432357	5.272123	1.35E-07	6.42E-07
1029	GCNT4	71.31714479	2.276969068	0.274737	8.287802	1.15E-16	2.20E-15
445	GPD1L	1047.743546	2.275192952	0.231412	9.831768	8.22E-23	3.62E-21
3933	CAPN14	12.97587596	2.271007418	0.421914	5.382627	7.34E-08	3.66E-07
714	TSPAN7	1399.464237	2.270938307	0.252932	8.978462	2.75E-19	7.54E-18
2522	FRMPD2	9.448024937	2.266343906	0.351613	6.445569	1.15E-10	8.95E-10
6833	TCL1B	1.144511046	2.266109116	0.582981	3.887105	0.000101	0.000291
3052	ANGPTL1	143.799241	2.264592033	0.377585	5.997567	2.00E-09	1.29E-08
3644	ACSM2B	3411.022323	2.263551789	0.405958	5.575833	2.46E-08	1.33E-07
79	PTPN3	1560.715606	2.26014694	0.17978	12.57171	3.02E-36	7.49E-34

6851	AQP5	1.943246178	2.256954009	0.581767	3.879484	0.000105	0.000299
1668	SLC2A12	159.8817892	2.255508358	0.307386	7.337716	2.17E-13	2.55E-12
3037	NIPAL1	25.4147054	2.254133716	0.375227	6.007385	1.89E-09	1.22E-08
1239	SCN4A	106.6255016	2.252348618	0.283612	7.941669	1.99E-15	3.16E-14
2151	SLC47A1	3750.164641	2.24813383	0.33021	6.808199	9.88E-12	9.01E-11
3259	SLC5A10	1019.101262	2.245117328	0.384048	5.845927	5.04E-09	3.03E-08
211	MAP7	1081.500652	2.243825261	0.202305	11.09129	1.38E-28	1.28E-26
2593	SULT1C4	681.5451183	2.243281868	0.351104	6.389215	1.67E-10	1.26E-09
1233	MED12L	6.717830413	2.240350708	0.281332	7.963375	1.67E-15	2.66E-14
4730	TCEAL2	27.66231359	2.238457675	0.455127	4.91831	8.73E-07	3.62E-06
1958	FRMPD1	21.91534517	2.237100957	0.31847	7.024522	2.15E-12	2.15E-11
2268	PDZK1P1	334.8896839	2.235159987	0.334608	6.679929	2.39E-11	2.07E-10
2138	FLRT3	715.2796016	2.235080085	0.327924	6.815842	9.37E-12	8.59E-11
4394	CDH12	11.1957773	2.233367894	0.437284	5.107365	3.27E-07	1.46E-06
6980	RNASE7	1.324626033	2.225326621	0.580423	3.833973	0.000126	0.000354
304	FAM160A1	94.19876827	2.222366027	0.211246	10.52029	6.97E-26	4.49E-24
12785	DEFB132	0.879779124	2.221952775	1.300641	1.708353	0.087571	0.134257
3317	ZNF98	6.777197751	2.218687603	0.38215	5.8058	6.41E-09	3.79E-08
2363	CUBN	7494.150935	2.217928835	0.336201	6.597024	4.19E-11	3.48E-10
5324	C22orf31	1.07085352	2.217569289	0.480995	4.610379	4.02E-06	1.48E-05
10370	LOC284551	1.524011	2.214653484	0.879774	2.517298	0.011826	0.022353
11755	TMPRSS11A	0.751831586	2.213084547	1.086776	2.036376	0.041713	0.069557
908	GATM	6556.134026	2.211193311	0.259892	8.508109	1.77E-17	3.82E-16
2844	HMGCLL1	9.177846551	2.210505084	0.359484	6.149103	7.79E-10	5.37E-09
436	GPHN	592.8160634	2.20964199	0.223756	9.875241	5.33E-23	2.40E-21
5833	GRIA4	190.5907855	2.2089384	0.507217	4.355013	1.33E-05	4.47E-05
5095	PPP4R4	9.948219645	2.203924271	0.466033	4.729114	2.26E-06	8.68E-06
5031	SCGB1D2	27.23778375	2.201973342	0.462674	4.759228	1.94E-06	7.57E-06
2058	RGS9BP	5.594039756	2.198610522	0.318987	6.892473	5.48E-12	5.22E-11
3876	C12orf59	120.370452	2.197378412	0.405485	5.419142	5.99E-08	3.03E-07
12158	ATP12A	1.045360673	2.197030478	1.151161	1.908534	0.056322	0.090802
5124	DACT2	37.64475583	2.195117322	0.465459	4.716028	2.40E-06	9.20E-06
9430	A2ML1	1.416046498	2.194860767	0.771118	2.846334	0.004423	0.009193
12051	EPHA5	4.860948547	2.194033967	1.128755	1.943764	0.051924	0.084454
2569	ALDH1L1	2104.057118	2.192359353	0.341949	6.41137	1.44E-10	1.10E-09
9471	CALCB	1.09959425	2.191428948	0.774197	2.830585	0.004646	0.009615
3496	AVPR1B	40.77314625	2.190031401	0.385718	5.677808	1.36E-08	7.65E-08
1999	KCNJ3	255.2946165	2.189778863	0.314564	6.96132	3.37E-12	3.31E-11
6921	FBXO40	2.508792813	2.18887447	0.567622	3.856216	0.000115	0.000326
8681	CPNE6	1.242316172	2.185345421	0.69626	3.13869	0.001697	0.003832
388	KIAA0664P	47.33077688	2.184787324	0.216331	10.09926	5.57E-24	2.81E-22
2470	T6GALNAC	172.9526339	2.183910733	0.336093	6.497932	8.14E-11	6.46E-10
4296	PRR15L	104.132348	2.183240135	0.423192	5.158978	2.48E-07	1.13E-06
2021	SLC16A12	1850.836723	2.18070808	0.314367	6.936815	4.01E-12	3.89E-11
768	ZDHHC23	343.4810958	2.180033199	0.246612	8.839935	9.58E-19	2.44E-17
901	SGSM1	162.6256133	2.176260735	0.255335	8.523142	1.55E-17	3.38E-16
11398	C3orf30	0.999186528	2.174555879	1.011193	2.150485	0.031517	0.054199

2604	TINAG	616.3307818	2.174405751	0.341082	6.375027	1.83E-10	1.38E-09
2134	CCDC160	24.77323382	2.173556221	0.318684	6.820421	9.08E-12	8.34E-11
12392	ELSPBP1	1.13203178	2.170909638	1.180993	1.838208	0.066032	0.104446
7285	43900	20.18802504	2.16996188	0.585529	3.705985	0.000211	0.000567
1508	CLIC5	336.0869541	2.167285362	0.286853	7.555391	4.18E-14	5.43E-13
5488	CBLN2	3.904066386	2.16575848	0.478343	4.527625	5.97E-06	2.13E-05
9276	TDGF3	1.362287257	2.163935562	0.744374	2.907053	0.003649	0.00771
3245	ENO3	147.6205016	2.163661014	0.36958	5.854381	4.79E-09	2.89E-08
4623	SLC17A4	633.2960497	2.161763213	0.434221	4.978483	6.41E-07	2.72E-06
4932	KCNV1	27.3527882	2.159085891	0.449009	4.808559	1.52E-06	6.04E-06
6402	SLC17A8	3.172220119	2.158274816	0.529543	4.075734	4.59E-05	0.00014
7058	NOL4	12.55468134	2.156093475	0.567738	3.797689	0.000146	0.000406
11126	UGT2B4	1.219299168	2.15449059	0.960135	2.243945	0.024836	0.043754
4473	LOC283392	108.6445079	2.153997529	0.426005	5.056275	4.28E-07	1.87E-06
5707	PKD1L2	82.38911346	2.150640981	0.486961	4.416455	1.00E-05	3.45E-05
109	TMEM38B	766.1604601	2.146535707	0.175738	12.21441	2.60E-34	4.70E-32
5129	ZCCHC16	2.863465319	2.13525566	0.452967	4.713928	2.43E-06	9.29E-06
5609	SCNN1A	2178.903459	2.133120354	0.47747	4.46755	7.91E-06	2.77E-05
809	COL4A4	642.5241408	2.132131075	0.244065	8.735906	2.42E-18	5.86E-17
4137	TUBBP5	17.51762841	2.127715605	0.40449	5.260248	1.44E-07	6.82E-07
9778	ABCG8	1.372209305	2.12763226	0.781409	2.722817	0.006473	0.012974
491	SOX6	746.7142826	2.120400054	0.219595	9.65597	4.64E-22	1.85E-20
1121	ASTN2	387.5847621	2.111016354	0.25975	8.127107	4.40E-16	7.69E-15
8542	RAB25	79.55573607	2.110394562	0.661146	3.192027	0.001413	0.003242
3476	LRP2	10957.69871	2.107191529	0.370289	5.690673	1.27E-08	7.14E-08
7294	FSD2	1.754017032	2.106266104	0.569111	3.700976	0.000215	0.000577
3770	DNASE1	155.7170966	2.102810801	0.382659	5.495257	3.90E-08	2.03E-07
13174	MSLNL	0.568447072	2.094348587	1.31826	1.588722	0.112123	0.166831
1656	OCLN	197.0268265	2.093591525	0.284814	7.350731	1.97E-13	2.33E-12
5123	CSDC2	218.6793619	2.092538643	0.443699	4.71612	2.40E-06	9.20E-06
9924	HYAL4	2.186792813	2.090873267	0.780441	2.679092	0.007382	0.014581
3760	ACSM2A	4297.262859	2.090334598	0.380077	5.499769	3.80E-08	1.98E-07
1960	PIP5K1B	119.0242017	2.089874491	0.297674	7.020685	2.21E-12	2.21E-11
4003	FAM196B	47.60149385	2.089321604	0.391344	5.338832	9.35E-08	4.58E-07
30	RAB3IP	1384.19147	2.086989656	0.151784	13.74977	5.11E-43	3.34E-40
1613	DLG2	52.09373569	2.083935675	0.281252	7.409489	1.27E-13	1.54E-12
1334	SLC10A5	14.62744659	2.08375812	0.266568	7.816992	5.41E-15	7.95E-14
6427	OC1001301	2.752025124	2.083685477	0.51307	4.061215	4.88E-05	0.000149
3046	GDA	1556.056669	2.082752746	0.346918	6.003585	1.93E-09	1.24E-08
1706	BTNL9	994.8285242	2.080532717	0.285417	7.289461	3.11E-13	3.58E-12
2908	TMSB15A	22.53992771	2.07967833	0.340707	6.104008	1.03E-09	6.97E-09
5141	LOC285768	76.35750871	2.078134594	0.441453	4.707483	2.51E-06	9.56E-06
494	KIF21A	1743.036791	2.077336063	0.215319	9.647709	5.03E-22	1.99E-20
3218	FMO1	1284.037994	2.073491777	0.352933	5.875036	4.23E-09	2.57E-08
559	CA2	3380.329733	2.071703417	0.219541	9.436505	3.85E-21	1.35E-19
1618	PTH1R	327.3851627	2.06962144	0.279544	7.40356	1.33E-13	1.61E-12
571	PANK1	496.1513469	2.066386754	0.219729	9.404254	5.24E-21	1.80E-19

990	GHR	488.0142278	2.065828173	0.247109	8.359979	6.27E-17	1.24E-15
11479	ASCL3	0.533906372	2.065373286	0.97085	2.127386	0.033388	0.057012
375	PAQR5	998.1037679	2.064458145	0.203486	10.14545	3.47E-24	1.81E-22
3287	CRNA0017	9.118578301	2.061041208	0.353948	5.823001	5.78E-09	3.45E-08
5368	RMST	3.654733233	2.055399568	0.448376	4.584098	4.56E-06	1.67E-05
14746	MAGEC2	0.777869863	2.053919292	1.894199	1.084321	0.278223	0.369777
451	NTN4	1997.906185	2.050178087	0.209148	9.802502	1.10E-22	4.77E-21
792	PLCL1	606.9059654	2.047944982	0.233513	8.770157	1.78E-18	4.42E-17
2463	TMEM125	164.7807719	2.046143285	0.314557	6.504845	7.78E-11	6.19E-10
4419	UNC80	19.92592429	2.044647933	0.401643	5.090707	3.57E-07	1.58E-06
11190	NKX2-3	1.189504409	2.043695265	0.918569	2.224868	0.02609	0.045701
11229	TBL1Y	1.178678321	2.043463532	0.925413	2.208164	0.027233	0.047537
5192	SLIT2	500.9163742	2.043295654	0.436642	4.679571	2.87E-06	1.09E-05
5710	HS6ST2	19.75435352	2.042170329	0.46256	4.414929	1.01E-05	3.47E-05
2661	TRIM10	77.18365426	2.042083128	0.32317	6.318907	2.63E-10	1.94E-09
63	CLCN5	1825.910651	2.027583915	0.157824	12.8471	8.93E-38	2.78E-35
8	PCCA	857.3545377	2.026234889	0.130972	15.47076	5.47E-54	1.34E-50
808	RNF152	223.7974174	2.026153124	0.231924	8.736285	2.41E-18	5.84E-17
4974	SPON1	1891.673709	2.024062782	0.42286	4.786603	1.70E-06	6.68E-06
641	NNT	2972.526592	2.022664343	0.220193	9.185877	4.08E-20	1.25E-18
6257	DHRS2	50.74192494	2.020469902	0.486656	4.151737	3.30E-05	0.000103
124	LIFR	2190.474269	2.01940808	0.168545	11.98139	4.45E-33	7.03E-31
1912	FRAS1	869.8213985	2.018332736	0.28526	7.075403	1.49E-12	1.53E-11
1101	MYLK4	100.3839016	2.01723612	0.247253	8.158591	3.39E-16	6.03E-15
4701	PKIA	269.726117	2.015664136	0.408908	4.929385	8.25E-07	3.44E-06
4409	EML6	114.3763884	2.010612992	0.394576	5.095626	3.48E-07	1.55E-06
12102	CGA	0.955326223	2.008547346	1.041585	1.928356	0.053811	0.087155
1767	ZNF711	255.7284684	2.007036612	0.277807	7.224561	5.03E-13	5.58E-12
1358	PEG3	158.8146246	2.006301324	0.257781	7.78297	7.08E-15	1.02E-13
7094	LGI3	12.17583345	2.002332224	0.528729	3.78707	0.000152	0.000421
6511	FAM26D	1.02882056	2.001923655	0.497561	4.023477	5.73E-05	0.000173
11823	MAFA	0.661510556	2.000253268	0.99353	2.013279	0.044085	0.073088
3824	SCIN	1860.892392	1.997889569	0.36637	5.4532	4.95E-08	2.54E-07
230	GRAMD1C	479.8106304	1.99591183	0.181815	10.9777	4.89E-28	4.17E-26
9523	CYP11A1	57.45141829	1.987152301	0.707396	2.809109	0.004968	0.010225
1049	ANK3	1660.72917	1.986177941	0.24099	8.241731	1.70E-16	3.17E-15
7971	FAM5B	7.327022664	1.98568444	0.580438	3.421009	0.000624	0.001534
132	HSPA4L	528.5932326	1.981230485	0.167591	11.82181	3.01E-32	4.47E-30
3602	SLC18A2	20.81385776	1.980716599	0.353401	5.604728	2.09E-08	1.14E-07
4943	NAT8	4741.483227	1.979180992	0.412075	4.802963	1.56E-06	6.20E-06
514	ATP6V1A	5483.097517	1.978520492	0.207315	9.54355	1.38E-21	5.24E-20
364	FAM47E	66.22152487	1.97836942	0.193476	10.2254	1.53E-24	8.22E-23
1813	EFR3B	114.7505432	1.976925525	0.275479	7.176308	7.16E-13	7.74E-12
5948	C16orf89	136.9705745	1.974188849	0.459901	4.292636	1.77E-05	5.82E-05
851	ABCG2	387.016309	1.972695356	0.228442	8.63543	5.85E-18	1.35E-16
537	ABAT	564.0674414	1.968236711	0.207058	9.505725	1.99E-21	7.25E-20
6805	C21orf130	23.88021607	1.967100779	0.504747	3.897199	9.73E-05	0.00028

1875	EMX2	729.6058833	1.962662821	0.275976	7.111718	1.15E-12	1.20E-11
5286	C6	276.1579109	1.961349465	0.423603	4.630159	3.65E-06	1.35E-05
691	TMEM150C	380.8509891	1.95933019	0.216763	9.039057	1.58E-19	4.48E-18
2518	BTC	19.35892129	1.959120618	0.303859	6.447473	1.14E-10	8.85E-10
1232	NEDD4L	2927.743997	1.957205192	0.245755	7.964047	1.67E-15	2.65E-14
3702	ADH6	184.8580793	1.956701005	0.353556	5.534347	3.12E-08	1.65E-07
3474	SUCNR1	123.824493	1.951075617	0.342788	5.691783	1.26E-08	7.09E-08
5553	NOS1	4.207079341	1.950387299	0.433593	4.498201	6.85E-06	2.42E-05
2338	GIPC2	693.1188316	1.94853449	0.294435	6.617873	3.64E-11	3.06E-10
5825	LOC57255	1.4659675	1.948410718	0.446758	4.361224	1.29E-05	4.35E-05
10944	KCNA4	0.911813481	1.936999829	0.840075	2.305746	0.021125	0.037835
4969	SLC23A3	762.1843583	1.934544457	0.403955	4.789009	1.68E-06	6.61E-06
592	FNIP2	1730.823399	1.930997266	0.20667	9.343375	9.33E-21	3.09E-19
1470	ALMS1P	7.442610349	1.930940221	0.253806	7.607929	2.79E-14	3.71E-13
7508	LSAMP	12.56920938	1.930180769	0.534013	3.614482	0.000301	0.000786
3300	ACSBG1	16.74892126	1.927901495	0.331462	5.816365	6.01E-09	3.57E-08
49	ACAA2	5027.364384	1.925493555	0.146189	13.17124	1.28E-39	5.14E-37
3765	DDIT4L	901.3412472	1.923599153	0.349805	5.499063	3.82E-08	1.99E-07
4848	DHDH	61.57542606	1.921885299	0.394905	4.866707	1.13E-06	4.59E-06
2693	COL4A3	486.3452727	1.920666553	0.305488	6.287206	3.23E-10	2.35E-09
7698	RBP4	1428.117668	1.92041153	0.542452	3.540241	0.0004	0.001018
466	KIAA1737	873.0007039	1.920106392	0.196965	9.748463	1.87E-22	7.88E-21
4271	CEL	29.09719664	1.919316095	0.371199	5.170584	2.33E-07	1.07E-06
4054	CPXM2	1519.659726	1.919140314	0.361819	5.304147	1.13E-07	5.47E-07
4162	FLJ37543	2.944885322	1.91840314	0.365954	5.242202	1.59E-07	7.47E-07
1731	GPR98	43.03266111	1.918124934	0.26393	7.267561	3.66E-13	4.14E-12
2557	ARNT2	892.5616862	1.917449606	0.298748	6.418274	1.38E-10	1.06E-09
2771	NAP1L2	94.20600329	1.916417765	0.308119	6.219725	4.98E-10	3.52E-09
76	AUH	547.0975047	1.914623435	0.152305	12.571	3.05E-36	7.49E-34
1337	DMRTA1	119.9916405	1.914448338	0.245124	7.810138	5.71E-15	8.37E-14
1071	IL17RD	445.1133131	1.91141923	0.232921	8.206302	2.28E-16	4.18E-15
4433	C8orf47	139.7610883	1.910459107	0.375936	5.081874	3.74E-07	1.65E-06
2114	AGMAT	569.3184335	1.908009064	0.279222	6.833307	8.30E-12	7.69E-11
3566	KCTD16	53.05380294	1.906901772	0.338942	5.626039	1.84E-08	1.01E-07
2922	SLC1A2	33.53379521	1.906839329	0.31287	6.094673	1.10E-09	7.36E-09
467	FAM59A	344.5313002	1.903099025	0.195248	9.7471	1.90E-22	7.97E-21
853	HLF	503.4138349	1.901753322	0.220243	8.6348	5.88E-18	1.35E-16
11581	PCDH8	0.503815087	1.898964332	0.909058	2.088937	0.036713	0.062141
4488	MYO7B	476.7096447	1.896691581	0.375793	5.04717	4.48E-07	1.96E-06
4727	PRDM16	96.84954496	1.894906012	0.385214	4.919103	8.69E-07	3.61E-06
1208	FRMD3	264.0220174	1.894193463	0.23667	8.003531	1.21E-15	1.96E-14
4556	KCNJ11	123.6155169	1.89354963	0.378183	5.006965	5.53E-07	2.38E-06
12143	C14orf39	0.454254745	1.893519174	0.989274	1.91405	0.055614	0.089771
7109	PLAC4	14.44244795	1.892332966	0.500473	3.781089	0.000156	0.000431
12150	PSG9	1.143359922	1.891384455	0.989665	1.911135	0.055987	0.090321
52	L2HGDH	270.3667487	1.890667386	0.144838	13.0537	6.05E-39	2.30E-36
4740	CYP3A4	10.43019836	1.890347284	0.384719	4.913576	8.94E-07	3.70E-06

5571	IL1RL1	162.5247243	1.889228146	0.420951	4.488002	7.19E-06	2.53E-05
1850	SLC3A1	11167.60937	1.888381066	0.264682	7.134537	9.71E-13	1.03E-11
4562	FUT6	348.113214	1.885883361	0.376936	5.003189	5.64E-07	2.42E-06
4069	TMEM35	12.2227754	1.88554171	0.355966	5.296965	1.18E-07	5.67E-07
7331	MAL	1502.022219	1.878770358	0.509375	3.688383	0.000226	0.000603
7348	UNC13C	13.07662722	1.877450634	0.509862	3.682275	0.000231	0.000617
5905	AMBP	61.35674906	1.874170797	0.433907	4.319288	1.57E-05	5.20E-05
6981	C15orf50	1.300167796	1.873758193	0.488846	3.83302	0.000127	0.000355
1861	FMO5	172.3120359	1.873369484	0.262889	7.126077	1.03E-12	1.09E-11
2234	ACOT11	367.6464112	1.870550527	0.278512	6.716233	1.86E-11	1.64E-10
11619	INSM1	0.769953512	1.868190809	0.901082	2.073276	0.038147	0.064353
1197	EFHD1	1201.136468	1.867301407	0.23287	8.018638	1.07E-15	1.75E-14
6318	FCAMR	434.1700028	1.866755135	0.453377	4.117447	3.83E-05	0.000119
11375	NORD115-[illegible]	0.674717787	1.864852393	0.8634	2.159894	0.030781	0.053041
3095	C18orf34	22.48347552	1.861628102	0.311983	5.967087	2.42E-09	1.53E-08
594	RRAGD	2005.960166	1.860935255	0.199255	9.339455	9.68E-21	3.20E-19
8467	PNMT	2.667431756	1.854404116	0.575829	3.220408	0.00128	0.002963
2623	AMDHD1	94.94493934	1.848916735	0.290967	6.354376	2.09E-10	1.56E-09
5966	SLC29A2	120.7852305	1.847752778	0.431296	4.284192	1.83E-05	6.03E-05
2482	GULP1	576.4552665	1.847174264	0.284732	6.487414	8.73E-11	6.90E-10
5337	SYT9	191.9602793	1.846698212	0.401195	4.602994	4.16E-06	1.53E-05
245	EPHX2	1162.256991	1.845397755	0.169693	10.8749	1.52E-27	1.21E-25
2715	SGK494	135.8631207	1.844889716	0.294401	6.266579	3.69E-10	2.66E-09
3810	MYOT	23.74179094	1.843972525	0.337281	5.467166	4.57E-08	2.35E-07
10280	GLB1L3	18.19976	1.843197849	0.722192	2.552226	0.010704	0.020408
9505	SLCO1A2	5.31044745	1.837494323	0.651826	2.818994	0.004817	0.009934
331	SLC25A4	1525.144511	1.837247951	0.176674	10.39906	2.50E-25	1.48E-23
2399	UGT8	538.5591317	1.834594767	0.279652	6.560269	5.37E-11	4.39E-10
624	WDR31	54.36138818	1.831790921	0.198343	9.235479	2.57E-20	8.08E-19
9478	PRSS22	6.904583524	1.831474348	0.647967	2.826494	0.004706	0.009732
5669	CYP2J2	2747.230225	1.830475135	0.412585	4.436602	9.14E-06	3.16E-05
4127	RAP1GAP	1689.63155	1.827093111	0.347104	5.263823	1.41E-07	6.70E-07
3970	WDR63	14.78514358	1.826633548	0.340778	5.36018	8.31E-08	4.10E-07
6473	ABCC8	3.087683321	1.826561363	0.451978	4.041264	5.32E-05	0.000161
2309	C2orf88	297.1611974	1.82085011	0.273917	6.647454	2.98E-11	2.53E-10
2647	TLL1	333.9242981	1.81993496	0.287669	6.32649	2.51E-10	1.86E-09
7045	LOC28562[illegible]	4.207099552	1.819625458	0.4785	3.80277	0.000143	0.000398
5976	RXRG	2.88638229	1.8192608	0.425075	4.279862	1.87E-05	6.13E-05
3565	PGR	193.0401842	1.817887038	0.323093	5.626509	1.84E-08	1.01E-07
5306	C22orf43	2.527733691	1.816748647	0.393616	4.615538	3.92E-06	1.45E-05
3484	APOM	567.3940648	1.81618596	0.31953	5.683928	1.32E-08	7.41E-08
11069	CHRNA4	5.280050649	1.815657612	0.800819	2.267251	0.023375	0.041392
8139	LOC64259[illegible]	0.97352738	1.813929214	0.540325	3.357111	0.000788	0.001897
4342	CPT1B	514.4315957	1.813128091	0.352947	5.137114	2.79E-07	1.26E-06
2242	KLHL32	56.04286792	1.810373571	0.270083	6.703027	2.04E-11	1.78E-10
6145	KY	38.75776129	1.807759652	0.430051	4.203596	2.63E-05	8.38E-05
2586	PXK	400.9189526	1.801832707	0.281768	6.394748	1.61E-10	1.22E-09

4232	CRHBP	34.07269824	1.801122597	0.346866	5.192568	2.07E-07	9.61E-07
800	SC5DL	1497.331197	1.800283714	0.205629	8.755021	2.04E-18	5.00E-17
5334	LONRF2	145.0141399	1.79731168	0.390247	4.605572	4.11E-06	1.51E-05
870	AKAP6	315.3314345	1.79536268	0.208736	8.60112	7.89E-18	1.78E-16
1373	PAR5	33.92902894	1.791540727	0.230786	7.762777	8.31E-15	1.19E-13
2119	ASPA	366.150302	1.790346646	0.262108	6.830565	8.46E-12	7.82E-11
154	ACADSB	1232.664708	1.789875458	0.154139	11.61209	3.58E-31	4.55E-29
3160	THSD7B	90.31184193	1.785940165	0.302094	5.911878	3.38E-09	2.10E-08
877	RAB40B	556.1327045	1.783205599	0.207888	8.577739	9.68E-18	2.16E-16
10426	PCDHB1	1.490634797	1.780780074	0.711802	2.50179	0.012357	0.023231
1226	ASB9	175.3666667	1.778295006	0.22271	7.984809	1.41E-15	2.25E-14
464	C4orf12	103.8405066	1.775872825	0.18215	9.749512	1.85E-22	7.82E-21
9037	CKMT2	533.0007873	1.77436127	0.59154	2.999564	0.002704	0.005864
889	ATP8A1	788.0314466	1.772333307	0.207358	8.547233	1.26E-17	2.78E-16
1534	OTUD7A	28.8882432	1.771269636	0.23572	7.514284	5.72E-14	7.31E-13
3632	CHAD	19.91716097	1.76628202	0.316415	5.582168	2.38E-08	1.28E-07
5804	SOX8	42.92335264	1.765501316	0.403721	4.373075	1.23E-05	4.14E-05
1075	CIT	1467.316323	1.764710217	0.215192	8.200629	2.39E-16	4.36E-15
476	C11orf54	3482.509305	1.764086418	0.181753	9.705967	2.84E-22	1.17E-20
3044	ADCY5	590.6728985	1.76385191	0.293759	6.004419	1.92E-09	1.24E-08
2660	CCDC68	255.4069869	1.763810341	0.279129	6.318983	2.63E-10	1.94E-09
561	PVRL3	292.0693624	1.760957708	0.18668	9.433031	3.98E-21	1.39E-19
5175	ABCA8	159.0410853	1.76093713	0.375618	4.688104	2.76E-06	1.04E-05
3749	FHDC1	210.0570351	1.758169392	0.319298	5.506356	3.66E-08	1.91E-07
8371	BEX1	79.28186067	1.757796738	0.53874	3.262793	0.001103	0.002583
3172	CASP12	13.30639554	1.753871177	0.297144	5.902432	3.58E-09	2.21E-08
10587	SLC7A14	1.312955947	1.75319114	0.718536	2.439949	0.014689	0.027196
4392	GADD45G	232.6719786	1.752932761	0.343172	5.108034	3.26E-07	1.45E-06
11103	PYY	1.887183359	1.752550961	0.777718	2.253454	0.024231	0.042776
8576	ZNF676	18.61212805	1.750443839	0.550361	3.180536	0.00147	0.00336
2409	EGOT	103.2429561	1.749765211	0.267108	6.55077	5.72E-11	4.66E-10
11461	GATSL2	1.446054757	1.748989057	0.82059	2.13138	0.033058	0.056534
8385	PPP1R1C	5.67744095	1.745942858	0.535964	3.257573	0.001124	0.002627
1815	FBP1	941.951908	1.74566381	0.243327	7.174138	7.28E-13	7.86E-12
7987	RTP2	4.281638658	1.74434236	0.510855	3.414558	0.000639	0.001568
337	ITGA6	5463.652438	1.742189243	0.167937	10.37407	3.25E-25	1.89E-23
2882	KCNJ15	1513.622668	1.741540409	0.284412	6.123305	9.17E-10	6.23E-09
1186	PABPC4L	204.3995673	1.740455572	0.216654	8.033348	9.48E-16	1.57E-14
9265	KIF1A	5.852340257	1.74007131	0.597697	2.911295	0.003599	0.007615
1140	ABARAPL	3718.21495	1.738791822	0.214507	8.105977	5.23E-16	9.00E-15
6726	ZNF556	6.438808538	1.737932283	0.442019	3.931805	8.43E-05	0.000246
7401	DEFB1	4684.57575	1.737678016	0.475218	3.656588	0.000256	0.000677
10117	KCNG3	2.15109823	1.737556617	0.66581	2.609688	0.009062	0.017559
3968	ERVFRDE1	6.558140398	1.735972044	0.323805	5.361169	8.27E-08	4.08E-07
7002	COCH	91.50414515	1.735642044	0.453992	3.823071	0.000132	0.000369
802	KLHL13	311.4282513	1.734885289	0.198267	8.750243	2.13E-18	5.20E-17
4629	C9orf106	2.936205091	1.732186294	0.348243	4.974075	6.56E-07	2.78E-06

2811	VIPR1	33.88971352	1.730170835	0.279989	6.179433	6.43E-10	4.49E-09
3920	NPY6R	303.2777063	1.729743567	0.320895	5.390371	7.03E-08	3.51E-07
1221	KIF13B	1934.565733	1.729291423	0.216373	7.992194	1.33E-15	2.13E-14
755	SAMD5	340.2245827	1.726892915	0.194572	8.87534	6.97E-19	1.81E-17
377	BCKDHB	434.1614775	1.726004891	0.17035	10.1321	3.98E-24	2.07E-22
6467	GPM6A	298.931836	1.722525546	0.426063	4.042892	5.28E-05	0.00016
12297	APOA2	0.847374194	1.722048814	0.922534	1.866651	0.06195	0.098747
10276	C3orf50	0.77655359	1.719587552	0.673035	2.554976	0.01062	0.020256
7141	SLC5A11	6.506020796	1.717780881	0.456065	3.766522	0.000166	0.000454
455	GK	524.8752238	1.717206135	0.175509	9.784161	1.32E-22	5.67E-21
11347	FAM123C	0.96346051	1.717198775	0.792582	2.166589	0.030266	0.052282
5261	TNFAIP6	1203.847043	1.716860995	0.369594	4.645265	3.40E-06	1.27E-05
11682	CST5	5.127648955	1.716204754	0.834903	2.055575	0.039824	0.066819
1106	PDGFD	1793.510457	1.716132043	0.210473	8.1537	3.53E-16	6.26E-15
4350	TAL2	18.34065383	1.715695249	0.334381	5.130962	2.88E-07	1.30E-06
3463	DIRAS2	585.4843069	1.715227857	0.301041	5.697655	1.21E-08	6.87E-08
684	ZNF844	247.7729199	1.715148529	0.189536	9.04922	1.44E-19	4.13E-18
10512	PRR18	1.767524665	1.714329376	0.694634	2.467962	0.013588	0.025337
1555	LNX1	329.0100697	1.706481292	0.227899	7.487882	7.00E-14	8.82E-13
465	SYDE2	90.60032641	1.706198182	0.175005	9.749429	1.86E-22	7.82E-21
4755	PDE1C	147.0424572	1.705866653	0.347675	4.906492	9.27E-07	3.82E-06
1313	CACNB2	132.6366081	1.704384708	0.217271	7.844508	4.35E-15	6.49E-14
8905	SRMS	1.367848497	1.704159077	0.558718	3.050126	0.002287	0.005035
6146	SLC2A2	952.3103005	1.703481221	0.405253	4.203503	2.63E-05	8.38E-05
6956	FABP4	238.3623358	1.702853389	0.443045	3.843524	0.000121	0.000342
1370	PLEKHH1	445.0723394	1.701546335	0.219131	7.764981	8.17E-15	1.17E-13
3147	NEBL	1286.660218	1.699802212	0.286897	5.924787	3.13E-09	1.95E-08
2177	EDNRB	3521.061608	1.699168903	0.250557	6.781578	1.19E-11	1.07E-10
387	PLEKHA7	649.6974239	1.69874813	0.16819	10.10017	5.51E-24	2.79E-22
8075	TREH	31.04998842	1.698583507	0.502293	3.381656	0.000721	0.001749
3305	SSTR1	78.29178597	1.69823281	0.292163	5.812628	6.15E-09	3.65E-08
11108	CPN1	11.10559719	1.6972357	0.754247	2.25024	0.024434	0.043115
6421	LOC440173	125.7781162	1.697123419	0.417397	4.065965	4.78E-05	0.000146
1874	ELOVL7	1790.470157	1.696263922	0.238496	7.112331	1.14E-12	1.19E-11
567	C14orf105	1154.268185	1.696159003	0.180202	9.412552	4.84E-21	1.67E-19
1628	CMTM4	1822.606007	1.694024836	0.229222	7.390337	1.46E-13	1.76E-12
659	TBC1D4	1495.955095	1.692293048	0.184971	9.148978	5.75E-20	1.71E-18
3777	PLLP	682.0307709	1.690100203	0.307927	5.48864	4.05E-08	2.10E-07
2643	PDZK1	3372.107665	1.68782831	0.26665	6.329745	2.46E-10	1.82E-09
779	C5orf30	283.9476365	1.686847968	0.191459	8.810507	1.25E-18	3.13E-17
5506	C9orf24	23.69100876	1.683648001	0.372811	4.516086	6.30E-06	2.24E-05
12163	C16orf11	0.797100386	1.683616734	0.882587	1.907593	0.056444	0.090953
2037	C9orf66	179.3061807	1.680046551	0.242699	6.922354	4.44E-12	4.27E-11
4875	ASXL3	41.47406316	1.679467132	0.346723	4.843826	1.27E-06	5.12E-06
3458	KATNAL2	127.1015752	1.679407124	0.294582	5.70098	1.19E-08	6.75E-08
3985	GMPR	245.9330243	1.679275911	0.313775	5.351856	8.71E-08	4.28E-07
981	EHHADH	3108.346282	1.678766447	0.200504	8.372717	5.63E-17	1.13E-15

7376	CDH20	3.224896107	1.678331804	0.457667	3.667149	0.000245	0.000652
4448	GDF6	277.2055217	1.67641278	0.33042	5.07358	3.90E-07	1.72E-06
2981	AQP1	12678.20892	1.674063814	0.276745	6.049115	1.46E-09	9.58E-09
3164	DACH1	160.7276483	1.67259842	0.283047	5.909267	3.44E-09	2.13E-08
3894	CYS1	1563.217824	1.671391301	0.309108	5.40715	6.40E-08	3.22E-07
750	APOOL	229.1025089	1.670331206	0.187985	8.885468	6.37E-19	1.66E-17
13399	SALL3	0.783797182	1.670086301	1.104509	1.512062	0.130518	0.190931
3578	LINGO4	17.83005004	1.668919343	0.297068	5.617971	1.93E-08	1.06E-07
12236	SLC6A15	17.54219448	1.667579153	0.883968	1.88647	0.059232	0.094884
8541	C1orf187	2.882891067	1.666953356	0.522132	3.192592	0.00141	0.003236
11059	ABCC13	1.348353423	1.66579529	0.733631	2.270619	0.02317	0.041067
5443	ALDH8A1	1015.815546	1.664488751	0.366246	4.544724	5.50E-06	1.98E-05
13513	CEACAM7	0.657852774	1.664096271	1.122009	1.48314	0.138037	0.200227
8357	LRRC31	24.04133648	1.661993979	0.508521	3.268289	0.001082	0.002538
6572	C21orf62	122.6824046	1.66186976	0.416114	3.993785	6.50E-05	0.000194
3774	MLANA	2.815593794	1.661152325	0.30257	5.49015	4.02E-08	2.09E-07
1679	DSG2	4899.644769	1.660745346	0.2268	7.322523	2.43E-13	2.84E-12
10143	ZNF705A	2.616819312	1.660337555	0.638461	2.600533	0.009308	0.017987
2704	THRB	384.655084	1.658845525	0.264286	6.276705	3.46E-10	2.51E-09
2139	DNAJA4	704.4029404	1.657976378	0.243306	6.814363	9.47E-12	8.68E-11
7016	CYSLTR2	198.4770285	1.657543393	0.434278	3.816778	0.000135	0.000378
605	C7orf41	807.1457659	1.657458369	0.178231	9.299516	1.41E-20	4.57E-19
6788	PTPN20B	87.23960891	1.657356965	0.424528	3.903996	9.46E-05	0.000273
568	MTSS1	1902.712031	1.656524232	0.176021	9.410921	4.92E-21	1.70E-19
588	CEACAM1	463.1745007	1.655440888	0.176884	9.358921	8.06E-21	2.69E-19
611	MSI2	335.1793001	1.655355761	0.178167	9.291022	1.53E-20	4.90E-19
8985	DACH2	3.33969726	1.655142263	0.548068	3.019958	0.002528	0.005515
6423	MDGA2	4.770179373	1.653912524	0.406895	4.06472	4.81E-05	0.000147
185	PRKAA2	1443.295751	1.653617237	0.145377	11.37471	5.59E-30	5.95E-28
7402	DIO1	47.54895152	1.653553938	0.45224	3.656365	0.000256	0.000677
1994	ALDH1B1	1352.457987	1.652102822	0.237097	6.968045	3.21E-12	3.16E-11
3286	NECAB1	222.8051626	1.650535814	0.283445	5.823119	5.78E-09	3.45E-08
2533	CRNA0008	70.45746534	1.650430021	0.256401	6.436921	1.22E-10	9.43E-10
460	ACSL1	7135.529579	1.649178687	0.168913	9.763471	1.62E-22	6.88E-21
3886	C1orf172	65.2920332	1.649012634	0.304792	5.410297	6.29E-08	3.17E-07
721	GOT1	2659.634427	1.64818086	0.18383	8.965774	3.08E-19	8.38E-18
6327	ZSCAN4	3.073820326	1.646620744	0.400164	4.114866	3.87E-05	0.00012
3425	ASB16	24.18558111	1.640910122	0.286599	5.725459	1.03E-08	5.90E-08
8881	ART3	2.24241376	1.64067658	0.536332	3.059066	0.00222	0.0049
911	ENTPD5	526.4984306	1.638637364	0.192826	8.498018	1.93E-17	4.15E-16
2539	PDZRN3	227.5793058	1.638165468	0.254702	6.431699	1.26E-10	9.74E-10
3341	SLC19A2	621.3790399	1.637568299	0.282738	5.791821	6.96E-09	4.08E-08
693	ZNF189	1277.409363	1.635816246	0.181053	9.034997	1.64E-19	4.64E-18
5569	TMIGD1	6.773928214	1.635491196	0.364319	4.489177	7.15E-06	2.52E-05
959	GK3P	54.30282728	1.634540632	0.19391	8.429393	3.47E-17	7.10E-16
5296	PLCH2	209.0630723	1.634465416	0.353516	4.623456	3.77E-06	1.40E-05
3017	TPPP	557.723228	1.628607782	0.270452	6.021803	1.72E-09	1.12E-08

7168	KCNMB2	52.26056648	1.624942539	0.432617	3.756076	0.000173	0.000472
827	HOOK1	1556.687422	1.624752133	0.187063	8.685594	3.77E-18	8.93E-17
191	ACADM	1864.197225	1.624673744	0.143674	11.30808	1.20E-29	1.23E-27
3301	USP44	5.148842637	1.623038353	0.279151	5.814185	6.09E-09	3.62E-08
536	TTC39B	184.1366743	1.622918753	0.170691	9.507918	1.95E-21	7.11E-20
1597	ZNF204P	210.766248	1.622517716	0.218482	7.426335	1.12E-13	1.37E-12
3855	CCDC150	141.8415185	1.622359479	0.298817	5.429274	5.66E-08	2.88E-07
814	ABCC4	1767.082443	1.621682376	0.185923	8.722334	2.73E-18	6.56E-17
1381	PPFIBP2	951.8688486	1.621391832	0.209303	7.746638	9.44E-15	1.34E-13
9027	KCTD8	1.678594382	1.620255873	0.539436	3.003612	0.002668	0.005793
4724	ARHGAP8	154.9781921	1.619399639	0.329103	4.920645	8.63E-07	3.58E-06
1325	IQGAP2	1325.079544	1.617787114	0.206619	7.829827	4.89E-15	7.22E-14
6868	CECR2	22.13760229	1.614864261	0.416629	3.876027	0.000106	0.000303
1702	PLS1	767.0801417	1.613601697	0.22124	7.293459	3.02E-13	3.48E-12
6188	A1CF	137.929169	1.61275977	0.385729	4.181066	2.90E-05	9.19E-05
6955	)C1001278	5.262317907	1.612355683	0.419479	3.843706	0.000121	0.000342
2637	C8orf79	254.527697	1.612067884	0.254393	6.336915	2.34E-10	1.74E-09
2523	AHCYL2	1258.67014	1.610983904	0.249995	6.444072	1.16E-10	9.03E-10
6139	RIMBP2	8.919750458	1.609363548	0.382688	4.205415	2.61E-05	8.32E-05
1587	SH2D4A	329.1997779	1.607356826	0.216062	7.439341	1.01E-13	1.25E-12
3967	FAM190A	29.76371386	1.60685863	0.299666	5.362169	8.22E-08	4.06E-07
747	BRP44L	996.5377643	1.606817369	0.180549	8.899609	5.60E-19	1.47E-17
5885	RERGL	47.89287933	1.606272677	0.370925	4.330455	1.49E-05	4.96E-05
639	DDAH1	3356.837729	1.605943794	0.174648	9.195306	3.74E-20	1.15E-18
2970	C4orf19	359.6163999	1.605880895	0.265159	6.056301	1.39E-09	9.19E-09
10755	C15orf55	0.452768072	1.6052422	0.674583	2.379607	0.017331	0.031586
165	HIBCH	905.060949	1.604436452	0.13864	11.57265	5.67E-31	6.74E-29
4300	SHROOM3	661.825381	1.604191147	0.311104	5.15644	2.52E-07	1.15E-06
2629	SPARCL1	14842.21	1.60339695	0.252405	6.352467	2.12E-10	1.58E-09
4244	CRIP3	19.44523219	1.6019267	0.308943	5.185184	2.16E-07	9.97E-07
995	PLCL2	586.7604333	1.60151069	0.191665	8.355793	6.50E-17	1.28E-15
13567	CER1	0.292302664	1.600789041	1.092745	1.464924	0.142942	0.206516
683	CAB39L	373.4463783	1.600066856	0.176722	9.054164	1.38E-19	3.95E-18
9998	MOGAT1	1.901612005	1.600026307	0.603002	2.653433	0.007968	0.015621
3526	CDH1	2821.015536	1.598815922	0.282732	5.654885	1.56E-08	8.67E-08
6268	TTLL10	2.153987056	1.598319565	0.385282	4.148444	3.35E-05	0.000105
1098	PDP2	149.1732675	1.597066307	0.195662	8.162392	3.28E-16	5.86E-15
4738	OSTalpha	91.51981649	1.596756289	0.324801	4.9161	8.83E-07	3.65E-06
955	NDRG2	2121.787184	1.595525079	0.189158	8.434859	3.32E-17	6.81E-16
5785	ELAVL3	7.70239939	1.593113518	0.363815	4.378908	1.19E-05	4.04E-05
4267	HRH2	257.1681169	1.590225063	0.307451	5.172286	2.31E-07	1.06E-06
511	IMPA2	2811.661769	1.590050484	0.166325	9.559893	1.18E-21	4.52E-20
3852	C12orf34	106.3671132	1.589949503	0.292689	5.432214	5.57E-08	2.83E-07
106	ZNRF3	615.6228988	1.588892639	0.130041	12.21844	2.48E-34	4.58E-32
4942	PCDHGB5	151.3111909	1.588187497	0.33064	4.803376	1.56E-06	6.19E-06
5457	LIPC	435.9914742	1.58430591	0.348974	4.539897	5.63E-06	2.02E-05
310	ARHGAP24	1366.348836	1.583421887	0.151124	10.47764	1.09E-25	6.92E-24

3819	CGN	308.9218592	1.582934239	0.290028	5.457875	4.82E-08	2.47E-07
5765	UST	201.2323288	1.5827596	0.360632	4.388847	1.14E-05	3.87E-05
828	ALDH5A1	924.9356337	1.582699372	0.182328	8.680511	3.94E-18	9.33E-17
392	MEGF9	1121.153678	1.578977332	0.156528	10.0875	6.27E-24	3.14E-22
5624	NLRP6	55.83520964	1.575927475	0.353403	4.459291	8.22E-06	2.87E-05
4429	MUC4	37.63383719	1.573040731	0.309371	5.084635	3.68E-07	1.63E-06
3285	ST3GAL4	898.5993541	1.57253204	0.270004	5.824104	5.74E-09	3.43E-08
1231	NCKAP5	160.9381486	1.572153299	0.19736	7.965917	1.64E-15	2.61E-14
15823	GFRA4	0.35097204	1.571507571	2.045272	0.768361	0.442273	0.547872
15045	FGF19	0.371462313	1.570393236	1.580745	0.993451	0.32049	0.417543
2007	MARVELD	178.0722946	1.569007033	0.225752	6.950147	3.65E-12	3.56E-11
2072	SDPR	1768.759993	1.568028831	0.22803	6.876425	6.14E-12	5.81E-11
4787	PLAG1	60.15743766	1.567991295	0.3205	4.892327	9.97E-07	4.08E-06
6537	CCDC151	79.9725983	1.567455518	0.390894	4.00992	6.07E-05	0.000182
3294	NPNT	1240.904442	1.56643004	0.269155	5.819799	5.89E-09	3.51E-08
9254	CPLX3	2.831614462	1.566240287	0.53712	2.915996	0.003546	0.00751
9431	CRNA0011	2.348218757	1.566079987	0.550238	2.846186	0.004425	0.009196
10940	FLJ32063	0.413378827	1.563581928	0.677802	2.306842	0.021064	0.037739
1292	CDKL2	164.6695409	1.563468165	0.198562	7.873966	3.44E-15	5.21E-14
6795	HRASLS5	47.31138029	1.562834518	0.400569	3.901535	9.56E-05	0.000276
3611	SCD5	509.1352325	1.562680135	0.278978	5.601455	2.13E-08	1.15E-07
1553	SFXN2	324.1911018	1.561549051	0.208525	7.488544	6.96E-14	8.79E-13
10597	GPR61	1.350775793	1.560701272	0.640473	2.436795	0.014818	0.027409
6782	KCNQ4	10.99187295	1.559866538	0.399374	3.905776	9.39E-05	0.000271
2025	RNF180	401.5452278	1.559430646	0.224858	6.935187	4.06E-12	3.93E-11
251	ANKRD57	760.1305547	1.556154541	0.143648	10.8331	2.40E-27	1.87E-25
5751	HSD17B13	16.99578135	1.551618045	0.352931	4.396377	1.10E-05	3.75E-05
123	CRY2	1665.998396	1.551429425	0.129244	12.00389	3.39E-33	5.40E-31
2748	EMCN	1213.643917	1.55110556	0.248562	6.240305	4.37E-10	3.12E-09
2402	ProSAPiP1	439.0199279	1.550848815	0.23648	6.558054	5.45E-11	4.45E-10
4502	CENPV	79.53204818	1.5505495	0.307799	5.037546	4.72E-07	2.05E-06
7394	CTSL3	4.184353788	1.550082957	0.423639	3.658968	0.000253	0.000671
6526	IYD	48.55679154	1.54931034	0.385867	4.015138	5.94E-05	0.000178
368	C12orf49	860.7470521	1.549098968	0.152022	10.18995	2.20E-24	1.17E-22
6837	RIC3	66.76114852	1.548603712	0.398633	3.884784	0.000102	0.000294
166	ALDH3A2	2777.504976	1.548574613	0.133934	11.56222	6.40E-31	7.56E-29
9081	C9orf128	1.20454943	1.548378881	0.518473	2.986423	0.002823	0.006093
8755	DDX25	2.115739149	1.546268016	0.498115	3.10424	0.001908	0.004271
941	COBLL1	1594.935344	1.540994512	0.182163	8.459427	2.69E-17	5.59E-16
878	LMO7	1642.339038	1.538977519	0.179439	8.576593	9.77E-18	2.18E-16
6625	LOXHD1	25.9546819	1.538772315	0.387186	3.974243	7.06E-05	0.000209
12813	TPPP2	0.364641999	1.534346398	0.902002	1.701045	0.088935	0.13605
2290	C6orf217	10.45167558	1.534150876	0.230231	6.66353	2.67E-11	2.29E-10
4376	CATSPERG	27.65213928	1.532867578	0.29956	5.11706	3.10E-07	1.39E-06
5483	PEBP4	3.932937737	1.532792095	0.338418	4.529283	5.92E-06	2.12E-05
2891	KITLG	1030.004182	1.531110722	0.250313	6.116787	9.55E-10	6.47E-09
855	CDKL1	106.6219421	1.529534103	0.177213	8.631026	6.08E-18	1.39E-16

883	LGR4	2462.67429	1.528104675	0.178489	8.561326	1.12E-17	2.48E-16
1184	MAGI3	306.6270645	1.527691449	0.189894	8.044975	8.63E-16	1.43E-14
2521	BEX5	133.1086012	1.527648332	0.236992	6.445991	1.15E-10	8.93E-10
5348	NRG1	135.0207107	1.52749489	0.332495	4.594032	4.35E-06	1.59E-05
156	MUT	1048.397271	1.526856887	0.131568	11.60504	3.89E-31	4.88E-29
6966	GRM1	10.51778243	1.526832326	0.397546	3.840641	0.000123	0.000345
3181	DNASE1L3	99.64295485	1.52433808	0.258627	5.893973	3.77E-09	2.32E-08
2626	COBL	600.7850674	1.522970952	0.239716	6.353234	2.11E-10	1.57E-09
9533	SLC30A2	57.70327455	1.52284284	0.5429	2.805016	0.005031	0.010345
3584	RNF43	93.92391613	1.522356498	0.271074	5.616028	1.95E-08	1.07E-07
3600	PRUNE2	5262.122088	1.51838072	0.270887	5.605216	2.08E-08	1.13E-07
6137	UGT2A3	2767.76612	1.515324392	0.360306	4.205654	2.60E-05	8.31E-05
15129	ADIG	0.315756367	1.514796524	1.569959	0.964864	0.334613	0.433522
728	ENPP4	1092.607162	1.51422638	0.169264	8.945965	3.69E-19	9.93E-18
6453	OC1002407	4.35482739	1.51270033	0.373721	4.047675	5.17E-05	0.000157
2322	RCAN1	3167.565026	1.512644588	0.22783	6.639352	3.15E-11	2.66E-10
3406	CDC42BPG	127.3683803	1.509300805	0.262828	5.742533	9.33E-09	5.37E-08
9279	FOXJ1	18.78214607	1.508189339	0.518901	2.906508	0.003655	0.007721
10339	C6orf103	0.635823919	1.508004844	0.595201	2.533606	0.01129	0.021403
5788	SLC44A4	285.9261855	1.507924659	0.344373	4.378755	1.19E-05	4.04E-05
5000	NOS1AP	16.01816113	1.507303759	0.315711	4.774312	1.80E-06	7.07E-06
3527	GATA2	315.1311536	1.501686803	0.265653	5.652812	1.58E-08	8.77E-08
284	EPB41L5	614.1576075	1.500872457	0.140914	10.651	1.73E-26	1.19E-24
1826	FAM63B	433.1823055	1.50070347	0.209416	7.166146	7.71E-13	8.28E-12
2653	B4GALT6	217.1988118	1.500049571	0.237241	6.322899	2.57E-10	1.90E-09
1783	OC1003037	30.32445587	1.498262299	0.207964	7.204422	5.83E-13	6.41E-12
6897	AGXT2	1131.500839	1.498006964	0.387536	3.865462	0.000111	0.000315
320	ZDHHC21	197.0118549	1.497722717	0.143399	10.44441	1.55E-25	9.52E-24
71	ETFDH	798.6613298	1.496847461	0.117986	12.68666	7.01E-37	1.94E-34
9366	CNGB1	2.395299309	1.496346238	0.520697	2.873736	0.004056	0.008489
2203	DHRS7	2508.923046	1.493750871	0.221249	6.751461	1.46E-11	1.30E-10
4607	EPCAM	1314.748375	1.493534556	0.299539	4.986107	6.16E-07	2.62E-06
3366	C7orf10	177.4683955	1.492635619	0.258778	5.768006	8.02E-09	4.67E-08
9251	C9orf79	0.54223194	1.492392505	0.51155	2.917393	0.00353	0.007479
7536	LOC221122	1.925472313	1.491899289	0.414323	3.600813	0.000317	0.000825
10491	BMP5	10.32960481	1.491559105	0.602827	2.474273	0.013351	0.024944
10057	ARPP21	10.62200417	1.487124088	0.564758	2.633206	0.008458	0.016485
293	CAT	4230.385827	1.487036686	0.140173	10.60861	2.72E-26	1.82E-24
8346	PCDH20	47.64135554	1.485378699	0.453532	3.275139	0.001056	0.00248
7333	MGC14436	4.765038812	1.484897909	0.402668	3.687648	0.000226	0.000605
6598	ATP2B2	383.2206232	1.482925365	0.372227	3.983925	6.78E-05	0.000201
7868	MB	15.01168495	1.482259486	0.42784	3.464514	0.000531	0.001323
4711	AJAP1	92.32330711	1.480723548	0.30046	4.928183	8.30E-07	3.45E-06
10657	KRTAP5-7	0.953039534	1.480482521	0.614117	2.410749	0.01592	0.029281
9105	CYP4Z1	0.95743345	1.480086486	0.497416	2.975551	0.002925	0.006296
10506	C8orf56	2.645767542	1.479219354	0.598999	2.469487	0.013531	0.025244
3320	IQUB	64.41028631	1.479024411	0.254846	5.803593	6.49E-09	3.83E-08

1640	PBX1	1131.682217	1.4779507	0.200439	7.373575	1.66E-13	1.99E-12
1018	EML5	51.26239976	1.477012122	0.177799	8.307186	9.80E-17	1.89E-15
4891	HEATR4	5.733131631	1.476953933	0.305624	4.83259	1.35E-06	5.40E-06
5412	C9orf173	4.377317876	1.476777775	0.323867	4.55983	5.12E-06	1.85E-05
13117	C20orf70	1.117309944	1.474899943	0.918205	1.606285	0.108211	0.161702
12249	CADM2	0.932206726	1.471961924	0.782154	1.881934	0.059845	0.095757
347	ZNF684	102.2192724	1.471492444	0.142779	10.30607	6.62E-25	3.74E-23
323	PPAP2B	5731.34766	1.470767711	0.14088	10.43985	1.63E-25	9.90E-24
9709	LIX1	91.35107324	1.470553751	0.535753	2.744833	0.006054	0.012222
7706	GAS2	132.0950181	1.468410496	0.415007	3.538279	0.000403	0.001024
5919	;ABARAPL	2.302508579	1.467477631	0.340209	4.313462	1.61E-05	5.32E-05
1920	EPHA4	376.6035944	1.465348372	0.207318	7.068134	1.57E-12	1.60E-11
7642	TNFAIP8L3	254.7202898	1.464646348	0.410936	3.564172	0.000365	0.000936
2611	LOC44104(	16.76941414	1.461475978	0.229552	6.366647	1.93E-10	1.45E-09
7400	DDC	1339.335067	1.460453448	0.399383	3.656777	0.000255	0.000677
2652	SPATA18	870.1009159	1.459218972	0.230773	6.323176	2.56E-10	1.89E-09
4374	ILDR1	185.9489678	1.457302226	0.284721	5.11835	3.08E-07	1.38E-06
3021	FUT1	135.3742499	1.453210072	0.241461	6.018399	1.76E-09	1.14E-08
10675	OR10Q1	0.667876785	1.453194257	0.604522	2.403873	0.016222	0.029787
1097	USP51	89.95482993	1.45275591	0.177915	8.165453	3.20E-16	5.72E-15
7259	SYT17	183.2529128	1.45264565	0.390245	3.722392	0.000197	0.000533
4664	HSD3B2	11.88828049	1.452435253	0.29316	4.954414	7.25E-07	3.05E-06
12177	MIMT1	0.39168289	1.452148532	0.762935	1.903372	0.056992	0.091739
178	MYO6	2314.642201	1.451130928	0.127132	11.41434	3.55E-30	3.90E-28
5458	ADCY1	253.4191945	1.451112806	0.319647	4.539741	5.63E-06	2.02E-05
227	SYNJ2BP	1406.420773	1.450604518	0.131984	10.99073	4.23E-28	3.66E-26
202	CPT1A	2784.718751	1.450154783	0.12946	11.20157	4.01E-29	3.89E-27
840	KRBA2	82.08215618	1.450123015	0.167639	8.65025	5.14E-18	1.20E-16
148	MPP5	991.4013543	1.449543318	0.124408	11.65152	2.25E-31	2.99E-29
8035	PAK6	55.92444282	1.449350164	0.426655	3.397008	0.000681	0.001662
224	INADL	1942.173581	1.447543298	0.131579	11.00133	3.77E-28	3.29E-26
15278	CA5A	0.280595793	1.447290569	1.562732	0.926128	0.354379	0.454629
2812	PBLD	1808.798369	1.44595459	0.234025	6.178623	6.47E-10	4.51E-09
865	STXBP1	943.9600637	1.445637511	0.167856	8.612351	7.16E-18	1.62E-16
7780	CD300LG	50.68687248	1.442279896	0.411184	3.507625	0.000452	0.001139
2327	MFAP3L	1037.803274	1.441488128	0.217371	6.631457	3.32E-11	2.80E-10
14787	CRH	0.730666039	1.438339467	1.342556	1.071344	0.284015	0.376478
7664	BCL2L10	10.37471565	1.438291864	0.404821	3.552907	0.000381	0.000974
489	SLC25A30	1095.130871	1.438161011	0.148872	9.660392	4.44E-22	1.78E-20
7374	CRHR2	2.558293503	1.437634125	0.391829	3.669036	0.000243	0.000647
1041	DNAJC28	37.65200213	1.437244687	0.17409	8.255751	1.51E-16	2.84E-15
10571	KRTAP5-2	0.9380923	1.436765949	0.587164	2.446958	0.014407	0.026713
7028	PLIN5	78.19555864	1.436371684	0.377074	3.809254	0.000139	0.000389
4878	NORD116-	57.39120564	1.434838951	0.296246	4.843407	1.28E-06	5.13E-06
2145	C6orf27	80.82388248	1.43468797	0.210598	6.81244	9.60E-12	8.77E-11
4814	OPY19L2P4	13.68964323	1.433161561	0.293582	4.881644	1.05E-06	4.28E-06
5227	LOC157381	2.980550357	1.431709498	0.306882	4.665341	3.08E-06	1.16E-05

7615	C11orf90	1.627013604	1.431592489	0.400362	3.57575	0.000349	0.000899
12377	ATP13A5	0.459553341	1.429686737	0.775942	1.842517	0.0654	0.103565
5170	CLDN10	523.8412077	1.42863302	0.3046	4.690192	2.73E-06	1.03E-05
4769	CLUL1	10.10515427	1.428281355	0.291597	4.898128	9.68E-07	3.98E-06
11546	TMC3	1.071686454	1.427002553	0.679839	2.09903	0.035814	0.0608
1608	CPEB4	2185.955124	1.426903617	0.192455	7.414225	1.22E-13	1.49E-12
6940	CAPN13	48.72443647	1.423709097	0.370016	3.8477	0.000119	0.000337
12285	DAPL1	17.70545999	1.421793188	0.76017	1.870362	0.061434	0.098019
15341	C13orf39	0.337679581	1.421616462	1.566497	0.907513	0.364136	0.465252
2018	MAGI1	784.4159749	1.420733078	0.204735	6.939381	3.94E-12	3.83E-11
14142	TACR3	0.31720469	1.420219216	1.114283	1.274559	0.202466	0.28062
5233	ELOVL4	68.55354426	1.419717944	0.304583	4.661191	3.14E-06	1.18E-05
2583	ARHGAP6	498.2784236	1.419279229	0.221858	6.397236	1.58E-10	1.20E-09
8692	TMEM82	68.51829544	1.419186219	0.452871	3.133753	0.001726	0.003892
820	ZNF433	110.4778992	1.418588746	0.162838	8.711642	3.00E-18	7.16E-17
9572	PRKG2	29.03660791	1.416846936	0.507673	2.790867	0.005257	0.010764
3427	CACNA2D2	35.56690054	1.416663181	0.247499	5.723912	1.04E-08	5.95E-08
1145	TACC2	1014.006608	1.416589343	0.174983	8.09557	5.70E-16	9.76E-15
6068	CNGA1	25.64963737	1.416032963	0.334231	4.236693	2.27E-05	7.33E-05
1953	RGN	387.8288141	1.415475206	0.201223	7.034369	2.00E-12	2.01E-11
5551	C1QTNF9E	4.181382053	1.415141063	0.314558	4.49883	6.83E-06	2.41E-05
14508	C2orf83	0.381298217	1.41395757	1.214706	1.164033	0.244411	0.33021
5539	ANK2	1927.816363	1.413926956	0.313996	4.503015	6.70E-06	2.37E-05
976	FAM135A	631.6496785	1.413457282	0.168628	8.382089	5.20E-17	1.04E-15
12914	DMRT1	0.88824395	1.411910522	0.845964	1.668996	0.095118	0.144371
7089	LOC72860€	1.58769752	1.411479838	0.372491	3.789295	0.000151	0.000418
6160	C19orf77	2289.099687	1.410933687	0.336134	4.19753	2.70E-05	8.59E-05
7586	ANXA13	485.7086179	1.410925762	0.393655	3.584165	0.000338	0.000874
5409	SDCBP2	137.9043009	1.40985866	0.309177	4.560036	5.11E-06	1.85E-05
7023	GABRQ	9.099189374	1.408680766	0.369419	3.813237	0.000137	0.000383
210	KIAA0564	731.8657728	1.4076084	0.126674	11.11206	1.10E-28	1.02E-26
891	ZNF697	404.4377701	1.407353308	0.164758	8.541925	1.32E-17	2.90E-16
4729	ENPP2	8283.258969	1.407304225	0.286109	4.918764	8.71E-07	3.61E-06
930	NUDT12	772.8833576	1.406733423	0.165895	8.479641	2.26E-17	4.76E-16
4004	LOC90246	41.60073611	1.406509649	0.263448	5.338845	9.35E-08	4.58E-07
836	RDH11	2977.007664	1.406391281	0.162317	8.664457	4.54E-18	1.06E-16
1213	MOBKL2B	911.3683943	1.406034787	0.175748	8.000303	1.24E-15	2.01E-14
3377	OC1001321	15.01549727	1.405359709	0.243897	5.762106	8.31E-09	4.82E-08
5194	CDH16	5920.607893	1.40498196	0.300256	4.679274	2.88E-06	1.09E-05
11074	ZNF99	1.027952391	1.404811247	0.620202	2.265086	0.023507	0.041608
3937	GOLM1	5109.777703	1.404722241	0.261068	5.380676	7.42E-08	3.69E-07
6531	SLC7A9	323.8765338	1.403703878	0.34982	4.012643	6.00E-05	0.00018
7766	SLC17A2	93.70260368	1.403609093	0.399763	3.511103	0.000446	0.001126
7282	CYP3A7	54.52000042	1.403179191	0.378497	3.707238	0.00021	0.000564
3706	LRRTM2	10.71824498	1.402362337	0.253429	5.533547	3.14E-08	1.66E-07
9581	PIGR	4397.300244	1.40173205	0.502857	2.787536	0.005311	0.010865
608	PHYH	1415.050342	1.40070683	0.150708	9.294168	1.48E-20	4.78E-19

4080	GPLD1	16.29503695	1.40056692	0.264674	5.291672	1.21E-07	5.82E-07
1574	ASAH1	8130.735045	1.399184663	0.187494	7.462554	8.49E-14	1.06E-12
7309	SLC4A11	70.72728991	1.399106838	0.378379	3.69763	0.000218	0.000584
10870	B4GALNT2	33.70126731	1.396850851	0.598785	2.33281	0.019658	0.035448
1147	BCL2	1840.928032	1.394818255	0.172386	8.091227	5.91E-16	1.01E-14
5435	BDNF	65.93373533	1.393852622	0.306295	4.55068	5.35E-06	1.93E-05
99	SUCLA2	1041.20946	1.392971178	0.112956	12.33196	6.09E-35	1.21E-32
3229	IMPG1	6.171476024	1.38953632	0.236879	5.866008	4.46E-09	2.71E-08
1116	HHAT	290.5343548	1.38905907	0.170672	8.138786	3.99E-16	7.01E-15
6114	GPR17	16.83092221	1.387832569	0.329292	4.214592	2.50E-05	8.02E-05
2478	RUNDC2A	153.7442598	1.387542224	0.213771	6.490775	8.54E-11	6.75E-10
9756	VTN	84.70243688	1.386674064	0.507864	2.730402	0.006326	0.012709
188	HSDL2	2106.312742	1.386470213	0.122441	11.32356	1.00E-29	1.05E-27
7177	LGSN	9.100550732	1.385622183	0.369206	3.752974	0.000175	0.000477
163	OSBPL1A	1624.551752	1.38135688	0.119271	11.58169	5.10E-31	6.14E-29
10233	HORMAD2	2.220857925	1.381193319	0.537196	2.571118	0.010137	0.019417
1739	CDC14C	9.546501196	1.381067264	0.190413	7.253017	4.08E-13	4.59E-12
7301	ANGPT4	5.626496365	1.378289721	0.372534	3.69977	0.000216	0.000579
4677	GPR113	13.64178428	1.37813727	0.278641	4.945923	7.58E-07	3.18E-06
920	C10orf118	471.5002491	1.377883391	0.162258	8.491908	2.03E-17	4.33E-16
9079	C7orf16	0.9327423	1.37766723	0.461223	2.98699	0.002817	0.006083
8217	KCNH6	52.80934003	1.377103626	0.414121	3.325366	0.000883	0.002106
1335	MSRA	506.8471079	1.37609049	0.176054	7.816305	5.44E-15	7.99E-14
285	TMEM192	892.5700245	1.375446054	0.12918	10.64753	1.79E-26	1.23E-24
4369	BTBD8	10.08001895	1.374749887	0.268501	5.120096	3.05E-07	1.37E-06
14837	PSG8	0.905745265	1.374331081	1.302003	1.055552	0.291173	0.384666
5943	CCDC144A	36.58412957	1.37409783	0.319855	4.296001	1.74E-05	5.74E-05
10058	FOXA3	8.067035352	1.373658092	0.521713	2.632976	0.008464	0.016495
1290	KIAA1161	237.4286407	1.373226137	0.174364	7.875635	3.39E-15	5.15E-14
7884	B3GALT1	2.67895824	1.371909982	0.397037	3.455368	0.00055	0.001366
1515	WLS	1457.053816	1.371890522	0.181729	7.549093	4.38E-14	5.67E-13
5494	SLCO4C1	2275.728834	1.371578431	0.30314	4.524577	6.05E-06	2.16E-05
4651	C1QTNF9	4.375714146	1.370541688	0.276311	4.960138	7.04E-07	2.97E-06
3461	C11orf93	101.0107142	1.369521275	0.240303	5.699132	1.20E-08	6.82E-08
1432	SATB2	394.1270024	1.368924207	0.178672	7.661661	1.84E-14	2.51E-13
195	PTAR1	1545.511475	1.367885524	0.121212	11.2851	1.55E-29	1.56E-27
2287	ATP11A	5545.045051	1.367862551	0.205182	6.66658	2.62E-11	2.24E-10
4186	C6orf186	19.15742582	1.365657645	0.261759	5.217231	1.82E-07	8.50E-07
1124	ATP7B	395.2128932	1.364306611	0.167919	8.124767	4.48E-16	7.82E-15
475	FANCC	432.7277682	1.363496203	0.140347	9.715204	2.60E-22	1.07E-20
2446	ZNF704	274.1288747	1.362844488	0.209222	6.513882	7.32E-11	5.87E-10
7079	AVPR1A	141.6827578	1.362375647	0.359289	3.79187	0.00015	0.000414
471	MUDENG	882.6220131	1.361457451	0.139846	9.735432	2.13E-22	8.86E-21
2328	PFKM	1802.707876	1.361199163	0.20533	6.629323	3.37E-11	2.84E-10
9776	TAAR1	0.588754231	1.360917108	0.499651	2.723735	0.006455	0.012942
2664	SLC25A5	6742.672342	1.359234503	0.215178	6.316784	2.67E-10	1.96E-09
7950	ESPNP	4.094527905	1.357469371	0.395946	3.42842	0.000607	0.001497

3469	GPC4	1190.045447	1.356846145	0.238243	5.695223	1.23E-08	6.96E-08
11525	ELF5	3.326245806	1.356467944	0.643082	2.109325	0.034917	0.059384
860	SECISBP2L	2088.153648	1.356325568	0.15736	8.619274	6.74E-18	1.54E-16
5612	MAN1C1	447.0652251	1.354537543	0.303273	4.466392	7.95E-06	2.78E-05
2142	CISH	891.0161366	1.353974902	0.198728	6.81321	9.54E-12	8.73E-11
2438	TBC1D1	3258.159695	1.353089895	0.207481	6.521507	6.96E-11	5.60E-10
881	ATP5A1	11597.94911	1.352678122	0.157888	8.567341	1.06E-17	2.36E-16
10265	SV2C	0.506304643	1.351055391	0.527777	2.559899	0.01047	0.019993
9840	G6PC2	1.83062145	1.350495816	0.498919	2.706845	0.006793	0.013531
7774	MAEL	28.54356054	1.34886277	0.384419	3.508834	0.00045	0.001135
3563	BAMBI	447.5830012	1.346622082	0.239309	5.627116	1.83E-08	1.01E-07
2798	ACOX2	252.2431326	1.346499939	0.217443	6.192425	5.92E-10	4.15E-09
492	NDUFS1	1885.626892	1.346050349	0.13947	9.65119	4.86E-22	1.94E-20
552	PRKCE	449.2335127	1.344934814	0.142249	9.454793	3.24E-21	1.15E-19
3596	ZNF471	115.0885773	1.344558564	0.239805	5.606873	2.06E-08	1.12E-07
8689	WNT11	36.02408033	1.343039983	0.428377	3.135183	0.001717	0.003874
2442	NUAK2	481.7101829	1.342520583	0.206038	6.51589	7.23E-11	5.80E-10
2851	GPRC5B	1324.138598	1.342383905	0.218546	6.142351	8.13E-10	5.59E-09
8255	SYT7	107.8222335	1.340980906	0.405008	3.310999	0.00093	0.002207
4832	CD36	3043.774924	1.339866323	0.274921	4.873641	1.10E-06	4.44E-06
1062	ATP6V1D	1931.250578	1.338673502	0.162831	8.221243	2.01E-16	3.72E-15
4305	TSPYL3	23.30155993	1.338002412	0.259579	5.154512	2.54E-07	1.16E-06
1068	GARNL3	133.3239982	1.33702349	0.162821	8.211617	2.18E-16	4.01E-15
4079	ACAD11	4547.753977	1.336770266	0.252596	5.292126	1.21E-07	5.81E-07
7113	GALR1	8.323614609	1.3356097	0.353361	3.779732	0.000157	0.000433
1259	SACM1L	1328.031239	1.334186227	0.168491	7.918433	2.41E-15	3.74E-14
7410	CACNB4	82.4092033	1.333828893	0.365295	3.65137	0.000261	0.00069
403	ZNF91	421.2319366	1.333710651	0.133122	10.01868	1.26E-23	6.14E-22
397	SESTD1	1007.028007	1.332453504	0.132556	10.05201	9.00E-24	4.44E-22
5259	USP2	701.2885129	1.332275639	0.286733	4.646392	3.38E-06	1.26E-05
7769	PRRG2	38.39164194	1.330941931	0.379199	3.509875	0.000448	0.001131
1749	ECHDC3	771.2375834	1.330275162	0.183585	7.246087	4.29E-13	4.81E-12
11636	C6orf222	5.561806552	1.329125381	0.64329	2.066137	0.038816	0.065385
2718	RBPMS2	296.6812335	1.326881117	0.211768	6.265742	3.71E-10	2.68E-09
3234	C17orf104	18.60647408	1.326361903	0.226222	5.863092	4.54E-09	2.75E-08
11943	SLC5A2	22.93277669	1.326203011	0.670431	1.978136	0.047913	0.078636
4304	RRH	2.366517153	1.325513861	0.257128	5.155083	2.54E-07	1.15E-06
2570	TMEM232	46.80784085	1.325375594	0.206724	6.411324	1.44E-10	1.10E-09
263	PPM1A	1966.271788	1.32455704	0.123038	10.7654	5.01E-27	3.74E-25
6099	CYP39A1	86.94426605	1.324199278	0.313659	4.221777	2.42E-05	7.79E-05
3591	GPRIN3	548.7828454	1.323332703	0.235923	5.609165	2.03E-08	1.11E-07
1210	ABCA5	637.6775468	1.322261849	0.165236	8.002247	1.22E-15	1.98E-14
441	ARHGAP5	2326.270641	1.32179395	0.134259	9.845118	7.20E-23	3.20E-21
12505	OR2G6	0.427020747	1.32127444	0.733044	1.802448	0.071475	0.112034
14925	NXF2	0.408751722	1.319098898	1.28248	1.028553	0.30369	0.398835
7195	WNT6	11.12723249	1.316547504	0.351233	3.748362	0.000178	0.000485
868	TMCC3	1133.718142	1.31576831	0.152904	8.605208	7.62E-18	1.72E-16

642	PTGR2	492.5939445	1.315423202	0.143199	9.186003	4.08E-20	1.25E-18
161	RCBTB1	753.2957166	1.314893769	0.11351	11.58393	4.97E-31	6.05E-29
12059	FRMPD4	3.146333661	1.313624698	0.676468	1.941888	0.052151	0.084767
7862	LOC201651	2.197316491	1.313210036	0.378853	3.466282	0.000528	0.001316
4906	PPM1L	190.8360792	1.312770995	0.272134	4.823981	1.41E-06	5.62E-06
2035	PDE7B	292.6828995	1.312641013	0.189618	6.922563	4.44E-12	4.27E-11
1016	STRBP	441.0930887	1.308859744	0.157485	8.310993	9.49E-17	1.83E-15
5522	PPM1H	641.8960312	1.307995405	0.290028	4.509896	6.49E-06	2.30E-05
87	CBR4	600.8409435	1.30790273	0.104889	12.46941	1.10E-35	2.47E-33
7687	ALB	40.77240457	1.306638041	0.368665	3.544241	0.000394	0.001004
5125	GRIP1	87.86654714	1.306117974	0.277005	4.715136	2.42E-06	9.24E-06
62	FBXO3	1036.264666	1.305487865	0.101365	12.87903	5.91E-38	1.87E-35
8820	GPR37	138.4500724	1.305449549	0.423558	3.082106	0.002055	0.004568
13485	SLC7A13	2.087475425	1.305430183	0.875119	1.491718	0.135773	0.197352
13619	RALYL	2.63981484	1.304777145	0.900359	1.449174	0.147289	0.211984
2817	ABHD2	4660.218423	1.304368311	0.211329	6.172228	6.73E-10	4.69E-09
548	ZNF772	182.2738971	1.304007477	0.137821	9.461629	3.03E-21	1.08E-19
9985	SERPINA5	607.4024246	1.303833895	0.490563	2.657829	0.007865	0.015439
560	SNX30	1101.306703	1.302719469	0.138063	9.435656	3.89E-21	1.36E-19
1343	LIMA1	2702.337089	1.302346724	0.166823	7.806753	5.87E-15	8.56E-14
3222	RORC	1313.262965	1.300747728	0.221487	5.872783	4.29E-09	2.61E-08
1835	DCLK2	279.1615604	1.299411657	0.181409	7.162879	7.90E-13	8.44E-12
1131	PHOSPHO2	50.73381037	1.298849584	0.160034	8.116091	4.81E-16	8.34E-15
4132	DSEL	604.6607255	1.298249892	0.246737	5.261673	1.43E-07	6.77E-07
3136	WDR17	112.5456717	1.298167051	0.218703	5.935758	2.92E-09	1.83E-08
470	VPS13A	1219.124402	1.29768821	0.133276	9.736879	2.10E-22	8.75E-21
8434	KCNF1	65.05992856	1.297122025	0.400923	3.235338	0.001215	0.002824
4834	STEAP2	501.1749881	1.295706751	0.265887	4.873145	1.10E-06	4.45E-06
11310	FAM83F	71.76376831	1.294392422	0.594875	2.175908	0.029562	0.051233
7314	RSPH10B2	4.837783543	1.29248134	0.34977	3.695235	0.00022	0.000589
5952	CACNA1D	258.0940193	1.29200991	0.301195	4.28962	1.79E-05	5.89E-05
1894	ZNF470	203.7402398	1.291520736	0.182123	7.091488	1.33E-12	1.37E-11
2073	NR1D2	1024.519771	1.291101283	0.187762	6.876276	6.14E-12	5.81E-11
2345	CTAGE1	7.638764368	1.290395572	0.19518	6.611311	3.81E-11	3.18E-10
2918	SERPINI1	662.7958071	1.289772783	0.211514	6.0978	1.08E-09	7.22E-09
6553	NRXN3	287.8262137	1.289207983	0.321871	4.005354	6.19E-05	0.000185
15858	SFTPC	0.277511937	1.288628643	1.704351	0.756082	0.4496	0.55572
2928	MYO5B	1306.917816	1.288153823	0.211516	6.090099	1.13E-09	7.55E-09
9794	GYPA	7.611373962	1.287914115	0.473728	2.718679	0.006554	0.013117
8144	AQP4	61.7354576	1.286647842	0.38344	3.355538	0.000792	0.001906
3141	ANKRD29	270.9219916	1.28660312	0.216936	5.930796	3.01E-09	1.88E-08
7106	TIMD4	166.3469578	1.285812263	0.339999	3.781808	0.000156	0.000429
4204	CD9	6516.400063	1.285263137	0.246883	5.20597	1.93E-07	9.00E-07
2292	LIMCH1	2001.028187	1.284188295	0.192746	6.662609	2.69E-11	2.30E-10
742	AMD1	2821.966387	1.283558042	0.14402	8.912365	5.00E-19	1.32E-17
7413	ANKS4B	289.6027624	1.283520951	0.351612	3.650389	0.000262	0.000692
12681	KRT85	0.592665767	1.283181712	0.735979	1.743503	0.081246	0.125581

15642	OPALIN	0.289392979	1.282881697	1.556675	0.824117	0.409873	0.513579
1178	IDH3A	1512.93708	1.282771149	0.159275	8.053791	8.03E-16	1.34E-14
10459	C15orf56	7.95307688	1.282746544	0.515224	2.489687	0.012786	0.023961
3011	TTC9	157.1833683	1.282598465	0.212815	6.026837	1.67E-09	1.09E-08
998	CFL2	1300.15142	1.281015609	0.153403	8.350639	6.79E-17	1.33E-15
9227	MYO3B	37.70554911	1.280850173	0.437367	2.92855	0.003405	0.007234
6480	ANO3	66.70728111	1.280653741	0.317283	4.036308	5.43E-05	0.000164
12133	CD164L2	2.029120563	1.280640905	0.668158	1.916673	0.055279	0.089305
7207	CNDP1	18.71656238	1.279910699	0.342134	3.740966	0.000183	0.000499
9085	OR2C1	0.79277811	1.279093798	0.428541	2.984767	0.002838	0.006123
8161	LOC64698	2.733196836	1.277584719	0.381652	3.347514	0.000815	0.001958
2064	ZNF323	225.8094647	1.277120852	0.185521	6.883975	5.82E-12	5.53E-11
8688	NPTX1	17.48054242	1.276976172	0.4073	3.135221	0.001717	0.003874
1069	AMOT	959.2426098	1.276204717	0.155517	8.206212	2.28E-16	4.18E-15
7140	C6orf223	885.6395055	1.275784588	0.338654	3.76722	0.000165	0.000453
5180	NR6A1	6.003068844	1.275356562	0.272255	4.684427	2.81E-06	1.06E-05
8335	BDH1	309.2162932	1.274228977	0.388652	3.27859	0.001043	0.002453
1733	ZBTB10	714.9221596	1.273747508	0.175383	7.262671	3.80E-13	4.29E-12
1168	MYEF2	691.6629872	1.272860114	0.157825	8.065003	7.32E-16	1.23E-14
13604	TTTY4C	2.917905453	1.272704695	0.87447	1.455402	0.145558	0.20973
4401	SLC1A1	1392.554171	1.271338348	0.249155	5.102602	3.35E-07	1.49E-06
7442	ACMSD	1072.193725	1.270985239	0.349232	3.639369	0.000273	0.00072
4251	CCDC64	870.8481528	1.270866802	0.245284	5.181196	2.20E-07	1.02E-06
5470	C1orf226	102.375577	1.270082246	0.280131	4.533887	5.79E-06	2.08E-05
8113	SERPINA6	538.6640376	1.269729884	0.377362	3.364752	0.000766	0.001851
10709	FOLR3	6.752331503	1.269446799	0.530266	2.393979	0.016667	0.030506
373	TJP2	1638.577692	1.268631345	0.124909	10.15642	3.10E-24	1.63E-22
11029	TRPM5	0.616395085	1.267950856	0.556038	2.280332	0.022588	0.040144
7265	CASQ2	122.5374512	1.267489369	0.340893	3.718147	0.000201	0.000541
5093	RASSF6	706.9076097	1.265141409	0.267511	4.729302	2.25E-06	8.67E-06
4034	LRRK2	3085.302356	1.262650298	0.237544	5.315446	1.06E-07	5.17E-07
5341	MARVELD	120.7921473	1.262083074	0.274367	4.599979	4.23E-06	1.55E-05
11323	LOC28578	0.70270839	1.261900806	0.580781	2.172764	0.029798	0.051583
715	SDHD	2723.592544	1.261170327	0.140526	8.974667	2.84E-19	7.79E-18
2495	CYFIP2	4162.124899	1.260331069	0.1948	6.469862	9.81E-11	7.71E-10
2192	TNFRSF21	2576.916185	1.259139674	0.186043	6.767999	1.31E-11	1.17E-10
1590	KGFLP2	151.2844009	1.259070483	0.169343	7.43505	1.05E-13	1.29E-12
3236	CMBL	5010.773082	1.257843779	0.214563	5.862363	4.56E-09	2.76E-08
4308	CCDC85A	183.8942177	1.257794111	0.244036	5.15414	2.55E-07	1.16E-06
8666	CHST9	337.698659	1.257056084	0.39967	3.145233	0.00166	0.003754
3692	PTPRB	2463.71153	1.25666373	0.226892	5.53859	3.05E-08	1.62E-07
5730	RAB19	15.62972392	1.255624904	0.28487	4.407712	1.04E-05	3.57E-05
1347	CPEB3	219.4336275	1.254205855	0.160734	7.802992	6.05E-15	8.80E-14
1981	KIAA1958	113.972756	1.253599236	0.179348	6.989764	2.75E-12	2.72E-11
1658	SH3YL1	1147.505823	1.253434395	0.170622	7.346274	2.04E-13	2.41E-12
12154	KCNA1	1.185292984	1.253398773	0.656519	1.909157	0.056242	0.090702
4827	LOC34234	8.963946141	1.252939591	0.256908	4.876992	1.08E-06	4.37E-06

8513	MTUS2	26.59788678	1.25276348	0.391524	3.199714	0.001376	0.003167
1499	MYO9A	854.9246271	1.251831445	0.165376	7.569603	3.74E-14	4.90E-13
5496	FZD9	7.008374844	1.251824597	0.276697	4.524168	6.06E-06	2.16E-05
5421	CRYM	271.3475037	1.251749002	0.274759	4.5558	5.22E-06	1.89E-05
9145	SCN2A	36.00587669	1.250698882	0.422501	2.960226	0.003074	0.006589
11478	PCDH11Y	2.349092145	1.246913546	0.586065	2.127602	0.03337	0.056986
3637	ITPR2	1678.047047	1.246808692	0.223475	5.579176	2.42E-08	1.30E-07
5111	NEAT1	18381.32343	1.246136725	0.263849	4.722908	2.32E-06	8.92E-06
2915	FAM134B	2871.166043	1.246094577	0.204268	6.10029	1.06E-09	7.12E-09
2288	LARS2	469.2957587	1.246051161	0.186928	6.665949	2.63E-11	2.25E-10
1800	METTL7A	4427.158065	1.245569297	0.173179	7.192368	6.37E-13	6.93E-12
9972	IZUMO1	1.133898379	1.245237944	0.467669	2.662647	0.007753	0.015239
724	LNX2	537.3911274	1.245195601	0.139056	8.954657	3.41E-19	9.23E-18
7907	HIST3H2A	22.7572347	1.245088465	0.36139	3.445277	0.00057	0.001414
735	RALGAPA	495.7870146	1.244861606	0.13943	8.928189	4.33E-19	1.15E-17
13294	IFITM5	0.92852447	1.244853897	0.805498	1.545447	0.122238	0.180236
2152	ERMP1	1318.168164	1.243843372	0.182704	6.807958	9.90E-12	9.02E-11
12182	HIST1H3A	0.389334502	1.243532147	0.653807	1.901987	0.057173	0.091992
9324	DLGAP1	11.10422469	1.243237122	0.430042	2.890968	0.003841	0.008074
2519	MFSD4	246.8350321	1.241802205	0.192624	6.446752	1.14E-10	8.89E-10
4066	KLHDC7A	1008.625882	1.240938304	0.234253	5.297438	1.17E-07	5.66E-07
681	LRP6	992.6491737	1.240553985	0.136918	9.060545	1.30E-19	3.74E-18
7885	BHMT	5416.236192	1.24012521	0.358917	3.455183	0.00055	0.001367
6561	KCNQ5	24.89260215	1.23976284	0.309835	4.001358	6.30E-05	0.000188
401	TSPYL1	2887.503522	1.238705032	0.123635	10.01907	1.26E-23	6.14E-22
6179	AIF1L	2452.967172	1.237803417	0.295719	4.185746	2.84E-05	9.02E-05
1044	TOM1L1	1165.460314	1.236522071	0.149955	8.245928	1.64E-16	3.08E-15
11220	SERPINB7	3.513657645	1.236121223	0.558394	2.213709	0.026849	0.046904
4031	KDR	4283.449506	1.23564576	0.232392	5.317066	1.05E-07	5.13E-07
15252	KRT84	0.457617469	1.234430843	1.324199	0.932209	0.351228	0.451361
699	BRMS1L	220.8336984	1.233408579	0.136637	9.026869	1.77E-19	4.95E-18
3987	LOC11511	300.6126906	1.233149911	0.230446	5.351145	8.74E-08	4.30E-07
5830	FGF12	77.38235432	1.23286046	0.283022	4.356061	1.32E-05	4.45E-05
11091	USH1G	5.63461158	1.232690229	0.545889	2.258132	0.023937	0.042304
6894	LOC40143	60.33335656	1.232394621	0.318755	3.866278	0.000111	0.000314
3176	NBEAL1	274.0935555	1.231915862	0.208909	5.896896	3.70E-09	2.29E-08
10544	SMCR5	0.894154033	1.23182818	0.501815	2.454746	0.014098	0.026209
1109	SLC44A3	783.0768461	1.23074234	0.150999	8.150658	3.62E-16	6.40E-15
11878	SLC5A7	1.267412726	1.229773649	0.615852	1.996866	0.04584	0.075645
5206	HIST1H2B	115.9644413	1.228605554	0.262827	4.67457	2.95E-06	1.11E-05
1288	PTPN21	511.9392562	1.22848434	0.155959	7.876992	3.35E-15	5.10E-14
4620	GPR116	5527.337402	1.228011155	0.246594	4.979893	6.36E-07	2.70E-06
12342	OR2T5	1.025773665	1.22722757	0.663023	1.850956	0.064176	0.101921
690	SLC30A9	2029.502005	1.225508836	0.135561	9.040248	1.56E-19	4.44E-18
13628	TMPRSS12	0.338682651	1.225054955	0.846964	1.446407	0.148063	0.212957
1693	KIAA1328	138.4899569	1.224166441	0.167646	7.302089	2.83E-13	3.28E-12
5264	SEMA6D	259.7853228	1.223883837	0.263524	4.644291	3.41E-06	1.27E-05

2066	MBP	1523.871626	1.223669913	0.1778	6.882285	5.89E-12	5.59E-11
10899	CSNK1A1P	0.603754454	1.223081763	0.526806	2.321693	0.020249	0.036417
182	TBC1D19	192.6957862	1.222526879	0.107433	11.37945	5.29E-30	5.70E-28
1916	RGP1	175.8642531	1.222144418	0.172802	7.072498	1.52E-12	1.56E-11
5560	MACC1	966.7851092	1.222090898	0.272025	4.49256	7.04E-06	2.48E-05
6887	SLC4A5	265.7694385	1.221042062	0.315584	3.869152	0.000109	0.000311
3950	ABHD3	431.6380865	1.220941453	0.227159	5.374831	7.67E-08	3.80E-07
1623	PER3	761.5186227	1.220930923	0.165051	7.397296	1.39E-13	1.68E-12
8734	ZNF208	127.2693701	1.220300624	0.391719	3.115247	0.001838	0.004125
3475	SORL1	3430.766384	1.220291668	0.214422	5.691074	1.26E-08	7.12E-08
1791	KBTBD7	144.9634213	1.220078436	0.169526	7.196982	6.16E-13	6.73E-12
15771	NXNL1	0.264536679	1.219205405	1.555106	0.784002	0.433039	0.538203
2694	FSD1L	90.78326203	1.217659007	0.193674	6.287143	3.23E-10	2.35E-09
5991	TNFSF15	24.86665235	1.217566525	0.284901	4.273648	1.92E-05	6.29E-05
3239	LOC22042	4.418524607	1.216422168	0.207536	5.861269	4.59E-09	2.78E-08
457	CDC14B	640.0746682	1.216117224	0.124463	9.770932	1.50E-22	6.44E-21
13457	PMP2	0.649435929	1.215196013	0.81013	1.500002	0.133614	0.194617
712	NKIRAS1	186.874114	1.215035053	0.135282	8.981503	2.67E-19	7.35E-18
3651	NORD116-2	51.77430962	1.214983795	0.218049	5.572057	2.52E-08	1.35E-07
8141	CLDN2	3851.627179	1.214411567	0.361852	3.356101	0.00079	0.001903
356	C4orf29	131.8113002	1.213833492	0.118513	10.24218	1.28E-24	7.06E-23
2948	HIGD1A	1984.72014	1.212661055	0.199705	6.072254	1.26E-09	8.39E-09
4956	FAM102A	1952.778929	1.211768963	0.252793	4.793515	1.64E-06	6.48E-06
1026	TMEM184C	713.3828377	1.21153152	0.146049	8.295405	1.08E-16	2.07E-15
7020	KIAA1324L	182.0554556	1.20998501	0.317149	3.815192	0.000136	0.00038
13617	MS4A10	1.900643782	1.20994352	0.834417	1.450047	0.147045	0.211664
1422	ATP10D	1198.092302	1.209854882	0.157592	7.677145	1.63E-14	2.24E-13
1161	ABHD6	731.7304668	1.209498146	0.149831	8.072426	6.89E-16	1.16E-14
531	CHIC1	578.2601774	1.208510186	0.126966	9.518341	1.76E-21	6.48E-20
951	BDH2	1865.852798	1.207994184	0.14306	8.443985	3.07E-17	6.32E-16
4248	SLC26A1	162.1553152	1.207748422	0.233053	5.182291	2.19E-07	1.01E-06
12811	UMODL1	0.892122779	1.206476934	0.709063	1.701508	0.088848	0.135938
8741	FUT3	37.01672753	1.20441891	0.387002	3.112177	0.001857	0.004164
9582	ALDH1A2	350.7084246	1.203724548	0.43188	2.787171	0.005317	0.010877
4751	STK32B	660.7368966	1.203569839	0.245216	4.908208	9.19E-07	3.79E-06
4596	AK7	63.25754835	1.203481024	0.241213	4.989295	6.06E-07	2.58E-06
328	AP1AR	665.5054626	1.203035936	0.11539	10.42583	1.89E-25	1.13E-23
4673	FAM184A	44.25787766	1.202657538	0.243025	4.948699	7.47E-07	3.13E-06
5082	OC1001909	41.58047701	1.202236762	0.254098	4.731385	2.23E-06	8.60E-06
4166	C9orf125	384.6226809	1.200230819	0.2291	5.238886	1.62E-07	7.60E-07
5587	GALNTL2	507.5708021	1.198985249	0.267613	4.4803	7.45E-06	2.62E-05
5718	NXNL2	40.87696766	1.198702607	0.271777	4.410617	1.03E-05	3.53E-05
14516	NGB	0.377899094	1.198324339	1.031787	1.161406	0.245477	0.331484
1933	SH3BGRL2	784.5749834	1.197979357	0.169878	7.052011	1.76E-12	1.79E-11
10592	PRSS45	1.482153714	1.197537766	0.491015	2.438904	0.014732	0.027262
13505	KFZP434L1	2.665625959	1.195694922	0.804123	1.486955	0.137027	0.198879
7029	NR4A1	4548.200312	1.195366411	0.313848	3.808749	0.00014	0.000389

7997	SOX2OT	1.281588213	1.194758107	0.350317	3.410507	0.000648	0.001589
2049	C15orf33	29.2092253	1.194539775	0.173028	6.903746	5.06E-12	4.85E-11
8913	LOC28595	14.28774614	1.194310688	0.391984	3.046837	0.002313	0.005086
1564	CPEB2	2197.633729	1.192558003	0.159549	7.474548	7.75E-14	9.71E-13
9451	KRTAP5-1	4.876739174	1.191737866	0.419961	2.837737	0.004543	0.009423
11671	FABP1	33.35006222	1.191716848	0.579217	2.057463	0.039642	0.066575
11142	SPTBN2	634.0975307	1.191333333	0.531903	2.239758	0.025107	0.044168
900	SCOC	1645.323812	1.191217353	0.139753	8.523711	1.55E-17	3.37E-16
1632	ZNF132	89.70859412	1.190857852	0.161282	7.383701	1.54E-13	1.85E-12
7901	PCDHGA1	74.65864848	1.189924751	0.345102	3.448038	0.000565	0.001401
6883	NEK5	13.13578608	1.189483556	0.307327	3.870418	0.000109	0.000309
2283	C9orf41	166.9430969	1.189273126	0.178322	6.669232	2.57E-11	2.21E-10
5796	ABHD1	21.09163686	1.189127896	0.271745	4.3759	1.21E-05	4.09E-05
10120	CHGB	71.43093744	1.188515674	0.455482	2.609361	0.009071	0.01757
9695	EDN2	204.6490375	1.187557237	0.432103	2.748322	0.00599	0.012111
1387	ARL6	148.0937055	1.187061548	0.153584	7.729076	1.08E-14	1.53E-13
2894	TIMP3	14580.48144	1.186513568	0.194045	6.114621	9.68E-10	6.56E-09
942	DLAT	1276.434693	1.186256235	0.140227	8.459543	2.68E-17	5.59E-16
10068	PCDH10	631.8174135	1.185642624	0.450868	2.629688	0.008546	0.016639
13921	LOC15762	0.401254744	1.18558749	0.882417	1.343568	0.179088	0.252159
277	EFHA1	985.1170108	1.185310427	0.110754	10.70217	9.94E-27	7.04E-25
6451	SAMD12	268.8919278	1.184970519	0.292734	4.047937	5.17E-05	0.000157
1594	RBM47	3246.855795	1.184899254	0.15951	7.428393	1.10E-13	1.35E-12
2208	BEX4	945.7071085	1.184736623	0.175592	6.747115	1.51E-11	1.34E-10
7947	UPB1	491.1170816	1.184594092	0.345448	3.42915	0.000605	0.001493
9235	LOC28457	11.49190585	1.183326163	0.404453	2.925743	0.003436	0.007294
1751	HABP4	722.4955562	1.181077269	0.163087	7.241999	4.42E-13	4.95E-12
5527	OC1001305	14.29400398	1.180265007	0.261887	4.506773	6.58E-06	2.33E-05
5339	VDR	583.5267482	1.179746789	0.256346	4.602158	4.18E-06	1.54E-05
1206	TRPM7	1260.224963	1.179452866	0.147306	8.006848	1.18E-15	1.91E-14
2562	SH3BP4	1856.756045	1.178985524	0.18379	6.414838	1.41E-10	1.08E-09
6478	ANGPT1	443.915227	1.178887534	0.292013	4.037102	5.41E-05	0.000164
13139	OR2T2	0.372546046	1.178860063	0.738249	1.596833	0.110303	0.16454
12433	GC	47.50414436	1.17884228	0.645424	1.826463	0.067781	0.106858
6344	C14orf37	104.4057562	1.178047917	0.28687	4.106552	4.02E-05	0.000124
407	DYNLL2	1335.571227	1.177867462	0.11763	10.01331	1.33E-23	6.40E-22
13242	DLK1	1.514005917	1.177858554	0.752362	1.565547	0.117455	0.173858
5535	AKR1C1	867.651966	1.177594427	0.261463	4.503859	6.67E-06	2.36E-05
795	ATP5S	118.5430808	1.176105401	0.134253	8.760353	1.95E-18	4.80E-17
637	HSPC159	707.9694972	1.17497968	0.127737	9.198424	3.63E-20	1.12E-18
313	KLHDC2	1408.85665	1.174529643	0.112205	10.46774	1.22E-25	7.61E-24
3908	C9orf45	89.92272081	1.174423122	0.217589	5.397433	6.76E-08	3.39E-07
4896	FAM174B	434.8668597	1.174228351	0.243175	4.828738	1.37E-06	5.50E-06
16270	LOC34802	0.306191697	1.174068843	1.849933	0.634655	0.525654	0.633304
2732	IQCH	44.10983553	1.17400668	0.187698	6.254772	3.98E-10	2.86E-09
3936	RAPGEF4	396.9796346	1.173708702	0.218105	5.381387	7.39E-08	3.68E-07
5595	APOLD1	4559.062418	1.173685561	0.262313	4.474374	7.66E-06	2.68E-05

1019	BAG1	1240.723596	1.173668535	0.14136	8.302688	1.02E-16	1.96E-15
12054	SPOCK3	0.938034888	1.173067344	0.603848	1.942652	0.052058	0.084652
1112	GPRASP2	381.1660167	1.172904661	0.144057	8.141974	3.89E-16	6.85E-15
521	RAPGEF2	1547.059645	1.172334424	0.122907	9.53835	1.45E-21	5.46E-20
813	BHLHB9	137.0928115	1.172300049	0.134366	8.724692	2.67E-18	6.43E-17
5865	CMYA5	424.762526	1.17110925	0.269944	4.338338	1.44E-05	4.80E-05
2924	ZNF833	27.27307026	1.171073836	0.192193	6.093208	1.11E-09	7.42E-09
15388	FAM74A1	0.442046719	1.170971865	1.310907	0.893254	0.371721	0.473493
5378	GPR89B	29.30768109	1.170178383	0.255641	4.577425	4.71E-06	1.72E-05
10164	C12orf39	126.5875175	1.169809259	0.450911	2.594325	0.009478	0.018277
6819	SULT1C2	1094.283431	1.169633764	0.300526	3.891954	9.94E-05	0.000286
9374	C18orf16	4.249606745	1.169156165	0.407254	2.870827	0.004094	0.008561
495	FBXL5	6193.795372	1.168951857	0.121253	9.640598	5.39E-22	2.13E-20
13277	LMAN1L	0.413165984	1.167453773	0.752397	1.551647	0.120747	0.17826
7382	IGSF9B	24.58927581	1.167367876	0.318692	3.663	0.000249	0.000662
3102	ADHFE1	134.8713368	1.166942375	0.195713	5.962527	2.48E-09	1.57E-08
5454	ID4	680.0622584	1.166451023	0.256909	4.540332	5.62E-06	2.02E-05
7107	ANO9	234.6931257	1.166205959	0.308386	3.781645	0.000156	0.00043
13046	C9orf135	0.847485201	1.165073749	0.714106	1.631514	0.102782	0.154425
3682	ZSWIM5	319.0216944	1.163431864	0.209674	5.548773	2.88E-08	1.53E-07
2103	MPL	19.2796068	1.162947682	0.169852	6.84682	7.55E-12	7.04E-11
3290	SEPHS2	3830.451143	1.161920042	0.199578	5.821874	5.82E-09	3.47E-08
2314	CTDSPL	1544.575221	1.161857226	0.174903	6.642883	3.08E-11	2.61E-10
8492	NTRK2	641.2855123	1.161784195	0.361888	3.210346	0.001326	0.00306
9881	TRIM74	5.680736179	1.161681868	0.431138	2.694457	0.00705	0.013986
16515	EIF4E1B	0.234297879	1.161194128	2.044848	0.567863	0.570128	0.676662
16746	ASZ1	0.218657265	1.161191409	2.296912	0.505545	0.613176	0.717716
13936	EDDM3A	0.330948516	1.160215441	0.865948	1.339821	0.180304	0.253597
9601	CDC20B	6.784033399	1.159968802	0.417312	2.779622	0.005442	0.011111
4417	SYN3	8.419979881	1.159199003	0.227654	5.091925	3.54E-07	1.57E-06
6644	TNN	22.2568608	1.157505316	0.291871	3.965814	7.31E-05	0.000216
507	CUL5	1166.139162	1.156132425	0.120672	9.580804	9.63E-22	3.72E-20
2267	ZNF493	258.3936263	1.155900884	0.172965	6.68286	2.34E-11	2.03E-10
2942	UBL3	2026.59747	1.155855843	0.190235	6.07593	1.23E-09	8.21E-09
12092	GJB7	0.664143666	1.155531779	0.598406	1.931017	0.053481	0.086692
1814	SLC35A3	397.7306841	1.153922418	0.160828	7.174877	7.24E-13	7.82E-12
1836	ZFP28	111.6277916	1.153732513	0.161074	7.162727	7.91E-13	8.44E-12
2347	PNPLA8	1235.783821	1.15308911	0.17443	6.610608	3.83E-11	3.20E-10
644	ABCD3	1671.204174	1.152805343	0.125689	9.171889	4.65E-20	1.41E-18
2682	EPS8	3947.547334	1.152342203	0.183016	6.296394	3.05E-10	2.23E-09
9270	NETO1	9.1720988	1.151617583	0.395862	2.90914	0.003624	0.007663
6369	CLEC3B	831.062412	1.151196185	0.281201	4.093851	4.24E-05	0.000131
7694	AKR7A3	396.4214036	1.151185869	0.325011	3.541991	0.000397	0.001012
6248	RGS5	41721.6105	1.150921552	0.276992	4.15507	3.25E-05	0.000102
6390	CGNL1	2628.211135	1.149961937	0.281893	4.07942	4.51E-05	0.000138
4119	TMEM116	366.2557024	1.149935132	0.218146	5.27141	1.35E-07	6.44E-07
10641	UCN3	2.274687386	1.149834121	0.475536	2.417973	0.015607	0.028749

1729	CCDC85C	582.3262428	1.149733481	0.158183	7.268356	3.64E-13	4.13E-12
9936	KCNJ1	51.09037898	1.147998349	0.42913	2.675174	0.007469	0.014734
1039	IVNS1ABP	8643.690114	1.146087237	0.138788	8.25783	1.48E-16	2.80E-15
5403	SETMAR	290.997973	1.144947354	0.250878	4.563766	5.02E-06	1.82E-05
535	DZIP3	765.0295996	1.144807415	0.120388	9.509294	1.92E-21	7.03E-20
3111	GPRASP1	293.6485754	1.144624534	0.19225	5.953843	2.62E-09	1.65E-08
5534	SLCO2A1	2416.413837	1.144433744	0.254065	4.504491	6.65E-06	2.36E-05
12142	GABRB1	1.133039224	1.144180409	0.597765	1.914098	0.055608	0.089768
13876	GRID2	1.061460294	1.143430205	0.842123	1.357795	0.174529	0.246536
1171	RAB11FIP2	730.3127583	1.142360554	0.141705	8.06156	7.53E-16	1.26E-14
8781	GLYAT	1125.831245	1.141450437	0.369108	3.092456	0.001985	0.004431
7026	LRIT3	11.68397396	1.141367438	0.299437	3.811711	0.000138	0.000385
1192	AFG3L2	2130.600712	1.141121526	0.142174	8.026223	1.01E-15	1.65E-14
10009	SHISA6	3.760494146	1.140212942	0.430267	2.65001	0.008049	0.015763
9163	ENTPD8	19.57063819	1.140001719	0.385943	2.953806	0.003139	0.006714
12277	SLC26A5	0.8050864	1.139186983	0.608372	1.872518	0.061135	0.097606
3190	PLEKHA6	1325.183692	1.138607187	0.193369	5.88826	3.90E-09	2.40E-08
1954	USP53	781.8303505	1.137698108	0.161755	7.033472	2.01E-12	2.02E-11
9459	FAM155B	95.42192249	1.137269391	0.401251	2.834311	0.004592	0.009517
9816	SLC22A2	4203.46799	1.137092764	0.419218	2.712415	0.006679	0.013338
4882	C10orf140	32.86684809	1.136690221	0.234888	4.839284	1.30E-06	5.23E-06
1648	PTPLAD1	2401.236323	1.136262703	0.154313	7.363372	1.79E-13	2.13E-12
412	DBT	754.0511235	1.134988953	0.113551	9.995432	1.60E-23	7.59E-22
5901	AFF3	334.9608855	1.134773455	0.262635	4.320731	1.56E-05	5.17E-05
7667	DCDC2	563.1436375	1.134608491	0.319456	3.551685	0.000383	0.000979
3942	ACOT4	190.8589387	1.134571629	0.210933	5.378824	7.50E-08	3.73E-07
4910	TNFRSF19	652.9557272	1.134009286	0.235192	4.821627	1.42E-06	5.68E-06
7582	LAMA1	705.1648048	1.133788465	0.316238	3.585237	0.000337	0.000871
3721	CASZ1	181.9852176	1.133755234	0.205237	5.524136	3.31E-08	1.74E-07
11752	CLRN1OS	0.623363226	1.133516804	0.556623	2.036416	0.041709	0.069557
2603	FITM2	95.35899862	1.133427502	0.17766	6.379754	1.77E-10	1.34E-09
2920	LIPE	86.21098323	1.133298111	0.185906	6.096086	1.09E-09	7.30E-09
645	BCL2L2	1133.446013	1.133090049	0.12354	9.171838	4.65E-20	1.41E-18
2683	MANEA	947.0146172	1.132737935	0.179919	6.295825	3.06E-10	2.23E-09
4062	EVC	1532.374268	1.132620024	0.213682	5.300491	1.15E-07	5.57E-07
1139	BPGM	868.9913801	1.132603003	0.139709	8.106865	5.19E-16	8.94E-15
1143	DNAJB14	476.8805187	1.130740433	0.139574	8.101396	5.43E-16	9.32E-15
9211	KCNIP3	518.7734437	1.13070446	0.385312	2.934513	0.003341	0.007109
5777	ASAH2B	10.85707292	1.13048415	0.257955	4.382483	1.17E-05	3.98E-05
1950	SUCLG1	2287.118648	1.129698878	0.160561	7.035958	1.98E-12	1.99E-11
6186	ALPK3	1457.963083	1.129555272	0.270083	4.182257	2.89E-05	9.15E-05
3345	SOCS2	459.9931742	1.128875429	0.195063	5.787241	7.16E-09	4.19E-08
510	AK3	1863.863426	1.128569706	0.118008	9.563484	1.14E-21	4.38E-20
2031	C14orf174	38.20478138	1.127749407	0.162827	6.926059	4.33E-12	4.18E-11
485	STX3	1480.107	1.127609584	0.116598	9.670913	4.01E-22	1.62E-20
10077	DAO	116.6559166	1.126817278	0.429146	2.625721	0.008647	0.016818
8601	SCUBE1	54.76217574	1.124882727	0.354324	3.174727	0.0015	0.003418

482	CTAGE5	1685.339235	1.124778497	0.116104	9.687719	3.40E-22	1.38E-20
98	ALG2	1045.119162	1.124437689	0.090875	12.37344	3.64E-35	7.28E-33
4310	RASGRF2	306.9579191	1.124019193	0.218206	5.151184	2.59E-07	1.18E-06
630	KTN1	4261.868496	1.123882913	0.122025	9.210239	3.25E-20	1.01E-18
7779	C1orf175	100.0859407	1.123587871	0.320329	3.50761	0.000452	0.001139
326	MRPL50	471.0306164	1.121401012	0.107526	10.42915	1.83E-25	1.10E-23
7993	FAM9C	3.051038363	1.121351664	0.328633	3.412166	0.000644	0.00158
314	MFSD8	477.8382299	1.12112739	0.107144	10.46375	1.27E-25	7.91E-24
7991	SLC7A8	1100.766199	1.120526609	0.328353	3.412563	0.000644	0.001579
8891	AMN	853.5015832	1.120407477	0.366664	3.055678	0.002246	0.00495
680	FBXO34	612.7838388	1.11989876	0.123601	9.06061	1.30E-19	3.74E-18
9003	GABRB2	20.49236711	1.118724177	0.371515	3.01125	0.002602	0.005664
7080	DPP4	4422.849179	1.118266195	0.294937	3.791541	0.00015	0.000414
1825	AGL	951.6965347	1.11818305	0.156034	7.166281	7.71E-13	8.28E-12
10119	HCN2	20.83710238	1.11769886	0.428334	2.609407	0.00907	0.017569
4226	TMEM233	56.36469046	1.117254517	0.215034	5.195705	2.04E-07	9.46E-07
338	AFTPH	1756.528427	1.117001978	0.107702	10.37124	3.35E-25	1.94E-23
1150	KBTBD3	161.9740866	1.116912943	0.138099	8.087787	6.08E-16	1.04E-14
10106	GSTA1	6314.68621	1.116850102	0.427145	2.614689	0.008931	0.017321
8992	PCDHGB4	89.83480485	1.116845949	0.370422	3.015061	0.002569	0.005601
6777	C10orf110	10.35786007	1.115444	0.285404	3.908295	9.29E-05	0.000269
1680	MOSC2	652.4695518	1.114548712	0.152263	7.319877	2.48E-13	2.90E-12
2297	TMEM220	281.1277667	1.114261792	0.167341	6.658647	2.76E-11	2.36E-10
3762	FLJ36777	11.15957763	1.110847324	0.20199	5.499526	3.81E-08	1.98E-07
6953	ZDHHC15	59.44876306	1.109325095	0.288518	3.844908	0.000121	0.00034
10738	SPINK5	89.66942474	1.109305992	0.465239	2.38438	0.017108	0.031229
2241	ATE1	254.550114	1.10897463	0.16533	6.707653	1.98E-11	1.73E-10
4470	T6GALNAC	180.6111883	1.108075969	0.219125	5.056812	4.26E-07	1.87E-06
2046	UQCRFS1	1856.460488	1.107997816	0.160278	6.912977	4.75E-12	4.55E-11
4994	ASAH2	4.838116162	1.106599772	0.23169	4.776204	1.79E-06	7.01E-06
1622	GOLGA4	1940.08884	1.106490743	0.149527	7.399948	1.36E-13	1.65E-12
3646	NCALD	884.7646518	1.106233752	0.198443	5.574576	2.48E-08	1.33E-07
9515	TRPV6	4.895568317	1.106198	0.393131	2.813819	0.004896	0.010085
3625	ACOT1	683.2140962	1.104628949	0.197591	5.59048	2.26E-08	1.22E-07
6960	BEX2	388.3256916	1.10452491	0.287478	3.842114	0.000122	0.000343
4445	C3orf18	209.1705491	1.103677943	0.217434	5.075913	3.86E-07	1.70E-06
16204	UCP1	0.227176918	1.103489614	1.688154	0.653666	0.513327	0.62094
4254	SOCS7	66.80412013	1.103184079	0.212962	5.180202	2.22E-07	1.02E-06
3480	CLIC4	13505.91774	1.102892973	0.193926	5.687184	1.29E-08	7.27E-08
1418	SCAPER	365.0745248	1.102595361	0.143588	7.678889	1.60E-14	2.22E-13
3189	PDP1	801.3199578	1.102466403	0.187231	5.888259	3.90E-09	2.40E-08
4970	MYO10	1958.35663	1.10235989	0.230196	4.788784	1.68E-06	6.62E-06
7399	LOC15869	6.084720449	1.101207935	0.3011	3.657282	0.000255	0.000675
9553	COL9A1	4.594124696	1.101136829	0.393265	2.799986	0.00511	0.010486
4612	KCTD1	322.4388763	1.09973999	0.220678	4.983454	6.25E-07	2.65E-06
5328	SALL2	269.531159	1.099628383	0.238587	4.608929	4.05E-06	1.49E-05
5467	DNAJC6	215.5256885	1.098799821	0.242272	4.535404	5.75E-06	2.06E-05

1384	OTUD1	751.532253	1.098055023	0.141874	7.739666	9.97E-15	1.41E-13
11887	SLC22A7	57.08090002	1.09687353	0.550547	1.992333	0.046335	0.076403
7120	ANKRD9	105.9937415	1.095002541	0.289851	3.777806	0.000158	0.000436
2838	FBXL2	195.7759643	1.095001594	0.177949	6.153451	7.58E-10	5.24E-09
6367	SNTB1	1751.459175	1.094395523	0.267246	4.095093	4.22E-05	0.00013
6425	SSTR2	206.3230873	1.09408531	0.269258	4.063341	4.84E-05	0.000148
3994	MARK1	134.1070306	1.0931817	0.204551	5.344304	9.08E-08	4.45E-07
9513	TMC4	411.151892	1.093129675	0.388362	2.814719	0.004882	0.010059
1151	KIAA1109	1876.330393	1.092962851	0.135163	8.086247	6.15E-16	1.05E-14
7902	KCNH1	9.606650734	1.092677473	0.316962	3.447349	0.000566	0.001404
1236	CYP4V2	1637.597786	1.092556474	0.137471	7.947556	1.90E-15	3.01E-14
9639	STK33	105.9941573	1.091563068	0.394589	2.766327	0.005669	0.011528
11416	SLC38A3	13.30713273	1.091375325	0.509165	2.143461	0.032076	0.055074
1782	SGPP1	930.6219986	1.091170622	0.151453	7.204664	5.82E-13	6.40E-12
4771	ZSCAN12P	26.65137653	1.090597928	0.222693	4.897312	9.72E-07	3.99E-06
8548	GRIP2	14.47891835	1.089279937	0.341379	3.190823	0.001419	0.003253
6728	PHKA1	625.2328419	1.089188512	0.277119	3.930407	8.48E-05	0.000247
7920	AR	140.1430968	1.0888767	0.31648	3.440585	0.00058	0.001437
5557	UHMK1	567.3335972	1.088521301	0.242252	4.49335	7.01E-06	2.47E-05
9035	ESRRB	7.389767842	1.087409831	0.362439	3.000257	0.002698	0.005852
9546	CCL16	3.878288212	1.087196475	0.387971	2.802264	0.005075	0.01042
3707	C9orf102	134.0476817	1.086548364	0.196374	5.533051	3.15E-08	1.66E-07
5581	MAP3K9	47.08365016	1.086282483	0.242379	4.48176	7.40E-06	2.60E-05
3072	ACSS1	1800.380198	1.086144547	0.181592	5.98124	2.21E-09	1.41E-08
3219	SPTLC3	744.3646259	1.085485794	0.184789	5.874196	4.25E-09	2.59E-08
2743	KLHDC1	73.50513577	1.08521582	0.173862	6.241824	4.32E-10	3.09E-09
1870	SETDB2	431.0371023	1.084605255	0.152334	7.119939	1.08E-12	1.13E-11
3530	NCOA7	1863.050189	1.08433167	0.191932	5.64956	1.61E-08	8.93E-08
10481	IL12B	6.088362311	1.08426226	0.437413	2.478807	0.013182	0.024653
4702	DUSP8	224.7387146	1.08335983	0.219779	4.929322	8.25E-07	3.44E-06
9589	C1orf114	17.24441836	1.082119986	0.388806	2.783186	0.005383	0.011003
8967	ADAMTS1	68.14182257	1.081918199	0.357759	3.024154	0.002493	0.00545
1727	DLD	2537.608148	1.081899882	0.148824	7.269662	3.60E-13	4.09E-12
8339	LOC13446	37.02854538	1.081759505	0.330056	3.277505	0.001047	0.002462
9447	CEND1	12.89618931	1.081311743	0.380769	2.83981	0.004514	0.009366
2129	TMEM33	1955.277103	1.081237404	0.158448	6.823922	8.86E-12	8.16E-11
3006	FAM164C	123.7450427	1.081076759	0.179294	6.029638	1.64E-09	1.07E-08
14996	ADAMTS1	0.769000219	1.080925626	1.072463	1.00789	0.313507	0.409779
4603	TSPAN12	3032.177871	1.080861622	0.21669	4.988045	6.10E-07	2.60E-06
10878	ANGPTL3	133.3916764	1.080070922	0.463578	2.329859	0.019814	0.035702
1659	ZNF737	379.727028	1.079855987	0.147006	7.345635	2.05E-13	2.42E-12
8159	ZNF750	5.793393452	1.079843927	0.322514	3.348211	0.000813	0.001954
3934	CYCS	4043.587969	1.079766716	0.200621	5.382121	7.36E-08	3.67E-07
5474	RALGAPA	534.8696723	1.079362651	0.23811	4.533046	5.81E-06	2.08E-05
2742	PTPN13	998.4692054	1.079248802	0.17287	6.243127	4.29E-10	3.07E-09
8410	NWD1	20.02978664	1.079089392	0.332467	3.245708	0.001172	0.002731
463	ZFP112	114.0473703	1.07862078	0.11053	9.758609	1.69E-22	7.17E-21

4918	CDYL2	112.1944628	1.078517582	0.223849	4.818069	1.45E-06	5.78E-06
3531	RAI2	500.3495907	1.077957566	0.19083	5.648776	1.62E-08	8.97E-08
1472	C10orf32	893.4104875	1.077867452	0.141718	7.605734	2.83E-14	3.77E-13
1809	ACO2	5870.850892	1.077801264	0.150105	7.180306	6.96E-13	7.54E-12
13300	XIST	4697.634654	1.07726956	0.698155	1.543023	0.122825	0.181028
1185	C14orf167	618.1658552	1.077254944	0.133908	8.044715	8.64E-16	1.43E-14
4148	RHOBTB2	790.6960386	1.077090309	0.205077	5.252134	1.50E-07	7.10E-07
651	ZC3H12C	447.8518633	1.076337864	0.1174	9.168139	4.81E-20	1.45E-18
7170	SLC38A11	49.67835963	1.076154609	0.286521	3.75594	0.000173	0.000472
11629	BNC1	42.01174484	1.075792359	0.520196	2.068051	0.038635	0.06511
6399	GLS2	37.87563393	1.075609748	0.263859	4.076454	4.57E-05	0.00014
5451	MECOM	863.3821335	1.075483774	0.236785	4.542028	5.57E-06	2.00E-05
5212	ADRB2	62.57437779	1.075354811	0.230096	4.673509	2.96E-06	1.11E-05
1115	FASTKD1	369.5100061	1.074742697	0.132048	8.139028	3.98E-16	7.00E-15
674	KIAA0232	1605.420526	1.074465547	0.118246	9.086672	1.02E-19	2.97E-18
5049	C22orf25	1047.469238	1.07432354	0.226346	4.746375	2.07E-06	8.04E-06
1204	GMFB	1494.65466	1.074152198	0.134112	8.00939	1.15E-15	1.88E-14
3792	LLGL2	751.9772887	1.073827642	0.196007	5.478523	4.29E-08	2.22E-07
1705	ERO1LB	701.7353756	1.073088885	0.147207	7.289679	3.11E-13	3.57E-12
1462	FNBP1L	2308.614584	1.072875694	0.140729	7.623722	2.46E-14	3.30E-13
2461	C14orf135	1096.163502	1.072627256	0.164852	6.506601	7.69E-11	6.12E-10
8628	LOC16263[illegible]	274.2121522	1.072051513	0.339071	3.161735	0.001568	0.003563
3751	SGMS2	1325.752908	1.07196496	0.194677	5.506369	3.66E-08	1.91E-07
4518	NBEA	507.5255032	1.071640718	0.213237	5.02559	5.02E-07	2.18E-06
4893	CD274	59.3392749	1.070895174	0.221694	4.830512	1.36E-06	5.46E-06
1034	ANKRD46	625.9830551	1.070712595	0.12933	8.2789	1.24E-16	2.36E-15
2727	AKAP9	1832.062695	1.07066644	0.171093	6.257816	3.90E-10	2.81E-09
765	LRBA	1613.014556	1.070169594	0.120994	8.84482	9.17E-19	2.35E-17
9819	HMP19	2.097252225	1.069947553	0.394706	2.710749	0.006713	0.013398
3849	ZNF542	284.0142632	1.069617688	0.196838	5.433987	5.51E-08	2.81E-07
7220	FAM110C	78.18166406	1.069452716	0.286128	3.737669	0.000186	0.000504
9890	PLIN4	144.005742	1.068861384	0.397204	2.690961	0.007125	0.01412
861	C1orf69	289.5364373	1.068526217	0.124027	8.615278	6.98E-18	1.59E-16
1146	GBA2	1615.414315	1.068502442	0.132039	8.092303	5.85E-16	1.00E-14
10269	TRPM1	1.419674196	1.068496588	0.417674	2.558207	0.010521	0.020084
885	SOS2	1291.278513	1.068294167	0.124814	8.559057	1.14E-17	2.52E-16
8464	PDE3B	245.6005057	1.066939485	0.331211	3.221333	0.001276	0.002955
7118	C3orf52	88.54601783	1.066779498	0.282366	3.778007	0.000158	0.000435
11167	DRD2	3.312453802	1.066250802	0.477624	2.232406	0.025588	0.044914
4328	TMTC1	1212.010874	1.065570375	0.207211	5.142453	2.71E-07	1.23E-06
2452	RGNEF	1439.680386	1.065183154	0.163605	6.51071	7.48E-11	5.98E-10
7967	GPT	236.6908262	1.065066137	0.311208	3.422364	0.000621	0.001527
1852	TOB1	1430.234825	1.064638598	0.149238	7.133833	9.76E-13	1.03E-11
4113	ZDHHC3	1588.672444	1.064578605	0.201899	5.272816	1.34E-07	6.40E-07
1287	ANKRD12	1156.527056	1.064512335	0.135142	7.876987	3.35E-15	5.10E-14
2116	OTUD3	339.4022921	1.064359561	0.155789	6.832038	8.37E-12	7.76E-11
130	CDADC1	241.2861791	1.064351686	0.08994	11.83401	2.60E-32	3.93E-30

722	MED21	537.2915414	1.064215352	0.118775	8.959909	3.25E-19	8.82E-18
1671	CD2AP	2021.22677	1.064127657	0.145117	7.332904	2.25E-13	2.64E-12
12598	CNKSR2	1.895522221	1.064079321	0.599784	1.774103	0.076046	0.118319
13794	FUT9	4.72050655	1.063907329	0.766281	1.388403	0.165014	0.234482
8819	CETN4P	2.262116897	1.062941435	0.344874	3.082112	0.002055	0.004568
14325	CDH19	1.838945818	1.062827335	0.869004	1.223041	0.221314	0.302826
3353	KLHL11	42.21326546	1.062803492	0.183926	5.778434	7.54E-09	4.41E-08
4588	CRB3	340.4853865	1.062227446	0.212777	4.992219	5.97E-07	2.55E-06
8599	GJB1	340.6517895	1.062042175	0.334501	3.175001	0.001498	0.003415
2417	C5orf41	775.7147632	1.061378354	0.162259	6.541277	6.10E-11	4.95E-10
1754	VPS13D	1525.725136	1.061120075	0.146584	7.239002	4.52E-13	5.05E-12
3329	CABLES1	828.263161	1.060789967	0.182996	5.796787	6.76E-09	3.98E-08
8455	EBF2	102.7911464	1.060751329	0.328723	3.226889	0.001251	0.002901
11000	CRNA0003	0.83163458	1.060078941	0.463149	2.28885	0.022088	0.039359
1627	SLK	1781.543633	1.059041778	0.14328	7.391424	1.45E-13	1.75E-12
13858	CA7	0.337802832	1.056829255	0.773735	1.36588	0.171977	0.243247
10013	HPGD	432.1270005	1.056786603	0.398958	2.648864	0.008076	0.01581
5182	CHDH	604.5976433	1.056750203	0.225597	4.684248	2.81E-06	1.06E-05
7035	SLC4A4	4688.708363	1.056738998	0.277667	3.805777	0.000141	0.000394
1485	BPHL	491.7801693	1.05668326	0.139155	7.593583	3.11E-14	4.11E-13
1240	WWP1	1735.573944	1.056553045	0.13306	7.940416	2.02E-15	3.19E-14
1230	PIK3C2A	2003.408042	1.056437803	0.132566	7.969157	1.60E-15	2.55E-14
8239	CCL2	2476.209338	1.055057368	0.318233	3.315365	0.000915	0.002177
1601	SATL1	29.68174845	1.054417617	0.14213	7.418705	1.18E-13	1.45E-12
604	RAVER2	557.2603886	1.053388962	0.113195	9.30599	1.33E-20	4.31E-19
5468	EFHA2	153.9061018	1.053292808	0.232291	4.534359	5.78E-06	2.07E-05
6790	RGL3	498.4259995	1.052505759	0.269637	3.903416	9.48E-05	0.000274
539	SPAG9	3401.71666	1.052458619	0.110825	9.496601	2.17E-21	7.89E-20
3638	GNA11	585.2918561	1.051337291	0.18846	5.57857	2.43E-08	1.31E-07
7287	C22orf45	107.9678616	1.050571308	0.283529	3.705341	0.000211	0.000568
6686	SLC25A34	100.9453228	1.050511874	0.26615	3.947063	7.91E-05	0.000232
435	C1orf26	199.5786162	1.050373489	0.106348	9.87677	5.25E-23	2.37E-21
1170	NAA30	616.8155354	1.050202611	0.130248	8.063082	7.44E-16	1.25E-14
4218	GPR160	854.963346	1.049480938	0.201846	5.199422	2.00E-07	9.29E-07
4151	ZNF154	87.95717982	1.049234001	0.199828	5.25068	1.52E-07	7.16E-07
1544	PEX7	188.3775712	1.049063337	0.139869	7.500335	6.37E-14	8.08E-13
4460	C21orf34	39.48500335	1.048841489	0.207153	5.063126	4.12E-07	1.81E-06
3382	FAM63A	768.0326037	1.048076434	0.18202	5.758026	8.51E-09	4.93E-08
14721	CCDC27	0.329542788	1.046951278	0.958452	1.092336	0.274685	0.365743
4172	DDO	186.7740185	1.046497758	0.199909	5.234866	1.65E-07	7.76E-07
1262	ZNF549	151.3830094	1.04631073	0.132247	7.911768	2.54E-15	3.94E-14
15775	KRTAP5-4	0.304037844	1.046076008	1.335852	0.783078	0.433582	0.538741
2558	DYNC2H1	523.6118794	1.045938879	0.162961	6.418329	1.38E-10	1.06E-09
15762	DSG4	0.256761341	1.045784495	1.329208	0.786773	0.431415	0.536491
16731	IRS4	0.254746281	1.045779238	2.046052	0.511121	0.609267	0.713779
2935	NHLRC2	152.3783619	1.045660937	0.171976	6.080273	1.20E-09	8.01E-09
1537	MARS2	208.2085102	1.045506962	0.139251	7.508097	6.00E-14	7.65E-13

4540	PRKAG2	1355.307226	1.044140671	0.208269	5.01343	5.35E-07	2.31E-06
3151	SLC39A8	1037.039942	1.043795211	0.176274	5.921432	3.19E-09	1.99E-08
9605	RGS6	7.953649164	1.04324426	0.375526	2.778091	0.005468	0.011158
2332	DTWD2	300.2676226	1.043159555	0.157525	6.62217	3.54E-11	2.98E-10
12902	CYP2B6	8.79615927	1.042911252	0.623624	1.672339	0.094458	0.143491
6012	PKDREJ	13.11058707	1.042659797	0.244757	4.259987	2.04E-05	6.67E-05
10070	SRRM4	14.30618974	1.042070065	0.396315	2.629396	0.008554	0.016649
8315	LOC14870	8.762805473	1.041529985	0.317056	3.284998	0.00102	0.002404
1175	PDE12	545.9198634	1.041522895	0.129306	8.0547	7.97E-16	1.33E-14
8568	GGT8P	58.53959778	1.041304825	0.326982	3.184595	0.00145	0.003316
222	KLHL9	1309.052611	1.041077692	0.094487	11.01819	3.12E-28	2.76E-26
2225	ARFGEF2	1938.034236	1.040914815	0.154683	6.729349	1.70E-11	1.50E-10
5459	AMIGO1	236.8968264	1.040747192	0.229313	4.538543	5.66E-06	2.03E-05
1316	RHPN2	1030.534375	1.040622204	0.132737	7.839721	4.52E-15	6.73E-14
1126	ZNF770	1325.016656	1.040620898	0.128171	8.119026	4.70E-16	8.18E-15
3877	TMEM25	317.1227505	1.040041187	0.192011	5.416572	6.08E-08	3.07E-07
11665	OLIG1	75.24923568	1.039782957	0.50508	2.058651	0.039528	0.066419
4681	MASP2	20.56561001	1.039183569	0.210349	4.940272	7.80E-07	3.27E-06
1260	SYNE2	3995.363187	1.03892178	0.131272	7.914252	2.49E-15	3.87E-14
12176	OVCH1	0.494695204	1.038628309	0.545585	1.903695	0.05695	0.091678
3109	ZNF582	37.59772425	1.037479934	0.174234	5.954527	2.61E-09	1.64E-08
9186	PDK4	16995.97097	1.037318893	0.352321	2.944247	0.003237	0.006908
1025	RWDD4A	666.6572719	1.037101193	0.124997	8.297021	1.07E-16	2.04E-15
2398	CDC14A	220.9445047	1.037056532	0.158073	6.560621	5.36E-11	4.38E-10
7856	NFKBID	67.57592434	1.036734512	0.298892	3.468593	0.000523	0.001305
3697	NINL	422.1210717	1.036699499	0.187257	5.536225	3.09E-08	1.64E-07
4219	TRAM1L1	119.6347863	1.036395904	0.199354	5.198763	2.01E-07	9.32E-07
10376	UGT1A9	4347.032443	1.036192565	0.412187	2.513888	0.011941	0.022557
9101	OC1001286	11.63828284	1.035838587	0.348092	2.975764	0.002923	0.006294
4167	FRK	155.9035691	1.034365582	0.197482	5.23776	1.63E-07	7.65E-07
1965	SPTLC2	1778.125773	1.033967782	0.147424	7.013569	2.32E-12	2.32E-11
10855	PPAPDC1A	27.26496005	1.03383681	0.442174	2.338075	0.019383	0.035001
5913	RAB11FIP4	396.0238127	1.033308893	0.239395	4.316327	1.59E-05	5.26E-05
6778	RBP7	528.7590092	1.032261792	0.264164	3.907651	9.32E-05	0.000269
9216	C13orf38	6.431077386	1.032115051	0.351894	2.93303	0.003357	0.007139
9423	PCDHGA7	80.91085932	1.031820578	0.36202	2.850172	0.00437	0.009089
2759	KPNA5	78.48783604	1.03161792	0.16552	6.232604	4.59E-10	3.26E-09
4094	SHE	443.2517583	1.031228408	0.195163	5.283921	1.26E-07	6.05E-07
9057	ACP5	2913.779042	1.031101332	0.344258	2.99514	0.002743	0.005937
7756	GRIK3	444.8208561	1.03110113	0.293233	3.516315	0.000438	0.001106
10976	BAI3	11.33472685	1.030977534	0.449294	2.294663	0.021752	0.038846
3497	EXOC6B	175.5801924	1.030546228	0.181527	5.677083	1.37E-08	7.68E-08
2048	ENOSF1	897.5647661	1.030293126	0.149147	6.907907	4.92E-12	4.71E-11
4830	ZNF483	25.30319271	1.029929888	0.211227	4.87594	1.08E-06	4.39E-06
14865	HRK	1.917469639	1.029584414	0.982681	1.04773	0.294763	0.388675
975	PPARA	1192.354576	1.02931726	0.122777	8.383604	5.13E-17	1.03E-15
2274	CCNG2	1969.255466	1.028926967	0.154161	6.674349	2.48E-11	2.14E-10

504	HADHB	4461.776344	1.028643893	0.107165	9.598663	8.10E-22	3.15E-20
1915	NUDT7	144.2555992	1.027848695	0.145329	7.072569	1.52E-12	1.56E-11
549	CD46	6404.624853	1.027278622	0.108613	9.458128	3.14E-21	1.12E-19
3051	AIFM1	2218.839809	1.027089491	0.171232	5.998232	1.99E-09	1.28E-08
4099	CRYZ	4576.78074	1.026646932	0.194378	5.281712	1.28E-07	6.12E-07
1134	ZNF780B	292.3652138	1.026168331	0.126504	8.111772	4.99E-16	8.62E-15
3008	TNIK	446.9093858	1.024762439	0.169972	6.029006	1.65E-09	1.08E-08
7811	CHRM3	26.71834053	1.024555575	0.29293	3.497613	0.000469	0.001178
432	WDFY3	2291.763746	1.02396216	0.103441	9.898973	4.21E-23	1.91E-21
8038	DSCR6	3.022252122	1.023686329	0.301417	3.396243	0.000683	0.001666
5424	LRRC70	115.5608674	1.023490722	0.224699	4.55495	5.24E-06	1.89E-05
10169	VTCN1	20.59729718	1.023472422	0.394662	2.593286	0.009506	0.018324
2783	CLN8	712.1530228	1.021316134	0.16443	6.21124	5.26E-10	3.70E-09
6576	FAM124A	65.31230103	1.020667237	0.255628	3.99279	6.53E-05	0.000195
832	GFM2	732.4449441	1.020615928	0.117644	8.67548	4.12E-18	9.70E-17
2155	COMMD8	448.3983861	1.020518387	0.14996	6.805293	1.01E-11	9.17E-11
2883	ZNF367	159.3351998	1.020394346	0.166641	6.123293	9.17E-10	6.23E-09
10124	UGT2B11	5.104811816	1.019759839	0.391103	2.607396	0.009123	0.017664
13086	HS3ST5	1.073386347	1.019605685	0.630511	1.617109	0.105855	0.158556
506	RCHY1	571.6628109	1.018240793	0.10625	9.583463	9.38E-22	3.64E-20
3857	ARL15	639.5023948	1.018186925	0.18759	5.427716	5.71E-08	2.90E-07
1281	NAP1L5	180.6439488	1.017793782	0.129021	7.88856	3.06E-15	4.68E-14
880	CPT2	930.9545987	1.017652233	0.118713	8.572382	1.01E-17	2.26E-16
1167	MCCC1	757.1875191	1.017363635	0.12613	8.065988	7.26E-16	1.22E-14
3603	ALAS1	1000.589357	1.016750822	0.181439	5.603808	2.10E-08	1.14E-07
10921	EHF	28.59842532	1.016214326	0.439598	2.31169	0.020795	0.037322
1395	TRIP11	765.8568005	1.015468751	0.131485	7.723048	1.14E-14	1.60E-13
577	CCDC121	103.3336231	1.014536104	0.108132	9.382392	6.45E-21	2.19E-19
2684	FAM84B	1516.284518	1.014007318	0.161073	6.295345	3.07E-10	2.24E-09
3851	PPP1R9A	509.5699498	1.013753331	0.18659	5.433048	5.54E-08	2.82E-07
1560	KIAA2018	672.0005117	1.013287509	0.135501	7.478067	7.54E-14	9.48E-13
3250	CX3CL1	4123.248884	1.013249153	0.173121	5.85284	4.83E-09	2.91E-08
10901	PKLR	639.2887581	1.01291578	0.436314	2.32153	0.020258	0.036426
8537	CADM4	311.351956	1.012256589	0.316989	3.193348	0.001406	0.003229
738	ZNF658	55.71484216	1.011845928	0.113458	8.918254	4.74E-19	1.26E-17
7347	DGKI	33.51210961	1.011149194	0.274574	3.682614	0.000231	0.000616
5415	ATP6V1G2	23.22188304	1.011103506	0.221808	4.558458	5.15E-06	1.87E-05
5012	ICA1	255.550464	1.010830787	0.212093	4.765982	1.88E-06	7.35E-06
11437	SIGLEC15	11.34234093	1.010802168	0.472744	2.138159	0.032504	0.055706
4795	ACACB	1092.016287	1.01061423	0.206685	4.889644	1.01E-06	4.13E-06
11089	GCNT7	0.619233853	1.010281626	0.447078	2.259746	0.023837	0.042135
1314	C8orf37	92.22547836	1.009950966	0.128799	7.841306	4.46E-15	6.65E-14
3853	OXNAD1	232.3845856	1.009139833	0.1858	5.431333	5.59E-08	2.85E-07
822	HOMEZ	334.9493199	1.008778492	0.115904	8.703605	3.22E-18	7.67E-17
3319	C4orf33	298.1023293	1.008698114	0.173788	5.804174	6.47E-09	3.82E-08
6978	SNURF	92.11893778	1.008617539	0.263044	3.834401	0.000126	0.000354
5660	ARHGAP2[illegible]	308.4746776	1.008445832	0.226998	4.442537	8.89E-06	3.08E-05

6330	TMEM101	1174.050775	1.008006646	0.24509	4.112809	3.91E-05	0.000121
382	ETFA	1667.822806	1.007761905	0.099595	10.11862	4.57E-24	2.34E-22
8257	ABCG5	3.66254806	1.006133382	0.303962	3.310059	0.000933	0.002214
1664	FAHD1	1679.309008	1.00605796	0.137023	7.342245	2.10E-13	2.47E-12
13131	TDRD12	0.587421154	1.00605666	0.629388	1.598469	0.109939	0.164108
3995	ZNF221	17.73135234	1.006041836	0.188269	5.343652	9.11E-08	4.47E-07
1243	ZYG11B	1301.26045	1.005596079	0.126687	7.937669	2.06E-15	3.25E-14
6715	ENPEP	8992.147341	1.00477329	0.255382	3.93439	8.34E-05	0.000243
4807	FAM171A1	587.8212788	1.004542954	0.205683	4.883935	1.04E-06	4.24E-06
918	DLST	2608.913255	1.004276697	0.11824	8.493543	2.00E-17	4.28E-16
11264	GRIK1	1.871403014	1.003960605	0.457674	2.193614	0.028263	0.049182
3058	DGKE	199.2019521	1.003237714	0.167407	5.9928	2.06E-09	1.32E-08
4438	TLN2	1624.994941	1.003167471	0.197485	5.079722	3.78E-07	1.67E-06
9757	VAT1L	61.18391642	1.002861516	0.36733	2.730141	0.006331	0.012718
2280	LRRC1	317.993179	1.002752743	0.15032	6.670793	2.54E-11	2.19E-10
3213	TEX9	105.5016549	1.002282812	0.170491	5.878801	4.13E-09	2.52E-08
13298	GPR26	1.539602399	1.001791228	0.649101	1.543351	0.122746	0.180925
7086	PLIN2	20008.12581	1.001143638	0.264179	3.789646	0.000151	0.000417
1148	CBWD1	729.1816645	1.001084872	0.123747	8.089803	5.98E-16	1.02E-14
2497	ADD3	3227.556635	1.000613934	0.154782	6.464665	1.02E-10	7.97E-10
4863	HIST1H2AC	1117.961169	1.000466506	0.206049	4.855472	1.20E-06	4.84E-06
11180	FLJ34503	1.209933554	1.000194466	0.448654	2.229323	0.025792	0.045223
14306	TRDN	1.382020034	0.999755183	0.8144	1.227597	0.219598	0.300877
2216	SNX13	1527.320363	0.999489037	0.14829	6.740079	1.58E-11	1.40E-10
1402	YOD1	344.4830656	0.998979605	0.129534	7.712117	1.24E-14	1.73E-13
8209	SLC25A27	172.4813128	0.998879701	0.300137	3.328077	0.000874	0.002088
1609	SPOPL	920.6979479	0.998810835	0.134774	7.411006	1.25E-13	1.53E-12
4928	LOC40237	5.894995657	0.998784637	0.20756	4.812026	1.49E-06	5.94E-06
7944	RUNDC3B	92.58648562	0.998682751	0.291166	3.429948	0.000604	0.00149
1277	IVD	1838.512395	0.998398445	0.126474	7.894116	2.92E-15	4.49E-14
1056	FAM108B1	392.6499389	0.997808979	0.121157	8.235691	1.79E-16	3.31E-15
7428	SPATA4	13.22417143	0.997418225	0.273641	3.644989	0.000267	0.000706
11239	PDZRN4	13.83983985	0.996888812	0.452251	2.204282	0.027505	0.047968
2999	FAM164A	376.1650036	0.996786607	0.165243	6.032231	1.62E-09	1.06E-08
4894	C13orf15	1415.654187	0.996039719	0.206247	4.829362	1.37E-06	5.49E-06
7648	MSI1	19.37231963	0.995977226	0.279689	3.561011	0.000369	0.000947
621	PRMT10	228.5545562	0.995256435	0.107641	9.246032	2.33E-20	7.35E-19
5432	ARSJ	494.7530537	0.995148839	0.218627	4.551809	5.32E-06	1.92E-05
2833	ACOT2	384.9068216	0.994638893	0.16148	6.159513	7.30E-10	5.05E-09
12084	LDHC	9.659169466	0.994561003	0.514664	1.932447	0.053304	0.086463
5450	ZSCAN23	19.95047591	0.99446882	0.218942	4.542155	5.57E-06	2.00E-05
5805	GNRHR	6.239651692	0.994313852	0.227451	4.371548	1.23E-05	4.16E-05
1718	NAPEPLD	676.3197871	0.993670527	0.136505	7.279345	3.35E-13	3.83E-12
1491	PPP1R13B	556.8321498	0.99257305	0.130872	7.584302	3.34E-14	4.39E-13
3642	PARD6B	748.1739712	0.991719674	0.17782	5.577109	2.45E-08	1.32E-07
4924	CYP7B1	227.9675539	0.991218046	0.205828	4.815757	1.47E-06	5.84E-06
9000	ROBO2	45.64139387	0.99113154	0.32905	3.012099	0.002594	0.00565

9008	SLC5A4	25.29980364	0.991017584	0.329358	3.008942	0.002622	0.005704
1899	ATPBD4	160.7366358	0.990900224	0.1398	7.087962	1.36E-12	1.40E-11
1605	SNX29	1787.390845	0.990648059	0.133562	7.417119	1.20E-13	1.46E-12
5806	SUSD1	428.4804718	0.990517668	0.226581	4.371583	1.23E-05	4.16E-05
2001	TTC7B	748.0120528	0.990386669	0.142356	6.957104	3.47E-12	3.40E-11
3144	SAMD4A	823.8522299	0.989994679	0.167037	5.926791	3.09E-09	1.93E-08
5355	VEPH1	449.5801882	0.989413434	0.215463	4.592037	4.39E-06	1.61E-05
6305	FAM198B	2621.667447	0.988968128	0.239781	4.124468	3.72E-05	0.000116
6878	CASC1	52.27342054	0.988551566	0.255302	3.872085	0.000108	0.000308
15335	OR10K2	0.276560016	0.988078106	1.085722	0.910066	0.362788	0.463711
1778	PLEKHF2	660.7029404	0.987967734	0.13706	7.208273	5.67E-13	6.25E-12
2227	PLA2G12A	1122.096837	0.987494866	0.146892	6.722605	1.79E-11	1.57E-10
9719	C6orf123	12.17678713	0.9872042	0.360007	2.742177	0.006103	0.012309
11094	SOSTDC1	65.89748368	0.98704109	0.437336	2.256939	0.024012	0.042424
13996	C1orf111	0.422589645	0.987018403	0.748324	1.318971	0.187179	0.262139
2555	TRUB1	643.5116703	0.986583776	0.153603	6.422936	1.34E-10	1.03E-09
581	KIAA1012	1062.139561	0.986450544	0.105252	9.372246	7.10E-21	2.40E-19
631	ACAD8	523.0937736	0.98566565	0.10709	9.204117	3.44E-20	1.07E-18
10510	TMEM132I	172.1815605	0.985611631	0.399242	2.468706	0.01356	0.02529
7312	HUNK	193.5353045	0.985254998	0.266612	3.695458	0.000219	0.000588
5247	SALL1	1442.870963	0.984914512	0.211681	4.65283	3.27E-06	1.22E-05
7929	DTNA	205.1406648	0.984315266	0.286401	3.436837	0.000589	0.001455
5198	GP1BA	19.637698	0.983640981	0.210254	4.678351	2.89E-06	1.09E-05
4871	TMEM8B	478.60696	0.982964986	0.202686	4.849698	1.24E-06	4.98E-06
3420	GKAP1	99.67283584	0.98281221	0.171553	5.728909	1.01E-08	5.79E-08
7636	NLRP14	5.556982543	0.982130766	0.275416	3.565986	0.000362	0.00093
8262	NRXN2	630.3822647	0.982086652	0.296919	3.307596	0.000941	0.002232
2262	C9orf82	517.133047	0.982072037	0.14686	6.687132	2.28E-11	1.97E-10
4764	CAPS2	60.30482646	0.981784551	0.200256	4.902637	9.46E-07	3.89E-06
4656	ZNF165	53.55399229	0.981086945	0.197894	4.957635	7.14E-07	3.00E-06
6256	FLT1	10056.70851	0.980998004	0.236249	4.152396	3.29E-05	0.000103
1451	RDX	2891.468712	0.980240949	0.128421	7.633053	2.29E-14	3.10E-13
9354	ADAMTS1	58.02310996	0.980234115	0.340534	2.878522	0.003995	0.008372
1698	ELMOD2	238.3086847	0.980094256	0.134361	7.294495	3.00E-13	3.46E-12
2392	IGF1R	2308.577443	0.979426203	0.149151	6.566661	5.15E-11	4.22E-10
8405	SMAD9	125.9050744	0.979294885	0.301582	3.247198	0.001165	0.002718
1860	DIP2C	1322.571966	0.979250444	0.137399	7.127038	1.03E-12	1.08E-11
1962	C2orf67	1345.718591	0.979186004	0.139516	7.018428	2.24E-12	2.24E-11
2938	TXNDC16	350.367188	0.978826969	0.161005	6.07947	1.21E-09	8.04E-09
5749	ZNF229	130.3604426	0.978632762	0.222535	4.39765	1.09E-05	3.73E-05
711	METTL14	318.9795088	0.978548418	0.108905	8.985364	2.58E-19	7.11E-18
1626	SPAG16	361.6376349	0.976426979	0.132096	7.391801	1.45E-13	1.75E-12
5934	ZNF813	76.12419317	0.976318125	0.226977	4.301401	1.70E-05	5.61E-05
3926	SLC45A4	761.5603886	0.976094901	0.181232	5.385895	7.21E-08	3.60E-07
2552	KIAA1549	471.0777463	0.976068537	0.151946	6.423784	1.33E-10	1.02E-09
5245	GPR155	582.5808909	0.975946191	0.209747	4.652978	3.27E-06	1.22E-05
509	SETD3	2519.552202	0.975400578	0.101872	9.574756	1.02E-21	3.93E-20

6426	ZNF233	28.04979529	0.975327097	0.240139	4.061509	4.88E-05	0.000149
5644	MPP6	206.3026448	0.975283239	0.219086	4.451598	8.52E-06	2.96E-05
6073	CCND1	18322.80926	0.974957583	0.230345	4.232605	2.31E-05	7.46E-05
13757	SCN7A	2.110188922	0.974514798	0.695138	1.401902	0.160945	0.229324
8975	TACR1	22.95148542	0.974083726	0.322328	3.02203	0.002511	0.005484
3118	DHTKD1	1304.340755	0.973675531	0.163641	5.950075	2.68E-09	1.68E-08
2672	NDUFA5	2575.170966	0.973548701	0.15433	6.308237	2.82E-10	2.07E-09
2190	PPARG	525.4274981	0.973501655	0.143807	6.769499	1.29E-11	1.16E-10
11174	TP53AIP1	3.426575259	0.97344305	0.436378	2.230734	0.025699	0.045081
664	EPM2A	164.7087357	0.972863216	0.106591	9.127033	7.04E-20	2.08E-18
2932	ATP6V1H	1335.228555	0.972643285	0.159825	6.085667	1.16E-09	7.76E-09
2038	UQCRC2	4449.710336	0.972297086	0.140462	6.922129	4.45E-12	4.28E-11
6022	DMGDH	1541.074653	0.972020444	0.228447	4.254915	2.09E-05	6.81E-05
1268	SENP8	80.1429281	0.971831046	0.122978	7.902457	2.73E-15	4.23E-14
13656	TSPAN19	1.226039907	0.971408243	0.675897	1.437214	0.150657	0.216264
3472	ZNF426	110.4923555	0.970455361	0.170417	5.694601	1.24E-08	6.98E-08
9774	ESPN	222.7379892	0.970419406	0.35624	2.72406	0.006448	0.012932
2944	FAM73A	816.8479356	0.970370438	0.15977	6.073535	1.25E-09	8.33E-09
2887	ATP5B	16525.29574	0.969728165	0.15841	6.121624	9.26E-10	6.29E-09
10628	CYP3A43	1.473267762	0.96961135	0.400223	2.422678	0.015407	0.028414
7567	SGK2	554.2989333	0.969445683	0.270059	3.589752	0.000331	0.000857
9010	TRIM7	78.28039435	0.969220506	0.322208	3.008055	0.002629	0.00572
10682	R5-ARHGA	14.43054	0.968809035	0.403302	2.402192	0.016297	0.029905
1755	FAM126B	682.3186027	0.968336108	0.133777	7.238416	4.54E-13	5.07E-12
3889	PELI2	292.6012638	0.967533894	0.178872	5.409088	6.33E-08	3.19E-07
3464	EYA3	123.3236745	0.967473607	0.169799	5.697747	1.21E-08	6.87E-08
12332	KCNA2	4.708778308	0.967307206	0.521456	1.855013	0.063594	0.10108
7092	ESM1	5234.461048	0.966669578	0.255194	3.787986	0.000152	0.00042
7593	PCOTH	15.87363111	0.966619848	0.26987	3.581795	0.000341	0.000881
4564	CCDC110	89.45063564	0.96633052	0.193168	5.002539	5.66E-07	2.43E-06
3255	ZIK1	76.97483066	0.966313859	0.165218	5.848728	4.95E-09	2.98E-08
3750	RFK	1096.314633	0.966121543	0.175454	5.506401	3.66E-08	1.91E-07
3963	CCNG1	4642.195396	0.965594425	0.179852	5.368831	7.92E-08	3.92E-07
2767	UNC13B	1408.023725	0.965330368	0.155115	6.223306	4.87E-10	3.45E-09
9798	C19orf26	11.25012101	0.965234831	0.355218	2.717302	0.006582	0.013167
6622	SLC15A2	62.99350843	0.96520775	0.242784	3.975581	7.02E-05	0.000208
2577	FBXL17	1000.342862	0.964984485	0.150689	6.403821	1.52E-10	1.15E-09
3094	C14orf159	1293.92419	0.964845663	0.161691	5.967219	2.41E-09	1.53E-08
1644	SLC35F5	1411.986039	0.964417773	0.130876	7.368965	1.72E-13	2.05E-12
2153	C18orf18	84.98032138	0.964302177	0.141651	6.807586	9.92E-12	9.04E-11
10373	LRRC2	38.77937931	0.964280106	0.383404	2.515051	0.011902	0.022489
3661	CLYBL	347.018573	0.964265476	0.173332	5.563101	2.65E-08	1.42E-07
13696	SVOP	5.665748742	0.964046319	0.67966	1.418425	0.156067	0.223355
1067	SLC25A44	1054.657536	0.963217358	0.117255	8.21472	2.13E-16	3.91E-15
2077	ZNF778	85.94888794	0.963135644	0.140115	6.87387	6.25E-12	5.90E-11
9259	ACSL6	26.97349755	0.962982872	0.330657	2.912331	0.003587	0.007594
2019	GLUD2	635.7393806	0.9628087	0.138751	6.939091	3.95E-12	3.83E-11

13094	IL1RAPL2	2.151833551	0.962252705	0.596615	1.612854	0.106776	0.159838
11770	HIST1H1T	0.735845181	0.960854129	0.473417	2.029614	0.042396	0.070602
565	FAM122A	545.1786004	0.960627543	0.102014	9.416636	4.66E-21	1.62E-19
10358	PM20D1	11.66118619	0.96034956	0.380532	2.523699	0.011613	0.021975
4012	FAM161A	138.7903718	0.960012572	0.180061	5.331599	9.74E-08	4.76E-07
6631	ASRGL1	721.903738	0.959613868	0.2416	3.971917	7.13E-05	0.000211
2828	PAR-SN	125.5618613	0.95957679	0.155657	6.164685	7.06E-10	4.89E-09
10038	ANK1	171.7758564	0.959262166	0.363467	2.639198	0.00831	0.016227
4713	ZMYND12	58.15611956	0.958552947	0.194548	4.927074	8.35E-07	3.47E-06
10405	CLEC18B	1459.398847	0.958009774	0.382018	2.507759	0.01215	0.022888
8916	CXXC4	6.613719536	0.957984531	0.314528	3.045787	0.002321	0.005102
9487	BCMO1	282.3958541	0.957649198	0.339252	2.822824	0.00476	0.009835
13646	EGR4	2.256005175	0.95714351	0.664717	1.439926	0.149888	0.215299
10526	SHISA9	375.6726157	0.956839131	0.388822	2.460864	0.01386	0.02581
5158	ATP9A	1543.536926	0.956643349	0.203661	4.697229	2.64E-06	1.00E-05
1665	PRKD1	401.6778894	0.956474662	0.130304	7.340336	2.13E-13	2.51E-12
12657	ALX4	0.703443261	0.955594297	0.544499	1.754997	0.07926	0.122744
1600	PPA2	960.3642344	0.955477558	0.128739	7.4218	1.16E-13	1.42E-12
7212	BLNK	354.8410297	0.95543489	0.255532	3.738996	0.000185	0.000502
13416	FOXN4	1.298325299	0.955283038	0.632957	1.509238	0.131238	0.191741
4231	C5orf4	802.9872822	0.954939197	0.183883	5.193188	2.07E-07	9.58E-07
5220	HRSP12	1711.437217	0.954613173	0.204391	4.670532	3.00E-06	1.13E-05
11262	PLD5	3.490351471	0.954062091	0.43487	2.193902	0.028242	0.049155
1222	ARMCX3	1990.304335	0.954041356	0.119394	7.9907	1.34E-15	2.15E-14
2911	MAPK10	247.0066244	0.953585608	0.156243	6.103235	1.04E-09	7.00E-09
5787	)C1001311	90.10184729	0.953556906	0.217768	4.378775	1.19E-05	4.04E-05
5687	ADM2	799.6974379	0.953513039	0.215385	4.427012	9.55E-06	3.29E-05
10095	VIP	9.779665314	0.953404019	0.364177	2.61797	0.008845	0.017175
1158	GFM1	1227.017996	0.952939154	0.117954	8.078928	6.53E-16	1.11E-14
4306	ATP6AP2	6491.616241	0.952814121	0.184859	5.154265	2.55E-07	1.16E-06
7778	KCNQ1	526.8708469	0.952667457	0.271571	3.507989	0.000452	0.001138
7321	SLC26A4	28.52169293	0.952611214	0.258079	3.691162	0.000223	0.000598
5536	MYO5C	375.7567631	0.952506204	0.2115	4.503572	6.68E-06	2.37E-05
14239	UGT2B28	1.011302006	0.95163622	0.764737	1.244397	0.213354	0.293696
7327	CCRL1	123.1942076	0.95140333	0.257903	3.688999	0.000225	0.000602
2818	PM20D2	597.2070807	0.951339228	0.154145	6.17173	6.75E-10	4.70E-09
3292	PLEKHB2	5144.093638	0.950891173	0.163374	5.820342	5.87E-09	3.50E-08
3025	ZNF573	27.48628765	0.95042276	0.157963	6.016748	1.78E-09	1.15E-08
1655	ADAL	186.290661	0.949494777	0.129149	7.35196	1.95E-13	2.31E-12
13088	C2orf54	13.41966116	0.949352419	0.587621	1.615586	0.106184	0.159024
2843	SYNM	1153.457242	0.949118806	0.154343	6.149401	7.78E-10	5.36E-09
1998	ZNF253	250.852717	0.948903716	0.136254	6.964221	3.30E-12	3.24E-11
8536	ST3GAL6	326.0885582	0.948805202	0.297093	3.193634	0.001405	0.003226
2441	UBA5	1783.455636	0.947969207	0.145418	6.518949	7.08E-11	5.69E-10
7448	THEM5	5.863831752	0.947776981	0.260528	3.637905	0.000275	0.000723
3276	PACSIN2	1420.459552	0.947688452	0.162514	5.831435	5.50E-09	3.29E-08
11783	LIPH	55.68474849	0.946850375	0.466898	2.027959	0.042564	0.070806

3866	SLC30A4	123.9139766	0.946737873	0.174515	5.424962	5.80E-08	2.94E-07
3741	CA5B	293.940085	0.945920364	0.171617	5.511824	3.55E-08	1.86E-07
1038	ZBTB26	83.16125364	0.945632975	0.114479	8.260345	1.45E-16	2.74E-15
725	FASTKD5	458.6524624	0.945315569	0.105631	8.949203	3.58E-19	9.68E-18
9056	NYX	11.09863128	0.944724663	0.315401	2.995309	0.002742	0.005934
3787	EZR	13310.68716	0.94390621	0.172234	5.480368	4.24E-08	2.20E-07
821	FAM179B	507.4298884	0.943557135	0.108373	8.706587	3.13E-18	7.48E-17
6961	GCSH	39.84987803	0.942965663	0.245429	3.84211	0.000122	0.000343
3270	SHPRH	286.5927239	0.942913833	0.16159	5.835215	5.37E-09	3.22E-08
4667	TSGA10	47.96285486	0.94271321	0.190323	4.953229	7.30E-07	3.07E-06
7819	MYH15	12.78797468	0.942673922	0.269644	3.495993	0.000472	0.001184
5419	HIST2H2BE	378.7782806	0.942535354	0.206852	4.556574	5.20E-06	1.88E-05
5840	ZNF714	127.0669992	0.94234312	0.216519	4.352245	1.35E-05	4.52E-05
4290	PPP1R3E	126.3628236	0.942288445	0.182592	5.160621	2.46E-07	1.12E-06
9019	RBM24	57.22473943	0.942201453	0.313532	3.005118	0.002655	0.00577
1703	ACBD5	1248.571027	0.94168386	0.129125	7.292811	3.04E-13	3.49E-12
3131	SGCB	3252.000033	0.941544162	0.158529	5.939262	2.86E-09	1.79E-08
949	SOCS4	622.951509	0.941531315	0.111446	8.448317	2.96E-17	6.10E-16
587	DNAJC27	204.3152048	0.941317996	0.100577	9.359211	8.03E-21	2.68E-19
8717	CLEC4F	10.24591557	0.940670967	0.301116	3.123944	0.001784	0.004012
3665	KLF9	3115.769488	0.940544676	0.169181	5.559414	2.71E-08	1.45E-07
2194	CCNC	1112.115173	0.940405534	0.13904	6.763553	1.35E-11	1.20E-10
2810	NDFIP2	1809.695172	0.940253938	0.152132	6.180525	6.39E-10	4.46E-09
7891	VCAM1	11945.00349	0.940096655	0.272255	3.452998	0.000554	0.001377
12581	LOC28385	1.916903433	0.93992014	0.528439	1.778673	0.075293	0.117306
12003	PROK1	1.06655096	0.939813497	0.479484	1.960052	0.04999	0.081634
16538	PSG2	0.717312107	0.939576166	1.669563	0.562768	0.573593	0.679828
329	BBS1	1126.133409	0.939413856	0.090126	10.42339	1.94E-25	1.16E-23
11272	C9orf11	0.763629669	0.939294276	0.428833	2.190349	0.028499	0.049557
773	PURA	515.1109299	0.939072461	0.106406	8.825339	1.09E-18	2.77E-17
3478	CLCN4	555.1569012	0.938770382	0.165051	5.687767	1.29E-08	7.25E-08
796	SECISBP2	943.6262454	0.938463081	0.107141	8.759154	1.97E-18	4.84E-17
10430	C9orf152	2.393558653	0.937463522	0.374973	2.500085	0.012416	0.023334
6846	SLC22A4	255.2505068	0.937181872	0.241484	3.880921	0.000104	0.000298
2229	LYSMD3	953.5067743	0.937066128	0.13943	6.720692	1.81E-11	1.59E-10
2906	ZNF583	78.5084336	0.937009733	0.153463	6.105752	1.02E-09	6.90E-09
705	ZNF181	270.3674817	0.936976395	0.1041	9.000697	2.24E-19	6.24E-18
15451	ZIC3	0.477676147	0.93664488	1.071404	0.874222	0.381997	0.484598
1996	ZFP30	194.8540786	0.935966038	0.134383	6.964891	3.29E-12	3.23E-11
4927	KLHL23	158.6446776	0.935681944	0.19442	4.812686	1.49E-06	5.92E-06
2450	CLCN3	1623.612593	0.935638229	0.14367	6.512412	7.40E-11	5.92E-10
1556	ZNF664	3481.163687	0.935020341	0.124904	7.485894	7.11E-14	8.95E-13
2745	C9orf5	2704.685675	0.93494854	0.149795	6.241527	4.33E-10	3.09E-09
10055	PROX1	17.06753709	0.93492878	0.354976	2.633781	0.008444	0.016461
9417	PTPRD	612.5243179	0.934873598	0.327793	2.852027	0.004344	0.009042
10708	ZYG11A	18.41456688	0.933797362	0.389955	2.394632	0.016637	0.030454
1719	NUDT9	706.453706	0.932711494	0.128142	7.278759	3.37E-13	3.84E-12

278	MOAP1	1063.31929	0.932506516	0.087182	10.69609	1.06E-26	7.48E-25
1141	NUBPL	273.1487424	0.931830424	0.114972	8.104841	5.28E-16	9.07E-15
10382	ATP2C2	21.33832237	0.930423555	0.370362	2.512198	0.011998	0.022652
15691	LOC28479€	0.258039914	0.930370053	1.148757	0.809893	0.418002	0.522163
16576	HOXB1	0.176551942	0.930366657	1.683148	0.552754	0.580432	0.686357
16943	TBX22	0.220673386	0.930363978	2.062627	0.451058	0.651948	0.754225
17169	UBE2U	0.1507643	0.93036064	2.359034	0.394382	0.693299	0.791505
8923	PGM5P2	4.477743941	0.93031208	0.305729	3.042932	0.002343	0.005147
4793	DSC2	782.2313126	0.929930372	0.190178	4.889781	1.01E-06	4.13E-06
3990	PRRG1	504.8291776	0.92808766	0.173517	5.34868	8.86E-08	4.35E-07
2827	PEX3	410.6641694	0.928068575	0.150521	6.1657	7.02E-10	4.87E-09
3240	SCAMP1	2105.122892	0.927983644	0.158345	5.860532	4.61E-09	2.79E-08
3113	PPP2R5A	1564.105693	0.927787828	0.155869	5.95236	2.64E-09	1.66E-08
1986	DCAF16	620.6919842	0.927694059	0.132827	6.984204	2.86E-12	2.83E-11
3821	FYCO1	1250.108682	0.927402676	0.169928	5.457609	4.83E-08	2.48E-07
2510	CC2D2A	513.549868	0.926630303	0.143571	6.454157	1.09E-10	8.50E-10
8057	RPL23AP64	5.634411192	0.926485137	0.273217	3.391026	0.000696	0.001694
14443	CYP1A1	4.946442555	0.92647293	0.782637	1.183784	0.236499	0.320877
945	MAPKSP1	1141.996299	0.926466398	0.109578	8.454884	2.79E-17	5.79E-16
1397	MIPEP	511.224648	0.926329231	0.119984	7.720441	1.16E-14	1.63E-13
602	CGRRF1	236.5836453	0.926254012	0.099513	9.307868	1.30E-20	4.25E-19
3905	TUFT1	548.5591648	0.92623715	0.171475	5.401585	6.61E-08	3.32E-07
758	UTP14C	1093.383455	0.926208769	0.104454	8.867172	7.50E-19	1.94E-17
5519	ZBTB20	39.12793403	0.926182907	0.205259	4.512254	6.41E-06	2.28E-05
7302	PFN2	2579.370529	0.925902616	0.250276	3.699528	0.000216	0.00058
6041	RAG1	91.38434534	0.925639753	0.217882	4.248349	2.15E-05	6.99E-05
2352	SEPSECS	613.4905109	0.925379209	0.1401	6.605132	3.97E-11	3.31E-10
1878	44084	1869.112947	0.925211789	0.130126	7.110111	1.16E-12	1.21E-11
4188	SLC12A2	783.9219089	0.924693379	0.177275	5.21616	1.83E-07	8.55E-07
5270	WASF3	235.36769	0.924076848	0.199091	4.64149	3.46E-06	1.29E-05
6864	TMEM117	124.416782	0.923582038	0.238249	3.876546	0.000106	0.000303
517	RANBP6	681.8719124	0.923483583	0.096775	9.542631	1.39E-21	5.28E-20
2212	PPP1R15B	2247.46197	0.923339571	0.136907	6.744298	1.54E-11	1.36E-10
8039	C2orf73	7.677231177	0.922962309	0.271765	3.396182	0.000683	0.001666
7489	PGM5	391.4681027	0.922716803	0.254873	3.620302	0.000294	0.00077
3991	SLC25A33	110.0265807	0.922237206	0.172541	5.345037	9.04E-08	4.44E-07
14739	PLCZ1	0.328250326	0.922172394	0.848297	1.087086	0.276999	0.368373
14765	IGLL1	0.385681389	0.921376616	0.853297	1.079785	0.280238	0.372025
11597	NTS	14.98958517	0.920647377	0.442376	2.081142	0.037421	0.063248
1094	LIN7C	923.5533943	0.920480314	0.112656	8.170736	3.07E-16	5.49E-15
3910	RAB7L1	978.5301919	0.919445783	0.170361	5.397045	6.77E-08	3.40E-07
807	ZNF782	75.18667077	0.91942644	0.105235	8.736882	2.40E-18	5.82E-17
11952	OMP	0.834512036	0.919398808	0.465663	1.974386	0.048338	0.079273
783	ZNF397	257.958822	0.9193674	0.104487	8.798891	1.38E-18	3.46E-17
2663	STXBP4	246.1706189	0.919309252	0.145526	6.317157	2.66E-10	1.96E-09
4554	ZNF699	37.6803003	0.919264988	0.183568	5.007763	5.51E-07	2.37E-06
10894	SLC6A13	3488.75005	0.919164813	0.395839	2.322065	0.020229	0.036394

2802	LMBRD1	2204.900613	0.919019309	0.14845	6.190756	5.99E-10	4.19E-09
3175	FLJ11235	57.81464838	0.918782459	0.155723	5.900121	3.63E-09	2.24E-08
10463	TRPA1	175.3107074	0.917946752	0.3689	2.488336	0.012834	0.024043
3197	DPP8	493.498907	0.917877373	0.155969	5.885001	3.98E-09	2.44E-08
348	WDR20	405.8754391	0.917836036	0.089111	10.29997	7.05E-25	3.97E-23
1906	EXOC5	1490.865102	0.917556147	0.129572	7.081449	1.43E-12	1.47E-11
13655	GOLGA6A	0.661133839	0.916917381	0.637996	1.437184	0.150666	0.216264
6095	FAT4	684.6653706	0.916531904	0.217048	4.222706	2.41E-05	7.76E-05
13445	ASTN1	10.15828792	0.916053313	0.609799	1.502222	0.13304	0.193937
10485	SOHLH2	15.4883147	0.915862191	0.369711	2.477239	0.01324	0.024752
1661	MAPK8	223.9125124	0.915461255	0.12466	7.343643	2.08E-13	2.45E-12
1389	EXOC6	414.4072584	0.915040296	0.118403	7.728212	1.09E-14	1.54E-13
3340	SVIP	403.9801727	0.91470675	0.157929	5.791888	6.96E-09	4.08E-08
8736	AGAP11	99.67524915	0.914468758	0.293624	3.114418	0.001843	0.004135
3208	TACC1	3929.554076	0.91419173	0.155476	5.879951	4.10E-09	2.51E-08
4935	ZNF431	158.9074745	0.914028934	0.190157	4.806715	1.53E-06	6.09E-06
5260	VAV3	1713.131635	0.913958376	0.196725	4.645873	3.39E-06	1.26E-05
2253	FARP2	250.6068508	0.913669624	0.136497	6.693701	2.18E-11	1.89E-10
9888	ESYT3	16.1207559	0.912952895	0.339246	2.691125	0.007121	0.014116
5532	SLC2A13	792.9397631	0.912671259	0.202601	4.504767	6.64E-06	2.35E-05
6942	LOC91316	445.6014393	0.912045501	0.237038	3.847678	0.000119	0.000337
10220	ZNF727	12.222741	0.911755739	0.354184	2.574241	0.010046	0.019267
2559	SAV1	823.6327824	0.911485305	0.142037	6.41725	1.39E-10	1.06E-09
10432	TRIM15	178.8158763	0.91146988	0.36464	2.49964	0.012432	0.023359
4756	PPAP2A	3485.183344	0.910918096	0.185656	4.906488	9.27E-07	3.82E-06
5036	ZNF540	87.05989054	0.910746552	0.191535	4.754995	1.98E-06	7.72E-06
3837	C3orf59	265.6723658	0.910341862	0.167289	5.441743	5.28E-08	2.69E-07
2849	HMBOX1	106.8468744	0.910232425	0.148154	6.143808	8.06E-10	5.54E-09
5756	EAF2	105.2652357	0.909881422	0.207123	4.392953	1.12E-05	3.81E-05
2378	TMEM135	978.4358851	0.909186282	0.138159	6.580738	4.68E-11	3.86E-10
7087	RAB3A	107.5868799	0.908569173	0.23975	3.789659	0.000151	0.000417
2473	ZNF441	239.4264601	0.908186536	0.139794	6.496585	8.22E-11	6.51E-10
8753	ALDH4A1	2167.48882	0.908035422	0.292419	3.105257	0.001901	0.004257
10846	LOC28410(	10.25097004	0.907755605	0.387657	2.341649	0.019199	0.034696
9218	SNCA	142.6367144	0.907430199	0.309401	2.932864	0.003359	0.007142
1862	ZNF718	85.38035937	0.907155025	0.12733	7.124446	1.05E-12	1.10E-11
5838	A2LD1	154.1192514	0.906855556	0.208328	4.353024	1.34E-05	4.51E-05
1320	TAB3	911.9785632	0.906564318	0.115691	7.83608	4.65E-15	6.90E-14
2017	FBXL3	1471.635258	0.906246827	0.130575	6.940416	3.91E-12	3.80E-11
4205	EDA	214.9297428	0.906169657	0.174089	5.205216	1.94E-07	9.03E-07
2266	CHM	798.0449565	0.905317304	0.135418	6.685348	2.30E-11	1.99E-10
10753	SPTBN4	64.97353337	0.904994143	0.38026	2.379934	0.017316	0.031564
1367	SNX4	1077.563555	0.904259742	0.116395	7.768891	7.92E-15	1.14E-13
9194	FBXL16	790.8126218	0.903585735	0.307154	2.941805	0.003263	0.006957
1569	CLN5	1249.305358	0.90309935	0.120895	7.470089	8.01E-14	1.00E-12
5746	SORBS2	1634.463397	0.902965785	0.205244	4.399471	1.09E-05	3.70E-05
1053	C1orf25	779.4544931	0.902636321	0.109567	8.238217	1.75E-16	3.25E-15

5899	MREG	165.2157741	0.902562971	0.208865	4.32127	1.55E-05	5.15E-05
2792	RIT1	1539.889311	0.901925101	0.145488	6.199288	5.67E-10	3.98E-09
5902	PCLO	315.7822843	0.901481277	0.208652	4.320496	1.56E-05	5.17E-05
3297	RARB	338.3706477	0.90105157	0.154879	5.817772	5.96E-09	3.55E-08
3921	RBBP8	1198.504589	0.900617292	0.167078	5.3904	7.03E-08	3.51E-07
7736	NRG3	118.0328086	0.900023458	0.255431	3.523546	0.000426	0.001079
7155	GALNAC	754.6049684	0.899857134	0.239176	3.762325	0.000168	0.000461
2200	SH3D19	1179.57776	0.899480177	0.133138	6.755984	1.42E-11	1.26E-10
5408	SLC25A40	230.1777044	0.899370563	0.19721	4.560461	5.10E-06	1.85E-05
3874	ERBB2	2526.964145	0.89891675	0.165827	5.420817	5.93E-08	3.00E-07
6970	RAB17	965.0223331	0.898849499	0.234069	3.840109	0.000123	0.000346
9883	RNF150	77.38340094	0.898720329	0.333687	2.693303	0.007075	0.014031
2934	SPIRE1	2039.691992	0.89868131	0.147789	6.080833	1.20E-09	7.99E-09
3848	CCDC87	19.90758433	0.898572793	0.165345	5.434533	5.49E-08	2.80E-07
4992	DNAJB4	800.7258922	0.898250241	0.188003	4.777838	1.77E-06	6.96E-06
1450	ARID4A	623.8917535	0.89823502	0.117645	7.635114	2.26E-14	3.05E-13
12798	AMAC1L3	0.420891555	0.8975521	0.526638	1.704307	0.088324	0.135274
1159	ZNF223	92.47683771	0.897312968	0.111087	8.077546	6.61E-16	1.12E-14
6006	LONRF3	70.2694282	0.897134669	0.210375	4.264447	2.00E-05	6.54E-05
2584	PCGF5	2326.435249	0.896975922	0.140229	6.396509	1.59E-10	1.21E-09
4275	COQ3	129.1060979	0.896953317	0.173539	5.168603	2.36E-07	1.08E-06
1200	ZBTB44	2067.384141	0.896198763	0.111823	8.014429	1.11E-15	1.81E-14
5792	GRTP1	150.1487524	0.896187885	0.204745	4.377101	1.20E-05	4.07E-05
8468	TFEC	1028.529598	0.895919929	0.278248	3.219866	0.001283	0.002969
12669	CTXN2	2.370187878	0.895346894	0.512236	1.74792	0.080478	0.124512
7957	ABTB2	393.2476189	0.895112966	0.261408	3.424205	0.000617	0.001519
7144	MAN2A1	1275.285475	0.894995352	0.237672	3.765668	0.000166	0.000456
5410	SPATA13	563.631063	0.89446575	0.196156	4.559976	5.12E-06	1.85E-05
1296	TSC1	979.5434357	0.894444307	0.113635	7.871189	3.51E-15	5.31E-14
9282	CD207	14.84576091	0.894417237	0.30778	2.90603	0.00366	0.00773
6078	LDB2	1256.848026	0.894290614	0.211417	4.229978	2.34E-05	7.54E-05
1409	MTM1	399.5394062	0.894026031	0.116281	7.688506	1.49E-14	2.07E-13
3421	ATP7A	664.3550283	0.893897093	0.156065	5.727713	1.02E-08	5.83E-08
4752	DDR1	3751.471505	0.893694243	0.182099	4.907748	9.21E-07	3.80E-06
1467	GCLC	884.5014781	0.893563812	0.117321	7.616374	2.61E-14	3.49E-13
7165	GPR157	21.43631929	0.893245842	0.237734	3.757328	0.000172	0.00047
8976	DSP	2796.076815	0.893132773	0.295595	3.021478	0.002515	0.005493
6905	BANK1	165.403198	0.893051672	0.23123	3.862185	0.000112	0.000319
14460	KLK2	2.130447866	0.892813624	0.756911	1.179548	0.23818	0.322876
7812	WDR65	4.904547177	0.892437314	0.255193	3.497107	0.00047	0.00118
6065	CTSF	1700.825016	0.891823781	0.210443	4.237838	2.26E-05	7.29E-05
1379	C16orf52	399.5913014	0.891815317	0.114991	7.755513	8.80E-15	1.25E-13
9602	C6orf138	108.8020841	0.890839755	0.320511	2.779438	0.005445	0.011115
6015	RAD54L2	158.9513392	0.890579848	0.209185	4.257387	2.07E-05	6.74E-05
9716	GRM8	141.0046866	0.890177113	0.324502	2.743213	0.006084	0.012274
1538	SLC12A6	798.5453449	0.889725148	0.118506	7.507828	6.01E-14	7.66E-13
1166	ALDH9A1	2720.044626	0.889347183	0.110233	8.06785	7.15E-16	1.20E-14

5097	ARHGAP42	757.8627409	0.889345883	0.188093	4.728229	2.26E-06	8.71E-06
4063	DAAM1	229.1059829	0.889111772	0.167781	5.299244	1.16E-07	5.61E-07
8387	SLC6A12	1234.6251	0.888599315	0.272947	3.255579	0.001132	0.002645
2265	ALS2CR8	180.7331755	0.888345315	0.132871	6.685786	2.30E-11	1.99E-10
117	NUMB	2097.913045	0.888303809	0.073478	12.08939	1.20E-33	2.01E-31
9479	FAM106C	7.599978386	0.887631629	0.314114	2.82583	0.004716	0.009752
2689	ZNF649	183.1502661	0.887409341	0.141045	6.291666	3.14E-10	2.29E-09
3272	CBX7	1779.740594	0.886000632	0.151868	5.834005	5.41E-09	3.24E-08
1045	PREPL	2412.716665	0.885898069	0.107456	8.244255	1.66E-16	3.12E-15
1083	RNF38	1183.317562	0.885773216	0.108224	8.184608	2.73E-16	4.94E-15
2415	MBIP	325.8299333	0.88575712	0.135343	6.544547	5.97E-11	4.84E-10
15450	RHOXF2B	0.441340312	0.88530673	1.012675	0.874226	0.381995	0.484598
10782	DOCK3	24.42187127	0.885151146	0.373976	2.366866	0.017939	0.03261
6295	LOC646471	89.3311749	0.885121368	0.214356	4.12922	3.64E-05	0.000113
3867	PDHA1	2161.079594	0.884396184	0.163084	5.422963	5.86E-08	2.97E-07
1011	ZNF260	553.9580512	0.884211335	0.106237	8.323014	8.58E-17	1.66E-15
1507	SIK2	1213.918211	0.884187499	0.117021	7.555779	4.16E-14	5.42E-13
1461	MMAA	447.657651	0.884031129	0.115948	7.624363	2.45E-14	3.29E-13
10659	TEDDM1	1.973129298	0.883999334	0.367027	2.408542	0.016016	0.029453
12341	TCEB3B	0.74752543	0.883183699	0.477054	1.851329	0.064122	0.101844
4548	TMEM106B	2722.064348	0.883075739	0.176254	5.010241	5.44E-07	2.34E-06
3389	ISCU	2477.908415	0.882958257	0.153414	5.755394	8.64E-09	5.00E-08
1972	PRDX3	4135.987199	0.881916224	0.125991	6.999811	2.56E-12	2.55E-11
1250	TSPYL4	749.2722493	0.881800852	0.111172	7.931831	2.16E-15	3.39E-14
1144	TAPT1	762.0703396	0.881777747	0.108903	8.096885	5.64E-16	9.66E-15
2305	ZNRF2	738.4658159	0.881359778	0.132553	6.649117	2.95E-11	2.51E-10
2673	DHRS4	951.6610089	0.881143864	0.139709	6.306972	2.85E-10	2.09E-09
599	ZNF24	1921.270763	0.881064489	0.094562	9.317354	1.19E-20	3.90E-19
1684	ISOC1	587.743741	0.881059801	0.120404	7.31751	2.53E-13	2.94E-12
7082	SNORD116-2	11.7920657	0.880645381	0.232303	3.790939	0.00015	0.000415
4233	ZNF192	441.6118767	0.880033384	0.16948	5.19255	2.07E-07	9.61E-07
10170	SCUBE2	103.1466607	0.879956099	0.339327	2.593238	0.009508	0.018325
9272	FAM13C	281.2532811	0.879148527	0.302273	2.908454	0.003632	0.007678
1196	DCTN6	705.3280616	0.879109541	0.109628	8.018992	1.07E-15	1.75E-14
3618	TMEM55A	429.4178774	0.878848933	0.157102	5.594129	2.22E-08	1.20E-07
3599	SUGT1L1	29.42019913	0.878683859	0.156747	5.605751	2.07E-08	1.13E-07
9006	UCKL1AS	19.65624731	0.877924278	0.291754	3.00912	0.00262	0.005702
4213	RASSF8	720.5020286	0.877745131	0.168729	5.202115	1.97E-07	9.17E-07
10480	AQP7P1	8.835360397	0.877727115	0.354025	2.479283	0.013165	0.024622
5199	TMTC2	508.0406586	0.8773622	0.187551	4.677987	2.90E-06	1.09E-05
14008	HTR4	1.025780012	0.877352471	0.666685	1.315993	0.188176	0.2633
3781	CRYL1	2883.767323	0.877268084	0.159918	5.48573	4.12E-08	2.13E-07
2875	HADH	1246.175862	0.877172846	0.143201	6.125475	9.04E-10	6.16E-09
11485	CSMD1	30.07636708	0.877145251	0.412569	2.126057	0.033498	0.057171
12329	NAPSA	539.6328401	0.87649854	0.472335	1.855673	0.0635	0.100954
4023	EPB49	1515.567853	0.875905361	0.164427	5.327027	9.98E-08	4.86E-07
2529	ZNF708	158.3107761	0.875876634	0.136017	6.439475	1.20E-10	9.29E-10

4198	PTPRG	1475.148783	0.875808609	0.168042	5.21185	1.87E-07	8.73E-07
906	C14orf104	221.0114466	0.875411521	0.102865	8.51028	1.74E-17	3.75E-16
9644	FGF17	3.154375188	0.874837195	0.316437	2.764645	0.005698	0.011582
1549	IREB2	1407.320124	0.874488627	0.116718	7.492297	6.77E-14	8.56E-13
4747	FAM175A	369.0623066	0.873777933	0.177966	4.909802	9.12E-07	3.76E-06
871	STX17	863.502238	0.873516289	0.101601	8.597531	8.14E-18	1.83E-16
5251	FGD4	1338.881453	0.873368304	0.187802	4.650478	3.31E-06	1.24E-05
1596	ZCCHC14	1076.40462	0.873239865	0.117581	7.426698	1.11E-13	1.37E-12
15248	TRIM40	0.299359491	0.872663281	0.934942	0.933388	0.35062	0.450715
16348	OC1001924	0.231983129	0.872660901	1.419277	0.614863	0.538645	0.645827
17065	RBP3	0.20402651	0.872656264	2.066815	0.422223	0.672862	0.772855
17075	PFN3	0.177860308	0.872656026	2.080259	0.419494	0.674855	0.77469
17083	C2orf14	0.152198729	0.872655645	2.095118	0.416519	0.67703	0.776823
17266	GSTA3	0.165282362	0.872653592	2.331156	0.374344	0.708149	0.803799
1583	PCNX	1474.654546	0.872634425	0.117241	7.443069	9.84E-14	1.22E-12
11433	NECAB2	86.7323009	0.872406974	0.407848	2.139048	0.032432	0.055602
9358	TRPC3	7.652883429	0.871751054	0.303008	2.876993	0.004015	0.008409
2412	ATP6V1C1	2536.840398	0.871362731	0.133099	6.546727	5.88E-11	4.78E-10
8278	SPAG5	520.5259536	0.87134657	0.263941	3.301291	0.000962	0.002279
2170	ZNF709	99.16001573	0.871101736	0.128236	6.792935	1.10E-11	9.92E-11
12218	GALNT9	184.2756598	0.870941247	0.460523	1.891199	0.058598	0.093999
2907	THAP9	155.8768628	0.870699536	0.142609	6.105503	1.02E-09	6.91E-09
3940	GPAM	324.7212703	0.870524879	0.161831	5.379223	7.48E-08	3.72E-07
6154	ZNF418	72.01866267	0.870497384	0.20721	4.201034	2.66E-05	8.46E-05
1743	USP38	877.2901515	0.870434318	0.120033	7.251654	4.12E-13	4.63E-12
10611	ADCYAP1	21.91454437	0.870398422	0.357746	2.433008	0.014974	0.02766
11903	TMC5	20.19731044	0.869699936	0.437719	1.98689	0.046935	0.077288
5651	ZNF610	70.38871008	0.869304042	0.195496	4.446651	8.72E-06	3.03E-05
8883	LOC349114	153.5708745	0.869303871	0.28421	3.058666	0.002223	0.004906
13357	ZIM2	0.538657834	0.86926165	0.570076	1.524818	0.127304	0.186815
4497	MPDZ	902.8053932	0.86920901	0.172449	5.040383	4.65E-07	2.03E-06
2967	MAP4K3	820.9979444	0.869207706	0.143498	6.057296	1.38E-09	9.15E-09
8372	HOXB8	145.757874	0.86882563	0.26639	3.261479	0.001108	0.002595
1078	SIRT5	448.2811242	0.868619981	0.105992	8.195129	2.50E-16	4.55E-15
2904	NFAT5	2326.65184	0.868346909	0.142145	6.108898	1.00E-09	6.77E-09
4403	PIK3R3	1755.023659	0.867997927	0.170133	5.101876	3.36E-07	1.50E-06
16028	RDH8	1.06943079	0.867881144	1.234986	0.702746	0.482214	0.589729
5789	ZNF793	101.52942	0.867716643	0.198163	4.378797	1.19E-05	4.04E-05
2069	TRAPPC6E	708.9677944	0.86765529	0.126101	6.880639	5.96E-12	5.64E-11
5860	NOSTRIN	681.8588104	0.867474404	0.199827	4.341136	1.42E-05	4.74E-05
1201	ATL2	997.9984794	0.867438148	0.108253	8.01309	1.12E-15	1.83E-14
1988	ARHGAP32	769.3581755	0.867388295	0.124303	6.978036	2.99E-12	2.95E-11
1964	AP4S1	84.13758919	0.86726189	0.123654	7.013639	2.32E-12	2.32E-11
2751	DMXL1	1560.809907	0.866588355	0.138898	6.239021	4.40E-10	3.14E-09
1177	ZNF555	87.69491522	0.866234455	0.107552	8.054075	8.01E-16	1.33E-14
2367	ZNF620	41.78546163	0.865895629	0.13136	6.59178	4.35E-11	3.60E-10
686	SNAPC3	593.9292913	0.865738506	0.095702	9.04615	1.48E-19	4.23E-18

10843	MST1P9	165.1931944	0.865691138	0.369453	2.343169	0.019121	0.034565
1548	LOC65350	148.4145291	0.865427982	0.115497	7.493047	6.73E-14	8.52E-13
6301	ACER2	92.77024499	0.865416768	0.20973	4.126347	3.69E-05	0.000115
4082	C1orf192	27.51695967	0.865151094	0.163536	5.290271	1.22E-07	5.86E-07
5505	ARL5B	299.3356513	0.865132201	0.191532	4.516914	6.27E-06	2.23E-05
597	TOPORS	574.7976156	0.864908541	0.092715	9.32864	1.07E-20	3.52E-19
3142	KLHL24	1865.711629	0.864817151	0.145824	5.930563	3.02E-09	1.88E-08
11486	HPSE2	1.968932198	0.863919079	0.406414	2.125714	0.033527	0.057214
4662	LRP11	995.2410627	0.863858834	0.17435	4.954733	7.24E-07	3.05E-06
9454	BTNL8	5.801135071	0.863796519	0.30455	2.836302	0.004564	0.009462
812	KIAA1826	439.5340366	0.862813434	0.098859	8.727759	2.60E-18	6.27E-17
5584	SATB1	564.7081002	0.862683611	0.192513	4.481175	7.42E-06	2.61E-05
2117	UBR3	1301.559818	0.861246366	0.126076	6.831175	8.42E-12	7.80E-11
4741	PTCH1	320.9902999	0.86123538	0.175287	4.913295	8.96E-07	3.70E-06
7405	PRKCD	1110.521727	0.861188202	0.235618	3.655018	0.000257	0.000681
13057	UGT3A1	741.2508244	0.860306236	0.528636	1.627408	0.10365	0.155599
6828	OC1002722	29.83540942	0.860155036	0.221197	3.888636	0.000101	0.000289
434	HDHD2	770.7498699	0.859995112	0.087065	9.877641	5.20E-23	2.35E-21
1877	OPHN1	164.6591299	0.859361104	0.120863	7.110229	1.16E-12	1.21E-11
1891	IKZF5	533.1141712	0.859289124	0.121162	7.092087	1.32E-12	1.37E-11
5819	CEP152	118.1990074	0.858833467	0.196809	4.363788	1.28E-05	4.31E-05
2675	SNRK	2229.621291	0.858644987	0.136238	6.302556	2.93E-10	2.15E-09
7488	HOXA7	280.6764469	0.858382433	0.237077	3.620694	0.000294	0.000769
6619	CYYR1	1066.290205	0.858167032	0.215804	3.976601	6.99E-05	0.000207
826	TMEM170A	679.4978895	0.858147025	0.098709	8.693698	3.51E-18	8.33E-17
1929	WWC2	1247.116985	0.857791984	0.121553	7.05692	1.70E-12	1.73E-11
3808	AGPAT5	1387.191724	0.857704434	0.156871	5.467577	4.56E-08	2.35E-07
9901	FAM23A	14.35964886	0.85605722	0.318537	2.687462	0.0072	0.014253
833	ZFYVE9	611.660309	0.856054795	0.098713	8.672166	4.24E-18	9.98E-17
1653	KIF3A	427.5727149	0.855995934	0.116385	7.354877	1.91E-13	2.27E-12
8669	CRNA0008	350.6965854	0.855819027	0.272154	3.144615	0.001663	0.00376
7631	SLC35F1	64.79543281	0.855099592	0.239716	3.567143	0.000361	0.000927
4121	C9orf103	153.6088988	0.854844095	0.162218	5.269739	1.37E-07	6.50E-07
4719	SLC25A25	470.1420956	0.854831673	0.173627	4.923392	8.51E-07	3.53E-06
5037	ZNF829	14.97127036	0.85466249	0.179739	4.755032	1.98E-06	7.72E-06
2286	HOXB2	412.9323401	0.854153336	0.128111	6.667269	2.61E-11	2.23E-10
1936	KIAA2026	746.8649306	0.854102133	0.121218	7.046013	1.84E-12	1.86E-11
8050	SESN3	162.2832215	0.853970528	0.251746	3.392195	0.000693	0.001688
4383	AASS	441.8073419	0.853911728	0.167001	5.113228	3.17E-07	1.42E-06
11492	FBXO2	147.5667551	0.853756474	0.402193	2.122755	0.033774	0.057606
6088	NKRD36B	159.332864	0.852989855	0.201811	4.226683	2.37E-05	7.64E-05
9193	LOC44090	20.14666102	0.852266518	0.289704	2.941848	0.003263	0.006956
12262	MMP21	1.096892958	0.852198082	0.453597	1.878754	0.060278	0.096355
2390	COX11	1141.14795	0.852098961	0.129712	6.569155	5.06E-11	4.15E-10
5504	MAP3K13	120.4502503	0.852027656	0.188589	4.517916	6.25E-06	2.22E-05
5987	ASXL2	740.0802287	0.851858809	0.199278	4.274736	1.91E-05	6.27E-05
3703	ACAD10	885.5177251	0.851844872	0.153932	5.533888	3.13E-08	1.66E-07

1300	CLASP2	896.6690017	0.851804387	0.108298	7.865355	3.68E-15	5.55E-14
3656	TRAK1	1423.91612	0.85171612	0.153016	5.566203	2.60E-08	1.40E-07
2361	ZNF420	142.7348018	0.851442218	0.129055	6.597498	4.18E-11	3.47E-10
5366	KLF10	3066.777414	0.851256703	0.185689	4.584315	4.55E-06	1.66E-05
2563	GAB1	717.5180691	0.850690619	0.132619	6.414522	1.41E-10	1.08E-09
3143	KCTD6	176.0451635	0.850448914	0.143448	5.928601	3.06E-09	1.91E-08
11975	PCDHA12	31.23404358	0.849948431	0.432245	1.966358	0.049257	0.080626
8186	LRRC4	111.1278116	0.849387736	0.254381	3.339036	0.000841	0.002013
1768	GNE	974.6212486	0.849344334	0.117595	7.222605	5.10E-13	5.65E-12
2725	BBX	1872.382884	0.849288351	0.135673	6.25982	3.85E-10	2.77E-09
9082	RASEF	128.4490404	0.849217759	0.284427	2.985718	0.002829	0.006106
1017	PCMTD2	1642.089993	0.84834412	0.102106	8.308451	9.70E-17	1.87E-15
12885	SLC6A3	2849.540989	0.848038104	0.505356	1.6781	0.093328	0.141972
1676	TRAK2	1839.204816	0.84740119	0.115635	7.328248	2.33E-13	2.73E-12
6204	VN1R1	14.63675369	0.847381656	0.202985	4.174606	2.99E-05	9.43E-05
2964	TPMT	1479.793287	0.847314077	0.139853	6.0586	1.37E-09	9.08E-09
10455	LOC28541	6.761137972	0.847042057	0.340121	2.490412	0.01276	0.023922
6405	EVI5L	430.2496609	0.846896558	0.207829	4.07496	4.60E-05	0.000141
9258	PNMA6A	409.6898251	0.84676877	0.290628	2.913586	0.003573	0.007565
1312	C5orf44	577.7576717	0.846454865	0.107888	7.845685	4.31E-15	6.43E-14
7675	PARD3B	82.78855776	0.845797917	0.238335	3.548773	0.000387	0.000988
3159	FAM82A1	135.1811528	0.845749475	0.143053	5.912143	3.38E-09	2.10E-08
1526	ZFP3	251.9648058	0.845636287	0.112453	7.519903	5.48E-14	7.04E-13
541	ZNF776	440.5760371	0.84535218	0.089066	9.491323	2.28E-21	8.27E-20
10073	KLF15	454.4174823	0.845341639	0.32176	2.627244	0.008608	0.01675
2487	LYPLAL1	653.4130567	0.845311726	0.130366	6.484137	8.92E-11	7.03E-10
12486	PON3	24.01074316	0.844983575	0.467472	1.80756	0.070675	0.110948
7879	C10orf108	157.1988215	0.84494976	0.244301	3.458649	0.000543	0.00135
6321	PPTC7	944.2581932	0.843831738	0.205012	4.116014	3.85E-05	0.00012
2823	CENPC1	314.8328365	0.843645552	0.136785	6.167675	6.93E-10	4.81E-09
823	OCIAD1	2978.483175	0.843354074	0.096915	8.702005	3.26E-18	7.77E-17
682	ZNF329	285.3415938	0.843060572	0.09308	9.057346	1.34E-19	3.84E-18
5637	TMEM64	1330.784679	0.842502952	0.18921	4.45274	8.48E-06	2.95E-05
3056	SPTBN1	13832.74181	0.842182939	0.140518	5.993435	2.05E-09	1.32E-08
2395	ARHGAP12	807.0174534	0.841413497	0.128194	6.56358	5.25E-11	4.30E-10
4189	PLEKHA1	3151.852327	0.841085639	0.161252	5.21596	1.83E-07	8.56E-07
1123	NAA35	306.5683009	0.840609089	0.103455	8.125349	4.46E-16	7.79E-15
8585	CHCHD10	1062.738801	0.840488415	0.264425	3.178551	0.00148	0.003379
3184	HERC1	1611.966452	0.840438004	0.142653	5.891468	3.83E-09	2.36E-08
1524	C9orf80	204.7612453	0.839844312	0.111635	7.523108	5.35E-14	6.88E-13
931	PPP2R1B	823.6685558	0.839053511	0.098951	8.479453	2.26E-17	4.76E-16
7802	KIF12	912.0726733	0.839040554	0.239638	3.501281	0.000463	0.001163
7745	PDE8B	89.60264095	0.83895727	0.238213	3.521883	0.000428	0.001084
9326	EBF3	84.03361632	0.83886774	0.290269	2.889971	0.003853	0.008098
1763	IDI1	701.9529195	0.838719959	0.116028	7.228619	4.88E-13	5.42E-12
1983	ZNF252	560.6964415	0.838600582	0.120043	6.985862	2.83E-12	2.80E-11
1707	RBM18	638.837195	0.838474161	0.11503	7.289191	3.12E-13	3.58E-12

2353	KRAS	1145.472716	0.838258513	0.12693	6.604078	4.00E-11	3.33E-10
6176	SHMT1	3038.036612	0.838010849	0.200143	4.187052	2.83E-05	8.97E-05
2382	TOM1L2	1262.209479	0.837963724	0.127434	6.575661	4.84E-11	3.99E-10
1495	CDK20	230.1916885	0.837363561	0.110534	7.575639	3.57E-14	4.69E-13
1638	DCAF10	1018.513222	0.836422688	0.113403	7.375652	1.64E-13	1.96E-12
9204	ZNF667	57.44047408	0.836412591	0.284876	2.936062	0.003324	0.007079
838	USP8	1274.79758	0.836210843	0.096644	8.652499	5.04E-18	1.18E-16
3084	GCC2	1330.148811	0.836189384	0.139987	5.97332	2.32E-09	1.48E-08
2474	ZNF440	242.2140826	0.835990254	0.128717	6.494808	8.31E-11	6.59E-10
12972	NRG4	8.350574726	0.835156505	0.50561	1.651779	0.09858	0.148956
2755	KIAA1632	906.4488246	0.835091272	0.133914	6.236034	4.49E-10	3.19E-09
7276	ZNF677	37.52029379	0.834928032	0.224995	3.710871	0.000207	0.000556
2376	CHD9	1566.84065	0.834559404	0.126736	6.585025	4.55E-11	3.75E-10
6881	ZNF285	47.04367985	0.834493728	0.215556	3.871357	0.000108	0.000308
2471	FAM8A1	1435.545511	0.833375033	0.128259	6.497602	8.16E-11	6.47E-10
8009	DHRS4L1	18.40057535	0.832535811	0.244421	3.406157	0.000659	0.001612
7704	ZNF257	35.08076284	0.832369434	0.235162	3.539555	0.000401	0.00102
3026	PRKAB1	1715.077289	0.832331628	0.138367	6.015374	1.79E-09	1.16E-08
8451	NGFR	537.1311982	0.831996555	0.257808	3.227201	0.00125	0.002899
9197	PTH2R	59.65752532	0.831823238	0.282771	2.941686	0.003264	0.006958
1345	LARP1B	509.4813864	0.831782793	0.106587	7.803798	6.01E-15	8.75E-14
9480	CCNI2	12.34993087	0.831733635	0.294346	2.825697	0.004718	0.009755
7077	NCOA2	741.7935389	0.830896698	0.219092	3.792455	0.000149	0.000413
11266	TRIM61	5.311711975	0.830846992	0.378852	2.193065	0.028303	0.049246
2164	CDC37L1	352.9333329	0.830265131	0.122147	6.797254	1.07E-11	9.66E-11
1330	STXBP3	1171.734294	0.830247652	0.106091	7.825785	5.04E-15	7.44E-14
6203	DEPDC4	7.581354766	0.830118421	0.198843	4.174734	2.98E-05	9.43E-05
1105	ZMYND11	1965.638678	0.829982377	0.101788	8.154042	3.52E-16	6.24E-15
8012	CYP4X1	84.85873038	0.829972326	0.243706	3.405636	0.00066	0.001615
788	EXOC8	507.686606	0.829834733	0.094463	8.784728	1.57E-18	3.90E-17
2243	DNAJC25	290.6488118	0.829801395	0.123798	6.702861	2.04E-11	1.79E-10
5873	FZD5	970.038212	0.829508623	0.191315	4.335831	1.45E-05	4.85E-05
5411	HIPK3	1433.595609	0.829483972	0.181908	4.559917	5.12E-06	1.85E-05
3334	FBXO30	445.1119383	0.829314919	0.143121	5.794501	6.85E-09	4.03E-08
10291	LOC64575[illegible]	2.853825272	0.829293762	0.325353	2.548904	0.010806	0.020582
2516	ISCA2	385.8921909	0.829229852	0.128594	6.448453	1.13E-10	8.80E-10
1851	ADCY9	1123.417824	0.828207136	0.116088	7.134334	9.73E-13	1.03E-11
4551	PDZD8	722.1223063	0.828164768	0.165334	5.009026	5.47E-07	2.36E-06
2377	PDHX	640.7614026	0.828071886	0.12576	6.58453	4.56E-11	3.76E-10
8148	TDRD6	22.44672106	0.82772831	0.24685	3.353168	0.000799	0.001922
849	THAP6	356.3446855	0.82769013	0.095825	8.637516	5.74E-18	1.33E-16
6454	C4orf32	526.5962801	0.827426232	0.204432	4.047442	5.18E-05	0.000157
158	DNAJC16	724.4599028	0.827127555	0.071331	11.59555	4.34E-31	5.35E-29
2475	SMC5	709.4336098	0.827116167	0.127359	6.494367	8.34E-11	6.60E-10
899	KIAA1704	294.7066543	0.826943064	0.097013	8.524038	1.54E-17	3.36E-16
3162	HERC3	922.1954081	0.826851969	0.139888	5.910826	3.40E-09	2.11E-08
5258	USP12	437.07421	0.826563547	0.177889	4.646505	3.38E-06	1.26E-05

2005	FAM188A	494.4415631	0.82627145	0.118868	6.951159	3.62E-12	3.54E-11
7459	RGPD5	81.31092293	0.825879315	0.227298	3.633464	0.00028	0.000735
3695	PPFIBP1	2707.820477	0.825802638	0.149137	5.537209	3.07E-08	1.63E-07
2528	SYPL1	3923.308386	0.825478391	0.128169	6.440546	1.19E-10	9.23E-10
1990	SSFA2	2431.830834	0.825207232	0.118268	6.977457	3.01E-12	2.96E-11
2371	GNPDA2	413.0922633	0.825163527	0.125247	6.588288	4.45E-11	3.68E-10
6809	CCDC147	12.52585875	0.825002213	0.211767	3.895799	9.79E-05	0.000282
2540	KIAA1370	762.6199162	0.824992021	0.12829	6.430659	1.27E-10	9.80E-10
5150	CLOCK	375.022527	0.824857366	0.175405	4.702587	2.57E-06	9.78E-06
15367	GABRA5	2.121318601	0.824074084	0.915721	0.899918	0.368164	0.469602
8933	SIX1	54.70194361	0.823895033	0.27101	3.040095	0.002365	0.005189
10027	FABP3	1627.784975	0.822989872	0.311411	2.642779	0.008223	0.016074
1866	AASDHPPT	1175.776587	0.822872664	0.115535	7.122291	1.06E-12	1.12E-11
938	SDHC	2508.308225	0.822853529	0.097174	8.467839	2.50E-17	5.22E-16
3776	ETNK1	1812.163939	0.822585954	0.149861	5.488989	4.04E-08	2.10E-07
3564	GSTA4	423.67883	0.82230732	0.146135	5.627054	1.83E-08	1.01E-07
5216	ZNF382	69.4631593	0.822292441	0.17599	4.67238	2.98E-06	1.12E-05
4081	B3GALNT1	777.7069455	0.822016739	0.15538	5.290362	1.22E-07	5.86E-07
478	LCMT2	279.4377295	0.821824615	0.084682	9.70486	2.87E-22	1.18E-20
8237	TLR3	1351.679513	0.821629567	0.247699	3.317051	0.00091	0.002165
8461	SEMA3G	417.0691119	0.821233936	0.254852	3.222392	0.001271	0.002945
9911	CAPN3	373.0610916	0.821223605	0.305863	2.68494	0.007254	0.014347
1886	FKTN	598.2485729	0.821144294	0.115667	7.099194	1.25E-12	1.30E-11
6276	KCNK5	618.3004136	0.820994204	0.198237	4.141481	3.45E-05	0.000108
3326	ABHD10	1128.448963	0.820851682	0.141535	5.799638	6.65E-09	3.92E-08
4364	GLS	5702.043682	0.820381912	0.160111	5.123818	2.99E-07	1.34E-06
12575	C1QL4	113.3743988	0.820206077	0.460892	1.779608	0.07514	0.117123
2990	C18orf32	1489.674757	0.81878223	0.135476	6.043749	1.51E-09	9.87E-09
3266	SERINC1	7823.122846	0.818668798	0.140258	5.836898	5.32E-09	3.19E-08
5640	TBC1D9	1201.488111	0.817718467	0.183677	4.451947	8.51E-06	2.96E-05
2566	XPO4	691.5310245	0.81757606	0.127503	6.412223	1.43E-10	1.10E-09
2002	FAM36A	1217.970912	0.817461394	0.117531	6.95531	3.52E-12	3.44E-11
4194	LYRM5	464.4043184	0.817362969	0.156798	5.212845	1.86E-07	8.69E-07
2800	IFT88	455.5732459	0.817024448	0.131953	6.191787	5.95E-10	4.16E-09
1907	ZNF274	518.1331452	0.816826554	0.115359	7.080748	1.43E-12	1.47E-11
3747	FGFR1OP	389.9033382	0.816724443	0.148296	5.507387	3.64E-08	1.91E-07
5340	SBNO1	460.5746584	0.816723592	0.177536	4.600329	4.22E-06	1.55E-05
6020	TIGD4	14.4956001	0.816681806	0.191918	4.255363	2.09E-05	6.80E-05
10865	BMX	28.45220037	0.816540451	0.349684	2.335083	0.019539	0.035249
3988	BEND7	541.6687312	0.81641031	0.152586	5.350493	8.77E-08	4.31E-07
3200	LACE1	80.96571358	0.816038605	0.138698	5.883544	4.02E-09	2.46E-08
3227	ZNF295	587.6515258	0.815359175	0.138949	5.868051	4.41E-09	2.68E-08
1919	DENND4C	1522.582462	0.815080758	0.115283	7.07026	1.55E-12	1.58E-11
2231	FDX1	658.6216031	0.814991694	0.121285	6.719655	1.82E-11	1.60E-10
14641	VN1R5	0.242825509	0.814956129	0.727727	1.119866	0.262771	0.351791
16834	SLC22A25	0.21227187	0.814951569	1.691809	0.481704	0.630016	0.733572
17357	SNTG1	0.189980568	0.814946515	2.320615	0.351177	0.725456	0.819246

17546	OR4A47	0.149277866	0.814942428	2.691973	0.302731	0.762095	0.851352
3571	FBXO48	38.96731642	0.81482512	0.14491	5.622959	1.88E-08	1.03E-07
7568	TEK	630.3190864	0.814368195	0.226863	3.589687	0.000331	0.000857
2989	AGPS	1826.060741	0.814167304	0.134707	6.043966	1.50E-09	9.86E-09
15751	ANKRD30A	0.822393735	0.814102603	1.031122	0.789531	0.429802	0.534858
2788	ZNF81	153.2079547	0.813844654	0.13121	6.202612	5.55E-10	3.90E-09
9001	FAM81A	79.489662	0.813385931	0.270056	3.011914	0.002596	0.005653
5627	TMPPE	42.78964973	0.813362459	0.182483	4.457193	8.30E-06	2.89E-05
1973	DDHD2	1112.689521	0.813326937	0.116203	6.999175	2.57E-12	2.56E-11
4495	CCNB1IP1	835.1607348	0.812933471	0.16126	5.041142	4.63E-07	2.02E-06
6857	LACTB2	967.3288447	0.812722062	0.209544	3.878518	0.000105	0.0003
7876	DNAH7	74.56589054	0.812630578	0.23475	3.461681	0.000537	0.001336
11460	CLEC18A	2167.93793	0.81217356	0.381022	2.131566	0.033043	0.056515
3614	EIF1B	789.9402335	0.810835285	0.144813	5.599207	2.15E-08	1.17E-07
1568	GHITM	5188.626756	0.81066574	0.10851	7.470919	7.96E-14	9.96E-13
3310	ZNF568	117.8005875	0.810539863	0.139491	5.810691	6.22E-09	3.68E-08
5309	FAM161B	101.5887917	0.810375062	0.175602	4.614836	3.93E-06	1.45E-05
3790	FBXO21	1416.105132	0.810241428	0.147869	5.47945	4.27E-08	2.21E-07
5722	DNAJB9	2789.264588	0.810198237	0.183738	4.409525	1.04E-05	3.55E-05
2591	YES1	1550.780216	0.809200655	0.126583	6.392637	1.63E-10	1.23E-09
7291	ZNF781	36.18312339	0.80872946	0.218454	3.702062	0.000214	0.000575
1624	SCRN3	358.6377277	0.808714411	0.109369	7.394348	1.42E-13	1.72E-12
3075	SS18L1	380.1959368	0.808627415	0.135225	5.979882	2.23E-09	1.42E-08
9696	COX7A1	335.7489322	0.808558889	0.294306	2.747339	0.006008	0.012146
13352	VWDE	17.19872608	0.808486481	0.528934	1.528521	0.126383	0.185533
1309	ZNF136	244.5523446	0.808415389	0.102992	7.849316	4.18E-15	6.26E-14
12406	TNMD	7.521298139	0.808341291	0.440751	1.834007	0.066653	0.105309
7552	DLL1	360.3017984	0.807999371	0.224785	3.594536	0.000325	0.000843
2507	ST7	937.9280926	0.807655712	0.125084	6.456896	1.07E-10	8.36E-10
6243	RDH10	1119.539694	0.80729283	0.194193	4.157171	3.22E-05	0.000101
5517	L3MBTL4	192.5333795	0.807216068	0.178863	4.513034	6.39E-06	2.27E-05
4313	TMEM161B	336.9619353	0.806911334	0.156717	5.148848	2.62E-07	1.19E-06
7556	TRIM6	356.658096	0.806889336	0.224562	3.593162	0.000327	0.000847
14780	LRRTM3	0.491042495	0.806722339	0.750858	1.074401	0.282643	0.374836
5604	LOC220729	184.2388013	0.806699308	0.18051	4.469008	7.86E-06	2.75E-05
5693	REST	314.6055778	0.806516548	0.182324	4.423544	9.71E-06	3.34E-05
1024	ACTR10	1068.432537	0.806315305	0.097178	8.297285	1.07E-16	2.04E-15
5317	TGFBR2	5636.626846	0.806004137	0.17472	4.613122	3.97E-06	1.46E-05
5335	CLMN	1025.215515	0.805596315	0.17497	4.604206	4.14E-06	1.52E-05
2143	UBR1	807.1111046	0.805427191	0.118226	6.812622	9.58E-12	8.77E-11
2794	ITM2B	35557.93359	0.805382388	0.129939	6.198157	5.71E-10	4.01E-09
15009	LMX1A	0.622187688	0.805377579	0.800545	1.006037	0.314398	0.410588
2240	DHFRL1	246.8906301	0.805285136	0.12005	6.707909	1.97E-11	1.73E-10
3258	ZBTB43	391.6948413	0.805080526	0.137696	5.846797	5.01E-09	3.01E-08
11046	CYP2C8	52.46890816	0.804718618	0.353805	2.27447	0.022938	0.040703
8364	ANKRD20A	13.48778665	0.804550478	0.246345	3.265948	0.001091	0.002557
3573	NT5DC1	867.1409983	0.804369068	0.143073	5.62209	1.89E-08	1.03E-07

1400	CHUK	603.6432234	0.804208495	0.104256	7.713801	1.22E-14	1.71E-13
3640	MANSC1	419.8767366	0.804009924	0.144146	5.577759	2.44E-08	1.31E-07
1728	TMED8	583.1422145	0.803873273	0.110593	7.268758	3.63E-13	4.12E-12
4237	PGRMC1	6942.689994	0.803869096	0.154961	5.187553	2.13E-07	9.86E-07
2857	LIN54	429.5804863	0.803239025	0.130853	6.138505	8.33E-10	5.72E-09
7350	FGF13	179.2117353	0.802823052	0.218125	3.680557	0.000233	0.000621
1786	C1orf27	825.9933412	0.802686752	0.111443	7.202643	5.91E-13	6.48E-12
5038	PXMP2	317.9428665	0.802661234	0.168829	4.754283	1.99E-06	7.75E-06
10645	LOC389791	2.826207071	0.802646735	0.332337	2.415158	0.015728	0.028961
7746	TMEM88	187.0249398	0.802170047	0.227849	3.520625	0.000431	0.001089
3734	SGK3	560.6472163	0.802101295	0.14543	5.515361	3.48E-08	1.83E-07
9877	FLJ35024	23.52829645	0.801871482	0.297536	2.69504	0.007038	0.013967
4575	PLEKHG3	775.480154	0.80184329	0.160442	4.997705	5.80E-07	2.49E-06
8742	PDZD2	1172.435446	0.801526947	0.257615	3.11134	0.001862	0.004176
3509	HSD17B12	2013.587684	0.801082087	0.141366	5.666729	1.46E-08	8.13E-08
7385	CCDC21	235.2508302	0.800880546	0.218712	3.661797	0.00025	0.000665
6056	OC1001286	37.19412776	0.800862835	0.18881	4.241635	2.22E-05	7.18E-05
9054	TCEAL5	7.157964121	0.800745875	0.267314	2.995524	0.00274	0.005931
8276	SOX9	1349.911041	0.800680303	0.242475	3.302118	0.00096	0.002273
5376	MTFR1	852.9062746	0.800653843	0.174883	4.578218	4.69E-06	1.71E-05
10796	RLN1	1.302188894	0.80043132	0.33865	2.363595	0.018099	0.032859
15728	SOHLH1	0.541747687	0.799805621	1.004896	0.795909	0.426085	0.531008
669	AP1G1	2799.875414	0.799774425	0.087798	9.109272	8.29E-20	2.43E-18
1681	ARHGEF12	7026.238759	0.799768778	0.10928	7.318499	2.51E-13	2.92E-12
7701	SRL	40.81263875	0.799622208	0.225865	3.540267	0.0004	0.001018
5983	NLN	405.6947453	0.799161396	0.186881	4.276306	1.90E-05	6.23E-05
454	ADH5	3233.862211	0.798623815	0.08156	9.791889	1.22E-22	5.27E-21
3909	ZNF429	193.5434486	0.798228395	0.147898	5.397142	6.77E-08	3.40E-07
3202	ZNF626	270.4499329	0.798191082	0.13568	5.882908	4.03E-09	2.47E-08
8126	GLCCI1	510.7210863	0.798043359	0.237498	3.36021	0.000779	0.001878
11397	ACVR1C	22.36539645	0.797752572	0.370953	2.150552	0.031512	0.054195
1536	GIN1	156.0397925	0.797720533	0.106225	7.509751	5.92E-14	7.56E-13
7046	SORD	603.0329454	0.797697485	0.209778	3.802575	0.000143	0.000398
11024	AP1M2	336.9965467	0.797652433	0.349574	2.281784	0.022502	0.040009
9013	ATP8B4	208.1374747	0.797563709	0.26517	3.007742	0.002632	0.005724
3411	ZNF674	78.15563472	0.797279892	0.138875	5.740973	9.41E-09	5.41E-08
5281	HIST4H4	20.94979308	0.797227691	0.172005	4.634903	3.57E-06	1.33E-05
4100	RNPC3	218.0671039	0.796969779	0.150905	5.281279	1.28E-07	6.13E-07
6550	LCOR	106.2384694	0.796880768	0.198919	4.006061	6.17E-05	0.000185
1650	THTPA	352.4431534	0.796712341	0.10826	7.359235	1.85E-13	2.20E-12
7278	C7orf46	82.59696922	0.796326969	0.214662	3.709674	0.000208	0.000559
6135	RGAG4	291.8301934	0.795784283	0.189199	4.206062	2.60E-05	8.30E-05
5742	FBXO10	228.1470167	0.795701265	0.180807	4.400822	1.08E-05	3.68E-05
6701	ZNF415	156.6375541	0.79559716	0.201904	3.940468	8.13E-05	0.000238
6034	TEF	907.6205614	0.795527847	0.187145	4.250856	2.13E-05	6.92E-05
6667	TBC1D24	339.2255159	0.795332225	0.201067	3.955568	7.64E-05	0.000224
1338	ATPAF1	1455.479056	0.795197401	0.101819	7.809935	5.72E-15	8.38E-14

1042	RAB21	1720.125433	0.795175488	0.096342	8.253644	1.54E-16	2.89E-15
999	RPRD1A	1102.491424	0.795024028	0.095216	8.349661	6.85E-17	1.34E-15
1582	ZNF552	133.2782257	0.794864216	0.106777	7.444146	9.76E-14	1.21E-12
2793	ZNF551	109.910101	0.794626676	0.1282	6.198332	5.71E-10	4.00E-09
8307	SLC16A13	189.7416199	0.793957819	0.241357	3.28956	0.001003	0.002368
6180	LOC92973	29.96692867	0.793501994	0.189575	4.185699	2.84E-05	9.02E-05
8055	SLC16A4	2601.242065	0.793190217	0.23389	3.3913	0.000696	0.001693
2054	TFAM	505.4395021	0.792951797	0.114941	6.8988	5.24E-12	5.00E-11
1699	FTSJD1	615.3090475	0.792890618	0.108696	7.294543	3.00E-13	3.46E-12
15634	VWC2	1.627032142	0.79260736	0.959196	0.826325	0.40862	0.512304
2108	KIDINS220	2311.787241	0.791733876	0.115707	6.842596	7.78E-12	7.23E-11
1646	ZNF417	162.5883883	0.79143061	0.107453	7.365368	1.77E-13	2.10E-12
9059	MYCN	15.74324603	0.791241078	0.264252	2.994268	0.002751	0.005952
6380	MLYCD	806.7575879	0.791220886	0.193632	4.08621	4.38E-05	0.000135
11260	LIMS3	30.82257015	0.790417084	0.360213	2.194305	0.028214	0.049113
12172	HAGHL	199.7761762	0.790087952	0.414686	1.905268	0.056745	0.091379
3725	HECTD1	3113.001793	0.790035904	0.143033	5.523441	3.32E-08	1.75E-07
929	DNAL1	486.4049137	0.789857512	0.093144	8.479998	2.25E-17	4.75E-16
2685	B3GALTL	453.0560227	0.789742759	0.125502	6.292668	3.12E-10	2.28E-09
8815	PNPLA7	271.2790173	0.789664047	0.256152	3.0828	0.002051	0.00456
4951	GCNT2	576.081259	0.789610286	0.164629	4.796306	1.62E-06	6.40E-06
2650	CRY1	484.0406382	0.7894914	0.124828	6.324648	2.54E-10	1.88E-09
2318	C11orf71	296.0128262	0.788511281	0.118742	6.640555	3.13E-11	2.64E-10
3028	ZNF570	68.48391593	0.787869287	0.131046	6.01217	1.83E-09	1.18E-08
3195	CD59	18501.53348	0.787866695	0.13384	5.88665	3.94E-09	2.42E-08
10010	DEGS2	41.20832248	0.787801043	0.297287	2.649969	0.00805	0.015763
2730	ZHX1	908.6939683	0.787678058	0.125907	6.256054	3.95E-10	2.84E-09
2247	PRKAR1A	7966.454004	0.787298747	0.117507	6.699994	2.08E-11	1.82E-10
14253	TAAR6	0.4222124	0.786655403	0.633475	1.241809	0.214307	0.294736
2962	IPP	206.6182182	0.786497862	0.129734	6.062386	1.34E-09	8.88E-09
6738	FAAH2	135.9891413	0.786461281	0.20033	3.92582	8.64E-05	0.000251
10997	LYG1	253.7176929	0.786232154	0.343466	2.289112	0.022073	0.039342
2133	FBXL4	334.2031968	0.786171501	0.115256	6.82111	9.03E-12	8.30E-11
3763	PHLPP2	387.5155526	0.786123512	0.142945	5.499493	3.81E-08	1.98E-07
4297	HCCS	621.975956	0.78604052	0.152375	5.158597	2.49E-07	1.13E-06
4504	ZNF468	489.4902681	0.785872906	0.156111	5.034061	4.80E-07	2.09E-06
2272	GUF1	608.3972898	0.785860695	0.11773	6.675101	2.47E-11	2.13E-10
4805	CEP78	186.5822185	0.785783375	0.160868	4.884653	1.04E-06	4.23E-06
1072	TRAF6	246.3513118	0.785752502	0.09576	8.20543	2.30E-16	4.20E-15
3082	SERAC1	167.1425764	0.785663066	0.13147	5.975983	2.29E-09	1.45E-08
8008	FAM110B	280.0846008	0.785534062	0.230605	3.406409	0.000658	0.001611
2275	MAGEE1	117.8968281	0.78502311	0.117629	6.673748	2.49E-11	2.15E-10
6098	TGFBR3	948.0988386	0.78340595	0.18556	4.221853	2.42E-05	7.79E-05
5533	MCF2L	986.107219	0.783156205	0.173861	4.504505	6.65E-06	2.36E-05
11408	GPNMB	12173.7679	0.783023043	0.364744	2.146774	0.031811	0.054661
4090	OSBPL8	2215.060176	0.782802213	0.148076	5.286503	1.25E-07	5.98E-07
5399	JMY	469.4210438	0.782749719	0.17143	4.566	4.97E-06	1.80E-05

5862	FOXO1	912.4962807	0.782700757	0.180341	4.340113	1.42E-05	4.76E-05
3042	MTHFD2L	167.5496314	0.782647085	0.130341	6.004625	1.92E-09	1.24E-08
2913	RICTOR	1021.13131	0.782376345	0.128227	6.101506	1.05E-09	7.07E-09
4388	UBE2W	841.256231	0.782269424	0.153098	5.109583	3.23E-07	1.44E-06
1838	GLUD1	6005.8871	0.781897077	0.109359	7.149848	8.69E-13	9.26E-12
3170	ANKRD6	440.8928301	0.78155931	0.132351	5.905193	3.52E-09	2.18E-08
904	RMND5A	1523.539043	0.78154336	0.091808	8.512842	1.70E-17	3.68E-16
9535	C22orf15	2.915701962	0.780743872	0.27844	2.803993	0.005047	0.010376
4047	LRRC40	509.6108854	0.780735839	0.14709	5.307867	1.11E-07	5.37E-07
7303	LOC730101	191.4536083	0.780361671	0.210983	3.6987	0.000217	0.000582
4390	AKD1	121.6880478	0.7798381	0.152651	5.108641	3.24E-07	1.45E-06
3139	PTPN4	210.5516604	0.779070732	0.131294	5.933782	2.96E-09	1.85E-08
6215	GBAS	1623.767934	0.778929403	0.186761	4.170721	3.04E-05	9.58E-05
11117	LRRC39	20.20093354	0.778883862	0.346788	2.245993	0.024704	0.043558
2640	HCFC2	548.9337451	0.77860311	0.122879	6.336341	2.35E-10	1.75E-09
3785	WDSUB1	321.99371	0.778583217	0.142049	5.481097	4.23E-08	2.19E-07
12700	OC1001302	50.37818979	0.777976917	0.448688	1.733894	0.082937	0.128004
5059	NELF	1166.433355	0.77783157	0.163997	4.742967	2.11E-06	8.16E-06
9653	TESC	155.9553943	0.777796992	0.281492	2.763126	0.005725	0.011625
7355	FAM108C1	218.0159801	0.777505414	0.211504	3.676086	0.000237	0.000631
5090	OGDH	6032.956341	0.777443315	0.16437	4.729827	2.25E-06	8.65E-06
9679	RNF157	449.0602441	0.777256863	0.282204	2.75424	0.005883	0.011914
3827	PNPLA4	588.0602749	0.77672531	0.142525	5.449733	5.04E-08	2.58E-07
1759	ATAD1	1112.310981	0.776714095	0.107414	7.230999	4.79E-13	5.34E-12
9060	FAM154B	12.94238999	0.77654618	0.259348	2.994225	0.002751	0.005953
2951	DENND4A	733.5601379	0.776294397	0.127897	6.069662	1.28E-09	8.51E-09
12121	HSPB7	153.9061453	0.776153248	0.403835	1.921958	0.054611	0.088312
5136	C6orf211	931.0326914	0.775871305	0.164704	4.710696	2.47E-06	9.42E-06
2160	ZNF134	233.6999589	0.775767846	0.11409	6.79964	1.05E-11	9.52E-11
4798	ZNF569	167.680603	0.775640687	0.158674	4.888268	1.02E-06	4.16E-06
11693	CNNM1	152.0988724	0.775400117	0.377699	2.052959	0.040077	0.06718
3818	AKT3	1266.179238	0.77531095	0.142041	5.458345	4.81E-08	2.47E-07
4528	CMTM8	84.91534336	0.774213664	0.154251	5.019167	5.19E-07	2.25E-06
1511	SEC24B	1286.960348	0.774200053	0.102502	7.553031	4.25E-14	5.52E-13
5009	MCC	664.3766613	0.774132734	0.162331	4.768839	1.85E-06	7.25E-06
2829	FNDC3A	2154.421916	0.773783786	0.125545	6.163386	7.12E-10	4.93E-09
7182	PODXL	5181.45662	0.773394958	0.206176	3.751135	0.000176	0.00048
6916	C1orf101	10.63956129	0.77334982	0.200495	3.857202	0.000115	0.000325
1992	TCTA	1337.866112	0.77297287	0.110851	6.973054	3.10E-12	3.05E-11
14060	IRX6	208.3178143	0.77293269	0.594847	1.29938	0.193814	0.270195
10584	CD22	129.3473422	0.772708685	0.316576	2.440828	0.014654	0.027138
7904	PDE2A	576.0007856	0.772244904	0.224072	3.446418	0.000568	0.001409
1573	ZNF721	507.1008276	0.771116633	0.103329	7.462744	8.47E-14	1.06E-12
12214	SIAH3	2.47957098	0.770821438	0.407336	1.892349	0.058444	0.093797
3047	THAP5	765.8102903	0.770338904	0.128323	6.003101	1.94E-09	1.25E-08
5025	C1orf115	1165.092291	0.770201622	0.161769	4.761117	1.93E-06	7.51E-06
6609	ARHGEF37	797.9607133	0.770119507	0.193509	3.979765	6.90E-05	0.000205

9113	ITGB8	3250.514845	0.769684745	0.258828	2.973736	0.002942	0.006328
4028	LARP4	1475.29241	0.769519772	0.144649	5.31992	1.04E-07	5.05E-07
7131	ZNF577	130.8558077	0.768548585	0.203656	3.773755	0.000161	0.000442
4900	PHACTR2	972.3912697	0.768172077	0.159162	4.826343	1.39E-06	5.56E-06
3482	C6orf57	102.7276882	0.768012954	0.135063	5.686324	1.30E-08	7.31E-08
1413	GRSF1	2114.999744	0.767712015	0.09992	7.68328	1.55E-14	2.15E-13
12772	ANKRD43	49.18882436	0.767653882	0.44824	1.712596	0.086787	0.13319
6350	HEY2	222.7999832	0.767528995	0.187127	4.101651	4.10E-05	0.000127
12787	SLC12A3	23.79796195	0.767525526	0.449382	1.707957	0.087644	0.134338
2986	TRIM23	501.9987363	0.767414494	0.126912	6.046833	1.48E-09	9.70E-09
4500	TCP11L2	209.714626	0.767409467	0.152282	5.039413	4.67E-07	2.03E-06
11595	FLJ46111	1.237263668	0.76736007	0.368595	2.081854	0.037356	0.063149
4128	ARFGEF1	1752.665196	0.767308075	0.145775	5.263631	1.41E-07	6.71E-07
1127	VPS36	1301.44631	0.767168717	0.094518	8.116681	4.79E-16	8.33E-15
1465	KLRAQ1	750.5390345	0.766965599	0.100636	7.621199	2.51E-14	3.36E-13
6711	CYB5A	5212.904623	0.766644008	0.194744	3.936681	8.26E-05	0.000241
4695	ZNF254	370.4584952	0.766637132	0.155446	4.931852	8.15E-07	3.40E-06
12844	LOC255167	24.40820006	0.766431451	0.452912	1.692229	0.090602	0.138267
12231	C21orf129	1.336071882	0.766188418	0.405941	1.887436	0.059102	0.094714
2199	ERLIN2	2001.341106	0.766102937	0.113395	6.75604	1.42E-11	1.26E-10
3068	FXC1	823.7012156	0.766027785	0.128022	5.983547	2.18E-09	1.39E-08
9170	NOXA1	129.9016146	0.76599155	0.259726	2.949234	0.003186	0.006809
2977	SBF2	1298.5435	0.76593777	0.126574	6.051293	1.44E-09	9.46E-09
8929	BMP4	220.6091101	0.765875128	0.251831	3.041223	0.002356	0.005172
3092	EPT1	1036.542238	0.765734552	0.128293	5.968621	2.39E-09	1.52E-08
11210	SLC19A3	209.6763864	0.765464777	0.345207	2.217408	0.026595	0.046502
2182	ZNF791	269.7214701	0.765216599	0.112956	6.774461	1.25E-11	1.12E-10
7101	STAG3L2	74.3402298	0.764987411	0.202228	3.782795	0.000155	0.000428
7954	SUGT1P1	6.272971231	0.764492627	0.223196	3.425205	0.000614	0.001514
3177	ZNF669	66.91998219	0.764058478	0.129569	5.896906	3.70E-09	2.29E-08
16264	FAM150A	0.529603076	0.763938392	1.200936	0.636119	0.524699	0.632239
5019	MEIS3P1	342.972577	0.76359341	0.160317	4.763022	1.91E-06	7.45E-06
8067	PARK2	252.6971795	0.763512529	0.225541	3.385256	0.000711	0.001728
8482	NET1	2337.766247	0.763303411	0.237513	3.21373	0.00131	0.003028
2016	PHKB	2053.930982	0.763148032	0.109947	6.941052	3.89E-12	3.78E-11
3013	RNF141	1210.428739	0.762992621	0.126665	6.023719	1.70E-09	1.11E-08
8222	BMP6	214.5159009	0.762550655	0.229452	3.323361	0.000889	0.00212
3067	ZBTB6	286.4609796	0.762522939	0.127413	5.984633	2.17E-09	1.39E-08
7171	DGKH	66.46970187	0.762387019	0.203001	3.755588	0.000173	0.000473
10910	CCDC144B	48.28389307	0.762331584	0.328881	2.317956	0.020452	0.036744
13383	TMPRSS4	16.38349851	0.762274456	0.502695	1.516374	0.129425	0.189558
2122	SDCCAG1	708.1965323	0.762196904	0.111612	6.828984	8.55E-12	7.90E-11
8924	SCRN1	2087.013918	0.762159392	0.250503	3.04252	0.002346	0.005153
5011	TSHZ1	1011.041307	0.761775754	0.159788	4.767407	1.87E-06	7.30E-06
1321	EPM2AIP1	851.3822853	0.761644368	0.097245	7.832205	4.79E-15	7.11E-14
10118	PLEKHH2	439.9444731	0.761509404	0.291821	2.609508	0.009067	0.017565
6239	LONRF1	552.4440919	0.76127432	0.183048	4.158883	3.20E-05	0.0001

760	PRPSAP2	543.4035497	0.761234513	0.085899	8.861964	7.86E-19	2.03E-17
6563	KIAA1529	105.0148555	0.761129768	0.190235	4.001004	6.31E-05	0.000188
10483	DGKB	10.07688723	0.760854592	0.306977	2.478543	0.013192	0.024666
3729	NDUFAF4	269.9911174	0.760772309	0.137823	5.519911	3.39E-08	1.78E-07
2141	SFRS5	3424.835897	0.760595249	0.111634	6.813314	9.54E-12	8.73E-11
11847	RASSF10	8.847951257	0.760432376	0.378889	2.007004	0.044749	0.074038
4279	HEATR5A	498.5987451	0.760371437	0.147144	5.167537	2.37E-07	1.09E-06
9907	REEP1	99.21909681	0.759975576	0.28296	2.685802	0.007236	0.014316
10065	ACSM5	673.2132472	0.759495123	0.28863	2.631376	0.008504	0.016561
2659	TTC21B	555.2764585	0.759467476	0.120172	6.319815	2.62E-10	1.93E-09
1818	FECH	660.5493855	0.759285646	0.105861	7.172482	7.37E-13	7.94E-12
4370	BBS12	293.7612062	0.759125589	0.148298	5.118913	3.07E-07	1.38E-06
3432	LOC72802	16.96367087	0.758871716	0.132631	5.721697	1.05E-08	6.02E-08
8283	CALCRL	2198.014795	0.758696205	0.229905	3.300046	0.000967	0.002288
12548	VNN1	863.4807625	0.758404443	0.423419	1.791143	0.07327	0.114454
2180	ZNF330	651.6999813	0.758219336	0.111858	6.778383	1.22E-11	1.09E-10
9380	DOK6	184.7746007	0.758003764	0.26441	2.86677	0.004147	0.008665
10724	BAIAP2L2	503.0256432	0.757938162	0.317397	2.387981	0.016941	0.030964
7625	MFHAS1	275.3857074	0.757903543	0.212349	3.569143	0.000358	0.000921
9376	GAS2L3	313.9396382	0.75790179	0.264052	2.870271	0.004101	0.008574
14194	CRISP3	0.380533637	0.757648843	0.601578	1.259437	0.207873	0.287059
8213	CTTNBP2	715.2747695	0.757606557	0.22778	3.326051	0.000881	0.002102
16139	HIST1H2A	0.156534844	0.757246838	1.131691	0.669129	0.503413	0.611401
16509	NXF2B	0.192271236	0.757245994	1.329661	0.569503	0.569015	0.675587
17124	SEMG2	0.197332878	0.757242731	1.870796	0.40477	0.685646	0.784826
17118	OR1B1	0.185881036	0.757242697	1.861394	0.406815	0.684144	0.783335
17620	KCNU1	0.196193451	0.757236329	2.6264	0.288317	0.773104	0.860023
17733	LCE1A	0.122864445	0.757232346	2.91407	0.259854	0.794977	0.87872
1293	EIF5	3779.84178	0.757172491	0.096183	7.872203	3.48E-15	5.28E-14
3098	MED9	481.5198012	0.756278111	0.126787	5.964932	2.45E-09	1.55E-08
9252	CHN2	179.2087556	0.756226893	0.259216	2.917367	0.00353	0.007479
3096	UBXN2B	932.062639	0.755617416	0.12665	5.966166	2.43E-09	1.54E-08
1385	CLPX	637.5241195	0.755546973	0.097682	7.734727	1.04E-14	1.47E-13
1687	LOC40002	234.2649046	0.755529478	0.103312	7.313105	2.61E-13	3.03E-12
5249	SLC25A15	240.8354111	0.75547715	0.162398	4.651999	3.29E-06	1.23E-05
1089	C18orf55	401.8418446	0.755417499	0.092406	8.175004	2.96E-16	5.33E-15
10147	TM7SF2	493.2350453	0.755061381	0.290433	2.599781	0.009328	0.01802
7135	NUP62CL	53.42558837	0.75505455	0.200197	3.771565	0.000162	0.000446
2633	NIPSNAP3	583.3258051	0.755014904	0.119034	6.342863	2.26E-10	1.68E-09
4574	HSP90AA1	16425.43954	0.754556486	0.15097	4.998072	5.79E-07	2.48E-06
8562	FAM107A	539.2526228	0.754541276	0.236851	3.185716	0.001444	0.003305
4884	ZFP106	1831.827501	0.754506425	0.15597	4.837504	1.31E-06	5.28E-06
3869	MTMR10	1590.878254	0.754139049	0.139092	5.421883	5.90E-08	2.99E-07
10741	SLC6A16	41.86101848	0.754025363	0.316353	2.383495	0.017149	0.031295
8984	CAND2	68.54219011	0.753900507	0.249635	3.020011	0.002528	0.005515
4917	KIAA0284	838.5816914	0.753548295	0.156394	4.818277	1.45E-06	5.77E-06
7388	C1orf51	197.1891925	0.753241419	0.205743	3.661078	0.000251	0.000666

6808	HSPH1	1771.85367	0.753150616	0.193288	3.896518	9.76E-05	0.000281
6629	PRKAR2A	245.6577023	0.753038076	0.189572	3.972309	7.12E-05	0.00021
11794	PRRG3	2.952767589	0.752963703	0.372039	2.023882	0.042982	0.071434
1408	CTR9	1260.479801	0.752638084	0.097871	7.690129	1.47E-14	2.05E-13
5721	COQ9	1877.29272	0.752489165	0.170639	4.409824	1.03E-05	3.54E-05
989	MTMR12	1207.546174	0.752321005	0.089972	8.361681	6.18E-17	1.23E-15
446	FBXO9	801.0962279	0.752154132	0.076569	9.823208	8.95E-23	3.93E-21
2936	MRPS35	1701.577714	0.751234686	0.123559	6.07997	1.20E-09	8.02E-09
2174	NAPG	700.2521156	0.750926073	0.110622	6.788246	1.14E-11	1.02E-10
1811	EIF4EBP2	5576.199995	0.750545784	0.104567	7.177673	7.09E-13	7.67E-12
532	TTC19	966.7661998	0.750436048	0.07884	9.518483	1.76E-21	6.48E-20
5725	RALGPS2	394.096993	0.750194089	0.170163	4.408688	1.04E-05	3.56E-05
2492	ISCA1	1180.016793	0.750011537	0.115841	6.474507	9.51E-11	7.48E-10
14165	FLJ16779	42.02601253	0.749930533	0.592014	1.266745	0.205247	0.284047
3620	C6orf72	965.1537035	0.74988681	0.134085	5.592637	2.24E-08	1.21E-07
3140	PKP4	2714.748659	0.749834761	0.126398	5.932339	2.99E-09	1.86E-08
2592	RCBTB2	1110.475094	0.749760275	0.117349	6.389171	1.67E-10	1.26E-09
10183	PKIB	117.1850532	0.749213773	0.289549	2.587524	0.009667	0.018607
4509	YAF2	523.9065486	0.749061971	0.148944	5.029158	4.93E-07	2.14E-06
1501	BBS7	328.1892672	0.748998422	0.098989	7.566514	3.83E-14	5.01E-13
3807	DENND5B	684.1511519	0.748622215	0.136901	5.468345	4.54E-08	2.34E-07
9133	TMEM200A	1106.625451	0.748476244	0.252434	2.965039	0.003026	0.006495
3275	PIAS2	319.6526474	0.748136728	0.128271	5.832485	5.46E-09	3.27E-08
2612	FAM13B	836.4566041	0.747882163	0.117484	6.365808	1.94E-10	1.46E-09
3152	FANCM	100.2850374	0.747838712	0.126372	5.917761	3.26E-09	2.03E-08
2101	PKN2	1301.246487	0.747469475	0.109138	6.848859	7.44E-12	6.94E-11
7185	C1orf229	12.40960405	0.747115801	0.199225	3.750117	0.000177	0.000482
2721	STX7	2192.241996	0.746909173	0.119235	6.264191	3.75E-10	2.70E-09
3739	B3GNT2	1258.97778	0.746813891	0.135465	5.512981	3.53E-08	1.85E-07
6557	MIPOL1	65.37530781	0.746477671	0.186482	4.002941	6.26E-05	0.000187
2910	EIF4E	526.3699749	0.746335047	0.122283	6.103335	1.04E-09	7.00E-09
8033	PTPLB	190.0685505	0.746208547	0.219563	3.398611	0.000677	0.001653
1258	ZNF304	334.5264753	0.746138312	0.094219	7.919175	2.39E-15	3.73E-14
8168	FAAH	451.0091178	0.745941663	0.222911	3.346362	0.000819	0.001965
3435	MTMR6	1195.875519	0.745908068	0.130386	5.720748	1.06E-08	6.05E-08
2619	SNX14	1201.457253	0.745823245	0.117313	6.357542	2.05E-10	1.53E-09
11776	SLC35F4	2.175727624	0.745292364	0.367345	2.028863	0.042472	0.070693
2215	PDPK1	1456.798925	0.745095787	0.110527	6.741308	1.57E-11	1.39E-10
11206	RGS9	77.55285642	0.744871329	0.335533	2.219961	0.026421	0.046215
6122	MTUS1	1752.972909	0.744869378	0.176898	4.210718	2.55E-05	8.15E-05
2696	NARS	3762.657955	0.744747114	0.118503	6.28463	3.29E-10	2.39E-09
3553	COL4A3BP	1592.854342	0.744179111	0.132116	5.632778	1.77E-08	9.78E-08
6825	STRN	309.1105771	0.744173256	0.191299	3.890097	0.0001	0.000288
10859	ATOH8	307.5853926	0.743883101	0.318397	2.336335	0.019474	0.035151
5762	DENND2C	44.54285485	0.743740792	0.169355	4.391602	1.13E-05	3.83E-05
3622	AFF4	3858.19107	0.743597511	0.132984	5.591642	2.25E-08	1.22E-07
8172	ZDHHC2	820.6553876	0.743548806	0.222273	3.345203	0.000822	0.001972

12629	HHLA2	1450.773917	0.743530668	0.421375	1.764535	0.077642	0.120505
11596	PCDHA10	94.55978458	0.743483382	0.357164	2.081631	0.037376	0.063178
3694	ANKRD26	202.0706119	0.743320466	0.134239	5.537287	3.07E-08	1.63E-07
9673	WDR16	3.105321526	0.743159385	0.269693	2.755573	0.005859	0.011872
3209	VPS13B	951.6558795	0.743029874	0.12637	5.879812	4.11E-09	2.51E-08
2419	SUZ12	889.9417952	0.742663338	0.113542	6.540873	6.12E-11	4.96E-10
3110	WASL	2495.833968	0.742644228	0.124728	5.954104	2.62E-09	1.65E-08
3743	DYNC2LI1	567.2353329	0.742248534	0.134692	5.51071	3.57E-08	1.87E-07
4704	C1D	286.7232185	0.741940437	0.150528	4.928918	8.27E-07	3.44E-06
8383	GRHL1	77.60301997	0.741937431	0.227736	3.257887	0.001122	0.002624
10773	RASD1	739.8343092	0.74171272	0.3124	2.374238	0.017585	0.031998
7708	UBE2Q2P1	23.43186634	0.74118018	0.209575	3.53659	0.000405	0.001031
1987	TRMT2B	356.4908373	0.74103274	0.106144	6.981384	2.92E-12	2.88E-11
10880	DPF3	120.5210927	0.740845615	0.318061	2.329256	0.019845	0.035753
3901	BRCC3	314.7474238	0.740805353	0.137093	5.403651	6.53E-08	3.28E-07
10780	UBD	2563.774128	0.740761008	0.312601	2.369667	0.017804	0.032373
6028	ZCWPW2	13.99126204	0.740368864	0.174101	4.252516	2.11E-05	6.87E-05
2945	CDC42SE2	2962.669441	0.740240963	0.121882	6.073423	1.25E-09	8.33E-09
3636	SGMS1	818.020792	0.740119817	0.132615	5.580969	2.39E-08	1.29E-07
8942	KLHL31	34.72827911	0.740074939	0.243857	3.034872	0.002406	0.005274
5276	KIAA1804	617.3374407	0.739425189	0.159392	4.639022	3.50E-06	1.30E-05
1865	FBXO8	557.5869735	0.739365717	0.103805	7.122633	1.06E-12	1.11E-11
3842	USP45	248.0105363	0.739147533	0.135889	5.439347	5.35E-08	2.73E-07
4068	AGPAT3	3240.684072	0.739116631	0.13953	5.297182	1.18E-07	5.67E-07
3623	ZNF594	149.7640842	0.739003491	0.132177	5.591033	2.26E-08	1.22E-07
5941	GALNT11	3161.455845	0.738792736	0.17191	4.297555	1.73E-05	5.70E-05
8052	C2orf15	34.04690906	0.738453828	0.21773	3.391598	0.000695	0.001692
6708	C15orf37	70.15974299	0.73833376	0.187489	3.938019	8.22E-05	0.00024
4016	HINT3	897.1542744	0.738240211	0.138525	5.329308	9.86E-08	4.81E-07
3986	KIF27	75.84505319	0.738091915	0.13793	5.351215	8.74E-08	4.30E-07
4862	SUCLG2	1592.99243	0.737921412	0.151961	4.855992	1.20E-06	4.83E-06
2852	RAB18	2046.637253	0.737305953	0.12004	6.142172	8.14E-10	5.59E-09
3615	TAF1B	305.6844817	0.737204848	0.131722	5.596652	2.19E-08	1.18E-07
2458	ANKS1A	873.2647164	0.736352958	0.11315	6.507756	7.63E-11	6.08E-10
10969	NLRP11	16.99460343	0.736140293	0.320474	2.297033	0.021617	0.038625
3911	ZNF396	51.84483624	0.735814247	0.136364	5.395961	6.82E-08	3.42E-07
5266	FAM199X	694.011322	0.735398013	0.158354	4.644007	3.42E-06	1.27E-05
8615	CCDC148	48.34161479	0.735363437	0.232055	3.168923	0.00153	0.003481
4284	MFSD6	899.2655445	0.735313396	0.142389	5.164102	2.42E-07	1.11E-06
2191	NAA15	1036.823335	0.735109508	0.108599	6.769052	1.30E-11	1.16E-10
5481	KIAA1147	1731.374822	0.734850403	0.162228	4.529733	5.91E-06	2.11E-05
11748	FAM83G	60.25004327	0.734569655	0.360477	2.037773	0.041573	0.069362
9595	C16orf3	4.086581094	0.734248581	0.264035	2.780877	0.005421	0.011075
1856	MRPL35	908.7039	0.734180504	0.102967	7.130255	1.00E-12	1.06E-11
4058	GTF3C4	319.7312544	0.734164682	0.138475	5.301773	1.15E-07	5.54E-07
7047	SDC2	4772.180049	0.734028801	0.193066	3.801954	0.000144	0.000399
8378	FAM171B	226.3151979	0.733889821	0.225194	3.25893	0.001118	0.002616

7203	APOO	311.6058818	0.733811923	0.196055	3.742886	0.000182	0.000495
2204	MTMR9	454.9005772	0.733723368	0.108679	6.751318	1.47E-11	1.30E-10
8580	ZNF835	16.92386223	0.73366242	0.230706	3.180079	0.001472	0.003364
11620	PHYHD1	175.2275659	0.73358015	0.353988	2.07233	0.038235	0.064496
6972	MAST4	642.450997	0.733577631	0.191152	3.837667	0.000124	0.000349
2720	MTMR15	543.290255	0.733446458	0.117084	6.264261	3.75E-10	2.70E-09
5769	TTBK2	157.8337252	0.733375218	0.167178	4.386795	1.15E-05	3.91E-05
4208	KIAA1107	119.6196142	0.732450257	0.140752	5.203841	1.95E-07	9.09E-07
6007	ZNF763	80.81107327	0.732320882	0.171779	4.26315	2.02E-05	6.58E-05
6244	CLDN23	150.9988024	0.73139232	0.175947	4.156893	3.23E-05	0.000101
4439	APIP	440.8036949	0.731195716	0.143949	5.079538	3.78E-07	1.67E-06
6348	LMTK2	500.2541568	0.730873436	0.178071	4.104399	4.05E-05	0.000125
5898	MOSC1	226.2207134	0.730686791	0.169043	4.322479	1.54E-05	5.13E-05
13382	CCDC54	0.679084265	0.730222409	0.48144	1.516745	0.129331	0.189435
4873	GRB10	3359.992639	0.730098631	0.150612	4.847536	1.25E-06	5.03E-06
15506	NEUROD1	0.403796951	0.730058881	0.850633	0.858254	0.390752	0.493947
3268	PIKFYVE	956.9623818	0.729988028	0.125073	5.836489	5.33E-09	3.20E-08
13846	BIRC7	170.0694837	0.729652163	0.532976	1.369015	0.170995	0.24205
4104	C1orf203	72.68021712	0.729495854	0.138174	5.279551	1.30E-07	6.19E-07
2420	TBC1D15	1382.312275	0.729459561	0.111524	6.540843	6.12E-11	4.96E-10
2666	PIGH	422.9511961	0.729063343	0.115435	6.315778	2.69E-10	1.98E-09
11826	C10orf107	29.34679333	0.728956617	0.362176	2.012715	0.044145	0.073168
12031	CADPS	4.217386852	0.72887445	0.373383	1.952081	0.050929	0.082973
5713	HDX	54.11311182	0.728843974	0.165122	4.413967	1.01E-05	3.48E-05
3868	STK3	526.4453864	0.728578094	0.134367	5.42229	5.88E-08	2.98E-07
6698	SLC22A23	593.5574825	0.728442009	0.184816	3.941441	8.10E-05	0.000237
4758	HCP5	1414.451584	0.728292091	0.148457	4.905739	9.31E-07	3.83E-06
2966	RBBP5	454.2875204	0.728249791	0.120223	6.057506	1.38E-09	9.14E-09
5342	C5orf36	84.09376191	0.727908189	0.158247	4.599815	4.23E-06	1.55E-05
6893	SLC25A42	696.2069901	0.727442646	0.188092	3.867482	0.00011	0.000313
2357	CDC42BPA	2194.470339	0.727333183	0.110181	6.601258	4.08E-11	3.39E-10
9346	SYNGR1	338.2859064	0.727145156	0.252543	2.879291	0.003986	0.008359
2937	LIG4	329.5007909	0.726709123	0.119532	6.079645	1.20E-09	8.04E-09
6093	PECR	623.7711048	0.726472241	0.171969	4.224448	2.40E-05	7.71E-05
15593	MYH4	0.546351187	0.726244896	0.867879	0.836804	0.402703	0.506213
5472	DAB2IP	2043.644998	0.726203936	0.160182	4.53361	5.80E-06	2.08E-05
3941	G2E3	331.6088227	0.726182575	0.135003	5.379012	7.49E-08	3.73E-07
9565	ZNF366	95.01273094	0.72608872	0.259746	2.795375	0.005184	0.010623
3261	PEX1	564.1839535	0.725360066	0.12418	5.841219	5.18E-09	3.11E-08
5344	CCDC126	195.9546368	0.724978413	0.157696	4.597315	4.28E-06	1.57E-05
2960	C1orf74	75.37236121	0.724801602	0.119552	6.062666	1.34E-09	8.87E-09
965	BTBD7	1014.888787	0.724764438	0.086095	8.418164	3.82E-17	7.77E-16
5086	ZNF462	438.3718016	0.724741261	0.153211	4.730363	2.24E-06	8.64E-06
2024	ZNF510	270.9371985	0.724593407	0.104477	6.935413	4.05E-12	3.92E-11
6341	ZNF665	25.60390873	0.724489303	0.176359	4.108028	3.99E-05	0.000123
3607	ZBTB33	700.8579099	0.724447231	0.129281	5.603663	2.10E-08	1.14E-07
6674	GDPD1	32.64722776	0.723870274	0.18315	3.952327	7.74E-05	0.000227

1911	CHMP5	1861.688044	0.723486889	0.102249	7.075768	1.49E-12	1.52E-11
10429	KCNJ16	3447.339307	0.722936192	0.289158	2.500144	0.012414	0.023332
10703	DHDPSL	558.0303398	0.722439574	0.301504	2.396123	0.01657	0.030345
15012	ROPN1B	0.432764318	0.722430628	0.718633	1.005284	0.31476	0.410997
13742	MAGEE2	0.718889985	0.722178934	0.514335	1.404102	0.160289	0.228629
4563	MMACHC	136.9768016	0.721920072	0.144292	5.003172	5.64E-07	2.42E-06
6930	MAML2	746.5851103	0.721864956	0.187425	3.851485	0.000117	0.000332
6054	ACO1	2292.87007	0.721759715	0.170156	4.241748	2.22E-05	7.18E-05
2317	BAHD1	715.2839918	0.721746783	0.10868	6.641056	3.11E-11	2.63E-10
3648	MMGT1	1080.488946	0.721731802	0.129511	5.572746	2.51E-08	1.35E-07
6489	HSD17B8	330.7238478	0.721701738	0.17895	4.032975	5.51E-05	0.000166
2884	USO1	2735.717707	0.721229289	0.117787	6.123142	9.17E-10	6.24E-09
7935	MYCT1	555.5471029	0.721206656	0.210023	3.433937	0.000595	0.001469
2457	PICALM	7250.789436	0.720805419	0.110753	6.508224	7.60E-11	6.07E-10
9670	TM4SF18	1095.485735	0.720683204	0.261439	2.756603	0.005841	0.011839
13850	ISM2	19.3438565	0.720637616	0.526612	1.368442	0.171174	0.242251
3302	LONP2	1484.369139	0.719974791	0.123843	5.81361	6.11E-09	3.63E-08
8550	HYAL1	919.0806651	0.719899209	0.225705	3.18956	0.001425	0.003267
10818	LOC154822	13.68987428	0.719865195	0.305717	2.354676	0.018539	0.03359
1132	CDKN2AIP	432.7495415	0.718942202	0.088624	8.112263	4.97E-16	8.60E-15
11383	SLC10A4	2.616459397	0.718923435	0.333112	2.1582	0.030912	0.05323
7227	FAM84A	362.5389889	0.718584616	0.192339	3.736041	0.000187	0.000507
1642	SLC39A9	2177.953154	0.718536154	0.097476	7.37144	1.69E-13	2.01E-12
8441	FAM65B	302.9991389	0.71836463	0.222357	3.230684	0.001235	0.002868
6171	CRIM1	6475.532466	0.71832427	0.171438	4.189995	2.79E-05	8.86E-05
3273	FH	1500.283431	0.718224457	0.123121	5.833483	5.43E-09	3.25E-08
3899	POLK	778.2206022	0.718148649	0.132894	5.403918	6.52E-08	3.28E-07
3267	BTBD1	1591.907064	0.718120856	0.12304	5.836482	5.33E-09	3.20E-08
3137	G3BP2	3307.531279	0.718053978	0.120983	5.935156	2.94E-09	1.83E-08
2991	SRP9	3761.247289	0.717661191	0.118755	6.043186	1.51E-09	9.90E-09
5728	CAP2	489.1256494	0.717660869	0.162806	4.408072	1.04E-05	3.57E-05
1528	ZFAND6	1362.132003	0.71744555	0.095429	7.518128	5.56E-14	7.13E-13
2950	PDCD6IP	3000.309151	0.717158151	0.118141	6.070342	1.28E-09	8.48E-09
6904	HMGN5	138.910739	0.717148668	0.185675	3.862395	0.000112	0.000319
9168	GPSM2	356.6621205	0.716603881	0.242955	2.949535	0.003183	0.006804
7535	OSBP2	175.8743887	0.716521571	0.19892	3.602057	0.000316	0.000821
7573	EFNB2	1817.770869	0.716501912	0.199688	3.588099	0.000333	0.000862
3373	KIAA0528	1094.693812	0.716326572	0.124266	5.764445	8.19E-09	4.76E-08
4529	GLRX5	738.5272628	0.715875689	0.142628	5.019183	5.19E-07	2.25E-06
1457	C9orf156	227.0386858	0.715627776	0.09381	7.628511	2.37E-14	3.19E-13
3254	ATRX	1867.751644	0.715535618	0.122324	5.849493	4.93E-09	2.97E-08
893	CUL3	1598.211452	0.71543783	0.083764	8.541148	1.33E-17	2.92E-16
3863	TRNT1	245.3941105	0.715354386	0.131847	5.425651	5.77E-08	2.93E-07
4492	FBXO33	345.7480442	0.715163367	0.141784	5.044029	4.56E-07	1.99E-06
7842	XYLB	146.7475585	0.714832056	0.205519	3.478173	0.000505	0.001262
11402	C10orf131	1.798583136	0.714594049	0.332569	2.14871	0.031657	0.05442
2860	PDSS2	489.7390476	0.714529971	0.116435	6.136707	8.42E-10	5.78E-09

2777	NMNAT1	249.7908314	0.714489211	0.114958	6.215244	5.12E-10	3.62E-09
3965	METTL9	4223.22675	0.714340368	0.13316	5.364533	8.12E-08	4.01E-07
3581	ZMPSTE24	2070.871674	0.714259065	0.127172	5.616466	1.95E-08	1.07E-07
2214	RAB14	2694.145916	0.714001883	0.10591	6.741586	1.57E-11	1.39E-10
3803	ZNF654	449.7609908	0.713467918	0.130369	5.472701	4.43E-08	2.28E-07
5004	TMEM14A	585.5261419	0.713447079	0.149503	4.772128	1.82E-06	7.14E-06
11443	SLITRK4	270.9055638	0.713095988	0.333817	2.13619	0.032664	0.055951
3547	IER3IP1	1338.501871	0.712919129	0.126465	5.637303	1.73E-08	9.55E-08
4030	FDFT1	1324.370374	0.712815916	0.134032	5.318252	1.05E-07	5.10E-07
5441	ATP1B1	21975.35448	0.712794928	0.156789	4.546197	5.46E-06	1.97E-05
3251	FMR1	1398.828744	0.712789697	0.121793	5.852456	4.84E-09	2.92E-08
2900	ZADH2	732.8176402	0.712498141	0.116562	6.112606	9.80E-10	6.62E-09
5157	TMED7	4432.596969	0.712442312	0.151661	4.697601	2.63E-06	1.00E-05
5147	RIMKLB	781.7400669	0.712296393	0.151412	4.704371	2.55E-06	9.70E-06
12219	GREB1	117.7753806	0.712072484	0.376514	1.891223	0.058595	0.093999
10583	HOXD1	53.51456837	0.711966853	0.291595	2.441628	0.014621	0.02708
3363	SYNJ1	492.6782962	0.711873164	0.123365	5.770455	7.91E-09	4.61E-08
4207	LRRC58	1495.599328	0.711547201	0.136704	5.20502	1.94E-07	9.04E-07
13124	GSTA2	1881.310977	0.711209878	0.443634	1.603144	0.108903	0.162649
3864	SMARCA1	1181.054982	0.71081659	0.131013	5.425525	5.78E-08	2.93E-07
7763	DENND1B	78.91994935	0.710800015	0.202357	3.512596	0.000444	0.00112
9443	SERTAD4	59.74426476	0.709986257	0.249938	2.840647	0.004502	0.009345
10085	CRNA0023	1.875462775	0.709976188	0.270616	2.623558	0.008702	0.016914
1425	SCO1	429.8758326	0.709945992	0.092504	7.674773	1.66E-14	2.28E-13
10580	NRIP3	74.84992547	0.709907062	0.290705	2.44202	0.014605	0.027059
5181	ABLIM1	3164.905928	0.709744727	0.151517	4.684272	2.81E-06	1.06E-05
13750	CLDN19	37.00544134	0.709606929	0.505981	1.402438	0.160784	0.229217
10810	PLEKHB1	174.6697883	0.709495697	0.300804	2.358665	0.018341	0.033256
3117	SFXN1	1457.961317	0.709447668	0.119231	5.950192	2.68E-09	1.68E-08
5541	PHLDB2	2204.598301	0.709246878	0.157527	4.502396	6.72E-06	2.38E-05
944	IKBKAP	873.9271081	0.70923715	0.083869	8.456463	2.76E-17	5.72E-16
1662	PNRC2	2076.724192	0.709211484	0.096579	7.343296	2.08E-13	2.46E-12
2393	CXorf23	262.3566657	0.709097127	0.108026	6.564155	5.23E-11	4.29E-10
15436	DHX40P1	0.878156247	0.708925882	0.808054	0.877325	0.38031	0.482927
2094	MRS2	721.6382986	0.708582127	0.103404	6.852586	7.25E-12	6.79E-11
15716	APOF	0.368697429	0.708553644	0.886171	0.799568	0.423961	0.528765
2940	PECI	1488.235967	0.708509382	0.116593	6.076756	1.23E-09	8.18E-09
3882	CADPS2	801.675534	0.708502861	0.130881	5.413323	6.19E-08	3.12E-07
3833	FBXO36	140.0831549	0.708300814	0.130118	5.443541	5.22E-08	2.67E-07
12310	SHISA2	50.80334253	0.708218224	0.380504	1.861262	0.062707	0.099848
5471	PCK2	933.6518834	0.708122972	0.156188	4.533773	5.79E-06	2.08E-05
2565	NEK1	415.8766761	0.708021594	0.11041	6.41264	1.43E-10	1.09E-09
4920	MBLAC2	234.5010014	0.708012251	0.146976	4.817197	1.46E-06	5.80E-06
6724	SCLY	407.7962231	0.707992167	0.18006	3.931984	8.42E-05	0.000246
2071	ITSN2	1521.809357	0.707965807	0.102912	6.879361	6.01E-12	5.69E-11
6370	CYP51A1	1339.814254	0.70785106	0.172945	4.092923	4.26E-05	0.000131
4191	PDK2	1630.755846	0.707822596	0.135729	5.214961	1.84E-07	8.60E-07

5329	43898	287.1778451	0.707718146	0.153557	4.608822	4.05E-06	1.49E-05
3314	SLC41A1	1199.572541	0.707455939	0.121823	5.807237	6.35E-09	3.76E-08
11684	DKK1	15.58427759	0.707185201	0.344108	2.055127	0.039867	0.066879
2602	STAM2	1026.574317	0.707062032	0.110822	6.380148	1.77E-10	1.33E-09
10283	C5	159.0643992	0.707002616	0.277042	2.551967	0.010712	0.020418
1854	C18orf25	704.6589759	0.706953025	0.099109	7.133094	9.81E-13	1.04E-11
5083	GK5	379.6916986	0.706910219	0.149415	4.731177	2.23E-06	8.61E-06
9238	SLC2A9	360.9428595	0.706845713	0.24177	2.923627	0.00346	0.007341
3394	BMI1	1523.702575	0.706813611	0.122925	5.749963	8.93E-09	5.15E-08
2976	FAM98B	293.1781736	0.706750237	0.11679	6.051481	1.44E-09	9.45E-09
11856	GABRR1	5.028255154	0.706535328	0.352514	2.004278	0.04504	0.074463
2627	CYB5D1	728.1259567	0.706489974	0.111206	6.352974	2.11E-10	1.58E-09
8958	FN3K	852.1190607	0.706258113	0.233257	3.027808	0.002463	0.00539
2462	RPP14	426.9587662	0.706226692	0.108566	6.50502	7.77E-11	6.18E-10
3572	KIAA1586	181.069221	0.706166314	0.1256	5.622352	1.88E-08	1.03E-07
5054	AADAT	88.45218015	0.706132428	0.148821	4.744828	2.09E-06	8.09E-06
9857	MYOM3	773.4132384	0.705580625	0.261214	2.701158	0.00691	0.01374
4122	ZNF292	1354.149422	0.705389771	0.133859	5.269654	1.37E-07	6.50E-07
3467	RBL2	2384.954089	0.705348707	0.123798	5.697556	1.22E-08	6.87E-08
3732	STRN3	789.041148	0.705078732	0.127788	5.517582	3.44E-08	1.80E-07
4256	ZFYVE1	739.6225337	0.704615988	0.136035	5.179671	2.22E-07	1.02E-06
7065	GNPNAT1	770.3218749	0.704342443	0.185537	3.796238	0.000147	0.000408
3502	TM7SF3	3277.185211	0.703647894	0.124058	5.671934	1.41E-08	7.90E-08
10720	DPY19L2	272.8961147	0.703403171	0.294378	2.389456	0.016873	0.030851
9886	ZNF878	5.154406049	0.703286817	0.261308	2.691412	0.007115	0.014107
1810	KIAA1468	646.8752399	0.703252054	0.097968	7.178362	7.06E-13	7.64E-12
10468	ANKRD31	2.569257417	0.703121303	0.282794	2.486342	0.012906	0.024167
8010	GRB7	296.1806128	0.702694719	0.206301	3.406158	0.000659	0.001612
4096	BDNFOS	56.6443122	0.702595933	0.132982	5.283411	1.27E-07	6.07E-07
7514	LMBRD2	240.6711669	0.702492626	0.194539	3.611063	0.000305	0.000795
5763	DOCK8	2201.52464	0.702281082	0.160003	4.38918	1.14E-05	3.87E-05
3343	EIF1AX	2048.889372	0.701876317	0.121195	5.791315	6.98E-09	4.09E-08
1918	AASDH	231.1499212	0.701832047	0.099261	7.070592	1.54E-12	1.58E-11
1576	PIK3C3	613.4595145	0.701746508	0.094076	7.459349	8.70E-14	1.08E-12
11699	PNMA3	104.353796	0.70160211	0.341968	2.051658	0.040203	0.067358
5107	MITF	1154.693259	0.701219867	0.148387	4.725606	2.29E-06	8.81E-06
4775	FCHO2	1619.177077	0.70057513	0.14308	4.896389	9.76E-07	4.01E-06
2430	SMARCAD	850.6701267	0.700367596	0.107226	6.531705	6.50E-11	5.25E-10
3832	MRPL42	888.3000861	0.699808744	0.128532	5.444646	5.19E-08	2.66E-07
11159	ARL4D	238.3554257	0.6996031	0.31301	2.235084	0.025412	0.044636
15200	OR7E37P	0.325532651	0.699540638	0.738257	0.947557	0.343355	0.44277
16222	PAR4	0.209999819	0.699539434	1.078014	0.648915	0.516393	0.623918
16334	LOC64392	0.186345191	0.699539176	1.133606	0.617092	0.537174	0.644615
16787	LOC34009	0.191966178	0.699537843	1.419596	0.492773	0.622173	0.726468
16949	OR1N2	0.231689139	0.699537259	1.552803	0.4505	0.65235	0.75442
16952	44086	0.166267786	0.699537047	1.553385	0.450331	0.652472	0.75443
17830	PRHOXNB	0.118110757	0.699525035	2.91407	0.240051	0.810291	0.890775

2686	MAPK1	5113.473381	0.698808596	0.111056	6.292387	3.13E-10	2.28E-09
11064	NR2E3	3.898752406	0.69874221	0.30802	2.268495	0.023299	0.041277
5574	GOT2	2739.938492	0.698561058	0.155706	4.486401	7.24E-06	2.55E-05
10795	FAM95B1	56.44313581	0.698441351	0.295496	2.363626	0.018097	0.032859
5967	C14orf33	39.14367432	0.698267646	0.163046	4.282637	1.85E-05	6.07E-05
8761	IPCEF1	163.0403633	0.698182115	0.225089	3.101808	0.001923	0.004303
3733	ZDHHC13	439.405913	0.698081938	0.12652	5.517581	3.44E-08	1.80E-07
2996	AKIRIN1	1035.456762	0.698042809	0.115693	6.033583	1.60E-09	1.05E-08
9575	HOMER1	167.9945745	0.697833324	0.250154	2.789611	0.005277	0.010803
4125	SLC25A46	824.3946627	0.697823185	0.132477	5.267491	1.38E-07	6.57E-07
5166	TRIM44	1559.393669	0.69771577	0.148719	4.691502	2.71E-06	1.03E-05
14123	SLC10A1	0.362630267	0.697596503	0.544721	1.280648	0.200317	0.278016
4175	FAM177A1	1053.627032	0.697264799	0.133259	5.232405	1.67E-07	7.86E-07
5256	TMEM170I	331.5390911	0.697142353	0.15	4.647602	3.36E-06	1.25E-05
13758	SERPINC1	6.236976347	0.6970126	0.497322	1.401531	0.161055	0.229455
11415	GALNTL1	174.0340472	0.696988302	0.325165	2.143489	0.032074	0.055074
8343	FAM186B	16.56034079	0.696909232	0.212715	3.276258	0.001052	0.002471
7176	ARHGAP18	1690.972515	0.696610222	0.185609	3.753096	0.000175	0.000477
4145	PPP2R5E	660.1733395	0.69631099	0.132559	5.252827	1.50E-07	7.08E-07
7599	MPZL2	1566.688418	0.695916371	0.194398	3.579847	0.000344	0.000887
5706	ZNF619	50.18553809	0.69555749	0.157483	4.416717	1.00E-05	3.44E-05
13213	CLEC18C	738.4760585	0.695460755	0.441457	1.575377	0.115169	0.17085
3155	ZNF765	220.4587048	0.69538325	0.117539	5.916187	3.29E-09	2.05E-08
11360	SYCP2	67.1602663	0.695311246	0.321371	2.16358	0.030497	0.05262
7803	FOXC1	415.2587344	0.695216842	0.198621	3.500212	0.000465	0.001168
5739	UEVLD	286.8547155	0.694682244	0.157798	4.402338	1.07E-05	3.66E-05
3201	C2orf24	3229.562543	0.694433642	0.118038	5.883144	4.03E-09	2.46E-08
6610	GADD45A	1485.164012	0.694418842	0.174511	3.979217	6.91E-05	0.000205
3153	RNF170	640.2213096	0.694296705	0.117348	5.916563	3.29E-09	2.04E-08
2606	RNF13	2328.819074	0.694205477	0.108935	6.372644	1.86E-10	1.40E-09
1908	OSBP	2365.778512	0.693650986	0.097965	7.080632	1.43E-12	1.47E-11
12618	TCTE1	1.146510481	0.693452186	0.392447	1.766995	0.077229	0.119969
4229	CNOT6L	1460.718012	0.693245099	0.133466	5.194175	2.06E-07	9.53E-07
3367	NDFIP1	4567.933075	0.693207783	0.1202	5.767129	8.06E-09	4.69E-08
2087	EXD2	583.7634575	0.693079313	0.101063	6.857891	6.99E-12	6.56E-11
4040	MYLIP	902.2032819	0.692925934	0.130437	5.312326	1.08E-07	5.25E-07
3168	NARS2	357.2446339	0.69269116	0.117277	5.906457	3.50E-09	2.16E-08
4255	LRPPRC	3717.784153	0.692687481	0.133725	5.179953	2.22E-07	1.02E-06
10252	ADRA2B	105.3928701	0.692177921	0.269895	2.564622	0.010329	0.019748
10015	SDR42E1	110.337919	0.692177352	0.261444	2.647522	0.008108	0.01587
3486	APC	944.2426576	0.691666681	0.121701	5.683326	1.32E-08	7.43E-08
7710	PPP2R3A	2023.40699	0.691653132	0.195617	3.535744	0.000407	0.001034
3020	SLMAP	857.4699528	0.691014647	0.114805	6.019046	1.75E-09	1.14E-08
3631	YIPF6	349.5075347	0.690877277	0.12375	5.582857	2.37E-08	1.28E-07
5098	ANKIB1	1940.80129	0.690810288	0.146109	4.728038	2.27E-06	8.72E-06
4617	ACSL3	1594.352501	0.69044427	0.138602	4.981499	6.31E-07	2.68E-06
8658	ABR	2055.556518	0.690020289	0.219223	3.147566	0.001646	0.003727

3754	HMGCS1	1145.073235	0.690011069	0.125356	5.504434	3.70E-08	1.93E-07
3076	YLPM1	1314.201802	0.689947952	0.115401	5.978704	2.25E-09	1.43E-08
4076	CASC4	4396.78496	0.689892874	0.130336	5.293189	1.20E-07	5.78E-07
3605	NCKAP1	4776.192947	0.689715248	0.123082	5.603703	2.10E-08	1.14E-07
3282	ERCC4	286.9844356	0.689621666	0.118295	5.829677	5.55E-09	3.32E-08
2279	ZNF828	434.9519046	0.689503226	0.103352	6.671432	2.53E-11	2.18E-10
3522	EGLN1	3210.953021	0.689338278	0.121851	5.657209	1.54E-08	8.56E-08
4395	PDCD4	1952.687979	0.689230422	0.134947	5.107401	3.27E-07	1.46E-06
5667	LYRM7	661.6499531	0.689182476	0.155287	4.438132	9.07E-06	3.14E-05
5404	RC3H2	415.4960782	0.689050167	0.151037	4.562127	5.06E-06	1.84E-05
5562	MSMP	35.61033882	0.689027132	0.153389	4.49203	7.05E-06	2.49E-05
4794	HSD17B4	2805.064256	0.688847418	0.140878	4.889689	1.01E-06	4.13E-06
6092	TXLNG	172.2291435	0.688772587	0.16303	4.224828	2.39E-05	7.69E-05
1946	FBXW2	1488.083975	0.688662806	0.097839	7.038759	1.94E-12	1.95E-11
1496	PPP6C	1721.678461	0.688240397	0.090872	7.57371	3.63E-14	4.75E-13
7146	KLF6	8570.670543	0.68812712	0.182752	3.765358	0.000166	0.000456
13084	GCM1	7.008517957	0.688066243	0.425381	1.617528	0.105764	0.158444
3872	AKAP11	1625.687754	0.687607334	0.126844	5.420902	5.93E-08	3.00E-07
3241	TSTD2	388.5959031	0.687360198	0.117285	5.860575	4.61E-09	2.79E-08
8438	LOC25845	624.7897328	0.68732017	0.212673	3.231817	0.00123	0.002857
3539	USP46	365.4431618	0.687135768	0.121795	5.641724	1.68E-08	9.32E-08
11983	CDH23	120.4380758	0.687048991	0.349744	1.964436	0.04948	0.080929
862	ZBTB5	423.1338796	0.686680393	0.079705	8.615319	6.97E-18	1.59E-16
11848	BCO2	52.37408454	0.686390652	0.342033	2.006799	0.044771	0.074068
1368	UBE3A	1872.602328	0.686263792	0.088346	7.767915	7.98E-15	1.14E-13
1959	PIK3R4	797.0557298	0.686179455	0.097704	7.023022	2.17E-12	2.17E-11
9343	NOX4	866.8289364	0.685687539	0.238042	2.880529	0.00397	0.008329
4679	NPAT	449.438306	0.685457224	0.138614	4.94507	7.61E-07	3.19E-06
8796	C10orf41	66.8644518	0.685418372	0.222019	3.087199	0.002021	0.004503
2375	LEMD3	800.516279	0.68519624	0.10405	6.58529	4.54E-11	3.75E-10
4359	STAG2	2565.860991	0.684559349	0.133528	5.126712	2.95E-07	1.33E-06
4272	KLHL15	318.5822979	0.684384016	0.132362	5.170562	2.33E-07	1.07E-06
10209	DCDC1	3.595440943	0.683771554	0.265207	2.578252	0.00993	0.019066
8374	KSR1	2351.799312	0.683404324	0.209585	3.260751	0.001111	0.002601
2711	TJP1	2214.52615	0.683367544	0.10902	6.268298	3.65E-10	2.64E-09
4301	UTP20	728.485909	0.683313852	0.132528	5.156008	2.52E-07	1.15E-06
14785	CDKL4	0.750327137	0.68325867	0.637247	1.072203	0.283629	0.376017
7979	AUTS2	854.350733	0.683252601	0.199842	3.41897	0.000629	0.001544
4622	PCMTD1	1744.441995	0.683179494	0.137195	4.979617	6.37E-07	2.70E-06
1219	SCFD1	944.4785481	0.683177551	0.085436	7.996398	1.28E-15	2.06E-14
4883	CROT	440.9457699	0.682904677	0.141165	4.837649	1.31E-06	5.27E-06
10393	NR2F1	332.3334784	0.682793069	0.272045	2.509857	0.012078	0.022779
2746	AKAP1	1188.344339	0.682780009	0.109396	6.241349	4.34E-10	3.10E-09
6570	LYPLA1	1629.391163	0.682430152	0.170839	3.994575	6.48E-05	0.000193
10897	F8	2295.565496	0.682263961	0.293834	2.321938	0.020236	0.0364
5652	MIB1	1244.712288	0.682240278	0.153435	4.446437	8.73E-06	3.03E-05
2963	AFF1	4080.391979	0.681887218	0.112501	6.061159	1.35E-09	8.94E-09

4653	TUBE1	240.8717256	0.681850504	0.137484	4.959472	7.07E-07	2.98E-06
6136	FAM45B	180.1431483	0.681749535	0.162101	4.205713	2.60E-05	8.31E-05
12275	LRRC43	15.38637486	0.681719114	0.363849	1.873629	0.060982	0.097377
8531	TAOK1	413.3478218	0.681692259	0.213351	3.19517	0.001397	0.003211
6659	JUB	838.2263065	0.68145163	0.172135	3.958814	7.53E-05	0.000222
7234	SLC30A1	396.1483705	0.681396982	0.182606	3.731506	0.00019	0.000516
2044	ACVR2A	345.5830711	0.681079009	0.09848	6.915946	4.65E-12	4.46E-11
4847	GNG12	4277.48986	0.68090733	0.139902	4.867045	1.13E-06	4.58E-06
10290	WBSCR27	131.479984	0.680602759	0.266901	2.550017	0.010772	0.020519
1773	SIK3	842.9676677	0.680113908	0.094228	7.217771	5.28E-13	5.84E-12
5112	C2orf69	455.3861958	0.680082066	0.144027	4.721901	2.34E-06	8.96E-06
6270	ARAP2	496.5296054	0.680053669	0.163979	4.147209	3.37E-05	0.000105
10128	LRRC55	13.02023952	0.679761385	0.260863	2.605814	0.009166	0.017738
10886	PLEK2	287.0480649	0.679199657	0.292059	2.325557	0.020042	0.036087
3885	PLD1	794.9510851	0.679153289	0.125479	5.412486	6.22E-08	3.14E-07
3100	ORC4L	693.1481209	0.678745584	0.113822	5.963232	2.47E-09	1.56E-08
6366	RORA	1097.510426	0.678455365	0.165674	4.095124	4.22E-05	0.00013
5588	ZNF121	62.56278215	0.678370225	0.151435	4.479602	7.48E-06	2.62E-05
11263	A4GNT	11.31423252	0.678303412	0.309206	2.193694	0.028257	0.049176
3860	TP53INP2	2697.890003	0.678285248	0.124989	5.426754	5.74E-08	2.91E-07
5401	ZNF525	242.7011655	0.678165602	0.148537	4.565642	4.98E-06	1.81E-05
2276	C2orf47	356.0906096	0.677689117	0.101552	6.673327	2.50E-11	2.15E-10
11553	UGT2B7	2933.559751	0.677521833	0.32309	2.097004	0.035993	0.061067
4889	KIF16B	592.7096465	0.677483095	0.140165	4.833457	1.34E-06	5.38E-06
4786	EPB41L4A	1484.842386	0.677399128	0.13846	4.892389	9.96E-07	4.08E-06
1873	TMLHE	273.2711457	0.677297113	0.095158	7.117633	1.10E-12	1.15E-11
4038	RNF11	2974.590179	0.676780371	0.12738	5.313079	1.08E-07	5.23E-07
7277	WWC1	2292.832955	0.676246818	0.182264	3.710252	0.000207	0.000558
10360	NNAT	7.36853923	0.675419622	0.267743	2.522637	0.011648	0.022038
9767	FSIP1	22.22733945	0.675329911	0.247789	2.725426	0.006422	0.012885
7004	TIGD2	149.5979518	0.675176666	0.176651	3.822097	0.000132	0.00037
3448	SH3RF1	673.3615896	0.675070458	0.118254	5.708631	1.14E-08	6.47E-08
957	HNRNPH2	2294.124667	0.674901816	0.080031	8.432994	3.37E-17	6.90E-16
2166	SPIN1	2658.516488	0.674814138	0.099292	6.796237	1.07E-11	9.72E-11
3414	ABHD13	489.9826948	0.674371004	0.117527	5.738003	9.58E-09	5.50E-08
4609	YTHDF3	2284.711234	0.674137153	0.135252	4.984311	6.22E-07	2.64E-06
15658	RP1-177G6.	0.521672037	0.673883105	0.822108	0.819701	0.412386	0.516234
2868	OPA1	1737.896734	0.673682262	0.1099	6.129964	8.79E-10	6.01E-09
2979	NAA16	244.6254313	0.672917026	0.111223	6.050161	1.45E-09	9.52E-09
11097	CLEC9A	19.65322864	0.672795179	0.298219	2.256045	0.024068	0.042512
10326	CYP26B1	130.4950686	0.672677792	0.265088	2.537566	0.011163	0.021187
15846	RIM6-TRIM	2.33977795	0.672468109	0.884585	0.760208	0.447131	0.553086
6387	AMOTL1	1390.945642	0.672208194	0.16465	4.082654	4.45E-05	0.000137
2551	ZNF585B	100.6821674	0.671995681	0.104582	6.425557	1.31E-10	1.01E-09
2360	MRPL44	778.2785026	0.671887968	0.101834	6.597851	4.17E-11	3.46E-10
3788	SCP2	3931.523161	0.671682006	0.122566	5.48017	4.25E-08	2.20E-07
5050	ZNF443	86.33253099	0.671613048	0.141515	4.745876	2.08E-06	8.06E-06

11694	ARG2	1403.823741	0.671144803	0.326957	2.052697	0.040102	0.067217
9616	FOXF1	135.2370736	0.671094991	0.241905	2.774209	0.005534	0.01128
14570	FLJ45983	1.570480838	0.670789489	0.585621	1.145433	0.25203	0.339055
6142	TMEM129	1303.746781	0.670722327	0.15953	4.204377	2.62E-05	8.35E-05
2400	RG9MTD1	426.5064274	0.670675349	0.102239	6.55989	5.38E-11	4.40E-10
5849	C3orf33	152.6430969	0.670649734	0.154275	4.347117	1.38E-05	4.62E-05
13796	LOC33953	271.2210772	0.670578626	0.483306	1.387482	0.165295	0.234829
1995	SMAD4	1721.730016	0.670527257	0.096228	6.968078	3.21E-12	3.16E-11
11570	C6orf142	26.45763614	0.670202075	0.320299	2.092424	0.036401	0.061667
8812	C6orf195	3.255874562	0.669518524	0.217123	3.083593	0.002045	0.004549
6141	ARL5A	1448.649773	0.66947578	0.159213	4.204916	2.61E-05	8.34E-05
6544	BCL7A	513.6845356	0.669461778	0.167062	4.007256	6.14E-05	0.000184
12044	CXCL10	926.3305371	0.669365009	0.344038	1.945613	0.051701	0.084139
2502	COX18	419.5563103	0.66894373	0.103577	6.458441	1.06E-10	8.29E-10
16470	PSG5	1.302327476	0.668767908	1.149728	0.581675	0.560786	0.667393
8005	RIF1	837.9064262	0.668574773	0.1962	3.407627	0.000655	0.001605
1821	ZNF527	124.3414382	0.668267097	0.093175	7.172136	7.38E-13	7.95E-12
6670	GSR	1246.923373	0.667936777	0.168911	3.954368	7.67E-05	0.000226
2127	ARPP19	4259.232979	0.667859089	0.097871	6.823894	8.86E-12	8.16E-11
4026	PIGO	890.8174617	0.667854332	0.12543	5.324534	1.01E-07	4.93E-07
5143	TMEM17	87.13244086	0.667842751	0.141889	4.706806	2.52E-06	9.59E-06
5110	ZNF124	71.95700371	0.667787719	0.14139	4.723017	2.32E-06	8.91E-06
2985	MUL1	954.6030441	0.667228228	0.110341	6.046942	1.48E-09	9.69E-09
1434	GIGYF2	1916.635953	0.667035528	0.087083	7.659797	1.86E-14	2.55E-13
4615	ZNF773	109.2092315	0.666963449	0.133876	4.981951	6.29E-07	2.67E-06
5719	LIN52	113.498167	0.666934459	0.151216	4.410461	1.03E-05	3.54E-05
6338	SREBF2	2086.50153	0.66674979	0.162263	4.109059	3.97E-05	0.000123
910	ZC3H14	1002.698862	0.666638593	0.078391	8.504061	1.83E-17	3.94E-16
4078	SRFBP1	273.5006949	0.666607424	0.125961	5.29218	1.21E-07	5.81E-07
3370	CDC40	651.9955504	0.666530331	0.115579	5.766865	8.08E-09	4.70E-08
3194	NDUFB6	838.8105349	0.666454134	0.113213	5.886713	3.94E-09	2.42E-08
7359	C9orf43	9.437977944	0.666093181	0.181299	3.67401	0.000239	0.000636
13830	MUM1L1	95.12122615	0.66599346	0.484406	1.374865	0.169173	0.239766
1694	JKAMP	978.0589908	0.665835668	0.091189	7.30175	2.84E-13	3.29E-12
7722	MAGI2	255.0219944	0.665595374	0.188455	3.531853	0.000413	0.001047
2250	SPG11	1698.401335	0.665450475	0.099367	6.696917	2.13E-11	1.85E-10
12494	LDHAL6A	1.774844648	0.665414053	0.368431	1.806077	0.070906	0.11124
3378	NF1	2057.92994	0.665268676	0.11548	5.76092	8.37E-09	4.85E-08
4263	CPNE3	2986.51232	0.665121781	0.128527	5.174967	2.28E-07	1.05E-06
5277	PCCB	1210.07344	0.664971123	0.143375	4.637984	3.52E-06	1.31E-05
5235	ENDOD1	2382.53965	0.664824337	0.142706	4.658714	3.18E-06	1.19E-05
4869	PEBP1	11050.62034	0.664751888	0.137007	4.85194	1.22E-06	4.92E-06
2733	43895	820.5482219	0.664553456	0.106257	6.254235	3.99E-10	2.86E-09
5221	IMPA1	614.5675113	0.664493924	0.142313	4.669245	3.02E-06	1.13E-05
1351	SRP54	1111.173713	0.664441749	0.085205	7.79815	6.28E-15	9.11E-14
10022	PCDHGA8	13.25962859	0.664251673	0.251174	2.64459	0.008179	0.015996
10113	DPY19L2P	143.1405992	0.664065556	0.254171	2.612676	0.008984	0.017412

8155	SSX2IP	719.1775262	0.663883668	0.19825	3.348712	0.000812	0.001951
6437	ZNF284	38.35458588	0.663735796	0.163691	4.054808	5.02E-05	0.000153
12510	EYA4	41.22862581	0.663583941	0.368527	1.800638	0.07176	0.112435
4483	KIAA1430	1295.947559	0.663307155	0.131383	5.04864	4.45E-07	1.95E-06
1305	TBCK	518.7749116	0.663231394	0.084433	7.855163	3.99E-15	6.00E-14
4657	KBTBD4	369.0443455	0.663057448	0.13378	4.956327	7.18E-07	3.02E-06
8076	ZNF135	129.9936061	0.662629613	0.195965	3.381361	0.000721	0.001751
2161	TTC8	278.9753644	0.662523073	0.097436	6.799554	1.05E-11	9.52E-11
3543	PDIK1L	251.705032	0.662501204	0.117456	5.640424	1.70E-08	9.38E-08
5683	ZNF347	126.5919778	0.662474832	0.149576	4.429029	9.47E-06	3.26E-05
10292	MTL5	95.83129841	0.66238875	0.259884	2.548785	0.01081	0.020587
3719	TAX1BP1	3806.036997	0.662349267	0.119871	5.525521	3.29E-08	1.73E-07
6830	H1F0	2611.930283	0.66233026	0.170355	3.887942	0.000101	0.00029
6242	MAN1A2	838.5608978	0.662211507	0.159256	4.158154	3.21E-05	0.000101
3647	APPL1	1221.574514	0.662095752	0.118785	5.573915	2.49E-08	1.34E-07
2620	VPS4B	1186.303317	0.661997528	0.104131	6.357347	2.05E-10	1.54E-09
2136	FEM1B	1704.139207	0.66190361	0.097055	6.81988	9.11E-12	8.36E-11
5904	RRAS2	583.3404022	0.661874399	0.153202	4.320268	1.56E-05	5.17E-05
3663	PRKAA1	2188.650393	0.661814436	0.119013	5.560843	2.68E-08	1.44E-07
4826	VPS13C	1909.271524	0.661796127	0.135692	4.877182	1.08E-06	4.37E-06
9416	QRICH2	41.61041352	0.661771492	0.232025	2.85216	0.004342	0.009039
12339	KRT7	155.1098938	0.661572178	0.357132	1.852461	0.06396	0.101603
1546	WDR11	1200.225473	0.661528704	0.08825	7.496057	6.58E-14	8.34E-13
2655	RAB3GAP2	1176.387129	0.66125698	0.104588	6.322521	2.57E-10	1.90E-09
3919	ZNF606	266.1068124	0.661176305	0.12265	5.390747	7.02E-08	3.51E-07
1961	GNB5	709.8931998	0.661048322	0.094187	7.018501	2.24E-12	2.24E-11
2974	44085	3636.801175	0.660967748	0.109209	6.052332	1.43E-09	9.41E-09
5531	PRKACB	1582.185504	0.660805368	0.146686	4.504883	6.64E-06	2.35E-05
6246	ZNF827	218.726264	0.660789244	0.158985	4.156299	3.23E-05	0.000102
3335	SOCS6	745.1546179	0.660680271	0.114018	5.794507	6.85E-09	4.03E-08
7662	FRY	799.5698452	0.66047288	0.18585	3.553793	0.00038	0.000971
3713	ZNF564	285.3366089	0.66027387	0.119404	5.529765	3.21E-08	1.69E-07
4163	FPGT	509.0371097	0.660157982	0.125951	5.241384	1.59E-07	7.50E-07
2874	RC3H1	800.1301421	0.660107446	0.107758	6.125841	9.02E-10	6.15E-09
1617	WBP4	351.1604609	0.659194566	0.089024	7.404691	1.31E-13	1.59E-12
2535	ARFIP1	876.3735866	0.659164024	0.102455	6.433669	1.25E-10	9.63E-10
5109	C5orf24	2151.294776	0.658975266	0.139467	4.72494	2.30E-06	8.83E-06
5287	COMMD10	595.7026351	0.658868059	0.142323	4.629378	3.67E-06	1.36E-05
6640	DYNLT3	1121.811491	0.65886278	0.166076	3.967226	7.27E-05	0.000215
4288	NCOA4	6184.70258	0.658499806	0.127584	5.161319	2.45E-07	1.12E-06
11830	MASP1	943.1299523	0.658024425	0.327048	2.012013	0.044219	0.073265
9367	ELTD1	2167.163034	0.657826613	0.228917	2.873648	0.004058	0.00849
6814	KIAA1614	137.937657	0.657778374	0.168949	3.893349	9.89E-05	0.000284
10754	FMN1	50.96114971	0.657625045	0.276333	2.379825	0.017321	0.03157
5520	LOC1002893	60.09691607	0.657616261	0.145742	4.512189	6.42E-06	2.28E-05
11138	C3orf36	41.0429804	0.657403118	0.293456	2.240211	0.025077	0.044132
4217	POLR3B	230.1359325	0.657233394	0.126381	5.200424	1.99E-07	9.24E-07

5001	KIAA0776	1252.060134	0.657088821	0.137654	4.773482	1.81E-06	7.10E-06
5330	CASKIN2	1004.267731	0.656902049	0.142542	4.608487	4.06E-06	1.49E-05
6004	BRAF	181.6619549	0.656753151	0.153993	4.264834	2.00E-05	6.53E-05
13927	PGA3	27.61646218	0.656696409	0.489493	1.341584	0.179731	0.252955
6834	C10orf72	808.5954856	0.656682824	0.168967	3.886467	0.000102	0.000292
3761	BRPF3	1084.573928	0.656618075	0.119393	5.499635	3.81E-08	1.98E-07
5834	MOSPD1	346.5766073	0.65643726	0.150747	4.354564	1.33E-05	4.48E-05
5445	DHRS4L2	436.3819388	0.656427149	0.144444	4.54452	5.51E-06	1.98E-05
5923	GOLIM4	1369.079439	0.656289217	0.152203	4.311926	1.62E-05	5.36E-05
4557	TTC33	644.4854671	0.656078794	0.13107	5.005577	5.57E-07	2.40E-06
1827	LRRC57	262.0277196	0.655869566	0.09153	7.165656	7.74E-13	8.31E-12
4022	TSNAX	1051.468903	0.655811079	0.123098	5.327567	9.95E-08	4.85E-07
8356	LRRC34	76.04847302	0.6558082	0.200542	3.270182	0.001075	0.002521
3822	ZNF484	98.76943203	0.655703665	0.12022	5.454218	4.92E-08	2.52E-07
10053	C14orf64	96.45724205	0.655690152	0.248889	2.634464	0.008427	0.016431
11732	LRRC16B	35.52003086	0.655542063	0.320958	2.042454	0.041106	0.068678
2195	NARG2	849.1515896	0.655453357	0.096945	6.761116	1.37E-11	1.22E-10
5042	CD164	8857.554074	0.655164665	0.137871	4.752006	2.01E-06	7.83E-06
3477	ASH1L	1597.98067	0.65514731	0.115134	5.690288	1.27E-08	7.15E-08
4224	FUT10	110.802425	0.654970553	0.126022	5.197286	2.02E-07	9.38E-07
3405	IBTK	1592.606865	0.654711148	0.113999	5.743114	9.30E-09	5.35E-08
5839	PAQR3	325.7435452	0.654076468	0.150266	4.352803	1.34E-05	4.51E-05
13498	IL20RA	6.778878049	0.653947222	0.439115	1.48924	0.136424	0.198109
3551	ZNF480	459.1955439	0.653943804	0.116052	5.634905	1.75E-08	9.67E-08
3193	BPNT1	552.202168	0.65356889	0.111004	5.887771	3.91E-09	2.40E-08
13265	KLHL4	217.292944	0.653503191	0.419397	1.558198	0.119186	0.176115
6049	HIBADH	2408.775188	0.653338705	0.153992	4.242684	2.21E-05	7.16E-05
12267	ADAMTSL	162.472385	0.653194583	0.347809	1.878025	0.060378	0.096465
2642	MRPL49	1362.167027	0.652828526	0.10312	6.330786	2.44E-10	1.81E-09
3738	MED13	1780.950102	0.652700628	0.118382	5.513498	3.52E-08	1.84E-07
3727	PIP4K2C	1014.270731	0.652441821	0.118161	5.521612	3.36E-08	1.77E-07
3548	STAU2	886.4684635	0.65237197	0.115728	5.637105	1.73E-08	9.55E-08
5518	PTP4A1	3642.488394	0.651896696	0.144463	4.512557	6.41E-06	2.28E-05
12519	BCAN	36.68300045	0.651722475	0.3624	1.79835	0.072122	0.112921
8912	TSSK4	13.33985599	0.651715708	0.21386	3.047393	0.002308	0.005077
1846	DUSP3	3361.646245	0.651555868	0.0912	7.144274	9.05E-13	9.61E-12
8833	WDR78	134.9443524	0.651448399	0.21171	3.07708	0.00209	0.004639
7912	LRMP	403.2219042	0.651410534	0.18914	3.444073	0.000573	0.00142
4088	LZTFL1	517.1712274	0.651266449	0.123161	5.287922	1.24E-07	5.93E-07
3130	MID2	462.5659211	0.651247616	0.109615	5.941221	2.83E-09	1.77E-08
2728	IPPK	257.3707111	0.651007463	0.104058	6.256226	3.94E-10	2.83E-09
4650	PCYOX1	3893.43571	0.650933163	0.131223	4.960493	7.03E-07	2.96E-06
9467	MAN1A1	2171.881482	0.650919921	0.229882	2.831542	0.004632	0.009592
5174	RNF160	810.5587082	0.650822169	0.13881	4.68858	2.75E-06	1.04E-05
4606	DIRC2	365.6489654	0.650760323	0.130501	4.98665	6.14E-07	2.61E-06
5628	GTF2I	2653.438787	0.650685495	0.145993	4.456968	8.31E-06	2.90E-05
6284	PLD6	125.7910429	0.650455887	0.15718	4.138298	3.50E-05	0.000109

8164	ZNF503	403.3423913	0.650419197	0.194343	3.346754	0.000818	0.001963
11666	IL17RB	463.2176901	0.650196435	0.315896	2.058259	0.039565	0.066477
3696	PAPD5	488.920244	0.650144124	0.117421	5.536885	3.08E-08	1.63E-07
4716	ABCB10	545.7321227	0.650072929	0.132016	4.924185	8.47E-07	3.52E-06
6646	C21orf91	414.0664188	0.649935212	0.163904	3.965344	7.33E-05	0.000216
4963	PKD2	2478.94692	0.649857646	0.135649	4.790732	1.66E-06	6.56E-06
6222	PARD6G	249.6444237	0.649813192	0.155887	4.168493	3.07E-05	9.66E-05
1993	PAFAH2	541.3673874	0.649158111	0.093106	6.972265	3.12E-12	3.07E-11
12960	\NKRD20A	3.04425071	0.648915056	0.392257	1.654312	0.098064	0.148314
3396	ZNF720	278.5880312	0.648863197	0.11285	5.749781	8.94E-09	5.16E-08
15286	LIPJ	0.40862788	0.648797239	0.701844	0.924418	0.355269	0.455556
3312	RSF1	676.9324622	0.64875709	0.111683	5.808919	6.29E-09	3.72E-08
7188	ISPD	114.2442172	0.648745915	0.173029	3.749341	0.000177	0.000483
1380	MRPS31	329.4659702	0.648669566	0.08373	7.747195	9.39E-15	1.33E-13
5226	EIF4E3	937.6272914	0.648646937	0.139008	4.666264	3.07E-06	1.15E-05
8592	UGCG	587.3715274	0.648587211	0.204209	3.176089	0.001493	0.003405
10086	TRPV4	600.9440164	0.648467521	0.247242	2.622809	0.008721	0.016948
8901	GPR137C	40.38694948	0.648445462	0.212501	3.051495	0.002277	0.005014
4836	ZNF287	123.3466733	0.648329905	0.133087	4.871482	1.11E-06	4.49E-06
8925	S1PR1	1752.151578	0.648071621	0.213027	3.042204	0.002349	0.005157
3492	C18orf19	595.9290788	0.647660751	0.114	5.681232	1.34E-08	7.51E-08
3900	BLZF1	615.4890341	0.647518592	0.11983	5.403647	6.53E-08	3.28E-07
5283	ZNF33B	1201.348735	0.647283858	0.139664	4.634576	3.58E-06	1.33E-05
7626	CBWD3	211.8395059	0.646963977	0.181298	3.568515	0.000359	0.000923
2930	FRS2	734.1109935	0.646886879	0.106259	6.087821	1.14E-09	7.66E-09
9469	MATN2	974.4854232	0.64673601	0.228446	2.831024	0.00464	0.009605
4687	OTUD6B	255.7670043	0.646657585	0.131023	4.935444	8.00E-07	3.34E-06
2898	ZFP14	228.4084997	0.646650446	0.105778	6.113259	9.76E-10	6.60E-09
8668	TMEM47	2040.463157	0.64656397	0.205594	3.144863	0.001662	0.003758
7289	PLAGL2	574.4545655	0.646263397	0.174498	3.703555	0.000213	0.000572
2501	TRIM13	886.3998829	0.646092871	0.100021	6.459565	1.05E-10	8.23E-10
2621	EID1	4810.440929	0.645805207	0.101592	6.356869	2.06E-10	1.54E-09
11831	RGS7BP	64.83675875	0.645768615	0.321084	2.011216	0.044303	0.073398
4530	RAD50	1611.381643	0.645758762	0.128673	5.018612	5.20E-07	2.25E-06
1566	USP33	1470.623565	0.645641879	0.086395	7.473141	7.83E-14	9.80E-13
5674	GABPA	939.1217269	0.645193339	0.145463	4.435456	9.19E-06	3.17E-05
7193	TMEM106A	389.2008485	0.644795768	0.172013	3.748527	0.000178	0.000485
11228	BARX2	804.9426101	0.644793408	0.291844	2.209376	0.027148	0.047394
10829	CELF6	124.8780765	0.644446116	0.274045	2.351604	0.018693	0.033835
4561	FAM35A	863.5699045	0.644439254	0.128792	5.003704	5.62E-07	2.42E-06
14235	CLGN	126.5563889	0.644303007	0.516957	1.246338	0.21264	0.292797
4569	GNAQ	2466.046439	0.644037133	0.128824	4.999367	5.75E-07	2.47E-06
4501	LANCL1	1802.922371	0.643929055	0.127805	5.038384	4.69E-07	2.04E-06
11441	TARP	31.4701207	0.643915758	0.301403	2.136393	0.032647	0.055932
2538	CCDC25	869.3228789	0.643912425	0.100102	6.432541	1.25E-10	9.69E-10
8446	IFIT1	1143.999261	0.643848241	0.199375	3.229329	0.001241	0.00288
4599	LMBR1	1127.597093	0.643745207	0.129048	4.988421	6.09E-07	2.59E-06

9356	SCAND3	107.5768137	0.643615798	0.223623	2.878129	0.004	0.008381
11219	TTLL7	94.84288638	0.643565674	0.290567	2.214863	0.026769	0.04677
16194	CCDC60	0.3782414	0.643072038	0.981321	0.655312	0.512267	0.619964
8757	CERKL	220.5295276	0.643050634	0.20724	3.102928	0.001916	0.004289
1975	NBR1	4052.590121	0.64291177	0.091899	6.995888	2.64E-12	2.62E-11
4277	PYROXD1	641.1500616	0.642855902	0.124391	5.168023	2.37E-07	1.08E-06
14856	SLC39A2	2.2731819	0.642296469	0.611158	1.050951	0.293281	0.386955
2426	ZNF14	195.7504802	0.642279042	0.098266	6.536135	6.31E-11	5.10E-10
5822	NIPSNAP3I	53.34773923	0.642173892	0.147231	4.361684	1.29E-05	4.35E-05
1654	PEX19	1857.547055	0.641927989	0.087295	7.353578	1.93E-13	2.29E-12
15980	OR2T11	0.232719112	0.641832376	0.894418	0.717598	0.473005	0.580186
16077	ZFATAS	0.179060589	0.641832193	0.929713	0.690356	0.489971	0.59737
16941	KRT40	0.200858619	0.641830221	1.418422	0.452496	0.650912	0.753115
17095	TMEM8C	0.203193121	0.64182956	1.546569	0.415002	0.67814	0.777551
17103	GRK1	0.162315241	0.641829358	1.558055	0.411943	0.680381	0.779755
17249	C3orf77	0.158929427	0.641828519	1.701342	0.377248	0.705989	0.802208
17356	PSG6	0.361361796	0.641828496	1.826373	0.351422	0.725271	0.819102
17305	HIST1H2BA	0.097182847	0.641827918	1.755175	0.365677	0.714606	0.809445
17379	PSG7	0.203492843	0.641827735	1.855023	0.345995	0.729347	0.822598
17383	SELV	0.198320678	0.641827709	1.859105	0.345235	0.729918	0.823001
17823	AMTN	0.170330454	0.641821032	2.65704	0.241555	0.809125	0.889842
17913	ANTXRL	0.09833784	0.641818478	2.844527	0.225633	0.821487	0.898848
17939	OR51T1	0.106656381	0.641817723	2.91407	0.220248	0.825678	0.902175
8361	DHRS11	161.7897165	0.641242347	0.1963	3.266639	0.001088	0.002551
4949	ZNF790	129.8033077	0.641104422	0.133579	4.799433	1.59E-06	6.30E-06
8402	C9orf129	68.63623267	0.641052274	0.197382	3.24778	0.001163	0.002713
3491	APPBP2	1134.368078	0.640895594	0.112796	5.681917	1.33E-08	7.48E-08
10518	AKR7L	57.16922057	0.640890167	0.259983	2.465126	0.013696	0.025524
4035	SPTY2D1	926.9448807	0.640858866	0.120593	5.314219	1.07E-07	5.20E-07
1910	VEZF1	1550.634264	0.640772348	0.090554	7.076121	1.48E-12	1.52E-11
16368	VGLL1	0.434268711	0.640689015	1.05243	0.608771	0.542676	0.649865
13334	SLC9A10	1.360263298	0.640187264	0.417771	1.532389	0.125427	0.184377
5039	MUTED	623.1237563	0.640139138	0.134651	4.754061	1.99E-06	7.76E-06
10415	LRP5L	86.41278071	0.64010194	0.255513	2.505163	0.012239	0.023035
3655	KDSR	2181.087102	0.640015458	0.11497	5.566795	2.59E-08	1.39E-07
6658	C1orf58	319.5617136	0.639992894	0.161626	3.95972	7.50E-05	0.000221
8887	TSPAN18	2028.548089	0.639926541	0.209341	3.056867	0.002237	0.004933
4984	KIAA0317	808.6855791	0.639776768	0.133787	4.782048	1.74E-06	6.82E-06
14982	CPNE4	1.031532266	0.639517161	0.632558	1.011002	0.312016	0.408211
2366	MAPK1IP1I	2321.580975	0.639461845	0.096997	6.592614	4.32E-11	3.58E-10
3503	XPA	236.9561773	0.638867879	0.112643	5.671639	1.41E-08	7.91E-08
4567	ZNF670	48.88680663	0.638826969	0.127745	5.000817	5.71E-07	2.45E-06
6751	CHMP4C	270.7597664	0.638742003	0.163007	3.918484	8.91E-05	0.000259
4877	SIPA1L1	1372.300365	0.638667079	0.131862	4.843459	1.28E-06	5.13E-06
3035	ZNF566	188.6958054	0.638586703	0.106289	6.008015	1.88E-09	1.21E-08
9509	RHOB	15223.32103	0.638538449	0.226703	2.816625	0.004853	0.010004
3005	ZNF562	413.4809899	0.638225566	0.105831	6.030598	1.63E-09	1.07E-08

4257	LOC344595	120.7102666	0.637412202	0.12307	5.179272	2.23E-07	1.03E-06
12205	DPYS	1155.297429	0.637145784	0.336223	1.895008	0.058091	0.093294
4736	EXTL2	363.2203448	0.637120877	0.129599	4.916096	8.83E-07	3.65E-06
10079	LOC28545(	2.591168756	0.637120709	0.242673	2.625433	0.008654	0.01683
4547	ZNF800	552.149522	0.636996464	0.127128	5.010687	5.42E-07	2.34E-06
4515	KLHL8	469.1409197	0.636962113	0.12669	5.02771	4.96E-07	2.15E-06
3257	ZNF559	285.1556384	0.636813058	0.108896	5.847876	4.98E-09	3.00E-08
4593	TMEM66	10567.38856	0.636807589	0.127599	4.99069	6.02E-07	2.57E-06
2432	SLAIN2	1297.087385	0.636772616	0.097524	6.529421	6.60E-11	5.32E-10
6928	INSR	6468.173782	0.636729382	0.165295	3.852085	0.000117	0.000331
3698	SPRYD4	423.8466172	0.636506004	0.114974	5.536075	3.09E-08	1.64E-07
14444	DGKK	0.439405998	0.636292283	0.537444	1.183922	0.236444	0.320877
6436	CAPN5	577.1108562	0.636075948	0.156869	4.05482	5.02E-05	0.000153
6328	PANK3	445.4573488	0.636075406	0.154641	4.113246	3.90E-05	0.000121
1974	TDRD7	501.1341004	0.635556877	0.090834	6.99694	2.62E-12	2.60E-11
2567	COPS4	795.1256321	0.635511709	0.099115	6.411888	1.44E-10	1.10E-09
10686	KIF26A	182.4478838	0.635177127	0.264534	2.401119	0.016345	0.029981
3451	VPS54	837.9014636	0.634216587	0.111145	5.706233	1.16E-08	6.56E-08
6038	APBB1	846.4893623	0.634189852	0.149226	4.249861	2.14E-05	6.94E-05
5239	C20orf7	189.5379586	0.634124585	0.136176	4.656647	3.21E-06	1.20E-05
9198	GATSL1	13.62055558	0.634111222	0.215599	2.941164	0.00327	0.006968
2027	ZNF506	430.4796204	0.634046708	0.09147	6.931739	4.16E-12	4.02E-11
1721	FAM175B	469.467254	0.633980864	0.087171	7.272871	3.52E-13	4.01E-12
8454	KIAA1467	467.0904596	0.633905492	0.196432	3.227099	0.001251	0.002899
7369	PLA2G4C	414.7632395	0.633740886	0.172659	3.67047	0.000242	0.000644
8099	AKAP3	19.00339616	0.63352886	0.187918	3.371296	0.000748	0.001811
4268	MDH1	3167.20227	0.633256471	0.122432	5.172307	2.31E-07	1.06E-06
5395	FAR1	1265.22512	0.633242693	0.138627	4.567954	4.93E-06	1.79E-05
9116	HOXB6	227.1301794	0.633092447	0.212992	2.972374	0.002955	0.006354
4996	CHMP2B	983.1879848	0.632727747	0.13248	4.776021	1.79E-06	7.01E-06
2983	SPG20	2189.764084	0.632608642	0.104605	6.047622	1.47E-09	9.66E-09
3575	CDC42BPE	3357.323442	0.632067252	0.112452	5.620777	1.90E-08	1.04E-07
13726	F5	601.4620727	0.632051057	0.449022	1.407618	0.159244	0.227404
5605	C9orf130	171.4417258	0.631749756	0.141392	4.468064	7.89E-06	2.76E-05
6269	TMEM133	394.9783418	0.631516646	0.152245	4.148015	3.35E-05	0.000105
7951	NUDT4	1076.440626	0.631353074	0.184193	3.427663	0.000609	0.001501
9845	NAALADL:	81.92063718	0.631255417	0.233306	2.7057	0.006816	0.01357
1535	GALK2	537.7220132	0.631039228	0.084015	7.511046	5.87E-14	7.49E-13
10306	GAS6	2949.098177	0.630906274	0.248075	2.543205	0.010984	0.020891
7194	ZNF460	57.39747832	0.630701381	0.168259	3.748401	0.000178	0.000485
3298	MEF2A	2453.045292	0.63012851	0.108321	5.817251	5.98E-09	3.56E-08
4292	DARS2	577.5822454	0.630100843	0.122117	5.159817	2.47E-07	1.13E-06
12001	ZNF826	349.4244181	0.630039523	0.32141	1.960237	0.049968	0.081612
6237	ARHGAP2(	3056.849461	0.629825729	0.151419	4.159491	3.19E-05	0.0001
7175	SOX13	768.9217875	0.629223038	0.167616	3.753964	0.000174	0.000476
4418	C8orf42	565.9333778	0.629097515	0.123568	5.091123	3.56E-07	1.58E-06
12090	ST14	1626.52246	0.628595586	0.325435	1.931557	0.053414	0.086598

2644	C4orf41	856.2587797	0.6282708	0.099261	6.329455	2.46E-10	1.82E-09
2455	FASTKD2	974.4104587	0.628249931	0.096515	6.509357	7.55E-11	6.03E-10
3635	SLC25A12	562.9575891	0.628131788	0.112537	5.581541	2.38E-08	1.29E-07
7366	LRP5	1684.404057	0.628104224	0.171031	3.672462	0.00024	0.000639
12209	IGSF22	10.31764432	0.627980491	0.331555	1.894048	0.058219	0.093467
6119	ZNF10	231.3809568	0.627954424	0.149104	4.211527	2.54E-05	8.12E-05
13184	CNKSR1	31.79047471	0.627926862	0.396276	1.58457	0.113064	0.168095
7822	LYSMD2	435.5661589	0.627770866	0.179719	3.493067	0.000478	0.001197
5469	FAM91A1	1770.577501	0.627755985	0.138451	4.53415	5.78E-06	2.07E-05
14401	CAPSL	0.884175537	0.627460557	0.523908	1.197655	0.231051	0.314481
2892	BTBD9	450.8740485	0.627450634	0.102597	6.115707	9.61E-10	6.52E-09
1675	TRMT5	371.8975114	0.627266895	0.085568	7.330617	2.29E-13	2.68E-12
9103	HEY1	459.7495422	0.627242116	0.21079	2.975668	0.002924	0.006295
4945	KLF12	720.2889318	0.627143644	0.130589	4.802418	1.57E-06	6.21E-06
1868	REV1	652.6867134	0.627138794	0.088066	7.121214	1.07E-12	1.12E-11
8505	FLJ37201	19.43825933	0.627112319	0.195813	3.202607	0.001362	0.003138
1828	DNAJA2	2049.828972	0.627050451	0.08751	7.165455	7.75E-13	8.31E-12
5191	SENP7	515.4064837	0.626792096	0.133936	4.679775	2.87E-06	1.08E-05
7116	HSD17B11	1589.155693	0.626423868	0.165762	3.779052	0.000157	0.000434
4531	ZFX	643.5726066	0.626299525	0.124799	5.018466	5.21E-07	2.25E-06
2055	LARP7	846.1576105	0.626255196	0.090781	6.898551	5.25E-12	5.01E-11
3062	CRBN	688.0451656	0.625956826	0.10448	5.99114	2.08E-09	1.33E-08
8320	SEMA5A	1280.724931	0.625596012	0.190542	3.283247	0.001026	0.002417
8874	SEPP1	12931.22675	0.625499271	0.204251	3.062409	0.002196	0.00485
3391	RFXAP	99.71309619	0.625279024	0.108684	5.75317	8.76E-09	5.06E-08
5139	PRKAB2	619.7563506	0.624837879	0.132695	4.708825	2.49E-06	9.50E-06
7258	PTGR1	1497.873892	0.624551253	0.16778	3.722438	0.000197	0.000533
4594	DICER1	1389.863928	0.624398792	0.125121	4.99035	6.03E-07	2.57E-06
12526	PACSIN1	41.51241918	0.624333145	0.347478	1.796756	0.072374	0.113244
3679	ARL1	3208.358754	0.624254302	0.112442	5.551792	2.83E-08	1.51E-07
1651	PRPF4B	1462.69126	0.624034894	0.084821	7.357104	1.88E-13	2.23E-12
12388	TPRG1	46.93645761	0.623966121	0.339179	1.839636	0.065822	0.104147
5151	MLLT4	2274.784297	0.623902782	0.13269	4.701974	2.58E-06	9.80E-06
4181	ITFG1	1467.258217	0.623788915	0.119373	5.225537	1.74E-07	8.14E-07
7358	AKAP7	266.7665825	0.623744566	0.169746	3.674569	0.000238	0.000635
6952	QDPR	1182.000088	0.623651724	0.162203	3.844873	0.000121	0.00034
13698	KCNJ10	61.29161629	0.622772243	0.439137	1.418172	0.156141	0.223428
3138	FAM76A	246.4241877	0.622742959	0.104932	5.934756	2.94E-09	1.84E-08
6802	WRN	266.0696105	0.622741112	0.159685	3.899817	9.63E-05	0.000277
5684	SLC17A5	1043.034265	0.622386922	0.140531	4.428821	9.47E-06	3.27E-05
4322	ZNF148	1442.898523	0.622300073	0.120923	5.146262	2.66E-07	1.21E-06
6363	CEP97	117.9587251	0.62223394	0.15192	4.095795	4.21E-05	0.00013
5757	ZNF671	204.1974428	0.622159631	0.14163	4.392862	1.12E-05	3.81E-05
4424	PSIP1	1460.335986	0.622121557	0.122275	5.087869	3.62E-07	1.60E-06
4246	NPTN	3189.08234	0.62207507	0.120012	5.183462	2.18E-07	1.01E-06
12713	SLC22A14	2.67883492	0.622058494	0.359936	1.728249	0.083944	0.129425
11384	GCNT3	420.2029329	0.621967828	0.288214	2.15801	0.030927	0.05325

10668	KBTBD10	13.02724488	0.621474983	0.258394	2.405144	0.016166	0.029703
5985	JHRF1BP1I	736.8843501	0.621461228	0.145346	4.275734	1.91E-05	6.24E-05
6835	CHML	661.778359	0.621269551	0.159858	3.886374	0.000102	0.000292
6949	ROCK2	1058.336904	0.621145023	0.161535	3.845255	0.00012	0.00034
14738	KCNIP1	10.00836149	0.621075864	0.571204	1.087311	0.276899	0.368266
8308	ITGAV	8629.883646	0.620996777	0.188784	3.289452	0.001004	0.002368
9928	ZC3H12B	42.60402629	0.620653786	0.231777	2.677808	0.007411	0.014631
1399	MTO1	426.3614212	0.62062717	0.080441	7.715271	1.21E-14	1.69E-13
12016	ABCB1	803.1345487	0.620618195	0.317227	1.956384	0.05042	0.082239
3736	PCBD2	462.1568043	0.620183482	0.112456	5.514901	3.49E-08	1.83E-07
10765	CDKL5	86.49266351	0.620142716	0.26092	2.37675	0.017466	0.031802
12830	AOX1	1421.745701	0.620052872	0.365722	1.69542	0.089996	0.137491
2092	KIF1B	1667.620339	0.619589082	0.090406	6.85339	7.21E-12	6.76E-11
3440	CAPN7	553.4939869	0.619474414	0.108366	5.716499	1.09E-08	6.20E-08
8103	STON1	548.6904444	0.619356289	0.183764	3.370389	0.000751	0.001816
3517	TGDS	266.5076098	0.619094427	0.10934	5.662129	1.50E-08	8.33E-08
4636	BCOR	838.9357363	0.618962357	0.124518	4.97085	6.67E-07	2.82E-06
1401	HELQ	268.1531729	0.618846386	0.08023	7.713373	1.23E-14	1.71E-13
6058	POLI	581.5338719	0.618835819	0.145927	4.240731	2.23E-05	7.21E-05
9450	CCNT1	184.9068791	0.618527545	0.217956	2.837855	0.004542	0.00942
6593	PPIP5K1	507.8960479	0.618294271	0.155138	3.985441	6.74E-05	0.0002
10060	ZNF880	128.1190358	0.617778285	0.234642	2.63285	0.008467	0.016498
5563	MCCC2	2169.022326	0.617732192	0.137538	4.491369	7.08E-06	2.49E-05
3452	FAM200B	442.9049532	0.617342119	0.108192	5.705995	1.16E-08	6.57E-08
1924	RABGAP1	1187.234546	0.617154631	0.087408	7.060637	1.66E-12	1.69E-11
10660	AMPD3	738.7620929	0.617150851	0.256274	2.408173	0.016033	0.02948
5169	KDM6A	579.8713871	0.616468703	0.131433	4.690378	2.73E-06	1.03E-05
7069	TWF1	2607.946475	0.616385052	0.162406	3.795335	0.000147	0.000409
4813	CDC73	1210.80436	0.616380491	0.126246	4.882358	1.05E-06	4.27E-06
8813	OPLAH	466.2408569	0.616367656	0.199921	3.083055	0.002049	0.004557
5152	CISD2	1150.188652	0.616203727	0.131057	4.701785	2.58E-06	9.81E-06
9361	NAALAD2	16.13217432	0.616104266	0.21421	2.876163	0.004025	0.008429
13712	GRIA3	40.81870605	0.616087053	0.435487	1.414708	0.157154	0.224648
8347	LRIG3	631.33828	0.616079242	0.188116	3.274997	0.001057	0.002481
4343	RABL3	691.6942675	0.616078596	0.119952	5.136041	2.81E-07	1.27E-06
3473	UBQLN1	2897.52525	0.615956771	0.108169	5.694393	1.24E-08	6.99E-08
7341	HNMT	1908.399215	0.615927966	0.167186	3.684084	0.00023	0.000613
2781	MPPE1	895.7706783	0.61540412	0.099063	6.212224	5.22E-10	3.68E-09
11502	GPR126	1373.199922	0.615244947	0.290618	2.117026	0.034258	0.05838
12871	FMO2	884.4932255	0.614865982	0.364871	1.685161	0.091957	0.140029
11274	PRSS8	933.3600522	0.614788451	0.280818	2.189281	0.028576	0.049683
9783	SCML2	23.77165706	0.614423563	0.225757	2.721615	0.006496	0.013016
1944	ZRANB1	813.4462804	0.614409247	0.087277	7.039784	1.93E-12	1.94E-11
4142	SLC35A5	953.8456393	0.614404074	0.116856	5.257804	1.46E-07	6.90E-07
3610	UFSP2	840.1991171	0.614371198	0.109678	5.601583	2.12E-08	1.15E-07
2645	;ABARAPL	1995.318491	0.614199754	0.097061	6.327988	2.48E-10	1.84E-09
8561	GPR137B	1208.12498	0.61412787	0.192738	3.186342	0.001441	0.003299

3834	EAPP	764.3596029	0.613949503	0.112787	5.443421	5.23E-08	2.67E-07
5187	CCNYL1	646.2133595	0.613803191	0.131112	4.6815	2.85E-06	1.08E-05
2862	ANKRD17	2209.651346	0.613742808	0.100061	6.133696	8.59E-10	5.88E-09
4269	RUFY2	360.3419682	0.613631083	0.118647	5.171901	2.32E-07	1.06E-06
7119	EHBP1	1412.303652	0.613587293	0.162416	3.777882	0.000158	0.000435
9934	BAIAP3	140.7678699	0.613453425	0.229186	2.676663	0.007436	0.014672
5907	ZFAND5	5462.213625	0.613374633	0.142021	4.31889	1.57E-05	5.20E-05
4456	BOD1L	1244.50786	0.613172286	0.12108	5.064171	4.10E-07	1.80E-06
4746	ZNF618	829.0945689	0.613152198	0.124869	4.910361	9.09E-07	3.75E-06
4981	THAP10	74.09710116	0.612887928	0.128141	4.7829	1.73E-06	6.80E-06
5223	DECR1	1902.834926	0.612701253	0.131282	4.667067	3.06E-06	1.15E-05
7466	ZNF614	315.9496399	0.61247682	0.168759	3.6293	0.000284	0.000746
8314	LEPROT	1069.859248	0.612359286	0.186353	3.286016	0.001016	0.002396
4170	DDX3X	7309.245723	0.612301162	0.116934	5.236297	1.64E-07	7.70E-07
9824	C20orf152	3.980510105	0.612298008	0.225933	2.71009	0.006726	0.013419
3133	DDX52	522.3317088	0.612129951	0.1031	5.937242	2.90E-09	1.81E-08
5662	CRAT	1522.167837	0.612122202	0.13781	4.441768	8.92E-06	3.09E-05
17684	RGPD8	0.411131399	0.611983757	2.251069	0.271864	0.785727	0.87091
3945	TLK1	1323.661013	0.611806149	0.113773	5.377421	7.56E-08	3.75E-07
4474	CCDC132	509.4003323	0.61165996	0.120985	5.055657	4.29E-07	1.88E-06
3188	CCNDBP1	1140.587011	0.611059131	0.103756	5.889409	3.88E-09	2.38E-08
5601	TET2	655.2133187	0.611041829	0.136658	4.471319	7.77E-06	2.72E-05
5085	RAB33B	393.2514196	0.610897238	0.129136	4.730668	2.24E-06	8.63E-06
3074	DDX24	2816.435598	0.610697582	0.10212	5.980208	2.23E-09	1.42E-08
5477	RCN2	992.8604449	0.610616443	0.134733	4.532033	5.84E-06	2.09E-05
4666	LRRC49	154.205989	0.610498262	0.123241	4.953714	7.28E-07	3.06E-06
5524	JMJD1C	1673.177384	0.60991363	0.135306	4.50767	6.55E-06	2.33E-05
1610	KIAA0391	655.0870033	0.609839916	0.082291	7.41081	1.26E-13	1.53E-12
3708	PPP2CB	2430.638082	0.609586172	0.110189	5.532163	3.16E-08	1.67E-07
4783	ERI2	325.0017232	0.609376932	0.12454	4.89302	9.93E-07	4.07E-06
7739	C17orf97	184.3597496	0.609340867	0.172947	3.523291	0.000426	0.001079
4543	C12orf26	102.3856432	0.609270561	0.121559	5.012136	5.38E-07	2.32E-06
4968	PHF6	609.3051972	0.609232138	0.127213	4.789075	1.68E-06	6.61E-06
2702	FAM168B	2758.168639	0.609172075	0.097022	6.278708	3.41E-10	2.48E-09
7679	WHAMML2	70.7981786	0.60903774	0.171679	3.547548	0.000389	0.000993
5688	C9orf3	517.5031869	0.609002866	0.137576	4.426652	9.57E-06	3.30E-05
4120	EARS2	742.1500268	0.609002744	0.115565	5.269795	1.37E-07	6.50E-07
7910	PHLPP1	303.6025597	0.608976169	0.176795	3.444542	0.000572	0.001417
2099	UBE2D3	6210.976039	0.608750062	0.088861	6.85061	7.35E-12	6.87E-11
13048	CKB	4298.476591	0.608684433	0.373223	1.630887	0.102914	0.1546
9733	EIF3C	288.9837375	0.608559508	0.222284	2.73776	0.006186	0.012458
2449	TRMT61B	282.7254116	0.608490183	0.093426	6.513047	7.36E-11	5.89E-10
13585	RAMP1	95.50970522	0.60820405	0.416824	1.459139	0.144527	0.208527
6157	ZNF43	408.2983311	0.608008147	0.144789	4.199273	2.68E-05	8.52E-05
5691	CNTLN	316.2806493	0.607408519	0.13724	4.425901	9.60E-06	3.31E-05
14711	LOC339240	0.561418247	0.607241659	0.554813	1.094497	0.273737	0.364728
4334	TMEM80	244.7434586	0.607146005	0.118114	5.140337	2.74E-07	1.24E-06

4298	METT5D1	448.636301	0.607076443	0.117697	5.157982	2.50E-07	1.14E-06
14608	PRSS55	0.682151343	0.606952631	0.536767	1.130756	0.258158	0.346396
6649	ATP11C	881.9602006	0.606647476	0.153116	3.962002	7.43E-05	0.000219
10883	FGFBP2	37.55148665	0.606419944	0.260509	2.327823	0.019921	0.03588
3024	SNX19	1759.936766	0.606416269	0.100779	6.017276	1.77E-09	1.15E-08
5377	SNX24	375.7774991	0.606182823	0.132413	4.577985	4.69E-06	1.71E-05
11444	MYO7A	569.9963078	0.60539308	0.283425	2.135994	0.03268	0.055973
1922	SUV420H1	940.2686622	0.604997804	0.085661	7.062736	1.63E-12	1.66E-11
3709	ZNF280D	720.2967133	0.604867468	0.109339	5.532029	3.17E-08	1.67E-07
11227	FCN3	376.5655931	0.604627771	0.273493	2.210765	0.027052	0.04723
11938	SLC16A10	116.1610934	0.604444056	0.30548	1.978672	0.047853	0.07857
7440	MYSM1	130.1861453	0.604428751	0.166049	3.640067	0.000273	0.000718
6524	SCYL2	911.0647634	0.604412527	0.150512	4.015723	5.93E-05	0.000178
3457	C6orf162	110.4526691	0.604340653	0.105986	5.702091	1.18E-08	6.71E-08
7030	SPIN4	136.7391033	0.603752637	0.158518	3.80873	0.00014	0.000389
5314	ZNF345	86.27214868	0.603627924	0.130827	4.613944	3.95E-06	1.46E-05
10193	C6orf164	11.4126555	0.603485381	0.233439	2.5852	0.009732	0.018715
7064	TTC30A	442.1677497	0.603125153	0.158863	3.796518	0.000147	0.000407
4428	ECHDC1	1095.572516	0.602930493	0.118554	5.085694	3.66E-07	1.62E-06
1500	ALKBH1	241.3607178	0.602523998	0.079629	7.566672	3.83E-14	5.00E-13
4358	ACTR6	491.71699	0.602237787	0.117469	5.126777	2.95E-07	1.33E-06
10745	INPP4B	321.3982024	0.602105097	0.252684	2.382837	0.01718	0.031339
9036	C20orf132	31.37035894	0.601883058	0.200641	2.999805	0.002702	0.00586
13323	GPC3	269.149781	0.601641782	0.391849	1.53539	0.124688	0.183443
8201	KREMEN1	754.6631862	0.601559316	0.180537	3.332055	0.000862	0.00206
6618	EFCAB6	58.23166016	0.601541688	0.151249	3.977174	6.97E-05	0.000207
8408	MRPS25	804.5351095	0.601510681	0.185284	3.246421	0.001169	0.002724
12921	CACNA1E	85.32511383	0.601427175	0.36077	1.667064	0.095502	0.144875
9520	TMOD1	252.9144522	0.601346336	0.213793	2.812752	0.004912	0.010113
3796	ZNF180	152.3187095	0.601151672	0.109789	5.475507	4.36E-08	2.25E-07
6479	SPAG1	237.5786254	0.601126043	0.148913	4.036756	5.42E-05	0.000164
4314	MNAT1	193.4393355	0.600938494	0.116721	5.14851	2.63E-07	1.19E-06
3797	COQ10B	847.1890087	0.600881016	0.109745	5.475244	4.37E-08	2.26E-07
6552	LOC64621	135.2605689	0.600727966	0.149982	4.005341	6.19E-05	0.000185
6347	SPATA6	109.3411277	0.600714523	0.146335	4.105071	4.04E-05	0.000125
5863	LEPROTL1	2128.059766	0.600426645	0.138367	4.339364	1.43E-05	4.78E-05
2648	INTS6	732.0737176	0.600333792	0.0949	6.32596	2.52E-10	1.86E-09
9207	VLDLR	1683.49463	0.60025199	0.20446	2.935789	0.003327	0.007083
13706	RNF186	421.4461051	0.59924726	0.42316	1.416124	0.156739	0.224153
2890	MPHOSPH	796.299772	0.599192932	0.097944	6.117706	9.49E-10	6.44E-09
4888	NUPL1	1374.105233	0.599190305	0.123958	4.83383	1.34E-06	5.37E-06
5104	PDHB	1111.839195	0.599087537	0.126749	4.726571	2.28E-06	8.77E-06
6925	KCNRG	7.720505881	0.599068998	0.155414	3.854655	0.000116	0.000328
3835	COQ6	367.0366884	0.598833886	0.110036	5.442159	5.26E-08	2.69E-07
4967	TANC1	966.3835945	0.598690655	0.125009	4.789172	1.67E-06	6.61E-06
5884	XPR1	1701.226354	0.598508592	0.138199	4.330782	1.49E-05	4.95E-05
11958	C1orf190	57.98037407	0.598297887	0.303403	1.971957	0.048615	0.079687

10469	RAPGEF3	543.7949899	0.59817697	0.240643	2.485748	0.012928	0.024205
12604	FXYD2	12894.53999	0.598062736	0.337701	1.770981	0.076564	0.119068
12940	PRODH	1438.470268	0.598018271	0.359777	1.662193	0.096474	0.146135
4193	43897	1628.939633	0.597379589	0.114587	5.213317	1.85E-07	8.67E-07
8065	FMO4	277.6924823	0.597364004	0.176407	3.386288	0.000708	0.001722
3315	NAF1	160.1663788	0.597336472	0.102876	5.806399	6.38E-09	3.77E-08
5829	KIAA0240	629.7411146	0.597284264	0.137112	4.356166	1.32E-05	4.45E-05
8774	LEPR	537.3961231	0.597202466	0.192965	3.094868	0.001969	0.004399
8809	ATP1A1	19498.77114	0.59691906	0.193559	3.083912	0.002043	0.004546
9175	GNG7	245.6707289	0.59691473	0.202526	2.947354	0.003205	0.006847
9466	FLJ13197	39.20690441	0.59636695	0.210618	2.831509	0.004633	0.009592
9428	IL6R	601.7149868	0.596180252	0.209366	2.847557	0.004406	0.009159
3538	ESCO1	418.7818298	0.596068625	0.105635	5.64274	1.67E-08	9.27E-08
8254	SCAI	259.6552171	0.596019917	0.179995	3.311318	0.000929	0.002205
2774	ZXDB	398.2640767	0.59589395	0.095862	6.216143	5.10E-10	3.60E-09
6950	LOC64567(	33.62940794	0.595328688	0.15483	3.845056	0.000121	0.00034
7378	TTC30B	210.3208329	0.595164583	0.162312	3.666804	0.000246	0.000653
4706	USP28	478.1440336	0.59514667	0.120755	4.92853	8.29E-07	3.45E-06
5100	KAT2B	819.2401686	0.595066707	0.125884	4.7271	2.28E-06	8.75E-06
7926	NMNAT3	173.690904	0.59505125	0.173077	3.438079	0.000586	0.001449
5456	MRPS36	609.4931713	0.594411325	0.13092	4.540246	5.62E-06	2.02E-05
7213	KIAA0664	2274.569613	0.594390133	0.158972	3.738969	0.000185	0.000502
5055	HSPA9	8973.88307	0.594371077	0.125287	4.744072	2.09E-06	8.12E-06
10001	HCG2P7	8.508303114	0.59412093	0.22402	2.652094	0.007999	0.015677
4020	ATG2B	961.6878181	0.594099311	0.111496	5.328418	9.91E-08	4.83E-07
12932	ENDOU	2.429490322	0.59366487	0.356724	1.664212	0.09607	0.145613
10949	C9orf153	3.543549592	0.593573906	0.257627	2.304007	0.021222	0.037992
5219	SMCR8	948.6849932	0.593339729	0.127021	4.67119	2.99E-06	1.12E-05
3693	ZNF33A	1080.868028	0.593313139	0.10713	5.538234	3.06E-08	1.62E-07
11737	NEURL3	74.96100324	0.593038395	0.290498	2.041454	0.041206	0.068814
5704	RAB11FIP5	2269.865508	0.592994426	0.134191	4.419039	9.91E-06	3.41E-05
10302	C1orf126	181.5931927	0.592896085	0.233019	2.544407	0.010946	0.020825
16502	ITLN2	0.389180455	0.592844824	1.036077	0.572201	0.567186	0.6737
5420	NBPF1	429.9374484	0.59251344	0.130055	4.555858	5.22E-06	1.89E-05
2277	NCOA1	1503.267027	0.592503874	0.088789	6.673139	2.50E-11	2.16E-10
8903	COX5A	2010.687148	0.592435052	0.194189	3.050822	0.002282	0.005024
2154	RAD17	701.3776174	0.59235526	0.087029	6.806447	1.00E-11	9.10E-11
13989	FAM81B	37.55253952	0.59231894	0.448184	1.321597	0.186302	0.261042
8092	KLF8	395.9180737	0.592311198	0.175579	3.373479	0.000742	0.001798
7242	PAFAH1B2	516.5666451	0.59228937	0.158852	3.728557	0.000193	0.000521
7622	BCR	1382.709936	0.591708967	0.165743	3.570032	0.000357	0.000918
4015	DHX29	899.9845912	0.591702348	0.111019	5.329747	9.83E-08	4.80E-07
4067	ZFP91	2159.38114	0.591511287	0.111662	5.297316	1.18E-07	5.66E-07
6730	ANKAR	50.14271872	0.591148061	0.150411	3.930221	8.49E-05	0.000247
8567	TBC1D8B	546.3556796	0.59065368	0.185466	3.184708	0.001449	0.003315
11171	AGBL4	5.745276696	0.590597779	0.264731	2.230938	0.025685	0.045064
3793	ZCCHC11	670.2148651	0.590212933	0.107765	5.476873	4.33E-08	2.24E-07

13305	LRRC3B	2.72497933	0.589905228	0.38273	1.541308	0.123242	0.181561
7042	PDE4D	1144.446489	0.589632999	0.155035	3.803233	0.000143	0.000398
5974	EFCAB7	139.1755616	0.589617139	0.137739	4.280671	1.86E-05	6.11E-05
4853	RANBP2	2481.883967	0.589580965	0.121244	4.862762	1.16E-06	4.68E-06
10154	TAL1	162.2537174	0.589513909	0.226933	2.597746	0.009384	0.018114
13414	STXBP6	115.9724442	0.589419333	0.39046	1.509552	0.131158	0.191652
10359	ALDH1A1	9752.854461	0.589392772	0.23363	2.522765	0.011644	0.022032
5061	THRA	949.6832005	0.589238764	0.124283	4.741086	2.13E-06	8.23E-06
5619	POC1B	504.0047168	0.589135067	0.132014	4.462676	8.09E-06	2.82E-05
3508	GSK3B	1253.818846	0.589039939	0.103939	5.66715	1.45E-08	8.12E-08
11761	TPD52L1	432.829581	0.588964843	0.289633	2.033483	0.042004	0.070004
4937	ISCA1P1	94.72584186	0.588952637	0.122558	4.8055	1.54E-06	6.13E-06
11324	SLC22A15	144.5962059	0.588634149	0.270984	2.172207	0.02984	0.051651
4001	DNAJC1	965.2232506	0.588354688	0.110186	5.339666	9.31E-08	4.56E-07
6010	PHF16	330.7542217	0.588259081	0.138059	4.260935	2.04E-05	6.64E-05
6501	FOXN3	2084.353934	0.587758976	0.145917	4.028048	5.62E-05	0.00017
3643	ABHD5	409.7686109	0.587747459	0.1054	5.576337	2.46E-08	1.32E-07
4993	GTPBP8	147.1578557	0.587669594	0.123006	4.777563	1.77E-06	6.97E-06
7201	PPP4R2	352.1048762	0.587598298	0.156873	3.745696	0.00018	0.00049
7638	C5orf55	113.427743	0.58734358	0.164744	3.565195	0.000364	0.000933
4789	CAB39	2248.459399	0.58730471	0.120073	4.891232	1.00E-06	4.10E-06
3513	PLEKHA3	427.0827667	0.587293841	0.103693	5.663793	1.48E-08	8.26E-08
4425	CPPED1	1373.920679	0.587139206	0.115418	5.087047	3.64E-07	1.61E-06
4926	CTDSPL2	685.7661135	0.586947397	0.121898	4.815051	1.47E-06	5.86E-06
7134	LDHB	11565.31294	0.586889139	0.155596	3.771881	0.000162	0.000445
1441	DHX15	2165.520342	0.586838829	0.076782	7.642924	2.12E-14	2.89E-13
4628	FNIP1	982.4457297	0.586826932	0.117949	4.975268	6.52E-07	2.76E-06
4870	SNRNP48	349.1046489	0.586766501	0.120967	4.850633	1.23E-06	4.95E-06
9080	LOC64728[illegible]	14.00017332	0.58667112	0.196413	2.986927	0.002818	0.006083
6189	TBL1X	1755.585401	0.586576606	0.140298	4.180939	2.90E-05	9.19E-05
3791	CGGBP1	2200.498809	0.586385236	0.107022	5.479117	4.27E-08	2.21E-07
9514	SLAIN1	150.19767	0.586353172	0.208341	2.814394	0.004887	0.010068
9951	MAP9	387.6084915	0.586228329	0.219475	2.671053	0.007561	0.014895
4415	RTN4	9375.721953	0.586171566	0.115103	5.092567	3.53E-07	1.57E-06
6456	CCDC122	59.76062637	0.58610978	0.144847	4.046392	5.20E-05	0.000158
10179	C12orf75	1704.050357	0.585840031	0.226219	2.589704	0.009606	0.018497
4905	RSBN1	686.2206232	0.585808743	0.121427	4.824358	1.40E-06	5.61E-06
3794	N4BP2L2	1297.378185	0.585671558	0.106941	5.476583	4.34E-08	2.24E-07
8868	LOC65365[illegible]	127.7148005	0.585601049	0.191124	3.063981	0.002184	0.004828
8210	UAP1	1234.899033	0.585416442	0.175935	3.327454	0.000876	0.002092
2881	DSTYK	697.5117564	0.585231499	0.095573	6.123374	9.16E-10	6.23E-09
3149	ADNP2	611.1803059	0.585117371	0.098801	5.922158	3.18E-09	1.98E-08
5035	PMPCB	1915.789154	0.585002845	0.123014	4.755578	1.98E-06	7.70E-06
8417	TECTA	15.71979081	0.584915514	0.180335	3.243494	0.001181	0.00275
6100	DNAJC13	1761.13824	0.584905378	0.138554	4.221498	2.43E-05	7.80E-05
4694	SMAD5	1716.692296	0.584823399	0.118581	4.931835	8.15E-07	3.40E-06
5695	PJA2	4795.802958	0.584378993	0.132131	4.422721	9.75E-06	3.35E-05

3881	BAG5	1296.079051	0.584128129	0.107887	5.414239	6.15E-08	3.11E-07
16446	STH	0.182741926	0.58412426	0.99325	0.588094	0.556469	0.663222
16984	TCEB3C	0.252472029	0.584123146	1.318646	0.442972	0.657786	0.759142
17015	IL1F7	0.256609357	0.584123065	1.337625	0.436687	0.662339	0.762967
16979	NT5C1A	0.189975315	0.584123012	1.31491	0.44423	0.656876	0.758271
17108	RBP2	0.216649431	0.584122558	1.425988	0.409627	0.68208	0.781473
17263	C9orf4	0.135060293	0.584121611	1.56092	0.374216	0.708243	0.803799
17375	DAZ2	0.292840862	0.584121567	1.682378	0.3472	0.728441	0.821765
17534	C11orf36	0.130112399	0.584119526	1.901413	0.307203	0.758689	0.848127
17649	LOC34035[illegible]	0.159378631	0.584118338	2.087594	0.279805	0.779627	0.865855
17979	POU3F4	0.109797503	0.584111881	2.794788	0.209	0.834448	0.909678
18027	CCDC63	0.111888648	0.584110657	2.91407	0.200445	0.841133	0.914466
18028	DMRTC1	0.091612751	0.584110412	2.91407	0.200445	0.841133	0.914466
3361	METAP1	942.4280803	0.584010096	0.10119	5.771427	7.86E-09	4.58E-08
4413	CREBL2	2981.337895	0.58397317	0.114643	5.093827	3.51E-07	1.56E-06
3922	METTL4	241.3240725	0.583914558	0.108329	5.390208	7.04E-08	3.52E-07
9871	SHROOM2	350.7054265	0.583859216	0.216523	2.696518	0.007007	0.013914
4329	ATG4A	443.3328496	0.583779152	0.113526	5.14225	2.71E-07	1.23E-06
6936	TMED5	3167.453037	0.583710759	0.151658	3.848871	0.000119	0.000335
3351	ZNF75D	426.0802008	0.583652717	0.100987	5.779476	7.49E-09	4.38E-08
3388	WDR7	589.3668503	0.583644892	0.101407	5.755477	8.64E-09	5.00E-08
11369	COL27A1	739.818069	0.583605246	0.270029	2.161268	0.030675	0.052885
10230	ST6GAL1	3102.488914	0.583538789	0.226901	2.571777	0.010118	0.019386
12426	SLC28A1	2118.107307	0.583495091	0.319117	1.828468	0.067479	0.106443
5853	KIAA1797	687.8177133	0.583479495	0.134318	4.344005	1.40E-05	4.69E-05
4087	MIER3	644.8837911	0.583448212	0.110329	5.288249	1.23E-07	5.92E-07
11713	KIAA0754	247.4312245	0.583436039	0.284766	2.048828	0.040479	0.067739
5205	CWF19L2	391.0471479	0.583357579	0.124796	4.674502	2.95E-06	1.11E-05
5369	IFIT5	837.9043591	0.583287362	0.127257	4.583538	4.57E-06	1.67E-05
6470	PRKCI	941.4300239	0.583229088	0.144303	4.041698	5.31E-05	0.000161
7674	SPHAR	147.507156	0.583183362	0.164307	3.549359	0.000386	0.000986
4846	MAT2B	2484.236227	0.583178235	0.119779	4.868803	1.12E-06	4.54E-06
5294	BDP1	949.587611	0.583139718	0.126099	4.624469	3.76E-06	1.39E-05
7965	PGAP1	398.2036698	0.583105139	0.170368	3.422619	0.00062	0.001526
5665	CAMK2D	1105.482621	0.583049303	0.13134	4.439237	9.03E-06	3.12E-05
7174	PI4K2B	509.0531472	0.582631038	0.155204	3.753959	0.000174	0.000476
15374	HPVC1	0.479600467	0.58209766	0.648525	0.897572	0.369414	0.470982
8098	PRDM5	47.23949857	0.582081313	0.172654	3.371368	0.000748	0.00181
13470	C17orf102	1.96751244	0.581723362	0.388603	1.496961	0.134403	0.195578
6227	HERPUD1	6054.601632	0.581639652	0.139647	4.165063	3.11E-05	9.80E-05
5508	MRPS28	306.1839894	0.581501723	0.128808	4.514467	6.35E-06	2.26E-05
2997	RNF6	876.3043888	0.581393454	0.096359	6.03362	1.60E-09	1.05E-08
6362	C14orf43	1444.053313	0.5811931	0.141891	4.09604	4.20E-05	0.000129
6460	ZNF256	102.6092558	0.581081953	0.143672	4.044507	5.24E-05	0.000159
2381	ACOX1	1601.654959	0.581028028	0.088332	6.577783	4.78E-11	3.93E-10
9927	SMPDL3A	2338.056153	0.580892039	0.216877	2.678437	0.007397	0.014605
11292	C10orf79	39.50271422	0.580567709	0.266272	2.180358	0.029231	0.05074

1352	MTPAP	459.2054317	0.580436448	0.074435	7.797876	6.30E-15	9.13E-14
9586	PI4KAP2	162.3536671	0.58022989	0.208366	2.784673	0.005358	0.010956
3104	TADA1	278.8848838	0.580209943	0.097324	5.961644	2.50E-09	1.58E-08
10241	IL6ST	2016.85592	0.579829807	0.225635	2.569768	0.010177	0.019478
6490	KCTD9	1057.767661	0.579826714	0.143795	4.032322	5.52E-05	0.000167
5608	MKKS	1060.230301	0.579759073	0.129772	4.467532	7.91E-06	2.77E-05
10338	RBM20	112.5356694	0.579466752	0.228695	2.533793	0.011284	0.021394
8127	LOC72832	22.4454559	0.579395006	0.172428	3.360208	0.000779	0.001878
3506	CBARA1	1545.673649	0.579302702	0.102222	5.667082	1.45E-08	8.12E-08
8435	SH3BGR	78.39801332	0.579241839	0.179044	3.235193	0.001216	0.002825
2889	ZNF595	167.0261526	0.579116323	0.094635	6.119482	9.39E-10	6.37E-09
7067	C14orf118	223.6896712	0.579065074	0.152563	3.795575	0.000147	0.000409
6472	ALG10B	371.8662557	0.578961576	0.143261	4.041302	5.32E-05	0.000161
8243	HIPK2	9294.255382	0.578854262	0.174639	3.314577	0.000918	0.002182
4987	ZNF268	407.3224988	0.578637195	0.121039	4.780577	1.75E-06	6.87E-06
6312	KLHL28	359.2801534	0.577711834	0.140239	4.119469	3.80E-05	0.000118
5672	LOC72917	28.43037599	0.577507241	0.130191	4.435839	9.17E-06	3.17E-05
11941	NLGN1	538.861577	0.577219062	0.291751	1.978465	0.047876	0.078588
5703	CHPT1	1187.316325	0.577149249	0.130595	4.419396	9.90E-06	3.40E-05
12677	ZNF157	2.063739361	0.576857181	0.330446	1.745694	0.080864	0.125031
4430	DCUN1D4	1165.8982	0.576805475	0.113465	5.083573	3.70E-07	1.64E-06
4498	MGA	799.3127665	0.576581859	0.114408	5.039719	4.66E-07	2.03E-06
13309	CRNA0011	1.197756485	0.576383192	0.374285	1.539957	0.123571	0.181991
5015	HSDL1	594.1952686	0.57604046	0.120901	4.764557	1.89E-06	7.40E-06
2956	ZNF17	133.1657825	0.575978381	0.094925	6.067725	1.30E-09	8.60E-09
14025	KIAA2022	27.01794069	0.575921136	0.438955	1.312027	0.189511	0.264856
2873	LOC40065	195.4500311	0.575870314	0.094002	6.126178	9.00E-10	6.14E-09
7416	NAT1	119.0790728	0.575846878	0.157778	3.649726	0.000263	0.000694
3847	TMEM168	701.5966535	0.5758245	0.10595	5.434876	5.48E-08	2.79E-07
6381	KIF13A	746.1994413	0.575703136	0.140898	4.085971	4.39E-05	0.000135
4259	ZSCAN2	278.0559072	0.575593283	0.111157	5.178199	2.24E-07	1.03E-06
5708	FAM117B	419.3385714	0.575574196	0.130332	4.416212	1.00E-05	3.45E-05
8291	PIH1D2	56.94395731	0.575531495	0.174531	3.297598	0.000975	0.002306
3408	GOLGA7	1263.918364	0.575390686	0.100215	5.741583	9.38E-09	5.39E-08
4446	AKTIP	893.988965	0.575331229	0.113363	5.075102	3.87E-07	1.71E-06
4005	MRPL19	1149.429492	0.575107669	0.10773	5.338412	9.38E-08	4.59E-07
6101	SETBP1	622.5165098	0.575065016	0.136224	4.221464	2.43E-05	7.80E-05
5526	PEX26	505.7567894	0.574887611	0.127556	4.506939	6.58E-06	2.33E-05
4168	OTUD7B	673.1130487	0.574881073	0.10978	5.236662	1.64E-07	7.69E-07
10929	KIF6	16.34021538	0.574857292	0.248893	2.309654	0.020907	0.037497
7623	UBXN7	318.5178718	0.574743937	0.161013	3.56955	0.000358	0.000919
790	ATXN7L3E	2678.138753	0.57461467	0.065488	8.774317	1.72E-18	4.27E-17
4367	GLO1	2393.055707	0.574541664	0.112179	5.121651	3.03E-07	1.36E-06
3069	ENOPH1	1000.826014	0.574409029	0.096013	5.982616	2.20E-09	1.40E-08
5118	LCLAT1	476.2680153	0.573967027	0.121643	4.718461	2.38E-06	9.10E-06
3207	C14orf101	433.372824	0.573933308	0.097606	5.880081	4.10E-09	2.51E-08
2839	INVS	293.7546493	0.573889231	0.093284	6.152037	7.65E-10	5.28E-09

3556	C1orf124	177.0743692	0.573661248	0.101873	5.631115	1.79E-08	9.87E-08
8450	LATS1	366.2654863	0.572893802	0.177508	3.227426	0.001249	0.002897
10582	STAG3L1	87.4809593	0.572817484	0.234601	2.441669	0.01462	0.02708
9722	HOXD4	84.2186799	0.572515548	0.208878	2.740913	0.006127	0.012353
5179	PHF3	1897.513302	0.572300981	0.122168	4.684532	2.81E-06	1.06E-05
6163	ATM	1746.471935	0.572286038	0.136409	4.195357	2.72E-05	8.66E-05
10041	C1orf88	216.5160156	0.572226198	0.216962	2.637445	0.008353	0.016306
3097	ALS2	672.6698902	0.57222397	0.095919	5.965697	2.44E-09	1.54E-08
10626	LOC44145	4.380842365	0.571778955	0.235921	2.423608	0.015367	0.028347
9796	DHCR7	695.5008877	0.571621777	0.210314	2.717942	0.006569	0.013144
3392	ZNF41	283.5437812	0.571510518	0.099339	5.753139	8.76E-09	5.06E-08
6723	ZNF625	26.4870966	0.571489022	0.145341	3.932057	8.42E-05	0.000246
4340	C5orf53	476.797793	0.571226061	0.111191	5.137326	2.79E-07	1.26E-06
3999	C11orf30	366.6410683	0.571115861	0.106933	5.340859	9.25E-08	4.53E-07
13272	C10orf93	14.26897806	0.5708468	0.367203	1.554579	0.120046	0.177293
12454	OC1003026	144.7967436	0.570756723	0.31372	1.819317	0.068863	0.108382
6696	LCORL	95.60779479	0.570477767	0.144727	3.941753	8.09E-05	0.000237
5613	DEPDC5	287.7472347	0.570169374	0.127664	4.466182	7.96E-06	2.78E-05
8081	HOXD8	557.427531	0.570017408	0.168715	3.378574	0.000729	0.001767
13803	ADH1C	9.121217282	0.569957035	0.411092	1.386447	0.165611	0.235176
7479	C5orf32	2612.946097	0.569920721	0.157295	3.623268	0.000291	0.000762
6721	ATP5G3	4118.191581	0.569801074	0.144891	3.932627	8.40E-05	0.000245
4437	SPATA7	235.7944387	0.569283183	0.112067	5.079851	3.78E-07	1.67E-06
7372	RNF5P1	245.5984372	0.569262214	0.155134	3.66948	0.000243	0.000646
5555	MOCS2	1221.243145	0.569203491	0.126581	4.496768	6.90E-06	2.43E-05
5275	TOLLIP	2033.721314	0.569068974	0.122669	4.639063	3.50E-06	1.30E-05
12590	C4orf37	1.440954402	0.568959713	0.32023	1.776719	0.075614	0.117722
7237	IPW	566.3316049	0.568783978	0.152489	3.729992	0.000191	0.000519
12963	HIST1H3H	11.28663571	0.568655578	0.343832	1.653875	0.098153	0.148414
13960	TDRD1	1.196391499	0.568586584	0.427038	1.331465	0.183036	0.256998
14255	FAM151A	192.935958	0.568582295	0.457998	1.241453	0.214439	0.294872
4303	ZNF808	252.2525378	0.568550486	0.110281	5.155449	2.53E-07	1.15E-06
8727	CTH	81.85838014	0.568545496	0.182392	3.117169	0.001826	0.004101
2000	GLYR1	2318.478033	0.568173643	0.081668	6.957083	3.47E-12	3.40E-11
11648	PCDHGA3	17.3085298	0.56812327	0.275596	2.061438	0.039261	0.066068
4295	SPATA5	180.4646066	0.568071081	0.110108	5.159213	2.48E-07	1.13E-06
4989	NOC3L	436.0361645	0.568024362	0.118835	4.779944	1.75E-06	6.89E-06
10162	SPNS2	1509.818425	0.568001045	0.218893	2.594874	0.009463	0.018252
4464	COG6	620.9261121	0.567703232	0.112183	5.060495	4.18E-07	1.84E-06
6974	AMFR	3484.926415	0.567660976	0.147986	3.835899	0.000125	0.000352
10814	OC1002407	20.5475891	0.5674283	0.240716	2.357248	0.018411	0.033371
3826	MED23	698.4705948	0.567348423	0.104103	5.449891	5.04E-08	2.58E-07
12491	ADSSL1	1420.804415	0.567026349	0.313925	1.806247	0.07088	0.111213
6813	BMPR2	3476.848863	0.566907374	0.145581	3.894105	9.86E-05	0.000284
1649	COG3	687.4731904	0.566707545	0.076994	7.360416	1.83E-13	2.18E-12
6124	USP37	532.9160965	0.566421322	0.134543	4.209967	2.55E-05	8.17E-05
10844	TBX3	205.9922466	0.566341056	0.241733	2.342839	0.019138	0.034592

13572	CDK15	1.785337106	0.566172085	0.386892	1.463386	0.143362	0.207046
7483	DPY19L4	1098.618969	0.565917914	0.156244	3.62202	0.000292	0.000766
3018	C13orf1	387.5712399	0.565750718	0.093955	6.021493	1.73E-09	1.12E-08
6412	ANKMY2	678.4923633	0.565723157	0.138963	4.071027	4.68E-05	0.000143
5959	KIAA1033	2002.439127	0.565491295	0.131932	4.286232	1.82E-05	5.98E-05
4320	TOR1AIP1	2037.353986	0.565439484	0.109867	5.146565	2.65E-07	1.20E-06
3223	KIAA0831	532.9559533	0.565312798	0.096285	5.871268	4.32E-09	2.63E-08
8180	CBX6	594.7399887	0.564801926	0.168957	3.342865	0.000829	0.001987
5105	BCAR3	402.9012415	0.56470266	0.119476	4.726501	2.28E-06	8.77E-06
11790	LOC441454	2.605238306	0.564609054	0.278805	2.0251	0.042857	0.07125
5497	FAM45A	455.1344254	0.564110204	0.124716	4.523159	6.09E-06	2.17E-05
3949	LUZP1	1213.747561	0.563824667	0.1049	5.374882	7.66E-08	3.80E-07
13147	LOC284749	63.80923508	0.563771477	0.353286	1.595794	0.110535	0.164797
4559	SENP6	1514.303396	0.563627516	0.112617	5.004802	5.59E-07	2.40E-06
3681	C2orf60	218.6964967	0.563488975	0.101549	5.548948	2.87E-08	1.53E-07
4420	IMPACT	879.3941792	0.563487011	0.110699	5.090265	3.58E-07	1.59E-06
12340	PCDHA3	41.1784738	0.563420066	0.304326	1.851368	0.064117	0.101844
2825	TADA2B	892.7985217	0.563371168	0.091368	6.165926	7.01E-10	4.86E-09
5929	PI4KA	1609.02665	0.563338711	0.130802	4.30681	1.66E-05	5.48E-05
10046	EXOC3L2	465.051877	0.563236415	0.213682	2.635862	0.008392	0.016375
5047	ANKRD13C	625.6189421	0.563138592	0.11858	4.749016	2.04E-06	7.94E-06
6310	THNSL1	324.9963108	0.5628298	0.136589	4.120612	3.78E-05	0.000117
4645	FBXW11	1464.323167	0.562816507	0.113374	4.96424	6.90E-07	2.91E-06
14407	LRRN2	258.9000005	0.562613467	0.470441	1.195929	0.231724	0.315265
4808	KIAA1712	300.2592473	0.5622567	0.115123	4.883982	1.04E-06	4.24E-06
5845	GRHPR	1801.927347	0.562154731	0.129283	4.348234	1.37E-05	4.60E-05
4708	NCOR1	2092.876972	0.562019925	0.114038	4.92836	8.29E-07	3.45E-06
9177	PRRG4	320.6762918	0.561818361	0.190639	2.947021	0.003209	0.006853
6449	SIAE	1927.94526	0.560985091	0.138503	4.05035	5.11E-05	0.000155
1713	ZDHHC6	828.8686872	0.560975812	0.077005	7.284944	3.22E-13	3.68E-12
5176	PDS5B	793.670755	0.56088793	0.119691	4.686125	2.78E-06	1.05E-05
5466	GRPEL2	645.7121488	0.560818157	0.123651	4.535504	5.75E-06	2.06E-05
7150	SPRY3	79.38120455	0.560792117	0.148975	3.764346	0.000167	0.000458
8163	BIVM	1370.077458	0.560706458	0.167526	3.346988	0.000817	0.001962
6126	ARMC1	720.1624064	0.560653302	0.133222	4.208414	2.57E-05	8.23E-05
2425	C15orf17	1306.896105	0.560611967	0.085761	6.536907	6.28E-11	5.08E-10
6262	SLC44A1	1822.516778	0.560570035	0.135051	4.150817	3.31E-05	0.000104
12325	FAM194A	1.445231894	0.560425142	0.301757	1.857204	0.063282	0.10064
2476	FBXO7	2664.430369	0.560141447	0.086276	6.492415	8.45E-11	6.69E-10
5106	MTX3	612.5773568	0.560074794	0.118515	4.725765	2.29E-06	8.80E-06
3165	MTCH2	1968.117497	0.559722255	0.094727	5.90879	3.45E-09	2.13E-08
5357	C3orf23	654.6035651	0.559582478	0.121972	4.587809	4.48E-06	1.64E-05
6859	RGPD3	246.8205359	0.559383883	0.144257	3.877679	0.000105	0.000301
3418	PROSC	1252.14659	0.559346875	0.097539	5.734621	9.77E-09	5.60E-08
8514	HOOK2	451.0062386	0.559307049	0.17481	3.199522	0.001377	0.003169
6588	KBTBD6	383.5129466	0.559169638	0.140229	3.987556	6.68E-05	0.000199
4027	ALAD	1153.684298	0.559121728	0.10506	5.321914	1.03E-07	5.00E-07

3957	PEX12	269.6689573	0.558905752	0.104043	5.371886	7.79E-08	3.86E-07
3928	RFC1	1328.175095	0.558583599	0.103739	5.384512	7.26E-08	3.62E-07
4854	RAB5A	1170.479418	0.558565642	0.114888	4.861836	1.16E-06	4.70E-06
4450	ITCH	1528.981756	0.558543808	0.110127	5.071833	3.94E-07	1.74E-06
4059	ATXN1L	1455.373133	0.558196121	0.105287	5.30165	1.15E-07	5.54E-07
6062	LETM1	956.0880739	0.558148097	0.131675	4.238843	2.25E-05	7.26E-05
4749	ZNF44	278.976894	0.55792462	0.113644	4.909396	9.14E-07	3.77E-06
5396	ANAPC13	1177.092495	0.557883403	0.122142	4.567486	4.94E-06	1.79E-05
9335	ST3GAL1	2197.574618	0.557827104	0.193396	2.884383	0.003922	0.008235
14333	DPEP1	192.5815438	0.557800209	0.456742	1.221259	0.221988	0.303578
2481	RBM23	1110.674441	0.557699016	0.085957	6.488115	8.69E-11	6.87E-10
7472	ARMCX2	974.1241453	0.557163104	0.153589	3.627625	0.000286	0.00075
5837	NIPSNAP1	2147.529229	0.556892465	0.127913	4.353696	1.34E-05	4.50E-05
3443	FEM1A	826.7359677	0.556615745	0.097439	5.712438	1.11E-08	6.34E-08
5782	CIRH1A	1307.455678	0.556204205	0.126948	4.381346	1.18E-05	4.00E-05
6975	TNFSF13	1276.512452	0.556153852	0.144998	3.8356	0.000125	0.000352
8395	STT3B	1422.841866	0.55587707	0.171048	3.249832	0.001155	0.002696
3423	ARHGEF9	585.8042822	0.555785181	0.097058	5.726315	1.03E-08	5.88E-08
8909	PRDX2	3509.977881	0.555785028	0.182337	3.048122	0.002303	0.005066
3836	USP14	1186.441864	0.555711741	0.102112	5.442155	5.26E-08	2.69E-07
4539	STAM	569.6712842	0.555409307	0.110751	5.014927	5.31E-07	2.29E-06
4542	CEP68	785.1467755	0.555128772	0.11075	5.012456	5.37E-07	2.32E-06
15722	CXADRP3	0.528838638	0.554945417	0.695984	0.797354	0.425246	0.53013
2599	SFRS2B	1347.113537	0.554914608	0.086961	6.381166	1.76E-10	1.33E-09
10695	SH2D1B	28.43886579	0.554481702	0.231102	2.399292	0.016427	0.030107
9381	CCDC89	70.90192643	0.55447757	0.193426	2.866611	0.004149	0.008669
14210	CACNA1S	1.310442715	0.55436517	0.442188	1.253687	0.209956	0.289609
13101	ALPL	1816.086734	0.554302668	0.34409	1.610925	0.107196	0.160381
14919	LOC38933[illegible]	201.4273461	0.554124592	0.537665	1.030613	0.302722	0.397725
5091	OPA3	753.2397777	0.554073888	0.117147	4.729718	2.25E-06	8.65E-06
2775	AQR	895.0176948	0.554002804	0.089131	6.215637	5.11E-10	3.61E-09
11718	RIMKLA	650.4259164	0.55394339	0.270616	2.046974	0.040661	0.068014
6236	RB1CC1	1488.298224	0.553869739	0.133102	4.161254	3.17E-05	9.95E-05
12786	BACE2	2714.69048	0.553756169	0.324167	1.708244	0.087591	0.134278
4985	ZXDA	81.99447978	0.553668063	0.11578	4.782052	1.74E-06	6.82E-06
2498	USP30	476.3732252	0.553483696	0.08563	6.463702	1.02E-10	8.02E-10
2953	UTP3	721.4235293	0.553423434	0.091192	6.06874	1.29E-09	8.56E-09
9599	NEDD9	2711.689596	0.553415066	0.199076	2.779913	0.005437	0.011103
4514	PDCL	495.3741428	0.553342369	0.110054	5.027925	4.96E-07	2.15E-06
12809	LOC39132[illegible]	8.105025367	0.55296823	0.324907	1.701929	0.088769	0.135838
12079	FNDC5	16.20723713	0.552942996	0.285803	1.934698	0.053027	0.086049
8732	ESRRA	1438.748101	0.552914488	0.177471	3.115519	0.001836	0.004121
4605	MTRF1	143.8934369	0.55276755	0.110836	4.98726	6.12E-07	2.61E-06
2939	ARMCX5	279.8433751	0.552535083	0.090906	6.078083	1.22E-09	8.11E-09
5866	ZNF675	121.586198	0.552528356	0.127393	4.337206	1.44E-05	4.82E-05
8234	CNKSR3	888.05011	0.552180225	0.16644	3.317594	0.000908	0.002161
7673	C14orf147	932.3310549	0.552171814	0.155545	3.549914	0.000385	0.000984

1563	MTFMT	219.8415127	0.552067934	0.07385	7.47551	7.69E-14	9.64E-13
4266	SNX2	2031.071508	0.552040453	0.106705	5.17352	2.30E-07	1.06E-06
3891	NDUFS2	2175.123406	0.55161324	0.101996	5.408191	6.37E-08	3.21E-07
3309	RDH14	466.9744572	0.551595197	0.094924	5.810908	6.21E-09	3.68E-08
7560	SGTB	369.7281844	0.551302055	0.153476	3.59211	0.000328	0.00085
3101	ZNF451	650.6306248	0.55123099	0.092446	5.962716	2.48E-09	1.57E-08
10131	CCDC39	17.09531492	0.550859488	0.211464	2.604979	0.009188	0.017777
16830	CCDC33	0.659798639	0.550718536	1.139255	0.483402	0.62881	0.732341
14135	IP6K3	135.0908172	0.550410474	0.430911	1.277318	0.20149	0.279406
4107	KRR1	685.3212306	0.550282986	0.104255	5.278233	1.30E-07	6.23E-07
2334	RNF146	695.7925364	0.550203113	0.083113	6.619961	3.59E-11	3.02E-10
6493	MAP3K1	920.7354908	0.550182962	0.136481	4.031202	5.55E-05	0.000167
4025	UFM1	2303.233731	0.54999007	0.103279	5.325309	1.01E-07	4.91E-07
5589	RNF5	1564.875791	0.549831758	0.122743	4.47952	7.48E-06	2.62E-05
8362	REP15	31.8813715	0.549640305	0.168266	3.266506	0.001089	0.002552
12305	FOXQ1	158.7929619	0.549584223	0.294894	1.863669	0.062368	0.099348
8634	CHAC2	31.15710284	0.549564339	0.173919	3.159889	0.001578	0.003583
3627	COMMD2	849.0322909	0.549316119	0.09831	5.587603	2.30E-08	1.24E-07
13072	CCL15	121.2489999	0.549269299	0.338652	1.621927	0.104819	0.157172
11733	TXK	42.88725861	0.549248396	0.268929	2.042352	0.041117	0.068689
7482	WHAMML1	190.8766369	0.548746294	0.15148	3.62256	0.000292	0.000764
9407	GTF2A1	186.1439384	0.548693325	0.192148	2.855575	0.004296	0.008951
12744	FAM86B2	9.322824436	0.548649716	0.319118	1.719272	0.085565	0.131604
10398	HNF1B	2380.083179	0.548594585	0.218731	2.508075	0.012139	0.022882
2707	MAP2K4	778.8666541	0.54854751	0.087428	6.274241	3.51E-10	2.54E-09
4426	ZMYM1	232.1321895	0.548491564	0.107825	5.086853	3.64E-07	1.61E-06
11296	PALM2	75.07488167	0.548481434	0.251685	2.179237	0.029314	0.050866
6746	ZNF596	98.58639704	0.548348881	0.13983	3.921552	8.80E-05	0.000256
7245	GNAI1	981.4954261	0.548306283	0.147086	3.727791	0.000193	0.000523
3814	TRIM33	1071.381098	0.548220436	0.100327	5.464344	4.65E-08	2.39E-07
5858	PRDM4	1110.768961	0.548175591	0.126256	4.341765	1.41E-05	4.73E-05
16464	MYH1	0.507633059	0.547718383	0.93939	0.583058	0.559854	0.666527
7765	PHF17	1934.017331	0.547644259	0.155954	3.511579	0.000445	0.001124
6760	HMGCR	804.0493623	0.547393919	0.139793	3.91575	9.01E-05	0.000261
5292	SFRS14	1191.450971	0.54726267	0.118322	4.625205	3.74E-06	1.39E-05
3211	FUBP3	1109.903382	0.546889777	0.093022	5.879113	4.12E-09	2.52E-08
4240	VDAC3	1782.699967	0.546741148	0.10542	5.186298	2.15E-07	9.92E-07
4812	TTC12	237.0170453	0.546623481	0.111952	4.882648	1.05E-06	4.26E-06
5653	TTC37	1845.320851	0.546585206	0.122936	4.446102	8.74E-06	3.03E-05
4412	ZNF780A	294.2048804	0.546574672	0.107294	5.0942	3.50E-07	1.56E-06
10593	C3orf15	180.518779	0.546486144	0.224114	2.438427	0.014751	0.027295
10416	ACSM3	680.1161694	0.54627878	0.218065	2.505114	0.012241	0.023036
5764	CCBL2	442.9924346	0.54616041	0.124438	4.389006	1.14E-05	3.87E-05
13899	GDPD4	2.120996539	0.545900282	0.404581	1.349299	0.177241	0.249953
8073	CCNI	5928.876348	0.545789163	0.161317	3.383329	0.000716	0.001739
8472	CYP2U1	397.4670378	0.545634037	0.16953	3.218511	0.001289	0.002981
6481	GALNT4	635.8548228	0.545296183	0.135099	4.036268	5.43E-05	0.000164

8023	RGL1	1774.968939	0.544965907	0.160188	3.402036	0.000669	0.001634
5156	C1orf55	660.9513247	0.54470518	0.115932	4.698495	2.62E-06	9.96E-06
11626	OCLM	4.476465574	0.544310368	0.263	2.06962	0.038488	0.064889
13852	PRODH2	381.4163526	0.544094802	0.397765	1.367881	0.171349	0.242464
692	TRIP4	561.0256421	0.543937655	0.0602	9.035487	1.63E-19	4.62E-18
6002	CBLL1	756.5691592	0.543894065	0.127508	4.265554	1.99E-05	6.51E-05
5053	ZC3H13	1333.611209	0.543836948	0.114611	4.745088	2.08E-06	8.08E-06
11049	DNAJB7	2.996381223	0.54351462	0.239061	2.273538	0.022994	0.040791
6368	ZNF550	195.5552369	0.543459017	0.132728	4.094527	4.23E-05	0.00013
6082	ZNF518A	652.2242614	0.543358803	0.128516	4.227954	2.36E-05	7.60E-05
10109	ZFP37	82.58056704	0.54295183	0.207723	2.613824	0.008954	0.017361
6492	C14orf21	225.5597993	0.542917341	0.134678	4.031218	5.55E-05	0.000167
8695	STARD4	203.2667286	0.542867267	0.173332	3.131945	0.001737	0.003915
10016	FLJ35390	132.2346396	0.542771222	0.205099	2.646392	0.008136	0.015921
9482	PALMD	523.2815452	0.542688815	0.192066	2.825528	0.00472	0.009758
5936	ATP6V1E1	3375.956288	0.542538586	0.126177	4.299822	1.71E-05	5.64E-05
6304	CCPG1	3465.3047	0.542486238	0.131527	4.124512	3.72E-05	0.000116
5434	SFRS2IP	2998.004051	0.542438629	0.119189	4.551066	5.34E-06	1.93E-05
6206	SLC20A2	563.5131028	0.542201985	0.129892	4.174249	2.99E-05	9.44E-05
5428	KIAA0649	674.905796	0.542021695	0.119039	4.553316	5.28E-06	1.91E-05
13588	TNNC1	4.890515956	0.541942018	0.371583	1.458469	0.144711	0.20875
5144	UBR2	804.5570963	0.541766799	0.115134	4.705528	2.53E-06	9.65E-06
14234	COLEC12	309.4487402	0.541756294	0.434183	1.24776	0.212119	0.2921
7709	FLVCR1	121.0726412	0.541553735	0.153136	3.536426	0.000406	0.001031
5661	PIGW	175.5355402	0.541283644	0.121851	4.442183	8.91E-06	3.08E-05
7523	NEK7	1752.397855	0.541184735	0.150048	3.606756	0.00031	0.000808
4325	NFE2L2	3295.081881	0.540769841	0.105133	5.143655	2.69E-07	1.22E-06
7257	SESN1	756.6234521	0.540602424	0.145221	3.722608	0.000197	0.000533
12627	TMC1	1.492261823	0.540432957	0.306217	1.764869	0.077586	0.120437
5827	C14orf126	226.9884539	0.540414065	0.123988	4.358607	1.31E-05	4.40E-05
5088	CYTSA	1366.172381	0.540407746	0.114252	4.729947	2.25E-06	8.65E-06
3984	PSEN1	1858.20283	0.540360898	0.100965	5.351956	8.70E-08	4.28E-07
11224	CLDN4	2130.872596	0.540304194	0.244319	2.211474	0.027003	0.047157
12083	PZP	2.695738004	0.540236591	0.279536	1.932619	0.053283	0.086436
3865	ATXN3	352.7934922	0.539979731	0.099536	5.424956	5.80E-08	2.94E-07
11276	MGAT5	270.9767004	0.539939534	0.246772	2.188014	0.028669	0.049834
4966	C11orf46	613.2601381	0.539849781	0.112708	4.789788	1.67E-06	6.59E-06
7478	PAWR	224.1544415	0.539836123	0.148988	3.623364	0.000291	0.000762
4421	KIAA1217	1848.950386	0.539685667	0.106038	5.08955	3.59E-07	1.59E-06
3862	API5	2133.841153	0.539399406	0.099407	5.426174	5.76E-08	2.92E-07
7353	FLJ10357	1006.979441	0.539358291	0.14663	3.678367	0.000235	0.000626
8647	CHKA	404.4392086	0.53925552	0.17107	3.152251	0.00162	0.003673
6767	AGAP1	432.0589797	0.539220091	0.137854	3.911533	9.17E-05	0.000266
4400	PPIG	1025.57301	0.539026005	0.105635	5.102708	3.35E-07	1.49E-06
5768	TOP2B	2109.838024	0.538736486	0.122769	4.388202	1.14E-05	3.88E-05
11200	HIST1H3E	40.34690748	0.538607551	0.242467	2.221365	0.026326	0.046073
6793	GGA2	2452.11132	0.538497683	0.137977	3.902819	9.51E-05	0.000274

1503	GLOD4	1292.155429	0.538482799	0.071229	7.559903	4.03E-14	5.26E-13
6333	UBE2Q2	1054.582145	0.538301407	0.130925	4.111533	3.93E-05	0.000122
3528	ATP6V1G1	2238.527362	0.538073171	0.095211	5.651396	1.59E-08	8.84E-08
2762	DDX5	10468.19551	0.538025338	0.086405	6.226754	4.76E-10	3.38E-09
12239	ARC	42.64792414	0.537985637	0.285373	1.885203	0.059402	0.095134
12613	FLRT1	8.466374901	0.537894043	0.304341	1.767406	0.07716	0.1199
3630	ZNF234	175.3519379	0.537348669	0.096217	5.584745	2.34E-08	1.26E-07
9638	LASS4	946.3169441	0.537333614	0.194238	2.766363	0.005669	0.011528
7332	ASAP2	1257.071649	0.536925571	0.145596	3.687788	0.000226	0.000605
5514	RAB9A	571.9105699	0.53691438	0.118953	4.513661	6.37E-06	2.27E-05
4818	TEAD1	2211.253839	0.536668296	0.109966	4.88033	1.06E-06	4.31E-06
4544	BCLAF1	2736.404914	0.536290869	0.107005	5.011838	5.39E-07	2.33E-06
4717	NUP160	1237.379205	0.536249713	0.108918	4.923432	8.50E-07	3.53E-06
10873	RAP1GAP2	332.4108942	0.536103445	0.229929	2.331603	0.019722	0.035553
8117	SPEF2	285.5777862	0.53605809	0.159379	3.363422	0.00077	0.001859
3898	TMEM128	604.4881577	0.536001353	0.099171	5.40482	6.49E-08	3.26E-07
8655	RRM2B	2051.344277	0.535634668	0.170125	3.148486	0.001641	0.003717
7248	TM9SF2	3961.723499	0.535586948	0.143738	3.726123	0.000194	0.000526
5960	AKAP10	486.1497649	0.535525076	0.124951	4.285883	1.82E-05	5.98E-05
11685	SCN4B	685.2631907	0.53548144	0.260562	2.055102	0.039869	0.066879
7143	RSRC1	218.5044542	0.535449092	0.142175	3.766124	0.000166	0.000455
11175	LOC641298	79.66272644	0.535411312	0.240029	2.23061	0.025707	0.04509
6503	KIAA1671	2042.478501	0.534915707	0.132867	4.025942	5.67E-05	0.000171
4496	KIAA0586	251.8045508	0.534637996	0.106066	5.040609	4.64E-07	2.02E-06
8460	AGPAT6	1963.011298	0.53457136	0.165854	3.223136	0.001268	0.002938
5880	C1orf9	1117.491766	0.534510923	0.123349	4.333328	1.47E-05	4.90E-05
9530	LOC286367	45.84031752	0.534441935	0.190431	2.806481	0.005009	0.010301
13353	LGALS2	882.7613897	0.534398803	0.349635	1.52845	0.126401	0.185545
4223	TUBGCP4	249.4664096	0.534209559	0.102783	5.19746	2.02E-07	9.38E-07
5886	KIF3B	2748.4348	0.533850232	0.123294	4.329912	1.49E-05	4.97E-05
7198	ALG10	106.8411764	0.533763253	0.142441	3.74725	0.000179	0.000487
6810	ZNF554	114.8354537	0.533653638	0.136988	3.895632	9.79E-05	0.000282
4052	TOB2	2035.923004	0.533522453	0.100558	5.305611	1.12E-07	5.43E-07
4009	RCOR3	877.9380937	0.53339524	0.099978	5.33515	9.55E-08	4.67E-07
3764	XIAP	1519.584976	0.53310373	0.096938	5.499402	3.81E-08	1.98E-07
11376	C10orf114	67.28599819	0.532923386	0.246743	2.159832	0.030786	0.053044
5063	SPTLC1	1727.775507	0.532864625	0.112427	4.739662	2.14E-06	8.29E-06
10158	SPTB	93.62088855	0.532764125	0.205177	2.596613	0.009415	0.018167
8619	LOC147727	491.1825729	0.532651972	0.16813	3.168092	0.001534	0.00349
3684	SNX27	1459.198301	0.532591588	0.096003	5.547648	2.90E-08	1.54E-07
5140	SMARCA5	1787.183537	0.532426015	0.11308	4.708406	2.50E-06	9.52E-06
13769	C8orf85	14.87353517	0.532412426	0.38126	1.396453	0.162578	0.231455
6876	CNIH	1392.233029	0.531993254	0.137372	3.872645	0.000108	0.000307
14753	CIB4	8.036766252	0.531921333	0.491425	1.082405	0.279072	0.370779
6662	SLC31A1	1135.612958	0.531732548	0.134386	3.956768	7.60E-05	0.000224
5159	AEBP2	933.2342081	0.531689665	0.113238	4.695347	2.66E-06	1.01E-05
8496	FAM60A	1338.492194	0.531664379	0.16575	3.207632	0.001338	0.003088

9869	CLEC1A	161.9146209	0.531606121	0.197048	2.697845	0.006979	0.013861
8560	DCK	626.5442234	0.531426881	0.166782	3.186364	0.001441	0.003299
12028	PHGDH	645.3470598	0.531247662	0.271902	1.953822	0.050722	0.082658
6233	BBS10	534.5486339	0.530824445	0.12751	4.163015	3.14E-05	9.88E-05
4823	PCM1	2341.464596	0.530818502	0.108805	4.878617	1.07E-06	4.34E-06
11388	AS3MT	516.7147618	0.530511965	0.246117	2.155525	0.031121	0.053563
7100	ARNTL	306.4111074	0.530487601	0.140234	3.782886	0.000155	0.000428
8818	MRPL34	817.0940235	0.530392876	0.172077	3.082293	0.002054	0.004566
9717	PPP1R12B	1425.373802	0.530360936	0.193339	2.743168	0.006085	0.012274
6037	FAM18B	602.2440058	0.530289854	0.124776	4.249922	2.14E-05	6.94E-05
2804	MED17	573.4025175	0.529857262	0.08561	6.189214	6.05E-10	4.23E-09
5878	CNN3	6796.65779	0.529673129	0.122213	4.334026	1.46E-05	4.88E-05
2223	RIC8B	478.3479328	0.529379468	0.078661	6.729914	1.70E-11	1.50E-10
5795	NUS1	1619.643421	0.529016119	0.120887	4.376109	1.21E-05	4.09E-05
3601	NCBP1	704.689235	0.528724321	0.094334	5.604809	2.08E-08	1.13E-07
7425	HLTF	953.7792822	0.528683748	0.144973	3.646765	0.000266	0.000701
6706	TUSC1	508.2168204	0.528675287	0.134205	3.939304	8.17E-05	0.000239
3839	DIS3	982.2264655	0.528593519	0.097146	5.44122	5.29E-08	2.70E-07
3728	MECP2	1601.340218	0.52858766	0.095735	5.52135	3.36E-08	1.77E-07
14132	GATA5	4.734609665	0.528539115	0.413229	1.279045	0.200881	0.278621
3964	FAM120A	4639.593211	0.528430946	0.098479	5.365948	8.05E-08	3.98E-07
7271	NUDT6	92.61014869	0.528352339	0.142262	3.713946	0.000204	0.00055
12595	TOMM20L	1.505454594	0.528316945	0.29766	1.774903	0.075914	0.118141
5048	MRRF	527.1909782	0.527909514	0.111171	4.748618	2.05E-06	7.95E-06
9738	STOX2	69.10295602	0.52788078	0.192874	2.736919	0.006202	0.012483
4990	ZNF567	122.627706	0.527875244	0.110473	4.778314	1.77E-06	6.94E-06
5374	MBTD1	451.2766678	0.527796823	0.115234	4.580232	4.64E-06	1.69E-05
5973	ZNF211	221.1652109	0.527704984	0.123273	4.280798	1.86E-05	6.11E-05
7846	ZBTB41	771.2554585	0.527610198	0.15182	3.475236	0.00051	0.001275
14109	GSTT1	1021.15911	0.52760018	0.410715	1.284591	0.198935	0.276372
3166	FGFR1OP2	644.9909975	0.527551897	0.08929	5.908318	3.46E-09	2.14E-08
13047	TSSK1B	1.799727682	0.527540904	0.323387	1.631298	0.102828	0.154482
8253	FER	76.07964043	0.527427485	0.159216	3.312658	0.000924	0.002195
6862	GXYLT1	865.3788004	0.527418575	0.136038	3.877007	0.000106	0.000302
9210	GLTP	1739.088641	0.527390369	0.179715	2.934587	0.00334	0.007108
4767	ZNF238	697.6267687	0.527278744	0.107602	4.900276	9.57E-07	3.94E-06
4347	BCL2L13	1768.635037	0.527278192	0.102717	5.133328	2.85E-07	1.28E-06
11513	PCDH19	19.01864334	0.527123327	0.249592	2.111944	0.034691	0.059062
10391	HGD	1317.627979	0.527103554	0.209991	2.51012	0.012069	0.022766
3226	PAFAH1B1	3295.42199	0.527065521	0.089809	5.868713	4.39E-09	2.67E-08
4940	UNC119B	1766.167649	0.526990424	0.109689	4.804415	1.55E-06	6.16E-06
4778	FASTKD3	218.4976146	0.526939936	0.107631	4.895812	9.79E-07	4.02E-06
8501	SLC16A14	137.3242315	0.526917583	0.164358	3.20592	0.001346	0.003104
11809	TMEFF1	120.9257392	0.526779042	0.261001	2.018306	0.043559	0.072302
11692	PLCB1	1202.359896	0.526650332	0.256504	2.053189	0.040054	0.067149
11965	LYNX1	314.3484838	0.526494995	0.26725	1.970045	0.048833	0.079998
5449	ARV1	409.4392076	0.526482292	0.115907	4.542266	5.57E-06	2.00E-05

15003	C3orf49	0.344282801	0.526417737	0.522974	1.006586	0.314134	0.410407
15674	TAS2R50	0.285160242	0.5264175	0.646328	0.814475	0.415373	0.519442
16193	PIRT	0.235388199	0.526417126	0.803088	0.655491	0.512152	0.61994
16297	C1orf180	0.317490726	0.52641712	0.837864	0.628285	0.529817	0.637231
16902	OR2T3	0.137836319	0.526415958	1.133326	0.464488	0.642298	0.744864
17149	TMEM211	0.12254552	0.526415132	1.321121	0.398461	0.69029	0.78899
17420	HIST1H1A	0.178522331	0.52641416	1.561065	0.337215	0.735955	0.828097
17512	PSG10	0.265668461	0.526413844	1.683023	0.312779	0.754449	0.844447
17922	SEMG1	0.158855504	0.526409003	2.348179	0.224178	0.822619	0.899685
18042	FAM74A4	0.161509895	0.526406396	2.662853	0.197685	0.843291	0.916041
18078	CNGA2	0.123727048	0.526405148	2.753446	0.19118	0.848384	0.919857
18116	OR51G2	0.079652997	0.5264031	2.91407	0.180642	0.856649	0.92687
8870	ZSCAN18	1076.529817	0.526338321	0.171824	3.063244	0.00219	0.004838
9401	UBXN10	246.5456739	0.526259484	0.184095	2.858625	0.004255	0.008871
13091	SLC24A5	17.40434969	0.526133601	0.326018	1.61382	0.106566	0.159549
11192	NHSL2	60.05954226	0.526093431	0.236507	2.224435	0.026119	0.045744
4904	TARSL2	404.22407	0.526037854	0.10903	4.824709	1.40E-06	5.60E-06
7547	ZKSCAN1	767.9150094	0.525909814	0.146258	3.595757	0.000323	0.00084
5895	TMEM9B	1454.158667	0.525638094	0.121576	4.323519	1.54E-05	5.11E-05
12141	DYNLRB2	20.84418831	0.525562231	0.274398	1.91533	0.05545	0.089522
13999	LOC28400	0.967514512	0.525520836	0.398598	1.318424	0.187362	0.262345
9854	DDX60	1065.875873	0.525405925	0.194501	2.701295	0.006907	0.013737
9179	C17orf91	265.4964171	0.525133305	0.178209	2.946728	0.003212	0.006858
4480	GABPB2	87.50088586	0.525075047	0.10392	5.05267	4.36E-07	1.91E-06
4998	TIMM17A	1026.326906	0.524747374	0.109898	4.774844	1.80E-06	7.05E-06
12877	QPRT	1305.484582	0.524268764	0.311606	1.682473	0.092477	0.140774
4654	ZFYVE21	805.4068141	0.524133591	0.105685	4.959384	7.07E-07	2.98E-06
14304	DMRTC1B	6.883229204	0.524034166	0.426696	1.228119	0.219402	0.30065
4274	DHX36	848.7852541	0.523988064	0.10136	5.169594	2.35E-07	1.08E-06
6337	TMEM136	259.2556583	0.523833099	0.127452	4.110058	3.96E-05	0.000122
3766	MFN2	2309.182688	0.523608738	0.095222	5.498837	3.82E-08	1.99E-07
11449	LRRC48	126.7779549	0.523515163	0.24523	2.134796	0.032778	0.056116
12793	GALNT3	110.7701667	0.522994523	0.306505	1.706317	0.087949	0.134752
8841	PTPRJ	1838.545493	0.522935735	0.170102	3.074247	0.00211	0.004679
5499	C2orf3	512.0295163	0.522921202	0.115641	4.521941	6.13E-06	2.18E-05
9022	LPIN1	591.1645541	0.522890737	0.174048	3.004285	0.002662	0.005784
9649	MBOAT2	249.3371441	0.522771926	0.189166	2.763568	0.005717	0.011615
12345	LOC14869	5.576517111	0.522586127	0.282444	1.850229	0.06428	0.102069
4144	PIGM	401.0971783	0.522549029	0.099446	5.254615	1.48E-07	7.02E-07
15624	TBC1D29	0.385102128	0.522535272	0.630954	0.828168	0.407576	0.511322
8671	PLXNA2	1283.619991	0.522380662	0.166218	3.14275	0.001674	0.003783
4757	PHAX	524.5388172	0.522311068	0.106465	4.905948	9.30E-07	3.83E-06
3196	DCAF6	1780.076372	0.522185364	0.088731	5.885049	3.98E-09	2.44E-08
6718	COG8	504.6280941	0.522158841	0.132752	3.933347	8.38E-05	0.000244
4176	TATDN3	379.9785099	0.522095927	0.099824	5.23017	1.69E-07	7.95E-07
4640	SHPK	383.5254542	0.522051155	0.105057	4.969215	6.72E-07	2.84E-06
5817	LOC64797	9445.948422	0.521896281	0.119553	4.365381	1.27E-05	4.28E-05

4086	RALGAPB	1749.217688	0.521895914	0.098671	5.289228	1.23E-07	5.89E-07
14270	LOC25455(	148.3037483	0.521784739	0.42186	1.236868	0.216136	0.29688
8685	SDHA	5896.624827	0.521728172	0.166321	3.136879	0.001708	0.003854
3265	C17orf42	168.5701385	0.521478931	0.089313	5.838811	5.26E-09	3.16E-08
3498	ZNF543	109.3415966	0.521312261	0.091831	5.676839	1.37E-08	7.69E-08
4331	XPNPEP3	216.9910864	0.52091121	0.101322	5.141135	2.73E-07	1.24E-06
7104	GSTCD	218.3503329	0.52084055	0.13772	3.781891	0.000156	0.000429
12556	KLRF1	26.43940976	0.52061004	0.29126	1.787441	0.073866	0.115312
12597	KCNK10	8.404786881	0.520299107	0.293231	1.774365	0.076003	0.118261
11803	KAAG1	9.066509695	0.520090633	0.257432	2.020302	0.043352	0.071994
3716	LOC33929(	604.9141971	0.520030855	0.09407	5.528115	3.24E-08	1.71E-07
5939	ELP4	289.2359613	0.520003138	0.120963	4.298876	1.72E-05	5.67E-05
5359	CLCN6	537.3307809	0.519885904	0.113335	4.587149	4.49E-06	1.64E-05
4476	CWC22	662.2794975	0.519683415	0.102811	5.054757	4.31E-07	1.89E-06
5641	TMEM41B	874.7449623	0.519552016	0.116703	4.451899	8.51E-06	2.96E-05
12849	C14orf73	166.9498046	0.519178001	0.30706	1.690805	0.090874	0.138606
15097	VSNL1	7.520026257	0.5190299	0.531217	0.977059	0.32854	0.426556
11318	ZNF662	80.73599975	0.51899694	0.238745	2.173857	0.029716	0.051463
7854	RB1	2181.787513	0.518493877	0.149426	3.469908	0.000521	0.001299
8584	INTU	113.5166029	0.51822929	0.163031	3.17872	0.001479	0.003378
7731	REEP3	623.4063821	0.517985678	0.146819	3.52805	0.000419	0.001061
9014	KIAA0895	311.3852977	0.51793166	0.172212	3.007522	0.002634	0.005727
14071	CNTD2	3.832091657	0.51690968	0.398796	1.296177	0.194914	0.271517
3007	NFX1	1017.208405	0.516878094	0.085732	6.029006	1.65E-09	1.08E-08
3033	MTERFD2	340.852264	0.516877244	0.086016	6.009096	1.87E-09	1.21E-08
5908	MAD2L1BI	398.988903	0.516799056	0.119667	4.318638	1.57E-05	5.21E-05
5592	PET112L	564.5355401	0.51677766	0.115461	4.475786	7.61E-06	2.67E-05
10566	FILIP1	272.008067	0.516754809	0.211121	2.447666	0.014378	0.026674
7228	C6orf203	247.7252863	0.516742927	0.138338	3.735378	0.000187	0.000508
12268	FGF1	145.1548302	0.516567937	0.275063	1.878001	0.060381	0.096465
9209	SGK1	3555.455037	0.516381598	0.17593	2.93516	0.003334	0.007096
6880	ROCK1	1494.167769	0.516152317	0.133313	3.871719	0.000108	0.000308
9393	KLHL21	1174.683672	0.515819151	0.180257	2.86158	0.004215	0.008796
1764	ASB7	443.7004317	0.515651636	0.071347	7.227406	4.92E-13	5.47E-12
5701	MRPL1	411.4106642	0.515627943	0.116654	4.420154	9.86E-06	3.39E-05
5303	43896	2534.220413	0.51540719	0.111563	4.619896	3.84E-06	1.42E-05
1645	RUNDC1	503.9509471	0.51512916	0.069917	7.367718	1.74E-13	2.07E-12
11056	KANK3	273.417304	0.514930805	0.226632	2.272104	0.02308	0.040919
15196	ZMAT4	47.87822083	0.514822851	0.542457	0.949058	0.342591	0.441901
11115	POU3F3	353.3677221	0.514797756	0.229107	2.246976	0.024642	0.043455
10529	RAB11FIP1	1634.32589	0.51463334	0.209177	2.460282	0.013883	0.025844
2635	RBM16	1038.04703	0.514523616	0.081152	6.340223	2.29E-10	1.71E-09
9918	DCPS	414.4249689	0.514129678	0.191763	2.681069	0.007339	0.014504
7244	ZFAT	388.8926069	0.514062837	0.13788	3.728342	0.000193	0.000522
8966	STRADB	782.3624031	0.513833235	0.169907	3.024199	0.002493	0.00545
3505	DDX10	543.9983383	0.513707623	0.090622	5.668659	1.44E-08	8.05E-08
4566	USP16	878.4285643	0.513646835	0.102696	5.001604	5.69E-07	2.44E-06

5726	FXN	184.0253163	0.513628022	0.116513	4.408319	1.04E-05	3.57E-05
13207	KHK	3581.235722	0.513403062	0.325465	1.577445	0.114693	0.170217
7293	GLCE	796.595772	0.513366685	0.138698	3.701316	0.000214	0.000576
15083	CRNA002(	0.747511785	0.513222503	0.52305	0.981211	0.326489	0.424286
7755	TOR1AIP2	436.1531674	0.513001897	0.14588	3.5166	0.000437	0.001105
10852	LPL	766.146373	0.512778304	0.219242	2.338865	0.019342	0.034937
12040	C15orf52	336.0113971	0.512758171	0.263264	1.947692	0.051452	0.083763
4056	MRPS18C	291.2239865	0.51274221	0.096708	5.301947	1.15E-07	5.54E-07
9264	BRWD3	231.1758473	0.512446964	0.175993	2.911752	0.003594	0.007604
10288	BNIP3	5498.896766	0.512438861	0.200952	2.550053	0.010771	0.020519
10227	SPDYE6	27.19435961	0.512428335	0.199179	2.572705	0.010091	0.01934
6235	TROVE2	1213.154998	0.512276228	0.123096	4.161584	3.16E-05	9.94E-05
5559	QRSL1	474.8132476	0.512020295	0.113966	4.492727	7.03E-06	2.48E-05
10772	DDI2	76.47888539	0.511749896	0.215545	2.374209	0.017587	0.031998
6000	MAT2A	5045.198128	0.511745249	0.11992	4.267401	1.98E-05	6.46E-05
6261	ASF1A	352.5271964	0.511745167	0.123289	4.150782	3.31E-05	0.000104
13730	FAM66A	1.679887173	0.511562156	0.363558	1.407101	0.159397	0.22756
5184	NDUFAF1	360.1559452	0.511560114	0.109231	4.683297	2.82E-06	1.07E-05
5998	USP19	963.0696075	0.511383801	0.119812	4.268207	1.97E-05	6.44E-05
11493	SYPL2	202.7861247	0.511274049	0.24088	2.122526	0.033794	0.057634
4108	MON2	1265.084931	0.511021094	0.096855	5.276146	1.32E-07	6.29E-07
5060	DCAF11	1464.817678	0.510850181	0.107726	4.742104	2.12E-06	8.19E-06
11140	CN5H6.4	10.12737858	0.510830396	0.228066	2.239834	0.025102	0.044163
5928	CYB5D2	564.7030986	0.510542213	0.118535	4.307112	1.65E-05	5.47E-05
8952	ZNF439	134.7549087	0.510540786	0.168434	3.031112	0.002437	0.005335
4117	PAPOLA	3578.348575	0.510453512	0.096824	5.271981	1.35E-07	6.43E-07
11265	DBNDD1	402.0288727	0.510409767	0.232741	2.193037	0.028305	0.049246
5131	MCPH1	388.9441238	0.510387949	0.108276	4.713778	2.43E-06	9.29E-06
5446	UCHL5	415.4205368	0.51024286	0.112284	4.54423	5.51E-06	1.98E-05
6324	GPD2	887.5279544	0.510175164	0.123971	4.115282	3.87E-05	0.00012
2397	ZNF45	265.8436904	0.509820559	0.077708	6.560742	5.35E-11	4.38E-10
12240	BICC1	790.0995535	0.509645825	0.270365	1.885027	0.059426	0.095158
12074	ZNF354C	48.72231483	0.509588375	0.263245	1.935796	0.052893	0.085866
8260	C9orf7	566.7896126	0.509541878	0.154005	3.308598	0.000938	0.002225
6438	CLINT1	2679.849076	0.509540057	0.125678	4.054327	5.03E-05	0.000153
3678	THUMPD1	1391.210825	0.509506264	0.091765	5.552264	2.82E-08	1.50E-07
3800	ZNF507	544.7273439	0.509416772	0.093053	5.474489	4.39E-08	2.26E-07
3232	RAB11A	3147.451974	0.50937126	0.086866	5.863852	4.52E-09	2.74E-08
7338	ZNF430	104.3888946	0.509188277	0.138175	3.685099	0.000229	0.000611
4484	WDR48	811.1377718	0.508992094	0.100818	5.048626	4.45E-07	1.95E-06
9510	EPAS1	15045.81587	0.508625338	0.180606	2.816216	0.004859	0.010015
5413	MKL2	1121.628547	0.508424889	0.111502	4.559768	5.12E-06	1.85E-05
10891	)C1001297	10.59505327	0.508415039	0.218845	2.323175	0.02017	0.0363
4201	LYRM2	792.2049184	0.50824876	0.097589	5.208037	1.91E-07	8.90E-07
6252	ZFYVE16	1202.887776	0.508161683	0.122339	4.15371	3.27E-05	0.000103
7137	NEDD1	526.4885329	0.508105187	0.1348	3.769333	0.000164	0.00045
5670	METTL10	280.4163457	0.507956696	0.114493	4.436592	9.14E-06	3.16E-05

12091	CNTNAP2	8.349758966	0.507783226	0.262904	1.93144	0.053429	0.086614
8622	IGSF3	1012.659378	0.50761467	0.160308	3.166497	0.001543	0.003508
4973	EP300	2298.334696	0.507576862	0.106013	4.787864	1.69E-06	6.64E-06
5290	PIBF1	353.7482842	0.507377668	0.109653	4.627128	3.71E-06	1.37E-05
8132	SLC25A23	2578.038546	0.507282456	0.151001	3.359453	0.000781	0.001882
4112	ATP5F1	3086.993608	0.507194448	0.096165	5.274231	1.33E-07	6.35E-07
6597	MCM9	57.14426452	0.507031498	0.127253	3.984447	6.76E-05	0.000201
4576	RNF111	784.9160424	0.507024595	0.101453	4.997622	5.80E-07	2.49E-06
6596	NAB1	1510.641734	0.50675939	0.127173	3.984809	6.75E-05	0.000201
15141	TMEM74	3.167797921	0.506450394	0.526444	0.962021	0.336039	0.434995
7874	PIK3CA	528.7149028	0.506368323	0.146259	3.462138	0.000536	0.001334
3673	MATR3	6482.59169	0.506315223	0.091156	5.554406	2.79E-08	1.49E-07
15740	FOXN1	0.656393253	0.506253528	0.638963	0.792305	0.428183	0.533216
7337	FAM174A	561.8134498	0.506216749	0.137333	3.686046	0.000228	0.000608
9937	RAPGEF6	377.2305692	0.506176265	0.189262	2.674469	0.007485	0.014764
5299	RETSAT	2013.810927	0.506155761	0.109501	4.622383	3.79E-06	1.40E-05
9841	MORF4	3.304307889	0.505770052	0.186886	2.706297	0.006804	0.013552
8379	ILVBL	1739.017219	0.50570803	0.155206	3.258308	0.001121	0.002622
11575	SLC9A7	63.9756469	0.505611343	0.241912	2.090065	0.036612	0.061998
6620	RNF14	1876.313337	0.505528469	0.12715	3.975842	7.01E-05	0.000208
14338	MYH14	844.7380378	0.505436921	0.415207	1.217313	0.223485	0.305519
14869	WNT8B	1.018954334	0.505242528	0.483005	1.04604	0.295542	0.389598
6212	PLEKHM3	424.1344965	0.50522247	0.121097	4.172031	3.02E-05	9.53E-05
1848	EIF4ENIF1	661.089672	0.505201284	0.070767	7.138986	9.40E-13	9.97E-12
5727	VAMP7	1156.353094	0.50513697	0.114592	4.408151	1.04E-05	3.57E-05
4466	WIPF2	1296.628999	0.505022695	0.099835	5.058594	4.22E-07	1.85E-06
11537	RASGRP1	485.4513914	0.504901838	0.239996	2.103793	0.035397	0.060138
10932	AFMID	670.1127606	0.504845765	0.218639	2.309034	0.020942	0.037548
4934	VAPA	2994.420226	0.504796447	0.105019	4.80673	1.53E-06	6.09E-06
6385	ZNF184	225.6867921	0.504745174	0.123587	4.084144	4.42E-05	0.000136
6066	HERC2	1318.80029	0.504738346	0.11912	4.237234	2.26E-05	7.31E-05
11199	HS3ST1	262.0983851	0.504518463	0.227106	2.221515	0.026316	0.04606
7908	RNF138	492.7864948	0.504464014	0.146439	3.444869	0.000571	0.001416
12910	GGT1	2979.196263	0.504360537	0.301897	1.670639	0.094793	0.143917
8407	RGPD6	672.0625309	0.504328876	0.155326	3.246901	0.001167	0.00272
4146	RNF115	477.5201821	0.504133012	0.095974	5.252835	1.50E-07	7.08E-07
8459	KLHL26	204.8295395	0.503876681	0.156329	3.223176	0.001268	0.002938
10101	PHF7	58.26666593	0.503506452	0.19245	2.616304	0.008889	0.017249
5767	VPRBP	652.2147456	0.503166715	0.114656	4.388493	1.14E-05	3.88E-05
14729	LRRC7	37.85836175	0.503124678	0.461663	1.08981	0.275797	0.367024
7138	PIGA	359.1971033	0.503061731	0.133484	3.76871	0.000164	0.000451
6514	IFT80	729.6420761	0.503054369	0.125067	4.022268	5.76E-05	0.000173
5891	PPIP5K2	1081.402175	0.502959315	0.116281	4.325385	1.52E-05	5.07E-05
6627	NUDT21	2033.275985	0.502926077	0.12657	3.973496	7.08E-05	0.000209
5402	SPIN3	193.9194243	0.502897341	0.110175	4.564544	5.01E-06	1.82E-05
4983	DCAF4	224.0394079	0.502776645	0.105139	4.782021	1.74E-06	6.82E-06
11258	SLC5A3	2880.670784	0.502755219	0.228785	2.197496	0.027985	0.048724

3347	ZNF322A	698.9459165	0.50248132	0.086883	5.783414	7.32E-09	4.29E-08
7328	LDOC1L	987.4344972	0.502447441	0.136202	3.688988	0.000225	0.000602
6824	KLHL2	459.9852219	0.502161711	0.129086	3.890124	0.0001	0.000288
9107	RRAGC	413.8393299	0.502147544	0.168789	2.974999	0.00293	0.006305
4799	CHURC1	1301.263171	0.50157146	0.102619	4.887716	1.02E-06	4.17E-06
10336	NDUFA4	3184.022653	0.501491495	0.197896	2.534112	0.011273	0.021378
6311	SELT	2612.300241	0.500879309	0.121571	4.120072	3.79E-05	0.000118
5257	ZNF627	396.1361281	0.500518172	0.107704	4.647159	3.37E-06	1.25E-05
7645	MOSPD2	323.8763954	0.500479226	0.140483	3.56257	0.000367	0.000942
5755	MRPS30	449.6641418	0.500285884	0.113882	4.393035	1.12E-05	3.81E-05
8365	OAT	1361.762323	0.500141236	0.153147	3.265755	0.001092	0.002558
6419	TYW3	553.6031404	0.499890479	0.122894	4.06765	4.75E-05	0.000145
13532	CCDC144C	2.076314066	0.499621682	0.338492	1.476023	0.139938	0.202699
9512	MAF	3996.941116	0.499396461	0.177383	2.815363	0.004872	0.01004
3560	WAC	2845.916297	0.499361727	0.088731	5.627791	1.83E-08	1.01E-07
9129	TMEM65	655.4713282	0.499358815	0.16835	2.966192	0.003015	0.006474
5554	ZNF461	116.6363445	0.499031863	0.110942	4.498129	6.86E-06	2.42E-05
6388	ZKSCAN3	60.31982679	0.499006417	0.122294	4.08037	4.50E-05	0.000138
9591	UAP1L1	563.1224724	0.498937849	0.179284	2.782939	0.005387	0.011009
8701	FAM92A1	160.2621184	0.498874722	0.159393	3.129836	0.001749	0.00394
13428	CYP3A5	729.4184884	0.498831591	0.331168	1.50628	0.131995	0.192675
2485	PACRGL	166.1665173	0.498771091	0.076916	6.484653	8.89E-11	7.01E-10
13280	GNAO1	79.12988403	0.498637845	0.321495	1.550999	0.120902	0.178449
8716	ELP3	873.4033123	0.498435691	0.159499	3.125015	0.001778	0.003998
15048	SLITRK5	73.82151431	0.498334227	0.502821	0.991076	0.321648	0.418968
9359	FZD4	3838.241925	0.498314628	0.17321	2.876933	0.004016	0.00841
5115	THAP1	228.9671889	0.498272184	0.105548	4.720796	2.35E-06	9.00E-06
5997	THUMPD3	697.0633171	0.497969814	0.11665	4.268938	1.96E-05	6.42E-05
15648	NTN3	1.104038679	0.497933398	0.604795	0.823309	0.410332	0.51399
13010	LOC127841	2.564190258	0.497867101	0.303437	1.64076	0.100847	0.151937
12678	TRPM6	20.72100771	0.49785073	0.285199	1.745624	0.080876	0.12504
3670	TCHP	360.6209366	0.497846179	0.089622	5.55497	2.78E-08	1.48E-07
3278	DIS3L	684.6331972	0.497749736	0.085359	5.831265	5.50E-09	3.29E-08
10678	EXOSC7	465.5716264	0.497736326	0.207175	2.402494	0.016284	0.029891
7952	ZNF100	192.8152249	0.497350436	0.145125	3.427047	0.00061	0.001504
5958	RIOK3	1981.545319	0.497339385	0.116011	4.287005	1.81E-05	5.96E-05
5232	C8orf40	511.0166856	0.497281777	0.106682	4.661326	3.14E-06	1.18E-05
6339	ABCA11P	66.78680284	0.497276806	0.121021	4.109007	3.97E-05	0.000123
3974	RCOR1	733.7637707	0.497239187	0.092841	5.355818	8.52E-08	4.20E-07
6988	LRRC8B	354.4342035	0.497237342	0.129813	3.830415	0.000128	0.000359
9134	MXI1	2137.45533	0.497082384	0.167655	2.96492	0.003028	0.006497
8000	DDX28	302.8495447	0.497009593	0.145762	3.409731	0.00065	0.001593
9982	LRRC37A4	189.5855012	0.496961286	0.186947	2.658304	0.007854	0.015421
5971	SOS1	1481.799246	0.496894516	0.116058	4.281415	1.86E-05	6.10E-05
3585	CDC23	852.4949412	0.496751151	0.088491	5.613551	1.98E-08	1.08E-07
11710	RAB39B	45.58588272	0.49671951	0.242292	2.050084	0.040356	0.067551
6376	PGBD1	111.1799816	0.496625151	0.121441	4.089432	4.32E-05	0.000133

6104	PPP1R2P3	57.87903918	0.496501254	0.117638	4.220582	2.44E-05	7.82E-05
4915	ALDH7A1	1869.869022	0.496476774	0.103017	4.81939	1.44E-06	5.74E-06
5772	ZNF644	856.4092658	0.496428544	0.113223	4.384539	1.16E-05	3.95E-05
6592	ZNF141	93.06198352	0.49623923	0.12451	3.985544	6.73E-05	0.0002
6027	PDCD10	598.6763123	0.496208914	0.116657	4.253574	2.10E-05	6.84E-05
4731	LOC72908	543.913259	0.495988343	0.100851	4.918042	8.74E-07	3.62E-06
10867	PARM1	1452.896049	0.495984089	0.212509	2.333947	0.019598	0.03535
5736	RAP1GDS1	839.7154189	0.495917308	0.112624	4.403288	1.07E-05	3.64E-05
6854	DLG1	1554.07951	0.495726754	0.127801	3.878894	0.000105	0.0003
14378	AGXT	23.709147	0.495703032	0.411906	1.203436	0.228807	0.311925
6444	PPM1D	418.1575443	0.495693896	0.122324	4.05231	5.07E-05	0.000154
5029	GDAP2	325.12478	0.495683036	0.104128	4.760339	1.93E-06	7.53E-06
16305	NKX2-2	2.016626943	0.495504581	0.791293	0.626196	0.531186	0.638551
5654	TMEM126I	755.9292218	0.49537447	0.111431	4.445557	8.77E-06	3.04E-05
10962	HCG4P6	8.684834597	0.495338975	0.215389	2.299746	0.021463	0.038377
15131	TUBB4Q	0.749695199	0.495334133	0.51362	0.964397	0.334847	0.433767
5391	PARG	377.8636441	0.495304758	0.108341	4.571701	4.84E-06	1.76E-05
6725	AAGAB	784.2837269	0.495248131	0.125956	3.931912	8.43E-05	0.000246
14748	TDGF1	3.579636014	0.49523453	0.456725	1.084318	0.278224	0.369777
8358	DYRK3	210.36454	0.494971138	0.151482	3.267521	0.001085	0.002544
1695	GOSR1	1202.463855	0.494920479	0.067792	7.300521	2.87E-13	3.31E-12
13162	PNPLA3	19.68857552	0.494865496	0.311011	1.591149	0.111576	0.16616
7985	TTC14	631.5515247	0.494838134	0.144832	3.416643	0.000634	0.001556
7924	ZBTB37	25.32895098	0.494761141	0.143885	3.438598	0.000585	0.001446
12585	LRCH2	110.6648609	0.494684524	0.278335	1.777296	0.075519	0.117621
6106	FLCN	713.3491653	0.494621011	0.117233	4.219131	2.45E-05	7.87E-05
9312	CBWD6	106.722129	0.494507542	0.170761	2.89591	0.003781	0.007958
4109	CTNND1	7715.943804	0.494492991	0.093733	5.275569	1.32E-07	6.31E-07
7225	NICN1	290.7030468	0.494461715	0.132348	3.736083	0.000187	0.000507
8218	PIK3C2B	636.8269044	0.494460554	0.148705	3.325102	0.000884	0.002108
3453	PDE8A	1225.293524	0.494455797	0.086662	5.70556	1.16E-08	6.58E-08
7866	GAN	32.85310763	0.494381173	0.142667	3.465284	0.00053	0.00132
6023	DNAJC19	563.5556384	0.494296408	0.116171	4.25492	2.09E-05	6.81E-05
5623	THEM4	365.1169133	0.494285496	0.110811	4.460598	8.17E-06	2.85E-05
6727	ARL8B	1960.003011	0.494211044	0.12571	3.931351	8.45E-05	0.000246
12570	PLA2R1	241.1844305	0.49411551	0.277354	1.781537	0.074825	0.116678
8524	C21orf49	9.31605926	0.493857386	0.154533	3.1958	0.001394	0.003206
9660	TWSG1	1456.741115	0.493814831	0.178811	2.761662	0.005751	0.01167
4837	C20orf194	833.3897934	0.493611098	0.101352	4.870272	1.11E-06	4.52E-06
3385	RNF185	1465.751217	0.493447188	0.085714	5.756902	8.57E-09	4.96E-08
6300	TMEM50B	971.7882901	0.493316693	0.119522	4.127403	3.67E-05	0.000114
5096	TFB2M	306.6598509	0.493277491	0.10431	4.728953	2.26E-06	8.68E-06
4472	RRAGB	271.5272014	0.49305175	0.097512	5.056337	4.27E-07	1.87E-06
3582	IARS2	2578.94546	0.49304469	0.087791	5.616127	1.95E-08	1.07E-07
10048	LOC59510	87.13914281	0.492987655	0.187062	2.635428	0.008403	0.016392
6540	MOBKL3	897.4871916	0.492948853	0.122982	4.008309	6.12E-05	0.000183
7349	ROD1	2507.659824	0.492729629	0.133845	3.681355	0.000232	0.000619

3146	SPATA5L1	181.0516613	0.492602691	0.083138	5.925129	3.12E-09	1.94E-08
9425	CCRN4L	206.1489057	0.492581687	0.172929	2.848461	0.004393	0.009136
7075	TMEM30A	5500.307631	0.492515446	0.129829	3.79358	0.000148	0.000411
12037	OVGP1	78.36208531	0.492180994	0.252517	1.949098	0.051284	0.08351
4601	TECPR2	594.9299953	0.492063375	0.09864	4.988465	6.09E-07	2.59E-06
665	PLRG1	748.7434825	0.491984286	0.053951	9.119031	7.58E-20	2.23E-18
11332	MTMR11	895.8308933	0.491953381	0.226685	2.170206	0.029991	0.051876
6638	ETAA1	424.2768835	0.491849221	0.12395	3.968121	7.24E-05	0.000214
9379	CNDP2	18492.09419	0.491675727	0.171506	2.866819	0.004146	0.008665
4281	IPO8	1600.042191	0.491446225	0.095126	5.166242	2.39E-07	1.09E-06
4698	PPM1B	967.654763	0.49109331	0.099582	4.931523	8.16E-07	3.40E-06
6148	SHOC2	1355.081095	0.490981377	0.116813	4.20314	2.63E-05	8.39E-05
6538	INO80D	689.5824889	0.490951794	0.122454	4.009275	6.09E-05	0.000183
6440	SMEK2	2379.724429	0.490920264	0.121106	4.05364	5.04E-05	0.000153
8758	BAG4	173.8867246	0.490917728	0.158223	3.102703	0.001918	0.004292
9201	SYNE1	2285.69876	0.490894391	0.167112	2.937509	0.003309	0.007048
4782	TTC5	215.8400739	0.490826645	0.100291	4.894027	9.88E-07	4.05E-06
9983	BOC	317.006628	0.490601652	0.184561	2.658211	0.007856	0.015424
3434	SP2	490.0559104	0.49042806	0.085717	5.721466	1.06E-08	6.03E-08
5379	ZNF235	80.00964908	0.490385063	0.107134	4.5773	4.71E-06	1.72E-05
5881	ZFP1	272.8681926	0.490298388	0.113162	4.332725	1.47E-05	4.91E-05
9304	C6orf136	268.8204889	0.490226948	0.16907	2.899543	0.003737	0.007873
4960	KRIT1	829.5179732	0.490038684	0.102266	4.791808	1.65E-06	6.53E-06
7310	WDR44	428.6926424	0.490026646	0.132535	3.697351	0.000218	0.000584
10748	KIAA1543	345.712904	0.489962966	0.205757	2.381274	0.017253	0.031464
14329	COL4A6	10.14311929	0.489611676	0.400582	1.22225	0.221613	0.30315
6982	TMF1	1139.752486	0.489513922	0.127724	3.832587	0.000127	0.000356
14551	LOC28427€	18.86068041	0.488905226	0.424976	1.15043	0.249967	0.336737
6508	KLHL7	661.5426398	0.488852244	0.12148	4.024129	5.72E-05	0.000172
8598	MDN1	656.1378576	0.488754809	0.153919	3.175401	0.001496	0.003411
7219	PTBP2	402.0459844	0.48849796	0.130687	3.737933	0.000186	0.000504
8331	TDP2	1315.71908	0.48839266	0.14889	3.280219	0.001037	0.00244
5852	VPS26A	1363.878784	0.48807998	0.112331	4.345012	1.39E-05	4.66E-05
7130	ACAP2	1299.394451	0.487990202	0.129307	3.773889	0.000161	0.000442
7566	NFXL1	203.3426961	0.487733473	0.135857	3.590057	0.000331	0.000856
8112	C9orf93	35.47062279	0.487633539	0.144923	3.364768	0.000766	0.001851
11124	SNPH	145.8295713	0.487320061	0.217103	2.244646	0.024791	0.04369
4136	EZH1	940.1210645	0.487036723	0.092586	5.260388	1.44E-07	6.81E-07
7563	RSPH3	177.4781687	0.486800816	0.135532	3.591781	0.000328	0.000851
5423	USP25	1046.920583	0.486787298	0.106864	4.5552	5.23E-06	1.89E-05
12988	PCA3	3.134970956	0.486750358	0.295208	1.648836	0.099181	0.149699
8897	ZNF678	42.60815236	0.48674659	0.159417	3.053292	0.002263	0.004987
4648	PCF11	1148.653177	0.48674033	0.098093	4.962042	6.98E-07	2.94E-06
7977	ZNF572	52.1160603	0.48665827	0.142333	3.419144	0.000628	0.001544
6200	ING3	344.4150366	0.486415351	0.116476	4.176111	2.97E-05	9.37E-05
3783	ARIH1	1489.830212	0.486297897	0.088682	5.483601	4.17E-08	2.16E-07
8296	PUS7L	192.8083148	0.486131749	0.147523	3.295291	0.000983	0.002323

7027	CS	4201.776774	0.486097589	0.127547	3.81113	0.000138	0.000386
4507	CHMP1B	1862.749176	0.485915967	0.0966	5.030194	4.90E-07	2.13E-06
5690	MYST3	1307.088877	0.485854187	0.109766	4.426285	9.59E-06	3.30E-05
13324	OC1001303	3.025224855	0.48566748	0.316353	1.535206	0.124733	0.183496
13385	ODZ1	473.109827	0.485651939	0.320447	1.515547	0.129634	0.189836
14124	ADAM21P1	0.905309756	0.485589804	0.379299	1.280228	0.200465	0.278201
5740	RAB12	1006.138055	0.485425632	0.11027	4.402151	1.07E-05	3.66E-05
15629	FLJ41941	1.074257717	0.485364708	0.587046	0.826791	0.408355	0.512136
4352	ZNF585A	165.2238023	0.485227573	0.094591	5.129743	2.90E-07	1.31E-06
10318	CA13	135.8746865	0.485177793	0.190947	2.540903	0.011057	0.021
13212	C2orf72	216.945793	0.48512475	0.307832	1.57594	0.11504	0.17067
4604	GOLGB1	3723.843507	0.484907783	0.097227	4.987371	6.12E-07	2.61E-06
9725	CAMK2N1	2953.605049	0.484706317	0.176886	2.740216	0.00614	0.012375
3742	GAPVD1	1066.454737	0.484532955	0.08791	5.511705	3.55E-08	1.86E-07
6786	USP9X	4327.506113	0.484283154	0.124043	3.904148	9.46E-05	0.000273
11545	AMT	329.3550221	0.484270705	0.230638	2.099703	0.035755	0.060704
5978	NEK4	338.207127	0.484049756	0.113133	4.278598	1.88E-05	6.17E-05
4571	ZNF326	360.9919537	0.483844507	0.096787	4.999091	5.76E-07	2.47E-06
3982	COX15	1301.069064	0.483755375	0.090369	5.353138	8.64E-08	4.26E-07
5362	ZNF586	152.0837087	0.483734983	0.105492	4.585507	4.53E-06	1.66E-05
11879	PAPLN	1526.8221	0.483558105	0.242167	1.996795	0.045847	0.075651
10853	SEC16B	520.8568896	0.483489367	0.20673	2.338746	0.019349	0.034944
3086	SMEK1	1413.978247	0.483443831	0.080955	5.971752	2.35E-09	1.49E-08
3972	ZBED5	929.8283985	0.483362994	0.09019	5.35936	8.35E-08	4.12E-07
11106	DNALI1	551.9388051	0.483348808	0.214736	2.250896	0.024392	0.04305
6046	YTHDC2	986.5873492	0.483227936	0.11383	4.245176	2.18E-05	7.08E-05
3686	DDX17	8826.284987	0.48317112	0.087128	5.545524	2.93E-08	1.56E-07
6183	PGGT1B	443.6446404	0.482910368	0.115434	4.183417	2.87E-05	9.10E-05
5478	WDR89	290.1835874	0.482876528	0.106564	4.53132	5.86E-06	2.10E-05
5087	INPP5A	499.9406022	0.482622431	0.102031	4.730145	2.24E-06	8.64E-06
4913	MRPL46	414.5660095	0.482600467	0.100124	4.820043	1.44E-06	5.73E-06
7123	AM120AO	1354.127698	0.48254698	0.127756	3.777087	0.000159	0.000437
6384	PEX2	917.5498055	0.48251624	0.118135	4.084449	4.42E-05	0.000136
8768	SNX16	273.596519	0.482188446	0.155726	3.096383	0.001959	0.004379
5305	C17orf75	263.8478559	0.481711388	0.104315	4.617857	3.88E-06	1.43E-05
12689	C10orf67	17.05250518	0.481711284	0.27681	1.740221	0.08182	0.12639
4356	C2orf43	397.8260812	0.481687577	0.093936	5.127823	2.93E-07	1.32E-06
9792	TMOD2	694.9353223	0.481492715	0.177089	2.718935	0.006549	0.01311
6660	GPR89A	799.6504539	0.481435891	0.121662	3.957172	7.58E-05	0.000223
5070	COX10	288.2835576	0.481409989	0.101623	4.737205	2.17E-06	8.38E-06
11719	LOC15837	123.6860365	0.48134683	0.235171	2.046793	0.040678	0.068038
15235	SOX10	1.514818662	0.481243831	0.513355	0.937449	0.348528	0.448408
12481	MBNL3	89.25099642	0.481211999	0.266067	1.808612	0.070511	0.110736
8411	KAZ	620.5488122	0.481058992	0.148227	3.24541	0.001173	0.002733
12118	F2RL1	1152.404379	0.480781312	0.250028	1.922907	0.054492	0.088141
2902	TOX4	1460.402451	0.480591031	0.078641	6.111216	9.89E-10	6.68E-09
13386	DNM1	653.9611605	0.480554788	0.317131	1.515321	0.129691	0.189906

7319	TSC22D1	8154.817635	0.480507327	0.130141	3.69221	0.000222	0.000595
11125	HOXD3	78.67770572	0.480453616	0.214088	2.244187	0.02482	0.043731
6225	ZCCHC10	347.6214022	0.480261115	0.115271	4.166382	3.09E-05	9.74E-05
5673	C4orf3	4180.491175	0.480236552	0.108266	4.435714	9.18E-06	3.17E-05
13661	RFX8	14.27246222	0.480231687	0.334589	1.43529	0.151204	0.21695
7485	TBC1D12	401.7343892	0.480162847	0.132599	3.621166	0.000293	0.000768
10475	EYS	12.60794825	0.479713019	0.193251	2.482327	0.013053	0.024425
5162	FAM120B	615.0996487	0.479552784	0.102181	4.69315	2.69E-06	1.02E-05
7824	INPP4A	673.586053	0.479187102	0.137201	3.492588	0.000478	0.001198
10599	RBM43	456.8050469	0.47917836	0.196678	2.436364	0.014836	0.027436
5798	FAM190B	1301.361349	0.479152244	0.109534	4.374469	1.22E-05	4.12E-05
6816	DOCK1	1625.899784	0.479064755	0.123062	3.892863	9.91E-05	0.000285
10478	HDAC11	532.7820126	0.479021591	0.193055	2.481266	0.013092	0.02449
9289	PLEKHA2	2565.143677	0.478802609	0.164899	2.903618	0.003689	0.007784
10204	OXR1	1235.542431	0.478764111	0.185506	2.580857	0.009856	0.018932
7948	CLDN12	1175.426033	0.478289643	0.139503	3.428522	0.000607	0.001497
7206	ADIPOR2	2184.237947	0.478144152	0.12781	3.741064	0.000183	0.000498
12396	RENBP	558.9687923	0.478143617	0.260263	1.837156	0.066187	0.104657
7295	TMED10P1	49.13558521	0.478124131	0.12919	3.700935	0.000215	0.000577
7981	SPCS3	4070.95083	0.478046401	0.139828	3.418809	0.000629	0.001545
5679	PNN	1630.419359	0.477941526	0.107827	4.432489	9.32E-06	3.22E-05
13534	UGT2B10	14.77608917	0.477925221	0.323907	1.475504	0.140077	0.202871
7650	IRAK1BP1	38.68601091	0.47791472	0.134258	3.55967	0.000371	0.000951
3650	SAP30L	1116.657676	0.477802964	0.085749	5.572094	2.52E-08	1.35E-07
5214	ATP2C1	2965.452873	0.477799241	0.102253	4.672717	2.97E-06	1.12E-05
13627	ITGA8	181.6667176	0.477681711	0.330133	1.446939	0.147914	0.212759
8569	RNF169	597.8749819	0.477672443	0.15001	3.184278	0.001451	0.003319
4626	RANBP9	793.8823104	0.477575234	0.09596	4.976809	6.46E-07	2.74E-06
7932	TMX1	1465.214526	0.477456261	0.138982	3.435372	0.000592	0.001462
13882	CHODL	12.73655311	0.47745189	0.352675	1.353803	0.175799	0.248224
7487	C6orf120	1020.024681	0.477408881	0.131852	3.620786	0.000294	0.000769
7371	VAMP2	1786.197665	0.477342705	0.130079	3.66965	0.000243	0.000646
13234	FCER1A	56.77568352	0.477187692	0.304206	1.568633	0.116733	0.172895
6935	SNRPN	2242.744127	0.47715508	0.12397	3.848959	0.000119	0.000335
13894	NTSR1	10.70292447	0.477080778	0.353432	1.349853	0.177063	0.249792
7651	SUOX	821.110642	0.477075814	0.134045	3.559066	0.000372	0.000953
7214	ZNF836	138.0280764	0.477050804	0.127593	3.738858	0.000185	0.000502
9945	ARGLU1	1524.855369	0.476961703	0.178479	2.672373	0.007532	0.014843
6048	DOPEY1	614.4730776	0.476822245	0.112385	4.242755	2.21E-05	7.16E-05
5970	RRN3	1275.737856	0.476740186	0.111328	4.28232	1.85E-05	6.07E-05
5503	CARKD	1008.456316	0.476634901	0.10548	4.518715	6.22E-06	2.22E-05
3125	DHX9	3233.973119	0.476525547	0.080143	5.945938	2.75E-09	1.72E-08
6542	ANO10	734.6305145	0.476335139	0.118863	4.007432	6.14E-05	0.000184
8858	NFKBIA	3744.165466	0.476331639	0.155287	3.067436	0.002159	0.004778
5811	C14orf142	235.8725922	0.476328612	0.108999	4.370009	1.24E-05	4.19E-05
7506	SIRT1	660.5186974	0.476177326	0.131711	3.615323	0.0003	0.000783
11616	SPATA17	24.14126839	0.476153389	0.229417	2.075493	0.037941	0.064022

6488	ALKBH8	208.1706879	0.476126783	0.118053	4.033157	5.50E-05	0.000166
12557	NPY1R	431.8207457	0.475999641	0.266374	1.786957	0.073944	0.115424
7612	C12orf29	479.502477	0.475759732	0.133033	3.576267	0.000349	0.000897
11157	ITPR1	1649.697759	0.475748564	0.212754	2.236144	0.025342	0.044522
13839	C9orf122	32.84269691	0.475638316	0.346751	1.371701	0.170156	0.241003
15523	PCSK9	3.312513526	0.475513249	0.557835	0.852427	0.393977	0.497478
8294	VHL	392.2150796	0.475362424	0.144203	3.296489	0.000979	0.002314
7594	CAPZA2	4268.316119	0.475323976	0.132716	3.581513	0.000342	0.000882
5650	KLF11	1106.287211	0.475317934	0.106857	4.448148	8.66E-06	3.00E-05
9812	PRDM11	23.8573808	0.475285798	0.175149	2.713609	0.006655	0.013295
10267	RPS6KA5	102.4881351	0.475279773	0.185756	2.558628	0.010509	0.020062
5498	UBXN4	3602.888932	0.475252758	0.10507	4.523193	6.09E-06	2.17E-05
13751	MPV17L	192.0968135	0.475007448	0.338719	1.402365	0.160806	0.229217
6579	MAEA	1258.841507	0.474741831	0.118938	3.991494	6.57E-05	0.000196
6697	FAM54B	1221.546951	0.474697029	0.120438	3.941428	8.10E-05	0.000237
5979	SETX	2088.72417	0.474675268	0.110959	4.277951	1.89E-05	6.18E-05
16152	AMAC1	0.561836181	0.474530816	0.71288	0.665653	0.505633	0.613578
15208	RD3	0.916207514	0.474376329	0.501852	0.945252	0.34453	0.444052
8745	SPATA24	62.54309935	0.474250427	0.152466	3.110526	0.001868	0.004186
6969	LAMP2	7577.394082	0.474076606	0.12345	3.840223	0.000123	0.000346
7149	BAZ2B	705.2371583	0.473584188	0.125794	3.764763	0.000167	0.000457
8208	RMI1	228.0308435	0.473171979	0.142137	3.328987	0.000872	0.002081
7844	MAPRE2	2543.514828	0.473016544	0.136035	3.477158	0.000507	0.001266
10990	RAET1E	10.09449591	0.472917641	0.206487	2.290306	0.022004	0.039244
14468	SH2D7	0.71405989	0.472620855	0.4012	1.178018	0.23879	0.323508
5137	C16orf72	1219.648937	0.472402206	0.10029	4.710374	2.47E-06	9.43E-06
7076	NEO1	1869.962542	0.471842865	0.124389	3.79329	0.000149	0.000412
10467	SHANK3	1398.433266	0.471453613	0.189614	2.486381	0.012905	0.024166
6589	C9orf95	536.9818108	0.471442771	0.11823	3.987494	6.68E-05	0.000199
8328	LOC22093(	148.3930029	0.471432003	0.143699	3.280691	0.001036	0.002437
11028	GLB1L	1002.799116	0.47087433	0.206472	2.280576	0.022574	0.040122
5045	ARFGAP3	1788.833807	0.470807407	0.099108	4.750429	2.03E-06	7.89E-06
7983	TRDMT1	176.6108766	0.470787573	0.137744	3.417855	0.000631	0.00155
14425	FAM150B	234.2898922	0.470785166	0.395358	1.190782	0.233739	0.31761
5564	TNRC6B	858.0494379	0.470703447	0.104809	4.491072	7.09E-06	2.50E-05
9241	SLC2A11	266.6542832	0.470651384	0.161093	2.921619	0.003482	0.007386
16565	NKX6-1	0.508030532	0.470511921	0.84727	0.555327	0.578671	0.684729
4185	BCDIN3D	143.3867113	0.470371761	0.090153	5.217486	1.81E-07	8.49E-07
6995	NLK	655.9537341	0.470246673	0.122919	3.825664	0.00013	0.000365
7659	WDR35	688.8172378	0.470165462	0.132281	3.554302	0.000379	0.00097
9759	DYX1C1	74.55989206	0.469833085	0.172182	2.728705	0.006358	0.012771
7499	MAP4K5	855.0208066	0.469696475	0.129832	3.617718	0.000297	0.000777
6296	HIRA	710.44868	0.469534519	0.113716	4.129022	3.64E-05	0.000113
10884	OC1001290	4064.838541	0.469490495	0.201692	2.327757	0.019925	0.035883
15789	LINGO2	1.762694953	0.469410727	0.602131	0.779582	0.435637	0.540815
11865	KBTBD11	2262.0827	0.469370814	0.234453	2.001982	0.045287	0.074814
12349	CSRNP3	15.00442278	0.469280292	0.253736	1.84948	0.064389	0.102201

8298	ADAT1	254.5272504	0.469232189	0.142421	3.294682	0.000985	0.002327
5263	MYST4	729.2509912	0.469113228	0.101009	4.644288	3.41E-06	1.27E-05
9352	TUBB1	20.38147116	0.469091297	0.162941	2.878905	0.003991	0.008365
6039	C14orf109	345.4634343	0.468874401	0.110328	4.249834	2.14E-05	6.94E-05
16116	AKAP4	0.304447779	0.468709661	0.693261	0.676094	0.498981	0.606846
16213	ST7OT2	0.228863618	0.46870955	0.719456	0.651477	0.514738	0.622302
17204	BOLL	0.191924843	0.468708055	1.212622	0.386524	0.699108	0.796467
17343	CDX2	0.213936514	0.468707703	1.316199	0.356107	0.72176	0.815731
17346	LOR	0.167635695	0.468707576	1.322727	0.35435	0.723077	0.817078
17434	PRB2	0.194963755	0.468707224	1.418027	0.330535	0.740996	0.8331
17566	BANF2	0.128091275	0.468706333	1.566005	0.299301	0.764711	0.853233
17709	DNAJC5G	0.089863485	0.46870507	1.766118	0.265387	0.790711	0.875193
17936	MTNR1B	0.131342548	0.468703003	2.121955	0.220883	0.825184	0.901786
18119	CFHR2	0.20921814	0.468700104	2.601571	0.18016	0.857027	0.927074
18205	C14orf165	0.071772388	0.468695788	2.91407	0.160839	0.87222	0.939104
7633	ZNF442	34.15754185	0.468665984	0.131398	3.566768	0.000361	0.000928
4946	RPRD2	1521.025157	0.468624312	0.097581	4.802433	1.57E-06	6.21E-06
7147	ATP11B	1528.406634	0.468543346	0.124441	3.765182	0.000166	0.000456
13728	AACSL	22.98108423	0.46848227	0.332856	1.407462	0.15929	0.227437
7585	APITD1	319.9742952	0.468442461	0.130696	3.584223	0.000338	0.000874
4494	MED20	305.6035516	0.46824572	0.092867	5.042089	4.60E-07	2.01E-06
4660	ANKFY1	1462.355385	0.468241285	0.094484	4.955778	7.20E-07	3.03E-06
12702	IQCA1	137.8905702	0.468103833	0.270077	1.733225	0.083056	0.128167
14946	PTPRT	6.299385661	0.468099169	0.457568	1.023014	0.306301	0.4017
9848	GCLM	493.1319788	0.467933021	0.172981	2.705119	0.006828	0.01359
15323	TMEM132C	4.187046536	0.467819141	0.511891	0.913903	0.360768	0.46149
9761	SNED1	440.1257299	0.467562159	0.17143	2.727425	0.006383	0.012818
4647	FUBP1	1263.714397	0.467517362	0.094202	4.962933	6.94E-07	2.93E-06
6306	CEBPZ	1244.088433	0.46732386	0.113327	4.123689	3.73E-05	0.000116
10732	CASC2	64.90181196	0.467176258	0.195715	2.387028	0.016985	0.031019
16397	ITIH5L	0.726898453	0.466906447	0.775785	0.60185	0.547274	0.654212
7734	USP6NL	395.5644858	0.466895452	0.132409	3.526154	0.000422	0.001069
8120	IDE	498.0626178	0.466301637	0.138667	3.362738	0.000772	0.001863
13630	GLIS1	224.1126022	0.466054027	0.322616	1.444611	0.148567	0.213651
4803	TBC1D13	968.8787452	0.465615038	0.095304	4.885583	1.03E-06	4.21E-06
12568	HIST2H2BA	7.309282891	0.465470178	0.261123	1.78257	0.074656	0.116434
8707	CNNM2	310.292902	0.465386254	0.148821	3.127155	0.001765	0.003973
7132	LRCH1	921.8707417	0.465175261	0.123295	3.772858	0.000161	0.000444
6530	LIAS	227.3200825	0.464995196	0.11588	4.012725	6.00E-05	0.00018
7618	YPEL2	1382.573354	0.464921564	0.130067	3.574491	0.000351	0.000903
12095	MAP2	631.2882933	0.464801202	0.24073	1.930796	0.053508	0.086715
5843	ABCE1	1385.239026	0.464733146	0.10684	4.349786	1.36E-05	4.57E-05
14412	ASFMR1	1.058513957	0.464692454	0.389107	1.194253	0.232379	0.316039
4019	SDAD1	784.6172838	0.464562063	0.087183	5.328611	9.90E-08	4.83E-07
8826	TMEM159	978.950155	0.464549221	0.150795	3.080675	0.002065	0.004587
9232	PTPRK	2001.970135	0.464318301	0.158618	2.927269	0.00342	0.00726
4317	KAT5	992.6326941	0.464309096	0.090211	5.146918	2.65E-07	1.20E-06

12173	LPCAT2	805.8351539	0.464287278	0.243749	1.904772	0.05681	0.091475
5778	APPL2	943.7517689	0.464236863	0.105934	4.382302	1.17E-05	3.98E-05
5809	ABI2	1452.141101	0.464024225	0.106175	4.37039	1.24E-05	4.18E-05
6461	C16orf63	926.4363711	0.463727992	0.114665	4.04419	5.25E-05	0.000159
15455	TAS2R43	0.447902932	0.463661819	0.530633	0.873791	0.382232	0.484781
6168	RRP15	481.4853242	0.462905348	0.11043	4.191829	2.77E-05	8.79E-05
8989	RBM7	610.7159487	0.462888774	0.153375	3.018011	0.002544	0.005548
10951	FAM21A	457.3343831	0.462860374	0.200929	2.303601	0.021245	0.038026
7099	HACL1	307.429039	0.462800805	0.122331	3.783191	0.000155	0.000428
13423	SLC10A6	17.86141628	0.462593206	0.306938	1.507123	0.131779	0.192431
6144	BPTF	1529.789773	0.462515481	0.110022	4.203836	2.62E-05	8.37E-05
11726	LRRIQ3	12.37227794	0.462324524	0.22609	2.044866	0.040868	0.068314
8564	NFIB	2698.43539	0.461979753	0.14504	3.185197	0.001447	0.003311
6744	DSTN	6883.13504	0.461754668	0.117716	3.922622	8.76E-05	0.000255
7307	TTC28	1237.246842	0.461750126	0.124867	3.697923	0.000217	0.000583
7426	SPRED2	1006.399087	0.46163809	0.126596	3.64655	0.000266	0.000702
5003	C12orf72	84.70318135	0.461107051	0.096608	4.772962	1.82E-06	7.11E-06
9700	LOC641367	4.691743823	0.461094801	0.16788	2.746566	0.006022	0.012169
4481	ZFR	2304.644686	0.461011063	0.091304	5.049186	4.44E-07	1.94E-06
9731	SHANK2	668.6236394	0.461006947	0.168379	2.737913	0.006183	0.012454
5464	VPS35	3062.37348	0.460977344	0.101624	4.536128	5.73E-06	2.06E-05
3554	POLR2B	1716.719702	0.460827875	0.081814	5.632622	1.77E-08	9.79E-08
13220	NME5	156.9980908	0.460761765	0.292815	1.573558	0.11559	0.171382
5635	PDXDC1	2939.786241	0.460550963	0.103412	4.453569	8.45E-06	2.94E-05
13029	GGTLC1	21.53128439	0.460369221	0.281606	1.634797	0.102092	0.153588
8327	RECK	371.1973337	0.460358751	0.140315	3.280891	0.001035	0.002436
4326	GMPR2	1368.266459	0.46020972	0.089477	5.143302	2.70E-07	1.22E-06
7247	ZNF587	474.5833581	0.46017901	0.123468	3.727102	0.000194	0.000524
9225	LRP2BP	81.04841832	0.460157484	0.157115	2.928795	0.003403	0.00723
5945	ZSCAN16	72.25237177	0.460023508	0.107153	4.293133	1.76E-05	5.81E-05
15961	ATP1A4	0.713307214	0.459927412	0.634763	0.724566	0.468718	0.575612
8574	CHCHD7	475.7531513	0.459878963	0.144563	3.181166	0.001467	0.003353
6509	SFMBT1	226.7330442	0.459835775	0.114283	4.023653	5.73E-05	0.000173
6087	DNAJA1	2904.875916	0.459822285	0.108786	4.226835	2.37E-05	7.63E-05
10797	PCBD1	1839.874546	0.459376861	0.194363	2.363494	0.018104	0.032865
9131	STAG1	864.7437067	0.459278052	0.154863	2.9657	0.00302	0.006483
12364	KCNN3	131.9733165	0.459177726	0.248639	1.846768	0.064781	0.102699
6543	OMA1	303.7323379	0.458918028	0.11452	4.007318	6.14E-05	0.000184
8607	PGBD4	38.39876865	0.458869671	0.144621	3.172917	0.001509	0.003436
6748	SMG1	2412.822993	0.458869264	0.117064	3.919813	8.86E-05	0.000257
2137	MFAP1	729.5542299	0.45884417	0.06731	6.816849	9.31E-12	8.54E-11
14747	SLC47A2	42.73275304	0.45883487	0.423137	1.084365	0.278203	0.369777
3699	TSG101	1479.758579	0.458784796	0.082873	5.535975	3.10E-08	1.64E-07
6286	ZNF845	188.3735118	0.458706946	0.110871	4.137298	3.51E-05	0.00011
7386	PDPR	1304.872527	0.458637295	0.12525	3.661789	0.00025	0.000665
8202	ZNF214	65.37649698	0.458583575	0.137637	3.331845	0.000863	0.002062
4684	RABEP1	1293.683292	0.458308759	0.092798	4.938777	7.86E-07	3.29E-06

8485	VCPIP1	879.9226021	0.458120101	0.142586	3.212932	0.001314	0.003035
5955	SETD2	1240.356028	0.458060049	0.10683	4.287762	1.80E-05	5.94E-05
10545	GLIS3	767.0727229	0.45793219	0.186571	2.454465	0.014109	0.026227
15333	KCNJ13	27.81841159	0.457709283	0.502894	0.91015	0.362743	0.463684
9864	MBOAT1	202.9961038	0.457564913	0.169535	2.698943	0.006956	0.013822
7732	UBXN8	213.7049799	0.457482071	0.129721	3.526651	0.000421	0.001067
7215	DCUN1D1	564.4771417	0.457361974	0.122335	3.738608	0.000185	0.000503
7081	GRINL1A	1241.982387	0.457176578	0.120596	3.790992	0.00015	0.000415
14580	SALL4	11.33195078	0.456897353	0.399953	1.142377	0.253297	0.340527
9154	ATF2	584.9485067	0.456691778	0.154449	2.956912	0.003107	0.006654
5365	CCDC93	851.7386668	0.456676923	0.099617	4.584338	4.55E-06	1.66E-05
6113	ZDHHC9	1965.907755	0.456630634	0.108342	4.214719	2.50E-05	8.02E-05
8794	SLC30A7	1372.156798	0.45656864	0.147878	3.08747	0.002019	0.004499
7504	CSPP1	276.6499339	0.456436556	0.126226	3.616024	0.000299	0.000781
6807	PTPN11	3140.956165	0.456277941	0.117094	3.896684	9.75E-05	0.000281
14862	SNX31	2.646507694	0.45624923	0.434805	1.04932	0.294031	0.387788
6408	CDYL	389.3655998	0.456218388	0.112016	4.072791	4.65E-05	0.000142
8988	ZNF516	267.0386792	0.455985442	0.151059	3.018596	0.002539	0.005538
6072	ZFYVE26	830.6331848	0.455771753	0.10763	4.234626	2.29E-05	7.39E-05
5723	MCART1	181.2590026	0.455767878	0.103359	4.409549	1.04E-05	3.55E-05
6491	CXorf56	163.6216367	0.455420379	0.112965	4.031526	5.54E-05	0.000167
6806	KIAA1598	922.4765414	0.455185878	0.116804	3.897018	9.74E-05	0.00028
5606	HNRPLL	530.6445478	0.455030737	0.101844	4.467913	7.90E-06	2.76E-05
7813	PHC3	1727.537839	0.455021411	0.130119	3.496955	0.000471	0.001181
10999	MERTK	615.0339829	0.455003241	0.198791	2.288849	0.022088	0.039359
3206	BTRC	500.9496753	0.454991046	0.077367	5.880917	4.08E-09	2.49E-08
5240	SDHB	1525.082463	0.45498138	0.097711	4.656391	3.22E-06	1.20E-05
6818	NAA50	2226.465602	0.45489027	0.116876	3.892089	9.94E-05	0.000286
6873	PPP1CB	7169.340993	0.454797952	0.11739	3.874233	0.000107	0.000305
3040	SAPS3	1954.651414	0.454277461	0.075652	6.004825	1.92E-09	1.23E-08
5149	DDX46	1140.697989	0.453807144	0.096496	4.702853	2.57E-06	9.77E-06
13270	HIST1H2AI	2.986046274	0.453806342	0.29166	1.555945	0.119721	0.176839
6630	SEC23IP	981.7523145	0.453736135	0.114232	3.972057	7.13E-05	0.000211
8094	SMC3	1148.324858	0.453660682	0.134481	3.37342	0.000742	0.001798
16347	MUC15	3.737970324	0.453652159	0.737702	0.614953	0.538586	0.645795
14550	DOC2A	434.2272637	0.453510736	0.394234	1.150361	0.249995	0.336737
5754	FAM173B	225.0084421	0.453479227	0.103219	4.393388	1.12E-05	3.80E-05
10447	STAG3L4	288.4158478	0.453320125	0.181681	2.495139	0.012591	0.023623
7139	EEF1E1	357.0529402	0.453192818	0.120258	3.768503	0.000164	0.000451
9205	WRB	909.1718355	0.453159604	0.15435	2.935924	0.003326	0.007081
5489	FBXO22	497.4189316	0.453042665	0.100083	4.526689	5.99E-06	2.14E-05
14988	GPC5	4.473908584	0.453037183	0.448572	1.009954	0.312517	0.408703
7837	YIPF4	665.2144365	0.452944327	0.13015	3.48017	0.000501	0.001253
6299	VBP1	888.0058028	0.452312076	0.109581	4.127665	3.66E-05	0.000114
5254	PPP2R2D	610.0066849	0.452262877	0.097299	4.64819	3.35E-06	1.25E-05
11010	SERP2	53.94410361	0.452227762	0.19774	2.286983	0.022197	0.039517
4725	SLC25A20	401.8572733	0.45216705	0.091911	4.919593	8.67E-07	3.60E-06

10091	COQ10A	169.7723983	0.451845619	0.172468	2.619886	0.008796	0.017085
7805	ZNF529	368.6748116	0.451652841	0.129052	3.499771	0.000466	0.001169
14542	HAVCR1	717.1888343	0.451479947	0.391561	1.153026	0.2489	0.335489
6401	NANP	193.0798948	0.451443794	0.11076	4.075856	4.58E-05	0.00014
13663	MLXIPL	452.0246146	0.451392626	0.314571	1.434945	0.151303	0.217059
12190	GGH	480.4748739	0.451371531	0.237622	1.899534	0.057494	0.092448
7192	CCDC82	678.2631271	0.451330935	0.120398	3.748669	0.000178	0.000485
8949	ZFAND1	662.1334023	0.451297216	0.148762	3.03369	0.002416	0.005292
6485	PINK1	2542.058747	0.451218285	0.111846	4.034279	5.48E-05	0.000166
9806	GDAP1	196.6522602	0.451150829	0.166191	2.714645	0.006635	0.013261
15461	MAGEA8	0.480763503	0.451123729	0.516906	0.872738	0.382806	0.485331
8609	APLF	79.8307732	0.451051085	0.142246	3.170928	0.00152	0.00346
7883	ACVR2B	298.097323	0.451018954	0.130528	3.455346	0.00055	0.001366
5810	KIAA0947	992.8157319	0.451002748	0.103195	4.370412	1.24E-05	4.18E-05
8616	MTERFD3	394.6036414	0.450897769	0.14229	3.168872	0.00153	0.003481
8588	ANKRD13A	2355.339092	0.450678513	0.141814	3.17795	0.001483	0.003385
5209	CNST	664.7227642	0.450589802	0.096409	4.673748	2.96E-06	1.11E-05
7205	TIGD7	256.0713574	0.450510793	0.12041	3.741468	0.000183	0.000498
14445	HAPLN4	28.11913756	0.450423205	0.380484	1.183817	0.236486	0.320877
8674	ZNF611	420.2102033	0.450238893	0.143332	3.14123	0.001682	0.003802
8072	ANUBL1	119.295249	0.450184505	0.133057	3.383393	0.000716	0.001739
8859	CDK8	143.1417918	0.450129078	0.146769	3.066919	0.002163	0.004785
6852	SPPL2A	894.0135411	0.45010909	0.116024	3.879448	0.000105	0.000299
11526	EDARADD	107.8909138	0.449646456	0.213214	2.108897	0.034954	0.059442
9800	CISD1	1058.64439	0.449546853	0.16546	2.716952	0.006589	0.013178
5021	MTX2	674.0457458	0.449367712	0.094352	4.762668	1.91E-06	7.46E-06
7048	RBM27	943.0708978	0.449333036	0.1182	3.80145	0.000144	0.0004
7049	MED22	596.6852626	0.449304354	0.118217	3.800683	0.000144	0.000401
6811	ST13	7311.695451	0.448892327	0.11524	3.895273	9.81E-05	0.000282
4534	SMCR7L	1257.263629	0.448762342	0.089448	5.017044	5.25E-07	2.27E-06
8032	MTERF	190.3593926	0.448692777	0.132018	3.398717	0.000677	0.001652
8646	KIAA0040	1241.432245	0.448637147	0.142298	3.152801	0.001617	0.003666
4903	FBXO11	1244.078504	0.448442757	0.092948	4.824682	1.40E-06	5.60E-06
6085	INPP5F	430.9066452	0.448317954	0.106055	4.227207	2.37E-05	7.62E-05
3917	CCDC43	495.5745671	0.44809972	0.083105	5.391975	6.97E-08	3.49E-07
7464	GRLF1	2434.981839	0.44805694	0.123414	3.630512	0.000283	0.000743
7817	PCNP	3189.216372	0.447965601	0.128135	3.496041	0.000472	0.001184
13455	C9orf47	20.71722886	0.447955981	0.298612	1.500127	0.133582	0.194585
7208	ZKSCAN2	185.0228106	0.447948549	0.119742	3.740959	0.000183	0.000499
6525	GALT	391.1211954	0.447701223	0.111491	4.015589	5.93E-05	0.000178
9169	ZNF680	254.4196469	0.447654799	0.151783	2.949311	0.003185	0.006808
7530	OXSM	207.7337407	0.447632986	0.124184	3.604594	0.000313	0.000814
6719	BIRC6	2044.071426	0.447610562	0.113814	3.932808	8.40E-05	0.000245
11995	FHOD3	837.6704793	0.447600342	0.228117	1.962155	0.049744	0.081285
4692	UPRT	310.3414675	0.447264446	0.09066	4.933404	8.08E-07	3.38E-06
8310	EML1	672.6444549	0.447123217	0.135963	3.288555	0.001007	0.002375
8934	RIN2	1798.569279	0.447121004	0.147079	3.040011	0.002366	0.00519

9775	CSNK2A1P	497.2078364	0.446977468	0.164094	2.723917	0.006451	0.012936
12052	SEMA6A	2039.362658	0.446769739	0.229922	1.943132	0.052	0.084572
7797	C14orf129	474.3168043	0.446437766	0.127463	3.502498	0.000461	0.001159
3321	YTHDC1	1578.75164	0.446315311	0.076927	5.801808	6.56E-09	3.87E-08
9395	TAF1L	17.73225365	0.446261871	0.156001	2.860631	0.004228	0.008821
3604	HTATSF1	1538.498068	0.446186696	0.079624	5.603678	2.10E-08	1.14E-07
7524	C6orf182	142.6945918	0.446052488	0.12369	3.606219	0.000311	0.000809
6902	KIAA0196	1282.269624	0.445602112	0.115316	3.864177	0.000111	0.000317
6343	ZSCAN12	183.0057308	0.445300587	0.108415	4.107369	4.00E-05	0.000124
7339	UBA3	810.3231088	0.445243323	0.120842	3.684506	0.000229	0.000612
5318	DSCR3	1329.739128	0.445167168	0.096505	4.612881	3.97E-06	1.46E-05
9444	MAP3K2	732.5661769	0.445050007	0.156678	2.840539	0.004504	0.009347
8220	LOC401588	65.46371601	0.444702863	0.133785	3.323999	0.000887	0.002116
7496	TMX2	1906.819328	0.44463097	0.122856	3.61911	0.000296	0.000773
7768	CCDC66	163.5029347	0.44462923	0.126679	3.509889	0.000448	0.001131
8479	ZNF846	75.75134146	0.444297529	0.138149	3.216082	0.0013	0.003004
3088	SLTM	1624.777402	0.444226311	0.074394	5.971236	2.35E-09	1.49E-08
12636	PLCD4	161.2745168	0.444153521	0.251961	1.762787	0.077936	0.120895
13471	CXorf57	211.6652984	0.444090566	0.296758	1.496476	0.13453	0.195748
10632	NEU3	92.49756089	0.444072107	0.183391	2.421451	0.015459	0.028494
4253	MYST2	943.9876187	0.444049408	0.085721	5.180191	2.22E-07	1.02E-06
8077	C14orf128	67.37686765	0.443932493	0.131358	3.379549	0.000726	0.001762
5689	BCAS2	636.8679788	0.443915969	0.100287	4.426465	9.58E-06	3.30E-05
8138	CHP	2882.262729	0.443819162	0.132186	3.35754	0.000786	0.001894
4614	CRK	1019.925027	0.443734801	0.089051	4.982939	6.26E-07	2.66E-06
9678	OSTM1	1560.220424	0.443605471	0.161063	2.754229	0.005883	0.011914
7465	OTUD4	1345.509219	0.443586662	0.122201	3.629981	0.000283	0.000744
6648	ERBB2IP	2504.655043	0.443538933	0.111931	3.9626	7.41E-05	0.000219
8350	PGRMC2	3003.361054	0.443489013	0.13543	3.274663	0.001058	0.002483
6001	TRIM37	471.1282495	0.443398463	0.103924	4.266582	1.98E-05	6.48E-05
11256	ZDBF2	224.6405436	0.443277972	0.201703	2.197677	0.027972	0.04871
8528	RBBP9	1025.472756	0.443168352	0.13869	3.195399	0.001396	0.003209
7463	TMOD3	1795.778767	0.44315509	0.122048	3.630986	0.000282	0.000742
6628	ZDHHC17	823.1840429	0.443136353	0.111533	3.973124	7.09E-05	0.00021
6334	RNF20	1132.241122	0.44311223	0.107796	4.110657	3.95E-05	0.000122
6616	CPSF2	980.8625739	0.442924084	0.111362	3.977353	6.97E-05	0.000206
8543	CASP8AP2	302.0556716	0.442851464	0.138739	3.191978	0.001413	0.003242
11118	MMRN2	2032.249316	0.442637778	0.197099	2.245759	0.024719	0.04358
5363	DNM1L	1613.690018	0.442503647	0.09651	4.58505	4.54E-06	1.66E-05
12852	FAM22F	3.293332135	0.442444629	0.261695	1.69069	0.090896	0.138629
5442	SON	5051.755896	0.442346415	0.097309	4.545804	5.47E-06	1.97E-05
8428	BTBD3	1266.541388	0.442338901	0.136574	3.238812	0.0012	0.002791
6495	ZNF607	163.2099007	0.442108477	0.10967	4.031251	5.55E-05	0.000167
7689	C2orf86	107.660563	0.442056968	0.124754	3.543426	0.000395	0.001007
6008	PLDN	1685.319954	0.442027508	0.103707	4.26229	2.02E-05	6.60E-05
10219	C11orf65	10.40473509	0.441983891	0.171677	2.574508	0.010038	0.019254
8962	OC1001288	87.40024929	0.441575422	0.145916	3.026233	0.002476	0.005416

6288	BIRC2	1620.593409	0.441548892	0.106783	4.135012	3.55E-05	0.000111
6371	RGPD4	151.7555819	0.441523126	0.107879	4.092751	4.26E-05	0.000131
13610	IDO1	674.4240844	0.441429904	0.303901	1.452543	0.146351	0.210773
7759	GFPT1	2597.997952	0.441295168	0.125556	3.514728	0.00044	0.001112
6383	ZNF839	235.6806509	0.440884421	0.107912	4.085601	4.40E-05	0.000135
9501	PMM1	1088.698499	0.440826481	0.156329	2.81986	0.004804	0.009912
10465	HIF1A	4462.21989	0.440766327	0.177167	2.487852	0.012852	0.024071
7549	MKLN1	2007.348919	0.440706255	0.122572	3.595475	0.000324	0.000841
11014	CDNF	29.20279207	0.440591185	0.192756	2.285746	0.022269	0.039631
7085	NR2C2	838.0819738	0.440417533	0.116216	3.789662	0.000151	0.000417
6693	MAGT1	3289.20602	0.440387875	0.111709	3.942292	8.07E-05	0.000236
11356	TCEAL6	19.82031417	0.440247473	0.203351	2.164965	0.03039	0.052456
6637	PPP1R3D	244.7070005	0.440159553	0.110889	3.96937	7.21E-05	0.000213
9400	MGC21881	178.6745745	0.440145112	0.153946	2.859091	0.004249	0.008859
10069	JUP	4127.365771	0.44012267	0.167379	2.62949	0.008551	0.016647
13647	ZSCAN1	3.629018112	0.440019317	0.305743	1.439179	0.1501	0.215586
8814	GRAMD3	932.9437637	0.439882287	0.142685	3.08289	0.00205	0.004559
10311	ZNF860	49.00763001	0.43984439	0.173012	2.542283	0.011013	0.020936
7888	SAR1B	746.9010418	0.439829033	0.127338	3.454015	0.000552	0.001372
8648	SETD7	2303.081327	0.439611808	0.139491	3.151542	0.001624	0.003681
5371	EIF2S1	1380.910752	0.439571742	0.095921	4.582661	4.59E-06	1.68E-05
16795	NPHS2	10.15834315	0.439411158	0.894705	0.491124	0.623339	0.727482
6946	CRNA0008	374.163928	0.439410171	0.114225	3.846883	0.00012	0.000338
8040	PIK3CB	1470.596878	0.439319129	0.12936	3.396086	0.000684	0.001666
7998	ARMCX1	489.6284888	0.439195594	0.128779	3.410447	0.000649	0.001589
7226	TBCEL	358.9306082	0.439169531	0.117547	3.736109	0.000187	0.000507
10277	LCA5	234.9769674	0.439156271	0.171897	2.554758	0.010626	0.020267
11474	C1001305	54.99878044	0.439089571	0.206334	2.128047	0.033333	0.056942
7705	ZNF302	695.2931972	0.438660996	0.123946	3.539118	0.000401	0.001021
10790	SCML1	275.4610489	0.438613134	0.185432	2.365354	0.018013	0.032722
8805	DEK	2252.420207	0.438461796	0.14212	3.085157	0.002034	0.004529
8390	ZNF624	110.0872207	0.438421709	0.134724	3.254223	0.001137	0.002656
14811	SLC39A5	691.1007138	0.438170468	0.412822	1.061402	0.288507	0.381813
13669	ZNF492	4.430286383	0.438083944	0.305918	1.43203	0.152135	0.218158
12631	THBS1	11007.36417	0.438038275	0.248314	1.764052	0.077723	0.120612
7982	UBE2CBP	59.49734504	0.43779906	0.128078	3.418217	0.00063	0.001548
6365	C1orf163	167.1381256	0.437510312	0.106831	4.095338	4.22E-05	0.00013
10541	LOC65311	37.3907496	0.437388287	0.178118	2.455617	0.014064	0.026153
11998	CMAH	299.3513527	0.437195708	0.22297	1.96078	0.049905	0.081522
6494	ZNF222	64.09477306	0.436816992	0.108358	4.031256	5.55E-05	0.000167
12183	GNAL	72.42364246	0.436807538	0.229702	1.901624	0.05722	0.092061
5046	C16orf62	1258.162907	0.436790628	0.09196	4.749815	2.04E-06	7.91E-06
7279	CREB1	1125.123574	0.436566267	0.117721	3.708472	0.000209	0.000561
7173	GSPT1	2743.6191	0.436539676	0.116271	3.754509	0.000174	0.000475
7209	ADSS	991.3833823	0.436515525	0.11672	3.739845	0.000184	0.000501
5814	ORC2L	406.0743771	0.436484481	0.09996	4.366607	1.26E-05	4.25E-05
10097	SP4	241.7673835	0.436460068	0.166773	2.617084	0.008868	0.017216

12523	C18orf56	40.10939941	0.436383339	0.242872	1.796766	0.072373	0.113244
6633	PPAPDC2	243.3230025	0.436342112	0.109868	3.971518	7.14E-05	0.000211
6389	EIF3A	4605.921913	0.436331115	0.106942	4.08007	4.50E-05	0.000138
17010	AGTR2	0.668530295	0.436197611	0.996774	0.437609	0.66167	0.762457
6057	ARID4B	995.1639744	0.436133536	0.102826	4.241482	2.22E-05	7.19E-05
11700	PPM1K	326.3018583	0.436048506	0.212562	2.051392	0.040229	0.067395
9847	C16orf5	1586.808159	0.435911285	0.161132	2.705309	0.006824	0.013584
6865	C11orf58	3446.811006	0.435888289	0.112446	3.876438	0.000106	0.000303
11862	CREB5	863.8720499	0.435810729	0.217647	2.002372	0.045245	0.074763
5794	APP	30293.74733	0.435809073	0.099582	4.376397	1.21E-05	4.08E-05
11078	COX7B	2616.580776	0.4357498	0.192522	2.263374	0.023613	0.041779
15110	ATP5L2	0.615446218	0.43547525	0.447635	0.972835	0.330635	0.428907
10052	APEH	1615.884591	0.43544545	0.16526	2.634906	0.008416	0.016411
11301	TIAM2	45.36185026	0.435308058	0.199853	2.178137	0.029396	0.050986
8238	ESF1	429.8581894	0.435305987	0.131252	3.316575	0.000911	0.002168
11994	MAPK8IP1	353.346386	0.435217322	0.221766	1.96251	0.049703	0.081232
5989	KIAA1143	594.2109147	0.434986352	0.101769	4.274262	1.92E-05	6.28E-05
5389	RLIM	1465.535266	0.434973297	0.095127	4.572563	4.82E-06	1.75E-05
12490	FGF2	550.1802787	0.434926659	0.240753	1.806527	0.070836	0.111166
6743	RG9MTD2	111.3799321	0.4348758	0.110842	3.923383	8.73E-05	0.000254
4625	CWC25	467.9261724	0.434841435	0.087356	4.977817	6.43E-07	2.73E-06
7486	FERMT2	1624.196035	0.4348045	0.120082	3.620882	0.000294	0.000769
11982	TMEM204	785.1342207	0.434393844	0.221069	1.96497	0.049418	0.080848
10743	UQCRHL	346.236207	0.434276176	0.182223	2.383218	0.017162	0.031313
2085	SNX1	2494.616891	0.434270986	0.063265	6.864302	6.68E-12	6.28E-11
7283	ZNF571	90.19007023	0.434206719	0.117146	3.706558	0.00021	0.000565
6322	TAF9B	713.1711027	0.433901166	0.105425	4.115749	3.86E-05	0.00012
7537	SLC33A1	937.4241252	0.433800107	0.120475	3.600741	0.000317	0.000825
8957	ZNF57	79.89088441	0.433625836	0.143211	3.027891	0.002463	0.005389
7475	SLC25A13	963.0059114	0.433253138	0.119476	3.626282	0.000288	0.000754
7336	C14orf45	163.5188226	0.433167461	0.11749	3.686851	0.000227	0.000607
10885	GALNT12	309.9755595	0.433098668	0.186233	2.325576	0.020041	0.036087
10788	LOC283922	55.63158981	0.43309759	0.183048	2.36603	0.01798	0.032666
2865	KIAA0562	742.5846225	0.432504449	0.070528	6.132366	8.66E-10	5.92E-09
9592	REV3L	606.1924767	0.432297393	0.155363	2.782493	0.005394	0.011023
9807	TMEM106C	1781.724482	0.432242547	0.159227	2.714633	0.006635	0.013261
6393	ZNF225	89.75983254	0.432040585	0.105939	4.078196	4.54E-05	0.000139
6855	ZNF76	524.5056096	0.432034739	0.111383	3.878826	0.000105	0.0003
6223	SNX9	1751.467927	0.431838524	0.103606	4.168067	3.07E-05	9.68E-05
10601	SLFN13	854.2244594	0.431543498	0.177197	2.435383	0.014876	0.027505
11314	N4BP2	145.3323219	0.431437497	0.198418	2.174389	0.029676	0.051408
5567	HBS1L	769.231476	0.431132651	0.096035	4.489315	7.15E-06	2.52E-05
16387	CRX	0.762293317	0.43113008	0.714414	0.603474	0.546193	0.653279
13785	GCNT1	272.670934	0.430827046	0.309513	1.391954	0.163936	0.233103
8677	ADCY6	1358.634948	0.430641648	0.137144	3.140065	0.001689	0.003815
13438	DEPDC7	197.827261	0.430588995	0.286425	1.503322	0.132756	0.193641
14845	TEKT2	19.56731964	0.430540578	0.408867	1.053009	0.292337	0.385995

11315	RUNDC2C	32.76488147	0.43052365	0.197996	2.174406	0.029675	0.051408
11789	NOTCH4	2095.111143	0.43048913	0.212566	2.025203	0.042846	0.071239
5888	EPC2	594.542078	0.430472945	0.099455	4.328337	1.50E-05	5.00E-05
8920	LOC15078(	52.32216171	0.429983003	0.141253	3.044071	0.002334	0.005129
7298	KIF5B	3382.910975	0.42992019	0.116189	3.700177	0.000215	0.000579
6391	IMMT	2254.387913	0.429863683	0.105386	4.078926	4.52E-05	0.000139
6583	UBE2K	1808.85463	0.429758329	0.107699	3.990357	6.60E-05	0.000196
11105	C17orf76	61.82695582	0.429736236	0.190899	2.25112	0.024378	0.043029
6499	USPL1	464.1657133	0.429715122	0.106639	4.029638	5.59E-05	0.000168
6600	C5orf51	925.4815752	0.429369531	0.10778	3.983756	6.78E-05	0.000201
11729	ALCAM	1639.787441	0.429303993	0.210124	2.043101	0.041042	0.068588
5383	STARD7	3333.322471	0.429269696	0.093797	4.576568	4.73E-06	1.72E-05
14660	GBP7	7.846062805	0.429024119	0.385351	1.113334	0.265565	0.355071
10421	EDA2R	318.2731626	0.428852302	0.171367	2.502541	0.012331	0.023193
8767	RNFT1	290.4960055	0.42882816	0.138474	3.096814	0.001956	0.004373
5731	C17orf39	331.4809042	0.428785158	0.09729	4.40729	1.05E-05	3.58E-05
4865	FAM149B1	556.9444609	0.42868586	0.088305	4.854602	1.21E-06	4.86E-06
10087	TAF13	157.7078184	0.428642494	0.163481	2.621978	0.008742	0.016988
13611	FLG	62.52792197	0.428630368	0.295185	1.452071	0.146482	0.210946
6613	JAK1	6325.304519	0.428469446	0.107686	3.978884	6.92E-05	0.000205
15228	CHRNB4	5.707687755	0.428217615	0.455874	0.939334	0.347559	0.447338
9484	SH3BGRL	3862.233111	0.428194834	0.151595	2.824604	0.004734	0.009784
11489	UQCRC1	3375.245708	0.428173668	0.201575	2.124145	0.033658	0.057423
10811	UQCRH	1361.228611	0.427942578	0.181442	2.358559	0.018346	0.033262
8466	PBRM1	784.7518762	0.427939985	0.132882	3.220451	0.00128	0.002963
7641	ZC3H7B	2618.146468	0.427871296	0.120045	3.564246	0.000365	0.000936
7697	INTS2	364.0304225	0.427804951	0.120831	3.540511	0.000399	0.001017
8749	PGPEP1	1483.261795	0.427772722	0.137575	3.109372	0.001875	0.0042
4573	CYP20A1	581.9925507	0.42770108	0.085573	4.998093	5.79E-07	2.48E-06
4536	USP7	2260.545863	0.427544441	0.085238	5.015865	5.28E-07	2.28E-06
10893	ZNF660	17.28260027	0.427346076	0.183975	2.322843	0.020188	0.036328
6086	SIKE1	750.5707738	0.427201898	0.101066	4.226964	2.37E-05	7.63E-05
5733	ZBTB34	321.6731004	0.427127588	0.096936	4.406272	1.05E-05	3.60E-05
2362	MRFAP1L1	1260.168247	0.427125354	0.064744	6.597129	4.19E-11	3.48E-10
2734	C17orf80	538.0337002	0.426905505	0.068285	6.251792	4.06E-10	2.91E-09
7723	ZNF175	217.3444274	0.426868432	0.120873	3.531545	0.000413	0.001049
15259	TNIP3	57.1927935	0.42674137	0.458261	0.931219	0.35174	0.451829
7538	ANGEL1	503.8258377	0.426674566	0.118499	3.600673	0.000317	0.000825
11465	KCNE3	1778.357774	0.426673091	0.200319	2.129965	0.033175	0.056716
13525	C17orf46	2.042608691	0.426491345	0.288289	1.47939	0.139036	0.201497
8153	ZNF749	58.69650086	0.426394568	0.127314	3.349153	0.000811	0.001949
12659	GBP4	2613.132258	0.426389212	0.242985	1.754799	0.079294	0.122777
7550	FAM200A	215.1092802	0.426290764	0.118575	3.595114	0.000324	0.000842
7521	CHCHD4	248.5515496	0.426287609	0.11817	3.607415	0.000309	0.000806
8770	ZNF322B	26.7983549	0.426197874	0.137665	3.095895	0.001962	0.004386
6781	DUSP11	532.934029	0.426147272	0.109061	3.907417	9.33E-05	0.00027
5386	TPP2	1024.748874	0.425959927	0.093097	4.575462	4.75E-06	1.73E-05

6886	REXO2	1014.692185	0.42573882	0.110029	3.869322	0.000109	0.000311
8318	ELK4	174.058161	0.425662683	0.12963	3.283671	0.001025	0.002415
10408	TMX4	1556.89579	0.425286403	0.169637	2.507045	0.012175	0.022929
5758	CTBP2	1729.698762	0.425270416	0.09681	4.392823	1.12E-05	3.81E-05
13049	BMP2	541.4629631	0.425171392	0.260744	1.630608	0.102973	0.154676
16567	HRG	4.104307278	0.425150177	0.766231	0.554859	0.578991	0.684984
7184	KIAA1279	736.1187061	0.424971404	0.113318	3.750265	0.000177	0.000482
12615	ANO2	42.3376564	0.424932303	0.240472	1.767079	0.077215	0.119957
8001	FAM96A	979.549053	0.424771062	0.124585	3.409485	0.000651	0.001594
15824	KIR3DX1	0.516388678	0.424726882	0.553366	0.767534	0.442764	0.548447
8785	WDR6	2108.279772	0.424707321	0.1374	3.091028	0.001995	0.00445
9713	DUSP19	135.8511068	0.424264222	0.154614	2.744022	0.006069	0.012248
5538	RAD23B	3269.190552	0.424049734	0.094169	4.503061	6.70E-06	2.37E-05
6850	DCLRE1A	324.8084776	0.423890295	0.109261	3.8796	0.000105	0.000299
12602	LRRC36	31.52006202	0.423792657	0.239128	1.772243	0.076354	0.118761
9856	FOXRED1	440.6753449	0.423740698	0.156866	2.701291	0.006907	0.013737
11601	CACHD1	268.9828001	0.423256148	0.203473	2.08016	0.037511	0.063378
8013	LRRC8A	1697.338748	0.423076551	0.124228	3.405646	0.00066	0.001615
9309	GAS8	668.8692777	0.423049258	0.146044	2.896717	0.003771	0.00794
10197	MYCBP2	1443.629604	0.42295874	0.163785	2.582402	0.009812	0.01886
8439	NMD3	1340.095577	0.422939433	0.130895	3.231136	0.001233	0.002864
3649	NECAP1	838.0344877	0.422890258	0.075895	5.572049	2.52E-08	1.35E-07
15216	C11orf20	4.116409146	0.422805801	0.448136	0.943476	0.345437	0.444987
5033	ZNF416	112.2303369	0.422796395	0.088861	4.757933	1.96E-06	7.62E-06
9094	NDUFB5	1793.299732	0.42264597	0.141869	2.979132	0.002891	0.00623
4493	PIGY	1358.655056	0.42260235	0.083812	5.042256	4.60E-07	2.01E-06
13531	NOVA1	220.800896	0.422580572	0.286212	1.476458	0.139821	0.202545
6220	MDM1	242.9412264	0.422516313	0.10133	4.169708	3.05E-05	9.61E-05
7280	WDR47	429.7139182	0.422509255	0.113942	3.708119	0.000209	0.000562
4147	NEK9	1877.935892	0.422478384	0.080436	5.252371	1.50E-07	7.10E-07
9445	SPATS2L	3600.186458	0.422261596	0.14866	2.840449	0.004505	0.009349
10274	FAM151B	20.13196962	0.422229411	0.165253	2.555056	0.010617	0.020255
10329	NDUFA6	1541.71963	0.422049255	0.16635	2.537113	0.011177	0.02121
7577	FBXO28	1223.401489	0.422011674	0.117652	3.586935	0.000335	0.000866
7843	ACTR8	411.1622329	0.421856982	0.121301	3.47776	0.000506	0.001264
11127	SH3TC2	30.25273271	0.421739148	0.187961	2.243753	0.024848	0.043772
3191	SMU1	1384.00371	0.421555741	0.071594	5.88816	3.91E-09	2.40E-08
5709	PRDM10	205.6428032	0.421090311	0.095373	4.415208	1.01E-05	3.46E-05
7126	DDX59	407.9869365	0.421007115	0.111535	3.774651	0.00016	0.000441
6457	SEH1L	826.5189582	0.420878239	0.10402	4.046147	5.21E-05	0.000158
9140	PPAT	318.1499958	0.420827595	0.142098	2.961536	0.003061	0.006565
15231	DCDC2B	0.689851613	0.420542228	0.448038	0.93863	0.347921	0.447744
6844	PEX13	696.2251856	0.420385248	0.108291	3.881989	0.000104	0.000297
6512	NUFIP2	2496.911224	0.420209859	0.104456	4.022832	5.75E-05	0.000173
10364	ZNF501	55.25107032	0.420193996	0.166609	2.522036	0.011668	0.022067
7923	ATF1	487.5171293	0.420149572	0.122152	3.439555	0.000583	0.001441
9612	ZNF615	289.5770046	0.42014623	0.151395	2.775159	0.005517	0.011251

4055	OSBPL9	2148.316578	0.420088052	0.079224	5.302527	1.14E-07	5.52E-07
5353	AP3M2	539.2922759	0.419945649	0.091445	4.592305	4.38E-06	1.61E-05
13093	HSPA2	529.0998945	0.41964717	0.260149	1.6131	0.106723	0.15977
9394	SRF	1235.897244	0.419595309	0.146648	2.861232	0.00422	0.008805
4339	CUL4A	1585.806202	0.41930545	0.081618	5.137383	2.79E-07	1.26E-06
6586	STRAP	2352.278985	0.419136157	0.105071	3.989061	6.63E-05	0.000197
5591	ZNF271	647.217421	0.419029267	0.093617	4.475987	7.61E-06	2.67E-05
6115	CCDC47	2998.028903	0.418794941	0.09938	4.214092	2.51E-05	8.04E-05
14532	PAR1	1.671018804	0.418687606	0.36212	1.156213	0.247594	0.333959
7733	SLC25A36	1788.966883	0.418637279	0.118718	3.52633	0.000421	0.001068
9180	MAML3	354.788715	0.41853219	0.142047	2.946442	0.003215	0.006864
9570	IFIH1	789.7389478	0.418438955	0.149831	2.792745	0.005226	0.010704
7894	PARS2	131.6623536	0.418092649	0.12115	3.451044	0.000558	0.001387
11325	ARID5B	2102.334828	0.417949051	0.192469	2.171513	0.029892	0.051737
4591	UVRAG	649.7660685	0.417817803	0.083718	4.990804	6.01E-07	2.57E-06
8926	ZNF275	687.6641717	0.41751911	0.137242	3.042206	0.002349	0.005157
9768	RAB6C	65.86468434	0.417488722	0.153181	2.725464	0.006421	0.012885
4638	YY1	1268.725936	0.417131232	0.083926	4.970238	6.69E-07	2.83E-06
7392	HTT	1655.643607	0.417018715	0.113932	3.660236	0.000252	0.000668
4578	NUDT15	443.5417239	0.416871599	0.083418	4.997401	5.81E-07	2.49E-06
7379	ERICH1	349.6003757	0.41680235	0.113684	3.666315	0.000246	0.000654
6914	SKIV2L2	1436.147351	0.416781571	0.108041	3.857615	0.000114	0.000325
11303	LASS6	823.8371009	0.416708952	0.191367	2.177543	0.02944	0.051053
4285	USP10	1752.495332	0.416673731	0.080686	5.164113	2.42E-07	1.11E-06
10716	HDGFRP3	503.1385807	0.416668401	0.174302	2.390493	0.016826	0.030777
9102	SLC25A3	8401.73383	0.416553892	0.139981	2.975787	0.002922	0.006294
12397	NKAPL	17.08679934	0.416445884	0.226732	1.836735	0.066249	0.104747
14507	PRSS35	67.19476676	0.416168427	0.357468	1.164213	0.244338	0.330135
9613	BRP44	1991.00512	0.416013537	0.149912	2.775047	0.005519	0.011254
10082	KILLIN	43.05275384	0.415940532	0.158459	2.624918	0.008667	0.01685
4948	SIAH1	618.478718	0.415673256	0.086603	4.799769	1.59E-06	6.29E-06
12451	FAM22G	2.351439938	0.41563905	0.228399	1.819797	0.06879	0.108293
10156	SMAD6	151.8284442	0.415569924	0.160004	2.597249	0.009397	0.018137
7163	MFN1	692.1198599	0.415565148	0.110591	3.757687	0.000171	0.000469
8830	HACE1	269.3459308	0.415453097	0.134915	3.079359	0.002074	0.004605
13454	PAX2	1082.198579	0.415419708	0.276826	1.50065	0.133446	0.194416
9460	ARL13B	360.1646553	0.415379381	0.146562	2.834146	0.004595	0.00952
7906	TRMT11	227.4121014	0.415205612	0.120501	3.445666	0.00057	0.001412
3956	ARMC8	729.6346589	0.415142355	0.077279	5.372009	7.79E-08	3.86E-07
16050	POU6F2	0.543437696	0.415006969	0.595058	0.697422	0.485538	0.592962
3786	UBE4A	1895.813454	0.414839212	0.075693	5.480553	4.24E-08	2.20E-07
8712	GTF2H2C	394.1983347	0.414797515	0.1327	3.125836	0.001773	0.003989
7933	ZNF597	49.42097676	0.414707597	0.120738	3.434772	0.000593	0.001465
5877	RAB28	293.1737332	0.414633156	0.095666	4.334165	1.46E-05	4.88E-05
6290	SYNRG	1284.307358	0.414550187	0.100282	4.133858	3.57E-05	0.000111
8085	ARHGEF3	762.4484388	0.414485786	0.122739	3.37697	0.000733	0.001777
7270	NUP153	1224.595026	0.414459967	0.11159	3.714118	0.000204	0.00055

10801	SLC43A2	1417.435897	0.414337271	0.175517	2.360661	0.018242	0.033105
6498	PPP1R2	1204.71193	0.414328729	0.102821	4.029618	5.59E-05	0.000168
4520	ZNF187	336.2678171	0.414165129	0.082438	5.023984	5.06E-07	2.19E-06
8511	ZNF701	140.0976023	0.414095946	0.129369	3.200892	0.00137	0.003155
5099	ZNF557	155.3231987	0.414080529	0.087593	4.727316	2.28E-06	8.75E-06
12464	SCAMP5	821.5503058	0.413657183	0.228112	1.813392	0.069771	0.109723
7434	FAM10A4	432.4194587	0.413339747	0.113464	3.642919	0.00027	0.000711
7269	FBXL20	145.6294955	0.41322541	0.111246	3.714522	0.000204	0.000549
4452	FAM20B	1314.194302	0.41314777	0.081483	5.070325	3.97E-07	1.75E-06
13837	ALG1L	5.19699509	0.412963122	0.300986	1.372032	0.170053	0.240892
11050	ACBD4	475.8400367	0.412707996	0.181546	2.273293	0.023009	0.040803
14736	TMEM195	248.4718053	0.412680904	0.379105	1.088566	0.276345	0.367603
11549	IDH2	4149.279209	0.412640605	0.196766	2.097116	0.035983	0.061066
4180	TBC1D22B	503.1905161	0.41243712	0.078919	5.226066	1.73E-07	8.12E-07
15723	IQCF1	1.07767524	0.412408384	0.517204	0.797381	0.42523	0.53013
9011	C16orf87	161.8378318	0.412129613	0.137012	3.007983	0.00263	0.005721
10425	FOXN2	613.1860885	0.411885631	0.164635	2.501806	0.012356	0.023231
6394	ABCD4	817.9945585	0.411874463	0.100998	4.078048	4.54E-05	0.000139
11817	ME3	468.26296	0.411740277	0.20427	2.01567	0.043834	0.072709
10457	GAB2	927.1144725	0.411672791	0.165335	2.489929	0.012777	0.023949
9627	C13orf27	289.1024716	0.411481383	0.148573	2.769564	0.005613	0.011429
9521	GTF2IRD2B	158.4475045	0.41144352	0.146298	2.812367	0.004918	0.010124
10094	UTRN	3135.159006	0.411343806	0.157072	2.618825	0.008823	0.017134
11293	CPNE8	751.9654777	0.411301199	0.188654	2.180191	0.029243	0.050757
8423	TOMM70A	2745.78404	0.411289232	0.126874	3.241726	0.001188	0.002765
3752	UBR7	1225.451901	0.411269495	0.07469	5.506349	3.66E-08	1.91E-07
6938	CRYZL1	385.3701365	0.411118942	0.106821	3.848655	0.000119	0.000336
13396	SH3PXD2B	1083.127246	0.411091802	0.271794	1.51251	0.130404	0.190807
15512	ARL13A	0.45698919	0.411065874	0.480287	0.855875	0.392067	0.495417
16032	MCART3P	0.342433612	0.411002087	0.585361	0.702135	0.482595	0.590004
16455	C1orf157	0.253692568	0.411001843	0.701668	0.58575	0.558044	0.664735
16638	TRPV5	0.332108622	0.411001788	0.763067	0.538618	0.59015	0.695274
17058	VENTXP7	0.270461345	0.411001269	0.965679	0.425608	0.670393	0.770335
17247	FAM138B	0.309474709	0.411000931	1.08904	0.377398	0.705878	0.802175
17330	NEUROG2	0.236007161	0.411000702	1.140584	0.360342	0.718591	0.812798
17409	PATE2	0.163640771	0.411000301	1.211351	0.339291	0.734391	0.826859
17626	LOC144742	0.1357169	0.41099937	1.430118	0.287388	0.773815	0.860505
17710	FFAR1	0.160381377	0.410998887	1.550591	0.265059	0.790964	0.87537
17989	CYP2A7	0.080188807	0.410996119	1.984348	0.207119	0.835917	0.910824
18313	AQP12A	0.098906131	0.410988967	2.91407	0.141036	0.887841	0.950026
18317	OR4X2	0.077780641	0.410988722	2.91407	0.141036	0.887842	0.950026
15739	C11orf34	0.395624195	0.410882584	0.518284	0.792774	0.427909	0.532909
6895	NSL1	660.4851002	0.410708513	0.106244	3.865708	0.000111	0.000315
8470	XRN1	1214.18466	0.410336297	0.127455	3.219469	0.001284	0.002972
9909	LRRCC1	284.1583157	0.410064145	0.152694	2.685532	0.007241	0.014324
7033	GOLPH3L	970.0390872	0.409964089	0.107663	3.807829	0.00014	0.000391
5311	LOC144438	747.0814867	0.409455486	0.088733	4.614454	3.94E-06	1.45E-05

13069	RHBDL3	7.890712653	0.409365016	0.252284	1.622635	0.104667	0.156969
11400	E2F5	140.5432428	0.40915164	0.190339	2.149594	0.031587	0.054311
5016	NUFIP1	186.9392046	0.409135456	0.085884	4.763801	1.90E-06	7.42E-06
11392	ETS1	6018.996325	0.409135123	0.189942	2.154005	0.03124	0.053751
11193	RIPK4	841.9117201	0.409007089	0.183901	2.224067	0.026144	0.045783
14368	PKD2L2	1.012750431	0.408990277	0.338703	1.207519	0.227232	0.309993
8313	RSC1A1	167.6993198	0.408858825	0.124398	3.286706	0.001014	0.00239
10787	IL28RA	113.0800988	0.408505618	0.172651	2.366075	0.017978	0.032666
10298	HDHD3	522.9138539	0.408142224	0.160225	2.547301	0.010856	0.020663
6749	USP22	4415.04625	0.408124919	0.104138	3.919092	8.89E-05	0.000258
10208	FAM3C	1633.116449	0.408052256	0.158222	2.578986	0.009909	0.019027
9118	NIPA1	845.1785149	0.407836441	0.137306	2.970279	0.002975	0.006396
14102	COLEC10	6.946999953	0.407602204	0.316372	1.288362	0.19762	0.274681
14164	CD1E	18.28444568	0.407545991	0.321737	1.266706	0.205261	0.284047
3283	FAM82A2	965.3851252	0.407430576	0.069925	5.826654	5.65E-09	3.38E-08
12524	MAFF	904.7384901	0.407350405	0.226715	1.796755	0.072375	0.113244
7006	WARS2	430.2048666	0.407087892	0.106533	3.821225	0.000133	0.000372
4459	KLHL12	997.9132156	0.407073931	0.080395	5.063405	4.12E-07	1.81E-06
5484	OSBPL2	932.462429	0.406975906	0.089858	4.529081	5.92E-06	2.12E-05
8836	NHEJ1	464.8125719	0.406955511	0.132317	3.075613	0.002101	0.00466
5272	NUP133	924.7012327	0.406923995	0.087701	4.639914	3.49E-06	1.30E-05
7406	RBM41	172.2119508	0.406903382	0.111351	3.654237	0.000258	0.000683
8680	MED13L	2587.069014	0.406822935	0.12961	3.138827	0.001696	0.00383
6011	COQ7	537.098306	0.406633077	0.095445	4.260411	2.04E-05	6.65E-05
9071	ICK	556.2065144	0.406586621	0.135966	2.990352	0.002787	0.006021
9961	CMC1	155.393254	0.40653932	0.152402	2.667546	0.007641	0.015035
7646	REEP5	5945.370238	0.406450161	0.11409	3.562553	0.000367	0.000942
12078	ECT2L	6.031112407	0.406430204	0.210059	1.934836	0.05301	0.086029
7632	TOMM20	6054.888254	0.406303766	0.113915	3.566743	0.000361	0.000928
12952	TMEM56	361.5620785	0.406204244	0.245325	1.655778	0.097767	0.147956
10401	C14orf148	6.674279369	0.406133155	0.161944	2.507855	0.012147	0.022888
6234	TCEAL1	350.0835105	0.405938043	0.09754	4.161764	3.16E-05	9.93E-05
6019	RALBP1	1458.606544	0.405893363	0.095381	4.255496	2.09E-05	6.79E-05
6690	TTLL11	55.40625052	0.405877132	0.102904	3.944225	8.01E-05	0.000235
11681	LNP1	134.3178863	0.40580731	0.197405	2.055711	0.03981	0.066803
10799	CCDC134	79.59692953	0.405738647	0.171858	2.360898	0.018231	0.03309
9184	ZNF641	172.858301	0.40570752	0.137738	2.945503	0.003224	0.006882
6032	C22orf30	878.2039104	0.405661131	0.095416	4.251517	2.12E-05	6.90E-05
5925	DET1	128.1531539	0.405427511	0.094053	4.310632	1.63E-05	5.39E-05
7772	ZER1	1303.425832	0.405346651	0.115512	3.509123	0.00045	0.001134
16748	GPR123	0.593718695	0.405310765	0.802384	0.505133	0.613465	0.717969
5108	WDR61	797.8288947	0.40517082	0.085749	4.725086	2.30E-06	8.83E-06
11989	GPR56	8951.807915	0.404782698	0.206166	1.963385	0.049601	0.081094
6541	ARHGEF7	1781.537662	0.404575392	0.10094	4.008077	6.12E-05	0.000183
5639	SS18	1713.980811	0.404212556	0.090791	4.452103	8.50E-06	2.96E-05
7431	BRD3	605.1739953	0.404151673	0.110904	3.64416	0.000268	0.000708
8169	TRIM68	296.0978461	0.404033389	0.120748	3.34609	0.00082	0.001967

13615	RUNX1T1	38.27848915	0.403925605	0.278483	1.450451	0.146933	0.211533
13316	N4BP3	263.1527341	0.403805465	0.262497	1.538326	0.123969	0.182481
8445	PRMT6	368.3721895	0.403794182	0.125032	3.229521	0.00124	0.002878
6735	SKP1	6037.881974	0.403620678	0.102765	3.927615	8.58E-05	0.00025
8224	CREBZF	1341.749731	0.403587095	0.121481	3.322234	0.000893	0.002128
8748	KATNAL1	566.9297339	0.403293233	0.129703	3.109369	0.001875	0.0042
8786	DLG3	826.1578422	0.402994026	0.130393	3.090616	0.001997	0.004456
10257	NRIP1	1919.346779	0.402983743	0.15724	2.562859	0.010381	0.019839
12695	CDH13	1558.551207	0.402891702	0.232158	1.735422	0.082666	0.127636
7335	GPN3	446.311526	0.402815987	0.109251	3.687056	0.000227	0.000606
9164	ODC1	2324.741152	0.402789962	0.1364	2.953011	0.003147	0.006731
12210	FLT4	1406.057672	0.402709791	0.212762	1.892772	0.058388	0.093732
10789	RABGAP1L	289.5148196	0.402557255	0.170141	2.366027	0.01798	0.032666
7180	STX12	1677.906817	0.402406462	0.10725	3.752042	0.000175	0.000479
6932	ZC3H15	1367.071971	0.402367526	0.104495	3.850604	0.000118	0.000333
9189	CCDC76	261.9750904	0.402210913	0.136686	2.942593	0.003255	0.006943
5525	C17orf71	451.2439941	0.402174575	0.089228	4.507274	6.57E-06	2.33E-05
13784	USH1C	2584.962254	0.40201581	0.288746	1.392281	0.163837	0.232979
12382	BOK	953.8541483	0.401867794	0.218227	1.841513	0.065546	0.103762
4335	ANKRD40	1445.171186	0.401858254	0.078184	5.139936	2.75E-07	1.24E-06
9554	ARHGAP31	1228.378677	0.40174446	0.143483	2.799945	0.005111	0.010486
5963	ZBTB4	2418.429388	0.401558759	0.093702	4.285474	1.82E-05	5.99E-05
6754	PLAA	888.2251776	0.40151327	0.102489	3.917613	8.94E-05	0.00026
13362	PCDHGA2	43.32270303	0.401500544	0.26366	1.522796	0.12781	0.187487
12094	IKZF2	216.085473	0.401432699	0.207895	1.930936	0.053491	0.086694
8563	SPEN	1649.976909	0.401362101	0.125987	3.185744	0.001444	0.003305
9606	SWAP70	1955.807078	0.401304515	0.144455	2.778065	0.005468	0.011158
14054	LEAP2	28.40741253	0.401200158	0.308557	1.300248	0.193516	0.269886
15508	TDRD9	71.01856228	0.40116349	0.468094	0.857015	0.391437	0.494748
6832	DPH3	558.7399566	0.401154045	0.103196	3.887319	0.000101	0.000291
5638	KIAA0368	2321.217851	0.401104301	0.090093	4.452136	8.50E-06	2.96E-05
10705	AKR7A2	1350.849604	0.400952658	0.167354	2.395831	0.016583	0.030363
13626	TTC22	91.86225993	0.400902769	0.277058	1.446998	0.147898	0.212751
7157	HSF2	215.0661268	0.400835214	0.106541	3.762254	0.000168	0.000461
11850	VPS37D	145.7804155	0.400764954	0.199715	2.006681	0.044784	0.074076
14284	KLKB1	86.53325765	0.400669927	0.324611	1.234308	0.217088	0.297914
6264	ZW10	348.5974479	0.400506388	0.096503	4.150196	3.32E-05	0.000104
7814	KIAA0355	983.9089209	0.400440282	0.114532	3.496323	0.000472	0.001183
6375	EIF4G2	17167.83997	0.400263938	0.097855	4.090389	4.31E-05	0.000132
9285	HIVEP1	571.6083296	0.400229787	0.137745	2.905588	0.003666	0.007738
7795	FAM178A	803.7681978	0.400228373	0.114259	3.502807	0.00046	0.001158
5121	METT10D	702.3291335	0.400197519	0.084834	4.717432	2.39E-06	9.14E-06
6424	GOLGA5	1296.879438	0.400176371	0.09848	4.063525	4.83E-05	0.000147
10350	C13orf31	386.1694333	0.400035366	0.158212	2.528476	0.011456	0.021695
7807	DCAF17	395.7428932	0.400030732	0.114328	3.49897	0.000467	0.001173
7391	FAM116A	600.3960008	0.399918289	0.109239	3.660953	0.000251	0.000666
9808	SKAP2	2427.809693	0.399900642	0.147337	2.714192	0.006644	0.013277

13870	NR4A3	401.4096378	0.399672887	0.293541	1.361557	0.173338	0.24496
15255	KIAA1210	2.382014158	0.39941187	0.428653	0.931784	0.351448	0.451572
7160	TTC35	879.7026905	0.399398239	0.106217	3.760216	0.00017	0.000465
6594	HADHA	7389.874757	0.399036219	0.100132	3.985094	6.75E-05	0.000201
16300	LOC64376[illegible]	1.316064994	0.398974037	0.636579	0.626747	0.530825	0.638325
9334	PIGK	1143.78247	0.398863095	0.138275	2.88457	0.003919	0.008231
15917	CALB1	12.85393549	0.398766034	0.541708	0.736127	0.461653	0.568463
4726	RAB4A	674.0993037	0.39876552	0.081058	4.919528	8.68E-07	3.60E-06
12174	GPR89C	84.553107	0.398762395	0.209385	1.904447	0.056852	0.091536
3107	PRPF18	348.306203	0.398746835	0.066925	5.9581	2.55E-09	1.61E-08
7863	BCL9	435.0071852	0.398574885	0.114995	3.466005	0.000528	0.001317
9370	SLC46A1	831.9263124	0.398428019	0.138696	2.87267	0.00407	0.008514
10758	TTC32	73.37282606	0.398418477	0.167497	2.378656	0.017376	0.031659
9215	CCBL1	145.6077492	0.39826527	0.135776	2.933242	0.003354	0.007135
11037	FAM73B	333.6438509	0.397996887	0.174694	2.278252	0.022712	0.040334
3726	ANKRD27	658.3929805	0.397950891	0.072052	5.523135	3.33E-08	1.75E-07
10051	UHRF1BP1	645.2252463	0.397864404	0.15099	2.635046	0.008413	0.016406
12541	MACROD1	168.0099065	0.397742447	0.221739	1.793741	0.072855	0.113868
7961	CCNT2	821.8844921	0.397511759	0.116119	3.423306	0.000619	0.001523
12784	ROR1	78.27833461	0.397314861	0.232458	1.709188	0.087416	0.13403
7125	DDX31	229.0126548	0.397312843	0.105241	3.775255	0.00016	0.00044
10834	ZNF92	210.8746705	0.397057363	0.169082	2.348306	0.018859	0.03412
7090	HBP1	2039.206025	0.396837456	0.104748	3.788485	0.000152	0.000419
10450	C20orf177	205.1987852	0.396766404	0.159067	2.494336	0.012619	0.02367
7204	RREB1	981.5245036	0.396763446	0.106028	3.74207	0.000183	0.000497
7960	SEC23B	2049.077981	0.396726145	0.115887	3.423388	0.000618	0.001523
6439	KIAA0494	3934.982774	0.396602968	0.097833	4.053873	5.04E-05	0.000153
5993	ZNF548	309.4416415	0.396413958	0.092772	4.27299	1.93E-05	6.31E-05
10989	RASGEF1[illegible]	225.5386188	0.396388137	0.173057	2.290508	0.021992	0.039227
8229	BRWD1	1181.140296	0.39623906	0.119323	3.320715	0.000898	0.002139
7559	UCHL3	311.809865	0.396106588	0.110267	3.592263	0.000328	0.00085
10188	KIAA1522	1363.463834	0.395936755	0.153091	2.586288	0.009702	0.018663
9222	LRCH3	267.8329195	0.395836278	0.135103	2.929887	0.003391	0.007207
13492	TMEM37	3059.690717	0.395751773	0.265523	1.490462	0.136103	0.197729
7666	BAT2L1	3927.859736	0.395646938	0.111375	3.552383	0.000382	0.000976
11866	[illegible]NFRSF10[illegible]	488.7616875	0.395570857	0.197615	2.001723	0.045315	0.074853
8422	ZBTB1	902.5481084	0.395554899	0.122002	3.242208	0.001186	0.00276
7043	[illegible]1GALT1C	610.0875688	0.395535614	0.104004	3.803096	0.000143	0.000398
7970	HSPA1L	73.25977252	0.394953965	0.115437	3.42139	0.000623	0.001532
8726	ZNF28	406.1970885	0.394922827	0.126668	3.11779	0.001822	0.004093
4077	EXOC1	955.3530959	0.394863928	0.074607	5.29256	1.21E-07	5.80E-07
10490	CD58	406.2492964	0.394795724	0.159557	2.474331	0.013349	0.024942
11183	NACC2	57.14319854	0.394779318	0.177274	2.22695	0.025951	0.045485
6534	YME1L1	3106.004445	0.394766517	0.098425	4.010843	6.05E-05	0.000181
13878	GABRR2	5.337357689	0.394741921	0.291132	1.355888	0.175135	0.247357
9765	MOBKL1B	883.7448089	0.394669307	0.144783	2.725939	0.006412	0.01287
8793	IPO11	783.7947151	0.39463387	0.127809	3.087682	0.002017	0.004497

6958	LZIC	277.0773323	0.39460059	0.102685	3.842841	0.000122	0.000343
8915	THSD1P1	110.6760044	0.39457165	0.12954	3.045951	0.002319	0.0051
10281	RPH3AL	297.7257223	0.394466367	0.154559	2.552208	0.010704	0.020408
10337	NDUFA8	1015.172511	0.394440171	0.155659	2.534004	0.011277	0.021383
7575	KIFAP3	1118.174446	0.394322981	0.109906	3.58782	0.000333	0.000863
8093	RPE	748.0977423	0.394293145	0.116881	3.373468	0.000742	0.001798
11435	NCEH1	2242.473452	0.394268524	0.184392	2.138213	0.032499	0.055705
10823	SDR39U1	570.5114174	0.394222954	0.16751	2.353425	0.018601	0.033688
15549	FBXL21	48.86944918	0.394189562	0.46573	0.846391	0.397334	0.500865
11551	KCNC4	265.3930797	0.394119461	0.187939	2.097059	0.035988	0.061067
6720	PIAS1	412.805402	0.394053675	0.100196	3.932822	8.40E-05	0.000245
12201	C4orf38	36.90397625	0.394015633	0.207845	1.895719	0.057997	0.093173
14195	CAPS	191.0789527	0.393945159	0.312873	1.259121	0.207987	0.287196
6165	MRPS22	562.4486745	0.393911591	0.093922	4.194051	2.74E-05	8.71E-05
7344	RBM25	1518.424072	0.393813143	0.106904	3.683806	0.00023	0.000613
5821	PTCD3	991.651781	0.393812258	0.090285	4.361897	1.29E-05	4.34E-05
9898	TMEM68	419.1957455	0.393774446	0.146483	2.688185	0.007184	0.014227
14667	BIK	10.06161356	0.393757087	0.354368	1.111153	0.266502	0.35613
5320	ATP6V0A2	426.1410958	0.393637739	0.085346	4.612268	3.98E-06	1.47E-05
5510	AIMP1	1039.050317	0.393530271	0.087179	4.514063	6.36E-06	2.26E-05
8216	ZNF544	464.1504725	0.393509608	0.11833	3.325528	0.000883	0.002105
11860	SLC2A8	429.8619023	0.393412965	0.196435	2.002763	0.045203	0.074707
10538	MCM3APAS	14.00529691	0.39336597	0.160117	2.456738	0.014021	0.026079
7196	DUSP16	975.223896	0.393186821	0.104923	3.747393	0.000179	0.000487
11041	HPRT1	804.7049581	0.392898133	0.172616	2.276138	0.022838	0.040544
7873	C5orf42	506.162495	0.392897703	0.113459	3.462901	0.000534	0.00133
6105	UBQLN2	1320.887149	0.392791782	0.093093	4.21935	2.45E-05	7.87E-05
13389	PATL2	27.18630125	0.392559079	0.259181	1.514612	0.129871	0.190126
6750	RG9MTD3	138.7577414	0.392482839	0.100148	3.919032	8.89E-05	0.000258
14675	LASS3	1.170840714	0.392470083	0.353815	1.109251	0.267322	0.357055
13621	EMX1	155.2003362	0.392334504	0.270864	1.448458	0.147489	0.212241
9409	GLMN	108.4555569	0.39218369	0.13739	2.854529	0.00431	0.008978
12968	FBXO15	40.79035978	0.39210294	0.237249	1.652707	0.09839	0.148707
12193	PEAR1	508.2963347	0.392036919	0.206479	1.898676	0.057607	0.092607
15385	BTNL3	1.986587543	0.391601057	0.438076	0.893911	0.37137	0.473123
10080	THAP2	176.3927984	0.391464035	0.149116	2.625232	0.008659	0.016838
10937	MEIS1	275.5902714	0.391188043	0.169506	2.307806	0.02101	0.037653
16330	LOC93432	0.817711448	0.391153294	0.632058	0.618857	0.536011	0.643377
10090	C17orf108	318.57876	0.390893889	0.149156	2.6207	0.008775	0.017046
7517	HIAT1	1189.337709	0.390710578	0.108244	3.609548	0.000307	0.0008
4914	TUG1	3895.146794	0.390630356	0.08105	4.819626	1.44E-06	5.74E-06
11448	DOCK9	1716.910193	0.39051551	0.182885	2.135301	0.032736	0.05605
12488	CDON	583.9892006	0.390279108	0.215974	1.807067	0.070752	0.111051
7304	SMYD2	1319.130274	0.390232628	0.105514	3.698383	0.000217	0.000582
13114	PYROXD2	133.7089299	0.390180841	0.24283	1.606807	0.108097	0.161568
7288	TWISTNB	546.5853821	0.390150443	0.10531	3.704784	0.000212	0.000569
12577	AKAP2	1896.32483	0.390111892	0.219262	1.779205	0.075206	0.117207

9385	BCAP29	1477.685541	0.389989216	0.136121	2.865026	0.00417	0.008709
7544	CLTC	10903.86517	0.389946243	0.108369	3.598317	0.00032	0.000832
7849	DNMBP	621.142471	0.389913097	0.1123	3.472071	0.000516	0.00129
12437	EIF5A2	257.6280585	0.389861393	0.213613	1.825085	0.067988	0.107144
10847	ASB1	459.5128694	0.389795677	0.166574	2.34008	0.01928	0.034836
13020	FOXD4	17.19969854	0.389658651	0.237956	1.637522	0.101522	0.152836
7186	TRAF3IP1	468.8030745	0.389487317	0.103864	3.749961	0.000177	0.000482
13050	MAP6	74.85811011	0.389430212	0.238926	1.629923	0.103118	0.154882
6308	AGGF1	924.7235269	0.389345942	0.094463	4.121678	3.76E-05	0.000117
9672	RAP2A	1418.61076	0.389254811	0.141242	2.755935	0.005852	0.01186
11585	KHDRBS3	319.3610993	0.389045623	0.186394	2.087224	0.036868	0.062378
8388	FRYL	1544.171829	0.388953016	0.119497	3.254922	0.001134	0.00265
6167	KDM3B	2205.170272	0.388948179	0.092785	4.191927	2.77E-05	8.79E-05
8301	TNPO1	2739.47779	0.388920284	0.1181	3.293141	0.000991	0.002339
8484	NUP35	281.4975765	0.388914657	0.121043	3.213027	0.001313	0.003035
9680	ZBTB3	74.16272556	0.388744739	0.141167	2.753797	0.005891	0.011928
8954	TIMM8A	122.8286098	0.388597291	0.128221	3.030685	0.00244	0.005341
6996	ZZZ3	762.8430151	0.388208761	0.101477	3.825574	0.00013	0.000366
9009	TBC1D23	933.6104903	0.388079346	0.128994	3.008513	0.002625	0.005712
11343	NBPF3	98.05874176	0.387984598	0.178956	2.168039	0.030156	0.05211
4790	WRNIP1	855.7651593	0.387711624	0.079267	4.891226	1.00E-06	4.10E-06
7806	ZNF75A	252.2699576	0.387267867	0.11067	3.499313	0.000466	0.001171
5069	ANXA7	2994.747074	0.387106275	0.081704	4.737903	2.16E-06	8.35E-06
9880	FAM172A	747.1170926	0.386955275	0.143609	2.694508	0.007049	0.013985
6712	ZBTB11	637.486203	0.386854763	0.09829	3.935837	8.29E-05	0.000242
8233	CNNM3	1102.296613	0.386763924	0.116549	3.318463	0.000905	0.002155
9954	C9orf40	100.1153517	0.386309452	0.144732	2.669135	0.007605	0.014975
7990	PPIL4	588.7817557	0.386111034	0.113115	3.413447	0.000641	0.001574
10635	MYO5A	1136.181618	0.386061489	0.159467	2.420956	0.01548	0.02853
6447	ANGEL2	612.479625	0.385921321	0.095265	4.051032	5.10E-05	0.000155
6766	ANKRD42	326.3489963	0.38573005	0.098611	3.911619	9.17E-05	0.000266
7715	ZNF655	1454.126394	0.385674355	0.109126	3.534196	0.000409	0.001039
10942	TAF4B	170.2922302	0.385342638	0.167112	2.305892	0.021117	0.037827
8886	SNTB2	1001.372558	0.385274859	0.126011	3.057459	0.002232	0.004924
5909	HMGN4	1731.039174	0.385273193	0.089223	4.318116	1.57E-05	5.22E-05
6120	ZNF264	738.264981	0.385149568	0.091452	4.211515	2.54E-05	8.12E-05
13449	MAOB	4512.506484	0.385031411	0.256345	1.502005	0.133096	0.193978
5791	CDC5L	873.869814	0.384943346	0.087946	4.377064	1.20E-05	4.07E-05
11814	HCG11	336.0437743	0.384797872	0.19076	2.017183	0.043676	0.072465
8263	ARSD	2190.666951	0.384704413	0.116343	3.306653	0.000944	0.00224
12252	ATG4D	430.4358031	0.384645865	0.204406	1.881773	0.059867	0.095776
8348	DIP2B	1149.823592	0.384567024	0.117431	3.274825	0.001057	0.002482
6329	ARID1B	1196.391974	0.384564832	0.093504	4.112823	3.91E-05	0.000121
8644	HYLS1	93.64887118	0.384510945	0.121939	3.153318	0.001614	0.00366
5847	MGEA5	4173.866942	0.384494045	0.08843	4.347984	1.37E-05	4.61E-05
15482	GLTPD2	30.27063441	0.384210143	0.443582	0.866154	0.386406	0.489209
9986	CRNA0017	24.53365599	0.384001198	0.144493	2.657574	0.007871	0.015449

8106	IFT74	298.4058142	0.383967918	0.113994	3.368331	0.000756	0.001829
4936	CSTF2T	822.3394563	0.383954587	0.07989	4.806048	1.54E-06	6.11E-06
13262	ITM2A	685.7025507	0.383954561	0.246146	1.559867	0.118791	0.175572
11843	C19orf71	10.48233116	0.383723663	0.191075	2.008231	0.044619	0.073847
5224	CELF1	1730.36393	0.383716496	0.08222	4.666924	3.06E-06	1.15E-05
10222	NRL	30.27923807	0.383546073	0.149031	2.573603	0.010065	0.019299
14016	NLGN4X	122.1366549	0.383544298	0.291858	1.314148	0.188796	0.264027
9620	RPS6KA3	2044.205792	0.383491597	0.138329	2.772309	0.005566	0.011341
8895	TFDP2	275.5155988	0.383447879	0.125548	3.054183	0.002257	0.004973
7603	ZMYM5	285.4573264	0.383320136	0.1071	3.579095	0.000345	0.000889
13965	CLDN20	1.546574714	0.383289529	0.288114	1.330339	0.183407	0.257426
7515	MTHFD1	1688.903335	0.383147161	0.106104	3.611061	0.000305	0.000795
6927	MRPL39	454.3309975	0.383065027	0.099435	3.852414	0.000117	0.000331
10002	N4BP2L1	432.4446457	0.383003979	0.144415	2.652103	0.007999	0.015677
5580	RNF103	1041.025593	0.382762485	0.085398	4.482115	7.39E-06	2.60E-05
14189	LRRIQ1	38.98042755	0.382728332	0.303516	1.260981	0.207316	0.286391
6471	C6orf35	404.9121689	0.382627545	0.094676	4.041461	5.31E-05	0.000161
10185	CLPB	380.4899116	0.382563671	0.147871	2.587145	0.009677	0.018624
7959	GTPBP10	323.5790596	0.382543683	0.111732	3.423762	0.000618	0.001521
13052	B3GAT2	10.99787157	0.382484257	0.234741	1.629392	0.10323	0.155027
10965	SPRY2	541.4369584	0.382336554	0.16636	2.298248	0.021548	0.038519
8974	C1orf84	17.58527621	0.381404506	0.126188	3.022512	0.002507	0.005475
9023	BMPR1A	467.7796033	0.381329697	0.126932	3.004199	0.002663	0.005785
10239	C15orf61	96.52527135	0.381326612	0.148368	2.570141	0.010166	0.019461
14243	FIBIN	468.5980637	0.381141843	0.306451	1.243727	0.2136	0.293953
15351	C1orf116	89.57392628	0.381111055	0.421097	0.905044	0.365442	0.466616
10725	TMEM51	1058.815048	0.381070495	0.15958	2.387952	0.016943	0.030964
5629	C6orf64	501.9514374	0.381067262	0.085514	4.456181	8.34E-06	2.91E-05
9968	ARSK	311.3624239	0.381062883	0.14303	2.664225	0.007717	0.015174
13789	TNFRSF11I	686.0986518	0.381015195	0.27425	1.389297	0.164743	0.234188
8801	TXNDC9	598.6372718	0.380998296	0.123442	3.086466	0.002026	0.004511
7794	C5orf33	791.8413888	0.380992891	0.108767	3.502839	0.00046	0.001158
10418	ZNF354B	170.5014928	0.380985554	0.152093	2.504958	0.012247	0.023041
11431	ESRP2	393.7278335	0.380886121	0.178032	2.139423	0.032401	0.055559
9899	CEP135	216.1987591	0.380778903	0.141663	2.687913	0.00719	0.014237
9088	CETN3	343.5801369	0.380773538	0.127661	2.982692	0.002857	0.006162
14172	FGFR3	563.2251793	0.380481588	0.300843	1.264717	0.205973	0.284877
7798	PRMT5	1067.311679	0.380380982	0.108607	3.502361	0.000461	0.001159
15592	SOX21	1.047918867	0.380359527	0.454505	0.836866	0.402668	0.506201
7654	C10orf137	251.6028468	0.380344875	0.10691	3.557601	0.000374	0.000958
10294	EIF2AK3	905.8929356	0.380338257	0.14927	2.547992	0.010834	0.02063
8264	DNAJA3	1280.36794	0.380090467	0.114968	3.306064	0.000946	0.002244
15192	C16orf81	1.260044005	0.380049897	0.399705	0.950826	0.341693	0.440858
15250	CDR1	12.72723463	0.379786054	0.407292	0.932466	0.351096	0.451267
10136	ZSWIM6	548.9862501	0.379550071	0.145819	2.602892	0.009244	0.017876
6683	ZNF561	720.7745108	0.379534205	0.096143	3.947594	7.89E-05	0.000232
8498	TRUB2	617.275443	0.379464148	0.118308	3.207431	0.001339	0.003089

16639	FSHR	0.997528642	0.379408118	0.70463	0.53845	0.590266	0.695343
13331	CNTNAP3	91.32451986	0.379391747	0.247426	1.533357	0.125188	0.184068
16421	KRT72	0.717239467	0.37912139	0.634029	0.597956	0.549869	0.656354
9580	HINT2	474.7825916	0.379099106	0.135974	2.788017	0.005303	0.01085
12748	C11orf52	265.828356	0.37896885	0.220471	1.718907	0.085631	0.131665
5981	RBM26	701.5992217	0.378868243	0.088581	4.277085	1.89E-05	6.21E-05
8376	MCEE	133.8740208	0.378868049	0.116253	3.258994	0.001118	0.002616
7784	TADA2A	135.9192351	0.378751352	0.108024	3.506172	0.000455	0.001145
8590	C9orf64	560.2706619	0.378691493	0.119194	3.177101	0.001488	0.003394
11540	RHOU	924.8763756	0.378499402	0.180119	2.101386	0.035607	0.06048
11353	CTPS	557.651218	0.378362047	0.174738	2.165305	0.030364	0.052424
8940	SNX25	410.0824189	0.378054291	0.124552	3.03531	0.002403	0.005268
8821	MAGEH1	606.4336386	0.377925711	0.122628	3.081889	0.002057	0.004571
9760	ZHX3	1382.594585	0.377871443	0.138533	2.72766	0.006379	0.01281
7634	TP53BP1	979.27022	0.377710454	0.105909	3.566371	0.000362	0.000929
7848	MSH3	514.7552438	0.37753203	0.108718	3.472593	0.000515	0.001287
8174	RAD21	3331.40095	0.377492602	0.112873	3.344407	0.000825	0.001977
8002	FYTTD1	1691.692138	0.377267372	0.110663	3.409166	0.000652	0.001596
6775	UBE4B	941.7141495	0.377236137	0.096512	3.908707	9.28E-05	0.000268
10146	ANP32E	1621.590244	0.377182796	0.145075	2.599919	0.009325	0.018014
4600	MTMR1	594.017762	0.377144726	0.075605	4.988381	6.09E-07	2.59E-06
5797	PPP2R5C	1697.615321	0.377139247	0.086189	4.375711	1.21E-05	4.09E-05
6704	ARID2	832.467435	0.377058067	0.095702	3.939937	8.15E-05	0.000238
12878	CYP27A1	1506.775355	0.376882754	0.224132	1.681522	0.092661	0.141036
14730	FAM181B	5.886400102	0.376687043	0.345733	1.089532	0.275919	0.367162
6052	PSMC6	1117.65439	0.376681096	0.088791	4.242329	2.21E-05	7.17E-05
5246	HNRPDL	3543.914899	0.376583536	0.080937	4.652799	3.27E-06	1.22E-05
12808	NRIP2	492.9803673	0.376517128	0.221212	1.702066	0.088743	0.13581
7859	:AMSAP1L	1487.276747	0.376333214	0.108518	3.467924	0.000524	0.001308
9464	SNX12	211.1940387	0.376194929	0.132823	2.832307	0.004621	0.009571
9507	IQCK	533.4343685	0.376085602	0.133505	2.817011	0.004847	0.009994
9633	EPC1	416.6729106	0.376029085	0.135815	2.768678	0.005628	0.011453
16709	NEUROD2	0.458400562	0.375933718	0.727255	0.516922	0.605211	0.709986
7412	TCF20	969.0623507	0.375559116	0.102866	3.650946	0.000261	0.000691
9404	ING5	287.6873684	0.375305981	0.131365	2.85698	0.004277	0.008914
8352	ZNF546	105.1226287	0.375108055	0.114601	3.273157	0.001064	0.002496
6634	ZNF318	634.0189643	0.375016342	0.094452	3.970425	7.17E-05	0.000212
11854	TRIB2	1746.555972	0.375005825	0.187071	2.004621	0.045004	0.074415
7380	CRNA0018	225.8689984	0.374826244	0.102314	3.663504	0.000249	0.000661
3402	GGNBP2	1325.004022	0.374541757	0.065162	5.747886	9.04E-09	5.21E-08
10019	C12orf23	2102.222134	0.374307418	0.141482	2.645624	0.008154	0.015952
10243	KIAA1009	205.0176111	0.374160839	0.145706	2.567925	0.010231	0.019578
8287	KLF3	1326.3371	0.374068407	0.113409	3.298395	0.000972	0.0023
12308	CETP	171.9281325	0.374064863	0.200894	1.862002	0.062603	0.099698
9594	CP110	404.3114805	0.373987829	0.134478	2.781025	0.005419	0.011071
5937	DCAF7	2591.361015	0.373884475	0.086961	4.299469	1.71E-05	5.65E-05
13579	USHBP1	150.0217227	0.373781764	0.2558	1.461228	0.143953	0.207793

9610	PNP	1474.046243	0.373652379	0.13463	2.775406	0.005513	0.011245
10594	PEPD	2147.503655	0.373619507	0.15324	2.438134	0.014763	0.027315
10934	TNS1	16397.43524	0.37357124	0.161837	2.308325	0.020981	0.037612
14371	MOV10L1	8.633921667	0.373516968	0.309536	1.206698	0.227549	0.31036
12555	SELENBP1	947.5276902	0.373395054	0.208779	1.788474	0.0737	0.115061
9867	PTS	314.4180373	0.37333981	0.138379	2.697948	0.006977	0.013861
7657	USP32	1185.046253	0.373266102	0.104953	3.556497	0.000376	0.000962
4621	SF3B1	5885.995732	0.373147582	0.074932	4.979795	6.37E-07	2.70E-06
14909	GRM5	38.01424499	0.373115567	0.360854	1.03398	0.301145	0.395919
4857	VTI1A	316.7169264	0.373079501	0.076759	4.860376	1.17E-06	4.73E-06
11141	FUT11	503.4253278	0.372781486	0.166433	2.239836	0.025102	0.044163
10470	SMC2	525.3594165	0.372673288	0.149936	2.485541	0.012935	0.024217
11255	KDM6B	1107.558851	0.372608305	0.169487	2.198453	0.027917	0.048618
5017	DCAF8	2524.077555	0.372485137	0.078192	4.763741	1.90E-06	7.42E-06
5044	ELAC1	122.3301165	0.372462667	0.078398	4.750941	2.02E-06	7.87E-06
11649	RNF138P1	17.20872503	0.372430853	0.180688	2.061182	0.039286	0.066103
15456	C1orf92	2.187933724	0.372193574	0.426071	0.873549	0.382364	0.484907
13199	GOLGA6L5	50.3870374	0.372151277	0.235707	1.578873	0.114365	0.169837
11287	ARAP1	3027.555286	0.37207398	0.170445	2.182956	0.029039	0.050429
6898	RBM12	1657.745765	0.372011341	0.09624	3.865455	0.000111	0.000315
13345	PCDH17	860.9231492	0.371865844	0.24313	1.529492	0.126142	0.185277
11033	TNK1	181.1432193	0.371672382	0.163063	2.279311	0.022649	0.040237
10664	PITPNC1	554.1758401	0.371618121	0.154414	2.406633	0.0161	0.029593
13497	DNAH6	37.464504	0.371405525	0.249394	1.489235	0.136426	0.198109
10542	CENPQ	96.42626581	0.371294809	0.151212	2.455464	0.01407	0.026161
5699	NDNL2	476.3490547	0.37118652	0.083965	4.420728	9.84E-06	3.38E-05
12328	SLC46A3	963.546467	0.370795667	0.199764	1.856167	0.06343	0.100851
9214	SPTAN1	6221.002911	0.370505609	0.126291	2.93375	0.003349	0.007124
12566	ZMAT1	600.4611942	0.370502444	0.207763	1.78329	0.074539	0.116269
9111	PPP3CA	1002.539195	0.370483154	0.124541	2.974787	0.002932	0.006308
10554	ZNF717	41.65062595	0.370460648	0.151111	2.451587	0.014223	0.026415
8690	ATRN	1919.916914	0.370301578	0.118121	3.134939	0.001719	0.003877
12429	ATP6V0C	4786.24887	0.37024168	0.202649	1.827013	0.067698	0.106762
9117	LOC158572	38.69691361	0.370231256	0.12458	2.971824	0.00296	0.006365
6784	ZFAND3	1837.361249	0.370175945	0.094799	3.904869	9.43E-05	0.000272
10842	CTSO	2242.384512	0.370148055	0.157931	2.343732	0.019092	0.034516
10216	SMCHD1	993.4146178	0.370115526	0.143649	2.576524	0.00998	0.019148
12580	STARD10	767.4444533	0.370093267	0.208041	1.778941	0.075249	0.117247
4241	DDX1	2261.166295	0.370020894	0.071352	5.185851	2.15E-07	9.94E-07
11578	SLC25A24	1096.830027	0.369978215	0.17707	2.089447	0.036668	0.062076
5664	MTRF1L	338.6973866	0.3699031	0.083293	4.440997	8.95E-06	3.10E-05
12269	GIMAP8	1037.606014	0.369863431	0.19694	1.878054	0.060374	0.096465
6688	BBS2	944.6969722	0.369667595	0.093678	3.946163	7.94E-05	0.000233
13753	COLEC11	142.6955978	0.369649446	0.263634	1.40213	0.160876	0.229284
9382	EFCAB2	215.3965612	0.369629148	0.128966	2.86609	0.004156	0.008682
7481	HAUS3	299.0338394	0.36919489	0.101911	3.622711	0.000292	0.000764
9257	SAMD8	109.0042584	0.369141735	0.12665	2.91466	0.003561	0.00754

8487	KLHL20	456.3598525	0.369103803	0.114907	3.212193	0.001317	0.003042
12413	CSRNP1	1252.635709	0.369063987	0.201436	1.832162	0.066927	0.105683
7422	CHD6	1086.378525	0.369033954	0.101149	3.648425	0.000264	0.000697
10535	MLLT3	406.1426139	0.3690164	0.150067	2.459003	0.013932	0.025922
11035	TMEM144	360.6217412	0.368966551	0.161922	2.278674	0.022686	0.040297
6231	GATAD2B	789.2048456	0.368953842	0.088614	4.1636	3.13E-05	9.85E-05
13341	KCNIP4	197.0387824	0.368729873	0.240765	1.531491	0.125648	0.184606
13227	PLVAP	13052.1636	0.36869923	0.234501	1.572273	0.115887	0.171725
9348	FAM18B2	241.8484823	0.368400652	0.12796	2.879034	0.003989	0.008364
6484	ARL3	649.4135452	0.368395551	0.091313	4.034439	5.47E-05	0.000165
14048	ARHGEF16	615.0296489	0.368371044	0.282899	1.302129	0.192872	0.269112
16065	MAGEA12	1.00877342	0.368300154	0.531151	0.6934	0.488059	0.595492
6871	POLR1B	602.9480246	0.368287235	0.095039	3.875102	0.000107	0.000304
8230	ZNF8	84.69908592	0.368106942	0.110866	3.320279	0.000899	0.002142
8349	MLL3	1815.493893	0.368069107	0.112398	3.274706	0.001058	0.002483
9952	RASGRP3	822.9221143	0.368007004	0.13779	2.670787	0.007567	0.014904
14057	GDF15	1776.977584	0.367949164	0.283032	1.300026	0.193592	0.269943
5078	PPFIA1	1138.95096	0.367923672	0.077741	4.732696	2.22E-06	8.55E-06
10253	NFIA	1586.850742	0.367692571	0.143374	2.564575	0.01033	0.019749
13920	FAM27A	27.68761522	0.367642229	0.27362	1.343622	0.179071	0.252153
15887	CDH4	144.0239253	0.367534035	0.49221	0.746702	0.455243	0.561668
12592	FZD1	3042.451674	0.367521596	0.206971	1.775719	0.075779	0.11796
13598	ANKRD20I	9.570409556	0.367470519	0.252317	1.456385	0.145286	0.209424
11614	CTNNAL1	561.7294275	0.367447509	0.17694	2.076679	0.037831	0.063848
14957	MAB21L1	3.344793414	0.367273601	0.359742	1.020937	0.307284	0.402693
10103	NRAS	2056.613961	0.367240541	0.140403	2.61561	0.008907	0.01728
9578	MFAP3	1494.456062	0.367144554	0.131655	2.788687	0.005292	0.01083
8449	CNOT6	927.0538892	0.367088605	0.113734	3.2276	0.001248	0.002896
9098	C12orf51	1055.493905	0.366973438	0.123289	2.97654	0.002915	0.006281
11679	SIRT4	27.05000228	0.366525056	0.178264	2.056078	0.039775	0.066755
9852	YEATS4	256.1558229	0.366508559	0.135558	2.703711	0.006857	0.013642
11567	DYSF	2800.184017	0.366454693	0.175088	2.092979	0.036351	0.061599
4467	SCYL3	235.4165623	0.36637962	0.072433	5.058154	4.23E-07	1.86E-06
8215	ZFP62	511.9982968	0.366294887	0.110133	3.325934	0.000881	0.002103
11888	ITPK1	1844.640702	0.366265855	0.183847	1.992234	0.046345	0.076415
7384	TCEAL8	1279.125428	0.366244796	0.100003	3.66234	0.00025	0.000663
9711	URB2	225.7729685	0.366039531	0.133366	2.744622	0.006058	0.012228
12334	LOC151009	51.76604901	0.365865475	0.197378	1.853631	0.063792	0.101369
14581	C1orf125	1.793216149	0.365721661	0.320196	1.142182	0.253378	0.340612
10492	SLC30A6	337.009421	0.365565979	0.147766	2.473944	0.013363	0.024965
9730	ZNF37A	529.8799934	0.365530876	0.133487	2.73832	0.006175	0.01244
7900	ZUFSP	151.0771783	0.365363693	0.105959	3.448165	0.000564	0.0014
8481	DNAJC3	3037.071487	0.365260663	0.113634	3.214359	0.001307	0.003022
14913	PLCXD3	96.8887439	0.365223289	0.353887	1.032035	0.302056	0.397009
8056	PATL1	1422.095615	0.365139788	0.107672	3.39122	0.000696	0.001693
8725	MORC3	803.626595	0.365025123	0.117072	3.117949	0.001821	0.004091
11226	LOC650623	56.90666351	0.364989575	0.165082	2.210961	0.027039	0.04721

13976	C8orf77	10.95952313	0.364923423	0.2754	1.325069	0.185148	0.259666
8473	HSPA4	2401.031555	0.364867028	0.113367	3.218453	0.001289	0.002982
4954	C13orf23	678.1645838	0.364759023	0.076084	4.794147	1.63E-06	6.46E-06
13868	ADAM22	133.2147436	0.364676805	0.267795	1.361775	0.173269	0.244898
7720	WAPAL	1463.094207	0.364529879	0.103186	3.532751	0.000411	0.001044
14147	PLA2G4A	160.9501837	0.364509571	0.28652	1.272197	0.203303	0.281681
8322	C17orf68	489.6585019	0.36447611	0.11103	3.282693	0.001028	0.002422
14833	CDO1	18.91349988	0.364469538	0.344898	1.056747	0.290627	0.384048
11802	SDC4	9299.78406	0.364342528	0.180331	2.020414	0.04334	0.071981
7115	RNMT	921.6482994	0.364268626	0.096383	3.779383	0.000157	0.000433
14288	TTBK1	45.15851354	0.363959298	0.295109	1.233304	0.217462	0.298326
10633	SNX18	904.6991083	0.363954077	0.150302	2.421487	0.015457	0.028494
9327	CNOT7	1764.307802	0.363885937	0.125938	2.889398	0.00386	0.008112
7266	ZNF613	118.6519507	0.363856691	0.097896	3.716781	0.000202	0.000544
14486	NDRG4	142.8013192	0.363690908	0.310121	1.172738	0.240901	0.325963
11243	MGC3771	13.4081658	0.363600174	0.165128	2.201933	0.02767	0.04824
9791	C9orf123	715.9622282	0.363489689	0.133674	2.719234	0.006543	0.013099
8359	GRK4	54.67534215	0.363282618	0.111194	3.267098	0.001087	0.002548
9949	MYO18A	1274.150418	0.363230573	0.135944	2.671916	0.007542	0.014859
7054	DTWD1	302.3853085	0.363157877	0.095594	3.798954	0.000145	0.000404
14529	C1orf150	2.464747126	0.363152601	0.313974	1.156634	0.247422	0.333796
5999	KPNA1	1498.202436	0.363074278	0.085066	4.268153	1.97E-05	6.44E-05
9755	OC1001293	76.93309253	0.363050823	0.132949	2.730748	0.006319	0.012697
11704	ORC5L	400.6511719	0.362988501	0.177005	2.050724	0.040294	0.067481
8853	MRE11A	367.6706612	0.362952175	0.118276	3.0687	0.00215	0.00476
15502	NAT2	10.61177421	0.36286684	0.421861	0.860156	0.389703	0.492747
14482	CCDC28B	160.8383821	0.362833293	0.309218	1.17339	0.240639	0.325699
11698	GYG1	1182.8786	0.362781416	0.17681	2.05181	0.040188	0.067339
15274	DNAH12	2.374960722	0.362605947	0.39123	0.926835	0.354012	0.454301
12755	ACADS	1364.690684	0.362591249	0.2112	1.716818	0.086012	0.132169
9306	ABCB7	510.2536239	0.362589772	0.125114	2.898073	0.003755	0.007908
15403	EVPLL	10.03272574	0.362522992	0.407435	0.889769	0.37359	0.47541
6567	ESD	2455.2519	0.362450172	0.090673	3.997312	6.41E-05	0.000191
12699	ENDOG	208.1853445	0.36219525	0.208782	1.7348	0.082776	0.127766
7832	CCDC28A	622.1633618	0.361886476	0.103834	3.485236	0.000492	0.001231
9166	TMTC3	1146.540765	0.361541261	0.122469	2.952104	0.003156	0.006749
16089	PROM1	761.1008738	0.361518208	0.527696	0.685089	0.493288	0.600966
10413	INTS4L2	9.907894301	0.361497993	0.14424	2.506224	0.012203	0.02297
6496	ATXN2	864.8938966	0.361320689	0.08965	4.030334	5.57E-05	0.000168
8731	OSCP1	191.7665474	0.361313597	0.115958	3.115898	0.001834	0.004117
13013	GTF2H2	159.3349651	0.361300089	0.220325	1.639848	0.101037	0.15219
8510	EML4	2128.312986	0.361295798	0.112862	3.201211	0.001369	0.003152
10825	SLC25A16	397.8482127	0.361269635	0.153536	2.353	0.018623	0.03372
9846	ZNF283	52.16547547	0.361092798	0.133457	2.705683	0.006816	0.01357
8687	CAPN2	5696.948644	0.360846465	0.115064	3.136049	0.001712	0.003864
9110	SMCR7	367.5056017	0.360771156	0.121273	2.974865	0.002931	0.006307
13520	ADAM21	2.883228064	0.360706126	0.24349	1.481403	0.138499	0.200793

9274	TNRC6A	1456.2574	0.360648464	0.124032	2.907702	0.003641	0.007695
11336	TTC38	1469.857659	0.36058597	0.166197	2.169632	0.030035	0.051933
8779	COPB1	3624.031064	0.360295934	0.116491	3.092907	0.001982	0.004425
7343	C21orf33	2082.083586	0.36006736	0.097742	3.683838	0.00023	0.000613
6614	MRPL30	839.9522899	0.360005087	0.090498	3.978042	6.95E-05	0.000206
7927	EPS15	1903.299946	0.359917777	0.10469	3.43794	0.000586	0.001449
13836	CNTN4	155.3747886	0.359917421	0.262234	1.372503	0.169907	0.240702
12496	HOXC10	790.9065834	0.359892485	0.199312	1.805674	0.070969	0.111326
8675	ANKRD49	276.772963	0.359890017	0.11458	3.140939	0.001684	0.003805
12680	EPHA2	1130.903547	0.35975887	0.206217	1.744561	0.081061	0.125306
13713	C9orf117	4.70252053	0.359683666	0.254282	1.414509	0.157212	0.224715
13600	RNASE4	1813.735248	0.359588387	0.24702	1.455704	0.145474	0.209665
12776	DNM3	105.0661277	0.359491756	0.210016	1.711735	0.086945	0.133382
14042	KCNAB1	294.4605751	0.359159218	0.275139	1.305372	0.191766	0.267683
8754	MMADHC	1747.440904	0.359077985	0.115667	3.10441	0.001907	0.004269
7833	MLLT10	587.0586745	0.359029969	0.103027	3.484822	0.000492	0.001232
7447	DYM	608.7121039	0.35900739	0.098681	3.638068	0.000275	0.000723
16644	TRPC7	2.52445973	0.358845797	0.668383	0.536887	0.591346	0.696428
10297	FAM107B	4077.593943	0.358835202	0.140869	2.547302	0.010856	0.020663
8620	IRGQ	654.8324553	0.358273012	0.113106	3.16759	0.001537	0.003495
5696	VAPB	1173.994443	0.358260183	0.081015	4.422157	9.77E-06	3.36E-05
12626	SH3RF3	385.9384503	0.358179049	0.202946	1.764902	0.07758	0.120437
10588	PRMT3	319.811836	0.357991629	0.146724	2.439902	0.014691	0.027197
14475	TMPRSS5	5.217315326	0.357979307	0.30447	1.175745	0.239697	0.32458
7655	UBAP2	651.0016631	0.357851114	0.100595	3.557343	0.000375	0.000959
11771	PIM3	2055.631235	0.357764366	0.176275	2.029584	0.042399	0.070602
14120	CPA6	12.85643181	0.357763744	0.279178	1.28149	0.200022	0.277665
14217	HYDIN	154.3161373	0.35764614	0.285713	1.251766	0.210655	0.290431
10533	NDUFA9	1721.316418	0.357593796	0.145392	2.459519	0.013912	0.02589
10040	C9orf46	282.8347273	0.357268091	0.135454	2.637564	0.00835	0.016302
6403	COG2	637.8695224	0.357231213	0.087651	4.075594	4.59E-05	0.000141
10610	SLC36A4	244.6500603	0.357124913	0.146765	2.433308	0.014962	0.02764
4907	TRA2B	2002.125815	0.357072982	0.074029	4.823435	1.41E-06	5.64E-06
6804	ZNF638	2123.639011	0.357012451	0.091578	3.898441	9.68E-05	0.000279
14867	HNF4G	572.0534239	0.356915028	0.341026	1.046592	0.295288	0.389314
9538	TRIM32	374.9954585	0.356786973	0.127283	2.803101	0.005061	0.010401
7969	DDB1	5491.120184	0.356730402	0.104262	3.421473	0.000623	0.001532
12000	ZNF681	77.56755484	0.356567189	0.181882	1.960434	0.049945	0.081581
9076	VTA1	842.7170655	0.356056523	0.11918	2.987549	0.002812	0.006074
8670	PJA1	504.6634081	0.355897068	0.11322	3.143412	0.00167	0.003775
7574	NT5C2	1112.269349	0.35546302	0.099073	3.587888	0.000333	0.000863
10201	TSC22D2	653.3843284	0.355398648	0.13765	2.581904	0.009826	0.01888
11186	CTBS	917.8275291	0.355360434	0.15966	2.22573	0.026032	0.045616
12943	ESR1	131.911343	0.355035999	0.213665	1.661648	0.096583	0.146267
9044	ADK	222.2812708	0.35462501	0.11829	2.997931	0.002718	0.005891
7299	AP3B1	1450.089806	0.354545269	0.095822	3.700026	0.000216	0.000579
15720	IL1RAPL1	1.124218938	0.354448743	0.444171	0.798001	0.42487	0.529749

10165	CHORDC1	406.4865726	0.354446632	0.136625	2.594294	0.009479	0.018277
16667	C11orf87	0.919220285	0.354306533	0.668671	0.529866	0.596205	0.701193
12410	CRYBG3	415.8358971	0.354280166	0.19333	1.832512	0.066875	0.10562
9850	EDEM3	1306.342316	0.354191851	0.130991	2.703951	0.006852	0.013635
8102	MLH3	529.2844201	0.35414583	0.105069	3.370605	0.00075	0.001815
5561	MTMR3	1188.112455	0.354137941	0.078833	4.492265	7.05E-06	2.48E-05
8783	PTCD2	127.9788494	0.353783589	0.114426	3.091822	0.001989	0.00444
7955	ATXN7	644.8500558	0.353776817	0.103307	3.42451	0.000616	0.001518
6060	FBXO38	915.0054971	0.353659654	0.083426	4.239214	2.24E-05	7.26E-05
14036	HCG27	42.51537926	0.353529745	0.270362	1.307619	0.191003	0.266732
10032	CCDC7	47.87940808	0.353492008	0.133854	2.640873	0.008269	0.016157
12043	CPNE2	1220.192707	0.353416464	0.18157	1.94645	0.051601	0.083985
16563	SLC28A2	0.28452586	0.353294209	0.635336	0.556075	0.57816	0.684206
17273	DDI1	0.218755594	0.353293519	0.945464	0.373672	0.708648	0.804158
17335	ARGFX	0.278141908	0.353293505	0.983285	0.359299	0.719371	0.813406
17347	LOC28635	0.203222441	0.353293352	0.998634	0.353777	0.723506	0.817476
17430	C8orf12	0.19330901	0.353293131	1.065607	0.331542	0.740235	0.832436
17531	PRB3	0.230406609	0.35329295	1.148803	0.307531	0.758439	0.847993
17582	OC1001019	0.289311771	0.353292946	1.189309	0.297057	0.766423	0.854434
17617	TRIML1	0.14644762	0.35329256	1.218005	0.290058	0.771772	0.858687
17822	CAPZA3	0.415469087	0.353292289	1.46222	0.241614	0.80908	0.889842
17808	C15orf60	0.111752358	0.353291573	1.442693	0.244883	0.806547	0.887739
18013	PRKACG	0.109919534	0.353290167	1.735724	0.20354	0.838713	0.912703
18122	FIGLA	0.088225903	0.353288772	1.965552	0.17974	0.857356	0.927328
18200	ZP2	0.09418208	0.353287359	2.186964	0.161542	0.871666	0.938765
18208	GIF	0.088638198	0.353287238	2.204842	0.160232	0.872698	0.939474
18230	KRTAP5-1	0.070089105	0.353286676	2.265113	0.155969	0.876058	0.941374
18441	CYLC1	0.090737435	0.353281655	2.91407	0.121233	0.903506	0.96008
18439	BSPH1	0.072356485	0.35328141	2.91407	0.121233	0.903507	0.96008
18440	C3orf79	0.074239866	0.35328141	2.91407	0.121233	0.903507	0.96008
18442	DEFB129	0.054627209	0.353281165	2.91407	0.121233	0.903507	0.96008
18444	GOLGA6D	0.05129112	0.353281165	2.91407	0.121233	0.903507	0.96008
8177	SLBP	845.2678804	0.353231111	0.105652	3.343343	0.000828	0.001984
12600	GIMAP7	710.6201688	0.352992364	0.199086	1.773064	0.076218	0.118558
8123	MTOR	1097.225641	0.352978231	0.105032	3.360685	0.000777	0.001876
12421	SLC27A4	647.5370911	0.352840825	0.19283	1.829804	0.067279	0.106171
8293	POMT2	461.93165	0.352818	0.107011	3.297018	0.000977	0.00231
8030	CALCOCO	1910.865179	0.352774735	0.103769	3.399625	0.000675	0.001647
12419	MPZL3	22.3473015	0.352633829	0.192703	1.829937	0.067259	0.106156
15672	HIST1H2BI	1.642793446	0.352581694	0.432272	0.815648	0.414702	0.518668
9031	MYNN	465.6383073	0.352565576	0.117457	3.001655	0.002685	0.005828
10616	TMEM177	195.0449146	0.352547281	0.145101	2.429662	0.015113	0.027904
10346	NEURL4	443.3685581	0.352532538	0.139315	2.530466	0.011391	0.021581
9813	TIFA	277.0057118	0.35234787	0.129855	2.713405	0.00666	0.013302
9919	TXNIP	20774.87659	0.35232786	0.131437	2.680586	0.007349	0.014523
9418	PSMD5	729.0100327	0.352182372	0.123502	2.851637	0.004349	0.009052
11148	MAPKAPK	1181.0469	0.352125748	0.157367	2.237615	0.025246	0.044389

12482	ETV3	127.2419636	0.352125185	0.194755	1.808043	0.0706	0.110857
10739	PSKH1	870.6945682	0.351603751	0.147471	2.38423	0.017115	0.031238
13284	GMCL1L	4.19671139	0.351582958	0.226954	1.549135	0.121349	0.179055
8538	PHF20L1	851.8933736	0.351480324	0.110074	3.193124	0.001407	0.003231
7468	SRBD1	409.980179	0.351319251	0.096814	3.628821	0.000285	0.000747
10489	CRADD	277.7734261	0.351276431	0.14192	2.475173	0.013317	0.024886
8713	ZNF160	861.81582	0.351185235	0.112361	3.125508	0.001775	0.003993
9025	NCOA3	1307.507536	0.351087799	0.11688	3.003821	0.002666	0.00579
13771	C1orf220	10.74051262	0.351073024	0.25155	1.395637	0.162824	0.231756
13952	LOC38764(	40.66880414	0.351039564	0.263185	1.333813	0.182265	0.256062
4627	ATMIN	1549.588198	0.350827406	0.070505	4.975935	6.49E-07	2.75E-06
14696	PURG	9.946737883	0.350606787	0.318522	1.100732	0.271013	0.361468
7839	PNPT1	540.2676235	0.350319004	0.100678	3.479599	0.000502	0.001256
8530	SLC35B4	940.3434952	0.350257524	0.109621	3.195166	0.001398	0.003211
10100	SEC62	5555.8557	0.350170022	0.133829	2.61655	0.008882	0.017239
11181	CLIP1	2178.376164	0.349848402	0.156941	2.229168	0.025803	0.045234
9302	BCAS3	630.1136312	0.34938813	0.120463	2.900382	0.003727	0.007854
7838	AHCTF1	1279.132005	0.349309843	0.100383	3.479784	0.000502	0.001255
8545	PPP2R2A	872.180664	0.34918676	0.109413	3.191467	0.001416	0.003247
7578	PURB	1901.160713	0.349144837	0.097342	3.586787	0.000335	0.000866
15024	ALS2CL	301.5546621	0.349095905	0.349345	0.999287	0.317656	0.414428
9921	CHD1	1035.446124	0.34893541	0.130212	2.679756	0.007368	0.014556
14214	C22orf24	1.739238587	0.348794608	0.278291	1.253347	0.21008	0.289698
8849	CEP76	80.75836034	0.348773532	0.113573	3.070925	0.002134	0.004727
10229	PDSS1	92.15480009	0.34873511	0.135588	2.572027	0.01011	0.019374
11731	AIG1	1717.055624	0.3485804	0.170655	2.042598	0.041092	0.06866
15449	RGAG1	5.276017673	0.348099117	0.398027	0.874562	0.381812	0.484446
15620	GALNT8	0.913092044	0.347986828	0.419395	0.829736	0.406688	0.510339
15721	SCN2B	5.560283405	0.347927428	0.436013	0.797974	0.424886	0.529749
15998	NXF5	0.802472559	0.347912568	0.487526	0.713628	0.475457	0.582537
9273	HIC2	143.6789902	0.347642671	0.119546	2.908022	0.003637	0.007688
5766	C3orf17	949.3280117	0.347619002	0.079207	4.388721	1.14E-05	3.88E-05
10579	SASH1	1604.300438	0.347200601	0.142171	2.442133	0.014601	0.027053
7801	NIPBL	1560.884233	0.346562705	0.09897	3.501679	0.000462	0.001162
8994	AP4E1	353.3399175	0.346540918	0.11496	3.014461	0.002574	0.00561
8710	CDS2	1109.591462	0.346524542	0.11083	3.126642	0.001768	0.003979
8861	EPS15L1	781.8754549	0.346422818	0.11299	3.065956	0.00217	0.0048
10127	SCCPDH	1206.946001	0.346418161	0.132904	2.606534	0.009146	0.017703
12616	C20orf160	205.880797	0.346396764	0.196023	1.767123	0.077208	0.119957
9157	DCUN1D2	186.8082476	0.346328432	0.117156	2.956136	0.003115	0.006668
8953	DNAJC24	360.9650644	0.345985596	0.114153	3.030885	0.002438	0.005338
12300	KLF7	194.5835123	0.345940847	0.185393	1.865991	0.062043	0.09887
10440	PRPF39	484.6857259	0.345920725	0.1385	2.497621	0.012503	0.023474
8259	RPAP3	452.6961273	0.345603548	0.104448	3.308845	0.000937	0.002223
7857	ZMYM6	475.7503587	0.345392402	0.099577	3.468583	0.000523	0.001305
9212	LRIG2	188.5368156	0.345133771	0.117621	2.934283	0.003343	0.007114
10422	MIER1	1200.94452	0.345099228	0.137903	2.502478	0.012333	0.023195

16331	FABP2	1.039184784	0.344944802	0.55815	0.618015	0.536565	0.644003
4402	INTS12	333.6223862	0.344478176	0.067514	5.102314	3.36E-07	1.49E-06
7315	STAT5B	2161.519083	0.344408974	0.093207	3.69509	0.00022	0.000589
14680	TSHR	16.148329	0.344370311	0.310749	1.108195	0.267777	0.357541
9038	LPIN2	1504.858769	0.344328317	0.114798	2.999434	0.002705	0.005866
6109	SLC25A17	508.6362116	0.344013949	0.081566	4.217621	2.47E-05	7.92E-05
14218	LOC40105[illegible]	29.69647629	0.343990337	0.274846	1.251576	0.210724	0.290506
8854	PHF10	1170.76583	0.343791113	0.112034	3.068637	0.00215	0.004761
12883	C6orf201	11.00160249	0.343680269	0.204692	1.679009	0.09315	0.141725
4511	KPNA6	1718.429124	0.343370488	0.068278	5.029007	4.93E-07	2.14E-06
11476	SLC3A2	3969.407418	0.343364988	0.161375	2.127746	0.033358	0.056976
6356	DYNC1I2	1964.590068	0.343360137	0.083793	4.097708	4.17E-05	0.000129
14772	SDK2	34.17529251	0.343265259	0.31884	1.076605	0.281657	0.373731
3959	GPBP1L1	2139.206961	0.343245697	0.063908	5.370908	7.83E-08	3.88E-07
5590	DDX20	379.7397828	0.343168204	0.076633	4.478097	7.53E-06	2.64E-05
16171	ANGPTL5	1.023408279	0.343163492	0.518686	0.661601	0.508227	0.616026
13895	LOC44166[illegible]	49.43176086	0.343072363	0.254172	1.349766	0.177091	0.249814
9962	ZNF397OS	366.8547102	0.343047137	0.12861	2.667342	0.007645	0.015043
7607	RBMXL1	501.0382804	0.343037695	0.095878	3.577841	0.000346	0.000893
9383	SMURF2	414.5690056	0.342989997	0.119682	2.865853	0.004159	0.008688
7032	TBRG1	904.4107532	0.342783202	0.090018	3.807961	0.00014	0.000391
15101	GIPR	6.996176146	0.342766604	0.351048	0.97641	0.328861	0.42686
11760	JAK2	524.2097435	0.342753237	0.168504	2.034099	0.041942	0.069906
14949	GGT3P	271.6968519	0.342488445	0.334867	1.022761	0.306421	0.401777
5250	SFRS1	3530.800183	0.342460105	0.073616	4.651959	3.29E-06	1.23E-05
10399	PLCD1	230.6819631	0.342404326	0.13652	2.508084	0.012139	0.022882
11393	FICD	104.2275895	0.342397001	0.158975	2.153777	0.031258	0.053777
7620	ALG13	581.5733715	0.342324889	0.095856	3.571223	0.000355	0.000914
11966	C16orf45	264.9464099	0.342324042	0.17379	1.96976	0.048866	0.080045
6357	OAZ2	2733.128568	0.342242315	0.083521	4.097676	4.17E-05	0.000129
8095	UBE2G1	1033.327362	0.342184777	0.101435	3.373449	0.000742	0.001798
12762	BSG	19325.42043	0.34207013	0.199439	1.715162	0.086316	0.132571
9317	MTAP	497.9478548	0.341993151	0.118166	2.894178	0.003802	0.007998
9018	ZNF785	194.870661	0.341836491	0.113706	3.006312	0.002644	0.005748
9820	SEMA4D	1186.630753	0.34181505	0.126094	2.710803	0.006712	0.013398
9119	EIF2C3	123.459923	0.341804063	0.115083	2.970075	0.002977	0.0064
12660	SYT15	60.82561824	0.34163903	0.194802	1.753773	0.079469	0.12304
7630	RAB2B	531.9927622	0.34145638	0.095722	3.567165	0.000361	0.000927
15107	NPM2	34.92243625	0.341365908	0.350658	0.973501	0.330304	0.428563
14706	TEX12	1.17846433	0.341174919	0.311031	1.096917	0.272678	0.363441
10665	USP1	827.0818804	0.340888729	0.141686	2.40594	0.016131	0.029647
8354	TM2D2	1159.825213	0.340882195	0.10422	3.270796	0.001072	0.002516
8325	GPATCH2	257.8560931	0.340866919	0.103863	3.281895	0.001031	0.002428
9433	UGGT2	496.5424909	0.340650859	0.119738	2.844979	0.004441	0.00923
13824	DNAJC22	430.3089683	0.340515795	0.247069	1.378219	0.168136	0.238399
8311	PRPF4	708.8693729	0.340501739	0.103545	3.288445	0.001007	0.002376
8173	VMA21	1450.298334	0.340472956	0.101793	3.344757	0.000824	0.001975

6959	INO80	740.9408317	0.340314887	0.08857	3.842313	0.000122	0.000343
9132	ZNF250	227.2853418	0.340271694	0.114758	2.965133	0.003026	0.006494
8430	CCDC111	169.104801	0.340263865	0.105141	3.236265	0.001211	0.002816
7509	EID2	248.1706046	0.340237522	0.094174	3.612873	0.000303	0.00079
6441	NGDN	394.9881106	0.340150369	0.083915	4.053516	5.05E-05	0.000154
16528	COL20A1	0.696729717	0.339969674	0.601872	0.564854	0.572173	0.678556
15314	FAM65C	356.6848098	0.339886066	0.370218	0.91807	0.358582	0.458964
9138	ZNF155	169.2559913	0.339638287	0.114616	2.963281	0.003044	0.006529
8302	ZNF230	124.5429477	0.339484784	0.103088	3.293164	0.000991	0.002339
6604	UNC50	661.5597269	0.339367858	0.085223	3.982134	6.83E-05	0.000203
8256	ZNF12	950.8511702	0.339355708	0.10251	3.310478	0.000931	0.002211
8523	CTNNB1	6623.577149	0.339186577	0.106133	3.195859	0.001394	0.003206
15564	SLC34A3	24.62125545	0.33916383	0.401878	0.843947	0.398699	0.502114
10473	APH1B	544.2603067	0.339158144	0.136543	2.483885	0.012996	0.024323
12203	PIK3IP1	1303.95583	0.338868996	0.178791	1.895331	0.058049	0.09324
8676	HEATR5B	838.9545499	0.338740706	0.107869	3.140308	0.001688	0.003813
7663	ASNSD1	870.0437504	0.338621211	0.095292	3.553513	0.00038	0.000972
8029	C10orf18	1292.63817	0.338619714	0.099596	3.39992	0.000674	0.001646
11085	MRPL15	1111.636223	0.338567925	0.149752	2.260857	0.023768	0.042028
9091	PRPF40A	1939.750604	0.338525686	0.113532	2.981758	0.002866	0.006179
9879	TMCO3	1254.770934	0.338490048	0.125611	2.694759	0.007044	0.013976
9588	COPS2	2015.433027	0.338330537	0.121558	2.783289	0.005381	0.011001
8728	SMNDC1	638.2745682	0.338311863	0.108555	3.116488	0.00183	0.00411
9378	YAP1	2617.644326	0.338296993	0.117872	2.870034	0.004104	0.008578
9705	TNKS2	1508.84635	0.337781386	0.12303	2.745529	0.006041	0.012202
8565	SRR	177.128335	0.337754572	0.106045	3.184999	0.001448	0.003313
7729	FOXO4	374.9665743	0.337738596	0.095717	3.528522	0.000418	0.00106
9426	SMARCA2	2229.350045	0.337633936	0.118544	2.848166	0.004397	0.009144
6398	PTK2	2602.088999	0.337385185	0.082763	4.076512	4.57E-05	0.00014
8664	PAN3	900.8968991	0.337223155	0.107189	3.146049	0.001655	0.003744
11120	C8orf55	757.4143301	0.337176534	0.150167	2.24535	0.024746	0.043619
9573	KFZP586I14	324.8615793	0.33715872	0.120827	2.790426	0.005264	0.010778
10251	RNF123	602.1906434	0.337123293	0.13145	2.564659	0.010328	0.019748
7097	ZC3H7A	1196.328026	0.337091437	0.089076	3.784315	0.000154	0.000426
9742	FAM133B	105.2988555	0.337088873	0.123237	2.735283	0.006233	0.01254
9560	LOC148189	45.12183451	0.337066388	0.12053	2.796546	0.005165	0.01059
9527	ECHS1	3022.39658	0.337008463	0.120038	2.807515	0.004993	0.010272
11277	HERC6	415.1323107	0.336867951	0.153989	2.187608	0.028698	0.049881
5968	OXSR1	1154.889661	0.336653259	0.07861	4.282561	1.85E-05	6.07E-05
11494	MGAT4A	1847.999576	0.336618331	0.158597	2.122474	0.033798	0.057636
12180	NIPAL2	185.4881506	0.336592461	0.176908	1.902642	0.057087	0.091869
12168	LPP	569.3979838	0.3364712	0.176455	1.906837	0.056542	0.091081
15550	OR5K2	0.846663141	0.336337522	0.397391	0.846364	0.39735	0.500865
11326	DPY19L3	320.3581163	0.336226733	0.154848	2.171331	0.029906	0.051754
7313	ASH2L	902.5061649	0.336216701	0.090984	3.695346	0.00022	0.000589
6275	KATNA1	250.9429696	0.336126169	0.081127	4.143192	3.43E-05	0.000107
15579	DUSP26	7.592065162	0.335886949	0.399668	0.840414	0.400676	0.504118

13204	CLEC14A	1462.601504	0.335832064	0.212777	1.57833	0.11449	0.169957
10507	ATG4C	246.5096424	0.33574862	0.135961	2.469448	0.013532	0.025244
10206	DHDDS	844.7321674	0.335745415	0.130152	2.579634	0.009891	0.018994
10454	C14orf1	677.7999294	0.335565407	0.134724	2.490754	0.012747	0.023901
10259	C14orf28	103.9875809	0.335394472	0.130898	2.562257	0.010399	0.019869
10312	VTI1B	1367.98323	0.335376199	0.131922	2.542225	0.011015	0.020937
10134	C10orf58	2133.008709	0.335188976	0.128746	2.603489	0.009228	0.017849
9504	RANBP10	772.2703354	0.335100212	0.118862	2.819238	0.004814	0.009928
5649	SIN3A	1366.573201	0.335075727	0.075321	4.448642	8.64E-06	3.00E-05
11241	)C1002718	275.5603792	0.335019741	0.152072	2.203034	0.027592	0.048113
11577	SVIL	1602.602768	0.335012345	0.160327	2.089553	0.036658	0.06207
10902	C10orf4	403.7618134	0.33489045	0.144274	2.321213	0.020275	0.036454
16363	ANKRD34I	1.413242092	0.334807421	0.549149	0.609684	0.542071	0.649339
13287	GPR75	17.36271015	0.334696473	0.216207	1.548035	0.121614	0.179405
12691	DIAPH2	429.4420763	0.334568895	0.192659	1.736582	0.082461	0.127359
10148	TMEM167A	2811.221181	0.334245419	0.128594	2.59924	0.009343	0.018046
9593	BTD	470.4697566	0.33414105	0.120126	2.781598	0.005409	0.011052
9495	ATP6V1B2	3328.577098	0.334081247	0.118439	2.820699	0.004792	0.009891
11687	PRKCH	971.6934749	0.334039703	0.162629	2.053998	0.039976	0.067046
10680	MTHFS	682.1057227	0.334015412	0.139034	2.402394	0.016288	0.029894
9770	PPP2R3C	450.6406124	0.333756972	0.122467	2.725289	0.006425	0.012889
7324	TRAPPC10	1102.510496	0.333654251	0.090408	3.690542	0.000224	0.000599
8062	ZNF146	1547.284211	0.333585599	0.098466	3.387816	0.000705	0.001713
10035	ACBD3	1421.806961	0.333250593	0.126211	2.640418	0.00828	0.016174
12444	C2orf63	58.33209048	0.33322143	0.182842	1.822457	0.068386	0.107717
12047	DAB2	9365.855523	0.333205401	0.171282	1.945356	0.051732	0.08417
7889	NUP54	606.0291983	0.333204399	0.096475	3.453792	0.000553	0.001373
9224	NFS1	800.6140787	0.33310943	0.113729	2.928967	0.003401	0.007227
8345	CBWD2	493.4874523	0.333107315	0.101691	3.275697	0.001054	0.002476
16450	RNF113B	0.854098515	0.33302484	0.567708	0.586613	0.557464	0.664246
9026	RBM12B	419.7220428	0.332945582	0.110844	3.00374	0.002667	0.005791
17881	TMEM207	0.57211079	0.332813108	1.444025	0.230476	0.817722	0.89638
11409	PXMP4	330.5606459	0.332644379	0.154961	2.146635	0.031822	0.054672
7590	TBC1D5	1347.764674	0.332573361	0.092821	3.582953	0.00034	0.000877
8586	ZNF140	451.2433888	0.332470897	0.104608	3.178254	0.001482	0.003382
8780	PAIP2	2357.275711	0.332362526	0.107461	3.092867	0.001982	0.004425
11497	SORT1	2428.759342	0.332313821	0.156771	2.119744	0.034028	0.058013
9320	C14orf102	227.5352671	0.332284715	0.114891	2.89218	0.003826	0.008045
9585	C10orf28	354.2529977	0.332279053	0.119321	2.78474	0.005357	0.010955
10190	HDAC5	1194.81317	0.332126206	0.12844	2.58584	0.009714	0.018686
9371	C3orf38	388.9714627	0.332106548	0.115622	2.872352	0.004074	0.008522
11161	MORC4	541.7908166	0.332049556	0.148643	2.233871	0.025492	0.044768
10033	SEC14L1	4071.607599	0.33194775	0.125709	2.640595	0.008276	0.016169
7611	ZNF383	106.0247547	0.331930022	0.092808	3.576509	0.000348	0.000897
16432	CNTFR	8.929430446	0.331903698	0.558531	0.594244	0.552349	0.658873
16333	CCDC129	0.674014261	0.331874787	0.537666	0.617251	0.537069	0.644529
9402	ZNF445	443.0965307	0.331829899	0.116109	2.857908	0.004264	0.00889

10516	GIT2	2586.002453	0.331797428	0.134579	2.465442	0.013684	0.025507
8972	C19orf12	767.0184629	0.331739197	0.109731	3.02321	0.002501	0.005464
11991	EML2	570.8174772	0.331726382	0.169011	1.962752	0.049675	0.081196
10089	PIGP	373.8426598	0.331598877	0.12652	2.62092	0.008769	0.017037
14279	CHRM5	2.384401882	0.331587025	0.268534	1.234805	0.216903	0.297746
6521	PPP2CA	3183.444963	0.331391741	0.082479	4.017889	5.87E-05	0.000177
12004	DST	3332.83546	0.331173116	0.169021	1.959359	0.050071	0.081759
14832	C19orf69	6.816265932	0.331151076	0.313303	1.056968	0.290526	0.38394
12159	TGFBRAP1	298.3567041	0.331113298	0.173526	1.908144	0.056373	0.090876
11861	PEX11A	180.5785913	0.330921492	0.165238	2.002692	0.04521	0.074713
12378	DIXDC1	1226.834527	0.330710881	0.17949	1.842508	0.065401	0.103565
7597	EFTUD1	518.3825326	0.330360102	0.092254	3.580971	0.000342	0.000883
10836	LRRC16A	457.9211686	0.330148958	0.140669	2.346989	0.018926	0.034234
12086	DDX21	1509.520953	0.330088255	0.170836	1.93219	0.053336	0.0865
9872	MSH2	519.1987698	0.329942872	0.122366	2.696369	0.00701	0.013918
12206	CCDC30	112.9627271	0.329821607	0.174068	1.894788	0.058121	0.093333
7653	PAIP1	1116.173221	0.329766839	0.092675	3.558299	0.000373	0.000956
14491	F2RL3	429.2950024	0.329195696	0.281072	1.171213	0.241513	0.326656
9115	C15orf29	326.6840153	0.329187864	0.110722	2.973105	0.002948	0.006339
9626	IFNAR1	2595.898408	0.32913714	0.118838	2.769622	0.005612	0.011428
7758	SMAD2	1611.89113	0.328775165	0.093525	3.515372	0.000439	0.00111
8406	C1orf43	5074.426641	0.328674671	0.101223	3.247045	0.001166	0.002719
9052	ALMS1	587.8535159	0.328583001	0.109681	2.995798	0.002737	0.005927
7984	CHTF8	1784.551317	0.32850766	0.096121	3.417642	0.000632	0.001551
9609	RSBN1L	408.2175301	0.328388103	0.118313	2.775599	0.00551	0.01124
5570	YWHAE	11139.13039	0.328223895	0.073124	4.488564	7.17E-06	2.52E-05
10471	VAV2	1144.796822	0.328100622	0.132078	2.48414	0.012986	0.02431
11905	PTGES2	715.2866622	0.328045358	0.165212	1.985602	0.047078	0.077511
11114	ALDH2	4193.345019	0.327899346	0.145929	2.246986	0.024641	0.043455
14866	PRPH2	17.79577236	0.327691057	0.31284	1.047473	0.294882	0.388805
13866	PKP2	405.9478099	0.32756677	0.240423	1.362463	0.173052	0.244626
7881	SEC22C	477.2031201	0.327540037	0.094715	3.458152	0.000544	0.001353
8369	TXNRD3IT	187.4085602	0.327501975	0.100345	3.263752	0.001099	0.002575
12612	PDK1	1468.166736	0.327443252	0.185258	1.767498	0.077145	0.119895
9298	ATF6	1370.482026	0.327284886	0.112822	2.900909	0.003721	0.007844
14614	EDN1	1062.604715	0.327108478	0.290015	1.127902	0.259361	0.347868
7712	ZSCAN29	418.7940838	0.327072566	0.092522	3.535089	0.000408	0.001036
6842	EXOC2	707.3559661	0.327069948	0.084226	3.883259	0.000103	0.000295
8104	NIPA2	993.8968868	0.327026334	0.097066	3.369099	0.000754	0.001824
7063	RNASEL	425.1578753	0.327010709	0.086127	3.796827	0.000147	0.000407
13872	PC	731.4064644	0.326970014	0.24022	1.36113	0.173473	0.245115
7820	C1orf109	273.2834336	0.326903091	0.093511	3.495871	0.000473	0.001184
11873	SLC41A2	743.7343633	0.32686766	0.163513	1.999029	0.045605	0.075289
8131	CEPT1	768.2739352	0.326755715	0.097263	3.35949	0.000781	0.001882
9506	SCAND2	349.6848901	0.326536332	0.115881	2.817857	0.004835	0.009969
8852	RAB6A	3807.225458	0.326530471	0.106398	3.06896	0.002148	0.004756
5352	SMG7	2244.441508	0.326519655	0.0711	4.592387	4.38E-06	1.60E-05

9655	RNF219	417.0245781	0.326499708	0.118179	2.762757	0.005732	0.011636
11628	AGPHD1	27.03300049	0.326453885	0.157842	2.068226	0.038619	0.065099
6799	ASB8	878.8251968	0.326320328	0.083667	3.900221	9.61E-05	0.000277
8791	PPP1R12A	1426.501738	0.326203571	0.105623	3.088378	0.002013	0.004487
8042	MRPL45	878.2082657	0.326142454	0.096046	3.395682	0.000685	0.001669
12199	TYMS	691.5735047	0.325877014	0.171807	1.896757	0.05786	0.092968
11147	ZNF350	261.2901319	0.32570111	0.145553	2.237686	0.025242	0.044385
10345	UBE2B	1607.533067	0.325690846	0.128703	2.53056	0.011388	0.021577
11867	ZEB1	1156.524451	0.325577987	0.162668	2.001492	0.045339	0.074888
14959	TRIM55	380.6432241	0.325502496	0.319454	1.018934	0.308234	0.403884
14072	CCDC48	44.66205901	0.325031004	0.250778	1.296089	0.194945	0.27154
9631	ZC3H8	92.08522315	0.324962595	0.117359	2.768961	0.005624	0.011445
6821	DYNC1LI2	2943.64636	0.324924525	0.083513	3.890729	9.99E-05	0.000287
13314	CRNA0009	59.14043382	0.324917034	0.211143	1.53885	0.123841	0.18232
14757	CLDN7	680.0797715	0.324702033	0.300113	1.081932	0.279283	0.370932
8633	TCEANC	49.15877881	0.324485604	0.102682	3.160108	0.001577	0.003581
11871	C5orf15	3952.601507	0.324132186	0.162117	1.999378	0.045567	0.075239
7937	SNX5	2961.499387	0.324126314	0.094413	3.433067	0.000597	0.001474
13286	PPIF	1686.242694	0.324022165	0.209306	1.548081	0.121603	0.179402
9648	GTF2IP1	5973.933167	0.323997033	0.117241	2.763519	0.005718	0.011615
9908	TMEM55B	482.0806582	0.323988881	0.120635	2.685699	0.007238	0.014319
13178	ABCC5	953.6661248	0.323902111	0.204169	1.586439	0.11264	0.167537
9969	CEP192	409.5081117	0.323896165	0.12162	2.663186	0.00774	0.015219
9941	DIAPH1	5423.541435	0.323767061	0.12109	2.67377	0.0075	0.014789
7827	TEX10	389.4211534	0.323663459	0.0927	3.49151	0.00048	0.001203
9809	FEZ2	1035.528682	0.323260737	0.11911	2.71396	0.006648	0.013285
7161	C19orf2	1249.272319	0.323242872	0.085968	3.760026	0.00017	0.000465
10411	IFT81	314.731614	0.323232955	0.128961	2.506438	0.012195	0.022961
13308	CATSPER3	18.65416152	0.323089363	0.20979	1.540061	0.123545	0.181967
12195	C8orf83	1130.807875	0.323028465	0.170189	1.898058	0.057688	0.092722
12919	C11orf63	108.3645911	0.322998874	0.193682	1.66768	0.095379	0.144711
11741	HSPA13	1537.873825	0.322898749	0.158272	2.040157	0.041335	0.069006
7493	CLUAP1	483.1333216	0.322489083	0.0891	3.619392	0.000295	0.000772
14767	RGL4	21.66159239	0.322370561	0.298806	1.078863	0.280649	0.372519
6979	RNF121	647.4562863	0.322331845	0.084068	3.834194	0.000126	0.000354
9233	AZI2	804.8275707	0.32230407	0.110155	2.925927	0.003434	0.007291
12029	RASA2	119.2275693	0.322181488	0.164938	1.953351	0.050778	0.082742
14422	SERINC5	118.0358858	0.322152927	0.270357	1.191581	0.233425	0.317249
10691	ANKS6	512.8968091	0.32210177	0.134216	2.399869	0.016401	0.03007
12450	RFX3	58.07683948	0.322008054	0.176901	1.820276	0.068717	0.108187
7397	LSM14A	3090.479386	0.321735621	0.087949	3.658193	0.000254	0.000673
9483	NFKB1	1426.895042	0.321718508	0.113885	2.824938	0.004729	0.009775
7022	DAZAP2	7380.098977	0.321699335	0.084356	3.813597	0.000137	0.000382
7688	PUM2	2978.730067	0.32168902	0.09077	3.544013	0.000394	0.001005
10556	VANGL1	736.3828873	0.321471522	0.131197	2.450291	0.014274	0.026505
12215	WWTR1	1293.372462	0.321299294	0.169875	1.891382	0.058573	0.093991
8705	RWDD2A	138.3258209	0.321092129	0.10266	3.12771	0.001762	0.003967

9933	PRRC1	2049.63614	0.320427931	0.11971	2.676709	0.007435	0.014672
11247	DARS	4185.062847	0.320005813	0.145377	2.201214	0.027721	0.048311
9230	STX18	512.3822918	0.319955213	0.109271	2.92809	0.003411	0.007243
8480	SUMO1	2013.299432	0.319942419	0.099491	3.215784	0.001301	0.003007
9557	CAND1	2478.503618	0.319890441	0.114338	2.797752	0.005146	0.010554
13134	HOXB3	353.0458203	0.319831589	0.200133	1.598093	0.110022	0.164196
7096	SNIP1	258.9607191	0.319773687	0.084494	3.784559	0.000154	0.000425
6094	HP1BP3	3389.970088	0.319693734	0.075692	4.223602	2.40E-05	7.73E-05
14035	MALAT1	6824.786127	0.3196705	0.244456	1.307679	0.190982	0.266722
13261	FOXRED2	390.3483319	0.319634462	0.20481	1.560641	0.118609	0.175314
9045	IFT57	886.7206903	0.319420536	0.106559	2.997591	0.002721	0.005897
13885	SFRS13B	55.95005137	0.318988933	0.235751	1.353076	0.176031	0.248498
11194	B2M	86813.30164	0.318970656	0.143451	2.223549	0.026179	0.04584
6316	PUM1	2053.029783	0.31893682	0.077446	4.118199	3.82E-05	0.000119
12724	SLC38A1	7986.49118	0.31893063	0.184911	1.724775	0.084568	0.130275
13273	RAPGEF5	1393.327294	0.318890356	0.205241	1.553739	0.120247	0.177575
11214	ZNF514	259.2075291	0.318849308	0.143907	2.215666	0.026714	0.046694
11395	RTN4IP1	181.802796	0.318526773	0.148084	2.150993	0.031477	0.054144
10111	MRPS18B	1461.336726	0.31846664	0.121852	2.613545	0.008961	0.017371
6997	ZNF227	289.1817645	0.318427341	0.083244	3.825249	0.000131	0.000366
15293	IGF1	302.9255161	0.318131272	0.344766	0.922747	0.356139	0.456463
7596	DBR1	347.6723527	0.317960403	0.088784	3.581275	0.000342	0.000882
10036	ZBTB24	318.0569917	0.317914628	0.120412	2.64022	0.008285	0.016182
7771	CHD2	1950.631088	0.317693901	0.090529	3.509321	0.000449	0.001133
11482	ANKRD5	177.6753201	0.317682817	0.149388	2.126567	0.033456	0.057113
9453	ARMC2	44.88313796	0.317682601	0.112003	2.836378	0.004563	0.009461
11452	ASPRV1	79.27192575	0.317577265	0.148822	2.133943	0.032847	0.056221
8519	TIRAP	148.0825106	0.317133798	0.099162	3.198139	0.001383	0.003182
10313	ORC3L	537.6359766	0.317101225	0.124749	2.541921	0.011025	0.020953
8297	PIGV	391.0856499	0.316560269	0.096074	3.294958	0.000984	0.002325
10249	EPB41L1	2253.203454	0.31633046	0.123298	2.565586	0.0103	0.019699
13659	RAET1G	9.555532697	0.316327302	0.22027	1.43609	0.150977	0.21666
9410	POLR2A	4624.475039	0.316268553	0.110796	2.854515	0.00431	0.008978
11365	MDM4	804.8754718	0.316242394	0.146234	2.162582	0.030573	0.052725
7005	HCG18	356.0178027	0.316162145	0.082724	3.821875	0.000132	0.000371
13486	MAP1LC3A	352.169351	0.315522936	0.211567	1.491361	0.135867	0.197473
14993	CELF4	31.10373387	0.31550535	0.312861	1.008454	0.313237	0.409508
6702	HAUS2	462.0919613	0.315479407	0.080065	3.940278	8.14E-05	0.000238
11023	CYB5RL	75.6617073	0.315265057	0.138134	2.282311	0.022471	0.039958
8847	ANAPC10	126.5065117	0.315061579	0.102518	3.073225	0.002118	0.004692
13141	SNTA1	1105.495195	0.315060445	0.197312	1.596765	0.110318	0.16455
8250	PITPNB	1345.914292	0.314903187	0.095046	3.313164	0.000922	0.002192
9699	ZNF799	157.8621017	0.314883527	0.114632	2.746909	0.006016	0.01216
11294	C1GALT1	615.7965439	0.314872563	0.144456	2.17971	0.029279	0.050814
9706	AMN1	198.6543706	0.314777621	0.114658	2.745354	0.006045	0.012204
8672	UXS1	1122.882063	0.314718337	0.10014	3.142771	0.001674	0.003783
8570	C15orf40	215.7841819	0.314410394	0.098763	3.183482	0.001455	0.003328

9651	ZRANB2	1193.894845	0.31405988	0.113647	2.763466	0.005719	0.011615
11751	DDX58	877.3889344	0.313980829	0.154173	2.036548	0.041695	0.069549
9664	UBLCP1	624.0117564	0.313913897	0.113763	2.759357	0.005792	0.011747
16291	ART5	39.68507076	0.313890975	0.498905	0.62916	0.529244	0.636776
17203	IGFN1	52.7897616	0.313857331	0.811675	0.386678	0.698994	0.79643
8769	FAM118B	270.4972091	0.313764059	0.101342	3.096091	0.001961	0.004383
11040	FRG1B	319.5257058	0.313757337	0.137837	2.276289	0.022829	0.040531
6713	NOL11	836.8196048	0.31372042	0.079712	3.935696	8.30E-05	0.000242
12336	IMPAD1	3457.047487	0.313548533	0.169163	1.853532	0.063806	0.101383
7872	USP24	1482.286364	0.313503896	0.090517	3.463492	0.000533	0.001328
11816	ZNF879	78.16228677	0.313476594	0.155492	2.016035	0.043796	0.072652
8082	C22orf39	512.3699634	0.313363097	0.092755	3.378387	0.000729	0.001768
14392	OC1001288	36.02924377	0.313246465	0.261005	1.200155	0.230079	0.313354
8694	C6orf130	357.9469232	0.313172293	0.099958	3.133028	0.00173	0.003901
10840	ZNF77	58.48941012	0.313124847	0.13352	2.345154	0.019019	0.034391
11401	LMAN1	6672.351148	0.313037847	0.145662	2.149074	0.031629	0.054377
9208	ZNF687	676.2457993	0.313025778	0.106641	2.935325	0.003332	0.007093
9427	PTPDC1	191.411395	0.31270012	0.109805	2.847787	0.004402	0.009154
9121	HUWE1	5262.84877	0.312651331	0.105314	2.968744	0.00299	0.006426
12294	RPS6KA2	1200.314776	0.312640923	0.167385	1.867799	0.06179	0.098515
13512	GLIPR1L2	27.70963469	0.312551826	0.210538	1.484539	0.137666	0.199703
9526	MAK16	384.8269327	0.312345371	0.111242	2.807799	0.004988	0.010264
15577	FERMT1	83.27747943	0.312212612	0.371035	0.841464	0.400088	0.503443
16622	PPP1R1B	6.785247147	0.312143349	0.575255	0.542617	0.587393	0.692638
8693	YPEL5	2544.089334	0.312024656	0.099591	3.133069	0.00173	0.003901
6307	DYRK1A	1497.883486	0.311914758	0.075654	4.122897	3.74E-05	0.000116
13327	C4orf36	5.348982422	0.311732789	0.203147	1.534516	0.124903	0.183704
10153	ZNF226	327.0793212	0.311692458	0.119953	2.59845	0.009365	0.018079
9434	UBR5	1722.157226	0.311659176	0.109563	2.844558	0.004447	0.00924
8723	CNOT1	4075.807996	0.311617055	0.099881	3.119896	0.001809	0.004065
6190	CPSF7	1825.605521	0.311563529	0.074541	4.179739	2.92E-05	9.24E-05
9311	TMBIM4	1916.268609	0.311050489	0.107395	2.896313	0.003776	0.007949
9894	ZSCAN20	55.65483969	0.310927758	0.115594	2.689835	0.007149	0.014162
10096	ZNF407	280.345983	0.310776684	0.118727	2.61757	0.008856	0.017193
6755	CNOT4	595.6994	0.310522223	0.079269	3.917329	8.95E-05	0.00026
10977	APLP2	19645.35414	0.31043591	0.135298	2.294466	0.021764	0.038859
13019	BNIP3L	5919.768905	0.310367497	0.18946	1.638168	0.101387	0.152645
9551	TMBIM6	31065.63236	0.310218294	0.110739	2.801335	0.005089	0.010444
15580	PROX2	0.840845137	0.310174189	0.369093	0.840368	0.400702	0.504118
10228	METTL3	483.5836595	0.310152171	0.120572	2.572333	0.010102	0.019359
10451	LOC441089	92.80506843	0.310036174	0.124378	2.492687	0.012678	0.023778
10712	TAB2	1784.623855	0.309629587	0.129371	2.393355	0.016695	0.030549
8991	TRAPPC2	337.8665666	0.30943103	0.102621	3.015294	0.002567	0.005597
13521	BACH2	44.43953618	0.309387353	0.20888	1.48117	0.138561	0.200854
8880	CBFA2T2	619.010355	0.309265876	0.101058	3.060273	0.002211	0.004881
11378	SERHL	81.42220362	0.309104017	0.143149	2.159313	0.030826	0.053104
15446	BMP3	4.048646889	0.309003071	0.35319	0.874891	0.381633	0.484293

7941	CCAR1	1062.612961	0.308954905	0.090015	3.432242	0.000599	0.001478
8448	CAMSAP1	701.175607	0.308778362	0.095653	3.228114	0.001246	0.002891
8546	C5orf22	508.1781229	0.308751636	0.096749	3.19128	0.001416	0.003249
15418	C7orf57	6.300818249	0.308698723	0.349132	0.884189	0.376594	0.478767
11285	FARP1	3033.354896	0.308661789	0.141328	2.184003	0.028962	0.050304
13092	ZNF177	77.20337224	0.308506666	0.191161	1.613856	0.106559	0.159549
8862	PARP2	304.1863013	0.308404483	0.100594	3.065838	0.002171	0.004801
7818	RBBP6	1074.379634	0.308341119	0.088197	3.496052	0.000472	0.001184
11225	NDUFA10	2029.102859	0.308137618	0.139361	2.211072	0.027031	0.047201
16744	SCGB2A1	5.595406308	0.308071352	0.607335	0.507251	0.611978	0.716399
5893	ZNF592	1178.730755	0.308069566	0.071249	4.323824	1.53E-05	5.10E-05
9158	SYNCRIP	3277.826665	0.308002536	0.1042	2.955887	0.003118	0.006673
8221	NGLY1	499.1146891	0.307996278	0.092661	3.323892	0.000888	0.002117
8286	LOC92249	529.7252753	0.307884799	0.093336	3.298658	0.000971	0.002298
14415	HPN	2985.619925	0.30777277	0.257853	1.193598	0.232635	0.316329
13291	WFS1	913.0527841	0.307742436	0.199083	1.545796	0.122154	0.180147
8756	MRPS9	521.7112375	0.307676267	0.099127	3.103852	0.00191	0.004276
6872	SLC9A6	532.0964128	0.307629407	0.07939	3.874915	0.000107	0.000304
16122	C1orf227	0.584470966	0.307097746	0.45505	0.674865	0.499761	0.607606
10980	ZNF823	135.4797795	0.306934972	0.13384	2.293297	0.021831	0.038972
9935	SNN	779.5941089	0.306874486	0.114665	2.676265	0.007445	0.014688
11327	LSS	626.9305717	0.306847851	0.141319	2.171316	0.029907	0.051754
8226	PEX11B	800.7591929	0.306750811	0.092332	3.322251	0.000893	0.002128
15261	VSIG2	12.6993761	0.306488038	0.329359	0.93056	0.352081	0.452208
9724	MED7	276.627505	0.305971755	0.111654	2.740365	0.006137	0.012371
11472	ACTR3B	138.2722038	0.305948086	0.14374	2.128487	0.033297	0.056891
12888	MINA	513.1263	0.305778232	0.182352	1.67686	0.09357	0.142312
7865	SFRS13A	1277.384684	0.305764265	0.088228	3.465609	0.000529	0.001319
11280	ZNF491	44.9492467	0.305445787	0.139763	2.185456	0.028855	0.050141
9021	C10orf88	193.2996028	0.305410728	0.101654	3.004418	0.002661	0.005782
4519	MRFAP1	5723.236678	0.305331253	0.060762	5.025064	5.03E-07	2.18E-06
9525	DHX40	1713.395549	0.305097971	0.108636	2.80845	0.004978	0.010244
11320	C22orf32	724.0726641	0.305080243	0.14036	2.17356	0.029738	0.051493
16301	GPR111	5.063725703	0.305071196	0.486816	0.626666	0.530878	0.63835
10900	DHRS12	328.410336	0.304846697	0.131307	2.321642	0.020252	0.036419
14810	ENTPD2	265.4309205	0.30475798	0.287043	1.061714	0.288365	0.381651
11051	CDK17	865.429975	0.304705582	0.134037	2.273289	0.023009	0.040803
11205	GAK	1552.108611	0.304699888	0.137253	2.219979	0.02642	0.046215
17396	C1orf65	0.910342314	0.30463838	0.888784	0.342759	0.73178	0.824536
11869	RASAL2	991.1170635	0.304536536	0.152193	2.000984	0.045394	0.074967
15666	TM6SF2	87.92177636	0.3039438	0.371764	0.817571	0.413602	0.517491
10698	STOM	9197.20253	0.303843965	0.126712	2.397907	0.016489	0.030211
17497	VCX3B	0.449660825	0.303786899	0.963889	0.315168	0.752634	0.843142
12857	C6orf170	66.1155116	0.303731251	0.179753	1.689712	0.091083	0.13886
13133	RTP4	259.9220969	0.303565313	0.189949	1.598138	0.110012	0.164193
4345	CTCF	1285.82735	0.303440606	0.059099	5.134419	2.83E-07	1.28E-06
17196	C6orf146	0.614384932	0.303270365	0.781094	0.388263	0.697821	0.795417

10861	ING2	219.784115	0.30321225	0.129805	2.335909	0.019496	0.035184
12460	ADAM10	2356.266856	0.30313231	0.166947	1.815736	0.069411	0.109191
15306	QRFPR	319.0140161	0.3028842	0.329443	0.919383	0.357895	0.458324
12241	NFIC	801.7485466	0.302700044	0.160582	1.88502	0.059427	0.095158
15474	C17orf55	33.53857771	0.302586181	0.348645	0.867893	0.385453	0.488255
12558	GALNTL4	859.1702032	0.302509039	0.169307	1.786749	0.073978	0.115468
14041	FGL2	2140.62032	0.302413495	0.231655	1.305446	0.191741	0.267667
16479	HIST3H2BI	1.348894797	0.30234965	0.523387	0.577679	0.563481	0.670234
16808	TM4SF20	0.543786792	0.302321648	0.618558	0.488752	0.625017	0.728903
14010	FLJ37307	4.206985821	0.302181708	0.229713	1.315475	0.18835	0.263516
11252	KIAA0020	584.1194685	0.302039322	0.137329	2.199379	0.027851	0.048516
13570	OC1001282	59.31156676	0.301995358	0.206272	1.464063	0.143177	0.20681
9540	HKR1	723.2495013	0.301984257	0.107739	2.802922	0.005064	0.010405
15272	MYCBPAP	6.78960943	0.301706414	0.325249	0.927618	0.353606	0.453839
14342	MDH1B	38.81488392	0.301629519	0.247923	1.216627	0.223746	0.305791
10012	TMEM186	234.8709509	0.301593994	0.113837	2.649348	0.008065	0.01579
10858	MRI1	525.8586843	0.301501902	0.12899	2.33741	0.019418	0.035053
12608	SGSM2	908.4548623	0.301069775	0.170213	1.768778	0.076931	0.119601
10286	PINX1	93.0883414	0.301069496	0.11805	2.550357	0.010761	0.020507
15326	POM121L1I	2.040261552	0.300948565	0.329866	0.912336	0.361592	0.462453
10746	ZNF425	51.44904043	0.300917425	0.126296	2.382629	0.01719	0.031354
17137	PCSK2	1.517245391	0.300840993	0.748755	0.401788	0.68784	0.786739
13373	LOC253039	362.222855	0.300823912	0.19798	1.519466	0.128645	0.188557
10688	DISP1	432.0154232	0.300787785	0.125287	2.400787	0.01636	0.03
11775	SEL1L	4296.326462	0.300702978	0.148207	2.02894	0.042464	0.070688
14034	REL	69.8306136	0.300453743	0.229725	1.307885	0.190912	0.266644
11045	RASA1	965.5333623	0.300343867	0.132042	2.274602	0.02293	0.040692
9075	KCTD18	403.0691799	0.30029202	0.100515	2.987529	0.002812	0.006074
7010	C11orf57	874.669575	0.300237402	0.078613	3.819171	0.000134	0.000374
11427	LOC28444(	81.50673417	0.300178881	0.140259	2.14018	0.03234	0.055474
13112	FXYD6	1046.947335	0.30003831	0.186682	1.607217	0.108007	0.161458
9150	B3GALNT2	417.348	0.300007743	0.101405	2.958503	0.003091	0.006623
9269	TMEM14B	850.9267402	0.299819583	0.103035	2.909882	0.003616	0.007646
12820	IL11RA	139.6139869	0.299688117	0.176345	1.699444	0.089235	0.136436
7368	SFRS6	2962.540038	0.299638514	0.081632	3.670597	0.000242	0.000644
11362	CUEDC1	276.143422	0.299597966	0.138504	2.163104	0.030533	0.052674
10177	MFSD9	154.2412825	0.299548955	0.115636	2.59044	0.009585	0.018461
12818	ECHDC2	1071.589929	0.299486333	0.176181	1.699877	0.089154	0.136342
10975	ZNF689	138.7661946	0.299390744	0.130446	2.295126	0.021726	0.038802
8303	HNRNPH3	2416.760506	0.299199966	0.09086	3.292978	0.000991	0.00234
10152	PPID	681.0725654	0.298968282	0.115054	2.598506	0.009363	0.018078
10644	MAGEF1	762.1950669	0.298951079	0.123729	2.416178	0.015684	0.028883
11857	RMND1	606.3502773	0.298522501	0.149	2.003502	0.045123	0.074594
6018	C15orf44	806.0068928	0.298447215	0.07012	4.256209	2.08E-05	6.77E-05
11039	SLC26A2	698.179337	0.298398312	0.131043	2.277102	0.02278	0.040449
7980	GLE1	807.6438792	0.298310901	0.087253	3.418936	0.000629	0.001544
15539	C22orf34	6.505296443	0.298255178	0.351272	0.849071	0.395842	0.499303

8187	C2orf56	262.1115523	0.298187453	0.08931	3.338792	0.000841	0.002014
15180	CRNA0016	1.69695805	0.298099576	0.312675	0.953385	0.340395	0.439553
9485	ADO	616.4023093	0.298086428	0.105589	2.823078	0.004757	0.009829
10309	TULP4	592.4846309	0.298044481	0.117228	2.542427	0.011009	0.020931
13185	CARD6	379.5549185	0.298043016	0.1881	1.58449	0.113082	0.16811
16448	OR10AD1	0.59643192	0.298041099	0.507309	0.587494	0.556872	0.663622
13528	OC1001909	186.7607807	0.297993521	0.201633	1.477903	0.139434	0.202029
10225	SHQ1	332.4864679	0.297862204	0.115761	2.573071	0.01008	0.019323
13564	INF2	2706.769019	0.297835369	0.203208	1.465668	0.142739	0.206268
14231	RFX2	243.4924577	0.297806784	0.238405	1.249165	0.211605	0.291465
11098	MACF1	5074.179613	0.297713582	0.131968	2.255952	0.024074	0.042518
9920	ZC3H6	380.3031095	0.297595985	0.111042	2.680028	0.007362	0.014546
10577	TOP1	2439.866669	0.297448557	0.121706	2.443985	0.014526	0.026919
15162	PTPRR	33.0234758	0.297405385	0.310554	0.957662	0.338233	0.437258
6986	PSPC1	552.3978859	0.297169146	0.07757	3.830961	0.000128	0.000358
9368	NOL9	159.9544154	0.296995849	0.10335	2.873681	0.004057	0.00849
11235	SLMO2	1487.762938	0.296927119	0.134615	2.205756	0.027401	0.047805
12656	KIAA1715	1069.661521	0.296924678	0.169181	1.755076	0.079246	0.122733
15884	FOXP2	94.02200214	0.296869624	0.397133	0.747531	0.454743	0.561121
13128	GIMAP6	1273.343013	0.296861125	0.185409	1.601113	0.109352	0.16327
12601	PLS3	3844.026793	0.296805357	0.167397	1.773065	0.076218	0.118558
10301	CCDC117	718.9524033	0.296736027	0.116583	2.545272	0.010919	0.020777
7855	USP42	346.5227429	0.296668394	0.085506	3.469564	0.000521	0.001301
11042	KIF1C	2418.812995	0.29648475	0.130269	2.275942	0.022849	0.040561
12520	FAM168A	228.5829379	0.296287906	0.16479	1.797975	0.072181	0.113005
13098	NHEDC1	4.233630673	0.296218001	0.183734	1.61221	0.106916	0.159999
13130	ITGA1	3314.096432	0.296189519	0.18511	1.600075	0.109582	0.163588
11566	ELF1	1490.459061	0.296026967	0.14143	2.093106	0.03634	0.061588
17447	PRDM7	0.425360751	0.295841593	0.903902	0.327294	0.743446	0.835231
14318	LOC283314	475.7573989	0.295797622	0.241538	1.224644	0.22071	0.302146
6861	TMEM57	829.7140043	0.295755865	0.076282	3.877157	0.000106	0.000302
10325	JRKL	466.2390037	0.295625653	0.11646	2.538433	0.011135	0.021139
16635	CAGE1	0.376857027	0.295586597	0.548284	0.539112	0.589809	0.694972
16844	TPO	0.350021088	0.295586503	0.616009	0.479841	0.63134	0.734801
17056	GPR52	0.29538182	0.295586351	0.694126	0.42584	0.670225	0.770232
17069	BTNL2	0.255721282	0.295586311	0.701323	0.421469	0.673412	0.773306
17185	C2orf78	0.379465498	0.295586311	0.757501	0.390212	0.69638	0.794236
17325	VCX	0.321829885	0.295586153	0.818225	0.361253	0.71791	0.812223
17374	CCDC38	0.233077063	0.295586006	0.851276	0.347227	0.728421	0.821765
17536	CRNA0016	0.359278868	0.295585912	0.963612	0.306748	0.759035	0.848418
17500	TAAR5	0.192607221	0.29558576	0.938556	0.314937	0.75281	0.84319
17631	ZNF716	0.291853132	0.295585653	1.040592	0.284055	0.776368	0.863161
17570	IL5	0.1797456	0.295585605	0.989198	0.298813	0.765082	0.853522
17571	APOBEC4	0.178809754	0.295585604	0.989597	0.298693	0.765174	0.853527
17669	FAM46D	0.248084736	0.295585475	1.072378	0.275636	0.782828	0.868426
17650	KBTBD13	0.154782171	0.295585386	1.057457	0.279525	0.779842	0.866005
17725	KRTAP5-6	0.185435724	0.295585209	1.12897	0.261818	0.793461	0.877441

17818	PTF1A	0.138359858	0.295584841	1.218914	0.242499	0.808394	0.889288
18087	MT1IP	0.1179037	0.295583396	1.572106	0.188017	0.850863	0.922098
18169	GHRHR	0.118369179	0.295582662	1.728619	0.170994	0.864229	0.932344
18175	CLRN1	0.099637775	0.295582466	1.750232	0.168882	0.86589	0.933827
18242	FLJ40504	0.139193587	0.295581882	1.899875	0.15558	0.876364	0.941652
18246	DEFB125	0.119079415	0.295581699	1.914454	0.154395	0.877298	0.942449
18269	IFNA1	0.080356247	0.295581087	1.983831	0.148995	0.881558	0.945832
18380	OR2D2	0.079034911	0.295579536	2.23242	0.132403	0.894665	0.954099
18467	OPTC	0.08150135	0.295577453	2.528087	0.116917	0.906925	0.962737
18496	PRG3	0.06138215	0.29557648	2.629432	0.112411	0.910498	0.964893
18497	DPRX	0.060350915	0.295576424	2.636428	0.112112	0.910734	0.96504
18529	NBPF22P	0.099196614	0.295575203	2.840496	0.104058	0.917124	0.970184
18548	GABRA6	0.144292475	0.295575079	2.91407	0.10143	0.919209	0.970766
18544	CCT8L2	0.058837416	0.295574099	2.91407	0.10143	0.919209	0.970766
18546	DEFB118	0.061312547	0.295574099	2.91407	0.10143	0.919209	0.970766
18552	LOC73227	0.05910443	0.295574099	2.91407	0.10143	0.919209	0.970766
18559	SNORA70	0.059769636	0.295574099	2.91407	0.10143	0.919209	0.970766
18545	CFC1B	0.042972694	0.295573854	2.91407	0.10143	0.919209	0.970766
18551	KRTAP13-	0.04257075	0.295573854	2.91407	0.10143	0.919209	0.970766
18555	OR10P1	0.042003151	0.295573854	2.91407	0.10143	0.919209	0.970766
18557	PRSS41	0.039662643	0.295573854	2.91407	0.10143	0.919209	0.970766
18560	SULT1C3	0.043781459	0.295573854	2.91407	0.10143	0.919209	0.970766
14547	IL18R1	237.9147131	0.295481284	0.256516	1.151901	0.249362	0.335996
12008	STARD8	755.0829196	0.295463644	0.150831	1.958907	0.050124	0.081818
8864	C9orf85	117.7368124	0.295442068	0.096397	3.06485	0.002178	0.004816
8955	AP3M1	1064.545788	0.295426103	0.097487	3.030418	0.002442	0.005345
8522	TMEM59	6774.354648	0.295058243	0.092301	3.196708	0.00139	0.003197
16983	C17orf99	0.411576554	0.295017648	0.665909	0.44303	0.657744	0.759138
10389	GNRHR2	376.3432456	0.294981998	0.117506	2.510347	0.012061	0.022756
10903	TM2D1	420.3468158	0.29476133	0.126995	2.321043	0.020285	0.036467
7730	EXOSC3	229.1034149	0.294760691	0.083543	3.528236	0.000418	0.001061
13900	LOC72881	27.35957706	0.294481959	0.218372	1.348531	0.177488	0.250283
14609	C20orf165	11.4116639	0.294462156	0.260483	1.130445	0.258289	0.346548
15400	C12orf27	77.83601169	0.294459045	0.330688	0.890443	0.373228	0.475041
10335	PRCP	3979.73174	0.293507025	0.115757	2.535551	0.011227	0.021293
11656	C11orf1	483.2217551	0.293445133	0.142483	2.059512	0.039445	0.066332
12049	OC1001300	87.04916819	0.29340529	0.150922	1.94408	0.051886	0.084406
11717	C4orf34	556.5021218	0.293338569	0.143282	2.047274	0.040631	0.067971
15915	WSCD2	11.08306194	0.293219691	0.397729	0.737235	0.46098	0.567745
15513	DLX6AS	2.406181962	0.293218514	0.342731	0.855535	0.392255	0.495622
9577	NDUFB3	743.9867487	0.293028741	0.105056	2.789254	0.005283	0.010812
9141	ALG5	694.6929723	0.292938246	0.098917	2.961445	0.003062	0.006566
13307	TPRN	321.3780528	0.292801903	0.190052	1.540641	0.123404	0.181773
12064	VPS37A	1039.506225	0.292697732	0.150877	1.939972	0.052383	0.085115
12970	FAS	771.6475787	0.292516098	0.17702	1.652444	0.098444	0.14877
10238	VRK3	771.8890389	0.292454345	0.113778	2.57039	0.010158	0.019449
4378	HNRNPK	11406.67881	0.292371352	0.057157	5.115194	3.13E-07	1.40E-06

9906	TCF7L2	583.3316556	0.292231561	0.10878	2.686441	0.007222	0.014291
9785	DDX18	1792.655146	0.292157147	0.107385	2.720653	0.006515	0.01305
8267	DNAJC21	1099.729736	0.292100034	0.088368	3.305501	0.000948	0.002248
11529	ALG11	19.79837638	0.29206219	0.13854	2.108146	0.035018	0.059536
11999	KDM1B	495.5141697	0.292060754	0.148951	1.960778	0.049905	0.081522
14320	KIF5C	137.1049608	0.292019686	0.238587	1.223956	0.220969	0.302459
13942	TSPYL5	448.2060576	0.291975473	0.218426	1.336724	0.181313	0.254907
15635	GOLGA6B	2.869193163	0.291911158	0.353403	0.826	0.408804	0.512502
14178	HOXA9	394.2104792	0.291841294	0.231008	1.26334	0.206467	0.285439
7831	DYNC1LI1	631.4679695	0.291814032	0.083725	3.485383	0.000491	0.00123
14068	KLF4	659.8844986	0.291612113	0.224853	1.296902	0.194665	0.27121
11512	MBNL2	2374.849738	0.291562409	0.138051	2.111985	0.034688	0.059061
11202	SNAPC1	355.3335329	0.291521644	0.131258	2.220983	0.026352	0.04611
15668	EFHB	16.47711603	0.291472209	0.356788	0.816933	0.413967	0.517881
16606	ADAM29	0.77646414	0.29145315	0.532251	0.547586	0.583976	0.6893
13741	DMD	552.2553772	0.291297262	0.207457	1.404137	0.160278	0.228629
14049	LOC90834	21.21899125	0.291243051	0.223738	1.301713	0.193014	0.269291
7665	ZBTB9	215.3573284	0.291064955	0.081923	3.552906	0.000381	0.000974
7540	THOC2	1088.067706	0.290925665	0.080828	3.599329	0.000319	0.000829
14838	MTMR9L	134.8345351	0.290787907	0.275583	1.055174	0.291346	0.384868
14490	NHLRC4	27.06158321	0.290732033	0.248209	1.171318	0.241471	0.326644
8527	CCDC55	713.7034367	0.290659348	0.090962	3.195403	0.001396	0.003209
13115	RAB27A	603.1153492	0.290549397	0.180836	1.606699	0.10812	0.161591
8017	AP2B1	5653.822995	0.290526683	0.085325	3.404961	0.000662	0.001617
10268	HIPK1	1911.518991	0.290297689	0.113478	2.55819	0.010522	0.020084
11979	GTF2H3	189.9649925	0.290156395	0.147609	1.965706	0.049333	0.08072
7527	TIAL1	1237.895691	0.290156127	0.080488	3.604942	0.000312	0.000813
9496	RHOT1	862.1028384	0.290087952	0.102842	2.820726	0.004792	0.009891
9965	DOLK	394.1593441	0.290077031	0.10886	2.664678	0.007706	0.015158
13191	SHROOM4	789.5733608	0.290047736	0.18332	1.582197	0.113605	0.168809
9128	HUS1	328.7055464	0.290027864	0.097771	2.96641	0.003013	0.00647
14548	FAM163A	24.30661723	0.289742825	0.251685	1.151211	0.249645	0.336355
14586	ARHGAP2(	39.5022012	0.289603317	0.253769	1.141207	0.253784	0.341041
8425	C18orf8	484.7756225	0.289591432	0.089347	3.241186	0.00119	0.002769
13012	ETS2	3908.968859	0.289230434	0.176377	1.639842	0.101038	0.15219
16411	OPCML	224.0924874	0.289132474	0.482568	0.599154	0.54907	0.655799
9411	MTF1	428.7823428	0.288895914	0.101247	2.85337	0.004326	0.00901
11107	PGM2	643.5705355	0.288806138	0.128311	2.250824	0.024397	0.043054
7598	NSMCE4A	339.6778021	0.288769258	0.080654	3.580365	0.000343	0.000885
12376	TMCO4	662.2867053	0.288705492	0.156662	1.842852	0.065351	0.103502
10546	RMND5B	639.1486567	0.288615792	0.117618	2.45384	0.014134	0.02627
14408	TLE2	321.6974536	0.288560928	0.241461	1.19506	0.232063	0.315705
10816	CRIPT	401.254382	0.28853447	0.122491	2.355559	0.018495	0.033517
10517	SLC24A1	343.2594899	0.288354041	0.116969	2.465216	0.013693	0.02552
11971	ARHGEF1(	1032.872347	0.28835148	0.146509	1.968151	0.049051	0.080314
10984	FAM76B	320.1908621	0.288238494	0.125753	2.292092	0.0219	0.039081
8719	USP34	2351.702343	0.287582843	0.092087	3.122943	0.001791	0.004025

10392	ATR	646.9682937	0.287385516	0.114499	2.509939	0.012075	0.022776
13347	EPB41L3	1422.31701	0.287271594	0.187871	1.529091	0.126242	0.185395
12226	ZNF85	114.4823514	0.28724104	0.151991	1.889851	0.058778	0.094234
8182	FIG4	489.7676128	0.287178154	0.085935	3.341805	0.000832	0.001994
11503	SMAD1	584.8510427	0.287103176	0.135696	2.115781	0.034363	0.058553
11008	FAM160B1	876.7698611	0.287010871	0.125476	2.287368	0.022174	0.039484
9500	WDR5B	321.5658979	0.286993656	0.10177	2.820013	0.004802	0.009908
9373	CAPRIN1	5003.253423	0.286972731	0.099935	2.871586	0.004084	0.008541
14950	FHL5	235.3204686	0.286836403	0.280534	1.022464	0.306561	0.401934
8342	C2orf64	602.8210123	0.286736829	0.087514	3.27647	0.001051	0.00247
12920	EEA1	1159.621122	0.28672236	0.17198	1.667181	0.095478	0.144851
13394	ZNF578	37.93349709	0.286700478	0.189528	1.51271	0.130353	0.190761
12554	LOC44035[illegible]	115.1614934	0.286586446	0.16021	1.788822	0.073643	0.114982
9702	EXOG	140.7640933	0.286441623	0.104316	2.745914	0.006034	0.01219
9109	BRD2	4120.030727	0.286346057	0.096256	2.974849	0.002931	0.006307
12803	RUFY3	1342.631431	0.286320653	0.168167	1.702594	0.088644	0.135711
12279	IGF2R	4651.093506	0.286301526	0.152912	1.872331	0.061161	0.097631
10619	TBCC	357.4487217	0.286284388	0.117935	2.427481	0.015204	0.028064
9815	GOLPH3	2575.71963	0.286270111	0.105538	2.712493	0.006678	0.013336
10565	TUBD1	157.9471501	0.286034708	0.116857	2.447723	0.014376	0.026672
14017	HIST1H2BI	621.7410278	0.285971782	0.217634	1.314006	0.188844	0.264075
11004	CEP57	783.2218281	0.285970985	0.124974	2.288241	0.022124	0.039408
12005	TPCN2	630.1219715	0.285933784	0.145945	1.959191	0.05009	0.081778
13007	ACLY	8948.451838	0.285618914	0.173947	1.641984	0.100593	0.15159
13680	C3orf70	210.4352824	0.28552414	0.199998	1.427634	0.153397	0.219791
15114	C9orf131	2.454107235	0.285450826	0.293735	0.971796	0.331152	0.429463
8581	EIF3J	1251.284969	0.285406946	0.089753	3.179927	0.001473	0.003365
7474	MORF4L1	4046.522485	0.285151881	0.078613	3.627265	0.000286	0.000751
12508	INSIG2	1663.774955	0.285066136	0.158304	1.800752	0.071742	0.112417
8630	WHAMM	357.9045577	0.284942358	0.09013	3.161444	0.00157	0.003566
9708	SENP2	691.2268494	0.284788256	0.103732	2.745411	0.006044	0.012204
8504	GPATCH8	1149.604285	0.284746746	0.088866	3.204224	0.001354	0.003121
10570	INTS9	351.0379497	0.284638931	0.116323	2.446968	0.014406	0.026713
13201	HIP1R	1539.282172	0.284366677	0.180149	1.578506	0.114449	0.169936
10562	AGTPBP1	411.7156822	0.284226878	0.116092	2.448299	0.014353	0.026637
11835	DHX33	265.2672076	0.284148654	0.141407	2.009437	0.044491	0.073679
10718	SLC10A7	214.0659144	0.284019689	0.118825	2.39024	0.016837	0.030792
15777	LOC34884[illegible]	1.548906812	0.283917269	0.362828	0.782513	0.433913	0.539084
7199	WDR92	160.6519087	0.283896802	0.075778	3.746437	0.000179	0.000488
8776	FAM114A[illegible]	593.1067921	0.283762511	0.091728	3.093511	0.001978	0.004418
12547	[illegible]LM2-AKA	2862.052875	0.283614843	0.158264	1.792036	0.073127	0.114242
11917	PDDC1	1164.599299	0.283484342	0.142946	1.983156	0.04735	0.077881
11603	PBXIP1	3735.195187	0.283438125	0.136283	2.079771	0.037547	0.063428
14501	ITGA9	238.6305509	0.283373897	0.242705	1.167567	0.242982	0.328438
12675	NHSL1	423.8763534	0.283320029	0.162277	1.745899	0.080828	0.124995
10663	AAK1	1257.247573	0.283307896	0.117709	2.406847	0.016091	0.029579
9619	ZNF79	103.5039808	0.282843125	0.10202	2.772426	0.005564	0.011338

8698	CSNK1G1	586.8378628	0.282781371	0.090315	3.131057	0.001742	0.003925
8822	SRP72	2244.927166	0.28267364	0.091722	3.081837	0.002057	0.004571
12063	C10orf12	81.6440731	0.282536122	0.145644	1.939915	0.05239	0.085115
17094	LYPD2	0.790745266	0.282499109	0.679828	0.415545	0.677743	0.777231
11238	CDC26	98.41154942	0.282319367	0.128063	2.204543	0.027486	0.047941
12632	DOCK4	1004.877049	0.282183993	0.159977	1.763906	0.077748	0.120622
8938	UBIAD1	457.1325174	0.282107077	0.092862	3.037909	0.002382	0.005224
9590	FAM65A	2268.61816	0.282098147	0.101359	2.783156	0.005383	0.011003
16501	EEF1A2	130.4393791	0.282060923	0.492556	0.572647	0.566884	0.673383
9449	FCF1	631.1925982	0.281900899	0.099324	2.838188	0.004537	0.009412
13431	TMC7	59.56577356	0.281728419	0.187219	1.504806	0.132374	0.193185
12281	TRIM52	289.402635	0.281518179	0.150367	1.872213	0.061177	0.097641
10084	HAT1	807.6067183	0.281410675	0.107264	2.623529	0.008702	0.016914
15394	JPH1	46.37625184	0.281238294	0.315397	0.891697	0.372555	0.47437
9256	AHCYL1	3847.294851	0.28122567	0.09648	2.914875	0.003558	0.007535
9817	ZBTB40	671.3689539	0.281209736	0.103708	2.711549	0.006697	0.013371
10500	CEP350	1205.275165	0.281141051	0.113751	2.47155	0.013453	0.025113
11827	UBA1	6964.958328	0.281050264	0.139666	2.012309	0.044187	0.073232
10075	MED1	1131.583971	0.280491661	0.106779	2.626832	0.008618	0.016767
8851	C4orf27	342.6256596	0.280441487	0.091351	3.069936	0.002141	0.004741
13810	CXorf36	1304.419798	0.280425626	0.202738	1.383195	0.166605	0.236468
14503	CDK18	5540.204465	0.280398063	0.240471	1.166037	0.2436	0.329228
8614	SAP18	3109.083718	0.280313456	0.08845	3.169186	0.001529	0.003478
10813	WDR36	938.0017221	0.280283151	0.118886	2.35758	0.018394	0.033344
16643	CELF5	34.55622138	0.280240538	0.522001	0.536859	0.591365	0.696428
10627	TMEM194E	190.3909936	0.280200907	0.115628	2.423295	0.01538	0.028368
10314	LOC550112	301.4765407	0.280051852	0.110176	2.54187	0.011026	0.020954
11564	CDKN1B	1466.07704	0.279935759	0.133742	2.093096	0.036341	0.061588
11562	RSL24D1	2243.437244	0.279742796	0.133635	2.093338	0.036319	0.061571
8068	BRD1	633.6816745	0.279729271	0.082644	3.384761	0.000712	0.001731
8928	WDR37	455.2435546	0.279680525	0.091944	3.041864	0.002351	0.005162
11339	KLHL5	920.4834159	0.279219767	0.12871	2.16938	0.030054	0.051952
12427	ZNF267	515.9994929	0.279125378	0.152734	1.827521	0.067621	0.106659
16021	ACTC1	84.91355512	0.278943778	0.395599	0.705118	0.480737	0.58816
14451	TSPAN33	2007.780748	0.27876505	0.23583	1.182058	0.237183	0.321709
13109	HERC5	274.7251091	0.278747086	0.173351	1.607989	0.107838	0.161242
12642	C13orf37	462.9486018	0.278662059	0.158344	1.759857	0.078432	0.121606
14101	GSTZ1	338.4680401	0.278468599	0.216121	1.288487	0.197576	0.27464
9243	FAM185A	95.90547364	0.278384264	0.095314	2.920721	0.003492	0.007406
15895	C15orf27	68.65197133	0.27835818	0.37405	0.744173	0.456772	0.56327
13035	FAM50B	279.4541626	0.278021752	0.170208	1.633427	0.102379	0.15395
8817	OCRL	938.845229	0.27776824	0.090116	3.08233	0.002054	0.004566
12022	MTHFR	886.4828402	0.277763949	0.142077	1.955021	0.050581	0.082461
12170	CIRBP	4981.42919	0.277762918	0.145699	1.906412	0.056597	0.091155
9353	MRPL32	968.5695435	0.277660088	0.096452	2.878737	0.003993	0.008367
9372	MAP2K5	309.3472539	0.277647875	0.096668	2.872179	0.004077	0.008526
12927	SH3GLB2	812.6905901	0.277571806	0.166688	1.665223	0.095868	0.145364

11523	NMT2	483.9971361	0.2773135	0.131418	2.110166	0.034844	0.059275
10044	AGA	546.599197	0.277214712	0.105162	2.636064	0.008387	0.016368
12779	ZFYVE28	258.8104584	0.277176995	0.161935	1.711652	0.086961	0.133395
11015	KIAA1191	4941.046145	0.277112272	0.121238	2.285693	0.022272	0.039633
14009	SEMA4G	391.4280416	0.276878457	0.210398	1.315974	0.188183	0.2633
15071	TNNI3K	22.8339331	0.27678103	0.280878	0.985415	0.32442	0.421933
12525	MAP3K5	830.7806991	0.27673842	0.154018	1.79679	0.072369	0.113244
8617	RPL7L1	749.6224013	0.276392836	0.087225	3.168732	0.001531	0.003483
12366	WASF1	355.6160004	0.27618089	0.149607	1.846039	0.064887	0.10285
10275	WDR59	707.7594417	0.276082131	0.108055	2.555027	0.010618	0.020255
9916	PHF2	870.7488201	0.275944796	0.102897	2.681763	0.007324	0.014476
12848	FRMD4B	874.7399542	0.275900278	0.163165	1.690923	0.090851	0.138604
8981	VPS11	1551.330549	0.275898485	0.09135	3.020224	0.002526	0.005513
9932	KPNA4	1811.771727	0.275527188	0.102923	2.677012	0.007428	0.01466
11724	HMGCL	1273.610791	0.275219311	0.134559	2.045349	0.04082	0.068247
11382	ZNF169	68.00160142	0.274958671	0.127397	2.158286	0.030906	0.053223
10805	DPM1	525.7333682	0.274791397	0.11644	2.359949	0.018277	0.033157
11602	BRF1	517.8653004	0.274689208	0.132059	2.080055	0.03752	0.063389
13039	PTPRM	3103.079894	0.274518667	0.168156	1.632525	0.102569	0.154188
10651	RNGTT	398.5385341	0.274418039	0.113698	2.413574	0.015797	0.029071
15095	FAM19A1	2.409296555	0.27430049	0.280571	0.977651	0.328247	0.42626
11407	AKT2	2158.274704	0.274191029	0.127724	2.146745	0.031814	0.054661
10872	UBN2	720.2196995	0.273978282	0.11748	2.332124	0.019694	0.035506
10656	GOPC	880.6736391	0.273963154	0.113609	2.411447	0.015889	0.029227
11686	WDR24	337.6804216	0.273041147	0.132864	2.055049	0.039874	0.066881
9542	REPS1	499.6907899	0.273009005	0.097412	2.802633	0.005069	0.010412
15183	RGS16	545.1783594	0.272969863	0.286477	0.952851	0.340666	0.439794
14299	TRIL	378.1527304	0.27294673	0.221978	1.229613	0.218842	0.299988
12309	SNX6	1671.993636	0.272535677	0.146378	1.861866	0.062622	0.09972
13835	C7orf63	127.7913327	0.272477359	0.198458	1.37297	0.169762	0.240513
11372	TAF2	671.1475921	0.272222754	0.126019	2.16017	0.03076	0.053018
10523	RAB10	3862.882176	0.272200426	0.110516	2.462993	0.013778	0.025665
10560	C16orf46	43.50478175	0.272158564	0.111142	2.448744	0.014336	0.026609
12019	FAM123B	178.3804061	0.272146163	0.139151	1.95576	0.050493	0.082346
10564	C14orf169	196.9967706	0.272054825	0.111144	2.447767	0.014374	0.026671
10232	C17orf85	588.7645361	0.271942986	0.105764	2.571233	0.010134	0.019413
10620	UTP23	403.6258678	0.271850669	0.112005	2.427129	0.015219	0.028089
9861	PRKRIR	981.0332976	0.271788097	0.100685	2.699402	0.006946	0.013808
12842	SHB	758.3137258	0.271773357	0.160552	1.692739	0.090505	0.13814
15690	C5orf40	1.173757958	0.27173912	0.335324	0.810377	0.417723	0.521848
13926	SPINT1	2079.605582	0.271674684	0.202369	1.342471	0.179443	0.252569
16493	PRSS12	27.05804699	0.27139224	0.47235	0.574557	0.565591	0.672173
13121	NDUFB1	818.7259488	0.271303084	0.169119	1.604218	0.108666	0.162332
10539	DNAJC11	1130.736556	0.271254553	0.110423	2.456508	0.014029	0.026093
11316	SP3	1951.753122	0.271166767	0.124714	2.174307	0.029682	0.051414
12559	DENND1C	499.4550704	0.271037453	0.151797	1.785528	0.074176	0.115767
7810	ZNF434	293.8373692	0.271023511	0.077484	3.497782	0.000469	0.001177

12288	CLSTN1	5818.519371	0.270973711	0.144988	1.868944	0.061631	0.098309
12540	USP11	1815.679043	0.270966443	0.151056	1.793809	0.072844	0.11386
12986	FAM125B	310.680424	0.270895979	0.1643	1.648785	0.099192	0.149699
11259	XRN2	2079.448035	0.270563175	0.123143	2.197154	0.028009	0.048762
10898	C5orf43	1609.709238	0.270380098	0.116448	2.321898	0.020238	0.036401
10792	TPRG1L	1477.341785	0.270282193	0.114295	2.364786	0.018041	0.032766
9897	RAB3GAP1	1576.80006	0.270206774	0.100497	2.688699	0.007173	0.014206
9727	PATZ1	827.7954835	0.270161549	0.09863	2.739135	0.00616	0.012413
10003	CDK12	1104.587167	0.270105058	0.101858	2.651776	0.008007	0.01569
11652	ENTPD4	331.1873805	0.270056678	0.131047	2.060756	0.039326	0.066149
10445	PIGF	273.1688262	0.269990095	0.108163	2.49613	0.012556	0.023562
12321	RNF19B	769.0204313	0.269811178	0.14514	1.858978	0.06303	0.100272
8993	SPIN2B	85.61209085	0.269636989	0.089437	3.014817	0.002571	0.005604
7785	FIP1L1	632.7295981	0.269554605	0.076887	3.505843	0.000455	0.001146
9195	ACP1	1843.675836	0.269398584	0.091581	2.941644	0.003265	0.006958
17230	OLIG2	7.835068414	0.269319956	0.705862	0.381548	0.702797	0.799508
12633	MICAL3	2301.164751	0.269248435	0.152644	1.763903	0.077748	0.120622
9566	TMCO1	2452.711751	0.26911042	0.096302	2.794438	0.005199	0.010653
10764	TEC	73.43197795	0.268945409	0.113136	2.377194	0.017445	0.031767
14149	FLJ22536	32.11768263	0.268827044	0.211393	1.271694	0.203482	0.281889
13232	CLK1	1939.507657	0.268791348	0.171263	1.569465	0.11654	0.172634
12721	C14orf2	921.5241233	0.26861969	0.155692	1.725328	0.084468	0.13015
8639	LINS1	260.3199043	0.268167947	0.08495	3.156768	0.001595	0.00362
11937	C17orf103	464.6391461	0.26816294	0.135523	1.978729	0.047846	0.078566
10817	CENPBD1	251.8788215	0.267963561	0.113795	2.354793	0.018533	0.033583
7995	FARS2	235.4523384	0.267799558	0.078508	3.411115	0.000647	0.001586
9234	XPO7	1525.676872	0.26777757	0.091521	2.925846	0.003435	0.007292
10892	NSF	1133.194927	0.267766168	0.115276	2.322818	0.020189	0.036328
14084	CROCCL2	71.0231687	0.26772696	0.207121	1.292614	0.196145	0.272979
11063	C12orf66	136.1001824	0.267717116	0.118007	2.268656	0.023289	0.041263
10321	RABGGTB	1252.132619	0.267528225	0.105318	2.5402	0.011079	0.02104
8743	CHD8	1251.973002	0.267388888	0.085954	3.110838	0.001866	0.004182
12846	CLK4	736.0758463	0.267356114	0.158014	1.691975	0.090651	0.138319
11945	RSPRY1	484.0766582	0.267347323	0.135208	1.977305	0.048007	0.078777
15929	MUC6	10.58640389	0.267079121	0.36365	0.734441	0.46268	0.569339
7649	CREBBP	2113.759378	0.266929606	0.074968	3.560595	0.00037	0.000948
15065	PLCB4	557.0499114	0.266817149	0.270434	0.986625	0.323827	0.421329
11307	UHRF2	642.5125831	0.266787985	0.122568	2.17666	0.029506	0.051149
12821	ARHGAP26	1048.640095	0.266781027	0.157045	1.698761	0.089364	0.136622
9470	TTC17	1327.547033	0.266696432	0.094207	2.830965	0.004641	0.009605
12928	LDOC1	626.7797123	0.266651235	0.160139	1.665123	0.095888	0.145382
11582	NR2F2	2502.691084	0.266471891	0.12758	2.08866	0.036738	0.062175
11271	FAM53B	1104.94284	0.266448588	0.121616	2.190909	0.028458	0.049491
13164	ASB14	9.754755385	0.26628293	0.167382	1.590871	0.111639	0.166216
14289	HECW2	611.626812	0.266222886	0.216038	1.232297	0.217838	0.29882
12732	DEXI	824.0818385	0.266136748	0.154501	1.722559	0.084968	0.130809
13590	LOC400752	28.94352483	0.266053725	0.182528	1.457603	0.14495	0.209048

11195	POLR2K	916.8930921	0.266017016	0.119657	2.223162	0.026205	0.045881
16313	KLHL33	1.205012816	0.265791238	0.425496	0.624662	0.532193	0.63946
9748	LANCL2	572.840113	0.265770978	0.097233	2.733337	0.00627	0.012607
10964	MGST3	2316.241244	0.265764748	0.115621	2.298585	0.021529	0.038488
13798	TSTD1	455.1771455	0.265683174	0.191557	1.386963	0.165453	0.235037
9943	MKRN2	489.2429106	0.265652656	0.099382	2.673048	0.007517	0.014818
8919	TTF1	272.611228	0.265586512	0.087245	3.044153	0.002333	0.005128
11173	SYAP1	1250.830562	0.265345513	0.118951	2.230721	0.0257	0.045081
15303	LPAR1	251.436698	0.265330332	0.288369	0.920106	0.357517	0.45793
10685	CCDC50	3353.487798	0.265258058	0.110469	2.401193	0.016342	0.029978
11261	ATF7IP	1039.197227	0.265137054	0.120843	2.194065	0.028231	0.049139
10770	FAM122B	713.2454266	0.265127985	0.111616	2.37535	0.017532	0.031908
8696	STK24	2740.572775	0.265091639	0.08466	3.131255	0.001741	0.003923
12473	SFRS18	1472.685136	0.264771677	0.146215	1.81084	0.070166	0.110263
12543	DUS4L	196.7315711	0.264758696	0.147651	1.793136	0.072951	0.114001
13826	PRICKLE2	578.3852981	0.26470761	0.192349	1.376183	0.168765	0.239257
11779	CHD7	392.5842952	0.264513195	0.13041	2.028324	0.042527	0.070768
7796	CPSF6	1333.30374	0.264474	0.075505	3.502744	0.00046	0.001158
15869	C2orf40	45.44307737	0.264427318	0.351075	0.753192	0.451334	0.557477
12807	GALC	1185.73532	0.264228777	0.155236	1.702114	0.088734	0.135807
9903	MTR	1966.024992	0.263874879	0.09822	2.686557	0.007219	0.014289
7494	HNRNPD	3440.149136	0.263800732	0.072889	3.619224	0.000295	0.000773
14334	SPRY1	1980.429299	0.263763805	0.216119	1.220457	0.222292	0.303972
13692	FZD3	106.3804859	0.263632834	0.185751	1.419278	0.155818	0.223064
8166	METTL2A	317.7779522	0.263372231	0.078701	3.346473	0.000818	0.001965
14148	RFTN2	109.5195843	0.263364369	0.207046	1.272008	0.20337	0.281754
13618	GATS	272.7941324	0.263303318	0.181665	1.449391	0.147228	0.211913
8839	VPS24	2534.704729	0.263280542	0.08563	3.074616	0.002108	0.004674
13695	RHOBTB1	1596.884758	0.263204919	0.185501	1.418888	0.155932	0.223178
15197	OC1001314	49.02277418	0.263118961	0.277263	0.948987	0.342627	0.441919
16163	LOC28573	39.18777655	0.263021945	0.39646	0.663426	0.507058	0.614913
9493	NPEPPS	2265.964701	0.262982206	0.093218	2.82115	0.004785	0.00988
11642	PGCP	1591.130016	0.262973693	0.127446	2.06342	0.039073	0.065785
15022	C9orf68	75.55183174	0.262923871	0.262626	1.001135	0.316762	0.413317
13759	STL	4.634709878	0.262904032	0.18759	1.40148	0.161071	0.22946
16165	EPX	1.025197408	0.262854672	0.396374	0.663147	0.507236	0.615053
9351	NCOA6	1302.767309	0.262850558	0.091303	2.878877	0.003991	0.008365
14798	NLRP9	6.74944228	0.262742407	0.246552	1.065669	0.286573	0.379587
12122	PIGX	665.2476421	0.262660918	0.136677	1.921761	0.054636	0.088345
14400	MORN1	89.7880351	0.262620638	0.219263	1.19774	0.231018	0.314458
15657	STEAP1	123.3116817	0.262611153	0.320235	0.820057	0.412184	0.516013
9146	TUBGCP3	470.0329763	0.262490771	0.088678	2.960044	0.003076	0.006592
9271	VPS37C	595.9371383	0.262305736	0.090184	2.908567	0.003631	0.007677
14723	HNF1A	706.6753297	0.262207716	0.240352	1.090931	0.275303	0.366516
13360	PLEKHG1	711.9106867	0.262201774	0.172161	1.523001	0.127759	0.18744
11355	EID2B	65.20453018	0.262160632	0.121093	2.164956	0.030391	0.052456
9893	ZNF35	116.7162794	0.261799736	0.097311	2.690351	0.007138	0.014142

14179	SNHG10	54.98168694	0.261796977	0.207239	1.263259	0.206496	0.28546
9280	ZCCHC4	181.1950125	0.261706804	0.090043	2.906465	0.003655	0.007721
13451	CRTC1	322.1082867	0.261645926	0.174257	1.501494	0.133228	0.194136
14093	SLC22A5	1876.789655	0.26160014	0.202828	1.289766	0.197132	0.274177
13148	NUDT9P1	6.419789574	0.261337925	0.163813	1.595343	0.110636	0.164925
9710	KIAA0430	2022.99797	0.261326694	0.095211	2.744713	0.006056	0.012226
12685	SNX7	842.1390137	0.261163747	0.14989	1.742365	0.081445	0.125849
15569	ALPK2	1829.757927	0.261141382	0.309908	0.842641	0.399429	0.502868
11345	ZNF563	81.05829438	0.260990997	0.120416	2.167411	0.030204	0.052183
14674	PLSCR4	803.6504263	0.260868242	0.235145	1.109391	0.267261	0.356998
14543	SLC40A1	5172.099391	0.260626184	0.226072	1.152846	0.248974	0.335566
10973	NFE2L1	12050.41435	0.260565802	0.11349	2.295942	0.021679	0.038725
13986	PER1	2536.010804	0.260487087	0.196783	1.323729	0.185593	0.260122
8965	BCCIP	1000.06079	0.260313323	0.086057	3.024891	0.002487	0.005438
18012	PGA4	0.736944411	0.260305866	1.27935	0.203467	0.83877	0.912703
12115	SLC41A3	787.8804174	0.260245613	0.135318	1.923209	0.054454	0.088096
13851	SPAG8	25.49600988	0.260209383	0.19016	1.368368	0.171197	0.242266
13263	CEP70	382.4848763	0.260187936	0.166812	1.559764	0.118816	0.175594
12704	HOOK3	1351.513255	0.260162237	0.150133	1.732876	0.083118	0.128242
15212	HIST1H4H	145.6012228	0.259938638	0.275227	0.944452	0.344939	0.444461
7767	LOC65125(	1806.909282	0.259831206	0.074024	3.510115	0.000448	0.00113
11060	TBK1	737.7470448	0.259741892	0.114405	2.270375	0.023185	0.041089
11160	RNF2	579.5654198	0.259534142	0.116178	2.23394	0.025487	0.044764
16015	PCOLCE2	1112.402627	0.25949042	0.366808	0.707428	0.4793	0.586623
13740	PER2	953.2941403	0.259336271	0.18461	1.404779	0.160087	0.228375
14290	)C1002717	35.11222716	0.259307553	0.21044	1.232217	0.217868	0.29884
12418	DMXL2	849.8798079	0.259108459	0.141556	1.830435	0.067185	0.106047
10519	FAM120C	309.2759055	0.25906752	0.105134	2.464162	0.013733	0.025591
11027	ZNF224	254.4253786	0.258957863	0.113526	2.281048	0.022546	0.040076
14055	TPI1P2	16.52492271	0.258685666	0.198954	1.300227	0.193523	0.269886
15359	GPER	635.3805995	0.258543628	0.286322	0.902981	0.366536	0.467769
9220	GNL3L	583.5649725	0.258470369	0.088215	2.930008	0.00339	0.007205
15573	LOC221442	46.04211789	0.258367263	0.306754	0.842261	0.399642	0.503023
9297	SEC16A	2201.226507	0.258217057	0.089003	2.901201	0.003717	0.007837
10841	ZSCAN21	223.7835832	0.258149212	0.110133	2.343971	0.01908	0.034497
10537	IPO5	3188.452545	0.258148998	0.105065	2.457048	0.014008	0.026058
11475	ZNF616	191.1421377	0.258091501	0.121282	2.128021	0.033335	0.056942
9268	LGALS8	1031.189251	0.257657604	0.088513	2.910974	0.003603	0.00762
11242	OSGEPL1	202.8775481	0.257612581	0.116962	2.202529	0.027628	0.048171
16177	HIST1H2B(	0.861548848	0.257600933	0.390038	0.660451	0.508964	0.616691
10993	UBE3C	1887.338601	0.257494428	0.112443	2.28999	0.022022	0.039269
10699	WDR12	403.7011787	0.257372163	0.107335	2.397851	0.016492	0.030213
10460	PRPF8	5892.768153	0.257221531	0.103318	2.489603	0.012789	0.023965
16483	RBM11	14.47256389	0.257160578	0.446171	0.576373	0.564363	0.671121
10161	UBE2N	1916.057867	0.257053419	0.099049	2.595226	0.009453	0.018235
14382	ADAMTS5	750.9597888	0.25695789	0.213658	1.20266	0.229108	0.312248
11081	TANK	857.9089235	0.256816742	0.113487	2.262954	0.023639	0.041814

11589	TMED4	1700.51118	0.256710875	0.123194	2.083802	0.037178	0.062881
9284	SUPT16H	1990.822609	0.256616244	0.088316	2.905658	0.003665	0.007737
9330	CALM1	9074.194902	0.256597346	0.08887	2.887331	0.003885	0.008162
8125	ATXN10	1742.763537	0.256579682	0.076356	3.36032	0.000779	0.001878
10740	PPP3CB	995.0665188	0.25657207	0.10763	2.383838	0.017133	0.031269
8606	BRD7	1064.410392	0.256384579	0.080796	3.173243	0.001507	0.003433
11730	METT11D1	581.9315873	0.256365533	0.125503	2.042698	0.041082	0.068649
14089	LNPEP	386.6390285	0.256342325	0.19849	1.291464	0.196543	0.273436
11631	EIF4B	11018.2502	0.256044313	0.123808	2.06807	0.038633	0.06511
10650	MLL	1826.593602	0.25599074	0.10604	2.414104	0.015774	0.029031
11165	UBTD2	712.991941	0.25597985	0.114637	2.232959	0.025552	0.044858
13144	C9orf72	302.3709969	0.255904504	0.160328	1.596135	0.110459	0.164722
15244	ALS2CR12	3.539831118	0.255859364	0.273781	0.93454	0.350026	0.450039
14923	IL18	698.1461543	0.255805448	0.248434	1.029672	0.303164	0.398199
11419	ARL6IP1	5563.83841	0.255487749	0.119253	2.142399	0.032161	0.055206
13650	ATP8B1	540.7772003	0.255206823	0.177506	1.437737	0.150509	0.21611
14958	PRKG1	145.8667888	0.255163033	0.249943	1.020883	0.30731	0.4027
10287	ERCC8	174.2585958	0.255119839	0.100036	2.550288	0.010763	0.020509
12951	OSBPL10	852.3497327	0.255104431	0.153896	1.657645	0.097389	0.147396
10958	SRPK1	723.9737161	0.255085178	0.110847	2.301244	0.021378	0.038242
10056	GORASP1	737.0003595	0.254915332	0.096805	2.633276	0.008457	0.016483
10819	CDC27	1486.775434	0.254802492	0.10822	2.354485	0.018548	0.033601
11456	COQ4	783.1824672	0.254798243	0.119476	2.13263	0.032955	0.056385
10260	NAE1	711.9856884	0.254751703	0.099426	2.562214	0.010401	0.01987
11357	ZNF816A	176.688402	0.254544764	0.117595	2.164597	0.030419	0.052499
10943	C9orf6	343.8014858	0.254437734	0.110346	2.305812	0.021121	0.037832
14828	FAM27C	30.87278896	0.254384531	0.240596	1.05731	0.29037	0.383838
13702	USP49	13.59093669	0.254359383	0.179449	1.417449	0.156352	0.223665
10076	IRF2BP2	4409.165589	0.254260747	0.096796	2.626764	0.00862	0.016769
14710	C17orf107	127.904135	0.254226542	0.232201	1.094858	0.273579	0.364543
15316	PRICKLE4	87.54264467	0.254193589	0.277092	0.91736	0.358954	0.459379
9499	MAP3K7	581.8992107	0.253917189	0.090037	2.820128	0.0048	0.009906
12934	PISD	781.3001697	0.253807847	0.152531	1.663972	0.096118	0.145663
12140	TATDN1	309.1841961	0.253551835	0.13237	1.915471	0.055432	0.0895
15590	LOC28317	85.43209964	0.253538685	0.3026	0.837867	0.402105	0.505559
11196	CSNK2A1	1474.974511	0.253148913	0.113873	2.223073	0.026211	0.045888
16391	GLRB	77.49833731	0.253053916	0.419944	0.60259	0.546782	0.653823
10927	ADNP	2053.127548	0.252657306	0.109356	2.310406	0.020866	0.037429
8762	MED4	822.4094047	0.252570915	0.081434	3.101551	0.001925	0.004307
14843	SLC43A1	146.7448346	0.252506112	0.239675	1.053536	0.292095	0.385728
9948	TFDP1	1013.201966	0.252457545	0.094481	2.672058	0.007539	0.014854
10887	C3orf63	1519.236961	0.252203664	0.108524	2.323939	0.020129	0.03624
16541	LOC44056	0.612863512	0.252075615	0.448438	0.562119	0.574035	0.680226
10552	TSPAN14	3024.370144	0.252067874	0.102779	2.452528	0.014186	0.026351
9100	RBM39	3670.484077	0.252043094	0.084684	2.976275	0.002918	0.006285
10701	PSMD6	937.846201	0.251959996	0.105111	2.397089	0.016526	0.03027
11197	DCTN3	777.7958783	0.251944542	0.113364	2.222431	0.026254	0.045959

9137	SEPHS1	997.9992079	0.251903538	0.084995	2.963732	0.003039	0.00652
14170	RAB9B	9.783237085	0.251728143	0.19894	1.26535	0.205746	0.284603
14998	RSPH4A	10.03834493	0.251717196	0.249796	1.007689	0.313604	0.409851
13297	ɔC1001255	45.40281538	0.251708898	0.163056	1.5437	0.122661	0.180814
15498	C20orf26	3.399525593	0.251696078	0.291966	0.862072	0.388648	0.49154
12443	QSER1	960.0776899	0.251656125	0.138048	1.822958	0.06831	0.107606
15068	UNC5CL	493.5220505	0.251606899	0.255192	0.985952	0.324157	0.421675
16934	CR2	14.35761208	0.251590385	0.553471	0.454569	0.64942	0.751699
14892	BHMT2	3921.569781	0.251480318	0.24205	1.03896	0.298823	0.393314
9571	ZNF20	114.0342516	0.251074277	0.089943	2.791484	0.005247	0.010745
15284	DSCAML1	256.8741625	0.251059745	0.271424	0.924972	0.354981	0.455246
11640	ATAD2B	284.2871523	0.251032075	0.121622	2.06403	0.039015	0.065698
16290	ADCY2	55.45348961	0.25093452	0.398153	0.630247	0.528533	0.63596
11759	C11orf95	580.1233276	0.25083283	0.123258	2.035021	0.041849	0.069763
14050	DLEU2L	5.357760375	0.250803796	0.192823	1.300698	0.193362	0.269736
12687	HSPA8	20739.7563	0.250687152	0.143906	1.742022	0.081505	0.125922
9336	TAF8	415.5478858	0.250602097	0.086886	2.884247	0.003924	0.008237
11445	VPS41	1385.914181	0.250533328	0.117294	2.135935	0.032685	0.055977
8556	RSRC2	1308.335618	0.250513912	0.078589	3.187658	0.001434	0.003286
14720	ZNF732	3.917559665	0.250384731	0.229201	1.092425	0.274646	0.36573
16792	ZFP57	17.04610857	0.250368547	0.509498	0.491403	0.623142	0.727339
13441	TMEM123	8326.444608	0.250225052	0.166503	1.502822	0.132885	0.193786
9185	N4BP1	1063.800776	0.25010646	0.084924	2.945047	0.003229	0.006891
12884	PDXP	366.4686636	0.250090937	0.148989	1.678582	0.093234	0.14184
12727	RP2	739.4390621	0.249905387	0.144981	1.723712	0.08476	0.130529
8321	TRIM39	410.0544516	0.249734375	0.076063	3.283246	0.001026	0.002417
11630	C3orf31	108.356656	0.249712935	0.120747	2.068076	0.038633	0.06511
13723	MYO19	675.1147108	0.249651358	0.177046	1.41009	0.158513	0.226409
15182	PYGM	85.16196307	0.249615687	0.261955	0.952895	0.340643	0.439794
11782	POMT1	651.3994907	0.249551252	0.123051	2.028039	0.042556	0.070798
13847	PSIMCT-1	11.23760377	0.249520212	0.182261	1.36903	0.17099	0.24205
10409	UTP15	251.4271791	0.249380986	0.099479	2.506876	0.01218	0.022937
12962	ANKRD32	165.7113506	0.249198081	0.150667	1.653971	0.098134	0.148396
13009	CCNL1	1276.439978	0.249138891	0.151832	1.64088	0.100822	0.151912
11149	FKRP	306.3203828	0.249137801	0.111355	2.237321	0.025265	0.044419
15184	SGPP2	347.8896679	0.249024612	0.261512	0.952251	0.34097	0.440129
9993	DDX6	3239.947762	0.248938124	0.093789	2.654234	0.007949	0.015591
9283	GGPS1	727.9548529	0.248936468	0.085664	2.905966	0.003661	0.007731
10374	LRRC37B2	534.5141062	0.248750343	0.098921	2.514626	0.011916	0.022514
14974	AMACR	2938.088119	0.248706206	0.245001	1.015123	0.310047	0.405852
12829	IPO7	4151.252573	0.248617562	0.146629	1.695558	0.08997	0.137462
9486	EPN2	755.39849	0.248609014	0.088066	2.822999	0.004758	0.009831
7526	SUDS3	927.0380972	0.248551652	0.068931	3.60578	0.000311	0.000811
16045	DLGAP2	6.407877972	0.248504664	0.355885	0.698272	0.485007	0.592516
8733	RPRD1B	783.5408372	0.24848774	0.079758	3.115524	0.001836	0.004121
12521	AMMECR1	336.4477707	0.248435057	0.138206	1.797573	0.072245	0.113095
12441	NDUFC1	1376.450254	0.248265951	0.136097	1.824184	0.068124	0.107331

15772	BAALC	129.3658246	0.248231472	0.316708	0.783786	0.433166	0.538295
7880	ZNF317	697.9090788	0.248156107	0.071749	3.458659	0.000543	0.00135
14248	TXNRD2	505.5251003	0.248009332	0.199587	1.242614	0.21401	0.294414
11334	ZNF621	556.2621267	0.247895668	0.11424	2.169958	0.03001	0.051899
13818	FHIT	82.6811985	0.247785229	0.179568	1.379897	0.167618	0.237765
9244	NKRF	322.3304004	0.247747754	0.084827	2.920624	0.003493	0.007407
15501	CRISPLD1	87.99345063	0.247540468	0.287685	0.860456	0.389538	0.49257
15916	CTSK	680.055708	0.247427005	0.335791	0.736848	0.461215	0.567999
10812	BAT1	4011.708419	0.247340495	0.104888	2.358144	0.018367	0.033297
13142	ZNF248	555.6189203	0.247276126	0.154894	1.596418	0.110395	0.164652
9481	FAM21C	1504.158351	0.247222316	0.087495	2.825549	0.00472	0.009758
8894	PRKAG1	1229.069701	0.2472115	0.080937	3.054376	0.002255	0.00497
10642	RBBP4	3075.099722	0.247180055	0.102245	2.417524	0.015627	0.028782
16833	LRRC67	1.337718027	0.246949743	0.512458	0.481893	0.629882	0.733459
10449	SLC35B3	520.4281977	0.24693606	0.098975	2.494938	0.012598	0.023632
14472	IPMK	79.15732707	0.246926666	0.209968	1.176021	0.239587	0.324498
16023	INE2	16.38716951	0.246878163	0.350854	0.703649	0.481651	0.589206
12605	GUSBL2	79.46557635	0.246644944	0.139288	1.770756	0.076601	0.119116
10521	ZNF84	793.5810358	0.246511122	0.100071	2.463372	0.013764	0.025642
9980	ZNF131	447.9319239	0.246261029	0.092602	2.659339	0.007829	0.015377
13880	RNF125	228.9165731	0.246200727	0.181728	1.354773	0.17549	0.247823
14854	LOC442421	10.40729021	0.246073454	0.23406	1.051324	0.29311	0.386778
15882	NPY5R	13.721054	0.245851212	0.328459	0.748498	0.45416	0.560508
8486	GPBP1	2121.392809	0.245808187	0.076517	3.212474	0.001316	0.00304
13756	HS6ST1	877.4689735	0.245675595	0.175248	1.401878	0.160952	0.229324
9926	RPAIN	473.2262887	0.245563381	0.091682	2.678433	0.007397	0.014605
11354	SEC63	2164.8222	0.245481199	0.113382	2.165086	0.030381	0.052448
16372	TPTE	0.921306249	0.245320659	0.40371	0.607666	0.543409	0.650584
14713	MYH10	1748.09102	0.245191619	0.224224	1.093512	0.274169	0.365254
14488	KIAA1377	252.6849755	0.245136448	0.209085	1.172425	0.241026	0.326088
12535	IMMP2L	265.2264556	0.245094004	0.136578	1.794531	0.072728	0.113726
11667	MAP1LC3B	317.4631302	0.245028402	0.119053	2.058149	0.039576	0.066489
12301	RYBP	1362.891031	0.244987113	0.13138	1.864721	0.062221	0.099145
9544	ELF2	677.2608056	0.244852102	0.087372	2.802401	0.005072	0.010416
12941	C6orf225	264.0662481	0.244827652	0.147308	1.662009	0.096511	0.14618
11658	DR1	1148.492968	0.244813902	0.11888	2.059333	0.039462	0.066349
9497	RBM8A	1732.526304	0.24479199	0.086791	2.820482	0.004795	0.009897
9786	FN3KRP	886.9412044	0.244709722	0.089944	2.720686	0.006515	0.01305
10550	TUBGCP5	321.6102324	0.244567726	0.099716	2.452645	0.014181	0.026347
11509	C1QBP	1391.914441	0.244535372	0.115749	2.112636	0.034632	0.058982
12046	ZSWIM7	290.715512	0.244515913	0.125679	1.945557	0.051708	0.084139
14004	LOC400927	24.25802892	0.244515706	0.185658	1.317022	0.187831	0.262902
14882	APBA1	158.9038562	0.244448933	0.234417	1.042795	0.297043	0.391234
11257	CYLD	1648.788276	0.244381642	0.111208	2.197519	0.027983	0.048724
7931	ECD	566.0912389	0.244218477	0.07109	3.435347	0.000592	0.001462
15127	CCNJL	270.1033388	0.244116415	0.252864	0.965406	0.334341	0.433227
9337	PRPF38A	731.1900027	0.244063926	0.084628	2.883977	0.003927	0.008244

11163	CASP7	689.1801197	0.243981092	0.109252	2.233199	0.025536	0.044838
9192	GFOD2	618.4411406	0.243935952	0.082913	2.942059	0.00326	0.006952
16467	DOK7	20.22564492	0.243738663	0.418774	0.582029	0.560547	0.667208
11690	ZNF449	223.165686	0.243679509	0.118666	2.053488	0.040025	0.067112
14201	ITGA2	890.5083593	0.243644867	0.193941	1.256286	0.209012	0.28849
14254	CYP46A1	49.45336723	0.243259142	0.195951	1.241426	0.214448	0.294872
6504	TMEM199	422.1317086	0.242796963	0.06031	4.025799	5.68E-05	0.000171
12105	POLH	733.9288491	0.242749983	0.125923	1.92776	0.053885	0.087253
11612	ZNF419	163.4259242	0.242703668	0.116846	2.077127	0.03779	0.063789
17301	TEPP	1.28093753	0.242559227	0.661418	0.366726	0.713823	0.808719
15574	AMPH	12.0498317	0.242532623	0.287986	0.842169	0.399693	0.503043
13797	LOC44094	165.9818515	0.242490176	0.174767	1.387507	0.165287	0.234829
13732	CYC1	2640.888879	0.242469721	0.172357	1.40679	0.15949	0.227655
9332	EIF4G3	1544.202515	0.242432523	0.08399	2.886434	0.003896	0.008184
15194	SAMD13	13.75110868	0.242219692	0.254952	0.95006	0.342082	0.441302
12311	KLHL22	490.2835497	0.242113059	0.130086	1.861177	0.062719	0.099859
13325	ANKRD50	779.737651	0.24208255	0.157751	1.534587	0.124885	0.183693
13102	ENOX2	280.4352096	0.242069009	0.15034	1.610148	0.107366	0.160622
9718	SET	5070.608378	0.242009857	0.088231	2.742917	0.00609	0.012283
10576	SNRNP27	619.0990377	0.241897181	0.098958	2.444442	0.014508	0.026888
12538	GTF2IRD2P	233.2212715	0.241820874	0.134803	1.793882	0.072832	0.11386
14258	ANXA4	15617.44613	0.241655712	0.19476	1.240789	0.214684	0.295113
13653	CDK19	986.1217463	0.241630033	0.168103	1.437391	0.150607	0.21622
16412	ANXA3	197.85015	0.241598418	0.403349	0.598981	0.549185	0.655801
12295	TSR2	987.6952727	0.241582694	0.129375	1.867305	0.061859	0.098617
12708	RAP2C	920.3519163	0.241576219	0.139641	1.729983	0.083633	0.128997
11335	NUCKS1	7292.746562	0.241505328	0.11131	2.169672	0.030032	0.051932
13499	PDGFC	897.6744337	0.241489775	0.162168	1.489133	0.136452	0.198133
15493	FAM71E1	33.79635369	0.241371327	0.279374	0.863973	0.387603	0.490376
8571	HNRNPU	8436.01502	0.241327571	0.075828	3.182558	0.00146	0.003338
9732	SRRM1	1423.253777	0.241309883	0.08814	2.737796	0.006185	0.012458
10543	ATP5C1	3826.901854	0.241285034	0.098292	2.454777	0.014097	0.026209
11898	EIF2C4	284.9435428	0.241169953	0.121254	1.98896	0.046706	0.076944
10404	C10orf84	685.1745709	0.241129678	0.096152	2.507798	0.012149	0.022888
10757	UBP1	971.5442316	0.240939811	0.101275	2.379067	0.017357	0.031626
12276	NDUFV2	1919.120546	0.240846551	0.128617	1.872594	0.061124	0.097597
8811	MAPKAPK	475.4170199	0.240427367	0.077969	3.083637	0.002045	0.004549
15495	ABCC6	717.3775797	0.240415392	0.278522	0.863182	0.388037	0.490863
8203	MBD1	926.4478218	0.240372507	0.07216	3.331121	0.000865	0.002067
10689	SSB	1781.172244	0.240311258	0.100097	2.400788	0.01636	0.03
16510	FAM189A1	93.64552393	0.240295123	0.421998	0.569423	0.569069	0.67561
12810	C5orf54	229.7135608	0.240275922	0.141209	1.701565	0.088837	0.135932
13967	MEF2C	1787.793291	0.240274605	0.18069	1.329763	0.183596	0.25767
10652	TPR	2610.535309	0.240263848	0.099564	2.413172	0.015814	0.029099
10123	MAP3K4	506.321945	0.239922758	0.092013	2.607481	0.009121	0.017661
10945	SMC1A	2057.520506	0.239887186	0.104051	2.305466	0.02114	0.03786
7121	ZDHHC5	2676.092845	0.23988682	0.063502	3.777626	0.000158	0.000436

15199	ERBB3	3775.774199	0.239883927	0.253098	0.947791	0.343236	0.442646
9632	MED28	501.6437176	0.239821017	0.086612	2.768906	0.005624	0.011446
15136	GPT2	647.6668582	0.239652324	0.24867	0.963737	0.335178	0.434084
15143	ANGPT2	2760.332041	0.239645163	0.249147	0.961863	0.336119	0.43507
14520	CAV2	4040.132279	0.23945969	0.206337	1.160526	0.245835	0.33186
16769	UGT2B15	1.49289866	0.239342475	0.48011	0.498515	0.618121	0.722511
11048	UBB	12952.7245	0.238955219	0.10507	2.274246	0.022951	0.040719
12036	NME7	283.3909765	0.238954972	0.122594	1.949157	0.051277	0.083506
9213	C8orf41	227.973017	0.23873165	0.081369	2.933933	0.003347	0.007121
14252	NHS	601.9340144	0.238658858	0.192192	1.241775	0.21432	0.294736
9503	C9orf78	1281.72774	0.238562734	0.084611	2.819516	0.00481	0.00992
16452	TNNC2	3.803131293	0.238489398	0.406731	0.586357	0.557636	0.66437
11569	UPF2	702.120073	0.238439851	0.113934	2.092789	0.036368	0.061617
12961	BCKDHA	1454.266115	0.238391313	0.144119	1.654124	0.098102	0.148361
11664	ZNF280C	82.21977155	0.238244105	0.115724	2.058722	0.039521	0.066417
17630	TMEM215	0.423392823	0.238123216	0.838456	0.284002	0.776409	0.863161
17545	DAZ1	8.445546119	0.238030207	0.786193	0.302763	0.76207	0.851352
11946	C1orf156	160.8559764	0.237989182	0.120366	1.977215	0.048017	0.078787
11820	ESYT1	3250.096268	0.237935925	0.118091	2.01486	0.043919	0.072825
17157	SCGBL	0.368236133	0.237878754	0.59961	0.396722	0.691572	0.790086
17190	LENEP	0.305200754	0.237878708	0.611266	0.389157	0.69716	0.794941
17298	KDM4DL	0.353190853	0.237878695	0.647434	0.367417	0.713308	0.808275
17394	FKSG29	0.247662844	0.23787855	0.693739	0.342894	0.731679	0.824469
17502	USP50	0.226945004	0.237878428	0.756373	0.314499	0.753142	0.843466
17765	AKAP14	0.217268186	0.237878035	0.938692	0.253414	0.799948	0.882622
17966	RAG2	0.288271976	0.237877739	1.114065	0.213522	0.83092	0.906538
17927	LOC12653(	0.182057987	0.23787769	1.066261	0.223095	0.823461	0.900318
17977	IFNW1	0.152641665	0.237877445	1.128884	0.210719	0.833106	0.908367
18183	LOC14477(	0.14742052	0.237876451	1.426329	0.166775	0.867547	0.935202
18184	HMX1	0.147334286	0.237876438	1.429856	0.166364	0.867871	0.935485
18254	OR52R1	0.126779248	0.237875827	1.567659	0.15174	0.879392	0.944285
18326	ATP1B4	0.150394278	0.237875328	1.701149	0.139832	0.888793	0.950577
18351	OR5A1	0.106072034	0.237874966	1.742656	0.136501	0.891425	0.952033
18409	DBX1	0.151467447	0.237874481	1.882057	0.126391	0.899423	0.957585
18446	ZPBP	0.091888416	0.23787378	1.95572	0.12163	0.903192	0.96008
18448	RIT2	0.08812687	0.237873725	1.965825	0.121005	0.903687	0.960168
18504	GPR6	0.10782819	0.237872761	2.157049	0.110277	0.91219	0.966216
18516	OR2T35	0.086262409	0.237872301	2.210611	0.107605	0.914309	0.967909
18525	OR5B12	0.069145459	0.237871785	2.269384	0.104818	0.91652	0.969755
18691	OR51A7	0.091362398	0.237867649	2.867948	0.08294	0.933899	0.979957
18675	CD300LD	0.068762358	0.237867032	2.91407	0.081627	0.934943	0.979957
18699	TTLL8	0.070693554	0.237867032	2.91407	0.081627	0.934943	0.979957
18672	ATXN8OS	0.068796644	0.237867032	2.91407	0.081627	0.934943	0.979957
18673	AWAT1	0.048087795	0.237866787	2.91407	0.081627	0.934943	0.979957
18679	FBXW12	0.050835768	0.237866787	2.91407	0.081627	0.934943	0.979957
18689	OR4F4	0.046877228	0.237866787	2.91407	0.081627	0.934943	0.979957
18690	OR4F5	0.047572343	0.237866787	2.91407	0.081627	0.934943	0.979957

18692	OR51G1	0.050153627	0.237866787	2.91407	0.081627	0.934943	0.979957
18694	RBMY1A1	0.050013461	0.237866787	2.91407	0.081627	0.934943	0.979957
18696	SPATA3	0.049681277	0.237866787	2.91407	0.081627	0.934943	0.979957
18700	ZCCHC13	0.050400456	0.237866787	2.91407	0.081627	0.934943	0.979957
18685	LOC285045	0.04924983	0.237866787	2.91407	0.081627	0.934943	0.979957
18677	DEFB126	0.030452161	0.237866542	2.91407	0.081627	0.934943	0.979957
18680	GNAT3	0.029368297	0.237866542	2.91407	0.081627	0.934943	0.979957
18682	KRTAP1-3	0.030873529	0.237866542	2.91407	0.081627	0.934943	0.979957
18687	OR11L1	0.030633055	0.237866542	2.91407	0.081627	0.934943	0.979957
18688	OR4D9	0.030675717	0.237866542	2.91407	0.081627	0.934943	0.979957
18698	SPINT3	0.030089998	0.237866542	2.91407	0.081627	0.934943	0.979957
16472	CBLN1	15.79190647	0.237791694	0.409208	0.581102	0.561172	0.667771
8355	GOLGA1	532.1940905	0.237742355	0.072698	3.270257	0.001074	0.002521
10750	MBD5	394.6693099	0.237726208	0.099855	2.380716	0.017279	0.031506
12624	CMPK1	3446.489242	0.237455708	0.134532	1.765055	0.077554	0.120417
13687	ZNF321	81.46293663	0.237310001	0.166701	1.423566	0.154572	0.221361
14814	KLF2	542.5000463	0.237293211	0.223784	1.060365	0.288978	0.382359
14075	C9orf150	339.4188644	0.237142256	0.183022	1.295704	0.195078	0.271667
11101	PNMA1	1168.681067	0.237131278	0.105175	2.254644	0.024156	0.042652
15356	C8orf46	92.49302186	0.236533061	0.261836	0.903362	0.366334	0.467572
14346	ZBED3	75.41060208	0.236515594	0.1946	1.215392	0.224217	0.306348
14077	TCTE3	44.66701955	0.236290168	0.182543	1.294434	0.195516	0.272238
13276	RAB30	58.90458449	0.236239513	0.152215	1.552013	0.120659	0.178144
11312	SFRS12	1459.931983	0.236208003	0.108597	2.17509	0.029623	0.05133
15503	CXCL12	1934.377802	0.235862241	0.274225	0.860104	0.389732	0.492752
9545	TTLL5	391.5346435	0.23576645	0.08413	2.802409	0.005072	0.010416
11283	PHC1	597.4803744	0.235718941	0.107914	2.184326	0.028938	0.050272
9827	MSL2	748.8124391	0.235677823	0.08697	2.709873	0.006731	0.013426
12797	LOH12CR2	27.90763794	0.235518554	0.138154	1.704749	0.088241	0.135158
16859	STAB2	4.544218811	0.235474146	0.493837	0.476826	0.633486	0.736518
11815	HDHD1A	497.5986675	0.23497796	0.116497	2.017036	0.043692	0.072484
14393	FGD5	1198.738876	0.234879732	0.195796	1.199617	0.230288	0.313616
12096	PARP11	264.6767286	0.234492577	0.121461	1.930593	0.053533	0.086748
15639	TCF21	117.6699448	0.234454713	0.284285	0.824718	0.409532	0.513283
13578	RAB11FIP3	2492.533862	0.234397089	0.160393	1.461388	0.143909	0.207745
11469	VAMP4	464.3276663	0.234276196	0.110055	2.128714	0.033278	0.056868
12625	KLC2	499.0902391	0.234122413	0.132653	1.764919	0.077577	0.120437
11100	MRPS10	791.2342431	0.233988153	0.103778	2.254694	0.024153	0.04265
11544	TSFM	643.2336051	0.233865438	0.11136	2.100077	0.035722	0.060654
11939	GTF3C3	598.0239877	0.233819643	0.118174	1.978612	0.04786	0.078574
14263	C16orf71	15.14678398	0.233810243	0.188569	1.239917	0.215006	0.295473
12553	TMPO	1236.606375	0.233695559	0.130582	1.789648	0.073511	0.114784
13781	ICOSLG	598.2510565	0.233652371	0.167741	1.392936	0.163639	0.232747
15839	FAM71F2	9.298382728	0.23357005	0.306924	0.761002	0.446656	0.552708
12475	IQSEC1	1593.988603	0.233564785	0.129037	1.810063	0.070286	0.110426
9247	MARK3	977.4401508	0.233563966	0.079987	2.920025	0.0035	0.007419
12081	CSNK1G3	758.4993691	0.233483042	0.120768	1.933313	0.053198	0.086311

12394	PMS1	279.9870208	0.233179944	0.126914	1.837306	0.066165	0.104639
13404	PGAM4	110.4098847	0.233174741	0.154284	1.511332	0.130704	0.191132
10936	AP3D1	3077.607624	0.23302664	0.100962	2.308053	0.020996	0.037632
12187	RLF	756.4923438	0.232975635	0.122587	1.900495	0.057368	0.092268
15163	PCDHGA6	55.99788253	0.232911026	0.243255	0.957478	0.338326	0.437349
13823	GCH1	282.6129941	0.232880266	0.168932	1.378543	0.168036	0.238275
17138	C21orf128	1.059117068	0.232817876	0.580271	0.401222	0.688256	0.787158
8232	RNF214	329.2922846	0.232773926	0.070131	3.319124	0.000903	0.00215
11697	EBAG9	793.3889265	0.232734304	0.113421	2.051942	0.040175	0.067323
12767	PRKD3	1125.85293	0.232700112	0.135786	1.713723	0.08658	0.132925
14047	SPDYE1	11.24282555	0.232698183	0.178697	1.302194	0.19285	0.2691
10484	POLR2C	1997.51334	0.232627408	0.093867	2.47826	0.013202	0.024684
12368	MRPL3	1577.189939	0.232366644	0.125941	1.84504	0.065032	0.103063
11808	ERLEC1	1809.827385	0.232340249	0.115102	2.018559	0.043533	0.072264
15714	HYMAI	4.519845207	0.232286416	0.290269	0.800246	0.423568	0.528363
15243	ST8SIA4	1216.471697	0.231965563	0.248177	0.934676	0.349955	0.450008
11951	IRAK4	597.5051198	0.231866481	0.117433	1.974455	0.04833	0.079267
14051	MCART2	6.3371091	0.231842441	0.17825	1.300662	0.193374	0.269736
10697	CRNKL1	768.7647403	0.231097351	0.096351	2.398505	0.016462	0.030165
9997	VPS53	809.8833962	0.231050452	0.08707	2.653632	0.007963	0.015613
14103	FZD6	985.7262778	0.231029305	0.179362	1.288064	0.197724	0.274806
13727	PPIL6	94.21510355	0.231000544	0.164114	1.407558	0.159262	0.227413
12109	FBXO31	647.2173141	0.23096115	0.119942	1.925614	0.054153	0.087657
16649	CBWD5	2.798029838	0.230892193	0.431918	0.534574	0.592945	0.698078
15108	ATP1B2	328.0300184	0.230591742	0.236976	0.97306	0.330523	0.428811
9645	KDM4C	602.6858024	0.230478199	0.083373	2.764414	0.005703	0.011589
9488	YTHDF2	1677.598182	0.230471274	0.081654	2.82253	0.004765	0.009843
12099	MGST2	970.7953931	0.230368655	0.119414	1.929157	0.053711	0.087015
10856	GPR107	2691.428037	0.23029923	0.098505	2.337947	0.01939	0.03501
15641	BAGE2	2.927531318	0.230071831	0.279113	0.824297	0.409771	0.513517
12324	SMC6	651.9999014	0.229931749	0.12377	1.857737	0.063206	0.100528
13380	KIAA0182	1601.861057	0.229923938	0.15153	1.517345	0.12918	0.189242
14221	GBE1	1806.697015	0.229918559	0.183748	1.251272	0.210835	0.290597
12903	NCK1	762.1349021	0.229867027	0.137452	1.67235	0.094455	0.143491
13992	KALRN	555.7199853	0.229850051	0.174103	1.320198	0.186769	0.261639
8832	LEO1	442.7879348	0.229802589	0.074683	3.077054	0.002091	0.004639
12603	CHKB	275.3713982	0.229796907	0.129732	1.771317	0.076508	0.11899
14256	DC1002868	21.0302126	0.229661206	0.185054	1.241052	0.214587	0.295031
10028	ZC3H11A	2891.732732	0.229186161	0.086729	2.64256	0.008228	0.016083
14630	PAX8	3076.577428	0.229174	0.204128	1.122696	0.261567	0.350442
13328	ACADVL	8149.796088	0.229093659	0.149317	1.534279	0.124961	0.183776
14673	DC1001709	36.07960125	0.22908812	0.206406	1.109892	0.267046	0.356734
8247	PRPSAP1	559.454756	0.228979642	0.069101	3.313709	0.000921	0.002188
15875	GPR20	11.96882063	0.228872736	0.305338	0.749571	0.453513	0.559956
12381	GSPT2	222.2018268	0.228858034	0.124268	1.841653	0.065526	0.103737
10602	FAM82B	865.0713277	0.228759424	0.093934	2.435322	0.014879	0.027507
13552	KIAA1530	215.3192946	0.228634366	0.155476	1.470541	0.141415	0.204537

13436	RNF168	132.3757232	0.228459139	0.151885	1.504157	0.132541	0.193356
9024	RNF8	294.3158244	0.228436923	0.076047	3.003879	0.002666	0.00579
14078	USP6	51.66632685	0.228318407	0.176411	1.294241	0.195582	0.272312
17577	KCNB2	0.650892482	0.228255314	0.766959	0.297611	0.766	0.854157
9784	YWHAB	6968.523442	0.228245125	0.083866	2.72154	0.006498	0.013018
11557	PHF15	1480.24059	0.228010648	0.108804	2.095615	0.036116	0.061254
12350	EIF4A2	7991.150017	0.227951599	0.123269	1.849223	0.064426	0.102243
13402	MINPP1	720.5943893	0.227939821	0.15078	1.511743	0.130599	0.191007
15254	P2RY14	116.6260354	0.227876658	0.244471	0.932121	0.351274	0.451378
14799	TCF4	2332.533618	0.227864644	0.213846	1.065557	0.286624	0.379628
15013	NEIL1	69.38884471	0.227719542	0.226577	1.005041	0.314877	0.411104
13375	C2orf18	2820.338255	0.227714102	0.149924	1.518867	0.128796	0.188736
10761	MTCH1	3568.932286	0.22709733	0.095496	2.378092	0.017402	0.031698
15483	ADAMTS1:	59.90390681	0.226939081	0.262061	0.865978	0.386502	0.4893
9624	NUP214	1379.179562	0.226873555	0.081878	2.77086	0.005591	0.011387
14300	OSTCL	5.664646038	0.226769326	0.18444	1.229504	0.218883	0.300023
14931	HSD17B7	432.2977416	0.226593645	0.220921	1.025678	0.305043	0.400425
11874	ZNF236	244.1657368	0.226547082	0.113365	1.998384	0.045675	0.075398
13502	MAN2C1	1386.968278	0.226426021	0.152152	1.488154	0.13671	0.198462
11964	ЭC1001327	132.1350866	0.226226322	0.114826	1.970167	0.048819	0.079982
12270	SNX3	4329.066807	0.226107913	0.120415	1.877736	0.060417	0.096515
12034	FOXO3	1719.127439	0.226043602	0.115905	1.950254	0.051146	0.083307
14734	ADCK1	152.7427058	0.22599199	0.207537	1.088925	0.276187	0.367418
10226	APTX	452.5131011	0.225890653	0.087794	2.572968	0.010083	0.019327
16150	ENPP3	5529.067897	0.225444494	0.3384	0.666208	0.505278	0.613248
9475	TRMT12	227.7659149	0.225378495	0.079678	2.828609	0.004675	0.009671
16765	SPDYE2	1.141750793	0.225354619	0.451048	0.499625	0.617339	0.72177
10769	USP3	518.8230304	0.22529559	0.094823	2.375953	0.017504	0.031859
14899	HOXA6	18.19647868	0.225061957	0.217046	1.036932	0.299768	0.394372
9915	NUP43	596.5293912	0.224983259	0.083875	2.682379	0.00731	0.014451
15099	LGMN	5218.349191	0.224570508	0.229927	0.976705	0.328715	0.426727
12082	JMJD5	109.2479003	0.224564136	0.116171	1.933052	0.05323	0.086356
13778	TMEM63A	1297.80819	0.224423109	0.161049	1.393506	0.163467	0.232553
11104	TAF1	801.160386	0.22433342	0.099573	2.252954	0.024262	0.042828
13177	RPGR	172.8124793	0.224166965	0.141296	1.586501	0.112626	0.167533
15058	PROCA1	27.70375729	0.224056116	0.226804	0.987885	0.323209	0.420721
15227	C17orf57	50.28103905	0.223961236	0.238298	0.939837	0.347301	0.447064
10948	TM9SF3	4863.172732	0.223908609	0.097179	2.304075	0.021218	0.037989
12664	RALB	2690.736597	0.223876139	0.127797	1.751811	0.079806	0.123522
11011	MED14	1366.901911	0.223573696	0.097786	2.286357	0.022233	0.039578
16080	C15orf26	1.465028874	0.223479263	0.32478	0.688094	0.491394	0.598993
11851	STIM2	521.8113006	0.223334524	0.111319	2.006252	0.044829	0.074146
15463	FGFR2	699.9289724	0.223262653	0.256107	0.871755	0.383342	0.485927
11481	HELZ	1284.642691	0.22297174	0.104829	2.127003	0.03342	0.057056
17085	LECT2	0.819860137	0.222960309	0.535536	0.416331	0.677167	0.77689
12463	ATP5J	1720.516835	0.222683437	0.122721	1.814551	0.069593	0.109451
15781	FAM166B	5.148977751	0.222659525	0.285086	0.781026	0.434787	0.540033

15313	TMEM139	706.7742805	0.222611678	0.242439	0.918219	0.358504	0.458894
12504	MCFD2	4617.64986	0.222292962	0.123313	1.802677	0.071439	0.111986
10723	TMCO7	389.5063254	0.222210863	0.093052	2.38804	0.016939	0.030963
13556	PHIP	1446.869462	0.222132383	0.151247	1.468671	0.141922	0.205209
9628	C12orf41	525.8521115	0.22182656	0.080103	2.769262	0.005618	0.011437
8759	COIL	383.9603026	0.221799061	0.071486	3.102677	0.001918	0.004292
12373	ERCC6	219.0465443	0.221697208	0.120286	1.843089	0.065316	0.103472
14849	ARHGEF15	655.1704693	0.221674723	0.210707	1.052051	0.292776	0.386471
17042	SLC44A5	126.906913	0.221618841	0.517026	0.428642	0.668184	0.768514
15120	DDIT3	853.2286736	0.221529306	0.229054	0.967148	0.33347	0.43227
10954	FXR2	683.515529	0.221527694	0.096229	2.302095	0.02133	0.038167
16223	SHISA3	130.0007868	0.221208085	0.340883	0.648927	0.516386	0.623918
16319	CYP4B1	13.15280554	0.221152793	0.354537	0.62378	0.532772	0.639921
17006	SLC15A1	192.9931153	0.22105409	0.504293	0.438345	0.661136	0.762021
12208	TIPRL	899.5808142	0.220822721	0.116568	1.894362	0.058177	0.093408
11785	ARIH2	1058.637518	0.220724622	0.108887	2.027089	0.042653	0.070942
12216	C20orf108	1564.902873	0.220664696	0.116677	1.891239	0.058593	0.093999
14321	LRRC8C	732.4038674	0.220593828	0.180287	1.223574	0.221113	0.302614
14186	CCDC75	69.51628373	0.220586799	0.174893	1.261268	0.207212	0.286319
11928	HBXIP	2097.233583	0.220493483	0.111333	1.980493	0.047648	0.078299
13409	ZNF629	848.7685141	0.220144663	0.145761	1.510308	0.130965	0.191442
14561	ACY1	1272.066567	0.22014463	0.191793	1.147827	0.25104	0.337932
9994	KCTD2	1014.674321	0.220061557	0.082913	2.654111	0.007952	0.015596
13473	GDE1	1697.49368	0.219947866	0.147025	1.495986	0.134657	0.195898
13535	LPHN2	2366.966005	0.21994179	0.149129	1.474844	0.140254	0.203113
12415	MRPL16	776.2835207	0.219890555	0.120081	1.831192	0.067072	0.105894
16478	KCNE1	11.96931608	0.219873993	0.380178	0.578345	0.563031	0.66974
12006	WDR43	892.9441807	0.219601638	0.112087	1.959211	0.050088	0.081778
12330	PIGB	334.4153255	0.219510879	0.118304	1.855482	0.063527	0.10099
10798	CSDE1	11467.86484	0.219453077	0.092935	2.361357	0.018208	0.033052
16064	HLA-G	719.7991116	0.21933608	0.316347	0.693339	0.488097	0.595492
12876	CASD1	792.1444728	0.219288305	0.130339	1.682447	0.092482	0.140774
10939	RTN3	3889.54893	0.219237364	0.095032	2.306978	0.021056	0.037729
11132	GPR108	1438.444604	0.219131297	0.097741	2.241955	0.024964	0.043958
12839	RAP1A	1674.083225	0.219086192	0.129396	1.69315	0.090427	0.138053
11458	GOLGA2	1547.022119	0.218927885	0.102696	2.131797	0.033024	0.056488
13420	PUS10	183.7382146	0.218906149	0.145166	1.507974	0.131561	0.192156
13153	ARL17A	65.31954803	0.218895763	0.137341	1.593808	0.110979	0.165384
12145	RAPGEF1	2824.343167	0.218889733	0.114389	1.913551	0.055678	0.089859
15223	KCNN2	24.89956671	0.218857708	0.232649	0.94072	0.346848	0.446599
15815	AKAP12	5264.292488	0.21865917	0.28335	0.771693	0.440296	0.5457
14983	KLHDC9	97.90381425	0.218568225	0.21629	1.010532	0.31224	0.408478
9974	VPS39	1811.161647	0.218551376	0.0821	2.66203	0.007767	0.015264
11606	ZNF605	448.2219254	0.218523418	0.105099	2.079215	0.037598	0.063497
12351	NDUFS4	808.2032078	0.218376355	0.118091	1.849228	0.064425	0.102243
14578	SQLE	525.0461134	0.21832317	0.191074	1.142613	0.253199	0.340442
11922	HDAC2	1417.299819	0.218281599	0.110141	1.981844	0.047497	0.07809

12838	CRAMP1L	557.4697555	0.217974732	0.128736	1.693197	0.090418	0.13805
13488	ZNF432	155.139192	0.217869203	0.146105	1.491184	0.135913	0.197513
13433	EVI5	425.9400469	0.217805934	0.144765	1.504544	0.132441	0.193254
14478	PLXNB1	1456.1724	0.217778896	0.18538	1.174771	0.240086	0.32504
16298	MYO3A	177.0440925	0.217715115	0.346714	0.627939	0.530044	0.637464
14246	SLC39A13	1537.841064	0.217447581	0.174913	1.243173	0.213804	0.294172
14045	C14orf106	340.2230626	0.217402309	0.166885	1.302707	0.192675	0.268911
12220	SNAPC5	98.09166183	0.21712018	0.114825	1.890885	0.05864	0.094059
12120	SAMM50	1087.306835	0.217054958	0.112903	1.922492	0.054544	0.088211
14405	KCTD14	340.0718416	0.216877184	0.18121	1.196826	0.231375	0.314833
14421	VPS18	1025.021575	0.216853428	0.181945	1.191865	0.233314	0.31712
14817	CCDC152	130.0612723	0.216771428	0.204599	1.059495	0.289375	0.382806
15889	EPHB1	85.55024971	0.216747105	0.290555	0.745976	0.455682	0.562139
14628	GATC	35.82395285	0.216467844	0.19252	1.124389	0.260848	0.349527
12229	C4orf52	492.1953282	0.216459419	0.1146	1.888828	0.058915	0.09443
13547	CALML4	717.493211	0.216443665	0.14704	1.472004	0.14102	0.20404
16736	OR56B4	0.710874645	0.216313052	0.42439	0.509703	0.610259	0.714728
11610	ULK2	543.615512	0.216234545	0.104025	2.078669	0.037648	0.06356
11633	USP47	1743.40081	0.21581826	0.104371	2.067804	0.038658	0.065137
11645	KIAA0753	336.7729028	0.215760612	0.10462	2.062335	0.039176	0.065941
12224	NXF1	1423.939312	0.215742461	0.114128	1.890351	0.058711	0.094135
15576	SMN1	4.027870482	0.215728331	0.256337	0.84158	0.400023	0.503394
10876	PTPN9	1249.012946	0.21557287	0.092503	2.330431	0.019783	0.035654
17842	CLEC2L	0.950619084	0.215418725	0.909883	0.236754	0.812847	0.892979
12438	TNKS	1152.099013	0.215298692	0.117967	1.825078	0.067989	0.107144
12026	TMEM167B	1370.913315	0.215271096	0.110157	1.95423	0.050674	0.082596
11891	GRPEL1	881.3282264	0.215248655	0.108087	1.991442	0.046432	0.076539
10956	C22orf29	302.9562509	0.215179919	0.093507	2.301216	0.021379	0.038242
14005	OC1003024	21.1833843	0.214909012	0.163212	1.316749	0.187923	0.263011
14987	FAM149A	898.9206457	0.214793461	0.21267	1.009983	0.312504	0.408703
11367	ANAPC16	2413.055914	0.214599502	0.099253	2.162157	0.030606	0.052776
12194	TEP1	688.5274913	0.214558919	0.113023	1.898372	0.057647	0.092664
14236	RGPD1	313.4169012	0.214364278	0.172011	1.246221	0.212683	0.292835
12782	BBS5	158.7303546	0.214352272	0.125378	1.709642	0.087332	0.133922
12738	SMAD3	1729.08491	0.21433099	0.124556	1.720759	0.085295	0.13125
12322	MEX3C	795.4197958	0.214158721	0.115221	1.85868	0.063073	0.100332
11212	CAMTA1	514.3948002	0.213833435	0.096468	2.216618	0.026649	0.046589
12431	MLLT6	2840.003492	0.213777215	0.117024	1.826787	0.067732	0.10679
10502	PCBP1	9271.473348	0.213736037	0.086506	2.470753	0.013483	0.025165
10638	SF3A1	2567.217179	0.213593208	0.088277	2.419585	0.015538	0.02863
13037	ZFP82	102.3362698	0.213495644	0.130749	1.632863	0.102498	0.154104
11750	CTNS	459.3809608	0.213458699	0.104804	2.036748	0.041675	0.069521
11926	TMEM131	1664.141097	0.213441208	0.10775	1.9809	0.047603	0.078237
12638	PAPOLG	350.6671836	0.213383941	0.121141	1.761446	0.078163	0.121227
11337	UBXN2A	215.5172968	0.213200359	0.098268	2.169588	0.030038	0.051934
10213	TSPAN3	4580.38515	0.213099794	0.082693	2.577015	0.009966	0.019127
12477	MGC57346	139.1573915	0.213094159	0.117794	1.809043	0.070444	0.110666

12834	ZFC3H1	730.176024	0.213068133	0.125777	1.694012	0.090263	0.137856
14634	GUCY1A3	3045.548228	0.212933612	0.189796	1.121907	0.261902	0.350803
8746	ADD1	5547.726639	0.212749397	0.068413	3.109767	0.001872	0.004196
8540	RBM45	149.6982646	0.212702283	0.06662	3.192786	0.001409	0.003234
14900	FAM86B1	75.45660135	0.212510086	0.205062	1.036322	0.300052	0.394719
14040	SH2B1	1046.489342	0.212077161	0.162441	1.305568	0.1917	0.267628
14030	SEC24A	1408.326121	0.212027862	0.161823	1.310242	0.190114	0.265604
10428	DHX57	503.2595236	0.211857133	0.084727	2.50046	0.012403	0.023314
16338	CHRM4	2.890003049	0.211826311	0.343756	0.616211	0.537755	0.645184
11812	C6orf70	357.0460529	0.211713932	0.104945	2.017388	0.043655	0.072436
12367	ERCC2	368.1166317	0.211675243	0.114669	1.845966	0.064897	0.102858
15247	C6orf165	43.51966681	0.211261198	0.226131	0.934241	0.35018	0.450178
11672	PDS5A	2635.160179	0.211209626	0.102657	2.05743	0.039645	0.066575
15540	FAM38B	388.8055503	0.211095701	0.248627	0.849045	0.395856	0.499303
9955	ZXDC	1089.554369	0.211081862	0.079089	2.66891	0.00761	0.014983
14373	CCDC73	51.05366715	0.211062675	0.174963	1.206325	0.227692	0.310512
12578	VDAC1	7697.308005	0.210882624	0.118533	1.779111	0.075222	0.117222
9290	BSDC1	1762.517969	0.210796891	0.072606	2.903318	0.003692	0.00779
15559	GPR88	6.933893423	0.21069842	0.249488	0.844523	0.398377	0.50187
12162	SMG5	2455.197532	0.210598655	0.110392	1.907733	0.056426	0.090953
10548	DNTTIP2	1195.755606	0.21050058	0.085792	2.453629	0.014142	0.02628
16243	EFHC2	109.1046777	0.21043032	0.327516	0.642504	0.520546	0.628161
10979	DHX35	261.7042982	0.210353048	0.091687	2.294244	0.021777	0.038878
13183	STYX	499.8721398	0.210297435	0.132707	1.584679	0.113039	0.168071
11767	ATP9B	564.1192621	0.210051359	0.103419	2.031066	0.042248	0.070376
17835	NPPC	0.86916411	0.209839476	0.880829	0.23823	0.811703	0.892124
14750	EVC2	150.0564606	0.209839298	0.1936	1.083881	0.278418	0.369984
12901	MLL2	1977.56966	0.209657361	0.125304	1.673189	0.09429	0.143259
17583	ARX	31.25283166	0.209557971	0.705731	0.296938	0.766514	0.854438
13379	TMX3	1455.363348	0.209475334	0.138016	1.517758	0.129075	0.189103
14970	PPP1R16B	517.6407277	0.209329115	0.206099	1.015674	0.309785	0.405617
11721	RIOK2	516.0109741	0.209309705	0.102309	2.045858	0.04077	0.06818
14471	ALOX12	5.329166723	0.209276734	0.177943	1.176089	0.239559	0.324483
14977	FOXJ3	3423.272509	0.208929542	0.205932	1.014556	0.310317	0.406125
10729	ZNF143	334.110941	0.208736099	0.087426	2.38758	0.01696	0.030984
14357	SCARF1	530.5306331	0.20856972	0.172151	1.211552	0.225684	0.308117
10417	ZFYVE20	501.6781325	0.208420407	0.083201	2.505033	0.012244	0.023039
12611	ZNF623	342.7643575	0.208314884	0.117833	1.767876	0.077082	0.119806
13688	C17orf86	149.2251079	0.208300427	0.146416	1.422663	0.154834	0.22172
10361	SFRS3	3861.187903	0.208252996	0.082557	2.522525	0.011652	0.022042
13059	SOCS5	717.8348727	0.20814259	0.128011	1.625974	0.103955	0.156032
13777	SBF1	1702.389781	0.208082594	0.149262	1.394076	0.163295	0.232325
8798	GANC	339.7573833	0.207958259	0.067367	3.086964	0.002022	0.004505
15601	PKDCC	468.1590287	0.207712123	0.248803	0.834845	0.403805	0.507338
11319	DHX16	650.0475098	0.207639816	0.095526	2.173646	0.029732	0.051486
9929	DCAF5	1467.764936	0.207620784	0.077541	2.677548	0.007416	0.014641
16534	IL12RB2	46.63712683	0.207274406	0.36791	0.563384	0.573173	0.679495

14473	GMCL1	437.9730226	0.207246118	0.176238	1.175942	0.239618	0.324518
13677	GCDH	344.687462	0.207115119	0.145023	1.428158	0.153246	0.219623
11351	CUL2	651.1236935	0.20695843	0.095574	2.165434	0.030354	0.052416
14281	NKTR	1297.570947	0.206907043	0.167589	1.23461	0.216976	0.297804
12929	ANAPC2	602.4316831	0.206723666	0.124188	1.664602	0.095992	0.145506
15554	AK3L1	1151.746905	0.206358007	0.244125	0.845298	0.397944	0.501421
12819	COX16	744.5578973	0.206339785	0.121398	1.699702	0.089187	0.136372
10420	NFATC2IP	706.5814669	0.206281499	0.082387	2.503807	0.012287	0.023112
12518	KIAA0100	3730.240595	0.206062464	0.114568	1.79861	0.07208	0.112865
15077	PLEKHJ1	786.0619731	0.205970181	0.209347	0.983872	0.325179	0.422752
11909	TAF3	188.6322165	0.205943501	0.103739	1.98521	0.047121	0.077556
13369	METTL8	337.6049567	0.205906048	0.13535	1.521282	0.128189	0.187945
15014	ROBO4	1576.392357	0.205773171	0.204775	1.004875	0.314957	0.411181
16226	PAQR6	56.3638055	0.205746535	0.31748	0.64806	0.516946	0.62447
17614	NR2E1	86.61004637	0.205715927	0.70878	0.290239	0.771633	0.858679
13193	BCL10	509.822352	0.205706332	0.130096	1.581192	0.113834	0.169125
12352	ATG5	670.5658302	0.205564852	0.111168	1.849141	0.064437	0.102254
14341	ZNF502	71.84283467	0.205493128	0.168843	1.217069	0.223578	0.305587
11341	UTP18	589.1349522	0.20543532	0.094723	2.168795	0.030098	0.05202
12694	KIAA1432	749.5112932	0.205387073	0.118346	1.735485	0.082655	0.127629
15659	PIP5K1P1	2.27094665	0.205366325	0.25073	0.819073	0.412745	0.516616
9747	RBMX	3500.320205	0.205003825	0.075	2.733389	0.006269	0.012606
17331	TAGLN3	7.780259217	0.204823156	0.568574	0.36024	0.718667	0.812798
13575	COX7A2	1442.417148	0.204814507	0.140022	1.462729	0.143542	0.20726
10372	SRP14	4166.092241	0.204788345	0.081397	2.515914	0.011872	0.022436
13215	PTEN	2313.662105	0.204784263	0.13001	1.575145	0.115223	0.170903
13937	SLFN12	169.4913168	0.20470317	0.152796	1.339718	0.180337	0.253626
14395	ZNF774	14.17295287	0.204669932	0.170688	1.199085	0.230495	0.313854
13249	MAP4K2	267.396598	0.204550256	0.130873	1.562972	0.118059	0.174672
13752	HS2ST1	1536.726798	0.204413198	0.145779	1.402209	0.160853	0.229267
11371	TNFAIP1	1716.504464	0.204051453	0.094439	2.160661	0.030722	0.052957
17345	IGLL3	0.558214825	0.203990588	0.575599	0.354397	0.723041	0.817078
15893	ETNK2	653.1651158	0.203837278	0.273697	0.744755	0.45642	0.562872
13064	MAPKBP1	429.0918579	0.203744241	0.125403	1.62471	0.104224	0.156376
11248	MFF	1428.717086	0.203669197	0.092533	2.201055	0.027732	0.048327
11172	SF1	3773.958231	0.203560694	0.091244	2.230946	0.025685	0.045064
12137	MIA3	1689.966213	0.20326502	0.106082	1.916104	0.055352	0.089385
15441	TUBB2A	723.1795697	0.203223743	0.232069	0.875704	0.381191	0.483888
11915	ARHGAP21	1501.778699	0.203060414	0.102385	1.983305	0.047333	0.07786
16061	HLA-J	70.53990973	0.202869122	0.29216	0.694377	0.487446	0.594884
16033	TCAP	8.860118073	0.202753861	0.288739	0.702206	0.482551	0.590004
11670	INTS4	518.0031814	0.202598129	0.098466	2.057535	0.039635	0.066571
12805	FKBP3	1028.856776	0.202591966	0.119004	1.702395	0.088681	0.135747
12501	SLC35D2	684.8010237	0.202496642	0.112283	1.803444	0.071319	0.111824
14934	ZNF534	4.585486714	0.202420606	0.19749	1.024968	0.305378	0.400812
15834	C8orf4	958.9928207	0.202226415	0.264437	0.764744	0.444424	0.550155
12326	KIAA0247	2636.392786	0.202161791	0.108897	1.856456	0.063389	0.100802

14984	P2RY8	496.3609003	0.201972747	0.199896	1.010388	0.31231	0.408514
12780	PWWP2A	655.3287297	0.201930773	0.117988	1.711459	0.086996	0.133429
15124	PPP1R3C	2026.610913	0.20188343	0.208888	0.966466	0.333811	0.43261
14011	NXT2	358.0647508	0.201793415	0.153454	1.315011	0.188506	0.263715
15093	TAS2R14	8.433584967	0.2017689	0.206334	0.977876	0.328135	0.426143
14095	TMEM19	1311.312995	0.201728814	0.156439	1.289506	0.197222	0.274264
10006	RAB7A	6023.449872	0.201640851	0.076068	2.650802	0.00803	0.01573
11455	ARCN1	4360.944995	0.201126536	0.094285	2.133187	0.032909	0.056312
13601	KLC4	935.8031903	0.200992421	0.138091	1.455504	0.14553	0.209729
12020	MDC1	678.2243736	0.200949032	0.102763	1.955455	0.050529	0.082398
14437	ACPL2	480.468918	0.200848167	0.169158	1.187342	0.235093	0.319183
14067	RFESD	31.42542663	0.200713707	0.154706	1.297389	0.194497	0.271013
11881	PCMT1	1106.1878	0.200627576	0.100525	1.995789	0.045957	0.075819
12387	WDFY1	1579.726398	0.200476587	0.108942	1.84021	0.065737	0.104022
12966	DGCR11	46.12605586	0.200127886	0.121061	1.653119	0.098307	0.148612
15328	ECM2	592.2571303	0.200096849	0.219525	0.911501	0.362031	0.462955
11414	VAMP3	2640.484665	0.200091818	0.093341	2.143659	0.03206	0.055056
14470	GSN	10234.99961	0.199885209	0.169943	1.176189	0.239519	0.324452
12348	FAM160A2	707.3662017	0.19983046	0.108044	1.849537	0.06438	0.102196
16930	NMBR	0.749955461	0.199815639	0.438902	0.455262	0.64892	0.751299
14636	PRPF40B	319.4516322	0.199794434	0.178125	1.121651	0.262011	0.350893
13561	ZFHX3	970.9990746	0.199782428	0.136207	1.466752	0.142443	0.205887
14098	LMO2	646.5226211	0.199418082	0.154702	1.289045	0.197383	0.274429
14220	LOC72960[illegible]	42.42292361	0.199276646	0.159234	1.251467	0.210764	0.290527
12243	ERC1	1353.056418	0.199244659	0.105747	1.884161	0.059543	0.095328
11253	DIP2A	750.481517	0.199054207	0.090507	2.19932	0.027855	0.048519
14684	KIAA1524	121.1715564	0.198783806	0.179698	1.106208	0.268637	0.358591
13620	C5orf28	295.4785628	0.198767159	0.137215	1.448579	0.147455	0.212208
11560	NOP14	676.167932	0.198755107	0.094933	2.093641	0.036292	0.061536
14582	MTMR8	12.02805308	0.198644512	0.173925	1.142126	0.253402	0.34062
14326	FAM35B	312.1907861	0.19842882	0.162249	1.222992	0.221333	0.30283
14700	EPHX1	2330.288521	0.198328761	0.18052	1.098655	0.271919	0.362577
14962	TNFRSF14	2782.486807	0.198294623	0.194682	1.018556	0.308414	0.404038
13267	BAP1	1301.203196	0.198171541	0.127246	1.557388	0.119378	0.176373
9583	MRPS14	313.227578	0.198140374	0.071105	2.786583	0.005327	0.010895
15880	BZRAP1	269.7392808	0.19808939	0.264523	0.748854	0.453945	0.560314
17401	S100A5	2.49506369	0.197952961	0.579898	0.341358	0.732834	0.825486
16847	PDZD9	0.734648742	0.197871316	0.413141	0.478944	0.631978	0.735289
11284	ZNF197	301.9206707	0.197786672	0.090551	2.184249	0.028944	0.050277
10857	SUN1	2571.988599	0.197700056	0.084567	2.337781	0.019399	0.035022
12149	NFU1	719.7211838	0.197607304	0.103371	1.911623	0.055925	0.090228
12472	ARF6	2785.696975	0.196976116	0.108762	1.811067	0.07013	0.110217
10988	CDC42	5549.947579	0.196694303	0.085861	2.290834	0.021973	0.039197
17639	USP9Y	348.6768203	0.19655277	0.696124	0.282353	0.777673	0.864174
13760	MDM2	2767.756084	0.196428494	0.140187	1.401188	0.161158	0.229568
10649	BICD2	983.0890599	0.196393899	0.081351	2.414166	0.015771	0.029029
13266	CCDC109A	672.1373212	0.196310046	0.126034	1.5576	0.119328	0.176311

17336	LMX1B	7.170031436	0.19630317	0.546958	0.3589	0.71967	0.813697
13877	C14orf138	111.0891599	0.196186776	0.14453	1.357415	0.174649	0.246689
13800	CRNA0018	41.76738765	0.196120713	0.141425	1.386746	0.165519	0.235063
13657	LOC642852	285.9460674	0.195936619	0.136364	1.436869	0.150755	0.216369
14167	TCFL5	244.3861448	0.195707441	0.154548	1.266319	0.205399	0.284183
11244	CTNNA1	10438.36878	0.195699284	0.08888	2.201839	0.027677	0.048247
11580	KLHL18	314.0461438	0.195508479	0.093593	2.088915	0.036715	0.062141
13887	PMPCA	1020.527937	0.195348838	0.144455	1.352316	0.176274	0.248805
14343	SLC38A7	282.45254	0.195258464	0.16051	1.216488	0.223799	0.305842
13820	USP54	423.3713254	0.194856483	0.141259	1.379427	0.167763	0.23794
13584	LOC729991	284.0918606	0.194700178	0.133387	1.459664	0.144382	0.208336
13972	GCFC1	573.5765692	0.19448818	0.146449	1.32803	0.184168	0.258365
15016	LOC284232	10.85350973	0.194468969	0.19357	1.004644	0.315068	0.411272
11969	RAB1A	4300.432128	0.194414978	0.09875	1.968769	0.04898	0.080211
10946	H2AFV	3603.360144	0.194339041	0.084313	2.304965	0.021169	0.037907
11676	POLR3F	278.9412475	0.194209548	0.094421	2.056856	0.0397	0.066646
14485	SCPEP1	1669.681526	0.194205485	0.165579	1.172888	0.240841	0.325904
14410	ULK3	897.3401767	0.19414488	0.1625	1.194736	0.23219	0.315834
12991	TMEM185B	546.7956858	0.194120221	0.117833	1.647422	0.099471	0.150084
11003	PIGG	702.5739752	0.194117088	0.084831	2.288276	0.022121	0.039408
15621	OC1001297	13.20967416	0.193992556	0.233884	0.82944	0.406856	0.510484
12355	WHSC1L1	877.5405156	0.193961544	0.104936	1.848383	0.064547	0.102403
14512	MKNK2	2738.527737	0.193869815	0.16675	1.162634	0.244978	0.330886
15106	NAGLU	1243.20423	0.193849631	0.198968	0.974275	0.32992	0.428093
13250	RBMS2	1605.070732	0.193689612	0.123932	1.562873	0.118082	0.174682
12950	TGOLN2	10584.8208	0.19339685	0.116663	1.657742	0.09737	0.147378
13214	PIGN	455.8745499	0.193273421	0.122692	1.575278	0.115192	0.170871
13209	RFC3	173.7734146	0.193268427	0.122545	1.577128	0.114766	0.170303
13100	ZNF22	929.2801502	0.193180301	0.119913	1.611	0.10718	0.160369
16580	WNT9B	5.14814305	0.192923586	0.349476	0.552037	0.580923	0.686772
14531	C5orf35	121.6054752	0.19276788	0.166718	1.156252	0.247578	0.333959
12529	ETF1	2783.331887	0.192586716	0.107241	1.795831	0.072521	0.113456
14890	HHEX	193.343207	0.192496422	0.185207	1.039357	0.298639	0.393124
14322	SEC11C	1180.232949	0.192449601	0.15728	1.223608	0.2211	0.302614
14930	ERG	820.5433342	0.192420971	0.187587	1.025771	0.305	0.400422
12775	NAA40	402.7185698	0.192107424	0.112215	1.711956	0.086905	0.13334
14212	ARHGEF10	1056.813644	0.192098619	0.153242	1.253566	0.21	0.289626
11083	COQ5	752.9847847	0.192084968	0.084952	2.261105	0.023753	0.042008
13862	ZNF25	431.4760972	0.191871741	0.140622	1.364447	0.172427	0.243813
12515	RNMTL1	314.3532891	0.19183263	0.106622	1.799186	0.071989	0.11275
12742	SLC25A32	536.6910741	0.191740169	0.111488	1.719827	0.085464	0.131469
15525	PCDHB14	247.3854555	0.191641871	0.224914	0.852067	0.394177	0.497666
15298	PDE5A	683.3192872	0.191483279	0.207855	0.921236	0.356927	0.457323
11233	UBAP1	1380.375242	0.191374731	0.086735	2.206429	0.027354	0.047731
14307	NBR2	132.0934192	0.19134604	0.15593	1.227126	0.219775	0.301099
12514	PPCS	1186.685532	0.191049881	0.106181	1.799281	0.071974	0.112735
15761	ST7OT3	2.251657607	0.190967759	0.242694	0.786867	0.43136	0.536456

15845	PDE6B	241.2065412	0.190863845	0.251042	0.760287	0.447083	0.553062
16366	ZDHHC11	57.54033847	0.190701201	0.313075	0.609123	0.542443	0.649665
11421	PHF8	725.705742	0.190682151	0.089031	2.141748	0.032214	0.055286
15384	TRIM78P	14.7065785	0.190540787	0.21312	0.894054	0.371293	0.47307
11498	DFFA	394.5276844	0.190444081	0.089882	2.118825	0.034105	0.05814
10982	ATG3	872.0602554	0.190202545	0.082964	2.292585	0.021872	0.039038
12815	EXOSC6	295.9239297	0.190167899	0.111819	1.700673	0.089004	0.136135
13066	PPIL2	1083.810781	0.190060059	0.117006	1.624364	0.104298	0.156463
12442	WDR5	783.1952279	0.190036187	0.10419	1.823933	0.068162	0.107382
12977	KLHDC5	678.7551579	0.189682697	0.114928	1.65045	0.098851	0.149286
14301	NEK8	111.1880806	0.189546291	0.154182	1.229369	0.218934	0.300071
14604	HIST2H4A	216.0548489	0.189513508	0.167382	1.132219	0.257542	0.345665
15904	LQK1	37.81383952	0.189509423	0.255618	0.741377	0.458465	0.565038
13518	IQCB1	420.8487217	0.189459729	0.127874	1.48161	0.138444	0.200728
13807	POLR1A	536.5267605	0.189351106	0.136674	1.385422	0.165923	0.235552
16734	COL24A1	28.25511227	0.189200771	0.371166	0.509748	0.610228	0.714728
12069	RAB5C	2866.131147	0.189042913	0.097514	1.938628	0.052547	0.08534
11254	HNRNPA3	4219.161854	0.188896604	0.085911	2.198741	0.027896	0.048587
16714	CXCL11	205.1962989	0.188856846	0.365969	0.516046	0.605822	0.710456
16232	OC1002722	51.18615678	0.188797277	0.292498	0.645466	0.518625	0.626267
13180	ARHGAP19	278.1132288	0.188597242	0.118892	1.586289	0.112674	0.167566
13683	DLEU1	149.3509518	0.188385748	0.132131	1.425752	0.15394	0.22052
12503	ZNF639	614.5178005	0.188317037	0.104447	1.802985	0.071391	0.111919
12261	PALB2	261.2494313	0.188307758	0.100212	1.8791	0.060231	0.096288
16008	HMCN1	682.3011647	0.188280541	0.265486	0.709191	0.478206	0.585539
12233	BBS9	334.6710388	0.188243742	0.099757	1.88702	0.059158	0.094789
12489	GTF2A2	623.1547823	0.188207914	0.104174	1.806677	0.070813	0.111138
12824	ZZEF1	1147.429119	0.188092679	0.110806	1.6975	0.089602	0.136953
13506	FAM165B	291.2966395	0.188066911	0.126545	1.486166	0.137235	0.199167
11715	MIS12	427.8512103	0.188024192	0.091791	2.048389	0.040522	0.067799
15937	VWF	20293.81287	0.187847111	0.256927	0.73113	0.4647	0.571537
14083	ATP5L	3221.641564	0.187846887	0.145313	1.292709	0.196112	0.272952
13855	UBE2H	1456.09256	0.187834063	0.137452	1.366542	0.171769	0.243006
12826	USP27X	137.3957327	0.187719471	0.110624	1.69692	0.089712	0.1371
10609	LUC7L2	1780.063129	0.187386516	0.077	2.433598	0.01495	0.027621
14546	LRIG1	997.9653673	0.18736398	0.162635	1.15205	0.2493	0.335937
15735	SLC16A8	6.001180897	0.187363115	0.235945	0.794095	0.42714	0.532086
13708	INO80C	328.8945801	0.187177266	0.132229	1.415558	0.156905	0.224358
14026	AIMP2	402.3702031	0.18715204	0.142655	1.311924	0.189546	0.264886
14657	NUDT16	1168.292042	0.187053887	0.167817	1.114631	0.265009	0.354412
13033	NDUFC2	1537.826271	0.186966597	0.114426	1.633946	0.10227	0.153809
15713	ETV3L	2.35841669	0.186945858	0.233619	0.800216	0.423586	0.528363
13041	RHBDD1	378.5434717	0.186892549	0.114484	1.632471	0.10258	0.15419
15606	RPL21P44	3.720481871	0.186649446	0.223863	0.833765	0.404413	0.507927
13023	LIMD1	846.6950075	0.186606455	0.114025	1.636538	0.101727	0.15311
14449	NIN	1163.921679	0.186575154	0.15774	1.182798	0.236889	0.321355
13636	KFZp761E1	437.0872343	0.186500564	0.129272	1.442696	0.149106	0.214318

16390	KCNJ12	57.88284051	0.186363008	0.309045	0.603029	0.54649	0.65356
15694	GSTM3	556.3020913	0.186246545	0.230395	0.808378	0.418873	0.523151
14533	LETMD1	1472.508657	0.186062582	0.160978	1.155825	0.247753	0.33415
16520	ALDH1A3	743.8925671	0.186061269	0.328744	0.565976	0.57141	0.677955
15023	CDKN2B	277.5766345	0.185996821	0.186077	0.99957	0.317518	0.414277
11087	KIAA1267	996.105592	0.185984239	0.082275	2.260515	0.023789	0.042058
13285	GTF2IRD2	393.417169	0.18591222	0.120024	1.548959	0.121391	0.179104
9598	ZNF2	116.6545318	0.185811221	0.066834	2.780198	0.005433	0.011094
14446	SLC9A3R1	4462.737188	0.185724117	0.156886	1.183814	0.236487	0.320877
12404	SNRNP200	5102.090931	0.18547956	0.101058	1.835383	0.066449	0.105004
12860	CIAPIN1	954.2091066	0.185468	0.109786	1.689354	0.091152	0.138924
13606	SPDYE3	72.2885808	0.18534175	0.127432	1.45444	0.145824	0.210077
14100	UCKL1	553.8987967	0.185313175	0.143814	1.288558	0.197552	0.274625
15957	ABCC6P2	106.5777406	0.185238975	0.255401	0.725286	0.468277	0.575214
11329	ZFP161	384.0887773	0.185164396	0.085299	2.17078	0.029948	0.051815
15822	TC2N	516.7602721	0.185005534	0.240737	0.768496	0.442193	0.547808
14058	RALA	1389.190628	0.184963422	0.142312	1.299701	0.193704	0.27008
13006	LOC643387	30.89418904	0.184412027	0.112302	1.642108	0.100568	0.151563
16881	PCDHGB1	91.79287807	0.18434797	0.392352	0.469853	0.63846	0.741334
14158	PLEKHM1	683.8826273	0.18411407	0.145176	1.26821	0.204723	0.283428
13332	TGS1	443.6075329	0.184043837	0.120039	1.533206	0.125225	0.184109
14096	BLCAP	1574.624721	0.183958575	0.142667	1.289426	0.19725	0.274283
12497	RAF1	1694.530323	0.183872081	0.101852	1.805293	0.071029	0.111406
13237	SAPS2	1042.502312	0.183859416	0.117282	1.567669	0.116958	0.173194
15437	ZNF843	9.129509854	0.183855552	0.209656	0.87694	0.380519	0.483161
12088	TSN	1796.837768	0.18378806	0.095136	1.931853	0.053378	0.086553
13494	SLC35A1	573.5189985	0.183711116	0.123316	1.489761	0.136287	0.197967
10981	RYK	1037.844958	0.183661812	0.080089	2.293227	0.021835	0.038975
12634	NUDT19	421.1698347	0.183462537	0.104009	1.76391	0.077747	0.120622
12474	HMGB1	4776.501264	0.183368388	0.101288	1.810371	0.070238	0.110369
16752	SPESP1	55.44831409	0.183325145	0.363589	0.50421	0.614114	0.718529
11521	PCBP2	13373.09602	0.183240271	0.086829	2.11035	0.034828	0.059254
14602	AP1S2	701.6633461	0.183175708	0.161632	1.133291	0.257092	0.345108
10793	GPATCH1	249.9125977	0.183086627	0.077446	2.364067	0.018076	0.032827
12200	LOC387647	494.780941	0.182770796	0.0964	1.895954	0.057966	0.093131
11423	TFCP2	505.9469415	0.182549962	0.085279	2.14062	0.032305	0.055432
13879	C14orf79	225.9379324	0.182412417	0.134577	1.355453	0.175273	0.247535
16146	HIST1H2BJ	14.75875765	0.182304391	0.273442	0.666702	0.504962	0.613017
14868	C9orf23	369.1387572	0.182138944	0.174111	1.046111	0.29551	0.389581
16627	C14orf132	327.9733255	0.182067609	0.33605	0.541787	0.587965	0.6931
14113	NIPAL3	1025.324285	0.181977485	0.141857	1.282823	0.199554	0.277153
11543	HNRNPR	2116.661336	0.181659242	0.086497	2.100182	0.035713	0.060643
10464	SFPQ	3599.007895	0.18148132	0.072937	2.488186	0.01284	0.024051
14651	ARSB	1298.593853	0.181221523	0.162328	1.116393	0.264254	0.35353
16126	STON2	529.8205925	0.1809205	0.268504	0.673809	0.500433	0.608272
10074	QRICH1	962.9228364	0.18089463	0.068856	2.62713	0.008611	0.016754
15324	OC1001339	6.400006127	0.180874672	0.198009	0.913466	0.360998	0.461754

11980	RIOK1	327.7608317	0.180857552	0.092008	1.965682	0.049335	0.08072
10436	DIDO1	1621.391839	0.180687278	0.072315	2.49862	0.012468	0.023417
10532	SEC31A	5351.219547	0.180652126	0.073442	2.45979	0.013902	0.025873
14438	MALT1	1138.307682	0.180644854	0.152158	1.18722	0.235141	0.319227
12314	NSUN3	109.5475467	0.180613805	0.097072	1.860621	0.062798	0.099959
12985	BAG3	1398.233236	0.180434806	0.109418	1.649036	0.09914	0.149653
11036	DCAF12	991.9512717	0.180409787	0.079184	2.278368	0.022705	0.040326
14420	DNAJB5	353.8977861	0.180375723	0.151327	1.191958	0.233278	0.317093
11842	UNG	903.4516704	0.180297006	0.089774	2.008335	0.044608	0.073835
17501	PROKR1	0.445739894	0.180266691	0.572826	0.314697	0.752991	0.843345
16260	IL1RL2	57.0926056	0.180251979	0.282541	0.637967	0.523495	0.63106
17674	SPACA5	0.289385987	0.18017087	0.656091	0.274613	0.783614	0.869051
17781	TAS2R30	0.23647207	0.18017073	0.723492	0.249029	0.803338	0.885565
17986	)C1001317	0.354177478	0.180170605	0.868725	0.207397	0.8357	0.910743
18093	MYT1L	0.369966744	0.180170435	0.968396	0.18605	0.852405	0.923451
18083	MYH7	0.235774733	0.180170306	0.952433	0.189168	0.849961	0.921261
18071	UOX	0.198819222	0.180170289	0.936368	0.192414	0.847418	0.919166
18273	'ABPC1L2/	0.138145599	0.180169452	1.21908	0.147791	0.882507	0.946592
18344	DVWA	0.179774353	0.180169234	1.319412	0.136553	0.891384	0.952033
18350	MYCNOS	0.137936927	0.180169143	1.316644	0.13684	0.891157	0.952033
18424	FGF21	0.273432028	0.180169119	1.445809	0.124615	0.900829	0.958357
18355	SNORA59E	0.121625071	0.180169075	1.322047	0.13628	0.8916	0.952033
18359	C20orf144	0.119322395	0.180169067	1.324399	0.136038	0.891791	0.952074
18408	CABP5	0.157578712	0.180168829	1.425406	0.126398	0.899417	0.957585
18420	RFX6	0.119991713	0.180168667	1.43754	0.125331	0.900261	0.957988
18423	)C1002718	0.116054457	0.18016865	1.442255	0.124922	0.900586	0.958196
18474	OR5C1	0.155923346	0.18016838	1.549168	0.1163	0.907415	0.962771
18490	C16orf82	0.098171676	0.180167993	1.592819	0.113113	0.909941	0.964616
18493	TAAR8	0.096523727	0.180167981	1.595675	0.11291	0.910102	0.96463
18542	TFDP3	0.098479517	0.180167294	1.752546	0.102803	0.918119	0.970735
18539	C17orf54	0.09669424	0.180167278	1.756176	0.102591	0.918288	0.970735
18615	BPY2	0.138832345	0.180166842	1.890783	0.095287	0.924087	0.973018
18619	DKK4	0.122226875	0.180166669	1.905364	0.094558	0.924666	0.973435
18623	KRT35	0.102541188	0.180166419	1.934262	0.093145	0.925789	0.974407
18633	OR7E5P	0.078580794	0.180166022	1.989381	0.090564	0.927839	0.976027
18637	PNLIPRP1	0.077225764	0.180165999	1.993721	0.090367	0.927996	0.976045
18635	ACTRT1	0.076698919	0.18016599	1.995433	0.090289	0.928057	0.976045
18704	OR51F2	0.078178336	0.180164632	2.235752	0.080583	0.935773	0.980651
18705	FKSG83	0.076567916	0.180164593	2.242141	0.080354	0.935956	0.980779
18778	tAKR	0.102520311	0.180163434	2.454698	0.073395	0.941492	0.982755
18801	C1orf185	0.061426867	0.180161876	2.629132	0.068525	0.945368	0.985142
18800	C16orf90	0.061290425	0.180161869	2.630049	0.068501	0.945387	0.985142
18811	C17orf77	0.060715406	0.180161841	2.633938	0.0684	0.945467	0.985171
18814	PRAMEF16	0.056768509	0.180161639	2.661975	0.06768	0.946041	0.985614
18923	PTPN20A	0.171380058	0.180161191	2.91407	0.061825	0.950703	0.986718
18876	)C1001285	0.079041261	0.180159966	2.91407	0.061824	0.950703	0.986718
18889	MBD3L1	0.061565997	0.180159721	2.91407	0.061824	0.950703	0.986718

18890	MC5R	0.058089788	0.180159721	2.91407	0.061824	0.950703	0.986718
18843	CTRB1	0.059156586	0.180159721	2.91407	0.061824	0.950703	0.986718
18841	CRISP1	0.040020147	0.180159476	2.91407	0.061824	0.950703	0.986718
18846	CXorf66	0.039936512	0.180159476	2.91407	0.061824	0.950703	0.986718
18856	FBP2	0.040130368	0.180159476	2.91407	0.061824	0.950703	0.986718
18874	KRTAP26-1	0.041183851	0.180159476	2.91407	0.061824	0.950703	0.986718
18898	OR11G2	0.03812668	0.180159476	2.91407	0.061824	0.950703	0.986718
18910	OR8B3	0.041344487	0.180159476	2.91407	0.061824	0.950703	0.986718
18912	OTOR	0.043403309	0.180159476	2.91407	0.061824	0.950703	0.986718
18915	PATE3	0.040061612	0.180159476	2.91407	0.061824	0.950703	0.986718
18956	UNCX	0.03940046	0.180159476	2.91407	0.061824	0.950703	0.986718
18833	C13orf28	0.021513198	0.18015923	2.91407	0.061824	0.950703	0.986718
18849	DEFB121	0.021626811	0.18015923	2.91407	0.061824	0.950703	0.986718
18851	EDDM3B	0.020928677	0.18015923	2.91407	0.061824	0.950703	0.986718
18865	IL9	0.02193968	0.18015923	2.91407	0.061824	0.950703	0.986718
18873	KRTAP10-2	0.020892739	0.18015923	2.91407	0.061824	0.950703	0.986718
18899	OR13C4	0.021066752	0.18015923	2.91407	0.061824	0.950703	0.986718
18902	OR4D10	0.021135509	0.18015923	2.91407	0.061824	0.950703	0.986718
18906	OR5K4	0.021626811	0.18015923	2.91407	0.061824	0.950703	0.986718
18919	PRAMEF9	0.020997434	0.18015923	2.91407	0.061824	0.950703	0.986718
18920	PRB1	0.021626811	0.18015923	2.91407	0.061824	0.950703	0.986718
18939	SNORA2B	0.021738035	0.18015923	2.91407	0.061824	0.950703	0.986718
18948	TBC1D21	0.021764886	0.18015923	2.91407	0.061824	0.950703	0.986718
18953	TRIM60	0.023367094	0.18015923	2.91407	0.061824	0.950703	0.986718
18962	ZP4	0.021202422	0.18015923	2.91407	0.061824	0.950703	0.986718
19613	BHLHE23	0.01877631	0.18015923	2.91407	0.061824	0.950703	NA
19684	DAZ3	0.019037914	0.18015923	2.91407	0.061824	0.950703	NA
19716	ESX1	0.020258979	0.18015923	2.91407	0.061824	0.950703	NA
19854	LCE1D	0.020534354	0.18015923	2.91407	0.061824	0.950703	NA
20202	SNORA4	0.018957247	0.18015923	2.91407	0.061824	0.950703	NA
12823	SCARB2	7597.948842	0.180153197	0.106103	1.697903	0.089526	0.136848
14538	CYB5R4	358.0733555	0.180142143	0.156129	1.153801	0.248582	0.335153
15102	TLR4	966.2982181	0.180133849	0.184682	0.975372	0.329376	0.427499
12024	ERP44	1812.064204	0.180085218	0.092121	1.95488	0.050597	0.082481
14359	HAGH	905.5641572	0.18000391	0.148689	1.210609	0.226045	0.308549
13442	BTBD6	906.5569053	0.179952521	0.119765	1.502547	0.132956	0.193875
13689	C9orf21	246.0236277	0.179844813	0.126418	1.422618	0.154847	0.221722
13120	SRRM2	10233.62904	0.179483295	0.111873	1.604355	0.108636	0.1623
12667	PPIL1	432.150211	0.17929651	0.102404	1.750866	0.079969	0.123745
17170	HCN4	10.80054099	0.179268216	0.455203	0.39382	0.693714	0.791933
14150	CCNJ	164.1198278	0.1791551	0.14091	1.271414	0.203581	0.282007
14406	TMEM67	241.3396798	0.17911533	0.149699	1.196499	0.231502	0.314984
16904	GSTO2	120.6911105	0.179058876	0.385812	0.46411	0.642569	0.74509
12918	LOC653566	1815.256156	0.178996929	0.107297	1.668233	0.095269	0.144556
16282	KCNMA1	1374.824983	0.178955365	0.282779	0.632844	0.526835	0.634201
17581	TEX15	112.2730405	0.178644488	0.601044	0.297224	0.766296	0.854381
12583	TAOK2	1594.319815	0.178529654	0.100404	1.77811	0.075386	0.117431

8899	HMG20A	910.5558934	0.178510024	0.058482	3.052419	0.00227	0.005
11963	RAD1	464.4464667	0.178345344	0.09052	1.97023	0.048812	0.079977
15350	ZBTB42	298.2173787	0.177983416	0.19661	0.905261	0.365327	0.4665
12371	SIAH2	752.6940636	0.177920573	0.096521	1.843342	0.065279	0.10343
13070	UCK1	807.4934669	0.177816424	0.109583	1.622664	0.104661	0.156969
13945	CEP120	709.7559529	0.17767401	0.133013	1.335764	0.181626	0.255293
15847	C6orf163	8.506333216	0.177462208	0.233467	0.760118	0.447184	0.553118
13487	ACVR1	1067.434136	0.17742967	0.118986	1.49118	0.135914	0.197513
12881	PPIL5	103.0421914	0.177408204	0.105587	1.680207	0.092917	0.141392
14718	HCG26	124.4073752	0.177364653	0.162342	1.09254	0.274596	0.365699
16315	SLC14A1	136.6944753	0.17736205	0.28408	0.624338	0.532406	0.639638
16924	COL9A3	14.17791342	0.177305316	0.386169	0.459139	0.646134	0.748338
14834	TUBB2C	4278.40658	0.177277477	0.167815	1.056389	0.290791	0.384238
15486	GNA14	122.760234	0.177183782	0.204688	0.865627	0.386695	0.489448
11845	NUP50	1215.63785	0.176930917	0.088133	2.007549	0.044691	0.073955
15583	GPR3	70.69043713	0.176747871	0.210422	0.839968	0.400926	0.504303
13169	CYB561D1	383.0876646	0.176603321	0.111108	1.58947	0.111954	0.166635
16784	MIP	1.293905526	0.176210038	0.356683	0.494024	0.621289	0.725566
13268	PANK4	340.6126172	0.17618031	0.113131	1.55731	0.119397	0.176387
13233	WDR3	654.8998133	0.176179488	0.112263	1.569352	0.116566	0.17266
12895	ALG14	220.7207467	0.176143251	0.105234	1.67383	0.094164	0.143134
14552	SLC35F2	447.4265768	0.176102143	0.153085	1.150356	0.249997	0.336737
13812	ZNF738	137.4518467	0.176044867	0.127311	1.382795	0.166728	0.236625
15134	APOL6	2771.776427	0.175887276	0.182515	0.963689	0.335202	0.434084
12739	ZNF324	138.8103342	0.175692284	0.102106	1.720677	0.085309	0.131262
14155	TESK2	71.06562122	0.175640755	0.138309	1.269912	0.204116	0.282648
12487	QTRTD1	444.3508546	0.175542265	0.097131	1.80727	0.07072	0.11101
16444	ELAVL4	1.692609874	0.175508011	0.298358	0.588247	0.556367	0.663181
15432	L3MBTL	114.3741671	0.175476421	0.199594	0.879166	0.379311	0.481783
13055	IP6K1	1114.037421	0.175393613	0.107729	1.628108	0.103502	0.1554
13221	LOC72864(	35.03533405	0.17502234	0.111237	1.573421	0.115621	0.171416
11021	ZNF200	202.902675	0.174859995	0.076583	2.283288	0.022413	0.039863
18251	ARHGDIG	0.674247772	0.174848099	1.145918	0.152583	0.878727	0.943673
13684	CBL	1019.449359	0.174828248	0.122654	1.425376	0.154049	0.22066
15032	CD83	371.3781002	0.174812083	0.175491	0.996133	0.319186	0.416147
15147	RILP	264.8199554	0.174590832	0.181694	0.960906	0.336599	0.435577
11556	PRKRA	867.2482675	0.17451489	0.083273	2.095693	0.036109	0.061248
15423	TMCC2	82.36371144	0.174354936	0.197465	0.882968	0.377254	0.479449
12763	PAPD4	1114.127921	0.174190552	0.101568	1.715016	0.086342	0.132602
14519	ABCG1	1414.306548	0.173989533	0.149875	1.160895	0.245685	0.33168
16147	ABCA10	17.98778325	0.173969347	0.261084	0.666336	0.505197	0.613244
14658	STIM1	1869.654541	0.173937337	0.156122	1.114109	0.265232	0.354674
13510	SUPT3H	132.3656432	0.173852919	0.117087	1.484824	0.13759	0.199625
14927	ATP8B5P	27.44441413	0.173779013	0.169262	1.026689	0.304567	0.399934
15458	C6orf124	24.52317439	0.173740591	0.199015	0.873001	0.382663	0.485222
16322	ACOT6	9.531111783	0.173621387	0.279217	0.621816	0.534063	0.641353
14156	CCDC45	483.624048	0.173517697	0.136718	1.269168	0.204381	0.282995

14897	TINAGL1	2502.337339	0.173412022	0.167152	1.037454	0.299524	0.394123
12405	44076	8910.730522	0.173399165	0.094516	1.834597	0.066565	0.105179
12461	UPF3A	530.34163	0.173171814	0.095403	1.815157	0.0695	0.109322
14493	LOC645332	100.1231795	0.173119606	0.147965	1.170006	0.241999	0.32729
14137	PTPN23	1052.978524	0.17270723	0.135311	1.276374	0.201823	0.279829
15637	HIST1H2BN	10.43643593	0.172684434	0.209351	0.824854	0.409454	0.513252
15896	PTER	1194.38686	0.172481907	0.231905	0.743761	0.457021	0.563542
12485	KIAA1967	1539.836781	0.172378962	0.095345	1.807946	0.070615	0.110863
14598	LBR	1111.663382	0.172234185	0.151583	1.136236	0.255858	0.343545
15487	DCI	1444.796006	0.172220816	0.199	0.865433	0.386801	0.489552
12769	ZBTB39	175.5520639	0.172132334	0.100455	1.713529	0.086615	0.132958
17682	ASCL1	3.019563794	0.171743161	0.629844	0.272676	0.785103	0.870308
16654	GOLT1A	193.2315706	0.171690575	0.322006	0.533191	0.593902	0.698995
13146	PPT2	475.1967113	0.17161799	0.107535	1.595921	0.110506	0.164768
16779	CDH8	20.21211243	0.171560128	0.345763	0.496179	0.619768	0.724005
11993	COG7	604.1022844	0.171471923	0.087375	1.962481	0.049706	0.081232
15424	KLHL25	147.8540403	0.171306251	0.194117	0.882489	0.377513	0.479747
12356	POGK	1268.642904	0.17124593	0.092655	1.848211	0.064572	0.102434
13097	MAP1LC3B	2769.351929	0.171218629	0.106188	1.61241	0.106873	0.159946
16238	RAMP3	1182.356829	0.170936018	0.265086	0.644832	0.519036	0.626532
10334	WDR45L	2040.549793	0.170855794	0.067381	2.535675	0.011223	0.021287
12104	UBE2A	1353.128754	0.170825677	0.088597	1.928116	0.053841	0.087189
14515	GGT7	414.5162441	0.170549436	0.146856	1.161335	0.245506	0.331484
12457	OPTN	3470.774689	0.170488041	0.093787	1.817824	0.069091	0.108718
12245	COPS8	1446.044973	0.170484026	0.090501	1.883772	0.059596	0.095397
13171	PSMD12	816.6955315	0.170060129	0.107026	1.588968	0.112068	0.166788
13788	EIF2A	2531.763578	0.169891662	0.122288	1.38928	0.164748	0.234188
12259	ANKRA2	264.895212	0.169857784	0.090346	1.880079	0.060097	0.09609
15685	BAIAP2	793.6167977	0.169771821	0.209251	0.811332	0.417175	0.521329
14574	TMEM20	88.56205867	0.169584855	0.148144	1.14473	0.252321	0.339354
13721	INPP1	551.222812	0.169253281	0.119976	1.410729	0.158325	0.226173
15786	RNF148	2.897304621	0.169235067	0.217001	0.779881	0.435461	0.540726
16299	OC1001339	14.69587965	0.16903644	0.2694	0.627455	0.530361	0.637806
15500	CD34	3892.57085	0.169027899	0.196338	0.860903	0.389292	0.492291
12771	ZNF652	882.625885	0.168991584	0.098648	1.713073	0.086699	0.133066
16820	HAPLN2	5.518179091	0.168916102	0.348063	0.485304	0.627461	0.731161
14606	WIPI2	1895.950371	0.16884171	0.14917	1.131878	0.257686	0.345786
12509	KIAA0141	1426.737585	0.168735794	0.093703	1.800749	0.071742	0.112417
15264	ZNF710	246.1135133	0.168630805	0.181327	0.92998	0.352382	0.452505
10126	SNW1	1178.097366	0.168448998	0.064616	2.606934	0.009136	0.017684
14327	TSPAN6	1127.302537	0.168302598	0.137651	1.222672	0.221453	0.302974
15043	LOC338758	35.49719493	0.168261282	0.16934	0.99363	0.320403	0.417457
12459	POGZ	1742.535441	0.168212376	0.092634	1.815872	0.06939	0.109167
13538	G3BP1	2273.764694	0.168199115	0.114121	1.47387	0.140517	0.203439
9405	WDR33	1228.658461	0.168137423	0.058867	2.856217	0.004287	0.008935
14839	MORN2	352.0232789	0.168034152	0.159294	1.054867	0.291486	0.385027
12500	BRD8	929.2668954	0.168028775	0.093121	1.804419	0.071166	0.111594

13950	WDR19	446.8031438	0.167970446	0.125914	1.334006	0.182202	0.25601
11538	ZCCHC3	582.4792498	0.167844047	0.079785	2.103709	0.035404	0.060145
14353	UBE2V2	744.4101804	0.16783384	0.138381	1.212835	0.225193	0.307562
12976	COMMD3	664.6334565	0.167729499	0.101625	1.650476	0.098846	0.149286
14187	TRAP1	2227.244443	0.167709278	0.132972	1.261234	0.207224	0.286319
17693	GRM4	3.29657219	0.167564389	0.623113	0.268915	0.787995	0.872954
14595	CRNA0012	32.7409342	0.167258117	0.147113	1.136936	0.255565	0.343222
13651	PSEN2	469.0914637	0.167251475	0.116329	1.437749	0.150505	0.21611
11756	GTF2B	474.3142246	0.167107895	0.082089	2.035704	0.04178	0.069661
10333	CSTF1	638.4679781	0.166962777	0.065843	2.535762	0.01122	0.021284
12900	MPHOSPH1	678.4456959	0.166844422	0.099711	1.673273	0.094273	0.143245
12711	UPF1	2033.706613	0.166823318	0.096507	1.728614	0.083878	0.129334
13008	C10orf119	1336.685596	0.166809012	0.101657	1.6409	0.100818	0.151912
14779	ZBTB46	257.2715392	0.166741903	0.155142	1.074767	0.282479	0.374661
13002	MAPK9	1204.887295	0.166558201	0.101334	1.643662	0.100246	0.151124
15181	MR1	362.3221122	0.166394436	0.174626	0.952861	0.340661	0.439794
14553	MBTPS2	840.1258113	0.166286216	0.14456	1.150291	0.250024	0.33675
13469	ZC3HAV1	1161.219955	0.166239395	0.111048	1.497006	0.134392	0.195576
13106	CUL4B	2079.670833	0.166235129	0.103357	1.608352	0.107758	0.161148
14139	WIPI1	683.2280147	0.166216771	0.130307	1.275582	0.202103	0.280177
16614	S100A14	53.88527809	0.166176366	0.304909	0.545004	0.585751	0.691062
14842	EID3	47.62883721	0.166158499	0.157679	1.053776	0.291986	0.385609
14603	ARHGEF17	2638.014833	0.166034143	0.14658	1.132724	0.25733	0.345404
13514	TBCE	422.3925135	0.166008004	0.111935	1.483073	0.138055	0.200238
14428	ZC4H2	176.3165436	0.165900244	0.139515	1.189122	0.234392	0.31843
12891	RPS6KB1	777.7288927	0.165311918	0.09864	1.675914	0.093755	0.142556
11849	CALCOCO2	2841.206464	0.165262192	0.082356	2.006692	0.044782	0.074076
12956	ZMYM2	1141.500157	0.165151598	0.099808	1.654692	0.097987	0.148243
12565	ZNF174	211.7992491	0.165027835	0.092527	1.78356	0.074495	0.11621
12935	MTIF2	809.0985447	0.164966575	0.099155	1.663717	0.096169	0.145729
14370	MRPS2	895.9622286	0.164811275	0.136567	1.206815	0.227504	0.31032
11968	XPC	1020.084321	0.164775472	0.083681	1.969091	0.048943	0.080158
12531	DCP1A	613.389341	0.164609949	0.09169	1.795297	0.072606	0.113571
14398	LATS2	982.4588124	0.164551189	0.137345	1.198086	0.230884	0.314318
15469	CCDC138	30.36951989	0.164402695	0.188901	0.87031	0.384131	0.48673
16082	P2RY1	93.37798267	0.164230643	0.238939	0.687334	0.491872	0.599502
13155	ZNF193	154.7360042	0.163957894	0.102918	1.593086	0.111141	0.165606
14350	EIF2AK2	404.6901111	0.163889758	0.135136	1.212778	0.225215	0.307562
12039	FBXL14	618.8501361	0.163833354	0.084114	1.947758	0.051444	0.083757
14111	OC1001305	44.96653395	0.163595857	0.127461	1.283496	0.199318	0.276865
15681	NUDT14	516.3215383	0.163168074	0.200797	0.8126	0.416447	0.520552
12853	C12orf4	445.4795851	0.16301889	0.096432	1.690501	0.090932	0.138673
10728	TBP	215.122491	0.162840456	0.068201	2.38767	0.016956	0.030979
14209	MRPL13	672.1689615	0.162770974	0.129797	1.25404	0.209828	0.289452
14317	TRIM38	1121.916142	0.162647671	0.132724	1.225456	0.220403	0.301748
14524	43899	462.7481501	0.1622021	0.13987	1.159659	0.246188	0.332245
13622	ATPAF2	299.9770943	0.162138537	0.111944	1.44839	0.147508	0.212253

16596	BET3L	2.236217315	0.161930024	0.295345	0.548273	0.583504	0.688909
14427	LOC72902(	27.87313254	0.161922634	0.136161	1.189197	0.234362	0.318412
13857	PHF1	1204.879824	0.161794641	0.118447	1.365966	0.171949	0.243226
12716	PAK1	1223.073781	0.161348778	0.093395	1.727598	0.08406	0.129574
14027	SUMO1P3	161.6498241	0.16118296	0.122874	1.31177	0.189598	0.26494
11532	TBCCD1	284.7984812	0.16118292	0.076497	2.107053	0.035113	0.059682
12539	FAM53C	933.5476257	0.161172077	0.089848	1.793836	0.072839	0.11386
16827	TSPAN10	23.20759632	0.161158902	0.332994	0.483969	0.628408	0.732004
12923	TERF1	593.9859284	0.16112933	0.096711	1.666095	0.095694	0.145145
16715	TIMP4	52.63902387	0.16097291	0.311959	0.516006	0.60585	0.710456
11886	NFYA	633.8580682	0.160876382	0.080737	1.992603	0.046305	0.076361
10024	PTPRA	1944.476565	0.160698021	0.06078	2.643924	0.008195	0.016025
15364	FAM115A	321.4020021	0.160529047	0.178165	0.901013	0.367581	0.468951
17148	KLB	29.11987096	0.160280521	0.402119	0.39859	0.690195	0.788927
13745	ABI1	1339.953661	0.160224037	0.114155	1.40356	0.16045	0.228809
15459	LEKR1	18.89032753	0.160048247	0.183366	0.872834	0.382753	0.485306
15704	ICA1L	124.0019593	0.159682926	0.198596	0.804058	0.421364	0.525927
15819	PI4KAP1	292.1025301	0.159665632	0.207226	0.770492	0.441008	0.546444
14487	FAM117A	257.3487274	0.159635723	0.136154	1.172464	0.241011	0.326088
12594	SCAMP2	2592.684249	0.159617952	0.089912	1.775269	0.075853	0.118056
16987	CXCL14	9575.495943	0.159581934	0.360796	0.442305	0.658268	0.759564
14737	FYN	1167.38948	0.159524708	0.146633	1.087919	0.276631	0.367934
13003	ACOT13	781.8876132	0.159494158	0.097044	1.643531	0.100273	0.151154
15224	JAG1	3946.782007	0.159390987	0.169495	0.940388	0.347018	0.446789
14941	WBP11P1	8.477398222	0.159276959	0.155491	1.02435	0.30567	0.401055
14961	TRPS1	467.7814314	0.159260774	0.156339	1.018686	0.308352	0.403984
15325	CLCN2	113.8087742	0.159237972	0.174498	0.912548	0.36148	0.462341
14526	BTN2A2	536.6955212	0.159229786	0.13747	1.158287	0.246747	0.332954
15821	FBXO17	2292.106724	0.159228214	0.207038	0.769077	0.441847	0.547415
13122	SETD1B	773.1200789	0.15905448	0.099166	1.60392	0.108732	0.162418
13274	MTIF3	911.1981196	0.158977571	0.102411	1.552349	0.120579	0.178052
13843	SPCS1	1436.695184	0.158872311	0.115924	1.370485	0.170536	0.24147
14605	C16orf61	446.0877735	0.158653968	0.140136	1.132139	0.257576	0.345686
17844	LRFN2	1.465168313	0.158576118	0.670079	0.236653	0.812926	0.892979
16938	C2orf62	17.29316647	0.158411372	0.349516	0.453231	0.650382	0.752636
13135	SAFB2	1481.097151	0.158169574	0.098981	1.597985	0.110046	0.164219
13365	RPAP2	221.509565	0.157898807	0.103741	1.522052	0.127996	0.187718
13733	SLC37A3	895.4811143	0.157894839	0.112262	1.406485	0.15958	0.227767
13464	SUB1	2860.931375	0.157716592	0.10531	1.497638	0.134227	0.195409
17992	REG4	0.55580467	0.157401241	0.761132	0.206799	0.836167	0.910944
16435	PCDHA7	33.52972058	0.157374336	0.265449	0.59286	0.553275	0.659856
15625	RTTN	262.1149218	0.157307464	0.189982	0.828011	0.407664	0.5114
16806	QPCT	765.9087177	0.157267323	0.321707	0.488853	0.624946	0.72888
13182	AMBRA1	842.3100492	0.157253647	0.099219	1.584921	0.112984	0.168002
12880	HMGXB4	435.8929741	0.157208195	0.093557	1.68035	0.092889	0.141361
14340	LRRC8D	858.130694	0.157171082	0.12914	1.217059	0.223582	0.305587
16462	LDLR	421.9910021	0.156933133	0.268964	0.583473	0.559575	0.666276

13924	MTMR2	1316.584133	0.156854089	0.116824	1.342658	0.179383	0.252524
16546	PNMA2	1678.16065	0.156543443	0.279928	0.559227	0.576007	0.68234
11798	LARP4B	1181.151475	0.155993387	0.077153	2.021875	0.043189	0.071754
15711	FOXP1	969.5319307	0.155761033	0.194477	0.800921	0.423177	0.527955
11832	HIF1AN	1117.078144	0.155420915	0.077281	2.011104	0.044314	0.073412
14576	GCA	505.2606716	0.155410476	0.135958	1.143081	0.253005	0.340227
13545	FAM21B	615.6603977	0.155333471	0.10549	1.472499	0.140886	0.203877
16939	HOMER2	85.36587154	0.155292138	0.342875	0.452912	0.650612	0.752857
15567	NUDT16P1	176.9678835	0.154882603	0.18369	0.843173	0.399132	0.502562
11618	ZNF398	353.2174259	0.154747817	0.074614	2.073975	0.038082	0.064248
15476	HOXC4	136.9919442	0.154617885	0.178261	0.86737	0.385739	0.488555
14972	MICA	415.1132142	0.154587244	0.152243	1.015397	0.309917	0.405736
13544	SLC30A5	1192.833832	0.154484135	0.104899	1.472693	0.140834	0.203816
15645	MNS1	147.0736506	0.154269314	0.18728	0.823735	0.41009	0.513753
12498	MOCS3	202.4866415	0.154193217	0.085421	1.805103	0.071059	0.111443
16167	)C1001343	5.03977695	0.154120386	0.232546	0.662752	0.50749	0.615283
13563	VGLL4	1606.843004	0.154109608	0.105088	1.466479	0.142518	0.20597
14643	ZNF761	318.6678415	0.153890063	0.13747	1.119447	0.262949	0.351982
12124	SFRS15	708.3447322	0.153870229	0.080154	1.919692	0.054897	0.088752
17350	CDHR2	451.6472023	0.153869391	0.43605	0.352871	0.724185	0.818142
15336	HSPA12A	1097.179227	0.152942138	0.168101	0.90982	0.362917	0.463846
12018	UMPS	539.9237636	0.152940141	0.078176	1.956354	0.050423	0.082239
15080	FMNL2	1886.592252	0.152933196	0.155511	0.983423	0.325399	0.422961
12123	RBM4	1756.29329	0.15280192	0.079514	1.921691	0.054645	0.088352
13419	TOP1P1	104.478197	0.152375015	0.100996	1.508718	0.131371	0.191892
13951	FLJ10038	266.9457358	0.152268505	0.114149	1.333939	0.182224	0.256022
14594	TUSC2	485.6256756	0.152230657	0.133739	1.13827	0.255008	0.342497
15617	NDUFV1	2738.111403	0.152013554	0.182864	0.831293	0.405808	0.509332
14773	IARS	2512.432143	0.151990594	0.141193	1.076476	0.281714	0.373782
13326	KIN	199.3955635	0.151893778	0.098981	1.534583	0.124886	0.183693
14846	TMEM201	309.1166181	0.15178879	0.14418	1.052773	0.292445	0.386112
12476	PSMD10	939.2756474	0.151773809	0.083849	1.81008	0.070283	0.110426
14091	THOC1	353.2803587	0.151472267	0.117353	1.290742	0.196793	0.273745
15029	TLE4	237.3092503	0.15117903	0.151659	0.996836	0.318844	0.41584
15457	C20orf94	24.75394045	0.151101603	0.172988	0.873482	0.382401	0.484922
16801	PSD3	371.5518642	0.151090158	0.308884	0.489148	0.624737	0.728853
14525	GNA13	2804.539087	0.151007018	0.130243	1.159422	0.246284	0.332352
15752	ARVCF	260.9688612	0.150921103	0.191191	0.789375	0.429893	0.534937
15614	STAG3L3	184.6445722	0.150786966	0.181194	0.832186	0.405304	0.508797
13533	PAK1IP1	263.3441456	0.150709847	0.102126	1.475723	0.140018	0.2028
14829	CASK	1032.186975	0.150540601	0.142397	1.057189	0.290425	0.383852
16063	GOLGA6L1	124.5320225	0.150524156	0.217092	0.693367	0.488079	0.595492
12147	UBE3B	1309.395735	0.150441606	0.07864	1.91304	0.055743	0.08995
13388	ZBTB49	78.00935008	0.150393483	0.099292	1.514653	0.12986	0.190125
13980	F11R	2814.81165	0.150331388	0.113536	1.324083	0.185476	0.260051
14518	EXTL3	1066.135143	0.150219191	0.129364	1.161212	0.245556	0.331529
14044	C12orf5	393.315146	0.150114126	0.115236	1.302672	0.192687	0.268911

17893	OC1001303	0.541839525	0.150111113	0.654753	0.229264	0.818664	0.896833
14796	EFR3A	2450.890912	0.150080459	0.140605	1.067392	0.285795	0.378607
15537	IL15	173.8296612	0.150041908	0.176642	0.849413	0.395651	0.499142
14537	C17orf100	59.22704753	0.150009558	0.129954	1.154332	0.248364	0.334882
17380	FAM138F	2.096984571	0.149915248	0.43356	0.345777	0.72951	0.822687
12562	PSME3	1787.711527	0.149822176	0.083978	1.784069	0.074412	0.116116
15701	HSPB8	5808.684841	0.149813687	0.185892	0.805918	0.42029	0.524687
11956	C6orf89	2487.761282	0.149745694	0.075906	1.972765	0.048522	0.079542
12990	GZF1	451.6374464	0.149694095	0.090863	1.647477	0.09946	0.150078
16458	TAS2R31	2.864131163	0.149692597	0.25579	0.585216	0.558402	0.665041
13456	HEXIM1	1071.170841	0.149593269	0.09972	1.500131	0.133581	0.194585
13913	RQCD1	149.7393937	0.149554336	0.111044	1.346802	0.178044	0.250833
14249	KIAA0922	414.5142631	0.149496421	0.120343	1.242258	0.214141	0.294574
12993	POLDIP3	1756.694202	0.149180107	0.090613	1.646347	0.099692	0.150383
13205	BBS4	453.2001616	0.149136688	0.094503	1.578116	0.114539	0.170017
15985	GPR4	1005.625992	0.149063073	0.208068	0.716416	0.473734	0.580899
18494	CDH7	0.652832599	0.148802835	1.318891	0.112824	0.91017	0.96465
14363	LIPT1	139.3384666	0.148795345	0.12306	1.20913	0.226613	0.309256
14188	DNAJC10	2452.365583	0.148732896	0.117938	1.261107	0.20727	0.286348
12753	NUBP1	561.5831677	0.14863422	0.086565	1.717019	0.085976	0.132142
15780	COQ2	198.3823841	0.148627733	0.190257	0.781193	0.434689	0.539945
12816	ZSCAN22	87.89303173	0.14862471	0.087399	1.700531	0.089031	0.136166
13770	RING1	716.6020599	0.148530955	0.106418	1.395732	0.162795	0.231732
13928	GTF2F2	353.3866926	0.1484636	0.110666	1.341542	0.179744	0.252956
12435	SENP5	707.1599422	0.148358241	0.081258	1.825776	0.067884	0.107004
16887	FOXD4L2	3.100609964	0.148324612	0.317041	0.46784	0.639899	0.74274
17059	P2RY4	0.983926954	0.148234853	0.349203	0.424495	0.671205	0.771223
13024	TAF9	1205.790664	0.1481473	0.090555	1.635993	0.101841	0.15327
12868	UBA2	2151.649212	0.148081504	0.087748	1.687576	0.091493	0.139365
12248	LSG1	823.3167606	0.148077111	0.07867	1.882268	0.0598	0.0957
14417	PHB	2950.846906	0.148074879	0.124097	1.193223	0.232782	0.316488
10909	TLK2	527.6451487	0.14805406	0.063868	2.318117	0.020443	0.036731
15736	FAR2	131.5423955	0.147963086	0.18648	0.793452	0.427515	0.532513
17047	DUOX2	5.220414987	0.147942587	0.345454	0.428255	0.668465	0.76864
13346	ZNF599	96.63957599	0.147764945	0.09662	1.529337	0.126181	0.185319
15345	NBN	2129.392084	0.147623425	0.162914	0.906142	0.364861	0.46607
13678	MAP2K1	1622.097088	0.147529219	0.103306	1.428074	0.153271	0.219642
11394	C12orf43	278.473953	0.147523594	0.068532	2.15263	0.031348	0.053927
13576	TTC3	3625.005817	0.147520072	0.100866	1.462535	0.143595	0.207322
14914	LOC202781	205.5866674	0.147400577	0.142852	1.031844	0.302145	0.397073
15377	SUMF1	1128.603801	0.147390396	0.164545	0.895746	0.370388	0.472133
16285	C15orf51	14.36178496	0.147283808	0.233047	0.631992	0.527392	0.634781
12889	RXRB	863.0093539	0.147224716	0.087825	1.676346	0.09367	0.14245
15475	ATL1	219.0796217	0.147140229	0.169548	0.86784	0.385482	0.488261
12827	METAP2	1544.502642	0.147095559	0.086698	1.696635	0.089766	0.137171
11600	PPP1R8	770.9915739	0.146967228	0.070644	2.080389	0.03749	0.06335
16309	APOBEC2	2.771918695	0.146593634	0.234348	0.625537	0.531619	0.638927

13762	SETD6	272.526911	0.14635424	0.10462	1.398909	0.16184	0.230507
12129	VPS52	1106.12541	0.146269292	0.07626	1.918024	0.055108	0.089057
12041	RIPK1	1113.583472	0.146160906	0.075051	1.947481	0.051477	0.083797
12698	MARK2	891.1767837	0.146155575	0.084242	1.734959	0.082748	0.127732
12108	MBTPS1	2750.802278	0.146067502	0.075833	1.926177	0.054082	0.087551
16214	GJA1	3588.693252	0.145695872	0.223808	0.650987	0.515055	0.622647
14504	SFRS11	2421.923079	0.145064244	0.124513	1.165055	0.243997	0.329742
14175	RPUSD2	150.2866275	0.144912454	0.114632	1.264157	0.206174	0.285098
13238	YRDC	308.3657576	0.144891437	0.09243	1.567572	0.116981	0.173209
14384	LOC28503[illegible]	34.64011018	0.144819278	0.120424	1.202573	0.229141	0.31225
15941	LDLRAD3	372.3472216	0.144813162	0.198889	0.728111	0.466546	0.573663
16823	GSTM2P1	1.467216418	0.144653096	0.298321	0.48489	0.627754	0.731416
13439	TSR1	1182.031363	0.144638711	0.096215	1.503284	0.132766	0.193641
15008	IFT122	910.9273839	0.144449706	0.143571	1.006118	0.314359	0.410564
14128	SAMD4B	1543.308887	0.144434885	0.112883	1.279511	0.200717	0.278472
14459	CAST	6648.918264	0.144425691	0.122444	1.179521	0.238191	0.322876
13787	HIATL1	1186.641074	0.144288108	0.103778	1.390355	0.164421	0.233758
14522	NCAPD3	332.6789466	0.144192132	0.124287	1.160154	0.245986	0.332019
13472	[illegible]NRNPA3P	68.15263999	0.144016824	0.096255	1.496198	0.134602	0.195839
14018	ZMIZ1	2852.189615	0.144013532	0.109629	1.313639	0.188968	0.264229
14560	STARD3NL	1299.586947	0.143943144	0.125359	1.148248	0.250866	0.337742
15222	QKI	3401.565059	0.143802203	0.152768	0.941311	0.346546	0.446239
16403	GRRP1	73.71598109	0.1434057	0.238814	0.600492	0.548178	0.655054
14613	COX7A2L	2199.80905	0.143383051	0.127075	1.128335	0.259179	0.347647
17442	KRT23	3.692963941	0.143375726	0.436429	0.32852	0.742518	0.834381
13984	ZNHIT6	377.684288	0.143311218	0.108258	1.323791	0.185572	0.260112
14497	OFD1	805.1403253	0.142831239	0.12221	1.16874	0.242508	0.327889
17210	AZGP1	790.128711	0.142778083	0.370346	0.385526	0.699848	0.797078
14348	N6AMT1	243.4002593	0.142422446	0.117362	1.213531	0.224927	0.307276
13608	MON1B	682.2760264	0.142356926	0.097952	1.453337	0.14613	0.210486
14782	ACAA1	1197.9783	0.142305093	0.132544	1.073643	0.282983	0.375236
15057	MORN4	187.9326817	0.142178002	0.14391	0.987962	0.323171	0.4207
15087	BTAF1	957.9721428	0.142129438	0.145005	0.980169	0.327003	0.424841
15344	ACYP1	79.77639553	0.142096769	0.156819	0.906121	0.364872	0.46607
14687	RAP1B	3112.324413	0.142015096	0.128573	1.10455	0.269354	0.359476
13977	WDTC1	1496.505043	0.141902716	0.107109	1.324848	0.185222	0.25975
13143	HNRNPH1	4094.747589	0.141753219	0.088805	1.596227	0.110438	0.164703
17048	CCDC8	319.6816026	0.141734568	0.331573	0.427462	0.669043	0.769235
13944	MAVS	2494.096964	0.141719635	0.106069	1.33611	0.181513	0.255152
12781	TAF4	375.3056468	0.141701267	0.082812	1.711118	0.087059	0.133514
17193	PCDHA13	10.91940916	0.141655503	0.364385	0.388752	0.69746	0.79512
13939	SMYD4	390.1660081	0.141606174	0.105781	1.338673	0.180677	0.25405
17867	RFPL4B	1.659697263	0.141473173	0.603506	0.234419	0.81466	0.893794
13001	PCID2	548.6837418	0.141265841	0.085902	1.644501	0.100073	0.150875
14492	[illegible]MPHOSPH[illegible]	237.3218262	0.141017152	0.120402	1.17122	0.24151	0.326656
16909	CLDN3	345.2209017	0.140817957	0.304076	0.463101	0.643292	0.745707
14775	CMTM6	2058.840332	0.140806124	0.13092	1.075513	0.282145	0.374303

15516	RPL32P3	137.0114615	0.140714955	0.164776	0.853975	0.393119	0.496618
14339	HLCS	567.4061325	0.140538781	0.115456	1.217253	0.223508	0.305529
14386	SH3GLB1	3427.47953	0.140490239	0.116875	1.202054	0.229343	0.312481
15401	TIGD1	131.9386263	0.140469401	0.15778	0.890286	0.373312	0.475118
13406	MAPKAP1	1325.222758	0.140432218	0.092961	1.510654	0.130877	0.191356
14622	PARP4	2384.558534	0.140087261	0.124392	1.126173	0.260092	0.348657
16203	TMEM217	38.53544288	0.140047294	0.214188	0.653853	0.513207	0.62085
13452	TRIM35	503.6913343	0.1399336	0.093198	1.501471	0.133234	0.194136
14640	ATP6AP1	4514.011137	0.139521354	0.124565	1.120065	0.262686	0.351701
14432	LAMP1	6462.996623	0.139482324	0.117377	1.188325	0.234705	0.318763
14225	RARS2	629.6815447	0.139330508	0.111387	1.250863	0.210984	0.290721
16202	TMEM151E	12.49415266	0.139216625	0.212924	0.653832	0.51322	0.62085
16419	PABPC5	27.59107851	0.13913901	0.232511	0.59842	0.54956	0.656064
15925	FAM35B2	94.17886129	0.139064883	0.189209	0.734981	0.462351	0.569077
16487	PCDHB4	150.4858181	0.139030445	0.241417	0.575894	0.564687	0.671349
16221	CG030	60.52310004	0.139013382	0.2142	0.648987	0.516346	0.623918
17472	FLJ30679	0.968766794	0.138929978	0.432893	0.320934	0.74826	0.839438
13881	ZNF70	164.7622718	0.138904825	0.10256	1.354382	0.175615	0.247981
15849	HOXA5	138.9655304	0.138762292	0.182877	0.758773	0.447988	0.554042
16188	YS1-DBNDI	32.66656663	0.138752776	0.211409	0.656323	0.511616	0.619483
12030	FRG1	548.3197634	0.138665949	0.071033	1.952128	0.050923	0.082971
13774	PPP5C	1042.312867	0.138382264	0.099199	1.394996	0.163017	0.23198
15410	DOT1L	515.0008523	0.138057059	0.155713	0.886614	0.375287	0.477352
14069	ZNF609	948.2072493	0.138011719	0.106417	1.296897	0.194667	0.27121
17802	HIST1H4B	0.608073135	0.137818444	0.560196	0.246018	0.805668	0.887086
14624	PPP3R1	1486.273341	0.137678947	0.122343	1.125349	0.260441	0.349077
15111	JAZF1	652.4123777	0.137669864	0.141523	0.972773	0.330666	0.428918
12740	MED6	274.6777732	0.137668769	0.080015	1.720535	0.085335	0.131292
14728	WNK1	3970.318963	0.137600219	0.126229	1.090087	0.275675	0.366886
15995	NEK11	230.0176163	0.137489182	0.192475	0.714324	0.475027	0.58212
13613	FAM48A	732.8175472	0.13741571	0.094643	1.451937	0.146519	0.210969
15582	TPD52	1614.420772	0.137395771	0.163546	0.840104	0.40085	0.50424
16131	FAM167B	360.2006995	0.137309844	0.204192	0.672455	0.501294	0.60913
15999	CCT6B	51.12345326	0.137282041	0.192392	0.713553	0.475503	0.582558
15958	PGM2L1	597.4923461	0.137161168	0.189165	0.725088	0.468398	0.575327
16810	PGBD5	501.6000501	0.137130895	0.281133	0.487779	0.625707	0.729594
12344	DDX19A	841.2999371	0.13706522	0.074081	1.850199	0.064285	0.102069
17870	SH2D6	1.436483789	0.137054499	0.586856	0.23354	0.815342	0.894321
16005	TMEM141	1320.224103	0.136991791	0.19262	0.711201	0.47696	0.584123
13679	C3orf19	354.6040189	0.136912424	0.0959	1.427658	0.15339	0.219791
14986	SERTAD2	1327.503941	0.136908063	0.135519	1.010248	0.312377	0.408574
17414	C12orf42	1.255295267	0.136893329	0.404791	0.338182	0.735226	0.827562
15079	ELOVL5	2236.970924	0.136624858	0.138934	0.983381	0.32542	0.422961
13053	ZNF766	360.4878474	0.13660291	0.083861	1.628929	0.103328	0.155162
16947	P2RY12	44.29304633	0.136522085	0.302929	0.450674	0.652225	0.754362
12257	VPS8	759.0105732	0.136421345	0.072531	1.880864	0.05999	0.095938
16506	C9orf96	7.151514404	0.136259052	0.238815	0.570562	0.568296	0.674856

18126	GABRA4	1.034852893	0.136018756	0.761203	0.178689	0.858182	0.928058
14872	ANAPC1	552.602907	0.135825323	0.129974	1.045016	0.296015	0.390143
14062	LOC72819(	153.6064288	0.135623224	0.104446	1.298498	0.194116	0.270578
14994	CUL9	590.5363085	0.135599622	0.134486	1.008282	0.313319	0.409588
17495	AKR1C4	3.435530102	0.135537007	0.429396	0.315646	0.752272	0.842828
15792	PRPS2	950.8864771	0.135201451	0.17362	0.778722	0.436143	0.54134
17362	PXDNL	136.6719972	0.135135927	0.385943	0.350145	0.72623	0.819885
16086	INSIG1	927.9027267	0.135074963	0.196989	0.685698	0.492903	0.600609
13407	C14orf119	831.1356556	0.135067326	0.089419	1.510491	0.130918	0.191402
13665	ZBTB22	478.9291437	0.135060963	0.09421	1.433615	0.151682	0.217572
12757	CDC16	1420.66805	0.134963181	0.078648	1.716033	0.086156	0.132365
11777	TERF2	603.9584392	0.134915625	0.066499	2.028839	0.042475	0.070693
13849	UQCC	994.1969268	0.134891818	0.09856	1.36862	0.171118	0.24219
15276	NPHP1	171.8603074	0.134711482	0.14541	0.926426	0.354225	0.454514
14906	AZIN1	2733.03532	0.13462494	0.130177	1.034165	0.301059	0.395885
15631	EXOC3	1840.06557	0.134622328	0.162855	0.826641	0.408441	0.512177
13816	ZSCAN5A	74.49801622	0.134610297	0.097465	1.381115	0.167244	0.237272
14131	FAM103A1	283.6635839	0.134204057	0.104922	1.279083	0.200868	0.278621
14173	PMS2	797.2568401	0.134108145	0.106078	1.264237	0.206145	0.285095
15828	GNG11	3036.920591	0.133699479	0.174389	0.766673	0.443276	0.548926
15774	STAMBPL1	302.9658091	0.133629053	0.170617	0.783209	0.433504	0.538678
16173	HCG4	131.8576183	0.133428461	0.201769	0.661294	0.508424	0.616188
16593	LIMS1	729.2331376	0.133427534	0.2428	0.549537	0.582637	0.688258
13947	ZNF500	188.9094178	0.132921781	0.099566	1.335015	0.181871	0.255601
15865	HELB	31.06156659	0.132824883	0.176169	0.753963	0.450871	0.557046
14760	ATG2A	651.6997369	0.132583115	0.122573	1.081666	0.279401	0.371039
14413	HERC4	793.2980424	0.132535423	0.11098	1.194226	0.23239	0.316039
14789	MRPS5	1056.813253	0.132360082	0.123582	1.071032	0.284155	0.376613
15225	AGFG1	2036.60283	0.13221985	0.140614	0.940301	0.347063	0.446816
13336	ARNT	1273.569497	0.132117984	0.086236	1.532059	0.125508	0.184469
15753	OSGIN2	1043.036466	0.132092155	0.167484	0.788686	0.430295	0.535404
13690	NSUN4	542.2857339	0.131933422	0.09275	1.422466	0.154891	0.221769
13871	MED18	228.0495327	0.1318625	0.096865	1.361295	0.17342	0.245059
16218	NR1I3	9.873226081	0.131798226	0.202853	0.649724	0.51587	0.623478
15088	SLC25A38	508.3608434	0.131775367	0.134526	0.979551	0.327308	0.42521
18213	ADH4	4.054104017	0.131631621	0.826513	0.159261	0.873463	0.940029
15193	ACP6	224.5015066	0.131583974	0.138479	0.950209	0.342006	0.441234
17314	SBF1P1	0.910512621	0.131542363	0.361522	0.363857	0.715965	0.810536
17607	OC1001446	1.974652333	0.13144931	0.449614	0.29236	0.770011	0.857215
14585	CXorf38	290.3392523	0.131298727	0.115035	1.141376	0.253714	0.340969
14237	FANCF	422.2356025	0.131214039	0.105333	1.245709	0.212871	0.293074
14639	SLC12A4	1706.347215	0.131125898	0.116941	1.121295	0.262162	0.351043
15347	CBR1	1919.011891	0.130947095	0.144581	0.905701	0.365094	0.466263
14088	PIP5K1A	1074.782496	0.1308425	0.101286	1.291808	0.196424	0.273289
14826	SERP1	3696.856554	0.130609226	0.123508	1.057492	0.290287	0.383754
15237	LOC283267	450.8925796	0.130484723	0.13922	0.937254	0.348628	0.448478
11001	KIAA0174	2955.127679	0.130448044	0.056996	2.288725	0.022095	0.039368

16097	PTPRN2	297.330411	0.130326227	0.191034	0.682213	0.495104	0.602878
15643	TCEAL3	384.6404621	0.130200035	0.157985	0.82413	0.409866	0.513579
14219	ANAPC4	461.8210953	0.129838228	0.10375	1.251452	0.21077	0.290527
15841	ASB13	1300.80533	0.129822345	0.170632	0.760832	0.446757	0.552729
14745	C1orf212	765.5282355	0.12966125	0.119543	1.084644	0.27808	0.36966
15759	FUZ	415.197392	0.129639164	0.16473	0.786978	0.431295	0.536432
13496	ZCCHC8	627.7005431	0.129517404	0.086954	1.489488	0.136359	0.19805
14286	KDM5A	1336.969093	0.129255333	0.104775	1.233643	0.217336	0.298194
12672	TERF2IP	1570.651856	0.129142762	0.073946	1.746458	0.080731	0.124875
14261	BAT2	4051.129104	0.129067343	0.104081	1.240069	0.21495	0.295437
15671	KIAA0907	811.0989031	0.128971718	0.158087	0.815829	0.414598	0.518582
15951	TUBGCP6	951.3445467	0.128933556	0.177465	0.726531	0.467513	0.574494
16798	PPP2R2B	47.81844637	0.128925548	0.262782	0.490618	0.623697	0.72777
15115	OGT	3120.261624	0.12889343	0.132708	0.971255	0.331421	0.429784
15842	GPR135	20.49081795	0.128629673	0.169055	0.760875	0.446732	0.552729
14682	ABCF1	1291.246396	0.128460503	0.115994	1.107474	0.268089	0.357884
17107	MYO1H	4.507692746	0.128408604	0.312648	0.410714	0.681283	0.780606
15287	HSPD1	9396.032892	0.128358408	0.138927	0.92393	0.355523	0.455851
14881	ZNF528	327.588175	0.12829226	0.123	1.043024	0.296937	0.391121
14388	XPO1	3127.901191	0.128278037	0.106752	1.201648	0.2295	0.312651
15484	NGFRAP1	1957.895091	0.128251251	0.148109	0.865927	0.38653	0.489303
17339	AMY2A	2.041338924	0.128242303	0.358142	0.358077	0.720286	0.814205
14951	C22orf23	39.20754178	0.128194374	0.125398	1.022299	0.306639	0.401984
16364	GPR176	226.975094	0.128134861	0.210268	0.609389	0.542267	0.649534
15460	KANK1	1904.356455	0.128045155	0.146722	0.872707	0.382823	0.485331
16976	GTF2H2B	92.23973786	0.128013771	0.28817	0.44423	0.656877	0.758271
14883	ZNF30	61.45171595	0.127935236	0.122705	1.042623	0.297123	0.391312
12795	NUP88	590.2755612	0.127799539	0.074909	1.706065	0.087996	0.134803
15295	LIN9	86.80024978	0.127581399	0.138316	0.92239	0.356325	0.456642
13524	CEP63	517.3491689	0.127455967	0.086136	1.479702	0.138953	0.201391
13969	KHDRBS1	3580.693332	0.127259475	0.095798	1.328411	0.184042	0.258244
15509	CLEC16A	764.600628	0.127052214	0.148314	0.856643	0.391642	0.494976
12915	ELAVL1	1714.845189	0.126514846	0.07581	1.668831	0.095151	0.14441
13089	ZNF830	305.5651123	0.126507189	0.078354	1.614558	0.106406	0.159345
14099	TMEM87A	1672.553771	0.1264671	0.098146	1.288557	0.197552	0.274625
17246	DOC2B	18.64144495	0.12634001	0.334651	0.377528	0.705781	0.802158
17004	ANKRD45	34.95707284	0.126325579	0.288121	0.438447	0.661062	0.761993
14521	CCDC125	229.2086173	0.126260057	0.108824	1.160223	0.245958	0.332004
15514	RAI1	788.8641825	0.126228691	0.147584	0.8553	0.392385	0.495755
15561	RAPH1	1017.169173	0.126155635	0.149404	0.844394	0.398449	0.50187
15741	CABC1	1101.073493	0.126126546	0.159274	0.791883	0.428429	0.533488
14726	MRPL48	435.5364758	0.12612549	0.115637	1.090706	0.275402	0.366577
15967	COX8A	2639.850048	0.126078746	0.174283	0.723413	0.469426	0.576265
15218	SLC25A11	1350.670414	0.125994654	0.133623	0.942912	0.345726	0.4453
15581	LARGE	259.8267786	0.125642085	0.149555	0.840109	0.400847	0.50424
14394	RWDD3	212.4712682	0.125626449	0.104762	1.199156	0.230467	0.313838
14852	ɔC1001306	20.89942782	0.125528066	0.119374	1.051549	0.293006	0.386697

15415	NDUFAB1	1156.567905	0.125312257	0.141644	0.884697	0.37632	0.478511
16378	CLCF1	263.4002247	0.125266211	0.206737	0.60592	0.544568	0.651693
17408	CES3	989.79462	0.125069995	0.368548	0.339359	0.734339	0.826849
14938	IMMP1L	68.60424657	0.125067084	0.122096	1.024331	0.305679	0.401055
14074	C6orf62	3369.863289	0.125009095	0.096469	1.295849	0.195028	0.271617
13279	C6orf47	635.5215904	0.124912893	0.080515	1.551433	0.120798	0.178309
13022	PPP4R1	1353.410998	0.124837055	0.076274	1.636694	0.101694	0.153073
15273	CFLAR	1786.820565	0.124729263	0.134504	0.92733	0.353755	0.454001
13938	LARS	2825.707413	0.124609655	0.093064	1.338969	0.180581	0.253951
15611	ACYP2	146.8870358	0.124522353	0.149622	0.832248	0.405269	0.508786
16555	DLL4	1505.768908	0.124445559	0.223193	0.55757	0.577138	0.683304
17171	FOXD4L6	1.317760176	0.124426129	0.316087	0.393645	0.693843	0.792034
17868	OR4F29	0.473581224	0.124400068	0.531401	0.234098	0.814909	0.893946
15220	KIAA1731	298.3773268	0.124395235	0.131972	0.942588	0.345892	0.445455
16721	LPHN1	409.1857025	0.124317201	0.241775	0.514186	0.607122	0.711649
17239	MSTN	14.31091859	0.124051936	0.327303	0.379013	0.704678	0.80123
15152	INPP5E	275.9988508	0.123919675	0.129259	0.958694	0.337713	0.436816
14676	FAM119A	231.2675329	0.123821622	0.111666	1.10886	0.267491	0.357256
16679	FAM195A	703.4612321	0.12368146	0.235218	0.525816	0.599016	0.703958
15518	TBC1D8	690.7537535	0.12346828	0.144661	0.853499	0.393383	0.496887
15060	C7orf60	374.6640038	0.123430321	0.124996	0.987475	0.32341	0.420926
17100	ID2B	9.835964672	0.123114126	0.29824	0.412802	0.679751	0.77917
15673	SASS6	91.5247617	0.123016599	0.15083	0.815597	0.414731	0.518672
15731	CCDC51	231.119768	0.12290359	0.154624	0.794854	0.426698	0.531604
13476	TAF11	522.8586414	0.122879682	0.082163	1.495559	0.134769	0.196023
15960	SLC48A1	777.3032584	0.122816591	0.169468	0.724717	0.468626	0.575568
16562	GJA5	564.8645163	0.122687646	0.220622	0.556099	0.578143	0.684206
13959	TRMT6	332.9857359	0.122667061	0.092123	1.331557	0.183006	0.256974
13763	WDR73	344.3229484	0.122520852	0.087653	1.397789	0.162176	0.230968
17843	GALR2	0.900195725	0.122499934	0.517658	0.236643	0.812934	0.892979
15873	NUCB2	2018.429745	0.122474553	0.163187	0.750518	0.452943	0.559323
17829	HTA	0.42494108	0.12246331	0.509713	0.240259	0.810129	0.890647
18039	TMEM40	0.385112638	0.122463197	0.616179	0.198746	0.842461	0.91541
18059	TAS2R1	0.331103161	0.122463149	0.629827	0.194439	0.845832	0.918089
18210	C9orf171	0.243954811	0.122462907	0.767116	0.159641	0.873164	0.939811
18292	USP17L2	0.226891279	0.122462762	0.843152	0.145244	0.884518	0.947815
18353	PRO1768	0.243514886	0.122462688	0.89669	0.136572	0.891369	0.952033
18477	OR3A1	0.307640697	0.122462462	1.058718	0.115671	0.907914	0.963144
18430	IFNA13	0.166810914	0.122462396	0.98956	0.123754	0.90151	0.95879
18498	NANOS2	0.259455924	0.122462327	1.092118	0.112133	0.910718	0.96504
18476	ARR3	0.146355143	0.122462208	1.056942	0.115865	0.90776	0.963033
18513	PRSS42	0.148129468	0.122462018	1.131733	0.108208	0.913831	0.967536
18563	OR2T8	0.144806859	0.122461779	1.219202	0.100444	0.919992	0.971435
18564	IFNA21	0.12513995	0.12246172	1.224604	0.100001	0.920343	0.97165
18624	IL19	0.145872555	0.122461488	1.317562	0.092946	0.925947	0.974521
18626	OR2A2	0.127763948	0.122461427	1.32107	0.092699	0.926143	0.974623
18627	MGC15885	0.125011286	0.122461419	1.323727	0.092513	0.926291	0.974726

18659	LOC64466S	0.186423947	0.122461267	1.423825	0.086009	0.93146	0.978432
18664	CRNA0009	0.178578734	0.122461223	1.437599	0.085185	0.932115	0.97891
18662	OR1F2P	0.126043267	0.122461051	1.435533	0.085307	0.932017	0.978892
18666	IFNA5	0.109630018	0.122460957	1.44543	0.084723	0.932482	0.979191
18732	PHOX2B	0.125943583	0.122460584	1.566209	0.078189	0.937678	0.981752
18712	ANP32D	0.111820503	0.122460476	1.575942	0.077706	0.938062	0.981752
18789	IFNK	0.11363336	0.122459857	1.729301	0.070815	0.943545	0.984322
18793	SNORA6	0.10878502	0.122459822	1.737738	0.070471	0.943819	0.984446
18792	FAM71E2	0.107985156	0.122459816	1.739173	0.070413	0.943865	0.984446
18795	CRNA002C	0.105893569	0.122459789	1.745435	0.07016	0.944066	0.984551
18932	SGCZ	0.187085672	0.122459672	1.862897	0.065736	0.947588	0.986718
18925	RLN3	0.134619439	0.122459244	1.891484	0.064742	0.948379	0.986718
18877	OC1001288	0.10532225	0.122458938	1.93499	0.063287	0.949538	0.986718
18867	IQCF6	0.090642376	0.122458713	1.959011	0.06251	0.950156	0.986718
18897	OR10W1	0.085783298	0.122458647	1.972388	0.062086	0.950494	0.986718
18979	TAAR9	0.070874578	0.12245697	2.261613	0.054146	0.956819	0.988177
19004	BPIL1	0.23848217	0.122456607	2.56681	0.047708	0.961949	0.989949
19161	RGS8	0.068630426	0.122454943	2.588555	0.047306	0.962269	0.989949
19181	SNORA54	0.09238297	0.122453291	2.85974	0.04282	0.965845	0.989949
19176	SNORA14E	0.072394966	0.122452654	2.91407	0.042021	0.966482	0.989949
19012	C1orf14	0.071167567	0.122452654	2.91407	0.042021	0.966482	0.989949
19034	FAM138D	0.047959867	0.122452409	2.91407	0.042021	0.966482	0.989949
19052	HIST1H4F	0.049364881	0.122452409	2.91407	0.042021	0.966482	0.989949
19086	CRNA0011	0.056599234	0.122452409	2.91407	0.042021	0.966482	0.989949
19096	OR10G4	0.048037958	0.122452409	2.91407	0.042021	0.966482	0.989949
19100	OR10V1	0.050038799	0.122452409	2.91407	0.042021	0.966482	0.989949
19105	OR1L4	0.049031868	0.122452409	2.91407	0.042021	0.966482	0.989949
19136	OR9I1	0.049398829	0.122452409	2.91407	0.042021	0.966482	0.989949
19150	PRAMEF2	0.048900744	0.122452409	2.91407	0.042021	0.966482	0.989949
19179	SNORA29	0.050451545	0.122452409	2.91407	0.042021	0.966482	0.989949
19072	LOC44004C	0.048205661	0.122452409	2.91407	0.042021	0.966482	0.989949
19165	RPL10L	0.050280664	0.122452409	2.91407	0.042021	0.966482	0.989949
19187	SPEM1	0.048634051	0.122452409	2.91407	0.042021	0.966482	0.989949
19164	RNU6ATAC	0.030463495	0.122452164	2.91407	0.042021	0.966482	0.989949
19182	SNORA55	0.030592725	0.122452164	2.91407	0.042021	0.966482	0.989949
19015	C7orf72	0.029144021	0.122452164	2.91407	0.042021	0.966482	0.989949
19027	DEFA6	0.030411723	0.122452164	2.91407	0.042021	0.966482	0.989949
19029	DPPA3	0.029174081	0.122452164	2.91407	0.042021	0.966482	0.989949
19053	HIST1H4K	0.029864509	0.122452164	2.91407	0.042021	0.966482	0.989949
19059	KLHL1	0.029091132	0.122452164	2.91407	0.042021	0.966482	0.989949
19076	LOC72827€	0.031254283	0.122452164	2.91407	0.042021	0.966482	0.989949
19093	OPN1LW	0.03043052	0.122452164	2.91407	0.042021	0.966482	0.989949
19095	OR10A3	0.029848293	0.122452164	2.91407	0.042021	0.966482	0.989949
19099	OR10H5	0.029594838	0.122452164	2.91407	0.042021	0.966482	0.989949
19103	OR13G1	0.028591521	0.122452164	2.91407	0.042021	0.966482	0.989949
19111	OR2V2	0.030475902	0.122452164	2.91407	0.042021	0.966482	0.989949
19119	OR4S1	0.028683554	0.122452164	2.91407	0.042021	0.966482	0.989949

19120	OR51F1	0.030331886	0.122452164	2.91407	0.042021	0.966482	0.989949
19124	OR5AP2	0.028945066	0.122452164	2.91407	0.042021	0.966482	0.989949
19125	OR5M8	0.031205749	0.122452164	2.91407	0.042021	0.966482	0.989949
19127	OR5W2	0.029149935	0.122452164	2.91407	0.042021	0.966482	0.989949
19131	OR6T1	0.031233063	0.122452164	2.91407	0.042021	0.966482	0.989949
19134	OR8I2	0.029144021	0.122452164	2.91407	0.042021	0.966482	0.989949
19147	PPIAL4E	0.033566368	0.122452164	2.91407	0.042021	0.966482	0.989949
19175	SMR3B	0.030738585	0.122452164	2.91407	0.042021	0.966482	0.989949
19192	SSX8	0.029192832	0.122452164	2.91407	0.042021	0.966482	0.989949
19039	FGF6	0.029516636	0.122452164	2.91407	0.042021	0.966482	0.989949
19064	KRTAP27-1	0.030078554	0.122452164	2.91407	0.042021	0.966482	0.989949
19123	OR52L1	0.028468418	0.122452164	2.91407	0.042021	0.966482	0.989949
19610	BASE	0.010756599	0.122451919	2.91407	0.042021	0.966482	NA
19618	C11orf94	0.010756599	0.122451919	2.91407	0.042021	0.966482	NA
19621	C14orf70	0.009569956	0.122451919	2.91407	0.042021	0.966482	NA
19630	C20orf71	0.00968232	0.122451919	2.91407	0.042021	0.966482	NA
19632	C20orf85	0.010108809	0.122451919	2.91407	0.042021	0.966482	NA
19643	C7orf66	0.009678283	0.122451919	2.91407	0.042021	0.966482	NA
19648	CATSPER4	0.00968232	0.122451919	2.91407	0.042021	0.966482	NA
19649	CDX4	0.010058465	0.122451919	2.91407	0.042021	0.966482	NA
19654	CELA3A	0.009628185	0.122451919	2.91407	0.042021	0.966482	NA
19678	CXorf27	0.011008287	0.122451919	2.91407	0.042021	0.966482	NA
19680	CYMP	0.01044637	0.122451919	2.91407	0.042021	0.966482	NA
19687	DEFA5	0.009560525	0.122451919	2.91407	0.042021	0.966482	NA
19693	DEFB109P1	0.009761189	0.122451919	2.91407	0.042021	0.966482	NA
19696	DEFB112	0.010267177	0.122451919	2.91407	0.042021	0.966482	NA
19711	DHRS7C	0.009518957	0.122451919	2.91407	0.042021	0.966482	NA
19724	FAM99B	0.010127223	0.122451919	2.91407	0.042021	0.966482	NA
19735	FTMT	0.010870212	0.122451919	2.91407	0.042021	0.966482	NA
19752	GLYCAM1	0.013239872	0.122451919	2.91407	0.042021	0.966482	NA
19765	GUCY2GP	0.010870212	0.122451919	2.91407	0.042021	0.966482	NA
19794	IL31	0.009626576	0.122451919	2.91407	0.042021	0.966482	NA
19796	IQCF2	0.009678283	0.122451919	2.91407	0.042021	0.966482	NA
19859	LCE2D	0.01012949	0.122451919	2.91407	0.042021	0.966482	NA
19871	LIPM	0.010870212	0.122451919	2.91407	0.042021	0.966482	NA
19883	LOC339568	0.009862242	0.122451919	2.91407	0.042021	0.966482	NA
19887	LOC642929	0.010870212	0.122451919	2.91407	0.042021	0.966482	NA
19893	LOC727924	0.01044637	0.122451919	2.91407	0.042021	0.966482	NA
19903	MLN	0.009497346	0.122451919	2.91407	0.042021	0.966482	NA
19908	MS4A12	0.010756599	0.122451919	2.91407	0.042021	0.966482	NA
19911	MSGN1	0.009678283	0.122451919	2.91407	0.042021	0.966482	NA
19924	NKX1-2	0.010267177	0.122451919	2.91407	0.042021	0.966482	NA
19939	OR10H4	0.010194136	0.122451919	2.91407	0.042021	0.966482	NA
19945	OR10T2	0.011535339	0.122451919	2.91407	0.042021	0.966482	NA
19975	OR2M7	0.009678283	0.122451919	2.91407	0.042021	0.966482	NA
19986	OR4A5	0.010756599	0.122451919	2.91407	0.042021	0.966482	NA
20019	OR52E2	0.009678283	0.122451919	2.91407	0.042021	0.966482	NA

20020	OR52E4	0.010058465	0.122451919	2.91407	0.042021	0.966482	NA
20022	OR52J3	0.009855685	0.122451919	2.91407	0.042021	0.966482	NA
20052	OR5P2	0.009676406	0.122451919	2.91407	0.042021	0.966482	NA
20064	OR6C68	0.011008287	0.122451919	2.91407	0.042021	0.966482	NA
20089	OR8G2	0.00968232	0.122451919	2.91407	0.042021	0.966482	NA
20107	PLGLA	0.009518957	0.122451919	2.91407	0.042021	0.966482	NA
20114	PPIAL4B	0.009567091	0.122451919	2.91407	0.042021	0.966482	NA
20186	SNORA14A	0.010959637	0.122451919	2.91407	0.042021	0.966482	NA
20188	SNORA1	0.011535339	0.122451919	2.91407	0.042021	0.966482	NA
20212	SNORA77	0.009678283	0.122451919	2.91407	0.042021	0.966482	NA
20480	TTTY1B	0.00968232	0.122451919	2.91407	0.042021	0.966482	NA
20486	TTTY6	0.010756599	0.122451919	2.91407	0.042021	0.966482	NA
14664	KPNA3	960.2171514	0.122435944	0.110137	1.111666	0.266282	0.355932
15063	AP3S2	1814.48561	0.122375783	0.123969	0.98715	0.323569	0.42105
14633	ARHGEF18	900.743414	0.122340098	0.109048	1.121894	0.261908	0.350803
14310	IKBKB	791.1187544	0.122326491	0.09971	1.226826	0.219888	0.30119
15185	ZCCHC2	497.9840054	0.122188211	0.128315	0.952253	0.340969	0.440129
15310	WWP2	1385.553495	0.122155767	0.132922	0.919007	0.358092	0.458471
17243	PCDHGB7	564.7998207	0.12212042	0.322872	0.378232	0.705259	0.801703
17711	GLIPR1L1	0.573131784	0.122108588	0.460676	0.265064	0.79096	0.87537
14185	C10orf46	1014.284059	0.122069455	0.096788	1.261205	0.207235	0.286319
16474	D2HGDH	556.697618	0.121947223	0.210544	0.5792	0.562454	0.669216
14000	ZC3H18	761.2003481	0.121704143	0.092328	1.318177	0.187444	0.262436
13301	KIAA0319L	2005.564124	0.121562559	0.078807	1.542541	0.122942	0.181174
15251	NUDCD1	399.4512879	0.121458711	0.130273	0.932341	0.35116	0.451321
14090	WDR26	2608.751234	0.121264642	0.093903	1.291381	0.196571	0.273456
17449	DCHS2	77.78216123	0.121234253	0.370807	0.326947	0.743708	0.83543
14402	AARS2	453.0620644	0.121204257	0.101227	1.197354	0.231169	0.314619
16463	DBF4	192.2112858	0.121122029	0.207623	0.583375	0.559641	0.666313
16002	IRAK2	206.860357	0.120820389	0.169536	0.712655	0.476059	0.583129
15695	NAV2	706.4879582	0.120655984	0.149325	0.808008	0.419086	0.523384
15201	HIVEP2	1182.17911	0.120589686	0.127301	0.94728	0.343496	0.442922
15930	HECTD2	324.6837246	0.12057866	0.164198	0.734348	0.462736	0.569372
15733	NUDT13	60.88469668	0.120405646	0.15147	0.794912	0.426664	0.531604
12683	PDCD7	424.7874742	0.120250473	0.068991	1.742998	0.081334	0.125698
16665	IL17RE	66.24843098	0.120212676	0.226796	0.530049	0.596078	0.701094
14315	C10orf57	503.174632	0.119961039	0.097855	1.225909	0.220233	0.301557
14267	C3orf1	1114.713851	0.11990363	0.096837	1.238198	0.215643	0.296265
14943	TIMM9	514.9535457	0.119832714	0.117044	1.02383	0.305916	0.401275
15765	VPS37B	653.3321585	0.119775003	0.152383	0.786011	0.431861	0.536948
15982	DUSP6	2312.026992	0.11974012	0.166917	0.717364	0.47315	0.580288
14989	BZW1	3459.999434	0.119546334	0.118386	1.009804	0.312589	0.40877
16951	BTBD18	1.751405258	0.119458321	0.265255	0.450352	0.652457	0.75443
14802	LDB1	1015.783865	0.119367958	0.112088	1.064947	0.2869	0.379916
15529	JRK	410.5071871	0.119364545	0.140158	0.851641	0.394413	0.497836
17093	LRRC50	13.54150663	0.119352117	0.287276	0.415462	0.677804	0.777231
14191	USP15	886.7560232	0.119304704	0.094691	1.259936	0.207693	0.286871

16950	PLA1A	1439.033291	0.119286798	0.264817	0.45045	0.652386	0.75442
15528	ARL6IP5	4686.646461	0.119002448	0.139708	0.851797	0.394327	0.497759
15242	SUV39H2	126.6403154	0.118689827	0.126907	0.935252	0.349658	0.449656
17950	ARMC4	3.859104615	0.118581562	0.546958	0.216802	0.828363	0.904503
16745	GCAT	169.2693946	0.118575317	0.234347	0.505982	0.612869	0.717399
15473	GPR180	362.5828169	0.11846781	0.136344	0.868888	0.384909	0.487597
14140	PPWD1	436.3008103	0.118269239	0.092754	1.27509	0.202277	0.280399
14568	SLU7	1184.537579	0.118225063	0.103169	1.145936	0.251822	0.338824
16279	BMS1P4	23.31334714	0.118191916	0.186633	0.633285	0.526547	0.63392
12833	CLP1	222.474746	0.118144889	0.069709	1.694826	0.090108	0.137631
14143	GON4L	878.3453243	0.118067157	0.092694	1.273737	0.202757	0.281004
15221	IRF2BP1	468.2318173	0.117971281	0.125175	0.942448	0.345963	0.445518
14577	SLC38A9	189.1504189	0.117967431	0.10324	1.142653	0.253183	0.340442
14324	RSAD1	758.4682006	0.117574836	0.096102	1.223439	0.221164	0.302641
13747	CHERP	986.1853815	0.117541698	0.083786	1.40288	0.160653	0.229065
14672	ZCCHC9	346.260559	0.117539421	0.105896	1.10995	0.267021	0.356725
13966	FAM193A	663.2061439	0.117536797	0.088391	1.329732	0.183607	0.25767
15357	TPRKB	343.8066286	0.117389968	0.129947	0.903365	0.366332	0.467572
15034	TMEM126A	305.8805574	0.117381993	0.117833	0.996174	0.319165	0.416147
15021	ZNF195	408.2255642	0.117253825	0.117114	1.001194	0.316733	0.413307
16516	ZNF219	596.5120597	0.117091401	0.206272	0.567655	0.570269	0.676789
16317	SC4MOL	1323.598009	0.116965446	0.187439	0.624019	0.532615	0.63981
15000	RNF139	956.527113	0.116905454	0.116084	1.007072	0.3139	0.410184
13372	IWS1	938.0951379	0.116674508	0.076783	1.519542	0.128626	0.188543
14409	NOL8	613.502223	0.116623333	0.097611	1.194776	0.232174	0.315834
15738	C1orf56	160.0734933	0.116472856	0.146841	0.793192	0.427666	0.53264
14815	NHLRC3	866.4416252	0.11641816	0.109859	1.059703	0.28928	0.382711
15678	ASCC3	997.2545076	0.116408273	0.143114	0.813393	0.415993	0.520084
15804	CDK16	2116.039326	0.11637662	0.150301	0.774291	0.438759	0.544172
13370	VIPAR	466.9314751	0.116339221	0.076496	1.52086	0.128295	0.188086
14891	ATP5SL	849.1212302	0.116277068	0.111916	1.038963	0.298822	0.393314
13612	TARDBP	2884.763275	0.116071233	0.079939	1.452	0.146502	0.210959
13405	CSTF3	498.8355049	0.115878256	0.076691	1.510969	0.130796	0.191253
14887	C1orf107	626.6441283	0.115821317	0.111331	1.040331	0.298186	0.392608
14895	C20orf112	788.7637928	0.115738479	0.111481	1.038192	0.299181	0.393705
15557	TBL1XR1	3820.227855	0.115444736	0.136659	0.844766	0.398241	0.501763
15697	SURF1	810.5369858	0.115376357	0.142829	0.807794	0.419209	0.523471
14029	C7orf64	293.6908067	0.115086125	0.087755	1.311447	0.189707	0.265054
16006	ACER3	506.9729015	0.11502167	0.161797	0.710903	0.477145	0.584313
15146	RBAK	549.1878227	0.114953758	0.119605	0.961115	0.336494	0.43547
15799	ITPKB	921.2514452	0.114888252	0.148064	0.775936	0.437787	0.543142
15109	DNAJC18	236.8592408	0.114770284	0.117952	0.973028	0.33054	0.428811
16871	SFTPD	21.18954715	0.11464873	0.242537	0.472707	0.636422	0.739406
14314	EIF2C1	831.1731027	0.114549798	0.093412	1.226282	0.220093	0.301386
17079	CA12	23952.20625	0.114355739	0.273663	0.417871	0.676041	0.77587
15338	NUP155	448.0289117	0.114258802	0.125676	0.909157	0.363267	0.464233
16057	TLE1	781.4997842	0.114081955	0.16377	0.6966	0.486053	0.593332

17080	CLDN18	5.626256895	0.114033662	0.273029	0.417661	0.676195	0.776001
15053	PFAS	504.2910492	0.113881384	0.115092	0.989481	0.322428	0.419844
14530	DCTN1	3728.531558	0.113797032	0.098406	1.156407	0.247515	0.333898
15160	CLPTM1	3158.124145	0.113752996	0.118744	0.957967	0.338079	0.437117
16451	FZD7	319.9450932	0.113741846	0.193972	0.586383	0.557618	0.66437
13931	TRIM26	1233.159461	0.113578172	0.0847	1.340954	0.179936	0.25317
17034	C10orf68	7.634225596	0.113420348	0.262107	0.432725	0.665215	0.765372
15056	RAB2A	1134.053855	0.113413452	0.114763	0.988242	0.323034	0.420549
17144	SYT6	28.06636611	0.113407809	0.283272	0.400349	0.6889	0.78763
16608	TM6SF1	219.5145531	0.113380836	0.207101	0.547466	0.584058	0.689314
15358	RNF19A	2007.116059	0.113377285	0.125515	0.903297	0.366369	0.467586
14813	ZNF182	220.0551526	0.113349237	0.106844	1.060884	0.288743	0.382073
15626	PGAP3	795.3507499	0.113323704	0.136942	0.827528	0.407938	0.511711
17499	PDZD3	150.2321149	0.113306005	0.359685	0.315015	0.75275	0.843172
17354	NEU4	5.892984479	0.113251085	0.321988	0.351724	0.725045	0.818924
16011	SPRED1	1635.566936	0.113243779	0.159847	0.708449	0.478666	0.585993
14759	SURF6	532.9979077	0.113192957	0.104639	1.081746	0.279365	0.371017
13243	YTHDF1	1213.783252	0.113055963	0.072235	1.565119	0.117555	0.173993
17267	MRO	142.6852282	0.112982575	0.301679	0.374512	0.708023	0.803799
14954	TXNL1	1416.3183	0.112942398	0.110539	1.021744	0.306902	0.402273
18397	C20orf114	0.875520779	0.11280378	0.875218	0.128886	0.897447	0.956181
16207	LRRC27	232.3149502	0.112285023	0.171865	0.653334	0.513541	0.621084
16989	RBM44	7.806136736	0.112093102	0.253678	0.441872	0.658582	0.759836
15226	SSR1	4013.517065	0.111998149	0.119152	0.939957	0.347239	0.447014
16246	RAB38	217.3134223	0.111964395	0.1746	0.641263	0.521352	0.629017
16049	DNAH14	60.81591892	0.11192951	0.160419	0.697734	0.485344	0.592761
15791	ACTR2	6839.130018	0.111916799	0.143713	0.778751	0.436127	0.54134
17353	HIST1H2AM	5.400035444	0.111750815	0.3177	0.351749	0.725027	0.818924
15340	LOC28535[illegible]	59.95094244	0.111492196	0.122809	0.907848	0.363959	0.465056
15667	ZNF821	171.0566641	0.111480441	0.136379	0.81743	0.413683	0.517559
14991	ZFP64	343.6772809	0.111459945	0.110426	1.009364	0.3128	0.408991
17119	SLC25A18	31.80944992	0.111324158	0.27363	0.406842	0.684124	0.783335
16382	C1orf204	41.2048185	0.111244159	0.183877	0.604993	0.545184	0.65231
16619	UBE2NL	4.673185856	0.111166144	0.204444	0.543747	0.586615	0.691874
14841	TXNDC15	1812.219574	0.111159798	0.105445	1.054199	0.291792	0.385379
17201	CDH6	3664.105545	0.111097611	0.286856	0.387294	0.698539	0.796004
15652	ANKRD28	861.0964134	0.11093433	0.134925	0.82219	0.410969	0.514656
14902	WDR1	6374.273284	0.110887489	0.107081	1.035543	0.300415	0.395144
16406	TP53INP1	1602.888939	0.110838568	0.184707	0.600078	0.548454	0.655263
17601	PCP2	7.739910491	0.110809149	0.377659	0.29341	0.769208	0.856614
14476	IFT46	578.3229991	0.110747235	0.094221	1.175399	0.239835	0.324745
12989	THRAP3	2649.281837	0.110731389	0.067194	1.647947	0.099364	0.149944
18503	PROKR2	0.615254575	0.110684446	1.001975	0.110466	0.91204	0.966162
17078	RP1L1	5.54794929	0.110620966	0.264493	0.418238	0.675773	0.775608
14375	CSRP2BP	560.8371679	0.110471666	0.091671	1.205083	0.228171	0.311123
17858	NRXN1	2.382218181	0.110131456	0.467661	0.235494	0.813825	0.893444
14659	RPAP1	507.5544162	0.110024577	0.09881	1.113497	0.265495	0.355002

13795	DCTN5	1330.937243	0.10991884	0.079207	1.387748	0.165214	0.234749
16876	VEGFA	28892.11738	0.109819403	0.233196	0.470933	0.637689	0.740658
17527	C8orf80	40.46912824	0.109556385	0.355619	0.308073	0.758027	0.847726
15198	AHSA1	1365.53436	0.109555067	0.115589	0.947797	0.343233	0.442646
16865	TTC39A	1137.402164	0.109507631	0.230508	0.475071	0.634736	0.737709
15802	RUSC2	1034.628303	0.109502885	0.141191	0.775565	0.438006	0.543308
16602	ZNF185	546.660687	0.109491964	0.19963	0.548476	0.583365	0.688909
15346	CACYBP	1148.786304	0.109483792	0.120839	0.906034	0.364918	0.466099
17482	TUBB8	3.035796482	0.109456893	0.343514	0.318639	0.75	0.840908
16355	RAGE	153.2179761	0.109453696	0.178762	0.612288	0.540347	0.647591
17045	AGBL3	30.37354061	0.109379634	0.255425	0.428226	0.668486	0.76864
15820	DAP	3662.813446	0.10929382	0.141905	0.770193	0.441186	0.54663
16247	CROCCL1	299.1956073	0.1092396	0.170549	0.640517	0.521837	0.629486
16445	PAX6	84.67252531	0.109164265	0.185621	0.588103	0.556463	0.663222
15653	ORAOV1	206.587505	0.108983256	0.132714	0.821186	0.41154	0.515325
13393	NOL7	678.4678847	0.108823255	0.071936	1.512782	0.130335	0.190749
16898	WSCD1	649.3320825	0.108767231	0.233478	0.465856	0.641318	0.743943
15281	SKA2	624.6293994	0.108701527	0.117456	0.925463	0.354725	0.455007
14783	CXorf42	21.50890988	0.108694443	0.101261	1.073404	0.28309	0.375353
15028	PPP2R5D	1000.161323	0.108611434	0.108859	0.997725	0.318413	0.415306
17365	FOLR1	1032.147846	0.108511773	0.310422	0.349562	0.726668	0.820237
15511	SPCS2	902.7546066	0.108271867	0.126474	0.856082	0.391953	0.495304
14233	WBP11	1703.52917	0.108127379	0.086607	1.248487	0.211853	0.291754
16504	ESAM	2341.602318	0.108091747	0.188998	0.57192	0.567376	0.673845
16615	INE1	23.15918504	0.108068838	0.19841	0.544674	0.585978	0.691288
15398	SLC11A2	1282.992355	0.108061995	0.121323	0.890697	0.373092	0.47493
15669	CYB5R1	1351.534813	0.108045588	0.132385	0.816149	0.414415	0.518409
13676	CDK13	1083.376267	0.107895149	0.075532	1.428472	0.153156	0.21951
17061	RNF207	241.9515302	0.107546533	0.25366	0.423979	0.671581	0.771564
13700	ZNF410	848.4762928	0.107535105	0.075851	1.417713	0.156275	0.223598
14431	LARP1	5139.092151	0.10722367	0.09022	1.188469	0.234649	0.318713
14886	PIAS4	226.2161451	0.107081191	0.102899	1.040647	0.29804	0.392441
17220	BCL11A	53.85425426	0.106792851	0.277946	0.384222	0.700814	0.797715
16275	AFAP1L1	876.0334555	0.106683025	0.168385	0.633566	0.526364	0.633849
17318	NMNAT2	52.35629922	0.106576393	0.294092	0.362391	0.71706	0.811606
15445	UBE2E1	1099.452411	0.106212131	0.121393	0.874943	0.381605	0.484288
17271	TUBA8	121.758147	0.106046796	0.283359	0.374249	0.708219	0.803799
14621	MEAF6	1072.112179	0.105672865	0.093807	1.126488	0.259959	0.348502
16666	BARD1	41.56319577	0.105625319	0.199359	0.529824	0.596234	0.701193
16388	TMEM182	135.737426	0.105590123	0.174965	0.603494	0.54618	0.653279
15755	PNPO	1475.753098	0.105589195	0.133944	0.78831	0.430515	0.53561
15914	ATP5O	3576.552934	0.105527709	0.143046	0.737717	0.460686	0.567419
15478	LRRC37B	116.3722933	0.105505713	0.121672	0.867135	0.385868	0.488638
14296	PPP3CC	417.002186	0.105315046	0.085592	1.230437	0.218533	0.299627
15497	ITFG2	530.392666	0.105250041	0.122037	0.862444	0.388443	0.491313
16671	FLJ42709	76.98920254	0.105189318	0.199521	0.527209	0.598049	0.703158
17592	C9orf169	3.983715062	0.105186583	0.356023	0.295449	0.767651	0.855316

16572	C3orf58	829.7839057	0.105054186	0.189644	0.553955	0.579609	0.685508
16380	C12orf60	45.45719742	0.10494342	0.173333	0.605445	0.544883	0.65203
16085	IFRD1	873.5201451	0.104753484	0.152552	0.686675	0.492288	0.599896
16054	MEGF8	1880.241586	0.104688363	0.150253	0.696747	0.485961	0.59333
15116	TJAP1	543.4281141	0.104640192	0.107857	0.970177	0.331958	0.430452
16081	NEK3	202.2121982	0.104589226	0.152041	0.687903	0.491514	0.599102
14704	FNTA	1260.806945	0.104588694	0.095335	1.097071	0.272611	0.3634
16607	ARHGEF3:	10.88765409	0.104576599	0.191	0.547523	0.58402	0.68931
15677	:TTNBP2N	1094.416691	0.104561579	0.128459	0.813967	0.415664	0.519706
16727	CPD	4480.798838	0.104392168	0.203548	0.512862	0.608048	0.71248
15154	TMEM188	385.8141079	0.104344328	0.108837	0.958719	0.3377	0.436816
16630	CAMK1D	97.71756333	0.104238347	0.192674	0.541008	0.588502	0.69364
13995	NCBP2	1513.711651	0.104219401	0.078971	1.319714	0.186931	0.261825
15033	TIGD6	193.4149958	0.104152069	0.104556	0.996137	0.319184	0.416147
14434	C2CD3	480.197358	0.103934281	0.087472	1.188195	0.234757	0.318793
15232	MASTL	259.3968581	0.103917836	0.110759	0.938231	0.348126	0.447979
14915	PPP1R10	1808.66664	0.103904937	0.100696	1.031865	0.302135	0.397073
16650	SLC37A4	3943.184405	0.103732264	0.194132	0.534338	0.593108	0.698228
15361	TRIM25	951.3782986	0.103730907	0.11497	0.902247	0.366926	0.468206
16281	SLC35D1	912.6484369	0.103685415	0.163707	0.633361	0.526498	0.63392
17161	LHX4	7.847791019	0.103570459	0.261656	0.395826	0.692233	0.790657
14637	CCDC90B	725.5682236	0.103546262	0.092342	1.121331	0.262147	0.351043
15923	CDKL3	46.96420311	0.103250827	0.140412	0.735342	0.462131	0.568877
16176	ATP6V0A1	2248.13249	0.103191271	0.156161	0.6608	0.50874	0.616458
15118	C4orf43	329.2124912	0.103039066	0.106459	0.967873	0.333108	0.431886
16691	CDH5	2960.655893	0.103034017	0.197924	0.520574	0.602664	0.707693
15427	UBE2J1	2115.28795	0.103019437	0.116919	0.88112	0.378253	0.480594
15935	UBA6	1142.285731	0.103019251	0.140811	0.731613	0.464405	0.571245
15969	ZNF331	454.8945494	0.102995915	0.14248	0.722881	0.469753	0.576594
14502	POLB	203.9371693	0.102952809	0.088183	1.167496	0.24301	0.328454
15773	MTERFD1	332.5094972	0.102746198	0.13109	0.783783	0.433168	0.538295
14860	MLL5	1990.989618	0.102568339	0.097687	1.049968	0.293733	0.387447
17770	NRADDP	1.009652934	0.102548488	0.405881	0.252656	0.800534	0.88302
15903	DRAM1	2226.152178	0.102302928	0.137988	0.741388	0.458458	0.565038
16325	SLC12A7	3949.951564	0.102284052	0.164766	0.620785	0.534741	0.64205
15360	SR140	1989.205692	0.102115703	0.113152	0.902467	0.366809	0.468087
16274	ZFP2	67.68140693	0.101973666	0.160889	0.633814	0.526202	0.633777
15171	ANKHD1	2417.027837	0.101918524	0.106654	0.955595	0.339277	0.438299
15319	SLC7A6OS	167.1709579	0.101862799	0.111258	0.915556	0.3599	0.4605
14719	GABPB1	368.6580541	0.101811397	0.0932	1.092401	0.274657	0.36573
16423	CDK14	1008.99683	0.101680716	0.170333	0.596952	0.55054	0.656994
16072	TMEM143	254.5541572	0.10167257	0.147031	0.691504	0.489249	0.596675
15734	KANK2	2788.651975	0.101280683	0.127492	0.794406	0.426959	0.531894
14857	LYSMD1	213.4645168	0.101088781	0.096242	1.050359	0.293553	0.387262
17030	)C1001327	2.479065115	0.101059728	0.232897	0.433924	0.664344	0.764656
16328	KDELC2	2019.340201	0.10097326	0.162728	0.620504	0.534926	0.642154
16788	CD24	41629.35246	0.100701274	0.204415	0.492632	0.622273	0.726541

17930	KCNN1	57.63120136	0.100550729	0.451768	0.222571	0.823869	0.900616
15085	ELP2	1205.217971	0.100296179	0.102309	0.980327	0.326925	0.424797
15492	C7orf36	209.5656814	0.100179677	0.115931	0.864131	0.387516	0.490299
15510	LOC90110	218.3418291	0.100050145	0.116817	0.856469	0.391738	0.495065
14793	COPA	5755.053521	0.099917197	0.093417	1.069585	0.284806	0.377373
15633	EAF1	763.8213268	0.099762163	0.12073	0.826327	0.408618	0.512304
15744	PCYT1A	445.589288	0.099598476	0.125862	0.79133	0.428751	0.533854
15594	CDV3	3528.01409	0.099527892	0.118947	0.836739	0.402739	0.506226
14973	IKZF4	241.1937338	0.099168468	0.097682	1.015212	0.310005	0.405824
15148	RWDD2B	361.2661807	0.099092089	0.10316	0.960569	0.336769	0.435767
16945	PHLDA1	3932.415686	0.099008522	0.21955	0.450961	0.652018	0.754234
15380	PSMG2	930.5244181	0.098928637	0.110599	0.894478	0.371066	0.472904
15245	INPPL1	2203.022871	0.098872513	0.105797	0.934551	0.35002	0.450039
17403	HIST1H1C	1445.397952	0.098409972	0.289058	0.340451	0.733517	0.82616
15462	MBNL1	4240.294484	0.098361739	0.112802	0.871982	0.383218	0.485801
17371	PCDHAC1	15.78527764	0.09809598	0.281735	0.348186	0.727701	0.821119
14566	TRAPPC4	912.9132356	0.097938923	0.085455	1.146082	0.251761	0.338787
17049	GJA4	1022.697875	0.097893982	0.229083	0.42733	0.669139	0.769278
14399	OGFOD1	722.3316052	0.097838747	0.081677	1.197873	0.230967	0.314409
16114	ZBTB8A	161.7905943	0.097825168	0.144602	0.676514	0.498714	0.606634
14744	VPS29	1473.665728	0.097820814	0.090128	1.085356	0.277764	0.369265
17002	SGK196	20.68496692	0.097813157	0.222926	0.438771	0.660828	0.761845
15863	RNF31	755.9063935	0.097509105	0.12916	0.754947	0.450281	0.556386
15544	COG5	927.8962185	0.097286914	0.114698	0.848201	0.396326	0.499767
17416	EGLN3	11629.01652	0.097231457	0.288124	0.337463	0.735768	0.828034
15230	LRRC28	248.8272384	0.097211991	0.103536	0.938916	0.347774	0.447585
15953	ACSL4	1995.110193	0.097098962	0.133724	0.726113	0.467769	0.574735
16888	HSP90AB2	32.92769669	0.097094714	0.207604	0.467693	0.640004	0.742775
15994	NME6	138.7770704	0.097045681	0.135804	0.7146	0.474856	0.581959
14122	CCNK	723.0164781	0.097033805	0.075761	1.280786	0.200269	0.277968
16749	LOC38933	42.22549561	0.096976024	0.192009	0.50506	0.613516	0.717985
15899	TIMM44	651.58274	0.096969491	0.130561	0.742715	0.457654	0.564217
17779	CCL3L3	6.931991353	0.096913802	0.387769	0.249926	0.802644	0.8849
15749	TMEM203	745.3983872	0.096909296	0.122602	0.790437	0.429273	0.534267
17558	MS4A2	34.49910558	0.096860404	0.321675	0.301112	0.763329	0.852148
16110	HIATL2	19.27618136	0.096780826	0.142764	0.67791	0.497829	0.605707
16449	LITAF	5107.415788	0.096673068	0.164782	0.586674	0.557423	0.664237
16511	ZNF391	29.99478877	0.096610316	0.169894	0.568649	0.569594	0.676193
16014	B3GNT1	1194.524827	0.09653844	0.136446	0.707521	0.479243	0.586589
15073	ACAD9	928.5257724	0.096029323	0.09746	0.985318	0.324468	0.42194
16921	C2orf77	85.5581208	0.095978279	0.208907	0.459431	0.645925	0.748228
15813	RAD18	114.9919106	0.095962809	0.124162	0.772884	0.439591	0.544895
15943	PDLIM5	3612.89756	0.095949022	0.131863	0.727643	0.466832	0.573943
16141	ZNF788	116.5529073	0.095941966	0.143507	0.668555	0.50378	0.61177
16108	HFE	309.1940832	0.095896295	0.141435	0.678023	0.497757	0.605663
15229	NAT15	1510.055795	0.095792695	0.101975	0.939372	0.34754	0.447338
15405	ST7L	252.2508642	0.095711552	0.107596	0.889547	0.373709	0.4755

17539	ATP1A2	107.350749	0.095612102	0.312734	0.30573	0.75981	0.849139
15782	SLC26A11	334.4582106	0.095471983	0.122249	0.780961	0.434825	0.540047
14177	HNRNPF	3481.319033	0.095460263	0.075532	1.263831	0.206291	0.285216
16684	NBPF10	402.5435317	0.09506906	0.18187	0.522732	0.601161	0.706267
14985	SMAP1	756.7173374	0.094954411	0.093975	1.010421	0.312294	0.408514
16087	C19orf55	71.29555087	0.094933253	0.138463	0.685624	0.49295	0.600629
18291	C12orf56	33.73584135	0.094711986	0.651757	0.145318	0.88446	0.947805
17963	FAM3B	14.0243878	0.094692887	0.442361	0.214063	0.830498	0.906229
14274	EXOSC9	424.9925617	0.094638463	0.076577	1.235862	0.21651	0.297311
14677	XRCC5	5945.855258	0.094630593	0.085345	1.108797	0.267518	0.357267
16459	RBM6	1029.981741	0.094509375	0.161524	0.585111	0.558473	0.665084
15395	DUT	849.1958911	0.094323032	0.105792	0.891591	0.372612	0.474412
16027	HECA	921.6731583	0.094321705	0.134223	0.702722	0.482229	0.589729
16588	LOC153684	43.13771952	0.094293196	0.171151	0.550936	0.581678	0.687332
16259	TCEA1	1565.271226	0.094293066	0.147779	0.638068	0.523429	0.631019
17798	NOX5	9.209069472	0.094156756	0.382045	0.246455	0.80533	0.886898
17187	KIAA0802	569.1839485	0.094116722	0.241234	0.390146	0.696428	0.794245
15812	THUMPD2	196.7149224	0.093787179	0.121317	0.773077	0.439477	0.544788
14130	USP48	1077.807485	0.093523447	0.073105	1.279304	0.20079	0.278534
15604	FAM122C	27.35675961	0.093383611	0.111987	0.833877	0.40435	0.507925
15794	SCLT1	173.7654825	0.093311758	0.120066	0.777171	0.437058	0.542407
13725	RAB5B	4101.297894	0.093301678	0.066182	1.409765	0.158609	0.226513
15585	SBDSP1	544.6543232	0.093178461	0.111032	0.839203	0.401355	0.504753
15732	FLJ33630	414.5333792	0.09310574	0.117136	0.794853	0.426699	0.531604
16566	ST7OT1	35.61114062	0.092966203	0.167475	0.555105	0.578823	0.684867
17183	KLF5	304.4518833	0.092411671	0.236578	0.390618	0.696079	0.794032
16900	CELSR2	1279.026621	0.092307547	0.198298	0.4655	0.641574	0.744112
16245	GPR125	677.1106523	0.092093194	0.143487	0.641822	0.520989	0.628618
17055	SPDYE5	9.74869418	0.091974475	0.215733	0.426335	0.669864	0.769862
15972	USP20	517.4258798	0.091948433	0.127453	0.721432	0.470643	0.577578
16832	ZMAT3	256.051057	0.091732647	0.19005	0.482676	0.629326	0.732855
16544	AMOTL2	2201.391715	0.091639963	0.163298	0.561182	0.574674	0.680862
16310	BEND3	81.7687532	0.091539727	0.146364	0.625424	0.531693	0.638977
13854	SPOP	1084.832406	0.091468353	0.066891	1.367422	0.171493	0.242633
15864	DHX30	1182.695358	0.091460672	0.121268	0.754203	0.450727	0.556903
16822	ADAM9	4642.580427	0.091288261	0.188248	0.484936	0.627722	0.731416
15082	CNBP	6802.691321	0.091239539	0.092792	0.983267	0.325476	0.422998
18629	AGR3	1.23684732	0.091144353	0.994113	0.091684	0.926949	0.975314
17162	ANXA9	122.6642636	0.090808331	0.22953	0.395628	0.69238	0.790778
14483	CWC27	400.3203719	0.090758873	0.077362	1.173173	0.240726	0.325794
16908	SNHG7	568.6223523	0.090582298	0.195386	0.463608	0.642929	0.745347
15139	CSTF2	224.1551583	0.090531128	0.093994	0.963163	0.335466	0.434339
16616	ECH1	5166.178858	0.090332659	0.165899	0.544504	0.586095	0.691376
18070	LGR5	2.434050246	0.090303925	0.468982	0.192553	0.847309	0.919098
16068	PTENP1	301.0353516	0.090024383	0.130048	0.69224	0.488787	0.59626
17310	C6orf154	42.22644654	0.089828119	0.246358	0.364624	0.715392	0.810075
17437	PDE3A	280.261638	0.089363697	0.270666	0.330163	0.741277	0.833273

14926	SUPV3L1	513.8372663	0.089214325	0.086753	1.028377	0.303773	0.398918
17896	RAPSN	1.991065461	0.089035506	0.390115	0.228229	0.819468	0.897441
15955	PNO1	531.260838	0.088733911	0.122219	0.726022	0.467825	0.574757
15618	PAPSS1	1023.781691	0.088707846	0.106727	0.831164	0.405881	0.509391
16257	IPO13	937.0005316	0.088605169	0.138822	0.638265	0.523301	0.630903
16777	ARHGEF35	496.5769243	0.088441413	0.177979	0.496922	0.619244	0.723479
16589	RAB36	560.6585161	0.088435249	0.160557	0.550803	0.581769	0.687398
16732	FLJ40852	37.44260575	0.088204387	0.172829	0.510356	0.609802	0.714364
15964	LRPAP1	2784.324918	0.087992044	0.121587	0.723694	0.469253	0.576161
16911	SYT11	849.1118507	0.087942309	0.190196	0.462378	0.64381	0.74622
14506	HNRNPUL2	2838.474097	0.087852499	0.075423	1.164797	0.244101	0.329838
16439	JHDM1D	716.3796131	0.087838546	0.148476	0.591601	0.554117	0.660701
15676	C2CD2	769.1583706	0.087694403	0.107732	0.814007	0.415641	0.519706
15743	NUMA1	6244.473499	0.087692415	0.110836	0.791192	0.428832	0.533854
17894	MLPH	183.8728626	0.087631054	0.382918	0.228851	0.818985	0.897112
16136	DNAJB1	3172.881878	0.087620743	0.130625	0.670778	0.502362	0.610238
15692	TNPO2	1823.281694	0.087476384	0.108028	0.809756	0.41808	0.522228
16519	PAK4	1116.144341	0.08745916	0.154401	0.566442	0.571093	0.677644
15913	STOML2	1700.463294	0.087310229	0.118274	0.7382	0.460393	0.567093
15386	METTL2B	260.0384139	0.087230885	0.097586	0.893887	0.371382	0.473123
14804	ZNF747	286.3090397	0.087193769	0.081967	1.063766	0.287435	0.380573
18128	TEX11	72.25636614	0.086940679	0.487246	0.178433	0.858383	0.928131
18008	OC1001246	5.678874937	0.086904354	0.426387	0.203816	0.838498	0.912672
16149	ZNF137	62.0021352	0.086887191	0.13041	0.666262	0.505244	0.613244
16522	MPHOSPH9	235.0813486	0.08687927	0.153561	0.565763	0.571555	0.678068
15505	LTV1	413.9777909	0.086822748	0.100971	0.859874	0.389858	0.492848
16771	ZNF713	42.43696078	0.086799531	0.174207	0.498256	0.618304	0.722638
15211	C4orf14	509.8617369	0.086492454	0.091576	0.944484	0.344922	0.444461
14732	CNOT10	381.0863473	0.086470996	0.079392	1.089166	0.276081	0.367327
15067	ZNF473	217.5268646	0.086469921	0.087685	0.986147	0.324061	0.421578
15137	CYB5B	1734.575647	0.08633382	0.089596	0.96359	0.335251	0.434119
15158	KHNYN	1319.289301	0.086163501	0.089932	0.958093	0.338016	0.437092
14126	MESDC2	1626.294373	0.085998176	0.067208	1.279575	0.200695	0.278472
15790	KIAA0495	499.1618754	0.085977109	0.110385	0.778886	0.436047	0.541289
16796	TMEM53	236.0311576	0.085928464	0.175005	0.491005	0.623423	0.727537
16304	LOC147804	139.4026863	0.085805916	0.137034	0.626163	0.531208	0.638551
15934	PTGES3	6022.279133	0.085742745	0.117144	0.731942	0.464204	0.571035
17000	NAPRT1	758.4595352	0.085668595	0.194966	0.439403	0.66037	0.761407
16968	ZNF37B	360.2177804	0.085533775	0.191364	0.446968	0.654898	0.756522
17888	QRFP	0.879749735	0.085492909	0.372286	0.229643	0.818369	0.896733
17432	SUSD3	164.1180056	0.085423834	0.25803	0.331061	0.740598	0.832748
16969	ULK4	65.93611305	0.085365672	0.191303	0.446232	0.65543	0.757091
17576	CCDC13	17.11739558	0.085271489	0.285857	0.298301	0.765474	0.853667
15984	RBM5	1361.379936	0.085247282	0.118861	0.717204	0.473249	0.580339
18647	TRIM72	0.709023549	0.085234267	0.955094	0.089242	0.92889	0.976665
15142	ZNF584	176.2471904	0.084956359	0.08831	0.962029	0.336035	0.434995
15536	KIAA0226	683.7722964	0.084802942	0.099793	0.849785	0.395445	0.498913

16583	SPHK2	303.1516257	0.084694241	0.153529	0.551649	0.581189	0.686962
15748	USP40	1042.547816	0.084629981	0.107044	0.790606	0.429174	0.534178
15217	MBD4	832.4524741	0.084526914	0.089643	0.942929	0.345717	0.4453
15795	ZNF142	495.7470001	0.084316163	0.108503	0.777089	0.437106	0.542432
17921	FAM153B	32.8606708	0.084292938	0.37558	0.224434	0.82242	0.899517
15670	FUNDC1	382.7130328	0.084154154	0.103154	0.815814	0.414607	0.518582
18250	NLRP4	0.769980626	0.084024617	0.547806	0.153384	0.878095	0.943099
17689	TMEM132E	20.08888853	0.08391832	0.310033	0.270675	0.786641	0.871698
17038	SEMA3F	2141.08799	0.083875566	0.194346	0.431578	0.666048	0.766241
17954	AVPR2	26.9590366	0.083562518	0.386346	0.21629	0.828762	0.904806
14803	ASTE1	175.2028425	0.083515387	0.078493	1.063992	0.287332	0.380464
17968	KSR2	24.12531539	0.083455607	0.391334	0.213259	0.831125	0.906631
16399	MTRR	1129.019667	0.0834473	0.138741	0.601462	0.547532	0.654441
17820	RNF183	54.51735743	0.083363814	0.34419	0.242203	0.808623	0.88944
14645	AMMECR1L	711.6711904	0.083220677	0.074356	1.119212	0.26305	0.352068
18084	NLRP2	89.00868591	0.083212654	0.439833	0.189192	0.849943	0.921261
15788	C17orf48	147.866873	0.083004642	0.106458	0.779697	0.435569	0.540765
15856	TMED10	7502.703381	0.082891855	0.109601	0.756306	0.449466	0.555625
15784	ZSWIM1	230.4572661	0.082867389	0.10624	0.779998	0.435392	0.540682
16340	NAPA	2831.701893	0.082718264	0.134376	0.615574	0.538175	0.64558
15061	CBX1	1253.122877	0.082702643	0.083758	0.987405	0.323444	0.420944
15311	POLR3A	412.4884726	0.082337836	0.089603	0.918919	0.358138	0.458485
17433	PIK3AP1	1620.987339	0.082322951	0.248944	0.330689	0.740879	0.833017
17488	CLIP4	434.477039	0.082279469	0.259619	0.316924	0.751301	0.842048
14981	COPS5	1106.350601	0.082273315	0.081364	1.01118	0.31193	0.408127
14935	MED29	1987.523914	0.0821671	0.080177	1.024817	0.305449	0.400878
17112	MLF1	313.8941542	0.082119653	0.200839	0.408884	0.682625	0.781915
15983	ZNF333	192.6869842	0.081869037	0.114132	0.71732	0.473177	0.580288
18165	RNASE13	0.808805563	0.081802262	0.477234	0.171409	0.863902	0.932196
16991	DLC1	1440.864599	0.081590041	0.184778	0.441557	0.65881	0.76001
16055	DYRK2	723.8719168	0.081583763	0.117104	0.696677	0.486005	0.593332
13804	ZNF207	2593.119389	0.08141976	0.058739	1.386121	0.16571	0.2353
16377	L3MBTL3	223.2405939	0.081247307	0.133992	0.606359	0.544276	0.651423
17217	SORBS1	2156.711141	0.081230045	0.211028	0.384926	0.700292	0.79726
16770	BTN3A3	966.4309684	0.081209893	0.162943	0.498396	0.618205	0.722566
17165	BCAM	3908.072079	0.081165499	0.205507	0.394952	0.692878	0.791195
15826	ACTR1B	1565.788766	0.0811293	0.105788	0.766906	0.443138	0.548805
18364	FAM71F1	1.136118776	0.081049458	0.598209	0.135487	0.892227	0.952276
16434	SLCO3A1	749.7624124	0.080912874	0.136461	0.592937	0.553223	0.659835
14023	LSM14B	782.3491803	0.080873875	0.061605	1.312784	0.189256	0.264552
15971	CALM2	7455.251348	0.080830539	0.111903	0.722327	0.470093	0.576939
17270	SDHAP2	273.1147592	0.080777943	0.215864	0.374207	0.70825	0.803799
16109	KIAA0913	1645.117329	0.08067497	0.118987	0.678016	0.497762	0.605663
17900	COL6A6	2.019905411	0.080500668	0.353188	0.227926	0.819704	0.89755
16837	CXXC5	987.0587021	0.080469254	0.167384	0.480746	0.630697	0.734245
16415	THAP4	1144.427971	0.08026884	0.134015	0.598954	0.549204	0.655801
18445	OR14I1	0.663637171	0.080232255	0.661051	0.121371	0.903397	0.96008

17202	SPATA1	2.706129877	0.07974551	0.20609	0.386945	0.698797	0.796252
15055	RNASEN	1040.333994	0.079694891	0.08061	0.988645	0.322837	0.42032
16433	ZFP36L2	3695.720638	0.079692372	0.134143	0.594084	0.552456	0.65896
17097	KIF20B	214.1131149	0.079518436	0.191789	0.414615	0.678424	0.777785
16537	CHCHD3	1145.351961	0.079141977	0.140612	0.562838	0.573545	0.679812
15940	LRP10	3639.723585	0.078970329	0.108189	0.729927	0.465435	0.572333
16199	ZCCHC6	660.2169045	0.07893764	0.120601	0.654536	0.512766	0.620468
15532	TOP3A	368.4589623	0.078840503	0.092651	0.850937	0.394804	0.498233
17139	MYOM2	134.3614893	0.078821481	0.196473	0.401182	0.688286	0.787158
15968	RERE	3275.778022	0.078589524	0.108654	0.723302	0.469495	0.576313
18212	YIPF7	0.594352812	0.078496529	0.492559	0.159365	0.873382	0.939993
16043	USP36	1141.366557	0.078321697	0.112097	0.698695	0.484742	0.592274
16895	MYL5	169.0769059	0.07809058	0.167344	0.466648	0.640752	0.743378
17373	PRKAR2B	160.1998724	0.078021097	0.224415	0.347665	0.728092	0.821466
14874	FTSJD2	997.947776	0.077877564	0.074573	1.044314	0.29634	0.390518
17274	ASAP3	460.2094713	0.077730322	0.208253	0.373249	0.708963	0.804377
17706	CSMD2	33.77949903	0.077663661	0.292495	0.265521	0.790608	0.875174
15156	SEC11A	2452.6631	0.077513872	0.080866	0.958548	0.337786	0.436879
17543	CCDC141	28.12925574	0.077409978	0.254689	0.30394	0.761174	0.850469
17697	LRRC66	44.98855668	0.077406131	0.28923	0.267628	0.788985	0.873852
17166	WEE1	677.0688843	0.077324103	0.1958	0.394914	0.692906	0.791195
16962	PDE7A	845.7485005	0.077223226	0.17185	0.449363	0.65317	0.754814
16073	STK19	342.3890343	0.077135714	0.111583	0.691286	0.489386	0.596806
16361	ACVR1B	1787.171049	0.077119758	0.126257	0.610816	0.541322	0.648521
15548	NAP1L4	2449.739487	0.077042788	0.091014	0.84649	0.397279	0.500841
15515	STAU1	3591.926884	0.076973034	0.090055	0.854737	0.392697	0.496116
17364	CCRL2	156.3052506	0.076967523	0.220128	0.349649	0.726602	0.82021
16216	TIPIN	77.38195616	0.076885123	0.118265	0.650109	0.515622	0.623255
15663	EIF2AK4	1301.89414	0.076804597	0.093871	0.818197	0.413245	0.517143
16220	TRAPPC2P[illegible]	169.3729095	0.076646807	0.118042	0.649318	0.516133	0.623741
18080	MAL2	1171.98441	0.076578317	0.402187	0.190405	0.848992	0.920414
15769	FAM40A	451.3606986	0.076514015	0.097504	0.784723	0.432616	0.537745
16613	PMS2L2	43.88795004	0.076498231	0.140308	0.545218	0.585604	0.690938
15081	RTF1	1345.629063	0.076454908	0.077748	0.983369	0.325426	0.422961
16425	VSIG10	549.1748605	0.076446093	0.128056	0.596975	0.550524	0.656994
18134	LRRN4CL	108.1470481	0.076226254	0.428506	0.177888	0.858811	0.928286
16657	SMURF1	1118.706995	0.076082746	0.142876	0.53251	0.594373	0.699461
17596	TDRD10	25.96894399	0.076038657	0.258198	0.294498	0.768377	0.855931
15987	TNS3	5543.201598	0.076027646	0.106195	0.715928	0.474036	0.581196
16430	ZNF700	342.3895821	0.076001815	0.127709	0.595117	0.551765	0.658256
16367	ZNF530	53.94494036	0.075967068	0.124766	0.608874	0.542608	0.649823
16044	DCTN4	2099.467731	0.075817814	0.108583	0.698248	0.485022	0.592516
16128	ZNF48	167.3210657	0.075761159	0.112546	0.67316	0.500846	0.608698
17302	PCDH1	1305.603106	0.075757112	0.20661	0.366667	0.713867	0.808722
16197	SRPK2	719.5232412	0.075643388	0.115442	0.655251	0.512306	0.619974
17060	NRP1	9084.039237	0.075608153	0.178323	0.423995	0.67157	0.771564
17942	COL21A1	212.6250692	0.075086286	0.341807	0.219675	0.826125	0.902512

16585	PGK1	20858.326	0.075046898	0.136143	0.551236	0.581472	0.687213
17469	CIB2	142.6194667	0.075038879	0.232836	0.322282	0.747239	0.838436
15094	DCTD	1618.598589	0.074685206	0.076396	0.977607	0.328269	0.42626
16187	44089	3565.85974	0.074631873	0.113691	0.656445	0.511538	0.619427
17087	PLEKHA5	1370.568505	0.074619446	0.179394	0.415954	0.677444	0.77707
16206	LOC72967ɛ	493.3705738	0.074407508	0.11388	0.653385	0.513508	0.621083
17846	EGR1	8272.216803	0.07432764	0.314754	0.236145	0.81332	0.893272
17436	GRAMD2	37.51841131	0.074278055	0.224953	0.330193	0.741254	0.833273
17105	CNTF	12.21504474	0.074246347	0.180354	0.41167	0.680581	0.779893
15064	TEX261	2539.649574	0.074162588	0.075145	0.986924	0.32368	0.421166
17225	RRN3P1	68.12589069	0.074102953	0.193619	0.382726	0.701923	0.798749
16974	CCNB3	10.68972295	0.074018611	0.166515	0.444515	0.65667	0.758271
15878	KCTD21	233.3936585	0.073899344	0.098634	0.749226	0.453721	0.560108
16174	B4GALT4	685.9909546	0.073717994	0.111504	0.661124	0.508533	0.616244
17696	C1orf53	33.48235182	0.07329044	0.273324	0.268145	0.788588	0.873481
16314	DEF8	935.5430944	0.073246002	0.117267	0.624611	0.532227	0.639462
17029	DDTL	386.0384178	0.073120068	0.168517	0.433904	0.664358	0.764656
16570	HSPE1	1791.893305	0.072884741	0.13149	0.554298	0.579375	0.685355
16402	ZNF19	89.5192323	0.072840012	0.121274	0.600622	0.548092	0.65499
15656	NIF3L1	378.2703976	0.072827223	0.088776	0.820349	0.412017	0.515837
16755	PMS2L4	62.77330152	0.072648422	0.144222	0.503726	0.614454	0.718825
17064	PRX	172.7788303	0.072514751	0.171484	0.422865	0.672394	0.772362
16542	LXN	496.7654114	0.072468268	0.128931	0.562071	0.574068	0.680226
16098	PAICS	2229.829015	0.07216085	0.105813	0.681966	0.495261	0.603032
17700	GBP1	2478.119062	0.072081858	0.269943	0.267026	0.789449	0.874213
16409	TBPL1	207.9445919	0.071976488	0.120095	0.599332	0.548952	0.655738
18450	RAB3B	6.951631634	0.071967054	0.597629	0.120421	0.90415	0.960555
17655	SIM2	41.82959375	0.071763943	0.257777	0.278395	0.780709	0.866792
16454	MGC23284	41.29732929	0.071339606	0.121716	0.586117	0.557797	0.664481
17743	FAM19A5	95.98520178	0.071264642	0.277393	0.256908	0.79725	0.880735
18300	TMC2	0.717092673	0.071238421	0.495934	0.143645	0.885781	0.948754
16805	LOC65227ɛ	55.33844482	0.071211644	0.145658	0.488895	0.624916	0.72888
17184	PDE9A	549.4441812	0.071131407	0.182215	0.39037	0.696263	0.794195
16198	CCDC149	349.2014425	0.071104455	0.108636	0.654518	0.512778	0.620468
16189	RBBP7	2089.796322	0.070883649	0.108038	0.6561	0.51176	0.619619
15956	MAN2A2	1406.374522	0.070762299	0.097528	0.725558	0.46811	0.575045
16003	TAF7	2998.786016	0.070707398	0.099281	0.712194	0.476345	0.583443
16617	PSMA6	1993.980538	0.070250878	0.129028	0.544463	0.586123	0.691376
17116	COX7C	5317.019984	0.070065512	0.172021	0.407307	0.683782	0.783058
16337	C16orf91	171.3047423	0.069970561	0.113556	0.616174	0.537779	0.645184
16846	ZNF354A	400.9368247	0.069924825	0.145929	0.47917	0.631818	0.735155
18453	LIPN	0.778915304	0.069860889	0.586323	0.119151	0.905156	0.961468
17082	ATP13A3	4895.904151	0.06984756	0.16754	0.416902	0.67675	0.776547
16759	SLC44A2	3785.119804	0.069809892	0.139098	0.501875	0.615756	0.720197
18339	CXorf58	0.629153519	0.069658718	0.504599	0.138048	0.890203	0.951429
16210	C7orf25	348.5062415	0.069651763	0.106695	0.652813	0.513877	0.621366
18991	KC6	0.743367404	0.069642125	1.341133	0.051928	0.958586	0.989376

16379	R3HCC1	660.9823613	0.069311505	0.114387	0.605937	0.544556	0.651693
15040	CDKAL1	293.655747	0.069128968	0.069517	0.994422	0.320017	0.417065
18017	HDC	16.2571539	0.069062055	0.340254	0.202972	0.839157	0.912933
16944	LOC72979	21.34075295	0.068899598	0.152799	0.450918	0.652049	0.754234
15921	C19orf42	1362.09366	0.06888849	0.093631	0.735742	0.461888	0.568649
16851	TXN	2437.30409	0.068835786	0.144041	0.47789	0.632729	0.735987
16182	CCDC53	666.329257	0.06881266	0.104378	0.659262	0.509727	0.617425
14752	BRAP	584.0501992	0.06865344	0.063411	1.082682	0.27895	0.370641
16481	BCORL1	397.3346505	0.068541866	0.118709	0.577396	0.563672	0.67038
15877	PRDM2	744.5284974	0.068515609	0.091422	0.749445	0.453589	0.55998
17677	GBP3	1374.813931	0.068487026	0.25011	0.273828	0.784217	0.869523
16215	FOXO3B	709.8364859	0.068485445	0.105293	0.650428	0.515416	0.623045
17244	NBEAL2	797.6960699	0.068465445	0.181197	0.377852	0.705541	0.801978
17564	NT5C1B	2.113146774	0.068331727	0.228141	0.299515	0.764547	0.853216
17421	NFIL3	1256.694011	0.068322255	0.202716	0.337034	0.736091	0.828203
16656	ACSS2	1214.490385	0.068108704	0.127912	0.532464	0.594405	0.699461
16612	PMF1	844.5895695	0.06801571	0.124752	0.545207	0.585611	0.690938
16104	PAK2	3047.610132	0.067930347	0.099859	0.680264	0.496337	0.604118
18226	POU4F3	0.610955053	0.067857446	0.432508	0.156893	0.875329	0.941365
15702	CHD4	5003.382583	0.067718558	0.084045	0.805743	0.420391	0.524779
16581	C15orf38	299.3936385	0.067694387	0.122652	0.551924	0.581	0.686822
18385	TFAP2C	27.34968193	0.067647004	0.514499	0.131481	0.895395	0.954617
15827	NGRN	2439.937005	0.067570448	0.088108	0.766907	0.443137	0.548805
18533	TFAP2B	5.260054231	0.067486944	0.6517	0.103555	0.917522	0.970344
17791	RPS6KL1	112.4600061	0.067420981	0.272727	0.247211	0.804745	0.886568
16677	C2orf34	79.87471408	0.067368531	0.128032	0.526185	0.598759	0.703741
16569	KIF24	73.23511215	0.067223009	0.121246	0.554434	0.579282	0.685286
16901	GALM	1083.460213	0.067161759	0.144447	0.464957	0.641962	0.744518
15239	TMEM127	2938.340693	0.06705139	0.071625	0.936148	0.349197	0.449151
16702	ABL1	1930.186518	0.06699945	0.129313	0.518119	0.604375	0.709278
15910	KDM5B	1191.138896	0.066803828	0.090432	0.738717	0.460079	0.566814
15299	BECN1	1317.116785	0.066591806	0.072304	0.920993	0.357054	0.457456
16356	SMARCC1	1508.523901	0.066525147	0.108685	0.61209	0.540478	0.647708
17022	TENC1	3265.640571	0.066364478	0.152171	0.436119	0.66275	0.763241
16611	ARID3B	97.96836276	0.066309416	0.121235	0.546949	0.584414	0.689609
16696	VARS2	807.0185684	0.06626672	0.127458	0.51991	0.603126	0.708066
16535	IQGAP1	6512.881155	0.066253143	0.117636	0.563203	0.573297	0.679559
16835	ERF	1185.734344	0.066239034	0.137661	0.481177	0.630391	0.733965
16420	ORMDL1	794.0988397	0.065754384	0.109899	0.598315	0.54963	0.656108
16878	LOC40139	1074.761138	0.065738992	0.139801	0.470232	0.63819	0.741151
16928	LARP6	386.6627352	0.065631747	0.143747	0.456579	0.647973	0.750291
16271	SBDS	2588.014048	0.06560359	0.103384	0.634564	0.525713	0.633304
16676	C3orf39	345.5445189	0.065495217	0.124435	0.526343	0.59865	0.703654
17036	PELI1	1034.027517	0.06540687	0.151125	0.432801	0.665159	0.765372
16273	SLC35E3	284.2518224	0.065263524	0.102953	0.633916	0.526136	0.633749
18415	HERC2P4	2.226005356	0.06525788	0.518073	0.125963	0.899761	0.957629
17604	ADCY4	305.1804092	0.065035408	0.221902	0.293081	0.76946	0.856747

15926	TM9SF1	1754.583033	0.065019345	0.088473	0.734905	0.462397	0.569098
18146	FAM106A	5.405536201	0.064783908	0.367941	0.176071	0.860238	0.929333
18492	RNF222	0.392283891	0.064755465	0.573057	0.113	0.910031	0.96463
18457	MEP1B	0.33397506	0.064755458	0.549878	0.117763	0.906255	0.962427
18523	LY6G6C	0.38492398	0.064755429	0.615306	0.105241	0.916185	0.969496
18505	PRPS1L1	0.293585282	0.064755404	0.587079	0.110301	0.912171	0.966216
18549	HPYR1	0.350299921	0.064755386	0.636965	0.101662	0.919025	0.970766
18618	WDR87	0.327776486	0.064755323	0.684084	0.09466	0.924585	0.973402
18571	OR2AG2	0.27529025	0.064755322	0.651401	0.099409	0.920813	0.97193
18640	ODF3	0.234227347	0.064755202	0.723942	0.089448	0.928726	0.976607
18707	AJC25-GN(	0.259376216	0.064755096	0.808509	0.080092	0.936164	0.980903
18693	POU1F1	0.224310883	0.064755085	0.791409	0.081823	0.934788	0.979957
18681	HIST1H3J	0.197643508	0.064755074	0.787065	0.082274	0.934429	0.979957
18774	OC1001909	0.312850142	0.064755051	0.879966	0.073588	0.941338	0.982718
18781	PATE4	0.229953291	0.064754928	0.899197	0.072014	0.942591	0.983656
18815	ADH7	0.265743366	0.064754844	0.961032	0.067381	0.946279	0.985725
18924	RGSL1	0.23985609	0.064754721	1.012001	0.063987	0.948981	0.986718
18847	CYP2F1	0.173876449	0.064754363	1.132104	0.057198	0.954387	0.986718
18885	LOC494141	0.15654308	0.064754327	1.131686	0.057219	0.95437	0.986718
18982	CLEC1B	0.17558778	0.064754148	1.219496	0.053099	0.957653	0.988615
19172	SLCO6A1	0.296204444	0.064754126	1.344077	0.048177	0.961575	0.989949
18983	FAM19A4	0.159548724	0.064754112	1.216737	0.053219	0.957557	0.988615
18987	NLRP13	0.154653	0.064754103	1.220008	0.053077	0.957671	0.988615
18984	GOLGA6C	0.136643154	0.06475406	1.220254	0.053066	0.957679	0.988615
18988	TRIM42	0.135983613	0.064754058	1.220774	0.053043	0.957697	0.988615
19206	TTC9B	0.136512173	0.064753778	1.320567	0.049035	0.960892	0.989949
19077	LOC72960(	0.116843705	0.06475335	1.441295	0.044927	0.964165	0.989949
19014	C4orf45	0.097357963	0.06475275	1.594222	0.040617	0.967601	0.989949
19180	SNORA40	0.095209293	0.064752736	1.597995	0.040521	0.967678	0.989949
19151	PRG1	0.140985157	0.064752471	1.710787	0.03785	0.969808	0.989949
19214	IQCJ	0.099264667	0.064752141	1.750974	0.036981	0.9705	0.990048
19216	LCE1E	0.095297079	0.064752108	1.759074	0.03681	0.970636	0.990054
19215	C3orf74	0.094677133	0.064752102	1.760376	0.036783	0.970658	0.990054
19244	ACCSL	0.076753486	0.064750962	1.995255	0.032452	0.974111	0.992182
19255	SYNPR	0.114051817	0.064750473	2.14825	0.030141	0.975955	0.993674
19257	CYLC2	0.094351994	0.064750108	2.194047	0.029512	0.976456	0.993693
19263	SNORA47	0.080704191	0.064749801	2.226053	0.029087	0.976795	0.993934
19311	GNG13	0.080439771	0.06474806	2.533099	0.025561	0.979608	0.994878
19332	LOC15130(	0.076818065	0.064747966	2.548604	0.025405	0.979732	0.994878
19418	TPD52L3	0.076944883	0.064747956	2.550212	0.025389	0.979744	0.994878
19405	SPINK9	0.06120751	0.06474726	2.630607	0.024613	0.980364	0.994878
19357	OR2T33	0.060947769	0.064747249	2.632361	0.024597	0.980377	0.994878
19346	CRNA002(	0.059278917	0.064747175	2.643865	0.02449	0.980462	0.994878
19277	BARHL1	0.059131866	0.064747168	2.644898	0.02448	0.98047	0.994878
19359	OR2T4	0.057158218	0.064747076	2.659097	0.024349	0.980574	0.994878
19328	KRTAP10-1	0.097881889	0.064746329	2.839871	0.022799	0.981811	0.994878
19323	IFLTD1	0.076084946	0.064745587	2.91407	0.022218	0.982274	0.994878

19299	DNAJB8	0.057487762	0.064745342	2.91407	0.022218	0.982274	0.994878
19274	ATOH1	0.058668532	0.064745342	2.91407	0.022218	0.982274	0.994878
19329	KRTAP11-1	0.05879604	0.064745342	2.91407	0.022218	0.982274	0.994878
19363	OR51A4	0.06029921	0.064745342	2.91407	0.022218	0.982274	0.994878
19378	PPBPL2	0.056123375	0.064745342	2.91407	0.022218	0.982274	0.994878
19425	ZG16	0.059110593	0.064745342	2.91407	0.022218	0.982274	0.994878
19356	OR2A5	0.059753838	0.064745342	2.91407	0.022218	0.982274	0.994878
19297	DEFB119	0.061482993	0.064745342	2.91407	0.022218	0.982274	0.994878
19421	UBQLN3	0.056314946	0.064745342	2.91407	0.022218	0.982274	0.994878
19370	OR7G3	0.039049697	0.064745097	2.91407	0.022218	0.982274	0.994878
19283	C4orf35	0.037861806	0.064745097	2.91407	0.022218	0.982274	0.994878
19333	LOC15444	0.040467749	0.064745097	2.91407	0.022218	0.982274	0.994878
19358	OR2T34	0.038857302	0.064745097	2.91407	0.022218	0.982274	0.994878
19375	PMCHL1	0.03893523	0.064745097	2.91407	0.022218	0.982274	0.994878
19385	RIPPLY2	0.041170719	0.064745097	2.91407	0.022218	0.982274	0.994878
19290	CDY2B	0.041852471	0.064745097	2.91407	0.022218	0.982274	0.994878
19334	LOC28466	0.040721824	0.064745097	2.91407	0.022218	0.982274	0.994878
19371	OR8B2	0.041214837	0.064745097	2.91407	0.022218	0.982274	0.994878
19348	NKAIN3	0.039758009	0.064745097	2.91407	0.022218	0.982274	0.994878
19354	OR10Z1	0.037790855	0.064745097	2.91407	0.022218	0.982274	0.994878
19383	PRSS48	0.038560768	0.064745097	2.91407	0.022218	0.982274	0.994878
19294	CLRN2	0.040487804	0.064745097	2.91407	0.022218	0.982274	0.994878
19301	FAM47A	0.037885413	0.064745097	2.91407	0.022218	0.982274	0.994878
19302	FAM71B	0.03744315	0.064745097	2.91407	0.022218	0.982274	0.994878
19338	LY6G6E	0.038022829	0.064745097	2.91407	0.022218	0.982274	0.994878
19364	OR52I1	0.040540779	0.064745097	2.91407	0.022218	0.982274	0.994878
19365	OR52I2	0.040011141	0.064745097	2.91407	0.022218	0.982274	0.994878
19369	OR6V1	0.038805087	0.064745097	2.91407	0.022218	0.982274	0.994878
19401	SNORA62	0.038736079	0.064745097	2.91407	0.022218	0.982274	0.994878
19416	TMEM88B	0.038469198	0.064745097	2.91407	0.022218	0.982274	0.994878
19417	TMEM95	0.039280716	0.064745097	2.91407	0.022218	0.982274	0.994878
19897	LY6G6D	0.019129642	0.064744852	2.91407	0.022218	0.982274	NA
19607	AMELY	0.019112282	0.064744852	2.91407	0.022218	0.982274	NA
19745	GALNTL5	0.018724626	0.064744852	2.91407	0.022218	0.982274	NA
19942	OR10K1	0.018533056	0.064744852	2.91407	0.022218	0.982274	NA
20068	OR6F1	0.018533056	0.064744852	2.91407	0.022218	0.982274	NA
20441	SPRR2F	0.020267937	0.064744852	2.91407	0.022218	0.982274	NA
20448	TAAR2	0.018724626	0.064744852	2.91407	0.022218	0.982274	NA
20487	TTTY6B	0.018533056	0.064744852	2.91407	0.022218	0.982274	NA
19657	CLDN17	0.019467615	0.064744852	2.91407	0.022218	0.982274	NA
19802	KRTAP10-1	0.019467615	0.064744852	2.91407	0.022218	0.982274	NA
19835	KRTAP3-3	0.020229646	0.064744852	2.91407	0.022218	0.982274	NA
19922	NEUROG1	0.019467615	0.064744852	2.91407	0.022218	0.982274	NA
19935	OR10G7	0.020540666	0.064744852	2.91407	0.022218	0.982274	NA
19950	OR13C5	0.019467615	0.064744852	2.91407	0.022218	0.982274	NA
19995	OR4D5	0.019467615	0.064744852	2.91407	0.022218	0.982274	NA
20010	OR4X1	0.018747901	0.064744852	2.91407	0.022218	0.982274	NA

20083	OR7G2	0.019467615	0.064744852	2.91407	0.022218	0.982274	NA
20465	TRIM48	0.018747901	0.064744852	2.91407	0.022218	0.982274	NA
19361	OR4K17	0.020707435	0.064744852	2.91407	0.022218	0.982274	0.994878
19396	SNORA2A	0.021727849	0.064744852	2.91407	0.022218	0.982274	0.994878
19398	SNORA36A	0.025077593	0.064744852	2.91407	0.022218	0.982274	0.994878
19641	C5orf48	0.019314616	0.064744852	2.91407	0.022218	0.982274	NA
19663	CSHL1	0.018275686	0.064744852	2.91407	0.022218	0.982274	NA
19728	FLJ25363	0.018074854	0.064744852	2.91407	0.022218	0.982274	NA
19732	FOXR1	0.020150484	0.064744852	2.91407	0.022218	0.982274	NA
19783	IFNA16	0.018952867	0.064744852	2.91407	0.022218	0.982274	NA
19808	KRTAP10-9	0.020095221	0.064744852	2.91407	0.022218	0.982274	NA
19836	KRTAP4-12	0.0185229	0.064744852	2.91407	0.022218	0.982274	NA
19878	LOC284379	0.018358592	0.064744852	2.91407	0.022218	0.982274	NA
19913	MUC7	0.01955704	0.064744852	2.91407	0.022218	0.982274	NA
19964	OR2B3	0.018279723	0.064744852	2.91407	0.022218	0.982274	NA
19966	OR2G3	0.018279723	0.064744852	2.91407	0.022218	0.982274	NA
19985	OR4A16	0.018275686	0.064744852	2.91407	0.022218	0.982274	NA
19987	OR4B1	0.018358592	0.064744852	2.91407	0.022218	0.982274	NA
20011	OR51A2	0.019354002	0.064744852	2.91407	0.022218	0.982274	NA
20014	OR51L1	0.018453088	0.064744852	2.91407	0.022218	0.982274	NA
20015	OR51M1	0.0201571	0.064744852	2.91407	0.022218	0.982274	NA
20029	OR5AN1	0.0185229	0.064744852	2.91407	0.022218	0.982274	NA
20082	OR7G1	0.018817199	0.064744852	2.91407	0.022218	0.982274	NA
20087	OR8D1	0.020257652	0.064744852	2.91407	0.022218	0.982274	NA
20102	OR9Q2	0.018273809	0.064744852	2.91407	0.022218	0.982274	NA
20118	PRAMEF22	0.018279723	0.064744852	2.91407	0.022218	0.982274	NA
20146	RNASE8	0.019314616	0.064744852	2.91407	0.022218	0.982274	NA
20197	SNORA38	0.01955704	0.064744852	2.91407	0.022218	0.982274	NA
20452	TCP10	0.01960569	0.064744852	2.91407	0.022218	0.982274	NA
20453	TCP10L2	0.018434801	0.064744852	2.91407	0.022218	0.982274	NA
20462	TNP2	0.019354002	0.064744852	2.91407	0.022218	0.982274	NA
20000	OR4F6	0.019014523	0.064744852	2.91407	0.022218	0.982274	NA
20113	POTEH	0.019582082	0.064744852	2.91407	0.022218	0.982274	NA
19789	IGFL4	0.019515169	0.064744852	2.91407	0.022218	0.982274	NA
19929	OCM2	0.020068549	0.064744852	2.91407	0.022218	0.982274	NA
19956	OR14C36	0.019323599	0.064744852	2.91407	0.022218	0.982274	NA
20447	SYCP1	0.019192865	0.064744852	2.91407	0.022218	0.982274	NA
19839	KRTAP4-4	0.01906623	0.064744852	2.91407	0.022218	0.982274	NA
19915	CRNA0002	0.01906623	0.064744852	2.91407	0.022218	0.982274	NA
20070	OR6K3	0.019169338	0.064744852	2.91407	0.022218	0.982274	NA
20140	RBMY2FP	0.019225344	0.064744852	2.91407	0.022218	0.982274	NA
17222	GALNT10	936.0913212	0.064419259	0.16819	0.383014	0.70171	0.798595
16029	KBTBD2	1295.53197	0.064265579	0.091466	0.702617	0.482295	0.589772
16913	CCDC58	281.2028789	0.064193566	0.139205	0.461145	0.644695	0.747157
15623	TINF2	1013.162619	0.064060296	0.077304	0.828683	0.407284	0.510989
16785	APBB2	1711.089089	0.063691792	0.129021	0.493656	0.621549	0.725826
17456	EXD3	190.8547842	0.063444061	0.195364	0.324748	0.745372	0.836868

16040	ARL8A	1008.518554	0.063365019	0.090675	0.698814	0.484668	0.592255
16512	RFX5	897.0067271	0.063224091	0.111223	0.568447	0.569732	0.676315
18057	NANOG	1.214271009	0.063212239	0.325166	0.1944	0.845863	0.918089
16428	BTN2A1	771.1685682	0.063191859	0.10596	0.596375	0.550925	0.657334
16603	CARD8	915.3411868	0.06307106	0.115053	0.548192	0.58356	0.688933
17471	BSN	34.69579168	0.062944316	0.195397	0.322136	0.74735	0.838464
16179	PGBD3	86.81687685	0.062798346	0.095142	0.660051	0.509221	0.616926
16694	ZNF286A	234.4365996	0.062768495	0.120689	0.520083	0.603006	0.70801
18287	HECW1	38.7617158	0.062658383	0.429047	0.146041	0.883889	0.947401
16120	ZNF512	803.3407187	0.062521559	0.092575	0.675359	0.499448	0.6073
14611	SNAP29	901.1749064	0.062403237	0.05523	1.129872	0.25853	0.346824
16438	TAOK3	1095.238669	0.062390047	0.105407	0.591896	0.55392	0.660506
14666	CIAO1	2055.718689	0.061908042	0.05571	1.111248	0.266462	0.356124
16158	SLC9A8	610.0083153	0.061848598	0.093113	0.664229	0.506544	0.61448
17172	ZNF138	182.0073352	0.061670794	0.156719	0.393512	0.693941	0.7921
17066	TDRKH	137.905525	0.061594763	0.145968	0.421974	0.673044	0.772979
17938	ERRFI1	5676.099765	0.06149794	0.279183	0.220278	0.825655	0.902175
15931	RTCD1	740.6173108	0.061360432	0.083604	0.733946	0.462982	0.569638
16312	PRPF38B	1108.657142	0.061314726	0.098132	0.624821	0.532089	0.639374
18256	CREB3L1	349.301657	0.061220818	0.406331	0.150667	0.880238	0.94507
16690	ZCCHC7	400.8298069	0.061124173	0.117386	0.520709	0.602569	0.707667
15818	C16orf80	540.0820858	0.060998037	0.07915	0.770667	0.440904	0.54635
17043	WDR34	1039.074087	0.060963104	0.14224	0.428592	0.66822	0.768514
16499	POLR1D	1743.184138	0.060955774	0.106327	0.573288	0.56645	0.672949
16169	NOM1	415.9354823	0.060924109	0.091979	0.662368	0.507736	0.615507
15997	DHX8	777.9321297	0.06087514	0.085247	0.714102	0.475164	0.582215
16799	C20orf30	3378.641264	0.060801646	0.124079	0.490022	0.624118	0.728218
16335	OXA1L	2531.988092	0.060789615	0.098572	0.616701	0.537432	0.644885
16831	FAM22D	54.70230274	0.060737228	0.125707	0.483165	0.628979	0.732494
16276	ARFGAP2	1592.295201	0.06066893	0.095759	0.633561	0.526367	0.633849
16824	MAPK6	1178.49914	0.06055795	0.124955	0.484637	0.627934	0.731582
16529	SERBP1	5492.547102	0.0603874	0.10693	0.564738	0.572252	0.678608
16592	PHF11	606.214891	0.060345068	0.109777	0.549708	0.58252	0.688161
17899	MYH7B	20.43727628	0.060234877	0.264013	0.228151	0.819529	0.897457
17135	CCDC15	43.46090935	0.060026149	0.149244	0.402201	0.687536	0.786483
16480	ARMCX6	223.4307844	0.059956957	0.103809	0.577568	0.563556	0.670282
17504	C16orf86	59.48187701	0.05992167	0.190788	0.314075	0.753464	0.843682
17289	PEX6	665.5234878	0.059758153	0.161148	0.370828	0.710765	0.805814
18550	INSRR	8.9913831	0.059681104	0.587691	0.101552	0.919112	0.970766
17698	ZNF454	13.34428439	0.059256881	0.221444	0.267593	0.789012	0.873852
17461	FAM41C	11.64449204	0.059229083	0.182693	0.324201	0.745786	0.837222
16993	C11orf61	250.9385168	0.05920654	0.134148	0.441353	0.658957	0.760091
16251	NOLC1	1498.935152	0.05915617	0.092408	0.640164	0.522066	0.629685
17444	FAM101B	907.7664505	0.059018781	0.179866	0.328126	0.742816	0.834667
17703	DCXR	574.2764258	0.058950241	0.221305	0.266376	0.78995	0.874643
17643	GFOD1	52.09484476	0.058934154	0.209206	0.281704	0.778171	0.864531
15595	PPHLN1	899.1137667	0.058761287	0.070246	0.83651	0.402868	0.506323

16517	BLMH	963.1211945	0.058727025	0.103489	0.567468	0.570396	0.676898
17117	KLF13	2346.278976	0.058697287	0.144255	0.406899	0.684082	0.783335
18107	CPO	3.82158732	0.058563521	0.32246	0.181615	0.855885	0.926484
17388	GLB1	1560.933544	0.058349258	0.169438	0.344369	0.730569	0.82355
18262	FAM153A	100.8135528	0.058335455	0.389838	0.14964	0.881048	0.945545
17072	PELI3	264.6443501	0.058302343	0.138586	0.420694	0.673979	0.77382
16052	ZBTB7A	739.682442	0.058272112	0.083587	0.697139	0.485716	0.593105
17007	PRPF3	708.3075599	0.058270059	0.133046	0.437968	0.66141	0.762291
17088	SLC39A10	817.0483415	0.058259324	0.140038	0.416025	0.677392	0.77707
18703	AQP2	29.27950827	0.058120227	0.72104	0.080606	0.935755	0.980651
16346	SUGT1	635.9976773	0.05783973	0.09403	0.615117	0.538477	0.645705
17261	TRAPPC6A	341.158479	0.057836167	0.154128	0.375249	0.707475	0.803475
16531	FDXACB1	77.11802837	0.057707964	0.102332	0.563931	0.572801	0.679177
16711	FUCA2	2050.018699	0.057658694	0.111616	0.516583	0.605447	0.710153
17315	ADI1	3075.033707	0.057603085	0.158512	0.363399	0.716307	0.810877
17084	PMVK	1119.861936	0.057480446	0.138021	0.416461	0.677073	0.776826
16825	C8orf38	139.4069628	0.057469992	0.118637	0.484418	0.628089	0.731676
17205	NF2	1135.812413	0.057379392	0.148442	0.386545	0.699093	0.796467
18628	C1orf230	1.108133033	0.056980265	0.620517	0.091827	0.926835	0.975247
17701	TTC25	85.32044974	0.056772351	0.212633	0.266997	0.789472	0.874213
18670	OVOL2	2.612048925	0.056627181	0.676927	0.083653	0.933332	0.979874
16632	TP53BP2	889.9628573	0.056571331	0.104616	0.540752	0.588679	0.693764
18118	C21orf71	2.295126235	0.056527943	0.313634	0.180235	0.856968	0.927074
16742	OGG1	348.9133718	0.056503076	0.111014	0.508973	0.610771	0.715072
17768	GPR44	6.090967662	0.055962668	0.221072	0.253143	0.800158	0.882705
17485	TMEM8A	1612.886319	0.055918818	0.175824	0.318038	0.750456	0.841275
16488	NUP98	2069.034148	0.055902305	0.097119	0.575606	0.564881	0.671533
16992	NPHP3	493.3085352	0.055580437	0.125895	0.441483	0.658863	0.760027
17797	TOX2	160.673468	0.05533366	0.224408	0.246576	0.805236	0.886859
18489	PTX4	0.639669892	0.055296164	0.488388	0.113222	0.909855	0.964577
17962	TTC18	90.17191838	0.055195229	0.257067	0.214711	0.829993	0.905728
17419	NDUFB9	2581.325389	0.05507202	0.163253	0.337342	0.735859	0.828037
16874	CEBPG	1023.047847	0.054934287	0.116392	0.471975	0.636945	0.739881
16349	NR2C1	404.9431601	0.054832063	0.089275	0.614193	0.539088	0.646319
16815	STK40	1283.294432	0.054774503	0.112424	0.487212	0.626108	0.729845
17132	MPI	1360.864714	0.054770709	0.135764	0.403426	0.686635	0.78559
17516	LOC44245	227.467901	0.054734912	0.17549	0.311897	0.755119	0.844991
16670	PSMA3	1183.764302	0.05472046	0.103764	0.527356	0.597946	0.70308
18328	CHRNA7	7.337230992	0.054712799	0.391488	0.139756	0.888853	0.950589
17810	KIAA0895L	541.5963912	0.054354456	0.222416	0.244382	0.806935	0.888082
18636	P2RX3	1.373972407	0.054242727	0.600364	0.09035	0.928009	0.976045
16653	EPRS	2765.920727	0.054128368	0.101495	0.533312	0.593818	0.698939
18041	ANKRD53	22.73145285	0.054072597	0.27346	0.197735	0.843252	0.916041
17092	FTO	2246.537674	0.054005957	0.129968	0.415533	0.677752	0.777231
16001	RNF41	911.4458817	0.053928361	0.075658	0.712793	0.475974	0.583061
16321	NFATC3	722.8610049	0.053865548	0.086593	0.622056	0.533905	0.641203
16240	RBM14	812.3144638	0.05364037	0.083346	0.643589	0.519842	0.627428

16418	PUS3	206.642398	0.053625173	0.089603	0.598478	0.549521	0.656058
18111	SLC22A20	2.878679698	0.053444179	0.294557	0.181439	0.856023	0.92649
17019	C4orf10	334.9505503	0.053361958	0.122397	0.435973	0.662856	0.763241
16782	SLC23A2	1123.544797	0.053355281	0.107576	0.495978	0.61991	0.724041
17106	TBC1D17	861.2427228	0.053247941	0.129541	0.411051	0.681035	0.780368
17292	SCAP	1163.075614	0.053219068	0.144261	0.368908	0.712196	0.807296
17694	HCN3	126.0582589	0.053215969	0.197854	0.268966	0.787956	0.872954
16610	RBM9	1916.081569	0.053146602	0.097151	0.547052	0.584343	0.689567
18099	RASGRF1	15.15137577	0.053097121	0.288501	0.184045	0.853978	0.924848
17133	PGAM5	317.2098402	0.05304797	0.131743	0.402663	0.687196	0.786186
17441	TRIM66	303.031458	0.053043897	0.161285	0.328883	0.742244	0.834168
16371	SEC22A	341.7199066	0.052899094	0.087013	0.607946	0.543223	0.650425
16953	IDS	4778.887811	0.052888239	0.117466	0.450244	0.652534	0.754458
16620	BAT5	776.7014194	0.05283959	0.097337	0.542854	0.587231	0.692558
16970	CAPRIN2	355.1450716	0.052656103	0.118131	0.445743	0.655783	0.757455
17027	UGP2	2745.200957	0.052586143	0.121098	0.434246	0.66411	0.764504
17538	ROGDI	498.4920535	0.052499948	0.171668	0.305822	0.75974	0.849108
17720	DZIP1	849.7164442	0.052435164	0.200033	0.262133	0.793219	0.877341
16255	NFRKB	537.7535329	0.052396993	0.08194	0.639454	0.522528	0.630106
17860	C10orf111	3.475599276	0.052394155	0.223004	0.234947	0.81425	0.893523
17276	TACO1	540.4736727	0.052377784	0.14033	0.373247	0.708965	0.804377
17556	C9orf37	141.3630857	0.052153811	0.172991	0.301482	0.763047	0.85193
17785	TCP10L	15.28893464	0.05191322	0.208938	0.248462	0.803777	0.885849
17231	PSMG1	365.2924553	0.051892538	0.136114	0.381243	0.703023	0.799718
18452	C2CD4A	21.36707981	0.051876727	0.433609	0.119639	0.904769	0.961108
16957	DYNC1H1	7265.438224	0.051828472	0.115248	0.449714	0.652917	0.754655
17154	ATXN1	999.9685376	0.051619781	0.129972	0.397162	0.691248	0.789828
16144	TAF5L	479.0219873	0.051521083	0.077214	0.66725	0.504612	0.612667
17952	TMEM98	942.2361495	0.051449711	0.237437	0.216688	0.828451	0.90455
16151	CCT2	2627.691858	0.051383393	0.077135	0.666146	0.505318	0.613258
16508	RABL2B	340.040236	0.051163984	0.089799	0.56976	0.56884	0.67542
16741	VDAC2	1803.730052	0.051155361	0.100458	0.509221	0.610598	0.714911
17890	YPEL4	35.99726965	0.051024231	0.222334	0.229493	0.818486	0.896766
16723	PHACTR4	1129.531025	0.050769137	0.098756	0.514085	0.607192	0.711689
16845	CNOT8	1395.249409	0.050574889	0.10555	0.479158	0.631826	0.735155
17949	TCN2	4291.993452	0.050391402	0.231628	0.217553	0.827777	0.903965
18002	PLA2G6	312.042432	0.050391016	0.24481	0.205838	0.836918	0.911256
17933	ADAMTS1[illegible]	31.01129625	0.0502777	0.226196	0.222275	0.8241	0.900752
17521	PNRC1	3856.286017	0.050270358	0.161851	0.310597	0.756107	0.845868
17784	TBX2	1176.257953	0.05007154	0.201505	0.248488	0.803757	0.885849
17268	NISCH	1333.832086	0.049929156	0.133406	0.374264	0.708208	0.803799
17550	[illegible]CRNA001[illegible]	8043.827114	0.049785805	0.164685	0.30231	0.762416	0.851439
16767	WDR25	166.9885409	0.049613909	0.099391	0.499181	0.617652	0.722049
16552	RRAGA	2681.929377	0.049388227	0.088384	0.558792	0.576303	0.682463
17529	ATP6V0D1	2272.573535	0.04922567	0.159986	0.307687	0.75832	0.847957
16675	HINFP	318.6573486	0.049197124	0.093431	0.526559	0.5985	0.70352
16461	UBE2G2	1681.854935	0.04913663	0.08416	0.583846	0.559324	0.666017

16547	SFRS2	2701.456976	0.048985548	0.087594	0.559232	0.576004	0.68234
17026	SYF2	1221.785752	0.04896881	0.11272	0.43443	0.663977	0.764396
16857	UBE2E3	1266.898175	0.048843973	0.102378	0.477097	0.633293	0.736408
17551	NUDT16L1	632.7608374	0.048333943	0.159895	0.302286	0.762434	0.851439
17625	FGFRL1	868.7027113	0.048139774	0.167369	0.287626	0.773633	0.860368
16860	MYL12B	7109.869105	0.048135535	0.101137	0.475942	0.634115	0.737206
16998	SERF1A	410.0979926	0.048125763	0.109431	0.439781	0.660096	0.76118
16269	IPO9	1591.906567	0.047824992	0.075365	0.634577	0.525705	0.633304
18225	GNMT	4.694798416	0.047530906	0.302412	0.157173	0.875109	0.94118
17068	YPEL1	199.5444206	0.047443286	0.112466	0.421846	0.673137	0.773035
16648	PEX5	1121.476629	0.047362794	0.088459	0.535422	0.592358	0.69743
17985	BHLHE41	3167.089829	0.047272379	0.228068	0.207273	0.835796	0.910743
17917	PCDHB13	107.8736602	0.047055184	0.209132	0.225002	0.821977	0.899234
17140	ZNF496	291.6352468	0.047021576	0.117277	0.400945	0.68846	0.787311
16647	CHMP7	931.1704995	0.046790579	0.087389	0.535429	0.592354	0.69743
17062	MAPK14	1388.240252	0.04673821	0.110352	0.423536	0.671904	0.771891
17096	TFG	2348.373073	0.046686445	0.112526	0.414894	0.678219	0.777596
16961	MBD2	1513.343622	0.04654879	0.103594	0.449337	0.653189	0.754814
18806	FSTL5	1.011291093	0.046541736	0.678034	0.068642	0.945274	0.985142
17621	C17orf69	52.09157337	0.046495807	0.161331	0.288202	0.773192	0.86006
17057	POLR3E	535.7592669	0.046484346	0.109195	0.425701	0.670326	0.770303
17208	MRPL47	618.7538186	0.046404438	0.120255	0.385885	0.699582	0.796909
17889	SLC25A35	97.46763115	0.046304801	0.20159	0.229698	0.818326	0.896733
18108	FLJ13224	2.478290308	0.046248641	0.254739	0.181553	0.855933	0.926484
16964	TRIM5	640.4929847	0.046176157	0.10288	0.448836	0.65355	0.755142
16849	WTAP	1803.06684	0.046129845	0.096373	0.478662	0.632179	0.735435
17756	TRIM22	2021.192015	0.046032062	0.180286	0.255329	0.798469	0.881437
16523	RHOA	9252.568252	0.046015071	0.081364	0.565547	0.571701	0.678166
16856	PHF23	716.7254811	0.045838735	0.09608	0.477087	0.6333	0.736408
17067	WWOX	287.6446847	0.045832463	0.108616	0.421967	0.673049	0.772979
17935	FLJ43663	187.8406506	0.045669486	0.206675	0.220972	0.825114	0.90176
17517	MIOS	772.9622997	0.045374789	0.145451	0.311959	0.755071	0.844991
17278	PCTP	619.4074072	0.045272316	0.121365	0.373025	0.70913	0.804471
16986	VRK1	154.9675122	0.04511666	0.101905	0.442732	0.657959	0.759252
16329	OBFC1	615.7131072	0.045108268	0.072827	0.619385	0.535663	0.642998
17258	TRIP12	3190.954591	0.045064195	0.119988	0.375571	0.707236	0.803252
17708	CAPN10	300.022954	0.045063579	0.169806	0.265383	0.790714	0.875193
16010	C20orf11	1326.96939	0.044987588	0.063457	0.708946	0.478358	0.585652
17664	ZRANB3	60.89970351	0.044976686	0.162637	0.276546	0.782129	0.867895
18174	TPTE2P1	12.72425422	0.044949525	0.264723	0.169798	0.865169	0.93315
17151	POLG2	109.2465937	0.044921268	0.112869	0.397994	0.690635	0.789291
16626	RNF167	2010.79377	0.044919664	0.082831	0.542308	0.587607	0.692751
17052	C1orf66	400.8977449	0.044856497	0.105093	0.426828	0.669505	0.769579
16885	TDG	441.7803801	0.044610001	0.09518	0.468691	0.639291	0.742122
17760	FBXO44	617.5602416	0.044555668	0.175032	0.254557	0.799066	0.881897
16405	INRNPA2B	11337.3939	0.044516801	0.074181	0.600112	0.548432	0.655263
16841	CTDSP2	4720.675262	0.044470642	0.092703	0.479709	0.631434	0.734801

17295	ZNF768	1224.736257	0.044463584	0.120766	0.36818	0.712739	0.807771
16536	VPS45	666.4430444	0.044219565	0.078508	0.563252	0.573263	0.679559
17713	FAM13A	3416.951895	0.044136924	0.167307	0.263808	0.791928	0.876269
17008	ATF7	1539.014337	0.044089018	0.100698	0.437833	0.661507	0.762314
18194	PLN	466.335012	0.044045404	0.269364	0.163517	0.870112	0.9374
17297	RFX7	519.5723396	0.043984337	0.119558	0.36789	0.712955	0.807923
17235	YIPF3	2511.973956	0.043613642	0.114747	0.380085	0.703882	0.80051
16431	PITPNA	2047.267578	0.043507415	0.073148	0.594786	0.551986	0.65848
18302	DNASE1L2	9.042095831	0.043398482	0.302968	0.143244	0.886097	0.948989
18075	TGFB2	239.1806211	0.043335472	0.22546	0.192209	0.847579	0.919213
17800	C6orf108	783.7906097	0.043251634	0.175644	0.246246	0.805491	0.886941
16967	RNF26	949.6294396	0.043245861	0.096646	0.447466	0.654539	0.756151
16579	UBN1	1019.121251	0.043041269	0.077953	0.552143	0.58085	0.686727
17672	KCTD20	1498.141214	0.043004218	0.156316	0.275111	0.783231	0.868772
17167	TRIM24	534.0694115	0.043002568	0.108922	0.394803	0.692988	0.791243
16518	C16orf70	523.6797938	0.042952789	0.075777	0.566834	0.570827	0.677369
17959	JPH4	27.25414251	0.042708341	0.198702	0.214937	0.829817	0.905666
18796	WNT7A	0.714965708	0.042628571	0.609532	0.069937	0.944244	0.984684
18091	AKAP5	88.4895098	0.042560368	0.228111	0.186577	0.851992	0.923054
17348	RBPMS	2849.388265	0.042552722	0.120284	0.353768	0.723513	0.817476
17223	SDHAF1	208.6011834	0.042546596	0.111079	0.383031	0.701697	0.798595
17606	AACS	619.1719725	0.042489999	0.145098	0.292836	0.769647	0.856859
18437	PEG10	1070.650171	0.042457519	0.34838	0.121871	0.903001	0.960011
17156	PCGF3	1061.270266	0.042279666	0.106479	0.39707	0.691316	0.789839
17152	FAM134A	3881.495301	0.04221604	0.106136	0.397754	0.690811	0.789447
18459	PDLIM4	241.9388895	0.042068338	0.357493	0.117676	0.906324	0.962435
17759	LOC283070	291.5886399	0.042023719	0.164913	0.254824	0.798859	0.881718
17767	C17orf79	777.9329453	0.041983422	0.16583	0.253172	0.800136	0.882705
16708	MCM3AP	1693.221539	0.04195178	0.081168	0.516848	0.605262	0.709986
17766	FGFR1	1992.471032	0.041707149	0.164674	0.253271	0.800059	0.882695
16659	SENP3	746.7721259	0.041687674	0.078349	0.532076	0.594673	0.699693
16912	ALKBH5	2945.194004	0.041532092	0.089885	0.462059	0.644039	0.746441
17003	SEC22B	1664.015146	0.041076147	0.093646	0.438632	0.660928	0.761916
18276	NCR1	3.314271168	0.041047421	0.278341	0.147472	0.88276	0.946759
17159	SNAP23	1487.59444	0.040680554	0.102604	0.396482	0.691749	0.790196
18565	IGSF9	11.59933589	0.040644291	0.40644	0.100001	0.920344	0.97165
17480	URB1	699.2514957	0.040481938	0.126829	0.319185	0.749586	0.84054
17817	C12orf35	1229.655638	0.040449029	0.166684	0.242669	0.808262	0.889192
18031	LAYN	305.9946608	0.040285594	0.201779	0.199652	0.841752	0.915046
17673	HS1BP3	1503.14896	0.040181388	0.146153	0.274927	0.783373	0.868833
16982	DDX50	818.7062485	0.040149462	0.090497	0.443656	0.657291	0.75866
17018	SAR1A	2564.162846	0.04002797	0.091718	0.436422	0.66253	0.76309
17014	SUPT6H	2620.926593	0.039933413	0.091417	0.436826	0.662237	0.762931
17719	LYST	681.2819004	0.039810766	0.151532	0.262722	0.792765	0.876968
16946	YWHAH	4815.38463	0.039790979	0.088251	0.450886	0.652072	0.754234
17814	MXD1	613.3039928	0.039783058	0.163435	0.243418	0.807681	0.888682
17296	SPRYD3	1286.15709	0.039720651	0.107913	0.368081	0.712813	0.807808

18088	NEURL2	54.5918376	0.039575974	0.21062	0.187902	0.850954	0.922133
18207	C10orf105	13.52788913	0.03945378	0.246334	0.160164	0.872752	0.939474
17252	SCFD2	350.0422107	0.03931186	0.104266	0.377034	0.706149	0.802296
17862	)C1001295	48.84493999	0.03925132	0.166993	0.235047	0.814172	0.893523
16716	C4orf23	156.5715134	0.039154334	0.075974	0.515362	0.6063	0.71087
16999	L3MBTL2	821.6864656	0.039119837	0.089016	0.439469	0.660321	0.761395
17755	STK38L	787.0978642	0.039063056	0.152789	0.255666	0.798209	0.881199
18508	PSORS1C3	29.61338087	0.039001726	0.35601	0.109552	0.912764	0.966677
17513	XRCC4	160.2902971	0.038996884	0.124712	0.312696	0.754512	0.844469
17130	TXNDC11	1881.772156	0.038839646	0.096183	0.403809	0.686353	0.785359
17658	CCDC104	1293.754802	0.03883234	0.13984	0.277691	0.78125	0.867175
18018	COX6C	2325.803185	0.038524465	0.189917	0.202849	0.839253	0.912987
18170	ZNF389	10.009876	0.038436952	0.225137	0.170727	0.864438	0.932518
17366	CABIN1	1343.748486	0.03829819	0.109717	0.349064	0.727041	0.820611
17632	DMTF1	941.6962609	0.038293061	0.135022	0.283606	0.776712	0.863401
17393	DCAF13	727.7094361	0.038158069	0.111198	0.343156	0.731481	0.824341
18064	TFRC	2499.459654	0.038135943	0.197246	0.193342	0.846691	0.918733
18115	PPAPDC3	92.05848292	0.038135739	0.210637	0.181049	0.856329	0.926611
17479	44081	4027.224245	0.038100385	0.119365	0.319193	0.749581	0.84054
18322	MCF2L2	16.03906318	0.037891066	0.269686	0.140501	0.888264	0.950219
17845	CEP290	612.1533624	0.03778607	0.159908	0.236298	0.813201	0.893223
17326	ISG20L2	486.7774771	0.037611333	0.104191	0.360984	0.718111	0.812403
17739	LOC72397	9.784168065	0.037459819	0.145357	0.257709	0.796631	0.880201
18500	KRTAP5-9	3.94704582	0.037457666	0.334761	0.111894	0.910908	0.965119
17028	KIAA0892	1155.544581	0.037206084	0.085712	0.434082	0.664229	0.764597
18327	CRNA000	5.671295407	0.03718762	0.26587	0.139872	0.888761	0.950577
17192	CCDC90A	323.6991988	0.037171841	0.095601	0.388822	0.697408	0.79512
16975	FOXJ2	762.2751916	0.036721415	0.08263	0.444408	0.656747	0.758271
18334	THSD4	514.1895791	0.036369498	0.261609	0.139022	0.889432	0.950931
18538	BCL8	2.816822786	0.036207121	0.352677	0.102664	0.91823	0.970735
18518	C2CD4D	7.635599255	0.036052491	0.336447	0.107157	0.914665	0.968158
16772	C2orf44	221.0557416	0.035988976	0.072296	0.497801	0.618624	0.72297
17385	TMEM14C	2169.170289	0.03583104	0.103803	0.345183	0.729957	0.823001
17525	C6orf153	535.9536675	0.035730408	0.115524	0.309291	0.7571	0.846786
17544	LOC22171	342.9692549	0.035715312	0.11778	0.303237	0.76171	0.851019
16866	SLC4A1AF	698.4771033	0.035688537	0.075152	0.474882	0.634871	0.737822
18443	ENPP1	330.8743712	0.035684056	0.293603	0.121538	0.903265	0.96008
17425	COBRA1	1217.208125	0.035608707	0.106609	0.334014	0.738369	0.830575
17812	GSTM4	739.393949	0.03548462	0.145623	0.243675	0.807483	0.888535
16811	ASB3	285.5912228	0.03540798	0.072631	0.487507	0.625899	0.729711
16893	MLH1	558.4053983	0.035372797	0.075728	0.467105	0.640425	0.743087
17329	NBAS	1282.928281	0.035301511	0.097992	0.360248	0.718661	0.812798
17476	C18orf10	1026.806026	0.035209998	0.110262	0.31933	0.749476	0.84054
17032	POLE3	1158.636137	0.03517112	0.081223	0.433017	0.665002	0.765307
18297	PCDHAC2	63.34149613	0.035144976	0.243448	0.144363	0.885214	0.948301
17580	LRSAM1	466.0556082	0.035128042	0.118206	0.297177	0.766332	0.854381
17786	WBP5	1497.985224	0.035098299	0.141489	0.248064	0.804085	0.886139

17653	MINK1	1657.33337	0.035089514	0.125969	0.278557	0.780584	0.866722
17455	CREB3	1247.112941	0.034947391	0.1075	0.325093	0.745111	0.836759
18591	HIST1H2AC	4.337029784	0.034620801	0.358467	0.09658	0.92306	0.972284
17101	CCT8	3502.06672	0.034616128	0.083901	0.412581	0.679914	0.779311
17683	NDUFB4	2228.011051	0.034609641	0.127309	0.271855	0.785734	0.87091
17219	TMEM183A	873.2558138	0.034455044	0.08962	0.384456	0.70064	0.797564
17796	GNS	9959.005507	0.034329342	0.139224	0.246577	0.805236	0.886859
17929	TOP3B	274.6544932	0.03424707	0.153603	0.222958	0.823568	0.900372
18386	OC1001309	149.4068678	0.03418265	0.260336	0.131302	0.895537	0.954716
17509	LSM3	615.8916055	0.034162202	0.108977	0.31348	0.753916	0.843995
17176	CCDC52	300.0422889	0.033909537	0.086338	0.392752	0.694503	0.792599
18073	ARHGAP39	102.5715987	0.033852291	0.176174	0.192152	0.847623	0.919213
17291	C7orf44	707.4036221	0.03378524	0.091324	0.369948	0.711422	0.806464
18171	EPOR	273.4604523	0.033749261	0.198448	0.170066	0.864958	0.933028
18284	GLB1L2	792.2315951	0.033737806	0.230424	0.146416	0.883593	0.947238
18243	C17orf28	255.6176495	0.033708427	0.217322	0.155109	0.876736	0.942
17514	NUPL2	442.4534121	0.033614777	0.10767	0.312203	0.754886	0.84484
17769	LOC151162	1441.794066	0.033578246	0.132863	0.252729	0.800478	0.883008
17841	UBAC1	700.083209	0.033420883	0.141036	0.236968	0.812682	0.89287
17323	CCDC115	1070.02992	0.033419054	0.092417	0.361613	0.717641	0.812012
17804	NUDT2	256.0637202	0.033344798	0.135657	0.245802	0.805836	0.887171
17381	GART	929.5598551	0.03325633	0.096168	0.345815	0.729482	0.822687
18499	ZNF404	58.03870634	0.033034267	0.294932	0.112007	0.910818	0.965076
18214	MID1	973.8568634	0.032748319	0.205759	0.159159	0.873544	0.940064
17098	MAPRE1	2828.771307	0.032734672	0.079007	0.414324	0.678637	0.777984
16907	CASC3	1836.74388	0.032655313	0.07044	0.463588	0.642943	0.745347
17727	ZNF217	1428.871372	0.032614169	0.124925	0.26107	0.794039	0.87798
17309	EDC3	591.9667042	0.032568871	0.089201	0.365118	0.715024	0.809705
17245	C2orf49	118.6921314	0.032529377	0.086136	0.377651	0.70569	0.802101
18650	ABCC11	3.58978294	0.032443696	0.366994	0.088404	0.929556	0.976956
17555	C12orf11	658.1726054	0.03244339	0.107538	0.301692	0.762887	0.851799
18383	ZSCAN5B	1.915606971	0.032354027	0.245526	0.131775	0.895163	0.954473
17642	EIF3L	6595.252817	0.03233298	0.11474	0.281795	0.778101	0.864503
18303	ATP6V0E2	914.43198	0.032305611	0.225701	0.143134	0.886184	0.94903
17646	ASNA1	1573.033554	0.032295113	0.115147	0.280469	0.779117	0.865436
17865	GNG10	723.1879966	0.032242176	0.137561	0.234385	0.814686	0.893794
17443	LSM1	460.6274347	0.032175603	0.097939	0.328527	0.742513	0.834381
17063	C2orf42	206.1745783	0.032165377	0.075983	0.423322	0.67206	0.772024
18425	NHLH1	5.309911493	0.032066436	0.257404	0.124576	0.900859	0.958357
17009	FAF1	859.6190897	0.031994858	0.073072	0.437851	0.661494	0.762314
17234	VCP	5975.671981	0.031623364	0.083154	0.380297	0.703725	0.800378
18836	C4orf50	1.59263395	0.031609981	0.52565	0.060135	0.952048	0.986718
17907	HEMK1	338.5739006	0.031554681	0.139292	0.226537	0.820784	0.89843
17282	CRTC3	1271.467169	0.031554409	0.084797	0.372118	0.709805	0.805083
17506	TARS	1722.412859	0.031376861	0.09993	0.31399	0.753529	0.843706
17919	POT1	736.6338605	0.031321482	0.139397	0.224692	0.822219	0.899398
17168	NSUN2	1195.412237	0.031124749	0.078911	0.39443	0.693264	0.791505

18000	EEF2K	982.2375357	0.03108285	0.150962	0.205898	0.836871	0.911256
18426	LY6G5B	46.0401136	0.030988993	0.249068	0.12442	0.900983	0.958437
17494	AGK	637.8984984	0.030892747	0.09772	0.316136	0.7519	0.842459
17721	PPPDE1	1063.132804	0.030560966	0.116547	0.26222	0.793152	0.877341
17883	XPO5	941.8860705	0.030461532	0.132245	0.230341	0.817827	0.896441
18095	BCAT2	712.5895915	0.030378351	0.164088	0.185135	0.853123	0.924127
18333	KLRD1	66.29263869	0.030375279	0.218553	0.138984	0.889463	0.950931
17805	PDE4A	685.5726687	0.030317147	0.123502	0.245479	0.806086	0.887396
18268	NR1D1	820.6058983	0.030284247	0.203036	0.149157	0.88143	0.945747
17801	TFIP11	961.5854027	0.030115356	0.122297	0.246248	0.80549	0.886941
17680	JARID2	381.2804622	0.029872827	0.109277	0.273369	0.78457	0.869816
18376	FIGN	128.3989321	0.029285157	0.220222	0.13298	0.894209	0.953769
17994	ZNF792	121.5214847	0.028804252	0.139384	0.206654	0.83628	0.910966
17744	GNPTAB	1608.164148	0.028717598	0.111813	0.256837	0.797305	0.880747
18414	HAAO	765.9530199	0.028535949	0.226496	0.125989	0.899741	0.957629
17970	TPP1	10053.11502	0.028463162	0.13352	0.213176	0.83119	0.906631
17926	BLOC1S2	812.1630891	0.028346016	0.127066	0.22308	0.823473	0.900318
18188	FLI1	865.0145372	0.028328096	0.17147	0.165207	0.868781	0.936313
17904	KIAA0556	475.0805991	0.028258644	0.124108	0.227695	0.819884	0.897595
17300	ZNF740	658.8526064	0.028166914	0.076778	0.366863	0.713721	0.808651
18985	LGALS4	175.2078998	0.027961891	0.52394	0.053369	0.957438	0.988615
17086	COPS3	895.6804196	0.027954669	0.067205	0.415962	0.677437	0.77707
17599	GLG1	6726.230594	0.027818556	0.094735	0.293646	0.769029	0.856511
17619	NOL6	845.5732641	0.027651457	0.095478	0.28961	0.772115	0.858972
18338	C6orf204	55.00742344	0.027420377	0.198403	0.138206	0.890078	0.951381
17771	CBX5	3668.336107	0.027413775	0.108561	0.25252	0.800639	0.883086
18139	C1orf89	72.24237805	0.027300333	0.154726	0.176443	0.859946	0.929206
17863	GMNN	224.5375801	0.027198254	0.115833	0.234805	0.81436	0.893544
18438	RAB15	361.9614608	0.026986748	0.221597	0.121783	0.903071	0.960033
18374	TNFRSF10A	118.7987022	0.026955871	0.202191	0.133319	0.893941	0.953638
18716	EPHA7	911.487844	0.026898172	0.35078	0.076681	0.938877	0.981752
18179	SDCBP	5086.93383	0.026799413	0.159682	0.167829	0.866717	0.934514
17678	PBX2	1366.49299	0.026688225	0.097446	0.273876	0.78418	0.869523
18398	C8orf39	8.019955445	0.026507214	0.205887	0.128746	0.897558	0.956248
17636	C1orf83	137.1204411	0.026371515	0.093385	0.282396	0.77764	0.864174
18695	SLCO4A1	568.6382187	0.026283128	0.31881	0.082441	0.934296	0.979957
18106	RPL23AP53	181.7251696	0.026192231	0.143902	0.182015	0.855571	0.926215
17415	SFRS7	1461.094184	0.026073456	0.077146	0.337975	0.735382	0.82769
17773	HSPA14	451.0338056	0.026054054	0.103406	0.251958	0.801074	0.883466
17795	ASB6	628.6776736	0.025966493	0.10514	0.246971	0.80493	0.886622
18172	TAF1D	777.9344684	0.025895062	0.152382	0.169935	0.865061	0.933088
18536	HIST2H2BE	62.34476591	0.025883933	0.250831	0.103193	0.91781	0.970543
18347	ITPRIP	1928.202099	0.025697513	0.18751	0.137046	0.890994	0.952033
18839	CHD5	4.122651688	0.025693186	0.417323	0.061567	0.950908	0.986718
19219	CLLU1OS	1.31429194	0.025510181	0.699831	0.036452	0.970922	0.990194
18040	AEN	816.9455667	0.025500103	0.128927	0.197787	0.843211	0.916041
18332	AFG3L1	231.9472217	0.025467167	0.182966	0.13919	0.8893	0.95086

17428	DNAJC8	1843.731789	0.025441224	0.07654	0.332393	0.739592	0.831808
18151	LOC64933(	9.55091171	0.025181586	0.144217	0.174609	0.861387	0.930199
17714	CRNA000	644.7262873	0.025145285	0.095329	0.263773	0.791955	0.876269
18026	C11orf74	237.9061617	0.025120793	0.125298	0.200489	0.841098	0.914466
18038	C6orf134	148.2881858	0.024987358	0.12569	0.198801	0.842419	0.91541
18049	BAT4	453.1681396	0.024815691	0.126052	0.196869	0.84393	0.916498
17958	BTF3L4	898.2825861	0.024741572	0.114906	0.21532	0.829518	0.905411
17877	MTF2	470.8565955	0.024678946	0.106816	0.231041	0.817283	0.896099
17873	AP2A2	2103.356539	0.024540448	0.105699	0.232173	0.816403	0.895335
18319	STBD1	867.9301164	0.024518547	0.174155	0.140785	0.888039	0.950134
17777	CDAN1	237.9129148	0.024495895	0.097941	0.25011	0.802503	0.884806
18065	C20orf29	251.5132056	0.024209157	0.125274	0.19325	0.846763	0.918755
18986	C1002407	1.93615423	0.024188256	0.454233	0.053251	0.957532	0.988615
18558	RPS15AP1	4.042969375	0.023911674	0.233775	0.102285	0.91853	0.970766
18278	UQCRB	4520.661407	0.023583834	0.160074	0.147331	0.882871	0.946791
18069	ZNF630	80.04308059	0.023566675	0.122233	0.192802	0.847114	0.918938
18154	PPRC1	756.8441254	0.023323887	0.134498	0.173414	0.862326	0.931059
18782	PRINS	4.418993226	0.02330578	0.323712	0.071996	0.942605	0.983656
18547	FCRLB	51.59983617	0.023253277	0.228229	0.101886	0.918847	0.970766
18066	CMAS	890.2196719	0.023160596	0.119881	0.193196	0.846805	0.918755
18994	RCOR2	15.24651783	0.023124143	0.451273	0.051242	0.959133	0.989784
18455	SPRY4	2135.17505	0.022686295	0.191483	0.118477	0.90569	0.96193
17615	HERPUD2	843.7244557	0.022616598	0.077949	0.290146	0.771705	0.858687
18337	ATPIF1	1942.817449	0.022567237	0.163243	0.138243	0.890048	0.951381
17679	PRPS1	922.4194435	0.022539268	0.082416	0.273481	0.784483	0.869769
17341	SUPT7L	1124.268349	0.022490308	0.062906	0.357523	0.7207	0.814627
18098	PGM3	417.4885147	0.022485467	0.122113	0.184137	0.853906	0.924821
18265	ZFAND2B	667.2538154	0.022482435	0.150385	0.1495	0.881159	0.945612
17957	EXOSC2	396.7521589	0.022427561	0.104018	0.215613	0.82929	0.905213
17850	GTF2H1	773.7723121	0.022363681	0.094883	0.235697	0.813668	0.893435
19035	FAM166A	0.946229719	0.022173071	0.562642	0.039409	0.968564	0.989949
17839	ZMYM4	1292.192283	0.022121749	0.093115	0.237574	0.812211	0.892435
17969	MOBKL2C	522.1265427	0.022029047	0.103313	0.213227	0.83115	0.906631
18348	KLHDC10	1160.48492	0.021988541	0.161178	0.136424	0.891486	0.952033
17912	C22orf28	1732.006262	0.021898809	0.096912	0.225967	0.821227	0.898664
18926	RNF212	90.91892742	0.021539612	0.375968	0.057291	0.954313	0.986718
18686	MYOZ1	18.75415929	0.021482623	0.259685	0.082726	0.93407	0.979957
17519	NFYC	969.4582601	0.021479866	0.069001	0.311299	0.755574	0.845368
17666	TOR1A	832.0142807	0.021301736	0.077088	0.276331	0.782294	0.867981
18857	FOXD1	84.20804847	0.021256681	0.331054	0.064209	0.948804	0.986718
18349	LUC7L3	2034.357745	0.021093626	0.154615	0.136426	0.891484	0.952033
17847	MED31	123.0304299	0.021081003	0.089302	0.236064	0.813383	0.893272
18405	IFIT3	1305.119583	0.021000483	0.165226	0.127101	0.89886	0.95727
18569	KCTD12	3725.8869	0.020923167	0.209864	0.099699	0.920584	0.971781
18665	LOC38970	3.551447135	0.020904757	0.24642	0.084834	0.932393	0.97915
18954	TSPAN11	144.2538504	0.020499197	0.31235	0.065629	0.947673	0.986718
18218	CCDC6	1691.392061	0.020083092	0.126918	0.158237	0.87427	0.940588

17838	BUD13	336.1119415	0.020077381	0.084488	0.237635	0.812164	0.892434
18434	ZDHHC14	191.9690557	0.020052999	0.162802	0.123174	0.901969	0.95907
18281	C14orf4	759.7403265	0.020029978	0.136443	0.146801	0.883289	0.947068
18798	AMY2B	313.7897235	0.019962755	0.291098	0.068577	0.945326	0.985142
18217	C8orf44	34.09598177	0.019817578	0.125144	0.158358	0.874175	0.940588
18304	NDUFS3	1500.982933	0.019758981	0.138479	0.142686	0.886538	0.949305
18054	SRCAP	2891.547269	0.019634996	0.100761	0.194866	0.845498	0.917943
18617	RCAN3	73.62986662	0.019560296	0.205855	0.09502	0.924299	0.973153
18010	ELMO2	913.3001512	0.019531457	0.095967	0.203523	0.838726	0.912703
18255	ANKRD10	1611.372088	0.019418087	0.128688	0.150893	0.88006	0.94495
18957	VWA2	4.282080126	0.01937378	0.332038	0.058348	0.953471	0.986718
18252	TGFBR1	2373.592426	0.019328132	0.12665	0.15261	0.878706	0.943673
18509	TEX264	1339.713718	0.01927505	0.176792	0.109027	0.913181	0.967058
18249	SERINC3	2674.879424	0.019260452	0.125339	0.153667	0.877872	0.942911
18980	IGSF1	28.22761768	0.019096967	0.3536	0.054007	0.956929	0.988239
18384	NDUFA12	1049.906994	0.018699645	0.141991	0.131696	0.895225	0.954487
18478	FRS3	98.37825836	0.018549678	0.160876	0.115304	0.908204	0.9634
18760	LOC44186	51.82815972	0.018496062	0.246517	0.07503	0.940191	0.982315
17826	GNPAT	1270.61276	0.018349664	0.076326	0.240412	0.810011	0.890617
18161	LRRC42	492.6540468	0.018259955	0.105877	0.172464	0.863073	0.931506
18960	YJEFN3	68.88985325	0.018174391	0.305659	0.05946	0.952586	0.986718
18006	BAT3	3633.545286	0.018012468	0.088014	0.204654	0.837842	0.912009
18358	ZNF512B	612.1697511	0.017820262	0.130179	0.136891	0.891117	0.952033
18201	CDK5RAP	953.3548072	0.017678893	0.109704	0.161152	0.871974	0.938942
18526	TRIM56	459.1665584	0.017459135	0.166831	0.104652	0.916652	0.969842
19025	DBC1	76.3426666	0.017307428	0.39519	0.043795	0.965068	0.989949
17851	SLC35E1	1949.534551	0.017091792	0.072528	0.235659	0.813697	0.893435
18340	GTF3C5	839.888944	0.017043308	0.12348	0.138025	0.890221	0.951429
18016	LRRFIP2	858.4740033	0.016832533	0.082869	0.203123	0.839039	0.912885
18159	SMG6	1061.510292	0.016830043	0.097433	0.172735	0.86286	0.931379
18020	CCNH	636.2605081	0.016819634	0.083235	0.202074	0.839859	0.913545
18253	MUS81	533.3387751	0.016790483	0.110463	0.152001	0.879186	0.944115
18484	KLHL36	373.704694	0.016339427	0.143753	0.113664	0.909505	0.964465
19254	SULT1E1	5.198161622	0.016210934	0.540639	0.029985	0.976079	0.993674
18203	URGCP	1138.268707	0.016208675	0.100568	0.161172	0.871958	0.938942
18776	TMEM26	157.0131327	0.016149815	0.219686	0.073513	0.941398	0.982718
18357	ZNF319	320.4447101	0.016147354	0.118124	0.136699	0.891269	0.952033
18701	ZNF320	1210.054725	0.016006395	0.194882	0.082134	0.93454	0.979957
18634	SH3BP5	1725.540218	0.015999161	0.17675	0.090519	0.927875	0.976027
19211	WEE2	1.091904041	0.01595894	0.328495	0.048582	0.961252	0.989949
18654	TRPM4	536.0278534	0.015918269	0.180399	0.088239	0.929686	0.977032
19051	HIST1H4E	3.681601502	0.01583235	0.36973	0.042821	0.965844	0.989949
19044	GLDN	56.20273372	0.01570531	0.320297	0.049034	0.960893	0.989949
19393	SNAP91	1.044761763	0.01569653	0.80361	0.019533	0.984416	0.994878
18974	LIPG	118.7302088	0.015270784	0.27649	0.055231	0.955955	0.987593
18652	ID2	3208.920785	0.015270352	0.173406	0.088061	0.929828	0.977032
18758	BMS1P5	106.3822181	0.015267113	0.203531	0.075011	0.940206	0.982315

18280	S100PBP	473.4911471	0.015239014	0.103702	0.14695	0.883171	0.946994
18371	CCDC71	279.8975612	0.014896956	0.111164	0.134008	0.893396	0.953212
18517	TCP11L1	354.6099245	0.014803393	0.137948	0.107311	0.914542	0.96808
19194	SYN2	9.305878278	0.0147723	0.397476	0.037165	0.970353	0.989949
18024	SMARCE1	2514.992852	0.014739106	0.073203	0.201347	0.840427	0.91396
18164	FAF2	1834.257648	0.014713659	0.085839	0.17141	0.863902	0.932196
18646	MAP2K6	88.20875706	0.014590355	0.164048	0.08894	0.92913	0.976665
18373	HCFC1	1421.584675	0.014560103	0.109001	0.133578	0.893736	0.953471
18562	C9orf91	431.3819758	0.014476224	0.143571	0.10083	0.919685	0.971164
17982	ILF3	4168.247564	0.014277172	0.068679	0.207883	0.83532	0.910528
17960	KDM2A	2586.041957	0.014168098	0.065928	0.214903	0.829843	0.905666
18308	TTC31	587.8533889	0.013998541	0.098288	0.142424	0.886745	0.94932
18019	DDX42	2334.758739	0.013762888	0.067953	0.202534	0.839499	0.913204
18572	TNPO3	986.9473132	0.013743271	0.138575	0.099176	0.920999	0.972028
19156	PXT1	1.488106984	0.013629745	0.31324	0.043512	0.965293	0.989949
18532	HTR7P1	91.91793724	0.013417661	0.129438	0.103661	0.917438	0.970344
18406	SIP1	89.2353347	0.013332857	0.105065	0.126901	0.899018	0.957387
18663	ZNF519	30.21586122	0.013253432	0.15543	0.085269	0.932047	0.978892
18967	)C1001300	39.28458513	0.013246455	0.237009	0.05589	0.955429	0.987366
19420	TYR	0.464149262	0.013242769	0.557587	0.02375	0.981052	0.994878
18722	)C1001339	12.57766915	0.01314958	0.169472	0.077591	0.938153	0.981752
18204	ALG9	702.5125054	0.013071757	0.081148	0.161085	0.872027	0.938947
18660	RELL1	836.7107242	0.013028536	0.151383	0.086064	0.931416	0.978432
18481	KDM3A	1745.728944	0.012904077	0.112846	0.114351	0.908959	0.964045
18275	PDCD2	581.5454691	0.012811472	0.086722	0.14773	0.882556	0.946593
19033	EPHB3	120.6837875	0.012627299	0.284182	0.044434	0.964559	0.989949
18790	AFAP1L2	451.9427964	0.012576141	0.177993	0.070655	0.943672	0.984402
19142	PCP4L1	2.352184313	0.012461731	0.283144	0.044012	0.964895	0.989949
18402	GBF1	1741.175087	0.011893556	0.092931	0.127983	0.898163	0.956652
19250	CABP1	178.0820206	0.011778349	0.394143	0.029883	0.97616	0.993674
19193	STAC	63.71179002	0.01177006	0.28682	0.041036	0.967267	0.989949
18488	GTPBP1	1082.178969	0.011765819	0.103741	0.113415	0.909701	0.964467
18554	NAA25	565.1124551	0.011643279	0.114409	0.101769	0.91894	0.970766
18746	ZFP41	263.7549957	0.011491193	0.150252	0.076479	0.939038	0.981867
18447	TSEN2	117.0724645	0.011395459	0.094074	0.121133	0.903586	0.960112
18813	SMPD1	1193.205424	0.011356788	0.167256	0.067901	0.945865	0.985483
18296	RFWD3	572.8405448	0.011224667	0.077601	0.144646	0.88499	0.948114
18114	NCOA5	895.4035866	0.010946866	0.060478	0.181007	0.856362	0.926611
18880	LOC284441	80.39964787	0.010933895	0.17519	0.062412	0.950235	0.986718
18821	HOXB4	139.2212657	0.010914626	0.16465	0.06629	0.947147	0.986438
18709	SEPT7P2	139.4066115	0.010895982	0.137195	0.07942	0.936699	0.981358
19422	UGT3A2	12.52660518	0.010740206	0.476168	0.022555	0.982005	0.994878
18787	PION	727.3072823	0.010577443	0.148578	0.071191	0.943246	0.984062
18777	ZNF266	565.5393945	0.010535588	0.143336	0.073503	0.941406	0.982718
18783	TICAM1	689.8942071	0.010496855	0.145693	0.072048	0.942564	0.983656
18522	CXorf26	411.5649474	0.010404847	0.098153	0.106006	0.915577	0.968914
18935	SLC25A45	162.718293	0.01032978	0.169693	0.060873	0.95146	0.986718

19229	VIPR2	8.913863973	0.010301843	0.292542	0.035215	0.971908	0.990711
18756	DCTPP1	639.8106375	0.010291548	0.136129	0.075602	0.939736	0.982073
18583	ERCC5	1523.991121	0.010061428	0.1042	0.096559	0.923077	0.972284
18922	PRH1	42.19182143	0.009796445	0.150113	0.065261	0.947966	0.986718
18362	PPP1R11	1539.163313	0.009771524	0.071916	0.135874	0.89192	0.952104
18818	ALG6	303.6624171	0.009610847	0.143073	0.067175	0.946443	0.985771
18791	RNASEK	2757.853641	0.009500402	0.134583	0.070591	0.943723	0.984403
18361	HNRNPA0	2775.633897	0.009473654	0.069674	0.135972	0.891843	0.952074
18742	TSEN15	443.4004114	0.009118013	0.116759	0.078093	0.937754	0.981752
18725	MGC16142	10.89499933	0.008959435	0.116543	0.076877	0.938722	0.981752
19242	ZNF702P	250.6874783	0.008953173	0.270987	0.033039	0.973643	0.991809
18407	PSMD11	1024.439848	0.008878085	0.070086	0.126675	0.899198	0.957526
18854	FAM66C	27.04201886	0.008830057	0.151959	0.058108	0.953663	0.986718
18964	C1orf97	107.0251812	0.008506134	0.150069	0.056681	0.954799	0.986871
19280	C11orf42	1.051376052	0.008389005	0.366348	0.022899	0.981731	0.994878
19246	ZNF280B	44.14526091	0.008385836	0.261021	0.032127	0.974371	0.99237
18422	HNRNPC	8127.074496	0.008381644	0.067111	0.124892	0.900609	0.958196
18763	TMEM181	915.6049162	0.008232598	0.110074	0.074792	0.94038	0.982432
19217	MGC16384	20.41036464	0.007805875	0.211769	0.03686	0.970596	0.990054
19003	BMP2K	575.2174219	0.007730535	0.163411	0.047307	0.962268	0.989949
19084	MPV17L2	231.2506793	0.007673661	0.19132	0.040109	0.968006	0.989949
19326	KCP	277.2220287	0.007658704	0.343177	0.022317	0.982195	0.994878
18755	INPP5K	856.2539119	0.007630916	0.100817	0.075691	0.939665	0.982052
18950	TCF12	1845.791502	0.007588096	0.127564	0.059484	0.952566	0.986718
19249	MYO15B	2493.673731	0.007428799	0.244456	0.030389	0.975757	0.9936
18997	ACSF3	402.4996675	0.007417604	0.14854	0.049937	0.960173	0.989949
19440	PRO0611	0.471963094	0.00734793	0.49685	0.014789	0.9882	0.996385
18757	GTF2E1	267.4272029	0.007142754	0.094654	0.075462	0.939847	0.982137
19449	CHRNG	0.302988765	0.007047646	0.572062	0.01232	0.990171	0.997858
19472	CXADRP2	0.346611338	0.007047584	0.661987	0.010646	0.991506	0.998328
19467	C9orf144	0.26293436	0.007047447	0.734753	0.009592	0.992347	0.998328
19513	LYPD4	0.259018577	0.007047396	0.774711	0.009097	0.992742	0.998328
19517	'CRNA0005	0.343386205	0.007047362	0.860966	0.008185	0.993469	0.998328
19542	'ABPC1L2I	0.260708472	0.007047338	0.817633	0.008619	0.993123	0.998328
19465	C3orf27	0.246536109	0.007047336	0.805927	0.008744	0.993023	0.998328
19459	BPESC1	0.293725356	0.007047137	0.986282	0.007145	0.994299	0.998328
19538	OR5K1	0.169023266	0.007046795	1.058253	0.006659	0.994687	0.998328
19498	IMP5	0.223059863	0.00704671	1.149544	0.00613	0.995109	0.998328
19507	LOC284632	0.186117291	0.007046669	1.133718	0.006216	0.995041	0.998328
19565	RNASE11	0.247426236	0.007046566	1.237169	0.005696	0.995456	0.998328
19544	PADI6	0.163274834	0.007046439	1.21667	0.005792	0.995379	0.998328
19511	LOC441601	0.145811633	0.007046135	1.317612	0.005348	0.995733	0.998328
19559	PRAMEF8	0.126315303	0.007046072	1.32246	0.005328	0.995749	0.998328
19492	HRH3	0.108808443	0.007045652	1.446507	0.004871	0.996114	0.998328
19500	ISL1	0.107552596	0.007045647	1.44817	0.004865	0.996118	0.998328
19475	FAM92A3	0.14172182	0.007045429	1.558785	0.00452	0.996394	0.998328
19456	ANXA10	0.107473063	0.007045209	1.582509	0.004452	0.996448	0.998328

19528	OR2C3	0.107307102	0.007045208	1.582766	0.004451	0.996448	0.998328
19561	PRY2	0.185420576	0.007045146	1.694113	0.004159	0.996682	0.998328
19569	44088	0.146904455	0.007044942	1.705436	0.004131	0.996704	0.998328
19480	GABRG2	0.128447644	0.007044802	1.721072	0.004093	0.996734	0.998328
19554	POU4F2	0.108940996	0.007044655	1.73746	0.004055	0.996765	0.998328
19494	HTR3C	0.106803581	0.007044641	1.741315	0.004046	0.996772	0.998328
19455	ACTL7B	0.088191048	0.007044442	1.769915	0.00398	0.996824	0.998328
19482	GJA8	0.088174462	0.007044442	1.769953	0.00398	0.996824	0.998328
19479	FOXD4L3	0.086521017	0.007044427	1.773793	0.003971	0.996831	0.998328
19491	HIST1H2BI	0.108052528	0.007043899	1.929029	0.003652	0.997087	0.998328
19541	OTX2	0.10307081	0.007042822	2.173124	0.003241	0.997414	0.998328
19555	PPP1R2P9	0.069341938	0.007042069	2.268489	0.003104	0.997523	0.998328
19583	TMEM146	0.068956115	0.00704206	2.27025	0.003102	0.997525	0.998328
19563	R3HDML	0.067666155	0.007042029	2.276227	0.003094	0.997532	0.998328
19536	OR5B2	0.067621664	0.007042028	2.276436	0.003093	0.997532	0.998328
19576	SNORA65	0.066684802	0.007042005	2.28087	0.003087	0.997537	0.998328
19499	IQCF3	0.126960764	0.007041846	2.405018	0.002928	0.997664	0.998328
19533	OR52N5	0.088676494	0.007040976	2.503989	0.002812	0.997756	0.998328
19591	USP29	0.085899782	0.007040928	2.512567	0.002802	0.997764	0.998328
19502	KCNG4	0.071738578	0.00704041	2.570955	0.002738	0.997815	0.998328
19531	OR4F15	0.071280006	0.007040395	2.57349	0.002736	0.997817	0.998328
19580	STARD6	0.069850315	0.007040348	2.581529	0.002727	0.997824	0.998328
19568	SEBOX	0.06929789	0.007040329	2.584691	0.002724	0.997827	0.998328
19483	GLT6D1	0.066277234	0.007040224	2.602567	0.002705	0.997842	0.998328
19501	KBTBD5	0.065462974	0.007040195	2.607564	0.0027	0.997846	0.998328
19526	OR1L1	0.068388632	0.007038276	2.91407	0.002415	0.998073	0.998328
19476	FGF16	0.068810894	0.007038276	2.91407	0.002415	0.998073	0.998328
19594	ZAR1	0.04905305	0.007038031	2.91407	0.002415	0.998073	0.998328
19529	OR2L3	0.048404328	0.007038031	2.91407	0.002415	0.998073	0.998328
19575	SNORA37	0.05027878	0.007038031	2.91407	0.002415	0.998073	0.998328
19523	OR11H6	0.046668589	0.007038031	2.91407	0.002415	0.998073	0.998328
19579	SNTN	0.04835586	0.007038031	2.91407	0.002415	0.998073	0.998328
19567	SCARNA8	0.049911135	0.007038031	2.91407	0.002415	0.998073	0.998328
19496	IFNA8	0.048552493	0.007038031	2.91407	0.002415	0.998073	0.998328
19466	C6orf10	0.051323388	0.007038031	2.91407	0.002415	0.998073	0.998328
19522	OR10A4	0.049693203	0.007038031	2.91407	0.002415	0.998073	0.998328
19486	GPR101	0.047471335	0.007038031	2.91407	0.002415	0.998073	0.998328
19505	KRTAP7-1	0.048899835	0.007038031	2.91407	0.002415	0.998073	0.998328
19508	LOC28478[illegible]	0.04845117	0.007038031	2.91407	0.002415	0.998073	0.998328
19516	MYF5	0.0488551	0.007038031	2.91407	0.002415	0.998073	0.998328
19596	ZIM3	0.047594359	0.007038031	2.91407	0.002415	0.998073	0.998328
19484	GLYATL3	0.047539826	0.007038031	2.91407	0.002415	0.998073	0.998328
19509	LOC28562[illegible]	0.049221649	0.007038031	2.91407	0.002415	0.998073	0.998328
19543	PABPN1L	0.048585279	0.007038031	2.91407	0.002415	0.998073	0.998328
19592	VCX2	0.029063939	0.007037786	2.91407	0.002415	0.998073	0.998328
19470	CSN2	0.030168934	0.007037786	2.91407	0.002415	0.998073	0.998328
19578	SNORD115-[illegible]	0.027970382	0.007037786	2.91407	0.002415	0.998073	0.998328

19457	ASB10	0.029242899	0.007037786	2.91407	0.002415	0.998073	0.998328
19477	FKSG73	0.028970029	0.007037786	2.91407	0.002415	0.998073	0.998328
19535	OR5B21	0.027996159	0.007037786	2.91407	0.002415	0.998073	0.998328
19577	SNORA71C	0.02928194	0.007037786	2.91407	0.002415	0.998073	0.998328
19464	C3orf24	0.029116467	0.007037786	2.91407	0.002415	0.998073	0.998328
19468	CLEC2A	0.032371485	0.007037786	2.91407	0.002415	0.998073	0.998328
19487	GPR119	0.028590529	0.007037786	2.91407	0.002415	0.998073	0.998328
19525	OR1D2	0.028822179	0.007037786	2.91407	0.002415	0.998073	0.998328
19527	OR2A12	0.030235827	0.007037786	2.91407	0.002415	0.998073	0.998328
19530	OR4C3	0.029014371	0.007037786	2.91407	0.002415	0.998073	0.998328
19590	USP26	0.030434678	0.007037786	2.91407	0.002415	0.998073	0.998328
19506	LCE3D	0.02902417	0.007037786	2.91407	0.002415	0.998073	0.998328
19512	LRRC30	0.028252527	0.007037786	2.91407	0.002415	0.998073	0.998328
19460	C10orf122	0.029733852	0.007037786	2.91407	0.002415	0.998073	0.998328
19462	C15orf43	0.028959785	0.007037786	2.91407	0.002415	0.998073	0.998328
19495	IFNA10	0.026672257	0.007037786	2.91407	0.002415	0.998073	0.998328
19497	IL3	0.029047982	0.007037786	2.91407	0.002415	0.998073	0.998328
19504	KRTAP10-4	0.028965044	0.007037786	2.91407	0.002415	0.998073	0.998328
19532	OR52A5	0.028203093	0.007037786	2.91407	0.002415	0.998073	0.998328
19537	OR5H6	0.027253271	0.007037786	2.91407	0.002415	0.998073	0.998328
19557	PRAMEF13	0.027130459	0.007037786	2.91407	0.002415	0.998073	0.998328
19564	RESP18	0.027462801	0.007037786	2.91407	0.002415	0.998073	0.998328
19573	SNAR-B2	0.029632699	0.007037786	2.91407	0.002415	0.998073	0.998328
19582	TMCO5A	0.028377906	0.007037786	2.91407	0.002415	0.998073	0.998328
19461	C10orf40	0.028741949	0.007037786	2.91407	0.002415	0.998073	0.998328
19463	C17orf105	0.027713295	0.007037786	2.91407	0.002415	0.998073	0.998328
19471	CST9L	0.028702563	0.007037786	2.91407	0.002415	0.998073	0.998328
19488	GPR139	0.028576597	0.007037786	2.91407	0.002415	0.998073	0.998328
19489	GPR149	0.027878303	0.007037786	2.91407	0.002415	0.998073	0.998328
19510	LOC29034	0.027579727	0.007037786	2.91407	0.002415	0.998073	0.998328
19515	MRGPRX2	0.027654182	0.007037786	2.91407	0.002415	0.998073	0.998328
19520	OPN1MW	0.028105695	0.007037786	2.91407	0.002415	0.998073	0.998328
19524	OR1C1	0.028832996	0.007037786	2.91407	0.002415	0.998073	0.998328
19539	OR5T1	0.026976198	0.007037786	2.91407	0.002415	0.998073	0.998328
19540	OR8D2	0.027822747	0.007037786	2.91407	0.002415	0.998073	0.998328
19546	PATE1	0.027951405	0.007037786	2.91407	0.002415	0.998073	0.998328
19550	PCNAP1	0.028945362	0.007037786	2.91407	0.002415	0.998073	0.998328
19553	POTEG	0.028209183	0.007037786	2.91407	0.002415	0.998073	0.998328
19556	PRAMEF12	0.028025867	0.007037786	2.91407	0.002415	0.998073	0.998328
19558	PRAMEF4	0.027628373	0.007037786	2.91407	0.002415	0.998073	0.998328
19570	SERPINA13	0.028079395	0.007037786	2.91407	0.002415	0.998073	0.998328
19574	SNORA11	0.029319955	0.007037786	2.91407	0.002415	0.998073	0.998328
19581	TBPL2	0.029804591	0.007037786	2.91407	0.002415	0.998073	0.998328
19584	TMEM202	0.027826479	0.007037786	2.91407	0.002415	0.998073	0.998328
19585	TMEM89	0.029459335	0.007037786	2.91407	0.002415	0.998073	0.998328
19588	UCMA	0.029443321	0.007037786	2.91407	0.002415	0.998073	0.998328
19552	PLUNC	0.03146396	0.007037786	2.91407	0.002415	0.998073	0.998328

19587	TSPY2	0.029148652	0.007037786	2.91407	0.002415	0.998073	0.998328
19521	OPRD1	0.029804914	0.007037786	2.91407	0.002415	0.998073	0.998328
19560	PRNT	0.028218847	0.007037786	2.91407	0.002415	0.998073	0.998328
19666	CSPG4PY2	0.009943144	0.007037541	2.91407	0.002415	0.998073	NA
20045	OR5K3	0.009943144	0.007037541	2.91407	0.002415	0.998073	NA
19604	ACTRT2	0.008597403	0.007037541	2.91407	0.002415	0.998073	NA
19609	ATXN3L	0.008597403	0.007037541	2.91407	0.002415	0.998073	NA
19611	BCYRN1	0.008597403	0.007037541	2.91407	0.002415	0.998073	NA
19612	BEYLA	0.008597403	0.007037541	2.91407	0.002415	0.998073	NA
19622	C14orf86	0.008597403	0.007037541	2.91407	0.002415	0.998073	NA
19627	C20orf166	0.009772179	0.007037541	2.91407	0.002415	0.998073	NA
19635	C21orf94	0.008597403	0.007037541	2.91407	0.002415	0.998073	NA
19640	C4orf11	0.008597403	0.007037541	2.91407	0.002415	0.998073	NA
19642	C5orf52	0.010046964	0.007037541	2.91407	0.002415	0.998073	NA
19652	CEACAM1	0.009746144	0.007037541	2.91407	0.002415	0.998073	NA
19653	CELA2B	0.010283642	0.007037541	2.91407	0.002415	0.998073	NA
19655	CETN1	0.008597403	0.007037541	2.91407	0.002415	0.998073	NA
19658	CLDN25	0.008597403	0.007037541	2.91407	0.002415	0.998073	NA
19659	CNBD1	0.010046964	0.007037541	2.91407	0.002415	0.998073	NA
19660	CRYGA	0.008597403	0.007037541	2.91407	0.002415	0.998073	NA
19664	CSN1S1	0.00984087	0.007037541	2.91407	0.002415	0.998073	NA
19667	CST11	0.009523784	0.007037541	2.91407	0.002415	0.998073	NA
19672	CT47A1	0.009670454	0.007037541	2.91407	0.002415	0.998073	NA
19679	CXorf51	0.008597403	0.007037541	2.91407	0.002415	0.998073	NA
19681	CYP11B2	0.014320994	0.007037541	2.91407	0.002415	0.998073	NA
19683	DAOA	0.008597403	0.007037541	2.91407	0.002415	0.998073	NA
19713	DMRTB1	0.008597403	0.007037541	2.91407	0.002415	0.998073	NA
19718	FABP12	0.009354564	0.007037541	2.91407	0.002415	0.998073	NA
19725	FERD3L	0.009670454	0.007037541	2.91407	0.002415	0.998073	NA
19736	GABRR3	0.009746144	0.007037541	2.91407	0.002415	0.998073	NA
19755	GOLGA2P	0.009354564	0.007037541	2.91407	0.002415	0.998073	NA
19762	GSTTP1	0.008597403	0.007037541	2.91407	0.002415	0.998073	NA
19774	HIGD1C	0.010768212	0.007037541	2.91407	0.002415	0.998073	NA
19776	HIST1H4L	0.009289	0.007037541	2.91407	0.002415	0.998073	NA
19778	HSFYL1	0.008597403	0.007037541	2.91407	0.002415	0.998073	NA
19780	HTN3	0.009670454	0.007037541	2.91407	0.002415	0.998073	NA
19781	HYALP1	0.008597403	0.007037541	2.91407	0.002415	0.998073	NA
19785	IFNA2	0.008597403	0.007037541	2.91407	0.002415	0.998073	NA
19786	IFNA4	0.008597403	0.007037541	2.91407	0.002415	0.998073	NA
19788	IFNA7	0.008597403	0.007037541	2.91407	0.002415	0.998073	NA
19798	KCNK18	0.009746144	0.007037541	2.91407	0.002415	0.998073	NA
19800	KRT76	0.008597403	0.007037541	2.91407	0.002415	0.998073	NA
19803	KRTAP10-	0.008597403	0.007037541	2.91407	0.002415	0.998073	NA
19804	KRTAP10-	0.008597403	0.007037541	2.91407	0.002415	0.998073	NA
19806	KRTAP10-	0.008597403	0.007037541	2.91407	0.002415	0.998073	NA
19810	KRTAP12-	0.008597403	0.007037541	2.91407	0.002415	0.998073	NA
19837	KRTAP4-2	0.008597403	0.007037541	2.91407	0.002415	0.998073	NA

19840	KRTAP4-5	0.008597403	0.007037541	2.91407	0.002415	0.998073	NA
19841	KRTAP4-7	0.008597403	0.007037541	2.91407	0.002415	0.998073	NA
19842	KRTAP4-8	0.008597403	0.007037541	2.91407	0.002415	0.998073	NA
19848	KRTAP9-2	0.008597403	0.007037541	2.91407	0.002415	0.998073	NA
19851	KRTAP9-9	0.008597403	0.007037541	2.91407	0.002415	0.998073	NA
19857	LCE2B	0.008597403	0.007037541	2.91407	0.002415	0.998073	NA
19860	LCE3A	0.009746144	0.007037541	2.91407	0.002415	0.998073	NA
19881	LOC28613[illegible]	0.008597403	0.007037541	2.91407	0.002415	0.998073	NA
19882	LOC28623[illegible]	0.008597403	0.007037541	2.91407	0.002415	0.998073	NA
19888	LOC64414[illegible]	0.010128162	0.007037541	2.91407	0.002415	0.998073	NA
19889	LOC64681[illegible]	0.009354564	0.007037541	2.91407	0.002415	0.998073	NA
19898	LYZL1	0.009772179	0.007037541	2.91407	0.002415	0.998073	NA
19904	MMD2	0.008597403	0.007037541	2.91407	0.002415	0.998073	NA
19916	[illegible]CRNA001[illegible]	0.008597403	0.007037541	2.91407	0.002415	0.998073	NA
19934	OR10C1	0.009138916	0.007037541	2.91407	0.002415	0.998073	NA
19937	OR10G9	0.008597403	0.007037541	2.91407	0.002415	0.998073	NA
19938	OR10H3	0.008597403	0.007037541	2.91407	0.002415	0.998073	NA
19943	OR10R2	0.008597403	0.007037541	2.91407	0.002415	0.998073	NA
19948	OR13C2	0.009354564	0.007037541	2.91407	0.002415	0.998073	NA
19955	OR14A16	0.008597403	0.007037541	2.91407	0.002415	0.998073	NA
19957	OR14J1	0.009632296	0.007037541	2.91407	0.002415	0.998073	NA
19958	OR1A1	0.008597403	0.007037541	2.91407	0.002415	0.998073	NA
19962	OR1S1	0.008597403	0.007037541	2.91407	0.002415	0.998073	NA
19968	OR2J3	0.008597403	0.007037541	2.91407	0.002415	0.998073	NA
19974	OR2M5	0.008597403	0.007037541	2.91407	0.002415	0.998073	NA
19976	OR2T12	0.008597403	0.007037541	2.91407	0.002415	0.998073	NA
19978	OR2T27	0.009772179	0.007037541	2.91407	0.002415	0.998073	NA
19981	OR2W1	0.008597403	0.007037541	2.91407	0.002415	0.998073	NA
19982	OR2W5	0.009354564	0.007037541	2.91407	0.002415	0.998073	NA
19989	OR4C12	0.008597403	0.007037541	2.91407	0.002415	0.998073	NA
19990	OR4C13	0.008597403	0.007037541	2.91407	0.002415	0.998073	NA
19992	OR4C45	0.008597403	0.007037541	2.91407	0.002415	0.998073	NA
19997	OR4E2	0.008597403	0.007037541	2.91407	0.002415	0.998073	NA
20003	OR4K15	0.008597403	0.007037541	2.91407	0.002415	0.998073	NA
20004	OR4K1	0.008597403	0.007037541	2.91407	0.002415	0.998073	NA
20005	OR4K5	0.008597403	0.007037541	2.91407	0.002415	0.998073	NA
20006	OR4L1	0.008597403	0.007037541	2.91407	0.002415	0.998073	NA
20008	OR4N5	0.008597403	0.007037541	2.91407	0.002415	0.998073	NA
20012	OR51B6	0.008597403	0.007037541	2.91407	0.002415	0.998073	NA
20016	OR51S1	0.008597403	0.007037541	2.91407	0.002415	0.998073	NA
20018	OR52A4	0.008597403	0.007037541	2.91407	0.002415	0.998073	NA
20021	OR52E8	0.008597403	0.007037541	2.91407	0.002415	0.998073	NA
20028	OR5AC2	0.008597403	0.007037541	2.91407	0.002415	0.998073	NA
20030	OR5AR1	0.008597403	0.007037541	2.91407	0.002415	0.998073	NA
20032	OR5B17	0.008597403	0.007037541	2.91407	0.002415	0.998073	NA
20033	OR5B3	0.008597403	0.007037541	2.91407	0.002415	0.998073	NA
20036	OR5D16	0.008597403	0.007037541	2.91407	0.002415	0.998073	NA

20037	OR5D18	0.008597403	0.007037541	2.91407	0.002415	0.998073	NA
20042	OR5H15	0.008597403	0.007037541	2.91407	0.002415	0.998073	NA
20046	OR5L1	0.008597403	0.007037541	2.91407	0.002415	0.998073	NA
20050	OR5M3	0.008597403	0.007037541	2.91407	0.002415	0.998073	NA
20053	OR5P3	0.008597403	0.007037541	2.91407	0.002415	0.998073	NA
20060	OR6C3	0.008597403	0.007037541	2.91407	0.002415	0.998073	NA
20061	OR6C4	0.008597403	0.007037541	2.91407	0.002415	0.998073	NA
20062	OR6C6	0.008597403	0.007037541	2.91407	0.002415	0.998073	NA
20065	OR6C70	0.008597403	0.007037541	2.91407	0.002415	0.998073	NA
20069	OR6K2	0.008597403	0.007037541	2.91407	0.002415	0.998073	NA
20071	OR6N1	0.008597403	0.007037541	2.91407	0.002415	0.998073	NA
20073	OR6P1	0.008597403	0.007037541	2.91407	0.002415	0.998073	NA
20077	OR7A10	0.008597403	0.007037541	2.91407	0.002415	0.998073	NA
20084	OR8B12	0.008597403	0.007037541	2.91407	0.002415	0.998073	NA
20086	OR8B8	0.008597403	0.007037541	2.91407	0.002415	0.998073	NA
20088	OR8D4	0.008597403	0.007037541	2.91407	0.002415	0.998073	NA
20094	OR8J1	0.008597403	0.007037541	2.91407	0.002415	0.998073	NA
20095	OR8J3	0.008597403	0.007037541	2.91407	0.002415	0.998073	NA
20096	OR8K1	0.008597403	0.007037541	2.91407	0.002415	0.998073	NA
20115	PRAMEF1(	0.008597403	0.007037541	2.91407	0.002415	0.998073	NA
20116	PRAMEF17	0.008597403	0.007037541	2.91407	0.002415	0.998073	NA
20139	RBMY2EP	0.009354564	0.007037541	2.91407	0.002415	0.998073	NA
20154	SCARNA15	0.009354564	0.007037541	2.91407	0.002415	0.998073	NA
20163	SERPINB12	0.009746144	0.007037541	2.91407	0.002415	0.998073	NA
20164	SFTA3	0.008597403	0.007037541	2.91407	0.002415	0.998073	NA
20191	SNORA28	0.008597403	0.007037541	2.91407	0.002415	0.998073	NA
20198	SNORA41	0.009772179	0.007037541	2.91407	0.002415	0.998073	NA
20205	SNORA56	0.009772179	0.007037541	2.91407	0.002415	0.998073	NA
20215	SNORA80	0.009648852	0.007037541	2.91407	0.002415	0.998073	NA
20434	SPANXN1	0.009772179	0.007037541	2.91407	0.002415	0.998073	NA
20450	TAS2R16	0.008597403	0.007037541	2.91407	0.002415	0.998073	NA
20451	TAS2R39	0.008597403	0.007037541	2.91407	0.002415	0.998073	NA
20456	TGIF2LY	0.009670454	0.007037541	2.91407	0.002415	0.998073	NA
20464	TRIM43	0.009670454	0.007037541	2.91407	0.002415	0.998073	NA
20466	TRIM64	0.008597403	0.007037541	2.91407	0.002415	0.998073	NA
20491	UBTFL1	0.009746144	0.007037541	2.91407	0.002415	0.998073	NA
20493	VCY	0.008597403	0.007037541	2.91407	0.002415	0.998073	NA
20494	VN1R2	0.008597403	0.007037541	2.91407	0.002415	0.998073	NA
20495	VN1R4	0.008597403	0.007037541	2.91407	0.002415	0.998073	NA
19639	C2orf51	0.009375785	0.007037541	2.91407	0.002415	0.998073	NA
19751	GHRH	0.009375785	0.007037541	2.91407	0.002415	0.998073	NA
19782	IFNA14	0.009375785	0.007037541	2.91407	0.002415	0.998073	NA
19923	NF1P1	0.009375785	0.007037541	2.91407	0.002415	0.998073	NA
19931	OR10A6	0.010649019	0.007037541	2.91407	0.002415	0.998073	NA
20108	PNLIP	0.009375785	0.007037541	2.91407	0.002415	0.998073	NA
20134	RBMY1A3I	0.009375785	0.007037541	2.91407	0.002415	0.998073	NA
19615	C10orf120	0.009308199	0.007037541	2.91407	0.002415	0.998073	NA

19624	C19orf41	0.009387947	0.007037541	2.91407	0.002415	0.998073	NA
19626	C1orf68	0.00914858	0.007037541	2.91407	0.002415	0.998073	NA
19646	CABP2	0.009378496	0.007037541	2.91407	0.002415	0.998073	NA
19662	CRYGC	0.009387947	0.007037541	2.91407	0.002415	0.998073	NA
19710	DEFB4A	0.010081801	0.007037541	2.91407	0.002415	0.998073	NA
19714	DUPD1	0.009546756	0.007037541	2.91407	0.002415	0.998073	NA
19717	EVX2	0.009308199	0.007037541	2.91407	0.002415	0.998073	NA
19720	FAM194B	0.009546756	0.007037541	2.91407	0.002415	0.998073	NA
19722	FAM27L	0.009546756	0.007037541	2.91407	0.002415	0.998073	NA
19726	FGF4	0.009387947	0.007037541	2.91407	0.002415	0.998073	NA
19727	FLJ25328	0.009387947	0.007037541	2.91407	0.002415	0.998073	NA
19730	FLJ44082	0.009387947	0.007037541	2.91407	0.002415	0.998073	NA
19766	H2BFWT	0.009498034	0.007037541	2.91407	0.002415	0.998073	NA
19793	IL28B	0.009387947	0.007037541	2.91407	0.002415	0.998073	NA
19811	KRTAP12-3	0.009387947	0.007037541	2.91407	0.002415	0.998073	NA
19823	KRTAP19-8	0.009387947	0.007037541	2.91407	0.002415	0.998073	NA
19849	KRTAP9-4	0.009387947	0.007037541	2.91407	0.002415	0.998073	NA
19865	LCE6A	0.009546756	0.007037541	2.91407	0.002415	0.998073	NA
19867	LELP1	0.009387947	0.007037541	2.91407	0.002415	0.998073	NA
19874	OC1002877	0.009450928	0.007037541	2.91407	0.002415	0.998073	NA
19875	LOC146481	0.009387947	0.007037541	2.91407	0.002415	0.998073	NA
19886	LOC388946	0.009387947	0.007037541	2.91407	0.002415	0.998073	NA
19895	LOC729121	0.009387947	0.007037541	2.91407	0.002415	0.998073	NA
19900	LYZL4	0.010081801	0.007037541	2.91407	0.002415	0.998073	NA
19901	MBD3L2	0.009387947	0.007037541	2.91407	0.002415	0.998073	NA
19932	OR10A7	0.009387947	0.007037541	2.91407	0.002415	0.998073	NA
19941	OR10J5	0.009387947	0.007037541	2.91407	0.002415	0.998073	NA
19953	OR13F1	0.009387947	0.007037541	2.91407	0.002415	0.998073	NA
19959	OR1A2	0.009387947	0.007037541	2.91407	0.002415	0.998073	NA
19967	OR2J2	0.009546756	0.007037541	2.91407	0.002415	0.998073	NA
19983	OR2Y1	0.009387947	0.007037541	2.91407	0.002415	0.998073	NA
19994	OR4D2	0.009546756	0.007037541	2.91407	0.002415	0.998073	NA
19999	OR4F21	0.009500523	0.007037541	2.91407	0.002415	0.998073	NA
20031	OR5AS1	0.009387947	0.007037541	2.91407	0.002415	0.998073	NA
20035	OR5D14	0.009546756	0.007037541	2.91407	0.002415	0.998073	NA
20044	OR5J2	0.009387947	0.007037541	2.91407	0.002415	0.998073	NA
20049	OR5M1	0.009387947	0.007037541	2.91407	0.002415	0.998073	NA
20079	OR7C2	0.009387947	0.007037541	2.91407	0.002415	0.998073	NA
20111	POTEB	0.009546756	0.007037541	2.91407	0.002415	0.998073	NA
20130	PRR21	0.009387947	0.007037541	2.91407	0.002415	0.998073	NA
20133	RAB9BP1	0.009387947	0.007037541	2.91407	0.002415	0.998073	NA
20141	RBMY3AP	0.009387947	0.007037541	2.91407	0.002415	0.998073	NA
20142	RETNLB	0.009387947	0.007037541	2.91407	0.002415	0.998073	NA
20161	SCGB1D1	0.009546756	0.007037541	2.91407	0.002415	0.998073	NA
20181	SNORA11E	0.009387947	0.007037541	2.91407	0.002415	0.998073	NA
20201	SNORA45	0.009387947	0.007037541	2.91407	0.002415	0.998073	NA
20442	SPZ1	0.009387947	0.007037541	2.91407	0.002415	0.998073	NA

20446	SYCN	0.009546756	0.007037541	2.91407	0.002415	0.998073	NA
20458	TLX3	0.00914858	0.007037541	2.91407	0.002415	0.998073	NA
20485	TTTY5	0.009387947	0.007037541	2.91407	0.002415	0.998073	NA
18929	SDCCAG3	563.2532322	0.006955368	0.111556	0.062349	0.950285	0.986718
18799	ARL2BP	2524.820016	0.006817174	0.099321	0.068638	0.945278	0.985142
18981	KRCC1	890.398742	0.006741122	0.125259	0.053818	0.957081	0.988343
19366	OR52N4	4.874695278	0.006733236	0.355016	0.018966	0.984868	0.994878
18785	ZFP90	656.3928909	0.006731661	0.093764	0.071794	0.942766	0.983719
19243	NR5A2	165.0918793	0.006709341	0.203975	0.032893	0.97376	0.991876
18869	KCTD7	434.4335633	0.006618771	0.107815	0.06139	0.951048	0.986718
18990	OC1001295	233.6277673	0.006502757	0.124034	0.052427	0.958188	0.989018
19157	RAD51L1	60.84531887	0.006502133	0.145055	0.044825	0.964247	0.989949
19024	CTCFL	8.179602675	0.006443278	0.138019	0.046684	0.962765	0.989949
19415	TKTL1	4.068589117	0.006435233	0.309867	0.020768	0.983431	0.994878
19199	TMEM111	985.0296346	0.006258603	0.135494	0.046191	0.963158	0.989949
18812	PI4KB	1674.502629	0.005916471	0.086573	0.068341	0.945514	0.985171
19245	FAM57A	573.5627008	0.005897429	0.183751	0.032095	0.974397	0.99237
18951	TCP1	3154.973847	0.005808139	0.092793	0.062592	0.950091	0.986718
18930	SFT2D1	387.2866019	0.005550191	0.086448	0.064203	0.948809	0.986718
18829	BAZ2A	2733.96537	0.005491972	0.087893	0.062485	0.950177	0.986718
19303	FAT3	56.53745625	0.005461367	0.30121	0.018131	0.985534	0.994878
19312	GPRC5D	4.011251065	0.005398681	0.302162	0.017867	0.985745	0.994878
19282	C1orf104	63.83021766	0.005271468	0.221292	0.023821	0.980995	0.994878
19345	CRNA002(	829.6935184	0.005249884	0.221958	0.023653	0.98113	0.994878
19068	OC1000096	97.37320856	0.005018309	0.120674	0.041586	0.966829	0.989949
19261	ZNF93	57.30835343	0.004960695	0.167971	0.029533	0.976439	0.993693
18949	TCEB3	1070.232307	0.00487099	0.075386	0.064614	0.948481	0.986718
19534	OR56B1	0.868752369	0.004813031	0.521346	0.009232	0.992634	0.998328
19391	SEMA6C	237.767461	0.004762617	0.188714	0.025237	0.979866	0.994878
18786	ARF3	5661.532338	0.004672991	0.065597	0.071238	0.943208	0.984062
19248	PARP3	450.9950747	0.004582854	0.149526	0.030649	0.975549	0.99344
19251	CREG1	4015.08931	0.004580805	0.153444	0.029853	0.976184	0.993674
19273	ANKRD44	140.1307503	0.004561083	0.192941	0.02364	0.98114	0.994878
19253	PRKDC	2636.915631	0.004501998	0.150052	0.030003	0.976065	0.993674
19270	BCAR1	2228.402451	0.004474996	0.159491	0.028058	0.977616	0.994408
19195	TAF5	87.18489629	0.004378076	0.108556	0.04033	0.96783	0.989949
19428	ZNF876P	15.25306939	0.004341954	0.180769	0.024019	0.980837	0.994878
19256	TIA1	1348.281521	0.004176269	0.138499	0.030154	0.975944	0.993674
19450	T6GALNAC	3.689943135	0.003805726	0.308304	0.012344	0.990151	0.997858
19430	CA11	189.4102449	0.003802983	0.224724	0.016923	0.986498	0.995155
19406	SREBF1	2189.583921	0.003776907	0.186418	0.02026	0.983836	0.994878
19547	PCDHA11	51.64975054	0.003729882	0.432681	0.00862	0.993122	0.998328
19493	HRNR	29.11461087	0.003568621	0.333525	0.0107	0.991463	0.998328
19269	AHI1	260.499614	0.003375918	0.119647	0.028216	0.97749	0.994332
19551	PEG3AS	0.644141608	0.003044734	0.536831	0.005672	0.995475	0.998328
19412	TASP1	164.402592	0.003035491	0.112075	0.027084	0.978392	0.994878
19439	OC1001309	10.4266765	0.00297803	0.199298	0.014943	0.988078	0.996312

19474	FAM157A	8.059767583	0.002961558	0.283568	0.010444	0.991667	0.998328
19278	BNIP2	881.944188	0.002950001	0.123747	0.023839	0.980981	0.994878
19490	GUCY2C	3.488892885	0.002888259	0.286173	0.010093	0.991947	0.998328
19000	ARHGAP1[illegible]	1231.470299	0.00287779	0.075058	0.038341	0.969416	0.989949
19454	PKD1L1	15.44194473	0.002863914	0.246179	0.011633	0.990718	0.998204
19262	C19orf52	196.7232099	0.00276426	0.094576	0.029228	0.976683	0.993872
19218	SF3B2	3614.300279	0.002738425	0.074823	0.036598	0.970805	0.990153
19324	IMP3	1209.104961	0.002727988	0.107031	0.025488	0.979666	0.994878
19343	NAAA	573.2430371	0.002714095	0.132191	0.020532	0.983619	0.994878
19589	UGT1A6	5282.48608	0.002655115	0.382756	0.006937	0.994465	0.998328
19432	DDT	903.3059188	0.002463681	0.151501	0.016262	0.987026	0.99561
19275	ATXN7L1	290.6785739	0.002448031	0.106465	0.022994	0.981655	0.994878
19337	LPGAT1	2271.606948	0.002403134	0.124323	0.01933	0.984578	0.994878
19443	KIAA1407	212.4228331	0.002313648	0.165369	0.013991	0.988837	0.996873
19342	MAP2K7	775.9092207	0.002286902	0.109098	0.020962	0.983276	0.994878
19426	ZNF324B	94.26443092	0.002262959	0.092418	0.024486	0.980465	0.994878
19447	A4GALT	1183.079668	0.00226073	0.18093	0.012495	0.990031	0.997819
19387	RNF44	1359.287718	0.002202385	0.10046	0.021923	0.982509	0.994878
19485	GOLGA2B	206.9293721	0.002085238	0.256585	0.008127	0.993516	0.998328
19458	ATHL1	2022.64646	0.002034874	0.356156	0.005713	0.995441	0.998328
19593	WDR27	128.0593596	0.001838564	0.218555	0.008412	0.993288	0.998328
19349	NOL10	626.1684999	0.001739859	0.073601	0.023639	0.981141	0.994878
19435	MED12	979.4686546	0.001630281	0.102311	0.015935	0.987287	0.995719
19279	BTBD10	573.6935165	0.001627295	0.093729	0.017362	0.986148	0.994878
19330	KTI12	174.3421718	0.001618705	0.091994	0.017596	0.985961	0.994878
19448	KIAA0467	890.724761	0.001602722	0.127626	0.012558	0.98998	0.997819
19549	PCDHGA1[illegible]	96.19802102	0.001416108	0.323434	0.004378	0.996507	0.998328
19478	FLJ42627	55.91311107	0.001335501	0.174279	0.007663	0.993886	0.998328
19586	TRPC6	144.1704842	0.001233922	0.234997	0.005251	0.99581	0.998328
19452	GCC1	593.6247301	0.001231216	0.102867	0.011969	0.99045	0.998029
19444	RBM4B	255.007031	0.001203211	0.087908	0.013687	0.98908	0.997036
19572	SLC9A3R2	1685.88133	0.001070628	0.232345	0.004608	0.996323	0.998328
19518	NLRX1	510.1305936	0.00097217	0.108891	0.008928	0.992877	0.998328
19438	C10orf76	645.9371226	0.000882995	0.058825	0.01501	0.988024	0.996309
19503	KIAA1919	212.7648942	0.000713549	0.141874	0.005029	0.995987	0.998328
19514	MMP24	1272.428877	0.000642075	0.209467	0.003065	0.997554	0.998328
19473	CYSLTR1	57.80957414	0.000543113	0.21233	0.002558	0.997959	0.998328
19598	RERG	1078.524329	0.000506191	0.25215	0.002007	0.998398	0.998551
19469	COX4NB	448.9453532	0.000467519	0.084603	0.005526	0.995591	0.998328
19601	IDH3B	1688.355076	6.87E-05	0.107116	0.000642	0.999488	0.999488
19602	ACTL6B	0	0	0	0	1	NA
19738	GAGE12D	0	0	0	0	1	NA
19740	GAGE12J	0	0	0	0	1	NA
19742	GAGE2B	0	0	0	0	1	NA
19599	NIP7	572.826337	-0.000167825	0.095021	-0.001766	0.998591	0.998693
19600	SEC23A	1863.846145	-0.000182543	0.158884	-0.001149	0.999083	0.999134
19597	SMARCB1	1323.346742	-0.000210609	0.099279	-0.002121	0.998307	0.998511

19566	RPF1	505.1202062	-0.000430167	0.099136	-0.004339	0.996538	0.998328
19519	NUP205	1152.258319	-0.000723111	0.11981	-0.006035	0.995184	0.998328
19595	ZDHHC4	1031.801594	-0.000784856	0.107206	-0.007321	0.994159	0.998328
19481	GIMAP5	1109.893834	-0.000812693	0.18905	-0.004299	0.99657	0.998328
19545	PARP9	1598.101721	-0.000931705	0.12614	-0.007386	0.994107	0.998328
19571	SLC22A16	2.801363715	-0.000987143	0.370003	-0.002668	0.997871	0.998328
19445	TMEM85	1473.977062	-0.001034201	0.07571	-0.01366	0.989101	0.997036
19548	PCDHA6	46.37063102	-0.001328961	0.274523	-0.004841	0.996137	0.998328
19441	GEMIN4	365.0600134	-0.001412204	0.099139	-0.014245	0.988635	0.996771
19433	NSD1	1700.96857	-0.00147293	0.091369	-0.016121	0.987138	0.995672
19453	MRPS18A	683.9879012	-0.001644469	0.138012	-0.011915	0.990493	0.998029
19434	CAMTA2	1000.399036	-0.001889069	0.118176	-0.015985	0.987246	0.995719
19309	GDI2	5781.410368	-0.00209779	0.117681	-0.017826	0.985778	0.994878
19315	GTPBP4	720.6728148	-0.002124315	0.082059	-0.025888	0.979347	0.994878
19446	MAPRE3	451.1814742	-0.002134922	0.168604	-0.012662	0.989897	0.997787
19437	C7orf28B	592.3061931	-0.002250663	0.147715	-0.015236	0.987844	0.996178
19258	E2F6	284.5300196	-0.002397795	0.080605	-0.029748	0.976268	0.993693
19436	FLJ39653	82.38823497	-0.002438324	0.158079	-0.015425	0.987693	0.996078
19380	PQLC3	783.3307539	-0.002484767	0.133755	-0.018577	0.985179	0.994878
19339	MAFG	648.7906032	-0.002507043	0.118238	-0.021203	0.983083	0.994878
19295	CRIPAK	249.1676273	-0.002547184	0.142382	-0.01789	0.985727	0.994878
19314	GTF3C1	1873.938526	-0.002563872	0.109551	-0.023403	0.981329	0.994878
19220	DNAJC14	1156.051294	-0.002655131	0.072903	-0.03642	0.970947	0.990194
19236	DEM1	124.8821655	-0.002877546	0.084134	-0.034202	0.972716	0.991173
19160	RFWD2	675.3664683	-0.003110357	0.075481	-0.041207	0.967131	0.989949
19285	C9orf86	1664.921792	-0.003281548	0.153672	-0.021354	0.982963	0.994878
19392	SIN3B	1055.679107	-0.003317566	0.121758	-0.027247	0.978263	0.994878
19451	C2CD4C	39.09957997	-0.003356644	0.277872	-0.01208	0.990362	0.997999
19293	CLDND1	1586.42234	-0.003475201	0.130582	-0.026613	0.978768	0.994878
19562	psiTPTE22	86.32787973	-0.003593924	0.363405	-0.00989	0.992109	0.998328
19188	SPG21	1944.294176	-0.00361874	0.071822	-0.050385	0.959816	0.989949
19005	BRF2	287.6755759	-0.004024286	0.105946	-0.037984	0.9697	0.989949
19442	PRSS16	267.4105971	-0.004238363	0.300397	-0.014109	0.988743	0.996829
19238	NDST1	3191.132242	-0.004373762	0.129122	-0.033873	0.972978	0.991337
19231	ZNF167	58.5452218	-0.004437126	0.126791	-0.034996	0.972083	0.990786
19268	ATP2B1	1115.75129	-0.004580551	0.160156	-0.028601	0.977183	0.994071
19234	CLN6	1218.983494	-0.005009402	0.145266	-0.034484	0.972491	0.990995
19240	C6orf192	375.6097412	-0.005072484	0.150603	-0.033681	0.973131	0.99139
19305	FLJ10213	12.13262816	-0.005137154	0.205629	-0.024983	0.980069	0.994878
19252	PDGFB	1102.105965	-0.005175164	0.173219	-0.029876	0.976166	0.993674
18965	ERH	1425.559747	-0.005339636	0.094483	-0.056514	0.954932	0.986956
19222	C2orf74	324.820066	-0.00535801	0.150432	-0.035618	0.971587	0.990696
19028	DENND1A	822.1413511	-0.005382344	0.120299	-0.044741	0.964314	0.989949
19162	RHOQ	2537.202423	-0.005402436	0.141177	-0.038267	0.969475	0.989949
18992	GCN1L1	2280.930037	-0.00554418	0.107355	-0.051643	0.958813	0.989558
18972	PSME4	1243.959523	-0.005796823	0.104709	-0.055361	0.95585	0.98755
18848	DAD1	2346.759857	-0.005823125	0.098809	-0.058933	0.953005	0.986718

19226	CCDC56	1620.866319	-0.006201725	0.175593	-0.035319	0.971826	0.990697
18888	MAP3K3	961.2535473	-0.006306932	0.100135	-0.062985	0.949779	0.986718
19239	MTSS1L	852.6895815	-0.006467721	0.191479	-0.033778	0.973054	0.991363
18918	PPP1CC	3232.957703	-0.006600586	0.113719	-0.058043	0.953714	0.986718
19018	CDC42EP2	195.0264296	-0.00668417	0.172466	-0.038756	0.969085	0.989949
18978	SIPA1L3	988.4952273	-0.00681778	0.124016	-0.054975	0.956158	0.987637
19173	SMAD7	829.9087261	-0.006874354	0.157402	-0.043674	0.965164	0.989949
19001	B9D1	264.0472791	-0.006885905	0.181	-0.038044	0.969653	0.989949
19388	RSPO3	52.26863469	-0.006970241	0.335932	-0.020749	0.983446	0.994878
18649	CSRNP2	794.1674562	-0.006976388	0.078659	-0.088692	0.929327	0.976767
18807	RWDD1	921.0972488	-0.007304433	0.105658	-0.069133	0.944884	0.985142
18852	ERGIC2	787.2947056	-0.007357289	0.120271	-0.061172	0.951222	0.986718
18645	CRCP	829.6142865	-0.007394169	0.083065	-0.089016	0.929069	0.976665
19228	ZNF395	8294.441566	-0.007712225	0.218503	-0.035296	0.971844	0.990697
18788	SNX15	443.7560496	-0.007823146	0.109705	-0.07131	0.943151	0.984062
19092	NQO2	1051.73475	-0.007824874	0.163042	-0.047993	0.961722	0.989949
19221	ANKS1B	111.2790181	-0.007830735	0.217623	-0.035983	0.971296	0.990498
18566	UBAP2L	2549.344118	-0.008064934	0.080639	-0.100013	0.920334	0.97165
18741	STAT6	4997.835994	-0.008140331	0.106089	-0.076731	0.938837	0.981752
19170	SH3TC1	1224.473021	-0.008159127	0.16351	-0.0499	0.960202	0.989949
19325	KCNH2	111.0375942	-0.008198102	0.4353	-0.018833	0.984974	0.994878
19159	RBMS3	81.37703103	-0.008327464	0.221706	-0.037561	0.970038	0.989949
19141	PCDHB12	77.45462351	-0.008379416	0.214378	-0.039087	0.968821	0.989949
18970	SCHIP1	455.1215799	-0.008515644	0.153186	-0.05559	0.955668	0.987457
19013	C2orf55	230.0024034	-0.00857193	0.193932	-0.044201	0.964744	0.989949
18784	DCP2	892.2403892	-0.008644364	0.120325	-0.071842	0.942728	0.983719
18775	TM2D3	993.7934282	-0.008720761	0.118533	-0.073572	0.941351	0.982718
19247	ESR2	17.02067681	-0.008733553	0.278881	-0.031316	0.975017	0.99295
19200	TNFSF10	5560.407983	-0.008807999	0.230281	-0.038249	0.969489	0.989949
19056	KCNK7	5.921250559	-0.008845793	0.226775	-0.039007	0.968885	0.989949
18927	RTKN	1242.063809	-0.009022126	0.147846	-0.061024	0.95134	0.986718
18824	ADAT2	102.9144293	-0.009029096	0.153396	-0.058862	0.953062	0.986718
18931	SFXN4	571.2238116	-0.009293908	0.156729	-0.059299	0.952714	0.986718
19310	GLOD5	13.08982565	-0.009430988	0.397008	-0.023755	0.981048	0.994878
18541	RPUSD4	435.2153316	-0.009740231	0.094831	-0.102712	0.918192	0.970735
18451	HARS2	794.7393332	-0.009760682	0.08132	-0.120028	0.904461	0.960833
18607	TMEM107	222.3741161	-0.009824502	0.101207	-0.097073	0.922668	0.972284
19233	DCAF4L1	9.45450325	-0.009839651	0.283798	-0.034671	0.972342	0.99099
19146	PPARGC1E	24.22953593	-0.009898312	0.206282	-0.047984	0.961729	0.989949
18661	KIF9	90.96295496	-0.009918473	0.115925	-0.08556	0.931817	0.978754
18602	RPS6KC1	428.5047598	-0.010079327	0.104191	-0.096739	0.922933	0.972284
18631	NIT1	838.1560837	-0.010539207	0.115991	-0.090862	0.927602	0.975853
19431	FLJ44054	0.85296285	-0.010584176	0.626646	-0.01689	0.986524	0.995155
18521	ACIN1	2236.985671	-0.010676324	0.100396	-0.106342	0.915311	0.968685
18480	GMEB1	116.3409791	-0.010783419	0.094082	-0.114617	0.908748	0.963873
19074	LOC541473	4.187170202	-0.010810783	0.245159	-0.044097	0.964827	0.989949
18648	GPATCH4	423.0251351	-0.010993219	0.123798	-0.0888	0.929241	0.97673

18861	HOXA10	581.6078876	-0.011071506	0.185204	-0.05978	0.952331	0.986718
19241	STX19	1.58863875	-0.011211266	0.334284	-0.033538	0.973245	0.991455
18969	PNMAL2	40.79471451	-0.011211963	0.201439	-0.055659	0.955613	0.987452
19232	ACPP	22.41364832	-0.011240781	0.324716	-0.034617	0.972385	0.99099
19032	EFCAB5	4.203755594	-0.011379517	0.25292	-0.044993	0.964113	0.989949
19264	C7orf71	0.707393787	-0.011579827	0.401594	-0.028835	0.976996	0.993972
18534	TTF2	227.4919375	-0.011664346	0.112605	-0.103587	0.917497	0.970344
19185	SOX5	35.44964721	-0.011826715	0.254202	-0.046525	0.962892	0.989949
18779	ZNF107	223.2361539	-0.011961377	0.163619	-0.073105	0.941722	0.982944
18655	YIPF5	1511.329191	-0.012043906	0.13752	-0.087579	0.930211	0.977382
19237	RAB27B	17.11666053	-0.012114624	0.35572	-0.034057	0.972832	0.99124
18702	DOCK7	785.8386534	-0.012139964	0.149846	-0.081016	0.935429	0.980395
18394	GAR1	308.3264144	-0.012218233	0.094141	-0.129786	0.896736	0.955579
18621	FAM111A	1006.261146	-0.012298755	0.131416	-0.093586	0.925438	0.974143
18377	TFB1M	161.1571183	-0.012339285	0.092725	-0.133075	0.894134	0.953769
18501	POLR1C	339.2444087	-0.012450434	0.111651	-0.111512	0.91121	0.965387
18630	PARD3	989.2223712	-0.012487009	0.137004	-0.091143	0.927379	0.975714
18868	KCNK1	491.8088673	-0.012727692	0.200783	-0.06339	0.949456	0.986718
18804	CIDEB	1133.690999	-0.01314532	0.191597	-0.068609	0.945301	0.985142
18820	FLJ44606	34.81291727	-0.013167764	0.198777	-0.066244	0.947184	0.986438
18458	DPY30	693.6956616	-0.013338735	0.113396	-0.117629	0.906361	0.962435
18222	HNRNPM	2976.384563	-0.01369757	0.086929	-0.157573	0.874794	0.940984
18511	DHRS1	369.943193	-0.013731677	0.126661	-0.108413	0.913668	0.967469
18388	KDM5C	2222.882365	-0.013802932	0.105325	-0.13105	0.895735	0.954824
17980	ZNF498	392.4065177	-0.014034758	0.06714	-0.209036	0.83442	0.909678
18432	HDDC2	908.2449373	-0.014037114	0.113795	-0.123354	0.901826	0.95897
18152	C15orf24	1511.433046	-0.01420929	0.081547	-0.174248	0.861671	0.930455
18241	ZNF576	200.0242089	-0.014284656	0.091334	-0.1564	0.875718	0.941374
19286	CAPN8	2.988390632	-0.014476678	0.582413	-0.024856	0.98017	0.994878
18671	ACCS	348.1275509	-0.014488004	0.175559	-0.082525	0.934229	0.979957
18805	CPM	1734.232348	-0.014528767	0.209893	-0.06922	0.944815	0.985142
18828	AVIL	38.69637556	-0.01455563	0.251614	-0.057849	0.953869	0.986718
18674	C18orf1	805.3438672	-0.014805629	0.181431	-0.081605	0.934961	0.979957
18528	C1orf93	851.845474	-0.014859583	0.142799	-0.10406	0.917122	0.970184
18401	LACTB	525.8036601	-0.014867146	0.115805	-0.128381	0.897847	0.956399
18047	R3HDM2	1323.709632	-0.015110919	0.076612	-0.197239	0.84364	0.916285
18643	C1orf183	81.1004467	-0.015131942	0.169505	-0.089272	0.928866	0.976665
18802	C1orf63	577.4399806	-0.015192294	0.219815	-0.069114	0.944899	0.985142
18382	UNKL	228.9352705	-0.015379655	0.116503	-0.132011	0.894976	0.954326
18105	SAP130	577.2231769	-0.015477936	0.084972	-0.182153	0.855463	0.926149
18537	ZNF814	276.6051397	-0.015499665	0.150331	-0.103104	0.917881	0.970566
18683	LOC14448(	8.121421214	-0.015547092	0.188777	-0.082357	0.934363	0.979957
18167	SP1	3271.033391	-0.015645947	0.091401	-0.171179	0.864083	0.932331
18177	EED	301.0058375	-0.015731712	0.093654	-0.167977	0.866601	0.934491
18510	FLJ37453	76.68203886	-0.015749786	0.144876	-0.108712	0.913431	0.967269
18678	FAM100A	396.5866658	-0.015904657	0.191789	-0.082928	0.933909	0.979957
18620	BHLHE40	7550.085533	-0.0159841	0.169651	-0.094217	0.924937	0.973667

17928	RNF4	1424.683889	-0.016008513	0.071727	-0.223185	0.823391	0.900318
18267	MYO1C	5390.352553	-0.016141077	0.108087	-0.149335	0.88129	0.945648
18248	SETD4	302.2280918	-0.016334258	0.106125	-0.153915	0.877676	0.942764
18223	ZNF805	341.7705918	-0.016365517	0.103891	-0.157525	0.874831	0.940984
18221	COG1	892.5358148	-0.016554031	0.104871	-0.157852	0.874574	0.940811
18568	C7orf23	857.376653	-0.016577559	0.166345	-0.099658	0.920616	0.971781
18003	ADAR	6232.921988	-0.016673057	0.081195	-0.205346	0.837302	0.911623
18410	DNAJC15	843.1868082	-0.016712887	0.132089	-0.126528	0.899314	0.957585
18668	OR2A7	140.7140324	-0.016804949	0.199891	-0.084071	0.933	0.97963
17988	ZNF764	192.8563085	-0.016957492	0.081785	-0.207341	0.835743	0.910743
18325	RBMS1	1732.269777	-0.016984885	0.121245	-0.140088	0.888591	0.950465
18030	ZNF346	267.4012112	-0.017213648	0.0858	-0.200626	0.840991	0.914466
19347	NELL1	6.604213331	-0.017274675	0.84114	-0.020537	0.983615	0.994878
18708	CMPK2	263.6703504	-0.017312986	0.217163	-0.079723	0.936457	0.981158
18062	ARID1A	1659.595439	-0.017360848	0.089518	-0.193936	0.846226	0.918307
18651	ELK3	646.392354	-0.017531161	0.199079	-0.088061	0.929828	0.977032
18237	TOPBP1	654.6803514	-0.01766026	0.112777	-0.156595	0.875564	0.941374
18142	BRD4	1529.286185	-0.01773505	0.100864	-0.175831	0.860427	0.929333
18324	DUS2L	199.5970827	-0.018027895	0.128646	-0.140136	0.888553	0.950465
18369	FUK	534.3379065	-0.018094106	0.134188	-0.134842	0.892737	0.952613
19296	DCAF12L2	3.833027543	-0.018178717	0.7556	-0.024059	0.980806	0.994878
18295	KDM4D	30.81859844	-0.01824037	0.126071	-0.144683	0.884961	0.948114
18257	TNRC6C	352.3831008	-0.01869929	0.124142	-0.150629	0.880269	0.94507
17813	ICMT	1925.134006	-0.018769943	0.077012	-0.243727	0.807443	0.888535
18029	ERMAP	375.4194243	-0.019003817	0.094832	-0.200394	0.841173	0.914466
18731	PCSK5	161.9918052	-0.019010152	0.245697	-0.077372	0.938327	0.981752
18436	BET1	865.7841515	-0.01919109	0.157136	-0.12213	0.902796	0.959845
18916	PCDHB6	135.1765504	-0.019216436	0.321077	-0.05985	0.952275	0.986718
18527	ARAP3	745.7645276	-0.01934053	0.18528	-0.104385	0.916864	0.970014
18711	LOC72874	249.2692189	-0.019430816	0.245604	-0.079114	0.936942	0.981508
18491	RAB8B	1253.670848	-0.019518814	0.172861	-0.112916	0.910097	0.96463
18053	ZNF263	665.1077954	-0.019605198	0.100564	-0.194953	0.84543	0.917923
18240	UBE2D1	438.7075097	-0.019606931	0.125648	-0.156046	0.875997	0.941374
18181	TRIM4	967.5560436	-0.019622685	0.117211	-0.167414	0.867045	0.934764
18150	NAPB	276.315053	-0.01987205	0.113749	-0.1747	0.861315	0.930173
18411	MAP2K3	1233.722018	-0.019910053	0.157569	-0.126358	0.899449	0.957585
18435	PDF	230.9485076	-0.020027668	0.163574	-0.122438	0.902552	0.959638
18625	SLC25A10	616.809617	-0.02004645	0.215896	-0.092852	0.926021	0.974547
18063	GMEB2	424.1524686	-0.020172722	0.104035	-0.193903	0.846252	0.918307
18456	ATP5G1	988.134885	-0.020190751	0.171281	-0.117881	0.906162	0.96238
18102	TUT1	530.7266145	-0.020225556	0.110053	-0.183781	0.854185	0.924919
18884	LOC374491	3.970260722	-0.020341467	0.344562	-0.059036	0.952924	0.986718
18032	PIGS	1446.008279	-0.020354375	0.102	-0.199553	0.841831	0.91508
18195	DOLPP1	221.0570809	-0.020449234	0.125374	-0.163105	0.870435	0.937697
18519	KCNQ1OT	179.1870984	-0.02054355	0.191979	-0.107009	0.914782	0.968229
18015	C6orf106	3747.625423	-0.020590688	0.101388	-0.203088	0.839067	0.912885
18431	TFEB	573.1622254	-0.020824246	0.168359	-0.12369	0.901561	0.958792

17589	GTF3C2	1180.432242	-0.021076169	0.071123	-0.296332	0.766976	0.85471
17793	MGAT2	875.6388361	-0.021097865	0.085381	-0.247102	0.804829	0.886576
18312	USP13	376.8031974	-0.021207393	0.149843	-0.141531	0.887451	0.949919
18163	TXN2	1779.861838	-0.021321202	0.124267	-0.171576	0.863771	0.932158
18540	NEDD4	866.3596618	-0.021348984	0.208022	-0.102628	0.918258	0.970735
18125	CA5BP	257.1314718	-0.021366369	0.119606	-0.17864	0.85822	0.928058
18310	C4orf46	171.6545806	-0.021445823	0.150987	-0.142037	0.887051	0.949595
18599	PLCH1	231.1636269	-0.021462622	0.221844	-0.096747	0.922928	0.972284
17923	C10orf26	1842.727022	-0.021709502	0.097018	-0.223768	0.822938	0.899968
18199	MRPL2	511.3432495	-0.021726706	0.134194	-0.161905	0.87138	0.938509
18157	LOC34892[illegible]	58.8838969	-0.02178809	0.126063	-0.172835	0.862781	0.931345
18046	POLDIP2	2700.776349	-0.022223421	0.11261	-0.197349	0.843555	0.916243
18404	PSTK	31.36050107	-0.022272637	0.174776	-0.127435	0.898596	0.957041
17906	CXorf40B	396.2672	-0.022363611	0.098427	-0.227209	0.820261	0.897908
18454	SYNJ2	307.6224536	-0.022642956	0.19029	-0.118992	0.905282	0.961549
18130	MRPL4	684.204396	-0.02266027	0.12714	-0.17823	0.858542	0.928204
18366	C8orf45	38.10595497	-0.022683242	0.16757	-0.135365	0.892323	0.952296
18120	TMEM194[illegible]	563.1178315	-0.022724701	0.126105	-0.180205	0.856992	0.927074
17990	FAM98A	995.1284912	-0.022757059	0.109915	-0.207042	0.835977	0.910838
18365	LOC40109[illegible]	101.8129857	-0.022815112	0.168356	-0.135517	0.892203	0.952276
17641	DDX47	962.9144117	-0.022889016	0.08117	-0.281988	0.777953	0.864387
19191	SSTR5	2.960577257	-0.022928855	0.616205	-0.03721	0.970318	0.989949
17751	SYS1	1438.309528	-0.023282275	0.090868	-0.256219	0.797781	0.880926
17772	HGSNAT	2349.954829	-0.023289583	0.092287	-0.25236	0.800763	0.883173
18298	ECSIT	932.7633809	-0.023346217	0.162159	-0.143971	0.885524	0.948582
18479	LOC40112[illegible]	6.030303853	-0.023384398	0.203128	-0.115122	0.908349	0.963501
18067	C15orf41	130.2634381	-0.023551881	0.122029	-0.193003	0.846957	0.918834
18844	CXorf48	0.90552434	-0.023552529	0.379565	-0.062051	0.950522	0.986718
17542	YY1AP1	1316.57225	-0.023590441	0.077506	-0.304368	0.760848	0.850153
18335	ABHD11	596.4816796	-0.023669254	0.170439	-0.138872	0.889551	0.950973
18346	HEATR7A	509.6432437	-0.023921883	0.175083	-0.136632	0.891322	0.952033
18391	BTN2A3	32.40051826	-0.024010483	0.183659	-0.130734	0.895986	0.954883
18642	C10orf128	39.62794207	-0.024113659	0.270939	-0.089	0.929082	0.976665
18155	C1orf201	167.1840691	-0.024176601	0.139603	-0.173181	0.862509	0.931206
18074	CNNM4	518.0439909	-0.024185883	0.12589	-0.192118	0.847649	0.919213
18301	NPC1	1876.968053	-0.024249519	0.168897	-0.143576	0.885835	0.94876
18261	DDX60L	517.7545744	-0.024296967	0.162334	-0.149673	0.881023	0.945545
17692	FDPS	1548.804949	-0.024317821	0.090442	-0.268878	0.788023	0.872954
17941	TOMM40L	437.392882	-0.024450961	0.111224	-0.219835	0.826	0.902426
18773	PPP1R14C	302.14254	-0.024507401	0.332135	-0.073787	0.94118	0.982691
17575	APEX1	2416.313375	-0.024549986	0.082293	-0.298323	0.765457	0.853667
18036	WDR60	708.1224514	-0.024564193	0.123432	-0.19901	0.842255	0.915338
18037	ARAF	1257.616072	-0.024928026	0.125391	-0.198803	0.842417	0.91541
18772	HFM1	3.113929964	-0.024934453	0.337641	-0.073849	0.941131	0.982691
18375	FGGY	321.1207148	-0.025153843	0.189156	-0.132979	0.89421	0.953769
17875	ARHGEF11	1009.141908	-0.025212462	0.108937	-0.23144	0.816973	0.895818
17837	C11orf59	2003.296146	-0.025408848	0.106795	-0.237921	0.811942	0.892239

17510	SLC25A14	131.7731689	-0.025463155	0.081311	-0.313157	0.754161	0.844221
18329	LAPTM4B	2385.656682	-0.025550819	0.183019	-0.139607	0.88897	0.950653
18644	CASQ1	3.260053711	-0.025563834	0.286876	-0.089111	0.928994	0.976665
18055	TAF1A	51.05133914	-0.025702015	0.131933	-0.194811	0.845541	0.917943
17640	SRPR	3780.045653	-0.025778823	0.091322	-0.282286	0.777725	0.864183
18274	SMTN	1254.954676	-0.025834844	0.174795	-0.147801	0.8825	0.946592
18004	SELK	606.0727586	-0.025849709	0.125999	-0.205159	0.837448	0.911731
17446	PKNOX1	405.5463028	-0.025905943	0.07911	-0.327467	0.743315	0.835132
18025	PACS2	1187.15528	-0.026084847	0.129896	-0.200813	0.840845	0.914363
18580	CUZD1	24.47146386	-0.02614232	0.270695	-0.096575	0.923064	0.972284
17685	NAA20	1195.326187	-0.026168669	0.096304	-0.27173	0.785829	0.870966
18416	POLN	17.44659921	-0.026358491	0.209302	-0.125935	0.899783	0.957629
18379	STAP2	703.8940592	-0.026376083	0.198881	-0.132623	0.894492	0.954011
18215	SLC27A1	782.0483689	-0.026682207	0.167821	-0.158992	0.873675	0.940154
17876	LAPTM4A	10277.66489	-0.026833554	0.115947	-0.23143	0.81698	0.895818
18429	ZDHHC20	159.2179763	-0.026943771	0.21746	-0.123902	0.901393	0.958717
17483	METTL13	607.4799663	-0.027006557	0.084806	-0.318452	0.750142	0.841019
17996	DNAJC30	388.4392462	-0.027098994	0.131301	-0.206388	0.836488	0.911092
17285	NKAP	330.7607773	-0.027159496	0.073075	-0.371668	0.71014	0.805291
17910	RGMB	310.7002523	-0.027180629	0.12018	-0.226166	0.821072	0.898595
17975	B4GALT5	3425.775047	-0.027384178	0.129177	-0.211989	0.832115	0.907388
18168	XBP1	4324.058721	-0.02768716	0.161848	-0.171069	0.86417	0.932331
18449	GVIN1	214.5443966	-0.027718055	0.229798	-0.120619	0.903992	0.96044
18963	SYT16	1.886843031	-0.027799821	0.489202	-0.056827	0.954683	0.986803
17398	UNK	636.3269407	-0.028035923	0.081937	-0.342165	0.732227	0.824944
17897	SFRS12IP1	847.7895015	-0.028217168	0.123562	-0.228364	0.819363	0.897441
18968	OCM	0.613674068	-0.028251243	0.506676	-0.055758	0.955535	0.987423
18113	CKAP2	456.9620946	-0.028308281	0.156242	-0.181183	0.856224	0.926564
18794	CLLU1	2.249862472	-0.028371721	0.40344	-0.070325	0.943935	0.984467
18259	SCRN2	774.6071455	-0.028386397	0.18894	-0.15024	0.880575	0.945295
17609	TP53RK	402.0845788	-0.028418574	0.097331	-0.29198	0.770302	0.857442
18512	RNF133	1.799789292	-0.028433557	0.262586	-0.108283	0.913771	0.967525
17507	METTL6	197.8995579	-0.028595755	0.091106	-0.313875	0.753616	0.843756
18288	CA14	7.265086246	-0.028764359	0.197065	-0.145964	0.88395	0.947414
17557	STAMBP	784.2460151	-0.028791443	0.09552	-0.301416	0.763097	0.851937
18669	SHBG	8.486303517	-0.028825288	0.343516	-0.083912	0.933126	0.97971
18667	ADRA1A	10.42662677	-0.02885403	0.342524	-0.084239	0.932866	0.979542
18189	PTPN14	298.7714912	-0.029053695	0.176322	-0.164776	0.86912	0.936589
17964	C15orf21	26.87352845	-0.029692418	0.138927	-0.213727	0.83076	0.906439
17695	CASP6	278.2131197	-0.029757124	0.110793	-0.268583	0.788251	0.873156
17370	TCTN3	1469.525507	-0.029816021	0.085613	-0.348264	0.727642	0.8211
17915	RECQL	1139.374428	-0.029853731	0.132369	-0.225534	0.821564	0.898882
17859	OLA1	1774.223186	-0.030000469	0.127544	-0.235216	0.814041	0.893444
18258	PPP1R2P1	3.77972694	-0.030006166	0.199414	-0.150471	0.880393	0.945152
18186	IFIT2	857.8553914	-0.030055306	0.181273	-0.165801	0.868313	0.935874
17686	C16orf58	2253.979862	-0.030383714	0.112105	-0.271028	0.786369	0.871516
17891	SLC38A2	4888.213771	-0.030637291	0.133543	-0.229419	0.818544	0.896779

17561	RAB22A	1378.830981	-0.030657642	0.102128	-0.30019	0.764033	0.852788
18427	ELFN1	127.202787	-0.03092338	0.248996	-0.124192	0.901163	0.958577
17559	INTS5	609.0676778	-0.031034164	0.103154	-0.300852	0.763527	0.852321
17587	TRIOBP	1866.274008	-0.031037322	0.10467	-0.296526	0.766828	0.854665
17378	R3HDM1	561.4046638	-0.031080721	0.08972	-0.346418	0.729029	0.822286
18110	ɔC1001316	29.27210016	-0.031191402	0.172014	-0.18133	0.856108	0.92649
18034	PAQR7	622.5676724	-0.031270497	0.156969	-0.199214	0.842095	0.915266
17794	VCL	3392.145875	-0.031440909	0.127248	-0.247084	0.804843	0.886576
18043	OTUB2	56.33338918	-0.031607596	0.159903	-0.197667	0.843305	0.916041
18759	FOSB	2560.818535	-0.031613597	0.421535	-0.074996	0.940218	0.982315
18247	DZIP1L	131.4315428	-0.031630234	0.205524	-0.153901	0.877688	0.942764
17728	UBOX5	308.8116063	-0.03189057	0.122323	-0.260707	0.794318	0.87824
18610	SNAP25	151.9747873	-0.032200493	0.3349	-0.09615	0.923402	0.972574
17603	ZFYVE27	807.6064837	-0.032253492	0.109999	-0.293216	0.769357	0.856682
18780	LMO3	23.74089819	-0.032285827	0.446689	-0.072278	0.942381	0.983578
17997	FEM1C	1551.372708	-0.03245821	0.15739	-0.206228	0.836613	0.911177
17585	CAPN1	3190.930733	-0.032687456	0.110112	-0.296858	0.766575	0.854458
18989	NAP1L6	5.501904884	-0.032693815	0.621576	-0.052598	0.958052	0.988929
17465	SETD5	1376.568592	-0.03291123	0.101863	-0.323092	0.746625	0.837939
18089	NBPF15	314.670744	-0.033121386	0.176862	-0.187273	0.851447	0.922617
17821	DAK	785.7610672	-0.033156951	0.136988	-0.242043	0.808747	0.889526
18096	COX6A1	3149.136769	-0.033187161	0.180045	-0.184327	0.853757	0.924762
19073	LOC44117	1.022192457	-0.03336971	0.797087	-0.041865	0.966607	0.989949
17125	CLCC1	889.1951705	-0.03358685	0.083058	-0.40438	0.685933	0.785108
17993	MYO1D	1893.174077	-0.033612434	0.162621	-0.206692	0.836251	0.910966
17537	CALM3	7746.25224	-0.033839311	0.110475	-0.306307	0.759371	0.848744
17688	BCL2L1	4459.774422	-0.033846387	0.12506	-0.270641	0.786667	0.871698
18543	SVEP1	463.1874634	-0.034012054	0.331792	-0.10251	0.918352	0.97075
18605	SOX11	146.8493059	-0.034040034	0.347204	-0.09804	0.9219	0.972284
17787	NCKIPSD	726.503511	-0.034048862	0.137389	-0.247828	0.804268	0.886291
17872	CRLS1	1344.945826	-0.034071625	0.146523	-0.232535	0.816123	0.895077
18381	GPC6	1533.987406	-0.034085662	0.25792	-0.132156	0.894861	0.954255
18768	SORCS2	529.0913822	-0.034224454	0.461519	-0.074156	0.940886	0.982489
18005	TPK1	234.8311	-0.034258499	0.167033	-0.2051	0.837494	0.911731
17405	TMBIM1	5454.705745	-0.034340504	0.101119	-0.339606	0.734153	0.826781
18048	SNHG8	683.791543	-0.034371924	0.1745	-0.196974	0.843848	0.916459
17021	SNX11	533.4984864	-0.034577139	0.079274	-0.436175	0.66271	0.763241
17486	POLA1	330.7419132	-0.034579324	0.108905	-0.317519	0.75085	0.841668
17852	ZNF841	265.2907631	-0.034657695	0.147072	-0.235651	0.813703	0.893435
18090	PLSCR1	1905.57115	-0.03471149	0.185559	-0.187065	0.85161	0.922742
18092	CD55	828.4792372	-0.034884823	0.18692	-0.18663	0.851951	0.923054
17908	C9orf9	136.0626142	-0.034888881	0.154127	-0.226365	0.820918	0.898504
17687	TARBP1	485.4885886	-0.034977559	0.129084	-0.270967	0.786416	0.871518
17937	FLNB	6460.366333	-0.035045984	0.158931	-0.220511	0.825473	0.902051
17648	TTL	757.210768	-0.035195337	0.125717	-0.279956	0.779511	0.865775
17361	DENR	1322.640937	-0.035221518	0.100576	-0.350199	0.726189	0.819885
17498	NUDCD2	373.5545001	-0.035264483	0.111935	-0.315044	0.752728	0.843172

17195	PITRM1	1521.762934	-0.035287054	0.090789	-0.38867	0.69752	0.79512
18418	KLHL10	1.378987525	-0.035356927	0.281096	-0.125782	0.899904	0.957706
16819	TRIM27	1076.745062	-0.035480243	0.073032	-0.485818	0.627096	0.730823
18153	ATP6V0B	2776.91932	-0.035795763	0.205745	-0.173982	0.86188	0.930629
17459	DHRS7B	569.2983516	-0.035974801	0.110849	-0.324538	0.745531	0.836998
17448	CCNY	2259.188713	-0.035975588	0.109986	-0.327092	0.743599	0.835355
16963	SDF2	655.3739369	-0.036052339	0.080258	-0.449206	0.653283	0.754878
17775	DHFR	252.36449	-0.036057603	0.143498	-0.251275	0.801601	0.883949
17016	ZBED4	433.3203092	-0.036320818	0.083176	-0.436676	0.662346	0.762967
18515	ABCA6	67.54233736	-0.036340892	0.337811	-0.107578	0.914331	0.967909
17438	CUL7	1255.355684	-0.036345484	0.110115	-0.330068	0.741349	0.833305
17407	USP31	545.6531776	-0.036530583	0.107619	-0.339443	0.734276	0.826825
17197	TIMM22	446.2283859	-0.036537007	0.094141	-0.388108	0.697936	0.795501
17726	HAUS6	466.0757421	-0.036707046	0.140256	-0.261715	0.793541	0.877479
17712	ZBTB38	3360.850517	-0.03675297	0.13901	-0.264391	0.791478	0.87589
18428	CDCA2	489.1881995	-0.036887912	0.297209	-0.124114	0.901225	0.958591
17182	LSM6	235.1607387	-0.037002974	0.094662	-0.390894	0.695875	0.793846
17306	VEZT	1430.437097	-0.037019981	0.101261	-0.36559	0.714671	0.809445
16751	ZNF3	601.9306971	-0.037096037	0.073545	-0.5044	0.61398	0.718443
16936	NCL	9242.820968	-0.037434295	0.082431	-0.454128	0.649736	0.751977
18825	AKR1B10	112.2926995	-0.037499804	0.622813	-0.06021	0.951988	0.986718
17463	MECR	331.9032563	-0.037507807	0.115991	-0.323368	0.746417	0.837801
17905	C3orf71	22.55543266	-0.037522342	0.164942	-0.227488	0.820045	0.897721
18570	GABBR2	8.107310131	-0.037612258	0.378566	-0.099354	0.920857	0.97193
16933	RPP38	230.4538048	-0.037767141	0.082993	-0.455062	0.649065	0.75142
17735	LIPT2	40.17642986	-0.037825412	0.145672	-0.259661	0.795125	0.878785
17406	CSNK1A1	5634.347402	-0.037951226	0.111803	-0.339447	0.734273	0.826825
17391	BRPF1	371.7980274	-0.038080658	0.110753	-0.343833	0.730972	0.823862
17953	ACN9	211.7612867	-0.03808247	0.176089	-0.216268	0.828779	0.904806
17344	TCEAL4	1944.276068	-0.038089085	0.107129	-0.355543	0.722183	0.816162
17460	ANKRD52	1174.161506	-0.038090557	0.117505	-0.324162	0.745816	0.837222
18149	TOP1P2	5.843121993	-0.03829002	0.218307	-0.175395	0.860769	0.929634
17397	STK16	812.9674738	-0.038305096	0.111865	-0.342423	0.732033	0.824773
17037	C17orf63	749.2115086	-0.038418274	0.088846	-0.432416	0.665439	0.765585
17562	FLJ90757	714.0152292	-0.038476751	0.128205	-0.300119	0.764086	0.852799
18156	DNAH10	25.16711208	-0.038616022	0.223081	-0.173103	0.86257	0.931221
17145	TYW1	543.187314	-0.038789371	0.096973	-0.400003	0.689154	0.787875
17143	WHSC1	1017.121649	-0.039267344	0.098062	-0.400433	0.688837	0.787604
17901	RASA4	321.0238062	-0.039621639	0.173837	-0.227924	0.819705	0.89755
17729	ZNF277	597.4960772	-0.039714063	0.152384	-0.260619	0.794386	0.878265
17141	BUB3	1408.133589	-0.03972534	0.099108	-0.400828	0.688547	0.787364
17492	RINT1	586.9645041	-0.039737396	0.125586	-0.316416	0.751687	0.842317
18553	LRRIQ4	0.978259889	-0.040008127	0.393683	-0.101625	0.919054	0.970766
17264	DGCR2	2850.193041	-0.040078856	0.107003	-0.374559	0.707988	0.803799
17530	MFSD1	1848.997448	-0.040112958	0.130407	-0.307597	0.758389	0.847985
16919	PHF5A	408.8407524	-0.040137696	0.087327	-0.459627	0.645784	0.748154
18021	MRVI1	879.9133134	-0.040491977	0.200499	-0.201956	0.839951	0.913594

18403	PAX9	4.052928075	-0.04051822	0.316652	-0.127958	0.898182	0.956652
17774	HYAL2	1947.987326	-0.040731781	0.161742	-0.251832	0.801171	0.883524
17241	PHTF1	354.704172	-0.04074468	0.107633	-0.378551	0.705021	0.801527
17983	DBP	354.8861607	-0.040939213	0.197132	-0.207674	0.835484	0.910655
18323	NRCAM	1165.157504	-0.041096768	0.29243	-0.140535	0.888237	0.950219
16713	RPA1	1569.915308	-0.041116877	0.079652	-0.516206	0.605711	0.710377
17493	KIAA0195	1793.603948	-0.041343281	0.130727	-0.316257	0.751807	0.842404
16864	SNAP47	553.2260697	-0.041475445	0.08729	-0.475147	0.634682	0.73769
17656	NDUFA1	2173.957022	-0.041488179	0.149094	-0.278269	0.780806	0.86682
17898	USP43	95.69069816	-0.041530214	0.181931	-0.228274	0.819433	0.897441
17478	IPO4	824.8809189	-0.041531285	0.130095	-0.319238	0.749546	0.84054
16862	TMEM62	415.1837023	-0.041577084	0.087384	-0.475798	0.634218	0.737238
18061	ZBTB47	500.7233144	-0.041791968	0.215209	-0.194192	0.846025	0.918163
17255	SPAG7	912.9404967	-0.041882231	0.111416	-0.375908	0.706985	0.803108
17638	SIPA1L2	1002.201994	-0.041914141	0.148443	-0.282358	0.777669	0.864174
17757	KIFC3	1253.963185	-0.042082541	0.164891	-0.255215	0.798557	0.881485
18072	AOC2	23.31205451	-0.042166933	0.21932	-0.192262	0.847537	0.919213
17427	ATP5H	2882.760194	-0.042396709	0.12738	-0.332837	0.739257	0.831479
16594	ANAPC7	901.4121929	-0.042528944	0.0774	-0.549469	0.582684	0.688272
17317	MED11	317.4872143	-0.042708659	0.117877	-0.362315	0.717116	0.811606
17861	HDAC9	141.9413242	-0.042750622	0.181924	-0.234991	0.814215	0.893523
18266	SERHL2	36.5256473	-0.043030701	0.287964	-0.149431	0.881214	0.945618
17553	C9orf119	314.0467377	-0.043115579	0.142667	-0.302211	0.762491	0.851455
17972	FBXL22	14.0655326	-0.043193544	0.203054	-0.21272	0.831545	0.906918
17943	SLFN5	670.9597552	-0.043202169	0.196956	-0.21935	0.826378	0.902738
17111	BAT2L2	3885.123097	-0.043266901	0.105797	-0.40896	0.682569	0.781897
17334	FBXW7	410.5723894	-0.043484478	0.120848	-0.359828	0.718976	0.813015
17102	SMARCA4	2030.370594	-0.043777536	0.106156	-0.412387	0.680056	0.779428
17212	EHD4	1633.57975	-0.043846899	0.113795	-0.385316	0.700003	0.797163
17013	LRRC47	973.3734893	-0.043982567	0.100637	-0.437043	0.66208	0.762795
18187	FATE1	33.03978647	-0.043999733	0.266402	-0.165163	0.868816	0.936313
16883	PSMD1	1802.303139	-0.044023866	0.093897	-0.468853	0.639175	0.742076
18372	MIR17HG	25.82019419	-0.044113058	0.330051	-0.133655	0.893675	0.953458
17874	EPS8L2	3492.608919	-0.044153204	0.190323	-0.231991	0.816545	0.89544
17240	PGAM1	6702.30428	-0.044213093	0.116685	-0.378909	0.704755	0.801271
16920	C15orf57	459.0451339	-0.044321008	0.096466	-0.459445	0.645914	0.748228
17792	RRP7B	78.13227905	-0.044440502	0.179763	-0.247217	0.80474	0.886568
16954	CSE1L	1832.762862	-0.045015087	0.100115	-0.449636	0.652973	0.754655
16678	C4orf42	363.861941	-0.045096036	0.085733	-0.526005	0.598885	0.703846
17699	NR3C1	3524.43425	-0.045116367	0.16865	-0.267514	0.789073	0.87387
17816	LOC28559:	5.278151408	-0.04514197	0.185569	-0.243262	0.807802	0.888737
16556	DUSP12	212.1491989	-0.045311407	0.081269	-0.557548	0.577153	0.683304
17624	ZNF518B	262.9140985	-0.045524045	0.158179	-0.287802	0.773499	0.860267
17207	MEMO1	307.9605682	-0.045582122	0.118138	-0.385837	0.699618	0.796909
17254	RNF187	3812.113295	-0.045661779	0.121459	-0.375945	0.706958	0.803108
18309	ZBTB16	216.2003323	-0.045922824	0.322276	-0.142495	0.886689	0.94932
18367	PALM3	212.5144646	-0.046030898	0.340115	-0.135339	0.892344	0.952296

17824	DEPDC6	624.3475151	-0.046120357	0.1914	-0.240963	0.809583	0.890297
17887	C14orf19	3.57828009	-0.046281904	0.201585	-0.22959	0.81841	0.896733
17050	HEBP1	1108.492339	-0.046292944	0.108338	-0.427303	0.669159	0.769278
16584	EIF2B5	964.1189883	-0.046311475	0.083971	-0.551515	0.581281	0.687029
16906	ZBTB2	361.8523376	-0.046347956	0.099946	-0.46373	0.642841	0.745317
17644	C1orf103	342.8728031	-0.046484065	0.165143	-0.281477	0.778344	0.864675
18202	IFI44L	1020.757105	-0.046715436	0.289705	-0.161252	0.871895	0.938942
16826	DHX38	1289.421223	-0.046783874	0.096577	-0.48442	0.628088	0.731676
17722	TMEM120I	421.4203469	-0.046824085	0.178643	-0.26211	0.793237	0.877341
17731	C20orf202	18.36119444	-0.046964678	0.180428	-0.260295	0.794636	0.878442
17431	HDAC6	1849.518413	-0.047057127	0.141969	-0.331461	0.740297	0.832457
17198	ZNF672	777.4438441	-0.047123991	0.121571	-0.387624	0.698294	0.795863
17054	ZKSCAN4	103.2942654	-0.047235573	0.110693	-0.426727	0.669578	0.769579
17675	GPRC5C	2258.930689	-0.047293171	0.172519	-0.274133	0.783982	0.869411
17218	CD47	2137.909797	-0.047373253	0.123119	-0.384778	0.700402	0.797339
17253	C21orf45	144.6692563	-0.047418088	0.126134	-0.375934	0.706966	0.803108
17411	CANX	28923.04117	-0.047555915	0.140251	-0.339078	0.734551	0.826945
17840	IRAK3	368.2534755	-0.047724393	0.201413	-0.236947	0.812698	0.89287
17634	WASH2P	345.4849404	-0.047903188	0.169143	-0.283211	0.777015	0.863688
18009	ANGPTL2	2226.875511	-0.04807712	0.236342	-0.203422	0.838805	0.912703
17520	RCL1	367.871341	-0.048172431	0.154907	-0.310977	0.755818	0.845593
16839	MRPS16	1468.509754	-0.04819278	0.100317	-0.480403	0.630941	0.73443
16797	UNC45A	1654.567989	-0.048254122	0.098326	-0.490754	0.6236	0.727701
18706	NTNG1	15.20100596	-0.04830094	0.60148	-0.080303	0.935996	0.980779
16955	CTPS2	440.14744	-0.048756565	0.108365	-0.449929	0.652762	0.754655
17925	EMID1	135.1285662	-0.048946818	0.218935	-0.223568	0.823094	0.900054
17984	S3-LOC44(	55.63245998	-0.049268245	0.237376	-0.207554	0.835577	0.910707
17389	DOM3Z	308.3045463	-0.049357866	0.143502	-0.343952	0.730882	0.823808
17424	TTC26	145.0345217	-0.049574471	0.14806	-0.334828	0.737755	0.829932
17515	C21orf119	71.57991848	-0.049726844	0.159455	-0.311855	0.75515	0.844991
18749	CYP2A6	0.425445999	-0.049929743	0.655778	-0.076138	0.939309	0.981934
16850	ABHD15	470.461102	-0.050042812	0.104625	-0.478306	0.632432	0.735686
16914	GTF2H4	355.9992607	-0.050136536	0.108804	-0.460798	0.644944	0.747401
16890	TOMM5	888.3024604	-0.050340033	0.107675	-0.467517	0.64013	0.742877
16699	LRRFIP1	3222.721386	-0.050368822	0.096931	-0.519636	0.603317	0.708163
18192	LAMC3	453.3429036	-0.050379174	0.307444	-0.163865	0.869838	0.937208
16925	TCERG1	865.1971833	-0.050422391	0.109894	-0.458828	0.646358	0.748553
17671	IL7	100.8096214	-0.050443387	0.183393	-0.275056	0.783273	0.868772
18638	)N1-GTF2/	11.19845742	-0.050557205	0.560215	-0.090246	0.928092	0.976045
17475	KCTD3	2766.783139	-0.050563675	0.158146	-0.319727	0.749175	0.840319
16668	CWC15	999.3489925	-0.050575946	0.095557	-0.529273	0.596616	0.7016
18751	MAB21L2	0.404040234	-0.050660158	0.665915	-0.076076	0.939359	0.981934
18710	GNAT1	0.352129351	-0.050660162	0.639996	-0.079157	0.936908	0.981508
18753	TERC	0.314836651	-0.050660209	0.66637	-0.076024	0.9394	0.981934
18752	MYOG	0.314279048	-0.05066021	0.666436	-0.076017	0.939406	0.981934
18771	ZBTB8B	0.330962134	-0.050660211	0.681873	-0.074296	0.940775	0.982489
18761	PPP3R2	0.310481217	-0.050660217	0.674845	-0.075069	0.94016	0.982315

18827	AQP12B	0.356973796	-0.050660297	0.786936	-0.064377	0.94867	0.986718
18886	LOC91948	0.39548438	-0.050660356	0.884574	-0.057271	0.954329	0.986718
18914	PAPOLB	0.292617198	-0.050660414	0.840911	-0.060245	0.951961	0.986718
18944	TAS2R46	0.213064683	-0.050660436	0.797642	-0.063513	0.949358	0.986718
19132	OR6W1P	0.231283049	-0.050660726	1.007091	-0.050304	0.95988	0.989949
19050	HESRG	0.157966906	-0.05066109	1.130833	-0.0448	0.964267	0.989949
19016	CACNG2	0.170562997	-0.050661242	1.219807	-0.041532	0.966872	0.989949
19060	KRT37	0.137331159	-0.050661327	1.219715	-0.041535	0.966869	0.989949
19235	PSAPL1	0.337762221	-0.050661348	1.46853	-0.034498	0.97248	0.990995
19155	PWRN2	0.168575743	-0.050661488	1.326838	-0.038182	0.969542	0.989949
18999	AOX2P	0.161224244	-0.050661492	1.307896	-0.038735	0.969102	0.989949
19223	HS3ST6	0.303867195	-0.050661498	1.42509	-0.03555	0.971641	0.990696
19144	PDILT	0.16065028	-0.0506615	1.311024	-0.038643	0.969175	0.989949
19224	OR2L1P	0.180476248	-0.050661732	1.425026	-0.035551	0.97164	0.990696
19227	PRSS33	0.136299872	-0.050661861	1.429525	-0.03544	0.971729	0.990697
19225	C6orf94	0.135699203	-0.05066187	1.43271	-0.035361	0.971792	0.990697
19230	C20orf173	0.115343663	-0.050661959	1.443125	-0.035106	0.971995	0.990748
19259	OR11A1	0.142561689	-0.050662707	1.70997	-0.029628	0.976364	0.993693
19260	RNU11	0.137579338	-0.050662717	1.712894	-0.029577	0.976404	0.993693
19266	KRT71	0.097428222	-0.050663034	1.754674	-0.028873	0.976966	0.993972
19265	HTR1A	0.096479824	-0.05066304	1.756618	-0.028841	0.976991	0.993972
19267	OR51I1	0.094880771	-0.050663052	1.759947	-0.028787	0.977035	0.993972
19276	BAGE	0.116304756	-0.050663533	1.91666	-0.026433	0.978912	0.994878
19289	CCL27	0.097235947	-0.050663767	1.949205	-0.025992	0.979264	0.994878
19284	C8orf86	0.095820143	-0.050663781	1.952696	-0.025946	0.979301	0.994878
19395	SNORA21	0.080255666	-0.050664021	1.984142	-0.025534	0.979629	0.994878
19386	RNF151	0.080127288	-0.050664022	1.984539	-0.025529	0.979633	0.994878
19424	XKRY2	0.081523707	-0.050665073	2.222987	-0.022791	0.981817	0.994878
19271	AMBN	0.079006163	-0.050665118	2.232531	-0.022694	0.981894	0.994878
19300	EPGN	0.078807613	-0.050665121	2.2333	-0.022686	0.981901	0.994878
19402	SPAG11B	0.077817485	-0.050665139	2.237169	-0.022647	0.981932	0.994878
19410	SSX4	0.07756287	-0.050665144	2.238174	-0.022637	0.98194	0.994878
19335	LOC33858[illegible]	0.076800997	-0.050665158	2.241206	-0.022606	0.981964	0.994878
19320	HSD3B1	0.076134092	-0.050665171	2.24389	-0.022579	0.981986	0.994878
19379	PPY2	0.082949209	-0.050666556	2.521381	-0.020095	0.983968	0.994878
19429	ZNRF4	0.076100261	-0.05066672	2.552248	-0.019852	0.984162	0.994878
19351	ODF4	0.075644056	-0.050666732	2.554587	-0.019834	0.984176	0.994878
19291	CELA3B	0.076053392	-0.050666733	2.554735	-0.019832	0.984177	0.994878
19400	SNORA5C	0.061066445	-0.05066735	2.631558	-0.019254	0.984639	0.994878
19298	DEFB131	0.060798506	-0.05066736	2.633373	-0.01924	0.984649	0.994878
19292	CHRNB3	0.05889044	-0.050667435	2.646602	-0.019144	0.984726	0.994878
19397	SNORA3	0.058818828	-0.050667438	2.64711	-0.019141	0.984729	0.994878
19288	CCDC70	0.058584254	-0.050667448	2.648776	-0.019129	0.984738	0.994878
19414	TFAP2D	0.058468235	-0.050667452	2.649604	-0.019123	0.984743	0.994878
19322	HTR5A	0.057883282	-0.050667476	2.653809	-0.019092	0.984767	0.994878
19382	PRSS38	0.057770393	-0.050667481	2.654626	-0.019086	0.984772	0.994878
19411	TAS2R60	0.05708424	-0.05066751	2.659641	-0.019051	0.984801	0.994878

19352	OR10G2	0.056985858	-0.050667514	2.660366	-0.019045	0.984805	0.994878
19427	ZNF645	0.056624077	-0.050667529	2.663048	-0.019026	0.98482	0.994878
19423	VWC2L	0.056413291	-0.050667538	2.66462	-0.019015	0.984829	0.994878
19336	LOC38842ε	0.056208618	-0.050667547	2.666154	-0.019004	0.984838	0.994878
19272	AMELX	0.141837343	-0.050668056	2.91407	-0.017387	0.986128	0.994878
19304	FEZF2	0.12771144	-0.050668546	2.91407	-0.017388	0.986127	0.994878
19403	SPAM1	0.076945531	-0.050668791	2.91407	-0.017388	0.986127	0.994878
19399	SNORA49	0.080697521	-0.050668791	2.91407	-0.017388	0.986127	0.994878
19353	OR10G3	0.076714595	-0.050668791	2.91407	-0.017388	0.986127	0.994878
19360	OR2Z1	0.076128921	-0.050668791	2.91407	-0.017388	0.986127	0.994878
19307	GAB4	0.077796117	-0.050669036	2.91407	-0.017388	0.986127	0.994878
19390	SDC4P	0.057291876	-0.050669036	2.91407	-0.017388	0.986127	0.994878
19419	TRIM49L	0.058013413	-0.050669036	2.91407	-0.017388	0.986127	0.994878
19381	PRAMEF18	0.055144776	-0.050669036	2.91407	-0.017388	0.986127	0.994878
19376	PMCHL2	0.057853085	-0.050669036	2.91407	-0.017388	0.986127	0.994878
19308	GBX1	0.055884655	-0.050669036	2.91407	-0.017388	0.986127	0.994878
19281	C19orf30	0.037939303	-0.050669281	2.91407	-0.017388	0.986127	0.994878
19373	PAX4	0.036522666	-0.050669281	2.91407	-0.017388	0.986127	0.994878
19374	PER4	0.039588499	-0.050669281	2.91407	-0.017388	0.986127	0.994878
19313	GRXCR2	0.039023232	-0.050669281	2.91407	-0.017388	0.986127	0.994878
19319	HNRNPCL1	0.035901018	-0.050669281	2.91407	-0.017388	0.986127	0.994878
19321	HTR1E	0.04110683	-0.050669281	2.91407	-0.017388	0.986127	0.994878
19367	OR5H2	0.038852112	-0.050669281	2.91407	-0.017388	0.986127	0.994878
19404	SPATA16	0.038456688	-0.050669281	2.91407	-0.017388	0.986127	0.994878
19372	PAGE2	0.040411543	-0.050669281	2.91407	-0.017388	0.986127	0.994878
19384	RBMXL3	0.036279039	-0.050669281	2.91407	-0.017388	0.986127	0.994878
19306	FLJ46361	0.038402317	-0.050669281	2.91407	-0.017388	0.986127	0.994878
19316	HMGB3L1	0.040855179	-0.050669281	2.91407	-0.017388	0.986127	0.994878
19341	MAGEB4	0.037203934	-0.050669281	2.91407	-0.017388	0.986127	0.994878
19350	NPSR1	0.038894471	-0.050669281	2.91407	-0.017388	0.986127	0.994878
19362	OR4N4	0.037029123	-0.050669281	2.91407	-0.017388	0.986127	0.994878
19368	OR6B2	0.039822624	-0.050669281	2.91407	-0.017388	0.986127	0.994878
19394	SNORA16A	0.040979173	-0.050669281	2.91407	-0.017388	0.986127	0.994878
19409	SSX3	0.03731163	-0.050669281	2.91407	-0.017388	0.986127	0.994878
19413	TBC1D28	0.038182109	-0.050669281	2.91407	-0.017388	0.986127	0.994878
19287	CCDC105	0.037214426	-0.050669281	2.91407	-0.017388	0.986127	0.994878
19317	HMGB4	0.037319861	-0.050669281	2.91407	-0.017388	0.986127	0.994878
19318	HMX3	0.038788305	-0.050669281	2.91407	-0.017388	0.986127	0.994878
19327	KRT28	0.038109067	-0.050669281	2.91407	-0.017388	0.986127	0.994878
19331	LOC14962(	0.039286496	-0.050669281	2.91407	-0.017388	0.986127	0.994878
19340	MAGEB1	0.035470493	-0.050669281	2.91407	-0.017388	0.986127	0.994878
19344	CRNA0005	0.039103636	-0.050669281	2.91407	-0.017388	0.986127	0.994878
19355	OR1I1	0.036420151	-0.050669281	2.91407	-0.017388	0.986127	0.994878
19377	POTEC	0.037298793	-0.050669281	2.91407	-0.017388	0.986127	0.994878
19389	SAMD7	0.037214426	-0.050669281	2.91407	-0.017388	0.986127	0.994878
19407	SRY	0.038604052	-0.050669281	2.91407	-0.017388	0.986127	0.994878
19408	SSTR4	0.039167423	-0.050669281	2.91407	-0.017388	0.986127	0.994878

19744	GAGE8	0.019886289	-0.050669526	2.91407	-0.017388	0.986127	NA
19853	LALBA	0.018229699	-0.050669526	2.91407	-0.017388	0.986127	NA
19884	LOC340017	0.017774032	-0.050669526	2.91407	-0.017388	0.986127	NA
19925	NKX2-6	0.017973188	-0.050669526	2.91407	-0.017388	0.986127	NA
19603	ACTL7A	0.017951967	-0.050669526	2.91407	-0.017388	0.986127	NA
19608	ARSH	0.018725566	-0.050669526	2.91407	-0.017388	0.986127	NA
19619	C12orf12	0.017736319	-0.050669526	2.91407	-0.017388	0.986127	NA
19620	C14orf177	0.01798535	-0.050669526	2.91407	-0.017388	0.986127	NA
19623	C15orf32	0.018280013	-0.050669526	2.91407	-0.017388	0.986127	NA
19631	C20orf79	0.019777014	-0.050669526	2.91407	-0.017388	0.986127	NA
19633	C21orf131	0.01798535	-0.050669526	2.91407	-0.017388	0.986127	NA
19636	C22orf33	0.019160126	-0.050669526	2.91407	-0.017388	0.986127	NA
19637	C22orf42	0.020281315	-0.050669526	2.91407	-0.017388	0.986127	NA
19638	C2orf27B	0.018343547	-0.050669526	2.91407	-0.017388	0.986127	NA
19656	CGB1	0.019025018	-0.050669526	2.91407	-0.017388	0.986127	NA
19661	CRYGB	0.01829716	-0.050669526	2.91407	-0.017388	0.986127	NA
19694	DEFB109P1	0.020609081	-0.050669526	2.91407	-0.017388	0.986127	NA
19712	KFZp434L1	0.01798535	-0.050669526	2.91407	-0.017388	0.986127	NA
19734	FTLP10	0.018144159	-0.050669526	2.91407	-0.017388	0.986127	NA
19737	GAGE10	0.017745983	-0.050669526	2.91407	-0.017388	0.986127	NA
19746	GALP	0.019518323	-0.050669526	2.91407	-0.017388	0.986127	NA
19748	GCG	0.019170341	-0.050669526	2.91407	-0.017388	0.986127	NA
19749	GDEP	0.018144159	-0.050669526	2.91407	-0.017388	0.986127	NA
19750	GDF2	0.017194806	-0.050669526	2.91407	-0.017388	0.986127	NA
19753	GML	0.020609081	-0.050669526	2.91407	-0.017388	0.986127	NA
19754	GNRH2	0.01907054	-0.050669526	2.91407	-0.017388	0.986127	NA
19759	GPX5	0.01798535	-0.050669526	2.91407	-0.017388	0.986127	NA
19764	GUCA2A	0.019335965	-0.050669526	2.91407	-0.017388	0.986127	NA
19772	HDGFL1	0.019295964	-0.050669526	2.91407	-0.017388	0.986127	NA
19779	HTN1	0.019340908	-0.050669526	2.91407	-0.017388	0.986127	NA
19784	IFNA17	0.01798535	-0.050669526	2.91407	-0.017388	0.986127	NA
19791	IL22	0.0192929	-0.050669526	2.91407	-0.017388	0.986127	NA
19792	IL28A	0.01907054	-0.050669526	2.91407	-0.017388	0.986127	NA
19799	KNCN	0.019365615	-0.050669526	2.91407	-0.017388	0.986127	NA
19801	KRTAP10-1	0.01798535	-0.050669526	2.91407	-0.017388	0.986127	NA
19815	KRTAP15-1	0.017736319	-0.050669526	2.91407	-0.017388	0.986127	NA
19838	KRTAP4-3	0.019047992	-0.050669526	2.91407	-0.017388	0.986127	NA
19850	KRTAP9-8	0.01798535	-0.050669526	2.91407	-0.017388	0.986127	NA
19856	LCE2A	0.017745983	-0.050669526	2.91407	-0.017388	0.986127	NA
19863	LCE3E	0.019605585	-0.050669526	2.91407	-0.017388	0.986127	NA
19864	LCE4A	0.017736319	-0.050669526	2.91407	-0.017388	0.986127	NA
19876	LOC200726	0.017194806	-0.050669526	2.91407	-0.017388	0.986127	NA
19879	LOC285194	0.017194806	-0.050669526	2.91407	-0.017388	0.986127	NA
19891	LOC653544	0.018742511	-0.050669526	2.91407	-0.017388	0.986127	NA
19907	MRGPRX1	0.019365615	-0.050669526	2.91407	-0.017388	0.986127	NA
19921	NEUROD6	0.017194806	-0.050669526	2.91407	-0.017388	0.986127	NA
19930	ONECUT3	0.020422888	-0.050669526	2.91407	-0.017388	0.986127	NA

19940	OR10J1	0.01798535	-0.050669526	2.91407	-0.017388	0.986127	NA
19947	OR12D3	0.017194806	-0.050669526	2.91407	-0.017388	0.986127	NA
19949	OR13C3	0.019311634	-0.050669526	2.91407	-0.017388	0.986127	NA
19954	OR13H1	0.017951967	-0.050669526	2.91407	-0.017388	0.986127	NA
19961	OR1M1	0.017886404	-0.050669526	2.91407	-0.017388	0.986127	NA
19977	OR2T1	0.018887649	-0.050669526	2.91407	-0.017388	0.986127	NA
19984	OR4A15	0.019044178	-0.050669526	2.91407	-0.017388	0.986127	NA
20001	OR4K13	0.01798535	-0.050669526	2.91407	-0.017388	0.986127	NA
20002	OR4K14	0.01798535	-0.050669526	2.91407	-0.017388	0.986127	NA
20007	OR4M2	0.01798535	-0.050669526	2.91407	-0.017388	0.986127	NA
20013	OR51D1	0.018526863	-0.050669526	2.91407	-0.017388	0.986127	NA
20024	OR56A1	0.018144159	-0.050669526	2.91407	-0.017388	0.986127	NA
20025	OR56A4	0.017194806	-0.050669526	2.91407	-0.017388	0.986127	NA
20027	OR5A2	0.01798535	-0.050669526	2.91407	-0.017388	0.986127	NA
20039	OR5F1	0.01798535	-0.050669526	2.91407	-0.017388	0.986127	NA
20040	OR5H1	0.018369583	-0.050669526	2.91407	-0.017388	0.986127	NA
20043	OR5I1	0.017194806	-0.050669526	2.91407	-0.017388	0.986127	NA
20047	OR5L2	0.017194806	-0.050669526	2.91407	-0.017388	0.986127	NA
20048	OR5M10	0.017194806	-0.050669526	2.91407	-0.017388	0.986127	NA
20057	OR6B1	0.018603915	-0.050669526	2.91407	-0.017388	0.986127	NA
20058	OR6B3	0.018662226	-0.050669526	2.91407	-0.017388	0.986127	NA
20059	OR6C1	0.017194806	-0.050669526	2.91407	-0.017388	0.986127	NA
20067	OR6C76	0.018679204	-0.050669526	2.91407	-0.017388	0.986127	NA
20072	OR6N2	0.01798535	-0.050669526	2.91407	-0.017388	0.986127	NA
20076	OR6Y1	0.019145335	-0.050669526	2.91407	-0.017388	0.986127	NA
20080	OR7D4	0.018742511	-0.050669526	2.91407	-0.017388	0.986127	NA
20081	OR7E24	0.020093928	-0.050669526	2.91407	-0.017388	0.986127	NA
20085	OR8B4	0.01798535	-0.050669526	2.91407	-0.017388	0.986127	NA
20099	OR9A2	0.018343547	-0.050669526	2.91407	-0.017388	0.986127	NA
20121	PRAMEF6	0.017194806	-0.050669526	2.91407	-0.017388	0.986127	NA
20122	PRB4	0.0190584	-0.050669526	2.91407	-0.017388	0.986127	NA
20143	RGR	0.01913409	-0.050669526	2.91407	-0.017388	0.986127	NA
20160	SCARNA4	0.020013024	-0.050669526	2.91407	-0.017388	0.986127	NA
20166	SMR3A	0.0195757	-0.050669526	2.91407	-0.017388	0.986127	NA
20185	SNORA13	0.017745983	-0.050669526	2.91407	-0.017388	0.986127	NA
20199	SNORA42	0.019228816	-0.050669526	2.91407	-0.017388	0.986127	NA
20216	SNORA84	0.018603915	-0.050669526	2.91407	-0.017388	0.986127	NA
20436	SPANXN5	0.017886404	-0.050669526	2.91407	-0.017388	0.986127	NA
20459	TMEM225	0.017194806	-0.050669526	2.91407	-0.017388	0.986127	NA
20461	'MPRSS11l	0.017951967	-0.050669526	2.91407	-0.017388	0.986127	NA
20501	ZNF735	0.01798535	-0.050669526	2.91407	-0.017388	0.986127	NA
19896	ST-3TM1:	0.018763731	-0.050669526	2.91407	-0.017388	0.986127	NA
20468	TSPY1	0.01875157	-0.050669526	2.91407	-0.017388	0.986127	NA
19647	CARTPT	0.018766443	-0.050669526	2.91407	-0.017388	0.986127	NA
19834	KRTAP3-1	0.018536526	-0.050669526	2.91407	-0.017388	0.986127	NA
20104	PGLYRP3	0.018453589	-0.050669526	2.91407	-0.017388	0.986127	NA
17040	NCDN	594.6628019	-0.050850439	0.118177	-0.430289	0.666985	0.767229

17780	FRMD6	475.411509	-0.05099691	0.204454	-0.24943	0.803028	0.885273
17468	ZNF815	25.90761181	-0.051243244	0.158948	-0.32239	0.747157	0.838392
17951	OM121L10	9.312970655	-0.051316774	0.236668	-0.21683	0.828341	0.904503
18270	PTN	343.0818358	-0.051497001	0.345803	-0.14892	0.881617	0.945844
17505	GLIS2	1573.458601	-0.051830136	0.165007	-0.314109	0.753438	0.843682
17511	ARSG	154.2293541	-0.051879264	0.165859	-0.312791	0.75444	0.844447
17129	LUZP6	5007.384858	-0.052017856	0.128714	-0.404134	0.686114	0.785162
16995	MICALL1	566.358517	-0.052140896	0.118294	-0.440774	0.659377	0.760485
17828	NMUR1	58.27375047	-0.052392114	0.218012	-0.240318	0.810084	0.890647
17736	LIMS2	629.8620939	-0.052519888	0.202656	-0.259157	0.795514	0.879165
17387	HD1-EIF4I	874.8885338	-0.05260895	0.152756	-0.344398	0.730547	0.82355
18277	SAMD11	109.7241935	-0.052694333	0.357705	-0.147312	0.882886	0.946791
18033	C9orf163	8.881018222	-0.052749382	0.264591	-0.199362	0.841979	0.915191
16071	XPNPEP1	1273.625654	-0.052752552	0.076281	-0.691558	0.489215	0.596671
17377	TMEM175	371.4824919	-0.052865562	0.152437	-0.346803	0.728739	0.822007
15906	BMS1	1021.252359	-0.052870235	0.071355	-0.740947	0.458726	0.565309
17351	PCGF6	154.8650081	-0.052881736	0.149989	-0.352571	0.72441	0.818349
17194	PCNXL3	1079.869083	-0.052964296	0.136261	-0.388696	0.697501	0.79512
16705	MED24	1211.694828	-0.053069737	0.102578	-0.517359	0.604906	0.709773
17232	VAT1	6884.152379	-0.053403345	0.140184	-0.380951	0.703239	0.799918
16498	ZNFX1	1915.149944	-0.05341081	0.093058	-0.573952	0.566001	0.672456
18086	KANK4	31.00457547	-0.053514237	0.284645	-0.188003	0.850874	0.922098
17524	STS	901.8488438	-0.053563268	0.172986	-0.309639	0.756836	0.846538
16707	CHRAC1	793.9268123	-0.053676977	0.103855	-0.516844	0.605265	0.709986
16497	RABGEF1	1046.607559	-0.053691189	0.093546	-0.573957	0.565997	0.672456
18290	PCDHGB2	156.2105	-0.053742835	0.369428	-0.145476	0.884335	0.947723
16621	GEMIN5	687.9149906	-0.053743286	0.099027	-0.542714	0.587327	0.69263
17815	RNLS	415.1522612	-0.053789962	0.221008	-0.243384	0.807708	0.888682
16793	ZNF574	360.7329946	-0.054044528	0.109976	-0.491421	0.623129	0.727339
17541	SLC20A1	1267.863768	-0.054371851	0.178526	-0.304559	0.760702	0.850038
17616	LOC64453	251.9841555	-0.05440101	0.187529	-0.290094	0.771745	0.858687
16817	IGHMBP2	291.9814058	-0.054899348	0.112826	-0.486583	0.626554	0.730278
17340	FAIM	285.494173	-0.054977689	0.15353	-0.358091	0.720275	0.814205
17070	TAF6L	223.1693971	-0.055037718	0.130643	-0.421283	0.673548	0.773417
17109	MESDC1	553.7945313	-0.055214912	0.134812	-0.409569	0.682122	0.781477
16758	CIZ1	1483.374673	-0.055329184	0.110251	-0.501849	0.615774	0.720197
16977	LOC20003	424.7068855	-0.055501032	0.1249	-0.444365	0.656779	0.758271
17229	TIMM13	849.8993684	-0.055669733	0.145785	-0.381863	0.702563	0.799288
18079	ARSE	1663.307932	-0.055688214	0.292369	-0.190473	0.848939	0.920408
17532	PBX3	430.7712415	-0.055745865	0.181361	-0.307374	0.758558	0.848078
17660	ARPM1	36.84951222	-0.055913224	0.201437	-0.277571	0.781342	0.867218
18535	SLC2A7	0.477115544	-0.05595048	0.540927	-0.103434	0.917618	0.970393
17924	HRC	138.4350175	-0.056264706	0.251488	-0.223727	0.82297	0.899968
16457	LTA4H	2241.877169	-0.056329629	0.09622	-0.585428	0.55826	0.664912
18321	GTF2A1L	7.907417518	-0.056359511	0.400509	-0.14072	0.888091	0.950138
18363	CCDC78	50.25236453	-0.056467782	0.416403	-0.135609	0.892131	0.952276
18228	CMTM5	1.591029219	-0.056660459	0.363253	-0.155981	0.876048	0.941374

16469	PTPMT1	663.8875494	-0.056801076	0.097603	-0.581957	0.560595	0.667208
17723	C1orf133	48.06915558	-0.057182336	0.218298	-0.261947	0.793362	0.87743
16289	COPB2	3316.716788	-0.05738265	0.09087	-0.63148	0.527727	0.635028
16756	RFFL	823.962288	-0.057578326	0.114328	-0.503624	0.614526	0.718866
15602	SPPL3	1353.379562	-0.057662021	0.069075	-0.83478	0.403842	0.507352
16843	TARS2	555.37513	-0.057698287	0.120282	-0.479692	0.631446	0.734801
17277	LAMB2	6930.732859	-0.057748016	0.154805	-0.373038	0.70912	0.804471
17627	TIE1	1353.347477	-0.057764229	0.201023	-0.287351	0.773844	0.860505
17705	CATSPER2	32.33156424	-0.057769803	0.217187	-0.265991	0.790246	0.874872
17005	ZSWIM3	98.21155539	-0.05786557	0.131983	-0.438433	0.661073	0.761993
16526	MNT	553.0888235	-0.057904843	0.102464	-0.565124	0.571989	0.678396
15927	KCTD10	1973.642787	-0.058081161	0.079043	-0.734801	0.462461	0.569104
17024	HLA-E	15327.71288	-0.058098491	0.133337	-0.435727	0.663035	0.763402
17662	AURKAPS1	9.736714653	-0.058217689	0.210443	-0.276644	0.782054	0.867861
18085	LOC643677	6.894750549	-0.058266895	0.309193	-0.188448	0.850525	0.921822
18140	HAL	5.768561326	-0.058331069	0.330509	-0.176488	0.85991	0.929206
16737	NRBF2	427.4804619	-0.058370388	0.114556	-0.509537	0.610376	0.714822
18193	GPD1	1534.102046	-0.058639133	0.358058	-0.16377	0.869912	0.937237
16674	HARBI1	61.38863078	-0.058900833	0.111781	-0.526933	0.59824	0.703279
16145	TMEM69	471.6897268	-0.058934425	0.088331	-0.667203	0.504643	0.612667
17035	KCTD11	729.3386399	-0.058955669	0.136231	-0.432762	0.665188	0.765372
17670	PCDH12	1271.144902	-0.05913234	0.214576	-0.275577	0.782873	0.868426
17999	DLEC1	6.901381197	-0.059155194	0.287067	-0.206068	0.836738	0.911256
16766	UPF3B	224.8208962	-0.059194944	0.118558	-0.49929	0.617575	0.722002
18173	HNF4A	1002.907455	-0.05955401	0.350853	-0.169741	0.865214	0.93315
15428	SMARCC2	2613.590297	-0.059588957	0.067641	-0.88096	0.37834	0.480674
16875	SCAMP4	1157.615317	-0.059628011	0.126385	-0.471797	0.637071	0.739984
17338	PITPNM1	808.2824977	-0.059658185	0.166535	-0.358232	0.72017	0.814168
16601	YWHAQ	6473.543561	-0.059690671	0.108858	-0.548333	0.583463	0.688909
17809	HSN2	20.13397999	-0.059795982	0.244222	-0.244843	0.806578	0.887739
15881	WDR53	128.7469355	-0.060103355	0.08028	-0.748668	0.454057	0.560417
16720	GATAD1	1357.816997	-0.060120429	0.116726	-0.515057	0.606513	0.711021
16629	MAGED2	5160.214354	-0.060142884	0.111096	-0.541361	0.588259	0.693394
18082	HRASLS2	14.76575052	-0.060224663	0.317452	-0.189713	0.849534	0.920901
17429	PHKA2	2526.670496	-0.060235596	0.181282	-0.332275	0.739681	0.83186
18076	ALS2CR11	11.54810566	-0.060447595	0.314755	-0.192047	0.847706	0.919223
16916	ZNF653	118.6457366	-0.060703729	0.131831	-0.460465	0.645182	0.747589
16288	NUP107	704.6349316	-0.06072972	0.096153	-0.631593	0.527653	0.634978
17259	C15orf34	16.56216734	-0.060737704	0.161906	-0.375142	0.707555	0.803475
17732	PRCD	11.26285189	-0.060801465	0.233857	-0.259994	0.794868	0.87865
16540	EHMT1	814.4885797	-0.060814977	0.108097	-0.562596	0.57371	0.679919
16062	MSL1	1228.953055	-0.060922598	0.087747	-0.694298	0.487495	0.594907
16625	CKAP5	1662.943387	-0.061003643	0.112478	-0.542363	0.587569	0.692748
17179	LPCAT3	2717.580375	-0.061380546	0.156672	-0.391778	0.695222	0.793193
16918	TUFM	3929.753227	-0.061392128	0.133541	-0.459723	0.645715	0.748118
16886	RPS3A	5589.380355	-0.061490471	0.131298	-0.468326	0.639551	0.742381
17224	ABHD8	251.2251662	-0.061698227	0.161232	-0.382667	0.701966	0.798749

16059	SNRNP40	459.2259909	-0.061822118	0.088888	-0.695502	0.486741	0.594097
16646	C2orf29	1204.515378	-0.061944148	0.115691	-0.535428	0.592354	0.69743
16973	AHNAK	31291.59623	-0.062035478	0.139603	-0.444372	0.656774	0.758271
16673	DAG1	2480.293546	-0.062256906	0.118156	-0.526905	0.598259	0.703279
17020	MCL1	9839.891825	-0.062627574	0.143634	-0.436022	0.66282	0.763241
15810	GOSR2	1194.819543	-0.062843248	0.081256	-0.773394	0.439289	0.544624
17704	FAM69B	238.4671239	-0.062954783	0.236495	-0.2662	0.790085	0.874744
16663	RAB11B	1968.63365	-0.063063129	0.118842	-0.530646	0.595664	0.700691
17955	RICH2	374.2886435	-0.063119064	0.29227	-0.215961	0.829018	0.905017
16117	TRA2A	1090.956984	-0.063125857	0.093362	-0.676141	0.498951	0.606846
16628	MPRIP	2281.646399	-0.0632406	0.116728	-0.541775	0.587973	0.6931
16267	RBM15	155.0864104	-0.063702373	0.100142	-0.636118	0.524699	0.632239
16763	MTDH	3817.142602	-0.063761069	0.127565	-0.49983	0.617195	0.721687
17491	DTX3	542.1812354	-0.063985269	0.202092	-0.316614	0.751536	0.842197
16121	WDR75	744.135234	-0.064022909	0.094811	-0.675266	0.499507	0.607334
17540	INTS4L1	25.01134068	-0.064030617	0.209899	-0.305054	0.760325	0.849665
17120	ETFB	1780.303945	-0.064125504	0.157883	-0.406159	0.684626	0.783841
15433	DPF2	939.4433119	-0.064274695	0.073185	-0.878249	0.379808	0.482352
17523	ITGA7	764.8378388	-0.064301164	0.207385	-0.310057	0.756518	0.846231
16025	NFYB	682.2457332	-0.064350695	0.091484	-0.703413	0.481799	0.589313
17730	CDKN1C	391.7963687	-0.064363305	0.247132	-0.260441	0.794523	0.878367
17574	MURC	9.544334014	-0.064382409	0.215704	-0.298476	0.76534	0.853615
18614	TSIX	74.97818493	-0.064392825	0.674575	-0.095457	0.923952	0.972944
16342	TRIM8	2438.532151	-0.064548987	0.104887	-0.615412	0.538283	0.645663
16256	ACTL6A	695.3913719	-0.064976844	0.101788	-0.638355	0.523243	0.630903
18135	)C1001285	0.847705254	-0.065025656	0.366116	-0.17761	0.85903	0.928472
16686	FBXW4	1250.291638	-0.065131781	0.124855	-0.521659	0.601908	0.707059
16235	CIR1	904.1590394	-0.0651838	0.101059	-0.645009	0.518921	0.626509
17073	C10orf78	184.4027401	-0.065551398	0.156007	-0.420183	0.674351	0.774162
18112	SLC6A17	38.0590533	-0.065607251	0.36173	-0.181371	0.856077	0.92649
18360	CRTAC1	219.462667	-0.065627866	0.482651	-0.135974	0.891842	0.952074
17286	LETM2	58.46028912	-0.065757244	0.177037	-0.371432	0.710316	0.805397
16224	RBM19	618.6372164	-0.065803616	0.101446	-0.648658	0.516559	0.62408
17895	GPX3	56720.58238	-0.065825619	0.288393	-0.228249	0.819452	0.897441
15684	EBNA1BP2	844.2698111	-0.066107678	0.081431	-0.811823	0.416893	0.52101
18022	SCN9A	371.8759409	-0.066197665	0.327995	-0.201825	0.840053	0.913655
18279	C9orf70	0.677223764	-0.066221431	0.449741	-0.147244	0.88294	0.946797
16396	PGBD2	120.3326967	-0.066290002	0.110093	-0.602126	0.54709	0.654032
16132	DPH2	358.6552767	-0.066295996	0.098608	-0.672318	0.501382	0.609198
16019	TMEM5	366.1578232	-0.066459179	0.094203	-0.705488	0.480507	0.587952
16127	HDAC8	262.3134443	-0.066741023	0.099139	-0.673205	0.500817	0.608698
18103	HSD17B6	25.60038413	-0.06695429	0.364545	-0.183665	0.854276	0.924966
16773	PI4K2A	834.1522073	-0.067055992	0.134737	-0.497681	0.618709	0.722993
16385	C7orf11	435.7467455	-0.067118488	0.111175	-0.603717	0.546032	0.653205
16861	SLC39A6	1823.632272	-0.067140297	0.1411	-0.475835	0.634192	0.737238
16600	VAMP8	2483.773955	-0.067257026	0.122646	-0.548386	0.583427	0.688909
17200	ABCA2	1573.382978	-0.067272241	0.173665	-0.387367	0.698484	0.795988

17269	NT5C3	823.1331225	-0.067274233	0.179741	-0.374283	0.708193	0.803799
17320	AP1S3	474.8187418	-0.067281115	0.185835	-0.362047	0.717317	0.811763
16800	CCDC9	402.8134909	-0.067586969	0.137956	-0.489916	0.624193	0.728263
15833	TMEM50A	2860.098981	-0.06761872	0.088399	-0.764927	0.444315	0.550055
16637	MAP7D3	415.4337195	-0.06776294	0.125816	-0.538586	0.590172	0.695274
17191	FLJ39582	52.41903088	-0.067768981	0.174179	-0.389077	0.697219	0.794962
17470	SAMD9L	1206.436363	-0.06800962	0.211062	-0.322225	0.747282	0.838436
17940	GDF3	9.784706585	-0.068082143	0.309232	-0.220165	0.825742	0.902195
18356	TMEM14E	0.583810512	-0.068112648	0.49735	-0.136951	0.891069	0.952033
16252	TMEM110	136.9825218	-0.068122313	0.106485	-0.639735	0.522345	0.629983
15091	ERCC3	887.6659636	-0.068128393	0.069653	-0.978118	0.328016	0.426045
16750	GTF3C6	1127.571459	-0.068172937	0.134996	-0.504999	0.613559	0.717993
16250	ACTR3	4668.181708	-0.068215568	0.106533	-0.640321	0.521964	0.629601
15214	XRCC6	5371.237778	-0.068300186	0.072333	-0.944249	0.345042	0.444536
17173	HEG1	2786.787767	-0.068526224	0.174169	-0.393447	0.69399	0.792109
16125	WDR77	620.7417656	-0.068538647	0.101618	-0.674476	0.500008	0.607817
15175	SEC24C	2664.3792	-0.068548553	0.071795	-0.954787	0.339686	0.43876
16867	MPP1	1203.681118	-0.068568955	0.144688	-0.473911	0.635564	0.73854
16345	RRS1	345.3520962	-0.068702442	0.11168	-0.615172	0.538441	0.645701
16140	C22orf13	3054.210134	-0.0687648	0.102795	-0.668953	0.503525	0.611499
17368	SPOCK2	2194.394733	-0.068892332	0.197563	-0.348711	0.727306	0.820816
15551	APH1A	3725.701861	-0.069047294	0.081622	-0.845942	0.397585	0.50113
17400	TTLL9	8.354126835	-0.069602232	0.203731	-0.341638	0.732623	0.825296
15339	GATAD2A	1548.930249	-0.06995739	0.076974	-0.908847	0.363431	0.464411
16471	RGL2	1027.358617	-0.069964917	0.120371	-0.581242	0.561077	0.667699
16219	MANBA	1162.668914	-0.069985879	0.107788	-0.64929	0.516151	0.623741
16960	SNAPC4	233.5994386	-0.069995857	0.155612	-0.449811	0.652847	0.754655
17250	JAM3	1052.056513	-0.0704307	0.186689	-0.377261	0.705979	0.802208
18653	MYH13	3.857981642	-0.070458456	0.799772	-0.088098	0.929799	0.977032
17948	MUC5B	5.894011448	-0.070516677	0.323768	-0.2178	0.827585	0.903805
16178	GORAB	197.6321995	-0.07071519	0.107134	-0.66006	0.509215	0.616926
16244	PMS2L5	52.53759377	-0.070728971	0.110182	-0.641931	0.520918	0.628572
16184	ZNF74	185.4770403	-0.07080652	0.107657	-0.657704	0.510728	0.618561
15556	PARP16	334.1002226	-0.070855463	0.08382	-0.845333	0.397925	0.501421
17251	CCDC88C	407.3097327	-0.071063712	0.188409	-0.377178	0.706041	0.802221
15072	ZKSCAN5	401.8442213	-0.071079891	0.072135	-0.985372	0.324441	0.421933
16978	NPHP4	211.0826786	-0.071174628	0.160196	-0.444298	0.656827	0.758271
17256	PIK3R1	2480.266227	-0.0712934	0.189761	-0.375702	0.707139	0.803235
14808	ADIPOR1	2748.394331	-0.071369986	0.067148	-1.062874	0.287839	0.381006
17579	FOXD4L1	6.290074928	-0.071451691	0.240151	-0.297528	0.766064	0.854179
16026	KIAA1429	1320.264468	-0.071522571	0.101763	-0.702835	0.482159	0.589716
16200	TSPAN31	1534.525361	-0.071598826	0.109475	-0.654018	0.5131	0.62082
17417	EPDR1	1650.83352	-0.071716274	0.212527	-0.337445	0.735782	0.828034
15494	MMS19	957.6684618	-0.071716754	0.083053	-0.863507	0.387859	0.490668
15430	SPATA2	395.483843	-0.071828138	0.081572	-0.880546	0.378563	0.480896
17864	ITGA10	115.3559173	-0.071945747	0.306379	-0.234826	0.814344	0.893544
18294	NPHS1	12.48780045	-0.07198106	0.496773	-0.144897	0.884792	0.948005

15874	MRPL33	494.6215831	-0.072273501	0.09631	-0.750429	0.452996	0.559354
18622	ADCYAP1R	0.621624938	-0.072326913	0.774265	-0.093414	0.925575	0.974235
15297	RABIF	289.5567085	-0.072462514	0.078632	-0.921534	0.356772	0.457154
15682	RBM15B	1127.488984	-0.072500324	0.089253	-0.812305	0.416617	0.520731
16111	IRF2	936.7582126	-0.072512453	0.107097	-0.677072	0.49836	0.606316
15318	TH1L	1472.526169	-0.072675306	0.079349	-0.91589	0.359724	0.460305
18007	PCDHGA9	170.4310605	-0.072856112	0.355946	-0.204683	0.83782	0.912009
15950	TMEM39A	543.9575701	-0.072859024	0.100284	-0.726529	0.467514	0.574494
16948	TPM1	8767.071308	-0.073092897	0.162203	-0.450627	0.652259	0.754362
17023	TP53I11	2559.33009	-0.073209535	0.1679	-0.43603	0.662815	0.763241
18260	DSG1	1.64185989	-0.073328432	0.488487	-0.150113	0.880675	0.945351
15371	SRP68	1886.496689	-0.07348601	0.081756	-0.898844	0.368736	0.47021
16660	C1orf31	340.6928974	-0.073567469	0.138424	-0.531466	0.595096	0.700149
15737	ING1	319.6895367	-0.073580869	0.09274	-0.793413	0.427537	0.532513
16137	TOMM34	813.945678	-0.07368611	0.109911	-0.670418	0.502592	0.610479
17882	FLJ33360	5.029483973	-0.073948106	0.321114	-0.230286	0.817869	0.896441
15547	PIP4K2B	1878.209411	-0.074015543	0.087408	-0.846784	0.397116	0.500667
18299	C6orf176	16.00976284	-0.074213486	0.51599	-0.143827	0.885637	0.948651
16786	SGK223	349.3889982	-0.074251199	0.150561	-0.493162	0.621898	0.72619
16990	BTG2	2592.723829	-0.074390175	0.168378	-0.441805	0.65863	0.759848
17262	MAPK13	488.06904	-0.074415598	0.198422	-0.375037	0.707633	0.803517
17311	FAM111B	99.7784867	-0.07453619	0.204584	-0.364331	0.715611	0.810276
17753	ALDH3A1	28.12985921	-0.074596995	0.291767	-0.255673	0.798203	0.881199
14921	ABT1	492.2080723	-0.074625279	0.072454	-1.029963	0.303027	0.398046
15470	SELS	1017.44765	-0.074891312	0.086055	-0.870276	0.38415	0.48673
17961	FLJ14107	1.183361089	-0.074960872	0.349098	-0.214727	0.82998	0.905728
17586	AGAP8	29.43970063	-0.074962294	0.252824	-0.2965	0.766848	0.854665
16680	PTPN12	2507.549223	-0.074984684	0.143029	-0.524262	0.600097	0.705185
16482	ASMTL	619.754462	-0.075080009	0.130136	-0.576934	0.563984	0.670711
17181	NRARP	440.8304618	-0.075097242	0.192063	-0.391003	0.695795	0.7938
15686	ASXL1	1739.338159	-0.075243563	0.092758	-0.811186	0.417259	0.521401
16994	DAPK1	1517.810498	-0.075449514	0.171117	-0.440925	0.659268	0.760404
16718	TMEM48	208.3604079	-0.075579164	0.146641	-0.515404	0.606271	0.71087
17175	C17orf51	183.0441878	-0.075653477	0.192649	-0.392701	0.69454	0.792599
15277	NAIF1	162.2476104	-0.075728516	0.081759	-0.926241	0.354321	0.454608
16652	MAP1D	67.83862306	-0.075895911	0.142216	-0.533667	0.593572	0.698691
17099	C19orf25	491.3397874	-0.076126892	0.183897	-0.413964	0.6789	0.77824
17281	BEND5	177.6803239	-0.076148346	0.204656	-0.37208	0.709834	0.805083
18211	PPEF2	0.789833715	-0.076161104	0.47703	-0.159657	0.873152	0.939811
16311	CXorf40A	435.7369896	-0.076182016	0.12189	-0.625007	0.531966	0.639266
16525	PIN1	694.619597	-0.076913481	0.136064	-0.565276	0.571886	0.678338
15796	FNTB	630.7244592	-0.077148491	0.099295	-0.776964	0.43718	0.54249
16404	PASK	165.39068	-0.077206615	0.128614	-0.600298	0.548308	0.655168
14556	NRF1	325.9713725	-0.077225168	0.067216	-1.148918	0.25059	0.337442
16105	C18orf21	242.6540453	-0.077408598	0.113834	-0.680014	0.496495	0.604272
16101	MCRS1	965.0404757	-0.077492707	0.113788	-0.681028	0.495854	0.603641
15546	CTTN	3901.360264	-0.077580263	0.091604	-0.846912	0.397044	0.500609

16417	OSTC	1966.601071	-0.077659558	0.129746	-0.598549	0.549474	0.656041
17612	TMEM169	74.13553943	-0.07799844	0.268103	-0.290927	0.771107	0.858192
17741	LOC619207	27.62982684	-0.07852081	0.304811	-0.257605	0.796712	0.880241
15986	CCT4	3357.290076	-0.078584874	0.109747	-0.716055	0.473957	0.581136
16492	FBRSL1	465.5039049	-0.078618177	0.136772	-0.574813	0.565418	0.672008
17242	PLGLB2	43.97376732	-0.078642889	0.207807	-0.378442	0.705102	0.801572
16802	FAM22A	22.78488714	-0.07874352	0.161015	-0.489045	0.62481	0.728872
14762	KIAA0406	666.6734018	-0.078771561	0.072857	-1.081175	0.279619	0.371279
15886	PCIF1	927.4392657	-0.078839896	0.105556	-0.746905	0.455121	0.561553
15337	KPNB1	4022.381395	-0.078961186	0.086815	-0.909534	0.363068	0.464009
17288	C6orf26	45.14510975	-0.079100383	0.213205	-0.371006	0.710633	0.80571
16804	ARHGEF5	421.5328584	-0.079325014	0.162256	-0.488889	0.62492	0.72888
18530	SLC6A14	0.657758606	-0.079326921	0.76321	-0.103938	0.917218	0.970232
16651	CDC42EP3	581.1947723	-0.079451661	0.148728	-0.534207	0.593199	0.698294
17128	JD7-PLA2C	318.7233797	-0.079465658	0.196649	-0.404098	0.68614	0.785162
14966	MLX	1083.133384	-0.079512676	0.078135	-1.017629	0.308855	0.404507
15853	AKT1	2656.281816	-0.079527359	0.105128	-0.75648	0.449362	0.555565
16228	KLHDC3	1502.488321	-0.079535209	0.122943	-0.646926	0.51768	0.62528
15619	TLE3	1297.734632	-0.07957624	0.095885	-0.829914	0.406587	0.510245
18271	SLC6A20	111.6218455	-0.079797677	0.537673	-0.148413	0.882017	0.946172
18081	F10	283.7487193	-0.079818747	0.420119	-0.189991	0.849316	0.920715
17155	ELOVL6	344.4674004	-0.079833584	0.201023	-0.397137	0.691266	0.789828
14731	FAM192A	1241.871189	-0.079836101	0.073285	-1.089393	0.275981	0.367219
15368	GPATCH3	245.8542328	-0.079872617	0.088775	-0.899717	0.368271	0.469708
16550	EEF1A1P9	7429.577572	-0.079931532	0.143013	-0.558912	0.576222	0.682463
16486	PAN2	951.9670938	-0.07993412	0.138786	-0.575954	0.564646	0.671349
15844	SLC39A7	2631.174187	-0.079976159	0.105151	-0.760582	0.446907	0.552879
16323	TMEM99	296.5850661	-0.080065132	0.128774	-0.62175	0.534107	0.641366
17974	C21orf81	1.42415078	-0.080285349	0.378583	-0.212068	0.832054	0.907371
15861	ZDHHC7	1370.493981	-0.080496485	0.106587	-0.755215	0.45012	0.556257
14884	MAML1	957.096898	-0.080641214	0.077428	-1.041505	0.297641	0.391969
16577	PAM	7039.289614	-0.080765641	0.146175	-0.552526	0.580588	0.686499
17522	CPAMD8	133.4208764	-0.080821288	0.260654	-0.310071	0.756507	0.846231
15612	ALG8	717.8412982	-0.080970755	0.097291	-0.83225	0.405268	0.508786
15640	SGPL1	1917.988929	-0.081044729	0.098275	-0.824669	0.40956	0.513285
15855	MRPS27	1551.493458	-0.081117558	0.107255	-0.756305	0.449466	0.555625
15609	DPH5	396.6403678	-0.081244648	0.097533	-0.832996	0.404847	0.508386
16774	PLA2G16	2447.97271	-0.08131159	0.163385	-0.497669	0.618717	0.722993
16704	INCA1	19.44082964	-0.081354899	0.157135	-0.517739	0.604641	0.709504
16942	SLC16A2	502.8704248	-0.081455156	0.18037	-0.4516	0.651557	0.753817
18101	KCNK15	12.77391692	-0.08147613	0.443267	-0.183808	0.854164	0.924919
17081	CCHCR1	635.978247	-0.081585521	0.195613	-0.417075	0.676623	0.776447
18330	LOC146336	2.937458813	-0.081597929	0.584687	-0.139558	0.889009	0.950653
17209	SCN1B	397.083946	-0.081720501	0.211883	-0.385687	0.699729	0.796989
17661	DNAH5	134.1318597	-0.081891783	0.295733	-0.276911	0.781848	0.867731
14452	EWSR1	3250.601855	-0.081941934	0.069348	-1.181597	0.237366	0.321935
14850	ENSA	3010.14657	-0.082075188	0.07803	-1.051838	0.292874	0.386574

16729	RHOT2	1288.732671	-0.082133165	0.160212	-0.512654	0.608194	0.712607
16722	TMTC4	472.7205955	-0.082212249	0.159875	-0.514229	0.607092	0.711649
17186	TRPV1	148.6294547	-0.082221535	0.210691	-0.390247	0.696354	0.794236
16047	SPAST	449.1870302	-0.08253769	0.11825	-0.697992	0.485183	0.592638
17831	MME	4115.468165	-0.082632411	0.344919	-0.239571	0.810663	0.891134
15836	CASP9	307.6042901	-0.082876935	0.108536	-0.763592	0.445111	0.550936
16154	ALS2CR4	423.52275	-0.08288104	0.124611	-0.665119	0.505974	0.613941
15798	DNAJC5	1938.290268	-0.082954162	0.106909	-0.775933	0.437789	0.543142
16783	NFATC1	413.4799827	-0.083012141	0.167996	-0.494132	0.621213	0.72552
15912	FURIN	2614.182587	-0.083015402	0.112437	-0.738325	0.460317	0.567036
15851	HSP90AB1	22437.37963	-0.08335861	0.109945	-0.758183	0.448341	0.554409
15434	LIG3	374.5743476	-0.083459795	0.095026	-0.878284	0.379789	0.482352
16927	CCDC106	403.6788498	-0.083546038	0.182798	-0.45704	0.647642	0.749952
15709	ZNF202	238.5640061	-0.083589478	0.104348	-0.801065	0.423094	0.527918
17275	ITGA4	947.1748362	-0.083615828	0.223984	-0.373312	0.708916	0.804377
17046	AHR	2390.334838	-0.083960537	0.196053	-0.428255	0.668465	0.76864
15898	MYCBP	439.0886042	-0.084046136	0.113096	-0.743142	0.457396	0.563933
16316	SEC61A2	100.0704693	-0.084322956	0.135084	-0.624227	0.532479	0.639686
17290	SV2A	105.4567005	-0.084475695	0.228161	-0.370247	0.711199	0.806258
15030	M6PR	3575.223891	-0.084534786	0.084828	-0.996538	0.318989	0.416001
15119	WDR82	2117.749707	-0.084803885	0.087654	-0.967481	0.333304	0.432111
17445	ARL17B	2.331754713	-0.084877503	0.259118	-0.327563	0.743242	0.835098
16284	SGSM3	888.3528368	-0.084881548	0.134217	-0.632421	0.527112	0.634483
14349	C20orf43	2349.641777	-0.085015336	0.070094	-1.212873	0.225178	0.307562
16633	TCF7L1	321.9929594	-0.085141097	0.157508	-0.540552	0.588817	0.693885
17752	CRNA001(	108.9708682	-0.085252065	0.33291	-0.256081	0.797888	0.880994
17518	NR1H4	1032.658797	-0.085350178	0.273887	-0.311626	0.755325	0.845138
18606	SPHKAP	0.799648334	-0.085389758	0.871419	-0.097989	0.921941	0.972284
15396	ZNF696	172.4940461	-0.08581811	0.096268	-0.891449	0.372688	0.474478
16661	IGFBP7	23184.32847	-0.085900941	0.161822	-0.530836	0.595532	0.70062
15268	SENP1	425.8538616	-0.085913157	0.092467	-0.929127	0.352823	0.452976
16076	C20orf111	862.4216748	-0.085963081	0.124476	-0.6906	0.489817	0.59722
14978	USP4	1093.754578	-0.086062955	0.085063	-1.011753	0.311656	0.40785
18216	MAP3K15	18.85513184	-0.086106179	0.542079	-0.158844	0.873792	0.940228
16162	DTX3L	2147.967676	-0.086117497	0.129757	-0.663684	0.506892	0.61475
14791	EP400	1157.60962	-0.086178303	0.080476	-1.070856	0.284234	0.376686
17395	LRRC4B	54.30123528	-0.08636942	0.251883	-0.342895	0.731677	0.824469
15650	NDEL1	751.084516	-0.08639502	0.104989	-0.822896	0.410567	0.514219
16039	FUNDC2	2422.66497	-0.086497403	0.123744	-0.699	0.484552	0.592163
15756	RABL2A	280.0660916	-0.086617485	0.109948	-0.787801	0.430813	0.535946
15399	DCUN1D5	297.8150759	-0.086726602	0.097385	-0.890552	0.37317	0.474998
15332	CD99L2	2199.278623	-0.086889067	0.095445	-0.910359	0.362633	0.463624
16689	KIAA1908	124.0188591	-0.087158174	0.167326	-0.52089	0.602444	0.70759
16302	GNPDA1	1727.291557	-0.087244529	0.139266	-0.62646	0.531013	0.638473
17104	CELF2	801.4452914	-0.087279957	0.211993	-0.411712	0.68055	0.779893
16624	BTN3A1	1513.974217	-0.087284692	0.160911	-0.542441	0.587515	0.692726
16513	ERI1	312.851266	-0.087577074	0.154122	-0.568232	0.569877	0.676447

16196	RRN3P3	72.92085471	-0.087697737	0.133807	-0.655406	0.512207	0.619964
13893	SART3	1160.638428	-0.087709015	0.064941	-1.350589	0.176827	0.249477
17869	CL14-CCL1	103.6720044	-0.087916516	0.376236	-0.233674	0.815238	0.894257
15890	DNASE1L1	771.8104057	-0.087978984	0.117962	-0.745823	0.455774	0.562217
15599	HEATR3	254.6571672	-0.087979155	0.105245	-0.835949	0.403184	0.506622
15145	LLPH	503.0893038	-0.088382283	0.091936	-0.961351	0.336376	0.435345
15202	SIRT3	781.7885227	-0.088640617	0.093627	-0.946746	0.343768	0.443244
15439	ELAC2	1322.797669	-0.088792739	0.101339	-0.876192	0.380926	0.483615
17177	SSPN	1226.558324	-0.088829252	0.226285	-0.392555	0.694648	0.792676
16205	GPI	12859.89474	-0.088889295	0.136003	-0.653585	0.513379	0.620965
16733	C15orf62	32.69135734	-0.088918941	0.174267	-0.510246	0.609879	0.714411
16889	MEIS2	258.1093949	-0.088925549	0.190137	-0.467693	0.640004	0.742775
14480	CRKL	1948.549719	-0.088928695	0.075729	-1.174295	0.240277	0.325253
14980	AGPAT1	2152.682702	-0.08896072	0.087959	-1.01139	0.31183	0.408023
16253	SMS	2432.762674	-0.088992319	0.139122	-0.639673	0.522385	0.629993
16376	PRNP	2982.471128	-0.089208475	0.147004	-0.606842	0.543956	0.65108
15947	CCDC120	181.488338	-0.08952334	0.123216	-0.726555	0.467499	0.574494
14591	TOR1B	601.7869278	-0.089542869	0.078632	-1.138755	0.254806	0.342296
16740	RBL1	124.7352258	-0.089641478	0.176027	-0.50925	0.610577	0.714911
17051	BCL6B	834.5559818	-0.089668034	0.20989	-0.427214	0.669224	0.769307
17526	CDH2	2065.778865	-0.089673835	0.290106	-0.309107	0.75724	0.846894
18263	FRG2C	1.776556423	-0.089679806	0.598584	-0.14982	0.880907	0.945545
15448	POLR3GL	576.6173973	-0.089792282	0.102672	-0.874554	0.381816	0.484446
17328	UTS2D	4.12685562	-0.089855171	0.249291	-0.360442	0.718516	0.812768
16442	MIDN	2260.193391	-0.089861305	0.152373	-0.589745	0.555361	0.662063
16853	SLC25A39	2761.674913	-0.090065019	0.188549	-0.477675	0.632882	0.736078
14781	ZC3H10	181.8246361	-0.090122143	0.083896	-1.074215	0.282726	0.374922
15342	LYPLA2P1	55.67653595	-0.090137855	0.099334	-0.907426	0.364182	0.46528
14056	EI24	2147.254558	-0.090142042	0.069335	-1.300095	0.193568	0.26993
15379	HEATR1	914.8134182	-0.090297039	0.100928	-0.894664	0.370967	0.472808
15534	AAMP	2110.217224	-0.090402037	0.106273	-0.850658	0.394959	0.498365
14964	ZNF786	116.2503843	-0.090476878	0.088884	-1.017922	0.308715	0.404379
16922	CLIC2	453.980885	-0.090567708	0.197196	-0.459277	0.646035	0.748268
16781	CLCN7	2239.783674	-0.090573898	0.182617	-0.495978	0.61991	0.724041
16351	DPYSL2	4222.705643	-0.090620684	0.147657	-0.613726	0.539396	0.646609
15568	FCHSD2	951.3179134	-0.090655204	0.107576	-0.842708	0.399392	0.502857
14464	SDCCAG8	462.7245526	-0.090704136	0.076955	-1.17866	0.238534	0.323251
18289	FAM55D	1.761616507	-0.090739872	0.621968	-0.145892	0.884007	0.947423
16780	CDK10	1259.149722	-0.090974797	0.183372	-0.496122	0.619808	0.724009
16135	CC2D1A	640.6031435	-0.090975021	0.13551	-0.671351	0.501997	0.609832
15598	PEX10	544.307708	-0.09100445	0.108837	-0.836153	0.403069	0.506511
17778	TMEM45B	34.43251236	-0.09119194	0.36463	-0.250094	0.802515	0.884806
17355	CC2D2B	9.229996914	-0.091215821	0.259576	-0.351403	0.725286	0.819102
16465	SLC25A43	678.5991177	-0.091386701	0.156858	-0.582609	0.560156	0.666846
17127	EXOC3L	279.8514623	-0.091540556	0.226455	-0.404233	0.686041	0.78514
16190	DBI	2398.963444	-0.091682527	0.139756	-0.65602	0.511811	0.619642
15292	RPP30	399.2728573	-0.091797641	0.099472	-0.92285	0.356086	0.456424

15309	NUP188	894.6005087	-0.091800665	0.099894	-0.918985	0.358104	0.458471
15472	PAAF1	525.6172558	-0.091825817	0.105668	-0.869004	0.384845	0.487548
17011	SPDYE7P	8.292225032	-0.091837886	0.210052	-0.437215	0.661955	0.762703
15044	NOP58	896.5089762	-0.091891911	0.092478	-0.993662	0.320388	0.417457
15411	KLC1	1022.614461	-0.091904887	0.103705	-0.886213	0.375503	0.477596
16067	EIF3K	3335.563688	-0.091957669	0.132644	-0.693268	0.488141	0.59551
15381	DIS3L2	420.6029397	-0.092250669	0.103167	-0.89419	0.37122	0.473039
16394	HDAC4	341.0650026	-0.092360381	0.153325	-0.602385	0.546918	0.653906
14540	PELO	933.1564594	-0.09243833	0.080145	-1.153384	0.248753	0.335337
15946	C19orf47	215.9813741	-0.092456353	0.127151	-0.727136	0.467143	0.574217
16923	PECAM1	4026.114348	-0.092639828	0.201705	-0.459283	0.646031	0.748268
13941	RNF114	1897.654292	-0.092764	0.069343	-1.337757	0.180976	0.254451
16640	OCIAD2	2332.142452	-0.09276869	0.172456	-0.537927	0.590628	0.695727
16762	WSB1	2876.635528	-0.092816006	0.185528	-0.50028	0.616878	0.721359
17618	ACY3	538.5044738	-0.092923762	0.320851	-0.289616	0.77211	0.858972
16809	CHST10	265.5285054	-0.0929591	0.190275	-0.488552	0.625159	0.728999
17360	ZNF486	118.1335958	-0.093212948	0.266102	-0.350291	0.72612	0.819855
15990	RNF217	87.26099595	-0.093259866	0.130438	-0.714975	0.474624	0.581808
16717	PLEKHM1I	150.0931225	-0.09344519	0.181326	-0.515345	0.606312	0.71087
15683	CREB3L2	2974.921466	-0.093503796	0.11514	-0.812091	0.416739	0.520851
15675	MAPKAPK	3925.272095	-0.093519152	0.114861	-0.814197	0.415532	0.519607
15776	C7orf70	449.4058158	-0.093560598	0.119487	-0.783021	0.433615	0.538748
16848	HSBP1L1	175.5197208	-0.093567959	0.195444	-0.478745	0.63212	0.73541
16265	LOC14578	47.94785607	-0.0937554	0.147368	-0.6362	0.524646	0.632239
17944	MGC16025	0.722161209	-0.093811209	0.427985	-0.219193	0.8265	0.902771
15315	TTC27	442.4894517	-0.09384998	0.102271	-0.917664	0.358795	0.459206
17303	ASS1	2828.564894	-0.093897482	0.256297	-0.366362	0.714095	0.808933
15800	TMEM60	404.4203116	-0.093925683	0.12106	-0.77586	0.437832	0.543161
15883	MTA1	622.780418	-0.09394466	0.125598	-0.74798	0.454472	0.560858
14979	POP4	674.3338052	-0.094007178	0.09292	-1.011705	0.311679	0.407852
16157	NSUN6	174.4755709	-0.094068043	0.141616	-0.664246	0.506533	0.61448
16985	GADD45B	2309.226665	-0.094108328	0.212499	-0.442866	0.657863	0.759186
18097	POF1B	117.705311	-0.094133315	0.511022	-0.184206	0.853852	0.924813
15041	GRWD1	659.857879	-0.094322835	0.094874	-0.994189	0.320131	0.417185
14280	EXOSC10	1002.947651	-0.094353853	0.076423	-1.234629	0.216969	0.297804
16400	HIP1	1262.910467	-0.094389955	0.157153	-0.600625	0.54809	0.65499
15933	LRRC14	564.8267632	-0.094408911	0.128969	-0.732027	0.464152	0.571007
17399	LOC14366	117.8131296	-0.09443605	0.276258	-0.34184	0.732471	0.825172
16094	IFT140	648.6105853	-0.094652074	0.138577	-0.68303	0.494588	0.602362
17622	CLSTN2	45.7549746	-0.09470171	0.328648	-0.288156	0.773228	0.86006
16587	GUSBL1	24.06838461	-0.094724224	0.17192	-0.550978	0.581649	0.687332
15260	MRPS23	781.8819906	-0.094778551	0.101847	-0.930596	0.352063	0.452208
14655	ZMYM3	801.7068409	-0.094782908	0.084974	-1.115437	0.264663	0.353986
17763	HOXB9	206.795065	-0.094983322	0.37426	-0.253789	0.799658	0.882402
15719	C14orf143	51.54552546	-0.095181834	0.119277	-0.797987	0.424878	0.529749
17363	PCDHGA1	24.83243087	-0.095194317	0.272168	-0.349763	0.726517	0.820161
17122	ADPRHL1	13.23105122	-0.095270289	0.23506	-0.405302	0.685255	0.78447

16598	LYSMD4	222.5939072	-0.095292487	0.173794	-0.548306	0.583482	0.688909
17946	SLC35F3	61.41132414	-0.095606627	0.4371	-0.218729	0.826861	0.903115
14216	ZNHIT3	547.7694159	-0.095664852	0.076414	-1.251921	0.210599	0.290373
17691	TNS4	13.41192458	-0.0957094	0.355668	-0.269098	0.787855	0.872915
15632	NUP37	291.9627095	-0.095947767	0.116097	-0.826443	0.408553	0.512285
14360	MORF4L2	4132.092344	-0.096165527	0.079436	-1.210602	0.226048	0.308549
15417	GEMIN8	465.6652187	-0.096296456	0.108893	-0.88432	0.376524	0.478708
17783	CHRNA3	1.893548492	-0.096303349	0.387557	-0.248488	0.803757	0.885849
15173	KTELC1	483.3998992	-0.096374569	0.100854	-0.955581	0.339284	0.438299
17283	LRRN3	18.75041206	-0.096689407	0.260042	-0.371823	0.710025	0.805253
16595	OSBPL11	790.7791654	-0.096799978	0.176395	-0.54877	0.583164	0.688797
15835	POLR2E	2565.481276	-0.096915261	0.126814	-0.764229	0.444731	0.5505
16024	BAZ1A	848.1091555	-0.097001174	0.137894	-0.703449	0.481776	0.589313
17834	LOC44089[illegible]	1.207656981	-0.097146407	0.407885	-0.238171	0.811749	0.892124
13296	SUPT4H1	1352.916568	-0.097149686	0.062912	-1.544224	0.122534	0.18064
15177	INTS8	627.0336856	-0.09756933	0.102248	-0.954244	0.33996	0.439056
15553	APAF1	557.9603653	-0.097613319	0.115413	-0.845774	0.397679	0.501183
15241	PCNT	506.7462323	-0.097619743	0.104354	-0.93547	0.349546	0.449541
16164	CXXC1	774.3796736	-0.09770607	0.147291	-0.663353	0.507105	0.614932
15588	MRPL22	581.4027789	-0.097745602	0.116577	-0.838464	0.40177	0.505202
15327	C10orf2	233.7327964	-0.097779732	0.107239	-0.91179	0.361879	0.462791
15885	SPG7	1567.003108	-0.097798698	0.130824	-0.747559	0.454726	0.561121
15945	RAB20	723.720155	-0.097834014	0.134515	-0.72731	0.467036	0.574122
15541	FAM86C	130.743537	-0.097880962	0.11536	-0.848485	0.396168	0.499633
14024	PHF12	821.9793652	-0.098065107	0.074739	-1.312098	0.189487	0.264841
14385	DUSP22	760.8154242	-0.09819215	0.081682	-1.202131	0.229313	0.312461
15531	FIGNL1	201.4648172	-0.098499612	0.115745	-0.851004	0.394767	0.498218
16958	LENG8	3134.145301	-0.098538912	0.219154	-0.449634	0.652975	0.754655
15412	XRRA1	443.7198954	-0.098646822	0.111393	-0.885576	0.375846	0.478001
15778	PARP1	2506.880105	-0.098886171	0.126378	-0.782466	0.433941	0.539084
15304	CYTH2	1308.192526	-0.099078247	0.107709	-0.919867	0.357642	0.458059
16106	PPARD	1111.086583	-0.099291833	0.14607	-0.679755	0.49666	0.604435
15090	MRPL43	1105.074796	-0.099624896	0.101762	-0.978998	0.327581	0.425508
15928	VAC14	1231.510652	-0.099737266	0.135732	-0.73481	0.462455	0.569104
15745	POLG	1403.141697	-0.099777639	0.126103	-0.791242	0.428803	0.533854
14266	VPS33B	275.0491178	-0.099778201	0.080538	-1.238903	0.215381	0.295927
16161	ZNF436	567.5189665	-0.100007689	0.150637	-0.6639	0.506754	0.614621
15019	MEF2D	1790.695406	-0.100093796	0.099858	-1.002363	0.316168	0.412625
15011	PARVA	1874.47901	-0.100146878	0.099623	-1.005255	0.314774	0.410997
15854	CDC42EP4	1130.760472	-0.100247099	0.132518	-0.756482	0.44936	0.555565
16791	MOCS1	1029.04045	-0.100485962	0.204463	-0.491462	0.6231	0.727339
16233	ZNF589	198.0587404	-0.100713059	0.156068	-0.645314	0.518724	0.626348
15089	DRG1	1134.29127	-0.100729464	0.102866	-0.979228	0.327468	0.425389
15001	PRUNE	850.1218753	-0.100736389	0.100033	-1.007029	0.313921	0.410184
15900	ME2	913.8048062	-0.100773721	0.135832	-0.741899	0.458149	0.564719
17715	TUBB2B	89.37930556	-0.100891485	0.382362	-0.263864	0.791885	0.876269
16725	KRT222	55.77941622	-0.100968232	0.196544	-0.513719	0.607448	0.711904

15454	LOC72875[illegible]	40.21710879	-0.101197945	0.115817	-0.873777	0.38224	0.484781
16932	HSD17B14	811.7477907	-0.101513924	0.223117	-0.45498	0.649124	0.75142
14861	NDST2	501.8826958	-0.101655966	0.096842	-1.049708	0.293852	0.387578
15596	TSC2	1400.435821	-0.101930692	0.121849	-0.83653	0.402857	0.506323
15660	SKI	1884.585867	-0.101991298	0.12452	-0.819073	0.412745	0.516616
15687	MITD1	249.5034629	-0.102007289	0.125806	-0.810832	0.417462	0.521621
16229	NOTCH1	1450.358532	-0.102076695	0.15786	-0.646628	0.517873	0.625474
15718	PLEKHA8	101.732504	-0.102539205	0.128358	-0.798855	0.424374	0.529212
16041	ZDHHC1	238.5939287	-0.10255475	0.146762	-0.698783	0.484688	0.592255
17535	OIT3	48.61227622	-0.10256672	0.334042	-0.307048	0.758807	0.848211
15808	TTC1	1500.936163	-0.102675874	0.132691	-0.773798	0.43905	0.544397
14397	PIGC	632.6166106	-0.102743349	0.085753	-1.198137	0.230864	0.314313
16143	MCM8	152.0901979	-0.102866057	0.154066	-0.667674	0.504342	0.612377
17312	ADAM20	1.829621621	-0.102942392	0.282777	-0.364041	0.715828	0.810428
15466	VRK2	401.4276629	-0.102979535	0.118237	-0.87096	0.383776	0.486382
15086	EIF3M	2625.279423	-0.103014	0.105087	-0.98027	0.326953	0.424805
16836	CD200	745.1408638	-0.103059738	0.214381	-0.480732	0.630707	0.734245
15587	ARMC6	556.0205337	-0.103106272	0.122934	-0.838716	0.401629	0.505057
15584	FAM131A	300.4189472	-0.103208375	0.122957	-0.839384	0.401254	0.504683
15431	FIZ1	362.594145	-0.10325113	0.117348	-0.879874	0.378928	0.481327
18182	FAM71A	0.432540577	-0.103585814	0.620296	-0.166994	0.867375	0.935068
14825	BFAR	1808.861899	-0.103786794	0.098141	-1.057532	0.290269	0.383754
15848	EEF1A1	107240.8691	-0.103822223	0.136704	-0.759467	0.447573	0.553564
16217	SLC36A1	604.305664	-0.103869363	0.159787	-0.650048	0.515661	0.623264
16778	TTC23L	10.25834763	-0.104061838	0.209485	-0.49675	0.619365	0.723577
16760	AQP11	47.41151905	-0.104077147	0.207881	-0.500656	0.616613	0.721136
14689	SLC35A4	2569.102246	-0.104083947	0.094249	-1.104345	0.269443	0.359545
16966	HIST1H2B[illegible]	765.6602133	-0.104115313	0.232448	-0.447909	0.654219	0.755826
16514	ZNF281	349.4733213	-0.104310434	0.183599	-0.568143	0.569938	0.676478
15811	MLEC	12323.85711	-0.104330242	0.13494	-0.773163	0.439426	0.544759
15443	ACTN4	11573.09321	-0.10463407	0.119587	-0.874962	0.381594	0.484288
17001	LOC40004[illegible]	188.4940237	-0.104675565	0.23838	-0.439111	0.660581	0.761605
16195	RNF144B	1109.770635	-0.105394084	0.160821	-0.655349	0.512243	0.619964
15372	ZBTB25	116.6941809	-0.105498434	0.117394	-0.898669	0.368829	0.470297
14567	INTS10	908.9553927	-0.10554431	0.092103	-1.145932	0.251823	0.338824
13718	UTP6	676.9770357	-0.105651227	0.074814	-1.412186	0.157895	0.225609
17257	IRX3	1090.65055	-0.105890056	0.281929	-0.375591	0.707221	0.803252
16078	COX17	636.487872	-0.105914286	0.153468	-0.690138	0.490108	0.5975
16286	SLC38A6	269.2166883	-0.105937493	0.16769	-0.631745	0.527554	0.634937
16168	LRP12	414.1865344	-0.105973693	0.159911	-0.662705	0.50752	0.615283
14822	CCT6A	3694.505172	-0.106027012	0.100213	-1.058018	0.290047	0.38354
15644	CCDC46	270.6891147	-0.106078092	0.128772	-0.823764	0.410074	0.513753
14844	ZMAT2	2045.860848	-0.106392411	0.100996	-1.053429	0.292144	0.385767
16042	CCDC159	229.438923	-0.10643426	0.15234	-0.698661	0.484764	0.592274
15613	TUBG2	361.7055048	-0.10661157	0.128095	-0.832287	0.405247	0.508786
16037	H3F3A	3024.637967	-0.106909233	0.152686	-0.700188	0.48381	0.59133
17422	MAP1LC3[illegible]	12.90413382	-0.107242954	0.319258	-0.335913	0.736937	0.829106

14758	TOE1	203.1394696	-0.107272853	0.099148	-1.081951	0.279274	0.370932
18293	KRT73	0.511109186	-0.107343458	0.740346	-0.144991	0.884718	0.947978
17707	TUBA4B	44.27536421	-0.107370918	0.404373	-0.265524	0.790606	0.875174
15562	TMEM165	1474.351207	-0.107430332	0.127229	-0.844385	0.398455	0.50187
14121	NAT10	1139.703662	-0.107459158	0.083873	-1.281206	0.200121	0.277783
17126	C2orf50	5.506652902	-0.107637464	0.266264	-0.404251	0.686028	0.78514
15157	C7orf28A	943.6044379	-0.107739758	0.112428	-0.958297	0.337913	0.436989
17807	GLP1R	7.772988823	-0.107871575	0.440279	-0.245008	0.806451	0.887698
14642	ZNF490	222.2386321	-0.108021957	0.096479	-1.119642	0.262866	0.351895
16571	KIAA1383	100.6380588	-0.108354103	0.195548	-0.554104	0.579507	0.68547
17981	C3orf48	0.36298423	-0.108367851	0.520892	-0.208043	0.835195	0.910442
18137	ZNF560	0.305526459	-0.10836794	0.611231	-0.177295	0.859277	0.928637
18220	TMEM212	0.230457341	-0.108368057	0.685768	-0.158024	0.874438	0.940716
18305	PDE6C	0.230163359	-0.108368137	0.759213	-0.142737	0.886498	0.949305
18396	VHLL	0.202851033	-0.108368254	0.838891	-0.12918	0.897215	0.955986
18507	HCRTR1	0.167402907	-0.108368527	0.989287	-0.109542	0.912773	0.966677
18567	C14orf23	0.238672047	-0.108368562	1.086858	-0.099708	0.920576	0.971781
18556	OR52W1	0.143639024	-0.108368679	1.058558	-0.102374	0.91846	0.970766
18612	)C1001339	0.168549629	-0.108368784	1.132728	-0.095671	0.923782	0.97287
18613	FOXD3	0.161486914	-0.108368787	1.133901	-0.095572	0.923861	0.9729
18684	LOC15018	0.183595799	-0.108369114	1.316263	-0.082331	0.934384	0.979957
18803	CALML5	0.303227401	-0.108369284	1.569196	-0.06906	0.944942	0.985142
18762	SLC25A31	0.106076785	-0.108369659	1.450153	-0.07473	0.94043	0.982432
18797	GABRA1	0.166283036	-0.108369738	1.552953	-0.069783	0.944366	0.98476
18809	T	0.110240372	-0.108370021	1.578294	-0.068663	0.945258	0.985142
18810	TRHR	0.11018402	-0.108370021	1.578378	-0.068659	0.945261	0.985142
18808	SEC14L3	0.108450728	-0.108370029	1.581005	-0.068545	0.945352	0.985142
18943	SPDYE4	0.108588095	-0.108370515	1.73809	-0.06235	0.950284	0.986718
18831	BMP10	0.089657081	-0.108370698	1.766582	-0.061345	0.951085	0.986718
18837	CAV3	0.087270226	-0.108370716	1.772042	-0.061156	0.951235	0.986718
18900	OR13D1	0.086821255	-0.10837072	1.773089	-0.06112	0.951264	0.986718
18891	MCHR2	0.085970784	-0.108370727	1.77509	-0.061051	0.951319	0.986718
18835	C18orf20	0.146378867	-0.108370804	1.884517	-0.057506	0.954142	0.986718
18966	VGLL2	0.107937983	-0.108371168	1.929276	-0.056172	0.955205	0.987186
18971	ASAP1IT1	0.091050308	-0.108371382	1.957927	-0.05535	0.95586	0.98755
18973	GLRA1	0.088703043	-0.108371406	1.964243	-0.055172	0.956001	0.987593
18975	OR10A2	0.087247075	-0.108371421	1.968264	-0.055059	0.956091	0.987633
18976	OR2K2	0.085492666	-0.10837144	1.973218	-0.054921	0.956201	0.987637
18977	PRR25	0.084799334	-0.108371448	1.975209	-0.054866	0.956245	0.987637
18993	OR4K2	0.144143317	-0.108371572	2.10465	-0.051492	0.958934	0.989631
19104	OR1E2	0.1075315	-0.108372038	2.15783	-0.050223	0.959945	0.989949
19026	DCAF8L1	0.08598384	-0.108372408	2.213899	-0.048951	0.960958	0.989949
19017	CCDC79	0.0843565	-0.108372433	2.219624	-0.048825	0.961059	0.989949
19145	PLA2G2C	0.069365633	-0.108372791	2.268381	-0.047775	0.961895	0.989949
19166	RPL3L	0.069196389	-0.108372795	2.269152	-0.047759	0.961908	0.989949
19057	KCNQ1DN	0.069193254	-0.108372795	2.269166	-0.047759	0.961908	0.989949
18996	ACCN5	0.0685796	-0.108372807	2.27198	-0.0477	0.961956	0.989949

19049	HEATR7B2	0.068163422	-0.108372816	2.273906	-0.047659	0.961988	0.989949
19205	TSGA13	0.06784367	-0.108372823	2.275396	-0.047628	0.962013	0.989949
19047	GSTA5	0.067825499	-0.108372823	2.275481	-0.047626	0.962014	0.989949
19102	OR11H12	0.06766628	-0.108372827	2.276226	-0.047611	0.962026	0.989949
19085	MRGPRX4	0.067639278	-0.108372827	2.276353	-0.047608	0.962029	0.989949
19110	OR2L2	0.067122172	-0.108372838	2.27879	-0.047557	0.962069	0.989949
19021	CRISP2	0.127083009	-0.108372885	2.396301	-0.045225	0.963928	0.989949
19038	FEZF1	0.067751222	-0.108374356	2.593717	-0.041783	0.966671	0.989949
19177	SNORA22	0.067437643	-0.108374366	2.59558	-0.041753	0.966695	0.989949
19036	FAM75A5	0.066958748	-0.108374381	2.598445	-0.041707	0.966732	0.989949
19189	SPINK4	0.091963257	-0.108375584	2.864837	-0.03783	0.969824	0.989949
19210	VSTM2B	0.088853464	-0.108375658	2.878299	-0.037653	0.969965	0.989949
19037	FAM99A	0.064856078	-0.108376102	2.91407	-0.037191	0.970333	0.989949
19061	KRTAP10-1	0.065998791	-0.108376102	2.91407	-0.037191	0.970333	0.989949
19071	LOC402644	0.072067057	-0.108376102	2.91407	-0.037191	0.970333	0.989949
19078	LOC84931	0.067954952	-0.108376102	2.91407	-0.037191	0.970333	0.989949
19094	OPRM1	0.065890352	-0.108376102	2.91407	-0.037191	0.970333	0.989949
19198	TGIF2LX	0.068260105	-0.108376102	2.91407	-0.037191	0.970333	0.989949
19065	KRTAP3-2	0.049694023	-0.108376347	2.91407	-0.037191	0.970333	0.989949
19008	C10orf96	0.047735988	-0.108376347	2.91407	-0.037191	0.970333	0.989949
19022	CSN3	0.048993065	-0.108376347	2.91407	-0.037191	0.970333	0.989949
19121	OR51V1	0.045551728	-0.108376347	2.91407	-0.037191	0.970333	0.989949
19169	SCARNA21	0.047168718	-0.108376347	2.91407	-0.037191	0.970333	0.989949
18995	ABCC12	0.047577074	-0.108376347	2.91407	-0.037191	0.970333	0.989949
19007	C10orf113	0.049077725	-0.108376347	2.91407	-0.037191	0.970333	0.989949
19010	C1orf141	0.046941009	-0.108376347	2.91407	-0.037191	0.970333	0.989949
19030	DRGX	0.050501758	-0.108376347	2.91407	-0.037191	0.970333	0.989949
19040	FSCB	0.04422701	-0.108376347	2.91407	-0.037191	0.970333	0.989949
19045	GNG8	0.048911759	-0.108376347	2.91407	-0.037191	0.970333	0.989949
19046	GPX6	0.045146212	-0.108376347	2.91407	-0.037191	0.970333	0.989949
19054	IL4	0.047304972	-0.108376347	2.91407	-0.037191	0.970333	0.989949
19067	LCE1B	0.048921913	-0.108376347	2.91407	-0.037191	0.970333	0.989949
19079	LRIT1	0.045962222	-0.108376347	2.91407	-0.037191	0.970333	0.989949
19081	MAGEB3	0.048269651	-0.108376347	2.91407	-0.037191	0.970333	0.989949
19083	MGC34034	0.050734979	-0.108376347	2.91407	-0.037191	0.970333	0.989949
19087	NKX6-2	0.049769233	-0.108376347	2.91407	-0.037191	0.970333	0.989949
19088	NOBOX	0.049589369	-0.108376347	2.91407	-0.037191	0.970333	0.989949
19098	OR10H2	0.048386132	-0.108376347	2.91407	-0.037191	0.970333	0.989949
19107	OR2D3	0.047695614	-0.108376347	2.91407	-0.037191	0.970333	0.989949
19118	OR4Q3	0.047137796	-0.108376347	2.91407	-0.037191	0.970333	0.989949
19122	OR52B4	0.048585322	-0.108376347	2.91407	-0.037191	0.970333	0.989949
19126	OR5V1	0.047197188	-0.108376347	2.91407	-0.037191	0.970333	0.989949
19130	OR6M1	0.047902075	-0.108376347	2.91407	-0.037191	0.970333	0.989949
19143	PDHA2	0.047073107	-0.108376347	2.91407	-0.037191	0.970333	0.989949
19149	PRAMEF1	0.04711278	-0.108376347	2.91407	-0.037191	0.970333	0.989949
19153	PRLHR	0.048529568	-0.108376347	2.91407	-0.037191	0.970333	0.989949
19154	PRR23B	0.044657607	-0.108376347	2.91407	-0.037191	0.970333	0.989949

19158	RAX2	0.048622193	-0.108376347	2.91407	-0.037191	0.970333	0.989949
19163	RNASE9	0.047402462	-0.108376347	2.91407	-0.037191	0.970333	0.989949
19171	SI	0.047302436	-0.108376347	2.91407	-0.037191	0.970333	0.989949
19183	SNORA71A	0.048887296	-0.108376347	2.91407	-0.037191	0.970333	0.989949
19190	SPP2	0.048357358	-0.108376347	2.91407	-0.037191	0.970333	0.989949
19196	TAS2R40	0.048138078	-0.108376347	2.91407	-0.037191	0.970333	0.989949
19202	TRIM49	0.04600387	-0.108376347	2.91407	-0.037191	0.970333	0.989949
19207	TTTY9B	0.048180684	-0.108376347	2.91407	-0.037191	0.970333	0.989949
19209	VPREB1	0.047553987	-0.108376347	2.91407	-0.037191	0.970333	0.989949
18998	ANXA8L1	0.029035212	-0.108376592	2.91407	-0.037191	0.970333	0.989949
19062	KRTAP2-2	0.029344984	-0.108376592	2.91407	-0.037191	0.970333	0.989949
19080	LYZL6	0.028847285	-0.108376592	2.91407	-0.037191	0.970333	0.989949
19091	NPVF	0.027975843	-0.108376592	2.91407	-0.037191	0.970333	0.989949
19109	OR2F2	0.027361135	-0.108376592	2.91407	-0.037191	0.970333	0.989949
19128	OR6C2	0.027332622	-0.108376592	2.91407	-0.037191	0.970333	0.989949
19129	OR6K6	0.026827102	-0.108376592	2.91407	-0.037191	0.970333	0.989949
19138	OTOL1	0.028001879	-0.108376592	2.91407	-0.037191	0.970333	0.989949
19168	SCARNA2(	0.03136196	-0.108376592	2.91407	-0.037191	0.970333	0.989949
19201	TNP1	0.029254779	-0.108376592	2.91407	-0.037191	0.970333	0.989949
19002	BMP15	0.028614563	-0.108376592	2.91407	-0.037191	0.970333	0.989949
19006	BSX	0.028089691	-0.108376592	2.91407	-0.037191	0.970333	0.989949
19009	C16orf78	0.02932377	-0.108376592	2.91407	-0.037191	0.970333	0.989949
19011	C1orf146	0.028904055	-0.108376592	2.91407	-0.037191	0.970333	0.989949
19019	CLCA1	0.042962981	-0.108376592	2.91407	-0.037191	0.970333	0.989949
19020	CRCT1	0.02865698	-0.108376592	2.91407	-0.037191	0.970333	0.989949
19023	CSTT	0.027384438	-0.108376592	2.91407	-0.037191	0.970333	0.989949
19031	DUSP21	0.02816384	-0.108376592	2.91407	-0.037191	0.970333	0.989949
19041	FSHB	0.027035676	-0.108376592	2.91407	-0.037191	0.970333	0.989949
19042	GFRAL	0.028173598	-0.108376592	2.91407	-0.037191	0.970333	0.989949
19043	GK2	0.027814613	-0.108376592	2.91407	-0.037191	0.970333	0.989949
19048	GSTTP2	0.02824956	-0.108376592	2.91407	-0.037191	0.970333	0.989949
19055	KCNC2	0.026793385	-0.108376592	2.91407	-0.037191	0.970333	0.989949
19058	KIF2B	0.027532106	-0.108376592	2.91407	-0.037191	0.970333	0.989949
19063	KRTAP24-1	0.026483807	-0.108376592	2.91407	-0.037191	0.970333	0.989949
19066	KRTAP4-11	0.029365119	-0.108376592	2.91407	-0.037191	0.970333	0.989949
19069	LOC283914	0.02579221	-0.108376592	2.91407	-0.037191	0.970333	0.989949
19070	LOC286094	0.028691363	-0.108376592	2.91407	-0.037191	0.970333	0.989949
19075	LOC643955	0.02579221	-0.108376592	2.91407	-0.037191	0.970333	0.989949
19082	MBD3L5	0.027339914	-0.108376592	2.91407	-0.037191	0.970333	0.989949
19089	NOTO	0.029288288	-0.108376592	2.91407	-0.037191	0.970333	0.989949
19090	NPBWR2	0.028014001	-0.108376592	2.91407	-0.037191	0.970333	0.989949
19097	OR10H1	0.027234363	-0.108376592	2.91407	-0.037191	0.970333	0.989949
19101	OR10X1	0.02579221	-0.108376592	2.91407	-0.037191	0.970333	0.989949
19106	OR2AG1	0.027442045	-0.108376592	2.91407	-0.037191	0.970333	0.989949
19108	OR2F1	0.027600819	-0.108376592	2.91407	-0.037191	0.970333	0.989949
19112	OR3A4	0.028793712	-0.108376592	2.91407	-0.037191	0.970333	0.989949
19113	OR4C15	0.026582753	-0.108376592	2.91407	-0.037191	0.970333	0.989949

19114	OR4D11	0.027373296	-0.108376592	2.91407	-0.037191	0.970333	0.989949
19115	OR4M1	0.02579221	-0.108376592	2.91407	-0.037191	0.970333	0.989949
19116	OR4N3P	0.027283075	-0.108376592	2.91407	-0.037191	0.970333	0.989949
19117	OR4P4	0.026333723	-0.108376592	2.91407	-0.037191	0.970333	0.989949
19133	OR8A1	0.028282224	-0.108376592	2.91407	-0.037191	0.970333	0.989949
19135	OR8K3	0.026582753	-0.108376592	2.91407	-0.037191	0.970333	0.989949
19137	OR9K2	0.02579221	-0.108376592	2.91407	-0.037191	0.970333	0.989949
19139	PAGE3	0.029011362	-0.108376592	2.91407	-0.037191	0.970333	0.989949
19140	PASD1	0.027826219	-0.108376592	2.91407	-0.037191	0.970333	0.989949
19148	PRAMEF14	0.02789109	-0.108376592	2.91407	-0.037191	0.970333	0.989949
19152	PRLH	0.031346963	-0.108376592	2.91407	-0.037191	0.970333	0.989949
19167	SAA3P	0.027895107	-0.108376592	2.91407	-0.037191	0.970333	0.989949
19174	SMCP	0.028183724	-0.108376592	2.91407	-0.037191	0.970333	0.989949
19178	SNORA25	0.03092437	-0.108376592	2.91407	-0.037191	0.970333	0.989949
19184	SNORD89	0.028477486	-0.108376592	2.91407	-0.037191	0.970333	0.989949
19186	SPATA19	0.029472425	-0.108376592	2.91407	-0.037191	0.970333	0.989949
19197	TAS2R41	0.031947057	-0.108376592	2.91407	-0.037191	0.970333	0.989949
19203	TRIM53	0.029018895	-0.108376592	2.91407	-0.037191	0.970333	0.989949
19204	TRYX3	0.027916338	-0.108376592	2.91407	-0.037191	0.970333	0.989949
19208	VENTXP1	0.027655804	-0.108376592	2.91407	-0.037191	0.970333	0.989949
19212	ZNF479	0.026333723	-0.108376592	2.91407	-0.037191	0.970333	0.989949
19213	ZSWIM2	0.027724553	-0.108376592	2.91407	-0.037191	0.970333	0.989949
15416	GUSBP1	110.3429587	-0.10840331	0.122571	-0.884413	0.376473	0.478675
17012	WDR88	3.535694576	-0.108458047	0.24807	-0.437207	0.661961	0.762703
16929	RNF208	108.1872663	-0.108584689	0.238352	-0.455565	0.648703	0.751091
14358	ILF2	2661.250107	-0.108690031	0.089759	-1.210916	0.225928	0.308428
17637	DNAH9	27.42614699	-0.108727137	0.384848	-0.282519	0.777545	0.864174
14391	TMEM218	226.0018006	-0.108823013	0.090646	-1.200531	0.229933	0.313176
18035	OR2B6	0.433849386	-0.108912875	0.546902	-0.199145	0.842149	0.915274
16496	LOC729375	22.24714021	-0.108979324	0.189842	-0.574053	0.565932	0.672456
15970	PUS7	398.6921575	-0.109198158	0.151118	-0.722604	0.469923	0.576767
14952	FBXO45	352.4290575	-0.109213951	0.106832	-1.022296	0.306641	0.401984
17178	DIRAS3	38.76828506	-0.109791367	0.280071	-0.392013	0.695049	0.793087
13376	RAD51C	192.6656128	-0.109818463	0.072302	-1.518893	0.128789	0.188736
17044	TAS2R4	2.947753058	-0.109925329	0.256582	-0.428422	0.668344	0.768611
16505	DOCK10	761.9134781	-0.109963683	0.192463	-0.571351	0.567762	0.674263
15952	PICK1	412.0827646	-0.110120131	0.151638	-0.726206	0.467713	0.574702
14511	TTC23	371.9323129	-0.110127897	0.094673	-1.16324	0.244732	0.330576
16719	MSH5	261.0428648	-0.110281873	0.214059	-0.515193	0.606418	0.710951
15962	TOM1	1523.980765	-0.11036607	0.152344	-0.724452	0.468788	0.575662
15334	SH3BP5L	744.5961128	-0.110975148	0.121926	-0.910184	0.362726	0.463684
15151	MEPCE	1398.649861	-0.111164972	0.115929	-0.958902	0.337608	0.436767
15807	NDUFB8	1755.284376	-0.111173452	0.143657	-0.773882	0.439	0.544385
17114	GRID2IP	3.761011613	-0.111344692	0.272828	-0.408113	0.683191	0.782472
16092	NDUFA7	718.4709862	-0.111413378	0.163025	-0.683415	0.494345	0.602141
17294	C3orf32	4.959314161	-0.111442553	0.302621	-0.368258	0.712681	0.807752
14722	UGGT1	2076.979173	-0.11156629	0.102263	-1.090978	0.275283	0.366514

17947	HHIP	24.55322207	-0.111604229	0.510462	-0.218634	0.826935	0.903146
15005	KDM1A	1058.496426	-0.111635246	0.110928	-1.00638	0.314233	0.410477
15993	DOCK6	1652.764241	-0.111761694	0.156401	-0.714584	0.474866	0.581959
15654	TAP2	1718.354227	-0.11193889	0.136318	-0.82116	0.411555	0.515325
17423	SIGLEC6	3.400754325	-0.112071838	0.333767	-0.335779	0.737038	0.829173
16813	OLFML2A	2635.597906	-0.112147838	0.230009	-0.487581	0.625847	0.729711
17761	GALNT13	10.85570602	-0.1122404	0.441458	-0.254249	0.799303	0.882109
13062	FBXO18	1343.134807	-0.112278453	0.069099	-1.624894	0.104185	0.156332
16075	LOC28490(	205.356556	-0.112590315	0.162991	-0.690777	0.489706	0.597121
15025	DNAL4	467.176957	-0.112615144	0.112772	-0.998609	0.317984	0.414829
13015	CNOT2	1343.595609	-0.112778839	0.068799	-1.639258	0.10116	0.152349
14377	TAB1	667.404386	-0.112904259	0.093795	-1.20373	0.228694	0.311792
14514	CNIH4	1204.169941	-0.112969658	0.097275	-1.161342	0.245503	0.331484
14336	BAZ1B	2108.755543	-0.112985413	0.092648	-1.219508	0.222651	0.304422
14786	UBE2D2	2254.398237	-0.113025634	0.105422	-1.072124	0.283665	0.376039
15203	RNH1	2993.469614	-0.113221689	0.1196	-0.94667	0.343807	0.443265
14705	TOMM22	1411.334215	-0.113262479	0.103247	-1.097006	0.272639	0.363413
14215	MGC2752	633.0897785	-0.113307766	0.090411	-1.253255	0.210113	0.289724
16490	LOC15153	428.3096197	-0.113415022	0.197225	-0.575054	0.565255	0.671896
15128	GTPBP5	388.3687768	-0.113417781	0.117521	-0.965084	0.334503	0.433407
14462	ERAL1	921.4081599	-0.113926105	0.096619	-1.179128	0.238347	0.323043
14888	EIF2B2	752.3434879	-0.114000097	0.109622	-1.039941	0.298367	0.392802
15767	DPH3B	16.99736262	-0.114180171	0.145343	-0.78559	0.432108	0.537182
15872	SMC4	998.6443439	-0.114230217	0.152087	-0.751087	0.4526	0.558935
15066	LMLN	384.0633651	-0.114289522	0.115882	-0.986262	0.324005	0.421533
17227	GPIHBP1	222.5938089	-0.114319045	0.29913	-0.382172	0.702334	0.79912
15234	FRAT2	120.8201664	-0.114354441	0.121937	-0.937815	0.34834	0.448195
14390	EIF1	14394.84721	-0.114365049	0.095237	-1.200842	0.229813	0.313034
15630	TMEM187	183.6049156	-0.114378808	0.138354	-0.826711	0.408401	0.51216
16320	ZNF682	70.14201484	-0.114425151	0.183696	-0.622905	0.533347	0.640572
16873	THNSL2	925.204744	-0.114575074	0.242637	-0.472208	0.636778	0.739732
16726	HSPG2	13183.55897	-0.114674067	0.223345	-0.51344	0.607644	0.71209
17189	RNF165	65.16528731	-0.114702475	0.294312	-0.389731	0.696735	0.794503
17665	LYPD6	23.43083648	-0.114782666	0.415189	-0.276459	0.782196	0.867921
16441	TCEA3	2084.709273	-0.114836703	0.194677	-0.589884	0.555268	0.661992
16096	CDKN1A	4572.607329	-0.115010644	0.168519	-0.682479	0.494936	0.602711
16280	PIGQ	1107.948164	-0.115048545	0.181658	-0.633324	0.526522	0.63392
16706	DYNC1I1	87.49413481	-0.115135672	0.222573	-0.517293	0.604952	0.709784
16687	CCDC62	2.883466743	-0.115554942	0.221661	-0.521313	0.602149	0.7073
16185	SYP	42.51443254	-0.11577625	0.176088	-0.657492	0.510865	0.618687
17146	TXLNB	59.27014659	-0.115810287	0.28959	-0.399912	0.689222	0.787906
16102	PRIM1	107.674977	-0.115815321	0.170087	-0.680918	0.495923	0.603688
16868	ZNF853	102.7222444	-0.115870479	0.244489	-0.473929	0.63555	0.73854
17369	LOC15117	8.322350586	-0.115909172	0.332517	-0.348582	0.727403	0.820878
14769	SLC7A6	727.5849688	-0.116122067	0.107725	-1.077945	0.281058	0.373012
15288	CYP2R1	198.563997	-0.11620386	0.125789	-0.923798	0.355592	0.45591
16186	NLRC5	1053.343548	-0.116274619	0.177055	-0.656716	0.511364	0.619254

16427	PRKCZ	387.5749444	-0.116558508	0.195403	-0.596502	0.55084	0.657272
16599	SYK	1065.007343	-0.116566725	0.212552	-0.548415	0.583407	0.688909
14670	SNRPD1	579.7989032	-0.11698292	0.105332	-1.110611	0.266736	0.356393
16956	CYR61	5360.880889	-0.117174978	0.260539	-0.44974	0.652898	0.754655
16672	EPSTI1	412.9465706	-0.117308999	0.222587	-0.527026	0.598176	0.703266
17484	IRX2	19.73292521	-0.117496642	0.369426	-0.318052	0.750445	0.841275
17572	GPR27	3.45911291	-0.117594594	0.393692	-0.298697	0.765171	0.853527
17916	ENPP7	54.61040292	-0.11777951	0.52345	-0.225006	0.821975	0.899234
16170	ENTPD7	402.3056462	-0.117822996	0.17791	-0.662261	0.507804	0.615551
16115	C6orf167	201.2159433	-0.117879405	0.174306	-0.676278	0.498864	0.606779
14380	KDM4A	945.8461954	-0.11793098	0.098012	-1.203233	0.228886	0.311988
14816	ZCRB1	1038.949443	-0.117961302	0.111316	-1.059694	0.289284	0.382711
15787	STK36	630.0677892	-0.118155943	0.151522	-0.779796	0.435511	0.540726
14094	BET1L	2142.338361	-0.118254553	0.091698	-1.289614	0.197185	0.274232
15402	FOXK1	1201.009867	-0.118286421	0.13293	-0.889842	0.37355	0.47539
15708	C5orf56	112.4227708	-0.118322914	0.147348	-0.803015	0.421966	0.526544
15939	FOSL2	3213.639482	-0.118402858	0.162058	-0.730621	0.465011	0.571847
15389	GHDC	1411.141843	-0.118481708	0.132681	-0.892984	0.371866	0.473646
16747	CTF1	106.7020141	-0.118506899	0.234564	-0.505222	0.613403	0.717938
15496	C2orf16	32.0245225	-0.118979069	0.137917	-0.862685	0.388311	0.491177
16365	NPIP	681.4403714	-0.119152603	0.195566	-0.60927	0.542346	0.649589
17074	SLCO1C1	20.16584364	-0.119402515	0.284171	-0.420178	0.674355	0.774162
13776	FUS	4306.936553	-0.119506518	0.085691	-1.394628	0.163128	0.232105
16814	IL33	320.9577062	-0.119613026	0.245487	-0.487247	0.626083	0.729845
13474	ZNF673	268.8468462	-0.11962536	0.079965	-1.495965	0.134663	0.195898
16545	OC1002681	13.00999721	-0.11976466	0.214172	-0.559199	0.576026	0.68234
13574	INRNPA1L	2081.490838	-0.11978286	0.08188	-1.462904	0.143494	0.207206
15349	C20orf96	246.361711	-0.119984703	0.132521	-0.905399	0.365254	0.466437
16159	CCDC96	56.10389677	-0.120053346	0.180797	-0.664022	0.506676	0.614595
15246	MLL4	896.9555607	-0.120136516	0.128579	-0.934338	0.35013	0.450144
12706	NDRG3	1017.544215	-0.120204459	0.069422	-1.731507	0.083361	0.128598
13948	ODF2	631.4050689	-0.120205402	0.090087	-1.33433	0.182096	0.255897
16172	SNORA8	329.0051196	-0.120345171	0.181943	-0.661445	0.508327	0.616109
15140	FAM119B	425.346696	-0.12042457	0.125097	-0.962649	0.335724	0.434645
14592	C11orf68	818.8041534	-0.120599727	0.105926	-1.138531	0.254899	0.34239
14021	INTS7	297.2482468	-0.120623676	0.091868	-1.313007	0.189181	0.26447
16618	DNHD1	202.4631594	-0.120684369	0.221692	-0.544379	0.58618	0.691402
17945	PGA5	22.07873415	-0.120802993	0.550979	-0.219252	0.826454	0.902771
15924	CCDC88A	1124.919725	-0.120882345	0.164413	-0.735235	0.462196	0.568922
14995	RPS27L	1867.480102	-0.1213101	0.120334	-1.008113	0.3134	0.409667
16262	B3GNT9	665.6478648	-0.12135906	0.190493	-0.637079	0.524073	0.631679
14855	RNF25	384.7555881	-0.121472894	0.115547	-1.051287	0.293127	0.386778
15104	TRIM41	852.8812657	-0.12151815	0.124624	-0.975078	0.329521	0.427632
15078	C22orf46	311.8198706	-0.121572377	0.123594	-0.983647	0.325289	0.422867
13940	STAT3	4942.174548	-0.121901128	0.091059	-1.33871	0.180665	0.25405
16413	CDH26	10.16804	-0.122024686	0.203729	-0.598957	0.549202	0.655801
15517	DPH1	690.3169843	-0.122050223	0.142988	-0.853568	0.393344	0.49687

14192	HPS6	412.9844226	-0.12212255	0.096951	-1.259635	0.207801	0.287
16669	FAM124B	48.95171234	-0.122226587	0.23118	-0.528708	0.597008	0.702019
14534	C17orf81	691.8064602	-0.122354316	0.105889	-1.155497	0.247887	0.334308
16574	SIGIRR	500.3962903	-0.122368248	0.220971	-0.553775	0.579733	0.685613
12845	C20orf4	991.2050768	-0.122374006	0.072318	-1.692164	0.090615	0.138275
17113	CTNNA3	3.882015623	-0.122407452	0.299421	-0.408813	0.682677	0.781929
16609	SOX7	277.0681184	-0.122497712	0.223858	-0.547211	0.584234	0.68948
14536	ARFIP2	900.776932	-0.122554312	0.106137	-1.154685	0.248219	0.33471
15814	PRDX5	4072.616497	-0.122559853	0.158624	-0.772642	0.439735	0.545038
16254	C21orf67	38.11273638	-0.122744283	0.191959	-0.639429	0.522544	0.630106
14648	ZNF23	271.27584	-0.122804683	0.109845	-1.117982	0.263575	0.352716
14631	TMEM11	441.8361858	-0.122879492	0.109483	-1.122361	0.261709	0.350609
17321	C1QL3	67.31169568	-0.123166864	0.340223	-0.362018	0.717339	0.811763
16287	FUNDC2P2	5.034743362	-0.123713086	0.195874	-0.631594	0.527652	0.634978
16278	RHOJ	351.8292381	-0.123817176	0.195423	-0.633584	0.526352	0.633849
16872	ITGB3	690.3104417	-0.123859237	0.262126	-0.472518	0.636557	0.739518
14924	C3orf75	305.5529531	-0.123890092	0.120336	-1.029531	0.30323	0.398259
14330	MTA3	691.5794081	-0.123895979	0.101397	-1.221888	0.22175	0.303316
15240	TNFRSF10I	2374.29359	-0.124012057	0.132486	-0.936041	0.349252	0.449192
15435	YY2	21.62390775	-0.124201237	0.141541	-0.877492	0.380219	0.482843
16118	TSSK6	18.8092301	-0.124371052	0.184025	-0.675837	0.499144	0.607006
12551	MAX	1298.127467	-0.124433305	0.0695	-1.790416	0.073387	0.114609
15938	MDFIC	1647.838334	-0.124670605	0.170611	-0.730731	0.464944	0.571801
14467	EIF4G1	6637.121243	-0.124922405	0.106035	-1.178127	0.238746	0.323471
15300	RHEB	1447.624411	-0.125005618	0.135747	-0.920872	0.357117	0.457507
14433	RAB1B	4350.66905	-0.125039168	0.105226	-1.188294	0.234718	0.318763
16341	PRELID2	148.0096411	-0.125042105	0.203211	-0.615332	0.538336	0.645663
15572	C12orf62	804.5289059	-0.125063063	0.148488	-0.842243	0.399652	0.503023
16138	PIM1	1211.184132	-0.125266653	0.186967	-0.669994	0.502861	0.610769
13635	MYL12A	5609.985583	-0.125335311	0.086861	-1.442949	0.149035	0.214245
17372	DNAI1	8.030826693	-0.125571214	0.360709	-0.348123	0.727748	0.821125
15413	TRAM1	8653.627911	-0.125604256	0.14189	-0.88522	0.376038	0.478214
12981	SMARCD1	1442.089145	-0.12567145	0.076168	-1.649924	0.098958	0.149425
17358	GLDC	332.6358168	-0.125703711	0.358435	-0.350701	0.725813	0.819602
16013	SPINT2	5400.178188	-0.125739313	0.177623	-0.707899	0.479008	0.586338
17657	GSTM1	492.9175521	-0.125759365	0.452121	-0.278154	0.780894	0.866869
15665	ITGB3BP	213.8925807	-0.125840188	0.153829	-0.81805	0.413329	0.517182
15710	DERA	1514.436434	-0.125994923	0.157306	-0.800952	0.423159	0.527955
16191	C9orf98	61.60337203	-0.126090881	0.19222	-0.655971	0.511843	0.619643
14545	RFT1	424.7986339	-0.126226628	0.109555	-1.152175	0.249249	0.335891
14376	FOXK2	851.5436561	-0.126485427	0.105021	-1.204377	0.228444	0.311473
14908	ATL3	566.050491	-0.126574719	0.122413	-1.033997	0.301137	0.395919
14969	POFUT1	2292.182657	-0.126575869	0.124592	-1.015922	0.309666	0.40549
16352	RAB3IL1	461.7170118	-0.126617007	0.206381	-0.613511	0.539538	0.64674
13597	PYGO2	901.7634167	-0.126619366	0.086927	-1.456622	0.145221	0.209345
13555	TATDN2	1015.001389	-0.126943091	0.086419	-1.468933	0.141851	0.205121
14601	DDHD1	416.2829505	-0.127114072	0.112146	-1.133474	0.257015	0.345028

14835	NDUFV3	710.6619668	-0.127255185	0.120502	-1.056042	0.290949	0.384418
13108	ILK	3237.866164	-0.127463803	0.079263	-1.608119	0.107809	0.161212
14942	POMGNT1	2052.262692	-0.127552421	0.124542	-1.024169	0.305755	0.401092
13036	GNB1	8317.857349	-0.127768398	0.078245	-1.63293	0.102484	0.154095
17474	IRX1	9.339951749	-0.127939599	0.400121	-0.319752	0.749156	0.840319
13786	RNF40	2285.477126	-0.127985917	0.092005	-1.391078	0.164202	0.233463
14717	ATN1	3668.838326	-0.128155943	0.117294	-1.092605	0.274567	0.365686
16343	ZC3HAV1L	91.84458639	-0.128311078	0.208528	-0.615319	0.538344	0.645663
13773	UBQLN4	867.0266033	-0.128349536	0.091978	-1.395434	0.162885	0.231826
9891	HNRNPL	4185.218887	-0.12849423	0.047755	-2.690722	0.00713	0.014128
15103	MED16	1006.133505	-0.128849961	0.132136	-0.975134	0.329494	0.427624
17307	SPTBN5	79.04009407	-0.128881143	0.352643	-0.365472	0.714759	0.809499
16855	LOC11323(	134.9723484	-0.128890526	0.270174	-0.477064	0.633316	0.736408
16757	DACT1	250.1506512	-0.128927177	0.256684	-0.50228	0.61547	0.719928
14242	SNRPB2	1103.741054	-0.128983273	0.103703	-1.243779	0.213581	0.293948
13744	FXR1	2135.605893	-0.12920341	0.092029	-1.403936	0.160338	0.228666
17528	FAM55B	0.986308863	-0.129264906	0.419675	-0.308012	0.758073	0.847729
14573	DTNBP1	392.7587611	-0.129322953	0.112938	-1.145074	0.252178	0.339185
16605	RND1	80.0167298	-0.129393754	0.236238	-0.547726	0.58388	0.689228
14106	GNL1	1064.283334	-0.129558101	0.100605	-1.287788	0.19782	0.27488
14285	MSH6	1011.717679	-0.129700255	0.105095	-1.234125	0.217156	0.297969
17464	FAM66E	1.121234378	-0.129725012	0.401445	-0.323145	0.746586	0.837939
17033	IGFALS	6.459601117	-0.129940011	0.300132	-0.432942	0.665057	0.765325
16854	CRYAB	24125.76555	-0.130058666	0.272396	-0.477461	0.633034	0.736211
17147	GLYATL2	24.65714826	-0.130232575	0.326201	-0.399241	0.689716	0.788425
14063	VPS4A	2326.563078	-0.130424083	0.100448	-1.298427	0.194141	0.270593
15123	ZWILCH	199.956804	-0.130618172	0.135131	-0.966607	0.333741	0.432563
14105	ABCF3	1031.221269	-0.131032551	0.101746	-1.287837	0.197803	0.274876
17248	IGDCC3	3.428200224	-0.131471853	0.34832	-0.377445	0.705843	0.802175
14015	NSMCE2	283.3136373	-0.13170601	0.1002	-1.314437	0.188699	0.26391
15002	CUX1	2266.520546	-0.131862621	0.130978	-1.006756	0.314052	0.410328
15100	NSA2	2189.953702	-0.132043612	0.135215	-0.976545	0.328794	0.426801
14807	ZNF622	827.3237198	-0.132084647	0.124239	-1.06315	0.287714	0.380866
14535	PHF13	452.6767754	-0.132107573	0.114384	-1.154947	0.248112	0.334589
15481	ITPRIPL2	1596.24391	-0.132144403	0.152547	-0.866252	0.386352	0.489173
16383	CES2	6346.449337	-0.132502813	0.219244	-0.604361	0.545603	0.652773
13806	VPS72	677.8175965	-0.132685792	0.095752	-1.385719	0.165833	0.23544
16532	MAK	5.020822332	-0.132731644	0.235406	-0.563842	0.572862	0.679208
13210	EIF2AK1	3596.463656	-0.132928399	0.084293	-1.576973	0.114802	0.170343
18524	OR2H1	0.521931405	-0.133145385	1.265772	-0.105189	0.916226	0.969496
14257	TRRAP	1694.683678	-0.133326678	0.107432	-1.241032	0.214594	0.295031
15920	TRIM69	32.41463368	-0.133545619	0.181438	-0.736039	0.461707	0.568463
13716	OSTF1	893.0373306	-0.133636899	0.094516	-1.41391	0.157388	0.224917
16790	PCDHB15	139.6497937	-0.133840573	0.271851	-0.492331	0.622486	0.726703
15628	C20orf12	124.1583588	-0.134017564	0.162087	-0.826823	0.408337	0.512136
14052	OTUB1	1551.899034	-0.134066481	0.103073	-1.300693	0.193364	0.269736
15966	C17orf72	61.16368726	-0.134162331	0.185432	-0.723511	0.469366	0.576227

12922	HECTD3	1160.221448	-0.134251616	0.080578	-1.666111	0.095691	0.145145
12957	CLASP1	1773.04626	-0.134498146	0.081293	-1.654488	0.098028	0.148295
13734	FBXL12	314.259356	-0.134563848	0.095704	-1.40604	0.159712	0.227939
17090	OSBPL6	30.87530472	-0.134564784	0.323693	-0.415717	0.677617	0.777178
16038	HELLS	64.70318111	-0.135077907	0.193026	-0.699791	0.484058	0.591596
11773	VPS33A	536.6750665	-0.135143964	0.066591	-2.029475	0.04241	0.07061
13458	GSK3A	1418.462983	-0.13521262	0.090151	-1.499847	0.133654	0.194661
14053	SLC35B2	1271.266416	-0.135491369	0.104193	-1.300387	0.193468	0.269848
13983	ERLIN1	1094.998022	-0.135600787	0.102433	-1.323798	0.18557	0.260112
12053	LOC49375	473.609049	-0.136069941	0.070038	-1.942809	0.052039	0.084628
15409	ANO6	5151.401923	-0.136215347	0.153561	-0.887043	0.375056	0.477089
17439	PTPN5	6.745172111	-0.136246629	0.413366	-0.329603	0.7417	0.833652
16410	HMGB3	406.081152	-0.136290983	0.227447	-0.599222	0.549025	0.655785
15996	SFXN5	405.9678092	-0.136413347	0.190986	-0.714259	0.475067	0.582132
13772	ORMDL3	1092.892206	-0.136650753	0.09793	-1.395396	0.162897	0.231826
16123	JAG2	737.4454567	-0.136654014	0.202552	-0.674661	0.499891	0.607726
13919	SKIV2L	867.1183191	-0.137072364	0.101993	-1.343935	0.17897	0.252028
15805	MZF1	300.1440133	-0.137311003	0.177348	-0.774246	0.438786	0.544172
15504	SH3BP2	2991.076426	-0.137442384	0.159819	-0.859988	0.389796	0.492801
14323	COASY	1351.461334	-0.137461422	0.112352	-1.223486	0.221146	0.302638
14741	DDX51	491.8377149	-0.137491459	0.126508	-1.086821	0.277116	0.368479
14806	PPP1R12C	1166.149503	-0.137524232	0.129343	-1.063249	0.287669	0.380832
11542	PWP1	1059.638217	-0.137819614	0.065618	-2.100323	0.0357	0.060628
13125	ZNF343	280.6909454	-0.138079343	0.086143	-1.6029	0.108957	0.162717
16503	TAGAP	260.9215016	-0.138169165	0.24153	-0.572058	0.567283	0.673775
16981	CD1C	27.03355638	-0.138190794	0.311451	-0.443701	0.657259	0.75866
17226	SYTL5	14.22450673	-0.13829365	0.361392	-0.382669	0.701965	0.798749
12384	UBE2Q1	1855.50734	-0.138299991	0.075129	-1.840835	0.065646	0.103902
16582	FAM162B	41.0828291	-0.138325864	0.250663	-0.55184	0.581058	0.686848
13890	ZNF362	687.9243506	-0.138352491	0.102373	-1.351459	0.176549	0.249138
13566	LOH12CR1	88.66703429	-0.138365321	0.094447	-1.464997	0.142922	0.206502
17053	KCNT1	2.533455977	-0.13849546	0.324534	-0.426752	0.66956	0.769579
11738	CNO	256.4415269	-0.138608291	0.067898	-2.041408	0.04121	0.068816
12933	AKIRIN2	610.7547291	-0.138975573	0.08352	-1.663976	0.096117	0.145663
14436	NT5C3L	812.3497264	-0.139122183	0.117154	-1.18752	0.235022	0.31911
15779	SOAT1	1514.256036	-0.139161342	0.178122	-0.781269	0.434644	0.539924
13583	C14orf166	1746.865324	-0.139182791	0.095349	-1.459715	0.144369	0.208332
14788	P4HTM	620.5838711	-0.139697089	0.130407	-1.071239	0.284062	0.376515
17590	C4orf22	1.046226438	-0.139908747	0.472832	-0.295895	0.76731	0.855034
17413	TM4SF5	176.5596209	-0.139980397	0.413833	-0.338253	0.735172	0.827549
16074	OC1001012	17.611077	-0.139984314	0.202538	-0.691152	0.48947	0.596871
16248	HSPA12B	242.9929025	-0.139994158	0.218555	-0.640545	0.521818	0.629486
13140	UIMC1	450.9223154	-0.140168164	0.087779	-1.59683	0.110304	0.16454
14724	ABHD4	906.7750166	-0.140184227	0.128527	-1.090699	0.275405	0.366577
16524	SLC6A10P	42.52594698	-0.14032666	0.248128	-0.565541	0.571706	0.678166
15373	CCDC112	188.3795968	-0.140696788	0.156575	-0.898591	0.368871	0.47032
13011	UBTF	2152.107942	-0.140730516	0.085814	-1.639939	0.101018	0.152183

16863	ATP8A2	18.95482061	-0.140870753	0.296234	-0.475539	0.634403	0.737409
14947	AXIN1	667.167152	-0.140912036	0.137756	-1.022911	0.30635	0.40171
15758	CD1D	172.0072697	-0.141187718	0.179391	-0.787037	0.43126	0.536432
15084	TEX2	1509.239876	-0.14137673	0.14417	-0.980626	0.326777	0.424633
14688	EIF3E	6033.546235	-0.141386907	0.128014	-1.10446	0.269393	0.359503
14661	TRIM34	195.1699025	-0.141415639	0.127045	-1.113113	0.26566	0.355173
16129	CYTSB	446.7437237	-0.141553507	0.210406	-0.672765	0.501097	0.608965
15117	CUTA	2149.083078	-0.141659159	0.146022	-0.97012	0.331987	0.43046
13568	TMUB2	949.396098	-0.141698147	0.09674	-1.464729	0.142995	0.206578
13508	ZNF16	228.9810817	-0.141882856	0.095518	-1.485411	0.137435	0.199427
14379	DHX32	885.7679081	-0.141894785	0.117919	-1.203322	0.228852	0.311963
16083	CCDC11	34.20987321	-0.141987734	0.206606	-0.687239	0.491932	0.599538
14190	GMPS	894.9562273	-0.142020427	0.112656	-1.260651	0.207435	0.286535
16358	CDHR3	96.92846758	-0.142214799	0.232505	-0.611662	0.540761	0.647968
13240	SFT2D3	341.2419653	-0.142477304	0.090938	-1.566755	0.117172	0.173466
13236	EIF4E2	1481.548592	-0.142537844	0.090924	-1.567654	0.116962	0.173194
15169	FOXP4	674.9890943	-0.142540818	0.149026	-0.956486	0.338827	0.437795
14692	ZBTB7B	1002.861663	-0.142558847	0.129272	-1.102783	0.270122	0.360377
17668	PLP1	3.20897674	-0.14256476	0.517056	-0.275724	0.78276	0.868423
14227	TMED2	6741.648642	-0.142618057	0.114043	-1.250565	0.211093	0.29083
12890	RBM22	1193.953438	-0.143230551	0.085458	-1.676042	0.09373	0.142529
16926	FOXC2	121.9821395	-0.14345604	0.313771	-0.4572	0.647528	0.749863
14880	TMED1	754.6935961	-0.143486225	0.137552	-1.043145	0.296881	0.391073
13371	CAMK2G	549.1464251	-0.143504361	0.094385	-1.520422	0.128405	0.188233
11617	C1orf77	1372.347793	-0.143525164	0.069178	-2.074737	0.038011	0.064135
11959	ANP32A	2564.951092	-0.143637612	0.072845	-1.971826	0.04863	0.079705
14356	LSM11	199.1986174	-0.143678025	0.1185	-1.212468	0.225333	0.307667
13430	MTHFSD	262.1169263	-0.143718139	0.095474	-1.505309	0.132245	0.19301
15188	SPA17	144.4151215	-0.143815649	0.151126	-0.951625	0.341287	0.440451
14763	LOC93622	258.239486	-0.143848663	0.133097	-1.08078	0.279795	0.371487
14564	FLYWCH1	756.4814878	-0.143883058	0.12542	-1.147208	0.251296	0.338207
14369	SRGAP2	1070.152172	-0.144138089	0.119379	-1.207396	0.22728	0.310036
13339	EIF2S3	4843.166345	-0.144699275	0.094473	-1.531649	0.125609	0.184576
15575	ZNF69	79.87169611	-0.144761341	0.171969	-0.84179	0.399906	0.503278
17322	CDHR5	3671.107503	-0.14487509	0.400563	-0.361679	0.717592	0.812003
14383	FANCL	251.4886019	-0.145479251	0.120973	-1.202574	0.229141	0.31225
16937	CCR3	2.774941599	-0.145481608	0.320565	-0.453828	0.649953	0.752183
15074	APOA1BP	1554.53512	-0.145483761	0.147675	-0.985162	0.324545	0.422011
13368	CHMP4A	971.6678567	-0.145560481	0.095682	-1.521288	0.128187	0.187945
14649	ARMC5	305.7446684	-0.145602442	0.130282	-1.117599	0.263738	0.352894
16858	ATP2A1	20.39792558	-0.145664121	0.305412	-0.476943	0.633403	0.736465
13123	PREP	623.6953761	-0.145756382	0.090902	-1.603449	0.108836	0.162561
15280	MRM1	135.2980762	-0.145757389	0.157492	-0.92549	0.354711	0.455007
14457	THOC7	778.0161312	-0.146240466	0.123958	-1.179757	0.238097	0.322815
14454	CHIC2	213.7768596	-0.146358537	0.123903	-1.181237	0.237509	0.322064
16466	C22orf36	531.1944432	-0.146418168	0.251352	-0.582523	0.560215	0.666875
14607	XAB2	1020.421296	-0.14647158	0.129402	-1.13191	0.257672	0.345786

13509	GRIPAP1	875.9905026	-0.146550912	0.098699	-1.48482	0.137592	0.199625
17481	CAPN6	412.6048796	-0.146880798	0.460942	-0.318653	0.749989	0.840908
16058	BST1	166.8127844	-0.146922993	0.211074	-0.696072	0.486384	0.593698
13634	CC2D1B	839.5313984	-0.147031865	0.10189	-1.443049	0.149007	0.21422
12665	KIAA1310	1455.882876	-0.14713899	0.084003	-1.751596	0.079843	0.12357
17651	SERPINE3	0.581058589	-0.147339141	0.527126	-0.279514	0.77985	0.866005
14800	FKBP4	2499.437298	-0.147474573	0.138443	-1.06524	0.286767	0.379792
13970	CFDP1	1372.095517	-0.14748652	0.111043	-1.328189	0.184116	0.258329
14963	HSBP1	3061.999257	-0.147613096	0.144999	-1.018031	0.308663	0.404338
12870	C12orf76	129.363625	-0.147926632	0.087685	-1.68702	0.0916	0.139506
13245	DGCR8	610.4225191	-0.147957812	0.09459	-1.564202	0.11777	0.174286
15051	RALGDS	1265.591764	-0.147968033	0.149432	-0.990205	0.322074	0.419461
12354	DEDD	715.1942865	-0.148099353	0.08012	-1.848479	0.064533	0.102389
12407	UBE2R2	1882.026763	-0.148131089	0.080773	-1.833914	0.066667	0.105322
16658	ZFP92	4.933187935	-0.148234305	0.278435	-0.532385	0.59446	0.699484
16339	EFCAB10	7.884842596	-0.148251241	0.240683	-0.615961	0.537921	0.645314
16426	CLU	34202.95006	-0.148355172	0.248543	-0.596899	0.550575	0.656996
16239	PARVB	921.1668304	-0.14849294	0.230511	-0.644189	0.519453	0.626996
16344	ADAP1	243.202132	-0.148662154	0.241654	-0.615185	0.538432	0.645701
15442	FBXW5	2208.853514	-0.148874513	0.17007	-0.875373	0.381371	0.484086
15490	GUCY1B3	1490.712258	-0.148878716	0.172078	-0.865182	0.386939	0.489631
14200	ATP2B4	3942.982745	-0.149094301	0.118557	-1.257572	0.208547	0.287868
14766	C15orf58	42.63016579	-0.149157142	0.138185	-1.0794	0.280409	0.372227
15465	AGFG2	488.5648316	-0.149557809	0.171685	-0.871117	0.38369	0.486306
17458	KRTAP5-1(	1.949813916	-0.149975916	0.461678	-0.32485	0.745295	0.836868
12250	NMT1	2425.065251	-0.150027764	0.07972	-1.881937	0.059845	0.095757
14709	USP35	168.1317472	-0.150036451	0.136987	-1.095257	0.273404	0.364334
16134	GUCA1B	21.53938431	-0.150162462	0.223653	-0.67141	0.50196	0.609825
15942	GJC1	731.2110939	-0.150232333	0.206444	-0.727714	0.466789	0.573926
12938	WSB2	2181.648623	-0.150377617	0.09042	-1.663095	0.096294	0.145884
14245	GNAI3	2142.381801	-0.150397891	0.120963	-1.243342	0.213742	0.294107
11932	THAP11	623.8239148	-0.150450116	0.076016	-1.979201	0.047793	0.078511
14597	AARS	3397.600994	-0.150611192	0.132546	-1.13629	0.255835	0.343538
16896	PLEKHG7	6.914770958	-0.150619353	0.323119	-0.466142	0.641114	0.743754
15426	C16orf7	428.8624249	-0.150866455	0.171023	-0.882139	0.377701	0.479925
14207	GPCPD1	864.0670954	-0.151134277	0.120475	-1.254484	0.209666	0.28927
14484	IGBP1	2094.038203	-0.151459596	0.129112	-1.173085	0.240762	0.32582
16034	PLAT	900.8429423	-0.151494686	0.215788	-0.702055	0.482645	0.590016
13018	STK38	1009.047557	-0.15151464	0.092478	-1.638384	0.101342	0.152589
16088	SDHAP1	250.9431602	-0.151616995	0.221214	-0.685386	0.4931	0.600774
17598	DCAF12L1	69.73476868	-0.151638339	0.516104	-0.293813	0.7689	0.856416
11136	NONO	5403.06051	-0.151798077	0.067738	-2.240974	0.025028	0.044053
17547	MEPE	1.113025349	-0.151887206	0.501857	-0.30265	0.762156	0.851372
14859	AIDA	1530.471154	-0.151922986	0.144662	-1.050194	0.293629	0.387336
15308	STXBP5	285.4037949	-0.15207501	0.165445	-0.919187	0.357998	0.458395
12701	DNAJC7	1630.464149	-0.152131028	0.087744	-1.733812	0.082951	0.128016
13159	PSME1	3740.267912	-0.152259778	0.09564	-1.592012	0.111382	0.16592

13222	ADSL	1017.206528	-0.152299445	0.096799	-1.573359	0.115636	0.171425
16959	PTPRVP	1.502344183	-0.152400534	0.33893	-0.449652	0.652962	0.754655
15705	LPAR6	1149.1449	-0.152725519	0.190024	-0.803715	0.421562	0.52614
12812	C1orf131	180.9550767	-0.153033567	0.089953	-1.701253	0.088896	0.136001
13722	HAX1	1664.421884	-0.153176264	0.108586	-1.410639	0.158351	0.226194
16683	ANP32C	1.670500832	-0.153279859	0.293182	-0.522816	0.601103	0.706241
15098	C6orf145	1296.814521	-0.153322222	0.156976	-0.976725	0.328706	0.426727
13175	RSL1D1	3810.098569	-0.153437873	0.096603	-1.58834	0.112209	0.166939
12912	PDE6D	360.6947382	-0.153986248	0.092247	-1.669288	0.09506	0.144306
10804	FTSJ2	628.9335747	-0.154064552	0.065277	-2.360184	0.018266	0.033139
15471	C7orf55	288.3474568	-0.154107633	0.177331	-0.869037	0.384827	0.487548
17426	TAS2R3	0.652990638	-0.154140646	0.462882	-0.333002	0.739133	0.831386
14213	NANOS1	120.951464	-0.154404844	0.123172	-1.253572	0.209998	0.289626
14448	C1orf151	1155.092466	-0.154453288	0.130575	-1.182872	0.23686	0.321338
15959	MAP3K14	1018.500691	-0.154471288	0.21316	-0.724673	0.468653	0.575568
16242	EGFR	2949.124442	-0.154535043	0.240304	-0.643083	0.52017	0.627747
16803	MGC27382	2.661340927	-0.154551742	0.316044	-0.48902	0.624827	0.728872
13714	RARS	1438.993828	-0.154596837	0.109305	-1.414363	0.157255	0.22476
17164	HCG22	1.826923229	-0.154763179	0.391762	-0.395044	0.692811	0.791178
16018	GSTM2	848.6220878	-0.154786234	0.219155	-0.706287	0.48001	0.587403
15488	COX5B	2996.489168	-0.154813686	0.178901	-0.865358	0.386843	0.489573
16728	TPH1	3.611946124	-0.154855554	0.301945	-0.512861	0.608049	0.71248
16548	POU3F1	9.278135744	-0.154979476	0.277206	-0.559077	0.576109	0.682398
13257	RBM28	363.0359133	-0.155130588	0.099341	-1.561599	0.118382	0.175033
15178	GLTPD1	641.4798434	-0.155243779	0.162813	-0.953513	0.34033	0.439506
12192	ANKRD11	2294.717387	-0.155254207	0.08175	-1.899123	0.057548	0.09252
16494	CPLX1	82.67403205	-0.155276216	0.270387	-0.574274	0.565783	0.672345
15703	ENG	7841.835568	-0.155416732	0.193037	-0.805115	0.420754	0.525198
17934	SNORD22	0.502063254	-0.15550535	0.703442	-0.221063	0.825043	0.901732
14889	TIMM10	515.7326827	-0.155748206	0.149768	-1.039927	0.298374	0.392802
14293	REPIN1	1885.539474	-0.155955015	0.12664	-1.231486	0.218141	0.299153
14441	PHRF1	1060.379661	-0.156161714	0.131823	-1.184636	0.236161	0.320546
17228	COX6A2	5.99917794	-0.1562868	0.409201	-0.381931	0.702512	0.799277
12114	FBXO42	490.1988985	-0.156292899	0.08125	-1.923616	0.054403	0.088026
12840	EIF4H	5562.35612	-0.156295017	0.092316	-1.693045	0.090447	0.138073
14937	HLA-C	33260.11617	-0.156489885	0.15278	-1.02428	0.305703	0.401055
13304	UBFD1	1457.155683	-0.156599498	0.101572	-1.541763	0.123131	0.181411
14461	HN1L	3260.853397	-0.15670075	0.132883	-1.179243	0.238302	0.323003
15729	DNM1P35	31.41242043	-0.156701253	0.197006	-0.795414	0.426373	0.531333
13483	PRIM2	117.7138235	-0.15673246	0.104964	-1.493204	0.135384	0.196815
11935	VPS25	921.0589752	-0.156770757	0.079226	-1.978777	0.047841	0.078563
14528	MGAT4B	2869.019289	-0.156892953	0.135622	-1.156836	0.247339	0.333707
14411	POP1	178.0644605	-0.156952209	0.131404	-1.194422	0.232313	0.315978
14712	ARMC9	218.5643566	-0.156995869	0.143469	-1.094286	0.27383	0.364827
16591	HAVCR2	1828.864926	-0.157065162	0.285407	-0.550321	0.5821	0.687706
17848	RAET1L	0.516772583	-0.157190661	0.665835	-0.236081	0.81337	0.893272
16004	PDXDC2	253.8607988	-0.157357182	0.221021	-0.711955	0.476493	0.583588

12764	EXOC7	3187.052883	-0.158003206	0.092136	-1.714891	0.086365	0.132626
17402	GDAP1L1	2.989393021	-0.158062383	0.46403	-0.340629	0.733383	0.826056
16880	CACNA2D1	197.4629437	-0.158097942	0.336336	-0.47006	0.638312	0.741206
14917	SURF2	210.9226961	-0.158160882	0.153306	-1.031667	0.302228	0.397144
15603	FKBP14	199.4356807	-0.158168894	0.18963	-0.834092	0.404229	0.507806
13724	MRP63	804.2088291	-0.158351281	0.112306	-1.409995	0.158541	0.226433
13589	E2F3	426.0828101	-0.158428063	0.10866	-1.458021	0.144835	0.208912
14474	GMDS	196.8134264	-0.158500979	0.134795	-1.175868	0.239648	0.324536
13997	CCDC41	177.0002741	-0.158636647	0.120297	-1.318711	0.187266	0.262242
17174	GPX2	8.788780108	-0.158673601	0.40342	-0.393321	0.694082	0.792169
15809	FLJ45244	57.87947417	-0.159119395	0.205686	-0.773603	0.439165	0.544505
14119	CLTA	2707.963378	-0.159219521	0.124235	-1.2816	0.199983	0.277631
16495	SGIP1	185.766662	-0.159638315	0.277998	-0.574242	0.565804	0.672345
14146	C19orf29	370.6011389	-0.159700664	0.125525	-1.27226	0.203281	0.28167
16789	RASAL1	301.0282791	-0.159709464	0.324353	-0.492394	0.622441	0.726694
12715	CWF19L1	429.70165	-0.159729916	0.092445	-1.727844	0.084016	0.129517
11624	GPKOW	434.6489535	-0.159744436	0.077136	-2.070937	0.038365	0.064693
17833	C17orf74	0.536429521	-0.159930897	0.66827	-0.239321	0.810857	0.891247
12792	GGA3	637.5464414	-0.159933086	0.093706	-1.706749	0.087869	0.13464
11912	DDX23	2056.806388	-0.1600086	0.080623	-1.984664	0.047182	0.077637
17237	FAM135B	43.48635776	-0.16002791	0.421551	-0.379617	0.70423	0.800812
15566	VEGFC	283.3434868	-0.160070714	0.189758	-0.843554	0.398919	0.502326
16735	NTRK3	42.28310287	-0.160073747	0.314007	-0.509778	0.610207	0.714728
14037	C2orf76	65.58262198	-0.160095013	0.122445	-1.307489	0.191047	0.266774
16894	KLHL14	34.79398564	-0.160151737	0.343049	-0.466848	0.640608	0.743256
14086	C17orf65	120.9406132	-0.160163342	0.123925	-1.292421	0.196211	0.273033
13901	FNBP4	1066.441968	-0.160286591	0.118865	-1.348481	0.177504	0.250288
15936	PRKCA	1186.47956	-0.160628836	0.219663	-0.731252	0.464625	0.571481
15757	LOC64699	13.12431319	-0.160743264	0.204094	-0.787594	0.430934	0.536063
17392	TTLL2	6.761446538	-0.160823042	0.468555	-0.343232	0.731424	0.824324
15355	ALX3	18.62485921	-0.160966393	0.178178	-0.903401	0.366313	0.467572
11594	SAFB	1451.980766	-0.161028322	0.077321	-2.082607	0.037287	0.063038
13863	CYTH3	747.3286393	-0.161135018	0.118124	-1.36412	0.17253	0.243941
15206	PRDXDD1P	47.5889439	-0.161229626	0.170473	-0.945777	0.344262	0.443765
15378	RPS10P7	28.09944764	-0.161436055	0.18043	-0.89473	0.370931	0.472794
14031	PEX14	384.4226761	-0.161482197	0.123252	-1.310184	0.190134	0.265613
14569	ASAP1	1445.931771	-0.161506322	0.140946	-1.14587	0.251849	0.338835
15076	MYO1B	2850.964304	-0.161606752	0.164152	-0.984492	0.324874	0.422383
17466	C14orf178	0.469846426	-0.161654468	0.500424	-0.323035	0.746669	0.83794
13099	STRN4	1461.987831	-0.161894174	0.100441	-1.61184	0.106997	0.160107
14756	ACACA	831.4489159	-0.162110646	0.149825	-1.081999	0.279253	0.370932
15520	ITM2C	2630.520689	-0.162140749	0.190144	-0.852728	0.39381	0.497363
15479	POPDC2	135.5530639	-0.162242664	0.187107	-0.867114	0.38588	0.488638
11800	EIF1AD	504.8517652	-0.162286773	0.080317	-2.02058	0.043323	0.071964
14590	SLC4A2	2680.743085	-0.16229123	0.142426	-1.139479	0.254503	0.341914
15331	ASPH	5524.077428	-0.162351144	0.178343	-0.91033	0.362649	0.463624
14685	CERK	1063.88277	-0.162366308	0.146979	-1.104688	0.269295	0.359445

13354	CLK3	846.5289139	-0.162398309	0.10626	-1.528317	0.126434	0.18558
15615	FAM162A	2013.601825	-0.162466738	0.195301	-0.831877	0.405479	0.508984
12620	CYFIP1	2979.414039	-0.162562561	0.092037	-1.76627	0.07735	0.120129
16113	PIGZ	64.75209683	-0.162635106	0.240331	-0.676713	0.498588	0.606518
12534	E2F4	1109.525079	-0.162883366	0.090758	-1.794698	0.072702	0.113693
13258	SUMO2	3108.186583	-0.162899605	0.104338	-1.561273	0.118459	0.175134
14778	C14orf156	521.5951568	-0.163018506	0.151682	-1.07474	0.282491	0.374661
16539	COL4A5	205.0734472	-0.163481115	0.290605	-0.562554	0.573739	0.679919
14038	PGM1	2990.608016	-0.163658648	0.125185	-1.307338	0.191098	0.266826
16507	FIGF	11.04121407	-0.164021083	0.287809	-0.569895	0.568749	0.675352
12975	C1orf52	278.6486058	-0.164045216	0.09935	-1.651185	0.098701	0.149105
16980	SERPINA1(	2.01900439	-0.164056114	0.369043	-0.444544	0.656649	0.758271
12506	RAE1	484.0027032	-0.164103041	0.091105	-1.801248	0.071664	0.112321
14848	C5orf13	2388.874197	-0.164193663	0.156021	-1.052381	0.292625	0.386297
15747	ERN1	177.7476372	-0.164204042	0.207617	-0.7909	0.429003	0.533999
17932	CEACAM8	0.426125826	-0.164222652	0.738209	-0.222461	0.823955	0.900644
14627	LOC90784	237.4523544	-0.164237635	0.146003	-1.124896	0.260633	0.349263
14903	CREB3L4	293.6282808	-0.16424538	0.158697	-1.034962	0.300687	0.395475
16642	SNCAIP	40.25952623	-0.164557355	0.306211	-0.537399	0.590992	0.696072
14389	TBCD	1304.821253	-0.164566315	0.136969	-1.201489	0.229562	0.312714
16107	PCDH18	363.2034448	-0.164583301	0.242617	-0.678368	0.497538	0.605466
15132	LUC7L	642.9071019	-0.16470824	0.170814	-0.964257	0.334917	0.433829
10522	RNF34	514.3788174	-0.16471274	0.066872	-2.463114	0.013774	0.025658
12015	NTAN1	666.556375	-0.164807672	0.084236	-1.95651	0.050405	0.08223
16381	MGC16121	49.19985562	-0.164985851	0.272664	-0.605089	0.54512	0.652274
13649	CTDP1	459.0335592	-0.165144536	0.114794	-1.438613	0.15026	0.215785
12796	PAF1	1358.187153	-0.165259663	0.09693	-1.704935	0.088207	0.135115
14617	SLC31A2	626.2613342	-0.165368718	0.146671	-1.127477	0.259541	0.348055
12674	ANXA11	3863.009772	-0.165487547	0.094784	-1.745944	0.080821	0.124994
17746	ZFY	193.9657637	-0.165736579	0.645753	-0.256656	0.797444	0.880801
16812	GDPD2	2.911115332	-0.165753835	0.340022	-0.487479	0.625919	0.729711
15159	DHRS3	2905.658067	-0.165848783	0.173113	-0.95804	0.338043	0.437098
14735	GAS2L1	623.8658338	-0.166046278	0.152542	-1.088526	0.276363	0.367603
17663	GSG1L	0.371929753	-0.166075671	0.600321	-0.276645	0.782053	0.867861
17750	OR13J1	0.373096654	-0.166075699	0.648168	-0.256223	0.797778	0.880926
17931	LOC15202	0.272684063	-0.166075844	0.746234	-0.222552	0.823884	0.900616
17892	LIM2	0.231619125	-0.166075854	0.724471	-0.229238	0.818684	0.896833
18068	C3orf16	0.249478459	-0.166075978	0.860571	-0.192983	0.846972	0.918834
18136	NHLH2	0.313581021	-0.166075989	0.93625	-0.177384	0.859207	0.928612
18158	OR9Q1	0.22884356	-0.166076122	0.960663	-0.172877	0.862748	0.931345
18133	HIST1H4A	0.199151621	-0.166076137	0.933143	-0.177975	0.858743	0.928276
18138	TAS2R42	0.200687412	-0.166076146	0.939902	-0.176695	0.859748	0.929095
18129	DNAI2	0.172979412	-0.166076161	0.932141	-0.178166	0.858592	0.928204
18190	SYCP3	0.232262084	-0.166076188	1.010575	-0.164338	0.869465	0.936909
18180	CD200R1L	0.177583289	-0.166076245	0.990122	-0.167733	0.866793	0.934544
18178	PIWIL3	0.155834935	-0.166076272	0.989371	-0.167861	0.866693	0.934514
18232	OC1001330	0.193774856	-0.166076321	1.059448	-0.156757	0.875436	0.941374

18378	MYBPC1	0.323792755	-0.166076375	1.252753	-0.132569	0.894534	0.954011
18285	LOC392196	0.135221749	-0.166076553	1.13517	-0.146301	0.883684	0.947284
18433	LUZP4	0.298462404	-0.166076562	1.346104	-0.123376	0.90181	0.95897
18341	BCAR4	0.156773721	-0.166076671	1.218577	-0.136287	0.891594	0.952033
18354	RXFP2	0.137955668	-0.166076715	1.219228	-0.136215	0.891652	0.952033
18460	C14orf166B	0.14855339	-0.166077148	1.420344	-0.116927	0.906918	0.962737
18475	MRGPRD	0.135519427	-0.166077172	1.430319	-0.116112	0.907564	0.962877
18611	OR11H4	0.116173703	-0.166078038	1.732201	-0.095877	0.923618	0.97275
18616	44166	0.102986517	-0.166078159	1.743718	-0.095244	0.924121	0.973018
18697	SPDYC	0.077159322	-0.166079091	1.993936	-0.083292	0.933619	0.979957
18676	CYCSP52	0.077106275	-0.166079092	1.994108	-0.083285	0.933625	0.979957
18726	NAA11	0.114908036	-0.16607941	2.150419	-0.077231	0.93844	0.981752
18750	CYP4F8	0.099203091	-0.166079642	2.176386	-0.07631	0.939173	0.981934
18747	BTBD17	0.096980422	-0.166079658	2.18063	-0.076161	0.939291	0.981934
18748	CELP	0.096758059	-0.16607968	2.186471	-0.075958	0.939453	0.981934
18754	H2BFM	0.093808736	-0.166079704	2.192645	-0.075744	0.939623	0.982052
18767	SERPINB11	0.078700834	-0.166080007	2.233714	-0.074352	0.940731	0.982489
18770	WNT8A	0.078022576	-0.166080017	2.236363	-0.074263	0.940801	0.982489
18769	SSX7	0.077855097	-0.16608002	2.237021	-0.074242	0.940818	0.982489
18765	OC90	0.077799663	-0.166080021	2.237239	-0.074234	0.940824	0.982489
18766	SCARNA1	0.077381927	-0.166080027	2.238891	-0.07418	0.940867	0.982489
18764	C6orf126	0.077349349	-0.166080028	2.23902	-0.074175	0.940871	0.982489
18816	KRTAP13-2	0.100251345	-0.166080806	2.463492	-0.067417	0.94625	0.985725
18817	METTL11B	0.098774098	-0.166080815	2.465685	-0.067357	0.946298	0.985725
18819	HCRTR2	0.099153428	-0.166080838	2.470846	-0.067216	0.94641	0.985771
18936	SLC6A5	0.080900648	-0.166081285	2.530913	-0.065621	0.947679	0.986718
18859	GPR32	0.077005521	-0.166081372	2.549907	-0.065132	0.948069	0.986718
18832	BTF3L1	0.058161851	-0.166082054	2.651799	-0.06263	0.950061	0.986718
18895	ODF1	0.058159229	-0.166082054	2.651818	-0.062629	0.950062	0.986718
18834	C14orf183	0.057175743	-0.166082089	2.658968	-0.062461	0.950196	0.986718
18882	LOC285692	0.057052084	-0.166082094	2.659878	-0.06244	0.950213	0.986718
18845	CXorf49B	0.056583003	-0.166082111	2.663353	-0.062358	0.950278	0.986718
18822	ACTL9	0.056472165	-0.166082115	2.66418	-0.062339	0.950293	0.986718
18907	OR6A2	0.056428661	-0.166082117	2.664505	-0.062331	0.950299	0.986718
18946	TAS2R8	0.056405896	-0.166082118	2.664675	-0.062327	0.950302	0.986718
18870	KPRP	0.116902525	-0.166082167	2.761402	-0.060144	0.952041	0.986718
18875	LBX1	0.097711984	-0.166082551	2.839942	-0.058481	0.953366	0.986718
18913	OTUD6A	0.076834467	-0.166083169	2.91407	-0.056994	0.95455	0.986718
18933	SIX6	0.079849212	-0.166083169	2.91407	-0.056994	0.95455	0.986718
18940	SNORA46	0.080294169	-0.166083169	2.91407	-0.056994	0.95455	0.986718
18896	OR10J3	0.075151695	-0.166083169	2.91407	-0.056994	0.95455	0.986718
18823	ADAM30	0.055307696	-0.166083414	2.91407	-0.056994	0.95455	0.986718
18830	BIRC8	0.059814823	-0.166083414	2.91407	-0.056994	0.95455	0.986718
18838	CELA2A	0.059350968	-0.166083414	2.91407	-0.056994	0.95455	0.986718
18850	DPPA5	0.054803385	-0.166083414	2.91407	-0.056994	0.95455	0.986718
18853	FAM25B	0.061064655	-0.166083414	2.91407	-0.056994	0.95455	0.986718
18872	KRT74	0.054661943	-0.166083414	2.91407	-0.056994	0.95455	0.986718

18881	LOC28537(	0.05480658	-0.166083414	2.91407	-0.056994	0.95455	0.986718
18904	OR52E6	0.057134904	-0.166083414	2.91407	-0.056994	0.95455	0.986718
18921	PRDM13	0.05926613	-0.166083414	2.91407	-0.056994	0.95455	0.986718
18938	SNORA27	0.056713559	-0.166083414	2.91407	-0.056994	0.95455	0.986718
18955	UBE2DNL	0.056725477	-0.166083414	2.91407	-0.056994	0.95455	0.986718
18958	WFDC8	0.058274744	-0.166083414	2.91407	-0.056994	0.95455	0.986718
18892	NCR2	0.056436099	-0.166083414	2.91407	-0.056994	0.95455	0.986718
18893	NDST4	0.056068868	-0.166083414	2.91407	-0.056994	0.95455	0.986718
18894	NXF4	0.056111345	-0.166083414	2.91407	-0.056994	0.95455	0.986718
18909	OR7E156P	0.05686431	-0.166083414	2.91407	-0.056994	0.95455	0.986718
18947	TAS2R9	0.058387757	-0.166083414	2.91407	-0.056994	0.95455	0.986718
18878	)C1001299	0.038530656	-0.166083659	2.91407	-0.056994	0.95455	0.986718
18864	IL1F10	0.038192494	-0.166083659	2.91407	-0.056994	0.95455	0.986718
18826	ALG1L2	0.038433304	-0.166083659	2.91407	-0.056994	0.95455	0.986718
18840	CIB3	0.037386878	-0.166083659	2.91407	-0.056994	0.95455	0.986718
18842	CRYGD	0.038328329	-0.166083659	2.91407	-0.056994	0.95455	0.986718
18855	FAM75A6	0.036412016	-0.166083659	2.91407	-0.056994	0.95455	0.986718
18858	GPR148	0.039108749	-0.166083659	2.91407	-0.056994	0.95455	0.986718
18860	HIST1H3I	0.037312786	-0.166083659	2.91407	-0.056994	0.95455	0.986718
18862	HTR3D	0.037040066	-0.166083659	2.91407	-0.056994	0.95455	0.986718
18863	IL17A	0.037752574	-0.166083659	2.91407	-0.056994	0.95455	0.986718
18866	INSL4	0.038215543	-0.166083659	2.91407	-0.056994	0.95455	0.986718
18871	KRT26	0.0377598	-0.166083659	2.91407	-0.056994	0.95455	0.986718
18879	LOC255025	0.037877616	-0.166083659	2.91407	-0.056994	0.95455	0.986718
18883	LOC285735	0.036300384	-0.166083659	2.91407	-0.056994	0.95455	0.986718
18887	MAGEB16	0.04004281	-0.166083659	2.91407	-0.056994	0.95455	0.986718
18901	OR2AK2	0.052350927	-0.166083659	2.91407	-0.056994	0.95455	0.986718
18905	OR5AU1	0.036225268	-0.166083659	2.91407	-0.056994	0.95455	0.986718
18908	OR6C75	0.03508121	-0.166083659	2.91407	-0.056994	0.95455	0.986718
18911	OTOP1	0.037099931	-0.166083659	2.91407	-0.056994	0.95455	0.986718
18917	PLAC1L	0.036242375	-0.166083659	2.91407	-0.056994	0.95455	0.986718
18928	SCGB1A1	0.040539474	-0.166083659	2.91407	-0.056994	0.95455	0.986718
18934	SLC17A6	0.035937317	-0.166083659	2.91407	-0.056994	0.95455	0.986718
18937	SNORA26	0.039957865	-0.166083659	2.91407	-0.056994	0.95455	0.986718
18941	SNORA71E	0.03957903	-0.166083659	2.91407	-0.056994	0.95455	0.986718
18942	SPANXC	0.037416355	-0.166083659	2.91407	-0.056994	0.95455	0.986718
18945	TAS2R7	0.041261944	-0.166083659	2.91407	-0.056994	0.95455	0.986718
18952	TPH2	0.036793251	-0.166083659	2.91407	-0.056994	0.95455	0.986718
18959	XAGE2	0.039016469	-0.166083659	2.91407	-0.056994	0.95455	0.986718
18961	ZNF679	0.037608765	-0.166083659	2.91407	-0.056994	0.95455	0.986718
18903	OR4D1	0.037171714	-0.166083659	2.91407	-0.056994	0.95455	0.986718
13156	PYCR2	1237.199644	-0.166324445	0.104451	-1.592375	0.1113	0.165826
12806	FTSJ3	981.7984111	-0.166454297	0.097785	-1.702247	0.088709	0.135779
14572	ARHGAP1(	389.8054608	-0.166562815	0.145456	-1.145107	0.252165	0.339185
15530	CITED2	2429.696261	-0.167094682	0.196245	-0.851458	0.394515	0.497932
16155	LRP4	317.051727	-0.167334734	0.251841	-0.664445	0.506405	0.614388
13834	POLR3K	219.7677214	-0.167524868	0.121993	-1.373239	0.169678	0.240412

15905	ANKRD37	355.1541763	-0.167527821	0.226123	-0.740872	0.458771	0.565309
14671	LOC144571	58.9079241	-0.167539055	0.150867	-1.110506	0.266781	0.356429
16730	HR	22.75811345	-0.167696519	0.327876	-0.511463	0.609027	0.71354
14967	NDUFB2	1704.463924	-0.167776299	0.164952	-1.017121	0.309096	0.404797
12996	SETD1A	725.7688799	-0.168003942	0.102058	-1.646156	0.099732	0.150419
14169	CTDSP1	3974.707897	-0.168563028	0.133181	-1.265665	0.205633	0.284467
13329	SUFU	741.9362101	-0.168587716	0.10991	-1.533875	0.12506	0.183908
17595	TMSB4Y	19.65654797	-0.168758582	0.572686	-0.294679	0.768239	0.855826
13350	SCMH1	1287.679039	-0.168782262	0.110395	-1.528901	0.126289	0.185423
14419	KIAA1841	147.4265742	-0.168900061	0.141658	-1.192311	0.233139	0.316927
11705	C2orf68	792.4538102	-0.168919685	0.082382	-2.050455	0.04032	0.067519
15527	KIAA0114	325.560832	-0.16893577	0.198308	-0.851885	0.394278	0.497729
16030	FBXO16	40.79451709	-0.16894625	0.240508	-0.702455	0.482395	0.589859
15768	SH2D3A	111.2014163	-0.169155727	0.215363	-0.785443	0.432194	0.537254
13478	PACS1	2337.439219	-0.1692195	0.113179	-1.495153	0.134875	0.196147
13594	IP6K2	1017.661099	-0.169330292	0.116202	-1.457201	0.145061	0.209161
14740	UACA	3163.958958	-0.169389257	0.155831	-1.087006	0.277034	0.368395
14223	SAT2	887.393189	-0.169535302	0.135505	-1.25114	0.210883	0.290638
15522	DAGLA	154.1688918	-0.169601924	0.198953	-0.852472	0.393952	0.497478
14686	GPR146	279.4537482	-0.169703228	0.153634	-1.104591	0.269337	0.359476
13964	LLGL1	471.4021435	-0.169710199	0.12756	-1.33043	0.183377	0.257403
13537	C21orf59	674.3854272	-0.169850273	0.115211	-1.474258	0.140412	0.203311
16327	EPHA1	58.85477201	-0.169872344	0.27372	-0.620607	0.534858	0.642113
13170	FLII	2909.842486	-0.17008825	0.107045	-1.588938	0.112074	0.166788
13096	CCDC59	468.2412208	-0.170111628	0.105486	-1.612649	0.106821	0.15988
13202	LRTOMT	200.9963754	-0.17028992	0.107883	-1.578469	0.114458	0.169936
12756	ASCC1	618.1783539	-0.170318578	0.099206	-1.716814	0.086013	0.132169
15354	THSD1	141.1568483	-0.170348099	0.188528	-0.903569	0.366224	0.467524
11744	TTC9C	310.2252631	-0.17052159	0.083662	-2.038214	0.041529	0.069312
13292	ARMC10	915.7544417	-0.170572475	0.110362	-1.545566	0.12221	0.180216
12424	PFDN1	1567.616779	-0.170602946	0.09326	-1.829327	0.067351	0.106257
16558	GJC3	1.589897133	-0.170605147	0.306359	-0.556879	0.57761	0.683762
14073	COMMD1	321.792058	-0.170667834	0.131687	-1.296012	0.194971	0.271558
14331	TMEM223	390.371977	-0.170722789	0.13977	-1.221455	0.221914	0.303519
16324	SLC29A4	940.5432205	-0.170774245	0.274691	-0.621695	0.534143	0.64137
16879	SLED1	6.635480411	-0.170813143	0.363309	-0.47016	0.638241	0.741167
13111	USP21	329.250793	-0.170903651	0.106309	-1.607616	0.107919	0.16134
16249	RHOBTB3	1237.82356	-0.170912493	0.266819	-0.640555	0.521812	0.629486
16020	SYT2	11.19858876	-0.17103683	0.242522	-0.705242	0.48066	0.588103
15816	TST	919.3612855	-0.171173117	0.221892	-0.771424	0.440456	0.545863
14268	CORO1C	4047.199314	-0.171286717	0.138383	-1.237777	0.215799	0.296459
15918	LMOD3	5.102943737	-0.171314925	0.232751	-0.736043	0.461705	0.568463
13434	LOC643837	137.749379	-0.171342605	0.1139	-1.504322	0.132498	0.193323
15563	OC1001328	4.653718526	-0.171902485	0.203616	-0.844246	0.398532	0.501935
14085	EHD1	1941.113422	-0.171923597	0.133017	-1.292494	0.196186	0.273017
14948	NDUFA13	2876.035941	-0.171947894	0.168095	-1.022922	0.306345	0.40171
14821	CCDC69	568.8487812	-0.172018066	0.162526	-1.058406	0.28987	0.383358

17412	FAM153C	26.6484135	-0.172032144	0.508146	-0.338549	0.73495	0.827346
12422	ACBD6	432.672718	-0.172066641	0.094055	-1.829419	0.067337	0.106253
15007	ANO8	207.25354	-0.172687191	0.171622	-1.006204	0.314317	0.410538
13391	RPL22	6667.317203	-0.172860241	0.114207	-1.513576	0.130134	0.190482
13790	EDC4	1197.979417	-0.17295715	0.124518	-1.389009	0.16483	0.234288
16306	SEC31B	154.9701173	-0.173274648	0.276724	-0.626164	0.531207	0.638551
15153	LMF1	428.391192	-0.173333409	0.180793	-0.95874	0.33769	0.436816
14481	ZNF487	41.81013978	-0.173343563	0.147717	-1.173484	0.240602	0.325671
12390	CDK5RAP1	405.3925701	-0.173473908	0.094334	-1.838928	0.065926	0.104295
16761	CRNA0017	38.93849224	-0.173508774	0.346784	-0.500336	0.616838	0.721356
15526	HOXD9	417.9235756	-0.173632614	0.203796	-0.851992	0.394218	0.497686
11921	C16orf88	261.6806701	-0.173744013	0.087659	-1.982043	0.047474	0.078059
13884	TMEM104	439.8800705	-0.173789018	0.128434	-1.353138	0.176012	0.248488
12271	COPS7A	1685.279422	-0.174248692	0.09291	-1.875464	0.060729	0.097005
15027	ATG16L1	964.2848974	-0.174382792	0.174742	-0.997943	0.318307	0.415195
13145	NEDD8	1673.633929	-0.174478302	0.109321	-1.596015	0.110485	0.164749
13991	GBA	2119.513926	-0.17451184	0.132139	-1.32067	0.186612	0.261438
17610	TMEM179	2.399809688	-0.174632734	0.598425	-0.291821	0.770424	0.857528
16231	COX4I2	147.2429809	-0.17467356	0.270551	-0.645622	0.518524	0.626184
15414	KBTBD8	55.66420155	-0.174921604	0.19769	-0.88483	0.376248	0.478451
12253	POM121	2535.048046	-0.175196378	0.09312	-1.881394	0.059918	0.095851
12607	TMEM138	458.0020978	-0.175213655	0.09904	-1.769125	0.076873	0.11952
13460	RAD51L3	184.7275023	-0.175283681	0.116926	-1.499095	0.133849	0.194916
15165	PRSS23	5037.133194	-0.175438603	0.183284	-0.957194	0.338469	0.437477
12591	AK2	2833.100685	-0.175753317	0.098927	-1.776592	0.075635	0.117745
13908	MPDU1	1477.223812	-0.175866711	0.130481	-1.347835	0.177711	0.250454
13697	SFT2D2	104.8971641	-0.175880735	0.124014	-1.418237	0.156122	0.223417
15215	MGC23270	26.76157759	-0.176090806	0.186565	-0.94386	0.345241	0.444763
13165	SLC35E2	1544.198791	-0.176117179	0.110704	-1.59089	0.111634	0.166216
13168	TLN1	10048.94022	-0.176177737	0.110814	-1.58985	0.111869	0.16652
8559	KHSRP	1772.030796	-0.176209928	0.055299	-3.186492	0.00144	0.003298
13925	ZNRF1	282.6387381	-0.176270952	0.131295	-1.342561	0.179414	0.252546
10235	SF3A3	1433.122519	-0.176301303	0.068578	-2.570826	0.010146	0.01943
12098	SRP19	566.9321581	-0.176324087	0.091355	-1.930088	0.053596	0.086835
11736	FKBP15	1143.974476	-0.176380031	0.086385	-2.041798	0.041172	0.068763
14019	C12orf44	792.6031235	-0.176514384	0.134412	-1.313234	0.189104	0.264401
16807	PCDHA1	12.33873896	-0.176563921	0.361278	-0.488721	0.62504	0.728903
12658	CBY1	537.2571336	-0.176789565	0.100742	-1.754882	0.07928	0.122765
14269	TECPR1	305.3192756	-0.176880041	0.142948	-1.237375	0.215948	0.296643
12207	OSGEP	330.8690994	-0.17729596	0.09358	-1.894587	0.058147	0.093368
13670	SOD1	4304.769617	-0.177356297	0.123876	-1.431727	0.152222	0.218267
15699	SERTAD1	595.7035264	-0.177376461	0.219907	-0.806598	0.419898	0.524264
13768	BCL2L11	886.7537086	-0.17742027	0.127054	-1.396418	0.162589	0.231455
14679	CTSH	2958.169442	-0.178056064	0.160611	-1.108618	0.267595	0.357322
13694	LIX1L	882.037808	-0.178123762	0.125528	-1.418991	0.155902	0.223151
16877	XK	51.4615309	-0.178171406	0.378559	-0.470658	0.637885	0.740842
15785	FBXO32	265.441066	-0.178249628	0.22858	-0.779812	0.435502	0.540726

10331	MKRN1	2282.46473	-0.178367501	0.070327	-2.536276	0.011204	0.021257
14059	GTPBP2	912.4114753	-0.178434644	0.13732	-1.29941	0.193803	0.270195
14033	CAMLG	1142.912036	-0.178489115	0.136407	-1.308506	0.190702	0.266368
12686	WHSC2	516.6391063	-0.178513032	0.102465	-1.742192	0.081475	0.125886
12062	HEATR6	384.0513042	-0.178638175	0.092078	-1.940067	0.052371	0.085105
11837	KDM2B	578.4823364	-0.178661696	0.088919	-2.009263	0.044509	0.073697
14563	ZNF547	54.01110093	-0.178722641	0.155736	-1.147603	0.251132	0.338011
14133	TAPBPL	932.9899813	-0.178780592	0.139803	-1.278801	0.200967	0.27872
16112	FAM24B	30.2774479	-0.178782708	0.264135	-0.676861	0.498494	0.606441
13553	PRR12	672.088497	-0.178854864	0.121657	-1.470162	0.141518	0.20467
11655	MAP4	6267.408357	-0.178916398	0.086871	-2.059573	0.039439	0.066328
11804	POM121C	1059.213823	-0.178918837	0.088593	-2.01957	0.043428	0.072114
11599	C1orf50	153.3670281	-0.178969377	0.086027	-2.080377	0.037491	0.06335
16283	NRTN	39.91103453	-0.179175114	0.283134	-0.632829	0.526845	0.634201
13662	DIMT1L	384.9092737	-0.179402947	0.125014	-1.43506	0.15127	0.217029
15662	PCDHB10	170.591568	-0.179479155	0.219288	-0.818464	0.413092	0.516985
14871	SKIL	586.9588357	-0.17966184	0.171842	-1.045507	0.295789	0.38987
12997	MKS1	240.994105	-0.179822078	0.10924	-1.646118	0.099739	0.150419
13935	STUB1	1511.923571	-0.179826444	0.134174	-1.34025	0.180164	0.253419
16568	RGS11	19.8470693	-0.17999711	0.324383	-0.554891	0.578969	0.684984
16634	MMRN1	405.121795	-0.180185529	0.333538	-0.540225	0.589042	0.694109
15862	NKPD1	4.031330691	-0.18023558	0.23871	-0.755041	0.450224	0.556352
15524	SLC24A4	11.12899279	-0.180622178	0.211944	-0.852217	0.394094	0.497593
12227	INPP5B	408.1528507	-0.180657717	0.095625	-1.889238	0.05886	0.094358
16354	AGAP7	11.73416289	-0.180765847	0.295104	-0.612549	0.540175	0.647424
15646	CRNA0011	33.20425747	-0.180810564	0.219497	-0.823749	0.410082	0.513753
16360	REPS2	1312.488393	-0.180941828	0.295946	-0.611402	0.540934	0.648095
14366	NARFL	567.1769655	-0.181231207	0.14998	-1.208367	0.226906	0.309583
16261	RASSF9	29.1504964	-0.18126467	0.284331	-0.637514	0.52379	0.631376
11426	RNF10	3656.763966	-0.181271394	0.084697	-2.140243	0.032335	0.05547
15407	KRT8	11784.168	-0.181297939	0.204286	-0.887471	0.374825	0.476858
14278	USMG5	903.5154747	-0.181319136	0.146813	-1.235033	0.216818	0.297651
13869	TSPAN9	4094.440165	-0.181365092	0.133189	-1.361717	0.173287	0.244906
16258	NOS2	57.63452519	-0.181582339	0.284478	-0.638301	0.523278	0.630903
11459	TBC1D20	1409.156834	-0.181709572	0.085237	-2.13182	0.033022	0.056488
17367	DEFA1B	4.84790995	-0.181867281	0.52152	-0.348725	0.727295	0.820816
16012	SCARNA9	15.99214143	-0.181941743	0.256936	-0.708121	0.47887	0.586206
15867	ID1	708.5944832	-0.181961355	0.241454	-0.753607	0.451085	0.55724
12799	TRAF3	226.9531143	-0.182308358	0.106986	-1.704035	0.088374	0.135341
16557	ACCN3	16.42624174	-0.182338513	0.327409	-0.556913	0.577587	0.683762
12788	STK25	1891.097709	-0.182427776	0.10681	-1.707972	0.087642	0.134338
15948	CLDND2	3.491218271	-0.182518579	0.251193	-0.726606	0.467467	0.574494
14013	MRPL21	580.5997175	-0.182531214	0.138837	-1.314715	0.188606	0.263816
12768	LMAN2L	902.244617	-0.182566238	0.10654	-1.713597	0.086603	0.13295
12112	ACTR5	205.2516061	-0.182756895	0.094975	-1.924266	0.054321	0.087909
13203	DMAP1	570.2037583	-0.182785183	0.115804	-1.578407	0.114472	0.169944
14076	SH2B3	1910.465893	-0.182818687	0.141183	-1.294903	0.195354	0.272032

12319	BACE1	1254.958921	-0.182987583	0.098412	-1.859404	0.06297	0.100193
13897	ATG4B	909.1663626	-0.182989998	0.135593	-1.349556	0.177158	0.249873
12946	RRM1	1058.175889	-0.183033895	0.110257	-1.660058	0.096903	0.146716
13310	FRMD8	888.5656657	-0.183050041	0.118879	-1.539797	0.12361	0.182034
14176	PHTF2	651.3687483	-0.183051476	0.144827	-1.263931	0.206255	0.285186
14992	ABCA1	2855.778845	-0.183552982	0.181987	-1.008606	0.313164	0.40944
16166	C11orf41	28.13573549	-0.183600158	0.277031	-0.662742	0.507496	0.615283
15909	GPR21	9.377741183	-0.183624483	0.248555	-0.738768	0.460048	0.566811
11872	GPN2	231.7545831	-0.183629776	0.091851	-1.999224	0.045584	0.075261
17995	GDNF	1.055426927	-0.183658281	0.889465	-0.206482	0.836415	0.911062
11406	RAB35	1134.529022	-0.183693615	0.085547	-2.147284	0.031771	0.054598
11635	MCM3	1158.767743	-0.183764002	0.088919	-2.066649	0.038767	0.06531
12213	SDHAF2	602.8047274	-0.183776915	0.097117	-1.892326	0.058448	0.093797
12230	ATP2A2	6639.312488	-0.184112339	0.097508	-1.888186	0.059001	0.094561
10679	UTP11L	486.2616281	-0.184189812	0.076668	-2.40243	0.016287	0.029894
11137	DCTN2	2561.686202	-0.184220133	0.082209	-2.240889	0.025033	0.044058
15429	CCDC103	243.4321662	-0.184260665	0.20917	-0.880912	0.378366	0.480676
14032	MRPS33	792.7985356	-0.184266306	0.140744	-1.309227	0.190457	0.266046
14365	CDRT4	175.4252698	-0.18432731	0.152499	-1.208714	0.226773	0.309431
12851	ZNF34	125.4699392	-0.184733114	0.109255	-1.690842	0.090867	0.138606
11112	RELA	1971.362111	-0.184796412	0.082204	-2.248023	0.024575	0.043349
14771	GIMAP4	1712.944099	-0.184874026	0.171684	-1.076829	0.281557	0.373624
11013	DDX19B	813.719066	-0.184896138	0.080879	-2.286071	0.02225	0.039601
12979	TULP3	874.7508724	-0.185020533	0.112102	-1.650459	0.098849	0.149286
12471	SUMO3	2275.457911	-0.185028609	0.102153	-1.811296	0.070095	0.11017
15125	SPATA2L	281.853451	-0.185098623	0.191525	-0.966446	0.333821	0.43261
12584	STX5	886.1296756	-0.185099804	0.104102	-1.778062	0.075394	0.117434
13922	TMEM49	2782.139919	-0.18513305	0.137836	-1.343143	0.179226	0.252335
14134	SFI1	323.7965778	-0.185174522	0.144836	-1.278515	0.201068	0.278841
13542	METTL5	568.9912956	-0.185229098	0.125754	-1.472952	0.140764	0.203745
12867	MYST1	665.1801761	-0.185239998	0.109724	-1.688242	0.091365	0.139181
14168	SERTAD3	539.0317404	-0.185278182	0.146352	-1.265978	0.205521	0.284332
13392	LZTR1	1139.302561	-0.18541098	0.122562	-1.512797	0.130331	0.190749
10807	MADD	1021.011554	-0.185625031	0.078675	-2.35939	0.018305	0.0332
12222	BRIX1	528.9209777	-0.185758982	0.098252	-1.89063	0.058674	0.094098
12272	WBP2	3269.864973	-0.185762427	0.09907	-1.875055	0.060785	0.097087
13993	CCDC142	176.8457556	-0.185823414	0.140769	-1.320062	0.186814	0.261684
16031	ANKRD36B	31.4242728	-0.185840935	0.264686	-0.702119	0.482605	0.590004
16590	C12orf74	1.403536368	-0.185942075	0.337841	-0.550383	0.582057	0.687697
14754	DLEU2	47.58467157	-0.186165867	0.172029	-1.082177	0.279174	0.370887
12035	TCF25	1974.360427	-0.186214143	0.09552	-1.949476	0.051239	0.083451
12480	STIP1	2252.834822	-0.186223857	0.102952	-1.808836	0.070476	0.11069
14229	LAP3	2626.389902	-0.1862948	0.149104	-1.249425	0.21151	0.291363
13067	MRPL10	1016.935976	-0.186462552	0.114823	-1.62392	0.104393	0.156593
15105	ZNF767	214.3949785	-0.186664055	0.191513	-0.974681	0.329719	0.427859
11267	SF3B3	3271.060091	-0.186733274	0.085159	-2.192763	0.028324	0.049276
12076	PAXIP1	314.9084634	-0.186757862	0.096485	-1.935612	0.052915	0.085892

11583	POLD3	386.094192	-0.186827648	0.089452	-2.088568	0.036747	0.062183
10662	TTC4	503.4745927	-0.187252041	0.077796	-2.406952	0.016086	0.029573
14805	JDP2	385.1461444	-0.187256641	0.176042	-1.063706	0.287462	0.380584
13225	ABCC1	1648.356511	-0.187257582	0.119091	-1.572395	0.115859	0.171716
17473	ZNF648	2.117558036	-0.187332962	0.584905	-0.320279	0.748757	0.839946
12038	GLRX3	792.6396733	-0.187432047	0.096173	-1.948902	0.051307	0.083541
16175	DGCR9	193.2702464	-0.18745669	0.283536	-0.66114	0.508523	0.616244
17071	HGFAC	2.541465644	-0.187518768	0.445598	-0.420825	0.673883	0.773755
14587	IFI27L1	95.48127087	-0.187690037	0.16455	-1.140626	0.254026	0.341342
15688	PEX11G	86.12024565	-0.187700992	0.231529	-0.810702	0.417537	0.521682
11916	TOMM6	1432.77816	-0.187934644	0.094757	-1.983327	0.047331	0.07786
15167	SLC16A1	2640.453934	-0.187968852	0.196467	-0.956746	0.338696	0.437712
12511	KIAA0146	661.6917165	-0.188692325	0.104799	-1.800513	0.07178	0.112457
13129	WBP1	1495.832028	-0.188791106	0.117979	-1.600212	0.109552	0.163556
14082	SARM1	590.6850042	-0.18881244	0.14602	-1.293058	0.195991	0.272804
14352	RAB23	328.8109678	-0.18892592	0.155779	-1.212782	0.225213	0.307562
12536	ING4	573.3382215	-0.18902131	0.105358	-1.794086	0.072799	0.113818
15825	MFSD3	229.1684304	-0.189128887	0.246446	-0.767425	0.442829	0.548493
15236	AFARP1	7.888394309	-0.189149873	0.201805	-0.937288	0.34861	0.448478
12874	ZNF232	145.8457504	-0.189355284	0.112396	-1.684713	0.092044	0.140139
15638	C14orf181	41.53038752	-0.189435327	0.229692	-0.824737	0.409521	0.513283
13444	DHPS	861.387008	-0.18950123	0.126136	-1.502361	0.133004	0.193916
11853	MRPS7	1117.908425	-0.189650349	0.094591	-2.004941	0.044969	0.074365
13791	C11orf2	1811.870387	-0.189654975	0.136565	-1.388757	0.164907	0.23438
12097	RBM34	691.7711798	-0.189727275	0.098289	-1.930306	0.053569	0.086799
16692	HIST1H4I	1.355378753	-0.189841572	0.364674	-0.520579	0.60266	0.707693
10862	TDP1	293.6132954	-0.189899141	0.081297	-2.335879	0.019498	0.035184
16212	CCDC36	11.29643284	-0.189966266	0.291313	-0.652103	0.514334	0.621852
15565	ATG16L2	439.8735095	-0.19051794	0.225846	-0.843575	0.398907	0.502326
12244	TRIM21	623.2276536	-0.190545423	0.101134	-1.884083	0.059554	0.095338
14228	AES	7723.541527	-0.1905664	0.15252	-1.249451	0.2115	0.291363
16237	FSTL3	2765.285295	-0.190776018	0.295843	-0.644855	0.519021	0.626532
10938	CCDC97	678.1568278	-0.190849433	0.08271	-2.30744	0.02103	0.037687
16332	MAPK12	357.9431204	-0.191110939	0.309426	-0.617631	0.536818	0.644267
11904	U2AF1	1319.14392	-0.191235464	0.09628	-1.986239	0.047007	0.077401
15453	LOC72872?	23.7151977	-0.191255332	0.218856	-0.873885	0.382181	0.484768
12843	FARSB	824.2881613	-0.19139817	0.113087	-1.69248	0.090555	0.138204
13609	BOLA1	217.6218282	-0.191456115	0.131787	-1.452772	0.146287	0.210697
14936	PRDM1	724.1390636	-0.191700316	0.187081	-1.024691	0.305509	0.40093
10767	PRKACA	1845.09044	-0.191864547	0.080745	-2.376168	0.017493	0.031846
11659	CLNS1A	1247.692906	-0.191975617	0.093224	-2.059285	0.039467	0.066352
14559	ST5	1008.23363	-0.192024851	0.167238	-1.148211	0.250882	0.337742
11605	ATF6B	1605.853045	-0.192521683	0.092592	-2.079241	0.037595	0.063497
16133	CX3CR1	392.4474859	-0.192525283	0.286698	-0.671526	0.501886	0.609773
11022	AMZ2	1566.294077	-0.192601876	0.084377	-2.282632	0.022452	0.039928
14012	AGXT2L2	1007.264316	-0.19266948	0.146537	-1.314821	0.18857	0.263785
11707	TRIM62	142.4652899	-0.19315545	0.094203	-2.050407	0.040325	0.067519

15954	HERC2P2	795.8238126	-0.193348979	0.266325	-0.725988	0.467846	0.574757
13856	RPL36AL	3290.535417	-0.193384392	0.141518	-1.366498	0.171783	0.243008
13016	BYSL	329.0463363	-0.193605205	0.118133	-1.638869	0.101241	0.15246
14208	PFKL	6436.104445	-0.193631764	0.154401	-1.254085	0.209811	0.28945
17565	ALPI	365.0076368	-0.193689923	0.647302	-0.299226	0.764767	0.853233
11519	ATP1B3	1995.24124	-0.193841582	0.091842	-2.110602	0.034807	0.059228
11944	RBM33	1154.391321	-0.194021659	0.098099	-1.977809	0.04795	0.07869
16375	TEKT4	5.642557055	-0.194023662	0.319574	-0.607132	0.543764	0.650889
11377	JAGN1	635.0858746	-0.19419731	0.089916	-2.159753	0.030792	0.05305
13467	AKAP8L	760.7889742	-0.194395948	0.129849	-1.497087	0.134371	0.195565
13975	TTLL4	346.2114355	-0.19453887	0.146734	-1.325792	0.184909	0.259348
15988	CCDC116	3.88443926	-0.194720961	0.27205	-0.715754	0.474144	0.581291
11825	YARS2	331.9995777	-0.194793461	0.096777	-2.012798	0.044136	0.073159
16066	STEAP4	960.7105213	-0.194832381	0.280956	-0.693463	0.488019	0.595492
16910	SEPT7L	0.650767751	-0.195042383	0.421591	-0.462634	0.643627	0.746051
14138	IL17RC	559.5639519	-0.195120494	0.152905	-1.276087	0.201925	0.27995
13550	LASS2	8733.385365	-0.19516537	0.132664	-1.47112	0.141259	0.20434
13546	RPF2	492.3676145	-0.195167229	0.132557	-1.472322	0.140934	0.203931
15205	HES1	968.3456044	-0.195214427	0.206403	-0.945792	0.344255	0.443765
14901	DPYD	786.7601177	-0.195219559	0.188434	-1.036012	0.300196	0.394883
13355	GGA1	992.9873598	-0.19534153	0.128005	-1.526051	0.126997	0.186392
11836	POMP	1747.930711	-0.195623503	0.097351	-2.009459	0.044489	0.073679
15305	RNF144A	537.6932741	-0.195754621	0.21284	-0.919727	0.357715	0.458123
9637	NUDT3	479.8507956	-0.195773972	0.070767	-2.766472	0.005667	0.011526
11346	ZNF7	382.517383	-0.196019239	0.090465	-2.166801	0.03025	0.052259
15404	GIPC3	392.7013964	-0.196039427	0.220342	-0.889706	0.373624	0.475422
13229	SLC1A4	782.0048958	-0.196178613	0.124797	-1.571976	0.115956	0.171809
13988	TCEA2	361.5041467	-0.197147661	0.149164	-1.321688	0.186272	0.261018
12507	IRF9	1108.341007	-0.197228338	0.109513	-1.800963	0.071709	0.112382
13278	TRMU	320.2455121	-0.197556513	0.127329	-1.551543	0.120772	0.178283
12447	BLVRA	1087.592251	-0.197596168	0.10848	-1.821496	0.068532	0.107921
11527	SNRPD3	1252.812258	-0.197636688	0.093729	-2.108608	0.034978	0.059479
16456	CLRN3	970.096873	-0.197657539	0.337562	-0.585545	0.558181	0.664859
14727	PTPRS	1059.655721	-0.197677433	0.181291	-1.090389	0.275542	0.366734
13408	PARP14	3276.498212	-0.197856035	0.131003	-1.510321	0.130961	0.191442
13775	LOC152217	259.7536536	-0.197943	0.141912	-1.394832	0.163067	0.232034
11902	TMEM184B	1833.313318	-0.197944915	0.099613	-1.987138	0.046907	0.07725
14824	KIAA1462	2112.286548	-0.198008686	0.187194	-1.05777	0.29016	0.383664
11288	PDCD11	803.3646448	-0.19801727	0.090717	-2.182806	0.02905	0.050444
15365	RPS2P32	25.67924308	-0.198062405	0.219933	-0.900556	0.367824	0.46923
13491	ARF4	3501.663445	-0.198099267	0.132893	-1.490671	0.136048	0.197663
14271	TRERF1	228.158429	-0.198116549	0.160186	-1.236788	0.216166	0.2969
16422	STOX1	45.55688242	-0.19820352	0.331585	-0.597746	0.55001	0.656482
15006	LOC441294	71.80088096	-0.198306649	0.197057	-1.006343	0.31425	0.410477
14477	CDH24	126.593073	-0.198376974	0.16882	-1.175077	0.239964	0.324897
15992	ABCD2	33.52777595	-0.19842735	0.277674	-0.714606	0.474853	0.581959
11896	FBXW8	436.7951858	-0.198499087	0.099764	-1.989686	0.046626	0.076825

15257	PALM	426.1071066	-0.198856528	0.213487	-0.931469	0.351611	0.451722
11012	SNUPN	440.1448786	-0.19893112	0.087009	-2.286321	0.022236	0.039578
17790	OPRK1	0.501064427	-0.199289612	0.805879	-0.247295	0.80468	0.886568
15750	HIST1H2BI	7.005282783	-0.199314467	0.25224	-0.790178	0.429424	0.534421
14910	HOXA4	397.5006312	-0.199394381	0.192952	-1.03339	0.301421	0.396255
13648	NDUFB10	1799.357203	-0.199493557	0.138665	-1.438677	0.150242	0.215775
11344	RANBP3	967.3377588	-0.199557265	0.092054	-2.167837	0.030171	0.052132
13079	ILKAP	379.1607275	-0.19957484	0.123206	-1.619846	0.105265	0.157753
11924	POLR3C	524.3258622	-0.19964623	0.100779	-1.98102	0.047589	0.078228
13105	RAB43	311.5606941	-0.199734143	0.124124	-1.609144	0.107585	0.160917
14152	METTL11A	361.4164654	-0.199828036	0.15726	-1.270688	0.20384	0.282325
14282	CEP110	288.6193093	-0.199896722	0.161942	-1.234373	0.217064	0.297904
14439	C14orf149	177.1558775	-0.200065513	0.168606	-1.186584	0.235392	0.319545
7334	EIF2B1	1031.771562	-0.200157594	0.054279	-3.68754	0.000226	0.000605
11657	SURF4	5276.989534	-0.200462582	0.097341	-2.059383	0.039458	0.066347
12898	SLC39A1	4096.520575	-0.200465463	0.119782	-1.673585	0.094212	0.143175
15046	PCYT2	521.4227729	-0.200521832	0.202006	-0.992651	0.32088	0.418023
13466	XRCC6BP1	121.9065383	-0.200624992	0.134003	-1.497164	0.134351	0.19556
14275	GALNACT	740.5487221	-0.200843052	0.162566	-1.235454	0.216662	0.297498
13027	SSBP3	590.5159807	-0.200999935	0.122938	-1.634972	0.102055	0.153556
12439	REXO4	523.9241869	-0.201025765	0.110155	-1.824929	0.068012	0.107171
13104	ALG12	743.6639493	-0.201046025	0.124941	-1.609132	0.107587	0.160917
14612	CAMKK1	293.8654259	-0.201100223	0.178052	-1.129444	0.258711	0.347043
12866	DCAKD	663.6463094	-0.2012164	0.119185	-1.688268	0.09136	0.139181
13825	NPM1	12562.44563	-0.201378013	0.14617	-1.377695	0.168297	0.238611
15026	DECR2	940.4478325	-0.201477212	0.201799	-0.998405	0.318083	0.414931
13898	LONP1	2341.757027	-0.201604933	0.149397	-1.349454	0.177191	0.249901
11430	LEMD2	904.7322702	-0.201604975	0.094224	-2.139629	0.032385	0.055536
11701	DERL2	711.4819732	-0.201614564	0.098294	-2.051135	0.040254	0.067426
11720	ATG10	129.4309091	-0.201675171	0.098557	-2.046273	0.04073	0.068118
12033	ZNF691	230.6792978	-0.201905608	0.103437	-1.951975	0.050941	0.08298
16303	KIAA1875	22.87727142	-0.202354677	0.323053	-0.626383	0.531064	0.638495
15571	GPR63	6.249679531	-0.202361407	0.240195	-0.842487	0.399515	0.502915
12861	HAUS1	286.868958	-0.20238418	0.119802	-1.689327	0.091157	0.138924
12825	SCAF1	1313.001522	-0.20247532	0.119298	-1.697222	0.089655	0.137023
15766	LOC25688(	4.656470019	-0.202565044	0.257745	-0.785911	0.431919	0.536982
15700	TCF7	236.1321809	-0.202606266	0.251218	-0.806496	0.419957	0.524304
14558	HOXC9	122.2932147	-0.202954042	0.176735	-1.148352	0.250823	0.337711
15390	ANKRD23	31.41601553	-0.203032835	0.227384	-0.892908	0.371906	0.473667
13127	ZNF558	438.0489012	-0.203119284	0.126843	-1.601344	0.109301	0.163206
12468	ZBED1	1911.677309	-0.203393143	0.112246	-1.812034	0.069981	0.110017
10778	KIAA0652	1898.860733	-0.20357939	0.085894	-2.370124	0.017782	0.032339
12693	ANKMY1	177.4289141	-0.20366683	0.117325	-1.735915	0.082579	0.127521
12567	UBR4	4014.699064	-0.203675729	0.11422	-1.783194	0.074555	0.116285
16489	PROK2	4.70358818	-0.203687531	0.354098	-0.57523	0.565136	0.671795
15452	FAM176A	743.0300628	-0.203707536	0.233045	-0.874111	0.382058	0.484643
16296	CYP27B1	51.00811719	-0.203836082	0.32437	-0.628406	0.529738	0.637174

13958	LOC25372[illegible]	14.55187651	-0.203971869	0.153116	-1.332137	0.182815	0.256724
12832	STK11	894.9660849	-0.204153812	0.120427	-1.695244	0.090029	0.13752
13271	TMEM115	1019.587104	-0.204183647	0.131262	-1.555545	0.119816	0.176966
16153	LRRC69	3.134953878	-0.204339441	0.306984	-0.665636	0.505644	0.613578
15707	[illegible]CRNA000[illegible]	8.257308514	-0.20438404	0.254389	-0.803431	0.421726	0.526278
9308	ZC3H4	838.3266774	-0.204603204	0.070632	-2.896768	0.00377	0.00794
13179	MMAB	759.0427941	-0.204627847	0.128988	-1.586412	0.112646	0.167537
12126	ICT1	371.1864393	-0.204726173	0.106678	-1.919097	0.054972	0.088859
15059	AATK	81.37939791	-0.204801419	0.20734	-0.987756	0.323272	0.420776
12303	ZBTB45	215.0978865	-0.204971936	0.109963	-1.864008	0.062321	0.099288
17199	OLFM4	3.56219439	-0.204990124	0.529041	-0.387475	0.698405	0.795943
14513	MYLK	4320.551197	-0.205001035	0.176373	-1.162313	0.245108	0.331039
13507	EFHC1	364.1329617	-0.205001978	0.137983	-1.485706	0.137357	0.199329
15035	UCP3	21.20261774	-0.205003754	0.205827	-0.996002	0.319249	0.416203
14129	ERAP1	2581.067699	-0.205054099	0.16028	-1.279348	0.200774	0.278532
13318	TMEM231	255.1014915	-0.205141136	0.133419	-1.537565	0.124155	0.182727
14345	NDUFA11	1496.834659	-0.205334911	0.168905	-1.215683	0.224106	0.306218
16527	PYGO1	9.145168047	-0.205475503	0.363607	-0.565103	0.572004	0.678396
14166	AVL9	331.1631376	-0.205662123	0.162405	-1.266353	0.205387	0.284183
12542	FZR1	790.7539603	-0.205786362	0.114757	-1.793232	0.072936	0.113986
14450	ARRDC4	1409.097194	-0.206000191	0.174201	-1.182543	0.23699	0.32147
12560	TFE3	1675.568438	-0.206020237	0.115395	-1.785341	0.074206	0.115805
13819	ODF2L	357.3858794	-0.206167724	0.149411	-1.379867	0.167628	0.237765
11484	MAPK3	1594.991116	-0.206271384	0.097017	-2.126128	0.033493	0.057165
16407	B3GNT8	82.21157441	-0.206300478	0.343907	-0.599874	0.548591	0.655386
13025	TBC1D10A	440.919193	-0.206412865	0.126194	-1.635678	0.101907	0.153357
13116	FAM160B2	758.5336702	-0.206426262	0.128498	-1.606449	0.108175	0.161661
12865	CECR5	619.4682888	-0.206503597	0.122305	-1.68843	0.091329	0.139148
14162	ZNF276	479.1627014	-0.206642386	0.163034	-1.267476	0.204985	0.283711
12217	CINP	419.5440455	-0.206791792	0.10934	-1.891282	0.058587	0.093999
12111	GDI1	3304.032417	-0.206959827	0.107529	-1.924692	0.054268	0.08783
12982	KIAA0427	682.4285516	-0.207145511	0.125564	-1.649717	0.099001	0.149478
17866	OR52N2	0.454940261	-0.207162144	0.884042	-0.234335	0.814725	0.893794
15037	ATP5D	1468.319589	-0.207241938	0.208205	-0.995376	0.319554	0.416544
12089	NPLOC4	2544.262065	-0.207510238	0.107423	-1.931716	0.053395	0.086573
10569	HNRNPA1	5959.593167	-0.207602429	0.084823	-2.447491	0.014385	0.026679
11931	EXOSC8	263.937468	-0.207779967	0.104948	-1.979838	0.047722	0.0784
12958	LENG1	160.5651505	-0.20789001	0.125659	-1.654397	0.098047	0.148311
13500	HLA-B	61142.69205	-0.207989374	0.1397	-1.488828	0.136533	0.198235
14387	FBXL13	12.98893448	-0.208095932	0.173161	-1.201751	0.22946	0.312619
16903	ADH1B	439.5461966	-0.20817885	0.448368	-0.464303	0.64243	0.744973
13440	C1orf112	94.25989469	-0.208246	0.138533	-1.50322	0.132782	0.193651
14635	ID3	1790.055924	-0.20829281	0.185686	-1.121749	0.261969	0.350862
11417	SP100	1989.330425	-0.208396293	0.097227	-2.143391	0.032082	0.055079
14770	EHD3	559.1036172	-0.208485659	0.193439	-1.077787	0.281129	0.373081
12822	ARHGAP2[illegible]	770.8639856	-0.208507182	0.122753	-1.698588	0.089397	0.136661
8452	USP39	802.9270719	-0.208551656	0.064623	-3.227201	0.00125	0.002899

17089	DDX43	18.95333559	-0.208744355	0.502017	-0.415812	0.677548	0.777144
11576	JOSD1	1450.863609	-0.209365616	0.100198	-2.089521	0.036661	0.06207
15375	PRR22	42.38419787	-0.209404531	0.233738	-0.895896	0.370308	0.472092
14174	C11orf51	449.7946835	-0.209544929	0.16576	-1.264149	0.206177	0.285098
11231	TXLNA	1859.103196	-0.209597734	0.09494	-2.207684	0.027266	0.047587
15578	JAM2	183.1719659	-0.209616036	0.249271	-0.840915	0.400396	0.503797
11291	MFSD11	433.2247297	-0.209680381	0.096154	-2.180679	0.029207	0.050703
9957	TBC1D25	456.2981864	-0.209818315	0.078624	-2.668617	0.007616	0.014993
15289	SOX17	139.2434866	-0.209872977	0.22722	-0.923656	0.355666	0.455975
12181	ACAT2	277.6476659	-0.210035152	0.110428	-1.902018	0.057169	0.091992
15348	FLJ12825	14.8168333	-0.210095021	0.231968	-0.905708	0.36509	0.466263
16416	THBS4	105.5110964	-0.210138302	0.350865	-0.598914	0.54923	0.655801
13246	CBFB	1187.586441	-0.210417622	0.13454	-1.563976	0.117823	0.174351
11536	FAM127C	506.7288976	-0.210446765	0.100012	-2.104218	0.035359	0.06008
15989	NPAS1	15.78972803	-0.210766491	0.294543	-0.715571	0.474256	0.581393
11992	SYVN1	1691.135241	-0.210988851	0.107497	-1.962741	0.049676	0.081196
13411	UBL5	1824.582966	-0.211156305	0.139842	-1.509959	0.131054	0.191543
12610	TSSC1	476.7940305	-0.211398056	0.119526	-1.768633	0.076955	0.119619
12942	EGLN2	1135.395538	-0.211494746	0.127275	-1.661714	0.09657	0.146258
15830	SPIRE2	126.0535379	-0.211942671	0.276474	-0.766591	0.443325	0.548933
16395	SFRP1	154.3560701	-0.21260435	0.352971	-0.602328	0.546956	0.653912
15262	RYR3	14.3975225	-0.212679494	0.228661	-0.930108	0.352315	0.452479
11977	SSU72	1812.691905	-0.212905693	0.108286	-1.966151	0.049281	0.080644
15253	WDR52	117.0567012	-0.212905811	0.228393	-0.932191	0.351238	0.451361
12835	MRPL27	837.2961721	-0.212981812	0.125758	-1.693585	0.090344	0.137959
11978	UBE2D4	303.5386735	-0.213167674	0.108418	-1.966169	0.049279	0.080644
10558	LSM12	826.1610387	-0.213168485	0.087023	-2.44956	0.014303	0.026554
15932	JAKMIP3	6.968189453	-0.213358086	0.291016	-0.73315	0.463467	0.5702
13629	HSD17B1	27.94852783	-0.213476072	0.14777	-1.444653	0.148555	0.21365
8795	RBM17	1211.595021	-0.213479821	0.069147	-3.087344	0.00202	0.004501
9536	SERPINB6	2976.22986	-0.213615622	0.076187	-2.803832	0.00505	0.01038
13192	WIPF1	2376.313111	-0.213647131	0.135103	-1.581362	0.113795	0.16908
13792	B3GALT4	159.9919122	-0.213678464	0.153873	-1.388665	0.164935	0.234403
14575	LOC440957	112.1306956	-0.213933705	0.18699	-1.144093	0.252585	0.339686
12291	ZNF777	339.9227987	-0.214158775	0.11462	-1.868424	0.061703	0.098401
16477	RAB26	22.23483569	-0.214286359	0.370457	-0.578437	0.562969	0.669707
10196	THADA	901.8854454	-0.214379469	0.083006	-2.582684	0.009804	0.018846
10369	ZCCHC17	577.9507487	-0.214496191	0.085147	-2.519134	0.011764	0.022239
12359	PSMD14	1212.835519	-0.214547407	0.116109	-1.847805	0.064631	0.102502
15979	HES2	32.76280356	-0.214940904	0.29941	-0.71788	0.472831	0.580009
13303	FANCE	62.27123495	-0.214995324	0.139431	-1.541948	0.123086	0.181358
15742	GP5	6.706532397	-0.215072902	0.271826	-0.791217	0.428818	0.533854
16318	EFCAB3	6.425004512	-0.215327091	0.345126	-0.623908	0.532688	0.639859
16362	OC1001315	628.3927185	-0.215329832	0.353117	-0.609797	0.541996	0.649289
16424	LAMA2	233.8469463	-0.215510673	0.360967	-0.597037	0.550482	0.656994
12302	YWHAZ	14739.10358	-0.215611208	0.115659	-1.864205	0.062293	0.099252
17628	ONECUT1	0.502982361	-0.21563161	0.756711	-0.284959	0.775676	0.862493

12916	CRLF3	373.8916644	-0.215833472	0.129373	-1.668307	0.095255	0.144546
15133	ADAMTS1	3138.076728	-0.216138456	0.224277	-0.963713	0.33519	0.434084
14020	BACH1	1599.269045	-0.216231813	0.164678	-1.313056	0.189164	0.264466
9621	SNAPIN	605.8812193	-0.216709256	0.078175	-2.772113	0.005569	0.011347
11209	PEF1	1549.720352	-0.21673404	0.09772	-2.217902	0.026562	0.046448
12247	PIN4	354.2669642	-0.217217327	0.115334	-1.883371	0.05965	0.095468
14916	FLJ10661	10.26799424	-0.217379063	0.210711	-1.031643	0.302239	0.397144
15764	PCDHB16	399.230167	-0.217393887	0.276581	-0.786004	0.431865	0.536948
10125	SNX17	2612.417185	-0.217483173	0.08342	-2.607078	0.009132	0.017678
17390	TTTY15	72.57227806	-0.217781616	0.633134	-0.343974	0.730866	0.823808
12165	TK2	1121.76838	-0.217911529	0.114237	-1.907545	0.05645	0.090956
13783	DISC1	276.889595	-0.217940965	0.15652	-1.392415	0.163797	0.232938
13765	LOC72789(	23.90349236	-0.217995664	0.156043	-1.397022	0.162407	0.231263
13095	KIAA0513	367.0684427	-0.21804861	0.135201	-1.612774	0.106794	0.159852
14403	DDX26B	223.6362476	-0.218076936	0.182155	-1.197206	0.231226	0.314675
14777	SMO	993.3837312	-0.21833295	0.203026	-1.075395	0.282198	0.374322
12974	SFRS17A	831.7456048	-0.218677386	0.132429	-1.651277	0.098682	0.149088
13004	CLPTM1L	3654.851446	-0.218730325	0.133117	-1.643144	0.100353	0.151263
13672	SELO	390.1762516	-0.218869334	0.152977	-1.430732	0.152507	0.218643
13640	ZNF414	144.3574124	-0.218880012	0.151909	-1.440867	0.149622	0.215011
12696	HDGF	5964.638963	-0.218936224	0.126177	-1.735149	0.082714	0.127694
15270	RASIP1	522.2355942	-0.219003744	0.235998	-0.927989	0.353413	0.453651
13189	TRAPPC9	787.1343375	-0.219216878	0.138475	-1.583075	0.113404	0.168537
15042	C7orf58	343.4772714	-0.21921862	0.220517	-0.994113	0.320168	0.417206
14372	C18orf54	133.0224747	-0.219373302	0.181807	-1.206627	0.227576	0.310375
12273	CBX3	3360.022167	-0.219399024	0.11703	-1.874719	0.060831	0.097153
13956	MRAS	810.0422086	-0.219409744	0.164628	-1.332763	0.182609	0.256454
15271	IRS1	604.0958851	-0.219442966	0.236541	-0.927716	0.353555	0.453803
15866	MYH3	39.52544406	-0.219445547	0.291144	-0.753735	0.451008	0.55718
14505	TRIM16L	158.750934	-0.219461158	0.188391	-1.164924	0.24405	0.329791
15052	SIK1	1890.33791	-0.219570223	0.221764	-0.990108	0.322121	0.419472
12644	RAD52	140.7899354	-0.220174207	0.12517	-1.758995	0.078578	0.121814
11328	IQCG	165.2575665	-0.22031422	0.101475	-2.171114	0.029923	0.051775
11158	RNASEH1	235.9510603	-0.220693525	0.098711	-2.23575	0.025368	0.044564
11487	ADPRH	517.0310842	-0.220757354	0.103857	-2.125587	0.033538	0.057227
10214	TGIF2	490.6220532	-0.220797753	0.085685	-2.576856	0.00997	0.019133
15387	KCNJ2	417.628271	-0.22097358	0.247296	-0.89356	0.371557	0.473315
13918	UNC119	399.0792784	-0.220984658	0.164296	-1.345038	0.178613	0.251544
13503	MDH2	3764.489042	-0.221121651	0.148591	-1.488121	0.136719	0.198462
15307	OBSL1	1916.575105	-0.221175509	0.240609	-0.91923	0.357975	0.458395
10433	RNPEP	1616.3491	-0.221251798	0.088515	-2.49959	0.012434	0.02336
11892	SCYL1	1870.315065	-0.22128452	0.111127	-1.991272	0.046451	0.076563
13639	CMTM1	115.6761976	-0.221403648	0.153578	-1.441636	0.149405	0.214714
13971	DENND2D	793.4305545	-0.222082255	0.167219	-1.328092	0.184148	0.258355
13814	PIGL	82.93871181	-0.222171273	0.16077	-1.381921	0.166996	0.236955
16604	BDKRB1	42.17427263	-0.222175048	0.405428	-0.548001	0.583691	0.689047
13081	AHCY	3829.950482	-0.222184368	0.137173	-1.619742	0.105288	0.157766

17418	PSMA8	0.438648095	-0.222274705	0.658783	-0.337402	0.735814	0.828034
11623	WIZ	1009.924852	-0.222424035	0.10739	-2.071183	0.038342	0.064659
13831	ANTXR2	1054.703852	-0.222466592	0.161827	-1.374721	0.169218	0.239812
10648	GPN1	880.54915	-0.222692315	0.092224	-2.41468	0.015749	0.028991
11942	C18orf22	345.5221031	-0.22269878	0.112578	-1.978173	0.047909	0.078636
10088	ZNF384	1265.893521	-0.22281239	0.084999	-2.621358	0.008758	0.017017
11389	NASP	1492.629909	-0.222849838	0.103386	-2.155508	0.031122	0.053563
12017	MCTS1	878.4340331	-0.222930957	0.113951	-1.956379	0.050421	0.082239
12992	NEU1	2742.036545	-0.222957931	0.135377	-1.646946	0.099569	0.15022
16551	GAPDHS	1.680771782	-0.223051844	0.399154	-0.558811	0.576291	0.682463
17215	C19orf34	0.418136621	-0.223224433	0.579831	-0.384982	0.700251	0.797259
15477	LPAR4	6.555228593	-0.223286607	0.257465	-0.867252	0.385804	0.488605
9721	SETDB1	843.8437679	-0.223299132	0.081446	-2.741699	0.006112	0.012324
17180	ROPN1	0.425272442	-0.223375227	0.570107	-0.391813	0.695197	0.793193
17652	C2orf71	0.385899393	-0.223386356	0.800922	-0.278911	0.780313	0.866469
9995	STX6	1077.339247	-0.2235789	0.084242	-2.654001	0.007954	0.015599
17467	HIST1H2A	0.341878619	-0.223783515	0.693194	-0.32283	0.746824	0.838066
17440	C17orf88	0.282867537	-0.223783547	0.67931	-0.329427	0.741833	0.833753
17605	FAM187B	0.243987332	-0.223783645	0.763703	-0.293024	0.769503	0.856747
17690	CCDC155	0.28807768	-0.223783667	0.830114	-0.269582	0.787482	0.872551
17762	OR7E91P	0.305873676	-0.22378369	0.88083	-0.25406	0.799449	0.882221
17645	OR1J1	0.224449129	-0.223783693	0.796889	-0.280822	0.778847	0.865185
17635	CPLX4	0.201608499	-0.223783706	0.791659	-0.282677	0.777424	0.864094
17918	B3GNT6	0.322250013	-0.223783768	0.994935	-0.224923	0.822039	0.899252
17911	EXD1	0.190873887	-0.223783962	0.989949	-0.226056	0.821158	0.898639
17978	LIPF	0.185619361	-0.223784065	1.064662	-0.210193	0.833517	0.908765
18045	SLC35D3	0.170492952	-0.223784201	1.131661	-0.197748	0.843242	0.916041
18185	KIR3DP1	0.314181694	-0.223784206	1.3455	-0.166321	0.867905	0.935485
18044	RPL29P2	0.147330613	-0.223784239	1.132247	-0.197646	0.843322	0.916041
18100	FBXO47	0.148664295	-0.223784383	1.216411	-0.183971	0.854036	0.92486
18245	SNORA61	0.107354566	-0.223784955	1.448434	-0.154501	0.877214	0.942411
18306	GALR3	0.125679146	-0.223785171	1.569061	-0.142624	0.886587	0.949306
18307	LOC28399	0.124135032	-0.223785176	1.571054	-0.142443	0.88673	0.94932
18311	ADAM5P	0.109164171	-0.223785269	1.579918	-0.141644	0.887362	0.949876
18314	C1orf189	0.106918066	-0.223785277	1.58337	-0.141335	0.887605	0.950026
18318	OR52D1	0.105391723	-0.223785283	1.585764	-0.141121	0.887774	0.950026
18316	GKN2	0.104843692	-0.223785286	1.586633	-0.141044	0.887835	0.950026
18390	SNORA5A	0.149764079	-0.223785436	1.709405	-0.130914	0.895843	0.954879
18393	C20orf186	0.126600428	-0.223785549	1.720536	-0.130067	0.896513	0.955394
18413	GKN1	0.08677659	-0.22378587	1.773194	-0.126205	0.89957	0.957629
18412	C2orf57	0.086207165	-0.223785874	1.774532	-0.12611	0.899645	0.957629
18514	LOC40094	0.186031213	-0.223786183	2.070145	-0.108102	0.913915	0.967573
18482	CPSF4L	0.089197681	-0.223786449	1.962896	-0.114008	0.909231	0.964281
18487	XAGE3	0.086532597	-0.223786472	1.970266	-0.113582	0.909569	0.964465
18483	C3orf51	0.08595863	-0.223786477	1.97189	-0.113488	0.909643	0.964465
18485	TAS1R2	0.085910317	-0.223786478	1.972027	-0.11348	0.90965	0.964465
18486	TSHB	0.085905532	-0.223786478	1.972041	-0.11348	0.90965	0.964465

18531	CCDC83	0.108314187	-0.223786974	2.159023	-0.103652	0.917446	0.970344
18561	SNORA76	0.087007841	-0.223787283	2.208043	-0.101351	0.919272	0.97078
18573	SPINK6	0.070418073	-0.223787634	2.263641	-0.098862	0.921248	0.972211
18574	TECTB	0.070262341	-0.223787637	2.264337	-0.098831	0.921272	0.972211
18576	ACPT	0.069386363	-0.223787652	2.268287	-0.098659	0.921409	0.972284
18609	ZPBP2	0.068990888	-0.223787659	2.27009	-0.098581	0.921471	0.972284
18589	GUCY2F	0.067283935	-0.223787689	2.278025	-0.098238	0.921744	0.972284
18601	PRKAG3	0.064982544	-0.223787732	2.289129	-0.097761	0.922122	0.972284
18632	PRR23C	0.102519349	-0.223788081	2.463238	-0.090851	0.927611	0.975853
18639	KLK12	0.090867564	-0.223788385	2.494936	-0.089697	0.928528	0.976451
18641	AADACL2	0.085667502	-0.223788452	2.511343	-0.089111	0.928994	0.976665
18656	FGF22	0.070463549	-0.223788921	2.578056	-0.086805	0.930826	0.977976
18657	LOC64649(	0.068307048	-0.223788975	2.590444	-0.08639	0.931156	0.978271
18658	REG1P	0.067015029	-0.223789008	2.598106	-0.086135	0.931359	0.978431
18727	NLRP5	0.085346089	-0.223790172	2.900648	-0.077152	0.938503	0.981752
18718	GPR151	0.084180496	-0.223790189	2.904211	-0.077057	0.938578	0.981752
18734	SERPINB1(	0.068803593	-0.22379048	2.91407	-0.076797	0.938785	0.981752
18721	OC1001302	0.069709059	-0.22379048	2.91407	-0.076797	0.938785	0.981752
18738	SPANXN2	0.064809932	-0.22379048	2.91407	-0.076797	0.938785	0.981752
18719	KLK9	0.065673168	-0.22379048	2.91407	-0.076797	0.938785	0.981752
18729	OR51I2	0.04716576	-0.223790726	2.91407	-0.076797	0.938785	0.981752
18713	BPIL2	0.049930771	-0.223790726	2.91407	-0.076797	0.938785	0.981752
18720	LDLRAD1	0.050587061	-0.223790726	2.91407	-0.076797	0.938785	0.981752
18723	LOC730811	0.048223874	-0.223790726	2.91407	-0.076797	0.938785	0.981752
18724	LOC73177(	0.047468465	-0.223790726	2.91407	-0.076797	0.938785	0.981752
18730	PAGE4	0.049120584	-0.223790726	2.91407	-0.076797	0.938785	0.981752
18733	PPP1R3A	0.043777559	-0.223790726	2.91407	-0.076797	0.938785	0.981752
18735	SNORA71D	0.047995435	-0.223790726	2.91407	-0.076797	0.938785	0.981752
18736	SNORA75	0.048812734	-0.223790726	2.91407	-0.076797	0.938785	0.981752
18740	SPRR4	0.048875972	-0.223790726	2.91407	-0.076797	0.938785	0.981752
18743	USP17	0.045035842	-0.223790726	2.91407	-0.076797	0.938785	0.981752
18744	WFDC10A	0.046454044	-0.223790726	2.91407	-0.076797	0.938785	0.981752
18745	XAGE5	0.04835227	-0.223790726	2.91407	-0.076797	0.938785	0.981752
18714	CDRT15	0.049358278	-0.223790726	2.91407	-0.076797	0.938785	0.981752
18715	DYTN	0.046071426	-0.223790726	2.91407	-0.076797	0.938785	0.981752
18717	FAM47B	0.045029058	-0.223790726	2.91407	-0.076797	0.938785	0.981752
18728	OR10A5	0.045865141	-0.223790726	2.91407	-0.076797	0.938785	0.981752
18737	SNORA9	0.048631934	-0.223790726	2.91407	-0.076797	0.938785	0.981752
18739	SPRR2B	0.047906644	-0.223790726	2.91407	-0.076797	0.938785	0.981752
10059	MAGOH	416.9267088	-0.224016402	0.085086	-2.632832	0.008468	0.016498
14287	ICAM2	714.4162313	-0.224155128	0.181728	-1.233464	0.217403	0.298265
17206	GH1	0.47859049	-0.224160499	0.58025	-0.386317	0.699262	0.796596
13395	BRI3BP	87.08660265	-0.224253756	0.148251	-1.512661	0.130366	0.190765
14046	ENO1	56158.12661	-0.224414821	0.172335	-1.302198	0.192849	0.2691
9421	TRAPPC3	1006.980944	-0.224570638	0.078782	-2.850532	0.004365	0.009081
10696	HMGN1	2076.290806	-0.224594053	0.093618	-2.399052	0.016438	0.030123
13905	NIT2	1580.632237	-0.224654931	0.166659	-1.347993	0.177661	0.250433

12978	LOC28402[illegible]	126.7338488	-0.225018345	0.136335	-1.650477	0.098845	0.149286
13401	SGK269	1485.194216	-0.225292062	0.149015	-1.511878	0.130565	0.190971
10809	MEN1	697.7181915	-0.225307085	0.095513	-2.358907	0.018329	0.033237
11749	SEC61B	1422.42884	-0.225456461	0.11067	-2.037188	0.041631	0.069454
14589	MET	9072.516523	-0.225521864	0.197886	-1.139656	0.25443	0.341838
14593	DBN1	1520.924249	-0.225539064	0.198101	-1.138503	0.254911	0.34239
17352	ADAMTS2[illegible]	1.589197263	-0.225685973	0.64095	-0.352112	0.724755	0.81869
15976	KIR3DL1	4.871321193	-0.22569592	0.313745	-0.719362	0.471918	0.578998
11970	SNRPE	698.3172149	-0.226157705	0.114881	-1.968628	0.048996	0.080231
11539	RBM10	1107.372398	-0.226170611	0.10753	-2.103322	0.035438	0.060197
15296	C20orf197	8.698257231	-0.226320234	0.245519	-0.921802	0.356632	0.457005
15908	B3GAT1	37.65628949	-0.226638921	0.306004	-0.740641	0.458911	0.565446
14247	SH2D3C	772.4510427	-0.22667	0.182343	-1.243097	0.213832	0.29419
13614	NUAK1	536.0288164	-0.226695489	0.156181	-1.451493	0.146643	0.211131
11251	EDEM1	1494.734586	-0.226714335	0.103048	-2.200077	0.027801	0.048436
10692	COMMD9	944.8563856	-0.226962854	0.094585	-2.39956	0.016415	0.030092
11453	TRIT1	351.3864339	-0.227356174	0.106555	-2.133704	0.032867	0.05625
12759	FNDC3B	4118.438609	-0.228152994	0.132954	-1.716032	0.086156	0.132365
10603	H3F3B	9310.274515	-0.228217722	0.093717	-2.435179	0.014884	0.027513
14250	LAMA5	4120.699598	-0.228716931	0.184129	-1.242157	0.214179	0.294604
13254	MGC70857	415.2668734	-0.228757072	0.14641	-1.562437	0.118185	0.174781
14656	CELSR1	2130.279762	-0.228874635	0.205341	-1.114609	0.265018	0.354412
11232	QSOX2	509.3052191	-0.228898337	0.103715	-2.206984	0.027315	0.047668
12894	MXD4	1961.469794	-0.229191715	0.136891	-1.674261	0.094079	0.143016
11950	DYNLT1	1329.800618	-0.229206706	0.116067	-1.97478	0.048293	0.079213
16840	LOC91149	0.722636055	-0.229231916	0.477507	-0.48006	0.631185	0.734671
10138	STT3A	3373.583171	-0.229395631	0.088143	-2.602541	0.009254	0.01789
12754	NCK2	1403.642951	-0.229466715	0.133649	-1.716939	0.08599	0.132154
16776	DNAJB3	3.434675535	-0.229549734	0.461683	-0.497202	0.619046	0.723291
14517	SAMD9	697.6822086	-0.229567333	0.197673	-1.161348	0.245501	0.331484
14918	DFNB31	215.9044302	-0.22957591	0.222557	-1.031536	0.30229	0.397183
9860	PRMT2	1898.97807	-0.229656869	0.085036	-2.700686	0.00692	0.013756
16631	OR2A25	0.769014192	-0.229679529	0.424645	-0.540874	0.588594	0.693707
13283	PIP4K2A	1246.153431	-0.229849286	0.148362	-1.549248	0.121322	0.179028
11299	WASF2	3960.883456	-0.229967137	0.105569	-2.178366	0.029379	0.050965
15161	ACE	1150.552119	-0.230189934	0.240356	-0.957704	0.338212	0.437258
10735	IDI2	39.92129477	-0.230222406	0.096507	-2.38554	0.017054	0.031139
14351	NPR2	348.9210046	-0.230244841	0.189832	-1.212885	0.225174	0.307562
13822	RPGRIP1L	277.0559236	-0.230255261	0.167015	-1.378647	0.168003	0.238246
12211	MYH9	27521.62051	-0.23034629	0.121712	-1.892553	0.058417	0.093771
15963	ABI3BP	1822.193341	-0.230348174	0.318164	-0.723991	0.469071	0.575973
13704	C4orf21	60.82335615	-0.230364577	0.162568	-1.417032	0.156474	0.223806
15706	CCDC17	10.64400444	-0.230388423	0.286694	-0.803604	0.421626	0.526187
13231	BTG1	6293.568615	-0.230434915	0.146738	-1.570387	0.116325	0.172329
12175	HPS5	696.8953217	-0.230549041	0.121061	-1.904398	0.056858	0.091539
13484	FBXL18	296.7116506	-0.230561612	0.154477	-1.49253	0.13556	0.197057
12947	TTPAL	501.0263609	-0.230652962	0.139021	-1.659126	0.09709	0.146989

12234	PMS2CL	104.1530863	-0.230750452	0.12231	-1.886609	0.059213	0.094869
12458	FIS1	1792.553635	-0.230839555	0.1271	-1.816208	0.069338	0.109095
10871	SIRT2	1345.078427	-0.230848699	0.098983	-2.332216	0.019689	0.035501
13063	IFNGR1	2449.046106	-0.230901944	0.142103	-1.624887	0.104187	0.156332
10352	PGAP2	397.619521	-0.231046102	0.091404	-2.527746	0.01148	0.021736
14790	CFI	3093.749155	-0.231125406	0.215839	-1.070824	0.284249	0.376686
12077	TCTN2	739.3294455	-0.23155286	0.119637	-1.935454	0.052935	0.085913
11593	GPS2	993.7286582	-0.231626117	0.111217	-2.082644	0.037284	0.063038
10375	ABCF2	1227.331484	-0.231637251	0.092123	-2.514446	0.011922	0.022524
14238	PSPH	400.2694158	-0.231667997	0.186038	-1.245271	0.213032	0.293274
12563	SRI	2103.78464	-0.231732731	0.129896	-1.783986	0.074426	0.116116
8930	C1orf174	359.8776917	-0.232003009	0.076286	-3.041236	0.002356	0.005172
14061	AGPAT4	253.7214121	-0.232023996	0.178632	-1.298891	0.193981	0.270409
15370	DCLK3	21.30167536	-0.23238492	0.258418	-0.89926	0.368514	0.469958
16578	BAAT	35.91758	-0.232436975	0.42078	-0.552396	0.580677	0.686564
9369	DPAGT1	782.6216982	-0.232505925	0.080937	-2.872684	0.00407	0.008514
14794	SIGLEC16	9.987110771	-0.232526321	0.217742	-1.067898	0.285566	0.378355
12906	FBXW9	158.7500485	-0.232710751	0.139217	-1.671569	0.094609	0.143688
10845	HMGXB3	1488.528509	-0.233259872	0.099611	-2.341708	0.019196	0.034694
12152	DMWD	478.3156479	-0.233464371	0.122229	-1.91005	0.056127	0.090524
11563	FARSA	1000.819089	-0.23361645	0.111605	-2.093238	0.036328	0.061581
14495	PTRH1	126.590887	-0.23376733	0.199924	-1.169282	0.24229	0.327616
15366	MAMSTR	19.20450892	-0.233816808	0.259678	-0.900409	0.367903	0.4693
13962	POLR3G	44.84501099	-0.233868746	0.175738	-1.330783	0.18326	0.257258
13161	RINL	330.227871	-0.233948875	0.147013	-1.591353	0.11153	0.166105
17025	FKBP6	0.550834233	-0.234206253	0.53772	-0.435554	0.66316	0.763501
15069	ZNF334	163.9976173	-0.234373421	0.237744	-0.985823	0.32422	0.421729
11609	TRIM3	203.4725931	-0.234465106	0.11279	-2.078771	0.037638	0.06355
15586	SLC7A5P2	5.148193436	-0.234654539	0.279619	-0.839193	0.401361	0.504753
14499	PDE1B	355.9406661	-0.234720705	0.200898	-1.168355	0.242663	0.328063
12886	DHODH	216.1107851	-0.234864944	0.13997	-1.677962	0.093355	0.142002
15829	SHANK1	5.488566495	-0.235149307	0.306724	-0.766647	0.443292	0.548926
15301	SUSD2	547.7941133	-0.235654055	0.255988	-0.920568	0.357276	0.45768
11342	LCMT1	669.7632739	-0.235696744	0.108705	-2.168223	0.030142	0.05209
11488	ATG12	602.0479121	-0.235820748	0.110989	-2.124719	0.03361	0.057346
11499	UBE2MP1	85.64833742	-0.236073006	0.111456	-2.118078	0.034168	0.058243
16917	GUCA2B	27.25689823	-0.236140025	0.513454	-0.459905	0.645584	0.748011
10967	TMEM18	709.6757033	-0.236151466	0.102766	-2.297953	0.021564	0.038542
10734	CCDC123	464.3970334	-0.236232441	0.099018	-2.385761	0.017044	0.031123
7480	TRPC4AP	2021.717698	-0.236317446	0.065228	-3.622927	0.000291	0.000763
14905	SP6	47.66449092	-0.236383386	0.228538	-1.034329	0.300982	0.39581
10458	DUSP28	85.47202183	-0.236504815	0.09499	-2.489774	0.012782	0.023958
14416	HEXDC	338.2629927	-0.236701451	0.198372	-1.193217	0.232784	0.316488
10968	ZNF71	168.1454301	-0.237252401	0.103264	-2.297524	0.021589	0.038582
11911	SRPRB	1446.538844	-0.237331905	0.11958	-1.984707	0.047177	0.077636
14932	RIMS3	89.24430547	-0.237374553	0.231427	-1.025697	0.305034	0.400425
11644	HMGN2	4299.179125	-0.237415072	0.115089	-2.062876	0.039124	0.06586

15725	MAOA	2994.646643	-0.237503123	0.298128	-0.796648	0.425655	0.530574
10240	C1orf128	779.6854331	-0.237532106	0.092429	-2.569881	0.010173	0.019473
13717	NDOR1	367.0283942	-0.237536605	0.168129	-1.412825	0.157707	0.225357
10357	OTUD5	1472.983016	-0.237665928	0.094161	-2.524048	0.011601	0.021956
14715	EBF1	539.8465163	-0.237966951	0.217753	-1.092829	0.274469	0.365604
13719	STK17A	856.4868477	-0.237973319	0.168536	-1.412006	0.157948	0.225668
13558	ENAH	1381.181259	-0.238074162	0.16214	-1.468323	0.142016	0.205315
11534	C16orf53	812.8262055	-0.238154154	0.113118	-2.105364	0.03526	0.059921
14404	RAMP2	807.7199828	-0.238219099	0.199034	-1.196878	0.231354	0.314827
16093	SELE	102.5914699	-0.238275949	0.348726	-0.683275	0.494433	0.602211
11168	C12orf73	154.2589237	-0.238366037	0.10678	-2.232302	0.025595	0.04492
11947	ESYT2	4528.962879	-0.23842264	0.120679	-1.97568	0.048191	0.079065
10528	SMN2	891.3220835	-0.238521028	0.096935	-2.460629	0.013869	0.025822
10503	PIPSL	125.8231662	-0.238581304	0.096585	-2.470179	0.013505	0.025203
10498	CNOT3	796.4149639	-0.238698916	0.09657	-2.471765	0.013445	0.025103
15317	C20orf107	2.22175048	-0.238937081	0.260709	-0.916489	0.359411	0.459934
14820	TRIB1	1607.621605	-0.239047053	0.22578	-1.058763	0.289708	0.383169
17490	GPR87	1.980314691	-0.239171628	0.75522	-0.316691	0.751478	0.842179
12375	MGRN1	2735.647413	-0.239225688	0.129812	-1.84286	0.06535	0.103502
11111	ANP32B	2540.278208	-0.239249799	0.106365	-2.249323	0.024492	0.043208
12452	HDDC3	328.3980635	-0.239494532	0.131629	-1.819472	0.068839	0.108353
14840	ARHGAP3[illegible]	191.472605	-0.239800722	0.227418	-1.054447	0.291678	0.385255
11852	IL17RA	316.8557614	-0.239874813	0.119601	-2.005628	0.044896	0.074249
16440	CA8	59.57636518	-0.240255221	0.406719	-0.590716	0.554711	0.661368
11906	GNPTG	1638.98158	-0.240560874	0.121161	-1.985468	0.047092	0.077523
11464	C20orf3	2864.41766	-0.240766142	0.113028	-2.13015	0.033159	0.056695
13343	WDR67	131.1846457	-0.240766535	0.157266	-1.530946	0.125783	0.184776
15803	PCDHB11	109.3581541	-0.240938446	0.310986	-0.774756	0.438484	0.543866
9622	ARF1	10211.99115	-0.240946562	0.086925	-2.77188	0.005573	0.011354
13264	44080	896.5180707	-0.241162151	0.154753	-1.558365	0.119147	0.17607
13515	TMEM70	669.1289698	-0.241218839	0.162698	-1.482615	0.138177	0.2004
15977	LHFPL1	1.712237086	-0.241371685	0.335591	-0.719243	0.471991	0.579051
15190	KCNK13	47.42043981	-0.241624607	0.254065	-0.951036	0.341586	0.44075
11189	AKAP8	915.7931727	-0.241719957	0.108633	-2.225113	0.026074	0.045676
11470	ZRSR2	262.8640969	-0.241721867	0.113553	-2.128715	0.033278	0.056868
12892	TIMM8B	621.8439393	-0.241813936	0.144321	-1.675526	0.093831	0.142661
10390	PSMD9	925.0759346	-0.24205573	0.09643	-2.510164	0.012067	0.022766
14136	CD302	996.0918729	-0.242143313	0.189647	-1.276813	0.201668	0.279634
13596	NMI	565.3620544	-0.242155144	0.166203	-1.456986	0.14512	0.209216
13432	TRIP10	1377.307712	-0.242277921	0.161016	-1.504686	0.132405	0.193215
12266	PPA1	1966.007495	-0.242293911	0.129002	-1.87822	0.060351	0.096441
12045	H3F3C	255.3405837	-0.24233998	0.124561	-1.945554	0.051708	0.084139
10378	RPA2	910.4680193	-0.242468462	0.096492	-2.512841	0.011976	0.02262
10581	GTF2F1	1796.158601	-0.242550154	0.099335	-2.441751	0.014616	0.027076
15062	TRPC4	64.03030215	-0.242687771	0.245831	-0.987212	0.323539	0.421039
16036	NGEF	215.1421859	-0.242764729	0.346562	-0.700494	0.483619	0.591133
15879	ACSBG2	3.317157027	-0.242839117	0.324177	-0.749094	0.453801	0.560171

13637	SESN2	622.6345256	-0.243171718	0.168554	-1.442692	0.149107	0.214318
14668	SGCE	774.2659584	-0.243291264	0.218947	-1.111188	0.266487	0.35613
16754	TULP2	0.742823906	-0.243441117	0.483112	-0.503902	0.61433	0.718723
11607	IFT172	576.4806152	-0.24364344	0.117187	-2.079101	0.037608	0.06351
12131	PABPN1	2189.466205	-0.243822097	0.127136	-1.917807	0.055135	0.089087
9377	RER1	2686.683566	-0.243822098	0.084949	-2.870233	0.004102	0.008574
12430	C9orf30	388.7007334	-0.244128227	0.133626	-1.826958	0.067706	0.106767
11840	HPS1	1452.52281	-0.244203879	0.121582	-2.008558	0.044584	0.073808
8979	UBE2L3	1934.806503	-0.244244518	0.080858	-3.020645	0.002522	0.005506
13398	ZNF286B	20.31949448	-0.244503427	0.161666	-1.512401	0.130432	0.190819
14265	NHEDC2	127.327934	-0.244620907	0.197432	-1.239012	0.215341	0.295892
14335	COX4I1	7404.799985	-0.244662901	0.200591	-1.219713	0.222574	0.304337
15696	TAS2R10	1.668989185	-0.244678497	0.302842	-0.80794	0.419125	0.523399
14241	TTN	173.1670919	-0.244713192	0.196701	-1.24409	0.213467	0.293811
11187	ALG1	678.7865007	-0.244832523	0.110017	-2.225412	0.026054	0.045649
11801	BTBD12	203.1764516	-0.244901568	0.121209	-2.020494	0.043332	0.071973
11422	DVL2	538.3885976	-0.244941687	0.114384	-2.141395	0.032242	0.05533
7828	CDC123	793.5882206	-0.244945971	0.070171	-3.490684	0.000482	0.001206
9689	44082	1939.279635	-0.244953229	0.089092	-2.749439	0.00597	0.012077
12117	BOD1	1005.518071	-0.244961106	0.127376	-1.923136	0.054463	0.088102
16156	SYT13	2696.001029	-0.24499978	0.368704	-0.66449	0.506377	0.614388
12837	DEAF1	747.1810838	-0.245044573	0.144718	-1.69325	0.090408	0.138045
10266	DIABLO	1041.678455	-0.245241079	0.095843	-2.558771	0.010504	0.020056
17533	CPB2	2.004768563	-0.245288312	0.798236	-0.307288	0.758624	0.848103
17188	TYRP1	3.514448882	-0.245633158	0.630076	-0.389847	0.69665	0.794452
15393	CCL14	497.8237703	-0.245686929	0.275485	-0.891835	0.372482	0.474307
15249	HKB-CPT1	454.6636302	-0.245920618	0.263533	-0.933167	0.350734	0.450832
14693	KIAA1683	84.04044359	-0.246083306	0.223225	-1.102401	0.270288	0.360573
14544	NECAB3	412.4686164	-0.246104062	0.213498	-1.152725	0.249023	0.33561
14831	UNC45B	5.221541595	-0.24619375	0.23288	-1.057168	0.290435	0.383852
15320	C6orf52	3.356613738	-0.246316865	0.269124	-0.915255	0.360058	0.460672
14328	FAM105A	419.302794	-0.246533321	0.20169	-1.222335	0.221581	0.303127
12103	CSNK1D	3123.138141	-0.246561304	0.127867	-1.92826	0.053823	0.087167
14620	AGAP5	38.16211286	-0.246637265	0.218901	-1.126707	0.259866	0.348402
11722	SF4	512.0817578	-0.246738499	0.120613	-2.045711	0.040785	0.068199
14381	PERP	2911.959216	-0.246748806	0.205151	-1.202767	0.229066	0.312213
12936	PRKX	702.2368033	-0.247059933	0.148517	-1.663515	0.096209	0.145779
12385	TXNL4A	857.1853204	-0.247297784	0.134348	-1.840723	0.065662	0.10392
10794	CDK7	542.6221973	-0.247623413	0.10476	-2.363722	0.018092	0.032854
9568	SETD8	564.4815809	-0.247811611	0.088724	-2.793046	0.005221	0.010696
16429	KLC3	33.60551881	-0.247839664	0.415719	-0.596172	0.55106	0.657456
9818	CDIPT	2391.693855	-0.24785398	0.091414	-2.711343	0.006701	0.013378
15689	CITED1	4.144637708	-0.247906952	0.305842	-0.810573	0.417611	0.521741
8986	IK	2140.888531	-0.248103655	0.082175	-3.0192	0.002534	0.005528
14157	ARL10	77.14303539	-0.248355703	0.195763	-1.268653	0.204565	0.283229
10555	C7orf30	462.1540539	-0.248361313	0.101329	-2.451048	0.014244	0.026452
9567	RUVBL1	730.9441982	-0.248657136	0.089011	-2.793544	0.005213	0.010681

12652	IDH3G	1135.972869	-0.248677181	0.141582	-1.756414	0.079018	0.122417
12749	FLJ35220	289.9421714	-0.248741665	0.144734	-1.718617	0.085684	0.131735
11166	MED30	125.4502673	-0.248798014	0.111429	-2.2328	0.025562	0.044872
14500	ERO1L	2002.562089	-0.24885752	0.213112	-1.167731	0.242915	0.328371
11805	CDR2	973.7573104	-0.249026449	0.123313	-2.019459	0.04344	0.072127
14997	LPAL2	5.93602637	-0.249191471	0.247265	-1.007792	0.313554	0.409814
9981	MARK4	758.6224291	-0.249256563	0.093758	-2.658513	0.007849	0.015413
16048	PADI4	4.748004059	-0.249344527	0.357277	-0.697902	0.485238	0.592669
14160	SLC4A7	863.2118762	-0.249462345	0.196777	-1.267743	0.20489	0.283619
11026	DRAM2	1153.949074	-0.249507597	0.109379	-2.281137	0.02254	0.04007
13073	TECR	1601.68319	-0.249634063	0.153922	-1.621818	0.104842	0.157195
11997	DALRD3	539.4374736	-0.249717889	0.127302	-1.961622	0.049807	0.081375
13526	729991-MI	41.79333737	-0.250010418	0.169042	-1.478983	0.139145	0.201625
15545	NAP1L3	63.88952977	-0.250011458	0.295142	-0.847089	0.396946	0.500517
14940	TSSK3	26.77349643	-0.250067772	0.244109	-1.02441	0.305642	0.401055
12296	DDRGK1	1106.090225	-0.250080713	0.133963	-1.866793	0.06193	0.098723
15655	GPR83	6.365214286	-0.250170333	0.304803	-0.820761	0.411783	0.515576
15330	LOC90586	3.874749734	-0.250801038	0.275456	-0.910495	0.362562	0.463573
10474	UBL4A	1076.310617	-0.250928053	0.101034	-2.483612	0.013006	0.024339
15589	COL11A2	20.75521101	-0.25106747	0.299501	-0.838286	0.40187	0.505296
10749	DNPEP	1458.7981	-0.251196637	0.105499	-2.381041	0.017264	0.031481
14812	TMEM176I	10751.62806	-0.251279729	0.236831	-1.061009	0.288686	0.382023
14681	SLC6A1	145.6761813	-0.251343451	0.226898	-1.107736	0.267976	0.357782
13288	ATP5I	1619.661458	-0.251381357	0.162517	-1.546799	0.121912	0.179831
10282	PHF20	988.63259	-0.251400339	0.098507	-2.552095	0.010708	0.020413
13624	ITGB7	425.0715004	-0.251527729	0.173758	-1.447576	0.147736	0.212549
11793	RILPL2	556.1660886	-0.251597936	0.124309	-2.023976	0.042973	0.071424
12723	C19orf63	3223.196665	-0.251783747	0.145966	-1.724944	0.084538	0.130238
13909	LOC21934	41.42320837	-0.251879142	0.186915	-1.34756	0.1778	0.250561
13160	MLKL	431.4620949	-0.251941444	0.15829	-1.591647	0.111464	0.166019
11662	PIK3R2	1368.461568	-0.251984256	0.1224	-2.058702	0.039523	0.066417
13349	SACS	646.5297679	-0.252144053	0.164909	-1.528986	0.126268	0.185406
9562	C19orf62	1082.625027	-0.252219222	0.09021	-2.795916	0.005175	0.010609
14650	SEC1	4.632809629	-0.252350999	0.225892	-1.117134	0.263937	0.353135
11765	TTLL1	149.7796415	-0.25238403	0.12423	-2.031594	0.042195	0.070298
10083	CCT3	4465.48378	-0.252403213	0.096187	-2.62409	0.008688	0.016889
9629	PSMA1	2429.028686	-0.252418952	0.09115	-2.769282	0.005618	0.011437
16374	DSCAM	3.914732923	-0.252674396	0.41607	-0.607288	0.54366	0.650805
10221	ZNF565	59.55830482	-0.252735973	0.098194	-2.573838	0.010058	0.019288
15150	CITED4	452.190135	-0.252776065	0.263482	-0.959368	0.337374	0.436492
11404	URM1	1112.904889	-0.252889217	0.117739	-2.147874	0.031724	0.054526
13994	GLYCTK	290.8162763	-0.252951414	0.191676	-1.319681	0.186942	0.261825
11507	HIRIP3	484.7380137	-0.253053869	0.119705	-2.113976	0.034517	0.058797
13749	BGLAP	34.49678617	-0.253066799	0.180452	-1.402402	0.160795	0.229217
16931	KFZP434K0	0.548013142	-0.253080545	0.556273	-0.454957	0.64914	0.75142
12127	RRP7A	662.0850742	-0.253167282	0.131932	-1.91893	0.054993	0.088886
15852	PRR15	32.04636451	-0.253557653	0.334978	-0.756939	0.449086	0.555295

11218	UBL7	739.9127914	-0.253570403	0.11448	-2.214969	0.026762	0.046761
16000	TSG1	14.09437542	-0.253591917	0.355643	-0.713051	0.475814	0.582902
12733	PPM1F	1378.760143	-0.253723584	0.14732	-1.722259	0.085023	0.130883
16022	TBX4	2.550762809	-0.253973092	0.360375	-0.704747	0.480968	0.588407
15783	SMTNL1	7.254025063	-0.254148358	0.325459	-0.780892	0.434866	0.540062
9347	RNPS1	1878.274838	-0.254171567	0.088278	-2.879214	0.003987	0.00836
14956	SBK1	50.90140673	-0.254242973	0.24894	-1.021302	0.307111	0.402493
13860	LRFN3	140.5710661	-0.254486878	0.186386	-1.365378	0.172134	0.243435
15036	XKR6	8.435869064	-0.254605276	0.255712	-0.995671	0.31941	0.416384
15919	POU2F3	6.517254397	-0.254953107	0.346368	-0.736076	0.461685	0.568463
9253	B4GALT3	818.096923	-0.255052565	0.08743	-2.917219	0.003532	0.007481
11396	MKI67IP	773.8934997	-0.255100258	0.118606	-2.150829	0.03149	0.054162
14907	MYOM1	81.53486141	-0.255134265	0.246719	-1.034108	0.301086	0.395893
13253	DOHH	274.8831301	-0.255231908	0.163346	-1.562526	0.118164	0.174763
15521	LOC16847	4.536827945	-0.255437465	0.299609	-0.852569	0.393898	0.497442
12655	LBH	2665.178126	-0.255569509	0.145587	-1.755437	0.079185	0.122647
8624	PTBP1	4506.516875	-0.255788939	0.080801	-3.165657	0.001547	0.003517
14699	MCTP1	150.3189928	-0.255850496	0.232864	-1.098714	0.271893	0.362567
11897	ADAM17	670.3000979	-0.255863033	0.128625	-1.989219	0.046677	0.076903
12110	IQCC	76.02800463	-0.25591343	0.132951	-1.924874	0.054245	0.0878
15352	PCDHGB6	85.75798978	-0.256340832	0.283507	-0.904177	0.365901	0.467173
8316	CUL1	1478.920942	-0.256356648	0.078064	-3.283944	0.001024	0.002413
8270	SPATS2	633.3917625	-0.256392312	0.077596	-3.304184	0.000953	0.002258
14818	UPP1	825.8091449	-0.256413276	0.242101	-1.059119	0.289546	0.383006
15726	GPR156	2.472319693	-0.256828097	0.322622	-0.796064	0.425995	0.530963
11621	C3orf62	105.8680052	-0.257279832	0.124194	-2.071588	0.038304	0.064607
9012	LGTN	1165.25558	-0.257405682	0.085578	-3.007835	0.002631	0.005723
14007	ZNF837	42.78316365	-0.257476958	0.195615	-1.316241	0.188093	0.263212
12100	EIF5B	3193.625973	-0.257617051	0.133581	-1.928548	0.053787	0.08713
9855	ENY2	573.8711598	-0.257660524	0.095385	-2.701278	0.006907	0.013737
15322	GDPD3	58.8152173	-0.257783704	0.282025	-0.914046	0.360693	0.461424
17115	ASB15	0.627068719	-0.257874328	0.633014	-0.407375	0.683732	0.783046
14022	FAT1	6951.391345	-0.257889393	0.196449	-1.312752	0.189267	0.264552
15031	NFASC	530.3852599	-0.257934947	0.258851	-0.996461	0.319026	0.416022
10047	HTRA2	684.5562591	-0.257943864	0.097871	-2.635544	0.0084	0.016388
16056	FGF20	1.150360058	-0.258061529	0.370444	-0.696628	0.486036	0.593332
12072	PTPN1	2144.384518	-0.258123717	0.133262	-1.936959	0.05275	0.085649
12731	CHMP6	586.6109624	-0.258186304	0.149877	-1.72266	0.08495	0.130791
12408	TSSC4	492.373539	-0.258257063	0.140833	-1.833781	0.066686	0.105345
13571	CCDC34	260.9459523	-0.258263911	0.176466	-1.463531	0.143322	0.207004
11734	AP1B1	2965.868667	-0.258441153	0.126553	-2.04216	0.041136	0.068709
12758	FAM86D	121.9898436	-0.258526216	0.150656	-1.716003	0.086161	0.132365
11677	ZNF335	466.3556702	-0.258565561	0.125714	-2.056781	0.039707	0.066653
12184	AKR1A1	3006.252096	-0.258580941	0.136002	-1.901297	0.057263	0.092122
14292	OC1001287	9.05326569	-0.258616954	0.209936	-1.231887	0.217991	0.298971
10636	CDK9	1033.808301	-0.258732999	0.106893	-2.420476	0.0155	0.028565
12401	MED27	223.4360513	-0.258733082	0.140911	-1.836145	0.066336	0.104851

14435	NUP210	930.3161707	-0.258751058	0.217792	-1.188065	0.234808	0.318841
9636	BCL7B	1269.945047	-0.258891073	0.093571	-2.766781	0.005661	0.011516
13802	TPBG	331.0606327	-0.258942806	0.18672	-1.386795	0.165504	0.235063
13755	MRPL54	731.6675691	-0.259071642	0.184789	-1.401989	0.160918	0.22931
11215	C1orf198	1105.9421	-0.259891996	0.117311	-2.215411	0.026732	0.046716
13577	KIF3C	329.9277726	-0.259916298	0.177803	-1.461821	0.14379	0.207589
12676	TES	2113.812832	-0.259960463	0.148911	-1.745745	0.080855	0.125027
11330	BANP	183.3256748	-0.259966464	0.119769	-2.170569	0.029964	0.051834
12456	GSTK1	3888.462624	-0.260118656	0.143095	-1.817807	0.069094	0.108718
13889	PAG1	1154.833605	-0.260129646	0.192464	-1.351576	0.176511	0.249103
13219	SILV	45.00491825	-0.260154253	0.165229	-1.574508	0.11537	0.171069
14638	LRRC17	153.0902795	-0.260187898	0.232049	-1.121262	0.262176	0.351043
13306	CCT6P1	73.10554595	-0.260196736	0.168829	-1.541184	0.123272	0.181591
10365	PSMB7	2012.991334	-0.260230299	0.103193	-2.521791	0.011676	0.02208
14163	AMDHD2	403.7875171	-0.260377271	0.205559	-1.266676	0.205271	0.284047
10920	HEXB	3343.363014	-0.260519979	0.112693	-2.311769	0.02079	0.037318
11900	POU6F1	309.5595346	-0.260522223	0.131047	-1.987998	0.046812	0.077106
11084	C18orf45	102.324261	-0.260564991	0.115241	-2.26104	0.023757	0.042012
12258	RECQL5	528.0735095	-0.260607368	0.138608	-1.88017	0.060085	0.096078
15944	MAPK15	96.43082786	-0.260930361	0.358755	-0.727322	0.467029	0.574122
12530	ZFYVE19	535.2583467	-0.260973156	0.14535	-1.795482	0.072577	0.113534
11432	ZNF600	194.1119813	-0.261287099	0.122133	-2.139366	0.032406	0.055563
9942	EIF3D	4106.972741	-0.261374786	0.097771	-2.673329	0.00751	0.014807
15015	HOXB5	86.37872324	-0.26149454	0.260241	-1.004816	0.314986	0.411191
14644	[illegible]	125.2336805	-0.261503356	0.233633	-1.119291	0.263016	0.352047
8577	C7orf42	4189.343758	-0.261867227	0.082337	-3.180438	0.001471	0.00336
16353	LRP1B	3.393607266	-0.261880029	0.427358	-0.612788	0.540017	0.647274
13198	FDX1L	217.0366792	-0.262429288	0.166213	-1.578872	0.114365	0.169837
8797	GORASP2	2243.584087	-0.262630891	0.085073	-3.087128	0.002021	0.004503
14615	ABCC9	685.0688072	-0.262814764	0.233081	-1.127567	0.259503	0.348034
10419	PSMA2	2266.348674	-0.262983018	0.105031	-2.503868	0.012284	0.02311
10203	PAPD7	643.3738688	-0.263110519	0.10194	-2.581039	0.00985	0.018924
9658	FAM188B	187.2932773	-0.263138385	0.095279	-2.761754	0.005749	0.011668
12185	ZNF532	1424.640346	-0.263172109	0.138435	-1.901057	0.057295	0.092165
12572	MAFK	1095.395144	-0.263238271	0.147834	-1.780636	0.074972	0.116889
10721	PPP2R1A	6365.076585	-0.263263244	0.110178	-2.389435	0.016874	0.030851
7375	KCMF1	1204.315577	-0.263301309	0.071795	-3.667403	0.000245	0.000651
10972	RBM3	4692.566747	-0.263387183	0.11471	-2.296119	0.021669	0.038711
10479	TBCA	1804.437371	-0.26368849	0.106349	-2.479463	0.013158	0.024612
9832	C7orf26	489.5758466	-0.2639862	0.097453	-2.708847	0.006752	0.01346
10568	HAUS4	866.2390816	-0.264098746	0.107906	-2.447492	0.014385	0.026679
11131	AKAP13	3464.849975	-0.264186138	0.117838	-2.241941	0.024965	0.043958
13197	SCRIB	961.1546087	-0.26435405	0.167369	-1.57947	0.114228	0.169659
14749	C6orf114	6.32788561	-0.26474673	0.244215	-1.084073	0.278332	0.369896
13077	SNRNP25	541.2963048	-0.264934666	0.163468	-1.620714	0.105079	0.157505
10137	CHCHD8	959.4411596	-0.265256809	0.10192	-2.602598	0.009252	0.01789
13957	WDR90	491.8377254	-0.265279142	0.199038	-1.332808	0.182595	0.256454

12641	MAP3K10	213.6982833	-0.265338186	0.150769	-1.759903	0.078424	0.121604
10726	ZNF101	101.081681	-0.265517001	0.111194	-2.387868	0.016946	0.030968
11855	SSH2	904.1418921	-0.265607091	0.132516	-2.004335	0.045034	0.074459
12688	PTDSS2	803.9581207	-0.265648333	0.152641	-1.740343	0.081799	0.126367
13118	POLR2J3	824.1215221	-0.266094877	0.165724	-1.605653	0.10835	0.161898
7829	C1orf144	3138.667871	-0.266175303	0.07627	-3.48991	0.000483	0.00121
11442	ZNF707	136.837952	-0.266218662	0.12462	-2.136246	0.032659	0.055948
11302	RCCD1	231.1179721	-0.266290602	0.122271	-2.177881	0.029415	0.051014
12467	XPOT	1772.557873	-0.266310913	0.146937	-1.812414	0.069922	0.109934
14920	C15orf28	5.602624188	-0.266476077	0.258646	-1.030272	0.302883	0.397909
12606	TAF1C	781.2799464	-0.266518079	0.150536	-1.770456	0.076651	0.119185
15422	PCDH9	20.13934362	-0.266673253	0.302014	-0.882983	0.377246	0.479449
10115	DNAJC9	443.1979031	-0.266749046	0.102205	-2.609945	0.009056	0.017548
13259	SDSL	358.2103756	-0.266749693	0.170889	-1.560957	0.118534	0.175231
11899	UBXN6	2683.498405	-0.26697022	0.134285	-1.98808	0.046803	0.077098
12007	CASP8	862.1575538	-0.26717641	0.136377	-1.959095	0.050102	0.081789
8730	MTA2	1396.876067	-0.267486294	0.085846	-3.115868	0.001834	0.004117
10172	PLCG1	1630.707691	-0.267749292	0.103282	-2.592422	0.00953	0.018364
9789	SF3B14	1121.684317	-0.267770917	0.098469	-2.719335	0.006541	0.013098
11151	ATF4	5644.075013	-0.267840319	0.119732	-2.237002	0.025286	0.044445
12167	SPATA20	1817.099185	-0.268092387	0.140558	-1.907346	0.056476	0.090982
17214	SHOX	0.547061902	-0.268097531	0.69619	-0.385092	0.700169	0.797259
13446	EPHB4	1130.380199	-0.268106276	0.178477	-1.502191	0.133048	0.193937
15440	DUOX1	31.65207152	-0.268304792	0.306264	-0.876058	0.380998	0.483675
11758	C10orf25	118.7174801	-0.268354806	0.13187	-2.03499	0.041852	0.069763
10011	MED19	216.1381285	-0.268764364	0.101447	-2.649315	0.008066	0.01579
10293	MLF2	3591.789912	-0.268875656	0.105523	-2.548033	0.010833	0.02063
11799	IFRD2	623.9590091	-0.268885517	0.133056	-2.020847	0.043296	0.071924
9579	U2AF2	2313.484447	-0.268899261	0.096443	-2.788174	0.005301	0.010846
11073	DPP3	762.5032146	-0.268924256	0.118719	-2.265211	0.0235	0.041598
13934	CRNA0021	679.8911319	-0.26901785	0.200703	-1.340376	0.180123	0.25338
11143	ENTPD1	2057.249652	-0.269133511	0.120167	-2.239663	0.025113	0.044174
8544	UBAC2	1761.916361	-0.269222274	0.084352	-3.191666	0.001415	0.003245
11987	DYRK1B	515.894102	-0.269787069	0.137357	-1.964132	0.049515	0.080966
12720	MGLL	7423.931612	-0.269830259	0.156251	-1.726908	0.084184	0.129724
13616	PPP1R3B	2871.101295	-0.270234419	0.186321	-1.450367	0.146956	0.211552
11467	DEDD2	579.1527275	-0.270351051	0.126954	-2.129519	0.033211	0.056769
11974	C19orf39	58.58899329	-0.270427651	0.137427	-1.967797	0.049091	0.080361
15468	CLDN1	1617.263947	-0.270503229	0.31075	-0.870485	0.384035	0.486649
14283	ADORA2A	319.1525183	-0.270591061	0.219231	-1.234273	0.217101	0.297914
15973	BCHE	38.89355932	-0.270659387	0.375274	-0.721231	0.470768	0.57769
12984	OGFRL1	475.5124699	-0.270793929	0.164205	-1.649124	0.099122	0.149638
12191	NEIL2	338.3016508	-0.271056899	0.142718	-1.899249	0.057532	0.092501
14297	MTMR7	74.21177011	-0.271187379	0.220462	-1.230085	0.218665	0.299775
10254	SRRT	1407.300763	-0.271238779	0.105772	-2.564378	0.010336	0.019758
12971	LRCH4	698.9661513	-0.271451952	0.164275	-1.652422	0.098449	0.14877
13961	IQCD	36.24342523	-0.271492248	0.203927	-1.331319	0.183084	0.257047

12697	PTK2B	736.0759874	-0.271870759	0.156686	-1.735134	0.082717	0.127694
10776	TMEM63B	1025.433741	-0.27192615	0.114607	-2.37269	0.017659	0.032121
15664	FLJ40292	1.538706511	-0.272391	0.332947	-0.818122	0.413288	0.517164
13886	SEL1L3	3332.996487	-0.272979746	0.201767	-1.352946	0.176073	0.248538
16294	WDR64	0.750028456	-0.273048958	0.434165	-0.628906	0.529411	0.636859
11150	CENPJ	115.0553398	-0.273228611	0.12213	-2.237195	0.025274	0.044429
10826	STX2	576.5771827	-0.273394428	0.116212	-2.352557	0.018645	0.033757
14079	PHOSPHO1	12.24033465	-0.273489411	0.211325	-1.294164	0.195609	0.27233
8135	DNAJB6	1511.904762	-0.27359019	0.081467	-3.358276	0.000784	0.00189
9780	DENND5A	2201.038086	-0.273614958	0.100511	-2.722226	0.006484	0.012996
14489	PCDHB19P	10.44557877	-0.273668558	0.233545	-1.171804	0.241276	0.326403
15266	RSPH1	63.54393272	-0.273680134	0.294511	-0.92927	0.352749	0.452917
15186	ADAMTSL3	469.7153182	-0.273783799	0.287576	-0.952041	0.341076	0.440237
15382	GPR82	23.81290212	-0.273983885	0.306397	-0.894213	0.371208	0.473039
12717	CCDC14	726.2594052	-0.274157315	0.158709	-1.727417	0.084093	0.129606
12651	ZNF784	146.6734298	-0.274268645	0.156137	-1.756588	0.078988	0.122381
15838	TCP11	2.358807485	-0.274302138	0.359796	-0.762382	0.445832	0.551759
14453	AK1	467.1148255	-0.274558816	0.232404	-1.181385	0.23745	0.322027
13735	ARRDC2	1643.004231	-0.274566759	0.195337	-1.405606	0.159841	0.228107
14374	BAG2	161.5817504	-0.274653674	0.227783	-1.205771	0.227906	0.310782
11297	PPIAL4C	34.64491539	-0.274934628	0.126199	-2.178588	0.029362	0.050945
13137	PAQR8	338.9300862	-0.274979188	0.172129	-1.59752	0.11015	0.164349
11203	PDK3	228.5336004	-0.27505211	0.123851	-2.220826	0.026363	0.046125
12856	ULK1	1409.246317	-0.275133247	0.16281	-1.689908	0.091046	0.138813
12855	MAMLD1	137.6899806	-0.275142037	0.16281	-1.689963	0.091035	0.138808
10099	ELL	530.4718131	-0.275499734	0.105292	-2.616529	0.008883	0.017239
12372	IFT27	431.477504	-0.275872048	0.149668	-1.843224	0.065296	0.103449
12144	ADARB1	621.5765592	-0.275881643	0.14416	-1.913716	0.055656	0.089832
17287	PENK	0.965835945	-0.275930077	0.742819	-0.371463	0.710292	0.805397
12561	PHPT1	1394.0784	-0.276129533	0.154784	-1.78397	0.074429	0.116116
12872	TRPC1	323.8595107	-0.276267925	0.163939	-1.685187	0.091953	0.140029
11895	MVD	377.4508368	-0.276274335	0.138839	-1.989891	0.046603	0.076794
12136	DUSP7	520.7654435	-0.276426039	0.144264	-1.916114	0.055351	0.089385
14600	LOC338799	274.1260539	-0.276485892	0.243503	-1.13545	0.256187	0.34394
10744	DHRSX	601.8526836	-0.276744226	0.116138	-2.3829	0.017177	0.031337
11387	TRAF2	382.4036754	-0.277039762	0.128501	-2.155931	0.031089	0.053515
12847	TBKBP1	298.2345628	-0.27706177	0.163801	-1.691454	0.09075	0.13846
10174	C17orf59	223.4802432	-0.277220176	0.106999	-2.590861	0.009574	0.018444
11586	EEF2	53272.81074	-0.277588001	0.133101	-2.085552	0.037019	0.062629
14112	LEPREL1	4312.116019	-0.277613195	0.216388	-1.28294	0.199513	0.277115
12202	GEN1	238.5407817	-0.278254685	0.146783	-1.895684	0.058002	0.093173
11541	PARP8	576.6962096	-0.278385551	0.132509	-2.100887	0.035651	0.060549
11627	CLK2	638.168726	-0.278893974	0.134784	-2.069199	0.038527	0.06495
11788	MTG1	452.9337922	-0.278907029	0.137649	-2.026212	0.042743	0.071073
11936	WDR85	237.3035731	-0.279078229	0.141034	-1.978806	0.047838	0.078563
9340	CYTH1	843.1475435	-0.279365518	0.096925	-2.88228	0.003948	0.008285
15096	CRIP1	1359.179036	-0.279409354	0.285861	-0.97743	0.328356	0.426345

14184	NETO2	1579.676142	-0.279421994	0.221519	-1.261388	0.207169	0.286289
12484	HLA-H	3996.464585	-0.279808137	0.154764	-1.807972	0.070611	0.110863
11223	POLL	610.088133	-0.279850526	0.126497	-2.212307	0.026945	0.04706
12705	ARL6IP6	357.4713242	-0.279968302	0.161678	-1.731644	0.083337	0.12857
15113	C1QTNF7	61.0652853	-0.279993968	0.28798	-0.97227	0.330916	0.429186
9219	PRDX6	5178.761251	-0.280192965	0.095608	-2.930646	0.003383	0.007192
6677	ISY1	504.7968308	-0.280384918	0.070951	-3.951798	7.76E-05	0.000228
13413	PPP1R15A	2237.995793	-0.280475352	0.185788	-1.509651	0.131132	0.191637
11822	MAP3K11	2117.379327	-0.280481862	0.139275	-2.013867	0.044024	0.072991
12564	TRIM59	179.3807355	-0.280500446	0.157234	-1.783967	0.074429	0.116116
14955	AFF2	82.91960629	-0.280656011	0.2747	-1.021683	0.306931	0.402284
16475	ADH1A	3.71885571	-0.280698173	0.484777	-0.579026	0.562572	0.669315
15421	RHBDL1	65.32086165	-0.280831679	0.317859	-0.88351	0.376961	0.479139
10815	PLK1S1	347.2626048	-0.280893871	0.119176	-2.356972	0.018425	0.033393
15647	SULT1B1	4.154223172	-0.280904575	0.341069	-0.8236	0.410167	0.513816
11213	TPCN1	1806.106108	-0.280939231	0.126794	-2.215709	0.026711	0.046693
9999	LOC646762	286.3652471	-0.280950641	0.105899	-2.653001	0.007978	0.015639
14440	SPP1	39420.75743	-0.280976934	0.237025	-1.185434	0.235846	0.32014
12160	DTNB	227.02363	-0.281019697	0.147311	-1.907667	0.056434	0.090953
10907	WDYHV1	181.2272618	-0.2811957	0.121264	-2.318865	0.020402	0.036665
17324	SNORA48	0.334287808	-0.281491337	0.77892	-0.361387	0.71781	0.812156
17238	LOC285740	0.271967688	-0.281491363	0.741835	-0.379453	0.704352	0.800905
17623	GLRA3	0.364255827	-0.281491443	0.977056	-0.288102	0.773269	0.86006
17359	FAM48B2	0.234816023	-0.281491445	0.803218	-0.350455	0.725998	0.819764
17477	CLEC4M	0.297572531	-0.281491448	0.881443	-0.319353	0.749459	0.84054
17613	KRT31	0.274487544	-0.281491546	0.968406	-0.290675	0.7713	0.858357
17788	OC1001334	0.355279279	-0.281491563	1.136284	-0.24773	0.804343	0.886324
17584	C8orf75	0.238078387	-0.281491579	0.947804	-0.296994	0.766471	0.854438
17549	HIST2H2AB	0.176163242	-0.281491637	0.930858	-0.3024	0.762347	0.851439
17633	GCM2	0.172391542	-0.28149171	0.992393	-0.283649	0.776679	0.863401
17734	OTOP2	0.280567931	-0.281491725	1.083602	-0.259774	0.795038	0.878738
17629	KIR3DL3	0.154773845	-0.281491735	0.989903	-0.284363	0.776132	0.862951
17920	C2orf53	0.272792503	-0.281491843	1.253448	-0.224574	0.822311	0.899448
17799	PGK2	0.194750322	-0.281491869	1.142355	-0.246414	0.805362	0.886898
17803	OC1001333	0.189432833	-0.281491872	1.144866	-0.245873	0.80578	0.88716
17886	CACNG7	0.214864629	-0.28149194	1.224578	-0.229868	0.818194	0.896647
17902	FLJ43859	0.206873806	-0.281491955	1.235591	-0.22782	0.819786	0.897565
17885	CNTNAP4	0.191043645	-0.281491982	1.224246	-0.229931	0.818145	0.896644
17878	OR52H1	0.136882811	-0.281492105	1.220066	-0.230719	0.817533	0.896274
17880	SNORA72	0.13668528	-0.281492105	1.220221	-0.230689	0.817556	0.896274
17976	OR1K1	0.190719092	-0.281492139	1.32873	-0.21185	0.832224	0.907455
17965	HOXC12	0.157001068	-0.281492221	1.317246	-0.213698	0.830783	0.906439
17973	TMCO2	0.117315782	-0.281492338	1.326491	-0.212208	0.831945	0.907302
18109	NLRP8	0.192215991	-0.281492513	1.550772	-0.181518	0.855961	0.926484
18124	CACNG1	0.133284539	-0.281492765	1.571518	-0.179122	0.857842	0.927751
18123	SERPINI2	0.118735757	-0.281492835	1.570961	-0.179185	0.857792	0.927749
18127	OR51Q1	0.114775223	-0.281492847	1.5765	-0.178556	0.858287	0.928078

18131	POM121L4I	0.112627889	-0.281492854	1.579618	-0.178203	0.858563	0.928204
18196	LOC643486	0.118942173	-0.281493197	1.727697	-0.16293	0.870574	0.937795
18197	C6orf191	0.117431661	-0.281493211	1.733402	-0.162393	0.870996	0.938198
18198	MUC21	0.11443499	-0.281493215	1.735095	-0.162235	0.871121	0.938281
18264	SMEK3P	0.175486847	-0.281493276	1.880034	-0.149728	0.880979	0.945545
18209	PANX3	0.09601429	-0.281493359	1.75758	-0.16016	0.872755	0.939474
18272	SNORA34	0.138715178	-0.281493523	1.896724	-0.14841	0.882019	0.946172
18282	C1orf49	0.114985856	-0.281493687	1.919271	-0.146667	0.883395	0.94713
18283	OR1L3	0.113824406	-0.281493693	1.9216	-0.146489	0.883535	0.947228
18286	FGF3	0.115318361	-0.281493703	1.925334	-0.146205	0.883759	0.947313
18315	DRD3	0.077020397	-0.281494122	1.994387	-0.141143	0.887757	0.950026
18320	VSX2	0.075554433	-0.281494137	1.999202	-0.140803	0.888025	0.950134
18387	CYP11B1	0.114467472	-0.281494337	2.147234	-0.131096	0.895699	0.954824
18392	PRR23A	0.115319685	-0.281494354	2.153121	-0.130738	0.895983	0.954883
18395	KRTAP17-1	0.100754672	-0.281494556	2.174752	-0.129438	0.897011	0.955821
18399	LRRC10	0.094583793	-0.281494598	2.187921	-0.128658	0.897628	0.95627
18400	OR52N1	0.094615404	-0.281494605	2.190141	-0.128528	0.897731	0.956328
18417	SPRR2C	0.078768397	-0.281494889	2.233452	-0.126036	0.899704	0.957629
18419	GPR50	0.075579879	-0.281494932	2.246144	-0.125324	0.900267	0.957988
18421	CPXCR1	0.074489304	-0.281494948	2.250638	-0.125073	0.900465	0.958147
18495	PRAMEF11	0.091839078	-0.281495662	2.498793	-0.112653	0.910306	0.964742
18502	HBZ	0.077959013	-0.281496019	2.545147	-0.110601	0.911933	0.966101
18506	KRT9	0.075699055	-0.281496055	2.554304	-0.110205	0.912247	0.966225
18520	GHSR	0.059046782	-0.281496616	2.645498	-0.106406	0.91526	0.968683
18581	DCAF4L2	0.073461416	-0.281497547	2.91407	-0.096599	0.923045	0.972284
18587	FOXR2	0.077337648	-0.281497547	2.91407	-0.096599	0.923045	0.972284
18592	IL21	0.078220444	-0.281497547	2.91407	-0.096599	0.923045	0.972284
18600	PLSCR5	0.075339349	-0.281497547	2.91407	-0.096599	0.923045	0.972284
18575	AADACL3	0.053324315	-0.281497792	2.91407	-0.0966	0.923044	0.972284
18577	ACSM4	0.059356555	-0.281497792	2.91407	-0.0966	0.923044	0.972284
18578	C18orf26	0.058051271	-0.281497792	2.91407	-0.0966	0.923044	0.972284
18579	CSH1	0.059120482	-0.281497792	2.91407	-0.0966	0.923044	0.972284
18582	DKFZp686A1(	0.075020294	-0.281497792	2.91407	-0.0966	0.923044	0.972284
18584	F13B	0.0576037	-0.281497792	2.91407	-0.0966	0.923044	0.972284
18585	F9	0.056178166	-0.281497792	2.91407	-0.0966	0.923044	0.972284
18586	FOXD4L5	0.058554815	-0.281497792	2.91407	-0.0966	0.923044	0.972284
18588	GAGE1	0.056949673	-0.281497792	2.91407	-0.0966	0.923044	0.972284
18590	H1FOO	0.057894096	-0.281497792	2.91407	-0.0966	0.923044	0.972284
18593	KRTAP4-1	0.06162046	-0.281497792	2.91407	-0.0966	0.923044	0.972284
18594	OC1001302	0.060739295	-0.281497792	2.91407	-0.0966	0.923044	0.972284
18595	MC2R	0.053153344	-0.281497792	2.91407	-0.0966	0.923044	0.972284
18596	OR2B2	0.054977845	-0.281497792	2.91407	-0.0966	0.923044	0.972284
18598	PAGE1	0.058022724	-0.281497792	2.91407	-0.0966	0.923044	0.972284
18603	SAG	0.057817391	-0.281497792	2.91407	-0.0966	0.923044	0.972284
18608	MPRSS11BI	0.052374962	-0.281497792	2.91407	-0.0966	0.923044	0.972284
18597	OTOP3	0.056023153	-0.281497792	2.91407	-0.0966	0.923044	0.972284
18604	SNORA38E	0.058170207	-0.281497792	2.91407	-0.0966	0.923044	0.972284

12116	SYNGAP1	293.5264367	-0.281586132	0.146415	-1.9232	0.054455	0.088096
11955	C3orf34	69.7720143	-0.28182796	0.142857	-1.972798	0.048519	0.079542
12075	CISD3	928.7331541	-0.281917744	0.145649	-1.935595	0.052917	0.085892
16940	FEV	0.420473955	-0.281977774	0.622925	-0.452667	0.650788	0.753017
10245	LOC38878(	699.7891027	-0.281989193	0.109863	-2.566733	0.010266	0.019641
13377	ATAD5	53.38600629	-0.282015451	0.185764	-1.518142	0.128979	0.188989
12743	FAHD2A	363.6172344	-0.282037718	0.164044	-1.719281	0.085563	0.131604
10319	DENND4B	937.7976374	-0.282110486	0.111026	-2.540944	0.011055	0.021
10879	WDR8	298.6993191	-0.282124173	0.121107	-2.329543	0.01983	0.035729
8641	RPIA	316.9682576	-0.28226067	0.089489	-3.154143	0.00161	0.003651
16623	TSPYL6	0.528965071	-0.282377337	0.520414	-0.542601	0.587405	0.692638
11501	PPAPDC1E	595.1236611	-0.282437743	0.133385	-2.117464	0.034221	0.058322
14626	LOC64284(	174.646478	-0.282553981	0.251142	-1.125079	0.260556	0.349183
11844	PPOX	265.9938418	-0.282772978	0.140849	-2.007627	0.044683	0.073947
14043	CEMP1	316.8892739	-0.282774297	0.216963	-1.30333	0.192462	0.268636
12189	WDR83	197.0549933	-0.283167214	0.14904	-1.899946	0.05744	0.092369
11295	C22orf27	102.5081711	-0.283207184	0.129949	-2.179367	0.029304	0.050854
9657	DRG2	639.0711417	-0.283251931	0.102546	-2.762192	0.005741	0.011654
12380	TNFSF12	1019.319207	-0.283446244	0.153886	-1.841928	0.065486	0.103682
17163	COL2A1	1.077469787	-0.28344923	0.717291	-0.395166	0.69272	0.791121
14396	ADAMTS8	16.294296	-0.283468354	0.236443	-1.198889	0.230571	0.313936
14928	RXFP1	30.14774654	-0.28347346	0.276118	-1.026637	0.304591	0.399939
15843	PTGFR	108.732265	-0.283491095	0.372573	-0.7609	0.446717	0.552729
13910	SLC45A3	133.5715311	-0.283783657	0.210611	-1.347428	0.177842	0.250603
10488	PIP5K1C	1258.43887	-0.283794426	0.114646	-2.475395	0.013309	0.024873
11169	C19orf44	220.6670548	-0.283860835	0.127162	-2.23228	0.025596	0.04492
10674	CRELD1	707.4848043	-0.283868983	0.118079	-2.40407	0.016214	0.029774
11451	TGIF1	1143.692036	-0.284083428	0.133109	-2.134216	0.032825	0.05619
13511	C6orf226	64.21994253	-0.284321855	0.191508	-1.484644	0.137638	0.199678
6890	SAE1	1344.730745	-0.28448039	0.073546	-3.868053	0.00011	0.000312
17591	FAM138E	0.43758361	-0.284599295	0.962029	-0.295832	0.767358	0.855039
10379	TTC13	261.5384806	-0.284814113	0.11335	-2.512696	0.011981	0.022627
15607	SCARNA7	10.26038202	-0.284931161	0.341726	-0.8338	0.404394	0.507927
13410	GNG2	659.0651801	-0.285080576	0.188783	-1.510094	0.13102	0.191507
12751	C21orf2	495.6039793	-0.285084089	0.165935	-1.718047	0.085788	0.131874
12067	SQSTM1	13597.45493	-0.28518049	0.147049	-1.939353	0.052458	0.085211
8053	SFRS4	1782.451138	-0.285258973	0.084111	-3.391451	0.000695	0.001692
14107	PLAGL1	685.4393455	-0.285316609	0.221647	-1.287259	0.198004	0.275117
15329	PDE6A	1.43004318	-0.285344168	0.31337	-0.910565	0.362524	0.463555
10285	OVCA2	588.1679935	-0.285456999	0.111881	-2.55143	0.010728	0.020446
12292	MPND	451.2312306	-0.285554516	0.152839	-1.868339	0.061715	0.098411
10919	RPL15	15116.19855	-0.285743007	0.123593	-2.311977	0.020779	0.037301
10589	BNIP1	226.7001073	-0.286079571	0.117252	-2.439865	0.014693	0.027197
15649	FAM40B	116.6248377	-0.286154064	0.347673	-0.823055	0.410477	0.514139
8027	PARN	1069.138413	-0.286338237	0.084215	-3.400094	0.000674	0.001645
7861	POLR2D	446.5934068	-0.286420477	0.082627	-3.466421	0.000527	0.001315
6059	HNRNPUL1	4361.384044	-0.286573855	0.067589	-4.239974	2.24E-05	7.23E-05

10258	SFRS8	864.4372183	-0.286579135	0.111843	-2.562327	0.010397	0.019867
7777	FAM134C	1832.638019	-0.28660088	0.081694	-3.508237	0.000451	0.001137
15467	C3orf45	1.663813957	-0.286700349	0.329268	-0.87072	0.383907	0.486517
11742	CCDC94	396.3135406	-0.286891714	0.140667	-2.039513	0.041399	0.069107
13539	PRTFDC1	109.5387216	-0.287099952	0.194796	-1.473852	0.140521	0.203439
14305	GPSM1	276.4748249	-0.287120775	0.233858	-1.227759	0.219538	0.300815
8665	WDR41	796.1700757	-0.28746684	0.091376	-3.145981	0.001655	0.003744
10173	FPGS	627.9145236	-0.287495958	0.110905	-2.592273	0.009534	0.018371
12719	USP18	243.6531168	-0.287520188	0.166472	-1.727135	0.084143	0.129672
11796	UBE2E2	426.3448983	-0.287571433	0.142162	-2.022837	0.04309	0.071601
9490	C12orf65	384.2174975	-0.287686191	0.101968	-2.821342	0.004782	0.009875
8091	DVL3	1902.790237	-0.287837915	0.085326	-3.373394	0.000742	0.001798
9153	CRTC2	878.7114354	-0.287932684	0.097375	-2.956958	0.003107	0.006653
9127	CUTC	288.238081	-0.288134174	0.09713	-2.966479	0.003012	0.006469
8788	ZNF259	712.8927032	-0.288170883	0.093272	-3.08959	0.002004	0.004471
10889	ZNF862	505.8437743	-0.288257892	0.124052	-2.323694	0.020142	0.036257
13040	AP1G2	845.7245716	-0.288360849	0.176644	-1.632442	0.102586	0.15419
9231	SUPT5H	2786.157406	-0.288395938	0.098508	-2.927648	0.003415	0.007252
14768	FKBP1AP1	13.82485673	-0.288403444	0.267533	-1.07801	0.281029	0.372999
13312	BAHCC1	443.7562625	-0.288409273	0.18739	-1.539087	0.123783	0.182262
16965	CYorf15A	99.67133876	-0.288497113	0.643273	-0.448483	0.653805	0.755392
12550	TMEM209	658.5668166	-0.288582339	0.16116	-1.790657	0.073348	0.114564
14527	AXIN2	83.13407956	-0.288663629	0.249495	-1.156989	0.247277	0.333646
12087	C3orf64	582.2093079	-0.288944116	0.149566	-1.931885	0.053374	0.086553
13282	PGP	334.2117537	-0.28900047	0.186457	-1.549954	0.121153	0.178792
12370	ZNF273	86.76029017	-0.289131364	0.15684	-1.843474	0.06526	0.103408
11250	CASP3	620.4116239	-0.289268624	0.131482	-2.200064	0.027802	0.048436
9199	EIF3H	3824.232312	-0.289896081	0.098662	-2.938276	0.0033	0.007032
13998	C10orf75	108.7927648	-0.290273951	0.22017	-1.318411	0.187366	0.262345
10141	MARS	1479.376355	-0.290387617	0.111628	-2.601385	0.009285	0.017946
14879	43891	177.2746485	-0.290828486	0.278782	-1.043211	0.296851	0.391073
13060	C5orf45	255.04651	-0.291092722	0.179066	-1.625615	0.104032	0.156135
9162	POC5	187.389562	-0.291328552	0.098619	-2.954081	0.003136	0.006709
11688	SOLH	1052.373781	-0.291505719	0.141951	-2.053572	0.040017	0.067104
15600	LY6K	27.30799576	-0.291566978	0.348912	-0.835647	0.403354	0.506803
10631	HSPA5	17240.67297	-0.29173368	0.120475	-2.421538	0.015455	0.028494
9067	STK35	936.4364364	-0.291811983	0.097535	-2.99187	0.002773	0.005994
10262	PPT1	4488.781412	-0.291910649	0.113951	-2.561731	0.010415	0.019894
11590	THOP1	498.7266046	-0.292179113	0.140225	-2.083649	0.037192	0.062899
12630	UROS	766.0550066	-0.292209424	0.165643	-1.764095	0.077716	0.12061
7749	RRP1B	670.6325737	-0.292343153	0.083065	-3.519444	0.000432	0.001094
11592	TMEM9	2398.025718	-0.292666067	0.140501	-2.083024	0.037249	0.062985
15010	CHAC1	59.53060538	-0.292751946	0.291171	-1.005428	0.314691	0.410943
15047	PDE4C	58.01991682	-0.292781301	0.295295	-0.991487	0.321448	0.418735
12944	TP53I3	735.7349464	-0.292859528	0.176251	-1.661608	0.096591	0.146268
10504	CIC	1573.609558	-0.292934992	0.118591	-2.470122	0.013507	0.025203
11113	WDFY2	174.8225213	-0.293071155	0.130383	-2.247778	0.02459	0.043372

13321	SMPD3	37.31192961	-0.293224941	0.190912	-1.535921	0.124558	0.183279
14230	LOC390595	9.65635121	-0.293270815	0.234778	-1.24914	0.211614	0.291465
11066	BCS1L	384.2178938	-0.293308093	0.129339	-2.267742	0.023345	0.041349
11438	GALNT1	1778.892908	-0.293534262	0.137294	-2.138005	0.032516	0.055722
13652	PLCXD2	60.13823044	-0.293798589	0.204374	-1.437552	0.150561	0.21617
9310	ATXN2L	2361.694947	-0.294203151	0.101577	-2.896353	0.003775	0.007948
9977	ANKRD54	411.8951049	-0.294559282	0.110711	-2.660624	0.0078	0.015323
12403	WDHD1	110.2568028	-0.294641222	0.160513	-1.83562	0.066414	0.104957
13293	C19orf23	16.80704052	-0.294717454	0.190702	-1.545434	0.122241	0.180236
9062	LOC285074	240.0567808	-0.294878345	0.098497	-2.993787	0.002755	0.00596
15871	ASMT	0.937153584	-0.29505186	0.391995	-0.752693	0.451634	0.557777
14222	MST4	147.9435938	-0.295324858	0.23605	-1.25111	0.210894	0.290638
10637	RPL7	1409.456852	-0.295393289	0.122082	-2.419632	0.015536	0.028629
15210	PMAIP1	125.605315	-0.295431016	0.312734	-0.944672	0.344826	0.444375
12596	FLJ45340	906.5837607	-0.295638979	0.16661	-1.77444	0.07599	0.118251
16009	UGT1A1	47.37056534	-0.295658427	0.416975	-0.709056	0.47829	0.585606
13200	HLA-F	3102.310542	-0.295708127	0.187318	-1.578645	0.114418	0.169901
7003	CAP1	6780.846439	-0.295781019	0.077384	-3.822252	0.000132	0.00037
11661	STARD13	849.2939306	-0.295885984	0.143695	-2.059126	0.039482	0.066366
13126	NBPF9	326.3238324	-0.295915793	0.184644	-1.602625	0.109017	0.162795
8789	ACTR1A	2397.080344	-0.295952049	0.095794	-3.08947	0.002005	0.004472
16996	DDX3Y	780.9522311	-0.296072516	0.672009	-0.440578	0.659518	0.760604
7992	PDCL3	447.7510215	-0.296098514	0.086776	-3.412223	0.000644	0.00158
14733	SOBP	139.9781006	-0.296345729	0.2721	-1.089106	0.276107	0.367337
10963	TET3	496.1315538	-0.296521357	0.128949	-2.299516	0.021476	0.038397
10354	C11orf31	975.7802655	-0.29652185	0.11741	-2.525534	0.011552	0.021869
10961	PDRG1	386.4608958	-0.296693498	0.128996	-2.300024	0.021447	0.038352
10511	GSS	1325.945248	-0.296707557	0.120218	-2.468081	0.013584	0.025331
11289	TNIP1	7723.692568	-0.296857676	0.136009	-2.182627	0.029063	0.050462
15343	LOC61303	26.17570525	-0.296960328	0.327503	-0.90674	0.364544	0.465713
12068	PDE4DIP	2134.146465	-0.297134775	0.153264	-1.938717	0.052536	0.085329
13973	SLC46A2	10.47015241	-0.297168298	0.223777	-1.327968	0.184189	0.258376
10151	TESK1	794.0532589	-0.297247021	0.114387	-2.598609	0.00936	0.018074
13149	ZNF423	135.0502906	-0.297253755	0.186327	-1.595336	0.110637	0.164925
11245	TMEM42	205.6938757	-0.297341248	0.13505	-2.201717	0.027685	0.048258
13811	SNORD1C	25.39837473	-0.29752399	0.215167	-1.382757	0.166739	0.236625
14944	TGFA	3178.641625	-0.297581733	0.290688	-1.023715	0.30597	0.40132
13181	MTP18	1100.833449	-0.297911219	0.187953	-1.585027	0.11296	0.167979
16597	GPR152	0.53558489	-0.298152586	0.543755	-0.548322	0.583471	0.688909
9125	PWP2	489.0793503	-0.298208082	0.100491	-2.967523	0.003002	0.006449
14066	OXER1	121.3366352	-0.298865124	0.230306	-1.297689	0.194394	0.270889
9151	CDK11B	602.815462	-0.298979631	0.101061	-2.95841	0.003092	0.006624
13461	MAP1B	2806.76962	-0.299176266	0.199655	-1.498464	0.134013	0.19514
12589	PMS2L3	51.88547438	-0.299198237	0.168379	-1.776936	0.075579	0.117676
13915	LOC20218	39.39090937	-0.299367366	0.222524	-1.345327	0.17852	0.251449
14159	AHSA2	613.2483569	-0.299464322	0.236145	-1.268139	0.204748	0.283443
8019	UBE2V1	3125.832244	-0.29951935	0.087965	-3.404993	0.000662	0.001617

6161	FAM32A	1537.372554	-0.299676196	0.071414	-4.196339	2.71E-05	8.63E-05
13527	TYW1B	86.93597526	-0.299989387	0.202834	-1.478991	0.139143	0.201625
9122	SLC35A2	661.2306308	-0.300001302	0.101067	-2.968335	0.002994	0.006434
12449	ADC	123.018894	-0.300281788	0.164952	-1.820423	0.068695	0.10816
15965	PCDHA8	9.006917316	-0.30049621	0.415301	-0.723563	0.469334	0.576224
14198	LBXCOR1	3.344741653	-0.300520342	0.238877	-1.258055	0.208372	0.287667
12718	PCGF2	804.7165107	-0.300557015	0.173993	-1.727409	0.084094	0.129606
9623	CCDC86	560.6635445	-0.300569389	0.10845	-2.771513	0.00558	0.011365
14311	LYVE1	368.1443327	-0.300797379	0.2452	-1.226741	0.21992	0.301213
9873	PCNA	1369.757888	-0.300854706	0.111588	-2.696118	0.007015	0.013928
15605	ADAMTS18	46.41113021	-0.301054271	0.36109	-0.833737	0.404429	0.507927
6599	DCP1B	344.7285085	-0.301139972	0.07559	-3.983848	6.78E-05	0.000201
8071	IFT52	633.3904786	-0.301399709	0.08906	-3.384225	0.000714	0.001734
8351	YIPF1	644.2361096	-0.301463894	0.092066	-3.274449	0.001059	0.002485
9805	GTF2H5	547.394703	-0.301545116	0.111059	-2.715191	0.006624	0.013241
12107	PPIL3	400.1969505	-0.301566341	0.156505	-1.926884	0.053994	0.087415
11986	NDUFAF3	1034.128934	-0.301681317	0.153589	-1.964213	0.049505	0.080957
9739	TBC1D9B	4304.984029	-0.301723464	0.110247	-2.736789	0.006204	0.012487
9539	ZNF646	487.738341	-0.301745118	0.107648	-2.803078	0.005062	0.010401
15480	ARGFXP2	1.356092555	-0.302110887	0.348668	-0.866471	0.386232	0.489052
12358	SSSCA1	336.7910419	-0.302282279	0.163568	-1.848052	0.064595	0.102454
10683	DFFB	80.79462916	-0.302297329	0.125862	-2.401818	0.016314	0.029932
15283	IRX5	151.7706827	-0.302354447	0.32675	-0.925338	0.35479	0.455031
13477	CNTD1	30.50711255	-0.30240189	0.202207	-1.495506	0.134783	0.196028
11654	GIGYF1	1219.47473	-0.302434571	0.146841	-2.059612	0.039436	0.066327
12225	TRMT61A	377.6703218	-0.302475834	0.16001	-1.890352	0.058711	0.094135
12794	ZNF83	1541.668411	-0.302492971	0.177288	-1.706227	0.087966	0.134768
14774	FAM7A3	8.683741538	-0.30288475	0.281562	-1.075732	0.282047	0.374198
10150	SAAL1	168.8267421	-0.30295544	0.116576	-2.598782	0.009356	0.018068
13136	RGS14	795.3324246	-0.302995542	0.189637	-1.597762	0.110096	0.164281
13038	RIBC1	51.78848173	-0.303040437	0.185605	-1.63272	0.102528	0.154138
11092	GMPPB	270.807754	-0.303119422	0.134247	-2.257921	0.023951	0.042324
9940	CD2BP2	1685.640515	-0.303176035	0.113388	-2.673792	0.0075	0.014789
11550	RPS6	29036.11937	-0.303330173	0.144642	-2.097113	0.035984	0.061066
8973	PSMA4	1994.428999	-0.303337561	0.100348	-3.022863	0.002504	0.00547
16698	LOC1001328	0.520458602	-0.303397236	0.583744	-0.519744	0.603242	0.708118
12574	RPL13P5	27.81075488	-0.303477328	0.170477	-1.780164	0.075049	0.11699
13290	TRIM45	92.28417557	-0.303558265	0.196318	-1.546255	0.122043	0.179997
13186	FBXO25	728.7130747	-0.303572548	0.191661	-1.583902	0.113216	0.168296
13228	WASH7P	1195.094357	-0.303636915	0.193122	-1.572256	0.115891	0.171725
9741	CETN2	1371.730252	-0.303700586	0.111024	-2.735445	0.00623	0.012535
7789	DDX55	407.293283	-0.303989002	0.086734	-3.50484	0.000457	0.00115
13381	PFKP	9084.572991	-0.304212371	0.20052	-1.517117	0.129237	0.189312
13479	SHF	77.81112371	-0.304257831	0.203512	-1.495034	0.134906	0.196178
15263	CPA3	178.3537053	-0.304454766	0.32736	-0.93003	0.352356	0.452501
10366	QARS	2747.957451	-0.304606743	0.120826	-2.521042	0.011701	0.022125
14294	GPR77	22.48985845	-0.304653107	0.247579	-1.230529	0.218499	0.299612

9325	KIAA0090	1339.611002	-0.304878778	0.105473	-2.890575	0.003845	0.008083
12512	MSX1	88.92617239	-0.304914134	0.169393	-1.800042	0.071854	0.112565
9671	MLLT1	1142.971934	-0.305141033	0.110715	-2.756102	0.005849	0.011856
13299	CD93	5483.453748	-0.305509219	0.197999	-1.542985	0.122834	0.181028
11178	ACOT8	381.4113316	-0.30561212	0.137055	-2.229853	0.025757	0.045166
14070	BVES	78.25785306	-0.305723309	0.235765	-1.296727	0.194725	0.271273
10563	SYMPK	1652.149774	-0.30572694	0.124889	-2.447987	0.014366	0.026657
12770	PWWP2B	156.3798687	-0.305777323	0.178457	-1.713456	0.086629	0.132969
13384	CTAGE9	218.0938201	-0.305816665	0.201728	-1.515984	0.129523	0.189688
10236	TNRC18	2217.828884	-0.30582676	0.118972	-2.57058	0.010153	0.019442
16180	INGX	0.724971346	-0.305861924	0.463601	-0.659753	0.509412	0.617119
15075	PNMAL1	298.869503	-0.306112166	0.310739	-0.985109	0.324571	0.422017
14795	ATF3	1696.701819	-0.306378456	0.286972	-1.067626	0.285689	0.378493
10802	CEP72	49.08438865	-0.306750941	0.129948	-2.360562	0.018247	0.033111
16554	LCNL1	0.833977733	-0.307451242	0.550433	-0.558563	0.57646	0.682566
14695	LOC728264	283.3291382	-0.307507145	0.279251	-1.101186	0.270816	0.361229
17017	XIRP2	0.582833259	-0.307509722	0.704384	-0.436566	0.662426	0.763015
11352	CLTCL1	158.4599338	-0.307555234	0.142033	-2.165385	0.030358	0.052418
11425	ITGB1	16241.88491	-0.307656566	0.143732	-2.140489	0.032315	0.055441
9087	PRDM15	154.3638257	-0.307731962	0.103153	-2.983258	0.002852	0.006152
9089	MSN	16331.01947	-0.307774642	0.103186	-2.982706	0.002857	0.006162
11611	GRK5	462.2382669	-0.307905392	0.148211	-2.077473	0.037758	0.063741
15760	GPR182	4.598379166	-0.308211168	0.391655	-0.786946	0.431313	0.536432
13516	DUSP5	609.4053941	-0.308296938	0.208029	-1.481991	0.138343	0.200626
14092	TIGD3	7.936267466	-0.308400524	0.239053	-1.290094	0.197018	0.274039
8059	C9orf25	768.355979	-0.308549177	0.091007	-3.390373	0.000698	0.001698
6705	UBE2Z	2970.218966	-0.308562675	0.078319	-3.939842	8.15E-05	0.000238
13832	LOC344967	5.102074478	-0.30875707	0.224747	-1.373799	0.169504	0.2402
9923	PDCD6	2129.264963	-0.308848877	0.115275	-2.679244	0.007379	0.014576
13632	FAM70B	157.5508646	-0.308875973	0.213978	-1.443493	0.148882	0.214072
10994	ZNF517	190.8063323	-0.308895416	0.134892	-2.289938	0.022025	0.039269
8389	ANXA5	9652.062661	-0.309006038	0.09495	-3.254422	0.001136	0.002655
9849	PMS2L1	273.9048802	-0.309007334	0.114258	-2.704461	0.006842	0.013616
10171	VMAC	60.31949558	-0.309372584	0.119305	-2.593133	0.009511	0.018328
11561	C10orf11	148.5848577	-0.309532047	0.147853	-2.093509	0.036304	0.061551
9687	PREB	1282.830861	-0.309553379	0.112568	-2.749913	0.005961	0.012062
12801	FLJ44635	29.35370679	-0.309665355	0.181797	-1.70336	0.088501	0.135513
10406	LAMC1	7519.449563	-0.309715326	0.12351	-2.507618	0.012155	0.022895
10310	TCTN1	1092.827771	-0.309973167	0.121922	-2.542383	0.01101	0.020932
11864	C19orf6	2499.287036	-0.310055357	0.154862	-2.002133	0.04527	0.074793
14922	VASH2	45.6607685	-0.310092388	0.301068	-1.029973	0.303022	0.398046
11511	NFKBIB	459.7801643	-0.310143663	0.146826	-2.112327	0.034658	0.059017
13154	FAM131B	37.86398613	-0.310315191	0.194791	-1.593069	0.111145	0.165606
13979	MRPL42P5	8.917397016	-0.310351099	0.234322	-1.324467	0.185348	0.25989
12318	RARA	1471.760688	-0.310453873	0.166948	-1.859589	0.062944	0.100159
11139	NDUFB11	1390.238218	-0.310480682	0.138613	-2.239915	0.025096	0.044162
11834	DNAJB2	2217.675528	-0.310491868	0.154495	-2.009724	0.04446	0.073641

14309	NEXN	304.9083897	-0.310643908	0.2532	-1.226872	0.219871	0.301187
14836	ST7OT4	2.167962998	-0.310699986	0.294222	-1.056004	0.290966	0.384418
7108	CCT7	4126.169166	-0.310700221	0.082169	-3.781219	0.000156	0.00043
16838	CMA1	1.262531067	-0.31095202	0.647136	-0.480505	0.630868	0.734389
13495	EPHB6	227.3268689	-0.310978913	0.208785	-1.489466	0.136365	0.19805
7962	MTMR14	742.6145252	-0.310999728	0.090848	-3.423305	0.000619	0.001523
9550	DDA1	630.7115575	-0.31110266	0.111044	-2.801614	0.005085	0.010436
11660	MDP1	257.3818595	-0.311153847	0.151101	-2.059244	0.039471	0.066352
14960	SCGB3A1	6.712310702	-0.311289312	0.305564	-1.018735	0.308329	0.403981
11838	TRAF4	749.4026308	-0.311310127	0.154935	-2.00929	0.044506	0.073697
16099	CTSE	23.3311955	-0.311425163	0.457178	-0.681191	0.495751	0.603591
10986	NADSYN1	1182.677612	-0.311512225	0.135924	-2.291806	0.021917	0.039104
14990	AKR1C2	296.7242794	-0.311518612	0.308508	-1.009757	0.312612	0.408773
14830	NR1I2	7.864871118	-0.311550032	0.294706	-1.057157	0.29044	0.383852
10881	DCBLD1	265.1656057	-0.311771478	0.133884	-2.328676	0.019876	0.035805
7098	MED26	200.0485821	-0.311960576	0.082452	-3.783525	0.000155	0.000427
9055	HSPC157	208.308765	-0.312003619	0.104159	-2.995443	0.00274	0.005932
9114	GNL3	796.2873462	-0.312214005	0.105004	-2.973346	0.002946	0.006335
8711	PPP1R7	1243.241487	-0.312358917	0.09992	-3.126097	0.001771	0.003986
14125	BRIP1	39.89990975	-0.312449486	0.244124	-1.279881	0.200587	0.278351
10129	LOC14841[illegible]	256.7967137	-0.312549258	0.11995	-2.605666	0.00917	0.017743
11573	TBL3	865.4348672	-0.312670039	0.149502	-2.091414	0.036491	0.061804
11673	SRD5A1	396.944138	-0.312690838	0.151983	-2.057405	0.039647	0.066575
15049	ABCB4	123.7687709	-0.31270619	0.315702	-0.990512	0.321924	0.419299
7316	NDE1	306.7513715	-0.312710388	0.084646	-3.694334	0.00022	0.000591
8007	GLT8D1	837.9610477	-0.31283096	0.091822	-3.406912	0.000657	0.001608
11506	C9orf89	284.6760525	-0.312863499	0.147983	-2.11418	0.0345	0.058772
6450	DKC1	854.0110056	-0.312892043	0.077281	-4.048733	5.15E-05	0.000156
9802	WBSCR16	1187.97291	-0.312970221	0.115221	-2.71626	0.006602	0.013203
8575	CPSF3	768.1520663	-0.313078967	0.098431	-3.180695	0.001469	0.003358
13517	NOVA2	125.8241229	-0.3132082	0.211352	-1.481926	0.13836	0.200636
14714	KCNAB3	12.01284965	-0.313265189	0.286589	-1.093083	0.274357	0.36548
12298	TRIM14	1258.594802	-0.313316286	0.167858	-1.86656	0.061963	0.098759
9656	CNTROB	596.863342	-0.313490674	0.113481	-2.762494	0.005736	0.011644
16326	ADAD2	0.917111573	-0.313501828	0.505155	-0.620606	0.534859	0.642113
10693	TMEM81	53.9387453	-0.313711683	0.130745	-2.399415	0.016421	0.030101
11076	FMNL3	1663.225051	-0.313719733	0.138559	-2.264168	0.023564	0.0417
8865	POLR3H	930.1029784	-0.313854567	0.102416	-3.064506	0.00218	0.004821
14110	RTDR1	58.36436407	-0.313880321	0.244452	-1.284018	0.199136	0.276631
10400	TIMM50	811.5871431	-0.313945912	0.125174	-2.508082	0.012139	0.022882
7882	WDR46	657.9361257	-0.314143564	0.090872	-3.456997	0.000546	0.001358
13821	TSC22D3	8152.858929	-0.314237915	0.227913	-1.378764	0.167968	0.238212
10575	MRPS11	524.8645727	-0.314383066	0.128559	-2.445439	0.014468	0.026816
16468	NLGN4Y	112.8199315	-0.314392404	0.540234	-0.581956	0.560596	0.667208
13675	NDUFS7	1118.067351	-0.314484956	0.220064	-1.429063	0.152986	0.219282
14898	CRNA002[illegible]	9.819188761	-0.314731969	0.303489	-1.037046	0.299714	0.394328
15238	KLHL38	4.489877761	-0.314956195	0.336312	-0.936501	0.349015	0.448947

8129	KIAA1609	429.8712766	-0.31496724	0.093746	-3.359776	0.00078	0.001881
13682	PRR5	342.2354408	-0.31502228	0.220772	-1.426915	0.153604	0.220055
15168	CXADR	180.0840869	-0.31503749	0.329299	-0.956691	0.338723	0.437718
11565	CLK2P	11.32923527	-0.315049312	0.150519	-2.093089	0.036341	0.061588
8146	COG4	1239.156558	-0.315057611	0.093932	-3.354089	0.000796	0.001916
13691	DOCK11	701.1820811	-0.315066109	0.221807	-1.420452	0.155476	0.22259
14494	MAMDC4	211.2305115	-0.315096032	0.269418	-1.169544	0.242185	0.327519
13429	LOC642826	97.28795938	-0.315169699	0.209244	-1.506233	0.132007	0.192678
13562	C7orf53	33.79471895	-0.31532563	0.215024	-1.466465	0.142522	0.20597
12778	ACVRL1	1312.278768	-0.315552892	0.18436	-1.711615	0.086968	0.133395
8828	TYK2	1447.062988	-0.315553648	0.10244	-3.080376	0.002067	0.00459
16007	RIMS4	4.076806686	-0.316234395	0.4453	-0.71016	0.477605	0.58484
11298	DUS3L	327.1263495	-0.316248565	0.145169	-2.178485	0.02937	0.050954
11446	ZMYND19	191.7426943	-0.31625385	0.148077	-2.135742	0.0327	0.055999
8959	MBD6	1237.401134	-0.316416342	0.104507	-3.027704	0.002464	0.005391
13226	LRRC6	114.7953058	-0.317084963	0.201662	-1.572358	0.115868	0.171716
12737	UQCR11	1568.675091	-0.317442204	0.184411	-1.721388	0.08518	0.131085
8998	EIF2B3	377.1174659	-0.317450292	0.105349	-3.01331	0.002584	0.005629
12582	ARRB1	390.4880179	-0.317698212	0.17865	-1.778325	0.075351	0.117386
12235	SIVA1	895.810105	-0.317779347	0.168445	-1.886551	0.059221	0.094874
16336	IGLON5	95.80429434	-0.317928557	0.515577	-0.616647	0.537468	0.644889
6232	NRD1	2660.674627	-0.317940825	0.076365	-4.163414	3.14E-05	9.86E-05
10496	GOLT1B	1191.835432	-0.318070525	0.128626	-2.472832	0.013405	0.025033
13211	CYBRD1	1969.674905	-0.318186313	0.201843	-1.576403	0.114933	0.170525
8323	PSMB5	1749.879292	-0.318311481	0.096969	-3.282597	0.001029	0.002422
13603	TTLL13	8.657877988	-0.318381798	0.21876	-1.455396	0.14556	0.20973
14653	KCTD19	3.831261311	-0.318489358	0.285394	-1.115963	0.264438	0.353733
10606	BIN3	369.0859897	-0.318490028	0.130832	-2.434338	0.014919	0.027572
12312	SUZ12P	130.2768364	-0.318498423	0.171147	-1.860966	0.062749	0.099898
12432	SERPINB9	792.434469	-0.318614655	0.174412	-1.826791	0.067731	0.10679
14308	MFSD7	193.0804862	-0.318625027	0.259687	-1.22696	0.219838	0.301163
8337	PRPF19	1891.699428	-0.31873637	0.097242	-3.277769	0.001046	0.00246
8845	COPG	4705.127913	-0.318742335	0.1037	-3.07371	0.002114	0.004685
11927	C6orf115	474.4531723	-0.318744677	0.16092	-1.980771	0.047617	0.078254
6739	DAP3	1457.322055	-0.318826193	0.081216	-3.925681	8.65E-05	0.000252
15130	LOC220594	125.8634529	-0.318875707	0.33051	-0.964798	0.334646	0.433536
11696	SPR	1161.371342	-0.319252922	0.155568	-2.052181	0.040152	0.06729
13666	SLC25A29	418.7190141	-0.319336398	0.222767	-1.433502	0.151715	0.217603
10850	VEGFB	2890.828383	-0.31938137	0.136543	-2.339048	0.019333	0.034926
10600	PNPLA2	1556.263241	-0.31941698	0.131153	-2.435447	0.014873	0.027503
15679	ARMC3	11.94358416	-0.319445048	0.392824	-0.813201	0.416103	0.520188
7095	PHF14	749.7881357	-0.319487544	0.084371	-3.78669	0.000153	0.000422
15860	C1orf170	184.6098473	-0.319560095	0.42294	-0.755569	0.449908	0.55603
11711	CORO7	890.4714682	-0.319720268	0.155967	-2.049919	0.040372	0.067572
13766	TCTEX1D4	5.430901116	-0.319845972	0.228968	-1.396905	0.162442	0.231297
13110	GJA9	6.725113102	-0.319873243	0.198952	-1.607792	0.107881	0.161295
10212	PELP1	1178.283744	-0.319964672	0.124154	-2.577161	0.009962	0.01912

11030	TTC7A	969.6398154	-0.320206503	0.140423	-2.2803	0.02259	0.040144
5490	MSL3	544.1494727	-0.320319863	0.07077	-4.526218	6.00E-06	2.14E-05
8872	LOC40783	52.39203294	-0.320475488	0.104627	-3.063042	0.002191	0.004841
14347	TTC16	11.51957519	-0.320520979	0.263852	-1.214778	0.224451	0.306647
13424	RASL11A	358.2795753	-0.320727036	0.21282	-1.507031	0.131803	0.192451
14276	C10orf125	202.5845697	-0.320898515	0.259771	-1.235314	0.216714	0.297528
13933	C11orf9	1044.367953	-0.320899629	0.239386	-1.340513	0.180079	0.253335
7579	FAM105B	760.5608837	-0.321127713	0.089557	-3.585749	0.000336	0.000869
9419	CASP10	610.957477	-0.321197947	0.11266	-2.851037	0.004358	0.009068
10791	PLXNB2	8720.600997	-0.321496403	0.135927	-2.365211	0.01802	0.032732
16370	CD1B	2.26534701	-0.321509291	0.528871	-0.607917	0.543243	0.650425
6651	WDR70	400.0091995	-0.321519868	0.08115	-3.962025	7.43E-05	0.000219
6158	RUFY1	1476.049884	-0.321718448	0.07662	-4.198862	2.68E-05	8.54E-05
9221	PTPN2	515.8859129	-0.321739897	0.109809	-2.929999	0.00339	0.007205
14206	HIGD1B	80.14128563	-0.32177755	0.256469	-1.254643	0.209608	0.289211
13256	CDK6	1198.573102	-0.321864853	0.206068	-1.561932	0.118304	0.17493
12911	TRAF1	456.4156365	-0.32204913	0.192772	-1.67062	0.094797	0.143917
14193	CPVL	1998.256419	-0.322494996	0.256053	-1.259488	0.207854	0.287054
11019	TEAD3	382.5845774	-0.322766393	0.141294	-2.284359	0.02235	0.039758
11188	WARS	3429.205081	-0.323132075	0.145209	-2.225288	0.026062	0.04566
11109	NCAPH2	808.2828163	-0.323556939	0.143834	-2.24951	0.02448	0.043193
8223	CDC42SE1	2033.081958	-0.323787061	0.097451	-3.322564	0.000892	0.002126
11119	EIF2C2	162.412878	-0.323981425	0.144272	-2.245628	0.024728	0.043591
8515	ATRIP	128.0800958	-0.324069063	0.10129	-3.199403	0.001377	0.00317
11099	LSM5	615.3954383	-0.324173512	0.143769	-2.254826	0.024144	0.042639
8474	TAF15	2176.936654	-0.324257556	0.100769	-3.217834	0.001292	0.002988
8412	C3orf10	2336.141472	-0.3242591	0.099918	-3.24525	0.001173	0.002734
16053	C15orf5	0.653936614	-0.324327812	0.465433	-0.69683	0.485909	0.593304
6258	PSMD3	2036.743824	-0.324870084	0.078257	-4.151337	3.31E-05	0.000104
6336	DDX27	963.3667628	-0.325072246	0.079092	-4.110065	3.96E-05	0.000122
10574	MAGOHB	255.920188	-0.325132807	0.132943	-2.445658	0.014459	0.026802
16549	C17orf78	0.445371633	-0.325147885	0.581794	-0.558871	0.57625	0.682463
10752	TMEM102	174.6824162	-0.325238441	0.136648	-2.380111	0.017307	0.031552
15018	KIAA1211	74.89821952	-0.325343685	0.324319	-1.003159	0.315784	0.412151
12617	ZNF775	64.11378302	-0.325368621	0.184127	-1.767086	0.077214	0.119957
4718	PSMF1	3179.289827	-0.325414366	0.066095	-4.923462	8.50E-07	3.53E-06
8904	ELMOD3	290.8607814	-0.325462418	0.106686	-3.050668	0.002283	0.005026
7589	NCSTN	3459.354862	-0.325495989	0.090838	-3.583251	0.000339	0.000876
16564	ST8SIA6	44.50613111	-0.325543537	0.586161	-0.555383	0.578633	0.684725
9754	CSNK2A2	693.6750255	-0.325548926	0.119178	-2.73162	0.006302	0.012665
11792	DOK4	1686.651042	-0.325566846	0.160838	-2.024185	0.042951	0.071395
9887	ATP13A1	1413.140962	-0.325655017	0.121001	-2.691337	0.007117	0.014109
10296	C11orf67	286.205304	-0.326061718	0.127985	-2.547665	0.010845	0.020646
11071	DCUN1D3	353.943316	-0.326072313	0.143939	-2.265346	0.023491	0.041587
9099	FAM104B	224.0439202	-0.326300944	0.109627	-2.976477	0.002916	0.006281
8516	VARS	1287.857257	-0.326424701	0.10204	-3.198979	0.001379	0.003174
11418	LOC72861	271.2354408	-0.326462453	0.152343	-2.142945	0.032118	0.055135

6569	EIF2S2	1431.846782	-0.326485291	0.081701	-3.996103	6.44E-05	0.000192
12954	MMP15	802.2457564	-0.326682903	0.197397	-1.654953	0.097934	0.148186
11145	DPY19L1	1137.215423	-0.326784901	0.145942	-2.23915	0.025146	0.044223
12316	LRRC32	2720.923282	-0.32683451	0.175704	-1.86014	0.062866	0.100051
7061	FADD	569.1092925	-0.326885012	0.086085	-3.797224	0.000146	0.000406
9398	C14orf179	328.4926803	-0.3269087	0.114312	-2.859793	0.004239	0.008841
16308	LPPR5	163.8072129	-0.326957833	0.522542	-0.625706	0.531508	0.638833
10187	RPUSD3	304.8304275	-0.327270185	0.126537	-2.586364	0.009699	0.018663
10224	NR1H2	1848.203512	-0.327356971	0.127221	-2.573144	0.010078	0.019323
15363	DDN	8.281069657	-0.3277599	0.363466	-0.901761	0.367184	0.468474
14244	PRRT2	96.86008584	-0.328020934	0.263783	-1.243527	0.213674	0.294034
9975	MRPL37	1523.759971	-0.32829994	0.123342	-2.661709	0.007775	0.015277
11162	SLC9A1	924.287306	-0.32835227	0.147001	-2.233674	0.025505	0.044787
16292	MT1H	25.57489749	-0.328623744	0.522486	-0.628962	0.529374	0.636854
10487	DGKQ	276.3328649	-0.32866975	0.13273	-2.476225	0.013278	0.024818
14087	TCEAL7	45.39485925	-0.328677996	0.254365	-1.292149	0.196306	0.273145
12021	SPSB3	801.1559751	-0.328709755	0.168113	-1.955288	0.050549	0.082424
9364	CDC37	3062.73899	-0.328747271	0.114373	-2.874348	0.004049	0.008475
15155	C10orf116	2975.823967	-0.329021242	0.343263	-0.95851	0.337806	0.436879
14151	CCDC65	31.17890457	-0.329040785	0.258823	-1.271298	0.203623	0.282044
11559	SLC25A26	354.7260021	-0.329299248	0.157267	-2.09388	0.036271	0.061505
15746	MORN5	3.687652913	-0.329669646	0.416764	-0.791023	0.428931	0.533943
9740	SERPINB1	1517.924421	-0.330042355	0.120634	-2.735909	0.006221	0.012519
13557	TMEM86A	362.1321541	-0.330062956	0.224755	-1.468542	0.141957	0.205244
12930	RASL12	329.318109	-0.330128562	0.198318	-1.664642	0.095984	0.145506
9090	RSU1	1865.54813	-0.330290093	0.110741	-2.982559	0.002858	0.006164
15204	CREG2	17.33882755	-0.330364061	0.349204	-0.94605	0.344123	0.443644
12198	C20orf135	134.5463009	-0.330367031	0.174153	-1.896992	0.057829	0.092926
12374	TMCC1	2271.336009	-0.330562724	0.179368	-1.842934	0.065339	0.1035
13333	CSRP2	1027.016683	-0.330649406	0.215687	-1.533007	0.125274	0.184167
11095	TOMM40	954.9591507	-0.330840129	0.146596	-2.256812	0.02402	0.042435
12013	MIB2	446.574598	-0.330923344	0.169122	-1.956715	0.050381	0.082203
13848	LOC646851	46.99413345	-0.331137373	0.241917	-1.368803	0.171061	0.242126
12965	CCDC84	141.0766355	-0.331222409	0.200315	-1.653504	0.098228	0.148505
6134	CAMKK2	1430.221818	-0.331300507	0.078758	-4.206585	2.59E-05	8.28E-05
6771	TRIAP1	471.5121017	-0.331308547	0.084734	-3.90999	9.23E-05	0.000267
11558	CHRNB1	351.5776735	-0.331343092	0.158198	-2.094481	0.036217	0.06142
11875	EMP2	1033.556572	-0.331366473	0.165839	-1.998123	0.045703	0.075438
11584	ATF7IP2	122.6746277	-0.331459363	0.158737	-2.0881	0.036789	0.062249
11399	GLRX2	123.4240578	-0.331511337	0.154178	-2.150187	0.03154	0.054235
14224	ASPHD2	152.3546551	-0.331878639	0.265303	-1.250942	0.210955	0.290702
10344	ANXA6	4152.686656	-0.332000489	0.131178	-2.530913	0.011377	0.021558
12331	CCS	653.6944153	-0.332106407	0.179021	-1.855128	0.063578	0.101062
7492	WDR55	607.5643377	-0.332163649	0.091771	-3.619503	0.000295	0.000772
11005	MOB2	520.0601349	-0.332420143	0.145296	-2.287876	0.022145	0.039439
8771	NOP10	1090.444193	-0.332493677	0.107416	-3.095379	0.001966	0.004393
11034	RGS12	963.7694361	-0.3325301	0.145893	-2.27927	0.022651	0.040238

11528	PKD1	1488.496702	-0.332551806	0.157719	-2.108505	0.034987	0.059489
9386	PKIG	1268.720352	-0.332577285	0.116084	-2.864967	0.004171	0.008709
7252	ATXN7L3	1009.348205	-0.332593347	0.089317	-3.723731	0.000196	0.000531
16443	TAC4	0.436916688	-0.332666494	0.565208	-0.588573	0.556147	0.66296
9681	TMEM93	296.5952582	-0.332829284	0.120899	-2.752957	0.005906	0.011958
9625	LOC728643	64.78718495	-0.3328522	0.120161	-2.770045	0.005605	0.011414
11361	ABHD14B	1933.028596	-0.332922429	0.153906	-2.163158	0.030529	0.052671
10912	GDF11	211.3299325	-0.332977265	0.143739	-2.316546	0.020528	0.036875
10218	SUN2	3857.446632	-0.333181319	0.129378	-2.575255	0.010017	0.019215
8873	MYO1E	1643.712048	-0.333396411	0.108851	-3.062858	0.002192	0.004843
15265	PIWIL1	1.810660882	-0.333521395	0.358746	-0.929686	0.352534	0.45267
9165	PCSK7	695.7816103	-0.333612257	0.112982	-2.952802	0.003149	0.006735
10992	RPTOR	888.3021383	-0.333726559	0.145736	-2.289941	0.022025	0.039269
14968	ST18	5.20152423	-0.333887392	0.328491	-1.016428	0.309426	0.405201
13729	C4orf39	38.28110614	-0.333921238	0.237313	-1.407092	0.1594	0.22756
13623	DNA2	84.89857487	-0.33437961	0.230989	-1.447599	0.147729	0.212549
12609	AVPI1	175.0224256	-0.33438242	0.189061	-1.768648	0.076953	0.119619
15806	KIF5A	4.876771874	-0.334766891	0.432593	-0.773861	0.439013	0.544385
12333	PAOX	191.0086616	-0.334827167	0.180604	-1.853927	0.06375	0.101318
10217	CTSL1	5484.311608	-0.335015177	0.130048	-2.576096	0.009992	0.01917
15187	C20orf54	104.8721467	-0.335105949	0.352077	-0.951798	0.341199	0.440367
8709	NACAP1	124.9954156	-0.335176773	0.107194	-3.126818	0.001767	0.003977
13685	KYNU	223.0382242	-0.335193099	0.235344	-1.424268	0.154369	0.221102
11054	FAM55C	381.2732315	-0.335419304	0.147562	-2.273069	0.023022	0.040823
10223	CLN3	1038.256151	-0.335532464	0.130399	-2.573115	0.010079	0.019323
15353	IHH	64.38030467	-0.33560568	0.371234	-0.904027	0.365981	0.467244
12493	TMEM97	318.5368203	-0.335634408	0.185821	-1.806223	0.070884	0.111213
9576	PGS1	570.964873	-0.335713862	0.120356	-2.789341	0.005282	0.010811
13883	GNRH1	48.68196475	-0.335864496	0.248141	-1.353521	0.175889	0.248333
9558	LOC143188	21.51973781	-0.335867574	0.120094	-2.796704	0.005163	0.010587
13289	FZD8	370.3946673	-0.335903129	0.217172	-1.546711	0.121933	0.179849
12391	PYGB	3492.554272	-0.336068672	0.182816	-1.838287	0.06602	0.104435
14496	RNF128	1925.775109	-0.336094747	0.287435	-1.169292	0.242286	0.327616
9947	CYB561	1372.855054	-0.336256915	0.125833	-2.672248	0.007535	0.014847
13173	KCNE4	855.9465352	-0.336378707	0.211732	-1.588699	0.112128	0.166831
12156	TRIM16	395.3044744	-0.336780089	0.176447	-1.90868	0.056303	0.090787
12586	DCHS1	1090.867866	-0.337168869	0.189719	-1.777201	0.075535	0.117628
13949	INHBB	1742.91329	-0.3373379	0.252874	-1.334014	0.182199	0.25601
17737	MC3R	0.400277583	-0.337339386	1.306021	-0.258296	0.796179	0.87985
11984	SLC29A1	1498.195018	-0.337574301	0.171841	-1.964461	0.049477	0.080929
12353	RASSF3	184.6752952	-0.337607844	0.182617	-1.848724	0.064498	0.102341
7103	EIF4A3	1403.003447	-0.337960272	0.089353	-3.7823	0.000155	0.000429
11876	CALD1	12197.87094	-0.338155997	0.169314	-1.997215	0.045802	0.075595
7501	C20orf72	429.5989349	-0.338178317	0.093497	-3.616992	0.000298	0.000779
12260	OSBPL3	1087.561867	-0.338214717	0.179914	-1.879869	0.060126	0.096128
12379	FANK1	83.6752375	-0.338288692	0.183655	-1.841981	0.065478	0.103678
13591	MCTP2	578.6249106	-0.338487433	0.232218	-1.45763	0.144943	0.209048

4720	PSMD7	1523.910361	-0.338524306	0.068791	-4.921091	8.61E-07	3.57E-06
9704	FLOT2	3498.498838	-0.338611382	0.123331	-2.745556	0.006041	0.012202
6430	RIC8A	1816.902596	-0.338758484	0.083429	-4.060432	4.90E-05	0.000149
12070	ZNF789	101.2739875	-0.338875571	0.174837	-1.938243	0.052594	0.085409
10822	IER5	550.7118088	-0.338937716	0.144016	-2.353478	0.018599	0.033686
14894	LRAT	73.00864896	-0.338974909	0.326433	-1.038423	0.299073	0.393591
10839	GET4	826.5853897	-0.339051907	0.144567	-2.345293	0.019012	0.034381
15406	BRD7P3	0.97077111	-0.339115064	0.381314	-0.889333	0.373824	0.475615
10295	TMEM222	899.314178	-0.339193229	0.133126	-2.547907	0.010837	0.020633
16681	C5orf60	0.262005746	-0.33919908	0.648127	-0.523353	0.600729	0.705886
16852	PHOX2A	0.264282151	-0.339199114	0.71	-0.477745	0.632831	0.736063
16988	C6orf25	0.243206499	-0.339199166	0.767269	-0.442087	0.658427	0.759702
17213	C1orf87	0.306274241	-0.339199182	0.880759	-0.385121	0.700147	0.797259
17211	CCDC37	0.182070818	-0.339199315	0.879977	-0.385464	0.699894	0.797084
17450	CEACAM1(	0.26296118	-0.339199351	1.038487	-0.326629	0.743949	0.835653
17404	HIST1H2BI	0.205105905	-0.339199403	0.997951	-0.339896	0.733935	0.826584
17410	OR51B4	0.206986434	-0.339199406	1.00016	-0.339145	0.7345	0.826935
17611	AAA1	0.282168279	-0.339199427	1.16454	-0.291273	0.770842	0.857946
17563	CACNG8	0.165597274	-0.339199619	1.131549	-0.299766	0.764356	0.853051
17569	RAD21L1	0.16780055	-0.339199621	1.133142	-0.299344	0.764677	0.853233
17573	AADACL4	0.16230859	-0.339199625	1.136248	-0.298526	0.765302	0.853615
17567	CXorf41	0.145170606	-0.339199659	1.133653	-0.299209	0.76478	0.853233
17745	C4orf40	0.215877396	-0.339199727	1.321564	-0.256665	0.797437	0.880801
17654	SCRT2	0.160338706	-0.339199733	1.218565	-0.27836	0.780736	0.866792
17740	SLC36A3	0.22825142	-0.339199771	1.316112	-0.257729	0.796616	0.880201
17659	PSMB11	0.145357569	-0.33919978	1.221549	-0.27768	0.781258	0.867175
17754	NPY2R	0.20585818	-0.339199784	1.326558	-0.255699	0.798183	0.881199
17738	OR2S2	0.162114207	-0.339199869	1.3148	-0.257986	0.796418	0.880065
17742	SNORA12	0.148136185	-0.339199925	1.31922	-0.257122	0.797085	0.880603
17748	LOC34737(	0.126638504	-0.33919998	1.322149	-0.256552	0.797525	0.88081
17747	CCL1	0.126520915	-0.339199981	1.322262	-0.25653	0.797542	0.88081
17836	SULT2A1	0.164672863	-0.339200036	1.424521	-0.238115	0.811792	0.892124
17855	GOLGA6L1	0.158278858	-0.339200064	1.441828	-0.235257	0.814009	0.893444
17956	C6orf221	0.123129877	-0.339200425	1.572367	-0.215726	0.829201	0.905167
17967	TLX1NB	0.102900344	-0.339200532	1.58976	-0.213366	0.831042	0.90662
18056	DDX53	0.105325842	-0.339200863	1.744035	-0.194492	0.845791	0.918089
18058	OR8S1	0.105018575	-0.339200864	1.744606	-0.194428	0.845841	0.918089
18060	FMR1NB	0.105460564	-0.339200868	1.746235	-0.194247	0.845983	0.918163
18077	MEG8	0.08786131	-0.339201014	1.770674	-0.191566	0.848082	0.919581
18160	OR52B2	0.088029818	-0.339201504	1.966092	-0.172526	0.863024	0.931505
18162	APOBEC1	0.086485644	-0.339201516	1.970399	-0.172149	0.863321	0.931723
18368	WFDC6	0.087033629	-0.339203144	2.510965	-0.135089	0.892542	0.952456
18370	CGB8	0.084221486	-0.339203186	2.523301	-0.134428	0.893064	0.952909
18389	MAGEB6	0.068033054	-0.339203616	2.592053	-0.130863	0.895884	0.954879
18461	C16orf92	0.107227693	-0.339204369	2.91407	-0.116402	0.907334	0.962737
18465	COX7B2	0.10563583	-0.339204369	2.91407	-0.116402	0.907334	0.962737
18471	PRAC	0.104311035	-0.339204369	2.91407	-0.116402	0.907334	0.962737

18473	TPRX1	0.084228187	-0.339204599	2.910345	-0.116551	0.907216	0.962737
18464	CARD18	0.12038205	-0.339204614	2.91407	-0.116402	0.907334	0.962737
18466	DSCR10	0.066569651	-0.339204859	2.91407	-0.116402	0.907334	0.962737
18463	C6orf127	0.071482103	-0.339204859	2.91407	-0.116402	0.907334	0.962737
18468	OR51B2	0.06675461	-0.339204859	2.91407	-0.116402	0.907334	0.962737
18462	C2orf80	0.069816311	-0.339204859	2.91407	-0.116402	0.907334	0.962737
18469	OR52K1	0.065649801	-0.339204859	2.91407	-0.116402	0.907334	0.962737
18470	OR56A3	0.066854609	-0.339204859	2.91407	-0.116402	0.907334	0.962737
18472	PRSS54	0.079565184	-0.339204859	2.91407	-0.116402	0.907334	0.962737
9726	PKM2	46904.2518	-0.339408827	0.123895	-2.739481	0.006154	0.012402
10806	WDR76	108.6946593	-0.339410539	0.143839	-2.35966	0.018292	0.033179
9502	C9orf69	708.5259644	-0.339542818	0.120424	-2.819558	0.004809	0.00992
10042	NPRL2	301.0630031	-0.339545013	0.128799	-2.636242	0.008383	0.016363
13356	DUSP4	152.4571956	-0.3395564	0.222666	-1.524961	0.127269	0.186777
11933	PLBD1	1085.084412	-0.339622465	0.171612	-1.979008	0.047815	0.07854
9987	AARSD1	542.4832038	-0.340147636	0.128016	-2.657078	0.007882	0.01547
6359	DAXX	1086.289822	-0.340563289	0.083127	-4.096894	4.19E-05	0.000129
14337	ACR	8.128243972	-0.340610362	0.279314	-1.219454	0.222672	0.304429
13005	LOC84989	7.371355555	-0.340822659	0.207498	-1.642535	0.100479	0.151441
14429	ASB12	2.909983559	-0.340931409	0.286727	-1.189046	0.234421	0.318449
11702	OAS1	1203.744999	-0.34093721	0.166218	-2.051143	0.040253	0.067426
14423	KIR2DL1	3.37175474	-0.340948334	0.286227	-1.191182	0.233582	0.317441
11706	CENPT	592.9636935	-0.341073562	0.166346	-2.050388	0.040327	0.067519
13754	SNHG4	4.391004907	-0.341149912	0.243315	-1.402089	0.160889	0.229284
9517	RPA3	403.0921586	-0.341309614	0.121306	-2.813629	0.004899	0.010089
10157	EIF3CL	10371.36561	-0.341316868	0.13142	-2.597144	0.0094	0.018141
9647	ABCC10	302.470087	-0.341387466	0.123535	-2.763483	0.005719	0.011615
8194	C14orf93	191.3598122	-0.341517373	0.102398	-3.335204	0.000852	0.002039
9914	RNF213	5092.397488	-0.341612902	0.127348	-2.68251	0.007307	0.014447
15850	ZBBX	5.02210686	-0.341642884	0.450579	-0.758231	0.448313	0.554409
9604	IGSF8	959.7923558	-0.342144782	0.123121	-2.778932	0.005454	0.011131
13731	FAM19A2	20.03421192	-0.34215048	0.243171	-1.407034	0.159417	0.227568
9173	EMD	1106.461185	-0.342170638	0.116088	-2.947511	0.003203	0.006845
15597	FAM183B	1.652225242	-0.342363639	0.409344	-0.836372	0.402946	0.506388
12320	A2M	30748.02688	-0.342444047	0.184183	-1.859258	0.062991	0.100217
12887	KIAA1257	4.033963239	-0.342564475	0.204291	-1.676844	0.093573	0.142312
8046	ZNF394	466.600653	-0.342861401	0.101024	-3.393845	0.000689	0.001679
6527	MORC2	775.6958484	-0.343126795	0.085462	-4.014978	5.95E-05	0.000179
9301	PCGF1	263.6958528	-0.343180446	0.11831	-2.900693	0.003723	0.007847
11177	MCAT	369.8984177	-0.343232893	0.153895	-2.230306	0.025727	0.04512
14426	LOC284837	14.5662028	-0.343483627	0.288714	-1.189703	0.234163	0.318164
10671	RAP2B	1112.46408	-0.343520831	0.142863	-2.404543	0.016193	0.029744
17236	LECT1	0.531612391	-0.34385701	0.904934	-0.37998	0.70396	0.800552
13853	SERINC4	7.348930712	-0.344128118	0.251627	-1.367612	0.171434	0.242566
9884	CREM	690.7271619	-0.344517335	0.127919	-2.693247	0.007076	0.014032
16892	PABPC1P2	0.523127755	-0.344517423	0.737207	-0.467328	0.640265	0.742946
10727	E4F1	384.8993794	-0.344522321	0.144291	-2.387693	0.016954	0.030979

10624	OC1001296	207.4486041	-0.34469847	0.142131	-2.425212	0.015299	0.028227
10347	ATSPER2I	26.80162489	-0.344740856	0.136267	-2.529893	0.01141	0.021614
8036	RFC5	286.1047435	-0.344761407	0.10149	-3.397	0.000681	0.001662
10830	RELB	560.5220562	-0.344775636	0.146663	-2.350799	0.018733	0.033905
9242	NOC2L	1415.746192	-0.345056331	0.118117	-2.9213	0.003486	0.007393
6254	HDAC1	1853.515333	-0.345365024	0.083162	-4.152927	3.28E-05	0.000103
10182	C8orf33	1103.10741	-0.34561883	0.133483	-2.589229	0.009619	0.018517
13166	SFMBT2	114.1704953	-0.345726054	0.217353	-1.590621	0.111695	0.166287
14864	DGCR5	1601.641909	-0.345801042	0.329789	-1.048551	0.294385	0.388202
7498	LYAR	230.0173361	-0.34581548	0.09559	-3.617688	0.000297	0.000777
12383	SLC6A9	121.1940673	-0.345850218	0.187851	-1.841089	0.065608	0.103851
9537	LAS1L	894.1243269	-0.346206031	0.123501	-2.803262	0.005059	0.010397
8340	ORMDL2	657.2111217	-0.346471779	0.105725	-3.277112	0.001049	0.002465
6210	NSFL1C	1843.35533	-0.346606225	0.083065	-4.172721	3.01E-05	9.50E-05
13138	CCNE2	57.03057366	-0.346648013	0.217047	-1.597109	0.110241	0.164473
9569	TPT1	88748.95563	-0.346652778	0.124113	-2.793033	0.005222	0.010696
6009	KARS	2960.72712	-0.346732706	0.081364	-4.261489	2.03E-05	6.62E-05
9752	STXBP2	1512.457734	-0.347145652	0.127073	-2.731869	0.006298	0.012658
10356	LOC401010	24.31423102	-0.347268103	0.137581	-2.524099	0.0116	0.021955
15020	PCDHB5	175.8899309	-0.347610493	0.347083	-1.00152	0.316576	0.413129
13580	SPDYA	15.17105226	-0.348432912	0.23847	-1.461119	0.143983	0.207821
15179	HES5	4.34272341	-0.349045842	0.366125	-0.953352	0.340412	0.439553
8105	IAH1	872.4962811	-0.349052473	0.10362	-3.368582	0.000756	0.001827
11490	SNRNP70	2464.270879	-0.349270041	0.164435	-2.124059	0.033665	0.05743
16148	SNORA81	1.017825108	-0.349327143	0.524285	-0.666292	0.505224	0.613244
11496	FZP686I15	70.25019342	-0.349466728	0.164801	-2.120533	0.033961	0.057905
15420	RIPPLY1	2.148558711	-0.349523864	0.395351	-0.884085	0.37665	0.478777
8879	ITSN1	919.8790814	-0.349585461	0.114211	-3.060871	0.002207	0.004872
11155	MTBP	53.94588102	-0.349600546	0.156332	-2.236273	0.025334	0.044515
11368	C3orf42	13.58867348	-0.349940495	0.161868	-2.161894	0.030626	0.052807
11716	ITPKC	1146.082225	-0.349952602	0.170876	-2.047997	0.04056	0.067858
15397	PRRT4	0.960178576	-0.350063602	0.392717	-0.891388	0.372721	0.474489
13737	AGAP6	110.4195065	-0.350181618	0.249195	-1.405253	0.159946	0.228223
13599	IED7-TICA	181.3142076	-0.350190745	0.240469	-1.456283	0.145314	0.20945
14251	KIAA1751	8.075801848	-0.350213645	0.282017	-1.241819	0.214303	0.294736
13448	NBPF16	86.54546317	-0.350250381	0.233177	-1.502077	0.133077	0.193965
9959	RABGGTA	466.6174858	-0.350272686	0.131273	-2.668286	0.007624	0.015005
11531	PPP4R1L	103.9645153	-0.350308334	0.166229	-2.107385	0.035084	0.059638
15543	KRT2	1.98865018	-0.350568235	0.413203	-0.848416	0.396206	0.499649
11784	CDKN2C	598.7627343	-0.350580988	0.172899	-2.027659	0.042595	0.070851
15763	DSC1	0.873579444	-0.350705742	0.446126	-0.786114	0.431801	0.536936
9390	POLR2J4	246.8955821	-0.350994561	0.122568	-2.863662	0.004188	0.008742
12315	ALKBH7	710.5044481	-0.351141296	0.188727	-1.86058	0.062804	0.09996
11102	HDAC10	511.9703954	-0.351167778	0.155785	-2.254188	0.024184	0.042698
7782	FBRS	1454.300773	-0.351300338	0.100179	-3.506733	0.000454	0.001143
12760	BIN1	1473.496244	-0.351394378	0.204812	-1.715692	0.086218	0.132443
12466	NID1	5621.041928	-0.351477303	0.193908	-1.812602	0.069893	0.109897

13799	FIGNL2	159.9794813	-0.351718178	0.253623	-1.386773	0.165511	0.235063
13782	ANG	602.5450629	-0.35189921	0.252649	-1.392839	0.163668	0.232772
7737	XRCC1	668.7163958	-0.351922051	0.099877	-3.52356	0.000426	0.001079
13235	CCNL2	1845.862407	-0.351952381	0.224484	-1.567829	0.116921	0.17316
12286	CBFA2T3	70.36710193	-0.35220509	0.188329	-1.870155	0.061462	0.098057
16263	SPATA21	0.824165829	-0.352541416	0.553922	-0.636446	0.524486	0.632137
10731	TRAPPC2L	844.7829856	-0.352862593	0.147819	-2.387131	0.01698	0.031016
9614	MRPL20	1129.11005	-0.352893651	0.12717	-2.774986	0.00552	0.011255
10714	HPS4	1286.341143	-0.352964723	0.147615	-2.391114	0.016797	0.03073
9963	FUT4	312.4987725	-0.352967104	0.132352	-2.666881	0.007656	0.015062
6717	UFD1L	1039.961881	-0.353376881	0.089829	-3.933872	8.36E-05	0.000244
13668	C11orf21	22.13039406	-0.35359902	0.246898	-1.432168	0.152096	0.218117
5422	PPME1	1187.405575	-0.353613511	0.077626	-4.555328	5.23E-06	1.89E-05
13463	PPP1R3G	118.5263581	-0.353867972	0.236248	-1.497867	0.134168	0.195337
10524	CYHR1	620.2278433	-0.353898373	0.143715	-2.462497	0.013797	0.025698
12517	B3GNT5	823.1930625	-0.354029758	0.196819	-1.798758	0.072057	0.112838
9635	SGTA	1708.303031	-0.354130653	0.127963	-2.767443	0.00565	0.011494
11306	EP400NL	87.5665349	-0.354159758	0.162705	-2.176696	0.029503	0.051149
9519	GINS3	117.4714323	-0.354246922	0.125923	-2.813204	0.004905	0.0101
7627	ITGB1BP1	1070.580624	-0.3544292	0.099328	-3.568263	0.000359	0.000923
9319	TMEM216	183.4470357	-0.3546813	0.122593	-2.893157	0.003814	0.008022
6061	C3orf37	704.2198137	-0.354784522	0.0837	-4.238783	2.25E-05	7.26E-05
13206	LCAT	144.554826	-0.354944054	0.224972	-1.577728	0.114628	0.170137
5671	ELOVL1	1527.692526	-0.354944253	0.080006	-4.436475	9.14E-06	3.16E-05
13435	MRPL41	654.1912812	-0.355135838	0.236098	-1.504186	0.132534	0.193356
11002	SRGAP1	533.6992559	-0.355231379	0.155219	-2.288579	0.022104	0.03938
9200	LYPLA2	1075.663897	-0.355443258	0.120979	-2.938067	0.003303	0.007036
7467	GNL2	1104.383065	-0.355627304	0.097991	-3.629196	0.000284	0.000746
7592	CCDC127	646.0457456	-0.355651384	0.099279	-3.582328	0.000341	0.000879
5771	UTP14A	381.5760186	-0.355711634	0.08112	-4.384988	1.16E-05	3.94E-05
10456	PRIC285	839.8446122	-0.355909191	0.142916	-2.490334	0.012762	0.023924
12178	AIM1	1974.902657	-0.356020431	0.187071	-1.903129	0.057024	0.091777
11017	LOC28583(	85.11740177	-0.35631207	0.155964	-2.284573	0.022338	0.039743
13586	RELL2	45.0420745	-0.356319071	0.244204	-1.459107	0.144536	0.208527
10476	TDRD3	559.5912382	-0.356479268	0.143615	-2.482186	0.013058	0.024432
8500	NSDHL	463.9480323	-0.356537019	0.11119	-3.206555	0.001343	0.003098
8152	CCDC77	125.1680816	-0.356717914	0.106501	-3.349439	0.00081	0.001947
5349	NVL	357.8882791	-0.356839223	0.077677	-4.59386	4.35E-06	1.59E-05
4195	VPS26B	1044.551166	-0.356857466	0.068458	-5.21282	1.86E-07	8.69E-07
5228	RNF216	1163.917151	-0.356937712	0.076515	-4.664917	3.09E-06	1.16E-05
12948	S1PR3	1328.408398	-0.356952641	0.215178	-1.658871	0.097142	0.147056
9979	RPP40	70.74714743	-0.356958305	0.134196	-2.659982	0.007814	0.015349
12135	APBB3	372.8505985	-0.356975744	0.186297	-1.916162	0.055344	0.089385
10071	ZFP36L1	7461.952589	-0.357096496	0.135893	-2.627774	0.008595	0.016727
13043	ATP2A3	822.3736067	-0.357177027	0.218845	-1.6321	0.102658	0.154275
9895	PPP1R3F	85.89681378	-0.357427954	0.132896	-2.689536	0.007155	0.014174
7357	ZNF526	236.5747961	-0.35775917	0.097342	-3.675272	0.000238	0.000633

14316	GOLGA8B	613.3660281	-0.357774673	0.291891	-1.225711	0.220307	0.301638
8892	WWC3	1660.332867	-0.357795881	0.117108	-3.055277	0.002249	0.004957
12654	CTAGE6	9.64346775	-0.357857803	0.203766	-1.756221	0.079051	0.122454
9698	AP1S1	978.2967729	-0.357921475	0.130302	-2.746852	0.006017	0.01216
11370	LOC22269	62.78354763	-0.358048754	0.16569	-2.160955	0.030699	0.052922
8876	POLR3D	339.8898254	-0.358182853	0.116977	-3.062001	0.002199	0.004855
14819	IL17REL	1.563811711	-0.358223874	0.338311	-1.058858	0.289664	0.383137
14312	FUT2	13.33685447	-0.358375136	0.292221	-1.226384	0.220054	0.301354
11957	FGFBP3	23.97746121	-0.358574898	0.18176	-1.972797	0.048519	0.079542
6332	CTBP1	2535.539148	-0.358728697	0.087228	-4.112544	3.91E-05	0.000121
9793	DUSP14	495.5031734	-0.358735954	0.131942	-2.718885	0.00655	0.013111
12925	EDNRA	751.5378491	-0.358753888	0.215344	-1.665957	0.095722	0.145172
12386	NBPF14	287.3557209	-0.359417052	0.19529	-1.84043	0.065705	0.103979
10160	RPSAP9	22.25779116	-0.359669092	0.13853	-2.596321	0.009423	0.018179
7320	TMEM109	2998.064195	-0.359687934	0.097428	-3.691815	0.000223	0.000596
10102	SPNS1	1034.873802	-0.359711266	0.1375	-2.616083	0.008894	0.017258
9303	ZNF26	278.1477694	-0.359726863	0.12404	-2.900087	0.003731	0.00786
12873	APLN	2132.201494	-0.359740788	0.213494	-1.685017	0.091985	0.140061
10242	C17orf44	68.85940533	-0.359901201	0.140054	-2.569732	0.010178	0.019478
9779	ZHX2	1309.508874	-0.360048354	0.132234	-2.722813	0.006473	0.012974
13981	OC1001322	24.03087588	-0.360075918	0.271964	-1.323986	0.185508	0.260077
7525	KEAP1	1510.282788	-0.360325711	0.099928	-3.605864	0.000311	0.00081
7111	ZNF746	426.5232406	-0.360456312	0.095345	-3.780543	0.000156	0.000431
9668	REXO1	675.245977	-0.360485006	0.130697	-2.758178	0.005812	0.011784
8737	PL-5283	1221.219629	-0.360604359	0.115823	-3.113412	0.001849	0.004149
13028	CYTIP	407.0158965	-0.360721976	0.220652	-1.634803	0.10209	0.153588
13565	CD109	2493.971811	-0.361042735	0.246356	-1.465532	0.142776	0.206307
13674	FBXO39	6.120645179	-0.361203822	0.252755	-1.429067	0.152985	0.219282
8195	C3orf26	308.4854543	-0.361301976	0.108367	-3.334071	0.000856	0.002047
15282	SEMA3C	2086.437638	-0.361308206	0.390434	-0.925401	0.354758	0.455019
4533	FAM104A	572.4331584	-0.361389057	0.072027	-5.017419	5.24E-07	2.26E-06
9826	KLHDC8B	448.5766575	-0.361409481	0.13336	-2.710036	0.006728	0.01342
4133	CASP2	809.0204135	-0.361590125	0.068724	-5.26146	1.43E-07	6.78E-07
10824	FAM3A	984.2322646	-0.361869387	0.153777	-2.353207	0.018612	0.033705
9714	MON1A	155.7597466	-0.36191892	0.131923	-2.743407	0.006081	0.012269
11555	ZAK	1498.219417	-0.362170178	0.172788	-2.09604	0.036079	0.061201
8090	CD3EAP	288.7288898	-0.36217063	0.107347	-3.373818	0.000741	0.001796
9843	C9orf114	318.3202401	-0.362220828	0.133849	-2.706192	0.006806	0.013553
12599	GIMAP2	325.6206958	-0.362230842	0.204233	-1.77362	0.076126	0.118434
10766	CENPN	255.3941874	-0.362531471	0.152557	-2.376362	0.017484	0.031833
10092	FBXO5	107.7211415	-0.362656483	0.138437	-2.619651	0.008802	0.017096
8108	C20orf196	69.85919303	-0.362715183	0.107699	-3.367858	0.000758	0.001831
14523	LOC12195	1.742053711	-0.363012968	0.31302	-1.159712	0.246166	0.332239
8856	CSNK1G2	1022.990348	-0.363122037	0.118363	-3.06787	0.002156	0.004772
14116	DLX6	6.735396362	-0.363405368	0.283463	-1.282021	0.199835	0.277484
14456	KIAA1984	25.24633896	-0.363426905	0.307801	-1.180722	0.237713	0.322317
14510	MAMDC2	49.34941066	-0.363539554	0.312475	-1.163418	0.24466	0.330502

8718	ZNF133	271.121814	-0.363541915	0.116399	-3.123228	0.001789	0.004022
14455	TMEM178	58.25034696	-0.363590419	0.307806	-1.181234	0.23751	0.322064
10330	RNF145	2809.313545	-0.363631604	0.143365	-2.536406	0.0112	0.021251
10377	RAI14	1695.39323	-0.364061602	0.144842	-2.513505	0.011954	0.022579
8304	UFC1	2163.318672	-0.364165374	0.110594	-3.292815	0.000992	0.002341
10672	BOLA3	357.8727498	-0.364224153	0.15148	-2.404445	0.016197	0.029749
14006	MAGIX	122.9321948	-0.364338973	0.276774	-1.316376	0.188048	0.263168
13378	KCNAB2	489.1041046	-0.36438203	0.240078	-1.517763	0.129074	0.189103
6769	MANBAL	1145.32244	-0.364391438	0.093168	-3.911105	9.19E-05	0.000266
7875	TM9SF4	2560.297782	-0.364509507	0.105287	-3.462066	0.000536	0.001334
7616	SLC35B1	1010.181322	-0.364586603	0.101986	-3.574858	0.00035	0.000902
13605	SCARNA17	21.20027165	-0.364641315	0.250648	-1.454795	0.145726	0.20995
9693	HSPBAP1	180.8460318	-0.364695176	0.13269	-2.748467	0.005987	0.012107
10104	PTPRE	753.0962733	-0.364785942	0.139507	-2.614824	0.008927	0.017318
10808	DENND3	960.2197104	-0.364981199	0.154703	-2.359245	0.018312	0.03321
10607	SLC39A3	366.5645548	-0.36551895	0.150155	-2.434271	0.014922	0.027574
10832	C7orf31	200.2995689	-0.365673461	0.155646	-2.349396	0.018804	0.034027
11961	PLXDC2	737.3193102	-0.365714763	0.185532	-1.971172	0.048704	0.079814
9556	ZNF598	511.5656871	-0.366203589	0.130836	-2.798949	0.005127	0.010516
7381	HEXA	3408.970975	-0.366801128	0.100129	-3.663294	0.000249	0.000661
9764	RPL17	5993.977631	-0.36686852	0.134582	-2.725977	0.006411	0.01287
10941	PQLC1	950.1031293	-0.367001092	0.159101	-2.306724	0.02107	0.037748
10145	CIDECP	88.68491858	-0.367155945	0.141207	-2.600134	0.009319	0.018005
6812	BTF3	8251.110802	-0.36732029	0.094322	-3.894321	9.85E-05	0.000283
8181	EIF3G	2167.538756	-0.367363105	0.109909	-3.34242	0.000831	0.00199
16476	KCNA7	0.444562975	-0.367383441	0.634905	-0.578643	0.56283	0.669582
5808	MRPL9	780.2993462	-0.367444831	0.084064	-4.371017	1.24E-05	4.17E-05
14697	KCNC1	3.893948689	-0.367486473	0.334309	-1.099243	0.271662	0.362308
10332	ZBTB48	339.6750835	-0.367513198	0.144925	-2.535883	0.011216	0.021279
13592	EFNB3	45.53686798	-0.367597157	0.252236	-1.457355	0.145019	0.209117
13779	RAB6B	728.8266291	-0.367698867	0.263881	-1.393429	0.16349	0.232569
10666	NHLRC1	50.29913139	-0.367888682	0.152928	-2.405631	0.016145	0.029669
13223	MAPK8IP3	1413.450517	-0.368138028	0.234031	-1.573034	0.115711	0.171523
11110	ADAMTS9	1068.091905	-0.368584604	0.163866	-2.24931	0.024493	0.043208
8631	RPL3	37621.6158	-0.369171178	0.116788	-3.161046	0.001572	0.00357
8054	NUCB1	9496.936936	-0.369195617	0.108865	-3.391321	0.000696	0.001693
11065	AFAP1	1063.547443	-0.36924397	0.162823	-2.267756	0.023344	0.041349
12532	LOC28299	89.48571117	-0.369370982	0.205762	-1.795133	0.072632	0.113603
8960	PSMB8	2990.207971	-0.369627463	0.12209	-3.027489	0.002466	0.005395
13045	RASGRP2	159.0528409	-0.369785757	0.226641	-1.631589	0.102766	0.154413
13150	HOXC8	154.1520309	-0.369839625	0.231889	-1.594902	0.110734	0.165057
10210	MYBBP1A	650.2753757	-0.370055028	0.143544	-2.577987	0.009938	0.019078
6606	SARS	2936.874908	-0.370416115	0.093045	-3.98105	6.86E-05	0.000204
17031	MOGAT2	0.556799407	-0.370503471	0.855255	-0.433208	0.664863	0.765192
13607	CCDC113	123.8376666	-0.370556307	0.254842	-1.454065	0.145928	0.210211
8399	NHP2L1	2140.724409	-0.370652085	0.1141	-3.248479	0.00116	0.002708
9736	SARS2	332.3783948	-0.370660471	0.135404	-2.73744	0.006192	0.012465

14418	ENPP6	14.46606954	-0.370733824	0.310893	-1.192481	0.233073	0.316858
9564	MVP	7298.704286	-0.370810628	0.132649	-2.795437	0.005183	0.010622
13551	LOXL4	249.0028884	-0.371140022	0.252344	-1.470769	0.141354	0.204463
8902	CUEDC2	992.2104292	-0.371460799	0.121747	-3.051097	0.00228	0.00502
6903	ZNF706	1023.599206	-0.37165269	0.096193	-3.863603	0.000112	0.000317
9255	TPST1	414.8519646	-0.371923712	0.127554	-2.915817	0.003548	0.007513
9801	DHX34	368.3817857	-0.372032074	0.136954	-2.716476	0.006598	0.013196
10499	POLRMT	746.1428153	-0.37216579	0.150571	-2.471702	0.013447	0.025105
6901	RABEPK	330.2424193	-0.372297453	0.096338	-3.864503	0.000111	0.000316
6866	THOC5	545.2194393	-0.372691035	0.096147	-3.876261	0.000106	0.000303
10477	TRAF3IP2	814.2964103	-0.372691511	0.150178	-2.481669	0.013077	0.024465
9139	USF2	2388.364644	-0.372691762	0.125833	-2.961798	0.003058	0.00656
10461	PDGFA	890.1668286	-0.372708741	0.14975	-2.488874	0.012815	0.024011
4685	ZNF212	276.3443733	-0.372709925	0.075497	-4.936777	7.94E-07	3.32E-06
9494	HLA-A	47056.03529	-0.372735568	0.132138	-2.820811	0.00479	0.00989
15857	UTS2	3.693369491	-0.372852486	0.493057	-0.756205	0.449526	0.555664
10139	TUBGCP2	1623.017435	-0.37286506	0.14327	-2.602526	0.009254	0.01789
10362	TMEM2	3065.614898	-0.37296335	0.14786	-2.522407	0.011655	0.022048
15698	IFIT1B	1.413529052	-0.373037372	0.462117	-0.807236	0.419531	0.523839
13911	GATA3	116.2363803	-0.373082523	0.276961	-1.347058	0.177961	0.250753
10896	ANO7	41.65863312	-0.373357175	0.160789	-2.322027	0.020231	0.036395
14361	RTN4RL2	91.04609036	-0.373469072	0.308518	-1.210525	0.226077	0.308568
5896	GTF2E2	477.7001074	-0.373893863	0.086489	-4.323007	1.54E-05	5.12E-05
8431	DGCR14	420.323968	-0.37397349	0.115565	-3.236052	0.001212	0.002817
12299	NEK6	2271.190957	-0.374011146	0.200403	-1.866294	0.062	0.09881
7647	CNP	1799.682394	-0.374121849	0.105045	-3.561538	0.000369	0.000945
8429	TCEB1	711.7330393	-0.374138919	0.115518	-3.238788	0.0012	0.002791
14875	MYOC	16.8880086	-0.374433524	0.358664	-1.043966	0.296501	0.390703
13654	BEST3	13.77516599	-0.374733518	0.260745	-1.437167	0.150671	0.216264
9408	CHD1L	698.8894558	-0.374782491	0.131279	-2.854847	0.004306	0.008971
15362	DCX	11.49778713	-0.374966722	0.415695	-0.902024	0.367044	0.468326
12327	MYADM	4024.12375	-0.375205695	0.202114	-1.856402	0.063396	0.100806
13034	ME1	314.0897852	-0.375253947	0.229714	-1.633571	0.102349	0.153916
13805	CSNK1A1L	1.854901602	-0.375302346	0.270829	-1.385753	0.165822	0.23544
7791	C12orf52	440.9904531	-0.375572804	0.107186	-3.503927	0.000458	0.001153
14442	RHOV	26.69827971	-0.375787009	0.317381	-1.184024	0.236404	0.320852
14847	WDR66	119.66936	-0.375873962	0.357052	-1.052714	0.292472	0.386122
10670	VWA5A	502.0893469	-0.375967364	0.156354	-2.404583	0.016191	0.029743
13056	EMP1	2486.115298	-0.375972332	0.230953	-1.627917	0.103543	0.155449
11282	U2AF1L4	149.0796476	-0.37614068	0.17219	-2.184451	0.028929	0.050261
6582	CRTAP	5498.088559	-0.376300156	0.094294	-3.99073	6.59E-05	0.000196
8635	EEFSEC	515.0602292	-0.376351326	0.119152	-3.158591	0.001585	0.003599
12750	IFI44	632.3486548	-0.376975439	0.219373	-1.718426	0.085719	0.131779
10615	CHST7	115.4371075	-0.376981003	0.155083	-2.430839	0.015064	0.027816
16266	CRNA001	0.515727328	-0.377013986	0.592622	-0.636179	0.524659	0.632239
9144	ATP6V0E1	4556.93429	-0.377054875	0.127362	-2.960503	0.003071	0.006584
14463	PPFIA4	465.7348976	-0.377165067	0.31997	-1.17875	0.238498	0.323224

12289	C11orf66	15.78588754	-0.377318702	0.201919	-1.86866	0.06167	0.098356
16201	MESTIT1	2.5028996	-0.377407692	0.577212	-0.653846	0.513211	0.62085
10849	C20orf117	271.1365917	-0.377482413	0.161332	-2.339787	0.019295	0.03486
8170	SCAMP3	1263.681611	-0.378181194	0.113041	-3.345533	0.000821	0.00197
12402	SEPX1	1333.884055	-0.378467566	0.206137	-1.835999	0.066358	0.104876
10598	ZEB2	1912.0002	-0.378516748	0.155348	-2.436579	0.014827	0.027422
10081	WDR91	1613.155264	-0.378580403	0.14421	-2.625202	0.00866	0.016838
7522	ALKBH3	319.1511914	-0.378730185	0.104992	-3.60723	0.000309	0.000806
14458	VANGL2	212.0152654	-0.378776596	0.321084	-1.179681	0.238127	0.322834
7296	ZNF18	197.2229886	-0.378893451	0.102379	-3.700874	0.000215	0.000577
9838	TEAD2	479.48261	-0.379064017	0.140017	-2.707267	0.006784	0.013516
9277	APBA3	291.478064	-0.379153265	0.130435	-2.90684	0.003651	0.007714
11548	PKN3	220.4953414	-0.379244082	0.180725	-2.098457	0.035865	0.060875
11520	DMPK	469.447543	-0.379250745	0.179698	-2.110485	0.034817	0.05924
8022	GGCX	855.3321765	-0.379283063	0.111476	-3.402377	0.000668	0.001632
14291	CCL28	203.5308365	-0.379397896	0.307983	-1.23188	0.217994	0.298971
7502	RNASEH2E	353.7955039	-0.379409255	0.104901	-3.616826	0.000298	0.000779
13367	MORN3	11.94581472	-0.379764099	0.249608	-1.521444	0.128149	0.187913
13827	C1orf106	639.5918864	-0.37994539	0.276121	-1.376012	0.168818	0.239315
13815	LOC64874C	16.82520322	-0.3799482	0.275069	-1.381285	0.167191	0.237214
6409	HARS	1524.980074	-0.380055511	0.093343	-4.071581	4.67E-05	0.000143
7764	DNM2	4288.693079	-0.380276619	0.108267	-3.512402	0.000444	0.001121
12338	FILIP1L	1273.452886	-0.380311297	0.20527	-1.852737	0.06392	0.101548
16414	SEMA3E	8.597474994	-0.380320988	0.635031	-0.598901	0.549239	0.655801
7012	SRRD	298.900359	-0.380481076	0.099651	-3.818147	0.000134	0.000376
7681	SIGMAR1	905.6728238	-0.380543885	0.107285	-3.547022	0.00039	0.000994
11364	HMGN3	2356.358563	-0.380654158	0.176008	-2.162705	0.030564	0.052718
12066	NUDT22	570.3445012	-0.380809804	0.196316	-1.939779	0.052407	0.085134
10402	DHX58	379.7289797	-0.380936032	0.151898	-2.507845	0.012147	0.022888
9640	PTTG1IP	13635.42719	-0.381068101	0.137754	-2.766287	0.00567	0.011529
12528	BTG3	433.870899	-0.381321848	0.212327	-1.795919	0.072507	0.113443
14001	BMP8B	86.26542187	-0.381465758	0.289443	-1.317928	0.187528	0.262533
12055	C12orf48	70.43229951	-0.381906569	0.196595	-1.942601	0.052064	0.084655
7584	RUSC1	694.0119639	-0.38191482	0.106532	-3.584965	0.000337	0.000871
14028	KIR2DL3	3.9398914	-0.381934747	0.291204	-1.311569	0.189666	0.265015
10915	SLC25A6	13409.52634	-0.381987988	0.165042	-2.314483	0.020641	0.037067
7062	AP4B1	286.1545227	-0.382127346	0.100637	-3.797101	0.000146	0.000406
10176	FAM113A	906.0516782	-0.382450172	0.147625	-2.590679	0.009579	0.01845
12619	PCDHB18	13.65041079	-0.382484999	0.216507	-1.766617	0.077292	0.120058
7162	SMYD5	818.3302404	-0.382592546	0.101753	-3.759996	0.00017	0.000465
7050	TMEM179E	942.0450351	-0.38270719	0.1007	-3.800487	0.000144	0.000402
11884	NDRG1	49502.9219	-0.382735184	0.191965	-1.993776	0.046177	0.076162
10605	NDUFS6	873.5551114	-0.382755557	0.157197	-2.434882	0.014897	0.027533
8937	C14orf153	519.8460706	-0.382801275	0.125959	-3.039096	0.002373	0.005204
12304	ABCG4	8.140950357	-0.382925339	0.205442	-1.863906	0.062335	0.099303
9051	RPL34	7657.481328	-0.382987778	0.127836	-2.995939	0.002736	0.005925
11797	NEURL1B	1283.594912	-0.383299077	0.189529	-2.022375	0.043138	0.071674

10960	SFRS16	424.1309533	-0.383299157	0.166624	-2.300385	0.021426	0.038322
14144	UPK3BL	166.7949209	-0.38347604	0.301113	-1.273529	0.20283	0.281086
10168	ZNF513	353.967008	-0.383619169	0.147918	-2.593466	0.009501	0.018316
11300	KCTD15	566.9693933	-0.383706171	0.176156	-2.178221	0.02939	0.050979
13000	CADM1	1097.500125	-0.383708201	0.233312	-1.644614	0.100049	0.150851
14939	OCA2	15.83871161	-0.383852691	0.374757	-1.024271	0.305707	0.401055
12242	TM4SF1	3199.751404	-0.384083133	0.203795	-1.884652	0.059477	0.09523
7869	CBLB	517.0734671	-0.384100187	0.110874	-3.464286	0.000532	0.001324
7850	KIAA1539	557.4669459	-0.384116813	0.110646	-3.471589	0.000517	0.001292
11439	TSNARE1	285.8088066	-0.384203228	0.179717	-2.137826	0.032531	0.055742
12999	BTBD19	54.30491043	-0.38427682	0.233608	-1.644961	0.099978	0.150755
11819	OR2A1	8.436510281	-0.384449356	0.190787	-2.015071	0.043897	0.072801
9712	CTSC	4925.494379	-0.384503577	0.14011	-2.744297	0.006064	0.012239
16035	SLITRK6	21.60481169	-0.384664449	0.548062	-0.701863	0.482765	0.590126
10860	IFFO2	684.5628716	-0.384761161	0.164697	-2.336174	0.019482	0.035163
15092	SCARNA1(	5.08532305	-0.384796747	0.393431	-0.978055	0.328047	0.426057
9436	PHKG2	466.1010049	-0.38482093	0.135323	-2.843717	0.004459	0.009263
15419	PHACTR3	26.6685137	-0.384851012	0.435311	-0.884083	0.376652	0.478777
6943	MYL6	14864.5553	-0.384864671	0.100027	-3.847596	0.000119	0.000337
12186	)C1002165	129.4726739	-0.385504628	0.202789	-1.901012	0.057301	0.092167
16739	NR5A1	0.675124683	-0.385589715	0.757173	-0.509249	0.610577	0.714911
5197	SARNP	847.7976644	-0.385840754	0.082465	-4.678856	2.88E-06	1.09E-05
15535	ASPDH	45.92242954	-0.385907347	0.454101	-0.849826	0.395422	0.498913
13090	FGD3	238.489209	-0.385952063	0.239102	-1.614174	0.10649	0.159458
9016	MSRB2	714.661827	-0.386041104	0.128394	-3.006686	0.002641	0.005742
13963	PRTG	51.52860726	-0.386369866	0.290331	-1.33079	0.183258	0.257258
16293	RXFP4	0.428616284	-0.386387544	0.614309	-0.628979	0.529363	0.636854
11894	NYNRIN	469.5244058	-0.386466103	0.19418	-1.990242	0.046564	0.076737
11123	LSR	1262.703831	-0.386479605	0.172183	-2.244585	0.024795	0.04369
7792	ALPK1	516.6292849	-0.38652871	0.110341	-3.503053	0.00046	0.001157
12662	H2AFY2	242.1573704	-0.386796972	0.220623	-1.753203	0.079567	0.123171
13269	GABBR1	392.3700848	-0.387020931	0.248722	-1.556037	0.119699	0.17682
8183	PRMT7	458.6155474	-0.387700327	0.116038	-3.341158	0.000834	0.001998
15797	SCARNA9I	0.563242318	-0.387717959	0.499545	-0.776143	0.437665	0.543057
10493	PPCDC	184.663518	-0.387943844	0.156833	-2.47361	0.013376	0.024986
13188	ZC3H12D	23.72983167	-0.388002623	0.245016	-1.583579	0.113289	0.168389
13840	DDR2	195.3969181	-0.388040591	0.28301	-1.371121	0.170337	0.241241
5020	PANK2	428.8415492	-0.388054774	0.081473	-4.762964	1.91E-06	7.45E-06
16103	XKR4	1.496043351	-0.388275921	0.570631	-0.680433	0.496231	0.604025
10591	ANKH	1389.198167	-0.388393185	0.159248	-2.438922	0.014731	0.027262
12255	C5orf25	213.0923045	-0.388612182	0.206582	-1.881157	0.059951	0.095887
8334	ATG9A	1465.662831	-0.388691077	0.118545	-3.278859	0.001042	0.002451
14809	SNORA53	2.791361781	-0.388918087	0.36593	-1.062822	0.287863	0.381012
14541	FAM154A	1.094731906	-0.389055314	0.337395	-1.153116	0.248863	0.335463
9518	MRPL52	504.4890168	-0.389109925	0.138299	-2.813543	0.0049	0.010091
9917	KCNMB3	55.69128449	-0.389143851	0.145138	-2.681193	0.007336	0.0145
11278	TUBA4A	1642.77246	-0.389167919	0.177931	-2.187187	0.028729	0.04993

6556	AAAS	721.780024	-0.389406664	0.097264	-4.003605	6.24E-05	0.000187
9665	MAST3	641.3631525	-0.38959474	0.141205	-2.759078	0.005796	0.011755
9441	RPL7A	14748.64377	-0.389663628	0.137151	-2.841123	0.004496	0.009333
16521	RFPL3	0.380971415	-0.389723178	0.688613	-0.565954	0.571425	0.677955
10175	ABTB1	553.5044902	-0.38984456	0.150476	-2.590734	0.009577	0.018449
12369	BNC2	621.284272	-0.389991838	0.211429	-1.844556	0.065102	0.103167
6607	PSMD2	3535.305573	-0.390006909	0.097969	-3.980909	6.87E-05	0.000204
13085	FAM13AOS	125.4626028	-0.390072771	0.2412	-1.617219	0.105831	0.158532
15693	TRPC5	0.730829251	-0.390120892	0.481991	-0.809394	0.418288	0.522454
9223	CCDC99	210.0098183	-0.390169095	0.133176	-2.929728	0.003393	0.00721
4759	ADPGK	1236.039176	-0.390225116	0.079553	-4.905212	9.33E-07	3.84E-06
6420	MKNK1	514.2909887	-0.390390262	0.095997	-4.066713	4.77E-05	0.000146
12188	TNFRSF10C	118.4925503	-0.390570741	0.205555	-1.900081	0.057422	0.092348
5741	NECAP2	1554.286096	-0.39060123	0.088749	-4.401202	1.08E-05	3.68E-05
10673	SHROOM1	291.9267653	-0.390619674	0.162476	-2.404168	0.016209	0.029769
10234	ARL16	491.7544717	-0.390621816	0.151933	-2.571008	0.01014	0.019422
8983	C22orf40	192.4256459	-0.390760657	0.129391	-3.019997	0.002528	0.005515
8752	MCOLN1	507.1790912	-0.39096621	0.125896	-3.105471	0.0019	0.004255
13504	SLC7A11	98.96827785	-0.391211597	0.263015	-1.487413	0.136906	0.198718
7169	LOC550643	454.1142484	-0.391213539	0.104154	-3.756094	0.000173	0.000472
9600	PSMG4	171.000814	-0.391477842	0.140834	-2.779719	0.005441	0.011108
11846	HYAL3	44.75989833	-0.391495712	0.195018	-2.007483	0.044698	0.07396
13397	TMEM176A	9135.513182	-0.391526142	0.258866	-1.512465	0.130416	0.19081
13450	C19orf73	8.216071385	-0.391593276	0.260743	-1.501839	0.133139	0.194026
9328	TMEM140	2648.668119	-0.391796647	0.135611	-2.889118	0.003863	0.008118
12125	LOC146880	302.1976195	-0.391798313	0.204128	-1.919379	0.054936	0.088809
8643	MUM1	705.3799094	-0.391828708	0.124249	-3.153567	0.001613	0.003658
7377	DNAJC2	594.5352978	-0.391905916	0.10688	-3.666796	0.000246	0.000653
8642	PIH1D1	893.2407683	-0.391924551	0.124264	-3.153968	0.001611	0.003653
10747	C17orf56	302.4345519	-0.392081041	0.164598	-2.382053	0.017216	0.0314
6709	CYBASC3	1027.061171	-0.392291838	0.099626	-3.937657	8.23E-05	0.00024
6211	RPN1	7665.397618	-0.392476604	0.094059	-4.172654	3.01E-05	9.50E-05
8549	TYSND1	466.2503108	-0.393442435	0.123342	-3.189844	0.001423	0.003264
13554	NPL	742.1178661	-0.393455773	0.267819	-1.469111	0.141803	0.205067
14784	LMTK3	39.403932	-0.393597281	0.366826	-1.072982	0.283279	0.375579
14965	HIST1H4C	0.809709711	-0.393996347	0.387084	-1.017858	0.308746	0.404392
17121	UTS2R	0.39245123	-0.394013208	0.971803	-0.405445	0.68515	0.784395
11880	SIDT1	66.6632703	-0.394027402	0.197371	-1.996382	0.045892	0.075719
11222	TMEM22	247.7563987	-0.394056324	0.178045	-2.213235	0.026881	0.046953
13968	NEK10	10.656065	-0.394104918	0.296638	-1.328573	0.183989	0.258188
12251	CTAGE4	64.80310587	-0.39413135	0.209437	-1.881862	0.059855	0.095765
12138	ZNF771	64.83462062	-0.394142764	0.2057	-1.916106	0.055352	0.089385
11554	NOS3	487.0321339	-0.394221575	0.188048	-2.096393	0.036047	0.061153
11639	P4HA1	3856.000916	-0.39423739	0.190929	-2.064833	0.038939	0.065576
14182	MSX2P1	1.480785616	-0.394242869	0.312258	-1.262555	0.206749	0.285749
5582	TRAFD1	886.7254252	-0.394413304	0.08801	-4.481481	7.41E-06	2.60E-05
11491	PCBP4	325.6247229	-0.394416643	0.185767	-2.123179	0.033739	0.057551

11829	SAP30	400.2184631	-0.394448094	0.196039	-2.012086	0.044211	0.073259
10004	MID1IP1	640.4811315	-0.394737007	0.148866	-2.651624	0.008011	0.015695
14579	HIST1H2AI	45.48586469	-0.394751267	0.345499	-1.142553	0.253224	0.340452
7867	C15orf63	741.6939223	-0.395185222	0.114066	-3.464531	0.000531	0.001323
9188	UBE2L6	2580.365564	-0.395369705	0.134319	-2.943517	0.003245	0.006923
11412	TNFAIP8L1	317.299929	-0.395468611	0.184405	-2.144568	0.031987	0.054941
9878	FAM127A	2040.56597	-0.395578898	0.146782	-2.695006	0.007039	0.013967
14002	DLX1	15.32415246	-0.395675814	0.300273	-1.317719	0.187598	0.262594
8842	VKORC1L1	994.4540696	-0.395684428	0.128712	-3.074182	0.002111	0.004679
10719	MRPS6	1020.23415	-0.395866666	0.16564	-2.389928	0.016852	0.030815
10623	GALNT7	528.1271952	-0.396064477	0.16325	-2.426122	0.015261	0.028159
9245	ARPC5L	607.283942	-0.396237091	0.135689	-2.920191	0.003498	0.007417
9882	HOXB7	399.6093121	-0.396275304	0.147107	-2.693785	0.007065	0.014013
10199	CDK5R1	89.66561212	-0.396275423	0.153462	-2.582231	0.009816	0.018866
10279	NPC2	5486.134818	-0.396429446	0.155307	-2.552546	0.010694	0.020392
13943	EME2	24.06942319	-0.396448375	0.296678	-1.336294	0.181453	0.255086
6182	GANAB	10705.01178	-0.396783629	0.094838	-4.183808	2.87E-05	9.09E-05
16350	CELF3	0.312289876	-0.39690682	0.646517	-0.613916	0.539271	0.646498
16821	IL17F	0.334808289	-0.396906868	0.817835	-0.485314	0.627453	0.731161
16573	DDX4	0.276698025	-0.396906875	0.716463	-0.553981	0.579592	0.685508
16869	P2RX2	0.316956304	-0.396906896	0.837948	-0.473665	0.635739	0.738699
16870	CRNA0002	0.21071838	-0.396906998	0.838798	-0.473185	0.636081	0.739053
17091	FAM55A	0.250677249	-0.396907032	0.955395	-0.415438	0.677821	0.777231
17077	BEND2	0.212157178	-0.396907077	0.946446	-0.419366	0.674949	0.774707
17319	NPY	0.278160363	-0.396907099	1.095401	-0.36234	0.717098	0.811606
17160	FNDC7	0.215614337	-0.396907122	1.002224	-0.396026	0.692085	0.790534
17508	TH	0.316035537	-0.396907137	1.264987	-0.313764	0.7537	0.843802
17142	PDE6H	0.176582397	-0.396907169	0.990554	-0.400692	0.688647	0.787433
17260	OR1G1	0.153794074	-0.396907267	1.058004	-0.375147	0.707551	0.803475
17462	HTR3E	0.212710611	-0.396907329	1.226874	-0.323511	0.746308	0.837727
17451	C3orf22	0.172433286	-0.396907403	1.21598	-0.32641	0.744114	0.835769
17454	ASB18	0.172903546	-0.396907409	1.221083	-0.325045	0.745147	0.836759
17554	MAGEA2	0.19717977	-0.39690747	1.314098	-0.302038	0.762623	0.851554
17453	OR2A14	0.136807716	-0.396907494	1.220125	-0.325301	0.744954	0.836637
17552	TBX20	0.159030276	-0.396907567	1.31222	-0.30247	0.762293	0.851439
17548	CGB2	0.154626814	-0.396907568	1.312849	-0.302325	0.762404	0.851439
17667	HIST3H3	0.132298642	-0.396907802	1.439657	-0.275696	0.782782	0.868423
17764	OR9A4	0.132805973	-0.396908001	1.564973	-0.25362	0.799789	0.882497
17782	CHRM2	0.096402637	-0.396908199	1.595887	-0.248707	0.803588	0.88579
17884	BRDT	0.11848563	-0.396908366	1.725985	-0.229961	0.818122	0.896644
17914	KRT83	0.095360259	-0.39690852	1.758942	-0.225652	0.821472	0.898848
17987	SNORA18	0.119055325	-0.396908748	1.9145	-0.207317	0.835762	0.910743
17991	C8B	0.116891796	-0.396908756	1.917879	-0.206952	0.836047	0.910865
18014	TAS2R38	0.096397851	-0.396908932	1.948879	-0.20366	0.838619	0.912703
18166	SNORA64	0.184004571	-0.396909237	2.319499	-0.171119	0.864131	0.932331
18104	SNORA68	0.100437047	-0.39690948	2.175662	-0.182432	0.855244	0.925963
18132	CLCA4	0.079582113	-0.396909764	2.230315	-0.177961	0.858753	0.928276

18191	KRT24	0.118988749	-0.396909961	2.417211	-0.164202	0.869572	0.936974
18342	CT45A3	0.241659523	-0.39691021	2.91407	-0.136205	0.891659	0.952033
18206	CST9	0.099100889	-0.396910283	2.471037	-0.160625	0.872389	0.939234
18233	NBPF6	0.078344969	-0.396910675	2.540992	-0.156203	0.875873	0.941374
18244	SLC32A1	0.074455731	-0.396910737	2.560765	-0.154997	0.876824	0.942042
18331	HBBP1	0.096533419	-0.396911463	2.849636	-0.139285	0.889225	0.950832
18336	GJA10	0.094311749	-0.396911499	2.860547	-0.138754	0.889645	0.951021
18345	GOLGA8F	0.097275291	-0.39691168	2.91407	-0.136205	0.891659	0.952033
18352	OR5AK2	0.097255838	-0.39691168	2.91407	-0.136205	0.891659	0.952033
18343	CT45A4	0.080519293	-0.396911925	2.91407	-0.136205	0.891659	0.952033
16119	:M189-UBI	4.289293432	-0.396912839	0.587614	-0.675465	0.49938	0.607256
16768	FLJ39609	0.436814294	-0.397124448	0.796416	-0.49864	0.618033	0.722452
12649	CRMP1	338.7108881	-0.397216559	0.226038	-1.757303	0.078866	0.122212
9543	TRPT1	492.2082254	-0.397267017	0.141756	-2.802469	0.005071	0.010416
11728	ACOT7	1072.337608	-0.397339197	0.194451	-2.043388	0.041014	0.068547
8143	STK4	1257.565049	-0.397351378	0.118402	-3.355949	0.000791	0.001904
10397	EEPD1	284.0625831	-0.397402262	0.158453	-2.508016	0.012141	0.022882
8133	UROD	1738.037928	-0.397454762	0.118326	-3.358972	0.000782	0.001885
11391	UQCR10	1627.988337	-0.397693194	0.184599	-2.154366	0.031212	0.053707
10707	ARRDC1	501.1702609	-0.3979053	0.166154	-2.394804	0.016629	0.030443
10278	FAM69A	236.3220488	-0.398033908	0.155877	-2.553516	0.010664	0.020337
8706	AGBL5	994.5809739	-0.398265575	0.127349	-3.127359	0.001764	0.003971
16645	MYADML	0.866455668	-0.398754031	0.743771	-0.536125	0.591872	0.696984
11207	ATP6V1F	3137.893561	-0.399057963	0.179791	-2.219569	0.026448	0.046257
7211	TRIM11	350.4004937	-0.399248338	0.106772	-3.739267	0.000185	0.000502
6789	H2AFZ	2322.703254	-0.399548175	0.102351	-3.903724	9.47E-05	0.000273
13673	SLC16A5	153.5468856	-0.399593375	0.279587	-1.429227	0.152939	0.219246
6224	NUB1	1930.233685	-0.399986873	0.096	-4.166548	3.09E-05	9.74E-05
13224	SULT1A1	773.3953014	-0.400275817	0.25447	-1.57298	0.115723	0.171529
10877	DOCK5	388.7406388	-0.400315867	0.171804	-2.330066	0.019803	0.035686
10821	NLRC3	189.2001401	-0.400329395	0.170039	-2.354341	0.018556	0.033611
7262	HDLBP	10569.98906	-0.400343609	0.107623	-3.719875	0.000199	0.000538
10250	ZNF643	30.08490493	-0.400482574	0.156104	-2.565489	0.010303	0.019702
11929	LRRC37A2	116.4921131	-0.400492608	0.202225	-1.98043	0.047655	0.078304
9286	TMEM87B	843.6925272	-0.400755597	0.13793	-2.905494	0.003667	0.00774
10930	FLVCR2	430.7480836	-0.400929618	0.173602	-2.309471	0.020917	0.037512
10561	LRRC37A	333.8898728	-0.401073575	0.163812	-2.448375	0.01435	0.026634
13348	NT5M	93.12703691	-0.401077575	0.262316	-1.528986	0.126268	0.185406
12995	C10orf47	199.4394621	-0.401492984	0.243893	-1.646183	0.099726	0.150419
12516	APC2	7.744393144	-0.401506689	0.223195	-1.798903	0.072034	0.112811
9946	CEP170	1080.441944	-0.401553734	0.150261	-2.67238	0.007532	0.014843
8367	ZNF251	310.7927402	-0.401629264	0.12302	-3.264754	0.001096	0.002567
10045	CHD3	1917.623252	-0.401638532	0.152368	-2.635969	0.00839	0.016371
12899	CFP	65.59960884	-0.401648029	0.240031	-1.673321	0.094264	0.143242
12967	STX1B	64.46724693	-0.401837688	0.243099	-1.652977	0.098336	0.148645
10255	C10orf35	153.0922575	-0.401863699	0.156747	-2.563765	0.010354	0.019791
10112	SPPL2B	973.0803514	-0.402487309	0.154017	-2.613266	0.008968	0.017384

5225	KCTD5	522.3903109	-0.40264667	0.086276	-4.666936	3.06E-06	1.15E-05
8553	THYN1	505.4320368	-0.402673903	0.126288	-3.188542	0.00143	0.003277
13465	NPAS3	43.06126399	-0.402746512	0.268946	-1.497501	0.134263	0.195447
7543	C19orf54	393.6917509	-0.402961927	0.111979	-3.598533	0.00032	0.000832
5265	PSMC2	1878.128807	-0.402998024	0.086774	-4.644206	3.41E-06	1.27E-05
16899	CCDC144N	0.595738036	-0.403057445	0.865459	-0.465715	0.641419	0.743977
10676	FKBP7	269.9510183	-0.403111109	0.167707	-2.403667	0.016232	0.029798
13916	44077	35.27792152	-0.403576902	0.299984	-1.345327	0.17852	0.251449
9743	FAM49B	1034.17938	-0.403724097	0.147659	-2.734172	0.006254	0.012581
11795	ZNF497	45.41874522	-0.403738932	0.19958	-2.022946	0.043079	0.071588
15837	SNAX-DISC	0.969735687	-0.403927147	0.529718	-0.762532	0.445742	0.551683
14851	ALOX12B	9.914048259	-0.404025276	0.384189	-1.051631	0.292969	0.386673
12025	PRRT3	44.61972053	-0.404052194	0.20676	-1.954212	0.050676	0.082596
8591	MYPOP	129.7822431	-0.404124394	0.127239	-3.176111	0.001493	0.003405
15538	PTTG2	0.820546114	-0.404311567	0.476125	-0.849172	0.395786	0.499279
10998	HBEGF	507.2818097	-0.404320249	0.176633	-2.289041	0.022077	0.039346
11668	FAM26E	168.0042209	-0.404377784	0.19648	-2.058108	0.03958	0.06649
7644	N6AMT2	133.2346048	-0.404783842	0.113599	-3.56326	0.000366	0.000939
13151	LRRC10B	55.40642245	-0.404957481	0.254037	-1.594088	0.110916	0.165316
4338	ANKLE2	1818.88829	-0.405007569	0.078834	-5.137445	2.78E-07	1.26E-06
16662	TERT	4.736015195	-0.405100262	0.763236	-0.530766	0.595581	0.700635
12282	LCA5L	87.08929067	-0.40512792	0.216461	-1.8716	0.061262	0.097769
13842	SEMA5B	3004.761459	-0.405129178	0.295606	-1.370506	0.170529	0.24147
6247	FBXO46	254.9383955	-0.405266888	0.097521	-4.155693	3.24E-05	0.000102
6414	NUP85	479.7921672	-0.405348502	0.099589	-4.070207	4.70E-05	0.000144
8838	RNASEH2C	746.3692702	-0.405369494	0.13184	-3.074715	0.002107	0.004673
5715	NAA38	326.679355	-0.405436494	0.091858	-4.413735	1.02E-05	3.48E-05
11403	GRAP	417.2547692	-0.405615968	0.188774	-2.148687	0.031659	0.05442
7816	CHCHD2	4166.545858	-0.40596113	0.116114	-3.496243	0.000472	0.001183
10779	C12orf61	19.06896332	-0.405977861	0.171292	-2.370086	0.017784	0.032339
5331	NUP93	651.5195927	-0.406022591	0.088114	-4.607948	4.07E-06	1.50E-05
5603	DUSP18	129.1155564	-0.406037739	0.090832	-4.470194	7.81E-06	2.73E-05
12085	FSTL1	13085.35793	-0.406189292	0.210202	-1.932381	0.053313	0.086469
15122	SCXB	1.527210697	-0.406296689	0.420285	-0.966716	0.333686	0.432521
9463	SERGEF	421.5330574	-0.406355204	0.143429	-2.833148	0.004609	0.009547
9630	CPOX	462.2699797	-0.406421473	0.146778	-2.768953	0.005624	0.011445
13644	CCDC136	17.88813196	-0.40643818	0.282221	-1.440141	0.149828	0.215243
6977	RCE1	247.1138261	-0.406540182	0.10602	-3.834544	0.000126	0.000353
11410	CD160	24.40314838	-0.406549581	0.189396	-2.146559	0.031828	0.054677
9392	GPAA1	2015.542433	-0.406643086	0.142104	-2.861594	0.004215	0.008796
5332	SAP30BP	1158.235933	-0.406809646	0.08831	-4.606635	4.09E-06	1.50E-05
4619	TPM3	6628.324239	-0.407091196	0.081736	-4.980568	6.34E-07	2.69E-06
14797	GRHL3	4.298823452	-0.407339092	0.381837	-1.066787	0.286068	0.378943
11133	CBLN3	147.8495131	-0.407346041	0.181732	-2.241469	0.024996	0.044
11032	FUT8	554.2634297	-0.407444597	0.178715	-2.279856	0.022616	0.040183
9187	KATNB1	370.8646896	-0.407646001	0.138467	-2.943991	0.00324	0.006913
9684	TIGD5	160.6885846	-0.407690019	0.14817	-2.751504	0.005932	0.012007

5552	SMARCAL[illegible]	598.4647541	-0.407752155	0.090641	-4.498531	6.84E-06	2.42E-05
9905	PSD4	812.3245749	-0.407813147	0.151806	-2.686416	0.007222	0.014291
13946	CD200R1	61.25266162	-0.407938442	0.305457	-1.335503	0.181712	0.255395
9396	GPS1	1630.281179	-0.408145288	0.1427	-2.860168	0.004234	0.008833
5278	PIAS3	681.5249924	-0.408201631	0.088025	-4.637344	3.53E-06	1.31E-05
9250	C17orf37	786.2201234	-0.408533279	0.140003	-2.91803	0.003523	0.007464
14204	ZNF883	65.15603464	-0.408773824	0.325672	-1.255169	0.209417	0.288988
13643	RPP25	250.5239738	-0.408887489	0.28385	-1.440506	0.149724	0.215121
10363	RHBDF1	495.9195116	-0.409483577	0.162357	-2.522116	0.011665	0.022064
11279	IRF1	1657.355549	-0.409577134	0.187367	-2.185962	0.028818	0.050082
5668	NADK	822.1913827	-0.409642358	0.09233	-4.436741	9.13E-06	3.16E-05
10974	RABEP2	456.6730483	-0.409713615	0.178502	-2.295284	0.021717	0.038789
16181	SNORD97	1.062009739	-0.409955439	0.621745	-0.659363	0.509663	0.617385
9064	JMJD8	1808.45215	-0.410187462	0.137059	-2.992782	0.002764	0.005978
9694	NFIX	1468.515128	-0.410273066	0.149274	-2.748455	0.005988	0.012107
10191	PLEKHH3	405.274759	-0.41028862	0.158679	-2.58565	0.00972	0.018694
12858	MYC	2329.790091	-0.410316117	0.242839	-1.689663	0.091092	0.138863
9281	PFDN4	373.8566408	-0.410398783	0.141208	-2.906339	0.003657	0.007723
10614	MLF1IP	180.6149333	-0.411024218	0.169035	-2.431592	0.015033	0.027761
9248	SEMA4C	826.4996434	-0.411191319	0.140836	-2.919655	0.003504	0.007427
13195	KCNA6	8.489865441	-0.411283583	0.260303	-1.580019	0.114103	0.169498
8427	ZNF32	643.5947091	-0.411303033	0.126963	-3.239552	0.001197	0.002785
13080	METRNL	638.6357392	-0.411339665	0.253941	-1.619822	0.105271	0.157753
8799	PLA2G15	989.4397637	-0.411445166	0.133295	-3.08672	0.002024	0.004508
16295	AQP7P3	0.814872559	-0.412019093	0.655354	-0.628697	0.529548	0.636984
13260	KLHL30	16.075416	-0.412019616	0.263995	-1.56071	0.118592	0.175304
7783	FCGRT	4236.240304	-0.412463176	0.11764	-3.506149	0.000455	0.001145
11031	GPX4	4656.596631	-0.412594433	0.180956	-2.28008	0.022603	0.040163
7871	HSPB11	343.3406843	-0.412653607	0.119124	-3.464078	0.000532	0.001325
12042	GYPE	9.380063679	-0.412831084	0.212082	-1.946559	0.051588	0.08397
9152	TSC22D4	1140.53567	-0.412900625	0.139625	-2.957207	0.003104	0.006649
8878	NOSIP	693.5246748	-0.413147419	0.134974	-3.06094	0.002206	0.004871
3671	ANAPC5	2322.072652	-0.413242504	0.074397	-5.554531	2.78E-08	1.49E-07
12783	PLCE1	355.4534089	-0.413318877	0.241806	-1.709297	0.087396	0.13401
8300	HSF1	1479.387082	-0.413523294	0.125559	-3.293464	0.00099	0.002337
8948	RFX1	373.3918593	-0.413699368	0.136368	-3.033691	0.002416	0.005292
10462	VILL	142.3240038	-0.413763601	0.166264	-2.488593	0.012825	0.024028
6169	MRPL18	777.2510777	-0.41400529	0.098791	-4.19073	2.78E-05	8.83E-05
14003	MST1P2	158.5945798	-0.414022603	0.314191	-1.31774	0.187591	0.262594
6909	DNAJB12	1416.538444	-0.414023946	0.10726	-3.860009	0.000113	0.000322
10737	FBXL15	241.7029807	-0.414154175	0.173634	-2.385216	0.017069	0.031161
14097	ABHD12B	4.628560182	-0.414206716	0.321288	-1.289206	0.197326	0.27437
12537	ZC3H12A	234.6237075	-0.414239547	0.230887	-1.794121	0.072794	0.113818
9341	PGD	1347.653534	-0.414403877	0.143779	-2.882229	0.003949	0.008286
9278	CCDC72	889.2963276	-0.414906523	0.142747	-2.906595	0.003654	0.007719
11146	NAB2	725.3332734	-0.415301031	0.185474	-2.239137	0.025147	0.044223
12434	SRGAP3	113.379394	-0.415362565	0.227434	-1.826297	0.067805	0.106889

13275	RGS18	131.0336638	-0.415496216	0.267666	-1.552295	0.120592	0.178058
13426	L1TD1	7.670095995	-0.415539296	0.275756	-1.506912	0.131833	0.192467
13569	NTN5	7.490594457	-0.415732544	0.28389	-1.464412	0.143081	0.206687
14912	PSAT1	777.8057717	-0.416025899	0.402815	-1.032797	0.301699	0.396587
14629	KCNK9	80.22054049	-0.416030581	0.370455	-1.123025	0.261427	0.350279
6941	EIF2B4	655.5867662	-0.416168055	0.108161	-3.847657	0.000119	0.000337
11413	WASH3P	412.5792419	-0.416457965	0.194214	-2.144328	0.032007	0.054969
10572	JUND	2392.430024	-0.416471537	0.170212	-2.446787	0.014414	0.026723
10924	GDF9	16.48295331	-0.416642649	0.180282	-2.311057	0.02083	0.037375
5487	LRRC59	2463.360516	-0.416644852	0.092002	-4.528654	5.94E-06	2.12E-05
9910	FAM46A	683.7584134	-0.416730875	0.155194	-2.68522	0.007248	0.014336
13241	GAPT	120.5688932	-0.416838293	0.266162	-1.566107	0.117324	0.173677
8766	DND1	66.57868372	-0.417071998	0.134646	-3.097548	0.001951	0.004363
13638	FGFR4	1173.471574	-0.417094179	0.289235	-1.442059	0.149286	0.214558
8931	WDR4	135.9138926	-0.417273823	0.137225	-3.040811	0.002359	0.005178
12113	OBSCN	728.4771999	-0.417307032	0.216904	-1.923927	0.054364	0.08797
5416	C11orf17	384.9928731	-0.417390498	0.091591	-4.557134	5.19E-06	1.88E-05
6486	PARK7	3527.002935	-0.417412132	0.103477	-4.033855	5.49E-05	0.000166
14141	DLEU7	5.377418832	-0.417548552	0.327535	-1.274822	0.202372	0.28051
3903	RAB8A	1594.69646	-0.417655605	0.077308	-5.402519	6.57E-08	3.30E-07
9344	PVR	1344.452358	-0.417673319	0.145017	-2.88016	0.003975	0.008338
14743	KLRC4	5.939052328	-0.417824104	0.384625	-1.086316	0.277339	0.368726
11637	TET1	46.17927604	-0.41797182	0.202323	-2.06586	0.038842	0.065424
6849	ARFRP1	710.678103	-0.418080838	0.107762	-3.879663	0.000105	0.000299
9971	WDR81	1389.142061	-0.418122504	0.157029	-2.662703	0.007752	0.015238
17076	C3P1	0.907119372	-0.418162257	0.997	-0.41942	0.674909	0.774707
8121	NPRL3	966.3500651	-0.418210536	0.124372	-3.362573	0.000772	0.001864
11588	ARRDC3	6098.237137	-0.418368733	0.20069	-2.08465	0.037101	0.062756
7345	PRPF6	2772.853098	-0.418716354	0.113666	-3.683728	0.00023	0.000613
15290	CREB3L3	468.8675563	-0.418823603	0.453584	-0.923365	0.355817	0.45614
13338	PLAC9	142.4359745	-0.41903038	0.273537	-1.531898	0.125548	0.1845
6102	RNF135	846.0317074	-0.419154102	0.099297	-4.221198	2.43E-05	7.81E-05
8700	PRICKLE3	143.2146694	-0.419278363	0.133933	-3.130519	0.001745	0.003931
8176	RRP8	330.6494671	-0.419816685	0.125568	-3.343329	0.000828	0.001984
12417	ANKRD36	179.4286794	-0.419820223	0.229327	-1.83066	0.067151	0.106002
13459	CECR4	3.018662501	-0.420581756	0.280529	-1.499247	0.13381	0.194873
7491	MRPL11	722.4019738	-0.420837539	0.116268	-3.619558	0.000295	0.000772
12343	SNORA7B	6.207964931	-0.420854327	0.227455	-1.850273	0.064274	0.102069
11833	PLK2	2051.520215	-0.420900363	0.209386	-2.010163	0.044414	0.07357
9728	ST3GAL5	365.1219543	-0.420923368	0.1537	-2.738609	0.00617	0.012432
5218	RNF220	1032.690758	-0.421041126	0.090127	-4.671625	2.99E-06	1.12E-05
4895	ASCC2	1234.330301	-0.421069753	0.087193	-4.829177	1.37E-06	5.49E-06
8603	HINT1	5859.545956	-0.421178926	0.132687	-3.174232	0.001502	0.003423
10198	MRPL38	932.9855924	-0.421308202	0.163153	-2.582291	0.009815	0.018864
8583	TUBG1	648.2261726	-0.421516721	0.132576	-3.17944	0.001476	0.00337
6545	PDZD11	883.2376959	-0.421538052	0.105201	-4.006995	6.15E-05	0.000184
3927	GRB2	3145.311905	-0.421811115	0.078328	-5.385202	7.24E-08	3.61E-07

9870	DOPEY2	829.8745693	-0.422450521	0.156665	-2.696521	0.007007	0.013914
15149	XG	10.79275419	-0.422722769	0.440137	-0.960434	0.336837	0.435827
9489	JMJD4	459.0122428	-0.422769107	0.149827	-2.821715	0.004777	0.009867
14691	OLFM1	246.8204636	-0.422831893	0.383397	-1.102856	0.27009	0.360359
4332	RAC1	6996.119399	-0.422928941	0.082271	-5.140676	2.74E-07	1.24E-06
4084	CCT5	3696.829992	-0.423010616	0.079963	-5.290106	1.22E-07	5.87E-07
8107	TTC15	814.1607324	-0.42324918	0.125657	-3.368292	0.000756	0.001829
8265	MRPL40	929.8509853	-0.423280739	0.128044	-3.305746	0.000947	0.002246
10874	APOL3	983.3491157	-0.423394832	0.181619	-2.33123	0.019741	0.035585
15070	PKHD1L1	5.323997728	-0.423577823	0.429753	-0.98563	0.324315	0.421824
11191	OBFC2A	416.6801835	-0.423745018	0.190474	-2.224692	0.026102	0.045717
5994	C8orf76	292.303747	-0.423771033	0.099241	-4.270112	1.95E-05	6.39E-05
9070	ARHGEF6	1108.596948	-0.424367124	0.141906	-2.990478	0.002785	0.006019
5897	PSMC1	520.3861883	-0.424393121	0.098177	-4.322727	1.54E-05	5.12E-05
12648	ZFP36	7627.679453	-0.42467557	0.241588	-1.757851	0.078773	0.122077
11390	HSPA1A	8532.137066	-0.424767605	0.197066	-2.155456	0.031126	0.053565
8621	LDLRAP1	421.5257314	-0.424793244	0.134138	-3.166844	0.001541	0.003504
3658	RNF130	2584.644212	-0.424978457	0.076363	-5.56523	2.62E-08	1.40E-07
9561	CDK11A	419.4539537	-0.425055443	0.15201	-2.796234	0.00517	0.010599
8419	NAT9	461.272182	-0.425416628	0.131181	-3.242962	0.001183	0.002754
9004	OC1001309	47.38693729	-0.425580243	0.141396	-3.00985	0.002614	0.00569
9069	TAP1	3761.690796	-0.425658302	0.142306	-2.991155	0.002779	0.006007
14180	BDKRB2	716.726501	-0.4258514	0.337171	-1.263013	0.206584	0.285561
12166	ANO1	1006.748374	-0.425915684	0.223296	-1.907403	0.056468	0.090978
9078	METTL12	46.14566705	-0.425921156	0.142588	-2.98707	0.002817	0.006082
11420	LIPA	4786.906757	-0.425987582	0.198849	-2.142272	0.032172	0.055219
5910	PSMD8	2924.602024	-0.426045746	0.09869	-4.31699	1.58E-05	5.25E-05
11753	CTSD	50743.43636	-0.426165179	0.209278	-2.036359	0.041714	0.069557
11598	PALLD	2701.451168	-0.426681213	0.205047	-2.080894	0.037444	0.063281
7614	ARMC7	354.3177691	-0.426695023	0.119329	-3.575774	0.000349	0.000899
7520	RBPJ	902.0135877	-0.42680817	0.118305	-3.607706	0.000309	0.000805
9288	SRXN1	1368.631088	-0.426851335	0.146974	-2.90426	0.003681	0.007769
8800	CHCHD1	475.6055082	-0.426967011	0.138336	-3.086459	0.002026	0.004511
6323	TP53	1011.986643	-0.427123294	0.103778	-4.115752	3.86E-05	0.00012
16241	PHF21B	5.358493552	-0.427381769	0.664413	-0.643247	0.520064	0.627656
5007	TBC1D10B	1212.878024	-0.427576503	0.089656	-4.769087	1.85E-06	7.24E-06
7604	DCAF15	390.1689431	-0.427667154	0.11951	-3.578511	0.000346	0.000891
6780	ZNRD1	346.768245	-0.427808679	0.109483	-3.90753	9.32E-05	0.00027
14104	COL9A2	95.99565641	-0.427932158	0.332263	-1.287931	0.19777	0.27485
7429	PES1	1629.316277	-0.428088802	0.117454	-3.644734	0.000268	0.000706
5697	SSH1	1252.336629	-0.428165663	0.096839	-4.421437	9.80E-06	3.37E-05
10495	KLHDC4	409.5796429	-0.428320663	0.173208	-2.472866	0.013403	0.025033
7495	PPP1R9B	1764.112353	-0.428338726	0.11835	-3.619252	0.000295	0.000773
6968	CHMP1A	2217.702143	-0.428379188	0.111549	-3.840278	0.000123	0.000346
9015	LZTS2	1405.878887	-0.428532457	0.142518	-3.006863	0.00264	0.005739
6772	NSMAF	719.1846493	-0.428610505	0.109635	-3.909441	9.25E-05	0.000268
11533	LOC339047	869.9973312	-0.428620115	0.203429	-2.106975	0.03512	0.059688

12987	PACSIN3	226.6508856	-0.428634385	0.259971	-1.648776	0.099193	0.149699
13239	TMEM200E	361.5132188	-0.428905388	0.27371	-1.567007	0.117113	0.173392
7497	SSBP1	1336.156071	-0.428922344	0.118542	-3.61831	0.000297	0.000775
9746	FAM118A	275.2820883	-0.428982855	0.156927	-2.733641	0.006264	0.012598
12864	SNORA39	6.204087377	-0.429111659	0.254121	-1.68861	0.091294	0.139106
12134	FITM1	5.722412498	-0.429443028	0.224064	-1.916608	0.055288	0.089311
9701	ARID3A	80.64728822	-0.429682868	0.156482	-2.745899	0.006035	0.01219
7513	SKP2	88.92300525	-0.42976178	0.118978	-3.612113	0.000304	0.000792
6836	PTRH2	321.644913	-0.429816347	0.110606	-3.886005	0.000102	0.000292
5081	MAN2B2	1419.109287	-0.429839163	0.090845	-4.731583	2.23E-06	8.59E-06
9675	C16orf55	147.4743662	-0.429902656	0.156025	-2.755349	0.005863	0.011878
7963	SSR3	4069.431071	-0.429937635	0.125593	-3.423263	0.000619	0.001523
7052	FEN1	374.0883726	-0.429972967	0.113147	-3.800133	0.000145	0.000402
8640	MRPL28	1119.032552	-0.430087431	0.136299	-3.155479	0.001602	0.003635
7909	ERP29	3106.953096	-0.430296709	0.124914	-3.444731	0.000572	0.001417
8659	LMO4	684.2355063	-0.43061832	0.136815	-3.147451	0.001647	0.003728
14694	UPP2	20.9386734	-0.430897618	0.391075	-1.101829	0.270536	0.360881
4899	SNRPA1	348.7220207	-0.430900516	0.089272	-4.826808	1.39E-06	5.55E-06
8116	ATP13A2	884.2288362	-0.431095662	0.128167	-3.36354	0.000769	0.001858
6520	PEA15	5695.087075	-0.431223625	0.107319	-4.018135	5.87E-05	0.000176
12453	KIFC2	403.4300275	-0.431642951	0.237235	-1.819474	0.068839	0.108353
4733	HDAC3	1064.668453	-0.432033174	0.087857	-4.917448	8.77E-07	3.63E-06
8341	ZNF511	450.4202514	-0.432246564	0.131903	-3.276995	0.001049	0.002466
7776	IL13RA1	3088.33897	-0.432435937	0.123253	-3.508511	0.000451	0.001136
8834	CCDC61	130.7945849	-0.432516237	0.140573	-3.076807	0.002092	0.004642
7179	ARPC5	4108.442329	-0.432796075	0.115341	-3.752311	0.000175	0.000478
13208	LOC400759	30.76382099	-0.432901997	0.274437	-1.577417	0.1147	0.170217
13014	SCN11A	9.633986726	-0.433002788	0.264067	-1.639745	0.101058	0.152208
15039	EPHA6	38.31919746	-0.433123696	0.435377	-0.994823	0.319822	0.416839
15507	LMOD2	0.486135355	-0.433129008	0.505068	-0.857566	0.391132	0.494395
7938	PRR4	34.32432921	-0.433169554	0.126189	-3.432708	0.000598	0.001476
7700	SRC	1190.431924	-0.433266324	0.122383	-3.540262	0.0004	0.001018
16408	FGL1	2.812881996	-0.433332454	0.722573	-0.599708	0.548701	0.655478
15258	ABP1	7397.890938	-0.433389817	0.465337	-0.931345	0.351675	0.451775
12470	CORIN	27.98542123	-0.43339706	0.239232	-1.811618	0.070045	0.110101
13248	CHRFAM7A	34.27299657	-0.433550552	0.277394	-1.56294	0.118067	0.174672
5912	PSMC4	1657.43388	-0.433703846	0.100478	-4.316394	1.59E-05	5.26E-05
2590	LASS5	939.295005	-0.433740924	0.06785	-6.392662	1.63E-10	1.23E-09
10194	LOC728554	190.4855563	-0.433938516	0.16786	-2.585123	0.009734	0.018717
11535	CYGB	852.9240612	-0.434070423	0.206183	-2.105267	0.035268	0.05993
5274	LASP1	6008.690361	-0.434384354	0.093634	-4.639156	3.50E-06	1.30E-05
11934	CKS2	343.8383229	-0.434481555	0.219549	-1.978971	0.047819	0.078541
10768	PTPLAD2	312.5653278	-0.434500624	0.182871	-2.375992	0.017502	0.031859
11067	PLEKHG5	301.4395479	-0.434575621	0.191635	-2.267725	0.023346	0.041349
13914	XKR9	33.75480402	-0.434624673	0.322832	-1.346286	0.17821	0.251049
8114	MED25	728.3286498	-0.434630124	0.129214	-3.363636	0.000769	0.001858
12492	DNAH1	247.3319944	-0.434716202	0.240675	-1.806241	0.070881	0.111213

9457	EPB41L2	1779.830544	-0.434851828	0.153366	-2.835379	0.004577	0.009487
10863	NFKBIE	869.189529	-0.434998854	0.186273	-2.335282	0.019529	0.035237
7727	CSNK1E	1963.724581	-0.43523544	0.123315	-3.529457	0.000416	0.001056
14929	C9orf50	10.81639232	-0.43559468	0.424619	-1.025848	0.304963	0.400401
8295	LMF2	1929.965747	-0.436124954	0.132348	-3.295291	0.000983	0.002323
13573	RNF126P1	1.686070111	-0.436138564	0.298106	-1.46303	0.143459	0.207172
12284	C2orf52	6.025173924	-0.436260597	0.233241	-1.870427	0.061425	0.098012
8782	ZBTB17	433.5348759	-0.436273403	0.141092	-3.092112	0.001987	0.004436
7156	CSNK2B	2637.302297	-0.436486755	0.116018	-3.762234	0.000168	0.000461
4152	ZNF438	233.9999076	-0.436849543	0.0832	-5.250578	1.52E-07	7.16E-07
8066	CENPL	106.7395302	-0.436865538	0.129017	-3.38612	0.000709	0.001723
8031	HDAC7	1771.661852	-0.436949623	0.128553	-3.398988	0.000676	0.001651
9851	MUTYH	132.5959694	-0.436957439	0.161607	-2.703833	0.006854	0.013639
9762	DVL1	909.392856	-0.437086461	0.160281	-2.727007	0.006391	0.012833
9690	C21orf82	6.688001927	-0.437236662	0.159052	-2.749018	0.005977	0.012091
13559	DGCR10	29.75406043	-0.4376257	0.2982	-1.467557	0.142225	0.205601
5622	RAVER1	1250.389611	-0.437839271	0.098141	-4.461317	8.15E-06	2.84E-05
11571	AMIGO2	306.9481996	-0.43803311	0.20937	-2.092146	0.036425	0.061704
7059	ZC3HC1	752.1832987	-0.438338302	0.115423	-3.797684	0.000146	0.000406
7717	MAPK7	441.7988082	-0.438755487	0.124159	-3.533824	0.00041	0.00104
12841	IL12A	4.910264005	-0.43891181	0.259276	-1.692837	0.090486	0.138122
6317	FLOT1	4361.700161	-0.439010095	0.106611	-4.117867	3.82E-05	0.000119
12478	PCSK4	76.33781906	-0.43906181	0.242719	-1.80893	0.070462	0.110685
8400	PPIAL4G	26.8669643	-0.439212019	0.135219	-3.248158	0.001162	0.00271
9662	CNRIP1	293.6751467	-0.439250092	0.159126	-2.760384	0.005773	0.011712
4414	NUDCD3	2457.788017	-0.439470006	0.086284	-5.093269	3.52E-07	1.56E-06
7581	C16orf42	870.4337501	-0.439822237	0.122672	-3.585343	0.000337	0.00087
8261	C21orf57	199.8544106	-0.439979961	0.133014	-3.307763	0.00094	0.002231
13251	HOXC13	5.066410237	-0.440008394	0.281556	-1.562772	0.118106	0.174704
7702	RPL5	19241.27835	-0.440045787	0.124318	-3.539677	0.000401	0.00102
6729	AMZ2P1	178.2870665	-0.44037657	0.112045	-3.930358	8.48E-05	0.000247
6840	EXOC4	1714.375303	-0.440432434	0.113391	-3.884206	0.000103	0.000294
9074	SSBP2	345.3335295	-0.440802166	0.147544	-2.987599	0.002812	0.006074
6533	KDM4B	1258.85407	-0.441009648	0.10995	-4.011017	6.05E-05	0.000181
13194	GRAPL	7.874280006	-0.441019132	0.278928	-1.58112	0.113851	0.169136
9474	SHMT2	4174.951154	-0.441122617	0.155943	-2.828747	0.004673	0.009668
7744	MAP1S	752.9818545	-0.441268181	0.125289	-3.522005	0.000428	0.001084
7452	TMCO6	198.2465204	-0.441274183	0.121339	-3.636701	0.000276	0.000726
10437	MTMR4	1234.965425	-0.441427188	0.176699	-2.498182	0.012483	0.023444
10911	LPIN3	937.4207	-0.441805059	0.19064	-2.317485	0.020477	0.036786
11725	CAMK4	20.81039799	-0.441833054	0.216049	-2.045057	0.040849	0.068289
11134	KLHL29	234.5781493	-0.44185218	0.197125	-2.241485	0.024995	0.044
7669	GIT1	1496.81334	-0.442178781	0.124506	-3.551478	0.000383	0.000979
8401	PPP2R5B	546.7633572	-0.442311309	0.136176	-3.248075	0.001162	0.002711
8889	COPE	2482.825468	-0.442431769	0.144762	-3.056269	0.002241	0.004942
8134	SMPD2	217.1553771	-0.442455581	0.131743	-3.358466	0.000784	0.001889
6906	PRR14	651.0370264	-0.442476215	0.114578	-3.861794	0.000113	0.000319

11647	MBLAC1	46.96339951	-0.442685743	0.214704	-2.061847	0.039222	0.066008
14877	C7	2037.996329	-0.442734271	0.4243	-1.043447	0.296741	0.390968
16268	KIAA1239	0.634666521	-0.442760096	0.697493	-0.634788	0.525567	0.633245
8520	MYEOV2	523.9481394	-0.44280245	0.138465	-3.197937	0.001384	0.003184
14870	TNNI1	15.21999609	-0.442993653	0.423615	-1.045745	0.295679	0.389751
6882	PARP6	826.6050043	-0.443105998	0.114476	-3.870748	0.000109	0.000309
5656	FAM136A	745.6437375	-0.443214475	0.099721	-4.444567	8.81E-06	3.05E-05
5186	C17orf95	440.5427378	-0.443235728	0.094664	-4.682184	2.84E-06	1.07E-05
13374	NACAD	135.7596237	-0.443372453	0.291879	-1.519027	0.128756	0.188705
8014	CPSF3L	1321.299714	-0.443447075	0.130214	-3.405533	0.00066	0.001615
7930	TBRG4	1161.827424	-0.443477918	0.129087	-3.435494	0.000591	0.001462
13387	NSUN5P1	161.3153664	-0.44352394	0.292731	-1.515127	0.12974	0.189963
16234	UTY	195.0160815	-0.443659391	0.687744	-0.645094	0.518867	0.626482
14716	GPR62	3.959955266	-0.44367633	0.406036	-1.092702	0.274525	0.365654
8171	CAPZA1	3185.008545	-0.443977501	0.13272	-3.345208	0.000822	0.001972
3664	NAGA	1326.875564	-0.444264088	0.079896	-5.56056	2.69E-08	1.44E-07
9042	RFTN1	1188.369388	-0.444408653	0.148196	-2.99879	0.002711	0.005876
9960	BRCA1	155.0849915	-0.444434844	0.166585	-2.667914	0.007632	0.01502
16307	SLITRK1	0.373169827	-0.444506586	0.710193	-0.625895	0.531384	0.638723
9461	FAM114A1	553.4127259	-0.444510932	0.156869	-2.833635	0.004602	0.009535
14332	ARHGEF4	10.79152278	-0.444684411	0.364091	-1.221356	0.221951	0.303549
6967	INTS3	2264.533055	-0.444712785	0.115796	-3.840482	0.000123	0.000345
8850	NOL12	261.2716515	-0.444822534	0.144884	-3.070199	0.002139	0.004738
11058	GCET2	22.10767569	-0.444981345	0.195896	-2.271519	0.023116	0.040974
13417	C7orf68	4644.037993	-0.445127109	0.294988	-1.508967	0.131307	0.191828
12153	ECSCR	270.4811694	-0.445155181	0.233056	-1.910078	0.056123	0.090524
10328	FEZ1	210.999463	-0.445197329	0.175458	-2.537337	0.01117	0.021199
12544	DDX12	80.80972519	-0.445281993	0.248417	-1.792479	0.073056	0.114156
6853	PHB2	3569.253525	-0.44536555	0.11481	-3.879167	0.000105	0.0003
14081	ADAMTSL5	33.81283935	-0.44554782	0.344518	-1.293249	0.195925	0.272731
14999	KISS1	5.346537294	-0.445553663	0.4423	-1.007357	0.313763	0.410032
11650	SIGLECP3	9.372098277	-0.445602717	0.216204	-2.061028	0.0393	0.066122
13641	BAIAP2L1	585.8334529	-0.445624059	0.309319	-1.440661	0.149681	0.215079
10424	PHLDB3	147.403916	-0.445833603	0.178165	-2.502363	0.012337	0.023198
8802	RRP12	632.0501958	-0.445855011	0.144503	-3.085433	0.002033	0.004526
12283	APBB1IP	1267.04456	-0.446104105	0.238385	-1.871358	0.061296	0.097814
11090	TBX6	51.54643548	-0.446489782	0.197591	-2.259661	0.023842	0.04214
5663	ZDHHC16	550.0829952	-0.446836413	0.100605	-4.441496	8.93E-06	3.09E-05
13990	TMIE	28.95889371	-0.446925585	0.338272	-1.321202	0.186434	0.261208
8150	NDUFAF2	182.2677028	-0.447063376	0.133366	-3.352162	0.000802	0.001928
10072	CCDC157	39.29381038	-0.447194343	0.170203	-2.627413	0.008604	0.016744
14364	GDF7	3.881138454	-0.447480751	0.370123	-1.209006	0.226661	0.309299
8392	RNF149	2033.668567	-0.447531882	0.137605	-3.252297	0.001145	0.002674
8978	ZNF337	611.274301	-0.447554027	0.148159	-3.02077	0.002521	0.005505
11080	STAT1	6734.582508	-0.447607031	0.197797	-2.262958	0.023638	0.041814
9555	FUCA1	2185.591583	-0.447690935	0.159911	-2.799618	0.005116	0.010496
4825	GOLGA3	1703.080844	-0.448471932	0.09195	-4.877327	1.08E-06	4.37E-06

10130	TNFAIP3	1611.003788	-0.448595017	0.172161	-2.605679	0.009169	0.017743
6896	SGSH	901.2154608	-0.448692735	0.116073	-3.865609	0.000111	0.000315
15425	CALML3	4.206074054	-0.448768211	0.508556	-0.882436	0.377541	0.479753
6823	CEP164	441.0378445	-0.449297023	0.115494	-3.890206	0.0001	0.000288
7943	COMMD6	1360.21694	-0.449403391	0.130999	-3.430576	0.000602	0.001486
2575	SFRS9	2068.371219	-0.449442066	0.070144	-6.407462	1.48E-10	1.13E-09
14303	ATP1A3	38.27135067	-0.449541515	0.365748	-1.229101	0.219034	0.300166
8277	NFKBIL1	416.4455414	-0.449789511	0.136233	-3.301612	0.000961	0.002277
12479	CAMK1G	54.09482956	-0.449862305	0.248701	-1.80885	0.070474	0.11069
9191	ARHGAP25	615.7000573	-0.450227853	0.153023	-2.942219	0.003259	0.00695
8738	SNRNP35	459.771338	-0.450312167	0.144649	-3.113138	0.001851	0.004152
10995	BATF2	221.6957584	-0.450487885	0.196747	-2.289681	0.02204	0.039291
12777	KAL1	264.841789	-0.450509917	0.263186	-1.711758	0.086941	0.133382
9295	FRAT1	63.32274754	-0.450744979	0.155348	-2.901521	0.003714	0.007831
14725	DNAH11	571.3088258	-0.451051936	0.413534	-1.090726	0.275393	0.366577
9596	KIF17	58.06314261	-0.451053124	0.1622	-2.780844	0.005422	0.011075
12012	NDUFS8	1429.302745	-0.451206379	0.230561	-1.956991	0.050349	0.082158
7564	INCENP	222.6148009	-0.451352083	0.125683	-3.591205	0.000329	0.000853
11510	TXNDC6	10.43387871	-0.451455469	0.213715	-2.112419	0.034651	0.059008
8205	GGCT	545.4018784	-0.451663761	0.135635	-3.329986	0.000869	0.002075
7949	LOC341056	8.926042163	-0.45168045	0.131744	-3.428463	0.000607	0.001497
15817	IL13	0.375880494	-0.451736871	0.585952	-0.770945	0.440739	0.54618
8964	TMEM161A	609.5540913	-0.452037499	0.149398	-3.025722	0.00248	0.005424
10348	TNFAIP8	821.6631318	-0.452195731	0.178782	-2.529314	0.011429	0.021648
6839	FLAD1	576.5281276	-0.452211836	0.116416	-3.884443	0.000103	0.000294
8332	EDF1	3851.866503	-0.452256336	0.137902	-3.279556	0.00104	0.002446
14183	SH3RF2	140.4327129	-0.452289261	0.358387	-1.262013	0.206944	0.285998
9821	SLC37A1	332.138223	-0.452361396	0.166877	-2.710747	0.006713	0.013398
10751	SULT1A3	632.9635668	-0.452396848	0.190045	-2.380466	0.017291	0.031524
13633	C16orf79	21.01243392	-0.452464598	0.313518	-1.443186	0.148968	0.21418
9938	FAM126A	467.3100644	-0.452556293	0.169217	-2.674417	0.007486	0.014765
6325	CTNNBIP1	411.1129779	-0.452574834	0.109979	-4.115088	3.87E-05	0.00012
8691	CMIP	2625.136034	-0.452629118	0.144387	-3.134827	0.00172	0.003878
12014	PITPNM3	52.28205305	-0.452857748	0.231441	-1.956684	0.050385	0.082203
5370	COPS7B	540.9100609	-0.453051015	0.098861	-4.582718	4.59E-06	1.68E-05
13113	PDE1A	333.6803708	-0.45306429	0.281957	-1.606858	0.108086	0.161564
4672	NACA	15671.85899	-0.453171091	0.091556	-4.949638	7.44E-07	3.12E-06
7414	C16orf68	184.3713883	-0.453260696	0.124186	-3.649849	0.000262	0.000694
4897	CCDC23	241.5431884	-0.453346835	0.0939	-4.828	1.38E-06	5.52E-06
6679	FIBP	1228.749684	-0.453352038	0.114781	-3.949714	7.82E-05	0.00023
10322	SLC4A8	38.92078526	-0.453354751	0.178492	-2.539913	0.011088	0.021056
8494	TRIO	1616.275242	-0.45359686	0.141332	-3.209442	0.00133	0.003069
11901	ACTR3C	117.8066453	-0.453599351	0.228263	-1.987182	0.046902	0.077248
15892	LOC339788	1.350392394	-0.453768218	0.609117	-0.744961	0.456295	0.562789
11811	TAS2R5	10.8643198	-0.453768438	0.224879	-2.017832	0.043609	0.072371
9508	SLC2A4RG	1694.298367	-0.453967204	0.161167	-2.816759	0.004851	0.010001
9438	TRIM47	995.5588831	-0.454127985	0.159784	-2.842145	0.004481	0.009306

7439	EHMT2	951.3665477	-0.454378358	0.12479	-3.641149	0.000271	0.000715
12455	LDB3	55.88873816	-0.454558482	0.249962	-1.818509	0.068986	0.108567
16079	CRNA0016	0.298270218	-0.454614601	0.659997	-0.688814	0.490941	0.598478
16641	FLJ43860	0.29939255	-0.45461466	0.845251	-0.537846	0.590684	0.695751
16460	IL1F9	0.268098259	-0.454614672	0.777451	-0.58475	0.558716	0.665333
16682	PRL	0.2406968	-0.454614734	0.869378	-0.52292	0.60103	0.706198
17216	RNF17	0.322294705	-0.454614763	1.180798	-0.385006	0.700233	0.797259
16971	PROP1	0.238924681	-0.454614819	1.020357	-0.445545	0.655926	0.757575
16816	GLRA4	0.181479563	-0.454614844	0.934214	-0.486628	0.626522	0.730278
17293	KRTAP1-1	0.245048339	-0.454614961	1.232855	-0.36875	0.712314	0.807383
17041	TBC1D3P2	0.165038074	-0.454614971	1.057272	-0.429989	0.667204	0.767435
17150	OR1F1	0.208451493	-0.454614972	1.141117	-0.398395	0.690339	0.788999
17279	IRGC	0.142489302	-0.454615167	1.22092	-0.372355	0.709629	0.804991
17376	DAZL	0.188451739	-0.454615167	1.309619	-0.347135	0.72849	0.821773
17280	C12orf40	0.141946809	-0.454615168	1.221326	-0.372231	0.709721	0.805049
17496	TMPRSS15	0.225561223	-0.454615188	1.442477	-0.315163	0.752638	0.843142
17386	MYH2	0.161720385	-0.454615227	1.318564	-0.344781	0.730259	0.823295
17489	TMEFF2	0.200348745	-0.45461524	1.434558	-0.316903	0.751317	0.842048
17382	C12orf54	0.146842647	-0.454615275	1.316769	-0.345251	0.729906	0.823001
17384	SNORD15A	0.146732245	-0.454615275	1.316859	-0.345227	0.729924	0.823001
17602	FOXB2	0.183728479	-0.454615431	1.549813	-0.293336	0.769266	0.856629
17487	ALPPL2	0.12873636	-0.454615474	1.432581	-0.31734	0.750986	0.841772
17608	TBR1	0.16407266	-0.454615507	1.555418	-0.292279	0.770074	0.857236
17819	KRTAP5-5	0.186416225	-0.454615859	1.87592	-0.242343	0.808515	0.889371
17718	LIN28A	0.12236626	-0.454615916	1.729394	-0.262876	0.792646	0.876886
17827	LOC415056	0.150934135	-0.454616087	1.89071	-0.240447	0.809984	0.890617
17832	C13orf30	0.141062938	-0.454616101	1.899217	-0.23937	0.810818	0.891247
17749	ZAR1L	0.086809306	-0.454616169	1.773117	-0.256394	0.797647	0.880876
17849	KFZP434H1	0.107924829	-0.454616367	1.929304	-0.235637	0.813714	0.893435
18141	DCAF8L2	0.242046169	-0.454616484	2.578114	-0.176337	0.860029	0.929245
17879	SDR9C7	0.086293147	-0.45461656	1.970942	-0.23066	0.817579	0.896274
17971	BARHL2	0.125444557	-0.454616651	2.134413	-0.212994	0.831332	0.906735
18001	LOC390858	0.087023859	-0.454617083	2.207988	-0.205897	0.836872	0.911256
17998	C13orf26	0.087641408	-0.454617083	2.208203	-0.205876	0.836887	0.911256
18117	PROL1	0.084271144	-0.454617855	2.517547	-0.18058	0.856697	0.926871
18121	FAM47C	0.082956752	-0.454617877	2.525748	-0.179993	0.857158	0.927165
18176	OR6S1	0.138744078	-0.454618148	2.700206	-0.168364	0.866297	0.934214
18229	GIP	0.122064981	-0.454618502	2.91407	-0.156008	0.876027	0.941374
18235	SPANXE	0.103112726	-0.454618747	2.91407	-0.156008	0.876027	0.941374
18236	SPRR2G	0.107413951	-0.454618747	2.91407	-0.156008	0.876027	0.941374
18219	CSH2	0.090449766	-0.454618863	2.871988	-0.158294	0.874225	0.940588
18224	CXorf64	0.087558471	-0.454618923	2.891578	-0.157222	0.87507	0.94118
18227	CASP14	0.084985853	-0.454618967	2.905991	-0.156442	0.875685	0.941374
18238	TTTY20	0.085319782	-0.454618992	2.91407	-0.156008	0.876027	0.941374
18231	LHX5	0.085106656	-0.454618992	2.91407	-0.156008	0.876027	0.941374
18234	OR5M11	0.08482072	-0.454618992	2.91407	-0.156008	0.876027	0.941374
18239	TTTY2	0.081781476	-0.454618992	2.91407	-0.156008	0.876027	0.941374

13536	KCNV2	1.807176338	-0.454635398	0.308363	-1.474352	0.140387	0.203289
6595	TAF6	1141.598562	-0.45488807	0.114149	-3.985023	6.75E-05	0.000201
11128	NLRP1	453.9220529	-0.455092186	0.202883	-2.243124	0.024889	0.043839
6912	C12orf47	287.3749156	-0.455249838	0.117986	-3.858515	0.000114	0.000323
12428	RSAD2	297.7135426	-0.455250985	0.24915	-1.827213	0.067668	0.106723
5803	NOB1	978.0785029	-0.455338091	0.104116	-4.373381	1.22E-05	4.13E-05
8204	TMEM150A	656.7817321	-0.455442703	0.136729	-3.330984	0.000865	0.002068
8124	FAM98C	245.023312	-0.4556803	0.135594	-3.360625	0.000778	0.001876
7934	RPS6KA1	1007.916605	-0.455776633	0.132709	-3.434405	0.000594	0.001467
6971	C15orf23	166.6756399	-0.456058156	0.118766	-3.839971	0.000123	0.000346
6443	GABARAP	7170.137725	-0.456059674	0.112528	-4.05284	5.06E-05	0.000154
10868	GRIN2C	12.554059	-0.456088479	0.195437	-2.333683	0.019612	0.035372
14262	SLC23A1	279.5613236	-0.456362379	0.368029	-1.240017	0.214969	0.295443
10983	VAMP1	175.9109012	-0.456543252	0.199152	-2.292437	0.02188	0.039049
13216	AGT	2583.454058	-0.45667452	0.289995	-1.574769	0.11531	0.171019
4735	XPO6	1702.582459	-0.457109206	0.092969	-4.916808	8.80E-07	3.64E-06
15558	ASB4	1.690014959	-0.457413904	0.54154	-0.844654	0.398304	0.50181
7548	LPCAT4	361.4210769	-0.457476103	0.127233	-3.595583	0.000324	0.000841
3494	MED8	587.5579965	-0.457561528	0.080579	-5.678392	1.36E-08	7.63E-08
7224	FKBPL	111.7959737	-0.45768209	0.122483	-3.736711	0.000186	0.000506
6612	IKBKG	383.0479136	-0.457701562	0.11503	-3.97898	6.92E-05	0.000205
7051	TSEN54	335.8257736	-0.457712399	0.120439	-3.80036	0.000144	0.000402
14953	KCNH7	0.673086209	-0.457724074	0.447916	-1.021897	0.30683	0.402205
5820	STARD3	837.8903388	-0.457746933	0.104906	-4.363406	1.28E-05	4.31E-05
8478	ATP5E	4171.469078	-0.457792473	0.142334	-3.216329	0.001298	0.003002
3516	COPZ1	3913.202545	-0.45785468	0.080854	-5.662721	1.49E-08	8.31E-08
8816	CCDC130	411.4886784	-0.458126453	0.148622	-3.082488	0.002053	0.004564
8258	ZMIZ2	2498.22248	-0.458219764	0.138438	-3.309916	0.000933	0.002215
12752	SLC22A17	666.2301742	-0.45826409	0.26683	-1.717441	0.085899	0.132034
5400	TMEM206	167.9777731	-0.45834555	0.100389	-4.565706	4.98E-06	1.81E-05
8885	LY6G5C	25.22973791	-0.458879536	0.150063	-3.05791	0.002229	0.004917
10661	MMD	434.8239325	-0.458904093	0.190568	-2.408091	0.016036	0.029484
11308	OC1001333	91.06587359	-0.45901318	0.210925	-2.176187	0.029541	0.051206
6875	ZNF642	93.43754394	-0.45901906	0.118503	-3.87348	0.000107	0.000306
3895	NUP62	1352.09268	-0.459244084	0.084943	-5.406511	6.43E-08	3.23E-07
15302	TBC1D26	0.866661869	-0.459322191	0.499107	-0.920287	0.357423	0.457838
6358	PRKD2	1227.654	-0.459550868	0.112152	-4.09757	4.18E-05	0.000129
10237	RASA4P	142.6416579	-0.459698162	0.178841	-2.570434	0.010157	0.019448
12973	KCNC3	344.6092258	-0.459736271	0.278339	-1.651713	0.098593	0.148965
12953	GALNT14	4580.666456	-0.459886412	0.277759	-1.655703	0.097782	0.147968
15715	CALR3	0.406807098	-0.460342472	0.575591	-0.799774	0.423842	0.528649
6127	SEPN1	2734.33904	-0.460614457	0.109454	-4.208296	2.57E-05	8.23E-05
14663	ZFR2	1.560929742	-0.460819722	0.414454	-1.111871	0.266194	0.355839
8652	MRPS21	984.2435879	-0.460958749	0.146333	-3.150067	0.001632	0.003698
11270	VWA1	2768.178922	-0.461196989	0.210402	-2.191979	0.028381	0.049361
6568	DNMT1	811.5797408	-0.461267971	0.115421	-3.99639	6.43E-05	0.000192
6417	UBE2O	663.3005266	-0.46130695	0.113354	-4.069612	4.71E-05	0.000144

8491	RNF122	242.1981417	-0.461475469	0.143723	-3.210867	0.001323	0.003055
16485	MYOD1	0.592953934	-0.46209419	0.802405	-0.575887	0.564692	0.671349
13437	LOC14913	11.66081561	-0.462180446	0.307294	-1.504036	0.132572	0.193388
8900	ABCB8	654.573905	-0.462183318	0.151423	-3.052274	0.002271	0.005002
11514	CRNA001	3.675865902	-0.462368065	0.218949	-2.111766	0.034707	0.059083
6029	MED15	1824.453599	-0.462377333	0.108737	-4.252235	2.12E-05	6.88E-05
5737	PRCC	1182.575067	-0.462513543	0.105053	-4.402671	1.07E-05	3.65E-05
15004	PTCHD1	1.361398566	-0.462823733	0.459819	-1.006534	0.314159	0.410412
16230	SNORD94	0.450177543	-0.463183432	0.716521	-0.646434	0.517998	0.625588
11743	ZNF521	323.3929164	-0.463299243	0.227211	-2.039068	0.041443	0.069176
10320	GLUL	11497.02612	-0.463342793	0.182352	-2.540924	0.011056	0.021
6287	FKBP1A	5695.55169	-0.463396841	0.112029	-4.136388	3.53E-05	0.00011
6908	PHC2	4257.299314	-0.46354928	0.120081	-3.860291	0.000113	0.000321
8299	CYB561D2	310.1036224	-0.463584679	0.140733	-3.294072	0.000987	0.002332
16775	LRRC26	0.492614704	-0.46418502	0.933078	-0.497477	0.618852	0.723107
15616	C17orf73	0.602803262	-0.464191318	0.558182	-0.831612	0.405628	0.509139
7364	C1orf57	765.6800159	-0.464222286	0.126399	-3.672686	0.00024	0.000639
5146	VPS16	842.8716931	-0.464874321	0.098814	-4.704516	2.54E-06	9.69E-06
4475	RAN	3630.116233	-0.464897861	0.091965	-5.055184	4.30E-07	1.88E-06
11948	OC1001322	270.1104303	-0.465025633	0.235412	-1.97537	0.048226	0.079116
15126	RORB	3.418978782	-0.465084475	0.481298	-0.966313	0.333888	0.432668
7093	ANKRD16	148.8841274	-0.465091208	0.1228	-3.787398	0.000152	0.000421
12128	DPRXP4	3.51303678	-0.46518421	0.242465	-1.918561	0.05504	0.088954
9046	MRPS26	754.6667909	-0.46521871	0.155202	-2.997498	0.002722	0.005898
4397	GNG5	1464.36252	-0.465371559	0.091148	-5.105653	3.30E-07	1.47E-06
6776	HEATR2	815.6570935	-0.465389129	0.119071	-3.908516	9.29E-05	0.000269
10353	ADAT3	40.4949649	-0.465408042	0.184172	-2.527031	0.011503	0.021779
11920	PARD6A	100.9884646	-0.465722396	0.234938	-1.982317	0.047444	0.07802
7164	C19orf56	1641.537413	-0.465977061	0.124009	-3.757613	0.000172	0.000469
11072	TLR1	278.3698815	-0.465982973	0.205698	-2.265373	0.02349	0.041587
10756	APCDD1	359.2184717	-0.466128544	0.195899	-2.37943	0.017339	0.031598
6487	CENPB	2132.487349	-0.466144554	0.11556	-4.033772	5.49E-05	0.000166
6445	TBC1D22A	488.8659046	-0.466190526	0.115059	-4.051761	5.08E-05	0.000155
10385	ECT2	448.1036817	-0.466707936	0.185856	-2.511121	0.012035	0.022715
9429	XRCC3	134.3013915	-0.466926575	0.163986	-2.847365	0.004408	0.009164
4123	PTDSS1	1542.422102	-0.467097644	0.088641	-5.269523	1.37E-07	6.50E-07
13337	SGCA	33.42542103	-0.467175242	0.304954	-1.531953	0.125534	0.184494
7346	RPL10A	9176.789131	-0.467223896	0.126838	-3.683624	0.00023	0.000614
6147	KRI1	594.1287291	-0.467287045	0.111175	-4.203185	2.63E-05	8.39E-05
5215	KDELR1	4530.441825	-0.4673732	0.100028	-4.672411	2.98E-06	1.12E-05
8432	MGC16275	41.84015624	-0.467422406	0.144441	-3.236083	0.001212	0.002817
6195	PDIA3P	3176.147585	-0.467492704	0.111911	-4.177344	2.95E-05	9.33E-05
10955	BRCA2	78.64282785	-0.467513848	0.203098	-2.301917	0.02134	0.038182
8344	CDC34	770.9486647	-0.46801192	0.142869	-3.275801	0.001054	0.002475
12499	PILRB	1299.621913	-0.468126376	0.259396	-1.804678	0.071125	0.111539
5655	C7orf49	1102.456836	-0.468418689	0.10538	-4.445023	8.79E-06	3.05E-05
5440	BRE	898.6940394	-0.468489648	0.103048	-4.546319	5.46E-06	1.97E-05

3132	SSRP1	2844.549805	-0.468942122	0.078968	-5.938388	2.88E-09	1.80E-08
6843	SART1	1159.669708	-0.468960158	0.120788	-3.882493	0.000103	0.000296
15712	C2orf61	0.529158326	-0.469007022	0.585815	-0.800606	0.42336	0.528149
16091	EIF1AY	242.5110025	-0.469060887	0.685966	-0.683796	0.494104	0.601885
13107	PCDHB3	100.5100653	-0.469304132	0.29179	-1.608362	0.107756	0.161148
14211	ACSM1	23.07003216	-0.469491861	0.374536	-1.25353	0.210013	0.289626
4499	EXT2	2186.874463	-0.469629394	0.093189	-5.039563	4.67E-07	2.03E-06
9249	FBXO22OS	59.92350187	-0.469634763	0.160855	-2.919613	0.003505	0.007427
6917	SLC25A28	632.7606979	-0.469686967	0.121772	-3.857113	0.000115	0.000325
8191	CTU2	221.5730749	-0.46969785	0.140732	-3.337534	0.000845	0.002023
11868	CD79B	137.3785513	-0.469939009	0.234854	-2.00098	0.045395	0.074967
7395	SS18L2	214.0205041	-0.470350298	0.128558	-3.658671	0.000254	0.000672
9446	RTEL1	489.986906	-0.470459127	0.165659	-2.839925	0.004512	0.009364
6663	ADCK2	904.9128291	-0.470901505	0.119018	-3.956557	7.60E-05	0.000224
8228	RAB32	799.9581792	-0.471000916	0.1418	-3.321582	0.000895	0.002132
4732	DAZAP1	1324.570947	-0.471016842	0.095786	-4.917411	8.77E-07	3.63E-06
9956	GIMAP1	243.3849846	-0.471205405	0.176571	-2.668649	0.007616	0.014993
8326	ADRBK2	413.9510029	-0.471326844	0.143655	-3.280963	0.001035	0.002435
12287	OSGIN1	315.0229873	-0.471469707	0.252199	-1.869437	0.061562	0.098208
5345	CCDC22	481.3594419	-0.471517871	0.10259	-4.596126	4.30E-06	1.58E-05
13595	FLJ26850	1.609317704	-0.471683655	0.323718	-1.457084	0.145093	0.209192
12412	SAMD3	65.20504202	-0.471717444	0.25742	-1.83248	0.06688	0.10562
15622	SFTA2	1.032102658	-0.471931622	0.568949	-0.82948	0.406833	0.510484
13317	D4S234E	17.24370948	-0.472229622	0.307003	-1.538192	0.124002	0.182515
7724	COMMD7	1512.71958	-0.472233255	0.133738	-3.53102	0.000414	0.00105
14465	C2orf66	1.297399155	-0.472244722	0.400725	-1.178474	0.238608	0.323328
5126	DNMT3A	387.7484313	-0.472383458	0.10019	-4.71488	2.42E-06	9.25E-06
11457	RAB41	2.318903517	-0.47243995	0.221593	-2.132017	0.033005	0.056467
12502	ADAMTS3	90.00489112	-0.472452587	0.261979	-1.803395	0.071326	0.111827
13218	ENHO	5.248086494	-0.474070448	0.301063	-1.574653	0.115337	0.171033
6675	SMUG1	574.3585354	-0.47422045	0.119985	-3.952336	7.74E-05	0.000227
12161	P2RY13	178.8554955	-0.474243066	0.248596	-1.907689	0.056431	0.090953
15831	LCN6	0.651004829	-0.474421397	0.619141	-0.766258	0.443523	0.549144
8269	SNX33	1499.677853	-0.47448167	0.143598	-3.304247	0.000952	0.002257
4444	SMPD4	1396.621246	-0.474650877	0.093502	-5.076368	3.85E-07	1.70E-06
7462	NAT6	127.9534539	-0.474671509	0.130674	-3.632486	0.000281	0.000737
5659	CDK2AP1	2282.329026	-0.47480045	0.106856	-4.443382	8.86E-06	3.07E-05
13217	HTR1F	40.82160957	-0.474810059	0.301521	-1.574717	0.115322	0.171024
6352	HMGB2	1339.228329	-0.474983473	0.115864	-4.099503	4.14E-05	0.000128
14430	RFPL4A	2.819331191	-0.475281809	0.399849	-1.188653	0.234576	0.318637
8048	ALDOA	48142.11904	-0.475719256	0.140199	-3.393168	0.000691	0.001683
10935	NDUFA3	841.1296387	-0.475726087	0.206103	-2.308201	0.020988	0.037621
12347	WBP2NL	9.414357195	-0.476087838	0.257386	-1.849703	0.064356	0.102166
12060	CLIP3	579.0933938	-0.476191348	0.245267	-1.941523	0.052195	0.084832
15499	FP91-CNT	0.961806793	-0.476459012	0.552983	-0.861616	0.388899	0.491825
12679	CCDC114	13.21314233	-0.476513065	0.273049	-1.745157	0.080958	0.125156
13358	RBP5	4263.369272	-0.476756599	0.312696	-1.524663	0.127343	0.186858

9931	C5orf34	31.23760904	-0.476789018	0.178105	-2.677007	0.007428	0.01466
15519	GRIA2	1.122261064	-0.476918822	0.559127	-0.85297	0.393676	0.497225
11176	LOC149837	48.63288762	-0.476947489	0.21385	-2.230285	0.025729	0.04512
5946	DNAJC17	209.7322158	-0.477058278	0.111124	-4.293041	1.76E-05	5.81E-05
9766	ELMO1	859.4102118	-0.477274831	0.175103	-2.725677	0.006417	0.012879
13026	FLT3	22.46348443	-0.477351183	0.291916	-1.635235	0.102	0.153485
9703	SOX4	1616.334147	-0.477406789	0.173862	-2.745899	0.006035	0.01219
11763	HAS3	74.43272578	-0.477488794	0.234881	-2.032896	0.042063	0.070091
4405	CDK2	519.8735352	-0.477851045	0.093693	-5.100154	3.39E-07	1.51E-06
10180	SAMD10	167.3734404	-0.477865539	0.184555	-2.58928	0.009618	0.018516
5327	GTDC1	398.1419968	-0.477962349	0.103703	-4.608941	4.05E-06	1.49E-05
13707	GGTLC2	508.7382861	-0.478169499	0.337676	-1.41606	0.156758	0.224164
9814	C11orf83	309.4399142	-0.478333292	0.176301	-2.713165	0.006664	0.01331
6152	WDR45	1281.041753	-0.478501393	0.113876	-4.201943	2.65E-05	8.43E-05
9315	FLJ35776	134.2444797	-0.478502842	0.165309	-2.894598	0.003796	0.007989
9863	NFKB2	1496.038639	-0.478682835	0.177347	-2.699138	0.006952	0.013816
6714	TSGA14	211.3939907	-0.478884493	0.12168	-3.935621	8.30E-05	0.000242
10888	ULBP3	30.8296157	-0.478915909	0.206091	-2.323804	0.020136	0.03625
10978	POLE2	25.81730276	-0.479469715	0.208966	-2.294492	0.021762	0.038859
12237	APLNR	1270.089659	-0.479613365	0.254251	-1.886376	0.059244	0.094896
6083	YWHAG	5802.37321	-0.479962954	0.113537	-4.227355	2.36E-05	7.62E-05
5117	ACTG1	47132.5205	-0.480096007	0.101734	-4.719143	2.37E-06	9.07E-06
4336	RNF7	1444.239526	-0.480174472	0.093459	-5.137797	2.78E-07	1.26E-06
6800	NOMO1	4506.115615	-0.480413758	0.123176	-3.90022	9.61E-05	0.000277
7060	OC1001909	142.1499206	-0.48058917	0.126554	-3.797496	0.000146	0.000406
10396	C9orf116	111.8352338	-0.480677734	0.191647	-2.50814	0.012137	0.022882
6528	FBXO4	200.7532212	-0.480914451	0.119798	-4.014366	5.96E-05	0.000179
9294	C21orf70	169.9587702	-0.480947087	0.165714	-2.902268	0.003705	0.007813
11480	CSPG4	2918.198048	-0.480952512	0.226109	-2.127086	0.033413	0.057049
10613	HSD11B1L	119.1841301	-0.481112832	0.19778	-2.432567	0.014992	0.027689
11434	CRNA0017	18.81463276	-0.481204632	0.224998	-2.138702	0.03246	0.055645
3805	C16orf57	768.10428	-0.481218633	0.087973	-5.470063	4.50E-08	2.32E-07
2779	EFTUD2	1798.054107	-0.481519026	0.077487	-6.214161	5.16E-10	3.64E-09
11774	C14orf49	27.53697672	-0.481839696	0.237437	-2.029336	0.042424	0.070626
10833	WDR54	488.348581	-0.482244802	0.205333	-2.348594	0.018844	0.034097
6792	PVRL2	2046.605966	-0.482389757	0.123598	-3.902906	9.50E-05	0.000274
10014	FAM102B	499.342476	-0.482463928	0.182159	-2.648585	0.008083	0.015821
15291	NKAIN4	237.4658325	-0.482815007	0.52306	-0.923059	0.355977	0.456314
7534	COPG2	315.0855092	-0.482841204	0.134033	-3.60241	0.000315	0.00082
6617	GEMIN6	205.2272082	-0.483123079	0.121469	-3.977328	6.97E-05	0.000206
8824	C14orf145	67.70616565	-0.48349963	0.156908	-3.081414	0.00206	0.004576
8804	KNTC1	359.8635626	-0.483542589	0.15673	-3.085193	0.002034	0.004529
8863	GFER	470.3745179	-0.483552494	0.157747	-3.065359	0.002174	0.004808
6682	RABL5	772.7789261	-0.483978921	0.12258	-3.948255	7.87E-05	0.000231
13932	S100A12	17.02804398	-0.484037269	0.36101	-1.340786	0.17999	0.253229
14698	VSIG8	1.546061186	-0.484181439	0.440598	-1.098918	0.271804	0.362473
6954	RPL26	10407.12967	-0.484256593	0.125954	-3.844708	0.000121	0.00034

12146	PRICKLE1	214.3135445	-0.48452625	0.253267	-1.913102	0.055735	0.089944
7408	OC1001281	53.19406015	-0.484609005	0.132652	-3.653237	0.000259	0.000685
13587	SUSD5	66.87648738	-0.484714104	0.332311	-1.458617	0.14467	0.208706
12362	INMT	301.2559333	-0.484823522	0.262465	-1.847194	0.064719	0.102618
16586	PGC	0.86013813	-0.484884971	0.879768	-0.551151	0.58153	0.687241
8750	SEC24D	1280.146873	-0.484893764	0.156054	-3.107226	0.001889	0.004231
15491	FAM157B	0.600841813	-0.484947842	0.560938	-0.86453	0.387297	0.490053
4570	STRADA	527.7924106	-0.484998261	0.097018	-4.99905	5.76E-07	2.47E-06
9029	HPS3	1968.98608	-0.485036944	0.161551	-3.002381	0.002679	0.005815
8532	ZNF485	52.01324328	-0.485123183	0.151842	-3.194912	0.001399	0.003213
12306	AGER	82.33730903	-0.485253291	0.260481	-1.862913	0.062475	0.09951
9676	PLXNA3	719.6982791	-0.485267869	0.176125	-2.75524	0.005865	0.011881
7210	GEMIN7	336.7879461	-0.485642124	0.129869	-3.739472	0.000184	0.000501
7973	MBD3	1419.245889	-0.485733902	0.142005	-3.420549	0.000625	0.001536
9384	SAT1	7418.864102	-0.48583346	0.169566	-2.865157	0.004168	0.008706
11204	MBOAT4	6.514561867	-0.485893148	0.218835	-2.220359	0.026394	0.046176
10970	SOD2	29477.80372	-0.485946195	0.211552	-2.297052	0.021616	0.038625
6309	GLA	617.4277352	-0.486078071	0.117946	-4.1212	3.77E-05	0.000117
9414	LYRM1	1104.749939	-0.486140483	0.170435	-2.852355	0.00434	0.009036
13523	PAPPA	133.97871	-0.486342717	0.328578	-1.480144	0.138835	0.201235
6229	MEA1	1185.99892	-0.486512567	0.116839	-4.163945	3.13E-05	9.84E-05
9597	FAM58A	324.9524348	-0.48664956	0.175032	-2.780342	0.00543	0.011091
3336	ARPC4	2893.497833	-0.486718701	0.083999	-5.794362	6.86E-09	4.03E-08
4933	EXT1	920.0095514	-0.486826343	0.101261	-4.807646	1.53E-06	6.07E-06
11500	KRBA1	1161.32154	-0.486908179	0.229903	-2.117884	0.034185	0.058266
7719	G6PC3	1513.252639	-0.486917491	0.137821	-3.53296	0.000411	0.001043
4033	DSN1	314.1618677	-0.486924468	0.09159	-5.316356	1.06E-07	5.15E-07
5996	FNBP1	1339.91839	-0.486954164	0.114056	-4.269446	1.96E-05	6.41E-05
5975	GTPBP3	272.7604859	-0.487319191	0.113858	-4.280053	1.87E-05	6.13E-05
12106	ADAMTSL1	305.8814004	-0.487534891	0.252934	-1.927521	0.053915	0.087294
5938	PRMT1	1745.020813	-0.487623187	0.113429	-4.29894	1.72E-05	5.66E-05
9066	PARP10	1908.246915	-0.487639717	0.162984	-2.99195	0.002772	0.005993
12400	CHRNA10	13.13524465	-0.487651062	0.265581	-1.836165	0.066333	0.104851
16070	SCT	0.992786689	-0.48808331	0.705632	-0.691697	0.489128	0.596602
6650	RDBP	1351.767806	-0.488099384	0.123194	-3.96203	7.43E-05	0.000219
9745	CHSY1	1957.667564	-0.48844867	0.178673	-2.73376	0.006262	0.012594
7393	ADPRHL2	995.3939921	-0.488457619	0.133495	-3.658992	0.000253	0.000671
15981	OPN5	0.404233297	-0.488949867	0.68152	-0.717441	0.473102	0.580269
4513	RAD23A	2838.702286	-0.489007916	0.097255	-5.028075	4.95E-07	2.15E-06
8792	SEPW1	3020.001285	-0.489267182	0.158434	-3.088139	0.002014	0.00449
10007	MCART6	18.63564924	-0.48929718	0.18461	-2.650443	0.008039	0.015746
11164	HIPK4	2.526522133	-0.489497718	0.219199	-2.233118	0.025541	0.044844
10549	CCND2	1433.800914	-0.489767817	0.199618	-2.453526	0.014146	0.026285
7420	C22orf26	16.14867308	-0.490290122	0.134369	-3.648845	0.000263	0.000696
3422	NKIRAS2	1007.427238	-0.4903835	0.085624	-5.727146	1.02E-08	5.85E-08
9226	BTN3A2	2654.616419	-0.490980249	0.167645	-2.928691	0.003404	0.007232
6017	WBSCR22	1170.56775	-0.491039805	0.115346	-4.257092	2.07E-05	6.75E-05

12714	GOLGA8A	1151.138694	-0.491165703	0.284254	-1.727911	0.084004	0.129508
7528	ATOX1	1152.237298	-0.491234636	0.136274	-3.604768	0.000312	0.000813
12710	C19orf18	7.430877129	-0.491303299	0.284191	-1.728777	0.083849	0.12931
7152	POLR1E	388.759414	-0.491311347	0.130557	-3.763193	0.000168	0.00046
11505	HLA-DOA	1585.910284	-0.491320207	0.232326	-2.114788	0.034448	0.058689
9275	COMMD4	913.993977	-0.491532744	0.169052	-2.907589	0.003642	0.007697
11689	TUSC3	723.6463902	-0.491635904	0.239404	-2.053587	0.040016	0.067104
6756	LYRM4	329.2982973	-0.491852021	0.125564	-3.917138	8.96E-05	0.00026
12980	REC8	108.2360875	-0.491891121	0.29807	-1.650253	0.098891	0.149335
14669	LRRTM4	6.045770731	-0.492178725	0.442983	-1.111055	0.266544	0.356162
13351	RNF182	30.40987934	-0.492419481	0.322141	-1.528584	0.126367	0.185524
8980	AURKAIP1	1221.362149	-0.492494418	0.163064	-3.020249	0.002526	0.005513
10629	UQCRQ	2599.036807	-0.492594051	0.203341	-2.422502	0.015414	0.028425
12527	SELP	178.4299981	-0.492612773	0.274152	-1.796863	0.072357	0.113244
11841	LOC150381	99.55324593	-0.492931999	0.245438	-2.008377	0.044603	0.073834
5447	CCDC92	1155.854757	-0.492987516	0.108531	-4.54236	5.56E-06	2.00E-05
12939	ANKK1	10.4143077	-0.493153222	0.296625	-1.662547	0.096403	0.146039
8594	AHDC1	805.3757917	-0.493309813	0.155333	-3.175826	0.001494	0.003407
13418	CYP2D6	11.60961773	-0.493352296	0.326991	-1.508763	0.131359	0.19189
12924	C4orf44	2.358190959	-0.493935826	0.296492	-1.665933	0.095727	0.145172
1365	PA2G4	2934.339179	-0.493988731	0.063563	-7.771699	7.74E-15	1.11E-13
8190	TRMT2A	617.9750758	-0.494059608	0.148008	-3.338068	0.000844	0.002019
7340	MLXIP	1019.041893	-0.4941399	0.134125	-3.684186	0.000229	0.000613
15661	SOX3	0.536994955	-0.494161569	0.603382	-0.818986	0.412795	0.516646
14702	C9orf144B	0.792225083	-0.494241431	0.450163	-1.097916	0.272241	0.362957
15724	ANGPTL7	3.655038111	-0.494362098	0.620317	-0.796951	0.42548	0.530388
13780	ADCY10	5.870414592	-0.4944652	0.354877	-1.393343	0.163516	0.232589
6797	SEC13	1540.819578	-0.494507104	0.126771	-3.900784	9.59E-05	0.000277
7671	STX8	397.7604935	-0.495061102	0.139424	-3.550755	0.000384	0.000982
6716	UBE2J2	745.4350487	-0.495346101	0.125904	-3.934325	8.34E-05	0.000243
2391	H2AFY	2610.955329	-0.495449894	0.075447	-6.566874	5.14E-11	4.21E-10
15489	AIRE	1.131343681	-0.495715844	0.572918	-0.865247	0.386903	0.489618
8855	LOC644936	35.58133755	-0.496104053	0.161685	-3.068331	0.002153	0.004765
5267	SNRPG	696.659995	-0.496127033	0.106834	-4.643888	3.42E-06	1.27E-05
11082	CDK3	178.3484097	-0.496138282	0.219274	-2.262641	0.023658	0.041844
11907	SLC12A5	10.30232857	-0.496223939	0.249928	-1.985465	0.047093	0.077523
8437	PPIC	854.8639856	-0.496400874	0.153521	-3.233441	0.001223	0.002841
10775	PRSS27	24.40941432	-0.496630424	0.209261	-2.373262	0.017632	0.032074
10343	SGOL2	106.6914428	-0.496693169	0.196197	-2.531599	0.011354	0.021518
13119	KIAA1244	253.4626819	-0.496710641	0.309367	-1.605572	0.108368	0.161912
13361	HSD17B2	61.79243484	-0.496914337	0.326296	-1.522895	0.127785	0.187464
9435	MFNG	438.3546497	-0.497035137	0.174751	-2.844251	0.004452	0.009248
4239	TMEM185A	535.7113402	-0.497339187	0.095894	-5.186356	2.14E-07	9.92E-07
11405	PLCD3	614.5176348	-0.497359447	0.231623	-2.147282	0.031771	0.054598
7686	EEF1B2	4796.838869	-0.497583838	0.140373	-3.544725	0.000393	0.001002
11821	LY75	654.112517	-0.497658064	0.24699	-2.014894	0.043916	0.072825
11290	MLC1	14.30610482	-0.497671377	0.228103	-2.181784	0.029126	0.050566

11349	FAT2	23.27505756	-0.49768386	0.229782	-2.165892	0.030319	0.052363
11436	TCTEX1D1	13.40136371	-0.497688319	0.232761	-2.138198	0.032501	0.055705
7360	NUDT5	799.6366663	-0.498193563	0.135599	-3.674009	0.000239	0.000636
5544	SAPS1	1913.943072	-0.498310139	0.110712	-4.500978	6.76E-06	2.39E-05
10349	SNX22	249.5133889	-0.498611616	0.197195	-2.528517	0.011455	0.021695
11237	ATAD3B	209.8374712	-0.498713463	0.226216	-2.204593	0.027483	0.047939
11675	STC1	4478.425553	-0.499151686	0.242662	-2.056982	0.039688	0.066637
8968	PTOV1	1974.797896	-0.499164865	0.165074	-3.023894	0.002495	0.005454
12879	NR4A2	363.277997	-0.499196611	0.296905	-1.681334	0.092698	0.141081
6676	BCAP31	5971.429334	-0.49921091	0.126321	-3.951934	7.75E-05	0.000228
11463	ELF3	1787.022237	-0.499269813	0.234355	-2.130396	0.033139	0.056665
9422	HOXC5	35.08821395	-0.499315552	0.175169	-2.85048	0.004365	0.009081
7232	SNHG11	145.3483657	-0.499452505	0.133817	-3.732364	0.00019	0.000514
5201	NANS	740.2396207	-0.499524705	0.106799	-4.677242	2.91E-06	1.10E-05
4468	SF3B4	1297.215284	-0.49952645	0.098776	-5.057188	4.25E-07	1.87E-06
8368	HMBS	332.5221293	-0.499574304	0.153053	-3.264063	0.001098	0.002573
11221	IER2	2365.994097	-0.499631438	0.225738	-2.213323	0.026875	0.046946
14447	WFDC2	1421.886734	-0.49970003	0.422126	-1.183771	0.236504	0.320877
9839	IDH1	1970.960641	-0.500021479	0.184718	-2.706939	0.006791	0.013528
14584	FMO6P	0.813094769	-0.500033467	0.438003	-1.141622	0.253611	0.340855
6219	LOC678655	368.0259233	-0.500102845	0.119936	-4.169742	3.05E-05	9.61E-05
10933	C19orf20	104.7237896	-0.500227287	0.216704	-2.308347	0.02098	0.037612
14652	FMN2	3.30275243	-0.500330195	0.44818	-1.116359	0.264268	0.35353
4669	RNF113A	296.7508799	-0.500444692	0.101097	-4.950138	7.42E-07	3.11E-06
9777	LTBP3	2457.498191	-0.500559348	0.183788	-2.723573	0.006458	0.012947
7897	CHID1	1854.727754	-0.500768894	0.145122	-3.450684	0.000559	0.001388
12736	TOX	145.293528	-0.501202737	0.291162	-1.721385	0.085181	0.131085
8457	C1orf85	1667.494178	-0.501582338	0.155514	-3.225323	0.001258	0.002916
9123	PNKP	520.1567184	-0.50173893	0.16905	-2.967988	0.002998	0.00644
9206	FGD6	824.2376912	-0.50177894	0.170918	-2.935786	0.003327	0.007083
13954	PAQR9	9.931804955	-0.50184575	0.376401	-1.333273	0.182442	0.256283
10192	ELP2P	9.842115392	-0.502077266	0.194201	-2.585345	0.009728	0.018709
10159	KLRG1	40.22553634	-0.50242785	0.193505	-2.596464	0.009419	0.018173
9842	BICD1	338.0092545	-0.502704513	0.185762	-2.706176	0.006806	0.013553
9291	FBXL7	422.1889485	-0.502768627	0.1732	-2.902817	0.003698	0.007802
7743	LRRC20	223.6383293	-0.50297264	0.142803	-3.522152	0.000428	0.001084
13071	MYOCD	42.79606892	-0.503308291	0.310236	-1.622339	0.104731	0.157052
10733	LOH3CR2A	37.75268315	-0.503325085	0.210856	-2.387052	0.016984	0.031019
3529	PHF21A	905.153249	-0.503343926	0.089065	-5.651444	1.59E-08	8.84E-08
4131	TSEN34	865.7914621	-0.503485289	0.095674	-5.262523	1.42E-07	6.74E-07
7071	ERI3	1049.247431	-0.503794957	0.132761	-3.794764	0.000148	0.00041
6373	MAP4K4	3452.389249	-0.503894585	0.12314	-4.092034	4.28E-05	0.000132
2789	DULLARD	2027.179446	-0.50413419	0.081293	-6.201466	5.59E-10	3.93E-09
11016	CSAD	380.7678081	-0.50415151	0.220574	-2.285633	0.022276	0.039636
11386	C11orf35	88.16461557	-0.504152359	0.233759	-2.156716	0.031028	0.053414
7640	RPS6KB2	631.6276295	-0.504186366	0.141444	-3.564577	0.000364	0.000935
12335	POLR2J2	338.0850901	-0.504264596	0.272039	-1.853645	0.06379	0.101369

8554	LAMB1	6926.768084	-0.504370254	0.158206	-3.188066	0.001432	0.003282
12790	IGFBP5	35035.61238	-0.504592844	0.295534	-1.707395	0.087749	0.134477
5666	RASSF1	468.4652147	-0.505080188	0.11378	-4.439085	9.03E-06	3.13E-05
7788	TRADD	817.9338086	-0.505537281	0.144231	-3.505065	0.000456	0.001149
6474	PTCD1	335.204619	-0.505702453	0.125139	-4.041132	5.32E-05	0.000161
13453	FGF7	148.8000809	-0.505779819	0.336951	-1.501048	0.133343	0.194281
11746	RTKN2	49.19298156	-0.50594513	0.248262	-2.037949	0.041555	0.069344
15207	TKTL2	0.495751181	-0.506012666	0.535103	-0.945636	0.344334	0.443828
12893	THEMIS	92.07263957	-0.506518152	0.302383	-1.675089	0.093917	0.14278
14554	GABRP	29.50778065	-0.506575853	0.440435	-1.150173	0.250073	0.336792
5071	TBC1D2B	1550.25225	-0.50660621	0.106953	-4.736729	2.17E-06	8.40E-06
7352	NLE1	313.0521672	-0.507037356	0.137816	-3.679094	0.000234	0.000624
12949	WNT9A	6.882465227	-0.507133998	0.305858	-1.65807	0.097303	0.147289
6884	NACC1	1145.892082	-0.507239648	0.13109	-3.869397	0.000109	0.000311
12828	CCR4	27.92540082	-0.507251278	0.299043	-1.696246	0.089839	0.137273
13230	LCN12	44.93137477	-0.507310025	0.322794	-1.571624	0.116038	0.171917
15391	H1FNT	0.386236357	-0.507345741	0.568247	-0.892826	0.37195	0.473692
9661	FJX1	328.8617454	-0.507735384	0.183867	-2.761434	0.005755	0.011676
3589	TAF12	406.0618553	-0.508092235	0.090548	-5.611277	2.01E-08	1.10E-07
8185	SF3B5	1248.649901	-0.508433003	0.152252	-3.339423	0.00084	0.00201
9828	LDHA	45037.01102	-0.508481742	0.18765	-2.709738	0.006734	0.01343
11275	SYNPO2	1327.977884	-0.509220785	0.232718	-2.188147	0.028659	0.049822
15176	LOC44245	0.3918086	-0.509314818	0.533592	-0.954502	0.33983	0.438917
13364	HSPA7	247.7648827	-0.509535896	0.334751	-1.522132	0.127976	0.187703
15949	PAN-P2RY	24.9174536	-0.509814919	0.701597	-0.726649	0.467441	0.574494
6641	MSTO1	556.8733144	-0.509831003	0.128514	-3.967124	7.27E-05	0.000215
11691	GPR162	75.80405966	-0.509954216	0.248356	-2.053323	0.040041	0.067133
7757	LOC39974	92.54902648	-0.510153842	0.145122	-3.515344	0.000439	0.00111
11440	TSPAN13	867.2066254	-0.510274865	0.238723	-2.137516	0.032556	0.055781
6035	ERCC1	919.1733779	-0.510469477	0.120101	-4.250342	2.13E-05	6.93E-05
6998	C11orf84	309.6551027	-0.510639677	0.133522	-3.824377	0.000131	0.000367
16227	RSPH6A	0.497079052	-0.5109292	0.788724	-0.647792	0.517119	0.624642
12469	FCN1	183.9212908	-0.511179523	0.282162	-1.811655	0.070039	0.110101
8184	TIMM17B	611.7807312	-0.511275761	0.153031	-3.340992	0.000835	0.001999
9106	KPTN	118.9658157	-0.512067693	0.17212	-2.975067	0.002929	0.006305
15174	TAS2R13	0.330402894	-0.512294113	0.536252	-0.955323	0.339414	0.438438
16084	FAM136B	0.2743611	-0.512322412	0.745957	-0.686799	0.492209	0.599838
16543	TULP1	0.284319064	-0.512322462	0.912005	-0.561754	0.574284	0.680441
16359	IL29	0.214136006	-0.5123225	0.837857	-0.611468	0.54089	0.648083
16794	OR51B5	0.274187457	-0.512322509	1.042734	-0.491326	0.623196	0.727359
16484	HIST1H2AI	0.212841356	-0.512322523	0.889489	-0.575974	0.564633	0.671349
16473	TEX13B	0.190348124	-0.512322543	0.88262	-0.580457	0.561607	0.668248
16701	CRYBA1	0.190262545	-0.512322629	0.987929	-0.518582	0.604052	0.708941
17435	GYPB	0.177110052	-0.512323095	1.550787	-0.330363	0.741125	0.833198
17342	SNORA52	0.132482615	-0.512323102	1.43603	-0.356763	0.721269	0.815223
17560	PDX1	0.174633896	-0.512323258	1.704479	-0.300575	0.763739	0.852508
17457	HHLA1	0.11409899	-0.512323338	1.577468	-0.324776	0.745351	0.836868

17578	KRTDAP	0.136810919	-0.512323448	1.721443	-0.297613	0.765999	0.854157
17588	MYH6	0.118520618	-0.512323543	1.728375	-0.296419	0.76691	0.854685
17597	PAX1	0.113309481	-0.51232356	1.740255	-0.294396	0.768456	0.85597
17806	FOXI3	0.160842295	-0.51232386	2.089645	-0.245173	0.806323	0.887607
18094	TEKT1	0.118967852	-0.512325465	2.765243	-0.185273	0.853015	0.92406
18144	CRYBA2	0.097906484	-0.512326058	2.91407	-0.175811	0.860442	0.929333
18145	CXorf61	0.09670454	-0.512326058	2.91407	-0.175811	0.860442	0.929333
18147	MAS1	0.096249164	-0.512326058	2.91407	-0.175811	0.860442	0.929333
18148	MYL7	0.094225501	-0.512326058	2.91407	-0.175811	0.860442	0.929333
18143	CCDC140	0.093495985	-0.512326058	2.91407	-0.175811	0.860442	0.929333
17776	FRMPD2L1	0.328603526	-0.512388867	2.044674	-0.250597	0.802126	0.884477
7181	UBE2M	1108.124764	-0.512392957	0.136571	-3.751839	0.000176	0.000479
12169	GPR179	3.668267978	-0.512627162	0.268849	-1.906751	0.056553	0.091091
12831	PCDHGB3	26.75666123	-0.51265331	0.302389	-1.695345	0.09001	0.137502
4938	BAK1	482.6967205	-0.512733791	0.106706	-4.805119	1.55E-06	6.14E-06
16183	GADL1	0.90450342	-0.512843459	0.779317	-0.658068	0.510494	0.618316
5295	IFNGR2	2518.12167	-0.51328608	0.111006	-4.62396	3.76E-06	1.39E-05
7815	AP3S1	1295.949056	-0.514014091	0.147018	-3.496255	0.000472	0.001183
10427	ABCB6	991.5479751	-0.514200237	0.205607	-2.500891	0.012388	0.023288
7922	C1orf91	277.4524025	-0.514426389	0.149552	-3.439784	0.000582	0.00144
8506	PVRL1	436.6129377	-0.514490924	0.160645	-3.202653	0.001362	0.003138
9050	LRRC33	191.2019594	-0.514508944	0.171711	-2.996372	0.002732	0.005917
5844	TIMELESS	411.6183406	-0.514558121	0.118302	-4.349526	1.36E-05	4.58E-05
10054	DSCC1	71.88607276	-0.514687247	0.195392	-2.634122	0.008436	0.016446
6983	MRPL24	1208.63449	-0.514995966	0.134378	-3.832452	0.000127	0.000356
7473	C15orf39	849.432544	-0.515201623	0.142036	-3.62726	0.000286	0.000751
5297	MAF1	2307.176329	-0.515205924	0.111444	-4.622989	3.78E-06	1.40E-05
9124	C2orf7	350.7299082	-0.515207826	0.173607	-2.967672	0.003001	0.006446
5565	COMMD5	519.1851763	-0.515215668	0.114732	-4.490582	7.10E-06	2.50E-05
8982	CROCC	325.7603983	-0.515233357	0.170598	-3.02016	0.002526	0.005513
5439	PSMA7	2540.066778	-0.515526523	0.11335	-4.548082	5.41E-06	1.95E-05
7516	C13orf34	97.37786195	-0.515638398	0.142836	-3.61001	0.000306	0.000799
5954	SSH3	663.8418965	-0.515643488	0.120234	-4.288653	1.80E-05	5.92E-05
7738	CDK5RAP3	1856.17363	-0.516066582	0.146468	-3.523409	0.000426	0.001079
6745	RPS4X	26003.68397	-0.516126894	0.131591	-3.92219	8.77E-05	0.000255
7308	DCLRE1C	247.5242589	-0.51620775	0.139603	-3.697696	0.000218	0.000584
4739	PARP12	912.6782024	-0.51673941	0.105142	-4.914676	8.89E-07	3.68E-06
7435	DDB2	892.5100648	-0.516795168	0.141881	-3.642453	0.00027	0.000712
11217	PCDHB9	148.2819598	-0.517073918	0.233427	-2.215144	0.02675	0.046744
15144	FAM170B	0.517566727	-0.517157868	0.537889	-0.961458	0.336322	0.435304
12420	HKDC1	976.1470172	-0.517347955	0.282735	-1.829799	0.06728	0.106171
13302	TMEM155	31.06853099	-0.517352486	0.335401	-1.542488	0.122955	0.181179
6013	ZNF444	385.8401265	-0.517572203	0.121509	-4.259522	2.05E-05	6.68E-05
12673	DHH	6.31243936	-0.517872077	0.296534	-1.746418	0.080738	0.124876
11787	TSNAXIP1	59.91996398	-0.51793187	0.255529	-2.026904	0.042672	0.070966
10882	IRF8	482.1874635	-0.518087091	0.222493	-2.328558	0.019882	0.035813
2863	MIF4GD	476.7118983	-0.518100545	0.084478	-6.132956	8.63E-10	5.91E-09

6036	PUF60	1946.772323	-0.518148926	0.121907	-4.250349	2.13E-05	6.93E-05
10133	HSPA1B	1623.889621	-0.518409149	0.199084	-2.603975	0.009215	0.017825
6260	BSCL2	1886.5665	-0.518461271	0.124898	-4.15108	3.31E-05	0.000104
8846	KLF16	486.3790238	-0.518704194	0.168765	-3.073531	0.002115	0.004687
10443	SNX10	2942.661803	-0.518762618	0.207785	-2.496635	0.012538	0.023533
9650	SNHG1	597.2594456	-0.518769823	0.18772	-2.763532	0.005718	0.011615
8305	CDC42EP1	1286.362352	-0.518913999	0.157661	-3.291323	0.000997	0.002353
12093	FZp779M0	7.356544799	-0.519656708	0.269118	-1.930965	0.053487	0.086694
5252	STX16	2115.749424	-0.519723808	0.11176	-4.650372	3.31E-06	1.24E-05
3228	CANT1	1119.039533	-0.520270322	0.088668	-5.86759	4.42E-09	2.68E-08
14117	TMOD4	1.158271333	-0.520362196	0.405939	-1.281872	0.199888	0.277537
8206	TAS2R20	23.96866372	-0.520365	0.156296	-3.329362	0.00087	0.002079
7541	SH3KBP1	1011.880617	-0.520674555	0.14468	-3.598792	0.00032	0.000831
6860	PLD2	558.6472715	-0.520702245	0.134294	-3.877344	0.000106	0.000302
8079	TXNRD1	2403.824443	-0.521208301	0.154236	-3.379291	0.000727	0.001763
13582	LIME1	717.693965	-0.521216006	0.356867	-1.460533	0.144144	0.208022
7254	RNF126	497.0474783	-0.5212672	0.140002	-3.723274	0.000197	0.000531
7919	ZBTB12	61.86743662	-0.521324579	0.151509	-3.440874	0.00058	0.001435
6172	IQCE	671.1963461	-0.521339662	0.124458	-4.188875	2.80E-05	8.90E-05
12814	PKD1L3	1.396302728	-0.52152774	0.306644	-1.700758	0.088988	0.136121
3081	NOP56	1462.571237	-0.52168507	0.087293	-5.976236	2.28E-09	1.45E-08
10926	RARRES3	2365.626338	-0.521692599	0.225753	-2.310899	0.020838	0.037384
11317	C21orf63	427.9952562	-0.521855781	0.240045	-2.17399	0.029706	0.05145
7750	ZGPAT	619.7462145	-0.522284434	0.148428	-3.518761	0.000434	0.001096
12573	GSTT2	100.0652456	-0.5225711	0.293493	-1.780526	0.07499	0.116908
6945	BTBD2	1997.807455	-0.523151083	0.135987	-3.847054	0.00012	0.000337
4682	PDCD2L	81.96584338	-0.523297428	0.105931	-4.93998	7.81E-07	3.27E-06
9034	EBP	766.9772488	-0.523312625	0.174382	-3.000947	0.002691	0.00584
8518	LMNA	6790.123992	-0.523747426	0.163737	-3.198719	0.00138	0.003176
10959	EFCAB4B	45.72130728	-0.523772861	0.227692	-2.300359	0.021428	0.038322
4788	RAB13	2510.264939	-0.523817091	0.107069	-4.892323	9.97E-07	4.08E-06
5393	SQRDL	1922.22076	-0.523845379	0.114641	-4.569425	4.89E-06	1.78E-05
16818	HAO1	0.789221713	-0.524204862	1.077586	-0.486462	0.626639	0.730334
6798	POLR2F	634.9541201	-0.524389089	0.134446	-3.900379	9.60E-05	0.000277
5548	TRIM28	3409.299635	-0.52496777	0.116676	-4.499362	6.82E-06	2.41E-05
7569	F8A1	154.9959641	-0.524973132	0.146248	-3.589615	0.000331	0.000858
10996	SLC7A1	1926.89318	-0.525392623	0.229471	-2.289578	0.022046	0.039298
15978	CPA1	0.563519532	-0.525446834	0.73168	-0.718138	0.472672	0.57985
7917	RANGRF	316.1685829	-0.525581189	0.152713	-3.441635	0.000578	0.001432
4721	MED10	558.6442051	-0.525859537	0.10686	-4.921031	8.61E-07	3.57E-06
4525	RPL4	24433.64725	-0.526307153	0.104812	-5.021459	5.13E-07	2.22E-06
7031	C7orf43	264.2570064	-0.526547911	0.138277	-3.807934	0.00014	0.000391
12854	CDSN	39.85924564	-0.52669478	0.31163	-1.690128	0.091003	0.138771
13793	SLC45A2	11.88869984	-0.526724084	0.379351	-1.388487	0.164989	0.234463
9682	GRASP	289.4722956	-0.526827925	0.191394	-2.75258	0.005913	0.01197
9068	ANXA1	3865.539319	-0.526859762	0.176133	-2.991256	0.002778	0.006006
9172	CHN1	209.1215019	-0.526954236	0.178706	-2.94872	0.003191	0.006819

3887	MOGS	1350.353685	-0.527170415	0.097449	-5.40968	6.31E-08	3.18E-07
12734	ALDH3B2	4.353958082	-0.527659955	0.306394	-1.722163	0.08504	0.130899
5745	PARL	1133.094015	-0.527878764	0.119962	-4.400393	1.08E-05	3.69E-05
4809	PLEKHM2	1580.714951	-0.527962281	0.108101	-4.883964	1.04E-06	4.24E-06
6578	PRR3	252.1457594	-0.529101079	0.132555	-3.991548	6.56E-05	0.000196
8058	)C1001347	16.83177894	-0.52920244	0.156076	-3.390681	0.000697	0.001696
11949	CASC5	68.67519858	-0.529221907	0.267936	-1.975182	0.048247	0.079145
2984	TMEM41A	742.2215975	-0.529259551	0.087518	-6.047419	1.47E-09	9.67E-09
5846	USP5	1944.902618	-0.529567473	0.121793	-4.348092	1.37E-05	4.60E-05
7114	CPSF1	1187.575039	-0.52958917	0.140115	-3.77967	0.000157	0.000433
10195	MYCL1	71.85456112	-0.52971419	0.205027	-2.583626	0.009777	0.018797
7458	FAM86A	320.8046804	-0.530064409	0.145873	-3.633738	0.000279	0.000734
8961	MAP3K6	703.5746728	-0.530366656	0.175245	-3.02643	0.002475	0.005413
6076	C6orf48	1748.193303	-0.530757352	0.125456	-4.230615	2.33E-05	7.52E-05
10271	UNC5B	2105.414628	-0.530859075	0.207613	-2.556966	0.010559	0.020151
12204	FFAR2	11.33944456	-0.53122011	0.280311	-1.895112	0.058078	0.093279
8147	RPS29	4643.735741	-0.531785477	0.158571	-3.353616	0.000798	0.001919
14498	ARG1	1.202683354	-0.532002477	0.45535	-1.168339	0.24267	0.328063
12032	GPR34	410.6496388	-0.532597414	0.27285	-1.951977	0.050941	0.08298
13541	C6orf227	3.858773607	-0.532815525	0.361619	-1.473418	0.140638	0.203578
4434	EIF4A1	10203.0575	-0.533145953	0.104911	-5.081901	3.74E-07	1.65E-06
4214	43892	718.6779584	-0.533209699	0.102501	-5.202002	1.97E-07	9.17E-07
10904	RASD2	328.995051	-0.533266871	0.229924	-2.319321	0.020378	0.036631
14302	HIST2H3D	0.616772744	-0.533300057	0.433828	-1.229289	0.218964	0.300091
6654	KCTD13	258.3554366	-0.533300148	0.134642	-3.960877	7.47E-05	0.00022
8006	INTS1	2202.174241	-0.533757269	0.156642	-3.407504	0.000656	0.001605
5986	IFNAR2	685.0951089	-0.533878895	0.124865	-4.275659	1.91E-05	6.24E-05
10098	)C1001322	193.8565934	-0.53390149	0.204036	-2.616702	0.008878	0.017234
14792	UGT1A4	2.989215924	-0.534066222	0.498861	-1.070572	0.284362	0.37681
7595	PEX16	437.2033542	-0.534261069	0.149172	-3.581507	0.000342	0.000882
15447	MYBPHL	0.454774334	-0.53447949	0.61116	-0.874533	0.381828	0.484446
3976	RPN2	8142.117108	-0.534603378	0.099829	-5.355176	8.55E-08	4.21E-07
12223	SCN5A	5.950319762	-0.534836926	0.282896	-1.890576	0.058681	0.094102
8338	PRKAR1B	389.8625479	-0.534897133	0.163193	-3.277694	0.001047	0.00246
6407	FLYWCH2	312.6853778	-0.534982846	0.13132	-4.073882	4.62E-05	0.000141
4457	PDIA3	13110.01929	-0.53543484	0.105733	-5.06403	4.10E-07	1.81E-06
14272	KCNJ9	1.363102517	-0.535511982	0.433166	-1.236274	0.216357	0.297142
10066	C6orf150	68.22138046	-0.535644852	0.203619	-2.630619	0.008523	0.016596
10017	HLA-L	74.97131863	-0.535891919	0.202532	-2.645965	0.008146	0.01594
2813	CHMP4B	3287.506923	-0.535904759	0.086764	-6.176611	6.55E-10	4.56E-09
11524	WISP1	205.2452475	-0.536145754	0.254167	-2.109423	0.034908	0.059375
3071	BLOC1S3	325.5524612	-0.536249808	0.089649	-5.981682	2.21E-09	1.41E-08
10446	NAALADL	51.47764791	-0.536434716	0.21495	-2.49563	0.012573	0.023593
13736	PCDHA2	20.47426581	-0.536462716	0.381708	-1.405426	0.159895	0.228166
7403	MLST8	916.4433676	-0.536475162	0.146722	-3.656397	0.000256	0.000677
8970	ENGASE	441.6333605	-0.536549677	0.17747	-3.023325	0.0025	0.005463
6845	NOP16	288.6202416	-0.536691524	0.138265	-3.881602	0.000104	0.000297

11006	PTPRC	1797.319032	-0.536772302	0.234617	-2.287866	0.022145	0.039439
8130	RPL23P8	110.649454	-0.536845198	0.159792	-3.359649	0.00078	0.001882
4174	DNAJB11	1428.523323	-0.536981181	0.102596	-5.233963	1.66E-07	7.79E-07
2513	SNRPC	865.4753426	-0.537025265	0.083244	-6.451182	1.11E-10	8.66E-10
4939	CAPNS1	8350.53809	-0.537568259	0.111884	-4.804704	1.55E-06	6.15E-06
5851	ZNF213	364.2240735	-0.537681912	0.123722	-4.345898	1.39E-05	4.65E-05
5079	ELF4	811.10093	-0.537688709	0.113616	-4.732516	2.22E-06	8.56E-06
1882	ATG7	699.9546295	-0.538111755	0.075734	-7.105297	1.20E-12	1.25E-11
13489	TMEM63C	26.01421571	-0.538854745	0.361413	-1.490967	0.13597	0.197579
4527	PSMB1	2811.052622	-0.538888213	0.107353	-5.019766	5.17E-07	2.24E-06
10774	SOX18	420.4077696	-0.539047391	0.227049	-2.37415	0.017589	0.032
7461	CCDC101	296.5806457	-0.539393447	0.148491	-3.632508	0.000281	0.000737
16401	PCDH15	0.597022438	-0.539423755	0.898077	-0.600643	0.548078	0.65499
14911	C1orf105	0.744529116	-0.539557318	0.52244	-1.032763	0.301715	0.396587
8604	MCM4	588.413637	-0.539705841	0.170042	-3.173965	0.001504	0.003426
11940	PPIEL	101.1846227	-0.539913541	0.27289	-1.978504	0.047872	0.078588
9950	HOMER3	852.4907052	-0.54015525	0.202228	-2.671021	0.007562	0.014895
7558	ORAI1	365.2346006	-0.540387107	0.15041	-3.592757	0.000327	0.000849
10063	TBXA2R	163.8117366	-0.540747141	0.205482	-2.631605	0.008498	0.016553
7860	GRN	8537.796869	-0.540851476	0.155977	-3.467519	0.000525	0.00131
9868	C19orf48	831.7653097	-0.541148831	0.200583	-2.697876	0.006978	0.013861
9448	PFKFB3	4562.068236	-0.541423812	0.190731	-2.838672	0.00453	0.009398
6510	SNX21	453.606851	-0.541712841	0.134634	-4.023602	5.73E-05	0.000173
12661	DRD4	10.96190453	-0.542348904	0.309337	-1.75326	0.079557	0.123166
9641	CCDC12	442.7207522	-0.542559192	0.19615	-2.766042	0.005674	0.011536
13530	MKX	10.11858608	-0.542575463	0.367428	-1.476684	0.13976	0.202472
13543	CGB7	2.120861059	-0.542734983	0.368506	-1.472798	0.140806	0.20379
3657	TXNL4B	280.478027	-0.54295437	0.097551	-5.565878	2.61E-08	1.40E-07
3904	RPL23AP82	128.4631678	-0.542980154	0.100505	-5.402502	6.57E-08	3.30E-07
13671	SGCD	140.8692363	-0.543248355	0.37948	-1.43156	0.15227	0.218319
11663	RDH5	404.7967784	-0.543421527	0.263962	-2.058714	0.039522	0.066417
5431	ZBTB8OS	185.3281768	-0.5436127	0.119413	-4.552356	5.30E-06	1.91E-05
4592	NSMCE1	1230.810241	-0.543623704	0.108926	-4.990747	6.01E-07	2.57E-06
10434	GJD3	122.1752283	-0.544359758	0.217806	-2.499291	0.012444	0.023375
3683	PPIA	5138.091842	-0.544527686	0.098149	-5.547965	2.89E-08	1.54E-07
7449	EPB41	1594.751403	-0.544951022	0.149814	-3.637509	0.000275	0.000724
4760	MRPL51	1651.60948	-0.545391079	0.111206	-4.90434	9.37E-07	3.86E-06
8309	DPM2	840.7112863	-0.545449562	0.165855	-3.288718	0.001006	0.002374
7451	WDR74	601.6009367	-0.545452542	0.149983	-3.636756	0.000276	0.000726
4348	PSMC3	2299.25634	-0.545677911	0.106326	-5.132128	2.86E-07	1.29E-06
6642	C8orf59	581.2755878	-0.545744876	0.137582	-3.966696	7.29E-05	0.000215
11890	CRB1	7.980306539	-0.545750702	0.274021	-1.991639	0.046411	0.076509
7001	THAP8	140.6558787	-0.54596686	0.142786	-3.823683	0.000131	0.000368
6639	TPRA1	665.1904614	-0.546209303	0.137656	-3.967931	7.25E-05	0.000214
2797	ACOT9	518.3931345	-0.546317877	0.088214	-6.193098	5.90E-10	4.13E-09
12730	FLJ45445	21.97858843	-0.546440271	0.317092	-1.723288	0.084836	0.130631
9753	ACSL5	793.8293652	-0.54644042	0.200034	-2.731736	0.0063	0.012662

12171	NAGS	107.2642611	-0.546550983	0.286757	-1.905974	0.056654	0.091239
2201	UBE2I	1694.528839	-0.546684014	0.080925	-6.75543	1.42E-11	1.27E-10
8896	CARD10	911.6844757	-0.547047987	0.179129	-3.053933	0.002259	0.004977
5620	ARHGDIA	6000.485494	-0.547117681	0.122618	-4.461978	8.12E-06	2.83E-05
14665	VCX3A	1.412574163	-0.547235685	0.492429	-1.111299	0.26644	0.356119
6298	ITFG3	2335.242247	-0.547297369	0.132577	-4.128161	3.66E-05	0.000114
7477	ANKZF1	835.8562013	-0.547528177	0.15109	-3.623862	0.00029	0.000761
8199	FAM89A	156.1082764	-0.54795629	0.164391	-3.33324	0.000858	0.002052
12969	LOC401387	1.280909125	-0.548132317	0.331659	-1.6527	0.098392	0.148707
4580	DHX37	504.8074995	-0.548405153	0.109761	-4.996338	5.84E-07	2.50E-06
11516	RPL21	6.580402774	-0.548421243	0.259821	-2.110769	0.034792	0.059213
16398	CXCL17	0.397688087	-0.548447329	0.911841	-0.601472	0.547525	0.654441
3948	C11orf73	382.0312171	-0.54872928	0.102053	-5.376914	7.58E-08	3.76E-07
11508	GSTP1	6457.117949	-0.548734785	0.259645	-2.113404	0.034566	0.058875
13560	CCDC154	9.165085969	-0.548802194	0.374073	-1.467098	0.14235	0.205766
9108	WASH5P	224.2728046	-0.548941595	0.184516	-2.975027	0.00293	0.006305
9823	DPP7	2799.283914	-0.549065114	0.202571	-2.71048	0.006719	0.013406
13829	PRDM12	1.316908061	-0.549073512	0.399216	-1.375381	0.169013	0.239557
7025	ATIC	1571.804604	-0.549163513	0.144063	-3.811965	0.000138	0.000385
5632	RPS25	9642.261718	-0.549341403	0.12334	-4.453887	8.43E-06	2.93E-05
9239	KAT2A	864.5173238	-0.549749366	0.188077	-2.923004	0.003467	0.007355
6185	PDIA6	8337.47724	-0.549785155	0.131445	-4.182637	2.88E-05	9.13E-05
12639	LOC647946	8.172614195	-0.549941639	0.312256	-1.761189	0.078206	0.121285
4754	SLC10A3	745.799349	-0.550305714	0.112147	-4.90699	9.25E-07	3.81E-06
12640	ALDH1L2	257.270907	-0.55056253	0.312808	-1.760066	0.078397	0.121571
7145	PREX1	1721.912154	-0.550573011	0.146217	-3.765461	0.000166	0.000456
8084	STARD5	68.60945671	-0.550784798	0.163066	-3.377679	0.000731	0.001772
15438	LOC150568	1.27973427	-0.551136509	0.628595	-0.876775	0.380609	0.483244
8999	DAAM2	860.3951443	-0.551139338	0.182948	-3.012551	0.002591	0.005643
12360	GRM2	7.791276724	-0.551616463	0.29858	-1.847465	0.06468	0.102572
8493	TNK2	756.5231972	-0.551620702	0.17184	-3.210084	0.001327	0.003062
10497	ADM	5018.768512	-0.551694021	0.223167	-2.472116	0.013432	0.025081
12256	TMEM90A	9.36251924	-0.551731479	0.293342	-1.880848	0.059993	0.095938
8996	MCAM	7681.850023	-0.551812406	0.183109	-3.013576	0.002582	0.005626
4901	USF1	819.5326325	-0.551818572	0.114344	-4.825954	1.39E-06	5.57E-06
5735	ALKBH4	286.0080252	-0.552180678	0.125348	-4.405195	1.06E-05	3.61E-05
7166	MPZ	41.2727336	-0.552445828	0.147038	-3.757162	0.000172	0.00047
6759	FAM96B	1113.173771	-0.552633549	0.141119	-3.916093	9.00E-05	0.000261
6762	HSCB	265.4023992	-0.552652759	0.141181	-3.914504	9.06E-05	0.000263
6130	HMG20B	1184.817599	-0.552711061	0.131364	-4.207474	2.58E-05	8.26E-05
7845	RHBDD2	2176.298738	-0.552780289	0.159048	-3.475547	0.00051	0.001274
6281	ZMAT5	336.0009964	-0.552820534	0.133545	-4.139589	3.48E-05	0.000109
9652	STK17B	949.2319944	-0.552831555	0.200059	-2.763336	0.005721	0.011619
6937	HRAS	569.1884952	-0.552875388	0.14365	-3.848769	0.000119	0.000335
8004	OAF	1903.408038	-0.552925233	0.162215	-3.408587	0.000653	0.001599
11447	SLFNL1	16.01457244	-0.552946157	0.258939	-2.13543	0.032726	0.056037
14801	CYP24A1	143.2694541	-0.552946413	0.519118	-1.065164	0.286802	0.379812

2978	IFT20	331.5583716	-0.552973959	0.091392	-6.050595	1.44E-09	9.50E-09
5850	DKN2AIPN	446.3020821	-0.553291264	0.12728	-4.347047	1.38E-05	4.62E-05
6957	ELL3	103.7691691	-0.553682822	0.144081	-3.842847	0.000122	0.000343
6669	LSM4	1211.995695	-0.553785046	0.140042	-3.954413	7.67E-05	0.000225
2914	ZNF282	608.1415133	-0.553866677	0.090787	-6.100732	1.06E-09	7.10E-09
13955	DPCR1	4.820223415	-0.553902365	0.415602	-1.33277	0.182607	0.256454
15542	MYADML2	0.651335027	-0.554104025	0.653027	-0.848516	0.396151	0.499633
5057	FAM127B	1049.377359	-0.55428767	0.116852	-4.743506	2.10E-06	8.14E-06
10742	CD69	185.7294265	-0.554372879	0.232609	-2.383284	0.017159	0.03131
10181	TYRO3	212.8300213	-0.554530302	0.214162	-2.589297	0.009617	0.018516
10351	CCDC57	380.8561898	-0.554547593	0.219358	-2.528046	0.01147	0.02172
7433	SYDE1	813.3964185	-0.554718603	0.15223	-3.64394	0.000268	0.000708
11608	HLA-DQB2	216.8383725	-0.554912739	0.266915	-2.078984	0.037619	0.063522
12164	EFCAB1	3.040287103	-0.554924033	0.2909	-1.907612	0.056441	0.090953
14562	AKR1CL1	1.328384659	-0.554945138	0.483526	-1.147705	0.25109	0.337977
7628	POLR2L	1333.184886	-0.555010807	0.155542	-3.568243	0.000359	0.000923
4318	C2CD2L	256.9368535	-0.555026642	0.107841	-5.14671	2.65E-07	1.20E-06
11055	EGFLAM	362.5126348	-0.555086865	0.244242	-2.272695	0.023045	0.040859
11331	TSPAN32	24.54969301	-0.556003502	0.256157	-2.170558	0.029965	0.051834
9391	NES	2267.288471	-0.556091429	0.194307	-2.861924	0.004211	0.008789
15172	CDH22	22.02164665	-0.556542143	0.582401	-0.9556	0.339274	0.438299
8087	ALKBH6	112.7752487	-0.556790804	0.164938	-3.37575	0.000736	0.001784
5172	DDX54	1512.746022	-0.556956045	0.118777	-4.689074	2.74E-06	1.04E-05
14344	HCG9	0.719462652	-0.556988555	0.458007	-1.216114	0.223942	0.306015
1742	PPM1G	2157.996727	-0.557038119	0.076811	-7.252037	4.11E-13	4.62E-12
11321	IL24	2.912418044	-0.557180397	0.256409	-2.173017	0.029779	0.051559
10444	SOX15	9.438057434	-0.557470964	0.223309	-2.496415	0.012546	0.023545
13366	DLX2	3.962534977	-0.557482939	0.366321	-1.521844	0.128048	0.18778
10184	CEBPD	2835.069346	-0.557584528	0.215521	-2.587153	0.009677	0.018624
8918	NUSAP1	424.8626916	-0.557855005	0.183237	-3.044438	0.002331	0.005124
11914	AQP3	2845.57986	-0.558259086	0.281425	-1.983685	0.047291	0.077803
8517	HYI	606.1448428	-0.55832022	0.174535	-3.198905	0.00138	0.003175
9047	TIAM1	221.7966312	-0.558420503	0.186349	-2.996634	0.00273	0.005914
9541	HESX1	12.9739501	-0.558607751	0.199305	-2.80278	0.005066	0.010408
9876	SEMA6B	691.0128939	-0.558753043	0.207285	-2.695573	0.007027	0.013946
8489	HSFX2	29.52256171	-0.55878759	0.173982	-3.211754	0.001319	0.003046
14623	OC1001923	1.615057522	-0.559154113	0.496838	-1.125426	0.260409	0.349058
8336	ANKS3	461.1173856	-0.559389423	0.17065	-3.277988	0.001046	0.002458
6355	DGAT1	642.9720382	-0.559922375	0.136595	-4.099133	4.15E-05	0.000128
10918	OC1001322	235.7222398	-0.559960274	0.242087	-2.313049	0.02072	0.037198
7039	DBF4B	90.56746951	-0.559988752	0.147209	-3.804028	0.000142	0.000396
10018	HOXA1	38.1927179	-0.559989595	0.211655	-2.645767	0.008151	0.015947
5429	MFSD5	865.3006806	-0.560040659	0.123007	-4.55291	5.29E-06	1.91E-05
8330	VASH1	1253.392386	-0.560351301	0.170813	-3.280493	0.001036	0.002438
7519	RBM38	431.7270524	-0.560716319	0.155394	-3.60836	0.000308	0.000803
4017	HAUS5	292.0553411	-0.560743776	0.105224	-5.329048	9.87E-08	4.82E-07
3303	PTP4A2	2151.094072	-0.560779954	0.09647	-5.812975	6.14E-09	3.64E-08

13681	RBM46	8.954116296	-0.560984697	0.393067	-1.427198	0.153523	0.219955
8160	GIPC1	1409.703058	-0.561308981	0.16766	-3.347901	0.000814	0.001956
6463	ZFPL1	524.2787567	-0.561439528	0.138835	-4.043919	5.26E-05	0.000159
13421	RPL23AP32	3.749591844	-0.561485952	0.372432	-1.50762	0.131652	0.192274
6848	POP5	347.1950154	-0.561562155	0.144734	-3.87997	0.000104	0.000299
11712	LOC72898	4.31161449	-0.561717321	0.274109	-2.049247	0.040438	0.067676
4406	TOR3A	907.1617164	-0.562262125	0.110282	-5.098388	3.43E-07	1.52E-06
15269	IL26	0.436782064	-0.562343408	0.605684	-0.928444	0.353177	0.453378
8074	NR2F6	752.4678496	-0.563455076	0.166549	-3.383119	0.000717	0.00174
8831	DEGS1	4059.656612	-0.563660736	0.183122	-3.078069	0.002083	0.004624
5495	HMOX2	1181.600227	-0.563806901	0.124613	-4.524471	6.05E-06	2.16E-05
7367	WDR18	588.9343964	-0.564034938	0.153645	-3.671026	0.000242	0.000643
11769	C15orf54	4.065877728	-0.564298433	0.278016	-2.029733	0.042384	0.070589
5406	C11orf49	570.9904872	-0.564727552	0.1238	-4.561594	5.08E-06	1.84E-05
10448	DTX4	731.9540763	-0.565384741	0.226599	-2.495085	0.012593	0.023625
10608	ZCCHC18	11.24860566	-0.565410408	0.232309	-2.433877	0.014938	0.027602
11859	LRRC24	16.52408726	-0.565475441	0.282347	-2.002768	0.045202	0.074707
8610	FAH	1563.543582	-0.565518653	0.178373	-3.170423	0.001522	0.003465
5237	SUV39H1	209.1680633	-0.565719961	0.121463	-4.657557	3.20E-06	1.20E-05
10202	C3orf47	25.43028007	-0.566048627	0.219282	-2.581377	0.009841	0.018907
5832	GRINA	6260.360248	-0.566590489	0.130085	-4.355548	1.33E-05	4.46E-05
9104	XYLT1	217.7579542	-0.566685431	0.190441	-2.975649	0.002924	0.006295
11976	C6orf174	266.3055871	-0.566957587	0.288343	-1.966261	0.049268	0.080637
10681	ZNF300	121.9597985	-0.567017825	0.236036	-2.402249	0.016295	0.029903
9549	SPN	300.9573734	-0.5672585	0.202466	-2.801751	0.005083	0.010433
13764	NEGR1	196.9850927	-0.567339339	0.405926	-1.397641	0.162221	0.231015
3711	C2orf28	2742.938289	-0.567605446	0.102614	-5.531468	3.18E-08	1.68E-07
10520	PTGDR	28.53022714	-0.567756812	0.230429	-2.463908	0.013743	0.025606
11020	LILRA1	24.02091309	-0.567778218	0.248575	-2.284135	0.022364	0.039778
8947	GPR124	1664.887307	-0.568063228	0.187253	-3.033665	0.002416	0.005292
14555	LPPR3	0.715642562	-0.568096766	0.494008	-1.149974	0.250154	0.336879
6110	HDGFRP2	907.4147249	-0.568106584	0.134743	-4.216235	2.48E-05	7.97E-05
5610	NCOR2	4254.40522	-0.568190891	0.127184	-4.467483	7.91E-06	2.77E-05
8157	LOC38815	222.6073788	-0.568256057	0.169708	-3.34843	0.000813	0.001953
11910	FAM46C	503.5418028	-0.568582436	0.286478	-1.984732	0.047174	0.077636
10186	CRNA000	67.5291061	-0.568774866	0.219883	-2.586714	0.00969	0.018646
7826	FTHL3	306.2769883	-0.568961115	0.162943	-3.491785	0.00048	0.001202
4723	TADA3	1488.47617	-0.569010913	0.115636	-4.920715	8.62E-07	3.58E-06
7740	GNAS	28657.60161	-0.56906827	0.161517	-3.523266	0.000426	0.001079
10991	C3orf14	123.37978	-0.569526924	0.248709	-2.289929	0.022025	0.039269
7545	C17orf58	276.8337469	-0.569638925	0.158326	-3.597895	0.000321	0.000833
7533	NUBP2	808.9642057	-0.569714161	0.158146	-3.602453	0.000315	0.00082
15552	TP53TG5	0.320939511	-0.569987911	0.673829	-0.845894	0.397612	0.501131
15902	SNORA31	0.303991369	-0.570030152	0.768305	-0.741932	0.458128	0.564719
15897	LOC28520	0.24689311	-0.570030203	0.766522	-0.743658	0.457083	0.563584
16561	LOC15052	0.291131579	-0.570030243	1.024693	-0.556293	0.57801	0.684112
16664	ATP4A	0.252305042	-0.570030289	1.074881	-0.53032	0.59589	0.700915

17158	OLFM3	0.243228006	-0.570030429	1.437561	-0.396526	0.691717	0.790196
16884	FOLR4	0.1683441	-0.57003051	1.216211	-0.468694	0.639289	0.742122
16891	C17orf98	0.163195523	-0.570030512	1.21944	-0.467453	0.640176	0.742886
17039	CACNG3	0.180070398	-0.570030531	1.322366	-0.431069	0.666419	0.766622
17304	C6orf58	0.163629735	-0.570030765	1.559077	-0.365621	0.714648	0.809445
17313	LY6G6F	0.163382596	-0.57003077	1.565785	-0.364054	0.715818	0.810428
17568	NKX2-1	0.127344129	-0.570031294	1.903867	-0.299407	0.764629	0.853233
17594	HOXD12	0.105876606	-0.570031439	1.933765	-0.294778	0.768163	0.855826
17593	DGAT2L6	0.107047864	-0.57003144	1.934377	-0.294685	0.768235	0.855826
17676	TECRL	0.153586822	-0.570031523	2.080494	-0.273989	0.784093	0.869485
17702	NKX6-3	0.124917088	-0.570031599	2.135562	-0.266923	0.789528	0.874225
17717	WFDC11	0.104003639	-0.570031779	2.16732	-0.263012	0.792541	0.876819
17811	TMEM114	0.154872397	-0.570031891	2.335766	-0.244045	0.807196	0.888319
17825	FLJ36000	0.138688103	-0.570031943	2.370024	-0.240517	0.809929	0.890617
18051	IAPP	0.122299339	-0.57003288	2.91407	-0.195614	0.844912	0.917412
18011	MAGEA11	0.107912093	-0.570032897	2.801499	-0.203474	0.838764	0.912703
18023	OR52K2	0.103060934	-0.570032948	2.827042	-0.201636	0.840201	0.913765
18050	GAGE4	0.106374994	-0.570033125	2.91407	-0.195614	0.844912	0.917412
18052	OR1D4	0.147444556	-0.570033125	2.91407	-0.195614	0.844912	0.917412
5760	TTLL12	738.8119918	-0.570182999	0.129818	-4.392175	1.12E-05	3.82E-05
12179	EGR3	214.355447	-0.570331188	0.299682	-1.903118	0.057025	0.091777
11863	SH2D1A	108.7328539	-0.570718117	0.285032	-2.002294	0.045253	0.074771
3579	HNRNPAB	2557.367659	-0.570947741	0.101637	-5.617511	1.94E-08	1.06E-07
8503	PPP1R16A	977.6627587	-0.571636955	0.178391	-3.204408	0.001353	0.00312
13172	OC1001336	20.50051955	-0.571768518	0.359876	-1.588791	0.112108	0.166825
6951	MRPL14	631.8598375	-0.571909363	0.14874	-3.845037	0.000121	0.00034
7117	PIK3CD	595.7024108	-0.572046947	0.151399	-3.778412	0.000158	0.000435
11007	SHC2	421.3321652	-0.572505475	0.250281	-2.287448	0.02217	0.039479
4211	ARPC1A	3177.056461	-0.572606115	0.110059	-5.20274	1.96E-07	9.14E-07
7133	CD40	989.0796552	-0.573122049	0.151934	-3.772167	0.000162	0.000445
8282	C17orf61	607.0609583	-0.573297866	0.173725	-3.300038	0.000967	0.002288
5962	SRA1	775.5725623	-0.573386683	0.133786	-4.285865	1.82E-05	5.98E-05
4202	CHAF1A	221.9896678	-0.573524333	0.11016	-5.206269	1.93E-07	8.99E-07
2706	DLGAP4	2076.777856	-0.57381623	0.091455	-6.27428	3.51E-10	2.54E-09
5900	HYOU1	4246.841947	-0.573846178	0.1328	-4.321146	1.55E-05	5.16E-05
12395	ITIH5	3654.864449	-0.57493363	0.312936	-1.837222	0.066177	0.10465
6045	SLC15A4	1894.684153	-0.574976359	0.135385	-4.246965	2.17E-05	7.03E-05
4216	AUP1	2350.344344	-0.575214082	0.110606	-5.200573	1.99E-07	9.24E-07
11839	ALDOC	1660.731404	-0.575338124	0.286413	-2.008769	0.044562	0.073777
3331	C1orf123	769.9612897	-0.575569139	0.099304	-5.796028	6.79E-09	4.00E-08
12010	PYHIN1	78.08560347	-0.57565287	0.293957	-1.958286	0.050196	0.081923
6636	GARS	2449.364925	-0.575718568	0.145023	-3.969844	7.19E-05	0.000212
10985	TMEM121	32.02810421	-0.575878149	0.251263	-2.291937	0.021909	0.039094
9659	LZTS1	830.8675438	-0.575940594	0.208551	-2.761635	0.005751	0.01167
5748	NUDC	1931.07896	-0.575957123	0.130958	-4.398027	1.09E-05	3.73E-05
4843	KIAA2013	1454.150023	-0.575975361	0.11828	-4.869589	1.12E-06	4.53E-06
2713	APEX2	509.061241	-0.576225658	0.091937	-6.267602	3.67E-10	2.65E-09

10050	CRIP2	3133.496502	-0.576245462	0.218668	-2.635253	0.008407	0.016397
6128	UBXN1	1811.428776	-0.576279654	0.136945	-4.208112	2.58E-05	8.24E-05
13817	TDH	0.795818967	-0.576370424	0.417618	-1.380139	0.167544	0.23768
12709	KMO	1205.520847	-0.57684457	0.333548	-1.72942	0.083734	0.129142
8391	POMZP3	188.2515922	-0.576896118	0.17732	-3.25341	0.00114	0.002664
15191	LGALS7	0.383230468	-0.577179471	0.606895	-0.951036	0.341586	0.44075
13602	TFAP2A	109.7744558	-0.577588076	0.396863	-1.455386	0.145562	0.20973
4443	SUMF2	3766.031467	-0.577767072	0.113802	-5.076957	3.84E-07	1.69E-06
7011	)C1002707	29.75118466	-0.577878775	0.151333	-3.818598	0.000134	0.000375
1354	ARPC2	4442.299217	-0.578077672	0.07423	-7.787661	6.83E-15	9.88E-14
8115	IRF5	533.9490127	-0.578105238	0.171872	-3.363576	0.000769	0.001858
6230	TELO2	537.2170446	-0.57816105	0.138848	-4.163978	3.13E-05	9.84E-05
8714	ACAP3	570.7081071	-0.578190031	0.185015	-3.125104	0.001777	0.003998
11643	DCLK1	601.9891196	-0.578557202	0.280445	-2.062997	0.039113	0.065847
14945	FUT5	0.726366397	-0.579467343	0.566139	-1.023542	0.306051	0.401399
5681	C17orf101	360.0908044	-0.579542132	0.130784	-4.431294	9.37E-06	3.23E-05
3753	ST3GAL2	842.0115085	-0.579651449	0.105301	-5.504733	3.70E-08	1.93E-07
6858	GPR137	634.355861	-0.580216533	0.149609	-3.878218	0.000105	0.000301
12836	SEC14L4	20.23729034	-0.580471635	0.342745	-1.693595	0.090342	0.137959
2315	DYNLL1	3714.770275	-0.580512068	0.087399	-6.642093	3.09E-11	2.62E-10
5992	RXRA	2114.095857	-0.580712848	0.135899	-4.273131	1.93E-05	6.31E-05
15213	GDF1	2.214746207	-0.580742272	0.614986	-0.944317	0.345007	0.444521
11579	ADRA2C	100.1716997	-0.580937518	0.278048	-2.089344	0.036677	0.062087
13490	NOX1	1.01051872	-0.581013626	0.389749	-1.490737	0.136031	0.197653
14863	RFPL1	0.829553468	-0.58133786	0.554347	-1.048689	0.294321	0.388145
5333	THOC3	604.5932096	-0.581416876	0.12623	-4.605997	4.10E-06	1.51E-05
10167	HSD17B7P2	48.4289488	-0.581659158	0.224272	-2.593546	0.009499	0.018314
9323	)C1002887	458.6308375	-0.581750709	0.201216	-2.89118	0.003838	0.008069
7682	HOXC6	272.1343578	-0.581763509	0.164047	-3.546321	0.000391	0.000997
5549	TAPBP	8172.53805	-0.581865808	0.129326	-4.499216	6.82E-06	2.41E-05
9737	SDHAP3	443.8597033	-0.581964944	0.212595	-2.73743	0.006192	0.012465
5961	RAD9B	27.14657491	-0.582140807	0.135827	-4.285902	1.82E-05	5.98E-05
9989	DUSP1	19627.54351	-0.582227165	0.219194	-2.656224	0.007902	0.015506
11216	)C1001331	21.45684448	-0.58230424	0.262839	-2.215444	0.02673	0.046716
9865	HEYL	1720.262607	-0.582375545	0.215819	-2.698441	0.006967	0.013842
9442	FANCB	10.75233213	-0.582765893	0.205148	-2.840708	0.004501	0.009345
17265	FAM27B	1.174600972	-0.58288854	1.55708	-0.374347	0.708146	0.803799
5288	PPP1CA	3302.346011	-0.58296507	0.125938	-4.628975	3.67E-06	1.36E-05
8420	FNDC8	7.038651922	-0.583014166	0.179781	-3.242921	0.001183	0.002754
11683	ANKRD55	5.083136914	-0.583375913	0.283853	-2.055206	0.039859	0.066873
6581	C8orf58	286.3580849	-0.583435029	0.146193	-3.990864	6.58E-05	0.000196
6162	PNPLA6	1214.052575	-0.583657373	0.139101	-4.19594	2.72E-05	8.64E-05
5325	PCYOX1L	257.8536347	-0.58366797	0.126604	-4.610197	4.02E-06	1.48E-05
4780	EIF3F	2696.179056	-0.583766964	0.119245	-4.895536	9.80E-07	4.02E-06
9836	SLC8A1	669.0475401	-0.584103941	0.21573	-2.707566	0.006778	0.013506
7037	ECE1	4304.365316	-0.584105495	0.153509	-3.805019	0.000142	0.000395
10021	S1PR5	75.36863943	-0.584203542	0.220898	-2.64467	0.008177	0.015994

13311	CYP27C1	2.957596226	-0.584403358	0.379623	-1.539429	0.123699	0.182153
12671	APOL5	1.950946603	-0.584450311	0.33448	-1.747339	0.080579	0.124648
7799	TRMT1	615.6235184	-0.584530889	0.166905	-3.50218	0.000461	0.00116
2987	CBX4	363.6925068	-0.584574159	0.096688	-6.046016	1.48E-09	9.74E-09
5076	MIER2	356.9868382	-0.584973184	0.12358	-4.733544	2.21E-06	8.52E-06
12254	CXorf50B	4.544005027	-0.58498677	0.310939	-1.881352	0.059924	0.095852
10667	CXorf21	64.7467647	-0.585162141	0.243272	-2.405386	0.016155	0.029686
3588	AATF	1247.871107	-0.585201374	0.104283	-5.611647	2.00E-08	1.09E-07
10246	SLIT3	1427.864198	-0.585514433	0.228149	-2.566372	0.010277	0.01966
15209	NPFFR2	1.273384193	-0.585623954	0.619759	-0.944922	0.344699	0.444239
7825	DGCR6L	819.6091246	-0.585762203	0.167739	-3.492096	0.000479	0.0012
13593	PCYT1B	15.08782093	-0.585947524	0.402063	-1.457351	0.14502	0.209117
7570	RAB3D	838.1552185	-0.586007141	0.163255	-3.589528	0.000331	0.000858
6103	MRPS15	1127.911013	-0.586040105	0.138849	-4.22071	2.44E-05	7.82E-05
12937	HIST1H2BC	17.29080508	-0.586288911	0.352464	-1.663401	0.096232	0.145803
11230	OC1001314	4.129803103	-0.586538738	0.265642	-2.208001	0.027244	0.047552
9603	NAMPT	4496.7265	-0.586609495	0.211053	-2.779436	0.005445	0.011115
10928	ESCO2	36.02118463	-0.587065816	0.254116	-2.310225	0.020876	0.037444
12896	GPRIN2	153.3543751	-0.587656261	0.351107	-1.673725	0.094185	0.143154
4280	PPP4C	1491.674702	-0.588277224	0.11386	-5.16668	2.38E-07	1.09E-06
8045	LRRC29	123.7151699	-0.58830765	0.173316	-3.39442	0.000688	0.001676
8775	CCDC18	50.52777917	-0.588377049	0.190198	-3.0935	0.001978	0.004418
14751	GALNT5	58.90301016	-0.588734367	0.543311	-1.083605	0.27854	0.370122
6773	RARG	458.4968157	-0.5888109	0.150615	-3.90937	9.25E-05	0.000268
15870	C14orf162	0.741874782	-0.58920481	0.782377	-0.753096	0.451392	0.557514
8840	TMEM71	46.48635959	-0.589439061	0.191722	-3.074443	0.002109	0.004676
13888	TPSD1	6.836798319	-0.589748769	0.436317	-1.351651	0.176487	0.249087
10531	AKR1E2	18.08832772	-0.589856028	0.23979	-2.459884	0.013898	0.025868
4258	KDELR2	6442.16254	-0.589893	0.113907	-5.178714	2.23E-07	1.03E-06
16060	PSG4	3.415565739	-0.589981932	0.848837	-0.695048	0.487026	0.594408
11468	OC1001281	2.699472004	-0.590066862	0.277105	-2.129395	0.033222	0.056782
4057	XKR8	199.679181	-0.590264429	0.111331	-5.301882	1.15E-07	5.54E-07
8248	LTB4R2	67.7260886	-0.590265394	0.178148	-3.313345	0.000922	0.002191
10304	OC1002708	34.71034485	-0.590597423	0.232175	-2.543765	0.010966	0.020861
3455	IL10RB	1268.241055	-0.590833968	0.103584	-5.703928	1.17E-08	6.64E-08
14761	CXCL6	127.5860071	-0.590839027	0.546303	-1.081522	0.279465	0.371099
11973	TG	44.86400494	-0.590896275	0.30025	-1.968015	0.049066	0.080326
6502	JMJD6	705.1186755	-0.591167869	0.146805	-4.026894	5.65E-05	0.00017
9896	SPRN	94.95632889	-0.591190329	0.219842	-2.689155	0.007163	0.014188
4375	RBX1	1027.5952	-0.591250849	0.115521	-5.118104	3.09E-07	1.38E-06
3205	UBE2F	585.6037536	-0.591286323	0.100537	-5.881284	4.07E-09	2.49E-08
12994	SEC14L5	29.08162083	-0.591403255	0.359217	-1.64637	0.099688	0.150383
13693	C3orf55	64.24374724	-0.592247907	0.417323	-1.41916	0.155852	0.223097
14619	HIST1H4D	0.606496161	-0.59237776	0.525534	-1.127193	0.259661	0.348151
2780	RANBP1	688.4259349	-0.592383109	0.095333	-6.213851	5.17E-10	3.65E-09
5636	YIPF2	980.6987431	-0.592744068	0.133116	-4.452848	8.47E-06	2.95E-05
11170	RFPL3S	5.475897933	-0.593301193	0.265924	-2.231097	0.025675	0.045054

10760	C17orf47	4.015375256	-0.593385871	0.249496	-2.378341	0.017391	0.03168
7167	SSNA1	723.7484516	-0.593550611	0.158019	-3.756207	0.000173	0.000472
8197	CTU1	59.04567623	-0.593602846	0.17807	-3.333529	0.000858	0.002051
4177	IQSEC2	495.4826886	-0.593681198	0.113514	-5.230027	1.69E-07	7.95E-07
6770	IL4R	1547.886948	-0.59370674	0.151803	-3.911037	9.19E-05	0.000266
10595	PRKCB	294.7263056	-0.593943067	0.243726	-2.436926	0.014813	0.027404
12747	ABCC2	813.8497161	-0.594018331	0.345569	-1.718956	0.085622	0.131661
7781	GSTO1	2799.083766	-0.594778771	0.169576	-3.50745	0.000452	0.00114
1452	OS9	9502.066675	-0.594790781	0.077928	-7.632537	2.30E-14	3.11E-13
8871	MAD2L1	164.6893055	-0.595163606	0.194301	-3.063094	0.002191	0.00484
9072	SRGN	3258.399735	-0.595168012	0.199068	-2.989775	0.002792	0.006032
4537	YEATS2	1302.350027	-0.595210994	0.118671	-5.015654	5.29E-07	2.28E-06
5102	IMP4	699.4931687	-0.595469145	0.125978	-4.726775	2.28E-06	8.76E-06
15195	POU4F1	4.993836832	-0.595758607	0.627452	-0.949488	0.342372	0.441648
11813	PMFBP1	11.12440529	-0.595768657	0.295316	-2.017395	0.043654	0.072436
9058	ZNF467	219.2595657	-0.595896475	0.198992	-2.994575	0.002748	0.005947
13841	SPDEF	6.695368728	-0.596215611	0.434917	-1.370873	0.170415	0.241333
13809	C10orf82	3.521872277	-0.596529032	0.43123	-1.38332	0.166567	0.236431
6064	PSMB6	1765.633587	-0.596714992	0.140775	-4.238781	2.25E-05	7.26E-05
12337	LILRA3	15.59969714	-0.596744777	0.322035	-1.853044	0.063876	0.101486
10525	PCDHB7	119.2557082	-0.59687552	0.24246	-2.461749	0.013826	0.025749
9476	SMAD5OS	4.451826413	-0.597052157	0.211129	-2.8279	0.004685	0.009692
10327	SLC2A10	322.7290954	-0.597448108	0.23544	-2.537579	0.011162	0.021187
5094	TXNDC5	7067.155959	-0.597790907	0.126402	-4.729279	2.25E-06	8.67E-06
5122	PIGU	617.9541548	-0.598439361	0.126887	-4.716335	2.40E-06	9.19E-06
7072	POLE4	490.180695	-0.598551725	0.157744	-3.794451	0.000148	0.00041
8281	ZNF311	35.16228892	-0.598569383	0.181353	-3.30058	0.000965	0.002284
12238	PLA2G7	228.9338048	-0.599058898	0.317583	-1.886306	0.059254	0.094904
4568	SHKBP1	1073.741127	-0.599087253	0.119821	-4.999852	5.74E-07	2.46E-06
9646	FAM193B	899.2596038	-0.59936775	0.216872	-2.763691	0.005715	0.011613
5600	PLCB3	574.2753844	-0.599502515	0.134041	-4.472541	7.73E-06	2.71E-05
8825	FAM115C	723.426893	-0.599536929	0.194608	-3.080749	0.002065	0.004586
10530	ELMO3	590.7617595	-0.599822976	0.243824	-2.46007	0.013891	0.025857
7893	RPS26	615.9820212	-0.599824389	0.173768	-3.451864	0.000557	0.001383
6274	RRP9	235.4223276	-0.599877356	0.144757	-4.14402	3.41E-05	0.000107
13664	CCDC146	1305.020811	-0.599892996	0.41823	-1.434362	0.151469	0.217282
10715	ETV7	156.7349996	-0.599901465	0.250952	-2.390502	0.016825	0.030777
8844	SIRPA	5277.403957	-0.599970882	0.195187	-3.073821	0.002113	0.004684
14196	GREB1L	20.8496954	-0.600014856	0.476564	-1.259043	0.208015	0.287215
6900	MYL6B	961.4840885	-0.600138801	0.155265	-3.865259	0.000111	0.000315
6584	ARHGEF1	1576.260814	-0.600270741	0.150435	-3.99022	6.60E-05	0.000197
9183	MYLK3	8.90491853	-0.60072081	0.203947	-2.945481	0.003225	0.006882
11727	GLT1D1	16.58211501	-0.601117914	0.294142	-2.043633	0.04099	0.068512
4247	TMEM219	2439.750163	-0.601375924	0.116035	-5.182707	2.19E-07	1.01E-06
10061	CACNA1C	371.0069929	-0.601596715	0.22855	-2.632227	0.008483	0.016526
10947	NODAL	7.401787223	-0.601767605	0.261126	-2.304512	0.021194	0.037949
703	TPD52L2	2719.546823	-0.601829176	0.066767	-9.013922	1.99E-19	5.54E-18

11009	NGF	110.5905498	-0.602086119	0.263246	-2.287163	0.022186	0.039502
3983	TOR2A	207.5286305	-0.602126039	0.112485	-5.352947	8.65E-08	4.26E-07
2699	WIBG	465.9188961	-0.602130617	0.095835	-6.283007	3.32E-10	2.41E-09
10441	C10orf95	5.019557557	-0.602142006	0.241104	-2.497437	0.012509	0.023484
12307	FGF14	133.4494231	-0.602483448	0.323427	-1.862814	0.062488	0.099524
7914	FAM158A	189.5170207	-0.602691519	0.175032	-3.443317	0.000575	0.001423
5501	CORO1B	1684.171246	-0.603371984	0.13345	-4.521345	6.14E-06	2.19E-05
6024	PSAP	46791.44917	-0.603713222	0.141886	-4.254903	2.09E-05	6.81E-05
10323	ACSF2	405.3010445	-0.603796537	0.237778	-2.539327	0.011107	0.021089
5273	DERL1	3102.415102	-0.6043522	0.130267	-4.63935	3.50E-06	1.30E-05
10690	ZNF215	45.22351253	-0.604637469	0.251865	-2.400641	0.016366	0.030009
7751	ZNF117	639.5633779	-0.604690134	0.171847	-3.518762	0.000434	0.001096
7716	HIGD2A	1494.296242	-0.604821795	0.171144	-3.533989	0.000409	0.00104
2729	TMEM214	2972.021906	-0.605908036	0.096852	-6.256005	3.95E-10	2.84E-09
11714	GAL3ST1	3311.29153	-0.60593305	0.295781	-2.048587	0.040503	0.067773
12955	ZNF488	5.106375434	-0.605966717	0.366173	-1.654863	0.097952	0.148202
10178	NXPH3	59.72047573	-0.606080388	0.234006	-2.590024	0.009597	0.018482
8673	ISOC2	1642.000415	-0.60618105	0.19294	-3.141812	0.001679	0.003795
12588	IL31RA	3.284333077	-0.606259798	0.341147	-1.77712	0.075549	0.117638
11350	HUS1B	3.018796462	-0.606289419	0.279929	-2.165871	0.030321	0.052363
3823	POFUT2	821.049159	-0.606527124	0.111223	-5.453254	4.95E-08	2.54E-07
5437	BRI3	2375.28871	-0.606531119	0.133342	-4.548675	5.40E-06	1.95E-05
12323	DGAT2	81.59926433	-0.606538868	0.326483	-1.857796	0.063198	0.100523
8284	MRPL12	1096.346831	-0.606556371	0.183855	-3.299108	0.00097	0.002295
14065	CCDC108	9.254796605	-0.606567505	0.46737	-1.297831	0.194346	0.27084
9432	KLHL17	100.254789	-0.606619913	0.213226	-2.844958	0.004442	0.00923
4357	ABL2	1251.970281	-0.607280506	0.118455	-5.126692	2.95E-07	1.33E-06
7602	RCSD1	617.0100844	-0.60746037	0.169715	-3.579307	0.000345	0.000888
11236	RPL9	1286.089475	-0.607548361	0.275537	-2.204959	0.027457	0.047898
12056	CGREF1	731.158299	-0.607877679	0.312982	-1.94221	0.052112	0.084711
10655	TNF	11.96773906	-0.608076874	0.252126	-2.411796	0.015874	0.029202
9126	DHRS13	253.9671509	-0.608498585	0.205085	-2.967061	0.003007	0.006458
5189	PFDN6	492.1652059	-0.608788463	0.130056	-4.680982	2.86E-06	1.08E-05
3740	NARF	835.2540847	-0.608926894	0.110466	-5.512326	3.54E-08	1.86E-07
1934	RNF216L	266.3472778	-0.609007657	0.086372	-7.050975	1.78E-12	1.80E-11
3449	JTB	2032.118228	-0.609202899	0.106753	-5.706642	1.15E-08	6.55E-08
6459	TMEM147	1478.934476	-0.609246721	0.150636	-4.044487	5.24E-05	0.000159
8596	FAM49A	137.5362706	-0.609476677	0.191911	-3.175837	0.001494	0.003407
9607	HOXD10	192.6249221	-0.609789208	0.219531	-2.777689	0.005475	0.01117
12393	ZNHIT2	158.090061	-0.609883243	0.331879	-1.83767	0.066111	0.104562
10078	WTIP	163.2588613	-0.610067788	0.232344	-2.62571	0.008647	0.016818
7505	NOTCH2	3193.347427	-0.610578693	0.168878	-3.615499	0.0003	0.000783
10037	MDGA1	116.3973518	-0.610596563	0.231306	-2.639782	0.008296	0.016201
7243	CLSTN3	3681.136867	-0.610609605	0.163772	-3.728423	0.000193	0.000521
5593	NOMO3	1759.497895	-0.610736356	0.136463	-4.475463	7.62E-06	2.67E-05
8787	GPR85	73.23345259	-0.610992917	0.197743	-3.089828	0.002003	0.004467
6273	BCAS4	722.4869091	-0.611076085	0.14746	-4.144001	3.41E-05	0.000107

9267	CCDC102B	283.3520737	-0.611080849	0.209916	-2.911073	0.003602	0.007619
6266	POU2F1	116.1178001	-0.61130124	0.147327	-4.149278	3.34E-05	0.000104
1169	NRBP1	2038.668835	-0.611402384	0.075825	-8.063292	7.43E-16	1.25E-14
6652	ARL4A	531.6913872	-0.611487834	0.154373	-3.961109	7.46E-05	0.00022
8317	SPSB2	206.5741885	-0.611565257	0.186238	-3.283784	0.001024	0.002414
9032	DHCR24	3444.17902	-0.611680481	0.203793	-3.001482	0.002687	0.005831
6783	RFNG	779.6945748	-0.611879839	0.156688	-3.905091	9.42E-05	0.000272
13480	GP9	1.473482899	-0.611974463	0.40939	-1.494844	0.134955	0.196236
4880	LMNB2	859.0354785	-0.612052106	0.126416	-4.841588	1.29E-06	5.17E-06
4851	STK10	1088.644415	-0.612179746	0.125875	-4.863394	1.15E-06	4.66E-06
15974	HSFY2	0.388627307	-0.612510691	0.849307	-0.721189	0.470793	0.57769
3371	CABLES2	148.1949513	-0.613169856	0.106331	-5.766628	8.09E-09	4.70E-08
12533	TACSTD2	372.6930397	-0.613246332	0.341669	-1.794857	0.072676	0.113662
14588	GRM7	0.758094067	-0.613299609	0.537858	-1.140263	0.254177	0.341521
4384	HSP90B1	27010.60258	-0.614098864	0.120109	-5.112845	3.17E-07	1.42E-06
7976	FASTK	1376.887576	-0.614215872	0.179622	-3.419495	0.000627	0.001542
15533	FGF23	0.642068028	-0.614252143	0.721999	-0.850765	0.3949	0.498322
2488	TXNDC12	1766.795349	-0.614354595	0.094778	-6.482051	9.05E-11	7.13E-10
6989	OR2A9P	269.6105432	-0.614527967	0.160441	-3.830253	0.000128	0.000359
12221	RSPH9	30.93498917	-0.614633353	0.325074	-1.890746	0.058658	0.094081
10827	GRAMD1B	94.48387397	-0.614673401	0.261286	-2.352491	0.018648	0.03376
11053	IL4I1	426.8358091	-0.614923371	0.270495	-2.273329	0.023006	0.040803
12409	SHISA7	1.494996135	-0.615448642	0.335801	-1.83278	0.066835	0.105572
10785	AKR1B1	6572.674711	-0.615820211	0.260231	-2.366436	0.01796	0.032642
11925	CCL3L1	48.96682717	-0.616047194	0.31099	-1.980921	0.0476	0.078237
8678	FAM18A	28.49192292	-0.61620927	0.19624	-3.140075	0.001689	0.003815
10837	RAB33A	36.96355186	-0.616688974	0.262803	-2.346584	0.018946	0.034269
4688	PRPF31	1170.135512	-0.616980985	0.125018	-4.935143	8.01E-07	3.35E-06
4649	PA2G4P4	53.57329109	-0.617007702	0.124358	-4.961532	6.99E-07	2.95E-06
4051	YKT6	2060.971122	-0.617060775	0.116296	-5.305943	1.12E-07	5.42E-07
5058	ATP6V1E2	47.03674514	-0.617091101	0.130096	-4.743359	2.10E-06	8.15E-06
6340	PITPNM2	537.5150075	-0.617673598	0.150358	-4.108014	3.99E-05	0.000123
4922	ZNF446	207.3761321	-0.617720572	0.128258	-4.816224	1.46E-06	5.83E-06
2995	CNPY2	1387.775765	-0.617945093	0.102369	-6.03645	1.58E-09	1.03E-08
12313	C11orf88	3.636587802	-0.617990873	0.332097	-1.860873	0.062762	0.099911
12151	NBPF7	1.331828123	-0.618063543	0.323529	-1.910381	0.056084	0.09047
12398	KIAA1409	28.98832338	-0.61814076	0.336568	-1.836598	0.066269	0.10477
6505	YIF1A	1125.575988	-0.618292506	0.153612	-4.025028	5.70E-05	0.000172
13859	C14orf50	13.78060482	-0.61931099	0.453545	-1.365489	0.172099	0.243403
2830	SLC35C1	605.1818477	-0.619370444	0.100492	-6.163377	7.12E-10	4.93E-09
4477	PSMC5	2538.076629	-0.619410365	0.122567	-5.053627	4.33E-07	1.90E-06
5040	CEP250	867.9738613	-0.619470483	0.130329	-4.753123	2.00E-06	7.79E-06
11483	DCT	4.121178661	-0.619513547	0.291353	-2.126334	0.033475	0.057141
8990	NQO1	1204.084861	-0.619639832	0.205414	-3.016538	0.002557	0.005575
4768	GLTSCR1	219.8006854	-0.620021718	0.126574	-4.898473	9.66E-07	3.97E-06
5229	RPS13	8200.200653	-0.620084989	0.132926	-4.664874	3.09E-06	1.16E-05
6265	MRPL53	577.2187695	-0.620214338	0.149456	-4.149812	3.33E-05	0.000104

9437	AGAP4	213.3220928	-0.620230317	0.218168	-2.842901	0.00447	0.009285
2525	PPIE	766.4774651	-0.620312135	0.096269	-6.443541	1.17E-10	9.06E-10
9844	FAM43A	502.9133573	-0.620718927	0.229373	-2.706159	0.006807	0.013553
12058	NOXO1	3.144500033	-0.620995081	0.319729	-1.942255	0.052106	0.084711
11870	GNAT2	1.4298977	-0.621398712	0.310645	-2.000347	0.045463	0.075073
16225	RAD51AP2	0.330108679	-0.621535846	0.958497	-0.648448	0.516695	0.624206
8306	FOXD2	66.12620515	-0.621751949	0.188943	-3.290682	0.000999	0.002359
6655	RANGAP1	1340.028131	-0.622023003	0.157042	-3.960864	7.47E-05	0.00022
2657	SEC61A1	9187.549527	-0.622037344	0.098409	-6.320958	2.60E-10	1.92E-09
10759	STC2	3543.872993	-0.622679909	0.261784	-2.378598	0.017379	0.031661
5243	APOBEC3F	258.9226665	-0.622718971	0.133803	-4.653997	3.26E-06	1.22E-05
13529	C14orf68	1.717634971	-0.622824079	0.421665	-1.47706	0.139659	0.202341
6150	AIFM2	453.6574586	-0.623163079	0.148272	-4.202829	2.64E-05	8.40E-05
15840	NRSN1	0.323771311	-0.623515811	0.81933	-0.761006	0.446653	0.552708
7787	JUN	8167.242613	-0.62368163	0.17793	-3.505214	0.000456	0.001148
8026	SRRM5	26.06909052	-0.623700431	0.183397	-3.400813	0.000672	0.001641
13833	ZDHHC22	1.033597889	-0.623704938	0.454073	-1.37358	0.169572	0.240279
6016	PHLDB1	2156.745777	-0.623880597	0.146543	-4.257313	2.07E-05	6.74E-05
6603	SBNO2	1436.990053	-0.624235857	0.156725	-3.982999	6.81E-05	0.000202
4772	C12orf10	1008.87295	-0.624291365	0.127479	-4.897212	9.72E-07	3.99E-06
5293	RRBP1	6454.006812	-0.624429994	0.135023	-4.624613	3.75E-06	1.39E-05
6856	SCO2	582.7273298	-0.624885611	0.161107	-3.878688	0.000105	0.0003
4670	CLPP	894.3479036	-0.625147146	0.126298	-4.949763	7.43E-07	3.12E-06
8151	ATP5EP2	37.04630167	-0.625153257	0.186566	-3.350849	0.000806	0.001937
11960	PLIN1	30.05857683	-0.625186893	0.31711	-1.971512	0.048665	0.079757
5486	AIP	1313.921238	-0.625450443	0.138108	-4.528689	5.94E-06	2.12E-05
15135	GPR142	0.327853847	-0.625612383	0.64918	-0.963697	0.335198	0.434084
2957	NAGK	1171.6766	-0.625677879	0.103124	-6.067234	1.30E-09	8.63E-09
3850	RAG1AP1	774.412454	-0.625755124	0.115165	-5.433543	5.52E-08	2.81E-07
5932	TPI1	21079.59809	-0.625884035	0.145451	-4.303067	1.68E-05	5.57E-05
11735	KIR3DL2	4.768194899	-0.625898917	0.306483	-2.042195	0.041132	0.068709
6080	FAM71D	14.1551025	-0.626413018	0.148107	-4.229451	2.34E-05	7.55E-05
6547	RBM42	1192.271488	-0.626462347	0.156367	-4.006362	6.17E-05	0.000185
11025	APOBEC3A	25.701621	-0.626574971	0.274633	-2.281498	0.022519	0.040036
10634	WDR93	24.09449797	-0.627031049	0.258948	-2.421455	0.015459	0.028494
9357	TBC1D2	486.7202305	-0.627080546	0.217928	-2.877465	0.004009	0.008398
10144	PIK3CG	189.6625884	-0.627146983	0.241195	-2.60017	0.009318	0.018004
12139	EDIL3	1228.49504	-0.627343942	0.32743	-1.915966	0.055369	0.089406
5291	PPPDE2	370.5550232	-0.62744833	0.135641	-4.62581	3.73E-06	1.38E-05
15717	CTRC	0.349085282	-0.627504951	0.78502	-0.799349	0.424088	0.528889
15876	C1orf110	0.312599891	-0.627590179	0.837375	-0.749474	0.453572	0.55998
15555	FAM71C	0.267736332	-0.627737935	0.742549	-0.845383	0.397897	0.501421
16373	CCK	0.314980296	-0.627737954	1.033299	-0.607508	0.543514	0.650669
15793	SEL1L2	0.252947203	-0.627737958	0.806571	-0.778279	0.436404	0.54163
15485	OR52B6	0.229158123	-0.627737965	0.724975	-0.865875	0.386559	0.489308
16753	SYT14L	0.275525807	-0.627737972	1.245041	-0.50419	0.614128	0.718529
16357	PBOV1	0.249136603	-0.627737984	1.025715	-0.612001	0.540537	0.647739

15608	ACER1	0.215479946	-0.627737985	0.753425	-0.833179	0.404744	0.508289
16211	SPACA4	0.235272112	-0.627738002	0.961644	-0.652776	0.513901	0.621366
16447	TCF23	0.199314839	-0.627738089	1.068118	-0.587705	0.55673	0.663493
16272	C7orf45	0.177217664	-0.627738093	0.99028	-0.6339	0.526146	0.633749
16277	PAGE5	0.175864095	-0.627738093	0.990866	-0.633525	0.526391	0.633849
17136	GABRG3	0.236716356	-0.627738146	1.561196	-0.402088	0.687619	0.786533
16575	SNORA24	0.176043965	-0.627738148	1.13376	-0.553678	0.579799	0.68565
16724	DAD1L	0.151892385	-0.627738229	1.221905	-0.513738	0.607436	0.711904
17299	LRTM1	0.194761022	-0.627738361	1.709847	-0.367131	0.713521	0.80847
17131	BLID	0.152579758	-0.627738424	1.555843	-0.403472	0.686601	0.78559
17316	SNORA10	0.118515341	-0.627738716	1.728383	-0.363194	0.71646	0.811003
17332	NLRP10	0.111449178	-0.62773873	1.743445	-0.360056	0.718805	0.812906
17452	IL25	0.112964175	-0.627738906	1.923344	-0.326379	0.744138	0.835769
17600	C4orf17	0.118522716	-0.627739141	2.138347	-0.293563	0.769092	0.856533
17724	INSL6	0.133068526	-0.62773929	2.397211	-0.261862	0.793428	0.877441
17871	SSX5	0.154296792	-0.627739437	2.690147	-0.233348	0.815491	0.894435
17903	KRT38	0.122645274	-0.627739951	2.755799	-0.227789	0.81981	0.897565
17909	SPAG11A	0.116554045	-0.627739977	2.773543	-0.226331	0.820944	0.898504
15801	SPERT	0.956880818	-0.627897188	0.809399	-0.775757	0.437892	0.543201
13715	CRNA0018	4.340727703	-0.627987312	0.444072	-1.414155	0.157316	0.224831
2581	POLR2G	856.9127262	-0.628196663	0.098146	-6.400644	1.55E-10	1.18E-09
2695	SIDT2	1152.671353	-0.628215142	0.099936	-6.286147	3.25E-10	2.37E-09
8499	DGKD	1058.200981	-0.628810889	0.1961	-3.20659	0.001343	0.003098
14277	FOXA1	10.38370886	-0.628839526	0.509047	-1.235326	0.216709	0.297528
15832	PRSS1	0.956071601	-0.6289069	0.822042	-0.765055	0.444239	0.549995
9988	TRIM58	23.33801571	-0.629128962	0.2368	-2.656796	0.007889	0.015481
9773	TPPP3	632.6990631	-0.62936738	0.231016	-2.724345	0.006443	0.012923
8329	PPDPF	3948.258982	-0.629492009	0.191882	-3.280624	0.001036	0.002438
9473	DFNB59	30.46534458	-0.629944561	0.222574	-2.830271	0.004651	0.009623
2263	EIF5AL1	1234.094448	-0.630109646	0.094228	-6.687051	2.28E-11	1.97E-10
4238	C19orf43	1819.33319	-0.63054764	0.121557	-5.187243	2.13E-07	9.87E-07
3395	TMEM43	2742.196262	-0.630797804	0.109705	-5.749942	8.93E-09	5.15E-08
5848	ENTPD6	1617.544918	-0.6308875	0.145103	-4.347853	1.37E-05	4.61E-05
2697	AP1M1	1619.658318	-0.631583747	0.100515	-6.28349	3.31E-10	2.41E-09
5255	INO80E	776.8418482	-0.631719619	0.135915	-4.647914	3.35E-06	1.25E-05
2401	CARS	1025.814785	-0.631747984	0.09633	-6.558196	5.45E-11	4.45E-10
5953	LIN37	149.6663333	-0.631894182	0.147311	-4.289531	1.79E-05	5.90E-05
8380	MSTO2P	100.2357738	-0.632082381	0.194012	-3.25796	0.001122	0.002624
5774	LOC375190	96.69223931	-0.632184228	0.144228	-4.383228	1.17E-05	3.97E-05
14362	CSAG1	1.644694962	-0.632187157	0.522583	-1.209735	0.226381	0.30896
5801	GTF2IRD1	472.9035746	-0.632399339	0.144583	-4.373944	1.22E-05	4.12E-05
9329	TNFRSF12A	2228.15253	-0.632481714	0.218951	-2.888696	0.003868	0.008128
13904	HSPC072	1.377673072	-0.632877353	0.469507	-1.347962	0.177671	0.250433
5599	CLEC2D	658.2624904	-0.632885337	0.141499	-4.472714	7.72E-06	2.70E-05
11778	PDZK1IP1	8590.907669	-0.63292487	0.312027	-2.028432	0.042516	0.070756
3688	HGS	1704.261305	-0.633004755	0.114186	-5.543642	2.96E-08	1.57E-07
11121	RPA4	2.546208549	-0.633538563	0.282198	-2.245015	0.024767	0.043653

10677	TMEM198	156.6981976	-0.633596513	0.263592	-2.4037	0.01623	0.029798
14466	ATCAY	1.47970598	-0.63359696	0.537677	-1.178398	0.238638	0.323348
5724	BRMS1	814.3757576	-0.633671792	0.143712	-4.409316	1.04E-05	3.55E-05
12011	RGS13	6.345281206	-0.634020079	0.323776	-1.958207	0.050206	0.081932
8433	HOXA3	284.2688998	-0.634072243	0.195946	-3.235956	0.001212	0.002818
3880	UBC	57253.21185	-0.634101931	0.117113	-5.414463	6.15E-08	3.11E-07
4061	ZNF341	93.41139841	-0.634456031	0.119682	-5.301191	1.15E-07	5.55E-07
7583	STX11	210.7711029	-0.634598701	0.177008	-3.585141	0.000337	0.000871
7178	C1orf159	122.3748748	-0.634634027	0.16912	-3.752565	0.000175	0.000478
5705	RGS3	1280.917032	-0.63520495	0.143809	-4.417001	1.00E-05	3.44E-05
3220	PSMB4	4079.487729	-0.635541391	0.108198	-5.873871	4.26E-09	2.59E-08
5770	MTX1	835.2531552	-0.635678466	0.144914	-4.38658	1.15E-05	3.91E-05
13482	HEPACAM	1.682468152	-0.635865708	0.425835	-1.493222	0.135379	0.196815
5611	SH3PXD2A	1527.15195	-0.635874933	0.142361	-4.466627	7.95E-06	2.78E-05
12859	CBLN4	90.13192603	-0.636168902	0.376523	-1.689588	0.091107	0.138874
7892	PEMT	312.6382527	-0.636446616	0.184349	-3.452408	0.000556	0.00138
9866	NLRP12	11.81435959	-0.636469183	0.235911	-2.697918	0.006977	0.013861
4124	LMBR1L	499.9604902	-0.63651841	0.12083	-5.267867	1.38E-07	6.56E-07
4380	SSR2	5980.35563	-0.636582828	0.124471	-5.114291	3.15E-07	1.41E-06
8721	TTC21A	66.53353071	-0.637255006	0.204185	-3.12097	0.001803	0.004051
2226	BRD9	687.982424	-0.63734724	0.094788	-6.723929	1.77E-11	1.56E-10
7370	ATAD2	340.9033391	-0.637359818	0.173675	-3.66985	0.000243	0.000645
11591	SCNN1D	65.84698941	-0.637381288	0.305947	-2.083308	0.037223	0.062946
6546	TMEM205	2266.781398	-0.637515974	0.159117	-4.006574	6.16E-05	0.000184
4018	EML3	952.8068168	-0.637632254	0.119657	-5.328842	9.88E-08	4.82E-07
16700	C10orf129	0.884842989	-0.637687903	1.228118	-0.51924	0.603594	0.708445
4526	ABHD12	1850.050796	-0.63771338	0.127041	-5.019732	5.17E-07	2.24E-06
3116	SLC35C2	1085.884708	-0.638270801	0.107257	-5.950851	2.67E-09	1.68E-08
4092	OAZ1	8582.267759	-0.638335851	0.12078	-5.285113	1.26E-07	6.02E-07
12246	TMEM200C	14.772018	-0.638553485	0.339007	-1.8836	0.059619	0.095427
5426	BAD	941.9435011	-0.638624581	0.140221	-4.554415	5.25E-06	1.90E-05
11424	LOC644165	40.72020053	-0.63896819	0.298505	-2.140564	0.032309	0.055435
5160	ZNF575	33.68736105	-0.639576279	0.136233	-4.69474	2.67E-06	1.01E-05
7038	ABCA3	1432.106244	-0.639659156	0.168149	-3.804124	0.000142	0.000396
9403	F2R	2977.427774	-0.639805414	0.223931	-2.85715	0.004275	0.008911
13082	C1orf223	1.415671029	-0.639866025	0.395059	-1.619671	0.105303	0.157777
6787	HSPBP1	685.3163366	-0.639890565	0.163906	-3.904002	9.46E-05	0.000273
3577	PRKRIP1	647.0822052	-0.640344604	0.11395	-5.619498	1.92E-08	1.05E-07
11930	TNFSF18	2.98496226	-0.640594223	0.323469	-1.980388	0.04766	0.078305
5947	ARSA	963.4542941	-0.640625067	0.149232	-4.292811	1.76E-05	5.81E-05
15888	MBL2	2.420015419	-0.64068882	0.858343	-0.746425	0.455411	0.561839
11703	SUMO4	1.479065189	-0.64080144	0.312438	-2.05097	0.04027	0.067447
14354	CD5L	31.51607542	-0.64082732	0.528459	-1.212635	0.22527	0.307615
14367	PGLYRP2	4.111810541	-0.641024323	0.530499	-1.208341	0.226916	0.309583
7197	LAMB2L	15.9205194	-0.641295044	0.171136	-3.747291	0.000179	0.000487
13658	CCDC67	3.482600346	-0.641476548	0.446686	-1.43608	0.15098	0.21666
2511	PTMA	15682.11495	-0.641485902	0.099395	-6.453899	1.09E-10	8.51E-10

13412	INSC	3.25174596	-0.641518067	0.42495	-1.509632	0.131137	0.191637
13319	CLDN16	5.125532938	-0.641561202	0.417283	-1.537473	0.124178	0.182747
7864	CDC7	131.268519	-0.641624164	0.185142	-3.465576	0.000529	0.001319
4881	NINJ1	1936.450378	-0.641626963	0.132553	-4.84055	1.29E-06	5.20E-06
7753	GLRX	2648.728094	-0.642153683	0.18254	-3.517877	0.000435	0.0011
13701	CAPNS2	0.963013827	-0.642204123	0.453038	-1.41755	0.156322	0.223639
6422	MGMT	399.0930535	-0.642426481	0.158015	-4.065617	4.79E-05	0.000146
5313	BZW2	804.8066401	-0.642508709	0.139252	-4.614005	3.95E-06	1.46E-05
8995	TACR2	13.83880609	-0.642700788	0.213218	-3.014284	0.002576	0.005613
9033	DKK3	1784.688288	-0.643124159	0.214306	-3.000956	0.002691	0.00584
12623	FAM183A	5.450627869	-0.643239719	0.3644	-1.765201	0.07753	0.120388
6226	DYNLRB1	3055.734041	-0.643288546	0.154406	-4.166216	3.10E-05	9.75E-05
13083	SKINTL	2.046835217	-0.643328824	0.397663	-1.617774	0.105711	0.158377
11273	LOC39995	219.9240958	-0.643448671	0.293902	-2.189328	0.028573	0.049682
10043	IL18RAP	31.64291165	-0.643524059	0.244115	-2.636155	0.008385	0.016365
11893	TSPAN1	2956.168127	-0.643574499	0.323233	-1.991052	0.046475	0.076596
5188	HEBP2	1115.94759	-0.643589878	0.137482	-4.681252	2.85E-06	1.08E-05
6303	LOC81691	63.4266598	-0.64374203	0.156033	-4.12567	3.70E-05	0.000115
13711	GAL3ST2	1.865583526	-0.643766294	0.455032	-1.414773	0.157135	0.224638
12057	HSF5	2.089571947	-0.644146006	0.331655	-1.942218	0.052111	0.084711
9492	HLA-DQA1	3569.431027	-0.644172692	0.228321	-2.821353	0.004782	0.009875
2677	ZDHHC18	598.7315414	-0.644254876	0.102253	-6.300567	2.97E-10	2.17E-09
5870	ZNF239	80.32712768	-0.644392769	0.148588	-4.336786	1.45E-05	4.83E-05
4744	T6GALNAC	1230.492376	-0.644533014	0.131225	-4.91168	9.03E-07	3.73E-06
6611	CCDC107	645.1061041	-0.644544774	0.161979	-3.979178	6.92E-05	0.000205
4860	PRR13	3652.562657	-0.644662092	0.132711	-4.857652	1.19E-06	4.79E-06
12983	LOC44092	9.939574702	-0.644671994	0.390839	-1.649457	0.099054	0.149546
5480	THOC6	498.5377087	-0.644745715	0.142326	-4.530065	5.90E-06	2.11E-05
10064	RGMA	53.91834363	-0.64474675	0.245007	-2.631547	0.0085	0.016554
5920	NCAPG2	323.4990525	-0.644759842	0.149487	-4.313143	1.61E-05	5.33E-05
2269	DDX56	1500.824411	-0.64480567	0.096546	-6.678711	2.41E-11	2.08E-10
16069	SMPX	2.252305962	-0.644925537	0.931775	-0.692147	0.488845	0.596294
3592	C1orf216	344.2259692	-0.645067493	0.115021	-5.608241	2.04E-08	1.12E-07
4236	TNIP2	676.2382996	-0.645195776	0.124347	-5.188691	2.12E-07	9.80E-07
9136	STAG3	30.35749563	-0.645214253	0.217643	-2.964559	0.003031	0.006503
12552	ABCA12	195.702226	-0.645314264	0.360551	-1.789802	0.073486	0.114754
15651	SUN3	0.541788829	-0.645346037	0.784706	-0.822404	0.410847	0.514536
3687	CLIC1	8101.389624	-0.645592721	0.116457	-5.543624	2.96E-08	1.57E-07
2701	LIMK2	1048.701411	-0.645682726	0.1028	-6.280988	3.36E-10	2.44E-09
2624	NOD1	378.3866886	-0.646159208	0.101704	-6.353327	2.11E-10	1.57E-09
13667	MEGF11	161.5060161	-0.646386248	0.451005	-1.433214	0.151797	0.217704
6378	OGFOD2	373.7909478	-0.647497051	0.158367	-4.088581	4.34E-05	0.000133
3953	RILPL1	371.349668	-0.647815523	0.120544	-5.374112	7.70E-08	3.82E-07
6963	GEMIN8P4	16.09272694	-0.647841114	0.168655	-3.841212	0.000122	0.000345
9750	CEACAM1	60.11225555	-0.648070509	0.237165	-2.732577	0.006284	0.012633
4635	PQBP1	1069.080323	-0.648089836	0.130357	-4.971663	6.64E-07	2.81E-06
3106	ELK1	777.4875749	-0.648194176	0.108761	-5.959798	2.53E-09	1.59E-08

12545	ABCC6P1	141.611129	-0.648360817	0.361798	-1.792052	0.073125	0.114242
6933	FAM125A	698.1100406	-0.648604897	0.16846	-3.850208	0.000118	0.000334
6435	ZNF205	336.6615588	-0.649133967	0.160059	-4.055593	5.00E-05	0.000152
6736	CCDC124	1025.409886	-0.649257568	0.165347	-3.926647	8.61E-05	0.000251
8363	PMS2L11	26.85609765	-0.649370908	0.19881	-3.266291	0.00109	0.002554
11923	RNF175	24.99614671	-0.649809046	0.327939	-1.981493	0.047536	0.078148
5859	CAMK1	726.6862203	-0.649936302	0.149706	-4.34143	1.42E-05	4.74E-05
4041	TRMT112	2364.352911	-0.650722168	0.122502	-5.311918	1.08E-07	5.26E-07
7610	NDUFA2	1477.907853	-0.65099714	0.182007	-3.576767	0.000348	0.000896
8080	CLDN15	196.2610648	-0.65133484	0.192748	-3.379211	0.000727	0.001763
7460	PSMB9	1204.234626	-0.651354981	0.179277	-3.633228	0.00028	0.000735
12157	CYP2D7P1	18.57658309	-0.651787785	0.341497	-1.908618	0.056311	0.090792
3854	P2RX4	873.4717273	-0.652033051	0.120081	-5.429942	5.64E-08	2.87E-07
6803	ENC1	745.4573274	-0.652183897	0.167281	-3.898741	9.67E-05	0.000279
11740	PCDHA9	5.274195976	-0.652312483	0.319608	-2.04098	0.041253	0.068875
8034	SLFN11	1411.583286	-0.653567302	0.192308	-3.398538	0.000677	0.001653
9007	SLC6A8	10884.9778	-0.653603169	0.217211	-3.009077	0.00262	0.005703
12571	CHRNA6	5.978963373	-0.653750591	0.367033	-1.781179	0.074883	0.11676
4200	CDKN2D	138.9304014	-0.654071656	0.125524	-5.210732	1.88E-07	8.78E-07
7154	BMF	377.5295508	-0.654095778	0.173835	-3.76273	0.000168	0.00046
12593	NPPA	1.471832174	-0.654105613	0.368369	-1.775683	0.075785	0.11796
8212	GNB4	1315.734187	-0.654593285	0.19676	-3.326867	0.000878	0.002096
9758	CDRT15P	4.203060944	-0.654682079	0.239881	-2.729194	0.006349	0.012753
4157	SLC5A6	522.777772	-0.654788366	0.124811	-5.24624	1.55E-07	7.32E-07
11708	HIST1H1E	3.720659931	-0.654823568	0.319377	-2.050316	0.040334	0.067525
9161	POTEF	9.577880331	-0.655224372	0.221768	-2.954548	0.003131	0.0067
5875	SIRT6	300.4692142	-0.655239907	0.151155	-4.334874	1.46E-05	4.87E-05
5132	EBPL	416.2015174	-0.655336425	0.139039	-4.713342	2.44E-06	9.31E-06
10342	LAMB4	9.917928786	-0.655373805	0.258826	-2.532104	0.011338	0.021489
12440	COL23A1	3409.56713	-0.655455418	0.359274	-1.824388	0.068093	0.107291
7968	NRP2	1742.792932	-0.655570413	0.191563	-3.422226	0.000621	0.001528
8963	TTLL3	579.9311999	-0.655614196	0.216658	-3.026034	0.002478	0.005419
11723	DLX3	4.256269565	-0.655804712	0.320597	-2.045572	0.040798	0.068216
12728	SCARNA16	1.506534387	-0.655936853	0.380534	-1.723726	0.084757	0.130529
6888	DLG4	206.4597362	-0.655971333	0.169571	-3.868425	0.00011	0.000312
13252	UROC1	1.536556831	-0.65598222	0.419772	-1.562709	0.118121	0.174713
5430	C17orf70	791.7802045	-0.656062159	0.1441	-4.552822	5.29E-06	1.91E-05
4976	UXT	1131.690177	-0.656080128	0.137097	-4.785525	1.71E-06	6.72E-06
13244	PIN1L	0.679315569	-0.656326898	0.419365	-1.56505	0.117571	0.174004
12964	CTNND2	4.454207233	-0.656581431	0.397059	-1.653611	0.098207	0.148484
2676	FANCG	231.3621509	-0.656688563	0.104202	-6.302069	2.94E-10	2.15E-09
13344	CRB2	58.04386548	-0.656697353	0.42913	-1.530299	0.125943	0.184997
9804	BATF3	75.3021313	-0.656756269	0.241863	-2.715404	0.00662	0.013234
12802	ITIH2	4.052820669	-0.656979383	0.385839	-1.702727	0.088619	0.135684
13738	TRH	0.790348579	-0.657176844	0.46773	-1.405035	0.160011	0.228299
1837	PSMA5	1332.611241	-0.657228818	0.091906	-7.151109	8.61E-13	9.18E-12
12746	SUSD4	86.18815418	-0.657440243	0.382428	-1.71912	0.085593	0.131626

7417	NUDT18	213.4971337	-0.657706546	0.180208	-3.649714	0.000263	0.000694
5779	ELOF1	854.8084866	-0.658764548	0.150326	-4.382248	1.17E-05	3.98E-05
8469	C19orf70	1135.200187	-0.659075162	0.204697	-3.219764	0.001283	0.002969
14742	GUCY1B2	19.65535911	-0.659319587	0.606698	-1.086734	0.277155	0.368505
1073	PSMB2	1668.537133	-0.65933112	0.080375	-8.203178	2.34E-16	4.28E-15
6342	C12orf24	158.1014172	-0.659402286	0.160522	-4.107854	3.99E-05	0.000123
7668	GPX7	284.1817971	-0.659455653	0.185678	-3.551601	0.000383	0.000979
5776	KIF22	644.0023883	-0.659722526	0.150522	-4.382908	1.17E-05	3.97E-05
8848	SCD	12969.19952	-0.659771644	0.214745	-3.072349	0.002124	0.004705
8413	CCDC163P	15.20247846	-0.659879073	0.203385	-3.244482	0.001177	0.002741
11075	GABRD	301.9935491	-0.660293351	0.291528	-2.264942	0.023516	0.04162
6044	PCDHGC3	3416.223866	-0.660310639	0.155477	-4.247003	2.17E-05	7.03E-05
4252	PTPN18	1826.140418	-0.660415287	0.127463	-5.181215	2.20E-07	1.02E-06
3744	MPV17	1212.888427	-0.660651422	0.1199	-5.510004	3.59E-08	1.88E-07
8667	EFNA1	2655.515912	-0.660693297	0.210088	-3.144848	0.001662	0.003758
8274	CCDC24	167.4823446	-0.661051082	0.200106	-3.303509	0.000955	0.002262
4187	SNAPC2	273.2362649	-0.661650738	0.126834	-5.216664	1.82E-07	8.53E-07
5633	ABCD1	610.5377727	-0.662030971	0.148644	-4.453805	8.44E-06	2.94E-05
9953	STK39	1574.347925	-0.662054799	0.248001	-2.669568	0.007595	0.014957
7956	PHACTR1	64.99492459	-0.662080648	0.19334	-3.424431	0.000616	0.001518
7229	OC1001342	149.8621169	-0.662086396	0.177265	-3.735013	0.000188	0.000509
14154	CNTN1	13.98783834	-0.662103025	0.521325	-1.270039	0.204071	0.282605
4555	WRAP53	182.5578725	-0.662216663	0.132251	-5.007282	5.52E-07	2.38E-06
9892	ROBO1	688.4008203	-0.662265689	0.246129	-2.69073	0.00713	0.014128
4471	ACD	394.305824	-0.662308069	0.130975	-5.05676	4.26E-07	1.87E-06
9389	SAMD14	113.085775	-0.662341306	0.231276	-2.863853	0.004185	0.008737
6353	CCDC102A	203.9214452	-0.662357498	0.161574	-4.099415	4.14E-05	0.000128
14181	GPR22	0.629021247	-0.662679202	0.524759	-1.262825	0.206652	0.285635
4085	MYD88	1059.178414	-0.662715927	0.125276	-5.290042	1.22E-07	5.87E-07
5658	USE1	421.5475529	-0.662857607	0.149157	-4.444035	8.83E-06	3.06E-05
5300	CHMP2A	2797.642715	-0.662867392	0.143431	-4.621507	3.81E-06	1.41E-05
5043	RPS18	23301.61616	-0.662929301	0.139534	-4.751015	2.02E-06	7.87E-06
8613	SYTL3	291.2908412	-0.663039203	0.209181	-3.169687	0.001526	0.003473
10414	CD82	843.996493	-0.663061786	0.264604	-2.505866	0.012215	0.022991
4671	TRNAU1AI	350.9781658	-0.663163637	0.133978	-4.949785	7.43E-07	3.12E-06
7008	OSBPL7	240.1028409	-0.663168087	0.173604	-3.820004	0.000133	0.000373
6468	BLOC1S1	1516.409996	-0.66325241	0.164069	-4.042516	5.29E-05	0.00016
8593	LRDD	271.4456632	-0.663481929	0.208908	-3.175959	0.001493	0.003407
7217	SLC6A6	1253.73172	-0.663528605	0.177489	-3.738412	0.000185	0.000503
12875	AMY1A	3.649053624	-0.663602088	0.394271	-1.683111	0.092354	0.1406
2737	TCF3	885.2033856	-0.663723538	0.106204	-6.249533	4.12E-10	2.95E-09
14509	C4orf31	219.3407694	-0.6641167	0.570634	-1.163822	0.244496	0.330303
4442	RAB4B	435.9667149	-0.664330322	0.130835	-5.077623	3.82E-07	1.69E-06
9375	DENND2A	406.6285641	-0.664336505	0.231446	-2.870376	0.0041	0.008572
8662	OLFML1	328.7989386	-0.664721739	0.211256	-3.146522	0.001652	0.003739
9217	GAS1	74.47804181	-0.664990134	0.226738	-2.932856	0.003359	0.007142
9587	TMEM108	26.94276958	-0.665088147	0.238891	-2.784061	0.005368	0.010976

2665	CDK4	1870.1662	-0.665295133	0.105333	-6.316085	2.68E-10	1.97E-09
8777	PRF1	465.4770369	-0.665412799	0.215106	-3.093423	0.001979	0.004419
4714	DPP9	1842.783494	-0.6657058	0.135116	-4.926909	8.35E-07	3.47E-06
4516	LRWD1	309.9905104	-0.665893299	0.132468	-5.026833	4.99E-07	2.16E-06
9462	CLSPN	38.1988753	-0.66599324	0.235048	-2.833434	0.004605	0.00954
7128	SMAGP	332.3489958	-0.666028968	0.176457	-3.774458	0.00016	0.000441
6991	MSRB3	916.7833259	-0.666433593	0.174058	-3.828795	0.000129	0.000361
7453	TCTEX1D2	100.438372	-0.666662204	0.183328	-3.636453	0.000276	0.000727
2954	SNF8	979.8253322	-0.666996153	0.109915	-6.068306	1.29E-09	8.58E-09
2947	DCLRE1B	209.7392155	-0.667255134	0.109874	-6.072908	1.26E-09	8.35E-09
13359	CTSG	13.95025185	-0.667395723	0.437935	-1.52396	0.127519	0.187102
3080	ACOX3	353.3559812	-0.668314388	0.11182	-5.976679	2.28E-09	1.45E-08
11674	EMR4P	14.15450316	-0.668430208	0.324962	-2.056951	0.039691	0.066637
3924	C19orf61	469.4656842	-0.668490558	0.124048	-5.388961	7.09E-08	3.54E-07
4385	TSPAN17	1007.929419	-0.669037239	0.130859	-5.112668	3.18E-07	1.42E-06
10625	SLFN12L	21.81137463	-0.669239983	0.276007	-2.424724	0.01532	0.028262
3629	EIF6	2553.154952	-0.66934385	0.119838	-5.585417	2.33E-08	1.26E-07
11211	ACTA1	1.808790718	-0.669566296	0.302052	-2.216725	0.026642	0.04658
5868	KIAA0415	619.7334297	-0.669947031	0.154471	-4.337027	1.44E-05	4.82E-05
5298	MYOF	4421.354649	-0.670067294	0.144959	-4.622454	3.79E-06	1.40E-05
4273	H6PD	2945.195207	-0.670078155	0.129611	-5.169898	2.34E-07	1.07E-06
11088	SPEF1	34.25178407	-0.670379544	0.296596	-2.260245	0.023806	0.042084
10067	CCL22	28.50675522	-0.670554504	0.254969	-2.629944	0.00854	0.016628
14161	C21orf29	1.201836101	-0.670558223	0.529029	-1.267526	0.204967	0.283706
10384	RS1	3.867458681	-0.671037221	0.267144	-2.511889	0.012009	0.022668
8956	FAM186A	8.251076471	-0.671065621	0.221458	-3.03022	0.002444	0.005348
4770	TXNDC17	790.7545628	-0.671139242	0.137025	-4.897924	9.69E-07	3.98E-06
7233	HLA-DMB	2089.346221	-0.671211338	0.179857	-3.73192	0.00019	0.000515
7890	GINS4	76.44028225	-0.671311745	0.194385	-3.453517	0.000553	0.001375
6573	ANKDD1A	77.58919255	-0.671359891	0.168106	-3.993676	6.51E-05	0.000194
8137	PSTPIP2	180.2419832	-0.671503723	0.199989	-3.357696	0.000786	0.001893
3281	ADRBK1	1738.402424	-0.671561243	0.115194	-5.829852	5.55E-09	3.31E-08
8158	FGD1	219.3506407	-0.672655873	0.20089	-3.34838	0.000813	0.001953
12317	ENTPD3	3.874365568	-0.672762787	0.361756	-1.859713	0.062926	0.100139
3093	RPL6	9112.896327	-0.673023584	0.112773	-5.967953	2.40E-09	1.52E-08
13443	FAM169B	12.46899952	-0.673069361	0.44798	-1.502454	0.13298	0.193896
11380	TMEM31	1.891414078	-0.673253888	0.311904	-2.15853	0.030887	0.053199
6623	ARHGAP23	667.7413921	-0.673363652	0.169403	-3.97491	7.04E-05	0.000208
9240	KIAA1045	11.98303728	-0.673381499	0.23048	-2.921649	0.003482	0.007386
3212	POLM	513.1877913	-0.673402488	0.114546	-5.87887	4.13E-09	2.52E-08
9782	AKR1C3	3255.936038	-0.67341616	0.247399	-2.721987	0.006489	0.013003
10508	NSUN5P2	386.2653281	-0.673507258	0.272783	-2.469023	0.013548	0.025272
2712	AP2M1	8053.935125	-0.67358008	0.107464	-6.267973	3.66E-10	2.64E-09
3198	RPS23	14682.57581	-0.67361412	0.114465	-5.884897	3.98E-09	2.44E-08
4541	PDCD5	961.5098349	-0.673847444	0.13443	-5.012609	5.37E-07	2.32E-06
5373	CTSA	6492.700892	-0.674001016	0.147143	-4.580576	4.64E-06	1.69E-05
9913	CCR2	166.9297669	-0.674871555	0.251447	-2.683952	0.007276	0.014386

7024	SMOX	413.2259747	-0.674879332	0.177	-3.812878	0.000137	0.000383
7450	RBKS	393.1710077	-0.675125546	0.185623	-3.637082	0.000276	0.000725
10029	FDPSL2A	5.506601189	-0.675545428	0.255671	-2.64224	0.008236	0.016097
7966	PAMR1	276.8291803	-0.676119377	0.197553	-3.422469	0.000621	0.001527
1023	C19orf50	1423.991047	-0.676504742	0.081522	-8.298396	1.06E-16	2.02E-15
10653	UNC5C	27.16203009	-0.67658911	0.280376	-2.413149	0.015815	0.029099
5315	TMEM208	911.9548748	-0.676808983	0.146701	-4.61354	3.96E-06	1.46E-05
1504	CTNNBL1	1027.726451	-0.676838145	0.089547	-7.558442	4.08E-14	5.32E-13
6050	BBC3	186.9300693	-0.67693347	0.159561	-4.242474	2.21E-05	7.16E-05
12761	PCDHA4	82.74429746	-0.677117128	0.394722	-1.715429	0.086267	0.132506
6143	TREX1	352.5704161	-0.677253101	0.161097	-4.204015	2.62E-05	8.37E-05
9769	REM2	6.053971326	-0.677272206	0.248501	-2.725435	0.006422	0.012885
11669	HOXA11AS	21.29863882	-0.677317531	0.329176	-2.057613	0.039627	0.066564
8744	DTL	149.8383382	-0.677408312	0.217778	-3.110551	0.001867	0.004186
6694	CLIP2	1103.449343	-0.677461692	0.171852	-3.942131	8.08E-05	0.000236
10049	FSCN3	1.876332382	-0.67772658	0.257172	-2.635309	0.008406	0.016396
5855	STK11IP	374.0489591	-0.678196187	0.156138	-4.343579	1.40E-05	4.69E-05
10093	TLR10	33.18413392	-0.678492802	0.259039	-2.619271	0.008812	0.017113
4327	XYLT2	945.3921829	-0.678549316	0.131937	-5.142967	2.70E-07	1.23E-06
6346	PQLC2	310.9337438	-0.678651257	0.16532	-4.105088	4.04E-05	0.000125
9978	HOXA2	25.12111384	-0.678651628	0.255079	-2.660559	0.007801	0.015325
10189	PCDHGA5	25.15134052	-0.678765286	0.262446	-2.586306	0.009701	0.018663
11061	PCDHA5	15.68713767	-0.679307987	0.299317	-2.269528	0.023236	0.041177
9958	RNFT2	58.45017796	-0.679710954	0.254711	-2.66856	0.007618	0.014994
6672	ANKRD34A	24.61584665	-0.679795108	0.171974	-3.952883	7.72E-05	0.000227
3445	SEMA4F	273.9058159	-0.67988979	0.11907	-5.710009	1.13E-08	6.43E-08
3428	RPL26L1	329.5679003	-0.679981174	0.118803	-5.723624	1.04E-08	5.96E-08
8246	SLC26A6	151.4602275	-0.680334751	0.205301	-3.31384	0.00092	0.002187
10163	METTL7B	977.4087485	-0.680502771	0.262289	-2.594473	0.009474	0.018271
3896	AKT1S1	1115.051015	-0.680538792	0.125885	-5.406045	6.44E-08	3.24E-07
8285	OC1001282	9.799428588	-0.680583774	0.206303	-3.298948	0.00097	0.002296
7601	LHFP	1611.132335	-0.68068947	0.190165	-3.579458	0.000344	0.000888
10706	CYP2E1	25.97129685	-0.68089688	0.284274	-2.395216	0.016611	0.030411
3214	RBMX2	412.5629502	-0.68093145	0.115845	-5.877933	4.15E-09	2.53E-08
4820	RRP1	487.9799346	-0.681096703	0.139576	-4.879765	1.06E-06	4.32E-06
11130	PLEKHG6	77.68967005	-0.681229096	0.303746	-2.242763	0.024912	0.043873
7415	ATP5J2	1825.367072	-0.681361946	0.18669	-3.649695	0.000263	0.000694
14114	SNORA63	1.336530525	-0.681472526	0.531402	-1.282406	0.1997	0.277337
15312	DUXA	0.320559494	-0.681499659	0.7421	-0.91834	0.358441	0.458843
2698	EIF3I	3482.935532	-0.681764275	0.108502	-6.283417	3.31E-10	2.41E-09
7703	PPP1R13L	863.4525285	-0.681942294	0.192659	-3.539642	0.000401	0.00102
13974	C12orf71	0.478833982	-0.682359168	0.51386	-1.327907	0.184209	0.258385
5944	PRDX4	2471.404706	-0.682393732	0.158901	-4.294455	1.75E-05	5.77E-05
9468	EPHX4	25.76364321	-0.682467561	0.241063	-2.831078	0.004639	0.009604
7476	QTRT1	691.3740091	-0.682609379	0.188264	-3.625805	0.000288	0.000755
12278	FCRL3	35.35973017	-0.682901017	0.364706	-1.872471	0.061142	0.097608
10284	LOC643719	6.886290878	-0.68292613	0.267632	-2.551732	0.010719	0.02043

8649	TBX21	39.88791655	-0.682994907	0.21672	-3.151504	0.001624	0.003681
6221	SERPINB8	293.7198903	-0.683339112	0.163924	-4.168638	3.06E-05	9.65E-05
5617	RABAC1	1474.247096	-0.683439636	0.153093	-4.464223	8.04E-06	2.80E-05
7373	RASSF4	4172.057165	-0.68346801	0.186261	-3.669403	0.000243	0.000646
6249	BCL6	1664.429664	-0.684198809	0.164669	-4.154992	3.25E-05	0.000102
8165	C12orf68	28.3010092	-0.684407905	0.204508	-3.346606	0.000818	0.001964
12002	TMPRSS9	1.777635509	-0.684507669	0.349208	-1.960172	0.049976	0.081617
6091	ABHD14A	286.8354777	-0.684566947	0.162029	-4.224969	2.39E-05	7.69E-05
3939	CYB5R3	9586.068806	-0.684600866	0.127265	-5.379333	7.48E-08	3.72E-07
8011	AGPAT2	1202.06887	-0.685034961	0.201128	-3.40597	0.000659	0.001613
10640	FGF11	888.0280843	-0.685397919	0.283453	-2.418027	0.015605	0.028747
16453	FLJ43950	0.256125461	-0.685445688	1.169108	-0.586298	0.557675	0.664377
16436	SERPINB13	0.264001819	-0.685445689	1.157231	-0.592315	0.553639	0.660251
15017	KHDC1L	0.282836634	-0.685445693	0.682803	-1.00387	0.315441	0.411731
15267	AGBL1	0.265433793	-0.685445705	0.737758	-0.929092	0.352841	0.452976
15392	C7orf34	0.240125171	-0.685445721	0.767902	-0.892621	0.37206	0.473801
16828	KCNH5	0.284980467	-0.685445733	1.41667	-0.483843	0.628497	0.732044
16386	C6orf15	0.163073655	-0.685445876	1.135808	-0.603487	0.546185	0.653279
16710	OR2B11	0.183108142	-0.685445883	1.326228	-0.516839	0.605269	0.709986
17123	FRG2	0.205175073	-0.685445906	1.692842	-0.404908	0.685545	0.784755
17110	NXPH1	0.196264928	-0.685446064	1.674521	-0.409339	0.682291	0.781624
17134	RPS4Y2	0.138456929	-0.685446243	1.70381	-0.402302	0.687462	0.786444
17327	LIN28B	0.140544038	-0.685446268	1.900024	-0.360757	0.718281	0.812549
17333	CFHR5	0.124943001	-0.685446375	1.904982	-0.359818	0.718983	0.813015
17337	DMRTC2	0.121745177	-0.685446379	1.910835	-0.358716	0.719808	0.813806
17716	LOC283332	0.188642677	-0.685446753	2.605108	-0.263116	0.792461	0.876779
17853	CLPS	0.144093611	-0.685447013	2.91407	-0.23522	0.814038	0.893444
17789	C18orf62	0.121135844	-0.685447108	2.769106	-0.247534	0.804495	0.886442
17857	MT1B	0.131215288	-0.685447258	2.91407	-0.23522	0.814038	0.893444
17854	GAGE2A	0.125715902	-0.685447258	2.91407	-0.23522	0.814038	0.893444
17856	LOC14584[illegible]	0.164375848	-0.685447258	2.91407	-0.23522	0.814038	0.893444
7562	PTGER4	236.475992	-0.685694539	0.190902	-3.591861	0.000328	0.000851
10950	MYH11	4001.299334	-0.685755182	0.297647	-2.303921	0.021227	0.037997
14654	AMHR2	0.579706057	-0.685796724	0.614618	-1.11581	0.264503	0.353796
11338	ANKRD7	8.699966367	-0.685916027	0.316173	-2.169432	0.03005	0.05195
5734	ZNF628	176.4368385	-0.685952723	0.155694	-4.405784	1.05E-05	3.60E-05
3861	MAN1B1	2319.976187	-0.686084959	0.126433	-5.426451	5.75E-08	2.92E-07
9902	NEFH	23.52288979	-0.68629734	0.255382	-2.687339	0.007202	0.014257
12196	KIR2DS4	5.201560423	-0.686352487	0.361641	-1.897886	0.057711	0.092751
9559	CARNS1	25.31445021	-0.686747925	0.245569	-2.796559	0.005165	0.01059
6863	SLC25A1	2190.860542	-0.68677317	0.17715	-3.876785	0.000106	0.000302
1410	ARPC3	4467.022888	-0.687045867	0.089365	-7.688071	1.49E-14	2.08E-13
5155	NOTCH2NL	430.1574117	-0.687056934	0.146215	-4.698941	2.62E-06	9.94E-06
9313	DNAH17	36.28984081	-0.687141321	0.237282	-2.895883	0.003781	0.007958
3316	THOC4	625.6030011	-0.687191839	0.11835	-5.806441	6.38E-09	3.77E-08
3555	MAST2	700.2707198	-0.68731933	0.122028	-5.63249	1.78E-08	9.79E-08
7921	DNLZ	112.7099663	-0.687531575	0.199842	-3.440384	0.000581	0.001437

4333	DDX49	890.0513685	-0.687634897	0.133772	-5.140343	2.74E-07	1.24E-06
7793	KDELC1	380.9439056	-0.68802548	0.196411	-3.502996	0.00046	0.001157
7040	RNF32	58.88849021	-0.688298215	0.180973	-3.803322	0.000143	0.000397
6522	RPS19BP1	1276.718849	-0.688757742	0.171462	-4.016968	5.90E-05	0.000177
5523	FAM108A1	619.5533728	-0.688855071	0.152797	-4.508294	6.54E-06	2.32E-05
3769	OGFR	1244.666543	-0.689194442	0.125383	-5.496708	3.87E-08	2.01E-07
11762	CLEC12B	4.040987217	-0.689215043	0.338975	-2.033233	0.042029	0.07004
3799	NOP2	651.3887365	-0.68924421	0.125894	-5.474783	4.38E-08	2.26E-07
1356	YARS	1526.726234	-0.689661311	0.088573	-7.786338	6.90E-15	9.97E-14
5648	SLC25A22	365.0002897	-0.689671035	0.155024	-4.448803	8.64E-06	3.00E-05
6632	C17orf90	313.6837115	-0.689880131	0.173699	-3.971688	7.14E-05	0.000211
8632	FAM116B	288.9449688	-0.689880709	0.218265	-3.160749	0.001574	0.003573
12684	CLDN6	1.168340313	-0.689938458	0.395915	-1.742644	0.081396	0.125783
5116	HSD17B10	1193.626951	-0.689986321	0.146169	-4.720462	2.35E-06	9.02E-06
8572	SNHG5	2009.831776	-0.690093436	0.216851	-3.182339	0.001461	0.003341
10367	PPM1J	23.95570035	-0.690185337	0.273971	-2.519194	0.011762	0.022239
9171	CCDC40	162.7885909	-0.690370666	0.23411	-2.948918	0.003189	0.006816
8898	CDC25A	36.17511859	-0.690413848	0.226178	-3.052526	0.002269	0.004999
9307	FAM159A	13.85557157	-0.690532671	0.238297	-2.897781	0.003758	0.007915
2067	HK1	3004.896938	-0.690763544	0.100374	-6.881868	5.91E-12	5.60E-11
723	CAPZB	4330.937899	-0.691008933	0.077137	-8.958205	3.30E-19	8.95E-18
3846	TMEM164	374.5080429	-0.691074434	0.127146	-5.435282	5.47E-08	2.79E-07
7136	MX1	1455.886881	-0.69124763	0.183305	-3.771023	0.000163	0.000447
9155	CD226	41.96178466	-0.691274695	0.233804	-2.956643	0.00311	0.006659
2549	MOBKL2A	1667.431217	-0.691646074	0.107631	-6.42609	1.31E-10	1.01E-09
3403	GRK6	476.4341376	-0.692258676	0.120465	-5.746532	9.11E-09	5.25E-08
3879	NAP1L1	8346.525854	-0.692372102	0.127852	-5.41541	6.11E-08	3.09E-07
7690	COX6B1	3828.340466	-0.692385497	0.195403	-3.54337	0.000395	0.001007
10800	LGALS3	8226.288239	-0.692490801	0.29333	-2.360794	0.018236	0.033096
8397	CRNA0017	192.5422208	-0.69336948	0.213373	-3.249561	0.001156	0.002698
10573	HOXA11	67.90519231	-0.693413596	0.283494	-2.445956	0.014447	0.026783
3841	C11orf24	807.0355746	-0.69372542	0.127517	-5.440259	5.32E-08	2.72E-07
11981	KFZp566F09	1.455012762	-0.694225395	0.353307	-1.964935	0.049422	0.080848
13415	ELFN2	147.0982465	-0.69427412	0.460015	-1.509244	0.131236	0.191741
4215	EEF1D	6850.06334	-0.694532028	0.133527	-5.201448	1.98E-07	9.20E-07
8249	TSPYL2	1013.981976	-0.694704504	0.209675	-3.313252	0.000922	0.002191
5350	STMN1	1319.764605	-0.6947869	0.151272	-4.59296	4.37E-06	1.60E-05
8061	PDE4B	555.218876	-0.694846539	0.205086	-3.388066	0.000704	0.001712
3365	FBXL19	354.6277102	-0.694914471	0.120449	-5.769386	7.96E-09	4.63E-08
6177	LRRC37A3	182.7381134	-0.694914812	0.165973	-4.186925	2.83E-05	8.97E-05
5312	LRRC23	430.0418943	-0.695015763	0.150627	-4.614142	3.95E-06	1.46E-05
13281	LOC647859	4.539551513	-0.695288499	0.448318	-1.550881	0.12093	0.178477
2980	AGAP3	1034.706688	-0.695551183	0.114984	-6.049116	1.46E-09	9.58E-09
8683	PRH2	6.214893032	-0.69595914	0.221802	-3.137754	0.001702	0.003843
4611	ZDHHC12	498.7841389	-0.695997073	0.139648	-4.983938	6.23E-07	2.65E-06
5418	EPN1	3126.544944	-0.696005497	0.152736	-4.556909	5.19E-06	1.88E-05
5460	SPARC	56555.11612	-0.69611467	0.153385	-4.538338	5.67E-06	2.04E-05

4565	IMPDH2	1962.320062	-0.696180483	0.139167	-5.002483	5.66E-07	2.43E-06
5802	ARL2	1222.407539	-0.696513221	0.159258	-4.3735	1.22E-05	4.13E-05
2753	C3orf21	377.2476773	-0.696786823	0.111701	-6.237961	4.43E-10	3.16E-09
12722	ERAS	1.762424134	-0.696921756	0.403943	-1.725299	0.084474	0.13015
8526	C1orf152	15.06583128	-0.696922286	0.218094	-3.195512	0.001396	0.003209
14904	SAGE1	1.115362781	-0.69749319	0.674194	-1.034559	0.300875	0.395696
1863	POLA2	233.9606729	-0.69787507	0.097956	-7.124361	1.05E-12	1.10E-11
6209	MICAL1	766.9680122	-0.698026175	0.167264	-4.173196	3.00E-05	9.48E-05
8078	C1orf135	11.77765893	-0.698095567	0.206571	-3.379441	0.000726	0.001762
5056	RAD9A	225.5754	-0.698104929	0.147164	-4.743714	2.10E-06	8.13E-06
14893	PTPRQ	0.925107216	-0.698165589	0.672203	-1.038624	0.29898	0.393494
9178	SLC27A5	84.33922548	-0.698236283	0.236927	-2.947055	0.003208	0.006853
11613	KEL	3.970561114	-0.698356792	0.336271	-2.07677	0.037823	0.063839
4368	BOLA2	984.7306597	-0.698485688	0.13641	-5.120497	3.05E-07	1.37E-06
6293	FAM38A	2756.202677	-0.698918219	0.169253	-4.129441	3.64E-05	0.000113
5513	SNX8	451.460489	-0.69895739	0.154851	-4.513755	6.37E-06	2.26E-05
5676	PIR	230.8298992	-0.699204411	0.15768	-4.434322	9.24E-06	3.19E-05
7110	RASSF5	628.9014097	-0.699490108	0.185022	-3.780582	0.000156	0.000431
12101	B3GNT3	248.3762888	-0.699528699	0.362733	-1.928497	0.053793	0.087134
4276	ZSWIM4	368.1191669	-0.699927339	0.135426	-5.16833	2.36E-07	1.08E-06
2617	MOV10	1014.929576	-0.699971252	0.110085	-6.358483	2.04E-10	1.53E-09
9532	CRNA001(	12.49925211	-0.700003189	0.24954	-2.805174	0.005029	0.010341
4249	PKN1	3449.101003	-0.700179808	0.135115	-5.182117	2.19E-07	1.01E-06
6922	SPDYE8P	18.58987638	-0.700184367	0.18161	-3.855437	0.000116	0.000327
6069	PDXK	2869.596959	-0.70020486	0.165274	-4.236629	2.27E-05	7.33E-05
16636	PRDM14	0.387402542	-0.700351414	1.299962	-0.538748	0.590061	0.695226
3105	SDF4	3687.646215	-0.700555073	0.117528	-5.960744	2.51E-09	1.59E-08
4602	SRD5A3	577.9432785	-0.700718787	0.140478	-4.988097	6.10E-07	2.60E-06
4952	RPS27A	9557.478129	-0.700876597	0.146154	-4.795468	1.62E-06	6.42E-06
12909	ADARB2	1.986457363	-0.700883878	0.419384	-1.671223	0.094678	0.143758
13699	HIST1H2AI	1.219884387	-0.700901832	0.494398	-1.417687	0.156282	0.223598
4451	SLC39A11	608.4934879	-0.701874148	0.138422	-5.070537	3.97E-07	1.75E-06
13907	PNLDC1	1.204035	-0.702271043	0.521004	-1.347918	0.177685	0.250435
7383	TMSB15B	29.69055035	-0.702356292	0.191774	-3.662426	0.00025	0.000663
10410	FAHD2B	100.4768216	-0.70239811	0.280218	-2.506609	0.01219	0.022952
10132	C5orf58	8.515054953	-0.702681538	0.269803	-2.604429	0.009203	0.017803
9634	FYB	1124.531147	-0.702836579	0.253935	-2.767787	0.005644	0.011483
2030	AP2A1	1913.519803	-0.703486018	0.101528	-6.928958	4.24E-12	4.09E-11
2803	MAZ	3300.006348	-0.703809525	0.113692	-6.190476	6.00E-10	4.19E-09
4643	CARS2	897.9863479	-0.704087106	0.14182	-4.964653	6.88E-07	2.90E-06
10355	NFATC2	110.6891041	-0.704417485	0.278925	-2.525473	0.011554	0.021871
7887	NKX3-1	48.94283219	-0.704423393	0.203914	-3.454508	0.000551	0.00137
11517	SMPDL3B	149.1540756	-0.704519261	0.333772	-2.110782	0.034791	0.059213
10494	GRM6	5.805271903	-0.705030959	0.285097	-2.472952	0.0134	0.025029
5013	HVCN1	211.4653714	-0.705173223	0.147983	-4.765236	1.89E-06	7.38E-06
5790	B9D2	205.3744099	-0.705278372	0.161113	-4.377528	1.20E-05	4.06E-05
8722	CENPK	55.63030079	-0.705407091	0.226071	-3.120284	0.001807	0.00406

9534	FADS2	1221.53118	-0.705471597	0.251519	-2.804848	0.005034	0.010349
4633	FKBP8	5336.736026	-0.70550346	0.1419	-4.971839	6.63E-07	2.81E-06
2614	RALY	2617.285396	-0.705579305	0.110899	-6.362375	1.99E-10	1.49E-09
2028	TBL2	1194.543128	-0.705746125	0.101822	-6.931189	4.17E-12	4.03E-11
6077	RACGAP1	442.4417041	-0.70604011	0.166891	-4.230557	2.33E-05	7.52E-05
4887	FRMD4A	1003.727653	-0.706175341	0.146091	-4.833812	1.34E-06	5.37E-06
6615	ZNF580	402.5030014	-0.706301985	0.17756	-3.977823	6.95E-05	0.000206
5213	SORBS3	2309.388285	-0.706442969	0.151174	-4.673048	2.97E-06	1.12E-05
2158	MKL1	1105.067328	-0.706994569	0.103966	-6.800269	1.04E-11	9.48E-11
10612	TRAT1	50.29860738	-0.707172334	0.290686	-2.432774	0.014984	0.027676
7551	LOC154761	117.5637427	-0.707435349	0.196785	-3.594973	0.000324	0.000842
6138	GAB3	178.1156267	-0.707694211	0.168275	-4.205573	2.60E-05	8.32E-05
9017	HPCAL1	5099.755159	-0.70780045	0.23544	-3.006283	0.002645	0.005748
4371	NCLN	1504.758329	-0.708100641	0.13833	-5.11891	3.07E-07	1.38E-06
6944	IL1R1	2114.664167	-0.70826858	0.184103	-3.847141	0.00012	0.000337
6678	ATAD3A	458.9335537	-0.708393619	0.179324	-3.950364	7.80E-05	0.000229
4506	ZNF428	550.1380911	-0.708981449	0.140919	-5.031143	4.88E-07	2.12E-06
8060	LHX6	121.6509113	-0.708990196	0.209236	-3.388463	0.000703	0.001709
9397	RYR1	78.60392614	-0.709003981	0.247905	-2.859977	0.004237	0.008837
11411	NUDT10	13.38497313	-0.709075444	0.330506	-2.145424	0.031919	0.054828
3407	MRTO4	700.1218885	-0.709334712	0.123531	-5.742159	9.35E-09	5.38E-08
8927	SKAP1	358.1185194	-0.709335497	0.23319	-3.041882	0.002351	0.005162
6319	LHPP	365.5355123	-0.709419548	0.172305	-4.117222	3.83E-05	0.000119
4014	ALDH16A1	588.5385111	-0.709515716	0.133118	-5.32996	9.82E-08	4.80E-07
8043	NID2	1312.120915	-0.710126234	0.209162	-3.395105	0.000686	0.001672
12998	LASS1	14.51473326	-0.710474401	0.431796	-1.645395	0.099888	0.150632
2050	RPS6KA4	675.4138142	-0.710515708	0.102951	-6.901487	5.15E-12	4.92E-11
3468	CPSF4	404.9419448	-0.710604231	0.124741	-5.696627	1.22E-08	6.91E-08
10030	MPP3	47.49876951	-0.71073517	0.26901	-2.642039	0.008241	0.016105
11495	DNAH8	2.958470087	-0.710830629	0.335038	-2.121644	0.033868	0.05775
3574	RPS3	17864.65079	-0.711113285	0.126512	-5.620898	1.90E-08	1.04E-07
10020	ANKRD24	61.9389248	-0.711145639	0.268824	-2.645398	0.008159	0.015961
2358	PRKCSH	6576.486821	-0.711407278	0.107768	-6.601288	4.08E-11	3.39E-10
7216	KRT18	10304.17104	-0.711611023	0.19034	-3.738636	0.000185	0.000503
7363	PLK4	72.15433838	-0.711784615	0.193796	-3.672857	0.00024	0.000639
10838	MPP4	1.967566882	-0.711929228	0.303477	-2.345905	0.018981	0.034328
3641	ZC3H3	503.7984296	-0.712406754	0.127724	-5.577726	2.44E-08	1.31E-07
7996	ZFPM1	81.03726396	-0.712555449	0.208904	-3.410916	0.000647	0.001587
8888	C2orf84	5.615393887	-0.712947048	0.233234	-3.056786	0.002237	0.004934
2955	SNRPB	2073.19847	-0.713231161	0.117545	-6.067743	1.30E-09	8.60E-09
12445	PRO0628	1.111492586	-0.713750181	0.391743	-1.821984	0.068457	0.107821
1989	COPS6	2250.176519	-0.713768344	0.10229	-6.977922	3.00E-12	2.95E-11
11954	SLC25A41	2.209069095	-0.713954522	0.361856	-1.973033	0.048492	0.079512
13746	RHO	0.730114926	-0.714637097	0.509267	-1.403265	0.160538	0.228917
8810	EBF4	245.8658869	-0.714696005	0.23176	-3.083775	0.002044	0.004547
11967	C1orf182	1.366356665	-0.71472636	0.362901	-1.969481	0.048898	0.080091
6605	KRT10	369.3211198	-0.714853378	0.17955	-3.981363	6.85E-05	0.000203

6129	ZFAND2A	420.2549906	-0.715233188	0.169972	-4.207944	2.58E-05	8.24E-05
4766	DONSON	183.9552593	-0.715262425	0.145951	-4.900693	9.55E-07	3.93E-06
4581	PUS1	341.5188992	-0.715338377	0.143183	-4.995976	5.85E-07	2.50E-06
4289	ADCY3	1047.610654	-0.715664216	0.138675	-5.16074	2.46E-07	1.12E-06
8697	CLEC7A	285.8217729	-0.715707015	0.228583	-3.131062	0.001742	0.003925
9041	IL32	9104.205866	-0.715910013	0.238729	-2.998844	0.00271	0.005876
11953	)C1001290	2.927175463	-0.716011537	0.362829	-1.973415	0.048448	0.079447
3515	ADCK4	750.6919566	-0.71614483	0.126452	-5.663359	1.48E-08	8.28E-08
4110	CD81	14652.84018	-0.716233637	0.135771	-5.2753	1.33E-07	6.32E-07
10005	PCDHB8	81.48329352	-0.716333311	0.270228	-2.650844	0.008029	0.01573
3279	ITGAE	190.3885027	-0.716448847	0.122876	-5.830643	5.52E-09	3.30E-08
5720	H1FX	1185.405542	-0.716564205	0.162481	-4.410138	1.03E-05	3.54E-05
13906	TNNT2	2.607318367	-0.716672675	0.53167	-1.347966	0.177669	0.250433
4323	ANKRD39	197.236032	-0.716817537	0.139296	-5.145999	2.66E-07	1.21E-06
6518	DNAJC4	828.4007575	-0.716970589	0.178344	-4.020152	5.82E-05	0.000175
4709	RPP21	227.5850854	-0.717273285	0.145538	-4.928425	8.29E-07	3.45E-06
9424	HOTAIR	15.13833125	-0.717360803	0.251726	-2.849763	0.004375	0.0091
1780	PSMD13	2152.220847	-0.717397533	0.099549	-7.20648	5.74E-13	6.32E-12
8653	SLIT1	6.937983254	-0.717613309	0.227844	-3.149581	0.001635	0.003704
10971	WIPF3	39.70520232	-0.717781096	0.312597	-2.296188	0.021665	0.038707
13340	GAD1	124.0993769	-0.718097029	0.468882	-1.531509	0.125644	0.184606
5753	C9orf167	540.0222513	-0.71826314	0.163483	-4.393516	1.12E-05	3.80E-05
10604	LOC645431	16.99751285	-0.718787307	0.295168	-2.435179	0.014884	0.027513
3672	CAD	798.8101017	-0.718819101	0.129411	-5.554565	2.78E-08	1.49E-07
2795	SLC38A10	2939.224758	-0.719128107	0.116043	-6.197097	5.75E-10	4.03E-09
6931	FKBP2	1460.824858	-0.719138529	0.186732	-3.851175	0.000118	0.000332
4777	SMYD3	230.5509052	-0.719150894	0.146885	-4.896013	9.78E-07	4.01E-06
6560	LOC441208	66.1538582	-0.719570141	0.179821	-4.00158	6.29E-05	0.000188
5092	TRAF5	406.9575892	-0.720656787	0.152368	-4.729712	2.25E-06	8.65E-06
10654	PLXNC1	300.1937202	-0.720734153	0.298683	-2.413041	0.01582	0.029105
15570	GABRA2	1.452519317	-0.720824091	0.855475	-0.842601	0.399452	0.502868
9229	GNLY	176.8330681	-0.721012286	0.246231	-2.928199	0.003409	0.007241
10717	C4orf47	77.79603639	-0.721197617	0.301714	-2.390334	0.016833	0.030787
11747	C8ORFK29	3.118631502	-0.72177649	0.354187	-2.037842	0.041566	0.069356
9142	FBXO27	295.0685304	-0.721838992	0.243783	-2.960988	0.003067	0.006575
11877	DTHD1	13.361292	-0.721921725	0.361474	-1.997162	0.045808	0.075598
9825	MST1R	23.38200512	-0.722370884	0.266548	-2.710101	0.006726	0.013419
10630	AHNAK2	3938.025859	-0.722720235	0.298462	-2.421478	0.015458	0.028494
5841	COTL1	3378.076632	-0.722861463	0.166169	-4.350149	1.36E-05	4.57E-05
6400	ATP8B2	638.6265088	-0.722861572	0.177346	-4.075985	4.58E-05	0.00014
1344	FTSJ1	569.1861381	-0.723022119	0.092635	-7.805065	5.95E-15	8.67E-14
4552	ZNF358	1308.61853	-0.723406247	0.144446	-5.00816	5.50E-07	2.37E-06
12682	SOAT2	5.325879954	-0.723538783	0.415027	-1.743354	0.081272	0.125612
13042	GNGT1	13.45355299	-0.724099063	0.443643	-1.632165	0.102645	0.154266
4140	TBC1D7	300.7859828	-0.724280309	0.137719	-5.2591	1.45E-07	6.85E-07
4111	RPL10	17490.2513	-0.724346936	0.13731	-5.275248	1.33E-07	6.32E-07
7953	FAM182B	34.43034692	-0.724804927	0.211571	-3.425817	0.000613	0.001511

3511	PXN	4837.14912	-0.724886836	0.127973	-5.664373	1.48E-08	8.24E-08
12959	SLC18A1	0.900035016	-0.724946354	0.438203	-1.654361	0.098054	0.148311
3121	GNB2	3962.338884	-0.724971595	0.121877	-5.948365	2.71E-09	1.70E-08
8589	LIN7B	35.2682555	-0.725410391	0.228297	-3.177485	0.001486	0.00339
8715	MNDA	361.2516072	-0.725411743	0.232122	-3.125135	0.001777	0.003998
6877	ADCK5	122.7852167	-0.725484113	0.187355	-3.872253	0.000108	0.000307
12668	LAD1	290.5270519	-0.725798023	0.415206	-1.748042	0.080457	0.124489
6689	IFI27L2	465.5032885	-0.725816389	0.183986	-3.944965	7.98E-05	0.000234
3022	RNF215	398.1581036	-0.72599182	0.120645	-6.017575	1.77E-09	1.15E-08
8240	SLC22A18	1097.235037	-0.726899328	0.219257	-3.315278	0.000916	0.002178
12897	PROM2	234.9061177	-0.726950209	0.434368	-1.67358	0.094213	0.143175
11766	ADORA1	154.9563276	-0.727113115	0.357909	-2.031556	0.042199	0.070299
5578	FAM129A	1169.030553	-0.727291589	0.162196	-4.484032	7.32E-06	2.57E-05
15975	ISX	0.598935911	-0.72748972	1.009169	-0.72088	0.470983	0.577887
6283	C11orf80	260.7510661	-0.727693702	0.175826	-4.138715	3.49E-05	0.000109
5882	FASN	1254.403994	-0.727833259	0.168014	-4.331968	1.48E-05	4.92E-05
4590	MAGED1	3461.644478	-0.727918896	0.145816	-4.992039	5.97E-07	2.55E-06
5869	PPAN	412.1378962	-0.728146938	0.167896	-4.33689	1.45E-05	4.83E-05
13103	FAM58B	0.625680514	-0.728227419	0.452416	-1.609643	0.107476	0.160775
2505	PSMD4	2274.512857	-0.728377195	0.11279	-6.457797	1.06E-10	8.31E-10
10906	PPFIA2	12.30413884	-0.728575941	0.314144	-2.31924	0.020382	0.036632
14646	C1orf194	1.181256759	-0.728805666	0.651613	-1.118464	0.263369	0.352471
8512	NDUFB7	1724.947207	-0.728855987	0.227715	-3.200745	0.001371	0.003156
3083	FTH1	50910.95011	-0.728870561	0.121983	-5.975194	2.30E-09	1.46E-08
6695	THAP7	348.1038477	-0.728989689	0.184929	-3.942007	8.08E-05	0.000237
3495	METTL1	456.2062261	-0.729590407	0.128486	-5.67838	1.36E-08	7.63E-08
6396	C1orf96	245.7581936	-0.729730893	0.178979	-4.077198	4.56E-05	0.00014
7260	P704P	16.91982203	-0.729770549	0.196052	-3.722333	0.000197	0.000533
5475	COL18A1	10572.30599	-0.730159237	0.16109	-4.532628	5.83E-06	2.09E-05
3063	CD63	19807.61708	-0.730222282	0.121892	-5.990749	2.09E-09	1.34E-08
8375	CHST2	859.6385571	-0.730571543	0.224169	-3.259019	0.001118	0.002616
4362	TAZ	466.9772371	-0.730624884	0.142562	-5.124974	2.98E-07	1.34E-06
16046	LOC285401	0.419170972	-0.730676199	1.046668	-0.698098	0.485116	0.592594
14298	SOST	2.126691179	-0.731030179	0.594301	-1.230068	0.218672	0.299775
9063	HLA-DRB5	3232.706799	-0.731149442	0.244227	-2.993728	0.002756	0.00596
6278	LOC80154	322.3074314	-0.731270228	0.176624	-4.140258	3.47E-05	0.000108
3393	IRAK1	2192.46949	-0.73144588	0.127153	-5.7525	8.79E-09	5.08E-08
7958	MMP25	59.16961516	-0.731473879	0.213643	-3.423813	0.000617	0.001521
10166	TRIM9	384.6443453	-0.731567665	0.282007	-2.594145	0.009483	0.018283
16655	MAGEB18	0.320379667	-0.731638119	1.372793	-0.532956	0.594064	0.699145
7471	AOC3	1005.350685	-0.731719383	0.201706	-3.627655	0.000286	0.00075
4522	SOX12	749.1698024	-0.731977863	0.145704	-5.023743	5.07E-07	2.20E-06
7531	HEXIM2	169.7316503	-0.732034368	0.203129	-3.603786	0.000314	0.000816
13501	HILS1	1.032875394	-0.73232858	0.491908	-1.488751	0.136553	0.19825
4829	RPUSD1	369.8952977	-0.732545471	0.150218	-4.876563	1.08E-06	4.38E-06
5028	APRT	1882.953482	-0.732948909	0.153961	-4.760601	1.93E-06	7.52E-06
3906	ETV5	546.6425133	-0.733020426	0.135786	-5.398356	6.73E-08	3.37E-07

3989	LOC374443	134.8314195	-0.733146039	0.137056	-5.349234	8.83E-08	4.34E-07
5575	CCND3	1500.770641	-0.73317856	0.163436	-4.486029	7.26E-06	2.55E-05
1591	C12orf32	553.4135173	-0.733227426	0.098652	-7.432475	1.07E-13	1.31E-12
11428	CECR7	13.39022959	-0.73348835	0.342739	-2.140076	0.032349	0.055483
4797	DGKZ	1067.106948	-0.73397699	0.15015	-4.888298	1.02E-06	4.16E-06
12513	SCARNA6	2.270487528	-0.734135607	0.407885	-1.799861	0.071883	0.1126
7284	RAD51AP1	92.63969834	-0.734221639	0.198103	-3.706264	0.00021	0.000566
9002	RDH13	497.4302492	-0.734339663	0.243845	-3.011503	0.0026	0.00566
9458	ZGLP1	22.95157959	-0.73448725	0.259099	-2.834771	0.004586	0.009504
4710	VPS28	2031.47578	-0.734635344	0.149062	-4.928378	8.29E-07	3.45E-06
6587	LOC441204	120.1111343	-0.734655394	0.184171	-3.988977	6.64E-05	0.000197
11129	NKD1	8.732691305	-0.734690758	0.327558	-2.242934	0.024901	0.043857
7273	ARHGAP11	185.9668112	-0.734772481	0.197887	-3.713091	0.000205	0.000552
1688	CHFR	459.5858297	-0.735128202	0.100527	-7.312767	2.62E-13	3.04E-12
780	YBX1	6666.586776	-0.735182713	0.083475	-8.807179	1.28E-18	3.22E-17
8211	P2RX7	177.2148298	-0.735462893	0.221057	-3.327021	0.000878	0.002095
13157	FST	52.63581165	-0.73552716	0.461982	-1.592111	0.11136	0.165901
7070	LAMA4	3917.008164	-0.735804828	0.193873	-3.795287	0.000147	0.000409
8488	SLC16A6	68.27093552	-0.735828452	0.229094	-3.211901	0.001319	0.003045
8083	GCHFR	304.2734667	-0.736009415	0.217898	-3.377772	0.000731	0.001772
2952	PABPC3	162.0638113	-0.736100841	0.121275	-6.069696	1.28E-09	8.51E-09
5388	AGRN	9814.100579	-0.736168596	0.160947	-4.57399	4.79E-06	1.74E-05
3308	PLSCR3	750.4105649	-0.736564035	0.126755	-5.810905	6.21E-09	3.68E-08
8471	DIAPH3	88.5092136	-0.737097034	0.228984	-3.218986	0.001286	0.002977
5678	CBX8	110.8029457	-0.73714145	0.166301	-4.432563	9.31E-06	3.21E-05
5823	TMEM229B	199.1340869	-0.737523684	0.169095	-4.361581	1.29E-05	4.35E-05
8483	MYO15A	18.50507762	-0.737540876	0.2295	-3.213684	0.00131	0.003028
10513	EPPK1	264.2087841	-0.7376933	0.298937	-2.467719	0.013598	0.025352
3238	SNRPF	640.3262551	-0.738113612	0.125923	-5.86162	4.58E-09	2.77E-08
8154	SIRPB1	338.5082593	-0.738189606	0.220426	-3.348929	0.000811	0.00195
16491	GSX2	0.350750179	-0.738366591	1.284349	-0.574896	0.565362	0.671982
8875	TNFRSF4	155.8512359	-0.738874939	0.241275	-3.062382	0.002196	0.00485
3178	TRAF7	1502.43359	-0.738878306	0.125309	-5.896435	3.71E-09	2.29E-08
6907	CNIH3	33.03576168	-0.73915805	0.191471	-3.860426	0.000113	0.000321
6267	S100A13	1756.108965	-0.739256645	0.178193	-4.148638	3.34E-05	0.000105
12265	DMKN	329.5019133	-0.73971226	0.393788	-1.878451	0.06032	0.096398
10482	IL17D	7.675363347	-0.739847495	0.298478	-2.478735	0.013185	0.024655
11780	HIST2H3C	1.090978475	-0.739946783	0.364825	-2.028224	0.042537	0.070779
11828	KCNK3	2030.312272	-0.740023875	0.367769	-2.012195	0.044199	0.073246
4349	EXOSC4	284.5810283	-0.740332533	0.144257	-5.132023	2.87E-07	1.29E-06
4155	EEF1G	18339.58235	-0.740382996	0.141065	-5.248534	1.53E-07	7.23E-07
6351	RPL19P12	69.48735236	-0.740441947	0.180603	-4.099838	4.13E-05	0.000128
12666	RPS28	562.9111411	-0.740517449	0.422879	-1.751131	0.079923	0.123684
3955	RPS15A	10381.31885	-0.740598574	0.137833	-5.373166	7.74E-08	3.83E-07
13054	CRYBB3	9.345862472	-0.740787073	0.454808	-1.628789	0.103358	0.155195
4354	IRF3	949.6639096	-0.740874576	0.144449	-5.128968	2.91E-07	1.31E-06
7895	FDXR	547.9823322	-0.740997396	0.214731	-3.450824	0.000559	0.001388

6411	NOMO2	1931.340535	-0.741009267	0.181998	-4.071529	4.67E-05	0.000143
1952	RFC4	237.8515208	-0.741255779	0.105377	-7.034324	2.00E-12	2.01E-11
13030	CALCR	213.8902399	-0.742013341	0.4539	-1.634751	0.102101	0.153591
9083	NT5E	987.381351	-0.742360296	0.248693	-2.985042	0.002835	0.006119
9831	CLEC5A	113.5077584	-0.742871965	0.274199	-2.709243	0.006744	0.013446
5452	FAM195B	1038.156587	-0.743142507	0.163641	-4.5413	5.59E-06	2.01E-05
16712	FAM48B1	0.254709611	-0.743153356	1.439277	-0.516338	0.605618	0.710311
16553	FGF10	0.233166366	-0.743153431	1.330121	-0.558711	0.576359	0.682487
15464	PGLYRP4	0.271573051	-0.743153461	0.852776	-0.871452	0.383507	0.486105
15138	HIST1H2AI	0.248929233	-0.743153471	0.771408	-0.963373	0.33536	0.434232
16559	NHEG1	0.209441509	-0.743153481	1.334886	-0.556717	0.577721	0.683852
15922	TMEM229A	0.215639432	-0.743153497	1.010462	-0.735459	0.46206	0.568825
15727	HIST1H3F	0.197588653	-0.743153525	0.933687	-0.795934	0.42607	0.531008
16369	PRDM9	0.195212887	-0.74315353	1.222064	-0.608113	0.543112	0.650348
16533	RXFP3	0.196203219	-0.743153533	1.318773	-0.563519	0.573082	0.679427
16192	CLCA3P	0.176660565	-0.743153566	1.133437	-0.655664	0.51204	0.619843
16208	CA6	0.175309335	-0.743153566	1.137899	-0.653093	0.513696	0.621215
16688	IGFL3	0.17417693	-0.743153594	1.426794	-0.520856	0.602467	0.70759
16693	C1orf129	0.173505593	-0.743153594	1.428606	-0.520195	0.602928	0.707961
16697	HMX2	0.172116996	-0.743153594	1.429661	-0.519811	0.603195	0.708105
16530	C1orf158	0.153801779	-0.743153632	1.316078	-0.564673	0.572296	0.678619
17153	FLJ46321	0.170522041	-0.743153648	1.869901	-0.397429	0.691051	0.789675
16703	TSPAN16	0.150711685	-0.743153654	1.435216	-0.517799	0.604598	0.709497
16829	TMEM190	0.182640433	-0.743153674	1.53603	-0.483815	0.628517	0.732044
17349	GPR78	0.151287739	-0.74315381	2.101589	-0.353615	0.723627	0.817558
17647	MYL10	0.163292803	-0.743154185	2.650898	-0.280341	0.779216	0.865497
17681	PSG11	0.136096622	-0.743154221	2.721189	-0.273099	0.784777	0.869997
17758	SPRYD5	0.134275147	-0.743154325	2.91407	-0.255023	0.798705	0.881599
16437	CFHR4	0.294808615	-0.743214826	1.254887	-0.592257	0.553679	0.660258
11062	ERAP2	2136.233724	-0.743415254	0.327621	-2.269131	0.02326	0.041216
14625	LOC400891	0.750486496	-0.743460001	0.66068	-1.125294	0.260464	0.349085
9837	SOCS1	221.9920221	-0.743658156	0.274662	-2.707541	0.006778	0.013506
7608	C10orf10	8482.020914	-0.743669114	0.207863	-3.577687	0.000347	0.000893
12904	CALN1	1.026915622	-0.743870171	0.444838	-1.672227	0.09448	0.143513
6742	ASPSCR1	384.718886	-0.743975687	0.189617	-3.92356	8.72E-05	0.000254
8047	PYGL	1740.467143	-0.744160331	0.219278	-3.393687	0.00069	0.00168
1142	PDAP1	2102.956471	-0.744236771	0.091855	-8.102302	5.39E-16	9.26E-15
7009	RNF39	7.400392888	-0.744362965	0.194881	-3.819585	0.000134	0.000374
13425	ACCN1	20.85383632	-0.744398072	0.493968	-1.506976	0.131817	0.192458
12232	CLEC17A	4.856932887	-0.744621124	0.394526	-1.88738	0.059109	0.094719
8447	FLJ39739	3.676910353	-0.744646145	0.230616	-3.228949	0.001242	0.002883
4908	RNF181	1693.955758	-0.744864818	0.154445	-4.82284	1.42E-06	5.65E-06
5540	OAS3	1445.460973	-0.744973698	0.165448	-4.502756	6.71E-06	2.37E-05
1790	VASP	1880.644886	-0.74515066	0.10353	-7.197416	6.14E-13	6.72E-12
1700	CPNE1	1909.30155	-0.745182729	0.102173	-7.293379	3.02E-13	3.48E-12
6684	GAS5	1994.590158	-0.745428154	0.188859	-3.947009	7.91E-05	0.000232
9147	ITPR3	2067.450673	-0.7460482	0.252104	-2.959283	0.003084	0.006608

4036	NR2C2AP	340.3529422	-0.746245364	0.140427	-5.314118	1.07E-07	5.21E-07
10559	GPC2	10.16293294	-0.74664176	0.304853	-2.449188	0.014318	0.026579
9563	PCDP1	142.8684704	-0.746800272	0.267146	-2.795473	0.005182	0.010622
3180	MRPL36	368.8494151	-0.746860749	0.126706	-5.894442	3.76E-09	2.32E-08
3659	MEX3D	282.3440304	-0.746862171	0.134205	-5.565073	2.62E-08	1.40E-07
9413	EGFL7	1365.120862	-0.747213345	0.26196	-2.852397	0.004339	0.009035
11208	PRSS30P	4.080471214	-0.748216002	0.337219	-2.218784	0.026501	0.046347
3966	MRPS17	251.9760193	-0.748282292	0.139494	-5.364251	8.13E-08	4.02E-07
6577	43893	92.14638857	-0.748852113	0.187605	-3.991648	6.56E-05	0.000196
9040	C8orf48	24.80014194	-0.74906652	0.249789	-2.998798	0.00271	0.005876
5142	STRA13	370.4121485	-0.749453191	0.159222	-4.706979	2.51E-06	9.58E-06
6549	ITPRIPL1	29.31558231	-0.749585305	0.187114	-4.006034	6.17E-05	0.000185
2209	COX19	279.7590025	-0.74959987	0.111103	-6.746894	1.51E-11	1.34E-10
8827	NCS1	582.6146014	-0.749635372	0.243338	-3.080637	0.002066	0.004587
7676	OC1002872	10.84971023	-0.750612131	0.211557	-3.548039	0.000388	0.000991
14319	DMRTA2	0.450703594	-0.750865135	0.613183	-1.224537	0.22075	0.30218
3831	TFPT	218.1162268	-0.750895182	0.137903	-5.445114	5.18E-08	2.65E-07
2912	TMEM79	118.1180296	-0.750958718	0.12305	-6.102858	1.04E-09	7.01E-09
3586	STOML1	431.4974059	-0.751035345	0.133802	-5.613023	1.99E-08	1.09E-07
3567	TALDO1	2635.528639	-0.751434176	0.133567	-5.625893	1.85E-08	1.01E-07
13031	CYP7A1	0.746119629	-0.751621613	0.459828	-1.634571	0.102139	0.153636
11322	ACTN3	1.127939203	-0.751904085	0.34603	-2.172945	0.029784	0.051564
8611	ZFPM2	103.8000968	-0.752252039	0.237291	-3.170168	0.001524	0.003468
4583	TMEM120A	1102.845015	-0.752696211	0.150717	-4.994109	5.91E-07	2.53E-06
12061	SLFN14	1.4932407	-0.752762458	0.387812	-1.941049	0.052252	0.084918
5207	RAB24	899.1566498	-0.754023567	0.161305	-4.67451	2.95E-06	1.11E-05
10803	HSPB9	1.967345025	-0.754029412	0.319437	-2.360495	0.018251	0.033114
14264	LOC340074	0.433059737	-0.754185422	0.608452	-1.239515	0.215155	0.295657
8911	NINJ2	54.42320877	-0.754885863	0.247694	-3.047658	0.002306	0.005073
14885	GOLGA6L6	0.397398355	-0.754943687	0.725381	-1.040755	0.297989	0.392401
4270	RPS7	7173.660755	-0.755416988	0.146071	-5.17156	2.32E-07	1.07E-06
4753	FAM50A	1419.758925	-0.755554404	0.153964	-4.907346	9.23E-07	3.81E-06
7699	RHOD	235.3595373	-0.755586733	0.213429	-3.540225	0.0004	0.001018
8424	EMB	608.3042841	-0.75573047	0.233139	-3.241545	0.001189	0.002766
5502	UGDH	1342.684012	-0.755844866	0.167178	-4.521196	6.15E-06	2.19E-05
4697	C10orf54	1606.499125	-0.755878043	0.153273	-4.93158	8.16E-07	3.40E-06
5427	YPEL3	1035.02584	-0.755912172	0.165979	-4.554265	5.26E-06	1.90E-05
2901	LIG1	505.2091868	-0.755951115	0.123694	-6.11147	9.87E-10	6.67E-09
6831	ST20	80.55468532	-0.756377517	0.194555	-3.887725	0.000101	0.00029
3439	TRAPPC1	1485.19601	-0.75658158	0.132349	-5.71656	1.09E-08	6.20E-08
5585	IL16	550.5700202	-0.756914916	0.168915	-4.481048	7.43E-06	2.61E-05
4524	NOC4L	317.2829173	-0.756935722	0.150721	-5.022113	5.11E-07	2.21E-06
7580	C14orf139	78.09880795	-0.757542148	0.211271	-3.585645	0.000336	0.000869
8663	NDN	533.4065339	-0.757606924	0.240792	-3.146315	0.001653	0.003741
2110	MRPL17	1322.709849	-0.757686444	0.110784	-6.83932	7.96E-12	7.39E-11
4521	ZMYND17	97.98396165	-0.757708171	0.150823	-5.023814	5.07E-07	2.20E-06
9159	B3GALT2	12.50049309	-0.757816418	0.256381	-2.955816	0.003118	0.006674

11304	FAM7A2	2.794616441	-0.757953469	0.348096	-2.177424	0.029449	0.051064
3916	B3GALT6	489.2934973	-0.758013768	0.140553	-5.393091	6.93E-08	3.47E-07
3773	DUS1L	1175.901902	-0.758154429	0.138088	-5.490377	4.01E-08	2.08E-07
10261	EEF1DP3	2.005097307	-0.758507849	0.296054	-2.56206	0.010405	0.019877
5879	INO80B	365.7029158	-0.759073407	0.175152	-4.333808	1.47E-05	4.89E-05
7606	CAV1	6592.48138	-0.759532759	0.212274	-3.57807	0.000346	0.000892
1858	TMEM39B	262.5975833	-0.759549855	0.106564	-7.127668	1.02E-12	1.08E-11
8235	HLA-DQB1	4683.778708	-0.75955745	0.22896	-3.317428	0.000909	0.002162
13844	RIMS2	10.50798007	-0.759615207	0.554321	-1.370352	0.170577	0.241511
10412	FAM167A	62.0401995	-0.760000335	0.303244	-2.506235	0.012202	0.02297
12048	KIF25	3.539733568	-0.760198584	0.390862	-1.944929	0.051783	0.084247
4075	TRIP6	1646.514509	-0.760253545	0.14362	-5.293496	1.20E-07	5.77E-07
8656	CRISPLD2	1542.874055	-0.76029625	0.241507	-3.148131	0.001643	0.003721
11313	GYS2	5.620691447	-0.760337625	0.349591	-2.174937	0.029635	0.051346
13865	C8orf34	13.52381461	-0.760339391	0.557961	-1.362711	0.172974	0.244533
8703	GPR114	38.61427889	-0.760348906	0.243079	-3.127989	0.00176	0.003964
7267	ADAMTS7	244.3661308	-0.760626037	0.204649	-3.716736	0.000202	0.000544
3354	ATP5G2	6434.964136	-0.760804782	0.131695	-5.777028	7.60E-09	4.44E-08
7680	TMEM191A	20.30532824	-0.760879796	0.214493	-3.547339	0.000389	0.000993
11547	EMR3	6.166575217	-0.761127469	0.362616	-2.098989	0.035818	0.060801
8907	OR51E2	59.56731135	-0.761272552	0.249705	-3.048691	0.002298	0.005058
2126	CSRP1	3698.391892	-0.761516111	0.111588	-6.824377	8.83E-12	8.14E-11
657	ACTB	94511.60027	-0.761673172	0.083196	-9.155213	5.43E-20	1.62E-18
6475	ISYNA1	945.1153028	-0.76221639	0.188768	-4.037841	5.39E-05	0.000163
7808	PLEKHF1	170.0288071	-0.762237257	0.21787	-3.498593	0.000468	0.001174
5343	CHCHD6	126.5135016	-0.762503899	0.165843	-4.597745	4.27E-06	1.57E-05
1724	MGAT1	4896.800572	-0.762752817	0.10489	-7.271923	3.54E-13	4.03E-12
9666	IL1B	136.9282871	-0.76275795	0.276461	-2.759004	0.005798	0.011757
13390	PCDH11X	4.411699176	-0.763117221	0.504071	-1.513909	0.130049	0.190373
4316	RAB31	2081.931843	-0.76334762	0.148301	-5.147289	2.64E-07	1.20E-06
2372	FAM189B	642.2405551	-0.763428468	0.115907	-6.586561	4.50E-11	3.72E-10
8384	CHST15	2202.049721	-0.763920673	0.234495	-3.257723	0.001123	0.002626
6031	GRIN3A	27.44066494	-0.764443828	0.17979	-4.251868	2.12E-05	6.89E-05
6263	UBASH3B	226.510743	-0.764940537	0.184316	-4.150163	3.32E-05	0.000104
11680	WDR38	1.259964201	-0.765020588	0.372119	-2.055852	0.039797	0.066786
10586	SLC22A3	265.2277184	-0.765371797	0.313668	-2.440072	0.014684	0.027189
10895	SYT1	18.80395604	-0.765379728	0.329611	-2.322069	0.020229	0.036394
11269	AMAC1L2	1.361882501	-0.765445894	0.349112	-2.192553	0.02834	0.049293
3429	BCL9L	1745.244772	-0.765526607	0.133769	-5.722766	1.05E-08	5.99E-08
5949	CTSB	31266.87002	-0.765571737	0.178413	-4.291011	1.78E-05	5.86E-05
11043	C6orf105	51.22447529	-0.766381104	0.336789	-2.275552	0.022873	0.040599
5075	TANC2	1059.298408	-0.766672784	0.161953	-4.733916	2.20E-06	8.51E-06
6372	TBC1D16	667.0176794	-0.766826545	0.187381	-4.092339	4.27E-05	0.000131
6021	TSPAN4	1798.287027	-0.766942108	0.18023	-4.255343	2.09E-05	6.80E-05
8893	HPSE	90.89832174	-0.767013752	0.251077	-3.054893	0.002251	0.004962
10300	FOS	7945.04583	-0.767067231	0.301335	-2.545563	0.01091	0.020762
5346	NT5C	546.7278186	-0.767181022	0.166948	-4.59533	4.32E-06	1.58E-05

4309	PNKD	1526.016835	-0.767263527	0.148925	-5.152011	2.58E-07	1.17E-06
2284	CDCA4	162.3502239	-0.767415426	0.115092	-6.667854	2.60E-11	2.23E-10
8534	COL15A1	2723.197789	-0.767519254	0.240303	-3.193959	0.001403	0.003223
6081	RASSF2	990.0915585	-0.767974496	0.181604	-4.228849	2.35E-05	7.57E-05
5041	KCNJ14	31.07322326	-0.768281107	0.161647	-4.752839	2.01E-06	7.80E-06
3992	PCNXL2	297.1557235	-0.768356527	0.143761	-5.344675	9.06E-08	4.45E-07
4802	TMEM134	607.8528951	-0.768599047	0.157307	-4.88599	1.03E-06	4.20E-06
1951	FAM100B	519.8311779	-0.768690842	0.109269	-7.034823	2.00E-12	2.00E-11
9092	GPR1	33.36535456	-0.768755731	0.25787	-2.981177	0.002871	0.00619
8028	SLC45A1	77.1408588	-0.769025205	0.226182	-3.40003	0.000674	0.001645
4558	LYN	989.7579791	-0.76907796	0.153663	-5.004957	5.59E-07	2.40E-06
5465	PORCN	156.7734462	-0.769213495	0.169592	-4.535662	5.74E-06	2.06E-05
6217	LAMP3	146.6170313	-0.769306912	0.184478	-4.170193	3.04E-05	9.60E-05
10107	KLHDC7B	64.30813298	-0.769316441	0.29423	-2.61468	0.008931	0.017321
3384	C8orf30A	480.4215434	-0.769375221	0.133627	-5.757637	8.53E-09	4.94E-08
12263	MPO	6.628731378	-0.769410083	0.409575	-1.878559	0.060305	0.096386
4608	C7orf59	1312.925781	-0.76981864	0.154416	-4.985343	6.19E-07	2.63E-06
2927	DDAH2	1105.685025	-0.770074286	0.126395	-6.092583	1.11E-09	7.44E-09
3235	SYNGR2	3492.860602	-0.770557588	0.131426	-5.863039	4.54E-09	2.75E-08
14690	MMP20	1.003821819	-0.770665201	0.697895	-1.10427	0.269476	0.359564
6763	PLK3	326.9972889	-0.770694963	0.196968	-3.912792	9.12E-05	0.000264
2582	ETHE1	391.9472773	-0.771140126	0.12054	-6.397396	1.58E-10	1.20E-09
6822	CRNA0018	54.08188788	-0.771738115	0.198377	-3.890252	0.0001	0.000288
4049	FHOD1	726.2452647	-0.771839655	0.145433	-5.307202	1.11E-07	5.39E-07
9751	LOC84856	259.5701577	-0.772108882	0.282579	-2.732369	0.006288	0.01264
3689	SAMD1	185.1741717	-0.772118519	0.139378	-5.539726	3.03E-08	1.61E-07
5018	GLTSCR2	6504.309127	-0.772121386	0.162103	-4.763157	1.91E-06	7.44E-06
10407	GPR174	22.66570629	-0.772175287	0.308004	-2.507026	0.012175	0.022929
7311	PAPSS2	1159.709175	-0.772257226	0.208932	-3.696217	0.000219	0.000587
2541	POLE	480.647575	-0.772394924	0.120133	-6.429511	1.28E-10	9.87E-10
8458	ABCA7	289.1521102	-0.773000385	0.239683	-3.225091	0.001259	0.002918
6413	HLA-DRB1	9477.090906	-0.773384434	0.189974	-4.070997	4.68E-05	0.000143
3550	SIX5	340.4493718	-0.773524538	0.137243	-5.636172	1.74E-08	9.60E-08
4835	RPS10	5634.591656	-0.773665424	0.158766	-4.872995	1.10E-06	4.46E-06
5995	BIN2	371.2591255	-0.773759533	0.181206	-4.270059	1.95E-05	6.39E-05
3691	SIRT7	266.7976169	-0.773933648	0.139716	-5.539315	3.04E-08	1.61E-07
8508	CPNE5	178.058395	-0.774062648	0.241711	-3.202432	0.001363	0.003139
9181	FOLH1	759.395987	-0.774259482	0.262788	-2.946327	0.003216	0.006865
3562	RUVBL2	1573.332701	-0.774319541	0.137598	-5.627388	1.83E-08	1.01E-07
12791	ZACN	0.601945992	-0.77435597	0.453618	-1.707065	0.08781	0.134561
3342	RPL41	17157.66896	-0.77476551	0.133775	-5.791537	6.97E-09	4.09E-08
6240	MARCKS	4517.034976	-0.775151194	0.186387	-4.158823	3.20E-05	0.0001
10248	SLC5A9	456.7433275	-0.775613155	0.302287	-2.565817	0.010293	0.019688
4902	GSDMD	1753.309461	-0.776121696	0.160839	-4.825449	1.40E-06	5.59E-06
2440	CSK	1213.185002	-0.77621118	0.11906	-6.519481	7.06E-11	5.67E-10
4953	RHBDD3	249.0136174	-0.776564796	0.161959	-4.79481	1.63E-06	6.44E-06
6691	C16orf75	115.5563089	-0.776798342	0.196956	-3.944019	8.01E-05	0.000235

3330	NCKAP5L	559.5206995	-0.777047293	0.134062	-5.796173	6.78E-09	3.99E-08
1220	CFL1	12620.2213	-0.777112735	0.09719	-7.995775	1.29E-15	2.07E-14
9885	MGC16703	4.430868063	-0.777136302	0.288667	-2.692157	0.007099	0.014077
5977	CASP1	565.231944	-0.777376647	0.181672	-4.279001	1.88E-05	6.16E-05
3587	ZMYND8	1013.737582	-0.777443218	0.138519	-5.612558	1.99E-08	1.09E-07
6455	TIMP2	6893.477401	-0.777602683	0.192121	-4.04747	5.18E-05	0.000157
7323	SYTL4	257.5701562	-0.77771084	0.210725	-3.69064	0.000224	0.000599
7886	PRKCQ	137.0328687	-0.778905458	0.22545	-3.454895	0.000551	0.001368
1329	CARM1	901.4897847	-0.778912405	0.099513	-7.827227	4.99E-15	7.36E-14
4699	PTPRF	5976.212724	-0.779083843	0.157983	-4.931431	8.16E-07	3.41E-06
1340	GNA12	1483.336618	-0.779342624	0.099799	-7.809153	5.76E-15	8.42E-14
6529	ASNS	649.4939577	-0.779441953	0.19423	-4.012987	6.00E-05	0.00018
13061	CPB1	6.637418418	-0.779521512	0.479581	-1.625422	0.104073	0.156185
10590	PCDHGA4	59.68000086	-0.780230147	0.319853	-2.439338	0.014714	0.027235
9387	PCDHB2	132.2653962	-0.780917351	0.272601	-2.864686	0.004174	0.008716
10247	SNORD17	8.280756304	-0.781103979	0.304402	-2.566028	0.010287	0.019678
8189	C17orf82	16.84696325	-0.781107121	0.233988	-3.338233	0.000843	0.002018
6415	NOTCH3	5745.105471	-0.781226217	0.19194	-4.070166	4.70E-05	0.000144
7617	FBXO24	5.906678058	-0.781311687	0.218571	-3.574637	0.000351	0.000903
11246	C14orf72	7.815769386	-0.78150796	0.354964	-2.201652	0.02769	0.048262
12423	PRSS37	0.793260742	-0.781547325	0.42722	-1.829378	0.067343	0.106254
9830	SGEF	59.65973035	-0.781574866	0.288468	-2.709401	0.00674	0.013441
1449	RNF166	332.7362534	-0.781736107	0.102384	-7.635341	2.25E-14	3.05E-13
13987	DCST1	0.417575995	-0.781766447	0.590853	-1.323115	0.185797	0.260371
6664	ANTXR1	2332.342481	-0.781775759	0.197593	-3.956491	7.61E-05	0.000224
8686	AGBL2	36.34074901	-0.781866245	0.249301	-3.136236	0.001711	0.003862
1341	PML	2479.23084	-0.782101962	0.100151	-7.809193	5.76E-15	8.42E-14
10000	CORT	8.451167221	-0.78220553	0.294889	-2.652539	0.007989	0.015659
6125	SEMA4A	363.5857398	-0.782750264	0.185934	-4.209827	2.56E-05	8.18E-05
3185	PANX1	547.5287909	-0.782774494	0.13287	-5.891302	3.83E-09	2.36E-08
3077	ITGB5	3560.429701	-0.782778436	0.13095	-5.977686	2.26E-09	1.44E-08
6458	IL3RA	359.2653403	-0.782975645	0.19354	-4.045546	5.22E-05	0.000158
5509	TCEB2	2033.259207	-0.782997524	0.173448	-4.514301	6.35E-06	2.26E-05
3179	PSME2	2262.105993	-0.783008776	0.132833	-5.894704	3.75E-09	2.31E-08
5828	PMP22	1862.329046	-0.783145092	0.179701	-4.358036	1.31E-05	4.41E-05
10905	GZMK	450.353741	-0.783160505	0.33768	-2.319238	0.020382	0.036632
13709	CNTN5	0.762493411	-0.783421865	0.553531	-1.415318	0.156975	0.224442
4126	CHEK1	162.4391801	-0.783536208	0.148794	-5.265919	1.39E-07	6.63E-07
1547	PRDX1	6354.617238	-0.783644357	0.104543	-7.495922	6.58E-14	8.34E-13
9314	HTR7	24.39908392	-0.783729001	0.270729	-2.894879	0.003793	0.007982
2622	DTD1	845.6373132	-0.783928909	0.123349	-6.355356	2.08E-10	1.55E-09
8969	CABP7	5.782840512	-0.78398376	0.25927	-3.023808	0.002496	0.005455
7404	RPL13AP3	12.30663422	-0.784148751	0.21449	-3.655878	0.000256	0.000679
2447	PLD3	4669.969637	-0.784349636	0.120415	-6.513712	7.33E-11	5.87E-10
6535	PDGFRB	5109.251043	-0.78449902	0.19562	-4.010314	6.06E-05	0.000182
4844	HLA-DMA	2561.025244	-0.784577577	0.16113	-4.869219	1.12E-06	4.53E-06
6469	C16orf48	468.5955549	-0.785071955	0.194244	-4.041676	5.31E-05	0.000161

6014	GINS1	136.1763056	-0.785158174	0.184418	-4.257488	2.07E-05	6.74E-05
10383	TAS2R19	2.065522965	-0.785514874	0.312692	-2.5121	0.012002	0.022656
9617	MANEAL	204.8346361	-0.785591123	0.283339	-2.772621	0.005561	0.011334
6666	CIITA	451.4229035	-0.785682099	0.198625	-3.955601	7.63E-05	0.000224
2052	ARHGAP1	3867.101954	-0.785739478	0.113864	-6.900692	5.17E-12	4.94E-11
1577	DDOST	5960.061385	-0.786196268	0.105445	-7.455994	8.92E-14	1.11E-12
14108	DUOXA2	0.637907471	-0.78672934	0.612255	-1.284971	0.198803	0.276207
4173	MAP2K2	1927.311724	-0.787125812	0.150363	-5.234853	1.65E-07	7.76E-07
6992	PIWIL4	62.63961642	-0.787125848	0.205605	-3.828339	0.000129	0.000362
8618	ITK	174.7044939	-0.787242605	0.248466	-3.168415	0.001533	0.003486
8477	RGS1	2802.037958	-0.787365229	0.244763	-3.216845	0.001296	0.002997
11646	MSH4	4.63124322	-0.787367713	0.381802	-2.062243	0.039185	0.06595
5586	PRR5L	302.4498348	-0.787375987	0.175734	-4.480498	7.45E-06	2.61E-05
5516	C8orf51	24.4162566	-0.787551174	0.174494	-4.513337	6.38E-06	2.27E-05
1323	SND1	4782.215983	-0.787593281	0.10056	-7.83209	4.80E-15	7.11E-14
14127	OR1J2	1.013203026	-0.787664547	0.615592	-1.279524	0.200713	0.278472
4811	RPS5	11463.60604	-0.788865563	0.161541	-4.883365	1.04E-06	4.25E-06
8651	VENTX	35.2304549	-0.788966353	0.250398	-3.150852	0.001628	0.003689
4824	C12orf57	1473.252502	-0.789057769	0.16174	-4.878557	1.07E-06	4.34E-06
11079	ANGPTL4	19583.07356	-0.789381337	0.348782	-2.263254	0.02362	0.041789
6465	RAB37	278.4729229	-0.789610096	0.19527	-4.043681	5.26E-05	0.00016
1444	ERGIC3	4333.900023	-0.789880562	0.103395	-7.639455	2.18E-14	2.96E-13
7978	DC1003026	7.530641602	-0.790699362	0.231261	-3.419081	0.000628	0.001544
4579	ZNF524	262.8219686	-0.790812729	0.158257	-4.997004	5.82E-07	2.49E-06
14827	STMN4	0.336343332	-0.790828613	0.747809	-1.057527	0.290271	0.383754
9065	RTN4R	99.49206915	-0.791136506	0.264362	-2.992624	0.002766	0.005981
4242	ABI3	476.0516698	-0.791170318	0.152567	-5.185717	2.15E-07	9.94E-07
13132	KIRREL2	2.684748493	-0.79125429	0.495077	-1.598244	0.109989	0.164171
10501	RASGEF1A	170.7983046	-0.791690363	0.320362	-2.471235	0.013465	0.025133
2281	COMT	1696.92513	-0.791805321	0.118709	-6.670122	2.56E-11	2.20E-10
8654	C9orf172	15.03958708	-0.792789166	0.251732	-3.149342	0.001636	0.003706
1454	GNAI2	6815.525914	-0.793281877	0.103941	-7.63202	2.31E-14	3.12E-13
2424	PMM2	1086.630675	-0.793846793	0.121418	-6.538131	6.23E-11	5.04E-10
7503	NACA2	57.07857215	-0.794659998	0.219736	-3.616429	0.000299	0.00078
9086	SLC17A7	7.53298448	-0.79479272	0.266375	-2.98374	0.002847	0.006143
9316	CHADL	31.43892603	-0.794892636	0.274626	-2.89446	0.003798	0.007991
14355	ITIH1	9.970444107	-0.795032166	0.655722	-1.212454	0.225339	0.307667
2040	TRIM65	428.1967803	-0.795042394	0.114884	-6.920377	4.50E-12	4.33E-11
6291	HOPX	220.1289007	-0.795303303	0.192416	-4.133253	3.58E-05	0.000111
11634	EN1	9.971792158	-0.795346937	0.384788	-2.066972	0.038737	0.065264
6170	MRPS12	667.0153678	-0.795916698	0.189947	-4.190212	2.79E-05	8.85E-05
2600	PPP2R4	4372.612932	-0.795925168	0.124739	-6.38073	1.76E-10	1.33E-09
8638	CACNA1A	22.02027498	-0.796270376	0.252236	-3.156849	0.001595	0.003619
3969	PTMS	5144.847756	-0.796341706	0.148554	-5.360638	8.29E-08	4.10E-07
10039	APOD	188.8792109	-0.796418021	0.301888	-2.638128	0.008337	0.016277
8200	PROS1	4479.956942	-0.796564586	0.239056	-3.332124	0.000862	0.00206
10116	CTSL2	29.16162536	-0.796801157	0.305327	-2.609661	0.009063	0.017559

14873	AVP	0.516317313	-0.79685527	0.762916	-1.044487	0.29626	0.390439
5190	TTYH2	224.9866405	-0.797187523	0.170316	-4.680633	2.86E-06	1.08E-05
5780	TIAF1	402.0824593	-0.797706317	0.18203	-4.382272	1.17E-05	3.98E-05
15038	SLC1A6	0.802718694	-0.797873286	0.801675	-0.995257	0.319611	0.416591
8720	ROPN1L	18.70792086	-0.797875656	0.255631	-3.121202	0.001801	0.004049
9350	MST1	568.5527806	-0.797898163	0.27715	-2.878943	0.00399	0.008365
3045	UBA7	925.7442147	-0.798151479	0.132934	-6.004141	1.92E-09	1.24E-08
2632	FAM129B	3715.520978	-0.798278782	0.125842	-6.343502	2.25E-10	1.67E-09
4892	PLXND1	4965.656662	-0.798503938	0.165281	-4.831197	1.36E-06	5.44E-06
11990	TPI1P3	0.744940793	-0.798728451	0.406848	-1.963211	0.049622	0.081121
3296	NHP2	1348.187607	-0.798768587	0.137273	-5.818847	5.93E-09	3.52E-08
11745	KLK14	1.263340938	-0.799442163	0.392252	-2.038081	0.041542	0.069328
2196	POLR2H	906.8807183	-0.799504878	0.118267	-6.760149	1.38E-11	1.23E-10
2596	BANF1	1916.431613	-0.799883604	0.12528	-6.384777	1.72E-10	1.30E-09
2959	RP9	165.0901684	-0.800123826	0.131954	-6.063646	1.33E-09	8.82E-09
16685	SNORA20	0.294060519	-0.800861	1.532981	-0.522421	0.601377	0.706479
17221	CGB5	0.181358392	-0.800861105	2.087602	-0.383627	0.701255	0.79817
16393	APOA5	0.226306597	-0.80086113	1.329438	-0.602406	0.546904	0.653906
16842	FAM90A7	0.229312276	-0.800861182	1.669379	-0.479736	0.631415	0.734801
15408	LGALS9B	0.26050821	-0.800861185	0.902697	-0.887187	0.374978	0.477022
15610	COX8C	0.249278642	-0.800861194	0.961896	-0.832586	0.405078	0.508645
16209	CALHM3	0.188752581	-0.800861245	1.226305	-0.653069	0.513712	0.621215
16882	KRT33A	0.162972984	-0.80086126	1.707204	-0.469107	0.638993	0.741909
15859	)C1001280	0.160775493	-0.800861297	1.059489	-0.755894	0.449713	0.555825
14776	MUSK	0.28551802	-0.800909171	0.744718	-1.075453	0.282172	0.374313
3971	NAA10	837.985083	-0.801019058	0.149442	-5.360078	8.32E-08	4.11E-07
4538	DAPK3	816.4472003	-0.801039066	0.15971	-5.015593	5.29E-07	2.28E-06
13078	TMEM105	2.663117933	-0.801352057	0.494489	-1.620568	0.10511	0.157537
15591	ALPP	0.348046827	-0.801418936	0.95729	-0.837175	0.402494	0.506016
3715	ZNF787	490.3995565	-0.80152019	0.144979	-5.528518	3.23E-08	1.70E-07
1779	TNKS1BP1	2876.604234	-0.801689136	0.111237	-7.207052	5.72E-13	6.30E-12
8555	PTPN22	119.3290595	-0.801853272	0.251537	-3.187817	0.001434	0.003284
10317	SPON2	2946.47494	-0.802052182	0.315636	-2.541069	0.011051	0.020996
3199	KPNA2	926.140599	-0.802151698	0.136336	-5.883634	4.01E-09	2.46E-08
12436	SNORD10	1.575415139	-0.802314377	0.439452	-1.825716	0.067893	0.10701
12712	XPNPEP2	107.6688023	-0.802499276	0.464236	-1.728643	0.083873	0.129334
7770	JUNB	4338.284887	-0.802776641	0.228745	-3.50949	0.000449	0.001133
5183	POLR2I	564.0788098	-0.802939068	0.171411	-4.684283	2.81E-06	1.06E-05
5165	GUK1	3825.136736	-0.802997619	0.17111	-4.692884	2.69E-06	1.02E-05
15894	GUCY2E	0.642656839	-0.803076262	1.078262	-0.744788	0.4564	0.562872
3119	GM2A	2993.39425	-0.803167047	0.134988	-5.949922	2.68E-09	1.69E-08
10684	PPIAL4D	1.2777606	-0.803177119	0.334416	-2.401728	0.016318	0.029937
9996	PCDHGB8I	3.907468257	-0.803212213	0.302656	-2.653876	0.007957	0.015603
11908	IL23R	1.434791477	-0.803721474	0.40485	-1.985232	0.047119	0.077556
7389	EVI2B	717.2856654	-0.803825081	0.219558	-3.661111	0.000251	0.000666
1955	ARRB2	1213.649593	-0.803910621	0.114333	-7.031337	2.05E-12	2.05E-11
6817	SAMHD1	1128.188374	-0.803942036	0.206537	-3.892477	9.92E-05	0.000285

6428	PRRT1	35.00034313	-0.804030161	0.197988	-4.060999	4.89E-05	0.000149
3815	CSDAP1	72.27842867	-0.804111201	0.147171	-5.463793	4.66E-08	2.39E-07
12155	PP14571	1.632822618	-0.804150776	0.421253	-1.908948	0.056269	0.090738
8175	FAM173A	172.1156886	-0.804219139	0.240538	-3.343424	0.000828	0.001984
4693	MARCKSL1	1603.124413	-0.804323039	0.163089	-4.931812	8.15E-07	3.40E-06
7455	TNFAIP2	2122.182481	-0.80440917	0.221315	-3.634682	0.000278	0.000732
9618	ISM1	54.6112505	-0.804419623	0.29015	-2.772427	0.005564	0.011338
5521	CHCHD5	325.7544004	-0.80463426	0.178355	-4.511411	6.44E-06	2.29E-05
2866	NXT1	305.2099189	-0.804674165	0.131243	-6.1312	8.72E-10	5.97E-09
1840	CHST14	773.0635726	-0.804729168	0.112571	-7.148621	8.77E-13	9.34E-12
3809	IFI35	812.9319159	-0.805216671	0.147281	-5.467201	4.57E-08	2.35E-07
4804	AMIGO3	72.27774337	-0.805275493	0.164844	-4.885076	1.03E-06	4.22E-06
9922	MGST1	3985.927213	-0.805406468	0.300598	-2.679343	0.007377	0.014573
4868	GUSBP3	55.96117498	-0.805508392	0.165975	-4.853187	1.21E-06	4.89E-06
1914	EIF5A	5672.103836	-0.805956797	0.11394	-7.073492	1.51E-12	1.55E-11
8244	ABLIM3	2841.716846	-0.805983638	0.243189	-3.314229	0.000919	0.002185
8393	C5orf49	104.0521554	-0.806047338	0.24796	-3.250715	0.001151	0.002688
3032	RPL23	11632.33802	-0.806216941	0.134148	-6.009906	1.86E-09	1.20E-08
3043	ZNF408	269.2245441	-0.806381975	0.134296	-6.004525	1.92E-09	1.24E-08
3666	ANKRD19	50.81714638	-0.806663238	0.145116	-5.558734	2.72E-08	1.45E-07
13631	HEPN1	1.020388849	-0.80674971	0.558644	-1.44412	0.148705	0.213834
3338	RPL14	6841.228426	-0.807381392	0.139361	-5.793448	6.90E-09	4.05E-08
9862	KIF19	14.08843079	-0.807464203	0.299146	-2.699234	0.00695	0.013813
11379	CXCL2	259.8136706	-0.808085885	0.374351	-2.158632	0.030879	0.05319
13548	MMEL1	3.492240003	-0.808229162	0.549179	-1.471705	0.141101	0.204142
7398	ALDH3B1	1140.705219	-0.808437716	0.221031	-3.657572	0.000255	0.000675
12549	PDC	0.613586624	-0.808921712	0.451752	-1.790633	0.073352	0.114564
5642	MAP1A	437.3157231	-0.80905279	0.181735	-4.45182	8.51E-06	2.96E-05
2532	BCKDK	2420.538333	-0.809192594	0.125702	-6.437373	1.22E-10	9.41E-10
4261	HHLA3	148.1726206	-0.809201839	0.156327	-5.176344	2.26E-07	1.04E-06
11824	MGC45800	41.50760243	-0.809337	0.402092	-2.012818	0.044134	0.073159
4440	ZNF688	312.3383345	-0.809396557	0.159361	-5.078997	3.79E-07	1.68E-06
3203	PFDN2	695.9842392	-0.809530947	0.137626	-5.882117	4.05E-09	2.48E-08
4192	PDLIM1	4160.648743	-0.809664848	0.155286	-5.214015	1.85E-07	8.64E-07
9498	ETV4	11.42094452	-0.809850796	0.287136	-2.820443	0.004796	0.009897
7088	IMPG2	3.622593284	-0.809986815	0.213756	-3.78931	0.000151	0.000418
7268	ZNF692	405.5329312	-0.810111931	0.217975	-3.716542	0.000202	0.000545
846	SLC24A6	715.4674291	-0.810219175	0.093745	-8.642817	5.48E-18	1.27E-16
9049	EPHA3	421.6576815	-0.810239231	0.270403	-2.996409	0.002732	0.005917
9176	C7orf54	2.323114965	-0.810331443	0.274969	-2.946997	0.003209	0.006853
10439	RPS26P11	1.150451271	-0.810699783	0.324521	-2.498139	0.012485	0.023444
7775	NPDC1	1033.567739	-0.811160985	0.231179	-3.5088	0.00045	0.001135
2173	NRSN2	1191.899113	-0.811221814	0.119498	-6.788562	1.13E-11	1.02E-10
10736	CYP4F11	166.0365873	-0.811920248	0.340397	-2.385214	0.017069	0.031161
11988	GRK7	0.689437478	-0.811938936	0.413519	-1.963485	0.04959	0.081082
12726	SNORA67	0.712234965	-0.812297904	0.471225	-1.723799	0.084744	0.130526
1631	MCM6	722.1532043	-0.812537537	0.110028	-7.384844	1.53E-13	1.83E-12

5397	ASL	1161.000769	-0.812703983	0.177966	-4.566627	4.96E-06	1.80E-05
8495	GLI2	85.44362739	-0.812888668	0.253386	-3.208108	0.001336	0.003083
2467	LSM2	510.8647769	-0.813083826	0.125111	-6.498901	8.09E-11	6.43E-10
11348	C10orf62	4.093934498	-0.813094627	0.375411	-2.16588	0.03032	0.052363
9148	HES4	126.5163849	-0.81330158	0.274852	-2.959051	0.003086	0.006612
1153	SP110	547.7627554	-0.813411225	0.100598	-8.085748	6.18E-16	1.05E-14
2700	RPS12	10089.7518	-0.813876297	0.129538	-6.282925	3.32E-10	2.41E-09
11530	ARMS2	1.087518878	-0.813920717	0.3862	-2.107513	0.035073	0.059624
9939	WNT3	22.34848676	-0.81403966	0.304442	-2.673871	0.007498	0.014787
8118	PTPRO	192.7029943	-0.814406972	0.242155	-3.363167	0.000771	0.00186
3576	TRANK1	623.4130939	-0.814624729	0.144934	-5.620677	1.90E-08	1.04E-07
3676	ALKBH2	278.4426645	-0.815231999	0.146798	-5.553445	2.80E-08	1.49E-07
8645	KIAA0748	53.67308488	-0.815509125	0.258641	-3.15305	0.001616	0.003663
5433	FLNA	19509.68907	-0.81569108	0.179209	-4.551627	5.32E-06	1.92E-05
1291	DBNL	2320.579613	-0.815863899	0.103606	-7.874673	3.42E-15	5.19E-14
7510	MSL3L2	83.54836217	-0.816197965	0.225917	-3.612823	0.000303	0.000791
9715	GBP6	5.232265437	-0.816459325	0.297618	-2.743314	0.006082	0.012272
15164	HGC6.3	1.615536154	-0.816499239	0.853014	-0.957194	0.33847	0.437477
2222	BAX	818.4241681	-0.816806246	0.121351	-6.730957	1.69E-11	1.49E-10
11518	CAMK2A	4.749670072	-0.817086638	0.387121	-2.110673	0.0348	0.059222
10923	RHOXF1	3.742718761	-0.817088057	0.353532	-2.311214	0.020821	0.037363
7823	SYTL2	1890.638752	-0.817147925	0.233949	-3.492842	0.000478	0.001197
9156	KCNB1	19.63358635	-0.81729133	0.276458	-2.956296	0.003114	0.006665
3789	HTATIP2	1331.056361	-0.817517506	0.149187	-5.47981	4.26E-08	2.20E-07
1043	DGUOK	736.0850696	-0.817654059	0.099104	-8.250505	1.58E-16	2.96E-15
8679	HS3ST3B1	12.71522697	-0.817760953	0.260454	-3.139757	0.001691	0.003819
4046	FADS1	912.3045799	-0.817827865	0.15408	-5.30781	1.11E-07	5.37E-07
7387	DGCR6	600.5974139	-0.818071509	0.223439	-3.661277	0.000251	0.000666
8140	KLRA1	40.99211051	-0.818254696	0.243775	-3.356604	0.000789	0.0019
6987	CECR1	2107.008008	-0.818679683	0.213707	-3.830858	0.000128	0.000358
1845	SFXN3	1706.734906	-0.818827307	0.114595	-7.145422	8.97E-13	9.53E-12
3091	TOMM7	2524.060065	-0.820300435	0.13742	-5.969277	2.38E-09	1.51E-08
4512	THAP3	308.6345367	-0.820783421	0.163215	-5.028835	4.93E-07	2.14E-06
3779	CENPO	82.46823816	-0.8211274	0.149677	-5.485981	4.11E-08	2.13E-07
11198	GFRA1	614.5297415	-0.82118638	0.369623	-2.221684	0.026305	0.046044
8268	CCBP2	26.28097525	-0.821245748	0.24849	-3.304945	0.00095	0.002252
4171	FAU	7822.154199	-0.821359467	0.156878	-5.235652	1.64E-07	7.73E-07
3293	CNPY4	387.2918682	-0.822668798	0.141355	-5.81989	5.89E-09	3.51E-08
6779	UBXN11	622.3858808	-0.822764004	0.210551	-3.907673	9.32E-05	0.000269
1704	ALDH18A1	2172.982563	-0.823032846	0.112856	-7.292756	3.04E-13	3.49E-12
3163	ORAI3	1272.241361	-0.823597321	0.139372	-5.909339	3.43E-09	2.13E-08
2070	RPL23AP7	95.1363471	-0.823623857	0.119713	-6.879999	5.99E-12	5.67E-11
5380	ZNF90	168.6017707	-0.823663704	0.17995	-4.577183	4.71E-06	1.72E-05
4453	RCN1	4087.920418	-0.823823599	0.162494	-5.069876	3.98E-07	1.75E-06
7238	RASSF7	918.309137	-0.823835401	0.2209	-3.729442	0.000192	0.00052
2211	ITPA	743.238417	-0.824035576	0.122166	-6.745208	1.53E-11	1.35E-10
13335	AQP10	0.48226933	-0.824332695	0.537977	-1.532282	0.125453	0.184402

1603	44083	6513.582395	-0.824437748	0.111139	-7.418058	1.19E-13	1.45E-12
9202	RND2	59.03740101	-0.824726111	0.280764	-2.937432	0.003309	0.007049
12050	MYO18B	1.939391341	-0.824827025	0.424294	-1.943998	0.051896	0.084416
8557	KLRK1	146.1519709	-0.825036337	0.258833	-3.187522	0.001435	0.003287
15294	KLK11	1.246149881	-0.825154917	0.894497	-0.922479	0.356279	0.456612
1689	TNFRSF1A	3609.911982	-0.825385487	0.11288	-7.312057	2.63E-13	3.05E-12
4639	MANF	1226.718782	-0.825874441	0.166193	-4.969355	6.72E-07	2.84E-06
9318	TFPI	5543.285591	-0.8262592	0.285574	-2.893325	0.003812	0.008018
2946	STAT5A	815.5113995	-0.826776277	0.136136	-6.073165	1.25E-09	8.34E-09
1125	TUBB	12582.79663	-0.826790253	0.101794	-8.12217	4.58E-16	7.98E-15
8003	REM1	41.77112155	-0.827664138	0.242783	-3.409073	0.000652	0.001596
1498	SMARCD2	1059.461148	-0.827747105	0.109325	-7.571403	3.69E-14	4.83E-13
5857	GPR161	83.85423802	-0.827940042	0.190669	-4.342298	1.41E-05	4.72E-05
8704	DIRC3	31.75082641	-0.82812414	0.26477	-3.127711	0.001762	0.003967
3723	GMPPA	881.4546113	-0.828367546	0.149965	-5.523725	3.32E-08	1.75E-07
6764	LDLRAD2	302.1037839	-0.828494718	0.211748	-3.912636	9.13E-05	0.000265
2171	MCM5	962.7967692	-0.829117911	0.122059	-6.792757	1.10E-11	9.93E-11
2125	RPL23A	4487.169471	-0.829621871	0.121559	-6.824869	8.80E-12	8.12E-11
7762	CLDN5	464.1854885	-0.829905322	0.236194	-3.513666	0.000442	0.001116
8950	H2BFXP	5.078393076	-0.829936477	0.27372	-3.032062	0.002429	0.005319
7190	RADIL	11.54703256	-0.829975168	0.221396	-3.748823	0.000178	0.000484
6084	GUCY1A2	82.8992104	-0.830013119	0.19635	-4.22721	2.37E-05	7.62E-05
8997	SCARNA12	4.916588675	-0.830376967	0.275562	-3.013396	0.002583	0.005628
11152	SLC8A3	5.161274126	-0.830758442	0.371374	-2.236986	0.025287	0.044445
7231	KCNMB4	124.597468	-0.830793079	0.222535	-3.733318	0.000189	0.000512
4921	MRPS34	1222.160252	-0.831039967	0.172551	-4.816206	1.46E-06	5.83E-06
8867	LBX2	17.51358972	-0.831199881	0.27127	-3.064106	0.002183	0.004826
9829	EOMES	93.92298735	-0.831339007	0.306829	-2.709455	0.006739	0.01344
8914	NTF3	29.87228147	-0.831400693	0.272935	-3.046148	0.002318	0.005097
8765	TMEM156	42.17606766	-0.831507591	0.268367	-3.098396	0.001946	0.004351
4944	AZI1	230.3565202	-0.831681556	0.17317	-4.802695	1.57E-06	6.21E-06
10307	AK5	11.69919945	-0.83214584	0.327285	-2.542571	0.011004	0.020924
2047	PLBD2	1014.738965	-0.832150819	0.120401	-6.911504	4.80E-12	4.59E-11
11885	C2orf70	5.037863632	-0.832170611	0.417443	-1.993496	0.046207	0.076206
14205	ART1	0.592642595	-0.832288184	0.663218	-1.254924	0.209506	0.289091
8735	PARP15	25.32955113	-0.832320085	0.267224	-3.114688	0.001841	0.004132
8381	PTCH2	21.38761522	-0.832492208	0.255528	-3.25793	0.001122	0.002624
10108	RP1	8.510087434	-0.832561718	0.318495	-2.614052	0.008948	0.017351
9455	XKR5	6.203184235	-0.832803913	0.293635	-2.836189	0.004566	0.009465
3875	SERF2	8584.126555	-0.8332567	0.153736	-5.420056	5.96E-08	3.01E-07
6228	GGTA1	487.3707441	-0.833272881	0.200077	-4.164763	3.12E-05	9.81E-05
13867	DPPA4	0.653346296	-0.834089229	0.61232	-1.362179	0.173141	0.244735
1339	MAP7D1	1622.891542	-0.834589571	0.106861	-7.81002	5.72E-15	8.38E-14
7091	SLC19A1	217.4849144	-0.834710195	0.220352	-3.788083	0.000152	0.00042
5135	TBC1D3	374.0842946	-0.835031466	0.177256	-4.71089	2.47E-06	9.41E-06
15054	SNORA23	0.462215436	-0.835032807	0.844486	-0.988806	0.322758	0.420246
12650	HIST1H2BI	2.725011835	-0.835103937	0.475276	-1.757091	0.078902	0.122258

4039	TUBA1A	4575.607167	-0.835738726	0.157309	-5.312708	1.08E-07	5.24E-07
12446	KIAA1644	127.9270425	-0.836101743	0.458991	-1.821607	0.068515	0.107903
10114	ANKRD22	180.2561851	-0.836354779	0.320437	-2.610044	0.009053	0.017545
12817	ASIP	6.372810844	-0.836399629	0.492045	-1.699845	0.08916	0.136342
9734	RAB39	21.56561298	-0.836843625	0.305682	-2.737624	0.006188	0.012462
7670	MTCP1	139.2602652	-0.836856415	0.235634	-3.551511	0.000383	0.000979
9321	SCARA3	342.7239716	-0.83729215	0.289501	-2.892189	0.003826	0.008045
7643	ADAM28	268.9630758	-0.837378178	0.234988	-3.563493	0.000366	0.000939
5969	KCNJ8	619.7992245	-0.837494205	0.195571	-4.282313	1.85E-05	6.07E-05
2220	RPL11	14548.33211	-0.837702854	0.124402	-6.733829	1.65E-11	1.46E-10
1772	C19orf66	756.1331969	-0.837736743	0.116053	-7.21856	5.25E-13	5.81E-12
6386	RNASE1	3213.98541	-0.837745683	0.205188	-4.082819	4.45E-05	0.000137
9874	HPCA	31.90078538	-0.83777581	0.310754	-2.695948	0.007019	0.013933
3613	S100A10	9453.760999	-0.837836137	0.149611	-5.600093	2.14E-08	1.16E-07
13190	FLJ43390	1.134320196	-0.838180079	0.529507	-1.582945	0.113434	0.168569
1551	STX4	1140.531821	-0.838323198	0.111899	-7.491761	6.80E-14	8.59E-13
13051	HIST1H3C	0.436681341	-0.838337982	0.514448	-1.629587	0.103189	0.154977
1521	C12orf45	154.9407097	-0.838390679	0.111314	-7.531743	5.01E-14	6.45E-13
3248	B4GALT1	5315.870381	-0.838892044	0.143311	-5.853657	4.81E-09	2.90E-08
4408	LOC388692	305.2510087	-0.839144646	0.164646	-5.096653	3.46E-07	1.54E-06
2245	C7orf27	934.5485144	-0.839212055	0.12523	-6.701348	2.07E-11	1.80E-10
9084	P2RY10	49.46216404	-0.839879086	0.281369	-2.984976	0.002836	0.006119
13748	BFSP2	4.164134148	-0.839933318	0.59878	-1.40274	0.160694	0.229107
10381	DAND5	4.587179844	-0.840024302	0.334372	-2.512242	0.011997	0.022652
2106	TBCB	1076.597924	-0.840523141	0.122806	-6.84432	7.68E-12	7.15E-11
14571	FAM170A	0.36530941	-0.841471798	0.734771	-1.145216	0.25212	0.339153
11154	DRD1	8.915955854	-0.841806098	0.376438	-2.236243	0.025336	0.044515
4852	PPP2R3B	144.6625817	-0.842569766	0.173266	-4.862875	1.16E-06	4.67E-06
10395	ANPEP	10424.86464	-0.842595492	0.33596	-2.50802	0.012141	0.022882
6392	LRRK1	510.3080836	-0.842604359	0.20661	-4.078228	4.54E-05	0.000139
5364	TLR5	155.0754034	-0.842667162	0.183804	-4.584588	4.55E-06	1.66E-05
4765	GALE	947.1173251	-0.84302637	0.172	-4.901306	9.52E-07	3.92E-06
3980	CKS1B	383.071691	-0.843084955	0.157487	-5.353376	8.63E-08	4.25E-07
6282	EHD2	6747.241121	-0.843246843	0.203719	-4.139272	3.48E-05	0.000109
7652	SLC9A5	23.73730858	-0.84425438	0.237261	-3.558337	0.000373	0.000956
6418	BCL3	926.4738047	-0.844294147	0.207551	-4.067883	4.74E-05	0.000145
12707	LOC340508	1.684170414	-0.844512655	0.487764	-1.731395	0.083381	0.128619
8353	HK2	2586.502366	-0.844639235	0.258103	-3.272495	0.001066	0.002502
843	PABPC1	17104.64922	-0.844807821	0.097674	-8.649294	5.18E-18	1.20E-16
11057	OC1001290	1.637053951	-0.845345444	0.372125	-2.271674	0.023106	0.040961
1022	C20orf20	409.3305242	-0.845535187	0.101885	-8.298896	1.05E-16	2.02E-15
15770	LGALS14	0.349684219	-0.845735497	1.07871	-0.784025	0.433025	0.538203
4160	CALHM2	379.4375018	-0.845762463	0.16126	-5.244715	1.57E-07	7.38E-07
5479	HLA-DPA1	12807.81146	-0.845776734	0.186658	-4.531158	5.87E-06	2.10E-05
7427	APOBEC3E	97.01517031	-0.845824514	0.232009	-3.645659	0.000267	0.000704
7571	ACBD7	13.98036356	-0.846211641	0.235754	-3.589382	0.000331	0.000858
5390	FGR	346.3943362	-0.846541194	0.185159	-4.571968	4.83E-06	1.76E-05

6574	GPR65	171.5724671	-0.846828844	0.212045	-3.993623	6.51E-05	0.000194
8608	PSORS1C1	34.49761144	-0.847156531	0.266995	-3.172933	0.001509	0.003436
3512	EFNB1	1190.430667	-0.847333057	0.149605	-5.663808	1.48E-08	8.26E-08
2238	ADRM1	1669.321962	-0.847346795	0.126241	-6.71213	1.92E-11	1.68E-10
8462	LRRC56	35.91552047	-0.847517078	0.263044	-3.22196	0.001273	0.002949
16017	GPR12	0.307258614	-0.847634164	1.200177	-0.706258	0.480028	0.587403
3645	IKBIP	1474.643228	-0.847673283	0.152029	-5.575717	2.47E-08	1.33E-07
3426	RPSAP58	5954.053768	-0.848229428	0.148155	-5.725283	1.03E-08	5.91E-08
11473	S100A1	2032.290856	-0.848439725	0.398621	-2.128438	0.033301	0.056893
6661	MFGE8	2194.082292	-0.84876456	0.214486	-3.957195	7.58E-05	0.000223
2909	C1orf35	211.1380101	-0.84894403	0.139082	-6.103891	1.04E-09	6.98E-09
14876	PTCHD3	0.327319542	-0.849064632	0.813446	-1.043788	0.296584	0.390786
11651	YBX2	67.27982144	-0.849277629	0.412105	-2.06083	0.039319	0.066148
13315	LCN2	42.24623592	-0.849341739	0.552001	-1.53866	0.123887	0.182374
8857	CBS	209.5396066	-0.849411007	0.276897	-3.06761	0.002158	0.004775
2128	THG1L	248.1076417	-0.849512975	0.12449	-6.823942	8.86E-12	8.16E-11
3639	IFFO1	463.2430554	-0.849514088	0.152296	-5.578033	2.43E-08	1.31E-07
1216	AP4M1	373.3967276	-0.849715224	0.106247	-7.997541	1.27E-15	2.04E-14
7975	MGC12916	5.906689225	-0.849742636	0.248457	-3.420073	0.000626	0.001539
4098	POR	2143.442664	-0.849962644	0.16092	-5.281909	1.28E-07	6.11E-07
9362	CD3G	66.83798064	-0.849974385	0.295562	-2.87579	0.00403	0.008438
9667	CFLP1	2.035993751	-0.850328078	0.308247	-2.758594	0.005805	0.01177
10034	BEND4	4.585907685	-0.85045212	0.322077	-2.640525	0.008278	0.01617
7396	GNG3	5.042067169	-0.850528271	0.232475	-3.658581	0.000254	0.000672
2546	C2orf79	298.6058128	-0.85052935	0.132315	-6.428065	1.29E-10	9.95E-10
754	EDEM2	1061.827619	-0.850807472	0.095849	-8.876529	6.90E-19	1.79E-17
5354	ABCB9	115.1002538	-0.850864597	0.185291	-4.592056	4.39E-06	1.61E-05
15050	A2BP1	1.632153979	-0.850943519	0.859392	-0.99017	0.322091	0.419461
7407	DMC1	9.969370815	-0.851702842	0.233131	-3.653327	0.000259	0.000685
7041	XAF1	1119.899857	-0.851734267	0.223943	-3.803357	0.000143	0.000397
2766	EMG1	598.2313123	-0.852009401	0.136905	-6.223381	4.87E-10	3.45E-09
15219	HIST1H2BM	0.305456364	-0.852274197	0.903935	-0.942849	0.345758	0.445312
3878	FAM109B	207.0702282	-0.852474689	0.157409	-5.415677	6.11E-08	3.09E-07
7354	LAMA3	485.0339627	-0.852629428	0.231924	-3.67633	0.000237	0.000631
2500	PLEKHO2	1599.936225	-0.852631118	0.131942	-6.46217	1.03E-10	8.09E-10
8773	SOX30	3.111042201	-0.852748935	0.275532	-3.094915	0.001969	0.004399
6555	LINGO1	364.786008	-0.852896498	0.212976	-4.00466	6.21E-05	0.000186
9608	CSPG5	13.94850076	-0.852917667	0.307156	-2.776823	0.005489	0.011199
5407	COL4A1	27940.26982	-0.853055475	0.187051	-4.560552	5.10E-06	1.85E-05
3364	OCEL1	391.6094172	-0.853481501	0.147931	-5.769471	7.95E-09	4.63E-08
7911	RHOF	321.6761959	-0.853730346	0.247854	-3.444485	0.000572	0.001418
4821	GATSL3	233.1538952	-0.853843357	0.174984	-4.879539	1.06E-06	4.32E-06
8279	BIRC3	3075.74006	-0.853893486	0.25867	-3.301093	0.000963	0.00228
11454	CYP8B1	216.0424587	-0.853944154	0.400272	-2.133408	0.032891	0.056286
6740	ACRC	22.00160277	-0.853973661	0.217549	-3.925426	8.66E-05	0.000252
4784	NTHL1	172.0518611	-0.854178713	0.174575	-4.892904	9.94E-07	4.07E-06
11122	GFI1B	1.180336539	-0.854382965	0.38064	-2.244594	0.024794	0.04369

13032	SULT4A1	19.96248863	-0.854584885	0.522921	-1.634251	0.102206	0.153725
3249	ZDHHC24	370.3592348	-0.854711419	0.146023	-5.853262	4.82E-09	2.91E-08
2418	STX10	299.8391018	-0.854742668	0.130678	-6.540815	6.12E-11	4.96E-10
10540	GRPR	9.88195009	-0.855460031	0.348299	-2.45611	0.014045	0.026119
7512	GFI1	54.15959447	-0.855679406	0.236881	-3.612275	0.000304	0.000792
4909	C17orf67	16.58587188	-0.855770032	0.177469	-4.822074	1.42E-06	5.67E-06
10890	PLAC8L1	2.364098779	-0.855822632	0.368349	-2.3234	0.020158	0.036282
7988	PLD4	183.6046561	-0.856037882	0.25071	-3.414461	0.000639	0.001568
1514	HAUS8	80.16982251	-0.856216797	0.113419	-7.549149	4.38E-14	5.67E-13
9299	EGR2	303.8540488	-0.85625887	0.295172	-2.900886	0.003721	0.007844
4490	MICB	132.7227131	-0.856517535	0.169738	-5.046129	4.51E-07	1.97E-06
1513	PIGT	3018.719783	-0.857474191	0.11357	-7.550149	4.35E-14	5.63E-13
5392	ZNF608	633.4341353	-0.857694468	0.187648	-4.570764	4.86E-06	1.77E-05
12212	NUP210L	0.759193136	-0.858218572	0.453514	-1.892377	0.058441	0.093797
11096	CPS1	127.7963955	-0.85827496	0.380346	-2.256562	0.024035	0.042458
15627	TMPRSS11	0.273057922	-0.858405881	1.037809	-0.827133	0.408162	0.511959
1620	C11orf10	1191.253974	-0.858421542	0.115983	-7.401251	1.35E-13	1.63E-12
16915	TEX101	0.250654709	-0.858568349	1.863819	-0.46065	0.64505	0.74748
16743	CLEC3A	0.219312936	-0.85856865	1.69113	-0.507689	0.611671	0.716082
17284	HELT	0.182121252	-0.858568704	2.309879	-0.371694	0.710121	0.805291
16095	KCNQ2	0.254743659	-0.858568769	1.257666	-0.682668	0.494816	0.602603
15891	NPBWR1	0.252411889	-0.858568826	1.151849	-0.745383	0.45604	0.56251
15754	CTRB2	0.248849176	-0.858568857	1.088967	-0.788425	0.430448	0.53556
15901	PLA2G4E	0.235717566	-0.858568862	1.157239	-0.741911	0.458141	0.564719
16392	TLX2	0.175246506	-0.858568904	1.424768	-0.602603	0.546773	0.653823
3621	NENF	1301.295182	-0.858721951	0.15357	-5.591745	2.25E-08	1.22E-07
4293	WDR13	2858.836246	-0.859134636	0.166506	-5.159797	2.47E-07	1.13E-06
12579	SEZ6	0.817081684	-0.859203122	0.482954	-1.779059	0.07523	0.117226
2717	GALNS	707.6108455	-0.859213851	0.137125	-6.265896	3.71E-10	2.67E-09
16738	GH2	0.295132276	-0.859741718	1.688003	-0.509325	0.610525	0.714911
9799	CYB5R2	73.23118755	-0.859878724	0.31647	-2.717092	0.006586	0.013174
10567	WNK4	124.7837102	-0.860671318	0.351639	-2.447601	0.014381	0.026676
7356	SDC1	4546.33895	-0.861136668	0.234272	-3.675801	0.000237	0.000632
13152	GFRA3	1.821418726	-0.861306517	0.540339	-1.594012	0.110933	0.165329
1276	SP140L	429.4048517	-0.861804506	0.109167	-7.894373	2.92E-15	4.48E-14
7300	TRO	229.4946117	-0.862012292	0.232985	-3.69987	0.000216	0.000579
7830	OR2A4	87.89560035	-0.862507449	0.247414	-3.486094	0.00049	0.001227
4469	PLEKHG2	1041.435425	-0.862609013	0.170583	-5.05684	4.26E-07	1.87E-06
14596	PSG1	0.929702836	-0.862962661	0.759354	-1.136443	0.255771	0.343476
2259	LSM10	402.9781958	-0.863161326	0.129039	-6.689131	2.24E-11	1.95E-10
736	CHST12	430.6764895	-0.863201244	0.096711	-8.925588	4.43E-19	1.18E-17
3460	ARFGAP1	1552.099774	-0.864121357	0.151614	-5.6995	1.20E-08	6.81E-08
5597	PVRIG	98.7600147	-0.864610633	0.193285	-4.47324	7.70E-06	2.70E-05
1575	ETV6	959.9473551	-0.864715596	0.115886	-7.461752	8.54E-14	1.06E-12
6774	HSP90B3P	7.669205287	-0.864760908	0.221205	-3.909323	9.26E-05	0.000268
9797	ZG16B	8.421500264	-0.865036978	0.318339	-2.717347	0.006581	0.013166
10730	ADAM32	7.727896606	-0.865839852	0.362705	-2.387171	0.016979	0.031016

7351	CTRL	14.15806564	-0.866042475	0.235336	-3.680027	0.000233	0.000622
12945	SLC7A5P1	0.548397902	-0.866248044	0.5216	-1.66075	0.096764	0.146517
4021	TP53I13	703.0158496	-0.866480546	0.162629	-5.327953	9.93E-08	4.84E-07
4197	RPL24	11139.57062	-0.866734502	0.166288	-5.21225	1.87E-07	8.71E-07
9803	IKZF3	39.52743521	-0.867081403	0.319284	-2.715709	0.006613	0.013223
1464	DNASE2	1858.961954	-0.867152039	0.11377	-7.621978	2.50E-14	3.34E-13
3884	LOC38895	631.7549086	-0.867584269	0.160282	-5.412853	6.20E-08	3.13E-07
7078	C3orf54	90.42332273	-0.867618024	0.228795	-3.792123	0.000149	0.000414
1272	EIF3B	4129.788172	-0.868251578	0.109939	-7.897603	2.84E-15	4.38E-14
1326	NUTF2	1897.96841	-0.868279787	0.110893	-7.829882	4.88E-15	7.22E-14
10200	C12orf63	2.360732053	-0.868480304	0.336339	-2.582156	0.009819	0.018868
7989	PDE6G	24.53928427	-0.868864159	0.25447	-3.414413	0.000639	0.001568
4964	NDUFS5	2859.679782	-0.8688838	0.181375	-4.790538	1.66E-06	6.57E-06
7629	TMEM184A	297.9781711	-0.868932662	0.243541	-3.567913	0.00036	0.000925
6462	RUNX3	455.8738121	-0.868991142	0.214886	-4.043961	5.26E-05	0.000159
1841	RPL19	19985.36378	-0.869217731	0.121621	-7.14692	8.87E-13	9.44E-12
2248	SCNM1	474.3285239	-0.869523287	0.129787	-6.699595	2.09E-11	1.82E-10
9790	SPAG17	141.2655772	-0.869525581	0.319764	-2.719273	0.006543	0.013099
12869	WNT2	15.66021237	-0.869566594	0.515303	-1.687487	0.09151	0.13938
9529	VWA5B2	5.489793393	-0.869669021	0.309809	-2.807112	0.004999	0.010282
534	SHISA5	3613.426898	-0.869724076	0.091451	-9.510246	1.90E-21	6.98E-20
6448	MPEG1	1362.650106	-0.869980136	0.21476	-4.050947	5.10E-05	0.000155
6025	FAM83H	878.8943598	-0.870815653	0.204675	-4.254624	2.09E-05	6.81E-05
4833	ZNF593	258.213211	-0.87099372	0.178728	-4.873286	1.10E-06	4.45E-06
4393	RIPK3	84.34293409	-0.871008518	0.170527	-5.107735	3.26E-07	1.45E-06
5876	TMEM163	105.8688642	-0.871040269	0.200955	-4.334505	1.46E-05	4.87E-05
4386	C17orf89	289.4866557	-0.87122643	0.170428	-5.112	3.19E-07	1.42E-06
1776	GYS1	2295.482285	-0.871594802	0.120902	-7.20912	5.63E-13	6.22E-12
3064	SHARPIN	1107.18561	-0.871969513	0.145567	-5.990174	2.10E-09	1.34E-08
6734	MS4A7	1396.90097	-0.87287553	0.222237	-3.927686	8.58E-05	0.00025
12411	PGLYRP1	0.986534157	-0.872975565	0.476393	-1.832468	0.066882	0.10562
10931	MADCAM1	1.931216389	-0.873087098	0.378073	-2.309305	0.020927	0.037525
13861	DAB1	1.540447495	-0.873463207	0.639787	-1.365241	0.172177	0.243478
2294	SLC25A19	245.6125912	-0.874173853	0.131265	-6.659611	2.75E-11	2.35E-10
2237	TCOF1	1069.487819	-0.874802663	0.130278	-6.714872	1.88E-11	1.65E-10
8403	LYZ	4935.350581	-0.874856852	0.269407	-3.247343	0.001165	0.002717
5463	GBAP1	788.752432	-0.874891752	0.192858	-4.536447	5.72E-06	2.05E-05
6314	RPL36A	89.8615422	-0.874938233	0.212438	-4.118549	3.81E-05	0.000118
3115	ZDHHC8	751.4882703	-0.875168601	0.147051	-5.951452	2.66E-09	1.67E-08
1522	NCAPD2	992.9964874	-0.875250085	0.116261	-7.528313	5.14E-14	6.62E-13
12913	RPE65	1.159513702	-0.875337654	0.52444	-1.669089	0.0951	0.144355
3128	GNB2L1	33821.08257	-0.875546663	0.147317	-5.943284	2.79E-09	1.75E-08
4545	FAIM3	194.9416039	-0.876184642	0.174827	-5.011714	5.39E-07	2.33E-06
10713	MBL1P	7.228853822	-0.876286655	0.366189	-2.392991	0.016712	0.030576
4184	TBX19	90.76924907	-0.876877359	0.168062	-5.217585	1.81E-07	8.49E-07
5594	CSF2RB	353.1371812	-0.876951522	0.195974	-4.474842	7.65E-06	2.68E-05
8196	HPDL	9.255297668	-0.877452262	0.263209	-3.333667	0.000857	0.00205

12414	UGT1A8	63.03548394	-0.877673347	0.47908	-1.831998	0.066952	0.105713
8661	TXNDC2	1.740922154	-0.877808918	0.278951	-3.146824	0.001651	0.003735
8475	POTEE	3.506175078	-0.87786006	0.272821	-3.217716	0.001292	0.002989
6277	NPAS2	598.6152577	-0.877876786	0.211983	-4.141267	3.45E-05	0.000108
13255	SORCS3	436.9645116	-0.877945229	0.561955	-1.562304	0.118216	0.174814
4997	EIF4EBP3	753.2789817	-0.879194343	0.184093	-4.775814	1.79E-06	7.02E-06
6377	NRBP2	1390.542173	-0.879208719	0.215033	-4.088706	4.34E-05	0.000133
3171	FAM109A	339.8738606	-0.879556685	0.148971	-5.904198	3.54E-09	2.19E-08
12766	GYG2	72.44781322	-0.879633021	0.51328	-1.713748	0.086575	0.132925
13828	OR2AE1	0.416656393	-0.879681845	0.639466	-1.375651	0.16893	0.239455
2809	C20orf24	1489.188934	-0.879872623	0.142341	-6.181459	6.35E-10	4.43E-09
14259	RPRM	3.341126817	-0.880130968	0.709311	-1.240825	0.21467	0.295113
9674	C6orf208	7.866196558	-0.880137306	0.319416	-2.755456	0.005861	0.011875
6919	CXCR7	2121.707758	-0.880268723	0.228249	-3.856608	0.000115	0.000326
6939	CRNA0020[illegible]	5.316499539	-0.880482808	0.228788	-3.848462	0.000119	0.000336
8808	BRSK1	42.69776987	-0.880511198	0.28548	-3.08432	0.00204	0.00454
14197	KLK6	3.567897434	-0.881166623	0.700126	-1.258583	0.208181	0.287424
6280	RIMBP3C	4.03728657	-0.881218849	0.212854	-4.140013	3.47E-05	0.000108
2325	RPL13A	14740.47519	-0.881276116	0.132858	-6.633239	3.28E-11	2.77E-10
7365	SARDH	196.0445468	-0.88282177	0.240373	-3.672717	0.00024	0.000639
8623	COMTD1	267.7963373	-0.883023792	0.278876	-3.166363	0.001544	0.003509
5558	AURKC	12.55934066	-0.883135124	0.196565	-4.492842	7.03E-06	2.48E-05
12882	CLEC4C	0.485816454	-0.883556147	0.526137	-1.679326	0.093089	0.141642
3524	TRABD	702.9199726	-0.883565653	0.15621	-5.656265	1.55E-08	8.60E-08
1295	FKBP9	4328.746046	-0.883864678	0.112289	-7.871346	3.51E-15	5.31E-14
12546	OR3A2	0.856956096	-0.883879046	0.493228	-1.792028	0.073128	0.114242
7964	IL1RAP	514.2506284	-0.884104131	0.258272	-3.423154	0.000619	0.001523
10966	PSPN	0.895203411	-0.885184709	0.385169	-2.298174	0.021552	0.038523
5375	OAS2	1256.704609	-0.885409026	0.193375	-4.57871	4.68E-06	1.71E-05
3570	SLC25A37	690.3893984	-0.88606423	0.157554	-5.623869	1.87E-08	1.03E-07
6374	RPL31P11	3.596591538	-0.886180845	0.216617	-4.091009	4.30E-05	0.000132
3902	C6orf1	326.181436	-0.886402618	0.164036	-5.403711	6.53E-08	3.28E-07
7261	BCL11B	134.5845995	-0.886483179	0.238176	-3.721971	0.000198	0.000534
11638	HOXA13	33.6534946	-0.886489538	0.429306	-2.064939	0.038929	0.065565
1003	DYRK4	340.0191479	-0.886808346	0.106326	-8.34044	7.40E-17	1.45E-15
10105	STRC	14.57353347	-0.886840498	0.339166	-2.614765	0.008929	0.01732
6112	TTYH3	6737.265135	-0.886988747	0.210443	-4.214875	2.50E-05	8.01E-05
11515	ATP10B	5.620148124	-0.887074127	0.420105	-2.111556	0.034725	0.059109
1785	SMAP2	1357.234507	-0.887242552	0.123177	-7.202983	5.89E-13	6.47E-12
8086	C3orf67	98.36843771	-0.887317469	0.262823	-3.376105	0.000735	0.001782
8921	CAPN12	967.4515382	-0.887354449	0.291553	-3.043543	0.002338	0.005137
13342	FLJ42393	2.039475074	-0.887792411	0.579726	-1.531401	0.12567	0.184625
8096	ZNF831	56.00719774	-0.887794421	0.263192	-3.373187	0.000743	0.001799
3325	ZWINT	234.3009152	-0.887956639	0.153074	-5.800846	6.60E-09	3.89E-08
8784	LDHAL6B	2.560293894	-0.888024276	0.287281	-3.091135	0.001994	0.004449
3156	RNPEPL1	2410.006597	-0.888612096	0.150229	-5.915058	3.32E-09	2.06E-08
3048	EZH2	149.0086717	-0.888750682	0.148052	-6.002946	1.94E-09	1.25E-08

3247	ERGIC1	9943.523125	-0.889489995	0.151952	-5.853765	4.81E-09	2.90E-08
9771	DNAJC12	140.6579306	-0.889580819	0.326519	-2.724442	0.006441	0.012921
1550	ARF5	2575.588005	-0.889868878	0.118776	-7.491995	6.78E-14	8.58E-13
6517	CD244	52.02868014	-0.890154815	0.221424	-4.020133	5.82E-05	0.000175
4705	GBP2	3282.80347	-0.890182138	0.180603	-4.928939	8.27E-07	3.44E-06
7418	LRP3	660.4976997	-0.890491647	0.244029	-3.649127	0.000263	0.000695
12908	OR7D2	4.642152699	-0.891442385	0.533324	-1.671484	0.094626	0.143691
11359	SFTPA2	1.061245571	-0.891460514	0.41198	-2.163844	0.030476	0.05259
11047	KCTD4	14.8576678	-0.892167562	0.392285	-2.274283	0.022949	0.040719
2824	CCDC137	413.8158491	-0.892236561	0.144674	-6.167213	6.95E-10	4.82E-09
2512	TFAP4	170.615848	-0.892239837	0.138295	-6.451705	1.11E-10	8.63E-10
3929	YDJC	389.4466506	-0.892277259	0.165711	-5.384524	7.26E-08	3.62E-07
3481	OSBPL5	551.2448018	-0.892444584	0.15694	-5.686526	1.30E-08	7.30E-08
7587	NOV	553.0080526	-0.892716517	0.24908	-3.584049	0.000338	0.000874
13519	PLA2G10	0.553186462	-0.89299983	0.602717	-1.481623	0.138441	0.200728
8421	DBH	10.11859314	-0.893271335	0.275463	-3.242796	0.001184	0.002755
12071	TBX10	0.80284089	-0.89348042	0.461041	-1.937963	0.052628	0.085457
9440	OSR2	27.49626596	-0.893577677	0.314464	-2.841593	0.004489	0.009321
7274	NRGN	204.3886694	-0.893631279	0.240675	-3.713025	0.000205	0.000552
2834	ARHGDIB	5347.265554	-0.893872237	0.145131	-6.15907	7.32E-10	5.06E-09
5799	HMGA1	550.2327009	-0.894293961	0.204444	-4.374265	1.22E-05	4.12E-05
6133	HPGDS	35.35722248	-0.894467541	0.212628	-4.206734	2.59E-05	8.28E-05
2321	SF3A2	968.2809139	-0.894659485	0.134735	-6.640149	3.13E-11	2.65E-10
6251	DACT3	168.0636582	-0.894709748	0.215367	-4.154357	3.26E-05	0.000102
8044	CCR1	489.649387	-0.895029786	0.263659	-3.394653	0.000687	0.001674
2556	C19orf28	1489.95123	-0.896233875	0.139599	-6.420071	1.36E-10	1.04E-09
3532	EHBP1L1	1123.529106	-0.896308688	0.158782	-5.644893	1.65E-08	9.17E-08
4199	PYCRL	338.8395717	-0.896523078	0.172031	-5.211403	1.87E-07	8.75E-07
10340	CDCP2	1.257574276	-0.896752416	0.354029	-2.532993	0.011309	0.021438
10263	TP73	17.650414	-0.897157792	0.350357	-2.560698	0.010446	0.019951
3714	PFDN5	7823.362585	-0.897276173	0.162274	-5.529384	3.21E-08	1.70E-07
8525	POU5F1B	84.38240685	-0.897471974	0.28083	-3.195788	0.001394	0.003206
2339	GRAMD1A	1435.668302	-0.897769221	0.135668	-6.617406	3.66E-11	3.06E-10
11077	THPO	80.2487748	-0.898161889	0.396704	-2.264058	0.023571	0.041709
7800	CEP170L	3.403712996	-0.898190025	0.256491	-3.501836	0.000462	0.001161
15321	SBK2	0.554226416	-0.898666883	0.982634	-0.914549	0.360429	0.461116
2768	OC1002867	170.546865	-0.898681499	0.144428	-6.222368	4.90E-10	3.47E-09
3416	SUV420H2	101.8450228	-0.898974786	0.156682	-5.73756	9.61E-09	5.51E-08
2961	MFSD10	1291.19805	-0.899345566	0.148347	-6.062446	1.34E-09	8.87E-09
6965	EVPL	334.6068432	-0.899542643	0.234195	-3.841006	0.000123	0.000345
8829	CCDC153	13.8205142	-0.899983253	0.292255	-3.079449	0.002074	0.004604
3412	MRPS24	1335.778871	-0.900547935	0.156884	-5.740229	9.45E-09	5.43E-08
11709	EMID2	6.019520125	-0.900567793	0.439252	-2.050231	0.040342	0.067533
6733	RRN3P2	21.2014366	-0.900769008	0.229317	-3.928045	8.56E-05	0.000249
4206	VASN	1551.643217	-0.900983938	0.173093	-5.205188	1.94E-07	9.03E-07
7761	CHRNA5	24.74607297	-0.901059526	0.256439	-3.513741	0.000442	0.001116
10316	KIAA0319	43.6804727	-0.901866825	0.3549	-2.541189	0.011048	0.020991

5448	CLEC4A	82.20957066	-0.901900525	0.198552	-4.542383	5.56E-06	2.00E-05
12663	SH3GL3	1.088179652	-0.902660989	0.515049	-1.752573	0.079675	0.123329
3593	MPG	891.1734181	-0.902881975	0.160992	-5.608244	2.04E-08	1.12E-07
2107	C19orf22	1093.79498	-0.903232091	0.131997	-6.842801	7.77E-12	7.22E-11
7899	KCNA5	50.79379498	-0.903515541	0.261983	-3.448751	0.000563	0.001398
2724	PRELID1	2913.340342	-0.904979911	0.14454	-6.261109	3.82E-10	2.75E-09
8443	F2RL2	37.83215705	-0.905144812	0.28019	-3.23047	0.001236	0.002869
5178	SNAI3	33.15524449	-0.905285236	0.193221	-4.685224	2.80E-06	1.06E-05
4715	ZCCHC24	621.9518013	-0.905374609	0.18381	-4.925595	8.41E-07	3.50E-06
4816	LOC15077(	352.9737177	-0.905636398	0.185533	-4.881269	1.05E-06	4.29E-06
7693	C6orf97	64.72790544	-0.906067363	0.255754	-3.542734	0.000396	0.001009
6118	LOC72839	112.8074144	-0.906268941	0.215185	-4.211575	2.54E-05	8.12E-05
6097	NFATC4	437.7711531	-0.90670747	0.214726	-4.222618	2.41E-05	7.76E-05
8128	C1orf228	9.347303031	-0.906784067	0.269871	-3.360062	0.000779	0.001879
7711	NPIPL3	933.5055405	-0.907322866	0.25666	-3.535118	0.000408	0.001036
5784	HEPH	460.9378055	-0.907385867	0.207207	-4.379119	1.19E-05	4.04E-05
7852	KCNA3	33.84987558	-0.907594686	0.261506	-3.470652	0.000519	0.001296
2491	DDX39	982.4217923	-0.907964273	0.140222	-6.475187	9.47E-11	7.45E-10
11757	KRT5	15.32700436	-0.908323171	0.446312	-2.035177	0.041833	0.069743
10315	TTLL6	156.5283741	-0.908396394	0.35739	-2.541749	0.01103	0.02096
3757	MCM2	532.0627619	-0.908466117	0.165099	-5.502552	3.74E-08	1.95E-07
419	DAGLB	526.1662105	-0.908577602	0.091219	-9.960407	2.27E-23	1.06E-21
6201	LILRA2	79.00822295	-0.908791526	0.217661	-4.175252	2.98E-05	9.41E-05
11381	C21orf15	5.120770168	-0.908809486	0.421067	-2.158346	0.030901	0.053219
5167	UFSP1	25.69626443	-0.909041366	0.193787	-4.690928	2.72E-06	1.03E-05
3186	FANCD2	107.6199784	-0.909364817	0.154379	-5.890476	3.85E-09	2.37E-08
13167	HTR6	23.38546652	-0.909738822	0.572109	-1.59015	0.111801	0.166432
14469	CELA1	0.318648764	-0.90977012	0.772606	-1.177534	0.238982	0.323747
4234	ARID5A	654.8642938	-0.909795158	0.175292	-5.190174	2.10E-07	9.73E-07
11156	TMPRSS3	171.4899118	-0.909801589	0.406832	-2.236307	0.025332	0.044515
10866	ACCN4	2.466284518	-0.909865156	0.38968	-2.334904	0.019548	0.035263
5956	RTN1	195.3014958	-0.909891864	0.21224	-4.287086	1.81E-05	5.96E-05
474	ACP2	1184.256252	-0.909992985	0.093601	-9.722062	2.43E-22	1.00E-20
5034	OC1002721	46.61601885	-0.910902608	0.191512	-4.75638	1.97E-06	7.67E-06
4389	ATP6AP1L	50.80306269	-0.911512882	0.178402	-5.109306	3.23E-07	1.44E-06
9574	G0S2	1092.391924	-0.911681069	0.32673	-2.790323	0.005266	0.01078
7511	C3orf35	7.282573216	-0.911714868	0.252385	-3.612402	0.000303	0.000792
6558	RPGRIP1	8.31452871	-0.912221788	0.227934	-4.002133	6.28E-05	0.000188
10380	GP6	16.54046774	-0.912251788	0.363087	-2.512491	0.011988	0.022638
6681	MEGF6	505.4445064	-0.912559982	0.231104	-3.948691	7.86E-05	0.000231
3533	LOC38879(	300.0019458	-0.91310982	0.161769	-5.644517	1.66E-08	9.19E-08
14014	KLRG2	1.35845317	-0.913169928	0.694605	-1.31466	0.188624	0.263824
5148	H2AFJ	1327.404884	-0.913527699	0.194193	-4.704227	2.55E-06	9.70E-06
272	GUSB	2050.088985	-0.913584806	0.085177	-10.72567	7.71E-27	5.56E-25
9904	SYCP2L	4.89275837	-0.913633024	0.34008	-2.686528	0.00722	0.014289
14203	C12orf50	0.36205663	-0.913801943	0.727791	-1.255582	0.209267	0.288802
830	EXOSC1	289.5752258	-0.913867109	0.105323	-8.676789	4.07E-18	9.61E-17

7066	OR51E1	180.306548	-0.913910562	0.240774	-3.795716	0.000147	0.000408
7752	NFE2	14.35687641	-0.914163262	0.259831	-3.518302	0.000434	0.001098
3914	C6orf125	662.665493	-0.91433285	0.169514	-5.393833	6.90E-08	3.45E-07
2348	FAM89B	837.9741537	-0.914582983	0.138357	-6.610292	3.84E-11	3.20E-10
5842	C5orf39	93.9272785	-0.914808027	0.210307	-4.349879	1.36E-05	4.57E-05
8682	MRC1	577.0257389	-0.91520377	0.291641	-3.13812	0.0017	0.003839
11962	RBMXL2	1.344999357	-0.915317435	0.464447	-1.970769	0.04875	0.079882
6173	HIST1H4J	16.19243863	-0.915319763	0.218519	-4.188736	2.81E-05	8.91E-05
697	RCC2	2019.251052	-0.915727449	0.101409	-9.030018	1.72E-19	4.83E-18
11144	FAM196A	10.60896337	-0.915903593	0.408962	-2.239578	0.025118	0.04418
17503	SPANXA2	0.181664473	-0.916275279	2.91407	-0.314431	0.753193	0.843475
16972	FAM181A	0.22978386	-0.916275859	2.057995	-0.445227	0.656155	0.757795
16997	SLCO1B1	0.180106834	-0.916276083	2.082531	-0.439982	0.65995	0.761057
15911	IL1F8	0.245871887	-0.916276492	1.240662	-0.738538	0.460187	0.566912
15560	OSTN	0.216690573	-0.916276627	1.08505	-0.844456	0.398415	0.50187
5065	TMEM154	65.44846247	-0.916792166	0.193453	-4.739106	2.15E-06	8.31E-06
3952	FBXL6	288.79109	-0.917367825	0.170697	-5.374252	7.69E-08	3.81E-07
9528	HAPLN1	384.2579462	-0.917450535	0.326818	-2.807225	0.004997	0.01028
6948	NT5DC3	987.9824116	-0.917813121	0.238676	-3.845443	0.00012	0.000339
2667	RPL35A	10028.9667	-0.917828041	0.145337	-6.315174	2.70E-10	1.98E-09
9048	INHBC	6.641002666	-0.918440844	0.30651	-2.996448	0.002731	0.005917
335	PABPC4	2671.668897	-0.918748032	0.088511	-10.38002	3.06E-25	1.79E-23
3433	STYXL1	538.1743167	-0.919403924	0.160689	-5.721647	1.05E-08	6.02E-08
9174	FAM177B	6.277276088	-0.919685009	0.312024	-2.94748	0.003204	0.006845
9005	PSORS1C2	8.737642712	-0.919867321	0.305631	-3.009728	0.002615	0.005692
6732	C2orf65	14.82705537	-0.919873981	0.234171	-3.928211	8.56E-05	0.000249
8319	BREA2	3.07834636	-0.920411382	0.280336	-3.283246	0.001026	0.002417
4929	CD320	890.5746542	-0.920491349	0.19131	-4.81151	1.50E-06	5.96E-06
2799	TUBB6	1831.532034	-0.92049547	0.148651	-6.192342	5.93E-10	4.15E-09
6477	GHRLOS	10.71726574	-0.920536127	0.228003	-4.037392	5.40E-05	0.000164
10987	PMCH	15.04767624	-0.920569905	0.401712	-2.291614	0.021928	0.03912
8100	LOC440461	3.288007801	-0.920602943	0.273078	-3.371208	0.000748	0.001811
9263	RPS27	664.1335664	-0.921577699	0.316491	-2.91186	0.003593	0.007603
13705	LOC646627	0.645085568	-0.922374694	0.651075	-1.416695	0.156572	0.223931
2428	RPL12	10094.11473	-0.922635326	0.141184	-6.534965	6.36E-11	5.14E-10
4209	FAM53A	18.10671834	-0.922676004	0.177327	-5.203258	1.96E-07	9.12E-07
3466	KIAA1274	621.0749844	-0.923001708	0.161996	-5.69768	1.21E-08	6.87E-08
9388	MIR155HG	82.0218407	-0.923313599	0.322392	-2.86395	0.004184	0.008736
1669	MCM7	1463.362205	-0.923704917	0.125892	-7.337295	2.18E-13	2.56E-12
10140	MYOZ2	1.401088444	-0.923937266	0.355086	-2.602011	0.009268	0.017915
4597	FAM156A	819.6480923	-0.924220152	0.185249	-4.989082	6.07E-07	2.59E-06
7443	WFDC1	118.8544775	-0.924372679	0.254033	-3.638793	0.000274	0.000721
3856	CARHSP1	2888.796846	-0.924377521	0.170287	-5.428341	5.69E-08	2.89E-07
2389	DBNDD2	704.1772702	-0.924744139	0.140763	-6.569521	5.05E-11	4.14E-10
13549	VIT	0.859700528	-0.924782516	0.628586	-1.471212	0.141234	0.204319
9548	HEPHL1	3.49607262	-0.924883872	0.330086	-2.801945	0.00508	0.010428
6164	KHDC1	19.53418145	-0.925101612	0.220539	-4.194736	2.73E-05	8.69E-05

7858	SGCG	2.360555547	-0.925494329	0.266857	-3.468133	0.000524	0.001307
7187	C14orf176	21.04133381	-0.925634261	0.246841	-3.749916	0.000177	0.000482
1769	ZNF668	211.9234359	-0.92584486	0.128193	-7.222274	5.11E-13	5.66E-12
7272	)C1001336	12.23888037	-0.925879276	0.249329	-3.71348	0.000204	0.000551
9030	C6orf147	6.980666982	-0.926300318	0.308554	-3.002072	0.002681	0.005821
7896	GZMA	383.8743191	-0.926451969	0.26848	-3.450726	0.000559	0.001388
6515	TCF19	712.1808701	-0.926655702	0.230424	-4.021521	5.78E-05	0.000174
3668	AGTRAP	1065.929942	-0.926755336	0.166817	-5.555519	2.77E-08	1.48E-07
2678	ARL6IP4	3101.494578	-0.926797808	0.147131	-6.299117	2.99E-10	2.19E-09
1152	OST4	2462.334447	-0.926836989	0.114627	-8.085674	6.18E-16	1.05E-14
1086	AP2S1	1266.587773	-0.927974878	0.113434	-8.180777	2.82E-16	5.09E-15
7588	FKBP5	5049.710128	-0.928573767	0.259134	-3.583372	0.000339	0.000876
10453	ITGAD	30.70652637	-0.92864641	0.372764	-2.491245	0.01273	0.02387
10031	BHLHA15	11.722707	-0.928659133	0.351595	-2.641272	0.00826	0.016139
13875	KDM5D	371.4623829	-0.92868707	0.682928	-1.35986	0.173874	0.245635
10687	FER1L5	3.277764194	-0.928707436	0.386787	-2.40108	0.016347	0.029982
11918	KRT1	2.82581138	-0.929102217	0.468596	-1.982735	0.047397	0.077952
10387	FAM3D	5.242782743	-0.92942658	0.370163	-2.510856	0.012044	0.022728
1359	OBFC2B	762.4244132	-0.930181255	0.119548	-7.78085	7.20E-15	1.04E-13
5744	C16orf54	90.65000873	-0.930346788	0.211423	-4.400411	1.08E-05	3.69E-05
2796	RPS17	11042.86749	-0.930385423	0.150176	-6.195301	5.82E-10	4.08E-09
2179	UBA52	8699.533894	-0.931183474	0.137364	-6.778952	1.21E-11	1.09E-10
13808	DNMT3L	0.972712253	-0.931511558	0.672927	-1.384269	0.166276	0.236036
9135	PCSK6	4256.609255	-0.931608929	0.314246	-2.964588	0.003031	0.006503
12119	WDR49	0.631409683	-0.931848147	0.484627	-1.922816	0.054503	0.088152
3381	RPS14	18306.30813	-0.931882956	0.161817	-5.758861	8.47E-09	4.91E-08
11116	ALOX12P2	15.26405693	-0.932013953	0.414881	-2.246458	0.024675	0.043509
2112	QPCTL	246.3457103	-0.932512975	0.136423	-6.835472	8.17E-12	7.59E-11
6516	ESPL1	140.1512432	-0.932720491	0.231957	-4.021085	5.79E-05	0.000174
6354	SHC4	14.71592532	-0.932976134	0.227597	-4.099241	4.15E-05	0.000128
13720	YSK4	0.40668604	-0.933646701	0.661338	-1.411755	0.158022	0.225757
13044	AKNAD1	1.341839185	-0.933727675	0.572208	-1.631799	0.102722	0.154358
13075	ASTL	0.427293584	-0.934079245	0.576114	-1.621345	0.104944	0.157323
2014	CNPY3	1592.294801	-0.93511762	0.134716	-6.941404	3.88E-12	3.78E-11
10835	SRD5A2	2.506387899	-0.935146565	0.398304	-2.34782	0.018884	0.034161
5864	CACNA1H	545.8785376	-0.935200522	0.215533	-4.339011	1.43E-05	4.78E-05
9834	IDO2	3.700158164	-0.93533397	0.345372	-2.708197	0.006765	0.013484
3438	C11orf75	481.379366	-0.93556996	0.163585	-5.719153	1.07E-08	6.10E-08
496	ARHGEF2	1494.636927	-0.935606877	0.097102	-9.635279	5.67E-22	2.24E-20
13163	CYP26C1	2.411012413	-0.935738547	0.588107	-1.591101	0.111587	0.166164
5384	MMP14	7115.873479	-0.935908148	0.204505	-4.576453	4.73E-06	1.72E-05
5134	C1orf213	62.31080386	-0.936094793	0.198666	-4.71191	2.45E-06	9.37E-06
3038	C19orf53	1675.353759	-0.936852542	0.155994	-6.005704	1.91E-09	1.23E-08
10062	GJB2	1092.641835	-0.936889415	0.355968	-2.63195	0.00849	0.016538
10466	BAI2	81.2524572	-0.937350617	0.376893	-2.487048	0.012881	0.024124
3771	MRPL55	559.6327196	-0.937381145	0.1706	-5.494621	3.92E-08	2.04E-07
3087	RASA3	846.5545827	-0.937789789	0.157043	-5.971542	2.35E-09	1.49E-08

5405	LTB4R	269.1175391	-0.937846571	0.205581	-4.561924	5.07E-06	1.84E-05
1165	CALR	27937.14789	-0.937850161	0.116242	-8.068057	7.14E-16	1.20E-14
2293	LPXN	502.702872	-0.938043378	0.1408	-6.662236	2.70E-11	2.31E-10
1202	NAGPA	359.8179389	-0.939225818	0.117234	-8.011573	1.13E-15	1.85E-14
4006	GYPC	1416.295871	-0.939320002	0.175997	-5.337147	9.44E-08	4.62E-07
2258	RPS11	24445.31102	-0.939360353	0.140416	-6.689839	2.23E-11	1.94E-10
8404	FHL1	6290.877586	-0.939737247	0.289393	-3.247273	0.001165	0.002718
8724	KLRC3	6.556520332	-0.940115838	0.301472	-3.118417	0.001818	0.004085
5783	CHRNE	40.50877146	-0.940778033	0.214772	-4.380359	1.18E-05	4.02E-05
11604	SNX32	5.473458143	-0.941085835	0.452531	-2.079607	0.037562	0.063448
2380	CSTB	3110.87886	-0.941108524	0.143065	-6.578208	4.76E-11	3.92E-10
7432	CST7	288.1273952	-0.94185807	0.258474	-3.643919	0.000269	0.000708
9707	FAM43B	28.90279931	-0.942102632	0.343161	-2.74537	0.006044	0.012204
4044	RAB40C	914.2669914	-0.942171857	0.177457	-5.309309	1.10E-07	5.33E-07
6753	OLR1	397.4777982	-0.94242625	0.240541	-3.917941	8.93E-05	0.000259
5289	CTSS	3613.151246	-0.942616874	0.203651	-4.628599	3.68E-06	1.36E-05
9077	ABCA9	36.76498202	-0.942715465	0.315569	-2.987355	0.002814	0.006077
10509	ACCN2	54.94959479	-0.943125601	0.382013	-2.46883	0.013556	0.025283
3768	DSE	859.3892439	-0.943157926	0.171543	-5.498081	3.84E-08	2.00E-07
7726	RHPN1	346.3546757	-0.943183315	0.267172	-3.530246	0.000415	0.001053
7619	TEKT3	4.065136275	-0.943213812	0.264029	-3.572383	0.000354	0.00091
8037	PBK	82.36913121	-0.943435073	0.277775	-3.396397	0.000683	0.001665
15233	NBLA0030	0.321381967	-0.94346237	1.005676	-0.938138	0.348174	0.448011
4265	DLG5	1244.993571	-0.944027	0.182449	-5.174186	2.29E-07	1.05E-06
7236	IFI27	2265.887021	-0.944165037	0.253093	-3.730513	0.000191	0.000518
9096	BEGAIN	9.088482976	-0.944609488	0.317184	-2.978113	0.0029	0.00625
2144	CSDA	5752.723365	-0.944671297	0.138667	-6.812527	9.59E-12	8.77E-11
3870	PDLIM2	625.9086092	-0.944941877	0.174298	-5.421428	5.91E-08	2.99E-07
12023	PLA2G12B	116.728777	-0.945961448	0.483859	-1.955037	0.050579	0.082461
4585	TNFSF4	107.334372	-0.946045582	0.189477	-4.992924	5.95E-07	2.54E-06
14424	AIPL1	0.401554428	-0.946447008	0.794676	-1.190984	0.23366	0.317524
8497	HLA-DOB	104.9045762	-0.946508802	0.295099	-3.207429	0.001339	0.003089
8214	RPH3A	7.280771867	-0.946908505	0.284704	-3.325943	0.000881	0.002103
2615	POLD2	2099.932792	-0.947055514	0.148858	-6.362157	1.99E-10	1.49E-09
5113	MFRP	970.6195829	-0.947204147	0.200605	-4.721736	2.34E-06	8.96E-06
11972	PRAP1	693.795784	-0.947822028	0.481602	-1.96806	0.049061	0.080325
14599	ADAM18	14.75968511	-0.948989979	0.835434	-1.135924	0.255988	0.343697
6548	GLIPR1	1136.396277	-0.949383162	0.236979	-4.006186	6.17E-05	0.000185
8699	PLSCR2	2.190272785	-0.949493326	0.303252	-3.131032	0.001742	0.003925
8049	IRS2	1422.511105	-0.949537791	0.279869	-3.392789	0.000692	0.001685
4663	NCF2	407.5891451	-0.949541269	0.191647	-4.954624	7.25E-07	3.05E-06
9203	ZNF80	7.690564087	-0.949570467	0.323274	-2.937359	0.00331	0.00705
2385	RPL13AP2	25.96334179	-0.949669693	0.14449	-6.572558	4.95E-11	4.06E-10
4864	MPST	1171.062635	-0.949956839	0.195658	-4.855184	1.20E-06	4.85E-06
5924	GPR183	391.4924609	-0.950152099	0.220389	-4.311249	1.62E-05	5.37E-05
6687	LMOD1	550.240201	-0.950215672	0.24076	-3.946735	7.92E-05	0.000232
1736	CXCL16	1721.808966	-0.950221293	0.130963	-7.25564	4.00E-13	4.51E-12

8910	RFPL2	3.231824325	-0.95099942	0.312006	-3.048016	0.002304	0.005068
9491	DNAH2	6.078673071	-0.9510275	0.337083	-2.821346	0.004782	0.009875
4029	RFXANK	990.7395812	-0.951913405	0.178988	-5.318306	1.05E-07	5.10E-07
3552	FES	359.7316878	-0.951930555	0.168977	-5.633487	1.77E-08	9.75E-08
7253	NPTXR	173.0388716	-0.952357621	0.25577	-3.723492	0.000196	0.000531
15636	MRAP	0.579149664	-0.953062099	1.154072	-0.825825	0.408903	0.512594
7714	TLR7	295.2644578	-0.954228866	0.269994	-3.534256	0.000409	0.001039
2822	ZCWPW1	106.6812151	-0.95425918	0.154678	-6.169346	6.86E-10	4.76E-09
13660	C3orf43	0.348447769	-0.954321159	0.66478	-1.435545	0.151132	0.216862
5892	GEM	1058.598867	-0.954368533	0.220663	-4.325013	1.53E-05	5.07E-05
2477	RPS16	13456.37297	-0.954542337	0.147047	-6.49142	8.50E-11	6.73E-10
4634	TEX14	4.625414627	-0.954637873	0.19201	-4.971801	6.63E-07	2.81E-06
4138	TSHZ3	225.8075056	-0.95528972	0.181615	-5.259975	1.44E-07	6.82E-07
8906	SDK1	662.0581693	-0.955531568	0.313305	-3.049845	0.00229	0.005039
3053	UBTD1	260.3256804	-0.955628235	0.159338	-5.997501	2.00E-09	1.29E-08
2869	MTCP1NB	648.3409629	-0.956292029	0.156007	-6.129806	8.80E-10	6.01E-09
10658	SUMO1P1	0.976494234	-0.956327175	0.396849	-2.409801	0.015961	0.029354
3619	ANAPC11	954.4678946	-0.956459997	0.171	-5.593325	2.23E-08	1.21E-07
2310	FANCI	340.0204399	-0.956513088	0.143893	-6.647385	2.98E-11	2.53E-10
10472	BRSK2	10.49856615	-0.95662795	0.385101	-2.484097	0.012988	0.02431
10596	CD300E	7.098241716	-0.95679826	0.392632	-2.436884	0.014814	0.027404
8064	KRT86	18.17357489	-0.956852885	0.282536	-3.386654	0.000708	0.00172
2232	GAA	3869.782024	-0.957151101	0.142467	-6.7184	1.84E-11	1.61E-10
7518	CLEC12A	68.80181999	-0.957185011	0.265268	-3.608363	0.000308	0.000803
8192	CLDN9	10.6396758	-0.95748715	0.28701	-3.33608	0.00085	0.002033
8271	MYB	13.23701895	-0.957491111	0.289789	-3.304099	0.000953	0.002258
6976	CABP4	7.34764881	-0.958057188	0.249838	-3.834716	0.000126	0.000353
12774	C21orf90	1.976487971	-0.958279436	0.559718	-1.712077	0.086883	0.133316
7057	GPR171	48.99494777	-0.958472832	0.252355	-3.798114	0.000146	0.000405
8935	HIST1H3D	12.89834977	-0.95854539	0.315356	-3.039565	0.002369	0.005197
2015	PSENEN	1062.156597	-0.959506898	0.138232	-6.941304	3.88E-12	3.78E-11
13320	KRT12	0.799450673	-0.959882268	0.624412	-1.537259	0.12423	0.18281
11311	NEFL	1078.450067	-0.959946029	0.441268	-2.175425	0.029598	0.051291
13978	LOC729668	0.305668157	-0.960660194	0.725137	-1.324798	0.185238	0.259755
3626	SLC27A3	650.0275727	-0.961014547	0.171916	-5.590012	2.27E-08	1.23E-07
2394	RNF24	286.1651765	-0.961123717	0.146422	-6.564067	5.24E-11	4.29E-10
14153	LOC285548	0.612831174	-0.961150605	0.756522	-1.270487	0.203911	0.282404
6297	FAM72A	8.268390709	-0.962140295	0.233029	-4.128841	3.65E-05	0.000113
4560	IGFBP4	24091.5988	-0.962665192	0.192386	-5.00383	5.62E-07	2.42E-06
1532	LTBR	2990.166242	-0.962751522	0.128112	-7.514899	5.70E-14	7.29E-13
4665	STAT4	109.2154979	-0.963109911	0.194402	-4.954206	7.26E-07	3.05E-06
3612	TSPAN15	745.1617807	-0.963212964	0.171963	-5.601274	2.13E-08	1.15E-07
13087	CXorf1	2.715762941	-0.964361371	0.596853	-1.615744	0.10615	0.158985
2407	C13orf29	111.2681411	-0.96484048	0.147272	-6.55142	5.70E-11	4.64E-10
583	CHPF2	1459.259082	-0.96524427	0.103007	-9.37068	7.21E-21	2.42E-19
1842	SLC35E4	100.7562259	-0.965538204	0.135098	-7.146943	8.87E-13	9.44E-12
3357	C1orf122	454.2229825	-0.965556617	0.167218	-5.774228	7.73E-09	4.51E-08

5282	IKZF1	525.9627187	-0.965563939	0.208338	-4.634614	3.58E-06	1.33E-05
6111	MYBL1	210.701104	-0.966588534	0.229306	-4.215275	2.49E-05	8.00E-05
7605	C6orf59	7.672987402	-0.966974767	0.270237	-3.578243	0.000346	0.000892
8866	CXCL9	1543.540066	-0.967296286	0.315687	-3.064102	0.002183	0.004826
3767	C17orf106	378.9210158	-0.967325362	0.175924	-5.498545	3.83E-08	1.99E-07
9300	HSF4	1051.916409	-0.967562186	0.333551	-2.900793	0.003722	0.007845
11678	KISS1R	172.643967	-0.968002781	0.470682	-2.056598	0.039725	0.066677
6562	LOC647121	43.14014574	-0.968003571	0.241934	-4.001104	6.30E-05	0.000188
13953	KLK5	0.598193218	-0.96824076	0.726224	-1.333254	0.182449	0.256283
5444	PTK7	703.0208781	-0.968417974	0.21309	-4.544643	5.50E-06	1.98E-05
2845	RIPK2	546.2810008	-0.96856072	0.157531	-6.148391	7.83E-10	5.39E-09
5752	MIF	9648.914934	-0.968691924	0.220347	-4.396206	1.10E-05	3.75E-05
3772	DPCD	558.1206574	-0.968724864	0.176376	-5.492394	3.97E-08	2.06E-07
3016	VAMP5	1051.165004	-0.969701558	0.16103	-6.02186	1.72E-09	1.12E-08
1297	RPS24	16620.60305	-0.970822648	0.12336	-7.869838	3.55E-15	5.37E-14
7725	C4A	33941.89368	-0.971092907	0.275029	-3.530873	0.000414	0.001051
14313	OBP2B	0.285405707	-0.971392801	0.792069	-1.226398	0.220049	0.301354
781	TMED9	5265.567879	-0.971663972	0.11034	-8.806067	1.30E-18	3.25E-17
10622	FAM66D	22.51559292	-0.971840663	0.400561	-2.426199	0.015258	0.028156
5133	C19orf57	18.53295814	-0.972018904	0.206259	-4.712605	2.45E-06	9.34E-06
13896	SPATA8	1.025429635	-0.972093511	0.720275	-1.349614	0.17714	0.249864
8070	ICOS	31.02585958	-0.972700844	0.287419	-3.384265	0.000714	0.001733
1051	RPL30	12644.80762	-0.972741758	0.118047	-8.240295	1.72E-16	3.20E-15
8110	FBLN2	875.3832839	-0.972832763	0.289078	-3.365293	0.000765	0.001848
12646	FOXG1	1.322048665	-0.972835527	0.55324	-1.758433	0.078674	0.121943
4324	FADS3	669.8677793	-0.973152498	0.189137	-5.145216	2.67E-07	1.21E-06
8602	CHST13	271.7266321	-0.973159922	0.306541	-3.174644	0.0015	0.003418
2184	ANKRD13D	400.6449012	-0.973448432	0.143702	-6.774061	1.25E-11	1.12E-10
8587	SHH	26.77051269	-0.973451412	0.306299	-3.178113	0.001482	0.003384
17233	NKX2-4	0.254166591	-0.973982513	2.558648	-0.380663	0.703453	0.800115
17308	CSAG2	0.171906959	-0.973982939	2.66542	-0.365414	0.714802	0.809501
16897	DPPA2	0.184961623	-0.9739835	2.090951	-0.465809	0.641352	0.743943
16905	S100A7A	0.170313924	-0.973983617	2.099167	-0.463986	0.642658	0.745149
16695	PPY	0.193000706	-0.973983742	1.873101	-0.519985	0.603074	0.708048
15907	SMYD1	0.174493121	-0.97398429	1.314601	-0.740897	0.458756	0.565309
7423	TP63	16.37819289	-0.974195865	0.267079	-3.647595	0.000265	0.000699
4742	RPL39	637.0572535	-0.975097886	0.198476	-4.912938	8.97E-07	3.71E-06
4410	RBP1	239.943694	-0.975581099	0.191493	-5.094603	3.49E-07	1.55E-06
9262	IL1A	2.703384095	-0.976215512	0.335257	-2.91184	0.003593	0.007603
10914	OGN	50.31514857	-0.977047766	0.422091	-2.31478	0.020625	0.037041
7613	ADAM11	17.61907777	-0.977078418	0.273215	-3.576231	0.000349	0.000897
4407	LRRC45	307.0885081	-0.97725502	0.191714	-5.097453	3.44E-07	1.53E-06
10869	TSPO2	1.948236288	-0.977712844	0.419003	-2.333429	0.019626	0.035393
2618	NSUN5	463.6709085	-0.978205731	0.153852	-6.358094	2.04E-10	1.53E-09
5193	GLI3	235.7535141	-0.978599515	0.209123	-4.679531	2.88E-06	1.09E-05
8533	APOE	15668.56091	-0.978623707	0.306389	-3.194061	0.001403	0.003223
11625	LRFN5	15.66237561	-0.979113567	0.47299	-2.070051	0.038448	0.064827

2336	RPL27	8579.211696	-0.979322812	0.147956	-6.619032	3.62E-11	3.03E-10
8292	KCNT2	79.02206221	-0.979689168	0.297102	-3.297488	0.000976	0.002306
1737	AMPD2	1126.395986	-0.979762825	0.135057	-7.254426	4.03E-13	4.55E-12
9584	CCL17	8.011329429	-0.980052141	0.351713	-2.786508	0.005328	0.010897
12647	UPK1B	88.40984307	-0.980145172	0.557432	-1.758322	0.078693	0.121962
12741	AFP	7.399822986	-0.980417664	0.569935	-1.720226	0.085391	0.131368
443	STAT2	2268.374361	-0.980805307	0.099694	-9.838132	7.71E-23	3.42E-21
4947	DNMT3B	90.41057728	-0.98147467	0.20442	-4.80127	1.58E-06	6.25E-06
6259	HES6	62.12501007	-0.981578399	0.236452	-4.151273	3.31E-05	0.000104
7441	MN1	222.6368473	-0.981757878	0.269719	-3.639933	0.000273	0.000718
7484	IL34	212.34033	-0.981802593	0.271113	-3.621383	0.000293	0.000767
7821	MDS2	2.205046434	-0.981858586	0.281007	-3.494072	0.000476	0.001192
2867	TKT	3380.850164	-0.981944372	0.160166	-6.130806	8.74E-10	5.98E-09
14708	MS4A3	0.586123824	-0.982692472	0.896921	-1.095629	0.273241	0.364142
8024	GPAT2	54.37374037	-0.982718881	0.288901	-3.401578	0.00067	0.001637
4282	MXRA7	6484.096036	-0.983673018	0.190406	-5.166194	2.39E-07	1.09E-06
1114	SNRPA	967.502144	-0.983706374	0.120859	-8.139313	3.98E-16	6.99E-15
7419	SPATC1	4.955302594	-0.983744094	0.269599	-3.64891	0.000263	0.000696
2271	AKNA	894.5606551	-0.983856253	0.14738	-6.675649	2.46E-11	2.12E-10
2790	CHEK2	145.3463307	-0.983993456	0.158718	-6.199627	5.66E-10	3.98E-09
12931	SDR16C5	1.221417593	-0.984644565	0.591506	-1.664639	0.095985	0.145506
10215	LOC28423	8.247348722	-0.984916289	0.382222	-2.576817	0.009971	0.019134
1355	GTF3A	2525.573647	-0.984919727	0.126487	-7.786709	6.88E-15	9.95E-14
879	C17orf62	1385.739743	-0.985042926	0.11486	-8.576053	9.82E-18	2.19E-16
6591	SECTM1	484.2354854	-0.985221051	0.247122	-3.986782	6.70E-05	0.000199
10303	CA9	5264.024522	-0.986045218	0.387536	-2.544398	0.010947	0.020825
9349	HIF3A	150.0227757	-0.98693854	0.342821	-2.878874	0.003991	0.008365
9246	MGC42105	42.38534526	-0.987959562	0.338327	-2.920132	0.003499	0.007417
2224	LMCD1	571.4632075	-0.988511127	0.146892	-6.729496	1.70E-11	1.50E-10
7678	PTPLA	38.14189363	-0.988747631	0.278689	-3.54785	0.000388	0.000992
9991	TMEM100	226.4767182	-0.989453597	0.37266	-2.65511	0.007928	0.015555
8231	NELL2	63.35447451	-0.989509303	0.298065	-3.319772	0.000901	0.002145
9944	FAM182A	3.639542224	-0.98995248	0.37038	-2.672803	0.007522	0.014827
3059	RPL8	30065.89167	-0.989977635	0.165193	-5.992846	2.06E-09	1.32E-08
7760	BTLA	22.36967894	-0.990070812	0.281708	-3.514531	0.000441	0.001113
5546	SMARCD3	371.7656552	-0.990451836	0.220105	-4.499907	6.80E-06	2.40E-05
9112	MRS2P2	4.163254597	-0.990635783	0.333079	-2.974179	0.002938	0.006319
8823	SYNPO2L	2.863245867	-0.991021314	0.321597	-3.081566	0.002059	0.004575
5210	SCAND1	880.7332908	-0.991817319	0.212213	-4.67368	2.96E-06	1.11E-05
1468	ZYX	3820.945542	-0.992074022	0.130259	-7.616187	2.61E-14	3.49E-13
4312	TRAF3IP3	156.5179066	-0.99311846	0.192831	-5.150193	2.60E-07	1.18E-06
8444	OR2W3	1.967127614	-0.993514705	0.307585	-3.230044	0.001238	0.002873
647	PPM1M	504.5480016	-0.993773155	0.108345	-9.172323	4.63E-20	1.41E-18
3890	HLA-DRA	28823.12947	-0.994163329	0.183826	-5.408183	6.37E-08	3.21E-07
3662	TLR2	706.3423528	-0.994671545	0.178863	-5.561082	2.68E-08	1.44E-07
13017	C11orf85	2.843721661	-0.995111803	0.607305	-1.63857	0.101303	0.152542
3569	RND3	1079.332345	-0.995726602	0.177045	-5.624147	1.86E-08	1.02E-07

6653	KIF2A	890.4988161	-0.995820713	0.251408	-3.960977	7.46E-05	0.00022
3262	C9orf100	131.2329289	-0.996214922	0.170575	-5.840335	5.21E-09	3.13E-08
2736	B3GAT3	843.033756	-0.996380335	0.159413	-6.250319	4.10E-10	2.93E-09
1414	PLEKHA9	83.37009362	-0.996382335	0.129681	-7.683316	1.55E-14	2.15E-13
10704	C10orf50	1.034165355	-0.996413719	0.415871	-2.395967	0.016577	0.030355
2972	C7orf13	174.8171392	-0.99655906	0.164568	-6.05561	1.40E-09	9.23E-09
7424	PDE11A	159.2895455	-0.996793979	0.273326	-3.646901	0.000265	0.000701
2919	TP53TG1	421.2516347	-0.996936192	0.163507	-6.097213	1.08E-09	7.25E-09
5120	OSMR	2880.914534	-0.997226022	0.211385	-4.717578	2.39E-06	9.14E-06
5321	RPL13AP6	7.53217659	-0.998296653	0.216455	-4.612023	3.99E-06	1.47E-05
1602	FAM110A	221.5001105	-0.998491576	0.134597	-7.418386	1.19E-13	1.45E-12
5951	C1orf21	691.8248254	-0.998656086	0.232787	-4.289999	1.79E-05	5.89E-05
3700	C11orf82	33.28489333	-0.999145537	0.18049	-5.535748	3.10E-08	1.64E-07
3951	CBX2	57.84304634	-0.999742938	0.18602	-5.374384	7.68E-08	3.81E-07
953	MBOAT7	1214.048533	-0.999792423	0.118448	-8.440768	3.15E-17	6.48E-16
9331	RPPH1	69.51174344	-1.000196973	0.34643	-2.887154	0.003887	0.008166
2316	RPS8	20963.87543	-1.000253374	0.150614	-6.641178	3.11E-11	2.63E-10
1607	POLD1	341.9924334	-1.000282364	0.134888	-7.415651	1.21E-13	1.48E-12
7728	E2F8	49.23118971	-1.000529024	0.283492	-3.529301	0.000417	0.001057
8193	CXCR2	36.31929008	-1.000596932	0.299932	-3.336084	0.00085	0.002033
6089	APOL4	190.2618182	-1.000754048	0.236814	-4.225915	2.38E-05	7.66E-05
13710	CEACAM5	0.81871246	-1.001386629	0.707762	-1.414864	0.157108	0.224616
7742	CTLA4	35.16009089	-1.001458365	0.284321	-3.522281	0.000428	0.001083
1844	CD151	9534.726154	-1.001515289	0.140157	-7.145654	8.96E-13	9.52E-12
5950	TLCD1	88.98254421	-1.001582193	0.233443	-4.290488	1.78E-05	5.87E-05
11810	HES7	2.334822257	-1.001739891	0.496433	-2.017876	0.043604	0.07237
9452	CCR9	2.972741843	-1.001998964	0.353206	-2.83687	0.004556	0.009448
6539	P2RY2	31.00621104	-1.002445867	0.250058	-4.008851	6.10E-05	0.000183
12635	VSTM1	1.466190748	-1.002489571	0.568638	-1.762965	0.077906	0.120858
7159	LOC38858	54.21167696	-1.002494415	0.266584	-3.760519	0.00017	0.000464
3437	LCP2	854.3610404	-1.002794514	0.175338	-5.719211	1.07E-08	6.10E-08
704	CKLF	349.963948	-1.003113776	0.111431	-9.00211	2.21E-19	6.16E-18
2065	NR1H3	838.1523847	-1.003304382	0.145777	-6.882446	5.88E-12	5.58E-11
1766	BUD31	1457.454895	-1.003440032	0.138867	-7.225904	4.98E-13	5.52E-12
8835	C8orf84	753.7854104	-1.003862778	0.326364	-3.075897	0.002099	0.004656
5208	CHSY3	133.9958068	-1.004045536	0.214803	-4.674252	2.95E-06	1.11E-05
2816	CDR2L	406.1909165	-1.004107349	0.162624	-6.174416	6.64E-10	4.62E-09
5631	OAZ3	20.23159351	-1.004350567	0.225417	-4.455514	8.37E-06	2.91E-05
10452	HSD17B3	17.44388191	-1.004431544	0.403048	-2.492087	0.0127	0.023816
6476	MND1	20.97023894	-1.004460119	0.248774	-4.037646	5.40E-05	0.000163
1304	LPPR2	517.5716674	-1.004639602	0.127881	-7.856037	3.96E-15	5.96E-14
8476	RAB3C	6.084886023	-1.004748252	0.312285	-3.217408	0.001294	0.002991
4387	AURKA	189.1303901	-1.004816096	0.196568	-5.111789	3.19E-07	1.43E-06
3112	ZNF581	448.705829	-1.005635821	0.168905	-5.95384	2.62E-09	1.65E-08
1697	RPL31	13720.40767	-1.006124505	0.137912	-7.295435	2.98E-13	3.44E-12
1816	TSTA3	891.1381239	-1.00767503	0.140482	-7.172996	7.34E-13	7.92E-12
3379	MAP3K8	205.794585	-1.007729738	0.174967	-5.759538	8.43E-09	4.89E-08

6602	CLEC10A	89.82799402	-1.007851199	0.253012	-3.983411	6.79E-05	0.000202
10122	LOC40080	1.303879752	-1.007920448	0.386406	-2.608448	0.009095	0.017613
7637	TNFSF9	212.3885422	-1.008052385	0.282684	-3.566009	0.000362	0.00093
1477	TARBP2	385.1367164	-1.008795349	0.132728	-7.600448	2.95E-14	3.92E-13
5675	DPM3	340.4488935	-1.008883065	0.227501	-4.434622	9.22E-06	3.19E-05
10957	KCNK4	2.196569011	-1.008993134	0.438449	-2.301276	0.021376	0.038242
3756	C21orf58	39.48551206	-1.009261244	0.183391	-5.503336	3.73E-08	1.94E-07
13322	GJD4	0.442083309	-1.009338646	0.657238	-1.535728	0.124605	0.183335
520	MYO9B	1805.814604	-1.009654435	0.105833	-9.540103	1.43E-21	5.38E-20
9053	SCTR	32.17378816	-1.009921795	0.337139	-2.995563	0.002739	0.005931
8547	DDX11L2	16.96672013	-1.010208555	0.316568	-3.191122	0.001417	0.00325
1349	RPL27A	13457.77257	-1.010845013	0.129587	-7.80052	6.17E-15	8.96E-14
212	AVEN	181.5817564	-1.011267935	0.091232	-11.08461	1.49E-28	1.38E-26
14145	C6orf218	0.302535605	-1.011316834	0.794439	-1.272994	0.20302	0.281329
4212	KIF21B	161.4037145	-1.011655822	0.194446	-5.202757	1.96E-07	9.14E-07
3806	CACNB3	320.6825263	-1.011764341	0.184966	-5.47001	4.50E-08	2.32E-07
9531	HBB	2770.099066	-1.012330484	0.360764	-2.806075	0.005015	0.010313
7840	LPHN3	97.32092888	-1.01285416	0.291137	-3.478964	0.000503	0.001258
2109	RPL32	12429.4544	-1.0129136	0.148091	-6.839783	7.93E-12	7.37E-11
10617	PRPH	23.76925154	-1.01304828	0.416957	-2.429626	0.015114	0.027904
4632	LYPD5	40.30337215	-1.013087307	0.203695	-4.973553	6.57E-07	2.78E-06
5933	PRR16	69.44621701	-1.013914149	0.235677	-4.302141	1.69E-05	5.59E-05
5773	NXN	449.8751084	-1.014036721	0.231308	-4.383923	1.17E-05	3.96E-05
8597	HTR1B	3.559415576	-1.014327405	0.319394	-3.175785	0.001494	0.003407
3537	MGC12982	82.99398607	-1.014357107	0.179747	-5.643235	1.67E-08	9.25E-08
3811	TMC6	513.5488331	-1.014440545	0.185566	-5.46675	4.58E-08	2.36E-07
5583	KIRREL	524.5428055	-1.014456113	0.226377	-4.481266	7.42E-06	2.61E-05
7696	FASLG	49.83735286	-1.014820258	0.28654	-3.541632	0.000398	0.001013
7835	ODF3L2	4.221026854	-1.01533965	0.291518	-3.482938	0.000496	0.001241
2102	RPL18A	1960.41427	-1.01551033	0.148299	-6.847732	7.50E-12	7.00E-11
8772	ZMYND10	58.57074859	-1.015550303	0.328105	-3.095199	0.001967	0.004395
13686	CDH10	0.865550593	-1.015632733	0.713322	-1.423807	0.154502	0.221277
6879	OTOA	30.05961464	-1.016196036	0.262468	-3.871688	0.000108	0.000308
5607	FBXL8	120.9371424	-1.016696042	0.227563	-4.467749	7.90E-06	2.76E-05
1616	ORAI2	1158.671793	-1.017423777	0.137371	-7.406407	1.30E-13	1.57E-12
4810	CPT1C	68.77412452	-1.017724529	0.208388	-4.883792	1.04E-06	4.24E-06
7658	EFCAB4A	389.558099	-1.017905103	0.286218	-3.556402	0.000376	0.000962
3685	LOC33952	43.6497953	-1.017997373	0.183525	-5.546911	2.91E-08	1.55E-07
4382	RASGRP4	62.31543594	-1.018269116	0.199123	-5.113782	3.16E-07	1.41E-06
13838	RFX4	0.344545153	-1.018994311	0.742764	-1.371894	0.170096	0.240935
2123	SNHG6	1005.602326	-1.019409443	0.149338	-6.826208	8.72E-12	8.05E-11
3470	OPN3	716.0729145	-1.019708664	0.179056	-5.694916	1.23E-08	6.97E-08
6442	ZNF469	139.3159031	-1.020006964	0.251633	-4.053542	5.04E-05	0.000154
1570	TUBA1C	4687.03873	-1.020207333	0.1366	-7.468563	8.11E-14	1.01E-12
6656	HIST2H2AA	503.3570149	-1.020384446	0.257665	-3.960117	7.49E-05	0.000221
7142	PCDHGC4	9.97392677	-1.020566213	0.270963	-3.766436	0.000166	0.000454
10700	CRNA001	4.238551934	-1.021352853	0.425989	-2.397605	0.016503	0.030231

8041	C9orf139	7.181283099	-1.021438294	0.300787	-3.395883	0.000684	0.001668
8509	WNT2B	42.05893138	-1.022388345	0.319303	-3.201933	0.001365	0.003145
3060	TGFB1I1	624.203315	-1.022526057	0.170651	-5.991901	2.07E-09	1.33E-08
2013	C17orf49	861.9678501	-1.022535011	0.147293	-6.942168	3.86E-12	3.76E-11
10289	SYNC	238.5793255	-1.02309211	0.401209	-2.550024	0.010772	0.020519
8882	TNFRSF9	56.37152107	-1.023389483	0.334567	-3.058845	0.002222	0.004903
13902	MORC1	0.36295556	-1.023457064	0.759191	-1.348089	0.17763	0.250429
5356	GRAP2	45.50599417	-1.023819007	0.223006	-4.590994	4.41E-06	1.61E-05
3675	TIMM16	179.2686728	-1.024271435	0.184436	-5.553523	2.80E-08	1.49E-07
2261	CLTB	1262.168872	-1.024517549	0.153197	-6.687563	2.27E-11	1.97E-10
5917	DAPP1	91.01185749	-1.024561484	0.237518	-4.313618	1.61E-05	5.32E-05
4691	)C1001342	9.095909284	-1.024678355	0.207704	-4.933363	8.08E-07	3.38E-06
4971	THBD	1212.68331	-1.025046487	0.21405	-4.788823	1.68E-06	6.62E-06
5196	GDPD5	244.5177202	-1.025490368	0.21917	-4.678974	2.88E-06	1.09E-05
4337	VSIG10L	71.02576687	-1.025660249	0.19963	-5.137813	2.78E-07	1.26E-06
4178	MEX3A	115.5540094	-1.025804085	0.196208	-5.228153	1.71E-07	8.03E-07
3804	IDUA	240.1298071	-1.025936957	0.187538	-5.470565	4.49E-08	2.31E-07
3944	PTRF	9046.62919	-1.02595803	0.190775	-5.377834	7.54E-08	3.75E-07
9061	C4orf6	40.07805424	-1.025999245	0.342664	-2.994183	0.002752	0.005953
5761	CABYR	35.37873306	-1.026247498	0.233683	-4.391622	1.13E-05	3.83E-05
1285	C20orf27	551.1586009	-1.026467292	0.130263	-7.879954	3.28E-15	5.00E-14
2815	GBGT1	198.8926054	-1.026794526	0.166294	-6.17458	6.63E-10	4.62E-09
7240	C9orf140	99.51117138	-1.027715177	0.275612	-3.728852	0.000192	0.000521
3350	SLMO1	36.07539689	-1.028118187	0.177853	-5.780707	7.44E-09	4.35E-08
487	PFN1	10879.4489	-1.028643187	0.106385	-9.669063	4.08E-22	1.64E-20
8684	C2orf58	17.02961525	-1.028648946	0.327863	-3.137431	0.001704	0.003847
12735	CRNA0018	10.05615866	-1.028649574	0.597355	-1.722007	0.085068	0.130932
8245	)C1001308	114.1937194	-1.028818309	0.310426	-3.314219	0.000919	0.002185
4975	GNAZ	150.8797282	-1.028890444	0.214985	-4.785871	1.70E-06	6.71E-06
2386	RPL36	7647.58728	-1.028981364	0.156586	-6.571338	4.99E-11	4.10E-10
5461	MGP	6068.796754	-1.029039799	0.226791	-4.537392	5.70E-06	2.04E-05
2835	ARHGAP15	179.0305094	-1.029329999	0.167168	-6.157453	7.39E-10	5.11E-09
8109	PNMA5	1.843980983	-1.029486289	0.305705	-3.367583	0.000758	0.001833
15256	MYH16	0.279724193	-1.029554057	1.105061	-0.931672	0.351506	0.451617
5883	RHGAP11	11.05187336	-1.029760392	0.23775	-4.331267	1.48E-05	4.94E-05
2359	STIL	102.6121532	-1.030328408	0.156103	-6.600321	4.10E-11	3.41E-10
11070	C15orf53	0.821454134	-1.030420927	0.454692	-2.266194	0.023439	0.041503
10851	BAI1	52.02107894	-1.030693048	0.440669	-2.338926	0.019339	0.034934
3173	EFEMP2	2321.694755	-1.030892124	0.174658	-5.902354	3.58E-09	2.21E-08
15369	FAM75A3	0.243759316	-1.031420644	1.146746	-0.899433	0.368422	0.469871
6397	LILRA5	73.58198231	-1.031682323	0.25307	-4.076663	4.57E-05	0.00014
16764	HCRT	0.198950828	-1.031691086	2.064694	-0.499682	0.617299	0.721765
16560	KRT3	0.195785432	-1.031691364	1.854341	-0.556365	0.577961	0.684095
16384	OR4N2	0.201761599	-1.031691456	1.708111	-0.603996	0.545847	0.653024
16389	GGNBP1	0.238940418	-1.031756224	1.710981	-0.60302	0.546495	0.65356
2598	RPSA	4741.595063	-1.032054138	0.161708	-6.382219	1.75E-10	1.32E-09
2814	APOBEC3D	158.6370218	-1.0322367	0.167175	-6.174579	6.63E-10	4.62E-09

13845	OR2H2	0.275036357	-1.032503336	0.75404	-1.369295	0.170907	0.241961
7436	GJC2	59.55021747	-1.032738256	0.283583	-3.641745	0.000271	0.000714
5781	TNFSF8	76.94810747	-1.033452791	0.235858	-4.381669	1.18E-05	3.99E-05
7555	PDZD4	70.17913986	-1.033601497	0.287656	-3.593189	0.000327	0.000847
8051	DOK5	104.3898766	-1.033737784	0.304787	-3.391678	0.000695	0.001691
11572	TEKT5	0.750260254	-1.034099982	0.494338	-2.091889	0.036448	0.061737
7438	CRTAM	57.31036	-1.034127138	0.28401	-3.641164	0.000271	0.000715
7928	C7orf51	7.476398142	-1.034226779	0.300841	-3.437785	0.000586	0.00145
5168	C16orf93	44.93642226	-1.034750077	0.220599	-4.690637	2.72E-06	1.03E-05
5982	SLC39A4	312.3541518	-1.034847569	0.241992	-4.276367	1.90E-05	6.22E-05
5630	CD40LG	34.76737944	-1.034963461	0.232286	-4.455555	8.37E-06	2.91E-05
1604	DRAP1	1241.079997	-1.035017712	0.139536	-7.417561	1.19E-13	1.46E-12
7936	IGF2	536.4011288	-1.035875479	0.301728	-3.433141	0.000597	0.001474
7221	FBLN5	1129.319911	-1.036850177	0.277439	-3.737225	0.000186	0.000505
243	RFC2	631.144692	-1.037238926	0.094981	-10.92053	9.20E-28	7.42E-26
2221	C11orf48	550.2075823	-1.037295683	0.154095	-6.731534	1.68E-11	1.48E-10
3401	LRRC41	3187.785222	-1.037677554	0.180522	-5.748216	9.02E-09	5.20E-08
1269	CDK5	369.4904991	-1.037824417	0.131337	-7.901998	2.74E-15	4.24E-14
1195	MOSPD3	358.4494952	-1.038167512	0.12945	-8.01981	1.06E-15	1.74E-14
12862	UGT1A5	2.606429319	-1.038492788	0.614745	-1.689305	0.091161	0.138924
6497	LRRC46	25.74356034	-1.039600993	0.257967	-4.029977	5.58E-05	0.000168
4773	CHST3	1054.827613	-1.041404844	0.212663	-4.89698	9.73E-07	4.00E-06
5556	IER5L	466.8365553	-1.041672065	0.231745	-4.49491	6.96E-06	2.46E-05
4849	KIF11	231.932654	-1.041849876	0.214076	-4.866735	1.13E-06	4.59E-06
15868	SLCO1B3	0.341768138	-1.042087191	1.383001	-0.753497	0.451151	0.557286
3004	MAD2L2	418.6735029	-1.042111742	0.172786	-6.031224	1.63E-09	1.06E-08
991	RHOC	6241.49476	-1.042283596	0.124692	-8.358853	6.33E-17	1.25E-15
11818	FAM83C	1.122316876	-1.042490762	0.517345	-2.01508	0.043896	0.072801
3122	ATF5	1286.26817	-1.042499372	0.175298	-5.947029	2.73E-09	1.71E-08
11882	LOC14547	0.683510524	-1.042667594	0.522717	-1.994708	0.046075	0.076007
7660	SH2D5	44.27292637	-1.043508878	0.293595	-3.554244	0.000379	0.00097
10442	SLC5A5	1.736292607	-1.043568803	0.417922	-2.49704	0.012523	0.023508
3041	EVL	1814.757468	-1.043869148	0.173843	-6.004661	1.92E-09	1.24E-08
3913	TTC39C	214.6192792	-1.044383573	0.193583	-5.39501	6.85E-08	3.43E-07
1308	SH3GL1	2077.154343	-1.044431185	0.133037	-7.850654	4.14E-15	6.20E-14
1229	DDX41	2360.704314	-1.04486336	0.13102	-7.974834	1.53E-15	2.43E-14
4353	ELL2	2202.893238	-1.04526715	0.203782	-5.12935	2.91E-07	1.31E-06
12027	FABP5L3	0.472583226	-1.045606484	0.535122	-1.953959	0.050706	0.082638
3784	THY1	6002.928111	-1.045805605	0.190761	-5.482282	4.20E-08	2.17E-07
9688	TSGA10IP	1.091672193	-1.046207687	0.380489	-2.74964	0.005966	0.012071
6671	KCNIP2	28.45822012	-1.046463053	0.264655	-3.954072	7.68E-05	0.000226
1900	DTYMK	399.1863555	-1.047006132	0.147733	-7.087153	1.37E-12	1.41E-11
2537	RAB34	1511.695208	-1.047358091	0.162809	-6.433067	1.25E-10	9.66E-10
8551	OLAH	2.579699464	-1.04749714	0.328478	-3.18894	0.001428	0.003273
2436	NRM	417.9119291	-1.047760529	0.160583	-6.524714	6.81E-11	5.48E-10
13923	MAS1L	0.386520442	-1.047844746	0.780432	-1.342647	0.179386	0.252524
1077	POP7	497.3849729	-1.047865519	0.127859	-8.195468	2.50E-16	4.54E-15

2872	RIMBP3	35.87826283	-1.047884756	0.171005	-6.127795	8.91E-10	6.08E-09
5550	IGSF6	212.2923052	-1.04841355	0.233029	-4.499072	6.83E-06	2.41E-05
1942	BLVRB	1391.76744	-1.048443361	0.148899	-7.041283	1.90E-12	1.92E-11
1977	SHFM1	1833.602665	-1.04921189	0.150036	-6.99306	2.69E-12	2.67E-11
11086	C17orf66	1.187148995	-1.04960754	0.464294	-2.260654	0.023781	0.042046
11135	UPK1A	0.953092906	-1.04997974	0.468425	-2.241512	0.024993	0.044
5217	PTPRU	784.688221	-1.050584931	0.22487	-4.671955	2.98E-06	1.12E-05
6416	SLC13A4	4.578773959	-1.051204822	0.258289	-4.069876	4.70E-05	0.000144
4655	CD48	465.322612	-1.05137103	0.212011	-4.959041	7.08E-07	2.98E-06
10820	KCNH8	5.541888402	-1.05157424	0.446621	-2.354513	0.018547	0.033601
5026	EVI2A	508.2322417	-1.051742061	0.220902	-4.761127	1.93E-06	7.51E-06
4432	CD247	188.1778448	-1.052057002	0.207003	-5.082335	3.73E-07	1.65E-06
5358	C1QTNF2	23.7269684	-1.052113976	0.229352	-4.587341	4.49E-06	1.64E-05
14632	GP2	0.720116122	-1.052405624	0.937712	-1.122312	0.26173	0.350612
4689	COLQ	57.05420464	-1.05344792	0.213518	-4.933767	8.07E-07	3.37E-06
15444	BEST2	0.313007447	-1.053705802	1.204191	-0.875032	0.381556	0.484288
6302	LOC80054	21.77205449	-1.053928759	0.255447	-4.125828	3.69E-05	0.000115
8156	PFKFB1	1.544475492	-1.053948254	0.314754	-3.348483	0.000813	0.001953
11739	C15orf2	1.39257099	-1.053978596	0.516342	-2.041241	0.041227	0.068838
1242	FBL	2339.153224	-1.053981257	0.132772	-7.938293	2.05E-15	3.23E-14
6123	S100Z	4.405902404	-1.05427155	0.250382	-4.210648	2.55E-05	8.15E-05
9729	ALK	5.459820632	-1.054810629	0.385181	-2.738484	0.006172	0.012435
2096	UCK2	213.7914526	-1.054963455	0.15396	-6.8522	7.27E-12	6.80E-11
1483	PLXNA1	1306.957699	-1.055010851	0.138899	-7.595517	3.07E-14	4.05E-13
7241	GAMT	1234.032552	-1.055085512	0.282963	-3.728711	0.000192	0.000521
4616	C14orf80	98.43766968	-1.055370899	0.211844	-4.981837	6.30E-07	2.67E-06
4779	FGD2	244.5926046	-1.055822081	0.215667	-4.895624	9.80E-07	4.02E-06
3409	BTK	224.4335687	-1.057063234	0.184124	-5.741041	9.41E-09	5.41E-08
6870	SH3D20	9.535683082	-1.05713269	0.272751	-3.875817	0.000106	0.000303
5856	KIR2DL4	9.366518708	-1.057141418	0.243449	-4.34235	1.41E-05	4.72E-05
7913	KLRC1	12.5912116	-1.057195963	0.307004	-3.443587	0.000574	0.001422
3845	DPEP2	91.0717122	-1.057870122	0.194621	-5.435552	5.46E-08	2.78E-07
6585	SRCIN1	54.95460152	-1.058038035	0.265197	-3.989635	6.62E-05	0.000197
2837	RPS9	15007.98587	-1.058143828	0.17194	-6.154162	7.55E-10	5.21E-09
12622	C18orf2	0.627412389	-1.058151216	0.599364	-1.765458	0.077487	0.120331
1903	SNRPD2	2602.768516	-1.058545984	0.149428	-7.083968	1.40E-12	1.44E-11
5906	HLA-DRB6	699.2485943	-1.058582532	0.245102	-4.318953	1.57E-05	5.20E-05
5702	SOD3	673.9357835	-1.058601926	0.23953	-4.419498	9.89E-06	3.40E-05
2091	SEC61G	1558.786246	-1.058626529	0.154441	-6.854562	7.15E-12	6.71E-11
8465	MRGPRF	82.03937125	-1.058870999	0.328793	-3.22048	0.00128	0.002963
11632	HPD	222.6843485	-1.059313961	0.512241	-2.068	0.03864	0.065112
7591	SCARNA2	7.439254845	-1.059360642	0.295692	-3.582645	0.00034	0.000878
7918	CD8A	726.624115	-1.05947253	0.307875	-3.44124	0.000579	0.001433
11786	DNER	253.5250252	-1.059591297	0.52277	-2.026878	0.042675	0.070966
2669	ZMYND15	100.8612646	-1.059591975	0.167869	-6.312012	2.75E-10	2.02E-09
2668	MARVELD	720.3716522	-1.059947898	0.167858	-6.314569	2.71E-10	1.99E-09
9420	MRAP2	9.695047202	-1.060211312	0.371916	-2.850672	0.004363	0.009078

2219	SAC3D1	110.017215	-1.06032137	0.157405	-6.736277	1.62E-11	1.44E-10
13982	SCGB2A2	0.991561847	-1.061161644	0.801559	-1.323873	0.185545	0.260111
2760	PSMC3IP	72.1490957	-1.061584143	0.170355	-6.231604	4.62E-10	3.28E-09
11286	CYP2C9	35.8679886	-1.061638766	0.48629	-2.183139	0.029026	0.05041
7202	SCN8A	41.0300184	-1.061670845	0.283475	-3.7452	0.00018	0.000491
13929	SLITRK3	0.531102808	-1.062153384	0.791775	-1.341484	0.179764	0.252965
8922	RACGAP1I	1.229185765	-1.062693435	0.349168	-3.043504	0.002338	0.005137
4728	KIAA0101	126.2761468	-1.062968834	0.216102	-4.918836	8.71E-07	3.61E-06
1853	BCL7C	672.6733938	-1.063670504	0.149109	-7.133515	9.78E-13	1.03E-11
1966	NLGN2	448.3682363	-1.06371009	0.151682	-7.012754	2.34E-12	2.33E-11
6349	CLECL1	15.68319707	-1.064302524	0.259311	-4.104344	4.05E-05	0.000125
6926	CCL4L2	173.8063822	-1.064624577	0.276231	-3.854105	0.000116	0.000329
1909	KRTCAP2	1850.295326	-1.064915369	0.150456	-7.077897	1.46E-12	1.50E-11
5698	OC1001348	5.381826797	-1.065122302	0.240919	-4.421085	9.82E-06	3.38E-05
6741	TIPARP	2298.127127	-1.065290543	0.271444	-3.924527	8.69E-05	0.000253
7786	RET	30.49978089	-1.065462472	0.303962	-3.505248	0.000456	0.001148
5119	HSF2BP	12.61194894	-1.065478212	0.225813	-4.718408	2.38E-06	9.10E-06
12073	GJB4	10.52634364	-1.06555088	0.550237	-1.936531	0.052803	0.085727
7411	BNIPL	8.099227739	-1.065631772	0.291879	-3.650935	0.000261	0.000691
10578	CCL20	673.6454674	-1.065661243	0.436295	-2.442526	0.014585	0.027026
10875	NCAM1	800.2386859	-1.065783868	0.457239	-2.330913	0.019758	0.035612
2776	IFI16	3090.880422	-1.066080511	0.171524	-6.215328	5.12E-10	3.62E-09
6645	IGFBP3	58607.86505	-1.066782899	0.269007	-3.96564	7.32E-05	0.000216
4678	MVK	797.9037865	-1.067144636	0.215795	-4.94517	7.61E-07	3.19E-06
1377	TMED3	1701.910114	-1.067381685	0.137597	-7.757279	8.68E-15	1.24E-13
3829	GPX8	1136.656016	-1.067535145	0.195968	-5.447484	5.11E-08	2.62E-07
3724	APOBEC3C	673.745862	-1.068514291	0.193447	-5.523546	3.32E-08	1.75E-07
7329	PABPC1L	371.3450354	-1.068695796	0.2897	-3.688972	0.000225	0.000602
2387	NUDT17	47.15987317	-1.068698181	0.162659	-6.570186	5.03E-11	4.13E-10
8507	SLC2A5	2218.319244	-1.069115735	0.333828	-3.202598	0.001362	0.003138
2572	EXOSC5	421.8432857	-1.069423963	0.166859	-6.409154	1.46E-10	1.12E-09
2754	COL4A2	23115.09356	-1.069455918	0.171466	-6.237138	4.46E-10	3.17E-09
7490	GRID1	72.32379167	-1.06982606	0.295522	-3.620118	0.000294	0.000771
4346	XRCC2	26.8731305	-1.070332504	0.208492	-5.133694	2.84E-07	1.28E-06
3634	PIK3R5	361.5796499	-1.070984891	0.191876	-5.581639	2.38E-08	1.29E-07
5577	C13orf33	204.4785795	-1.071049085	0.238784	-4.485433	7.28E-06	2.56E-05
6079	CYTL1	37.97197866	-1.071161931	0.253247	-4.229708	2.34E-05	7.54E-05
10646	CLCN1	1.443251612	-1.071636388	0.443752	-2.414946	0.015738	0.028973
1805	CRELD2	637.8909892	-1.072117999	0.14923	-7.184342	6.75E-13	7.33E-12
14202	ANKRD26P	0.346535667	-1.072573907	0.854008	-1.25593	0.209141	0.288648
9292	INA	2.126019882	-1.072915217	0.369666	-2.902392	0.003703	0.007812
7600	S100A2	98.71771987	-1.073078778	0.299775	-3.579608	0.000344	0.000887
2646	MAP3K12	282.8036986	-1.073120451	0.169608	-6.327058	2.50E-10	1.85E-09
4584	PTGIR	109.761145	-1.073695455	0.215034	-4.993147	5.94E-07	2.54E-06
5204	SLC39A14	8361.966571	-1.073708423	0.229618	-4.676065	2.92E-06	1.10E-05
4032	CDC6	123.4637232	-1.074059348	0.202021	-5.316585	1.06E-07	5.14E-07
6205	NUDT8	187.1850516	-1.074376689	0.257369	-4.174466	2.99E-05	9.44E-05

540	CASP4	1177.48602	-1.074859832	0.113239	-9.491967	2.27E-21	8.23E-20
8290	LAMC2	329.7964321	-1.07544197	0.32613	-3.297582	0.000975	0.002306
5692	WNT5A	196.8328187	-1.075681546	0.243098	-4.424895	9.65E-06	3.32E-05
5621	MICALCL	7.754089468	-1.076112431	0.241199	-4.46152	8.14E-06	2.84E-05
2068	PTP4A3	1077.149674	-1.076425351	0.156416	-6.881833	5.91E-12	5.60E-11
4941	PMEPA1	3231.390802	-1.076731403	0.22412	-4.804262	1.55E-06	6.16E-06
5285	SNAI2	342.9642066	-1.076793478	0.232469	-4.631992	3.62E-06	1.34E-05
6108	IER3	1912.606442	-1.076841684	0.255299	-4.217962	2.47E-05	7.91E-05
4750	GPBAR1	27.78820198	-1.076910399	0.219385	-4.908765	9.17E-07	3.78E-06
10403	KCNG1	27.87105708	-1.077096381	0.429493	-2.507833	0.012147	0.022888
1459	INPP5D	936.0754026	-1.077581401	0.141301	-7.626163	2.42E-14	3.25E-13
6404	LOC388242	35.98472978	-1.079217042	0.264821	-4.075274	4.60E-05	0.000141
7322	C1orf70	8.05005643	-1.079861523	0.29257	-3.690949	0.000223	0.000598
7223	TXNDC3	7.500457723	-1.080066763	0.289039	-3.736757	0.000186	0.000506
1506	GPR172A	834.1873224	-1.080546609	0.142998	-7.556378	4.14E-14	5.39E-13
8490	CHST6	8.525186044	-1.080688668	0.33649	-3.21165	0.00132	0.003047
5024	HLX	434.1086071	-1.080703432	0.226976	-4.761315	1.92E-06	7.50E-06
10244	TBX1	5.512688163	-1.081198413	0.421115	-2.567464	0.010245	0.019602
3454	DCBLD2	1620.931136	-1.081680066	0.189588	-5.705434	1.16E-08	6.59E-08
400	PPIH	280.0668234	-1.081799936	0.107952	-10.02109	1.23E-23	6.03E-22
5462	MYL4	10.90041414	-1.081928569	0.238462	-4.537106	5.70E-06	2.05E-05
4441	ZNF703	70.98097012	-1.081937004	0.213056	-5.07819	3.81E-07	1.68E-06
1735	ST3GAL3	356.5553317	-1.082505682	0.149087	-7.26091	3.84E-13	4.34E-12
2949	ROMO1	874.0951678	-1.082691233	0.178344	-6.070791	1.27E-09	8.46E-09
2508	ROBLD3	884.7652474	-1.082833015	0.16772	-6.456194	1.07E-10	8.39E-10
4372	P2RY11	128.4804441	-1.082977818	0.211581	-5.118507	3.08E-07	1.38E-06
5573	KIF18A	68.24394613	-1.084209631	0.24161	-4.487438	7.21E-06	2.54E-05
7546	KLRC2	7.244547661	-1.084836872	0.301603	-3.5969	0.000322	0.000836
14610	MTVR2	0.244830447	-1.084840785	0.959742	-1.130347	0.25833	0.34658
10647	EIF3IP1	0.588057717	-1.084880657	0.449235	-2.41495	0.015737	0.028973
9342	ANKRD1	27.8112005	-1.085088955	0.376558	-2.8816	0.003957	0.008302
2454	OPRL1	60.45585862	-1.086024836	0.166831	-6.509748	7.53E-11	6.01E-10
1849	PSMB3	1944.874364	-1.086225325	0.152174	-7.138053	9.47E-13	1.00E-11
1895	CALU	5632.031287	-1.086327382	0.153197	-7.091054	1.33E-12	1.38E-11
8021	GABRE	345.4191509	-1.087180476	0.31938	-3.404036	0.000664	0.001623
5614	FLJ42289	7.982049939	-1.087736771	0.243566	-4.465886	7.97E-06	2.78E-05
2163	RPL18	13478.23514	-1.088054103	0.160048	-6.798291	1.06E-11	9.59E-11
2213	LMAN2	5635.952045	-1.088271854	0.161413	-6.742154	1.56E-11	1.38E-10
6757	GPM6B	66.99027577	-1.088433208	0.27791	-3.916489	8.98E-05	0.000261
4377	GPR18	18.89642611	-1.08870653	0.212807	-5.115941	3.12E-07	1.40E-06
17272	GAGE2E	0.197226747	-1.089396724	2.91407	-0.37384	0.708523	0.804062
4363	ORC6L	56.88546565	-1.089451814	0.212612	-5.124135	2.99E-07	1.34E-06
14755	LRTM2	0.232314136	-1.089457265	1.006765	-1.082136	0.279192	0.370887
14662	FGF8	0.300898863	-1.089710336	0.979283	-1.112763	0.26581	0.35535
2925	CENPP	69.43741883	-1.089882301	0.178873	-6.093057	1.11E-09	7.42E-09
14171	HULC	0.271289149	-1.090167303	0.861752	-1.26506	0.20585	0.284727
913	MPZL1	3510.072054	-1.090442434	0.128359	-8.495285	1.97E-17	4.24E-16

16051	RLBP1	0.251260597	-1.090560871	1.564186	-0.697207	0.485673	0.59309
1412	MAD1L1	428.1724448	-1.09090115	0.141953	-7.684958	1.53E-14	2.12E-13
3759	PPL	776.2538847	-1.091111869	0.198362	-5.500611	3.78E-08	1.97E-07
5964	ETV2	11.43170675	-1.091434557	0.254706	-4.285083	1.83E-05	6.00E-05
8739	CLEC4D	6.984730209	-1.092558844	0.351018	-3.112541	0.001855	0.00416
3730	CARD16	296.949907	-1.092816669	0.198016	-5.518823	3.41E-08	1.79E-07
8890	FZD10	18.81840508	-1.093121756	0.357673	-3.056205	0.002242	0.004942
14565	PDYN	0.312754467	-1.093427722	0.953182	-1.147134	0.251326	0.338225
6796	CACNA2D	23.44109491	-1.094024713	0.280457	-3.900867	9.58E-05	0.000276
13021	SIX3	1.162554605	-1.094692143	0.66874	-1.636947	0.101642	0.153005
5482	C11orf45	69.40821088	-1.094915457	0.24172	-4.529685	5.91E-06	2.11E-05
7264	IL7R	162.2216531	-1.095309088	0.294529	-3.718844	0.0002	0.00054
5545	EMR2	395.9850408	-1.095329931	0.243387	-4.500367	6.78E-06	2.40E-05
8382	SERPIND1	3.012775907	-1.095378446	0.336216	-3.257965	0.001122	0.002624
11234	C12orf70	1.189107676	-1.095626647	0.496582	-2.206336	0.02736	0.047738
2063	ANXA2P1	50.17989361	-1.096657944	0.159282	-6.88501	5.78E-12	5.49E-11
8188	MEOX1	15.20097732	-1.096743107	0.328487	-3.338773	0.000841	0.002014
10008	FBXW10	1.294127693	-1.096793582	0.413823	-2.650391	0.00804	0.015746
12729	DRD5	0.418122423	-1.097334502	0.636774	-1.723273	0.084839	0.130631
985	ALG3	823.8367239	-1.097423122	0.131166	-8.366691	5.93E-17	1.18E-15
6155	DEPDC1B	55.32353156	-1.097592407	0.26132	-4.200182	2.67E-05	8.49E-05
9547	CPNE9	3.159169341	-1.097670276	0.39173	-2.802109	0.005077	0.010424
9833	LOC40146	1.666477402	-1.098400182	0.405575	-2.708252	0.006764	0.013483
9611	CLEC6A	2.051447094	-1.099371274	0.396121	-2.775344	0.005514	0.011246
4885	IFI30	5225.104996	-1.099790359	0.227444	-4.835436	1.33E-06	5.33E-06
6867	AQPEP	4.438623407	-1.099872328	0.283749	-3.876209	0.000106	0.000303
450	TMEM189	874.5490963	-1.100204824	0.112228	-9.803303	1.09E-22	4.75E-21
9889	NLRP7	4.044309259	-1.100741179	0.409038	-2.691047	0.007123	0.014118
4436	GAS7	403.8448539	-1.100806841	0.216672	-5.080519	3.76E-07	1.66E-06
8409	HSP90AB4	2.099194493	-1.101161512	0.339197	-3.246381	0.001169	0.002724
9970	FBLL1	4.997497788	-1.101647224	0.41367	-2.663103	0.007742	0.015221
7576	REEP6	86.3336864	-1.101797798	0.307135	-3.58734	0.000334	0.000864
2791	CSF1	921.4609606	-1.102220824	0.177795	-6.199385	5.67E-10	3.98E-09
3947	HSPA6	252.529742	-1.10260288	0.205056	-5.377095	7.57E-08	3.76E-07
2531	ADCY7	623.1839927	-1.102772822	0.171282	-6.438331	1.21E-10	9.35E-10
3812	NAIP	97.74659697	-1.102779208	0.201775	-5.465378	4.62E-08	2.38E-07
5793	DUSP2	180.9499159	-1.102850566	0.251971	-4.37689	1.20E-05	4.07E-05
255	RHOG	1281.95603	-1.102970687	0.102008	-10.81261	3.00E-27	2.31E-25
8708	VAX2	8.025583443	-1.103230123	0.352797	-3.127092	0.001765	0.003974
4748	TLR6	41.347098	-1.103626177	0.224797	-4.909426	9.13E-07	3.77E-06
6153	TCF15	18.72706119	-1.104284484	0.262809	-4.201845	2.65E-05	8.43E-05
11240	WISP3	2.086396017	-1.104470088	0.501182	-2.203733	0.027543	0.048031
1777	GIYD2	616.2439459	-1.104565226	0.153237	-7.208228	5.67E-13	6.25E-12
5280	SIGLEC5	44.1751662	-1.105274148	0.238432	-4.635595	3.56E-06	1.32E-05
2307	BFSP1	31.45786813	-1.105288381	0.166251	-6.648312	2.96E-11	2.52E-10
14683	CST4	0.451954281	-1.105368589	0.998081	-1.107494	0.26808	0.357884
5914	ADAMTS2	265.0801877	-1.105712499	0.256191	-4.315972	1.59E-05	5.27E-05

4105	SPATA9	6.197589843	-1.106123264	0.209533	-5.278982	1.30E-07	6.20E-07
3557	NLRP3	128.1228	-1.106576572	0.196573	-5.62933	1.81E-08	9.97E-08
8266	PAK3	13.19256788	-1.106762167	0.33482	-3.305545	0.000948	0.002248
5032	ENOX1	26.64540617	-1.107225464	0.232663	-4.758933	1.95E-06	7.58E-06
384	HM13	3934.21161	-1.107436061	0.109505	-10.11311	4.83E-24	2.47E-22
11305	GRIA1	14.69110624	-1.108138907	0.508933	-2.177378	0.029452	0.051066
1516	LOC65036{8}	35.43545095	-1.108356689	0.146936	-7.543151	4.59E-14	5.93E-13
13625	GAL	1.003200688	-1.108555515	0.765918	-1.447356	0.147797	0.212622
7878	DUSP27	3.974148145	-1.109217654	0.320594	-3.459883	0.00054	0.001345
9853	UPK3A	2.569743511	-1.10973129	0.41071	-2.701984	0.006893	0.013712
439	BID	495.9587057	-1.109750274	0.112555	-9.859612	6.23E-23	2.78E-21
6559	TMEM52	10.56309299	-1.11025495	0.277435	-4.001863	6.28E-05	0.000188
1135	ADAP2	527.4955579	-1.110464751	0.13693	-8.109702	5.07E-16	8.76E-15
5940	ZAP70	152.0437817	-1.110702941	0.258434	-4.297812	1.72E-05	5.69E-05
14115	SCN10A	0.357095514	-1.111457671	0.866848	-1.282183	0.199779	0.277425
727	TAX1BP3	2150.775158	-1.111937154	0.124277	-8.947212	3.65E-19	9.83E-18
7877	ABCA4	12.1678148	-1.112022935	0.321268	-3.461357	0.000537	0.001337
3915	CCNA2	179.1295495	-1.112118321	0.206193	-5.393585	6.91E-08	3.46E-07
1712	PGLS	1173.98815	-1.112437352	0.152705	-7.284889	3.22E-13	3.68E-12
3327	FKBP1B	56.23148175	-1.112860503	0.191938	-5.798032	6.71E-09	3.95E-08
11373	MYBPH	1.091873671	-1.112880384	0.515198	-2.160104	0.030765	0.053022
7102	PHKG1	6.81935612	-1.113534954	0.294396	-3.782436	0.000155	0.000429
992	REEP4	338.56349	-1.113859214	0.133261	-8.358458	6.35E-17	1.26E-15
9028	COL6A4P2	4.453598861	-1.113915034	0.370927	-3.003056	0.002673	0.005803
8119	ERC2	13.41296443	-1.114203935	0.331317	-3.362955	0.000771	0.001862
3540	LSMD1	607.1018982	-1.114269005	0.197513	-5.641485	1.69E-08	9.33E-08
3624	CECR6	26.42493196	-1.114937695	0.199425	-5.590749	2.26E-08	1.22E-07
13891	CHRND	0.515845337	-1.115152186	0.825178	-1.351408	0.176565	0.249143
7361	ERMN	16.27838085	-1.115559522	0.303672	-3.673569	0.000239	0.000637
458	PTPN6	1004.73741	-1.117240712	0.114397	-9.766334	1.57E-22	6.72E-21
12614	LPAR3	1.112098704	-1.117666691	0.632373	-1.767417	0.077158	0.1199
2373	44078	724.2594295	-1.11775941	0.169721	-6.585859	4.52E-11	3.74E-10
7456	RANBP17	38.53854902	-1.118240917	0.307682	-3.634408	0.000279	0.000732
1956	S100A16	2507.875292	-1.118478245	0.1592	-7.025637	2.13E-12	2.14E-11
10386	CYP4F3	81.39129776	-1.118875902	0.445591	-2.510996	0.012039	0.022721
4745	PDCD1LG2	113.5634472	-1.118900861	0.227819	-4.911349	9.05E-07	3.74E-06
12905	CYP4F2	19.67214148	-1.119361172	0.669429	-1.672113	0.094502	0.143536
7015	CNR1	63.70820033	-1.120732916	0.293589	-3.817347	0.000135	0.000377
1985	FBXO6	348.0326282	-1.121881691	0.160625	-6.984469	2.86E-12	2.82E-11
5927	CCR6	128.8858923	-1.122222652	0.260545	-4.307214	1.65E-05	5.47E-05
7609	ISLR2	12.75659536	-1.122239676	0.313756	-3.576788	0.000348	0.000896
9692	C3orf57	5.189996306	-1.122651275	0.408462	-2.748484	0.005987	0.012107
13065	TTTY10	0.471897385	-1.12296436	0.691254	-1.624533	0.104262	0.156421
4855	CRNA0011	285.1028308	-1.124354957	0.23127	-4.861661	1.16E-06	4.70E-06
3400	FAM176B	297.9319385	-1.125189674	0.19574	-5.748388	9.01E-09	5.19E-08
1350	MTHFD1L	494.1962005	-1.125737799	0.144334	-7.799548	6.21E-15	9.02E-14
584	TWF2	998.3819452	-1.126111666	0.120185	-9.369823	7.27E-21	2.44E-19

8097	MATN3	73.22827552	-1.126910168	0.33415	-3.37247	0.000745	0.001803
3386	CCDC109B	219.8266758	-1.12793458	0.195958	-5.756009	8.61E-09	4.99E-08
5988	LOC400931	35.11793238	-1.128021037	0.263886	-4.274661	1.91E-05	6.27E-05
3244	RASAL3	294.8089685	-1.128160435	0.192646	-5.85612	4.74E-09	2.86E-08
9093	FLG2	2.232994295	-1.128469028	0.378772	-2.97928	0.002889	0.006228
8088	LILRA4	24.81289965	-1.128704629	0.334432	-3.374992	0.000738	0.001789
4379	ITGAX	735.1261701	-1.128787761	0.220676	-5.115126	3.14E-07	1.40E-06
1579	POLR2J	1130.654567	-1.128907072	0.151414	-7.455758	8.94E-14	1.11E-12
5128	NPR1	1223.680158	-1.129624434	0.239597	-4.714692	2.42E-06	9.25E-06
13540	HIST1H3B	0.341409461	-1.130182116	0.767013	-1.473486	0.14062	0.203567
10908	TCERG1L	1.864499417	-1.130599057	0.487646	-2.318484	0.020423	0.036699
739	EFNA4	121.684751	-1.1307129	0.126801	-8.917239	4.78E-19	1.27E-17
5302	BGN	20233.5436	-1.130780235	0.24475	-4.620141	3.83E-06	1.42E-05
1318	RP9P	213.4831581	-1.131236427	0.144308	-7.83902	4.54E-15	6.76E-14
7056	C12orf53	13.00589718	-1.131487932	0.29787	-3.7986	0.000146	0.000404
2656	PLEKHA4	728.3241516	-1.131893172	0.179057	-6.321414	2.59E-10	1.91E-09
4610	SLC9A9	557.1599188	-1.132253605	0.227178	-4.984003	6.23E-07	2.65E-06
8860	CNTN2	4.959830589	-1.132572394	0.369407	-3.065922	0.00217	0.0048
2993	PLCXD1	195.7411121	-1.133361302	0.187571	-6.042292	1.52E-09	9.95E-09
1235	NME2	7203.087095	-1.133622456	0.14253	-7.953558	1.81E-15	2.88E-14
5234	MCOLN2	45.02411088	-1.133970477	0.243343	-4.659977	3.16E-06	1.18E-05
839	SLC12A9	928.0796518	-1.134164683	0.131089	-8.651854	5.07E-18	1.18E-16
2011	RPL29	8345.527503	-1.13416552	0.163285	-6.945941	3.76E-12	3.66E-11
4291	GPR55	16.65105564	-1.134290914	0.219816	-5.160182	2.47E-07	1.13E-06
1307	RIN3	787.1436892	-1.134316845	0.144428	-7.853873	4.03E-15	6.05E-14
2202	MEX3B	67.70984316	-1.134353737	0.167985	-6.752729	1.45E-11	1.29E-10
8101	WNT5B	85.88005052	-1.135696346	0.336918	-3.370841	0.000749	0.001813
982	ADAM15	1622.172533	-1.135806821	0.135677	-8.371416	5.69E-17	1.14E-15
7290	OC1001282	4.610402539	-1.135809482	0.306765	-3.702536	0.000213	0.000574
5171	FPR3	825.2939564	-1.135812782	0.2422	-4.689557	2.74E-06	1.04E-05
14064	FAM75C1	0.535852951	-1.135997718	0.875271	-1.297882	0.194328	0.270835
2291	VIM	83217.49551	-1.136075635	0.17051	-6.6628	2.69E-11	2.30E-10
12745	LGI1	0.90928759	-1.136241105	0.660929	-1.719157	0.085586	0.131625
5507	POMC	17.87234108	-1.136668155	0.251718	-4.515641	6.31E-06	2.25E-05
7939	NDUFA4L2	30565.71053	-1.136854279	0.331196	-3.432571	0.000598	0.001476
7073	EMILIN3	11.33735994	-1.136963999	0.299658	-3.794209	0.000148	0.00041
7691	CEBPE	1.778787445	-1.13699094	0.320888	-3.543264	0.000395	0.001007
11462	LOC26102	1.010408667	-1.137469792	0.533681	-2.131365	0.033059	0.056534
6985	DHRS9	38.03039318	-1.137628788	0.296893	-3.831785	0.000127	0.000357
13864	CHRNA9	0.303002292	-1.137998776	0.834803	-1.363195	0.172821	0.244335
4792	PRAM1	68.65239113	-1.138048959	0.23271	-4.890415	1.01E-06	4.12E-06
429	CD276	1927.001609	-1.138073411	0.114845	-9.909647	3.78E-23	1.72E-21
8869	OC1001335	9.649951607	-1.138077903	0.371524	-3.063273	0.002189	0.004838
5931	OC1001285	2.667491159	-1.138216674	0.264465	-4.303841	1.68E-05	5.55E-05
4886	CD96	234.205473	-1.138846734	0.235595	-4.833917	1.34E-06	5.37E-06
6395	CTSW	270.2698633	-1.138926307	0.279326	-4.077407	4.55E-05	0.00014
9465	CXCL1	203.3095522	-1.139378505	0.402368	-2.831682	0.00463	0.009589

1517	TMEM86B	75.70879217	-1.139396649	0.151169	-7.537232	4.80E-14	6.20E-13
5677	TTK	64.21424969	-1.139687928	0.257037	-4.433942	9.25E-06	3.19E-05
3998	C2orf81	107.1974694	-1.139783953	0.213372	-5.341779	9.20E-08	4.51E-07
9287	IGF2BP1	140.7025938	-1.140172861	0.39247	-2.905118	0.003671	0.007748
9984	CRLF1	35.12166689	-1.140355646	0.429014	-2.658088	0.007859	0.015428
776	EFHD2	1658.831134	-1.140377568	0.129239	-8.823815	1.11E-18	2.79E-17
4073	TPST2	878.4706639	-1.140602131	0.215429	-5.294563	1.19E-07	5.74E-07
7542	JAKMIP1	32.25088667	-1.14069318	0.31698	-3.598625	0.00032	0.000831
6067	A1BG	64.26450308	-1.140807239	0.269256	-4.236889	2.27E-05	7.32E-05
5714	SCARB1	6835.38432	-1.141264991	0.258559	-4.413936	1.02E-05	3.48E-05
1117	G6PD	900.6723201	-1.142003968	0.140332	-8.13785	4.02E-16	7.06E-15
6483	ZNF804A	17.08149741	-1.142042089	0.282998	-4.03552	5.45E-05	0.000165
10669	LOC64730(	8.124372219	-1.142238082	0.47499	-2.40476	0.016183	0.029731
4351	PID1	103.1833793	-1.142443884	0.222686	-5.130286	2.89E-07	1.30E-06
4801	CCL4	279.2079199	-1.142861965	0.233906	-4.885994	1.03E-06	4.20E-06
3360	C9orf142	327.4451334	-1.143256653	0.198087	-5.771477	7.86E-09	4.58E-08
2342	LRRC61	500.0219299	-1.144018798	0.172928	-6.615585	3.70E-11	3.10E-10
3256	C20orf118	51.73773928	-1.144088676	0.195625	-5.848371	4.96E-09	2.99E-08
1248	B4GALT2	796.8652338	-1.14422376	0.14424	-7.932767	2.14E-15	3.37E-14
4462	HMOX1	7000.787022	-1.1443185	0.226064	-5.061916	4.15E-07	1.82E-06
7692	HRH4	2.145323468	-1.144663157	0.323069	-3.54309	0.000395	0.001008
5185	LOC25735ε	5.286042751	-1.145020081	0.244512	-4.682873	2.83E-06	1.07E-05
2578	SLC29A3	253.8092742	-1.14518446	0.178829	-6.403791	1.52E-10	1.15E-09
2579	C7orf50	1416.27984	-1.145442645	0.178883	-6.403307	1.52E-10	1.16E-09
6250	TNFRSF25	82.24006798	-1.145925716	0.275814	-4.154712	3.26E-05	0.000102
14618	KRT82	0.245686851	-1.146130101	1.016676	-1.127331	0.259603	0.348096
5367	CENPE	99.48261628	-1.146550287	0.250116	-4.584078	4.56E-06	1.67E-05
15383	C14orf115	0.380340348	-1.146774362	1.282602	-0.8941	0.371268	0.47307
16100	CSRP3	0.225350421	-1.147171408	1.6843	-0.681097	0.49581	0.603626
2045	DGKA	303.4291555	-1.147201377	0.165928	-6.913847	4.72E-12	4.52E-11
3883	LOC72923<	34.43191548	-1.147343044	0.211962	-5.412972	6.20E-08	3.13E-07
5543	ABLIM2	152.9490878	-1.14735743	0.254902	-4.501166	6.76E-06	2.39E-05
11653	LCT	0.577155201	-1.14751036	0.556836	-2.060769	0.039325	0.066149
9976	TUSC5	1.486528127	-1.147564182	0.431205	-2.661295	0.007784	0.015294
12926	LHX1	11.1221726	-1.147665962	0.689036	-1.665611	0.095791	0.145257
5145	CLDN14	20.42602608	-1.148376629	0.244092	-4.704696	2.54E-06	9.69E-06
10388	C7orf69	0.747914052	-1.148556429	0.457518	-2.510409	0.012059	0.022754
4053	LRRC25	367.8099643	-1.149065383	0.21663	-5.304276	1.13E-07	5.47E-07
2787	CHAF1B	88.26150933	-1.149181448	0.18522	-6.204421	5.49E-10	3.86E-09
7945	CXCR1	27.1679929	-1.149732464	0.335216	-3.429826	0.000604	0.00149
1804	DUSP10	183.3176339	-1.149770906	0.160035	-7.184505	6.75E-13	7.33E-12
4707	MCM10	41.01384507	-1.149871641	0.233318	-4.928345	8.29E-07	3.45E-06
12692	CYorf15B	90.20554777	-1.151211986	0.663013	-1.736335	0.082505	0.127417
2168	SHISA4	189.484804	-1.1514491	0.169457	-6.794922	1.08E-11	9.80E-11
11615	PITX1	33.52915237	-1.152771477	0.555113	-2.076643	0.037834	0.063848
8312	TMEM84	11.67051416	-1.154539885	0.351172	-3.287675	0.00101	0.002382
1558	TAF10	953.745461	-1.155110373	0.154356	-7.483437	7.24E-14	9.11E-13

2198	B4GALT7	918.4016397	-1.155204821	0.170892	-6.759836	1.38E-11	1.23E-10
6452	CUX2	2.433251885	-1.155478988	0.285465	-4.04771	5.17E-05	0.000157
767	YIF1B	469.9453278	-1.155544195	0.130689	-8.841961	9.41E-19	2.40E-17
1692	NMRAL1	822.0606303	-1.155932407	0.158256	-7.304211	2.79E-13	3.23E-12
619	TMSL3	32658.98984	-1.156102949	0.124963	-9.251549	2.21E-20	7.01E-19
1286	C1RL	1328.409212	-1.156506454	0.146774	-7.879499	3.29E-15	5.01E-14
7454	CHRNB2	10.68814592	-1.156795134	0.318265	-3.634689	0.000278	0.000732
10527	C7orf65	0.901969472	-1.157830588	0.470543	-2.460625	0.01387	0.025822
9261	ODZ2	79.78532827	-1.157990965	0.397633	-2.912209	0.003589	0.007596
5890	FCRL6	46.37324069	-1.159602592	0.26808	-4.325577	1.52E-05	5.06E-05
5990	LRRC3	39.77114033	-1.159816786	0.271375	-4.273853	1.92E-05	6.29E-05
12130	KRT13	5.930490014	-1.160176417	0.604946	-1.917818	0.055134	0.089087
7457	CD8B	172.1170477	-1.160344412	0.319275	-3.634315	0.000279	0.000733
13739	MYF6	0.532815807	-1.160402732	0.825973	-1.404892	0.160053	0.228343
5738	SLAMF6	213.4153762	-1.160433945	0.263583	-4.402538	1.07E-05	3.65E-05
6565	ZNF695	6.771440025	-1.161324989	0.29047	-3.998085	6.39E-05	0.000191
1543	TNFRSF1B	1661.741447	-1.161559401	0.154856	-7.500879	6.34E-14	8.05E-13
7529	PIPOX	338.0087831	-1.161584964	0.322247	-3.604637	0.000313	0.000814
3355	CHPF	3311.168098	-1.162015467	0.201155	-5.776707	7.62E-09	4.45E-08
6410	PCDHB17	10.09710909	-1.162572652	0.285533	-4.071591	4.67E-05	0.000143
3888	MGC87042	14.31846015	-1.1627905	0.214968	-5.409135	6.33E-08	3.19E-07
5918	EFS	123.3624897	-1.163008419	0.26962	-4.313507	1.61E-05	5.32E-05
2285	MYL9	5817.075534	-1.163456657	0.174496	-6.667538	2.60E-11	2.23E-10
1525	CD97	1985.228092	-1.163482729	0.15467	-7.522361	5.38E-14	6.91E-13
4489	IFI6	2065.886621	-1.163828905	0.230636	-5.04617	4.51E-07	1.97E-06
8415	LOC644172	30.72581348	-1.164978547	0.359161	-3.24361	0.00118	0.002749
4183	P2RX5	45.83168134	-1.165223141	0.223195	-5.220656	1.78E-07	8.35E-07
2687	ARHGAP30	737.3191104	-1.165315108	0.185196	-6.292327	3.13E-10	2.28E-09
3960	GRAMD4	2155.342865	-1.165434389	0.217024	-5.370062	7.87E-08	3.90E-07
2968	ATXN7L2	96.28220551	-1.165582423	0.192449	-6.056592	1.39E-09	9.18E-09
330	SHC1	4429.550288	-1.165877902	0.112073	-10.40283	2.41E-25	1.43E-23
2680	GPC1	1455.614307	-1.167210938	0.185296	-6.299159	2.99E-10	2.19E-09
3049	NLGN3	18.92269728	-1.16739834	0.194505	-6.0019	1.95E-09	1.25E-08
11985	HIGD2B	0.489456744	-1.168179939	0.594711	-1.96428	0.049498	0.080951
6707	SCUBE3	37.98072755	-1.168474987	0.296646	-3.938959	8.18E-05	0.000239
4011	PDIA5	1749.882396	-1.168581422	0.219065	-5.334401	9.59E-08	4.68E-07
2434	HLA-DPB1	7838.078625	-1.169247448	0.179123	-6.527625	6.68E-11	5.38E-10
13767	NEUROG3	0.459421321	-1.16933781	0.837327	-1.396512	0.16256	0.231448
4330	CD300A	440.6768745	-1.169821685	0.227494	-5.142211	2.72E-07	1.23E-06
2542	E2F1	146.6400505	-1.170042239	0.181987	-6.429245	1.28E-10	9.88E-10
10423	KCNK2	36.50999298	-1.170123754	0.467602	-2.50239	0.012336	0.023198
11574	NPC1L1	98.28172467	-1.171100713	0.559992	-2.091282	0.036503	0.061819
6151	ELOVL2	126.496311	-1.171416189	0.278759	-4.202248	2.64E-05	8.42E-05
7218	GPR173	24.64981819	-1.171601058	0.313405	-3.738297	0.000185	0.000503
6406	GLI1	52.90615399	-1.171930755	0.287602	-4.074836	4.60E-05	0.000141
4097	KCNE2	10.61244066	-1.172028164	0.22188	-5.282265	1.28E-07	6.10E-07
7656	C11orf70	42.94310878	-1.17239585	0.329628	-3.556729	0.000376	0.000961

4129	C17orf87	42.14710522	-1.172675752	0.222807	-5.263186	1.42E-07	6.72E-07
3054	LCP1	3362.772734	-1.173025991	0.195608	-5.996831	2.01E-09	1.29E-08
2821	APOB48R	268.2897882	-1.173046318	0.190116	-6.170154	6.82E-10	4.74E-09
9930	C9	28.69113954	-1.174006196	0.438527	-2.677161	0.007425	0.014656
9363	VSX1	5.422528907	-1.17446134	0.408408	-2.875704	0.004031	0.008439
4227	CD28	99.63717627	-1.174586719	0.226098	-5.19503	2.05E-07	9.49E-07
14703	C10orf53	0.512164698	-1.175222007	1.07112	-1.09719	0.272558	0.363355
6075	HRCT1	89.49368707	-1.17527017	0.277738	-4.231576	2.32E-05	7.49E-05
3280	NHG3-RCC	188.8195112	-1.175373708	0.201593	-5.830443	5.53E-09	3.30E-08
4872	SCML4	39.83723739	-1.175406081	0.242376	-4.84951	1.24E-06	4.98E-06
6564	FPR2	32.04817628	-1.175878956	0.293909	-4.000822	6.31E-05	0.000188
5351	CCR5	425.9021697	-1.17592804	0.256033	-4.592877	4.37E-06	1.60E-05
3304	HIC1	201.4662162	-1.17604389	0.202319	-5.812828	6.14E-09	3.64E-08
1081	TMEM173	990.0512571	-1.176179423	0.143664	-8.187008	2.68E-16	4.86E-15
3289	KLHL6	299.4255023	-1.17656891	0.202096	-5.821842	5.82E-09	3.47E-08
13468	C21orf99	0.486145475	-1.176724409	0.786016	-1.497074	0.134374	0.195565
6841	HLA-DQA2	928.5602631	-1.176867433	0.303026	-3.883716	0.000103	0.000295
1469	TICAM2	261.5833944	-1.177013903	0.1546	-7.613309	2.67E-14	3.56E-13
402	VOPP1	1700.083374	-1.177284275	0.117509	-10.01865	1.26E-23	6.14E-22
3269	SYNPO	9691.726336	-1.177455619	0.201749	-5.836237	5.34E-09	3.20E-08
4230	NOL3	2190.377697	-1.178026232	0.226829	-5.193459	2.06E-07	9.57E-07
2264	C6orf129	227.2415848	-1.178784069	0.176289	-6.686671	2.28E-11	1.98E-10
9781	SLC4A3	135.7328978	-1.178858637	0.433072	-2.722083	0.006487	0.013
5238	RFPL1S	8.984602871	-1.178938395	0.253161	-4.65688	3.21E-06	1.20E-05
3873	HHIPL1	40.30918919	-1.179066646	0.217503	-5.420922	5.93E-08	3.00E-07
13427	MS4A6E	0.345295458	-1.179091169	0.782479	-1.506867	0.131845	0.19247
448	PHF19	355.5205669	-1.179189773	0.120133	-9.815731	9.63E-23	4.21E-21
11450	FOXE3	0.481982154	-1.179816116	0.552815	-2.134197	0.032827	0.05619
733	POLD4	1653.112698	-1.18008549	0.132159	-8.929262	4.29E-19	1.15E-17
1190	RPL38	6853.476639	-1.181323626	0.147152	-8.027892	9.92E-16	1.63E-14
6752	FAM155A	41.55112191	-1.181703308	0.301571	-3.918498	8.91E-05	0.000259
5528	C6orf41	32.972776	-1.181789023	0.262238	-4.506546	6.59E-06	2.34E-05
6532	EGFL8	104.5629906	-1.182262123	0.294665	-4.012222	6.01E-05	0.00018
5453	ZBTB32	8.827712731	-1.184247977	0.260784	-4.541104	5.60E-06	2.01E-05
10023	HIST1H3G	1.068842774	-1.184413165	0.447883	-2.644471	0.008182	0.016001
3390	CCDC85B	369.6913072	-1.184418925	0.205871	-5.753195	8.76E-09	5.06E-08
2084	C7orf47	237.6051845	-1.185525062	0.172664	-6.866072	6.60E-12	6.21E-11
9859	LOC148824	1.891641245	-1.185828404	0.439081	-2.700706	0.006919	0.013756
2758	GMFG	753.6262915	-1.18597221	0.190256	-6.233559	4.56E-10	3.24E-09
1035	TRPV2	462.2480897	-1.186305945	0.143306	-8.278104	1.25E-16	2.37E-15
4613	PIWIL2	5.849005594	-1.186573076	0.238122	-4.983046	6.26E-07	2.66E-06
12621	UNC93A	31.51785596	-1.186748706	0.671885	-1.766297	0.077346	0.120129
6432	ATAD3C	140.6672195	-1.186832196	0.292506	-4.05746	4.96E-05	0.000151
2858	RNASE6	499.7514367	-1.187588445	0.193503	-6.137316	8.39E-10	5.76E-09
2628	P4HA2	2829.48643	-1.188057301	0.187018	-6.352643	2.12E-10	1.58E-09
5716	P2RX1	37.13883795	-1.188829516	0.269455	-4.411972	1.02E-05	3.51E-05
7741	NCCRP1	9.352541138	-1.188913368	0.337447	-3.523261	0.000426	0.001079

1691	SLC1A5	1893.802374	-1.189684641	0.162866	-7.304678	2.78E-13	3.22E-12
5729	C5orf20	14.50858484	-1.189849718	0.269937	-4.407882	1.04E-05	3.57E-05
1663	TEAD4	438.889226	-1.190228638	0.162093	-7.342862	2.09E-13	2.46E-12
679	ANXA2P2	3434.233291	-1.190467436	0.131307	-9.066263	1.23E-19	3.56E-18
13703	CEACAM20	0.285165369	-1.190699931	0.84023	-1.417112	0.15645	0.223789
586	PPIB	10989.13691	-1.190738802	0.127209	-9.360512	7.94E-21	2.65E-19
14414	MAGEA10	0.306863693	-1.191208466	0.997629	-1.194039	0.232463	0.316116
4838	DAPK2	142.3312801	-1.191224435	0.244595	-4.870197	1.11E-06	4.52E-06
484	C19orf10	2291.475274	-1.191537088	0.123174	-9.673636	3.90E-22	1.58E-20
10394	HORMAD1	1.107056564	-1.191592453	0.474841	-2.509455	0.012092	0.022803
5336	ODF3L1	11.60488565	-1.192307231	0.258987	-4.603735	4.15E-06	1.52E-05
4130	SLC16A3	6333.518743	-1.192587872	0.226611	-5.262713	1.42E-07	6.74E-07
2896	RPL22L1	638.1292329	-1.193019715	0.195116	-6.114405	9.69E-10	6.56E-09
11806	IFNB1	0.311197071	-1.193645781	0.591138	-2.019234	0.043463	0.07216
4861	SRPX	129.7111406	-1.193714125	0.245818	-4.85608	1.20E-06	4.83E-06
2526	HRH1	385.1671185	-1.193825275	0.185287	-6.443104	1.17E-10	9.08E-10
11695	ST8SIA2	6.349278366	-1.194074364	0.581749	-2.052561	0.040115	0.067234
8932	KNDC1	190.8237277	-1.194331061	0.392791	-3.04063	0.002361	0.005181
14853	CGB	0.329893583	-1.194606983	1.136165	-1.051438	0.293058	0.386738
4486	SOCS3	3295.369212	-1.194808855	0.236702	-5.047733	4.47E-07	1.95E-06
1266	RPLP2	11049.03837	-1.194920002	0.151123	-7.90694	2.64E-15	4.08E-14
5815	AIFM3	22.48330515	-1.195767168	0.27387	-4.366189	1.26E-05	4.26E-05
2384	NME2P1	88.4057308	-1.196044959	0.181923	-6.574456	4.88E-11	4.02E-10
3436	ICAM1	4289.36361	-1.196046027	0.209086	-5.720363	1.06E-08	6.06E-08
1095	TOP1MT	504.7717809	-1.19672719	0.146525	-8.167412	3.15E-16	5.64E-15
6382	NRN1	508.6400934	-1.197008291	0.292953	-4.086005	4.39E-05	0.000135
4161	ADRA1B	226.4921385	-1.197539	0.228418	-5.242759	1.58E-07	7.45E-07
7469	PIP5KL1	6.151458135	-1.197782363	0.330115	-3.628381	0.000285	0.000748
5485	MS4A14	99.09753397	-1.197866045	0.264501	-4.528782	5.93E-06	2.12E-05
939	DOK1	340.0168545	-1.199129986	0.141639	-8.466079	2.54E-17	5.30E-16
14701	DSPP	0.249036118	-1.19917246	1.091769	-1.098376	0.272041	0.362715
3633	CCNE1	44.57558387	-1.199429146	0.214876	-5.581962	2.38E-08	1.28E-07
4143	MAPK11	394.803147	-1.199792125	0.228308	-5.25515	1.48E-07	7.00E-07
5153	ARNTL2	53.96565715	-1.199836235	0.255224	-4.701106	2.59E-06	9.84E-06
10917	PAPL	0.702707145	-1.200224741	0.518844	-2.313267	0.020708	0.03718
7129	PSD2	23.76847909	-1.200232995	0.318028	-3.773983	0.000161	0.000442
2006	RPL37A	14990.07963	-1.200795134	0.172755	-6.950862	3.63E-12	3.55E-11
13874	GAD2	0.542037472	-1.200960508	0.883157	-1.359849	0.173878	0.245635
8241	TRNP1	150.749229	-1.201081672	0.362311	-3.315055	0.000916	0.002179
7297	RMRP	38.90802044	-1.202646647	0.324992	-3.700547	0.000215	0.000578
5889	PTGER2	167.9065223	-1.202831488	0.278041	-4.3261	1.52E-05	5.05E-05
3590	ITGAL	786.9191709	-1.203151872	0.214493	-5.609279	2.03E-08	1.11E-07
16236	DIRC1	0.228091211	-1.203715243	1.866358	-0.644954	0.518957	0.626514
3674	LOC283663	35.08720506	-1.204253579	0.216829	-5.553923	2.79E-08	1.49E-07
2703	STX1A	51.78248624	-1.205818722	0.192052	-6.278611	3.42E-10	2.48E-09
709	RNASEH2A	315.4112735	-1.207349284	0.13434	-8.98729	2.53E-19	7.01E-18
2723	PLXDC1	1052.826705	-1.207545367	0.192846	-6.261716	3.81E-10	2.74E-09

1426	HIVEP3	183.1417729	-1.208054309	0.157429	-7.67365	1.67E-14	2.30E-13
12280	KRT4	1.32912807	-1.208212545	0.645324	-1.872257	0.061171	0.09764
4982	NCR3	14.33288641	-1.208698065	0.252716	-4.782824	1.73E-06	6.80E-06
1970	HMHA1	887.7247126	-1.209884389	0.172795	-7.00183	2.53E-12	2.51E-11
1672	FAM78A	290.3672001	-1.21006325	0.165026	-7.332556	2.26E-13	2.65E-12
14896	SLC7A3	0.256101527	-1.210224811	1.166567	-1.037425	0.299538	0.394123
6055	TNXB	489.165473	-1.210313244	0.285334	-4.241745	2.22E-05	7.18E-05
4696	CDKN2BAS	8.717885809	-1.211997252	0.245756	-4.931711	8.15E-07	3.40E-06
12725	HIST1H2AI	0.314721016	-1.212282714	0.702929	-1.724617	0.084596	0.130308
8272	DNASE2B	6.346684722	-1.212927919	0.367115	-3.303946	0.000953	0.002259
1494	IFITM3	12538.78789	-1.214540513	0.160308	-7.576316	3.56E-14	4.66E-13
6829	LTK	13.96298346	-1.214562963	0.312343	-3.888554	0.000101	0.000289
8941	LCTL	1.414926928	-1.215280204	0.40042	-3.035012	0.002405	0.005273
6429	JPH3	4.842743597	-1.215409861	0.299305	-4.060769	4.89E-05	0.000149
5173	TSPAN2	228.363954	-1.215648668	0.259275	-4.68864	2.75E-06	1.04E-05
5323	ABCC3	3721.648844	-1.216150023	0.263762	-4.610791	4.01E-06	1.48E-05
6043	CHST5	8.014505316	-1.216652698	0.286409	-4.247954	2.16E-05	7.00E-05
1224	TMUB1	719.3943105	-1.216672269	0.152327	-7.987243	1.38E-15	2.21E-14
9615	DNAH3	5.648509941	-1.217201252	0.438726	-2.7744	0.00553	0.011274
2165	MGC72080	109.8116593	-1.217389337	0.179114	-6.796711	1.07E-11	9.69E-11
5542	SYCE2	9.429879861	-1.217443369	0.270465	-4.501301	6.75E-06	2.39E-05
2146	ARL11	67.82192147	-1.217885839	0.178811	-6.811006	9.69E-12	8.85E-11
14479	PLA2G2F	0.293144353	-1.218108536	1.037066	-1.174572	0.240166	0.325126
1390	GLIPR2	519.3953255	-1.21846138	0.157688	-7.727062	1.10E-14	1.55E-13
5074	CRYGS	25.19107121	-1.218709307	0.257392	-4.734831	2.19E-06	8.47E-06
7239	ATOH7	6.686937011	-1.218757534	0.326844	-3.728866	0.000192	0.000521
2836	MRPL23	1041.6479	-1.219381531	0.198059	-6.156664	7.43E-10	5.13E-09
3731	LPCAT1	7221.50216	-1.220170188	0.221112	-5.518331	3.42E-08	1.80E-07
4210	GPR39	192.0742274	-1.220212072	0.234519	-5.203052	1.96E-07	9.13E-07
10026	PCSK1N	201.0747302	-1.220829346	0.461919	-2.642948	0.008219	0.016068
14678	C9orf57	0.295209386	-1.220915529	1.101242	-1.108671	0.267572	0.357316
6026	FBXO41	226.7246727	-1.221269864	0.287081	-4.254101	2.10E-05	6.83E-05
5338	LOC349196	9.51664001	-1.222585932	0.265637	-4.602461	4.18E-06	1.53E-05
1673	LSM7	431.2079388	-1.222793836	0.166763	-7.332507	2.26E-13	2.65E-12
5089	C20orf46	134.1898265	-1.222863195	0.258545	-4.729793	2.25E-06	8.65E-06
3828	PFKFB4	645.4146155	-1.223511311	0.224525	-5.44934	5.06E-08	2.59E-07
1180	LOC728855	228.4618041	-1.224407669	0.152082	-8.050983	8.21E-16	1.36E-14
4977	CD2	466.3156402	-1.224473114	0.255906	-4.784853	1.71E-06	6.74E-06
6197	NUPR1	5157.550295	-1.225247443	0.293348	-4.176774	2.96E-05	9.35E-05
14539	DYDC1	0.6295847	-1.22551927	1.062323	-1.153623	0.248655	0.335228
7044	CA3	28.7352484	-1.225541058	0.322248	-3.803096	0.000143	0.000398
4680	SLC1A3	505.71662	-1.22564388	0.248072	-4.940672	7.79E-07	3.26E-06
3419	C1orf113	265.6125985	-1.225646602	0.213851	-5.73131	9.97E-09	5.71E-08
2719	RPL35	8457.018914	-1.22588892	0.195676	-6.264877	3.73E-10	2.69E-09
2337	C20orf199	2370.895093	-1.226036608	0.185243	-6.618522	3.63E-11	3.04E-10
2189	LMNB1	431.9764375	-1.226677555	0.181176	-6.770648	1.28E-11	1.15E-10
2313	GINS2	150.7290654	-1.226723033	0.18466	-6.643138	3.07E-11	2.60E-10

12425	POPDC3	3.813434657	-1.227502983	0.671061	-1.829197	0.06737	0.10628
5438	FAM179A	9.718476991	-1.227617677	0.269886	-4.548661	5.40E-06	1.95E-05
2969	CHTF18	158.6326087	-1.228252428	0.202804	-6.05636	1.39E-09	9.19E-09
1119	C21orf122	33.09076057	-1.228431833	0.151076	-8.131218	4.25E-16	7.44E-15
2356	NLRC4	91.37902693	-1.228802898	0.186143	-6.601405	4.07E-11	3.39E-10
14080	SLC27A6	0.480175514	-1.229195538	0.950035	-1.293842	0.19572	0.272465
7905	RASL11B	34.18582434	-1.231261377	0.357318	-3.445838	0.000569	0.001412
1561	SSR4	3409.19013	-1.231582674	0.164707	-7.477435	7.58E-14	9.52E-13
1859	TSPO	1587.483651	-1.231769454	0.172815	-7.127686	1.02E-12	1.08E-11
6255	SLC7A7	2455.107303	-1.231878594	0.296655	-4.152558	3.29E-05	0.000103
10925	RGS22	7.75642944	-1.232193831	0.533193	-2.31097	0.020835	0.03738
1366	PLEC	8066.210984	-1.232590467	0.158638	-7.76983	7.86E-15	1.13E-13
9237	ABRA	2.416389764	-1.232716902	0.4216	-2.923898	0.003457	0.007335
13801	OR1Q1	0.342113262	-1.232825339	0.888948	-1.386836	0.165492	0.235063
5014	TREML2	6.653813252	-1.233119644	0.25879	-4.764936	1.89E-06	7.39E-06
1331	THBS3	690.3616004	-1.233162574	0.157627	-7.823291	5.15E-15	7.58E-14
4553	CCL3	171.6609578	-1.233991203	0.246408	-5.007912	5.50E-07	2.37E-06
1589	FLT3LG	126.7693444	-1.234731986	0.166001	-7.438094	1.02E-13	1.26E-12
5813	MGAT5B	14.05802243	-1.234810833	0.282725	-4.367539	1.26E-05	4.24E-05
3231	KIF4B	4.650431414	-1.234957133	0.210565	-5.864981	4.49E-09	2.73E-08
7851	SLC24A3	112.3353164	-1.235193243	0.3558	-3.471595	0.000517	0.001292
4264	MSR1	1555.767165	-1.235763658	0.238828	-5.17429	2.29E-07	1.05E-06
14878	C14orf48	0.3070873	-1.235795524	1.184639	-1.043183	0.296864	0.391073
2524	CSF2RA	250.6334904	-1.235797687	0.191774	-6.444045	1.16E-10	9.03E-10
3520	CDK1	254.1562649	-1.235918698	0.218456	-5.657527	1.54E-08	8.55E-08
2785	C1orf86	467.8438674	-1.236029102	0.199109	-6.207795	5.37E-10	3.78E-09
1028	RHBDF2	1204.93618	-1.236360716	0.149142	-8.289827	1.13E-16	2.16E-15
2105	GNGT2	53.75395208	-1.236457192	0.180653	-6.844368	7.68E-12	7.15E-11
5322	SERPINE2	4704.121953	-1.236552295	0.268164	-4.611175	4.00E-06	1.47E-05
1431	RPLP1	22857.2444	-1.236776924	0.161423	-7.661711	1.83E-14	2.51E-13
14557	DEFA4	0.660596238	-1.236816971	1.076974	-1.148419	0.250796	0.337696
12357	CAPN9	1.195376335	-1.236919639	0.66927	-1.848163	0.064579	0.102437
8167	CACNA1I	8.05707887	-1.237190109	0.369711	-3.346375	0.000819	0.001965
2561	FAM128B	1578.376169	-1.237618708	0.192912	-6.415473	1.40E-10	1.07E-09
3993	GPA33	4.550254138	-1.237652339	0.23158	-5.344388	9.07E-08	4.45E-07
3358	FAM72D	24.551703	-1.239123057	0.21463	-5.773291	7.77E-09	4.54E-08
1765	SIL1	1475.951419	-1.239167668	0.171485	-7.226115	4.97E-13	5.52E-12
6758	43894	30.07868363	-1.239669211	0.316544	-3.916263	8.99E-05	0.000261
4179	HSPB2	446.6996038	-1.239777296	0.237138	-5.22808	1.71E-07	8.03E-07
13581	FOXE1	5.272153181	-1.240389198	0.849047	-1.460919	0.144038	0.207885
3233	BMP8A	184.3051184	-1.240825202	0.211615	-5.863605	4.53E-09	2.75E-08
11333	ARID3C	0.924520615	-1.241571991	0.572123	-2.170113	0.029998	0.051884
2364	PROCR	923.3551009	-1.241649326	0.188295	-6.594174	4.28E-11	3.55E-10
7014	MPP2	43.96118301	-1.241712905	0.325252	-3.817689	0.000135	0.000376
1378	NME1	1245.291235	-1.243042064	0.160252	-7.756815	8.71E-15	1.24E-13
6869	LOC39981	3.80083563	-1.243631367	0.320856	-3.87598	0.000106	0.000303
3979	LRRC8E	151.9285416	-1.24370049	0.232296	-5.353953	8.61E-08	4.24E-07

7200	SLC26A8	1.594365343	-1.243869387	0.332061	-3.745908	0.00018	0.000489
6737	KIAA0087	9.480697944	-1.244917241	0.317099	-3.925962	8.64E-05	0.000251
15730	OR1E1	0.281302798	-1.244977226	1.565758	-0.795127	0.426539	0.531507
6685	GPR120	10.75401732	-1.24535027	0.315518	-3.947006	7.91E-05	0.000232
13892	C7orf33	0.281213972	-1.245538637	0.921802	-1.3512	0.176631	0.249219
7561	AICDA	14.10851263	-1.245773388	0.346816	-3.592031	0.000328	0.000851
5596	ODF3B	294.3502352	-1.245853641	0.278488	-4.473633	7.69E-06	2.69E-05
2344	NPM3	228.2453807	-1.246986791	0.18861	-6.611459	3.81E-11	3.18E-10
5360	)C1001889	13.09878173	-1.247492301	0.271981	-4.586694	4.50E-06	1.65E-05
1244	GNB1L	79.47308198	-1.247503165	0.157187	-7.936425	2.08E-15	3.28E-14
5476	SEZ6L2	1958.503389	-1.248273677	0.275399	-4.532595	5.83E-06	2.09E-05
10270	WNK2	43.77425099	-1.248652211	0.488259	-2.557356	0.010547	0.02013
5824	ICAM4	17.15010721	-1.249242357	0.286437	-4.361312	1.29E-05	4.35E-05
4455	)C1002722	29.80366852	-1.250403511	0.246644	-5.069675	3.98E-07	1.75E-06
8416	PTGES	330.5466691	-1.250521309	0.38555	-3.243474	0.001181	0.00275
8806	SCN3A	26.99918975	-1.250886017	0.405507	-3.084743	0.002037	0.004535
2992	C20orf134	27.37369132	-1.25095189	0.207018	-6.042728	1.52E-09	9.93E-09
3019	CACNB1	89.17491027	-1.251090444	0.207807	-6.020447	1.74E-09	1.13E-08
6313	KIF26B	94.03493593	-1.251508313	0.303835	-4.119037	3.80E-05	0.000118
1484	C19orf40	30.65471107	-1.253863587	0.165103	-7.594427	3.09E-14	4.08E-13
13917	LOC72767	0.301574172	-1.255381243	0.933305	-1.345093	0.178595	0.251537
3996	SPC25	42.35337443	-1.255609864	0.234983	-5.343402	9.12E-08	4.47E-07
4381	PFN4	7.654078671	-1.255736307	0.245547	-5.114046	3.15E-07	1.41E-06
10762	C3orf65	0.505171712	-1.256361949	0.528321	-2.378026	0.017406	0.031701
4399	TWIST1	23.20373171	-1.256478559	0.246156	-5.10441	3.32E-07	1.48E-06
6643	RHD	5.866193044	-1.256534895	0.316782	-3.96656	7.29E-05	0.000215
3748	DTX1	72.40789092	-1.257144937	0.228284	-5.506944	3.65E-08	1.91E-07
7554	C20orf203	2.481047842	-1.257710072	0.349952	-3.593954	0.000326	0.000845
4995	CYP4F22	3.60839391	-1.258031679	0.263396	-4.776207	1.79E-06	7.01E-06
8386	PRSS50	13.77579335	-1.258301166	0.386291	-3.25739	0.001124	0.002628
2181	CDK2AP2	2418.353466	-1.258572964	0.185746	-6.775759	1.24E-11	1.11E-10
16124	MAGEA9B	0.217075871	-1.25929565	1.867156	-0.674446	0.500028	0.607817
13645	ACTL8	1.357125251	-1.259410794	0.874536	-1.44009	0.149842	0.215248
4196	IGSF21	65.9187422	-1.260614012	0.241846	-5.212471	1.86E-07	8.70E-07
3346	SDF2L1	517.3247027	-1.260766449	0.217918	-5.785509	7.23E-09	4.23E-08
16130	IL1F5	0.219930813	-1.261422319	1.875656	-0.672523	0.501251	0.609114
2230	TFAP2E	9.204746635	-1.261549267	0.187711	-6.720688	1.81E-11	1.59E-10
1430	Γ6GALNA(	395.5071975	-1.262663105	0.164765	-7.663425	1.81E-14	2.48E-13
2739	UCP2	2124.18781	-1.262683596	0.202118	-6.247265	4.18E-10	2.99E-09
6335	C19orf51	31.90596018	-1.262718857	0.307195	-4.110475	3.95E-05	0.000122
1382	PRC1	336.3975347	-1.26395753	0.163179	-7.745828	9.50E-15	1.35E-13
3447	RHOH	134.9010828	-1.264460027	0.221473	-5.709319	1.13E-08	6.45E-08
7286	DUSP5P	33.26152538	-1.264825798	0.341329	-3.705595	0.000211	0.000567
4508	GATA6	127.7253032	-1.265201175	0.251539	-5.029843	4.91E-07	2.13E-06
5177	CHRD	344.0702881	-1.266265141	0.27025	-4.685533	2.79E-06	1.06E-05
7972	SCG5	113.4371566	-1.266814548	0.370323	-3.420837	0.000624	0.001535
677	IL18BP	746.9721928	-1.267151867	0.139559	-9.079714	1.09E-19	3.15E-18

5732	PHYHIP	27.138907	-1.267322401	0.287578	-4.406882	1.05E-05	3.59E-05
2850	CDCA7L	592.600687	-1.267730948	0.206345	-6.143731	8.06E-10	5.54E-09
7148	CYP2B7P1	5.122536557	-1.268411728	0.336887	-3.765095	0.000166	0.000457
1871	C17orf96	82.29561659	-1.268778614	0.178218	-7.119234	1.09E-12	1.14E-11
13761	C19orf75	0.462750866	-1.269157931	0.906832	-1.399551	0.161648	0.230249
4911	XKRX	4.388117102	-1.269345765	0.26327	-4.82146	1.43E-06	5.69E-06
9643	TFR2	71.74276049	-1.269561902	0.459066	-2.765531	0.005683	0.011552
3344	PRDM6	20.25144642	-1.269801743	0.219321	-5.7897	7.05E-09	4.13E-08
2324	SELL	288.4935785	-1.269971164	0.1914	-6.635166	3.24E-11	2.73E-10
3322	GPRIN1	161.1853176	-1.270910218	0.219065	-5.801521	6.57E-09	3.88E-08
83	TUBA1B	17259.36167	-1.271202462	0.101271	-12.55251	3.85E-36	9.09E-34
2651	C19orf38	46.20840436	-1.271871044	0.201131	-6.323593	2.56E-10	1.89E-09
2574	BLM	53.4355315	-1.271913303	0.198477	-6.40838	1.47E-10	1.12E-09
4190	SAMSN1	308.9031293	-1.271915185	0.243882	-5.215284	1.84E-07	8.59E-07
1092	MIIP	240.5345054	-1.272737386	0.155747	-8.171813	3.04E-16	5.45E-15
15170	LOC28468	0.288354105	-1.273201229	1.331067	-0.956527	0.338806	0.437795
10514	DUSP9	41.14530831	-1.273382973	0.516237	-2.466662	0.013638	0.025425
1336	NAV1	1076.386542	-1.273496748	0.163	-7.812847	5.59E-15	8.20E-14
1252	CIB1	3064.317251	-1.273575028	0.160585	-7.930842	2.18E-15	3.41E-14
5493	SIT1	82.63229967	-1.273938297	0.281559	-4.524582	6.05E-06	2.16E-05
11366	WBSCR28	1.152821556	-1.274006082	0.589109	-2.162598	0.030572	0.052725
5051	CYBB	2127.367036	-1.274189068	0.268495	-4.745663	2.08E-06	8.06E-06
1463	LAT2	311.5174561	-1.274241847	0.167167	-7.622551	2.49E-14	3.33E-13
1324	CD74	87964.9048	-1.274863986	0.162777	-7.831969	4.80E-15	7.11E-14
13403	FAM74A3	0.888586873	-1.275582554	0.843882	-1.511565	0.130645	0.191059
16090	SPANXB2	0.282488288	-1.275723449	1.863014	-0.684763	0.493493	0.601179
2082	UBE2T	79.37300871	-1.275910744	0.18576	-6.868612	6.48E-12	6.10E-11
9039	MOBP	1.028109778	-1.275963482	0.425446	-2.999117	0.002708	0.005871
4235	LGI2	89.84195458	-1.276212201	0.245928	-5.18937	2.11E-07	9.77E-07
6801	FSD1	4.338328449	-1.276385307	0.327277	-3.90002	9.62E-05	0.000277
1493	POC1A	60.08549063	-1.276869659	0.168431	-7.580964	3.43E-14	4.50E-13
3975	LCK	283.8101964	-1.27689792	0.238422	-5.355613	8.53E-08	4.20E-07
2298	AXL	2782.627805	-1.276919486	0.191801	-6.657518	2.78E-11	2.38E-10
10585	RTP1	0.485942095	-1.277020532	0.523311	-2.440271	0.014676	0.027177
6601	FAM131C	214.6922977	-1.277618568	0.320715	-3.983651	6.79E-05	0.000202
6047	NXPH4	484.1335194	-1.277632888	0.301067	-4.24368	2.20E-05	7.13E-05
1155	C1orf54	270.9772969	-1.2782569	0.158178	-8.081154	6.42E-16	1.09E-14
308	CKAP4	2313.947345	-1.278646898	0.121773	-10.50027	8.61E-26	5.48E-24
1270	PSMG3	582.6031172	-1.278984162	0.161896	-7.900017	2.79E-15	4.30E-14
8943	VWCE	317.1387229	-1.279054036	0.421448	-3.034901	0.002406	0.005274
732	ACTN1	4091.775979	-1.279108114	0.143134	-8.936452	4.02E-19	1.08E-17
6934	ST6GAL2	55.56621507	-1.279765455	0.332466	-3.849313	0.000118	0.000335
2042	RAPGEFL1	109.7358113	-1.281538625	0.185195	-6.919938	4.52E-12	4.34E-11
617	RPLP0	27227.23621	-1.281998079	0.138465	-9.258669	2.07E-20	6.58E-19
5361	RASL10A	11.70942602	-1.283425638	0.279888	-4.58549	4.53E-06	1.66E-05
2249	ACRBP	67.54926346	-1.283448736	0.191644	-6.697062	2.13E-11	1.85E-10
7470	LINGO3	3.211899578	-1.283819585	0.353888	-3.627759	0.000286	0.00075

14971	PNLIPRP2	0.288216078	-1.284110071	1.264448	-1.01555	0.309844	0.405667
6994	GPR37L1	5.33390341	-1.284177713	0.335635	-3.826119	0.00013	0.000365
1406	GALNT6	130.6391417	-1.285269358	0.167007	-7.695908	1.40E-14	1.96E-13
11179	C3orf72	0.620427629	-1.285888635	0.576814	-2.229296	0.025794	0.045223
7084	TPSB2	350.3187953	-1.285917411	0.339226	-3.790742	0.00015	0.000416
6149	MUSTN1	66.72278484	-1.286891716	0.306177	-4.203096	2.63E-05	8.39E-05
4957	PLEKHN1	43.56891386	-1.287253893	0.268567	-4.793051	1.64E-06	6.49E-06
1980	FAM83D	113.4823073	-1.287789659	0.184228	-6.990199	2.74E-12	2.72E-11
9677	NPB	6.113669587	-1.287864537	0.467534	-2.754593	0.005877	0.011903
2878	·ADD45GIF	1209.934334	-1.288417494	0.210366	-6.12465	9.09E-10	6.19E-09
6042	C15orf48	165.8796633	-1.288698392	0.303369	-4.247964	2.16E-05	7.00E-05
5130	FOXL1	121.6025854	-1.289107197	0.273471	-4.713864	2.43E-06	9.29E-06
6003	C6orf122	13.01204993	-1.289737661	0.3024	-4.265006	2.00E-05	6.53E-05
5066	HOXC11	33.51538269	-1.289914049	0.272198	-4.738888	2.15E-06	8.31E-06
603	RELT	213.0915282	-1.289967853	0.138602	-9.306996	1.31E-20	4.27E-19
866	VKORC1	3384.374423	-1.290649178	0.149903	-8.609894	7.31E-18	1.66E-16
1726	TRAIP	38.79536627	-1.291416808	0.177625	-7.270488	3.58E-13	4.07E-12
719	GAPDH	129486.2198	-1.291509748	0.144001	-8.968767	3.00E-19	8.17E-18
1435	PSRC1	82.26731045	-1.29176722	0.168842	-7.650735	2.00E-14	2.73E-13
1701	CXCR4	4867.800759	-1.292161209	0.17717	-7.293352	3.02E-13	3.48E-12
3840	ENO2	4514.715771	-1.292402516	0.23756	-5.440328	5.32E-08	2.71E-07
3099	FBF1	107.5859585	-1.294034743	0.216955	-5.964524	2.45E-09	1.55E-08
1625	RPS15	5017.927321	-1.29414106	0.175042	-7.393336	1.43E-13	1.73E-12
6551	ITGB6	464.2284704	-1.294186873	0.32308	-4.005775	6.18E-05	0.000185
2716	FBN1	2483.378653	-1.294730264	0.206632	-6.265877	3.71E-10	2.67E-09
1217	CST3	7343.195196	-1.295203007	0.16195	-7.997537	1.27E-15	2.04E-14
1948	C8orf73	89.88889674	-1.295804831	0.184139	-7.037112	1.96E-12	1.97E-11
6379	ODZ4	297.3069434	-1.295845629	0.317095	-4.086616	4.38E-05	0.000134
11996	LCE5A	0.422474147	-1.296464006	0.660743	-1.962129	0.049747	0.081285
933	TPM4	8323.725164	-1.296642606	0.152994	-8.475118	2.35E-17	4.93E-16
8370	C2orf85	2.85791045	-1.296772607	0.397438	-3.262828	0.001103	0.002583
13462	MRGPRE	1.634028374	-1.297219693	0.865925	-1.498075	0.134114	0.195273
4465	GLT8D2	109.0751575	-1.297260881	0.256367	-5.06018	4.19E-07	1.84E-06
12907	EVX1	0.813887027	-1.297376061	0.776171	-1.671507	0.094622	0.143691
12365	)C1001895	0.557162111	-1.297812724	0.702791	-1.846655	0.064797	0.102716
6621	AREG	103.9471431	-1.29792019	0.326476	-3.975551	7.02E-05	0.000208
2349	FAM20C	2441.720361	-1.29836675	0.196433	-6.609723	3.85E-11	3.21E-10
2499	ARHGAP9	253.5619039	-1.298758929	0.200955	-6.462949	1.03E-10	8.05E-10
924	HCLS1	1494.232874	-1.299223884	0.153129	-8.484488	2.17E-17	4.60E-16
5576	DSCR9	2.239168402	-1.299893104	0.289767	-4.485995	7.26E-06	2.55E-05
16500	OR3A3	0.29362928	-1.299959754	2.268911	-0.572944	0.566682	0.673184
2135	RPS21	6878.428037	-1.300079182	0.190627	-6.820033	9.10E-12	8.36E-11
6193	C12orf69	23.90556398	-1.301195458	0.311461	-4.177717	2.94E-05	9.32E-05
14583	SLC6A11	0.354520876	-1.301371136	1.139808	-1.141746	0.253559	0.340809
6159	RAC3	62.26735182	-1.301975307	0.310115	-4.198366	2.69E-05	8.56E-05
175	TAGLN2	11511.69587	-1.303879751	0.113838	-11.45386	2.25E-30	2.52E-28
1586	NT5DC2	559.1457572	-1.305179371	0.175431	-7.439839	1.01E-13	1.25E-12

1396	DDX11	382.0075828	-1.305239807	0.169025	-7.722154	1.14E-14	1.61E-13
864	H2AFX	451.3300265	-1.305329144	0.151547	-8.61337	7.09E-18	1.61E-16
10763	KHDRBS2	0.779468613	-1.305361829	0.548936	-2.377985	0.017408	0.031702
4637	LILRB5	117.7670431	-1.306002122	0.262746	-4.970587	6.68E-07	2.82E-06
4072	MALL	1443.184249	-1.306193894	0.246697	-5.294733	1.19E-07	5.74E-07
8394	EACAM22	1.757491011	-1.307251667	0.402191	-3.250323	0.001153	0.002692
273	UNC93B1	708.7556605	-1.308273544	0.122065	-10.71784	8.39E-27	6.03E-25
8015	F12	147.6947389	-1.308553524	0.384252	-3.40546	0.000661	0.001615
2308	LPAR5	171.8012879	-1.308801994	0.196865	-6.648225	2.97E-11	2.52E-10
8426	ACRV1	0.879683863	-1.308898996	0.403876	-3.240847	0.001192	0.002772
1294	ICAM3	513.9078105	-1.309357957	0.166328	-7.872152	3.49E-15	5.28E-14
12670	COL29A1	0.75416863	-1.309467487	0.749284	-1.747626	0.080529	0.124581
8627	IFNG	17.50200457	-1.309630841	0.414206	-3.161785	0.001568	0.003563
4116	KCNQ3	33.6346035	-1.30966359	0.248419	-5.271995	1.35E-07	6.43E-07
231	DNTTIP1	603.4843623	-1.310265663	0.119379	-10.97571	5.00E-28	4.24E-26
9406	CD177	26.09860949	-1.31090499	0.459054	-2.855667	0.004295	0.00895
4411	ADAMTS4	1844.40914	-1.311055231	0.25736	-5.094252	3.50E-07	1.56E-06
2654	PLCB2	464.5181387	-1.31145539	0.207419	-6.322727	2.57E-10	1.90E-09
15166	SCGB1C1	0.37014225	-1.311482891	1.370248	-0.957113	0.33851	0.437501
2709	UBQLNL	15.7249112	-1.311715101	0.209137	-6.272045	3.56E-10	2.58E-09
16016	LCN15	0.251116269	-1.313479969	1.857289	-0.707203	0.47944	0.586758
3307	WDFY4	286.1207627	-1.314455228	0.226157	-5.812126	6.17E-09	3.66E-08
1173	CENPH	118.8608787	-1.315228769	0.163192	-8.059377	7.67E-16	1.28E-14
15376	SSX2	0.356259949	-1.315720539	1.46874	-0.895816	0.370351	0.472116
267	C22orf9	2201.263459	-1.316617012	0.122567	-10.742	6.46E-27	4.74E-25
4299	ADAMTS6	24.13161174	-1.317133925	0.255425	-5.156637	2.51E-07	1.15E-06
3027	SIRPB2	115.1476214	-1.317895724	0.21916	-6.013394	1.82E-09	1.18E-08
10135	SYCE1	5.141143119	-1.318065286	0.506342	-2.603112	0.009238	0.017867
6053	CDCP1	1431.631479	-1.318410653	0.310806	-4.241906	2.22E-05	7.18E-05
4587	SELM	1382.442313	-1.318450616	0.264095	-4.99234	5.97E-07	2.55E-06
6768	SSPO	26.36225551	-1.319392298	0.337314	-3.91147	9.17E-05	0.000266
1057	CNN2	1821.056309	-1.320503717	0.160368	-8.234191	1.81E-16	3.35E-15
4064	GLI4	173.9883582	-1.32064343	0.249258	-5.298291	1.17E-07	5.64E-07
3134	BOP1	248.9203154	-1.320903499	0.222508	-5.936441	2.91E-09	1.82E-08
4164	NFKBIZ	520.9715559	-1.321079204	0.25205	-5.241336	1.59E-07	7.50E-07
1580	IL27RA	452.337463	-1.321177382	0.177447	-7.445478	9.66E-14	1.20E-12
8650	GPR109A	79.25070854	-1.321205161	0.419247	-3.151377	0.001625	0.003682
13076	GPHA2	0.519693137	-1.32154551	0.815415	-1.620704	0.105081	0.157505
7019	TPSAB1	236.7461144	-1.321706699	0.3464	-3.81555	0.000136	0.000379
6253	PTGS2	225.9235997	-1.322650002	0.318471	-4.15312	3.28E-05	0.000103
16935	CT45A6	0.239269091	-1.323453327	2.913995	-0.454171	0.649705	0.751977
4479	GZMB	144.0111172	-1.324185172	0.262066	-5.052876	4.35E-07	1.90E-06
2550	TRIM36	57.06345382	-1.324400019	0.206102	-6.425951	1.31E-10	1.01E-09
14933	PHGR1	0.35743836	-1.324761354	1.292017	-1.025343	0.305201	0.400606
9415	C7orf4	0.660469338	-1.324799139	0.464468	-2.852296	0.00434	0.009036
6624	ZCCHC12	5.059801759	-1.324995868	0.333341	-3.9749	7.04E-05	0.000208
4785	HLA-DPB2	18.78320656	-1.325025884	0.27081	-4.892824	9.94E-07	4.07E-06

5712	KAZALD1	42.01638135	-1.325412653	0.300253	-4.414319	1.01E-05	3.48E-05
13330	IGFL1	0.804162969	-1.326014704	0.864701	-1.533495	0.125154	0.184032
6096	KIAA1755	67.22067226	-1.326129986	0.314045	-4.222741	2.41E-05	7.76E-05
181	DTX2	387.7363063	-1.32617221	0.116254	-11.40758	3.83E-30	4.15E-28
11385	C17orf50	0.53198766	-1.326217208	0.6148	-2.157153	0.030994	0.05336
9043	SCN1A	2.167370564	-1.326251283	0.442314	-2.998439	0.002714	0.005882
8660	LOC121838	12.40049556	-1.327585698	0.421874	-3.146879	0.00165	0.003735
3061	PVT1	178.5969073	-1.32829225	0.221702	-5.991334	2.08E-09	1.33E-08
529	KIAA1949	1957.555039	-1.328341916	0.13945	-9.525565	1.64E-21	6.08E-20
13074	KLK7	2.238579138	-1.328639495	0.81928	-1.621716	0.104864	0.157216
11587	NBPF4	1.203741697	-1.330209595	0.637873	-2.085383	0.037035	0.062649
4841	TBC1D3H	20.01504528	-1.330304171	0.273173	-4.869824	1.12E-06	4.52E-06
3065	CCDC81	13.20949421	-1.330906566	0.222228	-5.988937	2.11E-09	1.35E-08
5646	ACAN	887.4964875	-1.331253193	0.299214	-4.44916	8.62E-06	2.99E-05
2554	COL5A3	842.1153489	-1.331526234	0.2073	-6.423186	1.33E-10	1.02E-09
9260	DISC2	0.635476955	-1.33153803	0.457211	-2.912307	0.003588	0.007594
4774	GPR141	14.3841948	-1.331708926	0.271951	-4.896876	9.74E-07	4.00E-06
427	SIPA1	1063.312693	-1.332557406	0.134211	-9.9288	3.12E-23	1.43E-21
14823	PTH	0.274335331	-1.33267307	1.259563	-1.058044	0.290035	0.38354
5685	COL14A1	1203.07531	-1.332693169	0.300976	-4.427902	9.52E-06	3.28E-05
7986	TTC24	3.843363744	-1.332756355	0.390141	-3.416087	0.000635	0.001559
10551	LOC440356	1.24568308	-1.332982126	0.543508	-2.452554	0.014185	0.026351
4416	LRFN4	184.7738143	-1.33318241	0.261817	-5.092048	3.54E-07	1.57E-06
7661	ACOXL	3.493516825	-1.333503606	0.375212	-3.554005	0.000379	0.000971
2480	KIAA1024	34.94351127	-1.333768292	0.205559	-6.488483	8.67E-11	6.85E-10
9190	ALOX15	0.996159971	-1.334351125	0.453491	-2.942399	0.003257	0.006946
4658	FAM171A2	31.12715097	-1.334549883	0.269286	-4.955884	7.20E-07	3.03E-06
3813	BCAT1	769.6525471	-1.334551593	0.244192	-5.465162	4.62E-08	2.38E-07
3507	CDH11	1267.231029	-1.334820673	0.235539	-5.667079	1.45E-08	8.12E-08
2414	TMC8	374.6331445	-1.336098938	0.20414	-6.545025	5.95E-11	4.83E-10
2943	AOAH	361.8945759	-1.336705598	0.220063	-6.074188	1.25E-09	8.30E-09
9787	ITLN1	2.395082022	-1.336927659	0.491437	-2.720443	0.006519	0.013057
6761	TAS1R3	8.319128057	-1.337276644	0.341545	-3.915379	9.03E-05	0.000262
9149	ARSI	34.96123982	-1.337352008	0.452041	-2.958477	0.003092	0.006623
3525	SLA2	105.2045862	-1.337451752	0.236497	-5.655249	1.56E-08	8.65E-08
2368	FAM72B	49.06646264	-1.337781326	0.203004	-6.589917	4.40E-11	3.64E-10
627	TBXAS1	562.692743	-1.337841623	0.145061	-9.222633	2.90E-20	9.06E-19
2576	TMEM54	995.2342864	-1.33822492	0.208966	-6.404027	1.51E-10	1.15E-09
5310	RIBC2	9.808188766	-1.338393657	0.290022	-4.6148	3.93E-06	1.45E-05
7695	C1orf130	22.87271409	-1.338577908	0.37795	-3.541683	0.000398	0.001013
2120	C3AR1	595.4428636	-1.338657509	0.196007	-6.829628	8.51E-12	7.87E-11
2503	ERCC6L	27.38462115	-1.339066223	0.207342	-6.458249	1.06E-10	8.29E-10
11182	AZU1	1.572763854	-1.339203112	0.601112	-2.227877	0.025889	0.04538
3523	GGN	10.2562844	-1.339340868	0.236748	-5.657247	1.54E-08	8.56E-08
12462	OR7A5	1.15920638	-1.339707556	0.73819	-1.814855	0.069546	0.109387
14199	LCE1C	0.513718888	-1.340958385	1.066147	-1.257762	0.208478	0.287793
1497	CHST11	336.5529815	-1.341139322	0.177085	-7.573415	3.64E-14	4.76E-13

5023	PKNOX2	32.46559027	-1.341578714	0.28172	-4.762093	1.92E-06	7.48E-06
580	RPS20	19602.0467	-1.342033825	0.143135	-9.37601	6.85E-21	2.32E-19
1690	SEC14L2	532.5216488	-1.342245484	0.183677	-7.307655	2.72E-13	3.15E-12
1311	MTHFD2	769.5866627	-1.342372549	0.171032	-7.848654	4.21E-15	6.29E-14
2710	C19orf35	19.78152931	-1.343249162	0.214293	-6.268293	3.65E-10	2.64E-09
2256	SLA	887.072439	-1.343425143	0.200726	-6.692819	2.19E-11	1.90E-10
526	RPL37	13851.15247	-1.34377715	0.141028	-9.52847	1.60E-21	5.95E-20
10783	GPR150	1.645462976	-1.344002834	0.567835	-2.36689	0.017938	0.03261
8136	AANAT	1.115011712	-1.34416017	0.400294	-3.357934	0.000785	0.001892
1923	GALK1	554.1240078	-1.34477943	0.190458	-7.060771	1.66E-12	1.69E-11
2388	CD86	407.4172526	-1.344986544	0.204716	-6.570026	5.03E-11	4.13E-10
2841	TRAPPC5	753.5831845	-1.345018225	0.218661	-6.151156	7.69E-10	5.31E-09
1571	PRAF2	605.8730942	-1.345179746	0.180127	-7.46796	8.14E-14	1.02E-12
1696	APOBEC3C	458.7005587	-1.345903168	0.184481	-7.29562	2.97E-13	3.44E-12
6292	TRIM31	3.189915284	-1.346246446	0.325803	-4.132087	3.59E-05	0.000112
10207	POU5F2	0.693433654	-1.346254874	0.52188	-2.579625	0.009891	0.018994
7946	KCNJ4	14.1047407	-1.346311224	0.392586	-3.42934	0.000605	0.001493
4972	ST8SIA1	50.42372501	-1.346662732	0.28125	-4.788136	1.68E-06	6.64E-06
2807	CASS4	77.86014941	-1.347084863	0.217891	-6.182391	6.31E-10	4.41E-09
1404	TCIRG1	1147.588597	-1.347518971	0.174884	-7.705206	1.31E-14	1.82E-13
1424	IL23A	7.96895728	-1.347841692	0.1756	-7.67565	1.65E-14	2.27E-13
6434	POU5F1	398.4636864	-1.347928372	0.332346	-4.055796	5.00E-05	0.000152
2879	C19orf60	482.4665287	-1.348049474	0.220111	-6.124397	9.10E-10	6.20E-09
11913	CLC	0.638656259	-1.348124439	0.679351	-1.98443	0.047208	0.077673
3489	CMKLR1	590.2862767	-1.348466432	0.237321	-5.682045	1.33E-08	7.48E-08
12228	GLP2R	2.025557867	-1.348965179	0.714077	-1.889102	0.058878	0.094379
9296	ALAS2	24.35976254	-1.349537817	0.465153	-2.901276	0.003716	0.007836
3536	RDH16	5.564515783	-1.349920923	0.239181	-5.643931	1.66E-08	9.21E-08
1619	FCGR2A	1794.265728	-1.350442446	0.182408	-7.403431	1.33E-13	1.61E-12
5894	STAR	5.603034297	-1.350555672	0.312369	-4.323584	1.54E-05	5.11E-05
1407	GEFT	311.3400428	-1.350743139	0.17555	-7.694369	1.42E-14	1.98E-13
4315	ANKLE1	11.31613021	-1.351406014	0.262515	-5.147928	2.63E-07	1.20E-06
6174	CCR8	5.784237142	-1.351746195	0.322731	-4.188461	2.81E-05	8.92E-05
2323	NFE2L3	1008.167916	-1.351818835	0.203705	-6.636175	3.22E-11	2.72E-10
4007	GZMH	139.3293958	-1.352386306	0.253407	-5.336812	9.46E-08	4.63E-07
4734	NKG7	543.4233741	-1.353244051	0.275222	-4.916916	8.79E-07	3.64E-06
8366	CCDC135	13.32600703	-1.354551501	0.414843	-3.265216	0.001094	0.002563
689	NME4	1862.881639	-1.355563339	0.149907	-9.042679	1.53E-19	4.35E-18
7013	C1QTNF3	620.878426	-1.356265565	0.355242	-3.817864	0.000135	0.000376
7748	ESPNL	26.36862702	-1.356568891	0.385443	-3.519508	0.000432	0.001094
3339	OASL	244.9843941	-1.356623184	0.234224	-5.791995	6.96E-09	4.08E-08
11429	C19orf45	0.606998693	-1.357065664	0.634251	-2.139636	0.032384	0.055536
8595	C17orf64	0.993035323	-1.357680456	0.427503	-3.175839	0.001494	0.003407
8764	MIA	2.214994906	-1.358157043	0.438041	-3.100526	0.001932	0.00432
6566	ART4	28.03412341	-1.358268041	0.33974	-3.997969	6.39E-05	0.000191
898	GPX1	5575.603257	-1.360358095	0.159519	-8.527856	1.49E-17	3.25E-16
4761	TLR8	163.1227091	-1.360473326	0.27742	-4.904017	9.39E-07	3.87E-06

233	CCM2	1281.864795	-1.360535849	0.124012	-10.97099	5.27E-28	4.43E-26
1978	LHFPL2	1702.337381	-1.360958332	0.194628	-6.992616	2.70E-12	2.67E-11
5491	FOXF2	19.39856745	-1.36102045	0.300714	-4.525966	6.01E-06	2.15E-05
12346	TFF1	2.974628035	-1.361070335	0.73573	-1.84996	0.064319	0.102116
12800	C9orf53	0.343872972	-1.361784269	0.799463	-1.703374	0.088498	0.135513
9686	C2orf39	1.642661276	-1.361906444	0.495009	-2.751279	0.005936	0.012013
10536	FLJ45079	0.697950891	-1.362751134	0.554477	-2.457722	0.013982	0.026012
4979	AHRR	88.93535558	-1.363036055	0.284921	-4.783905	1.72E-06	6.77E-06
10272	FAM83E	1.340591434	-1.363130654	0.533313	-2.555969	0.010589	0.020206
2933	S1PR4	84.83519248	-1.363691104	0.224236	-6.081511	1.19E-09	7.96E-09
9511	GTSF1L	1.502887315	-1.363938065	0.484349	-2.816024	0.004862	0.01002
2193	ARHGAP4	961.0693474	-1.36421609	0.201683	-6.764166	1.34E-11	1.20E-10
4517	KIAA1324	67.11712672	-1.364614392	0.271532	-5.025605	5.02E-07	2.18E-06
4458	USH2A	15.18653311	-1.364686902	0.269508	-5.063629	4.11E-07	1.81E-06
6279	VSIG1	90.59443016	-1.364726087	0.329631	-4.140163	3.47E-05	0.000108
2749	PIK3R6	78.79075491	-1.365903089	0.2189	-6.239841	4.38E-10	3.12E-09
1747	LILRB2	330.9696487	-1.36688938	0.188612	-7.247096	4.26E-13	4.78E-12
280	BCL2L12	141.1716233	-1.367199275	0.127928	-10.68728	1.17E-26	8.17E-25
9685	KRT32	1.243078267	-1.367692912	0.497103	-2.751329	0.005935	0.012012
11093	FABP7	5441.474525	-1.370137567	0.607041	-2.257077	0.024003	0.042413
11358	LOC64532[illegible]	2.131513649	-1.370704794	0.633359	-2.164183	0.03045	0.052549
2351	HCFC1R1	2635.17527	-1.370965433	0.207491	-6.607353	3.91E-11	3.26E-10
6947	CRYBB2	5.138997981	-1.3709786	0.356503	-3.845632	0.00012	0.000339
4245	CCDC91	1263.365555	-1.371745994	0.264627	-5.183693	2.18E-07	1.00E-06
7557	SYT3	9.38150757	-1.372126377	0.381892	-3.592968	0.000327	0.000848
6608	RNASE10	2.520842111	-1.373077171	0.345011	-3.979804	6.90E-05	0.000205
5127	ASPHD1	250.6877549	-1.373569417	0.291336	-4.714731	2.42E-06	9.25E-06
6700	CACNA1F	26.7992879	-1.373871734	0.348591	-3.941218	8.11E-05	0.000237
1480	SASH3	526.4105126	-1.373937893	0.180821	-7.598311	3.00E-14	3.97E-13
927	FCHSD1	318.7143514	-1.374156201	0.162019	-8.481436	2.22E-17	4.70E-16
12080	SNORA74E	0.411189752	-1.375034031	0.711118	-1.933624	0.053159	0.086256
4000	C6orf132	39.94323065	-1.376189265	0.257712	-5.340019	9.29E-08	4.55E-07
9524	MYPN	1.074988734	-1.376560337	0.490132	-2.808552	0.004976	0.010242
5398	FLRT2	133.1101577	-1.37674354	0.301501	-4.566291	4.96E-06	1.80E-05
2115	CORO1A	1374.448503	-1.376860878	0.201531	-6.831995	8.37E-12	7.76E-11
574	SDC3	2516.528399	-1.377596444	0.146668	-9.392598	5.85E-21	2.00E-19
1677	IL2RB	527.1047679	-1.377624744	0.188018	-7.327105	2.35E-13	2.75E-12
2074	PON2	2615.790764	-1.377766596	0.200391	-6.87538	6.18E-12	5.84E-11
5382	BEND6	53.96528168	-1.378859509	0.301281	-4.576652	4.72E-06	1.72E-05
12363	SNORA74A	0.519658194	-1.37928526	0.746842	-1.846824	0.064773	0.102694
8629	SLITRK2	135.1383206	-1.379455205	0.43632	-3.161566	0.001569	0.003565
12804	PAGE2B	0.325153559	-1.379561756	0.810322	-1.702486	0.088664	0.135732
3935	LGR6	12.97312107	-1.379983678	0.256435	-5.381411	7.39E-08	3.68E-07
6213	ZNF541	48.55202312	-1.380423809	0.330911	-4.171586	3.02E-05	9.54E-05
8884	DUOXA1	1.660356155	-1.380473509	0.451338	-3.058625	0.002224	0.004906
4763	CNGA4	5.190269859	-1.380592238	0.281561	-4.903356	9.42E-07	3.88E-06
3145	ADORA2B	55.44230929	-1.380815353	0.233036	-5.925328	3.12E-09	1.94E-08

6285	GFAP	55.41382548	-1.381159932	0.333832	-4.137296	3.51E-05	0.00011
3930	KCNE1L	2.67251732	-1.381173044	0.256539	-5.383879	7.29E-08	3.64E-07
4523	ARRDC5	6.042754897	-1.381489743	0.275026	-5.023121	5.08E-07	2.20E-06
257	GMIP	416.0656154	-1.381643234	0.128004	-10.79378	3.68E-27	2.81E-25
137	RGS19	316.7400119	-1.381835511	0.117461	-11.76418	5.97E-32	8.54E-30
14549	PNPLA5	0.810481626	-1.382502504	1.201167	-1.150966	0.249746	0.336468
10786	CSF3	2.131196767	-1.382771012	0.584408	-2.366107	0.017976	0.032666
696	TRAM2	1166.064489	-1.383789527	0.153211	-9.031901	1.69E-19	4.75E-18
9683	CRNA0011	0.807623284	-1.38513656	0.5033	-2.752108	0.005921	0.011986
9228	LYG2	1.272243849	-1.385704695	0.473207	-2.928327	0.003408	0.007239
4449	SIGLEC8	168.093459	-1.386239618	0.273246	-5.073233	3.91E-07	1.72E-06
3485	GZMM	42.00723999	-1.386877997	0.244012	-5.683645	1.32E-08	7.42E-08
10711	MS4A15	1.071476468	-1.387019783	0.579442	-2.393718	0.016679	0.030522
9439	LOC285847	0.949258746	-1.387369625	0.488151	-2.842088	0.004482	0.009307
4912	WFIKKN1	100.1791847	-1.387810491	0.287852	-4.821261	1.43E-06	5.69E-06
986	CDC25B	1246.454586	-1.387825094	0.165891	-8.365878	5.97E-17	1.19E-15
7122	COL28A1	12.18277298	-1.388213116	0.367507	-3.777375	0.000158	0.000436
3669	OSR1	39.65023263	-1.388370351	0.249923	-5.555183	2.77E-08	1.48E-07
3780	SLC2A3	3936.041536	-1.388777656	0.253156	-5.485851	4.11E-08	2.13E-07
10710	SPIC	2.082785572	-1.389139612	0.580315	-2.393768	0.016676	0.03052
3148	CD180	161.1483978	-1.38937265	0.234515	-5.924463	3.13E-09	1.95E-08
129	MAN2B1	2817.204695	-1.389918371	0.116658	-11.91451	9.94E-33	1.51E-30
4341	LRGUK	17.54017133	-1.39117045	0.270803	-5.137196	2.79E-07	1.26E-06
1172	C7orf29	428.3787344	-1.392059048	0.172715	-8.059862	7.64E-16	1.28E-14
10702	SOX2	2.054561886	-1.392272517	0.580834	-2.397025	0.016529	0.030273
1282	PUSL1	136.960225	-1.392344139	0.176548	-7.886485	3.11E-15	4.75E-14
8456	CXorf65	1.647166972	-1.392526284	0.431564	-3.2267	0.001252	0.002903
1346	LILRB1	323.3486107	-1.392856937	0.178492	-7.80346	6.02E-15	8.77E-14
6635	CDX1	2.927063355	-1.393551295	0.351021	-3.969996	7.19E-05	0.000212
4722	XCL1	26.22113662	-1.394144872	0.283306	-4.920978	8.61E-07	3.57E-06
2588	IL12RB1	105.9888766	-1.39446723	0.218119	-6.393138	1.63E-10	1.23E-09
7230	FSCN2	10.18936661	-1.394916957	0.373562	-3.734096	0.000188	0.000511
4024	PDZD7	18.68979731	-1.395403904	0.261976	-5.326456	1.00E-07	4.88E-07
256	CMTM7	365.003514	-1.395577934	0.129227	-10.7994	3.46E-27	2.65E-25
2608	FAM54A	26.77909153	-1.395679278	0.219075	-6.370786	1.88E-10	1.41E-09
4478	COL8A1	1084.779472	-1.395801534	0.27622	-5.053221	4.34E-07	1.90E-06
1864	JOSD2	352.4453093	-1.395845448	0.195964	-7.122953	1.06E-12	1.11E-11
1879	CD300LB	41.62436525	-1.397110851	0.196503	-7.109874	1.16E-12	1.21E-11
8945	ONECUT2	41.30107418	-1.397846793	0.460728	-3.033993	0.002413	0.005288
2295	NME3	905.4331195	-1.398214302	0.209959	-6.659477	2.75E-11	2.35E-10
1796	CAPG	3841.372915	-1.399480793	0.19451	-7.19489	6.25E-13	6.82E-12
2888	SCARF2	218.9178395	-1.399969026	0.228696	-6.121519	9.27E-10	6.29E-09
1527	CD53	1394.701045	-1.40058478	0.186288	-7.518385	5.55E-14	7.12E-13
2299	SH2B2	67.40388356	-1.400592218	0.210406	-6.656605	2.80E-11	2.39E-10
2009	MICALL2	463.4403867	-1.400617469	0.201554	-6.9491	3.68E-12	3.59E-11
1559	IFITM2	4549.139071	-1.400915524	0.187287	-7.48003	7.43E-14	9.34E-13
7565	MMP1	143.471116	-1.400926133	0.390187	-3.5904	0.00033	0.000855

2453	KLRB1	123.665128	-1.401055649	0.215202	-6.510434	7.49E-11	5.99E-10
7083	HS3ST2	131.1028562	-1.401952754	0.369828	-3.790825	0.00015	0.000416
2899	FAM198A	20.40590959	-1.402548028	0.229432	-6.113128	9.77E-10	6.61E-09
1817	MAFB	1326.769919	-1.402669111	0.195558	-7.172662	7.36E-13	7.93E-12
5579	SPNS3	68.73905115	-1.402715148	0.312858	-4.483554	7.34E-06	2.58E-05
15121	RBPJL	0.286605602	-1.402769415	1.450355	-0.96719	0.333449	0.43227
4641	GSDMB	81.28804	-1.403004214	0.282439	-4.967458	6.78E-07	2.87E-06
11466	ATP2B3	2.154852177	-1.403646806	0.659067	-2.129748	0.033192	0.056742
702	GPSM3	671.6012329	-1.40412316	0.155745	-9.015537	1.96E-19	5.47E-18
12789	OOEP	3.282157981	-1.404288607	0.822354	-1.707644	0.087702	0.134417
11340	TTC29	0.522579749	-1.40454939	0.647458	-2.16933	0.030058	0.051954
4423	UBASH3A	48.555984	-1.404571652	0.276052	-5.088076	3.62E-07	1.60E-06
922	LENG9	110.2360488	-1.405633223	0.165634	-8.486375	2.13E-17	4.53E-16
6590	CHST8	2.08180791	-1.405837674	0.352566	-3.98744	6.68E-05	0.000199
12587	SNORA57	0.549809802	-1.406250902	0.791276	-1.777194	0.075536	0.117628
2167	ADORA3	352.0834848	-1.406488482	0.206958	-6.796003	1.08E-11	9.73E-11
3243	TOP2A	669.7949822	-1.407282585	0.240244	-5.857727	4.69E-09	2.84E-08
3549	C10orf55	10.12910946	-1.407398144	0.249685	-5.636697	1.73E-08	9.57E-08
980	FTL	79459.07808	-1.407643691	0.16804	-8.376845	5.44E-17	1.09E-15
3838	LOC14582(	104.5158474	-1.408006868	0.258743	-5.441722	5.28E-08	2.69E-07
8360	C20orf106	1.055081181	-1.408060281	0.430996	-3.266995	0.001087	0.002548
9642	IL2	0.625914656	-1.409045672	0.509448	-2.76583	0.005678	0.011542
3306	ETV1	323.2733723	-1.40915681	0.242438	-5.812447	6.16E-09	3.65E-08
1074	AGAP2	158.1965605	-1.40919042	0.171832	-8.200957	2.38E-16	4.35E-15
1084	SC65	773.0248739	-1.410598385	0.172362	-8.183939	2.75E-16	4.97E-15
4510	GHRL	5.536244868	-1.411358591	0.280644	-5.029004	4.93E-07	2.14E-06
8657	MT1G	395.439588	-1.412296335	0.44862	-3.14809	0.001643	0.003721
7246	MYBPC3	2.382503096	-1.412379701	0.378889	-3.727684	0.000193	0.000523
1647	ARHGAP22	152.7189628	-1.412904567	0.19184	-7.365021	1.77E-13	2.11E-12
9305	NOG	62.56482851	-1.412954229	0.487337	-2.899339	0.00374	0.007877
1474	RTN2	165.4583662	-1.413281124	0.185861	-7.603977	2.87E-14	3.82E-13
479	RPS2	13694.27712	-1.413839875	0.145784	-9.69819	3.07E-22	1.26E-20
1319	NAT14	348.4608903	-1.414291908	0.180472	-7.836607	4.63E-15	6.88E-14
2625	TMPRSS13	10.92219752	-1.414852152	0.222695	-6.353327	2.11E-10	1.57E-09
6519	FER1L4	441.465926	-1.415219349	0.352111	-4.019247	5.84E-05	0.000176
1806	IL10RA	1107.44834	-1.415328231	0.197028	-7.183399	6.80E-13	7.38E-12
13912	OTOS	0.92907774	-1.415649767	1.051063	-1.346875	0.178021	0.250818
8946	OR2L13	0.723586585	-1.416076263	0.46675	-3.03391	0.002414	0.005289
3264	RAD54B	123.1200889	-1.416565522	0.242575	-5.83971	5.23E-09	3.14E-08
4842	GBP5	713.1415731	-1.416883287	0.290965	-4.869597	1.12E-06	4.53E-06
11374	C1QTNF8	0.724336823	-1.417149719	0.65612	-2.159894	0.030781	0.053041
4828	GSC	5.608848583	-1.419885018	0.291151	-4.876795	1.08E-06	4.38E-06
44	PLIN3	1576.577613	-1.421070162	0.10691	-13.29227	2.57E-40	1.14E-37
7684	ROR2	263.1287461	-1.421091015	0.400829	-3.545383	0.000392	0.001
4283	SCN3B	95.969344	-1.421194336	0.275182	-5.164562	2.41E-07	1.10E-06
2206	NCAPG	152.1642526	-1.421360465	0.210595	-6.749269	1.49E-11	1.32E-10
3410	CKAP2L	59.30882821	-1.421426569	0.247595	-5.740932	9.42E-09	5.41E-08

2079	IFITM1	2912.42909	-1.421549923	0.206842	-6.872653	6.30E-12	5.94E-11
3002	SHCBP1	125.4342596	-1.422623112	0.235867	-6.031459	1.62E-09	1.06E-08
964	TGFB1	3201.417802	-1.422733997	0.168943	-8.421363	3.72E-17	7.57E-16
3001	TYMP	2353.59845	-1.422901346	0.235905	-6.031683	1.62E-09	1.06E-08
2855	SPAG4	826.2630262	-1.423686728	0.231851	-6.140534	8.22E-10	5.65E-09
6507	PTCHD2	6.651483218	-1.424009535	0.353853	-4.024292	5.71E-05	0.000172
9654	IFITM4P	0.662664473	-1.424903091	0.515744	-2.762811	0.005731	0.011635
1898	AMICA1	354.3858317	-1.425271469	0.201083	-7.087992	1.36E-12	1.40E-11
2095	44075	200.9406267	-1.425807572	0.208071	-6.852511	7.26E-12	6.79E-11
7672	C1orf173	11.69101446	-1.426228049	0.401711	-3.550385	0.000385	0.000983
3079	NCF1B	42.42429369	-1.426259716	0.238621	-5.977084	2.27E-09	1.45E-08
3654	ADAMTS1(	279.9564299	-1.428891413	0.25666	-5.567249	2.59E-08	1.39E-07
1567	S100A6	9901.62634	-1.430955782	0.191533	-7.47105	7.96E-14	9.95E-13
8778	DCC	2.742877442	-1.431069104	0.462689	-3.092943	0.001982	0.004425
5775	FAM129C	4.54478343	-1.431989514	0.326717	-4.382968	1.17E-05	3.97E-05
1685	ALOX5AP	360.8120375	-1.432155292	0.195817	-7.313752	2.60E-13	3.02E-12
1215	C7orf40	507.8104311	-1.432249159	0.179078	-7.997897	1.27E-15	2.04E-14
2806	PDLIM3	615.2692751	-1.432579297	0.231708	-6.182688	6.30E-10	4.40E-09
2329	IKBKE	235.5655892	-1.432946959	0.216216	-6.627383	3.42E-11	2.88E-10
4427	LTC4S	65.43559382	-1.432954878	0.281713	-5.086571	3.65E-07	1.61E-06
2861	POLQ	43.19351055	-1.434035628	0.233757	-6.134739	8.53E-10	5.84E-09
1738	RHBDL2	43.26475846	-1.434288699	0.197712	-7.254436	4.03E-13	4.55E-12
2548	C19orf24	804.534282	-1.434424931	0.223193	-6.426846	1.30E-10	1.00E-09
1872	NDC80	101.3037229	-1.434482876	0.201523	-7.118209	1.09E-12	1.14E-11
1093	C1orf59	98.40220627	-1.434602428	0.175569	-8.171142	3.05E-16	5.48E-15
819	MXD3	204.8861781	-1.434875981	0.16466	-8.714159	2.93E-18	7.01E-17
9925	MEP1A	1.626459747	-1.43601046	0.536023	-2.67901	0.007384	0.014583
2090	PRR19	13.62903985	-1.436665249	0.209568	-6.855365	7.11E-12	6.67E-11
13493	KCNK16	0.252363766	-1.436747414	0.96411	-1.490232	0.136163	0.197801
854	C1orf38	782.9725664	-1.437005422	0.166454	-8.633052	5.97E-18	1.37E-16
1436	OIP5	27.42574071	-1.437114065	0.187855	-7.650136	2.01E-14	2.74E-13
6184	PLXNA4	61.08601534	-1.437613557	0.343667	-4.183162	2.87E-05	9.11E-05
3034	TMEM160	102.5614192	-1.438696526	0.23944	-6.0086	1.87E-09	1.21E-08
3817	ASPM	185.6116289	-1.439422437	0.263484	-5.46304	4.68E-08	2.40E-07
2885	FABP5	117.1993657	-1.439635374	0.235129	-6.122739	9.20E-10	6.25E-09
1979	DDIT4	11726.66586	-1.439657054	0.205949	-6.99035	2.74E-12	2.72E-11
6192	LOC73066(	3.260549258	-1.440164274	0.344669	-4.178399	2.94E-05	9.29E-05
777	RRAS	1357.5362	-1.44036808	0.163291	-8.820884	1.14E-18	2.86E-17
5231	TSLP	11.81686354	-1.440485389	0.3089	-4.663279	3.11E-06	1.17E-05
4071	FRRS1	146.0111651	-1.440936804	0.272127	-5.295082	1.19E-07	5.73E-07
2405	CORO2A	174.3363913	-1.441280221	0.219975	-6.552019	5.68E-11	4.63E-10
8288	IGJ	4002.932146	-1.442163919	0.4373	-3.297882	0.000974	0.002304
5972	GJA3	8.416840377	-1.442821015	0.337035	-4.280924	1.86E-05	6.11E-05
7446	SMC1B	3.865229894	-1.443257007	0.396648	-3.638631	0.000274	0.000722
10534	LOC28340	0.531622395	-1.443986938	0.587177	-2.459204	0.013925	0.02591
7275	TFF3	105.5260495	-1.444147197	0.389009	-3.71237	0.000205	0.000553
628	APOL2	2455.404236	-1.444822565	0.156721	-9.219087	3.00E-20	9.35E-19

907	FSCN1	2215.95183	-1.445940962	0.169925	-8.509307	1.75E-17	3.78E-16
1615	PXDN	3262.278764	-1.446792624	0.19529	-7.408436	1.28E-13	1.55E-12
3221	SGOL1	18.00705903	-1.448334107	0.246608	-5.873025	4.28E-09	2.60E-08
2738	CD5	158.4306461	-1.448602809	0.231836	-6.248392	4.15E-10	2.97E-09
1554	MICAL2	1223.032633	-1.448940228	0.193491	-7.488397	6.97E-14	8.79E-13
2504	CCDC3	551.6515193	-1.449062709	0.224382	-6.458009	1.06E-10	8.30E-10
1752	MAP6D1	56.12917313	-1.449517347	0.200164	-7.241664	4.43E-13	4.96E-12
3158	TBC1D10C	174.473949	-1.449558009	0.245164	-5.912604	3.37E-09	2.09E-08
6626	CYP21A2	148.5046679	-1.44966979	0.364776	-3.974134	7.06E-05	0.000209
11807	INS-IGF2	1.545948407	-1.450628554	0.718431	-2.019163	0.04347	0.072166
3844	TIGIT	96.9643058	-1.451410417	0.266956	-5.436899	5.42E-08	2.76E-07
2053	RECQL4	111.0421972	-1.452092673	0.21048	-6.898956	5.24E-12	5.00E-11
6580	AMZ1	48.76735287	-1.453077856	0.364087	-3.991015	6.58E-05	0.000196
5253	LANCL3	4.421542596	-1.453138475	0.312505	-4.649974	3.32E-06	1.24E-05
3459	C14orf182	6.02646677	-1.45399752	0.255104	-5.699623	1.20E-08	6.80E-08
847	ZNHIT1	1736.403268	-1.454180571	0.168318	-8.639504	5.65E-18	1.31E-16
8149	TNFSF11	12.24375211	-1.454848721	0.43398	-3.352342	0.000801	0.001927
12576	INSL5	0.454545017	-1.455787016	0.818061	-1.779558	0.075148	0.117126
3954	CCDC74B	144.8041754	-1.455893063	0.270916	-5.373969	7.70E-08	3.82E-07
5625	NNMT	21229.27609	-1.456263404	0.326597	-4.458896	8.24E-06	2.87E-05
1784	C21orf56	119.194262	-1.457266525	0.202298	-7.203555	5.87E-13	6.45E-12
3559	ASGR1	25.60467065	-1.457320378	0.258921	-5.628446	1.82E-08	1.00E-07
1375	LOC654342	795.5479614	-1.45774883	0.187837	-7.760701	8.45E-15	1.20E-13
1415	PARVG	441.1951208	-1.457750045	0.189745	-7.682672	1.56E-14	2.16E-13
3375	FHAD1	18.99285128	-1.458635811	0.253119	-5.762652	8.28E-09	4.81E-08
4598	FMO3	341.0617835	-1.458817472	0.292441	-4.988416	6.09E-07	2.59E-06
4225	RPLP0P2	17.20169843	-1.459614219	0.280924	-5.19576	2.04E-07	9.46E-07
4002	LY6E	6674.059442	-1.460021073	0.273435	-5.339556	9.32E-08	4.56E-07
615	LAGE3	258.8359972	-1.460493387	0.157506	-9.272606	1.82E-20	5.79E-19
5161	EFEMP1	3118.835453	-1.461201728	0.311295	-4.693942	2.68E-06	1.02E-05
11622	ARL5C	0.475938363	-1.461373573	0.705482	-2.071455	0.038316	0.064622
12643	LRRC18	0.483934184	-1.461435225	0.830778	-1.759117	0.078558	0.121791
1130	IL15RA	403.0788686	-1.461503838	0.180072	-8.116232	4.81E-16	8.34E-15
1505	ITGAM	508.450719	-1.461709088	0.193398	-7.558019	4.09E-14	5.33E-13
1913	GPR19	12.13331049	-1.463129339	0.206806	-7.074885	1.50E-12	1.53E-11
2871	C19orf76	21.57890418	-1.463249974	0.238731	-6.129282	8.83E-10	6.03E-09
15991	FAM25A	0.27670052	-1.463864557	2.047877	-0.71482	0.47472	0.581889
1080	NFAM1	351.835445	-1.46518214	0.178896	-8.190149	2.61E-16	4.74E-15
2236	DOCK2	718.3224003	-1.465363871	0.21821	-6.715382	1.88E-11	1.64E-10
8436	GPR15	4.451177253	-1.465410606	0.45317	-3.233685	0.001222	0.002839
12765	CCL24	0.651470458	-1.465946856	0.854959	-1.714641	0.086411	0.132687
8069	LOC400696	17.33347076	-1.466192983	0.433221	-3.384402	0.000713	0.001733
2605	SNX20	71.06185307	-1.466227557	0.230063	-6.373154	1.85E-10	1.39E-09
3830	CD3D	199.57228	-1.466352262	0.269218	-5.446703	5.13E-08	2.63E-07
7999	CFHR3	6.671510808	-1.466580404	0.430058	-3.410195	0.000649	0.001591
10025	FIBCD1	116.4418543	-1.467192249	0.554948	-2.643836	0.008197	0.016027
9182	CBLC	39.99727503	-1.467846083	0.498272	-2.945873	0.00322	0.006875

1442	CD33	134.7696861	-1.467890286	0.192083	-7.641943	2.14E-14	2.91E-13
3595	LOC91450	5.518827751	-1.467905737	0.261788	-5.607223	2.06E-08	1.12E-07
2589	KIF7	106.4806473	-1.46831232	0.229672	-6.393083	1.63E-10	1.23E-09
1732	C16orf13	1347.252539	-1.46927218	0.20221	-7.266088	3.70E-13	4.19E-12
3055	CD80	28.68381783	-1.469389827	0.245158	-5.993654	2.05E-09	1.32E-08
2988	FBXO43	12.26409926	-1.469718093	0.243142	-6.044696	1.50E-09	9.82E-09
3216	CD3E	561.0521725	-1.469734686	0.250089	-5.876844	4.18E-09	2.55E-08
2383	IRF7	597.4411814	-1.469792697	0.223561	-6.574446	4.88E-11	4.02E-10
1238	MYO1F	719.1311597	-1.469867045	0.18507	-7.942239	1.99E-15	3.14E-14
6208	GRIK2	5.226369281	-1.470426145	0.352325	-4.173491	3.00E-05	9.47E-05
12148	AWAT2	0.390048917	-1.470978569	0.769147	-1.91248	0.055815	0.090058
1458	BEST1	129.2232415	-1.471180891	0.192904	-7.626473	2.41E-14	3.24E-13
772	CD99	3026.070383	-1.471431343	0.166671	-8.828336	1.06E-18	2.70E-17
12465	SLC39A12	0.348491677	-1.471581279	0.811561	-1.813271	0.06979	0.109744
4150	S100A8	250.7351322	-1.47202092	0.280295	-5.251687	1.51E-07	7.12E-07
5935	SPTA1	4.502233967	-1.472069702	0.342284	-4.300729	1.70E-05	5.62E-05
2671	PRKCDBP	1075.964909	-1.472285419	0.233292	-6.310902	2.77E-10	2.04E-09
6070	SEMA3A	73.2945761	-1.472289229	0.347528	-4.23646	2.27E-05	7.33E-05
5202	NRN1L	5.051510412	-1.472416915	0.314813	-4.677115	2.91E-06	1.10E-05
2560	NOD2	98.02102694	-1.472891585	0.229549	-6.416452	1.39E-10	1.07E-09
45	ANXA2	11411.2323	-1.473144233	0.110957	-13.2767	3.16E-40	1.38E-37
1070	GSG2	18.69018017	-1.473630238	0.179577	-8.206138	2.28E-16	4.18E-15
4106	RAB40AL	6.847416715	-1.473744704	0.279177	-5.278895	1.30E-07	6.20E-07
5200	GJB5	5.494994863	-1.473791927	0.315094	-4.677308	2.91E-06	1.10E-05
2252	MC1R	152.6452633	-1.474064781	0.220204	-6.694077	2.17E-11	1.89E-10
5911	B3GNT4	181.333	-1.474182544	0.341507	-4.316692	1.58E-05	5.25E-05
2496	SPSB1	1278.660579	-1.474719382	0.228028	-6.467262	9.98E-11	7.84E-10
8178	MEIG1	0.815881251	-1.474934732	0.441172	-3.343219	0.000828	0.001985
934	FMNL1	692.78783	-1.47526861	0.174087	-8.474336	2.36E-17	4.96E-16
312	PLOD3	3029.478411	-1.475301027	0.140891	-10.47123	1.17E-25	7.36E-24
13813	VAX1	0.343662369	-1.475769841	1.067453	-1.382515	0.166814	0.236713
6198	SLC4A10	7.069487185	-1.476132751	0.353415	-4.17677	2.96E-05	9.35E-05
7925	PF4V1	37.25904273	-1.476822424	0.429534	-3.438194	0.000586	0.001448
7940	SCARNA5	4.382079075	-1.477109099	0.430336	-3.432454	0.000598	0.001477
4644	RNF112	20.21327346	-1.477229289	0.297549	-4.964654	6.88E-07	2.90E-06
1788	FBLIM1	1069.223716	-1.477532408	0.205226	-7.199534	6.04E-13	6.62E-12
687	CD37	575.7902678	-1.47760264	0.163376	-9.04417	1.51E-19	4.30E-18
4796	TIFAB	7.771428905	-1.478514479	0.302417	-4.888997	1.01E-06	4.14E-06
943	SSBP4	676.8111078	-1.478885373	0.174855	-8.45778	2.73E-17	5.66E-16
1416	MS4A6A	1664.769822	-1.479752564	0.192669	-7.680282	1.59E-14	2.20E-13
5154	CDA	377.5577668	-1.479998199	0.314951	-4.699137	2.61E-06	9.94E-06
9822	CCL25	1.177549144	-1.480469546	0.546169	-2.710643	0.006715	0.013401
7362	IGF2BP2	239.3132632	-1.480651913	0.403111	-3.673066	0.00024	0.000638
5537	C1orf186	426.3734306	-1.481985088	0.329084	-4.503365	6.69E-06	2.37E-05
8612	NDST3	5.080568458	-1.482107542	0.467586	-3.169697	0.001526	0.003473
10299	MAT1A	130.5551129	-1.482509675	0.582167	-2.546539	0.01088	0.020706
5800	CCL23	4.251619554	-1.482965107	0.339025	-4.374204	1.22E-05	4.12E-05

7421	TAT	2.285891695	-1.485246804	0.407047	-3.648832	0.000263	0.000696
10913	TFAMP1	0.575799131	-1.485334268	0.641267	-2.316249	0.020545	0.036901
4700	ASPN	435.3850332	-1.485979664	0.301436	-4.929661	8.24E-07	3.44E-06
1876	PLEK	829.8583315	-1.486166148	0.208994	-7.11104	1.15E-12	1.20E-11
4686	TTYH1	11.79880546	-1.487806204	0.301402	-4.936288	7.96E-07	3.33E-06
14260	OR12D2	0.367536983	-1.488170731	1.199441	-1.24072	0.214709	0.295127
5942	FRZB	2773.976984	-1.488503645	0.346443	-4.296532	1.73E-05	5.72E-05
5103	TMEM90B	183.5332747	-1.489688452	0.315169	-4.726629	2.28E-06	8.77E-06
844	TMEM180	99.94807076	-1.490097487	0.172319	-8.647326	5.27E-18	1.22E-16
4668	ELN	768.2090961	-1.490344416	0.300982	-4.951606	7.36E-07	3.09E-06
7053	S100P	8.938071018	-1.490456263	0.392248	-3.799784	0.000145	0.000402
7747	SLC25A2	1.384975045	-1.490554542	0.423466	-3.519888	0.000432	0.001092
12569	ARHGAP3(	0.893217297	-1.491255739	0.837034	-1.781594	0.074815	0.116673
2311	HSPB1	14784.15635	-1.491527626	0.224437	-6.645646	3.02E-11	2.56E-10
5682	SFTA1P	22.45378993	-1.491943689	0.336795	-4.429831	9.43E-06	3.25E-05
10784	MYL2	0.346133772	-1.493388158	0.631034	-2.366575	0.017954	0.032632
1015	FLJ36031	423.4772199	-1.493399817	0.179617	-8.314366	9.22E-17	1.78E-15
1599	DOK2	218.6296656	-1.493990196	0.201283	-7.42232	1.15E-13	1.41E-12
8740	PTHLH	1698.678543	-1.494209359	0.480101	-3.112282	0.001856	0.004163
1529	KIF23	128.1549368	-1.494329867	0.198773	-7.517759	5.57E-14	7.14E-13
3108	FOXS1	151.5800994	-1.495558967	0.251053	-5.957136	2.57E-09	1.62E-08
9990	FAM163B	0.823364014	-1.495647143	0.563313	-2.65509	0.007929	0.015555
4463	SH2D4B	4.294077047	-1.496210521	0.295651	-5.060731	4.18E-07	1.83E-06
710	ZNF385A	673.3498581	-1.496497178	0.166531	-8.986292	2.56E-19	7.06E-18
8760	GDF5	13.62476381	-1.498113627	0.482967	-3.101896	0.001923	0.004303
2304	LILRA6	112.4421758	-1.498801481	0.225396	-6.649651	2.94E-11	2.50E-10
4008	MFSD2B	7.237000153	-1.499213139	0.280929	-5.336624	9.47E-08	4.63E-07
903	LOC541471	509.5474433	-1.499665679	0.176133	-8.514404	1.67E-17	3.63E-16
7007	MACROD2	378.4182642	-1.50027357	0.39273	-3.820117	0.000133	0.000373
16160	DSCR4	0.401722884	-1.500814913	2.260324	-0.663982	0.506702	0.614595
6175	CEACAM3	3.579083771	-1.501978063	0.358664	-4.187698	2.82E-05	8.94E-05
1203	PTGFRN	1811.286841	-1.502148241	0.187508	-8.011102	1.14E-15	1.85E-14
6289	WNT3A	2.695839476	-1.503594189	0.363648	-4.134746	3.55E-05	0.000111
9744	ANKRD34C	1.085011441	-1.503680972	0.550009	-2.733921	0.006259	0.01259
2757	TMEM150B	25.9137326	-1.506056184	0.241555	-6.234833	4.52E-10	3.22E-09
3544	DLGAP3	11.81805963	-1.506609044	0.267134	-5.639908	1.70E-08	9.41E-08
1278	UBE2S	211.4813969	-1.506741957	0.190877	-7.893792	2.93E-15	4.50E-14
5686	PDPN	89.64161674	-1.507448051	0.340464	-4.427631	9.53E-06	3.28E-05
2601	RASL10B	81.20613668	-1.507680811	0.236293	-6.380553	1.76E-10	1.33E-09
2183	CTGF	4230.755238	-1.509026138	0.222761	-6.774208	1.25E-11	1.12E-10
11764	MUC2	0.603198926	-1.509361893	0.742868	-2.031805	0.042173	0.070269
15680	KCNA10	0.264905283	-1.510729457	1.858785	-0.812751	0.416361	0.520477
972	ASF1B	170.9107684	-1.510866506	0.180036	-8.392024	4.78E-17	9.64E-16
1163	PAFAH1B3	311.9460045	-1.511297192	0.187249	-8.071045	6.97E-16	1.17E-14
6913	PRND	122.79737	-1.512340727	0.391952	-3.858483	0.000114	0.000323
4589	RRAD	1922.811283	-1.513498575	0.303175	-4.992154	5.97E-07	2.55E-06
4991	EYA2	110.0201237	-1.514032353	0.316858	-4.778266	1.77E-06	6.94E-06

1652	ECE2	129.849819	-1.514710376	0.205921	-7.355778	1.90E-13	2.25E-12
3798	ASGR2	30.62382078	-1.51531897	0.276778	-5.474859	4.38E-08	2.26E-07
6915	AMH	9.763911905	-1.515350031	0.392862	-3.857204	0.000115	0.000325
544	SRM	1013.003296	-1.515619176	0.159922	-9.477253	2.61E-21	9.41E-20
11363	CARD17	0.329516103	-1.515759967	0.700821	-2.162836	0.030554	0.052705
1205	PSMB10	1671.56965	-1.51596361	0.18928	-8.009095	1.16E-15	1.88E-14
4930	CORO2B	134.4684383	-1.516482715	0.3152	-4.811172	1.50E-06	5.97E-06
663	HCK	562.8685496	-1.516989146	0.166196	-9.127722	7.00E-20	2.07E-18
1209	LY86	279.946511	-1.517061151	0.189561	-8.003021	1.21E-15	1.97E-14
12690	KLF14	0.308074224	-1.517284576	0.872756	-1.738499	0.082123	0.126847
1133	TGFB3	503.5488026	-1.518100135	0.187134	-8.112354	4.96E-16	8.60E-15
2514	COL12A1	1917.814768	-1.518118096	0.235365	-6.450071	1.12E-10	8.72E-10
8987	FOLH1B	11.86620126	-1.518763732	0.503052	-3.019097	0.002535	0.00553
1756	CD300LF	109.8017153	-1.519478214	0.209924	-7.238218	4.55E-13	5.07E-12
10371	FADS6	1.908252784	-1.519548952	0.603963	-2.515962	0.011871	0.022436
7034	RELN	74.57941861	-1.519975274	0.399359	-3.80604	0.000141	0.000393
4278	METRN	297.8663168	-1.520125291	0.294161	-5.167658	2.37E-07	1.09E-06
778	FAM113B	216.3948524	-1.520331945	0.172401	-8.818599	1.16E-18	2.92E-17
4045	CLEC4E	133.246476	-1.520781104	0.286482	-5.308469	1.11E-07	5.36E-07
7021	HIST1H1D	1.935349612	-1.521211711	0.398766	-3.814798	0.000136	0.000381
1803	SKA3	57.66613656	-1.521831062	0.211737	-7.187371	6.61E-13	7.18E-12
2023	LYL1	142.2703743	-1.522113153	0.219467	-6.935514	4.05E-12	3.92E-11
2472	CXCR6	154.8919766	-1.523294662	0.234458	-6.497088	8.19E-11	6.49E-10
9772	SLC28A3	1.060742258	-1.524087611	0.559438	-2.72432	0.006443	0.012923
3274	BUB1	176.2232146	-1.524554622	0.261389	-5.832516	5.46E-09	3.27E-08
6315	HBA2	2710.407024	-1.524747047	0.370241	-4.118257	3.82E-05	0.000118
1055	C21orf7	593.6332612	-1.524793899	0.185143	-8.235761	1.78E-16	3.31E-15
7677	C21orf96	0.931571262	-1.525133018	0.429865	-3.547935	0.000388	0.000991
2530	SLAMF1	39.7635956	-1.525722285	0.236957	-6.43881	1.20E-10	9.33E-10
700	PPP1R14B	1252.521958	-1.526842883	0.169216	-9.023066	1.83E-19	5.12E-18
2156	MS4A4A	575.4522605	-1.527770173	0.224498	-6.805287	1.01E-11	9.17E-11
3352	LC22A18A	73.63278139	-1.528072422	0.264422	-5.778909	7.52E-09	4.40E-08
1241	KCNS3	469.8881472	-1.528520512	0.19253	-7.939136	2.04E-15	3.22E-14
2210	FLJ23867	604.4807767	-1.528843923	0.226645	-6.745549	1.52E-11	1.35E-10
4294	PADI2	382.623394	-1.529649399	0.296461	-5.159692	2.47E-07	1.13E-06
1274	ACTA2	10200.99702	-1.530365773	0.193829	-7.895429	2.89E-15	4.45E-14
2876	HMMR	120.8330365	-1.53087542	0.249926	-6.125307	9.05E-10	6.17E-09
2926	DLX5	50.36339103	-1.530887409	0.251252	-6.093036	1.11E-09	7.42E-09
1191	ITGB2	3201.188403	-1.530935437	0.190733	-8.026601	1.00E-15	1.65E-14
10777	HCN1	1.507552368	-1.530943717	0.645368	-2.372202	0.017682	0.03216
6571	SPIN2A	2.117233468	-1.531313363	0.383409	-3.993944	6.50E-05	0.000194
4404	KCNJ5	135.3046199	-1.53171591	0.300251	-5.101448	3.37E-07	1.50E-06
2681	CENPM	80.8900949	-1.532125756	0.243333	-6.296407	3.05E-10	2.23E-09
3003	EPHX3	43.51621151	-1.533395245	0.254245	-6.031182	1.63E-09	1.06E-08
656	PILRA	263.9669991	-1.53343798	0.167465	-9.156761	5.35E-20	1.60E-18
7318	C20orf103	48.07267081	-1.533497459	0.415281	-3.692678	0.000222	0.000594
4344	C2orf48	3.913921028	-1.534136698	0.298747	-5.135243	2.82E-07	1.27E-06

1120	E2F2	44.46836456	-1.534693682	0.188813	-8.128101	4.36E-16	7.63E-15
3490	AG2	1019.552012	-1.535140076	0.270177	-5.68197	1.33E-08	7.48E-08
6116	H2AFB1	1.907914264	-1.535870428	0.364575	-4.212765	2.52E-05	8.08E-05
11184	LHX8	5.939497232	-1.536344592	0.68995	-2.226747	0.025964	0.045505
2520	SULF1	2774.346191	-1.536479975	0.238356	-6.446162	1.15E-10	8.92E-10
10438	NMUR2	0.964135067	-1.536583761	0.615098	-2.498114	0.012486	0.023444
1725	UNC13D	269.7848688	-1.537953694	0.211526	-7.270746	3.58E-13	4.06E-12
1545	CDC42EP5	112.591006	-1.538838142	0.205182	-7.499881	6.39E-14	8.10E-13
12448	NPFFR1	0.45142773	-1.539015043	0.845236	-1.820812	0.068635	0.108075
3925	OPN1SW	1.939257688	-1.539134954	0.28573	-5.386684	7.18E-08	3.58E-07
8807	LOC283731	1.461722571	-1.539354555	0.499073	-3.084428	0.002039	0.004539
7915	PNCK	2734.265116	-1.539828449	0.447198	-3.443284	0.000575	0.001423
7250	HRNBP3	2.451523645	-1.540041335	0.413418	-3.725143	0.000195	0.000528
632	CD4	2182.274737	-1.540267907	0.167345	-9.204159	3.44E-20	1.07E-18
4919	IL22RA1	174.5805164	-1.540384695	0.319713	-4.818018	1.45E-06	5.78E-06
10694	ARL14	7.698231208	-1.542184069	0.642771	-2.399273	0.016428	0.030107
2421	SNHG3	54.16057972	-1.542207956	0.235803	-6.540228	6.14E-11	4.97E-10
14858	SPRR2E	0.359260343	-1.54311898	1.469098	-1.050385	0.293541	0.387262
1925	ORC1L	44.55522961	-1.54413584	0.218709	-7.060239	1.66E-12	1.69E-11
36	GLT25D1	3439.995179	-1.544257073	0.114611	-13.47388	2.23E-41	1.21E-38
1941	STK32C	130.1466124	-1.545280936	0.219441	-7.041885	1.90E-12	1.92E-11
3710	MLLT11	205.1673166	-1.545528812	0.279396	-5.531686	3.17E-08	1.68E-07
8333	SPINK1	48.35490833	-1.546327455	0.471561	-3.27917	0.001041	0.002449
4659	POM121L9P	11.9370973	-1.547633232	0.312285	-4.955841	7.20E-07	3.03E-06
1419	C13orf18	89.09206855	-1.54811199	0.201612	-7.67867	1.61E-14	2.22E-13
3717	VPREB3	10.21599952	-1.548664171	0.280179	-5.527409	3.25E-08	1.71E-07
2544	SNAI1	218.0979403	-1.54924006	0.240997	-6.428469	1.29E-10	9.93E-10
928	C5AR1	600.5426385	-1.54948585	0.182716	-8.48028	2.25E-17	4.75E-16
11471	TBC1D3G	26.62858456	-1.550784534	0.728555	-2.128576	0.033289	0.056883
8578	FAM180B	0.872770656	-1.553031321	0.48831	-3.180418	0.001471	0.00336
4850	SLC7A5	2497.294875	-1.553229765	0.31923	-4.865553	1.14E-06	4.61E-06
1612	NME1-NME	80.07705378	-1.553388316	0.209617	-7.410601	1.26E-13	1.53E-12
5455	CCDC64B	159.357968	-1.553611682	0.342186	-4.540249	5.62E-06	2.02E-05
7158	STK31	2.334638866	-1.554427718	0.41333	-3.760742	0.000169	0.000464
6920	GOLGA9P	12.99545824	-1.554593779	0.403097	-3.856627	0.000115	0.000326
11919	GRIN1	1.555587809	-1.554720902	0.784305	-1.982292	0.047447	0.07802
1006	RPL13	25706.3349	-1.554829671	0.186475	-8.338016	7.55E-17	1.47E-15
13422	SSX6	0.383581316	-1.555589893	1.031889	-1.507516	0.131678	0.192298
1271	STAB1	3222.527863	-1.555714684	0.196938	-7.899497	2.80E-15	4.32E-14
186	TACC3	392.642642	-1.555872412	0.137135	-11.34553	7.81E-30	8.23E-28
9973	OC1001280	3.986274401	-1.556175584	0.584471	-2.662539	0.007755	0.015242
2029	BST2	3397.316715	-1.556642512	0.224639	-6.929532	4.22E-12	4.08E-11
8251	DIO3	11.7545859	-1.556711994	0.469871	-3.313063	0.000923	0.002192
75	CTSZ	8068.996228	-1.556871457	0.123791	-12.57657	2.84E-36	7.42E-34
422	GPR132	132.245351	-1.556909936	0.156365	-9.956894	2.35E-23	1.09E-21
4139	PREX2	508.216289	-1.556926993	0.296002	-5.259848	1.44E-07	6.83E-07
8600	TM4SF4	29.74880703	-1.558805957	0.490981	-3.174881	0.001499	0.003416

12290	MGAT4C	0.712984709	-1.559624979	0.834617	-1.868672	0.061669	0.098356
3288	ANXA2P3	4.752696421	-1.5601386	0.267934	-5.822853	5.79E-09	3.45E-08
969	TRIP13	153.0492189	-1.56016573	0.18569	-8.401973	4.39E-17	8.88E-16
1963	MELK	97.89274	-1.560612811	0.222445	-7.015731	2.29E-12	2.28E-11
1839	SLC2A1	7902.225142	-1.561573724	0.21844	-7.148753	8.76E-13	9.33E-12
4491	C1QTNF4	4.887519027	-1.562368879	0.309646	-5.045669	4.52E-07	1.97E-06
3501	MMP28	105.2448499	-1.562761635	0.275464	-5.673197	1.40E-08	7.85E-08
14273	SPACA3	0.295758254	-1.563928779	1.265273	-1.23604	0.216444	0.29724
734	CYTH4	527.7757053	-1.565368247	0.175316	-8.92882	4.31E-19	1.15E-17
9875	FOXA2	30.58427276	-1.565462581	0.58071	-2.695772	0.007023	0.013939
4923	CTXN1	71.72965023	-1.565479071	0.325056	-4.81603	1.46E-06	5.83E-06
1342	RHEBL1	17.1089736	-1.566697451	0.200677	-7.80707	5.85E-15	8.55E-14
10916	SERPINB5	1.280415763	-1.567710732	0.677482	-2.314025	0.020666	0.037109
2408	MYOZ3	22.92248836	-1.567721412	0.239304	-6.551172	5.71E-11	4.65E-10
2921	DC1001446	9.745873506	-1.568479013	0.257302	-6.095871	1.09E-09	7.30E-09
2741	CENPI	17.61097681	-1.569260961	0.251345	-6.24345	4.28E-10	3.06E-09
3210	RAET1K	3.722968799	-1.569813338	0.267002	-5.879406	4.12E-09	2.51E-08
216	P4HB	32952.80672	-1.570811028	0.142093	-11.05478	2.08E-28	1.89E-26
472	C17orf53	44.27878115	-1.571442267	0.161432	-9.73439	2.15E-22	8.93E-21
566	SELPLG	724.0874559	-1.571917893	0.16699	-9.413243	4.81E-21	1.67E-19
2443	TBC1D3B	190.4550332	-1.571988937	0.241263	-6.515662	7.24E-11	5.81E-10
9456	CES4	3.038545229	-1.57268323	0.55454	-2.836013	0.004568	0.009469
2140	LOC92659	45.37476297	-1.572905285	0.230837	-6.813931	9.50E-12	8.70E-11
4898	WNT10A	30.36227481	-1.573505557	0.325919	-4.827904	1.38E-06	5.52E-06
3157	LTBP2	1787.59335	-1.573594544	0.266134	-5.912798	3.36E-09	2.09E-08
8063	CASKIN1	3.437720989	-1.573751404	0.464586	-3.387425	0.000706	0.001715
7553	MAP7D2	1117.114323	-1.573902389	0.437879	-3.594379	0.000325	0.000844
13400	C1orf94	0.298698481	-1.574181154	1.041192	-1.511903	0.130559	0.190971
4505	ENKUR	39.7220688	-1.574988974	0.312883	-5.033796	4.81E-07	2.09E-06
1489	C1orf162	543.9168134	-1.575699151	0.207702	-7.586357	3.29E-14	4.33E-13
3337	CD84	240.8072868	-1.576471144	0.272112	-5.793462	6.90E-09	4.05E-08
2534	NEIL3	23.22522583	-1.576689997	0.245036	-6.434512	1.24E-10	9.58E-10
3701	DEPDC1	86.80860773	-1.577318774	0.284951	-5.53541	3.11E-08	1.64E-07
3946	BCL2L14	3.315829028	-1.577387833	0.293347	-5.377211	7.56E-08	3.76E-07
235	PDIA4	6851.119109	-1.577673746	0.144068	-10.95086	6.58E-28	5.49E-26
1437	SIGLEC9	104.3851913	-1.579770116	0.206523	-7.64936	2.02E-14	2.76E-13
4037	CMTM2	6.987919903	-1.581229855	0.297599	-5.313288	1.08E-07	5.23E-07
4013	CLDN11	62.49011304	-1.58133817	0.296631	-5.330998	9.77E-08	4.77E-07
3311	BHLHE22	19.88305495	-1.58163926	0.27225	-5.809501	6.27E-09	3.71E-08
4646	ZDHHC8P1	25.03825729	-1.58211423	0.318716	-4.964021	6.90E-07	2.91E-06
411	SH3BP1	245.7053947	-1.582143115	0.158133	-10.00516	1.45E-23	6.90E-22
996	ITGA3	7461.624433	-1.583129179	0.189478	-8.355201	6.53E-17	1.29E-15
5818	FCRL1	4.349866367	-1.584736611	0.363127	-4.364142	1.28E-05	4.30E-05
13247	FAM75A2	0.393100652	-1.585094149	1.013744	-1.563604	0.117911	0.174467
13313	NPPB	0.577533032	-1.585374563	1.03016	-1.53896	0.123814	0.182294
1247	DFNA5	750.572253	-1.585396943	0.199837	-7.933432	2.13E-15	3.35E-14
3332	SLC7A2	2283.323477	-1.585419404	0.273573	-5.79523	6.82E-09	4.01E-08

1716	ZNF579	251.6977455	-1.585637203	0.217731	-7.282542	3.28E-13	3.74E-12
13481	ABCB11	0.347909723	-1.585798614	1.06119	-1.494359	0.135082	0.196405
1275	VAV1	271.4050473	-1.585952627	0.200884	-7.894885	2.91E-15	4.47E-14
708	NPEPL1	1659.522842	-1.587176817	0.176578	-8.988542	2.51E-19	6.94E-18
5268	IGSF10	16.3722129	-1.588697277	0.342155	-4.643204	3.43E-06	1.28E-05
9236	TREML4	1.52655266	-1.588743266	0.543067	-2.925501	0.003439	0.007298
13743	SFTPA1	0.325204926	-1.58906692	1.131786	-1.404035	0.160308	0.22864
13903	SERPINA1[illegible]	0.324825031	-1.589436094	1.179015	-1.348106	0.177624	0.250429
14295	HAND1	0.418756246	-1.590130029	1.292256	-1.230507	0.218507	0.299612
1592	EFNA3	147.8450429	-1.591266589	0.214101	-7.432317	1.07E-13	1.31E-12
4618	MUC1	2354.420557	-1.592088208	0.319605	-4.981431	6.31E-07	2.68E-06
7754	AQP8	0.823044594	-1.592478502	0.452695	-3.517777	0.000435	0.0011
973	MX2	363.925428	-1.592942051	0.189871	-8.389591	4.88E-17	9.83E-16
4154	WNT1	2.607000063	-1.593682333	0.303572	-5.249768	1.52E-07	7.19E-07
2439	MAP4K1	169.9210789	-1.594572689	0.244533	-6.520899	6.99E-11	5.62E-10
7974	HBE1	1.233592706	-1.595263231	0.466442	-3.420069	0.000626	0.001539
5008	TLE6	25.66314276	-1.595295072	0.334514	-4.768992	1.85E-06	7.25E-06
11044	WIF1	1.16513136	-1.595646734	0.701423	-2.27487	0.022914	0.040668
8373	CALHM1	0.658023728	-1.595977569	0.489446	-3.260782	0.001111	0.002601
376	SLC15A3	876.0495661	-1.596867504	0.157548	-10.13574	3.83E-24	2.00E-22
7068	MMP17	44.54732842	-1.596880583	0.42075	-3.795323	0.000147	0.000409
6993	MEOX2	83.30987053	-1.597453327	0.417408	-3.827076	0.00013	0.000363
4776	PTGS1	1849.71468	-1.599128547	0.3266	-4.896284	9.77E-07	4.01E-06
4287	OLFM2	139.8709443	-1.599875941	0.309878	-5.16292	2.43E-07	1.11E-06
2124	BUB1B	104.3010718	-1.600106544	0.234418	-6.825872	8.74E-12	8.06E-11
1611	CENPF	401.67354	-1.60087324	0.216022	-7.410706	1.26E-13	1.53E-12
3183	SIGLEC14	83.40339984	-1.601698055	0.271804	-5.892847	3.80E-09	2.34E-08
209	S100A11	7874.232928	-1.602659224	0.143854	-11.14085	7.94E-29	7.44E-27
7325	HBA1	735.0302524	-1.602831188	0.434333	-3.690325	0.000224	0.000599
1634	CSF3R	349.9839772	-1.603362322	0.217185	-7.382467	1.55E-13	1.86E-12
4431	SULT1A2	35.48305046	-1.60356915	0.315471	-5.083096	3.71E-07	1.64E-06
1792	[illegible]C1002332	39.55222132	-1.604356649	0.222921	-7.19698	6.16E-13	6.73E-12
731	RPS19	16846.77585	-1.604688581	0.179469	-8.941327	3.85E-19	1.03E-17
7916	DSC3	4.608998411	-1.604794709	0.466274	-3.441741	0.000578	0.001431
2257	[illegible]NKRD13[illegible]	116.5946334	-1.604908522	0.239904	-6.689787	2.23E-11	1.94E-10
8702	SPINK2	2.618276168	-1.604943837	0.512987	-3.128622	0.001756	0.003956
7124	MUC13	92.3749073	-1.605240996	0.424993	-3.777098	0.000159	0.000437
818	CCNB1	361.4942225	-1.605897669	0.184285	-8.714184	2.93E-18	7.01E-17
355	RBCK1	3614.67831	-1.606324292	0.156689	-10.2517	1.16E-24	6.42E-23
8089	[illegible]OM121L8[illegible]	1.097550221	-1.606506228	0.476069	-3.374522	0.000739	0.001792
1063	WDR62	50.74153247	-1.606565976	0.195439	-8.220274	2.03E-16	3.74E-15
8377	PRKY	109.8985662	-1.607667693	0.493306	-3.258968	0.001118	0.002616
14975	SP9	0.399574448	-1.607916988	1.584315	-1.014897	0.310155	0.405966
1938	LFNG	400.8222021	-1.607994551	0.228248	-7.044937	1.86E-12	1.88E-11
8453	ABCA13	5.512611294	-1.608305241	0.498368	-3.227143	0.00125	0.002899
9810	MPPED1	0.738509974	-1.608433276	0.592685	-2.713807	0.006651	0.01329
4978	KRTCAP3	150.7134019	-1.609672882	0.336468	-4.78403	1.72E-06	6.77E-06

3652	TBX15	216.4614022	-1.609784208	0.288987	-5.57044	2.54E-08	1.36E-07
5618	GSTM5	52.96674853	-1.610023184	0.360692	-4.46371	8.06E-06	2.81E-05
417	LGALS3BP	13647.66796	-1.610264956	0.161472	-9.972405	2.01E-23	9.46E-22
1371	POU2F2	147.1365632	-1.611344379	0.207544	-7.763851	8.24E-15	1.18E-13
1753	LY96	232.953417	-1.611816119	0.222588	-7.241251	4.45E-13	4.97E-12
10305	INSM2	0.421570088	-1.612061274	0.633777	-2.543579	0.010972	0.02087
1149	SLC37A2	375.0978567	-1.612617881	0.199371	-8.088517	6.04E-16	1.03E-14
2761	PRDM8	46.65665216	-1.612716162	0.258892	-6.229307	4.69E-10	3.33E-09
5861	CXCL3	19.28171068	-1.613229574	0.371631	-4.340939	1.42E-05	4.74E-05
2020	ISG15	898.6272136	-1.615796178	0.232889	-6.938044	3.98E-12	3.86E-11
1333	CD248	1633.140841	-1.61599736	0.206696	-7.818248	5.36E-15	7.88E-14
1211	LYPD3	19.71046914	-1.616196673	0.201991	-8.001335	1.23E-15	1.99E-14
113	LEPRE1	1097.471427	-1.617999115	0.132968	-12.16837	4.58E-34	7.95E-32
426	NFKBIL2	123.392728	-1.618645863	0.162939	-9.934044	2.96E-23	1.36E-21
1723	PTPN7	247.4864321	-1.61899691	0.222633	-7.272041	3.54E-13	4.03E-12
5984	C1orf61	2.48752778	-1.620166933	0.378899	-4.275985	1.90E-05	6.23E-05
3606	PLAC2	9.575499886	-1.62138876	0.289337	-5.603806	2.10E-08	1.14E-07
1967	C9orf16	696.7838455	-1.621932328	0.231326	-7.011462	2.36E-12	2.35E-11
2456	C16orf59	27.94426445	-1.621982711	0.249198	-6.508807	7.57E-11	6.05E-10
8535	NEFM	60.41713769	-1.622046978	0.507897	-3.193654	0.001405	0.003226
2631	ACAP1	206.0089976	-1.623329395	0.255893	-6.343786	2.24E-10	1.67E-09
1183	CYP2S1	61.22622069	-1.623932756	0.201845	-8.045456	8.59E-16	1.42E-14
1261	44079	528.1706419	-1.624100815	0.205238	-7.913271	2.51E-15	3.90E-14
6331	WNT16	2.845439157	-1.625008474	0.395117	-4.112724	3.91E-05	0.000121
1137	SLCO2B1	2537.218923	-1.62542446	0.200472	-8.107994	5.15E-16	8.87E-15
5547	C22orf41	6.668148415	-1.62577738	0.361318	-4.499576	6.81E-06	2.41E-05
283	FHL3	406.4100896	-1.626653473	0.152686	-10.65359	1.68E-26	1.16E-24
6699	UCHL1	764.7858863	-1.626869262	0.412759	-3.941453	8.10E-05	0.000237
10854	C16orf73	4.153737078	-1.627339288	0.695896	-2.338479	0.019362	0.034966
2444	KIF15	40.6757851	-1.627815544	0.249849	-6.515185	7.26E-11	5.82E-10
3961	LAX1	60.83717802	-1.628154746	0.303209	-5.369749	7.88E-08	3.90E-07
360	EMP3	1435.782769	-1.628220395	0.159125	-10.23233	1.42E-24	7.74E-23
6199	PTGDS	387.3393702	-1.628443781	0.389905	-4.176516	2.96E-05	9.36E-05
10618	KIAA1486	0.663523055	-1.632053836	0.672211	-2.427891	0.015187	0.028035
3660	HAR1A	11.50432236	-1.6322052	0.293383	-5.563393	2.65E-08	1.42E-07
8582	RCVRN	0.52628516	-1.632929341	0.513549	-3.179697	0.001474	0.003367
295	RGS10	514.1634064	-1.634469626	0.154428	-10.58401	3.53E-26	2.35E-24
1118	PAQR4	390.3454602	-1.634499361	0.200881	-8.136651	4.06E-16	7.12E-15
1552	HSD3B7	3296.314271	-1.635953011	0.218457	-7.48868	6.96E-14	8.79E-13
6361	ADAM33	13.87896381	-1.638857911	0.400089	-4.096238	4.20E-05	0.000129
291	SERPINH1	6865.11464	-1.639538434	0.154458	-10.61476	2.54E-26	1.71E-24
2075	S100A3	50.27516272	-1.639594638	0.238486	-6.87502	6.20E-12	5.85E-11
3129	TREML1	4.779513575	-1.642511146	0.276415	-5.9422	2.81E-09	1.76E-08
2093	CCDC74A	280.4267247	-1.642963186	0.239744	-6.852997	7.23E-12	6.77E-11
670	RPL28	12953.55849	-1.643963963	0.18066	-9.09978	9.05E-20	2.65E-18
14616	KRT77	0.313399143	-1.644114423	1.458265	-1.127445	0.259554	0.348055
5615	SLC22A1	9.578183978	-1.644232307	0.368214	-4.465426	7.99E-06	2.79E-05

3483	KRT80	348.6585479	-1.645450732	0.289388	-5.68597	1.30E-08	7.32E-08
6294	TGM3	3.697167457	-1.646721332	0.398779	-4.129406	3.64E-05	0.000113
1091	NCKAP1L	825.7244237	-1.646931387	0.201507	-8.173083	3.01E-16	5.40E-15
6433	KCND3	13.5460236	-1.64763402	0.406221	-4.056005	4.99E-05	0.000152
9788	C3orf66	12.41497263	-1.647932467	0.605854	-2.720016	0.006528	0.013072
2301	GGT5	912.9598342	-1.648130355	0.247643	-6.655277	2.83E-11	2.41E-10
2846	SLC2A14	83.12582656	-1.648791937	0.268216	-6.147248	7.88E-10	5.43E-09
10621	NTF4	0.464984056	-1.649399065	0.679755	-2.426461	0.015247	0.028138
12522	MAGEA6	0.568056476	-1.649531609	0.917662	-1.797538	0.07225	0.113095
5874	PPBP	8.442661716	-1.650408601	0.380721	-4.334961	1.46E-05	4.86E-05
1032	CD101	47.78997213	-1.650488938	0.199337	-8.279906	1.23E-16	2.34E-15
730	CYBA	3126.61243	-1.651611589	0.184716	-8.941345	3.84E-19	1.03E-17
3720	VGLL3	160.0024974	-1.651987281	0.299013	-5.524806	3.30E-08	1.74E-07
6815	CCNO	24.45992471	-1.652039561	0.42433	-3.893294	9.89E-05	0.000284
244	LIMK1	1044.86292	-1.652170495	0.151364	-10.91522	9.75E-28	7.83E-26
1223	CDKN3	65.49841763	-1.652276635	0.206797	-7.989837	1.35E-15	2.17E-14
9333	APOH	45.35036327	-1.652989946	0.572995	-2.884824	0.003916	0.008225
11268	OBP2A	0.382174862	-1.653622714	0.754166	-2.192651	0.028333	0.049285
2273	CNIH2	8.456122483	-1.655232903	0.247989	-6.674628	2.48E-11	2.14E-10
2842	HIST2H2AC	5.058705986	-1.655843192	0.269197	-6.151056	7.70E-10	5.31E-09
11201	TAC3	0.38388212	-1.656546645	0.745824	-2.221095	0.026344	0.046101
4355	C2orf89	67.90419225	-1.65779311	0.323229	-5.128854	2.92E-07	1.31E-06
5279	C20orf195	2.940790146	-1.657807115	0.357584	-4.636137	3.55E-06	1.32E-05
1708	IL2RG	783.3549923	-1.658393273	0.22753	-7.288674	3.13E-13	3.59E-12
12495	BTG4	0.361808895	-1.65856548	0.918539	-1.805656	0.070972	0.111326
7718	MGC2889	1.517900429	-1.660416081	0.469905	-3.533518	0.00041	0.001041
643	RAD51	52.7436721	-1.660426989	0.180768	-9.185424	4.10E-20	1.25E-18
1237	ULBP2	44.33311715	-1.66065434	0.208951	-7.947589	1.90E-15	3.01E-14
6090	RUNDC3A	70.87770803	-1.660996496	0.393122	-4.225143	2.39E-05	7.69E-05
5006	TREX2	19.60892519	-1.661371913	0.34833	-4.769536	1.85E-06	7.23E-06
2763	C1orf127	7.713132119	-1.66213342	0.266983	-6.225605	4.80E-10	3.40E-09
5700	KCND2	11.9921035	-1.662206411	0.376007	-4.420683	9.84E-06	3.38E-05
3356	CCL8	74.90014528	-1.6626203	0.28793	-5.774381	7.72E-09	4.51E-08
3204	SDS	335.9624007	-1.662774079	0.282695	-5.88187	4.06E-09	2.48E-08
6196	XCR1	7.023941513	-1.663477629	0.398245	-4.17702	2.95E-05	9.34E-05
2246	PTAFR	148.6068438	-1.663579183	0.248275	-6.700537	2.08E-11	1.81E-10
2580	HSH2D	75.62443182	-1.663591995	0.259848	-6.402171	1.53E-10	1.16E-09
3450	C19orf36	31.4268156	-1.663805445	0.291565	-5.70647	1.15E-08	6.55E-08
2312	STMN3	1893.825754	-1.664488158	0.250503	-6.644594	3.04E-11	2.58E-10
2573	MATN1	5.062120033	-1.664668282	0.259741	-6.408952	1.47E-10	1.12E-09
299	LRFN1	46.76977963	-1.66676926	0.158033	-10.54697	5.25E-26	3.44E-24
2998	LOC65378(	13.88115697	-1.667141922	0.276335	-6.033053	1.61E-09	1.05E-08
585	AIF1	886.0540505	-1.667151984	0.178058	-9.362959	7.75E-21	2.60E-19
3545	SIRPG	81.25658681	-1.667332239	0.295634	-5.639855	1.70E-08	9.41E-08
1789	EXO1	48.84704544	-1.668183803	0.231729	-7.198858	6.07E-13	6.65E-12
2149	CHST1	265.603355	-1.668353784	0.245015	-6.809196	9.81E-12	8.95E-11
668	ARHGEF19	140.156634	-1.668995903	0.183105	-9.11497	7.87E-20	2.31E-18

4454	FCGR3B	97.13912104	-1.669827181	0.329368	-5.069791	3.98E-07	1.75E-06
2466	OXCT2	8.117033067	-1.671248824	0.257018	-6.502449	7.90E-11	6.28E-10
1188	)C1002707	210.8771201	-1.671329611	0.208181	-8.028271	9.89E-16	1.63E-14
10639	ADCY8	83.55354	-1.671336933	0.690957	-2.418871	0.015569	0.028683
3324	SNHG9	31.38260578	-1.671437398	0.288133	-5.800918	6.60E-09	3.89E-08
5965	CYP4F12	32.00642567	-1.671560285	0.390144	-4.284471	1.83E-05	6.02E-05
5500	MSX2	30.32617581	-1.671975574	0.369762	-4.521757	6.13E-06	2.19E-05
1392	FCGR3A	4314.826847	-1.673280367	0.216574	-7.726144	1.11E-14	1.56E-13
2416	KIF14	56.35145366	-1.673943189	0.255895	-6.54153	6.09E-11	4.94E-10
5319	DIO2	143.9925594	-1.674115285	0.362946	-4.612571	3.98E-06	1.47E-05
6063	LRRN4	128.0165604	-1.676094512	0.395414	-4.238829	2.25E-05	7.26E-05
1103	SERPING1	15179.4307	-1.676274297	0.205526	-8.156039	3.46E-16	6.15E-15
1720	CCR10	29.23425549	-1.677205374	0.230531	-7.275409	3.45E-13	3.94E-12
1164	LIMD2	521.196962	-1.677209771	0.207874	-8.068392	7.12E-16	1.20E-14
3973	DNAJC5B	25.92319112	-1.677421275	0.313086	-5.35771	8.43E-08	4.16E-07
763	CSF1R	2474.38009	-1.678410414	0.189653	-8.849914	8.76E-19	2.25E-17
11889	KRT33B	0.605722696	-1.680567611	0.843774	-1.991728	0.046401	0.0765
8018	TMEM151A	9.42331153	-1.681760754	0.493914	-3.404964	0.000662	0.001617
4321	TRIM17	10.41945379	-1.682003078	0.326829	-5.146437	2.65E-07	1.20E-06
8324	GPR31	0.792113259	-1.682814717	0.51269	-3.282322	0.00103	0.002424
6554	C1orf177	2.625042845	-1.683220705	0.420262	-4.00517	6.20E-05	0.000185
1249	CD52	476.885928	-1.684258956	0.212344	-7.931751	2.16E-15	3.39E-14
1748	CCDC88B	386.1669424	-1.685302312	0.232557	-7.246839	4.27E-13	4.78E-12
2333	DLGAP5	85.46540246	-1.686140894	0.254653	-6.621327	3.56E-11	2.99E-10
11791	GUCA1C	0.500165062	-1.686214123	0.832803	-2.024746	0.042893	0.071305
5203	BTN1A1	1.752541886	-1.688288804	0.361028	-4.676341	2.92E-06	1.10E-05
3907	NPFF	14.53325175	-1.688349012	0.312755	-5.39832	6.73E-08	3.37E-07
8747	UGT1A7	12.52300795	-1.689263493	0.543214	-3.109759	0.001872	0.004196
8552	BCORL2	3.027278081	-1.689382776	0.529795	-3.188748	0.001429	0.003275
3931	LEF1	564.6924056	-1.692209952	0.31434	-5.383377	7.31E-08	3.65E-07
1678	CD6	218.1349448	-1.692796301	0.231102	-7.324883	2.39E-13	2.79E-12
589	COPZ2	347.0391071	-1.693363035	0.180956	-9.357872	8.14E-21	2.71E-19
10273	C3orf20	1.686447434	-1.693795088	0.662775	-2.555613	0.0106	0.020225
3349	MMP16	71.38513827	-1.693882592	0.293016	-5.780856	7.43E-09	4.35E-08
8951	CYP2C18	6.735850237	-1.694480279	0.558968	-3.031444	0.002434	0.00533
4806	RHCE	9.061292502	-1.695109853	0.347045	-4.884408	1.04E-06	4.23E-06
12264	MSMB	0.555699497	-1.695758913	0.9027	-1.87854	0.060307	0.096386
2254	KLHDC8A	48.39026374	-1.695827614	0.253364	-6.693252	2.18E-11	1.90E-10
3782	MSC	1276.409678	-1.697073352	0.309441	-5.484322	4.15E-08	2.15E-07
3114	PBX4	30.53927666	-1.697303685	0.285181	-5.951678	2.65E-09	1.67E-08
4065	SERPINF2	4672.201144	-1.697670357	0.320444	-5.297862	1.17E-07	5.65E-07
10864	AKR1B15	1.536345091	-1.698725756	0.727431	-2.33524	0.019531	0.035238
1947	PLEKHG4	331.1436517	-1.699435466	0.241479	-7.037601	1.96E-12	1.97E-11
5248	NANOS3	2.120151707	-1.699524192	0.3653	-4.652413	3.28E-06	1.23E-05
205	PDLIM7	898.6318073	-1.701153003	0.152008	-11.19123	4.50E-29	4.30E-27
3359	DCST2	6.822548528	-1.70149529	0.294797	-5.771749	7.85E-09	4.58E-08
749	KCNMB1	172.5481506	-1.702139851	0.191527	-8.88722	6.27E-19	1.64E-17

2022	LY9	58.55191048	-1.702283115	0.245443	-6.935556	4.05E-12	3.92E-11
1502	PHLDA2	92.80641278	-1.703009537	0.225201	-7.562184	3.96E-14	5.17E-13
8252	OC1001338	1.080742085	-1.703433897	0.514182	-3.312902	0.000923	0.002193
9097	CLCA2	1.816069552	-1.703938511	0.572266	-2.977531	0.002906	0.006261
3893	CYP1B1	2066.692683	-1.705182565	0.315343	-5.407392	6.39E-08	3.22E-07
413	WAS	345.2984306	-1.70524153	0.170734	-9.987693	1.73E-23	8.19E-22
2235	OC1001267	126.3015307	-1.705264009	0.253918	-6.715806	1.87E-11	1.64E-10
2708	CXCR3	108.6719429	-1.705538358	0.271875	-6.273238	3.54E-10	2.56E-09
4675	DPY19L2P1	25.45090754	-1.70622201	0.344863	-4.947533	7.52E-07	3.15E-06
5084	MAPK8IP2	54.233478	-1.706715901	0.360772	-4.730737	2.24E-06	8.62E-06
8219	APOC1P1	2.137863059	-1.710195811	0.514374	-3.324807	0.000885	0.00211
4925	PYY2	3.991249864	-1.7106023	0.35523	-4.815472	1.47E-06	5.84E-06
3380	KRT81	10.19343308	-1.711040041	0.297111	-5.758934	8.46E-09	4.91E-08
415	LAPTM5	7801.615691	-1.712652027	0.171575	-9.981934	1.83E-23	8.64E-22
1847	ASB2	64.81163982	-1.71269873	0.239895	-7.139368	9.38E-13	9.95E-12
3225	FAM46B	59.62996953	-1.712867855	0.291854	-5.868913	4.39E-09	2.67E-08
4262	SLC26A10	32.27210771	-1.713068516	0.331009	-5.175287	2.28E-07	1.05E-06
4074	FFAR3	6.978518707	-1.713072182	0.323578	-5.294152	1.20E-07	5.75E-07
4961	SRPK3	9.401397512	-1.713625132	0.357618	-4.79177	1.65E-06	6.53E-06
622	DEF6	275.3498154	-1.71409544	0.18544	-9.243389	2.39E-20	7.53E-19
344	SLC43A3	1459.779509	-1.715144865	0.16619	-10.32036	5.70E-25	3.25E-23
1945	EIF4EBP1	1219.960171	-1.716646083	0.243859	-7.039499	1.93E-12	1.94E-11
7256	ECEL1	10.5343677	-1.717105704	0.461268	-3.722578	0.000197	0.000533
3348	CILP	28.19693041	-1.717735493	0.297044	-5.782773	7.35E-09	4.30E-08
994	NCAPH	77.2385955	-1.718082584	0.205618	-8.355692	6.51E-17	1.28E-15
5903	GPR109B	35.54677009	-1.718384996	0.39775	-4.320268	1.56E-05	5.17E-05
6271	NKAIN2	2.509690502	-1.719257404	0.414624	-4.146543	3.38E-05	0.000106
936	LOC645166	265.5654776	-1.720077399	0.203115	-8.46848	2.49E-17	5.21E-16
4042	LOC389458	4.057514509	-1.720521359	0.323915	-5.311637	1.09E-07	5.27E-07
2639	PCDHGA12	68.81365195	-1.721135063	0.271626	-6.336407	2.35E-10	1.75E-09
6722	STRA8	2.157704517	-1.721603556	0.437825	-3.932177	8.42E-05	0.000245
4874	EXTL1	4.292087751	-1.722250262	0.355422	-4.845654	1.26E-06	5.08E-06
15275	KRTAP2-1	0.307408267	-1.722362526	1.858929	-0.926535	0.354168	0.454471
3036	VCAN	7846.291607	-1.722987518	0.286796	-6.007712	1.88E-09	1.21E-08
2217	CCL5	1719.056507	-1.723388972	0.255734	-6.738985	1.59E-11	1.41E-10
3923	MS4A1	40.95844105	-1.723859338	0.319815	-5.390182	7.04E-08	3.52E-07
2750	FAM20A	559.4329345	-1.724797054	0.276436	-6.239419	4.39E-10	3.13E-09
10149	HBG2	9.403011952	-1.725002117	0.663779	-2.598762	0.009356	0.018068
1932	APOBEC3H	26.75464252	-1.725900694	0.244648	-7.054633	1.73E-12	1.76E-11
2270	OXTR	68.74096242	-1.726165158	0.258504	-6.677511	2.43E-11	2.10E-10
2464	PTCRA	4.804366271	-1.727177703	0.265535	-6.504518	7.79E-11	6.20E-10
10308	RPS4Y1	2672.770312	-1.727740882	0.679517	-2.5426	0.011003	0.020924
2051	FAM26F	307.4719394	-1.72787493	0.250372	-6.901225	5.16E-12	4.93E-11
5005	LOC389634	8.491841335	-1.729805858	0.362558	-4.771109	1.83E-06	7.18E-06
616	CNTNAP1	359.5421118	-1.730567797	0.186759	-9.266313	1.93E-20	6.13E-19
2853	STYK1	9.220704428	-1.731382243	0.281943	-6.140897	8.21E-10	5.64E-09
12850	RPRML	0.404963981	-1.731568352	1.024087	-1.690842	0.090867	0.138606

958	ISG20	340.1473216	-1.731932888	0.205378	-8.432887	3.37E-17	6.90E-16
2423	CNN1	497.3741278	-1.7319558	0.264901	-6.538118	6.23E-11	5.04E-10
265	EMILIN2	530.3656124	-1.73416111	0.161223	-10.75627	5.54E-27	4.10E-25
8790	PITX2	52.5843099	-1.73627471	0.562182	-3.088458	0.002012	0.004487
5244	LOC150197	4.219017452	-1.736381276	0.373129	-4.653569	3.26E-06	1.22E-05
6523	SIGLEC12	22.08292248	-1.737331901	0.432502	-4.016936	5.90E-05	0.000177
11504	TDRG1	0.569805389	-1.73747692	0.821206	-2.115762	0.034365	0.058553
6794	LTF	518.4623777	-1.738006855	0.445415	-3.901992	9.54E-05	0.000275
3167	HTR2B	70.1243154	-1.738251561	0.294262	-5.907161	3.48E-09	2.15E-08
751	LSP1	1095.293158	-1.740954973	0.195941	-8.885093	6.39E-19	1.67E-17
327	FANCA	163.9912048	-1.742107839	0.167078	-10.42691	1.87E-25	1.12E-23
3609	CD27	314.2433843	-1.742826391	0.311118	-5.601818	2.12E-08	1.15E-07
8207	KRTAP1-5	0.541363422	-1.742984417	0.523553	-3.329149	0.000871	0.002081
1021	SIGLEC10	395.2536509	-1.743660091	0.21009	-8.299571	1.04E-16	2.01E-15
784	PHLDA3	1231.932356	-1.743937382	0.19822	-8.797968	1.39E-18	3.48E-17
234	S1PR2	147.796171	-1.74397308	0.159139	-10.95879	6.03E-28	5.05E-26
14226	SSX1	0.408039913	-1.743984051	1.394306	-1.25079	0.211011	0.290737
2187	ITGB1BP2	6.150763311	-1.744866717	0.25762	-6.773023	1.26E-11	1.13E-10
8142	SLC13A3	617.1718504	-1.744925767	0.519928	-3.356089	0.000791	0.001903
7804	LOC554202	24.35818954	-1.745551766	0.49871	-3.500136	0.000465	0.001168
5473	FLJ40330	60.45443043	-1.746995863	0.385367	-4.533328	5.81E-06	2.08E-05
5271	FCAR	9.558974658	-1.74776432	0.376561	-4.64138	3.46E-06	1.29E-05
1082	CEBPA	446.1045159	-1.748162779	0.21357	-8.185416	2.71E-16	4.92E-15
500	CD68	7811.840182	-1.748193502	0.181859	-9.612909	7.05E-22	2.76E-20
5643	CD70	1263.329094	-1.748570751	0.392792	-4.451643	8.52E-06	2.96E-05
5915	TTC36	4.6700227	-1.748682446	0.405189	-4.315715	1.59E-05	5.27E-05
10435	HBD	9.682573947	-1.74897411	0.699781	-2.499315	0.012443	0.023375
480	LPAR2	232.1246087	-1.749418959	0.180471	-9.693615	3.21E-22	1.31E-20
7292	UGT1A3	52.91800144	-1.749452298	0.472618	-3.701619	0.000214	0.000576
2169	NEK2	70.57885335	-1.75033054	0.257611	-6.794466	1.09E-11	9.82E-11
5211	FCER2	3.921177219	-1.750395489	0.374528	-4.673601	2.96E-06	1.11E-05
269	DOK3	296.6654147	-1.750428233	0.162983	-10.73993	6.61E-27	4.80E-25
8198	SLC7A10	2.103435069	-1.751357986	0.525387	-3.333464	0.000858	0.002051
1940	RGS2	1017.194446	-1.751766029	0.248753	-7.042202	1.89E-12	1.91E-11
9143	DSG3	1.31164691	-1.752648119	0.591932	-2.960895	0.003067	0.006576
6838	ALOXE3	2.025199199	-1.75321742	0.451335	-3.884517	0.000103	0.000294
5064	CNR2	2.130323185	-1.753653774	0.370031	-4.739213	2.15E-06	8.30E-06
3735	NTM	285.324574	-1.754781896	0.318189	-5.51491	3.49E-08	1.83E-07
1598	SNHG12	389.5365791	-1.755303235	0.23641	-7.424836	1.13E-13	1.39E-12
12483	PAX7	0.413141258	-1.75592194	0.971172	-1.808044	0.0706	0.110857
11781	OR1J4	0.389020641	-1.756174913	0.865925	-2.028091	0.042551	0.070796
804	LST1	304.3759987	-1.756341443	0.20075	-8.748909	2.15E-18	5.25E-17
404	ADAM19	365.9630285	-1.756540538	0.175369	-10.01628	1.29E-23	6.24E-22
2971	PRR7	69.69163572	-1.757950814	0.290292	-6.055803	1.40E-09	9.22E-09
6575	RGS20	23.45584096	-1.759571976	0.440672	-3.992928	6.53E-05	0.000195
3598	DKK2	21.69375585	-1.760375774	0.314033	-5.605707	2.07E-08	1.13E-07
8944	TTTY14	2.246431849	-1.760618362	0.580259	-3.034193	0.002412	0.005286

7409	MOGAT3	64.66821631	-1.761117895	0.482093	-3.653066	0.000259	0.000686
12009	SPINLW1	0.491677445	-1.761522876	0.899303	-1.958764	0.05014	0.081839
9345	AHSG	1.037573866	-1.762857353	0.612176	-2.879656	0.003981	0.00835
7683	MYEOV	487.3322414	-1.764353167	0.497547	-3.546101	0.000391	0.000997
9723	GABRA3	0.734434499	-1.767672823	0.645029	-2.740455	0.006135	0.012369
1629	LTA	17.94050462	-1.768694465	0.239323	-7.390396	1.46E-13	1.76E-12
51	HN1	1106.752706	-1.769566824	0.135567	-13.05307	6.10E-39	2.30E-36
6847	GPR25	0.82886174	-1.769587506	0.456084	-3.879959	0.000104	0.000299
2403	SEMA3B	750.6897217	-1.76981214	0.270021	-6.55436	5.59E-11	4.56E-10
545	RAC2	907.5542034	-1.77050449	0.186883	-9.473862	2.70E-21	9.70E-20
3154	TMEM91	1355.219759	-1.771564513	0.29943	-5.91646	3.29E-09	2.04E-08
1058	B3GNT7	97.7112505	-1.772176105	0.215301	-8.231155	1.85E-16	3.44E-15
4819	FXYD7	3.206723908	-1.772274783	0.363176	-4.87993	1.06E-06	4.32E-06
15189	MMP27	0.342270791	-1.772296869	1.863316	-0.951152	0.341527	0.440732
806	TLR9	26.40098654	-1.773073615	0.202823	-8.741978	2.29E-18	5.57E-17
9691	IL17C	0.666634102	-1.77407093	0.645433	-2.748651	0.005984	0.012103
2905	IL10	21.86384119	-1.774183193	0.290444	-6.108527	1.01E-09	6.78E-09
1621	LTBP4	1151.247381	-1.774666094	0.239785	-7.401066	1.35E-13	1.63E-12
718	SLC2A6	147.3214293	-1.775129718	0.197888	-8.970395	2.95E-19	8.07E-18
673	KIFC1	126.2206637	-1.775725992	0.195315	-9.091614	9.76E-20	2.84E-18
926	SIGLEC7	98.89272077	-1.775973545	0.209365	-8.482662	2.20E-17	4.66E-16
2406	PSTPIP1	134.4950295	-1.776095067	0.271082	-6.551885	5.68E-11	4.63E-10
6005	CDRT1	1.850541912	-1.776151694	0.416492	-4.264556	2.00E-05	6.54E-05
1279	PIM2	794.1885053	-1.776358664	0.225057	-7.892923	2.95E-15	4.52E-14
2638	CD209	195.8275436	-1.776884249	0.280403	-6.336886	2.34E-10	1.74E-09
831	EBI3	125.0262478	-1.777125929	0.204844	-8.675523	4.12E-18	9.70E-17
2012	FUT7	12.55861367	-1.777370709	0.255924	-6.944912	3.79E-12	3.69E-11
2634	NAPSB	371.2179657	-1.778097849	0.280394	-6.341422	2.28E-10	1.69E-09
325	FXYD5	1929.021235	-1.778415759	0.170443	-10.43408	1.73E-25	1.05E-23
8877	CALML6	1.164624057	-1.778869533	0.581027	-3.061593	0.002202	0.004861
1630	ATP10A	200.9728817	-1.778884745	0.240845	-7.38601	1.51E-13	1.82E-12
1518	FCGR2C	143.022179	-1.780344353	0.23626	-7.535528	4.86E-14	6.28E-13
925	CD44	5125.096267	-1.780919697	0.209931	-8.483352	2.19E-17	4.64E-16
425	TNFAIP8L2	157.2781046	-1.782012488	0.179189	-9.944891	2.65E-23	1.22E-21
2973	C6orf81	9.739744995	-1.782093381	0.294341	-6.054512	1.41E-09	9.29E-09
5747	HTR2A	4.086554096	-1.782695241	0.405229	-4.39923	1.09E-05	3.71E-05
9966	KRT6B	1.793711648	-1.782763178	0.669054	-2.664603	0.007708	0.01516
7281	FOXL2	1.121085044	-1.782880299	0.48088	-3.707541	0.000209	0.000563
1897	PIF1	26.04749329	-1.783606336	0.251619	-7.088522	1.36E-12	1.40E-11
1968	MESP1	42.31719516	-1.785809501	0.254909	-7.005684	2.46E-12	2.45E-11
2296	PRR11	38.69889869	-1.786670254	0.268303	-6.659153	2.75E-11	2.35E-10
259	QSOX1	5616.539716	-1.786743359	0.165607	-10.78903	3.88E-27	2.94E-25
775	LOXL3	150.3590647	-1.787175995	0.202519	-8.82473	1.10E-18	2.78E-17
6973	GATA1	1.742206961	-1.787492453	0.465884	-3.836779	0.000125	0.00035
4482	C9orf170	2.577283003	-1.78870577	0.354261	-5.049116	4.44E-07	1.94E-06
948	RUNX2	263.8038138	-1.788706401	0.211709	-8.448899	2.94E-17	6.08E-16
1253	ROBO3	116.0157563	-1.789397077	0.225651	-7.929937	2.19E-15	3.43E-14

7036	C21orf121	1.481217867	-1.789583892	0.470237	-3.805704	0.000141	0.000394
726	SULF2	4805.609598	-1.791393668	0.200187	-8.948589	3.60E-19	9.72E-18
2848	PPEF1	18.80329796	-1.793006143	0.291822	-6.144167	8.04E-10	5.53E-09
8529	TPTE2	1.125236812	-1.793493721	0.561287	-3.195327	0.001397	0.00321
872	NCF1	170.9934655	-1.793892235	0.208693	-8.59586	8.26E-18	1.86E-16
3594	MEST	509.1361368	-1.79445848	0.320013	-5.607448	2.05E-08	1.12E-07
301	ITGA5	5755.25714	-1.796549854	0.170481	-10.53813	5.76E-26	3.75E-24
970	CEACAM21	34.32753779	-1.797250041	0.21408	-8.39524	4.65E-17	9.40E-16
2089	MEF2B	37.20606703	-1.7973332	0.262166	-6.855701	7.10E-12	6.66E-11
1327	TNFRSF8	25.539326	-1.797764407	0.229627	-7.829071	4.91E-15	7.26E-14
771	CLEC2B	550.9245034	-1.798147376	0.203665	-8.828932	1.06E-18	2.69E-17
10831	EPB42	1.958167209	-1.798939614	0.765404	-2.350315	0.018758	0.033946
6999	CCKBR	1.376132347	-1.800712009	0.470857	-3.82433	0.000131	0.000367
7685	HBG1	12.95787242	-1.801744469	0.508238	-3.54508	0.000392	0.001001
176	BMP1	1291.62018	-1.802587039	0.157398	-11.45239	2.29E-30	2.55E-28
309	LRP1	8952.001554	-1.804087929	0.171962	-10.4912	9.48E-26	6.01E-24
1740	MEFV	43.3901506	-1.804539332	0.248826	-7.252208	4.10E-13	4.62E-12
1428	PPFIA3	170.0081942	-1.805183913	0.235414	-7.668129	1.75E-14	2.40E-13
241	PLEKHO1	1132.894798	-1.805787331	0.165315	-10.92332	8.92E-28	7.26E-26
743	PRR24	235.1335622	-1.807086416	0.20288	-8.907149	5.24E-19	1.38E-17
591	KCNK6	127.5620314	-1.807309785	0.193309	-9.349341	8.82E-21	2.93E-19
1722	TK1	641.7554393	-1.807424254	0.248527	-7.272551	3.53E-13	4.02E-12
516	HTRA1	7260.983756	-1.807732383	0.189416	-9.543711	1.38E-21	5.24E-20
6918	CAMP	2.097271607	-1.807842454	0.468757	-3.856672	0.000115	0.000326
4815	MEG3	43.36818385	-1.808209255	0.370412	-4.881614	1.05E-06	4.28E-06
5347	KCNG2	1.269958381	-1.808281572	0.393545	-4.594851	4.33E-06	1.59E-05
97	CCNF	122.3801927	-1.808848396	0.146044	-12.38564	3.13E-35	6.32E-33
1760	HAUS7	674.2579794	-1.810012053	0.250312	-7.231024	4.79E-13	5.34E-12
7342	CYP26A1	7.963163585	-1.810515528	0.491469	-3.683888	0.00023	0.000613
6892	EPHA8	0.715013657	-1.810611606	0.468145	-3.867629	0.00011	0.000313
4817	CORO6	37.79918625	-1.810705235	0.371016	-4.880399	1.06E-06	4.31E-06
650	PLOD2	10904.6675	-1.811875056	0.19758	-9.170321	4.72E-20	1.42E-18
8280	MMP8	1.721144722	-1.812503574	0.549079	-3.30099	0.000963	0.002281
5304	CHI3L1	632.8031732	-1.812521674	0.392427	-4.618747	3.86E-06	1.43E-05
3260	UPK3B	9.983088568	-1.81375384	0.310302	-5.845132	5.06E-09	3.04E-08
8751	CEACAM6	1.468149456	-1.814181835	0.584039	-3.106269	0.001895	0.004244
12065	ZSCAN10	0.39959218	-1.814336466	0.935268	-1.93991	0.052391	0.085115
748	MKI67	611.6789269	-1.815700426	0.204055	-8.89809	5.68E-19	1.49E-17
399	PLOD1	7878.628834	-1.817085495	0.181115	-10.03278	1.09E-23	5.37E-22
11754	OLIG3	0.486717552	-1.817382954	0.892467	-2.036359	0.041714	0.069557
1808	MMP2	2396.380021	-1.819253313	0.253347	-7.180863	6.93E-13	7.51E-12
2931	KCNH4	5.182838243	-1.820569519	0.299088	-6.087077	1.15E-09	7.69E-09
12293	MAGEA3	0.82266637	-1.820853185	0.974607	-1.868294	0.061721	0.098413
769	RCN3	484.3815626	-1.820883701	0.206002	-8.839162	9.64E-19	2.46E-17
1802	XCL2	26.07130693	-1.822065936	0.253374	-7.191201	6.42E-13	6.99E-12
1064	COL5A2	2967.423798	-1.823182903	0.221814	-8.219413	2.05E-16	3.77E-15
2880	C1orf200	2.171764081	-1.82356714	0.297772	-6.124034	9.12E-10	6.21E-09

8122	C7orf52	4.988069025	-1.823861183	0.542688	-3.360794	0.000777	0.001876
1289	CBR3	129.4946232	-1.824721918	0.231664	-7.876592	3.36E-15	5.12E-14
1750	NUF2	65.45354383	-1.824808836	0.251968	-7.242235	4.41E-13	4.94E-12
3519	LPPR4	64.52432001	-1.825082247	0.322566	-5.658013	1.53E-08	8.53E-08
3932	RTN4RL1	168.5127101	-1.825504244	0.339143	-5.382693	7.34E-08	3.66E-07
2233	SLAMF7	491.8925525	-1.826422585	0.271875	-6.717885	1.84E-11	1.62E-10
250	B3GNTL1	90.04909498	-1.826550932	0.168375	-10.84808	2.04E-27	1.60E-25
4879	CCDC42B	5.792321039	-1.828363069	0.377554	-4.842655	1.28E-06	5.15E-06
701	CSTA	92.06090411	-1.829465482	0.202801	-9.021004	1.86E-19	5.21E-18
5743	ICAM5	8.703447363	-1.829731046	0.415773	-4.400791	1.08E-05	3.68E-05
4134	CNFN	27.74283722	-1.829957749	0.347864	-5.260552	1.44E-07	6.81E-07
1969	MATK	130.4166032	-1.830684728	0.261436	-7.002433	2.52E-12	2.50E-11
12628	LOC285501	0.35503479	-1.830884903	1.037484	-1.764736	0.077608	0.120462
3070	DARC	412.6839135	-1.831414517	0.306155	-5.981983	2.20E-09	1.41E-08
7105	NCAN	1.244497592	-1.834391586	0.485059	-3.781787	0.000156	0.000429
3012	TSHZ2	88.82369164	-1.83499824	0.304594	-6.024398	1.70E-09	1.10E-08
2431	SIGLEC11	19.85971795	-1.836056252	0.281171	-6.530023	6.58E-11	5.30E-10
1179	TAGLN	6701.294121	-1.836095067	0.22802	-8.052357	8.12E-16	1.35E-14
1540	JPH2	362.6394658	-1.836288907	0.24463	-7.506395	6.08E-14	7.74E-13
1991	S100A9	481.154111	-1.836651326	0.26336	-6.973932	3.08E-12	3.03E-11
501	KCND1	79.1642775	-1.838305702	0.191262	-9.611433	7.15E-22	2.80E-20
6826	JSRP1	27.18301786	-1.838371547	0.472661	-3.889406	0.0001	0.000289
1801	PTPRCAP	338.6336361	-1.838535038	0.255666	-7.19115	6.42E-13	6.99E-12
2679	GFRA2	80.77912616	-1.840228966	0.29214	-6.299124	2.99E-10	2.19E-09
8837	C14orf184	1.364396293	-1.840563927	0.59847	-3.075447	0.002102	0.004662
7500	UBL4B	1.354026988	-1.840720052	0.508806	-3.617723	0.000297	0.000777
729	RPL39L	111.1578406	-1.843377821	0.206114	-8.943501	3.77E-19	1.01E-17
289	FCER1G	1041.347156	-1.843391777	0.173364	-10.63307	2.09E-26	1.42E-24
1670	ZNF296	35.95880439	-1.846020739	0.251726	-7.333455	2.24E-13	2.63E-12
352	PLP2	2117.16099	-1.846428967	0.179731	-10.27331	9.30E-25	5.18E-23
9339	CRYBA4	0.575863642	-1.847984843	0.640897	-2.883433	0.003934	0.008256
4048	PLEKHG4B	50.33425653	-1.848830775	0.348322	-5.307826	1.11E-07	5.37E-07
2080	IL9R	5.640138337	-1.850467602	0.269358	-6.869911	6.42E-12	6.05E-11
2178	CH25H	49.27580991	-1.850496282	0.272946	-6.779714	1.20E-11	1.08E-10
610	DUSP23	1469.30325	-1.85070553	0.199156	-9.292747	1.50E-20	4.83E-19
5711	CNTN6	6.186907875	-1.850940261	0.419261	-4.414769	1.01E-05	3.47E-05
258	C1QTNF6	593.6287545	-1.85428062	0.171863	-10.78929	3.87E-27	2.94E-25
3271	PVRL4	23.45813287	-1.856228838	0.318171	-5.834062	5.41E-09	3.24E-08
3014	LOC1002160	7.226186784	-1.857117939	0.308324	-6.023263	1.71E-09	1.11E-08
3541	CKM	1.874176784	-1.858961486	0.32956	-5.640744	1.69E-08	9.37E-08
56	STAC3	86.73718971	-1.859076453	0.143084	-12.99288	1.34E-38	4.70E-36
5080	GTSF1	7.07452862	-1.861384614	0.39334	-4.732254	2.22E-06	8.57E-06
342	FERMT3	652.5637665	-1.862907057	0.180361	-10.32877	5.22E-25	2.99E-23
3616	ADRA2A	112.8437375	-1.863133449	0.332931	-5.596163	2.19E-08	1.19E-07
3542	ANGPTL6	6.845527448	-1.863154036	0.330312	-5.640593	1.69E-08	9.38E-08
122	RUNX1	1630.082779	-1.863232768	0.154903	-12.02842	2.52E-33	4.05E-31
8396	C6orf118	0.575287469	-1.863758887	0.57354	-3.249571	0.001156	0.002698

508	CD300C	66.24644164	-1.864504151	0.194671	-9.577699	9.92E-22	3.83E-20
423	OSCAR	116.9703196	-1.865180099	0.187397	-9.953105	2.44E-23	1.13E-21
179	FKBP11	726.7645883	-1.865263932	0.163463	-11.41094	3.69E-30	4.03E-28
372	ANKRD58	32.6001992	-1.865867615	0.183471	-10.16984	2.70E-24	1.42E-22
199	CMTM3	1223.094981	-1.865883856	0.165904	-11.24675	2.40E-29	2.37E-27
7222	HIST1H1B	0.490238856	-1.866088611	0.499325	-3.737222	0.000186	0.000505
2370	SYTL1	94.88339215	-1.866355881	0.283255	-6.58895	4.43E-11	3.66E-10
4876	IRGM	1.347582994	-1.866708458	0.385386	-4.843737	1.27E-06	5.12E-06
8273	SCGB3A2	0.572227216	-1.868582305	0.565612	-3.303646	0.000954	0.002261
9552	UTF1	0.657821809	-1.86997748	0.667597	-2.801058	0.005094	0.010452
1682	FPR1	340.0579895	-1.870760131	0.255632	-7.318178	2.51E-13	2.93E-12
10205	XKR7	0.439216535	-1.871824839	0.72558	-2.579764	0.009887	0.01899
3369	UCN	8.841781462	-1.872042238	0.324617	-5.766927	8.07E-09	4.70E-08
7853	IL5RA	2.943418759	-1.872700852	0.539683	-3.47	0.00052	0.001299
5164	C8G	19.39034669	-1.873067756	0.399131	-4.692865	2.69E-06	1.02E-05
2493	C2	2526.536513	-1.874273149	0.289632	-6.471226	9.72E-11	7.64E-10
1937	TNC	2700.531267	-1.874759748	0.2661	-7.045313	1.85E-12	1.87E-11
380	PLB1	93.4525357	-1.876027439	0.185325	-10.12292	4.37E-24	2.26E-22
3397	FGF18	7.382541272	-1.877769522	0.326613	-5.749224	8.97E-09	5.17E-08
13158	MRGPRX3	0.342889938	-1.878852623	1.180199	-1.59198	0.111389	0.16592
4361	CST6	5.087950658	-1.879269251	0.366583	-5.126456	2.95E-07	1.33E-06
1182	CCR7	80.0138869	-1.879282851	0.233471	-8.049306	8.33E-16	1.38E-14
5230	NKD2	182.6067552	-1.879513285	0.403015	-4.663637	3.11E-06	1.16E-05
2340	CCDC80	1676.175084	-1.880083491	0.284123	-6.617136	3.66E-11	3.07E-10
5372	APOC4	1.524606838	-1.881285161	0.410543	-4.582431	4.60E-06	1.68E-05
1904	ITGA11	387.3551271	-1.881873831	0.265668	-7.083562	1.40E-12	1.45E-11
15285	RAX	0.394441398	-1.881895677	2.034988	-0.92477	0.355086	0.455351
3795	PHEX	12.96488525	-1.88211037	0.343706	-5.475924	4.35E-08	2.25E-07
1843	CDCA7	111.5269823	-1.88211117	0.263352	-7.146737	8.89E-13	9.45E-12
716	LILRB3	152.221851	-1.883044183	0.20985	-8.97328	2.88E-19	7.88E-18
192	SPI1	723.9862878	-1.884403632	0.166685	-11.3052	1.24E-29	1.26E-27
135	TMEM149	239.0724294	-1.884899496	0.159832	-11.79299	4.24E-32	6.16E-30
2410	IRF4	92.71683946	-1.885018303	0.287761	-6.550638	5.73E-11	4.66E-10
3704	PODXL2	235.3932064	-1.887227562	0.341028	-5.533932	3.13E-08	1.66E-07
13447	LGALS7B	0.50925928	-1.889658375	1.257887	-1.502249	0.133033	0.193937
6320	KCNS2	2.459710909	-1.88998161	0.459072	-4.116965	3.84E-05	0.000119
5068	ODZ3	257.7192339	-1.891023195	0.399073	-4.738544	2.15E-06	8.33E-06
8917	OR7C1	0.785547877	-1.891077771	0.621101	-3.04472	0.002329	0.005119
5921	LILRP2	1.609869214	-1.892089062	0.438795	-4.31201	1.62E-05	5.36E-05
3333	SRRM3	76.99618448	-1.892587014	0.326599	-5.794838	6.84E-09	4.02E-08
564	ROM1	117.3667488	-1.894473222	0.201028	-9.423936	4.34E-21	1.51E-19
8637	TRIML2	0.596835601	-1.896187897	0.600423	-3.158085	0.001588	0.003604
1479	NCF1C	59.35571308	-1.897052802	0.249646	-7.598986	2.98E-14	3.96E-13
8111	PON1	1.311995796	-1.897384553	0.56385	-3.365053	0.000765	0.001849
3442	ITGA2B	18.3195167	-1.89771084	0.332202	-5.712515	1.11E-08	6.34E-08
4118	CXCR2P1	76.86448596	-1.897791601	0.360007	-5.271545	1.35E-07	6.44E-07
1794	HTRA3	287.8184033	-1.898475351	0.263801	-7.196625	6.17E-13	6.74E-12

10553	C10orf91	0.420530302	-1.898723333	0.774246	-2.452351	0.014193	0.026361
593	HCST	264.424095	-1.898855569	0.203258	-9.342095	9.44E-21	3.12E-19
3284	CES8	838.3411402	-1.901189491	0.326382	-5.825044	5.71E-09	3.41E-08
5436	L1CAM	190.5447842	-1.901500869	0.417953	-4.549561	5.38E-06	1.94E-05
196	NCF4	251.1150164	-1.902556754	0.168781	-11.27234	1.80E-29	1.80E-27
9522	POU3F2	0.98445278	-1.902741449	0.677335	-2.809157	0.004967	0.010225
22	IMPDH1	710.2966822	-1.902816342	0.135422	-14.051	7.60E-45	6.77E-42
11068	TRY6	0.557815767	-1.905690367	0.840429	-2.267522	0.023358	0.041367
16142	FAM197Y2	0.374288082	-1.906548639	2.852712	-0.668329	0.503924	0.611908
2354	FBLN7	203.352206	-1.909607517	0.289188	-6.603333	4.02E-11	3.35E-10
4461	OC1001323	6.298723256	-1.909917368	0.377304	-5.062014	4.15E-07	1.82E-06
13295	C8A	0.455894788	-1.911179075	1.236701	-1.545385	0.122253	0.180239
6899	CACNG4	12.45455294	-1.911760844	0.494591	-3.865338	0.000111	0.000315
133	GALNT2	3817.23231	-1.913268871	0.161864	-11.82023	3.07E-32	4.52E-30
4	ARPC1B	3643.968242	-1.91518261	0.120445	-15.90093	6.24E-57	2.45E-53
7539	KPNA7	1.31252143	-1.916956356	0.53259	-3.599306	0.000319	0.000829
3521	TLL2	16.88339343	-1.917235959	0.338885	-5.657491	1.54E-08	8.55E-08
3859	MT1L	199.8053291	-1.917701743	0.353358	-5.427072	5.73E-08	2.91E-07
8145	DYDC2	4.187467043	-1.918295259	0.571738	-3.3552	0.000793	0.001909
997	RRM2	431.9142912	-1.920717503	0.229925	-8.353672	6.62E-17	1.30E-15
13176	LOC38903	0.538164317	-1.920863537	1.210471	-1.586872	0.112542	0.16742
1000	CARD9	78.90881577	-1.922157257	0.230262	-8.347686	6.96E-17	1.36E-15
1263	FN1	51328.97885	-1.924131919	0.243247	-7.910211	2.57E-15	3.99E-14
1466	CCDC19	10.8779074	-1.925312842	0.252744	-7.617635	2.58E-14	3.45E-13
572	CDT1	139.1789728	-1.925572153	0.204764	-9.403843	5.26E-21	1.80E-19
542	TRPM2	343.7965708	-1.926680276	0.203104	-9.486191	2.40E-21	8.67E-20
4220	IL11	3.459288046	-1.928059385	0.370893	-5.19843	2.01E-07	9.34E-07
1641	PRRX1	299.665106	-1.928501536	0.261592	-7.372181	1.68E-13	2.00E-12
596	ALOX5	810.0223572	-1.931482275	0.207048	-9.328678	1.07E-20	3.52E-19
10771	FAM9A	0.405885688	-1.931636879	0.813333	-2.374963	0.017551	0.031939
1302	LOC28305(	32.85082578	-1.932237594	0.245755	-7.862463	3.77E-15	5.67E-14
5307	SYNGR4	2.497540669	-1.933373255	0.418893	-4.615432	3.92E-06	1.45E-05
157	GNA15	168.0121695	-1.935497165	0.166808	-11.60316	3.97E-31	4.96E-29
1160	FZD2	100.9610509	-1.936172496	0.23971	-8.077162	6.63E-16	1.12E-14
381	GPR68	78.64562943	-1.939927762	0.191687	-10.12031	4.49E-24	2.31E-22
10848	SERPINA9	1.73862518	-1.940192272	0.829109	-2.340092	0.019279	0.034836
797	TPM2	3075.726889	-1.941301637	0.22166	-8.758006	1.99E-18	4.89E-17
1181	SKA1	48.5060845	-1.941443278	0.241153	-8.050682	8.23E-16	1.37E-14
1957	ACTG2	600.1289801	-1.943260611	0.276607	-7.025336	2.14E-12	2.14E-11
9130	FGFBP1	1.150157511	-1.943683972	0.655364	-2.965808	0.003019	0.006481
13930	TCHHL1	0.383655073	-1.943758076	1.449175	-1.341286	0.179828	0.253037
12197	SPANXN3	0.441533081	-1.944275858	1.024877	-1.897081	0.057817	0.092914
2856	COL13A1	42.55900499	-1.945204421	0.316821	-6.139751	8.27E-10	5.67E-09
2244	RAB40A	13.61277226	-1.946074824	0.290388	-6.701643	2.06E-11	1.80E-10
3705	BTBD11	222.0539942	-1.947128567	0.351864	-5.533755	3.13E-08	1.66E-07
9399	DBX2	0.408733308	-1.947440635	0.681084	-2.859327	0.004245	0.008854
9516	TP53TG3B	1.306372193	-1.950910892	0.693355	-2.813727	0.004897	0.010087

2459	ITGBL1	273.0323488	-1.951502849	0.299899	-6.507197	7.66E-11	6.10E-10
11309	PITX3	0.467981223	-1.953887144	0.897899	-2.176065	0.02955	0.051217
6140	HEMGN	3.597943121	-1.954226753	0.464724	-4.205135	2.61E-05	8.33E-05
11477	MC4R	0.550210032	-1.954351791	0.918551	-2.127646	0.033366	0.056985
253	PRSS36	38.85414188	-1.954746767	0.180638	-10.82134	2.73E-27	2.11E-25
5308	TNFRSF13I	5.916083445	-1.956131415	0.423822	-4.61546	3.92E-06	1.45E-05
4103	RAB42	346.3265808	-1.957357737	0.37073	-5.279745	1.29E-07	6.18E-07
8521	GUCA1A	0.776265664	-1.957428705	0.612307	-3.196811	0.00139	0.003196
1102	CDC45	55.06889575	-1.958942114	0.240176	-8.156288	3.45E-16	6.14E-15
3299	MESP2	6.777007203	-1.960214363	0.337011	-5.816474	6.01E-09	3.57E-08
462	UHRF1	116.1826723	-1.96063719	0.200905	-9.759025	1.69E-22	7.16E-21
229	CDCA5	144.993772	-1.96112391	0.178544	-10.984	4.56E-28	3.91E-26
3291	SPATA12	3.952618796	-1.961978547	0.337009	-5.821748	5.82E-09	3.47E-08
9912	SFRP5	1.251626812	-1.962259119	0.730963	-2.684484	0.007264	0.014365
519	RAD54L	48.00125818	-1.962840016	0.205728	-9.540963	1.42E-21	5.34E-20
9858	KRT39	0.61392611	-1.964565345	0.727354	-2.700976	0.006914	0.013747
6238	NXF3	1.639267353	-1.964741877	0.472358	-4.159437	3.19E-05	0.0001
983	TGM2	13778.76233	-1.965842731	0.234828	-8.37141	5.69E-17	1.14E-15
658	SERINC2	4902.823204	-1.965903091	0.214787	-9.15282	5.55E-20	1.65E-18
1393	CFB	4757.241038	-1.966340268	0.254575	-7.724021	1.13E-14	1.59E-13
303	KCTD17	256.1178927	-1.967455402	0.186843	-10.52997	6.29E-26	4.07E-24
528	COL6A1	8315.547951	-1.968901861	0.206693	-9.525753	1.64E-21	6.08E-20
857	ADAMTSL4	559.9426934	-1.969099205	0.228282	-8.62572	6.37E-18	1.46E-16
12637	MAGEC1	0.467702125	-1.969448118	1.117264	-1.762742	0.077944	0.120897
3135	BEAN	18.94237708	-1.969737488	0.331831	-5.935964	2.92E-09	1.83E-08
1364	LTBP1	1997.829585	-1.970918259	0.253576	-7.772481	7.70E-15	1.11E-13
845	TMEM158	47.25441944	-1.971249372	0.228073	-8.643076	5.47E-18	1.27E-16
7624	CHGA	2.184740885	-1.973497692	0.55291	-3.569296	0.000358	0.00092
53	CENPW	59.09872579	-1.976454888	0.151434	-13.05156	6.23E-39	2.30E-36
13873	MAGEB10	0.367204242	-1.976541823	1.452296	-1.360978	0.173521	0.245165
2965	GPR97	33.28470616	-1.976865268	0.326334	-6.057794	1.38E-09	9.12E-09
5930	SLC7A4	2.953742432	-1.977039152	0.45909	-4.306429	1.66E-05	5.48E-05
2616	DRP2	3.807059945	-1.977282939	0.310811	-6.361679	2.00E-10	1.50E-09
4661	ADAMDEC	164.1046634	-1.980193265	0.399651	-4.954803	7.24E-07	3.04E-06
8162	GPR128	1.32492526	-1.981294291	0.591877	-3.347478	0.000816	0.001958
1376	PCDHGC5	18.69739982	-1.981852324	0.255427	-7.758964	8.56E-15	1.22E-13
9663	C20orf201	0.914911491	-1.982029906	0.718119	-2.76003	0.00578	0.011724
5010	LGI4	2223.950329	-1.982345561	0.415693	-4.768777	1.85E-06	7.25E-06
1129	FHL2	1404.472529	-1.982700072	0.244287	-8.116275	4.81E-16	8.34E-15
1113	GPR133	40.69131458	-1.983671621	0.243669	-8.140834	3.93E-16	6.91E-15
3871	TNFRSF13C	3.682892339	-1.983738342	0.365908	-5.421414	5.91E-08	2.99E-07
3404	HTR1D	6.581074902	-1.984227059	0.345481	-5.743372	9.28E-09	5.34E-08
8936	KRT6A	6.362308909	-1.98453843	0.652953	-3.039328	0.002371	0.005201
11052	IL22RA2	0.502694234	-1.985165411	0.873225	-2.273372	0.023004	0.040803
9720	LOC731789	0.72073468	-1.98720948	0.724716	-2.742053	0.006106	0.012312
3583	WNT10B	9.936398386	-1.987917059	0.353966	-5.616124	1.95E-08	1.07E-07
5381	ADRB3	0.974212549	-1.988154755	0.434407	-4.576714	4.72E-06	1.72E-05

1374	SRCRB4D	57.93172539	-1.989249631	0.256276	-7.762137	8.35E-15	1.19E-13
9900	DLL3	1.753445233	-1.990611912	0.740618	-2.687772	0.007193	0.014241
6506	TDRD5	3.836163728	-1.99142826	0.494793	-4.02477	5.70E-05	0.000172
15112	MMP23A	0.653720511	-1.99176468	2.04809	-0.972499	0.330803	0.429067
3398	FAM132A	38.79706191	-1.993060167	0.346683	-5.748934	8.98E-09	5.18E-08
3962	FAM69C	3.98968744	-1.993111919	0.371196	-5.369431	7.90E-08	3.91E-07
3820	HOXD13	15.92572833	-1.997677209	0.366015	-5.457914	4.82E-08	2.47E-07
7809	MAGEA5	0.904520746	-1.998195906	0.57126	-3.497874	0.000469	0.001177
3224	SYBU	872.3185211	-1.999457223	0.340599	-5.870422	4.35E-09	2.64E-08
12917	FRG2B	0.565018622	-1.999488058	1.198515	-1.668305	0.095255	0.144546
10828	SERPINA1[illegible]	1.072633902	-1.999684756	0.850202	-2.352012	0.018672	0.033801
1761	ZNF474	8.162932528	-2.000814362	0.276701	-7.230954	4.80E-13	5.34E-12
10110	CES7	2.233115027	-2.001158326	0.765679	-2.613575	0.00896	0.017371
1710	DLX4	23.64495443	-2.001647926	0.274728	-7.285924	3.19E-13	3.66E-12
1711	E2F7	57.56910415	-2.002198653	0.274811	-7.285723	3.20E-13	3.67E-12
2609	SNCG	535.0641895	-2.004706875	0.314719	-6.369838	1.89E-10	1.42E-09
1246	SH2D2A	128.8017942	-2.005017448	0.252688	-7.934767	2.11E-15	3.32E-14
607	SEMA4B	3753.637181	-2.00748791	0.215926	-9.297116	1.44E-20	4.66E-19
7017	APOB	1066.513303	-2.007873489	0.526158	-3.816106	0.000136	0.000379
2769	PCDH7	117.2624439	-2.010038955	0.323143	-6.220286	4.96E-10	3.51E-09
2228	ZNF385D	33.19388368	-2.011464013	0.299255	-6.721568	1.80E-11	1.58E-10
4365	CD1A	4.513443288	-2.013952718	0.393098	-5.123288	3.00E-07	1.35E-06
1892	F13A1	1961.035321	-2.015592253	0.284214	-7.091815	1.32E-12	1.37E-11
5602	TAS1R1	2.712495462	-2.01600217	0.450975	-4.470316	7.81E-06	2.73E-05
1834	TSKU	1755.431325	-2.016186819	0.281391	-7.165071	7.77E-13	8.32E-12
8539	FRMD5	7.64575769	-2.017532558	0.631858	-3.193014	0.001408	0.003232
655	SP140	105.8103046	-2.018798998	0.220459	-9.157234	5.32E-20	1.59E-18
1046	TMIGD2	9.296651566	-2.021231707	0.245191	-8.243489	1.67E-16	3.13E-15
3561	VWA3B	1.830950601	-2.021455016	0.359199	-5.62767	1.83E-08	1.01E-07
1557	ZNF683	46.19618081	-2.023624476	0.270363	-7.48483	7.16E-14	9.02E-13
7834	SLC9A11	2.783752224	-2.024098466	0.580935	-3.484209	0.000494	0.001235
5647	CLEC4G	2.242699226	-2.025222009	0.455206	-4.449025	8.63E-06	2.99E-05
6513	MCHR1	396.3447361	-2.02616003	0.503729	-4.022319	5.76E-05	0.000173
590	LRP8	72.14000889	-2.026781693	0.216624	-9.356238	8.26E-21	2.75E-19
20	LGALS9	1311.559419	-2.029052331	0.143577	-14.13212	2.41E-45	2.36E-42
6187	PRIMA1	1200.295075	-2.030178809	0.485532	-4.181351	2.90E-05	9.18E-05
1824	CDKN2A	165.9589835	-2.030200102	0.283235	-7.167901	7.62E-13	8.18E-12
4980	C20orf200	1.365351859	-2.030270934	0.424441	-4.783403	1.72E-06	6.78E-06
114	TYROBP	1784.296246	-2.031996895	0.167465	-12.13389	6.98E-34	1.20E-31
4503	AP3B2	43.77933891	-2.032223592	0.403457	-5.037033	4.73E-07	2.06E-06
2460	SLC12A8	256.009844	-2.032934773	0.312438	-6.506685	7.68E-11	6.12E-10
2594	IGFBP6	741.4560748	-2.032995232	0.318224	-6.388562	1.67E-10	1.27E-09
2527	FOSL1	240.1830318	-2.033323664	0.315677	-6.441145	1.19E-10	9.20E-10
5759	LEFTY2	6.622318914	-2.038981249	0.464173	-4.392717	1.12E-05	3.81E-05
11858	SPRR2A	0.612652783	-2.038985454	1.017935	-2.003061	0.045171	0.074666
968	DGKG	122.9430531	-2.039445397	0.242729	-8.402167	4.38E-17	8.88E-16
1004	SLC11A1	325.1450257	-2.039527508	0.244562	-8.339521	7.46E-17	1.46E-15

10952	RHAG	1.102156667	-2.04022192	0.885694	-2.30353	0.021249	0.03803
6929	SSTR3	0.773331566	-2.040715902	0.529831	-3.851638	0.000117	0.000332
12399	HAND2	0.703459624	-2.041834618	1.11177	-1.836561	0.066275	0.104771
3323	IL27	2.456574472	-2.041849128	0.351976	-5.80111	6.59E-09	3.89E-08
10368	PYDC1	1.57120065	-2.042188198	0.810661	-2.519163	0.011763	0.022239
3499	PGF	4273.689417	-2.042343568	0.359799	-5.676342	1.38E-08	7.71E-08
144	LAIR1	926.3263229	-2.042614706	0.17512	-11.66406	1.95E-31	2.65E-29
867	IL21R	125.0348343	-2.042701766	0.237341	-8.606595	7.53E-18	1.70E-16
319	CDCA8	121.3620546	-2.044697247	0.195762	-10.4448	1.55E-25	9.51E-24
1799	HPX	10.39544449	-2.050985136	0.285157	-7.192479	6.36E-13	6.93E-12
1926	MGAT3	654.7843926	-2.05197842	0.290678	-7.05929	1.67E-12	1.70E-11
856	DPYSL3	2780.242094	-2.052108386	0.23785	-8.627759	6.26E-18	1.43E-16
8566	SNORD15E	1.03636637	-2.053120394	0.644635	-3.184935	0.001448	0.003313
3376	PTK6	63.66955668	-2.053338547	0.35632	-5.762618	8.28E-09	4.81E-08
14039	CT45A2	0.440686433	-2.054062469	1.571619	-1.306972	0.191222	0.266981
6874	TSPAN8	78.73277237	-2.054706747	0.5304	-3.87388	0.000107	0.000305
3667	NKX3-2	2.054985343	-2.055951294	0.369895	-5.558206	2.73E-08	1.46E-07
31	SH3BGRL3	4753.511924	-2.056712932	0.150784	-13.64017	2.31E-42	1.46E-39
173	OLFML2B	1674.922561	-2.056836514	0.179302	-11.47136	1.84E-30	2.08E-28
2765	PNPLA1	23.30893479	-2.057009484	0.330483	-6.224253	4.84E-10	3.43E-09
201	GAL3ST4	430.4144154	-2.057288159	0.183464	-11.21359	3.50E-29	3.41E-27
1108	TNFSF13B	156.7320064	-2.057713974	0.252416	-8.152071	3.58E-16	6.33E-15
1666	VNN2	236.5537617	-2.059054804	0.280524	-7.340021	2.14E-13	2.51E-12
81	TMSB10	29126.75151	-2.05972749	0.163888	-12.56788	3.17E-36	7.67E-34
103	TMEM44	503.6637714	-2.061422258	0.168533	-12.23157	2.11E-34	4.01E-32
1446	NUDT11	14.0061834	-2.063753889	0.270168	-7.638766	2.19E-14	2.97E-13
3653	TCL1A	6.566343268	-2.064010809	0.37067	-5.568327	2.57E-08	1.38E-07
28	NUDT1	220.5542171	-2.064881989	0.149005	-13.85784	1.14E-43	7.98E-41
2121	PSD	32.37756356	-2.06511898	0.302401	-6.829068	8.55E-12	7.90E-11
3430	PGAM2	114.6406229	-2.065363348	0.360908	-5.72269	1.05E-08	5.99E-08
3039	PCSK1	30.61183527	-2.066828089	0.344165	-6.005339	1.91E-09	1.23E-08
3124	ATG9B	58.86254674	-2.068088224	0.347808	-5.946059	2.75E-09	1.72E-08
2239	NMB	843.0128294	-2.071638698	0.308823	-6.70817	1.97E-11	1.73E-10
13475	REXO1L1	0.447570487	-2.071962496	1.385323	-1.495653	0.134744	0.196001
1530	CELSR3	68.36980464	-2.074919606	0.276077	-7.515717	5.66E-14	7.25E-13
557	LILRB4	492.683216	-2.076076261	0.219852	-9.443046	3.62E-21	1.27E-19
8502	SHD	0.688377417	-2.076824302	0.647816	-3.205884	0.001346	0.003104
2994	PDCD1	112.5298961	-2.076949169	0.343751	-6.042023	1.52E-09	9.96E-09
3546	FAM178B	2.091889685	-2.077795838	0.368432	-5.63956	1.70E-08	9.42E-08
416	CCNB2	123.0354925	-2.07804129	0.208265	-9.977854	1.91E-23	8.98E-22
2036	PRG4	11.23449616	-2.079472098	0.300394	-6.922476	4.44E-12	4.27E-11
8908	LY6D	0.998067188	-2.085130304	0.683959	-3.048621	0.002299	0.005059
12703	C20orf123	0.422241442	-2.085194715	1.203104	-1.733179	0.083064	0.128169
2078	PPAP2C	175.5998119	-2.086090805	0.3035	-6.873445	6.27E-12	5.91E-11
10505	CRNA0023(	5.849485989	-2.086193124	0.844577	-2.470105	0.013507	0.025203
6647	SLC34A2	1416.536327	-2.086551716	0.52645	-3.963439	7.39E-05	0.000218
2613	SPIB	16.47056406	-2.087084154	0.327959	-6.363863	1.97E-10	1.48E-09

14647	MAGEA1	0.454859047	-2.087959756	1.867665	-1.117952	0.263588	0.352716
1829	ZBP1	45.95151049	-2.088740215	0.291503	-7.165418	7.75E-13	8.31E-12
1492	SPC24	10.32175607	-2.09349778	0.276033	-7.584219	3.34E-14	4.39E-13
307	TPX2	451.4910376	-2.093926565	0.199307	-10.50605	8.10E-26	5.17E-24
10155	SYT4	0.881302556	-2.094669642	0.806459	-2.597368	0.009394	0.018132
2641	BMPER	29.60051961	-2.095182109	0.330826	-6.333193	2.40E-10	1.78E-09
498	HAPLN3	249.7675729	-2.098293809	0.217866	-9.631117	5.91E-22	2.33E-20
2004	SMOC2	1057.634886	-2.099717891	0.301987	-6.952998	3.58E-12	3.50E-11
2752	MOXD1	447.5012035	-2.099928612	0.336631	-6.23808	4.43E-10	3.16E-09
4286	PDGFRA	574.3970563	-2.101900198	0.407086	-5.163287	2.43E-07	1.11E-06
4631	CACNA1G	2.466696531	-2.103515953	0.422943	-4.973524	6.57E-07	2.78E-06
396	C9orf110	78.9210367	-2.103548674	0.209261	-10.05226	8.98E-24	4.44E-22
1353	MDFI	79.62348673	-2.10372972	0.269891	-7.794749	6.45E-15	9.35E-14
437	GTSE1	97.203229	-2.104363247	0.213209	-9.869974	5.62E-23	2.52E-21
825	FCGR2B	351.451872	-2.105109832	0.241959	-8.700274	3.31E-18	7.87E-17
916	HK3	140.7664186	-2.105963467	0.247929	-8.494221	1.99E-17	4.26E-16
652	CEP55	164.7507012	-2.106097205	0.229759	-9.166552	4.88E-20	1.47E-18
598	GPR153	205.1198845	-2.106596472	0.225869	-9.326634	1.09E-20	3.58E-19
3123	DLK2	35.36865996	-2.107794865	0.354429	-5.947023	2.73E-09	1.71E-08
887	LOC60672[illegible]	49.18504268	-2.107935779	0.246442	-8.553492	1.19E-17	2.64E-16
7151	FAM83A	1.144183997	-2.109964109	0.560522	-3.764287	0.000167	0.000458
2341	CR1	154.7842315	-2.110098764	0.318954	-6.615678	3.70E-11	3.10E-10
2427	SIX2	15.43821065	-2.110667169	0.32296	-6.53539	6.34E-11	5.12E-10
706	MYO1G	424.7884442	-2.110940862	0.234582	-8.998752	2.28E-19	6.34E-18
10557	THEG	0.543563393	-2.112009752	0.862106	-2.449825	0.014293	0.026537
145	CD14	2879.298571	-2.112101641	0.181106	-11.66223	1.99E-31	2.69E-29
3918	SIRPD	2.389995505	-2.112282829	0.391793	-5.391321	6.99E-08	3.50E-07
1927	LIF	1119.587697	-2.112509501	0.299289	-7.058425	1.68E-12	1.71E-11
86	NUMBL	287.523387	-2.115645168	0.169142	-12.50809	6.74E-36	1.54E-33
9120	ZIC5	8.800717552	-2.116520708	0.71278	-2.96939	0.002984	0.006413
11568	PDCL2	0.433967844	-2.11796118	1.011994	-2.09286	0.036362	0.061612
8939	GPR112	1.815702752	-2.119199216	0.697671	-3.037532	0.002385	0.00523
2484	OTOF	6.949189558	-2.119548346	0.326837	-6.485037	8.87E-11	7.00E-10
4169	GRIN2A	328.7224979	-2.119848348	0.404827	-5.236433	1.64E-07	7.70E-07
569	PRSS53	89.65392966	-2.120159951	0.225396	-9.406376	5.14E-21	1.77E-19
770	ARL4C	2938.579274	-2.121145569	0.240147	-8.832689	1.02E-18	2.60E-17
3192	GPRC5A	264.6653582	-2.123260921	0.360608	-5.88801	3.91E-09	2.40E-08
2172	TBC1D3C	45.23044679	-2.123691801	0.312798	-6.789329	1.13E-11	1.02E-10
225	OLFML3	1012.925911	-2.124294952	0.193173	-10.99683	3.96E-28	3.45E-26
2282	C4orf48	22.84680344	-2.124652249	0.318558	-6.669591	2.57E-11	2.20E-10
6923	NPW	1.060687005	-2.125882313	0.551455	-3.85504	0.000116	0.000328
5750	PTPRZ1	4.472073434	-2.126956468	0.483709	-4.397185	1.10E-05	3.74E-05
6827	LEP	5.991517086	-2.130192191	0.547793	-3.888684	0.000101	0.000289
395	FOLR2	586.1328465	-2.132285409	0.211953	-10.0602	8.28E-24	4.11E-22
3374	C19orf59	14.59481359	-2.133503217	0.370139	-5.764052	8.21E-09	4.77E-08
4762	GBX2	1.840672013	-2.137219955	0.435844	-4.903636	9.41E-07	3.87E-06
759	CLEC11A	297.6626505	-2.137579964	0.241157	-8.863857	7.73E-19	2.00E-17

1798	MYLK2	5.617662114	-2.137789377	0.297164	-7.193972	6.29E-13	6.86E-12
8626	AADAC	0.712780558	-2.139774053	0.676505	-3.162983	0.001562	0.003548
4260	TSKS	2.454970217	-2.141398198	0.413595	-5.177525	2.25E-07	1.03E-06
4962	TPRXL	1.369049571	-2.143310768	0.447289	-4.79178	1.65E-06	6.53E-06
6207	GPR115	25.4998657	-2.144116559	0.513735	-4.173588	3.00E-05	9.47E-05
932	ASCL2	23.43711667	-2.146971181	0.253254	-8.477537	2.30E-17	4.84E-16
223	RCC1	452.9939328	-2.150139025	0.195259	-11.01175	3.35E-28	2.95E-26
1982	TBX18	37.78652439	-2.150698168	0.307811	-6.987067	2.81E-12	2.78E-11
896	C5orf62	2150.007216	-2.150869296	0.252076	-8.532631	1.43E-17	3.13E-16
6218	IFNE	4.855231264	-2.152698886	0.516266	-4.169748	3.05E-05	9.61E-05
3372	BPI	5.065411338	-2.154951444	0.373712	-5.766347	8.10E-09	4.71E-08
3943	OMD	40.02824424	-2.155302845	0.400765	-5.377978	7.53E-08	3.74E-07
782	LAT	314.5611051	-2.156357773	0.244884	-8.805637	1.30E-18	3.26E-17
5871	VWA3A	5.147555838	-2.157355892	0.497481	-4.336557	1.45E-05	4.83E-05
4435	LOC729467	1.867862501	-2.158043531	0.424679	-5.081593	3.74E-07	1.65E-06
3126	ALOX15B	102.2517502	-2.158697193	0.363077	-5.945561	2.76E-09	1.73E-08
4822	SLC8A2	4.322255262	-2.16118472	0.442939	-4.879196	1.07E-06	4.33E-06
33	MMP19	450.9055907	-2.161481204	0.160092	-13.50146	1.53E-41	9.11E-39
3746	KCNH3	16.3142777	-2.162857297	0.392592	-5.509177	3.61E-08	1.89E-07
275	TREM2	606.165395	-2.163624486	0.202025	-10.70968	9.17E-27	6.53E-25
1008	SPHK1	189.9149317	-2.164639778	0.259808	-8.331679	7.97E-17	1.55E-15
3456	GPR158	8.616085136	-2.169380534	0.380356	-5.70355	1.17E-08	6.65E-08
8418	C4BPA	15.81386415	-2.169763661	0.66899	-3.243345	0.001181	0.002751
2486	WBSCR26	26.42847509	-2.170425869	0.334702	-6.48466	8.89E-11	7.01E-10
1040	SFRP4	394.2287859	-2.17066894	0.262901	-8.256604	1.50E-16	2.82E-15
2148	FAIM2	22.85138004	-2.171193098	0.318816	-6.810172	9.75E-12	8.90E-11
9835	SPRR3	1.048267793	-2.171551266	0.801979	-2.707742	0.006774	0.013501
9322	CYP2C19	0.763076738	-2.174157473	0.75176	-2.892088	0.003827	0.008047
10231	FCRL4	0.596864138	-2.174178499	0.845536	-2.571361	0.01013	0.019407
1473	FAM90A1	24.32992179	-2.174262906	0.285871	-7.605746	2.83E-14	3.77E-13
1674	TM7SF4	11.15137282	-2.178822224	0.297158	-7.332196	2.26E-13	2.65E-12
2060	LAG3	221.3914447	-2.179575521	0.316421	-6.888204	5.65E-12	5.38E-11
101	JAK3	728.2834473	-2.180073768	0.177219	-12.30161	8.88E-35	1.72E-32
95	CEBPB	1447.897386	-2.180589894	0.175881	-12.39811	2.68E-35	5.52E-33
5077	EPHA10	32.62843822	-2.184868773	0.461577	-4.733483	2.21E-06	8.52E-06
4890	GDF10	8.560487414	-2.188437554	0.452793	-4.833196	1.34E-06	5.39E-06
300	MMP11	672.2418718	-2.193607149	0.208112	-10.5405	5.62E-26	3.67E-24
459	LOC728875	65.83858415	-2.194794506	0.224785	-9.763973	1.61E-22	6.86E-21
4422	MYBPC2	10.77687582	-2.196220206	0.431553	-5.089113	3.60E-07	1.59E-06
13522	MAGEA4	0.485149478	-2.198599859	1.484349	-1.481188	0.138556	0.200854
2691	CARD14	113.1607804	-2.199982801	0.349776	-6.289693	3.18E-10	2.32E-09
110	C17orf60	32.14873243	-2.200906748	0.180445	-12.1971	3.22E-34	5.74E-32
1939	CD38	95.767914	-2.201729616	0.312602	-7.043225	1.88E-12	1.90E-11
762	CENPA	39.84851206	-2.201792815	0.248615	-8.856244	8.28E-19	2.13E-17
219	KIF4A	141.8643316	-2.202388272	0.199796	-11.02321	2.95E-28	2.64E-26
7942	CYP2W1	2.181693979	-2.202652641	0.641794	-3.432024	0.000599	0.001479
3295	C6orf141	19.962014	-2.205850287	0.379053	-5.819368	5.91E-09	3.51E-08

1486	RGS4	182.7120414	-2.207091108	0.29068	-7.592856	3.13E-14	4.13E-13
4958	WDR69	4.06692248	-2.207614886	0.460587	-4.793048	1.64E-06	6.49E-06
2331	C8orf31	23.90616283	-2.20969118	0.333571	-6.624349	3.49E-11	2.93E-10
7172	LHB	1.020047628	-2.211085824	0.588766	-3.755461	0.000173	0.000473
252	P2RY6	143.7657034	-2.211584418	0.204282	-10.82612	2.59E-27	2.01E-25
2369	RLTPR	65.88560873	-2.211969189	0.335705	-6.58903	4.43E-11	3.66E-10
4447	SULT2B1	22.70922273	-2.214075197	0.436335	-5.074259	3.89E-07	1.71E-06
1636	ADAMTS1	82.77159902	-2.214455669	0.300002	-7.381464	1.57E-13	1.88E-12
340	CRNA001	421.2934132	-2.214609652	0.213944	-10.35134	4.13E-25	2.38E-23
2692	MEGF10	6.327211116	-2.216942449	0.352574	-6.287881	3.22E-10	2.34E-09
9964	GOLGA8E	0.648254584	-2.220370641	0.833143	-2.665054	0.007698	0.015143
1360	COL8A2	242.7813253	-2.225138397	0.286061	-7.778557	7.34E-15	1.06E-13
9477	DMBX1	3.003317978	-2.225673519	0.787391	-2.826645	0.004704	0.009729
7572	WFDC13	0.552853294	-2.227112299	0.620494	-3.589254	0.000332	0.000858
2081	PLA2G5	15.41051572	-2.227584347	0.324313	-6.868631	6.48E-12	6.10E-11
1391	SPEG	69.59290202	-2.228328484	0.288406	-7.726368	1.11E-14	1.56E-13
262	PLAU	1339.115223	-2.228490775	0.206793	-10.77641	4.45E-27	3.33E-25
766	HOXD11	85.33781489	-2.228770095	0.252016	-8.843748	9.26E-19	2.37E-17
5616	CHRDL1	142.3616743	-2.228817679	0.499179	-4.46497	8.01E-06	2.79E-05
1928	H19	3814.403797	-2.229643837	0.315909	-7.057864	1.69E-12	1.72E-11
2770	BTBD16	56.84481953	-2.230088896	0.358523	-6.220208	4.96E-10	3.51E-09
2131	ZBED2	28.29665457	-2.233817428	0.327381	-6.823296	8.90E-12	8.18E-11
1054	C3	42520.39258	-2.233827863	0.271175	-8.23758	1.76E-16	3.27E-15
3500	REEP2	49.29715723	-2.237088288	0.394147	-5.675776	1.38E-08	7.73E-08
4595	DPEP3	2.208275788	-2.241607864	0.44919	-4.990336	6.03E-07	2.57E-06
2062	TMEM45A	1437.695059	-2.246274697	0.326173	-6.88675	5.71E-12	5.43E-11
3938	IL13RA2	76.28917216	-2.246281498	0.417516	-5.380104	7.44E-08	3.71E-07
3277	ZDHHC19	2.373904248	-2.247427785	0.385405	-5.831337	5.50E-09	3.29E-08
6117	MMP10	5.403472776	-2.247556796	0.533521	-4.212684	2.52E-05	8.09E-05
1533	ANKRD35	20.41697602	-2.248602842	0.299223	-7.514808	5.70E-14	7.29E-13
9811	CACNG6	0.702987343	-2.249271009	0.828865	-2.713674	0.006654	0.013294
523	ITGB4	1479.892788	-2.253913403	0.236383	-9.534999	1.50E-21	5.62E-20
2185	MT1M	57.10993524	-2.256008613	0.333063	-6.773523	1.26E-11	1.13E-10
5030	ZNF114	109.645402	-2.25662623	0.474069	-4.760119	1.93E-06	7.54E-06
6191	LOC72915	10.6523382	-2.256644412	0.539915	-4.179629	2.92E-05	9.24E-05
152	FOXM1	371.3879447	-2.258573014	0.194426	-11.61664	3.39E-31	4.37E-29
4955	CLIC6	517.7949627	-2.259107444	0.471253	-4.793836	1.64E-06	6.47E-06
960	BASP1	653.1093704	-2.260742641	0.268272	-8.427065	3.54E-17	7.24E-16
5831	NPAS4	1.695869903	-2.261852665	0.519269	-4.355844	1.33E-05	4.46E-05
4250	CXCL13	215.929984	-2.261945151	0.436502	-5.181976	2.20E-07	1.01E-06
837	IL1RN	93.64417407	-2.262435174	0.261323	-8.657623	4.82E-18	1.13E-16
1757	BCL2L15	31.05690731	-2.262790255	0.312713	-7.235986	4.62E-13	5.16E-12
3217	LY6H	65.78343973	-2.265188218	0.385476	-5.876344	4.19E-09	2.56E-08
217	C15orf42	48.43358427	-2.266716337	0.205301	-11.04096	2.42E-28	2.19E-26
3103	LRRC4C	35.22180022	-2.267956798	0.380375	-5.962426	2.49E-09	1.57E-08
9338	TAC1	1.326651232	-2.268871616	0.786839	-2.883526	0.003933	0.008255
737	TRIB3	2105.02475	-2.268930323	0.254331	-8.921187	4.61E-19	1.23E-17

563	S100A4	1874.690226	-2.269094452	0.240596	-9.431137	4.06E-21	1.41E-19
3608	CRABP2	111.85482	-2.272497695	0.405551	-5.603486	2.10E-08	1.14E-07
8636	LOC642587	1.259374701	-2.273920866	0.720036	-3.158066	0.001588	0.003604
835	LTB	203.9538344	-2.275346739	0.262521	-8.667308	4.42E-18	1.04E-16
345	C2orf27A	40.97589701	-2.275387844	0.220613	-10.31394	6.10E-25	3.46E-23
213	FCGR1C	98.06797796	-2.275478736	0.205436	-11.07634	1.63E-28	1.50E-26
5926	GOLGA8DP	0.933018683	-2.27562691	0.528198	-4.308285	1.65E-05	5.44E-05
238	LGALS9C	19.92211594	-2.276492585	0.208164	-10.93606	7.75E-28	6.38E-26
5301	ACTBL2	4.470984518	-2.277905389	0.492926	-4.621191	3.82E-06	1.41E-05
139	FCGR1B	265.6535998	-2.278027851	0.193998	-11.74256	7.71E-32	1.09E-29
4683	LGALS12	146.8020528	-2.278586373	0.461307	-4.939416	7.84E-07	3.28E-06
803	SYN1	62.85176511	-2.281702428	0.260784	-8.7494	2.14E-18	5.24E-17
515	BATF	82.79148033	-2.282001981	0.239113	-9.543611	1.38E-21	5.24E-20
10324	SERPINB3	0.742078172	-2.282126096	0.898825	-2.53901	0.011117	0.021106
954	PPP1R14A	144.6895208	-2.283290848	0.270518	-8.440452	3.16E-17	6.49E-16
167	CD72	170.5449922	-2.284605613	0.197772	-11.55171	7.24E-31	8.49E-29
2350	KRT17	49.9839047	-2.286147701	0.345917	-6.608942	3.87E-11	3.23E-10
346	VMO1	76.51624453	-2.287157818	0.221884	-10.30792	6.49E-25	3.68E-23
2111	ABCA17P	51.86287405	-2.289022604	0.33486	-6.835761	8.16E-12	7.57E-11
4149	SP5	37.02736806	-2.28915814	0.435852	-5.252149	1.50E-07	7.10E-07
5163	LOC339674	1.437001511	-2.28951669	0.487856	-4.693014	2.69E-06	1.02E-05
5067	SLC6A7	1.156992761	-2.290982847	0.483466	-4.738665	2.15E-06	8.32E-06
15279	TGM7	0.457536956	-2.291418732	2.474214	-0.92612	0.354384	0.454629
3722	SHC3	5.236940868	-2.294692444	0.415392	-5.524159	3.31E-08	1.74E-07
140	C1QC	5012.356969	-2.29478333	0.195859	-11.71649	1.05E-31	1.47E-29
646	INHBA	153.5768266	-2.295598456	0.250281	-9.17209	4.64E-20	1.41E-18
254	CERCAM	1446.496571	-2.297249702	0.21233	-10.81925	2.79E-27	2.15E-25
2113	SERPINA1	51702.96246	-2.297999573	0.336275	-6.833697	8.28E-12	7.68E-11
2714	TWIST2	5.132212694	-2.298286068	0.366744	-6.266731	3.69E-10	2.66E-09
1189	SLCO5A1	6.774153855	-2.299065467	0.286378	-8.028083	9.90E-16	1.63E-14
4859	ALX1	5.370762184	-2.301385484	0.473663	-4.858697	1.18E-06	4.77E-06
4156	CP	16700.28664	-2.302765249	0.43886	-5.247148	1.54E-07	7.29E-07
4307	C13orf16	3.367032573	-2.303865405	0.446991	-5.154167	2.55E-07	1.16E-06
3755	HFE2	3.657990127	-2.30395737	0.418633	-5.503528	3.72E-08	1.94E-07
2726	ADRA1D	5.46654097	-2.304988116	0.368278	-6.258823	3.88E-10	2.79E-09
503	CACNA2D4	164.5878623	-2.305672493	0.240192	-9.599307	8.05E-22	3.14E-20
4043	CDHR1	575.3721958	-2.306286954	0.434272	-5.310695	1.09E-07	5.29E-07
1880	ZNF365	72.49226911	-2.309896657	0.324961	-7.108232	1.18E-12	1.23E-11
6121	LRIT2	2.589984019	-2.31324299	0.549311	-4.211172	2.54E-05	8.14E-05
334	FKBP10	5697.926873	-2.313246318	0.222734	-10.38571	2.88E-25	1.69E-23
512	FAM128A	1012.005667	-2.315718046	0.242248	-9.559287	1.19E-21	4.54E-20
406	MEIS3	145.3288872	-2.317206296	0.231339	-10.01651	1.29E-23	6.24E-22
876	FOXP3	47.24368085	-2.317393696	0.270131	-8.578769	9.59E-18	2.15E-16
461	NBL1	3070.120923	-2.317482921	0.237375	-9.762962	1.62E-22	6.90E-21
14240	VSTM2A	0.465898416	-2.317643678	1.862642	-1.244278	0.213397	0.293736
4319	DKKL1	1.465844498	-2.318588801	0.450514	-5.146535	2.65E-07	1.20E-06
3518	TMEM92	234.1863018	-2.319601999	0.409941	-5.658378	1.53E-08	8.51E-08

685	CD163L1	164.382037	-2.320836812	0.256527	-9.04713	1.47E-19	4.20E-18
633	EPR1	107.7321964	-2.32172356	0.252277	-9.203063	3.48E-20	1.08E-18
1048	GSDMA	21.0939734	-2.326013731	0.282217	-8.241929	1.69E-16	3.17E-15
351	IQGAP3	240.6571788	-2.327526301	0.226528	-10.27478	9.16E-25	5.11E-23
6924	SPATA22	4.849166609	-2.32839036	0.604009	-3.854896	0.000116	0.000328
558	EMR1	92.44068716	-2.332111555	0.246984	-9.442376	3.64E-21	1.28E-19
6747	C20orf56	1.103167234	-2.335148827	0.595587	-3.920751	8.83E-05	0.000256
1762	P4HA3	353.2556531	-2.336229786	0.323119	-7.230241	4.82E-13	5.36E-12
449	C7orf61	6.709175771	-2.336438796	0.238147	-9.810908	1.01E-22	4.41E-21
2564	GNASAS	3.260372301	-2.336778396	0.364352	-6.413527	1.42E-10	1.09E-09
1775	UPK2	2.997957641	-2.337089509	0.324045	-7.212244	5.50E-13	6.08E-12
717	CD7	200.7931824	-2.337134915	0.26052	-8.971054	2.94E-19	8.03E-18
1637	APOC2	336.7766976	-2.339118577	0.316946	-7.380191	1.58E-13	1.89E-12
499	EPHB2	182.3320365	-2.339192558	0.2431	-9.622364	6.43E-22	2.53E-20
1917	GNB3	15.75949825	-2.339740902	0.330832	-7.072302	1.52E-12	1.56E-11
4360	PRSS21	2.976087977	-2.340462419	0.456542	-5.126499	2.95E-07	1.33E-06
1881	GPR45	2.771536901	-2.344985283	0.329981	-7.106422	1.19E-12	1.24E-11
11018	FOXB1	0.504220659	-2.346280165	1.027028	-2.284533	0.02234	0.039743
7326	LOC28376	1.691180291	-2.346692063	0.636084	-3.689278	0.000225	0.000602
6326	PPP1R1A	1416.709223	-2.348143999	0.570627	-4.115024	3.87E-05	0.00012
2670	GCK	7.286789779	-2.348555959	0.372095	-6.311716	2.76E-10	2.03E-09
653	ANLN	396.9373167	-2.348907235	0.256283	-9.165298	4.94E-20	1.48E-18
1328	TRPM8	9.886980049	-2.351385804	0.300383	-7.827966	4.96E-15	7.32E-14
2157	DMP1	3.383554341	-2.352293323	0.34567	-6.80502	1.01E-11	9.18E-11
5262	CDK5R2	7.030487889	-2.352362069	0.506416	-4.645115	3.40E-06	1.27E-05
4840	TNFRSF17	24.5150112	-2.352586645	0.483068	-4.870089	1.12E-06	4.52E-06
94	ADA	348.7396412	-2.353152384	0.189649	-12.40793	2.37E-35	4.94E-33
3328	PANX2	57.71397634	-2.355039686	0.406251	-5.797007	6.75E-09	3.98E-08
2118	KLHL35	8.344926423	-2.357243214	0.34509	-6.830816	8.44E-12	7.81E-11
10121	HS3ST4	0.670436206	-2.357497162	0.903752	-2.608566	0.009092	0.017609
4800	FAM19A3	3.203902854	-2.358130477	0.482517	-4.88715	1.02E-06	4.18E-06
2260	RUFY4	18.12172626	-2.358765162	0.352648	-6.688724	2.25E-11	1.95E-10
2465	FOXH1	6.90780787	-2.360569021	0.362925	-6.504294	7.81E-11	6.21E-10
26	LGALS1	7099.418345	-2.360988163	0.1693	-13.94558	3.35E-44	2.52E-41
661	RNASET2	6512.788707	-2.362308243	0.258489	-9.138928	6.31E-20	1.87E-18
1386	LOC65443	128.9252983	-2.365239453	0.305869	-7.732857	1.05E-14	1.49E-13
4135	CATSPER	6.363025727	-2.365431119	0.449669	-5.260385	1.44E-07	6.81E-07
4624	MUC16	9.72191211	-2.365651667	0.475184	-4.97839	6.41E-07	2.72E-06
228	SIGLEC1	599.5638928	-2.365927226	0.21535	-10.98641	4.44E-28	3.82E-26
4396	PEX5L	12.5351086	-2.367224071	0.463539	-5.106854	3.28E-07	1.46E-06
8414	FAM5C	1.03062646	-2.369860578	0.73044	-3.244428	0.001177	0.002742
3424	SCARA5	10.67339359	-2.369997295	0.413925	-5.72567	1.03E-08	5.90E-08
1128	APBA2	95.20055679	-2.371904531	0.292242	-8.116224	4.81E-16	8.34E-15
3737	CCL18	271.6045134	-2.372358056	0.430235	-5.514099	3.51E-08	1.84E-07
5835	SERPINA4	21.83513599	-2.376092345	0.545661	-4.354518	1.33E-05	4.48E-05
107	C1QB	6377.491931	-2.378248786	0.194714	-12.21409	2.61E-34	4.70E-32
3031	ADAM23	30.92479912	-2.380540406	0.396084	-6.010196	1.85E-09	1.20E-08

10431	OR2AT4	0.611201819	-2.380542726	0.952298	-2.499786	0.012427	0.023351
2585	VNN3	7.988155062	-2.381858723	0.372377	-6.396362	1.59E-10	1.21E-09
3775	ARL9	3.842398777	-2.384778417	0.434468	-5.488963	4.04E-08	2.10E-07
1002	DISP2	40.01331467	-2.389082932	0.286391	-8.342033	7.30E-17	1.43E-15
940	FBN2	47.21653697	-2.392720308	0.282771	-8.461678	2.64E-17	5.50E-16
279	PKMYT1	67.54491243	-2.394383642	0.223877	-10.69508	1.07E-26	7.54E-25
447	BCL2A1	146.9234882	-2.396661596	0.24403	-9.821157	9.13E-23	4.00E-21
1090	HSPB6	406.4919711	-2.397036916	0.293277	-8.173291	3.00E-16	5.40E-15
2506	FCGBP	1440.962981	-2.39802466	0.371362	-6.457375	1.07E-10	8.33E-10
1398	TNFRSF6B	463.4311548	-2.398675208	0.31076	-7.718735	1.17E-14	1.65E-13
5626	CPA2	1.264321402	-2.401330987	0.538692	-4.457704	8.28E-06	2.89E-05
8225	MIXL1	0.795366929	-2.401338073	0.722799	-3.322275	0.000893	0.002128
5566	GOLGA8G	1.366119127	-2.401806123	0.534901	-4.490188	7.12E-06	2.51E-05
7532	FAM133A	2.97463939	-2.402948378	0.666939	-3.602948	0.000315	0.000819
5515	C14orf34	1.200417595	-2.40327563	0.532466	-4.513485	6.38E-06	2.27E-05
50	ECM1	683.8592357	-2.407070824	0.183359	-13.12761	2.29E-39	8.97E-37
1138	MT1F	510.8217288	-2.411171318	0.297394	-8.107678	5.16E-16	8.89E-15
1888	SPOCK1	2101.334197	-2.413052364	0.339993	-7.097355	1.27E-12	1.32E-11
84	FAM78B	60.76018528	-2.413401047	0.192527	-12.53536	4.78E-36	1.12E-33
886	APOC1	3190.606328	-2.418075106	0.282681	-8.55408	1.19E-17	2.63E-16
141	PYCARD	504.7872692	-2.418261083	0.206574	-11.70652	1.18E-31	1.64E-29
389	COL3A1	19731.8278	-2.419558831	0.239613	-10.09778	5.65E-24	2.84E-22
3514	C21orf125	3.971133912	-2.419678308	0.427223	-5.663741	1.48E-08	8.26E-08
7444	CHP2	1.319187025	-2.421438246	0.665468	-3.638701	0.000274	0.000721
2379	TFPI2	1596.198041	-2.421535157	0.368094	-6.578572	4.75E-11	3.91E-10
2207	SCG2	218.8476474	-2.421664221	0.358839	-6.748617	1.49E-11	1.33E-10
3977	DPYSL4	62.37976633	-2.422101441	0.452298	-5.355097	8.55E-08	4.21E-07
1448	PLAC8	109.4901602	-2.42233549	0.317181	-7.637085	2.22E-14	3.01E-13
6214	ELANE	3.807151708	-2.423737856	0.581056	-4.171265	3.03E-05	9.55E-05
1403	HIST1H2BH	3.011574824	-2.426692025	0.314665	-7.711985	1.24E-14	1.73E-13
629	CPE	10424.55255	-2.426725741	0.263381	-9.213743	3.15E-20	9.81E-19
149	KIF2C	138.0584068	-2.427989912	0.208396	-11.65084	2.27E-31	2.99E-29
10142	PWRN1	1.431075703	-2.428420954	0.93356	-2.601248	0.009289	0.017952
1176	POSTN	3584.050609	-2.428767224	0.301548	-8.054333	7.99E-16	1.33E-14
2690	NCAM2	6.937268909	-2.429462453	0.386159	-6.291356	3.15E-10	2.29E-09
8558	OXT	3.634500219	-2.431602216	0.763058	-3.186656	0.001439	0.003296
829	FMOD	849.8216133	-2.432549155	0.280279	-8.679028	3.99E-18	9.44E-17
116	OC1001307	267.1998609	-2.432706267	0.200821	-12.11383	8.92E-34	1.51E-31
3009	C2orf82	4.496172601	-2.435815212	0.404055	-6.028419	1.66E-09	1.08E-08
14707	DSCR8	0.567853178	-2.436869831	2.222083	-1.09666	0.27279	0.363565
1746	EFNA5	1020.600597	-2.437175275	0.336287	-7.247303	4.25E-13	4.77E-12
1417	GOLGA7B	151.7988973	-2.437598458	0.31741	-7.679647	1.60E-14	2.21E-13
208	KDELR3	665.6004885	-2.438188039	0.218373	-11.16524	6.03E-29	5.69E-27
1833	TNFSF14	32.48946976	-2.441607133	0.340764	-7.165103	7.77E-13	8.32E-12
950	MEI1	73.66383082	-2.44229367	0.289177	-8.445672	3.02E-17	6.24E-16
3023	PTTG3P	1.278387554	-2.443006422	0.405983	-6.017514	1.77E-09	1.15E-08
573	CXCR5	13.18456024	-2.444382102	0.260242	-9.392713	5.85E-21	2.00E-19

288	SLAMF8	761.2219608	-2.444456304	0.229833	-10.63579	2.03E-26	1.38E-24
740	PRG2	20.6783878	-2.445146663	0.274307	-8.913902	4.93E-19	1.30E-17
3617	EREG	17.40818104	-2.44535424	0.436974	-5.596114	2.19E-08	1.19E-07
2630	ACTN2	48.06693541	-2.446492721	0.385138	-6.352253	2.12E-10	1.58E-09
2041	XIRP1	13.4639891	-2.447173362	0.353628	-6.920199	4.51E-12	4.33E-11
2893	HPCAL4	16.83302293	-2.450627222	0.400727	-6.115459	9.63E-10	6.52E-09
203	CRYBB1	12.17892173	-2.450684645	0.218812	-11.19998	4.08E-29	3.94E-27
493	TNFRSF11/	58.14583756	-2.453322597	0.254217	-9.650489	4.89E-22	1.95E-20
1348	PRELP	1068.621332	-2.453390726	0.314497	-7.801007	6.14E-15	8.93E-14
3510	CPZ	104.6029567	-2.454056445	0.433195	-5.665013	1.47E-08	8.21E-08
1212	FNDC4	177.7504635	-2.454398541	0.306779	-8.000538	1.24E-15	2.00E-14
5387	MT3	475.0607646	-2.459691668	0.537655	-4.574849	4.77E-06	1.73E-05
6431	TGM5	2.862942845	-2.462724137	0.606861	-4.058134	4.95E-05	0.000151
4586	ASAM	111.271437	-2.463953965	0.493533	-4.992477	5.96E-07	2.55E-06
2649	CHI3L2	228.6296204	-2.466726717	0.390021	-6.324598	2.54E-10	1.88E-09
105	RIN1	240.9750782	-2.467833957	0.201837	-12.22689	2.23E-34	4.17E-32
9763	CNGA3	0.579967341	-2.469394896	0.905669	-2.726599	0.006399	0.012847
1770	NALCN	43.4091076	-2.469589357	0.342074	-7.219457	5.22E-13	5.78E-12
3417	INHBE	187.5734422	-2.474467009	0.431326	-5.736883	9.64E-09	5.53E-08
8440	ANKFN1	0.880722991	-2.474744436	0.765943	-3.230976	0.001234	0.002865
2132	SPRED3	2.639541116	-2.47535123	0.362784	-6.82321	8.90E-12	8.19E-11
65	TMEM132/	962.0634573	-2.476180383	0.19292	-12.83527	1.04E-37	3.14E-35
1614	IL17B	6.215748881	-2.47727729	0.33434	-7.409448	1.27E-13	1.54E-12
1255	TDO2	73.31933351	-2.477800551	0.312786	-7.921708	2.34E-15	3.66E-14
4931	MYLPF	1.574569638	-2.477934532	0.515101	-4.810582	1.50E-06	5.98E-06
912	CDH3	54.51873396	-2.478928587	0.291719	-8.497654	1.93E-17	4.16E-16
6156	HMSD	1.100838708	-2.479557897	0.59039	-4.199867	2.67E-05	8.50E-05
5598	CCL21	326.2049091	-2.481068648	0.554703	-4.47279	7.72E-06	2.70E-05
3050	GOLGA8C	6.006049119	-2.482015226	0.413714	-5.999354	1.98E-09	1.27E-08
2923	IGSF5	2.679172266	-2.486752617	0.408056	-6.094153	1.10E-09	7.38E-09
3431	ANKRD33	4.631801916	-2.486754854	0.434574	-5.722276	1.05E-08	6.00E-08
7430	RSPO1	1.113578333	-2.488807669	0.682853	-3.64472	0.000268	0.000706
4582	CLEC4GP1	2.455019282	-2.489036209	0.498213	-4.99593	5.86E-07	2.50E-06
112	C1QTNF1	1641.742297	-2.489081803	0.204454	-12.17432	4.26E-34	7.45E-32
3825	WT1	138.4983519	-2.489815493	0.456726	-5.451439	5.00E-08	2.56E-07
5645	LOC28569(	0.762283673	-2.492148342	0.559893	-4.451113	8.54E-06	2.97E-05
3127	C19orf21	147.2120989	-2.494892626	0.419649	-5.945189	2.76E-09	1.73E-08
1455	CEACAM4	16.9656138	-2.496303076	0.327186	-7.629618	2.35E-14	3.17E-13
80	SEMA7A	192.7175591	-2.496819232	0.198622	-12.57073	3.06E-36	7.49E-34
1890	RNASE3	3.042986535	-2.49869487	0.352239	-7.09375	1.31E-12	1.35E-11
666	OSM	108.4118166	-2.499328212	0.274099	-9.118349	7.63E-20	2.24E-18
2756	FXYD1	268.7128187	-2.502260707	0.401279	-6.235719	4.50E-10	3.20E-09
200	VSIG4	1037.808997	-2.502401039	0.222729	-11.23519	2.74E-29	2.68E-27
2819	PI15	53.13228449	-2.509750265	0.406664	-6.171564	6.76E-10	4.70E-09
108	COL6A2	12281.54848	-2.510800432	0.205553	-12.21485	2.59E-34	4.70E-32
100	CDCA3	90.97769897	-2.512574544	0.203874	-12.32415	6.71E-35	1.32E-32
218	FAM64A	51.94214517	-2.515478578	0.22789	-11.03814	2.50E-28	2.25E-26

578	PDE10A	654.8161452	-2.516061968	0.268215	-9.380751	6.55E-21	2.22E-19
1085	PTX3	61.3866892	-2.519186377	0.307862	-8.182849	2.77E-16	5.01E-15
91	FCGR1A	304.1417452	-2.519697913	0.202867	-12.42046	2.02E-35	4.36E-33
1273	HTRA4	28.24512874	-2.522135051	0.319371	-7.897189	2.85E-15	4.39E-14
5425	POM121L2	1.131884029	-2.527845874	0.554962	-4.554987	5.24E-06	1.89E-05
7847	CRLF2	0.743582945	-2.528867619	0.728203	-3.472751	0.000515	0.001287
3010	FAM57B	5.505370399	-2.529637833	0.41965	-6.027976	1.66E-09	1.08E-08
7183	C1QL2	0.754807667	-2.52989042	0.674478	-3.750884	0.000176	0.000481
1581	GLT25D2	76.24904246	-2.532646544	0.34022	-7.444144	9.76E-14	1.21E-12
6033	DYSFIP1	1.038731711	-2.534693127	0.596185	-4.25152	2.12E-05	6.90E-05
1317	RDM1	7.192274115	-2.534754208	0.323354	-7.83894	4.54E-15	6.76E-14
74	C1QA	6201.014057	-2.536992277	0.201095	-12.61588	1.73E-36	4.57E-34
11768	SPRR1B	0.64621662	-2.537980492	1.250218	-2.03003	0.042354	0.070545
379	CFH	2999.11845	-2.538574198	0.250617	-10.12931	4.10E-24	2.12E-22
4572	CHST4	4.226672464	-2.539479895	0.50806	-4.998388	5.78E-07	2.48E-06
667	LOXL1	405.2162873	-2.542211765	0.278808	-9.118136	7.64E-20	2.25E-18
576	MMP23B	36.62133416	-2.543565229	0.270944	-9.387781	6.13E-21	2.09E-19
298	KCNN4	60.84073561	-2.545103018	0.241257	-10.54935	5.12E-26	3.36E-24
1315	LRG1	349.6196641	-2.54900291	0.325133	-7.839881	4.51E-15	6.72E-14
4158	EN2	16.73618225	-2.549929833	0.486094	-5.245758	1.56E-07	7.34E-07
9472	HBQ1	0.799304093	-2.550629403	0.901101	-2.83057	0.004647	0.009615
7903	KERA	1.805663461	-2.552185774	0.740335	-3.447338	0.000566	0.001404
2083	HRASLS	21.95319047	-2.55355847	0.371845	-6.867268	6.54E-12	6.16E-11
473	CD163	2729.64578	-2.554818498	0.262751	-9.72333	2.40E-22	9.94E-21
6891	CACNA1B	3.310766415	-2.556422198	0.660977	-3.86764	0.00011	0.000313
2176	IL8	413.995204	-2.560350413	0.37743	-6.783652	1.17E-11	1.06E-10
10953	S100A7	0.584554873	-2.56328453	1.113277	-2.302468	0.021309	0.038133
6673	HMHB1	1.392305291	-2.564541199	0.648812	-3.952672	7.73E-05	0.000227
834	PRRX2	29.87578297	-2.566012518	0.296038	-8.667836	4.40E-18	1.04E-16
1427	HAS2	32.2912655	-2.566484463	0.3346	-7.670315	1.72E-14	2.36E-13
5957	LPO	1.842684027	-2.568274276	0.599087	-4.286982	1.81E-05	5.96E-05
6765	HOXB13	31.08475502	-2.570173368	0.656967	-3.912178	9.15E-05	0.000265
7153	AKR1D1	2.808572398	-2.571380759	0.683355	-3.762876	0.000168	0.00046
8236	LCN1	0.773301804	-2.573696314	0.775847	-3.317273	0.000909	0.002163
8579	AHSP	3.266405597	-2.573712215	0.809313	-3.180121	0.001472	0.003363
6889	CSF2	0.887988854	-2.575449986	0.66581	-3.868144	0.00011	0.000312
848	NAV3	67.17974873	-2.576637247	0.29825	-8.639196	5.66E-18	1.31E-16
164	KIF18B	77.34471592	-2.5781843	0.222681	-11.57791	5.33E-31	6.37E-29
789	AEBP1	13063.32381	-2.581255945	0.294147	-8.775396	1.70E-18	4.23E-17
146	HJURP	112.1266108	-2.581652156	0.221526	-11.65393	2.19E-31	2.92E-29
151	EME1	45.53243166	-2.582804118	0.222303	-11.61839	3.32E-31	4.31E-29
59	PTTG1	218.4473227	-2.583077526	0.199791	-12.9289	3.09E-38	1.03E-35
902	COL16A1	469.2393115	-2.583517669	0.303424	-8.514537	1.67E-17	3.63E-16
2547	IGDCC4	244.4379049	-2.585857298	0.402307	-6.427578	1.30E-10	9.98E-10
2186	GPR172B	20.0203276	-2.587288785	0.381987	-6.773234	1.26E-11	1.13E-10
10922	HTR2C	0.815318719	-2.58754487	1.119412	-2.311522	0.020804	0.037336
518	MDK	1578.301954	-2.589131023	0.271349	-9.541704	1.41E-21	5.32E-20

5572	AGRP	1.199677084	-2.589566678	0.57702	-4.487827	7.20E-06	2.53E-05
3252	DPF1	1.994900725	-2.590667618	0.442715	-5.851769	4.86E-09	2.93E-08
966	'SF12-TNF!	331.0820687	-2.592054371	0.308061	-8.41409	3.96E-17	8.03E-16
609	S100B	39.37429758	-2.595080297	0.279212	-9.294291	1.48E-20	4.78E-19
11185	CIDEA	1.084401888	-2.596066749	1.166379	-2.225749	0.026031	0.045616
799	TNNI2	11.18797511	-2.596374694	0.296489	-8.757068	2.00E-18	4.92E-17
10264	OR4C6	0.67003985	-2.597892939	1.014656	-2.560369	0.010456	0.019968
1930	IGF2BP3	253.7015111	-2.598578519	0.368289	-7.055805	1.72E-12	1.74E-11
8605	SERPINB4	0.988824155	-2.600147437	0.819279	-3.173704	0.001505	0.003428
7249	ZIC1	2.08167368	-2.600848823	0.69812	-3.725506	0.000195	0.000527
648	PPM1N	20.66182129	-2.60202619	0.283702	-9.171698	4.66E-20	1.41E-18
247	MRC2	1738.552552	-2.60411674	0.23965	-10.86634	1.67E-27	1.32E-25
1867	CLVS1	18.43554896	-2.608627259	0.366314	-7.121281	1.07E-12	1.12E-11
1394	TRPV3	4.763610501	-2.610160909	0.337946	-7.723601	1.13E-14	1.59E-13
378	ADAM8	336.4151387	-2.610524332	0.25766	-10.13165	4.00E-24	2.07E-22
5072	CCNA1	20.27152962	-2.610898805	0.551286	-4.736013	2.18E-06	8.42E-06
237	GPR84	35.08603079	-2.612619394	0.238898	-10.93613	7.74E-28	6.38E-26
7841	CACNG5	1.480680506	-2.61465113	0.751715	-3.478249	0.000505	0.001262
120	CDC20	190.1853484	-2.615218844	0.216903	-12.0571	1.78E-33	2.91E-31
971	PLTP	5355.234872	-2.616712569	0.311706	-8.394799	4.67E-17	9.42E-16
2854	KLF1	1.937523818	-2.620957342	0.426815	-6.140732	8.21E-10	5.64E-09
4965	SLC13A5	1.297165958	-2.621935982	0.547362	-4.79013	1.67E-06	6.58E-06
1572	ARTN	32.27502009	-2.623294524	0.351387	-7.465534	8.30E-14	1.03E-12
1198	ELOVL3	5.897658792	-2.626776171	0.32771	-8.01555	1.10E-15	1.79E-14
974	CAPN11	38.61783008	-2.631401287	0.313756	-8.386777	5.00E-17	1.01E-15
859	UNC13A	29.51983221	-2.631573427	0.30522	-8.621895	6.59E-18	1.50E-16
11772	SPRR1A	0.583914364	-2.633182012	1.297476	-2.029465	0.042411	0.07061
688	HSD11B1	48.836246	-2.635037765	0.291365	-9.043759	1.51E-19	4.31E-18
17	PLAUR	668.9492725	-2.639261441	0.183237	-14.40357	4.91E-47	5.67E-44
7721	CA1	13.07383167	-2.641144705	0.747804	-3.531866	0.000413	0.001047
4485	MKRN3	3.813990682	-2.641371123	0.523245	-5.048056	4.46E-07	1.95E-06
5052	VGF	12.58520722	-2.64575356	0.557523	-4.745548	2.08E-06	8.07E-06
128	CFD	321.4431732	-2.64597357	0.221962	-11.92084	9.22E-33	1.41E-30
525	TNFRSF18	33.81095557	-2.648575549	0.277905	-9.530501	1.57E-21	5.84E-20
3997	PI16	21.02412615	-2.650284792	0.496126	-5.341958	9.19E-08	4.51E-07
3313	HAR1B	3.088398921	-2.652136637	0.456587	-5.808615	6.30E-09	3.73E-08
1460	GPR64	130.6476457	-2.657465837	0.348519	-7.625022	2.44E-14	3.28E-13
3462	PF4	3.389297643	-2.658499319	0.466474	-5.699138	1.20E-08	6.82E-08
4487	C21orf88	1.796332107	-2.659399214	0.526907	-5.047185	4.48E-07	1.96E-06
353	C9orf109	14.54447787	-2.660458491	0.259313	-10.25966	1.07E-24	5.95E-23
183	TROAP	80.95761687	-2.666587513	0.234342	-11.37902	5.32E-30	5.70E-28
2100	CCDC42	1.284545807	-2.669284877	0.389728	-6.849104	7.43E-12	6.94E-11
134	PLK1	163.5850071	-2.671374337	0.226174	-11.81117	3.42E-32	5.00E-30
4986	OTP	1.058549321	-2.674915522	0.559531	-4.780641	1.75E-06	6.87E-06
2886	PLAC1	1.474020445	-2.678015095	0.437403	-6.12253	9.21E-10	6.26E-09
3413	PRAME	436.7464505	-2.678053003	0.466668	-5.738669	9.54E-09	5.48E-08
9196	EFNA2	1.089933468	-2.678365535	0.910496	-2.941655	0.003265	0.006958

12274	PAX3	0.705471742	-2.678832974	1.429527	-1.873929	0.06094	0.097319
2043	KRT36	1.946440654	-2.680846465	0.387464	-6.918948	4.55E-12	4.37E-11
369	INSL3	7.86686688	-2.682159388	0.2634	-10.18285	2.37E-24	1.26E-22
383	NTNG2	54.61405098	-2.682466994	0.265223	-10.11401	4.79E-24	2.45E-22
4703	TF	367.6775606	-2.684674917	0.544657	-4.929109	8.26E-07	3.44E-06
6040	CRNN	0.783762069	-2.685058453	0.631981	-4.248638	2.15E-05	6.98E-05
4712	MTTP	155.7566429	-2.686323264	0.545208	-4.927154	8.34E-07	3.47E-06
671	AIM2	56.13388406	-2.691232564	0.295826	-9.097338	9.26E-20	2.70E-18
513	TUBB3	613.7186466	-2.691743759	0.2819	-9.548592	1.31E-21	5.02E-20
5530	REG1A	1503.918909	-2.693297383	0.597781	-4.505493	6.62E-06	2.35E-05
220	KIF20A	226.9741305	-2.695226406	0.244599	-11.01896	3.10E-28	2.75E-26
863	ULBP1	23.26247178	-2.698118359	0.313232	-8.613815	7.07E-18	1.61E-16
1471	FNDC1	271.7320563	-2.698421951	0.354742	-7.606706	2.81E-14	3.75E-13
5512	LOC648691	1.384863078	-2.703089281	0.598811	-4.514097	6.36E-06	2.26E-05
2674	XKR3	1.685705343	-2.703220975	0.428794	-6.304234	2.90E-10	2.12E-09
634	FBLN1	1357.782386	-2.703343463	0.293754	-9.202757	3.49E-20	1.08E-18
4089	PRKCG	1.152597473	-2.704643485	0.511601	-5.286623	1.25E-07	5.97E-07
332	FAP	240.8051286	-2.704933675	0.260332	-10.39032	2.74E-25	1.62E-23
553	MXRA8	2135.139034	-2.710714093	0.286741	-9.453514	3.28E-21	1.16E-19
1541	WNT4	29.74519894	-2.711731901	0.361289	-7.50572	6.11E-14	7.77E-13
38	ZP3	237.4612736	-2.714900244	0.202152	-13.42997	4.04E-41	2.08E-38
1162	MT1X	2282.038221	-2.715514911	0.336435	-8.071437	6.95E-16	1.17E-14
5002	KCNJ6	1.274042152	-2.716077792	0.569045	-4.773043	1.81E-06	7.11E-06
1299	GXYLT2	126.9934851	-2.716974253	0.34534	-7.867534	3.62E-15	5.46E-14
490	MXRA5	1921.735815	-2.718702825	0.281553	-9.656113	4.63E-22	1.85E-20
1227	EPS8L1	165.2192414	-2.721573029	0.340983	-7.98154	1.45E-15	2.31E-14
3120	MAGEL2	6.424930742	-2.726587406	0.458366	-5.94849	2.71E-09	1.70E-08
3816	ANKRD2	16.42624723	-2.728660059	0.499427	-5.463576	4.67E-08	2.40E-07
9735	KIAA0408	5.233676092	-2.731290251	0.997742	-2.737471	0.006191	0.012465
8763	KLK8	0.810070625	-2.734243662	0.881767	-3.100868	0.00193	0.004316
2303	T6GALNA(	8.76729901	-2.736330154	0.411423	-6.650893	2.91E-11	2.48E-10
147	RARRES2	6980.354584	-2.73763833	0.234908	-11.65407	2.19E-31	2.92E-29
6703	ZIC4	1.526468633	-2.738890784	0.695125	-3.940139	8.14E-05	0.000238
786	MIAT	360.9092655	-2.739288427	0.31167	-8.789059	1.51E-18	3.76E-17
2840	DPT	66.687987	-2.740019944	0.445419	-6.151555	7.67E-10	5.30E-09
12773	LHX3	0.662953148	-2.741719804	1.600954	-1.712554	0.086795	0.133192
390	SLC17A9	256.1132332	-2.74329802	0.271676	-10.09769	5.66E-24	2.84E-22
5195	CPA5	1.595751195	-2.747223702	0.587117	-4.679178	2.88E-06	1.09E-05
3504	ISL2	2.2379686	-2.747716341	0.484488	-5.671377	1.42E-08	7.92E-08
440	CATSPER1	6.665287465	-2.749025088	0.279035	-9.851886	6.73E-23	3.00E-21
271	APOL1	10804.6783	-2.750260083	0.25631	-10.73023	7.34E-27	5.31E-25
11249	IRX4	0.664082857	-2.750679374	1.25008	-2.200403	0.027778	0.048403
5887	EPYC	2.268540517	-2.753323814	0.63589	-4.329873	1.49E-05	4.97E-05
2205	INHA	38.6678621	-2.755524315	0.408201	-6.750403	1.47E-11	1.31E-10
350	PYCR1	401.6881015	-2.756441895	0.268224	-10.27663	8.98E-25	5.03E-23
1265	PKD2L1	12.00412815	-2.756765995	0.348621	-7.907634	2.62E-15	4.06E-14
2319	RYR2	108.3383181	-2.759774815	0.4156	-6.640451	3.13E-11	2.64E-10

5284	HTR3A	13.04466196	-2.761366103	0.596123	-4.632208	3.62E-06	1.34E-05
2801	PAX5	4.020018445	-2.764331341	0.446484	-6.191339	5.97E-10	4.17E-09
1717	CYP19A1	10.48793516	-2.766587234	0.379977	-7.280942	3.31E-13	3.78E-12
5717	PRTN3	5.289301982	-2.76805035	0.627534	-4.411	1.03E-05	3.53E-05
1869	SHOX2	20.1886562	-2.771709039	0.389211	-7.121351	1.07E-12	1.12E-11
1088	C2CD4B	35.41895837	-2.772015148	0.338988	-8.177331	2.90E-16	5.23E-15
1020	GPR35	263.8712333	-2.773050503	0.334046	-8.301413	1.03E-16	1.98E-15
9992	CPLX2	2.109391103	-2.77448759	1.045227	-2.654435	0.007944	0.015584
746	NKAIN1	63.98895297	-2.778080953	0.312092	-8.901475	5.51E-19	1.45E-17
1883	DCN	2463.641949	-2.78160354	0.391559	-7.103928	1.21E-12	1.26E-11
1639	BLK	11.90047498	-2.785908507	0.377771	-7.374603	1.65E-13	1.97E-12
171	RNASE2	44.68307488	-2.790676976	0.242401	-11.51264	1.14E-30	1.31E-28
962	JAKMIP2	22.54111555	-2.791268421	0.331254	-8.426362	3.57E-17	7.27E-16
1935	TMEM130	583.1675131	-2.792654749	0.396129	-7.049868	1.79E-12	1.81E-11
1730	C19orf33	1174.686808	-2.796128227	0.384738	-7.267619	3.66E-13	4.14E-12
274	EMILIN1	1032.432653	-2.797083711	0.26109	-10.71309	8.84E-27	6.32E-25
12416	CDH18	0.804594798	-2.801705015	1.530296	-1.830825	0.067127	0.105972
12361	LOC116437	0.725489363	-2.802153498	1.516813	-1.847396	0.06469	0.102579
3897	SP7	1.02243475	-2.803715952	0.51868	-5.405485	6.46E-08	3.25E-07
2411	PRSSL1	2.398365927	-2.804214468	0.428123	-6.550026	5.75E-11	4.68E-10
6962	TLX1	1.583137311	-2.804498415	0.730019	-3.841676	0.000122	0.000344
5680	GRP	2.236093961	-2.805373922	0.632991	-4.431934	9.34E-06	3.22E-05
4010	TPSG1	67.77374271	-2.809539699	0.526659	-5.33465	9.57E-08	4.68E-07
2479	GNG4	67.01072157	-2.811788478	0.433282	-6.489517	8.61E-11	6.81E-10
8803	KRT75	1.249625317	-2.814035318	0.912064	-3.08535	0.002033	0.004527
2162	MMP7	3650.828501	-2.814379982	0.413976	-6.79841	1.06E-11	9.59E-11
2490	TMEM59L	18.18184688	-2.815232636	0.434627	-6.47736	9.33E-11	7.35E-10
892	CCIN	4.65267158	-2.821212927	0.3303	-8.541355	1.33E-17	2.92E-16
9697	ADIPOQ	3.399150331	-2.821874977	1.027309	-2.74686	0.006017	0.01216
2929	ZCCHC5	1.40863083	-2.822453041	0.46347	-6.089832	1.13E-09	7.56E-09
177	LOXL2	4294.125815	-2.82330016	0.247242	-11.41917	3.35E-30	3.71E-28
623	CAMK2N2	36.28352735	-2.828848215	0.3062	-9.238577	2.50E-20	7.86E-19
2448	OC1001889	11.68573612	-2.829724747	0.434471	-6.513027	7.37E-11	5.89E-10
6360	CST1	4.28841587	-2.829897919	0.690826	-4.096396	4.20E-05	0.000129
810	CASP5	9.01988946	-2.836747817	0.324752	-8.735111	2.43E-18	5.89E-17
1741	NPTX2	4943.689805	-2.839453758	0.391529	-7.252213	4.10E-13	4.62E-12
322	SSC5D	482.5550434	-2.842219791	0.272224	-10.44072	1.62E-25	9.84E-24
1234	HGF	451.3493114	-2.842859823	0.357121	-7.960501	1.71E-15	2.72E-14
2326	LAIR2	8.845752209	-2.844160855	0.428824	-6.632464	3.30E-11	2.78E-10
1820	GUCY2D	3.65257112	-2.844321973	0.396574	-7.172244	7.38E-13	7.95E-12
1245	MT1DP	10.61440187	-2.846191941	0.358679	-7.935204	2.10E-15	3.31E-14
7074	KRT79	0.671444649	-2.847125967	0.750493	-3.793673	0.000148	0.000411
18	TIMP1	13339.05319	-2.848905947	0.200239	-14.22751	6.18E-46	6.73E-43
606	LYPD1	152.2601442	-2.849104581	0.306395	-9.298799	1.42E-20	4.59E-19
1657	FABP6	556.4725694	-2.84921091	0.387757	-7.347921	2.01E-13	2.38E-12
8843	LOC150622	0.874740361	-2.855972127	0.929058	-3.074053	0.002112	0.004681
4311	CXorf59	3.573926595	-2.858769838	0.555054	-5.150436	2.60E-07	1.18E-06

7790	RSPO2	1.449969828	-2.86294469	0.816868	-3.504782	0.000457	0.00115
6107	SLC30A10	2.00760627	-2.863948708	0.678806	-4.219099	2.45E-05	7.87E-05
2826	SYT5	4.390609144	-2.865140468	0.464685	-6.165775	7.01E-10	4.86E-09
4549	TCAM1P	3.713716175	-2.865487661	0.571946	-5.010071	5.44E-07	2.34E-06
5385	C13orf36	3.242149889	-2.865997063	0.6263	-4.57608	4.74E-06	1.72E-05
5222	TNR	2.172304265	-2.868295112	0.614328	-4.668993	3.03E-06	1.14E-05
935	GAP43	7.904884891	-2.871145347	0.338965	-8.470324	2.45E-17	5.13E-16
1531	CES1	162.7951017	-2.874951534	0.382536	-7.515512	5.67E-14	7.26E-13
791	C16orf74	279.513276	-2.876278648	0.327942	-8.770703	1.78E-18	4.40E-17
1633	LEFTY1	37.4012344	-2.878841776	0.389915	-7.38325	1.54E-13	1.85E-12
3858	TGM4	1.738384532	-2.881807283	0.530985	-5.427282	5.72E-08	2.91E-07
761	BEST4	40.55747806	-2.882710289	0.325368	-8.859858	8.01E-19	2.06E-17
811	LOC286467	10.91385827	-2.885389991	0.330391	-8.733246	2.47E-18	5.98E-17
4302	CFHR1	8.797593966	-2.886434164	0.559825	-5.155954	2.52E-07	1.15E-06
7437	CRABP1	6.274899723	-2.889327842	0.793486	-3.641308	0.000271	0.000715
2003	MAGED4B	192.1016267	-2.890134227	0.415533	-6.955241	3.52E-12	3.44E-11
1523	PLA2G1B	6.014667599	-2.896913859	0.384809	-7.528177	5.15E-14	6.62E-13
3479	WIT1	10.63048688	-2.901222007	0.510105	-5.687502	1.29E-08	7.26E-08
5316	EPS8L3	156.263552	-2.902810147	0.629235	-4.613235	3.96E-06	1.46E-05
3677	MNX1	14.28637237	-2.909631703	0.523989	-5.552845	2.81E-08	1.50E-07
215	CDC25C	36.92180433	-2.912045145	0.263324	-11.05879	1.99E-28	1.81E-26
2731	NDP	4.834969514	-2.912466411	0.465618	-6.255059	3.97E-10	2.85E-09
744	ZFHX4	153.7735925	-2.918735795	0.327726	-8.906037	5.29E-19	1.39E-17
136	CARD11	489.0553722	-2.920761885	0.247827	-11.78548	4.64E-32	6.68E-30
897	KIAA1199	83.23117797	-2.923754282	0.342811	-8.528752	1.48E-17	3.23E-16
713	UNC5A	75.66982533	-2.925124052	0.325787	-8.978642	2.74E-19	7.54E-18
764	MT1E	1732.966172	-2.928393689	0.330921	-8.849219	8.81E-19	2.26E-17
96	PCOLCE	1140.982648	-2.934707243	0.236868	-12.38964	2.97E-35	6.07E-33
115	BIRC5	140.3926484	-2.938579879	0.242309	-12.1274	7.56E-34	1.29E-31
89	COL1A2	25117.00828	-2.939277564	0.236055	-12.45167	1.37E-35	3.02E-33
1512	AQP9	631.1722858	-2.940259981	0.389356	-7.551595	4.30E-14	5.57E-13
444	PODN	325.7147936	-2.943948006	0.299335	-9.834969	7.96E-23	3.51E-21
221	MT2A	7170.572267	-2.947235106	0.267468	-11.01903	3.09E-28	2.75E-26
14976	CLDN22	0.721470183	-2.947429454	2.904596	-1.014747	0.310227	0.406033
170	TMEM119	351.3456789	-2.949861526	0.256096	-11.51857	1.06E-30	1.23E-28
9167	REG3G	24.37418477	-2.958617645	1.002367	-2.95163	0.003161	0.006759
1488	SLC30A3	6.372880768	-2.961163245	0.390019	-7.592348	3.14E-14	4.14E-13
2595	DES	292.4988656	-2.961961998	0.463698	-6.387697	1.68E-10	1.27E-09
77	AURKB	74.97271666	-2.962716546	0.235676	-12.57112	3.04E-36	7.49E-34
317	STEAP3	1942.216949	-2.967902601	0.284017	-10.44975	1.47E-25	9.08E-24
757	DMBT1	13.78958081	-2.973589532	0.335292	-8.868655	7.40E-19	1.92E-17
142	C1S	11496.20469	-2.97421488	0.254442	-11.68915	1.45E-31	2.00E-29
7000	HSPB3	2.075962703	-2.974470406	0.777862	-3.823905	0.000131	0.000368
614	ACHE	199.077218	-2.976389009	0.320861	-9.276256	1.76E-20	5.60E-19
595	FCRLA	18.16280669	-2.98203162	0.319559	-9.331713	1.04E-20	3.43E-19
7235	KRT6C	1.211194568	-2.984191012	0.79978	-3.731266	0.000191	0.000516
431	DERL3	338.852194	-2.991206163	0.302143	-9.899962	4.16E-23	1.89E-21

2289	FCRL2	6.380164672	-2.992183027	0.449001	-6.664094	2.66E-11	2.28E-10
570	THBS2	4621.936099	-2.99468245	0.318391	-9.405674	5.17E-21	1.78E-19
8625	HBM	1.864089752	-2.997728229	0.947589	-3.163531	0.001559	0.003542
2847	HAS1	3.863387287	-2.998896036	0.487911	-6.146397	7.93E-10	5.46E-09
1831	OR13A1	2.988360885	-3.002941862	0.419114	-7.16497	7.78E-13	8.32E-12
3362	HHIPL2	2.785805455	-3.003183879	0.52039	-5.771022	7.88E-09	4.59E-08
2536	TRIM29	97.2241812	-3.003622295	0.466862	-6.433636	1.25E-10	9.63E-10
5916	DPYSL5	5.107421593	-3.009976757	0.697509	-4.315322	1.59E-05	5.28E-05
6466	MAGEC3	3.17770532	-3.021443458	0.747323	-4.043023	5.28E-05	0.00016
4546	RETN	4.532957183	-3.024432882	0.603491	-5.01156	5.40E-07	2.33E-06
6166	GCKR	9.740669152	-3.02697445	0.722028	-4.192325	2.76E-05	8.78E-05
481	MFAP2	89.25282572	-3.029724459	0.312617	-9.691504	3.28E-22	1.34E-20
4535	CPN2	116.0571602	-3.035669736	0.605115	-5.01668	5.26E-07	2.27E-06
4743	ERN2	2.539351951	-3.035789106	0.617932	-4.912818	8.98E-07	3.71E-06
4737	GRIN2B	2.782147491	-3.037867844	0.61794	-4.916119	8.83E-07	3.65E-06
979	ITIH3	118.480884	-3.040080066	0.362854	-8.378247	5.37E-17	1.08E-15
4165	KLK13	3.056916303	-3.042694394	0.580576	-5.240821	1.60E-07	7.52E-07
2571	CALB2	21.53944088	-3.044141703	0.474883	-6.410295	1.45E-10	1.11E-09
127	UBE2C	256.2786836	-3.044381052	0.255272	-11.92601	8.66E-33	1.34E-30
1372	COX6B2	2.709328151	-3.045191672	0.392235	-7.763692	8.25E-15	1.18E-13
824	WDR86	24.67594665	-3.054262767	0.351047	-8.700429	3.31E-18	7.86E-17
3493	LHFPL5	1.210469773	-3.057365162	0.538387	-5.678755	1.36E-08	7.61E-08
2772	CD19	15.92207284	-3.059281721	0.492006	-6.21798	5.04E-10	3.56E-09
483	MOCOS	192.4036892	-3.061470626	0.31601	-9.687895	3.39E-22	1.38E-20
197	TRIM46	44.81454562	-3.063385966	0.272106	-11.25806	2.11E-29	2.10E-27
612	LOX	10798.48518	-3.065291781	0.330401	-9.277494	1.74E-20	5.56E-19
3628	XDH	13.13650614	-3.066508843	0.548972	-5.585913	2.32E-08	1.26E-07
1007	PLA2G2D	54.34920262	-3.066986637	0.367939	-8.335581	7.71E-17	1.50E-15
13	GRIN2D	50.92910648	-3.074317548	0.208144	-14.77015	2.28E-49	3.44E-46
7112	CSAG3	1.021249007	-3.083777514	0.815827	-3.779939	0.000157	0.000432
7330	CSMD3	1.066549201	-3.087946911	0.837215	-3.688355	0.000226	0.000603
9355	SPRR2D	0.888119313	-3.093287679	1.074649	-2.878416	0.003997	0.008374
852	ZBTB7C	258.7198207	-3.100237072	0.359015	-8.63539	5.85E-18	1.35E-16
2088	SERPINA3	1519.259042	-3.10034284	0.452164	-6.856682	7.05E-12	6.62E-11
7127	FLJ25758	0.98533338	-3.104887184	0.82258	-3.774572	0.00016	0.000441
2778	MATN4	1.91444741	-3.108597159	0.500212	-6.214564	5.15E-10	3.63E-09
1478	GRIK4	26.71791042	-3.110401623	0.409302	-7.599282	2.98E-14	3.95E-13
816	FAM180A	16.93652089	-3.116742991	0.35753	-8.71744	2.85E-18	6.84E-17
2610	OPN4	9.209396022	-3.117705582	0.489466	-6.369608	1.90E-10	1.42E-09
888	APLP1	158.6586669	-3.123062906	0.365268	-8.55005	1.23E-17	2.72E-16
9266	BRS3	3.687328042	-3.123647875	1.073013	-2.911099	0.003602	0.007619
324	IGFBP2	1283.221862	-3.125071844	0.299422	-10.43703	1.68E-25	1.02E-23
1122	LUM	2931.405863	-3.132088756	0.385403	-8.126792	4.41E-16	7.70E-15
21	FCHO1	131.3380216	-3.133057985	0.221833	-14.12348	2.72E-45	2.54E-42
14764	GAGE2D	0.82198859	-3.136687605	2.904001	-1.080126	0.280086	0.371848
7263	XAGE1D	1.796656886	-3.140454896	0.844384	-3.719226	0.0002	0.000539
12863	MAGEB2	0.873558376	-3.148061718	1.864145	-1.688743	0.091269	0.139078

2300	ENTHD1	2.227750281	-3.151552637	0.473473	-6.656252	2.81E-11	2.39E-10
1943	LOC15222[illegible]	3.459590144	-3.153778049	0.447919	-7.040962	1.91E-12	1.93E-11
4060	FAM92B	2.580349913	-3.155985297	0.59529	-5.301594	1.15E-07	5.54E-07
1411	STRA6	33.20916492	-3.161156217	0.411221	-7.687239	1.50E-14	2.09E-13
988	MFAP4	1122.088222	-3.165666414	0.378552	-8.362562	6.14E-17	1.22E-15
358	FKBP9L	295.736287	-3.166490027	0.309382	-10.23488	1.38E-24	7.58E-23
11883	CT45A5	0.841093776	-3.169633186	1.589287	-1.994374	0.046111	0.07606
297	LEPREL2	519.2482738	-3.172775237	0.300266	-10.56654	4.26E-26	2.81E-24
1823	MGC4473	16.23827525	-3.175542323	0.442983	-7.168543	7.58E-13	8.15E-12
2451	PKP3	170.3565053	-3.177786896	0.487986	-6.512049	7.41E-11	5.93E-10
2302	CHRNA1	25.56883588	-3.180314842	0.478068	-6.652426	2.88E-11	2.45E-10
2916	PDIA2	4.035770682	-3.188186305	0.522699	-6.099475	1.06E-09	7.15E-09
3843	KLK10	13.16983809	-3.189097676	0.586484	-5.437652	5.40E-08	2.75E-07
1475	SFN	256.2105848	-3.190310967	0.419642	-7.602456	2.91E-14	3.86E-13
180	SERPINF1	1925.399823	-3.196002279	0.280092	-11.41056	3.70E-30	4.03E-28
5417	RTP3	3.372157575	-3.197234272	0.701585	-4.55716	5.19E-06	1.88E-05
7773	IGF2AS	1.262001609	-3.197431244	0.911211	-3.50899	0.00045	0.001134
6692	CAMKV	1.865913193	-3.202135258	0.812084	-3.943106	8.04E-05	0.000236
2365	GRIN3B	7.926522003	-3.211380689	0.48705	-6.593536	4.29E-11	3.56E-10
1443	PNOC	24.74276553	-3.217854968	0.421168	-7.640308	2.17E-14	2.94E-13
1332	POU2AF1	184.2430697	-3.218198535	0.411547	-7.819769	5.29E-15	7.79E-14
1036	SLC1A7	33.31525381	-3.218460385	0.389216	-8.269089	1.35E-16	2.55E-15
1745	MAST1	12.00876128	-3.219330556	0.44407	-7.249608	4.18E-13	4.70E-12
3488	CRYGN	1.297183041	-3.226925114	0.567823	-5.682982	1.32E-08	7.44E-08
70	COL5A1	4139.64062	-3.228227362	0.25414	-12.70253	5.72E-37	1.60E-34
424	RARRES1	634.0639216	-3.229318821	0.324463	-9.952818	2.45E-23	1.13E-21
1037	C9orf44	23.68679792	-3.234117651	0.391355	-8.263908	1.41E-16	2.66E-15
1280	C1QL1	1110.343535	-3.261366568	0.413398	-7.889168	3.04E-15	4.66E-14
2330	TREML3	2.688062007	-3.262190035	0.492382	-6.625328	3.46E-11	2.91E-10
895	GJB3	14.44753196	-3.270069335	0.383003	-8.537971	1.37E-17	2.99E-16
391	MFSD2A	187.1148143	-3.274598597	0.324552	-10.0896	6.14E-24	3.08E-22
3387	FGF5	16.78841878	-3.277766449	0.569458	-5.755938	8.62E-09	4.99E-08
3558	SNTG2	2.613250598	-3.281444708	0.58297	-5.62884	1.81E-08	9.99E-08
111	COL6A3	8185.683341	-3.282158208	0.269509	-12.17829	4.06E-34	7.16E-32
1667	KIAA0125	40.22370053	-3.283773204	0.447402	-7.339641	2.14E-13	2.52E-12
3057	CXorf22	6.908038162	-3.289358624	0.548889	-5.992758	2.06E-09	1.32E-08
1445	TMPRSS6	30.13247963	-3.293589463	0.431131	-7.639414	2.18E-14	2.96E-13
393	TM4SF19	25.38520761	-3.298999964	0.327171	-10.08341	6.54E-24	3.26E-22
3801	TUBA3C	11.4827268	-3.300625855	0.603016	-5.473531	4.41E-08	2.27E-07
4999	SERPINB2	4.09630827	-3.307729688	0.692787	-4.774523	1.80E-06	7.06E-06
6074	KRT78	0.979428282	-3.311290244	0.782447	-4.231965	2.32E-05	7.48E-05
1218	KREMEN2	17.14214882	-3.312214663	0.414212	-7.996417	1.28E-15	2.06E-14
27	MYBL2	227.1746833	-3.318923314	0.23883	-13.89658	6.64E-44	4.82E-41
363	F3	481.4647238	-3.321941867	0.32479	-10.22796	1.49E-24	8.02E-23
10256	DEFB103B	1.033348653	-3.323835873	1.296788	-2.56313	0.010373	0.019825
5101	RPSAP52	1.194663742	-3.324140248	0.70322	-4.727027	2.28E-06	8.75E-06
1362	LOC84740	57.45993004	-3.325141574	0.427726	-7.774005	7.60E-15	1.09E-13

917	MT1A	65.30830261	-3.330877272	0.392131	-8.494301	1.99E-17	4.26E-16
428	ADD2	62.58684025	-3.337021897	0.336153	-9.927102	3.17E-23	1.45E-21
1099	ITIH4	401.8088423	-3.339485154	0.409169	-8.161633	3.31E-16	5.89E-15
741	P2RX6	13.0200662	-3.346347309	0.375458	-8.912696	4.98E-19	1.32E-17
3680	AMPD1	11.12565603	-3.353908245	0.604144	-5.551509	2.83E-08	1.51E-07
270	ISLR	665.0850878	-3.355673246	0.312446	-10.74	6.60E-27	4.80E-25
1797	PPP1R14D	188.7242082	-3.36333827	0.467516	-7.194067	6.29E-13	6.86E-12
302	RGS17	11.01138621	-3.365416742	0.319588	-10.53048	6.25E-26	4.06E-24
2831	KRT16	4.351951416	-3.371534693	0.547068	-6.162918	7.14E-10	4.94E-09
4114	MMP12	40.13113328	-3.37222766	0.639572	-5.272629	1.34E-07	6.41E-07
1187	SCRG1	11.15701094	-3.375105884	0.42037	-8.028884	9.84E-16	1.62E-14
5872	NKX2-5	2.099454363	-3.382852093	0.78018	-4.335987	1.45E-05	4.84E-05
1519	FCRL5	101.6989716	-3.38889563	0.44984	-7.533564	4.94E-14	6.37E-13
354	PLXNB3	159.3389873	-3.389221078	0.330578	-10.25242	1.15E-24	6.39E-23
4856	NKX2-8	1.501533985	-3.393998626	0.698161	-4.861338	1.17E-06	4.71E-06
2306	(FZp434J02	6.996729887	-3.403723266	0.511915	-6.649004	2.95E-11	2.51E-10
798	CD79A	193.7437345	-3.404647495	0.388781	-8.757229	2.00E-18	4.92E-17
1100	NTRK1	8.65012324	-3.407038922	0.417504	-8.160492	3.34E-16	5.95E-15
5022	CNPY1	1.970105644	-3.413368707	0.71671	-4.762553	1.91E-06	7.46E-06
5980	TUBAL3	10.58381353	-3.414882847	0.798265	-4.277879	1.89E-05	6.18E-05
1578	PPYR1	15.67000903	-3.425849152	0.459475	-7.456009	8.92E-14	1.11E-12
3029	PRSS3	6.364374777	-3.428312184	0.570345	-6.010945	1.84E-09	1.19E-08
1440	APOA1	5.681034569	-3.428750508	0.44862	-7.642889	2.12E-14	2.89E-13
1758	CCL11	13.53947457	-3.428949796	0.474186	-7.231237	4.79E-13	5.34E-12
1357	EGFL6	19.87816747	-3.430609351	0.440624	-7.785804	6.93E-15	1.00E-13
138	LAMB3	1002.394394	-3.434351676	0.292047	-11.75957	6.31E-32	8.96E-30
42	SPOCD1	36.5041716	-3.438663275	0.258441	-13.30539	2.15E-40	1.01E-37
8025	KRT27	1.300332314	-3.451844143	1.014884	-3.401222	0.000671	0.001639
456	COL22A1	169.7279602	-3.466736666	0.3547	-9.773716	1.46E-22	6.28E-21
6464	FETUB	1.374190891	-3.469415531	0.857946	-4.043862	5.26E-05	0.000159
1686	IBSP	31.44174488	-3.479573936	0.475783	-7.313362	2.61E-13	3.03E-12
1087	B3GALT5	31.46049635	-3.486637352	0.426216	-8.180442	2.83E-16	5.10E-15
7507	DUSP13	1.619637076	-3.506559744	0.970143	-3.614479	0.000301	0.000786
530	SYCE1L	34.3231721	-3.511971906	0.368794	-9.522857	1.68E-21	6.23E-20
5027	LOC283867	1.301306912	-3.520529665	0.739476	-4.760845	1.93E-06	7.52E-06
6194	FAM9B	1.36353698	-3.522965699	0.843344	-4.177376	2.95E-05	9.33E-05
1079	ADAM6	37835.56508	-3.52368937	0.430226	-8.19032	2.61E-16	4.73E-15
6911	APCS	14.43777991	-3.523945666	0.913125	-3.859213	0.000114	0.000323
1388	CHIT1	281.0071633	-3.527489491	0.456392	-7.729071	1.08E-14	1.53E-13
2688	DQX1	2.929496221	-3.527801682	0.560704	-6.291737	3.14E-10	2.29E-09
1322	LMO1	14.19550304	-3.534509587	0.451278	-7.832215	4.79E-15	7.11E-14
967	MFAP5	79.42434284	-3.541361519	0.421377	-8.404269	4.31E-17	8.73E-16
47	CTHRC1	858.5130515	-3.550461908	0.268172	-13.2395	5.19E-40	2.16E-37
421	TGM1	64.30746039	-3.554010976	0.3569	-9.957997	2.33E-23	1.08E-21
2057	HAS2AS	1.755933386	-3.561222694	0.51665	-6.892911	5.47E-12	5.21E-11
952	FXYD3	55.71335799	-3.561693864	0.421836	-8.44332	3.08E-17	6.35E-16
78	IL2RA	112.9036247	-3.562209982	0.283343	-12.57206	3.01E-36	7.49E-34

620	BCAS1	19.31996747	-3.565077418	0.385425	-9.249741	2.25E-20	7.11E-19
987	KRT15	16.58070271	-3.582377931	0.428321	-8.363778	6.07E-17	1.21E-15
10486	LIPI	1.251032247	-3.600137646	1.453652	-2.476616	0.013263	0.024793
281	B4GALNT1	229.516752	-3.601512555	0.337329	-10.67656	1.31E-26	9.14E-25
6272	SOX1	7.09697428	-3.603844255	0.86923	-4.146019	3.38E-05	0.000106
3	C1R	9903.624723	-3.623095412	0.221996	-16.32054	7.05E-60	4.61E-56
32	CLIC3	46.32045867	-3.624658896	0.267185	-13.56609	6.36E-42	3.90E-39
5854	RTBDN	1.748751848	-3.628195725	0.835243	-4.343881	1.40E-05	4.69E-05
5241	KRT34	1.91248431	-3.63427278	0.780615	-4.655655	3.23E-06	1.21E-05
850	MAGED4	96.82405615	-3.634316194	0.420842	-8.635824	5.83E-18	1.34E-16
72	ATP8B3	287.2075602	-3.641321972	0.287563	-12.66268	9.52E-37	2.59E-34
204	PTGIS	769.0778674	-3.646962537	0.325667	-11.19844	4.15E-29	3.99E-27
19	COL1A1	30108.95519	-3.660865625	0.258232	-14.17664	1.28E-45	1.32E-42
1857	KCNK12	2.727484603	-3.667927147	0.514533	-7.128659	1.01E-12	1.07E-11
3078	OTX1	4.053721046	-3.670071024	0.613982	-5.977489	2.27E-09	1.44E-08
2509	CRYAA	51.14089849	-3.682205794	0.570507	-6.454266	1.09E-10	8.50E-10
1683	SLPI	651.6444134	-3.683980055	0.503437	-7.317661	2.52E-13	2.94E-12
408	FLNC	1068.787767	-3.702839706	0.369797	-10.01318	1.33E-23	6.40E-22
3215	CRHR1	2.375279082	-3.708451926	0.631016	-5.876952	4.18E-09	2.55E-08
477	CCL13	20.65452404	-3.71060034	0.382339	-9.704993	2.87E-22	1.18E-20
923	DNAJB13	103.2559714	-3.720614834	0.438519	-8.484496	2.17E-17	4.60E-16
5394	ZNF280A	1.485550226	-3.720815209	0.814534	-4.568029	4.92E-06	1.79E-05
1010	GREM2	9.089382844	-3.721585097	0.446975	-8.326156	8.35E-17	1.62E-15
2494	C5orf38	26.46103166	-3.722302118	0.575244	-6.470824	9.75E-11	7.66E-10
543	RSPO4	32.61796825	-3.723112978	0.392673	-9.481459	2.51E-21	9.05E-20
4950	ZAN	3.0885827	-3.734248627	0.778512	-4.796647	1.61E-06	6.39E-06
119	SYT12	154.0070105	-3.736007826	0.309733	-12.06202	1.68E-33	2.76E-31
4095	OC1002718	30.83755227	-3.755681301	0.710832	-5.283498	1.27E-07	6.07E-07
2056	TUBA3D	268.0755836	-3.756384888	0.544763	-6.895446	5.37E-12	5.12E-11
1193	LEMD1	4.252529273	-3.759439941	0.468463	-8.025049	1.01E-15	1.67E-14
2786	DIO3OS	3.578380621	-3.768127351	0.607236	-6.205375	5.46E-10	3.84E-09
6446	ADAM7	1.466767561	-3.768577797	0.930222	-4.051267	5.09E-05	0.000155
1405	RASGEF1C	25.58944761	-3.781312225	0.491043	-7.700575	1.35E-14	1.89E-13
3187	F2	245.7412091	-3.786520587	0.64285	-5.890211	3.86E-09	2.37E-08
1256	LHX2	4.660495287	-3.78925285	0.478355	-7.921429	2.35E-15	3.66E-14
756	SFRP2	1314.478809	-3.790869569	0.427409	-8.869417	7.35E-19	1.91E-17
2820	C21orf84	2.954827322	-3.792015069	0.614441	-6.171486	6.77E-10	4.70E-09
1060	WNT7B	27.32133885	-3.793746309	0.461095	-8.227696	1.91E-16	3.53E-15
8275	CT45A1	1.566272629	-3.799688348	1.150441	-3.302812	0.000957	0.002267
505	C10orf81	17.78242181	-3.805958276	0.396829	-9.590939	8.73E-22	3.39E-20
2445	TNNT3	10.38910858	-3.820407717	0.586443	-6.514548	7.29E-11	5.84E-10
1901	CCL7	3.712615303	-3.825168438	0.539836	-7.085799	1.38E-12	1.43E-11
4839	REG3A	1.602848771	-3.834706336	0.787399	-4.87009	1.12E-06	4.52E-06
841	PADI1	240.4263251	-3.838628593	0.443756	-8.650317	5.14E-18	1.20E-16
7836	ZFP42	1.693052578	-3.845936279	1.104455	-3.482203	0.000497	0.001244
890	SPINK13	119.7934523	-3.848736267	0.450453	-8.544155	1.29E-17	2.85E-16
46	SERPINE1	19219.96684	-3.869531224	0.291984	-13.25255	4.36E-40	1.86E-37

4676	WFDC12	1.975798936	-3.8763555	0.783651	-4.946533	7.55E-07	3.17E-06
1298	CDHR4	2.763501037	-3.878785455	0.493006	-7.867619	3.61E-15	5.46E-14
1423	SCNN1B	248.5573791	-3.882318893	0.505773	-7.676018	1.64E-14	2.26E-13
3758	AGR2	17.89763688	-3.885388618	0.706177	-5.502003	3.75E-08	1.96E-07
104	TREM1	126.7210999	-3.886009237	0.317753	-12.22964	2.16E-34	4.07E-32
131	MFI2	169.4745265	-3.898194514	0.329584	-11.82764	2.81E-32	4.20E-30
11	ADAMTS14	121.3054014	-3.899525106	0.259437	-15.03069	4.62E-51	8.24E-48
11153	ST8SIA3	1.693173811	-3.914125007	1.75005	-2.23658	0.025314	0.044488
2159	KLF17	2.528702714	-3.921366395	0.576697	-6.799698	1.05E-11	9.52E-11
3568	REG1B	22.48248116	-3.924800776	0.697829	-5.624301	1.86E-08	1.02E-07
294	COL10A1	65.46914471	-3.93524311	0.371315	-10.59812	3.04E-26	2.03E-24
1310	C10orf90	3.009132478	-3.940299722	0.502015	-7.848964	4.19E-15	6.28E-14
249	TCHH	94.71515842	-3.958745181	0.364876	-10.84956	2.00E-27	1.58E-25
121	CILP2	23.12524859	-3.959329099	0.328498	-12.05281	1.87E-33	3.04E-31
61	AIM1L	15.06807595	-3.966329555	0.307585	-12.89509	4.80E-38	1.54E-35
978	TRPC2	47.99359665	-3.968225621	0.473526	-8.380169	5.29E-17	1.06E-15
1254	VSTM2L	102.3507282	-3.977991788	0.502045	-7.923583	2.31E-15	3.61E-14
90	HAMP	35.66505995	-3.999745958	0.321368	-12.44601	1.47E-35	3.20E-33
6178	TNNI3	5.589066275	-4.0285316	0.962178	-4.186888	2.83E-05	8.97E-05
698	CCL19	104.6061992	-4.036989422	0.44719	-9.027463	1.76E-19	4.93E-18
1104	PTGER1	7.681849298	-4.058942522	0.497668	-8.155917	3.47E-16	6.15E-15
5114	GATA4	2.327734839	-4.061350908	0.860172	-4.721556	2.34E-06	8.97E-06
16	ADAM12	594.5671151	-4.066844275	0.279251	-14.56341	4.80E-48	5.88E-45
662	PKP1	426.3622951	-4.077218196	0.446303	-9.135534	6.51E-20	1.93E-18
29	GFPT2	598.4004892	-4.079223847	0.296207	-13.77151	3.78E-43	2.56E-40
1013	C4orf26	3.081471893	-4.108523711	0.493956	-8.317585	8.98E-17	1.74E-15
143	WFDC3	23.56516951	-4.110608208	0.352093	-11.67477	1.72E-31	2.35E-29
48	PDGFRL	191.1753376	-4.111842973	0.310854	-13.22759	6.08E-40	2.48E-37
1771	NMU	23.37499993	-4.116272632	0.570226	-7.218669	5.25E-13	5.81E-12
365	COL17A1	37.80089064	-4.11677049	0.40275	-10.22164	1.59E-24	8.52E-23
1456	ZPLD1	18.41600249	-4.118119172	0.539756	-7.629601	2.35E-14	3.17E-13
2076	CXorf30	17.81897789	-4.11948514	0.59923	-6.874634	6.21E-12	5.87E-11
874	EYA1	55.42917967	-4.123234733	0.479981	-8.590411	8.67E-18	1.94E-16
2658	C12orf36	140.93455	-4.130018544	0.653502	-6.319828	2.62E-10	1.93E-09
1066	KIRREL3	12.73162431	-4.133421247	0.503076	-8.216295	2.10E-16	3.86E-15
264	NIPAL4	21.99891162	-4.135239	0.384269	-10.76132	5.24E-27	3.89E-25
246	COL7A1	194.0523049	-4.138808402	0.380658	-10.87279	1.55E-27	1.24E-25
341	CPNE7	122.958388	-4.17310292	0.403301	-10.34737	4.30E-25	2.47E-23
174	HS3ST3A1	68.72163622	-4.176523849	0.364482	-11.4588	2.12E-30	2.39E-28
5816	C17orf93	2.439561215	-4.195236591	0.960898	-4.365954	1.27E-05	4.27E-05
5568	CALY	2.372642892	-4.219233698	0.93986	-4.489214	7.15E-06	2.52E-05
869	PLA2G4D	9.644115441	-4.224551211	0.491131	-8.601675	7.86E-18	1.77E-16
4050	MS4A8B	2.295219916	-4.23117183	0.797369	-5.306418	1.12E-07	5.41E-07
1714	KRT14	10.34437205	-4.233390827	0.581182	-7.284104	3.24E-13	3.70E-12
7621	NEUROD4	2.208282865	-4.239426507	1.187282	-3.5707	0.000356	0.000916
248	MARCO	151.4774629	-4.240969815	0.390732	-10.85392	1.91E-27	1.51E-25
362	GREM1	166.5109665	-4.249833409	0.415402	-10.23064	1.45E-24	7.83E-23

1301	TUBA3E	57.20718486	-4.259292759	0.541534	-7.865237	3.68E-15	5.55E-14
2188	ZIC2	15.22406809	-4.263032779	0.62947	-6.772413	1.27E-11	1.13E-10
752	B4GALNT4	58.72475345	-4.297133579	0.483654	-8.884735	6.41E-19	1.67E-17
884	CDH15	17.63149014	-4.299962338	0.502349	-8.55972	1.13E-17	2.51E-16
240	CCL26	10.84532106	-4.301079504	0.393755	-10.92324	8.93E-28	7.26E-26
1709	CRNA0016	2.116703724	-4.30553912	0.590868	-7.286799	3.17E-13	3.64E-12
2515	TCN1	16.66553758	-4.306966849	0.667757	-6.449903	1.12E-10	8.72E-10
118	CPXM1	309.5813209	-4.329879185	0.358285	-12.08501	1.27E-33	2.11E-31
6181	CHAT	7.043462034	-4.340232251	1.037223	-4.184474	2.86E-05	9.06E-05
676	UCN2	4.432871209	-4.345668969	0.478478	-9.082276	1.06E-19	3.08E-18
2895	FGB	6994.175212	-4.351883677	0.711735	-6.11447	9.69E-10	6.56E-09
189	LRRC15	81.00174194	-4.353475931	0.384774	-11.31438	1.11E-29	1.16E-27
442	MGC29506	435.2585413	-4.357003358	0.442877	-9.837946	7.73E-23	3.42E-21
35	SRPX2	339.5623014	-4.366844835	0.324003	-13.47777	2.11E-41	1.18E-38
266	PCBP3	110.1375045	-4.370409448	0.406749	-10.74473	6.27E-27	4.62E-25
57	MUC12	68.25812062	-4.38920126	0.338785	-12.95572	2.18E-38	7.50E-36
468	TMEM145	49.78045127	-4.392225681	0.450794	-9.743309	1.97E-22	8.25E-21
1174	WFDC10B	2.754738589	-4.441344327	0.55136	-8.055251	7.93E-16	1.32E-14
150	ITPKA	161.8034711	-4.480254267	0.384899	-11.64008	2.58E-31	3.37E-29
187	KRT19	5889.731722	-4.484275856	0.395293	-11.34418	7.93E-30	8.31E-28
214	PPP2R2C	96.94831468	-4.504625868	0.406824	-11.07265	1.70E-28	1.56E-26
1807	LOC153910	4.263865158	-4.529436487	0.630709	-7.181503	6.89E-13	7.48E-12
2483	OC1001923	3.331046101	-4.530043728	0.698433	-6.486008	8.81E-11	6.96E-10
5694	NR0B1	3.478056356	-4.534665782	1.02521	-4.423157	9.73E-06	3.35E-05
1429	GSG1	14.57657475	-4.535412035	0.591715	-7.664862	1.79E-14	2.46E-13
2468	UCA1	3.335949974	-4.536184822	0.698009	-6.498746	8.10E-11	6.43E-10
1565	IGFL2	5.920994396	-4.53760483	0.607094	-7.474302	7.76E-14	9.72E-13
1889	C20orf141	2.623563206	-4.557157532	0.642237	-7.095755	1.29E-12	1.33E-11
3415	RPTN	2.88858545	-4.571982747	0.796845	-5.737605	9.60E-09	5.51E-08
533	C5orf46	412.4407849	-4.612931071	0.484917	-9.512825	1.86E-21	6.82E-20
1606	TEX19	4.726435047	-4.624410753	0.623551	-7.416246	1.20E-13	1.47E-12
1509	SCNN1G	217.4515473	-4.631510449	0.61304	-7.554987	4.19E-14	5.44E-13
162	NEB	538.4447327	-4.638307265	0.400484	-11.58174	5.10E-31	6.14E-29
159	LOC96610	31921.69925	-4.665869346	0.402383	-11.59559	4.34E-31	5.35E-29
2859	PADI3	178.9104322	-4.675302163	0.761863	-6.13667	8.43E-10	5.78E-09
774	ANXA8	34.07457804	-4.67924834	0.530251	-8.824595	1.10E-18	2.78E-17
6	PODNL1	89.16416399	-4.688374156	0.29508	-15.88849	7.61E-57	2.49E-53
1905	ORM1	9.217636032	-4.693877026	0.662677	-7.083208	1.41E-12	1.45E-11
276	MYO1A	17.40108236	-4.698474241	0.438875	-10.70572	9.57E-27	6.80E-25
5	TGFBI	54976.30749	-4.713795184	0.296329	-15.90731	5.64E-57	2.45E-53
1660	EPO	460.7134212	-4.731506015	0.644286	-7.3438	2.08E-13	2.45E-12
58	IL6	275.661551	-4.747108594	0.366832	-12.94081	2.65E-38	8.95E-36
23	MMP9	1470.67855	-4.747954968	0.338872	-14.01104	1.33E-44	1.14E-41
1832	TMEM196	4.050984931	-4.761928264	0.664588	-7.16523	7.77E-13	8.32E-12
2278	APOC3	5.887331454	-4.772122517	0.71526	-6.671868	2.53E-11	2.17E-10
374	CST2	6.583379433	-4.817696072	0.474853	-10.14567	3.46E-24	1.81E-22
172	CHRDL2	72.05467005	-4.819274007	0.418839	-11.50627	1.23E-30	1.40E-28

1264	SLAMF9	6.119658661	-4.829482635	0.610585	-7.909604	2.58E-15	4.00E-14
6665	ORM2	2.903256753	-4.832057796	1.221426	-3.956078	7.62E-05	0.000224
155	IL20RB	1174.226487	-4.857221177	0.418395	-11.60918	3.70E-31	4.68E-29
1194	GJB6	9.338147802	-4.878752412	0.60823	-8.021223	1.05E-15	1.72E-14
2343	FGG	5033.429729	-4.879737428	0.737837	-6.613575	3.75E-11	3.14E-10
453	C5orf27	470.8978544	-4.880175298	0.498358	-9.792515	1.21E-22	5.25E-21
2545	KRT25	7.48264301	-4.886773175	0.760208	-6.428207	1.29E-10	9.94E-10
4577	ROS1	6.626206679	-4.88868144	0.978222	-4.997519	5.81E-07	2.49E-06
1921	SAA4	52.23792175	-4.927847513	0.697716	-7.062827	1.63E-12	1.66E-11
1156	PLA2G2A	29.01248503	-4.954673933	0.613171	-8.080417	6.45E-16	1.09E-14
1481	BARX1	6.169843852	-4.96243419	0.653244	-7.596597	3.04E-14	4.02E-13
1997	C8orf22	208.694212	-4.977511301	0.714675	-6.964724	3.29E-12	3.23E-11
1439	LOC44230[illegible]	6.030585335	-4.98887137	0.652413	-7.646803	2.06E-14	2.81E-13
1361	IVL	4.381996421	-5.002661414	0.643501	-7.774132	7.60E-15	1.09E-13
1734	UGT1A10	151.8632599	-5.012493332	0.690287	-7.26146	3.83E-13	4.33E-12
1306	C10orf99	936.654206	-5.013175031	0.638226	-7.854858	4.00E-15	6.01E-14
385	C11orf86	146.0070484	-5.12899227	0.50752	-10.106	5.20E-24	2.65E-22
2061	TBX5	27.43821979	-5.135182459	0.745658	-6.886782	5.71E-12	5.43E-11
88	SLC38A5	848.1739267	-5.139112924	0.412589	-12.45576	1.30E-35	2.90E-33
1433	MMP3	7.963782393	-5.161199744	0.673748	-7.660432	1.85E-14	2.53E-13
405	COMP	258.8131634	-5.168951743	0.51605	-10.01637	1.29E-23	6.24E-22
60	IL1R2	1077.100167	-5.186235971	0.401662	-12.91194	3.85E-38	1.26E-35
842	ANXA8L2	21.80479808	-5.188827989	0.599895	-8.649558	5.17E-18	1.20E-16
290	IGFBP1	1568.022742	-5.234519664	0.492418	-10.63023	2.16E-26	1.46E-24
1781	SP8	4.53032703	-5.295079966	0.734765	-7.20649	5.74E-13	6.32E-12
2597	UMOD	157.7338612	-5.299245574	0.830301	-6.38232	1.74E-10	1.32E-09
801	GAS2L2	8.845580445	-5.316912672	0.607506	-8.752033	2.10E-18	5.13E-17
882	HPR	17.89028041	-5.335584224	0.623067	-8.563415	1.10E-17	2.44E-16
919	EDN3	8.361573072	-5.351099386	0.630108	-8.492356	2.02E-17	4.32E-16
43	CADM3	964.6506064	-5.385110733	0.404795	-13.30329	2.22E-40	1.01E-37
92	TRIM54	221.5710216	-5.393107972	0.434475	-12.41292	2.22E-35	4.74E-33
260	STMN2	22.27191206	-5.4153612	0.502073	-10.786	4.01E-27	3.02E-25
66	APCDD1L	171.3027669	-5.440783944	0.424815	-12.80741	1.49E-37	4.43E-35
190	CXCL5	325.1004735	-5.462220885	0.482885	-11.31163	1.15E-29	1.19E-27
1257	TFF2	8.309304719	-5.462420825	0.689619	-7.920922	2.36E-15	3.68E-14
4642	RTL1	5.262749584	-5.513472456	1.110044	-4.966896	6.80E-07	2.87E-06
339	SYT8	10.69459309	-5.537971275	0.534448	-10.36205	3.69E-25	2.13E-23
41	WISP2	319.6295661	-5.581829523	0.419298	-13.31232	1.96E-40	9.38E-38
54	KCNK17	38.62632639	-5.601393703	0.429838	-13.03142	8.11E-39	2.94E-36
239	HMGA2	35.07081449	-5.671112906	0.518935	-10.92836	8.44E-28	6.92E-26
649	CRP	55.0150962	-5.771972754	0.629364	-9.171116	4.68E-20	1.41E-18
5657	S100G	5.861718122	-5.850293554	1.316331	-4.444395	8.81E-06	3.05E-05
357	DMRT3	10.40219493	-5.908495448	0.576928	-10.2413	1.29E-24	7.11E-23
198	ZP1	23.70564793	-5.912525919	0.525331	-11.25485	2.19E-29	2.17E-27
82	COL11A1	301.1608749	-5.942181432	0.473146	-12.55888	3.55E-36	8.49E-34
660	FGA	5327.516734	-5.966342314	0.652285	-9.146827	5.86E-20	1.74E-18
226	SLN	60.09492008	-6.062858554	0.551374	-10.99591	4.00E-28	3.47E-26

9	CPA4	120.6453979	-6.063593211	0.400187	-15.15192	7.36E-52	1.60E-48
268	CIDEC	116.5420446	-6.14671231	0.572246	-10.74139	6.51E-27	4.76E-25
7	FAM101A	216.0635704	-6.216751695	0.398475	-15.60134	7.13E-55	2.00E-51
2374	C4orf7	11.5473766	-6.416514104	0.974356	-6.585388	4.54E-11	3.75E-10
2	PTPRH	141.639771	-6.49595744	0.387452	-16.76585	4.34E-63	4.25E-59
956	WFDC5	21.32369033	-6.663110099	0.790083	-8.433426	3.36E-17	6.88E-16
153	LOC55908	115.6207058	-6.671782876	0.574381	-11.61561	3.43E-31	4.40E-29
24	PI3	72.25546181	-6.692262098	0.478101	-13.99758	1.61E-44	1.32E-41
371	SBSN	12.86503031	-6.869624727	0.674952	-10.17795	2.49E-24	1.31E-22
67	KCNS1	148.5805055	-6.873958763	0.538478	-12.76553	2.55E-37	7.47E-35
236	PAEP	83.01309762	-7.106995337	0.649326	-10.94519	7.01E-28	5.82E-26
292	MMP13	24.72373128	-7.113674876	0.670387	-10.61129	2.64E-26	1.77E-24
169	SAA2	1929.471898	-7.166806912	0.622144	-11.51954	1.05E-30	1.22E-28
1136	SLC18A3	247.2307102	-7.258705208	0.895072	-8.109632	5.08E-16	8.76E-15
961	MUC17	62.4056893	-7.364171065	0.873923	-8.426566	3.56E-17	7.26E-16
10	PTPRN	524.7743098	-7.746379139	0.513351	-15.08983	1.89E-51	3.70E-48
69	SAA1	4546.029676	-7.808028156	0.613616	-12.72461	4.32E-37	1.23E-34
15	TNNT1	139.4531062	-7.874964738	0.539106	-14.60744	2.52E-48	3.51E-45
14	LBP	3344.393946	-8.543478614	0.58505	-14.60299	2.69E-48	3.51E-45
1	HP	2198.908358	-8.712206253	0.499128	-17.45484	3.16E-68	6.20E-64
1096	APOA4	10.97467917	-10.09141712	1.235622	-8.167077	3.16E-16	5.65E-15
