# Supplementary material for: Prognostic and therapeutic implications of extracellular matrix associated gene signature in renal clear cell carcinoma
Source: Sci Rep. 2021 Apr 7;11:7561. doi: 10.1038/s41598-021-86888-7 (PMC8026590; doi:10.1038/s41598-021-86888-7)
Supplement: Supplementary file 7 — Supplementary Information 7. [file 41598_2021_86888_MOESM7_ESM.pdf]

Prognostic and therapeutic implications of extracellular matrix associated gene signature in renal clear cell carcinoma

Pankaj Ahluwalia[1], Meenakshi Ahluwalia[1], Ashis K. Mondal[1], Nikhil Sahajpal[1], Vamsi Kota[2], Mumtaz V. Rojiani[1], Amyn M. Rojiani[1], and Ravindra Kolhe[1]*

[1] Department of Pathology, Medical College of Georgia, Augusta University, GA, U.S.A.
[2] Department of Medicine, Medical College of Georgia, Augusta University, GA, U.S.A.
[*] Correspondence: rkolhe@augusta.edu; Tel.: (706)-721-2771; Fax :(706)-434-6053

Supplementary table 5: GSEA analysis of Low-risk patients.

	GS	SIZE	ES	NES	NOM p-val	FDR q-val	FWER p-val	RANK AT MAX	LEADING EDGE
	follow link to MSigDB								
1	GO_ACTIVE_ION_TRANSMEMBRANE_TRANSPORTER_ACTIVITY	221	-0.56	-2.4	0	0	0	2162	tags=36%, list=11%, signal=40%
2	GO_ACTIVE_TRANSMEMBRANE_TRANSPORTER_ACTIVITY	326	-0.54	-2.38	0	0	0	2162	tags=31%, list=11%, signal=34%
3	GO_SECONDARY_ACTIVE_TRANSMEMBRANE_TRANSPORTER_ACTIVITY	222	-0.53	-2.27	0	0	0.001	2162	tags=33%, list=11%, signal=36%
4	GO_PYROPHOSPHATE_HYDROLYSIS_DRIVEN_PROTON_TRANSMEMBRANE_TRANSPORTER_ACTIVITY	30	-0.73	-2.25	0	0	0.001	2224	tags=40%, list=11%, signal=45%
5	GO_ANION_TRANSMEMBRANE_TRANSPORTER_ACTIVITY	339	-0.5	-2.25	0	0	0.001	1691	tags=27%, list=8%, signal=29%
6	GO_ORGANIC_ANION_TRANSMEMBRANE_TRANSPORTER_ACTIVITY	219	-0.52	-2.21	0	0	0.001	1691	tags=30%, list=8%, signal=32%
7	GO_PROTON_TRANSMEMBRANE_TRANSPORTER_ACTIVITY	96	-0.58	-2.21	0	0	0.002	2224	tags=27%, list=11%, signal=30%
8	GO_INORGANIC_ANION_TRANSMEMBRANE_TRANSPORTER_ACTIVITY	143	-0.54	-2.18	0	0.001	0.003	2047	tags=32%, list=10%, signal=36%

9	GO_ORGANIC_ACID_TRANSMEMBRANE_TRANSPORTER_ACTIVITY	155	-0.52	-2.15	0	0	0.003	1604	tags=31%, list=8%, signal=33%
10	GO_ANTIPORTER_ACTIVITY	74	-0.59	-2.13	0	0.001	0.005	2012	tags=31%, list=10%, signal=34%
11	GO_SYMPORTER_ACTIVITY	140	-0.52	-2.12	0	0.001	0.006	2162	tags=34%, list=11%, signal=38%
12	GO_BICARBONATE_TRANSMEMBRANE_TRANSPORTER_ACTIVITY	21	-0.75	-2.09	0	0.001	0.008	1956	tags=62%, list=10%, signal=69%
13	GO_SOLUTE_SODIUM_SYMPORTER_ACTIVITY	72	-0.56	-2.07	0	0.001	0.012	2313	tags=43%, list=12%, signal=49%
14	GO_PROTON_EXPORTING_ATPASE_ACTIVITY_PHOSPHORYLATIVE_MECHANISM	17	-0.77	-2.06	0	0.002	0.016	2224	tags=47%, list=11%, signal=53%
15	GO_PRIMARY_ACTIVE_TRANSMEMBRANE_TRANSPORTER_ACTIVITY	103	-0.53	-2.05	0	0.002	0.018	2224	tags=27%, list=11%, signal=30%
16	GO_ATPASE_COUPLED_ION_TRANSMEMBRANE_TRANSPORTER_ACTIVITY	57	-0.59	-2.04	0	0.002	0.024	2224	tags=33%, list=11%, signal=37%
17	GO_COA_HYDROLASE_ACTIVITY	21	-0.72	-2.01	0	0.005	0.052	1969	tags=43%, list=10%, signal=47%
18	GO_SOLUTE_CATION_SYMPORTER_ACTIVITY	98	-0.53	-2	0	0.005	0.056	2162	tags=37%, list=11%, signal=41%
19	GO_SOLUTE_ANION_ANTIPORTER_ACTIVITY	28	-0.67	-2	0	0.005	0.06	1470	tags=39%, list=7%, signal=42%

20	GO_CHLORIDE_TRANSMEMBRANE_TRANSPORTER_ACTIVITY	101	-0.51	-1.99	0	0.005	0.069	2158	tags=33%, list=11%, signal=36%
GS	GS DETAILS	ES	NES	NOM p-val	FDR q-val	FWER p-val	RANK AT MAX	LEADING EDGE	
follow link to MSigDB									
1	HALLMARK_FATTY_ACID_METABOLISM	154	-0.51	-2.1	0	0	0	3898	tags=47%, list=19%, signal=58%
2	HALLMARK_OXIDATIVE_PHOSPHORYLATION	182	-0.5	-2.07	0	0	0.001	5345	tags=51%, list=27%, signal=69%
3	HALLMARK_BILE_ACID_METABOLISM	112	-0.42	-1.67	0	0.004	0.013	3145	tags=36%, list=16%, signal=42%
4	HALLMARK_PROTEIN_SECRETION	95	-0.42	-1.63	0	0.004	0.019	5636	tags=47%, list=28%, signal=66%
5	HALLMARK_ANDROGEN_RESPONSE	96	-0.4	-1.52	0.014	0.012	0.066	3703	tags=28%, list=19%, signal=34%
6	HALLMARK_PEROXISOME	101	-0.37	-1.41	0.016	0.035	0.218	3755	tags=31%, list=19%, signal=38%

7	HALLMARK_APICAL_SURFACE	43	-0.42	-1.4	0.062	0.033	0.235	660	tags=14%, list=3%, signal=14%
8	HALLMARK_PANCREAS_BETA_CELLS	39	-0.42	-1.34	0.124	0.048	0.36	2894	tags=36%, list=14%, signal=42%
9	HALLMARK_ADIPOGENESIS	189	-0.3	-1.26	0.054	0.083	0.595	3145	tags=28%, list=16%, signal=33%
10	HALLMARK_HEME_METABOLISM	190	-0.21	-0.88	0.834	0.764	1	4723	tags=32%, list=24%, signal=42%
