# Supplementary material for: Prognostic and therapeutic implications of extracellular matrix associated gene signature in renal clear cell carcinoma
Source: Sci Rep. 2021 Apr 7;11:7561. doi: 10.1038/s41598-021-86888-7 (PMC8026590; doi:10.1038/s41598-021-86888-7)
Supplement: Supplementary file 8 — Supplementary Information 8. [file 41598_2021_86888_MOESM8_ESM.pdf]

Prognostic and therapeutic implications of extracellular matrix associated gene signature in renal clear cell carcinoma

Pankaj Ahluwalia[1], Meenakshi Ahluwalia[1], Ashis K. Mondal[1], Nikhil Sahajpal[1], Vamsi Kota[2], Mumtaz V. Rojiani[1], Amyn M. Rojiani[1], and Ravindra Kolhe[1]*

[1] Department of Pathology, Medical College of Georgia, Augusta University, GA, U.S.A.
[2] Department of Medicine, Medical College of Georgia, Augusta University, GA, U.S.A.
[*] Correspondence: rkolhe@augusta.edu; Tel.: (706)-721-2771; Fax :(706)-434-6053

Supplementary table 6: GSEA analysis of High-risk patients.

GS	GS DETAILS	ES	NES	NOM p-val	FDR q-val	FWER p-val	RANK AT MAX	LEADING EDGE	
follow link to MSigDB									
1	GO_EXTRACELLULAR_MATRIX_STRUCTURAL_CONSTITUENT	160	0.64	2.29	0	0	0	2951	tags=54%, list=15%, signal=63%
2	GO_CYTOKINE_ACTIVITY	221	0.62	2.28	0	0	0	3337	tags=46%, list=17%, signal=54%
3	GO_CHEMOKINE_ACTIVITY	47	0.71	2.13	0	0	0	3217	tags=62%, list=16%, signal=73%
4	GO_COLLAGEN_BINDING	64	0.66	2.11	0	0	0	2459	tags=44%, list=12%, signal=50%
5	GO_SERINE_TYPE_ENDOPEPTIDASE_INHIBITOR_ACTIVITY	88	0.62	2.08	0	0	0.001	1980	tags=40%, list=10%, signal=44%
6	GO_CHEMOKINE_RECEPTOR_BINDING	60	0.67	2.07	0	0	0.001	3217	tags=52%, list=16%, signal=61%
7	GO_ENDOPEPTIDASE_REGULATOR_ACTIVITY	176	0.56	2.05	0	0	0.002	2741	tags=38%, list=14%, signal=43%

8	GO_CYTOKINE_RECEPTOR_BINDING	248	0.54	2.04	0	0	0.00 2	3234	tags=35%, list=16%, signal=42%
9	GO_PEPTIDASE_REGULATOR_ACTIVIT Y	210	0.54	2	0	0.0 01	0.00 7	2741	tags=34%, list=14%, signal=39%
10	GO_METALLOENDOPEPTIDASE_ACTIVI TY	101	0.58	1.98	0	0.0 01	0.00 9	2166	tags=35%, list=11%, signal=39%
11	GO_COMPLEMENT_BINDING	20	0.77	1.96	0	0.0 01	0.01 6	1820	tags=60%, list=9%, signal=66%
12	GO_RECEPTOR_REGULATOR_ACTIVITY	477	0.49	1.95	0	0.0 01	0.02	3343	tags=37%, list=17%, signal=43%
13	GO_IMMUNE_RECEPTOR_ACTIVITY	124	0.55	1.94	0	0.0 01	0.02 4	4055	tags=55%, list=20%, signal=68%
14	GO_EXTRACELLULAR_MATRIX_STRUC TURAL_CONSTITUENT_CONFERRING_T ENSILE_STRENGTH	40	0.66	1.93	0	0.0 02	0.03 3	2121	tags=50%, list=11%, signal=56%
15	GO_CYTOKINE_BINDING	127	0.55	1.93	0	0.0 02	0.03 4	3067	tags=44%, list=15%, signal=52%
16	GO_CCR_CHEMOKINE_RECEPTOR_BIN DING	39	0.66	1.9	0	0.0 02	0.04 5	3119	tags=51%, list=16%, signal=61%
17	GO_CHEMOKINE_BINDING	29	0.68	1.88	0.00 2	0.0 04	0.07 5	3427	tags=55%, list=17%, signal=66%
18	GO_GLYCOSAMINOGLYCAN_BINDING	208	0.51	1.88	0	0.0 04	0.07 6	3340	tags=40%, list=17%, signal=48%

19	GO_LIPOPOLYSACCHARIDE_BINDING	25	0.7	1.88	0	0.0<br>03	0.07<br>7	1520	tags=44%,<br>list=8%,<br>signal=48%
20	GO_C_C_CHEMOKINE_BINDING	20	0.74	1.87	0	0.0<br>04	0.09<br>2	3427	tags=70%,<br>list=17%,<br>signal=84%
GS	GS DETAILS	ES	NES	NOM<br>p-val	FD<br>R q-<br>val	FW<br>ER<br>p-<br>val	RA<br>NK<br>AT<br>MA<br>X	LEAD<br>ING<br>EDGE	
follow link<br>to MSigDB									
1	HALLMARK_EPITHELIAL_MESENCHYM<br>AL_TRANSITION	194	0.69	2.55	0	0	0	3319	tags=58%,<br>list=17%,<br>signal=69%
2	HALLMARK_ALLOGRAFT_REJECTION	195	0.63	2.35	0	0	0	4998	tags=62%,<br>list=25%,<br>signal=81%
3	HALLMARK_INFLAMMATORY_RESPON<br>SE	197	0.56	2.07	0	0	0	3270	tags=43%,<br>list=16%,<br>signal=51%
4	HALLMARK_COAGULATION	136	0.58	2.05	0	0	0	2205	tags=35%,<br>list=11%,<br>signal=39%
5	HALLMARK_E2F_TARGETS	187	0.52	1.92	0	0	0.00<br>1	4619	tags=42%,<br>list=23%,<br>signal=54%
6	HALLMARK_IL6_JAK_STAT3_SIGNALIN<br>G	87	0.56	1.88	0	0	0.00<br>1	3500	tags=44%,<br>list=18%,<br>signal=53%

7	HALLMARK_G2M_CHECKPOINT	184	0.5	1.83	0	0	0.00 2	4421	tags=39%, list=22%, signal=50%
8	HALLMARK_INTERFERON_GAMMA_RE SPONSE	196	0.48	1.8	0	0.0 01	0.00 5	5114	tags=43%, list=26%, signal=57%
9	HALLMARK_COMPLEMENT	195	0.48	1.78	0	0.0 01	0.00 5	3305	tags=33%, list=17%, signal=40%
10	HALLMARK_TNFA_SIGNALING_VIA_NF KB	197	0.47	1.72	0	0.0 01	0.01 1	5692	tags=45%, list=28%, signal=63%
11	HALLMARK_APICAL_JUNCTION	194	0.47	1.71	0	0.0 01	0.01 1	4113	tags=38%, list=21%, signal=47%
12	HALLMARK_HYPOXIA	190	0.46	1.7	0	0.0 01	0.01 5	2913	tags=28%, list=15%, signal=32%
13	HALLMARK_APOPTOSIS	158	0.43	1.58	0	0.0 1	0.11 1	6215	tags=45%, list=31%, signal=65%
14	HALLMARK_ANGIOGENESIS	36	0.54	1.54	0.01 5	0.0 14	0.17 5	1461	tags=28%, list=7%, signal=30%
15	HALLMARK_INTERFERON_ALPHA_RES PONSE	92	0.45	1.52	0.00 8	0.0 18	0.22 6	6562	tags=49%, list=33%, signal=72%
16	HALLMARK_GLYCOLYSIS	196	0.41	1.5	0.00 3	0.0 2	0.26 7	4402	tags=39%, list=22%, signal=49%
17	HALLMARK_MYOGENESIS	197	0.4	1.49	0.00 4	0.0 23	0.31 5	2850	tags=23%, list=14%, signal=26%

18	HALLMARK_KRAS_SIGNALING_UP	193	0.39	1.43	0.00 6	0.0 36	0.45 9	3404	tags=34%, list=17%, signal=41%
19	HALLMARK_KRAS_SIGNALING_DN	188	0.39	1.43	0.00 6	0.0 37	0.48 5	3005	tags=31%, list=15%, signal=36%
20	HALLMARK_ESTROGEN_RESPONSE_LA TE	196	0.35	1.3	0.03 5	0.1 16	0.90 6	4075	tags=36%, list=20%, signal=44%
